# Investigating the effects of Carpesii fructus extract on the liver transcriptome of olive flounder (*Paralichthys olivaceus*) as a potential antiparasitic agent

**DOI:** 10.1590/1678-4685-GMB-2023-0146

**Published:** 2024-03-04

**Authors:** Sang Yoon Lee, Hwa Jin Lee, Na Young Kim, Min Sun Kim

**Affiliations:** 1 CellQua, Inc, Seongnam, South Korea.; 2 Kongju National University, Department of Biological Sciences, Gongju, South Korea .; 3 National Institute of Fisheries Science, Pathology Research Division, Busan, South Korea .

**Keywords:** Paralichthys olivaceus, Carpesii fructus extract, scuticociliatosis, transcriptome, differentially expressed genes

## Abstract

Olive flounder (*Paralichthys olivaceus*), a popular aquaculture species, is plagued by the disease scuticociliatosis caused by *Miamiensis avidus*, which has a high mortality rate and is typically treated with chemicals such as formalin and hydrogen peroxide. However, Carpesii fructus extract has shown potential as a natural therapeutic agent by reducing the motility of *M. avidus*. However, despite its potential importance, the effect of the extract on fish metabolism remains unknown. In this study, the effect of Carpesii fructus extract and formalin on fish metabolism was analysed by whole transcriptome analysis in the liver of *P. olivaceus*. A total of 37,796 transcripts were generated and differential expression genes (DEGs) were identified in the liver of *P. olivaceus* treated with Carpesii fructus extract or formalin. In addition, functional analysis of DEGs between treatment groups was presented using Gene Ontology. These results will be crucial for the study of scuticociliatosis in various fish species, including *P. olivaceus*, and for the development of therapeutic agents for other diseases.

The olive flounder (*Paralichthys olivaceus*) is a widely cultivated species in South Korea, with a well-established market in Asia, the United States and worldwide (Stieglitz et al., 2021). As a result, numerous studies have been conducted on *P. olivaceus* in various fields, such as breeding, feed nutrition, and diseases in aquaculture (Liu et al., 2018; Hamidoghli et al., 2020; Jung et al., 2020; Park et al., 2021). The genome assembly of *P. olivaceus* has also been completed and its data are continuously updated (Wei et al., 2017). Despite significant progress, disease problems persist in *P. olivaceus* aquaculture, and chemical treatments such as antibiotics, formalin (37% formaldehyde) and hydrogen peroxide have been commonly used (Harikrishnan et al., 2010; Lee et al., 2017). However, alternative disease prevention and treatment methods have recently emerged, such as vaccines, specific wavelengths of light, and natural extracts (Roh et al., 2018; Fan et al., 2019; Wu et al., 2022). One of the significant challenges in *P. olivaceus* aquaculture is scuticociliatosis, a parasitic infection that causes high mortality rates. Among the parasites causing this disease, *M. avidus* has been reported to be particularly lethal compared to *Pseudocohnilembus persalinus*, *Pseudocohnilembus hargisi* and *Uronema marinum* (Song et al., 2009; Whang et al., 2013). Although formalin and hydrogen peroxide have been commonly used to treat scuticociliatosis in fish, Carpesii fructus, a medicinal plant used in traditional Chinese medicine to treat parasitic infections, has recently been found to be effective (Peng et al., 2015; Zhang et al., 2015). In addition, Carpesii fructus has been reported to have antioxidant and anti-hemolytic properties (Kang et al., 2013). While studies have been conducted on the toxicity of Carpesii fructus extracts in zebrafish and Hirame natural embryo (HINAE) cells and its efficacy in treating scuticociliatosis in fish (Xia et al., 2017; Woo et al., 2022), very few studies have investigated the safety and reactivity of this treatment. Therefore, this study aims to examine the effects of formalin and Carpesii fructus extract, both of which can be used as therapeutic agents for scuticociliatosis, on the liver of *P. olivaceus*. Rather than focusing only on specific genes whose functions and mechanisms have been previously reported, this study aims to provide integrated information on whole-transcriptome expression analysis by RNA-seq. This approach will provide valuable data for further research into disease treatment in fish.

The entire process of the fish experiment was conducted under the guidelines of the Institutional Animal Care and Use Committee (IACUC) of Sejong University (SJ-20210406). The ethics committee of Sejong University reviewed all experiment methods. In addition, the present study’s authors have obtained the Animal Welfare & Ethics Course certification under the research ethics and compliance training program.

The experiment used juvenile olive flounder (*Paralichthys olivaceus*) with an average weight and length of 22.9±2.5 g and 13.6±0.22 cm, respectively. The fish were obtained from a local aquafarm (latitude: 36.9848180; longitude: 126.3755814) and reared in tanks with a seawater recirculation system, maintaining a constant water temperature of 20-21 °C. For two weeks prior to the *in vivo* experiment, all fish were fed the basal diet (Suhyup Feed, Gyeongsangnam-do, South Korea) twice daily. The Carpesii fructus powder was extracted with 50% ethanol and has been shown to have antiparasitic potential against *M. avidus* and its immune effect on Hirame Natural Embryonic (HINAE) cells has been verified (Woo et al., 2022). The Carpesii fructus extract was incorporated into a powdery feed (Suhyup Feed) at a concentration of 100 ppm. A powdery feed mixture was subsequently formed into feed pellets (about 0.25 g). During the *in vivo* experiment, Carpesii fructus feed pellets were orally administered once a day for two days. Stimultaneously, formalin (37% formaldehyde; Junsei Chemical, Tokyo, Japan) and Carpesii fructus extract were administered by immersion at a concentration of 100 ppm for the same period, respectively. All fish including control groups were fed the basal diet (Suhyup Feed) daily. For each group, ten fish were sampled at 0, 3, 6, 12, 24, 72 and 168 h after immersion or oral treatment. The fish were anaesthetised with 200 ppm tricaine methanesulphonate (MS-222) and the liver was stored in an ultra freezer at -80°C for total RNA extraction. Total RNA was extracted from each tissue using Ribo EX Reagent (GeneAll, Seoul, South Korea) and purified using the Hybrid-RTM Kit (GeneAll) according to the manufacturer’s instructions. The quantity and quality of total RNA was measured using ND-1000 Nanometer (Thermo Fisher Scientific, USA) and Bioanalyzer (Agilent, Santa Clara, CA). Table S1 shows the MIxS descriptions used in this study.

Whole-transcriptome next-generation sequencing (NGS) libraries were prepared using the MGIEasy RNA Directional Library Prep Kit (MGI, Shenzhen, China) and sequenced using the MGISEQ-2000 platform (MGI) in 150 bp paired-end mode. Adaptor sequences, low-quality sequences (limit=0.05), and ambiguous nucleotides (maximal two nucleotides) were removed using CLC Genomics Workbench version 11.0 (CLC bio, Qiagen, Denmark). The trimmed reads were then mapped to the *P. olivaceus* reference genome assembly (GCF_001970005.1) containing 37,796 transcripts. Duplicate sequences were removed using OmicsBox 2.1.2 (BioBam, Valencia, Spain). CLC Genomics Workbench version 11.0 (CLC bio) was used for mapping with default parameters: mismatch cost = 2, insertion cost = 3, length fraction = 0.9, similarity fraction = 0.9, and maximum hits for a read = 10, minimum read count fusion gene table = 5. The percentages of reads mapped in pairs, reads mapped in broken pairs, and reads not mapped for each group in the *P. olivaceus* genome assembly were 74.63% - 82.32%, 2.45% - 5.25%, and 14.43% - 22.25%, respectively (Table 1). Principal-component analysis (PCA) was performed to evaluate differences in expression in the liver of *P. olivaceus* according to the type and method of treatment. The formalin-treated group and the Carpesii fructus extract-treated group were classified based on the type of material treated, but there was no difference depending on the treatment method (Figure 1A ). Additionally, the whole-transcript information of *P. olivaceus* used as a reference for RNA-seq data in this study is included in Table S2.

**Table 1 -:** Summary of the RNA sequencing data.

Group	Time	Treatment	Number of reads	Reads mapped in pairs (%)	Reads mapped in broken pairs (%)	Reads Not mapped (%)
Experiment-I formalin (100 ppm)	0h	Control	47,941,374	81.1	3.5	15.4
	3h	Immersion	43,557,390	81.64	2.96	15.4
	6h	Immersion	51,860,140	82.2	2.72	15.08
	12h	Immersion	54,575,220	82.17	2.81	15.02
	24h	Immersion	51,547,922	82.32	3.03	14.65
	72h	Immersion	53,928,464	81.99	3.58	14.43
	168h	Immersion	51,259,002	82.3	3	14.7
Experiment-II Carpesii Fructus (100 ppm)	0h	Control	43,170,318	74.63	3.12	22.25
	3h	Immersion	38,023,916	78.05	2.62	19.33
	6h	Immersion	41,873,994	79.85	2.98	17.17
	12h	Immersion	41,358,076	80.66	2.86	16.48
	24h	Immersion	44,460,316	80.85	2.99	16.16
	72h	Immersion	51,138,610	80.44	3.72	15.84
	168h	Immersion	49,849,982	81.68	3.26	15.06
	3h	Oral	45,029,262	75.64	2.99	21.37
	6h	Oral	52,813,362	80.47	4.28	15.25
	12h	Oral	43,441,910	80.51	2.45	17.04
	24h	Oral	47,118,490	80.8	2.87	16.33
	72h	Oral	44,633,086	79.64	5.25	15.11
	168h	Oral	43,231,022	81.89	3.3	14.81

**Figure 1 -:**
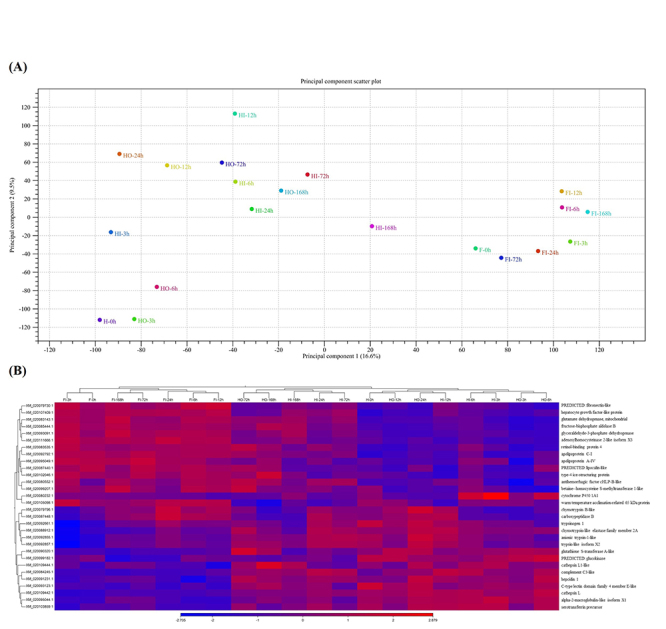
Description of transcript features in *P. olivaceus* RNA-seq. (A) Principal component analysis (PCA) plot of RNA-seq in formalin and Carpesii fructus extract treated groups. The x-axis indicates principal component 1 (16.6%), the y-axis principal component 2 (9.5%). Each sample was given its own colour and label. (B) Heatmap examination was used to cluster 30 differentially expressed genes (DEGs) with the highest coefficient of variation in the formalin and Carpesii fructus extract treated groups, respectively. Selected genes (FDR p-value cutoff = 0.05, minimum fold change =1.5) were represented by different saturated colours according to their expression value. Red indicates highly expressed genes and blue indicates low expressed genes.

*In silico* prediction was performed using InterProScan v 5.56-89.0 (Jones et al., 2014), Gene Ontology (GO) (Götz et al., 2008), GO-Slim and EggNOG-Mapper 2.1.0 with EggNOG 5.0.2 (Huerta-Cepas et al., 2019) for functional annotation in OmicsBox 2.1.2 (BioBam). The functional annotation results were combined with Refseq (GCF_001970005.1) to construct a functional reference of the whole transcriptome of *P. olivaceus*. Differential gene expression analysis was performed using CLC Genomics Workbench version 11.0 (CLC bio) for formalin and Carpesii fructus extract treatment in liver with p-values below 0.05. Expression patterns of differentially expressed genes (DEGs) were identified using the fold change (FC) value based on up-regulation (FC≥2.0) and down-regulation (FC≤-2.0). In the liver of *P. olivaceus*, 161 and 158 differentially expressed genes were identified in common in all sampling periods after formalin and Carpesii fructus treatment, respectively (Table S3). Using EggNOG, the whole transcriptome of *P. olivaceus* was examined for four functional categories of information, namely ‘information storage and processing’, ‘cellular processes and signalling’, ‘metabolism’ and ‘poorly characterised’. As a result, genes belonging to the ‘signal transduction mechanisms’ category within ‘cellular processes and signaling’ were the most abundant (Figure S1). GO annotation was performed for three categories (biological process; BP, molecular function, MF, cellular component, CC) under the condition of level 7 for each group of DEGs (Figure S2A-C). In addition, 30 DEGs based on RPKM values were clustered using the heatmap function. The heatmap was used to more intuitively show the clustering between specific genes and experimental groups based on RNA-seq DEGs. Blue indicates low gene expression and red indicates high gene expression (Figure 1 B ). In the heat map, specific genes in the liver of *P. olivaceus* showed different expression patterns according to formalin or Carpesii fructus treatment. 30 DEGs included genes involved in some immune responses. The expression patterns of cytochrome P450 1A1 (CYP1A1) and glutathione S-transferase, which are genes involved in phase I (drug oxidation) and phase II (drug conjugation) of drug metabolism, hepcidin, a key regulator of iron homeostasis, and cathepsin L, a gene involved in tumour invasion and metastasis, were different in each treatment group.

To validate the expression pattern of immune-related genes identified in the whole transcriptome data, qRT-PCR was performed. Total RNA in each tissue was normalised and cDNA was synthesised using HyperscriptTM RT Master Mix (Geneall) containing random hexamer and oligo (dT)18. Bet-actin (ACTB; EU090804.1) was used as an internal control gene to correct for the concentration of each sample in qRT-PCR. The qRT-PCR primers were designed from the whole transcriptome reference and the amplification efficiency was confirmed using serially diluted cDNA series from a single sample (Table S4). qRT-PCR was performed using QuantStudioTM 7 Flex Real-time PCR (Applied BiosystemsTM, Waltham, USA) with PowerUPTM SYBRTM Green Master Mix (Applied Biosystems) and gene-specific primers, following 2 amplification steps (45 cycles) at 95 °C for 15 s and 60 °C for 60 s. The Ct value of qRT-PCR was calculated using the delta-delta Ct method and analysed by one-way ANOVA with a significance level of p < 0.05 using IBM SPSS 25.0 software (Livak and Schmittgen, 2001). To validate the expression pattern of the differentially expressed genes (DEGs) identified by RNA-seq, drug metabolism, xenobiotic biodegradation and metabolism-related genes were selected for qRT-PCR analysis. As shown in Figure S3, most of the qRT-PCR results of the analysed genes showed differences in expression levels compared to the high-throughput sequencing data, but the expression patterns were similar. For the validation by qRT-PCR of genes involved in the immune system, including CYP1A1, CYP24A1, CYP25, CYP27B1, UDP-glucuronosyltransferases (UGT) in phase I and II of drug metabolism, and nuclear receptor subfamily 1 group D member 1 (NR1D1), the formalin-treated group tended to have less difference in expression compared to the control group for six genes common to the treatment period. However, the Carpesii fructus extract treated group showed different expression patterns. CYP1A1 and UGT showed a tendency for large expression differences compared to the control group at the beginning of treatment, and CYP24A1 showed a particularly large expression difference in the oral administration group compared to the control group.

In this study, Carpesii fructus extract was selected as a potential therapeutic agent for scuticociliatosis in fish as an alternative to formalin, which is traditionally used for parasitic infections. Although the efficacy of Carpesii fructus extract as a therapeutic agent has recently been reported, there is still a lack of knowledge regarding its physiological response in fish. Therefore, an RNA-seq based study was conducted on the liver of *P. olivaceus* for both Carpesii fructus extract and formalin treatments to obtain whole transcript expression profiles for each time period. Although it was not possible to analyse all functions and mechanisms of whole transcript expression in the liver of *P. olivaceus* for both treatments, the expression patterns of genes related to drug metabolism, xenobiotic biodegradation and metabolism showed slightly different trends between the Carpesii fructus extract and formalin-treated groups. In the formalin-treated group, the selected genes showed a small difference in expression compared to the control group. However, in the Carpesii fructus extract treatment group, a significant difference in the expression of the selected genes was observed depending on the treatment period and method. This difference is probably due to the influence of components present in the Carpesii fructus extract, although not all components could be identified. Therefore, further experiments with different fish are needed to verify the safety and efficacy of Carpesii fructus extract as a therapeutic agent for scuticociliatosis.

The whole-transcriptome raw data and biological sample information used in the present study were submitted to the National Center for Biotechnology Information (NCBI). The Sequence Read Archive (SRA) raw-data can be accessed in NCBI with the following information: BioProject (PRJNA867739), BioSample (SAMN30183003), and SRA (SRR20994465, SRR20994466, SRR20994467, SRR20994468, SRR20994469, SRR20994470, SRR20994471, SRR20994472, SRR20994473, SRR20994474, SRR20994475, SRR20994476, SRR20994477, SRR20994478, SRR20994479, SRR20994480, SRR20994481, SRR20994482, SRR20994483).

## Supplementary material

The following online material is available for this article:

Figure S1 - Classification of EggNOG annotated genes in the *P. olivaceus* genome.

Figure S2 - Gene ontology (GO; to level 7) analysis of DEGs for formalin and Carpesii fructus extract treated groups.

Figure S3 - qRT-PCR validation for drug metabolism, xenobiotic biodegradation and metabolism-related genes.

Table S1 - MIxS descriptions used in this study.

Table S2 - Transcript information excluding duplicated sequences from the *P. olivaceus* genome assembly.

Table S3 - List of commonly expressed genes according to the duration and method of treatment with formalin or Carpesii Fructus.

Table S4 - Primer sequences for qRT-PCR validation of differentially expressed genes in the whole transcriptome assembly.

## References

[B1] Fan Y, Yang X, Ye S, Li H, Li R (2019). Houttuynia cordate Thunb boosts the non-specific immune response
and enhances resistance to Edwardsiellosis in the olive flounder
(Paralichthys olivaceus). Pak J Zool.

[B2] Götz S, García-Gómez JM, Terol J, Williams TD, Nagaraj SH, Nueda MJ, Robles M, Talón M, Dopazo J, Conesa A (2008). High-throughput functional annotation and data mining with the
Blast2GO suite. Nucleic Acids Res.

[B3] Hamidoghli A, Won S, Lee S, Lee S, Farris NW, Bai SC (2020). Nutrition and feeding of olive flounder Paralichthys olivaceus: A
review. Rev Fish Sci Aquac.

[B4] Harikrishnan R, Kim M-C, Kim J-S, Balasundaram C, Heo M-S (2010). Immune enhancement of chemotherapeutants on lymphocystis disease
virus (LDV) infected Paralichthys olivaceus. Fish Shellfish Immunol.

[B5] Huerta-Cepas J, Szklarczyk D, Heller D, Hernández-Plaza A, Forslund SK, Cook H, Mende DR, Letunic I, Rattei T, Jensen LJ (2019). eggNOG 5.0: A hierarchical, functionally and phylogenetically
annotated orthology resource based on 5090 organisms and 2502
viruses. Nucleic Acids Res.

[B6] Jones P, Binns D, Chang H, Fraser M, Li W, McAnulla C, McWilliam H, Maslen J, Mitchell A, Nuka G (2014). InterProScan 5: Genome-scale protein function
classification. Bioinformatics.

[B7] Jung J-Y, Kim S, Kim K, Lee B-J, Kim K-W, Han H-S (2020). Feed and disease at olive flounder (Paralichthys olivaceus) farms
in Korea. Fishes.

[B8] Kang HJ, Kim H, Jeong S-I, Kim HS, Jeon IH, Mok JY, Shim J, Jang SI (2013). Antioxidant and antihemolytic activities of ethanol extract of
Carpesii fructus and Farfarae flos. Korean J Herbology.

[B9] Lee J-H, Park J-J, Choi J-H, Shin D-H, Park KH (2017). Anti-scuticociliate effects of aquatic hydrogen peroxide
preparation in olive flounder Paralichthys olivaceus. J. Fish Dis.

[B10] Liu H, Wu Z, Zhu X, Song Z, Hu J, Wang L, Li J, You F (2018). Comparative performance of growth, vertebral structure and muscle
composition in diploid and triploid Paralichthys olivaceus. J Fish Dis.

[B11] Livak KJ, Schmittgen TD (2001). Analysis of relative gene expression data using real-time
quantitative PCR and the 2(-Delta Delta C(T)) Method. Methods.

[B12] Park S-J, Seo BS, Park HS, Lee B-J, Hur S-W, Nam T-J, Lee K-J, Lee S, Choi YH (2021). Effect of fishmeal content in the diet on the growth and sexual
maturation of olive flounder (Paralichthys olivaceus) at a typical fish
farm. Animals.

[B13] Peng W, Liu Y-J, Wu N, Sun T, He X-Y, Gao Y-X, Wu C-J (2015). Areca catechu L.(Arecaceae): A review of its traditional uses,
botany, phytochemistry, pharmacology and toxicology. J Ethnopharmacol.

[B14] Roh HJ, Kim A, Kang GS, Kim BS, Kim D (2018). Blue light-emitting diode light at 405 and 465 nm can inhibit a
Miamiensis avidus infection in olive flounder, Paralichthys
olivaceus. Aquaculture.

[B15] Song J-Y, Kitamura S, Oh M-J, Kang H-S, Lee J-H, Tanaka S, Jung S-J (2009). Pathogenicity of Miamiensis avidus (syn. Philasterides
dicentrarchi), Pseudocohnilembus persalinus, Pseudocohnilembus hargisi and
Uronema marinum (Ciliophora, Scuticociliatida). Dis Aquat Org.

[B16] Stieglitz JD, Hoenig RH, Baggett JK, Tudela CE, Mathur SK, Benetti DD (2021). Advancing production of marine fish in the United States: Olive
flounder, Paralichthys olivaceus, aquaculture. J World Aquacult Soc.

[B17] Wei X, Xu Z, Wang G, Hou J, Ma X, Liu H, Liu J, Chen B, Luo M, Xie B (2017). pBACode: A random-barcode-based high-throughput approach for BAC
paired-end sequencing and physical clone mapping. Nucleic Acids Res.

[B18] Whang I, Kang H-S, Lee J (2013). Identification of scuticociliates (Pseudocohnilembus persalinus,
P. longisetus, Uronema marinum and Miamiensis avidus) based on the cox1
sequence. Parasitol Int.

[B19] Woo SJ, Jeong MG, Jeon EJ, Do MY, Kim NY (2022). Antiparasitic potential of ethanolic extract of Carpesii Fructus
against Miamiensis avidus in hirame natural embryo cell line and their
effects on immune response- and biotransformation-related
genes. Comp Biochem Physiol C Toxicol Pharmacol.

[B20] Wu X, Xing J, Tang X, Sheng X, Chi H, Zhan W (2022). Protective cellular and humoral immune responses to Edwardsiella
tarda in flounder (Paralichthys olivaceus) immunized by an inactivated
vaccine. Mol Immunol.

[B21] Xia Q, Luo J, Mei X, Wang Y, Huang W, Wang J, Yang R, Ma Z, Lin R (2017). A developmental toxicity assay of Carpesii fructus on zebrafish
embryos/larvae. Toxicol Res (Camb).

[B22] Zhang J, Wang G, Tian X, Yang Y, Liu Q, Chen L, Li H, Zhang W (2015). The genus Carpesium: A review of its ethnopharmacology,
phytochemistry and pharmacology. J Ethnopharmacol.

