# Supplementary material for: Investigating the effects of Carpesii fructus extract on the liver transcriptome of olive flounder (Paralichthys olivaceus) as a potential antiparasitic agent
Source: Genet Mol Biol. 2024 Mar 4;47(1):e20230146. doi: 10.1590/1678-4685-GMB-2023-0146 (PMC10941726; doi:10.1590/1678-4685-GMB-2023-0146)
Supplement: Figure S2 - [file 1415-4757-GMB-47-1-e20230146-s2.pdf]

Genetics and Molecular Biology

https://doi.org/10.1590/1678-4685-GMB-2023-0146

Supplementary Material to “Investigating the effects of Carpesii fructus extract on the liver transcriptome of olive flounder (*Paralichthys olivaceus*) as a potential antiparasitic agent”

(A)

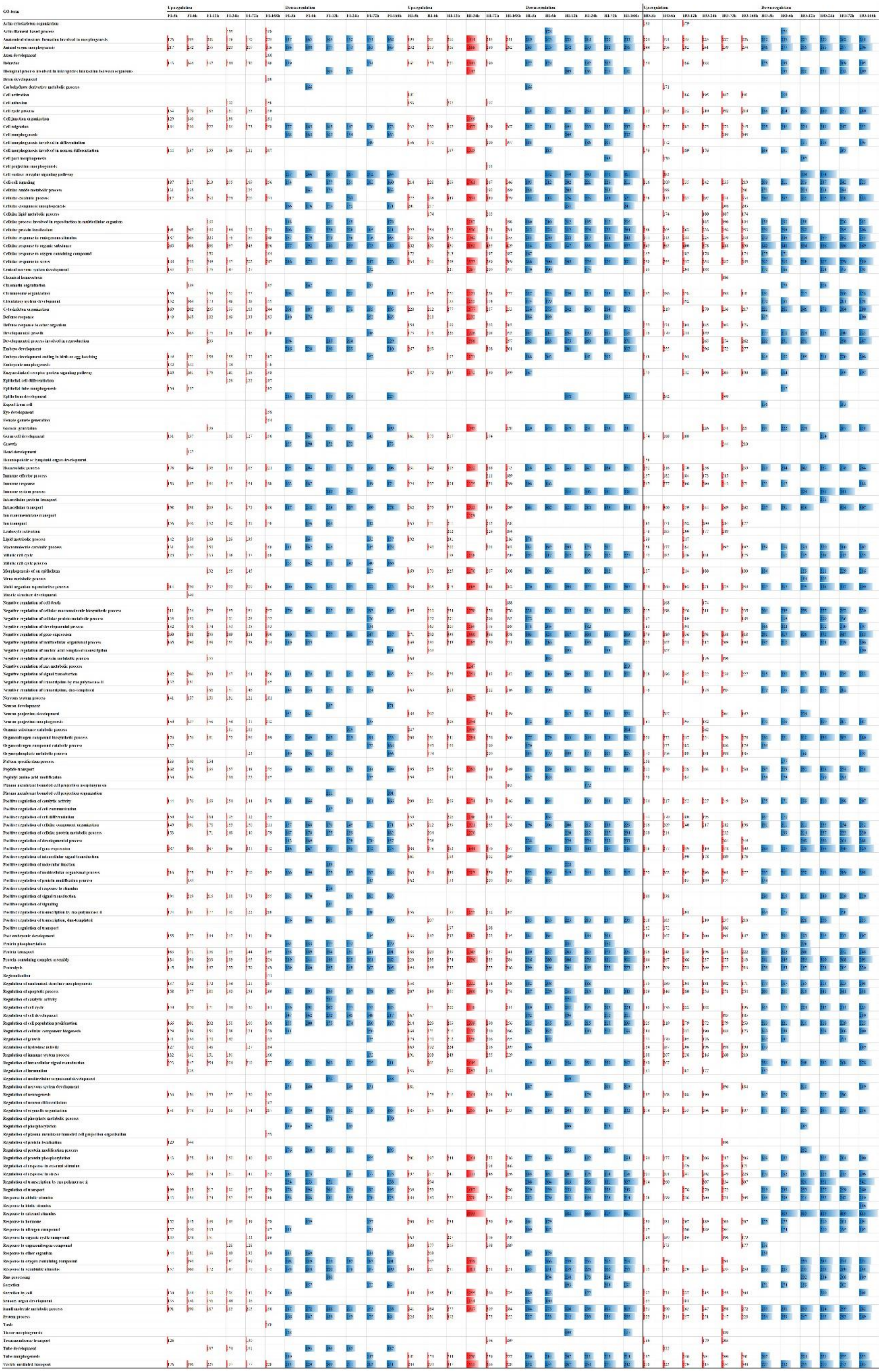

(B)

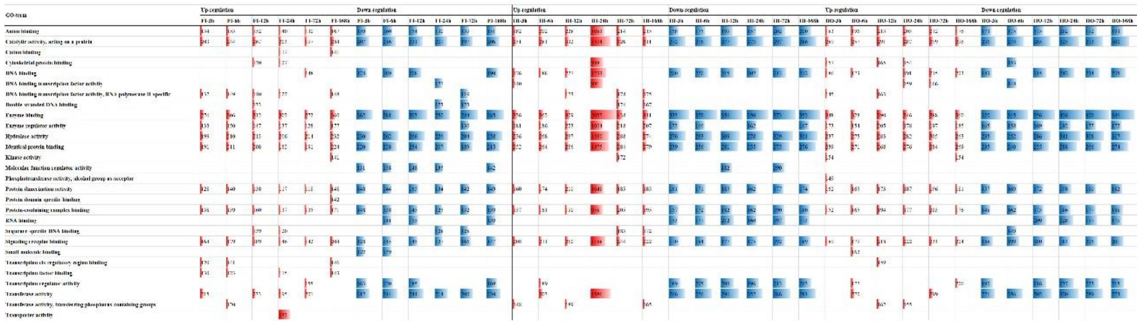

(C)

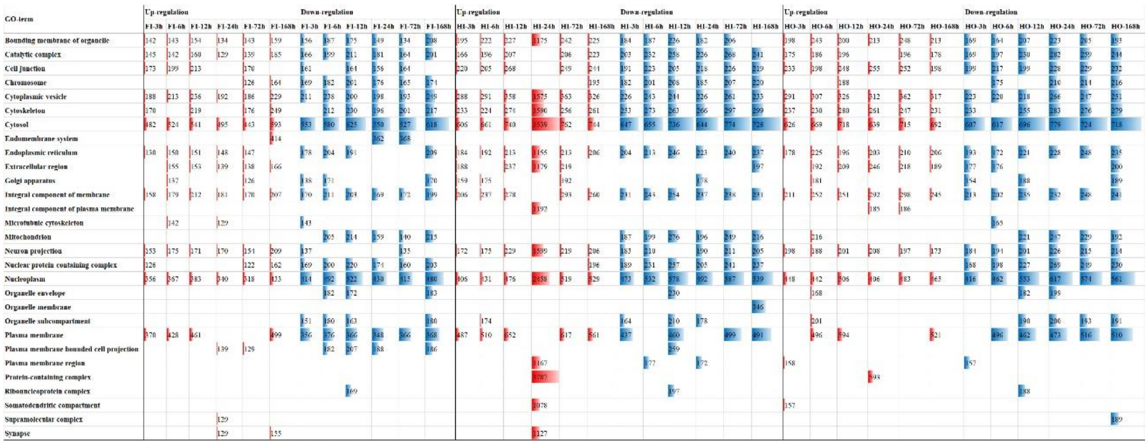

Figure S2 - Gene ontology (GO; to level 7) analysis of DEGs for formalin and Carpesii fructus extract treated groups. GO was categorised into three domains: (A) biological process, (B) molecular function, and (C) cellular component. The x-axis and y-axis represent the number of DEGs and GO terms, respectively.
