# Supplementary material for: Investigating the effects of Carpesii fructus extract on the liver transcriptome of olive flounder (Paralichthys olivaceus) as a potential antiparasitic agent
Source: Genet Mol Biol. 2024 Mar 4;47(1):e20230146. doi: 10.1590/1678-4685-GMB-2023-0146 (PMC10941726; doi:10.1590/1678-4685-GMB-2023-0146)
Supplement: Figure S3 - [file 1415-4757-GMB-47-1-e20230146-s3.pdf]

Genetics and Molecular Biology

https://doi.org/10.1590/1678-4685-GMB-2023-0146

Supplementary Material to "Investigating the effects of Carpesii fructus extract on the liver transcriptome of olive flounder (*Paralichthys olivaceus*) as a potential antiparasitic agent"

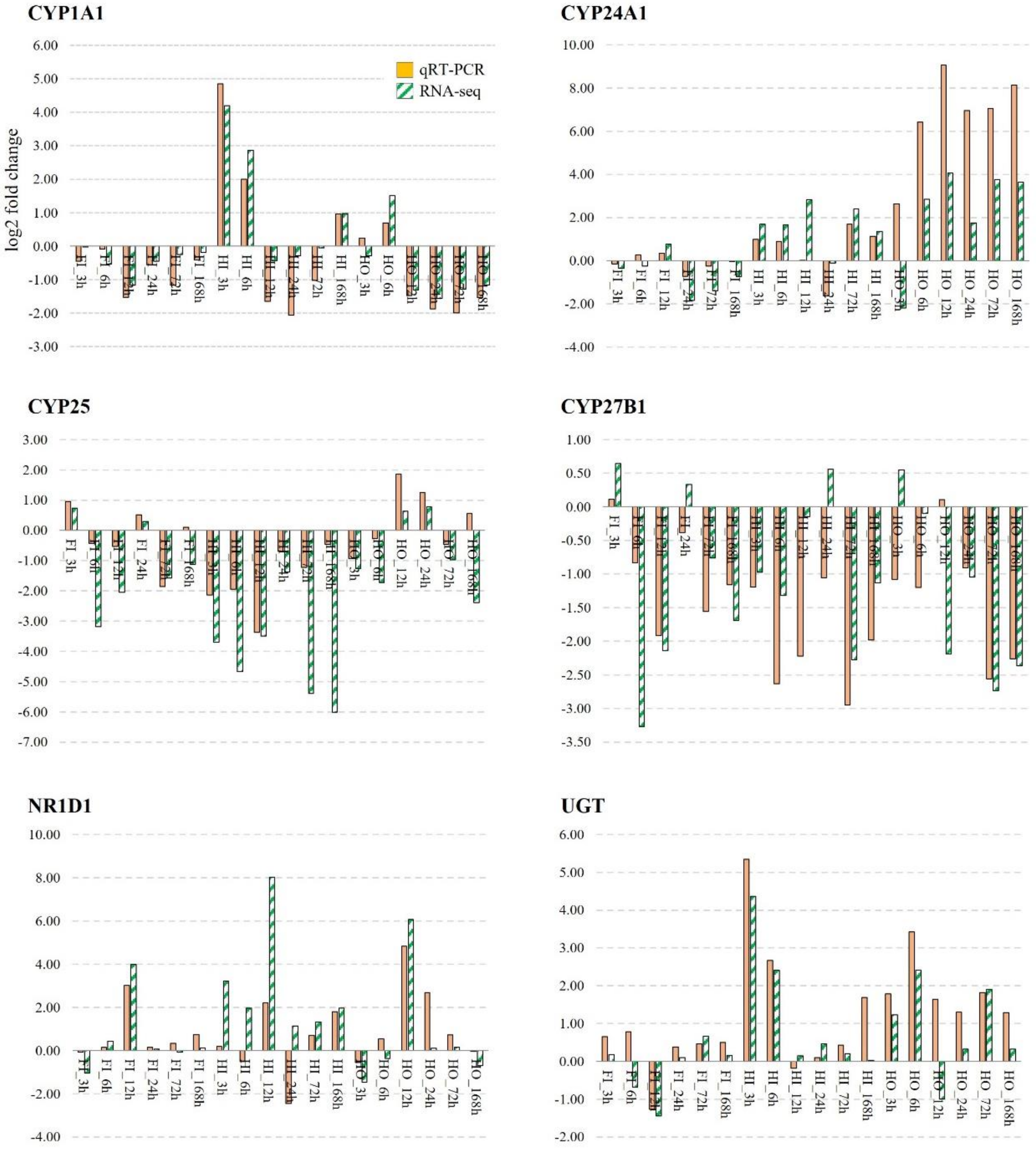

Figure S3 - qRT-PCR validation for drug metabolism, xenobiotic biodegradation and metabolism-related genes. Comparison of log2 fold change values of 6 differentially expressed genes in *P. olivaceus* liver after formalin and Carpesii fructus treatment between qRT-PCR and RNA-Seq.
