# Supplementary material for: Investigating the effects of Carpesii fructus extract on the liver transcriptome of olive flounder (Paralichthys olivaceus) as a potential antiparasitic agent
Source: Genet Mol Biol. 2024 Mar 4;47(1):e20230146. doi: 10.1590/1678-4685-GMB-2023-0146 (PMC10941726; doi:10.1590/1678-4685-GMB-2023-0146)
Supplement: Table S1 - [file 1415-4757-GMB-47-1-e20230146-s4.pdf]

Genetics and Molecular Biology

https://doi.org/10.1590/1678-4685-GMB-2023-0146

Supplementary Material to "Investigating the effects of Carpesii fructus extract on the liver transcriptome of olive flounder (*Paralichthys olivaceus*) as a potential antiparasitic agent"

Table S1- MIxS descriptions used in this study.

Item	Description
Investigation_type	Eukaryote
Project_name	Bioproject: PRJNA867739, Biosample: SAMN30183003
Submitted to SRA	SRR20994465, SRR20994466, SRR20994467, SRR20994468, SRR20994469, SRR20994470, SRR20994471, SRR20994472, SRR20994473, SRR20994474, SRR20994475, SRR20994476, SRR20994477, SRR20994478, SRR20994479, SRR20994480, SRR20994481, SRR20994482, SRR20994483
Latitude_longitude	latitude: 36.9848180; longitude: 126.3755814
Country	Republic of Korea
Geolocation_name	Seosan-si, Chungcheongnam-do
Collection_date	March-2020
Environment	Marine (Sea water)
Env_biome	ENVO:00000447 (marine biome)
Env_feature	ENVO:01001254 (animal aquaculture process)
Env_material	ENVO:00001999 (marine water body)
Seq_method	MGISEQ-2000, MGIEasy RNA Directional Library Prep Kit
Genome reference	GCF_001970005.1
Mapping method	CLC Genomics Workbench 11.0
