# Supplementary material for: Investigating the effects of Carpesii fructus extract on the liver transcriptome of olive flounder (Paralichthys olivaceus) as a potential antiparasitic agent
Source: Genet Mol Biol. 2024 Mar 4;47(1):e20230146. doi: 10.1590/1678-4685-GMB-2023-0146 (PMC10941726; doi:10.1590/1678-4685-GMB-2023-0146)
Supplement: Table S2 - [file 1415-4757-GMB-47-1-e20230146-s5.pdf]

Genetics and Molecular Biology
https://doi.org/10.1590/1678-4685-GMB-2023-0146

Supplementary Material to "Investigating the effects of Carpesii fructus extract on the liver transcriptome of olive flounder (*Paralichthys olivaceus*) as a potential antiparasitic agent"

Table S2 - POL transcriptome references.

SeqName	Description	Length	e-Value
XM_020077987.1	protocadherin gamma-C5-like isoform X4	5657	0
XM_020077988.1	PREDICTED: uncharacterized protein LOC109648310	1725	9.44E-151
XM_020077989.1	receptor-type tyrosine-protein phosphatase mu-like	742	7.65E-172
XM_020077990.1	serine/threonine-protein kinase SBK1-like	1004	4.25E-153
XM_020077991.1	major facilitator superfamily domain-containing protein 6-A-like	936	3.52E-178
XM_020077992.1	transcription factor SOX-6-like isoform X1	5182	0
XM_020077993.1	probable RNA-binding protein 23	478	7.10E-77
XM_020077994.1	transcription factor SOX-6-like isoform X2	394	8.86E-83
XM_020077995.1	diacylglycerol kinase zeta-like	488	1.39E-81
XM_020077996.1	zinc transporter ZIP10-like	1010	3.57E-97
XM_020077997.1	adenylate cyclase type 3-like	271	5.62E-62
XM_020077998.1	transcription factor SOX-6-like isoform X2	5179	0
XM_020077999.1	neuroblast differentiation-associated protein AHNAK-like	1769	0
XM_020078000.1	NADH-cytochrome b5 reductase 3-like	493	1.08E-119
XM_020078001.1	misshapen-like kinase 1	645	4.94E-61
XM_020078002.1	chromodomain-helicase-DNA-binding protein 5-like	321	4.91E-62
XM_020078003.1	inactive ubiquitin carboxyl-terminal hydrolase 53-like	840	1.28E-171
XM_020078004.1	ribosome-binding protein 1-like	345	2.05E-74
XM_020078005.1	macrophage mannose receptor 1-like	569	2.51E-103
XM_020078006.1	serum response factor-like	730	6.77E-61
XM_020078007.1	EMILIN-1-like isoform X1	709	3.92E-100
XM_020078008.1	pre-mRNA-splicing factor 18 isoform X1	1913	0
XM_020078009.1	homeodomain-interacting protein kinase 2-like	1033	2.55E-115
XM_020078010.1	PREDICTED: calpain-7-like, partial	301	5.61E-71
XM_020078011.1	laminin subunit alpha-5-like	552	2.58E-116
XM_020078012.1	microtubule-actin cross-linking factor 1-like	849	5.41E-170
XM_020078013.1	polypyrimidine tract-binding protein 3-like	565	2.02E-114
XM_020078014.1	steroid hormone receptor ERR2-like isoform X2	793	0
XM_020078015.1	pre-mRNA-splicing factor 18 isoform X2	1886	0
XM_020078016.1	cohesin subunit SA-2-like	384	1.02E-60
XM_020078017.1	PREDICTED: bestrophin-1-like, partial	562	7.35E-140
XM_020078018.1	homeobox protein Nkx-2.5-like	1134	3.60E-104
XM_020078019.1	LARGE xylosyl- and glucuronyltransferase 2-like	390	1.34E-93
XM_020078020.1	epidermal growth factor receptor kinase substrate 8-like	602	1.23E-89
XM_020078021.1	protein AF1q	418	1.38E-55
XM_020078022.1	lipase member H	921	4.18E-80
XM_020078023.1	solute carrier family 12 member 3-like	608	2.21E-97
XM_020078024.1	EH domain-containing protein 4-like	301	8.35E-70
XM_020078025.1	transmembrane protein 138	1591	5.74E-94
XM_020078026.1	rho GTPase-activating protein 18-like	717	4.22E-133
XM_020078027.1	histone H4	269	1.99E-39
XM_020078028.1	histone H2A-like	263	2.14E-33
XM_020078029.1	perilipin-2-like isoform X2	417	6.52E-64
XM_020078030.1	mediator of RNA polymerase II transcription subunit 15-like	642	9.24E-135
XM_020078031.1	caspase recruitment domain-containing protein 11-like	356	1.57E-70
XM_020078032.1	HSPB1-associated protein 1 homolog	1156	5.64E-125
XM_020078033.1	transmembrane protein 138	1601	6.26E-94
XM_020078034.1	protein NLRC3	907	1.11E-130
XM_020078035.1	beta-adrenergic receptor kinase 2-like	282	2.57E-65
XM_020078036.1	PREDICTED: gamma-adducin-like	729	1.79E-40
XM_020078037.1	hydroxysteroid dehydrogenase-like protein 2	575	1.79E-88
XM_020078038.1	ELM2 and SANT domain-containing protein 1-like	874	0
XM_020078039.1	nuclear transcription factor Y subunit beta	1057	2.81E-78
XM_020078040.1	methylmalonic aciduria and homocystinuria type D homolog, mitochondrial-like	510	1.54E-99
XM_020078041.1	transmembrane protein 138	1515	2.71E-94
XM_020078042.1	25-hydroxycholesterol 7-alpha-hydroxylase-like	970	6.07E-152
XM_020078043.1	eukaryotic translation initiation factor 4H-like	456	1.11E-76
XM_020078044.1	ATP-binding cassette sub-family A member 1-like	660	9.72E-141
XM_020078045.1	WD repeat-containing protein 34-like	974	3.67E-146
XM_020078046.1	inactive ubiquitin carboxyl-terminal hydrolase 53-like	425	1.16E-103
XM_020078047.1	programmed cell death 6-interacting protein-like	753	4.54E-117
XM_020078048.1	plexin-A1 isoform X1	492	9.93E-69
XM_020078049.1	RNA polymerase II degradation factor 1-like	576	1.68E-86
XM_020078050.1	PREDICTED: uncharacterized protein LOC109623555	936	0
XM_020078051.1	ubinuclein-2-like isoform X1	1165	5.84E-142
XM_020078052.1	transmembrane protein 258	500	1.91E-37
XM_020078053.1	ATP-binding cassette sub-family E member 1-like	465	4.74E-85
XM_020078054.1	mediator of RNA polymerase II transcription subunit 16-like	914	0
XM_020078055.1	prosaposin receptor GPR37L1-like	1214	1.03E-140
XM_020078056.1	activin receptor type-2B-like	1316	4.11E-74
XM_020078057.1	activin receptor type-2B-like	944	9.09E-76
XM_020078058.1	activin receptor type-2B-like	948	9.72E-76
XM_020078059.1	CREB-regulated transcription coactivator 3-like	413	1.30E-39
XM_020078060.1	coagulation factor X-like	515	8.38E-80
XM_020078061.1	growth arrest-specific protein 8-like	468	2.54E-81
XM_020078062.1	5-hydroxyisourate hydrolase-like	1520	1.06E-79
XM_020078063.1	tubulin polyglutamylase ttll6-like	801	0
XM_020078064.1	APOBEC1 complementation factor-like	705	5.09E-169

XM_020078065.1	ERI1 exoribonuclease 2-like	1526	0
XM_020078066.1	rho GTPase-activating protein 44-like	675	1.77E-139
XM_020078067.1	COMM domain-containing protein 1	1000	4.55E-78
XM_020078068.1	protein IWS1 homolog	642	2.17E-129
XM_020078069.1	BMP-binding endothelial regulator protein-like	257	3.53E-42
XM_020078070.1	zinc finger protein aebp2-like	2509	0
XM_020078071.1	wiskott-Aldrich syndrome protein family member 3-like	541	1.32E-134
XM_020078072.1	breast cancer anti-estrogen resistance protein 3-like isoform X3	473	4.85E-117
XM_020078073.1	potassium voltage-gated channel subfamily G member 2-like	1162	1.34E-177
XM_020078074.1	inositol 1,4,5-trisphosphate receptor type 1-like	261	4.00E-59
XM_020078075.1	ubiquitin-conjugating enzyme E2 G1-like	286	2.12E-45
XM_020078076.1	atrial natriuretic peptide-converting enzyme-like	570	2.01E-107
XM_020078077.1	PREDICTED: natterin-3-like	1295	0
XM_020078078.1	zinc finger protein 467-like	540	8.70E-60
XM_020078079.1	fatty acid desaturase 2	2088	0
XM_020078080.1	protein FAM184A-like	350	8.07E-54
XM_020078081.1	PREDICTED: kalirin-like, partial	702	2.28E-174
XM_020078082.1	arylsulfatase B-like	658	2.31E-115
XM_020078083.1	GSK-3-binding protein FRAT2	1126	3.29E-94
XM_020078084.1	complement component C6-like	791	1.18E-129
XM_020078085.1	methyl-CpG-binding domain protein 2-like	482	1.78E-115
XM_020078086.1	MHC class II regulatory factor RFX1-like	705	1.98E-106
XM_020078087.1	LYR motif-containing protein 4	159	2.21E-33
XM_020078088.1	laminin subunit beta-1-like	947	9.43E-170
XM_020078089.1	protocadherin gamma-A2-like	2658	0
XM_020078090.1	homeobox protein aristaless-like 4	1525	0
XM_020078091.1	homeobox protein Hox-B6b	919	3.04E-94
XM_020078092.1	kelch-like protein 17 isoform X1	519	4.09E-129
XM_020078093.1	dynein heavy chain 10, axonemal-like	1092	0
XM_020078094.1	vacuolar protein sorting-associated protein 13C-like	381	3.41E-58
XM_020078095.1	iroquois-class homeodomain protein IRX-5-like	999	0
XM_020078096.1	protein NipSnap homolog 3B-like	864	2.81E-176
XM_020078097.1	BUB3-interacting and GLEBS motif-containing protein ZNF207-like	713	5.01E-79
XM_020078098.1	WD40 repeat-containing protein SMU1-like	696	1.18E-152
XM_020078099.1	chloride channel CLIC-like protein 1	433	2.81E-84
XM_020078100.1	malonyl-CoA decarboxylase, mitochondrial	3220	0
XM_020078101.1	putative polypeptide N-acetylgalactosaminyltransferase-like protein 3	658	3.91E-162
XM_020078102.1	receptor-type tyrosine-protein phosphatase gamma-like	1168	2.90E-80
XM_020078103.1	steroid 17-alpha-hydroxylase/17,20 lyase-like	538	2.44E-91
XM_020078104.1	A-kinase anchor protein 2-like	974	4.71E-157
XM_020078105.1	gamma-soluble NSF attachment protein-like	459	3.62E-111
XM_020078106.1	homeobox protein Hox-C8a-like	990	1.03E-140
XM_020078107.1	DDB1- and CUL4-associated factor 6-like	326	4.57E-61
XM_020078108.1	disintegrin and metalloproteinase domain-containing protein 17-like	1420	1.55E-80
XM_020078109.1	protein Wnt-7b isoform X1	2066	0
XM_020078110.1	zinc transporter 6-like	372	1.85E-70
XM_020078111.1	probable ATP-dependent RNA helicase DDX5	1337	3.19E-68
XM_020078112.1	integrator complex subunit 11-like	345	9.82E-71
XM_020078113.1	dual specificity phosphatase DUPD1	479	1.63E-117
XM_020078114.1	laminin subunit alpha-5-like	502	2.53E-124
XM_020078115.1	protein Wnt-7b isoform X2	1891	0
XM_020078116.1	PREDICTED: urocortin-3	941	3.21E-93
XM_020078117.1	1110055E19Rik protein	651	8.26E-159
XM_020078118.1	PREDICTED: extensin-like, partial	533	4.83E-63
XM_020078119.1	filamin-C-like isoform X2	739	0
XM_020078120.1	dynein heavy chain 5, axonemal-like	569	3.21E-120
XM_020078121.1	sorting nexin-2-like	483	1.48E-79
XM_020078122.1	gastrula zinc finger protein XlCGF49.1-like	1567	1.54E-161
XM_020078123.1	anaphase-promoting complex subunit 10	805	1.33E-134
XM_020078124.1	ETS domain-containing protein Elk-3-like	2598	0
XM_020078125.1	kelch-like protein 7	418	1.05E-101
XM_020078126.1	chloride intracellular channel protein 2-like	783	2.00E-85
XM_020078127.1	membrane-associated phosphatidylinositol transfer protein 2-like	311	5.36E-74
XM_020078128.1	ectonucleotide pyrophosphatase/phosphodiesterase family member 2-like	735	3.96E-165
XM_020078129.1	olfactory receptor 4S1-like	936	0
XM_020078130.1	transmembrane O-methyltransferase-like	660	1.74E-119
XM_020078131.1	TCF3 fusion partner homolog	225	5.99E-26
XM_020078132.1	protein kinase C-binding protein 1-like	1023	1.69E-130
XM_020078133.1	regulator of G-protein signaling 13-like	1078	5.71E-113
XM_020078134.1	solute carrier family 25 member 44-like	2572	0
XM_020078135.1	translation factor GUF1, mitochondrial-like	513	2.45E-99
XM_020078136.1	claudin-15-like isoform X2	505	2.67E-52
XM_020078137.1	multiple epidermal growth factor-like domains protein 10	527	5.95E-110
XM_020078138.1	dual specificity protein phosphatase 14-like	1117	1.05E-124
XM_020078139.1	putative E3 ubiquitin-protein ligase UBR7	678	4.63E-134
XM_020078140.1	tumor protein p53-inducible nuclear protein 1	526	2.12E-98
XM_020078141.1	neuropilin and tolloid-like protein 2	2145	0
XM_020078142.1	ras-related C3 botulinum toxin substrate 1-like	272	2.83E-63

XM_020078143.1	PREDICTED: uncharacterized protein LOC109623661	1088	0
XM_020078144.1	arginyl aminopeptidase-like 1	760	0
XM_020078145.1	discoidin domain-containing receptor 2-like	350	2.58E-36
XM_020078146.1	gamma-aminobutyric acid receptor subunit rho-2-like	359	1.03E-73
XM_020078147.1	vesicle-fusing ATPase-like	462	1.83E-89
XM_020078148.1	kinetochore protein Nuf2-like	842	5.66E-73
XM_020078149.1	BTB/POZ domain-containing protein KCTD9	583	3.92E-116
XM_020078150.1	retinol dehydrogenase 11-like	704	3.02E-170
XM_020078151.1	phosphatidylinositol 4-phosphate 3-kinase C2 domain-containing subunit alpha	5593	0
XM_020078152.1	E3 ubiquitin-protein ligase RNF123-like	429	2.86E-106
XM_020078153.1	spindle assembly checkpoint kinase-like	969	0
XM_020078154.1	nuclear pore complex protein Nup98-Nup96-like	1034	9.77E-77
XM_020078155.1	multidrug resistance-associated protein 1-like	821	0
XM_020078156.1	fer3-like protein	1090	4.44E-112
XM_020078157.1	inosine-5'-monophosphate dehydrogenase 1a-like	682	2.50E-168
XM_020078158.1	phosphatidylinositol 4-phosphate 3-kinase C2 domain-containing subunit alpha	4809	0
XM_020078159.1	ectonucleotide pyrophosphatase/phosphodiesterase family member 5-like	984	0
XM_020078160.1	nephronectin isoform X2	780	6.67E-107
XM_020078161.1	rho GTPase-activating protein 28-like	514	2.67E-80
XM_020078162.1	retinoblastoma-like protein 2	442	8.50E-92
XM_020078163.1	PREDICTED: tetraspanin-12-like	1277	3.20E-83
XM_020078164.1	L-fucose kinase-like	649	1.95E-87
XM_020078165.1	tensin-1 isoform X4	432	4.92E-79
XM_020078166.1	elongation factor 1-beta-like	578	3.36E-67
XM_020078167.1	transmembrane protein 160	469	8.08E-67
XM_020078168.1	meiosis-specific nuclear structural protein 1-like	927	0
XM_020078169.1	PREDICTED: tetraspanin-18-like	1990	1.97E-128
XM_020078170.1	PREDICTED: trichohyalin-like, partial	982	3.42E-170
XM_020078171.1	protein patched homolog 1-like	794	0
XM_020078172.1	complement C5-like	706	2.03E-175
XM_020078173.1	PREDICTED: uncharacterized protein LOC109623692	1151	1.13E-85
XM_020078174.1	neuroblast differentiation-associated protein AHNAK-like	1402	0
XM_020078175.1	coiled-coil domain-containing protein 186-like	1365	0
XM_020078176.1	microtubule-actin cross-linking factor 1-like	663	1.32E-147
XM_020078177.1	post-GPI attachment to proteins factor 2-like	557	1.33E-120
XM_020078178.1	vacuolar protein sorting-associated protein 13C-like	672	2.47E-143
XM_020078179.1	traf2 and NCK-interacting protein kinase-like	414	4.58E-101
XM_020078180.1	ammonium transporter Rh type C	2299	0
XM_020078181.1	dynactin subunit 1-like	739	3.53E-174
XM_020078182.1	calcium-activated potassium channel subunit beta-2-like	456	4.43E-91
XM_020078183.1	coagulation factor XIII A chain	470	2.53E-91
XM_020078184.1	interferon regulatory factor 2-binding protein 2-B-like	1105	4.19E-172
XM_020078185.1	neuronal acetylcholine receptor subunit non-alpha-3-like	648	5.04E-145
XM_020078186.1	protocadherin gamma-A3-like	2536	0
XM_020078187.1	nuclear factor NF-kappa-B p100 subunit-like	575	1.76E-140
XM_020078188.1	ectonucleotide pyrophosphatase/phosphodiesterase family member 2-like	561	1.86E-126
XM_020078189.1	cohesin subunit SA-2-like	740	7.52E-143
XM_020078190.1	laminin subunit beta-2-like	443	3.71E-105
XM_020078191.1	UPF0428 protein CXorf56 homolog	1148	5.41E-138
XM_020078192.1	polyhomeotic-like protein 2	552	1.54E-80
XM_020078193.1	phosphatidylinositol 3-kinase regulatory subunit alpha-like	610	1.03E-58
XM_020078194.1	nuclear receptor coactivator 3-like	276	2.80E-62
XM_020078195.1	thrombospondin-type laminin G domain and EAR repeat-containing protein-like	634	2.46E-108
XM_020078196.1	probable ATP-dependent RNA helicase DDX31	663	9.17E-95
XM_020078197.1	laminin subunit alpha-4-like	531	4.11E-66
XM_020078198.1	sphingomyelin phosphodiesterase 5-like isoform X1	4940	0
XM_020078199.1	echinoderm microtubule-associated protein-like 6	628	1.50E-129
XM_020078200.1	beta-arrestin-2 isoform X2	404	2.08E-75
XM_020078201.1	major histocompatibility complex (MHC) class Ia chain	598	1.50E-140
XM_020078202.1	WD repeat- and FYVE domain-containing protein 4-like	636	9.76E-152
XM_020078203.1	ankyrin-1 isoform X3	421	1.56E-99
XM_020078204.1	pre-mRNA-splicing factor 38A-like isoform X1	642	2.55E-39
XM_020078205.1	pre-mRNA-splicing factor 38A-like isoform X2	639	1.65E-39
XM_020078206.1	pre-mRNA-splicing factor 38A-like isoform X3	555	3.29E-23
XM_020078207.1	pre-mRNA-splicing factor 38A-like isoform X4	552	2.15E-23
XM_020078208.1	PREDICTED: semaphorin-7A	3967	0
XM_020078209.1	Williams-Beuren syndrome chromosomal region 27 protein-like	630	1.24E-138
XM_020078210.1	protein NLRC3-like	1278	0
XM_020078211.1	aminopeptidase B-like	877	4.52E-159
XM_020078212.1	aminopeptidase B-like	860	2.93E-159
XM_020078213.1	syntaxin-binding protein 4-like	381	5.79E-77
XM_020078214.1	ubiquitin-fold modifier-conjugating enzyme 1	955	4.78E-117
XM_020078215.1	vacuolar protein sorting-associated protein 8 homolog	407	8.01E-98
XM_020078216.1	alanine--tRNA ligase, cytoplasmic-like	566	4.57E-108
XM_020078217.1	F-box/LRR-repeat protein 16-like	3296	0
XM_020078218.1	perilipin-2-like isoform X2	420	1.46E-100
XM_020078219.1	phospholipase D3-like	531	4.21E-115
XM_020078220.1	leucine-rich repeat flightless-interacting protein 1-like	499	4.47E-82
XM_020078221.1	sestrin-1 isoform X2	634	1.21E-137

XM_020078222.1	potassium voltage-gated channel subfamily E member 4	1327	1.50E-105
XM_020078223.1	PREDICTED: uncharacterized protein LOC109623749, partial	1155	9.96E-74
XM_020078224.1	transcription cofactor vestigial-like protein 2	1037	1.33E-89
XM_020078225.1	zinc finger protein 710 isoform X1	2367	0
XM_020078226.1	receptor-type tyrosine-protein phosphatase mu-like	441	2.92E-109
XM_020078227.1	transmembrane protein 53-A-like isoform X3	1212	0
XM_020078228.1	inositol 1,4,5-trisphosphate receptor type 1-like	790	2.50E-177
XM_020078229.1	Krueppel-like factor 13	1274	2.75E-100
XM_020078230.1	extracellular sulfatase Sulf-2 isoform X1	324	1.98E-66
XM_020078231.1	serine/threonine-protein kinase N2-like	706	2.78E-147
XM_020078232.1	zinc finger protein 710 isoform X2	2337	0
XM_020078233.1	formin-binding protein 1-like	435	1.29E-68
XM_020078234.1	2,4-dienoyl-CoA reductase, mitochondrial-like	618	5.09E-137
XM_020078235.1	zinc finger and BTB domain-containing protein 14	494	7.91E-121
XM_020078236.1	synembryn-B isoform X2	457	5.03E-59
XM_020078237.1	protein shisa-7-like	501	3.92E-121
XM_020078238.1	3-ketoacyl-CoA thiolase, peroxisomal-like	592	2.84E-63
XM_020078239.1	arginase-2, mitochondrial	266	2.27E-59
XM_020078240.1	PREDICTED: spatacsin-like	364	4.88E-77
XM_020078241.1	ras GTPase-activating protein 2-like	390	4.76E-71
XM_020078242.1	carnitine O-acetyltransferase-like	956	3.86E-89
XM_020078243.1	MOB kinase activator 2-like	1543	1.48E-131
XM_020078244.1	zinc finger protein OZF-like isoform X2	334	1.05E-78
XM_020078245.1	PREDICTED: kalirin-like, partial	748	2.40E-158
XM_020078246.1	myosin heavy chain, cardiac muscle isoform-like	509	6.70E-101
XM_020078247.1	latent-transforming growth factor beta-binding protein 1-like	599	2.32E-126
XM_020078248.1	serine/threonine-protein phosphatase with EF-hands 2-like	491	5.78E-103
XM_020078249.1	spectrin alpha chain, non-erythrocytic 1-like	715	1.43E-175
XM_020078250.1	E3 ubiquitin-protein ligase ARIH2	520	1.85E-59
XM_020078251.1	xanthine dehydrogenase/oxidase-like	682	1.49E-93
XM_020078252.1	serine/threonine-protein phosphatase 6 regulatory ankyrin repeat subunit B-like	391	2.23E-85
XM_020078253.1	mitochondrial intermediate peptidase	710	6.22E-168
XM_020078254.1	protein FAM60A-like isoform X1	1968	3.07E-142
XM_020078255.1	nuclear receptor coactivator 2 isoform X1	547	1.75E-47
XM_020078256.1	immunoglobulin-binding protein 1-like	500	3.96E-101
XM_020078257.1	E3 ubiquitin-protein ligase HECTD3-like	801	1.43E-68
XM_020078258.1	transmembrane channel-like protein 7 isoform X1	596	1.83E-88
XM_020078259.1	transmembrane channel-like protein 8 isoform X2	591	1.64E-67
XM_020078260.1	brefeldin A-inhibited guanine nucleotide-exchange protein 3-like	502	7.98E-121
XM_020078261.1	phosphatidylinositol 4-phosphate 3-kinase C2 domain-containing subunit alpha	634	1.52E-144
XM_020078262.1	voltage-dependent L-type calcium channel subunit alpha-1D-like	502	2.23E-123
XM_020078263.1	histone-lysine N-methyltransferase 2C-like	472	1.02E-102
XM_020078264.1	transmembrane protein 263-like	5524	3.96E-70
XM_020078265.1	vacuolar protein sorting-associated protein 13A-like	555	5.41E-64
XM_020078266.1	SH3 domain-containing protein 19-like	718	2.09E-148
XM_020078267.1	DNA mismatch repair protein Msh2-like	528	1.48E-87
XM_020078268.1	actin-binding LIM protein 1-like	323	4.47E-75
XM_020078269.1	rab effector MyRIP-like	525	1.53E-85
XM_020078270.1	rho GTPase-activating protein 23-like	266	3.11E-63
XM_020078271.1	PREDICTED: opsin-5-like	841	4.34E-110
XM_020078272.1	zinc finger C3H1 domain-containing protein-like	411	1.00E-60
XM_020078273.1	60S ribosomal protein L27a	607	5.41E-91
XM_020078274.1	sodium/potassium/calcium exchanger 3-like	483	1.88E-111
XM_020078275.1	transcription factor 4-like	288	4.32E-43
XM_020078276.1	nuclear pore complex protein Nup133-like	470	2.71E-58
XM_020078277.1	NAD(P) transhydrogenase, mitochondrial-like	607	1.71E-121
XM_020078278.1	F-box only protein 41-like	681	1.52E-147
XM_020078279.1	laminin subunit alpha-5-like	583	3.84E-131
XM_020078280.1	dehydrogenase/reductase SDR family member 12-like isoform X2	409	1.52E-81
XM_020078281.1	39S ribosomal protein L40, mitochondrial-like	504	2.34E-50
XM_020078282.1	extracellular serine/threonine protein kinase FAM20C-like	511	4.96E-106
XM_020078283.1	protocadherin alpha-5-like	2385	0
XM_020078284.1	RNA helicase Mov10l1 isoform X1	3779	0
XM_020078285.1	serine/threonine-protein phosphatase 4 regulatory subunit 4-like	340	1.17E-78
XM_020078286.1	arf-GAP with coiled-coil, ANK repeat and PH domain-containing protein 2-like	715	2.78E-69
XM_020078287.1	acidic leucine-rich nuclear phosphoprotein 32 family member E-like	414	5.93E-53
XM_020078288.1	extended synaptotagmin-1-like	437	4.91E-90
XM_020078289.1	inaD-like protein	483	2.47E-58
XM_020078290.1	RNA helicase Mov10l1 isoform X1	3796	0
XM_020078291.1	multidrug resistance-associated protein 4-like	469	3.21E-83
XM_020078292.1	sorting nexin-13-like	536	1.36E-104
XM_020078293.1	xanthine dehydrogenase/oxidase-like	616	1.73E-134
XM_020078294.1	ATP-binding cassette sub-family E member 1-like	457	6.45E-104
XM_020078295.1	ribonuclease kappa	881	1.66E-37
XM_020078296.1	keratin, type I cytoskeletal 50 kDa-like	491	3.77E-49
XM_020078297.1	vacuolar protein sorting-associated protein 13C-like	550	1.41E-132
XM_020078298.1	RNA helicase Mov10l1 isoform X1	3773	0
XM_020078299.1	PREDICTED: uncharacterized protein LOC109623829, partial	731	2.76E-151
XM_020078300.1	PREDICTED: beta-taxilin-like	695	1.31E-119

XM_020078301.1	beta-1,4-glucuronyltransferase 1	1189	0
XM_020078302.1	RIB43A-like with coiled-coils protein 2	548	1.45E-106
XM_020078303.1	BTB/POZ domain-containing protein KCTD14	928	2.54E-171
XM_020078304.1	semaphorin-3G isoform X2	388	2.47E-84
XM_020078305.1	translation initiation factor IF-2, mitochondrial-like	503	6.74E-102
XM_020078306.1	calmodulin-binding transcription activator 1-like	453	1.33E-109
XM_020078307.1	multiple C2 and transmembrane domain-containing protein 2-like isoform X1	4207	0
XM_020078308.1	E3 ubiquitin-protein ligase Siah2-like	1067	2.76E-71
XM_020078309.1	phospholipase D1B	455	1.57E-81
XM_020078310.1	synaptosomal-associated protein 25-like	292	3.16E-67
XM_020078311.1	ubiquitin carboxyl-terminal hydrolase MINDY-1-like	575	2.59E-46
XM_020078312.1	transmembrane protein 244-like	591	1.26E-108

XM_020078313.1	receptor-type tyrosine-protein phosphatase O-like	595	7.84E-146
XM_020078314.1	multiple C2 and transmembrane domain-containing protein 2-like isoform X1	4158	0
XM_020078315.1	CD59 glycoprotein-like	482	5.19E-58
XM_020078316.1	ankyrin repeat and fibronectin type-III domain-containing protein 1-like	562	1.62E-117
XM_020078317.1	protein IWS1 homolog	315	9.57E-51
XM_020078318.1	NEDD4-binding protein 3 homolog	3403	0
XM_020078319.1	PREDICTED: otopetrin-1-like, partial	905	0
XM_020078320.1	switch-associated protein 70-like	512	2.77E-84
XM_020078321.1	NAD kinase 2, mitochondrial-like	824	1.41E-71
XM_020078322.1	multiple C2 and transmembrane domain-containing protein 2-like isoform X1	4119	0
XM_020078323.1	putative helicase mov-10-B.1	323	3.93E-61
XM_020078324.1	chromodomain-helicase-DNA-binding protein 5-like	434	7.81E-91
XM_020078325.1	cerebellar degeneration-related protein 2-like	527	2.87E-95
XM_020078326.1	ubiquitin-conjugating enzyme E2 H-like	357	7.76E-29
XM_020078327.1	eukaryotic translation initiation factor 4 gamma 1-like	308	6.30E-73
XM_020078328.1	synaptotagmin-1-like isoform X1	341	1.62E-51
XM_020078329.1	multiple C2 and transmembrane domain-containing protein 2-like isoform X1	4204	0
XM_020078330.1	sortilin-related receptor-like	283	3.66E-66
XM_020078331.1	cytochrome P450 3A19-like	575	1.71E-110
XM_020078332.1	glucose-6-phosphate isomerase-like	561	7.90E-79
XM_020078333.1	stabilin-1 isoform X1	428	1.43E-100
XM_020078334.1	phosphatidylinositol 4-phosphate 3-kinase C2 domain-containing subunit alpha	817	2.82E-150
XM_020078335.1	FRAS1-related extracellular matrix protein 1-like	727	6.36E-172
XM_020078336.1	phosphatidylinositol 3-kinase regulatory subunit beta-like	563	7.09E-138
XM_020078337.1	zinc finger protein 512-like isoform X1	651	8.64E-151
XM_020078338.1	DENN domain-containing protein 4B-like	445	9.65E-96
XM_020078339.1	transmembrane protein 263-like	762	6.51E-53
XM_020078340.1	zinc finger C3H1 domain-containing protein-like	554	1.95E-135
XM_020078341.1	transcription initiation factor TFIID subunit 3-like	451	5.24E-41
XM_020078342.1	protein prune homolog	496	3.73E-118
XM_020078343.1	protein NipSnap homolog 3B-like	567	5.59E-134
XM_020078344.1	transmembrane protein 263-like	872	5.64E-52
XM_020078345.1	importin subunit beta-1-like	586	5.07E-130
XM_020078346.1	ropporin-1-like protein	306	3.10E-71
XM_020078347.1	V-set and transmembrane domain-containing protein 5	553	1.88E-94
XM_020078348.1	dehydrodolichyl diphosphate synthase complex subunit nus1-like	584	7.56E-94
XM_020078349.1	pyruvate dehydrogenase E1 component subunit beta, mitochondrial-like	520	9.58E-122
XM_020078350.1	ATP-binding cassette sub-family A member 1 isoform X2	567	4.68E-101
XM_020078351.1	host cell factor 1 isoform X1	610	2.36E-88
XM_020078352.1	PDZ and LIM domain protein 5-like	894	4.56E-75
XM_020078353.1	bone morphogenetic protein receptor type-2-like	323	3.09E-77
XM_020078354.1	BLOC-1-related complex subunit 5	1975	6.36E-140
XM_020078355.1	NAD-dependent protein deacetylase sirtuin-3-like	475	4.06E-117
XM_020078356.1	B-cell lymphoma/leukemia 11A-like	400	1.09E-79
XM_020078357.1	ubiquitin-like domain-containing CTD phosphatase 1	576	6.35E-78
XM_020078358.1	cell adhesion molecule-related/down-regulated by oncogenes-like	419	2.08E-100
XM_020078359.1	solute carrier family 22 member 13-like	476	3.06E-91
XM_020078360.1	tRNA (adenine(58)-N(1))-methyltransferase, mitochondrial-like isoform X1	457	1.06E-41
XM_020078361.1	tRNA (adenine(58)-N(1))-methyltransferase, mitochondrial-like isoform X2	668	7.91E-41
XM_020078362.1	40S ribosomal protein S13	576	1.00E-108
XM_020078363.1	protein Niban-like	455	3.80E-106
XM_020078364.1	laminin subunit gamma-3	572	3.03E-138
XM_020078365.1	filamin-C-like isoform X1	644	1.62E-136
XM_020078366.1	vesicle-fusing ATPase-like	444	9.50E-94
XM_020078367.1	synaptic vesicular amine transporter	428	6.31E-80
XM_020078368.1	tributyltin binding protein type 2	450	5.49E-45
XM_020078369.1	centrosomal protein of 57 kDa-like	815	1.33E-102
XM_020078370.1	RNA-binding protein 39-like	320	2.17E-63
XM_020078371.1	ankyrin repeat and SOCS box protein 13-like	1495	0
XM_020078372.1	bone morphogenetic protein receptor type-2-like	660	7.34E-151
XM_020078373.1	C-type natriuretic peptide 2-like	611	2.90E-47
XM_020078374.1	alpha-1,3-mannosyl-glycoprotein 4-beta-N-acetylglucosaminyltransferase B-like	255	6.24E-54
XM_020078375.1	ATP-binding cassette sub-family F member 3-like	480	8.46E-77
XM_020078376.1	2'-deoxynucleoside 5'-phosphate N-hydrolase 1	341	4.76E-70
XM_020078377.1	PREDICTED: mitofusin-1-like, partial	630	1.69E-103
XM_020078378.1	mortality factor 4-like protein 1	1497	0
XM_020078379.1	dynein heavy chain 9	368	2.57E-76

XM_020078380.1	phosphofurin acidic cluster sorting protein 2-like	386	7.11E-59
XM_020078381.1	RING finger protein 145-like	333	2.09E-60
XM_020078382.1	traf2 and NCK-interacting protein kinase-like	598	5.31E-75
XM_020078383.1	DNA repair protein complementing XP-G cells homolog	380	1.19E-79
XM_020078384.1	protein downstream neighbor of son homolog	510	5.59E-110
XM_020078385.1	anoctamin-5 isoform X2	290	4.86E-56
XM_020078386.1	DNA replication complex GINS protein PSF1	296	5.90E-70
XM_020078387.1	titin-like isoform X1	7177	0
XM_020078388.1	oocyte zinc finger protein XlCOF6.1-like isoform X1	685	1.22E-128
XM_020078389.1	PREDICTED: interleukin-20-like, partial	457	6.61E-90
XM_020078390.1	dynein heavy chain 5, axonemal-like	434	4.04E-107
XM_020078391.1	tubulin polyglutamylase complex subunit 1-like	509	1.48E-101
XM_020078392.1	3-mercaptopyruvate sulfurtransferase-like	817	0
XM_020078393.1	long-chain-fatty-acid--CoA ligase 5	421	5.84E-102
XM_020078394.1	ephrin type-B receptor 3-like	482	5.12E-116
XM_020078395.1	leucine-rich repeat-containing protein 31-like	597	1.39E-120
XM_020078396.1	fibroin heavy chain-like	992	1.16E-55
XM_020078397.1	ubiquinone biosynthesis protein COQ9, mitochondrial isoform X2	1419	0
XM_020078398.1	ubiquinone biosynthesis protein COQ9, mitochondrial isoform X2	1434	0
XM_020078399.1	bisphosphoglycerate mutase	2546	0
XM_020078400.1	bisphosphoglycerate mutase	2537	0
XM_020078401.1	AMP deaminase 3-like isoform X1	3926	0
XM_020078402.1	AMP deaminase 3-like isoform X2	4077	0
XM_020078403.1	tyrosine--tRNA ligase, mitochondrial	1757	0
XM_020078404.1	amyloid beta A4 precursor protein-binding family A member 2-like isoform X2	3417	0
XM_020078405.1	protocadherin gamma-A11-like	1746	0
XM_020078406.1	amyloid beta A4 precursor protein-binding family A member 2-like isoform X2	3051	0
XM_020078407.1	proteasome subunit alpha type-4	981	4.10E-171
XM_020078408.1	CD82 antigen	1566	3.86E-164
XM_020078409.1	zona pellucida sperm-binding protein 3-like	1283	0
XM_020078410.1	cyclin-dependent kinase inhibitor 1B	1534	1.33E-156
XM_020078411.1	annexin A2-A-like	1849	0
XM_020078412.1	tyrosine-protein kinase CSK-like isoform X1	2881	0
XM_020078413.1	tyrosine-protein kinase CSK-like isoform X1	2779	0
XM_020078414.1	tyrosine-protein kinase CSK-like isoform X1	2428	0
XM_020078415.1	calcium-independent phospholipase A2-gamma-like isoform X2	4196	0
XM_020078416.1	calcium-independent phospholipase A2-gamma-like isoform X1	4009	0
XM_020078417.1	protocadherin gamma-A11-like	1723	0
XM_020078418.1	stomatin-like protein 1 isoform X1	1504	0
XM_020078419.1	stomatin-like protein 1 isoform X2	1501	0
XM_020078420.1	BET1-like protein isoform X1	1694	6.87E-67
XM_020078421.1	BET1-like protein isoform X1	1695	8.51E-62
XM_020078422.1	death-associated protein kinase 2 isoform X1	2205	0
XM_020078423.1	death-associated protein kinase 3-like	2185	0
XM_020078424.1	small acidic protein	1455	2.30E-112
XM_020078425.1	kielin/chordin-like protein	6998	0
XM_020078426.1	centrosomal protein of 41 kDa isoform X1	1626	0
XM_020078427.1	centrosomal protein of 41 kDa isoform X1	1678	0
XM_020078428.1	acid trehalase-like protein 1	2755	0
XM_020078429.1	acid trehalase-like protein 1	2564	0
XM_020078430.1	protocadherin alpha-13-like	1776	0
XM_020078431.1	p53-induced death domain-containing protein 1	3877	0
XM_020078432.1	forkhead box protein B1	2206	0
XM_020078433.1	glucoside xylosyltransferase 1 isoform X1	3197	0
XM_020078434.1	glucoside xylosyltransferase 1 isoform X2	3101	0
XM_020078435.1	WD repeat-containing protein 61	1225	0
XM_020078436.1	excitatory amino acid transporter 2 isoform X2	2090	0
XM_020078437.1	excitatory amino acid transporter 2 isoform X2	1959	0
XM_020078438.1	excitatory amino acid transporter 2 isoform X2	1741	0
XM_020078439.1	excitatory amino acid transporter 2 isoform X2	2016	0
XM_020078440.1	LIM domain only protein 3 isoform X2	1713	2.57E-111
XM_020078441.1	LIM domain only protein 3 isoform X2	1541	4.39E-112
XM_020078442.1	LIM domain only protein 3 isoform X2	1599	8.16E-112
XM_020078443.1	protocadherin gamma-A11-like	1593	0
XM_020078444.1	LIM domain only protein 3 isoform X2	1409	1.08E-112
XM_020078445.1	PREDICTED: hyaluronidase-4-like	5636	0
XM_020078446.1	glycine cleavage system H protein, mitochondrial-like	1069	1.25E-103
XM_020078447.1	PREDICTED: caveolin-2	2133	4.18E-110
XM_020078448.1	G1/S-specific cyclin-D2-like	1279	0
XM_020078449.1	putative tRNA (cytidine(32)/guanosine(34)-2'-O)-methyltransferase	1474	0
XM_020078450.1	putative tRNA (cytidine(32)/guanosine(34)-2'-O)-methyltransferase	1468	0
XM_020078451.1	PQ-loop repeat-containing protein 1-like	4157	1.71E-156
XM_020078452.1	ras-specific guanine nucleotide-releasing factor 1-like isoform X1	6931	0
XM_020078453.1	COP9 signalosome complex subunit 6	1335	0
XM_020078454.1	alpha-1,3-mannosyl-glycoprotein 4-beta-N-acetylglucosaminyltransferase C-like isoform X1	1848	0
XM_020078455.1	alpha-1,3-mannosyl-glycoprotein 4-beta-N-acetylglucosaminyltransferase C-like isoform X1	1808	0
XM_020078456.1	alpha-1,3-mannosyl-glycoprotein 4-beta-N-acetylglucosaminyltransferase C-like isoform X1	1714	0
XM_020078457.1	RING finger protein 141	1593	3.08E-169
XM_020078458.1	RING finger protein 141	1782	2.71E-168

XM_020078459.1	RING finger protein 141	1594	3.19E-169
XM_020078460.1	alpha-ketoglutarate-dependent dioxygenase alkB homolog 3	2071	0
XM_020078461.1	alpha-ketoglutarate-dependent dioxygenase alkB homolog 3	1197	0
XM_020078462.1	alpha-ketoglutarate-dependent dioxygenase alkB homolog 3	1304	0
XM_020078463.1	rhombotin-1-like isoform X1	1515	3.11E-110
XM_020078464.1	rhombotin-1-like isoform X2	1414	1.59E-94
XM_020078465.1	brevican core protein-like	3381	0
XM_020078466.1	leucine-rich repeat-containing protein 61	1708	3.17E-171
XM_020078467.1	nucleotide exchange factor SIL1 isoform X1	1825	0
XM_020078468.1	calcium and integrin-binding family member 2	1521	2.57E-133
XM_020078469.1	synaptotagmin-12 isoform X1	3171	0
XM_020078470.1	synaptotagmin-12 isoform X1	3508	0
XM_020078471.1	troponin I, fast skeletal muscle-like	1004	4.63E-119
XM_020078472.1	beta-parvin isoform X1	1660	0
XM_020078473.1	beta-parvin isoform X2	1545	0
XM_020078474.1	chondroitin sulfate proteoglycan 4-like	8958	0
XM_020078475.1	nucleotide exchange factor SIL1 isoform X1	1741	0
XM_020078476.1	lysM and putative peptidoglycan-binding domain-containing protein 4-like isoform X1	3411	0
XM_020078477.1	lysM and putative peptidoglycan-binding domain-containing protein 4-like isoform X1	3322	0
XM_020078478.1	PREDICTED: uncharacterized protein LOC109623993	3103	0
XM_020078479.1	high affinity choline transporter 1-like	2546	0
XM_020078480.1	OTU domain-containing protein 7B-like	4733	0
XM_020078481.1	OTU domain-containing protein 7B-like	4558	0
XM_020078482.1	ceramide synthase 2-like	1557	0
XM_020078483.1	ceramide synthase 2-like	1613	0
XM_020078484.1	nucleotide exchange factor SIL1 isoform X1	1822	0
XM_020078485.1	ceramide synthase 2-like	1543	0
XM_020078486.1	ceramide synthase 2-like	1599	0
XM_020078487.1	multiple epidermal growth factor-like domains protein 11	5177	0
XM_020078488.1	peroxisomal membrane protein 11A-like	1290	7.21E-172
XM_020078489.1	vasopressin V1a receptor	2297	0
XM_020078490.1	homeobox protein aristaless-like 4	2133	0
XM_020078491.1	neuroplastin-like isoform X1	2151	0
XM_020078492.1	pyroglutamyl-peptidase 1-like	3176	1.31E-138
XM_020078493.1	structural maintenance of chromosomes protein 1B-like isoform X1	4360	0
XM_020078494.1	structural maintenance of chromosomes protein 1B-like isoform X2	4048	0
XM_020078495.1	protein Spindly	2056	0
XM_020078496.1	mucin-5AC-like isoform X1	4564	0
XM_020078497.1	mucin-5AC-like isoform X2	4561	0
XM_020078498.1	ankyrin repeat, SAM and basic leucine zipper domain-containing protein 1	1870	0
XM_020078499.1	dnaJ homolog subfamily B member 9	2230	2.26E-130
XM_020078500.1	lipoma HMGIC fusion partner-like 3 protein isoform X1	2078	5.82E-161
XM_020078501.1	lipoma HMGIC fusion partner-like 3 protein isoform X1	2084	6.20E-161
XM_020078502.1	lipoma HMGIC fusion partner-like 3 protein isoform X1	2050	1.57E-158
XM_020078503.1	PREDICTED: uncharacterized protein LOC109624010	1824	2.52E-125
XM_020078504.1	PREDICTED: sarcospan	2429	4.09E-141
XM_020078505.1	low-density lipoprotein receptor class A domain-containing protein 3 isoform X1	2729	5.56E-171
XM_020078506.1	low-density lipoprotein receptor class A domain-containing protein 3 isoform X2	2726	4.06E-170
XM_020078507.1	pituitary homeobox 2 isoform X2	1503	2.19E-167
XM_020078508.1	microtubule-associated proteins 1A/1B light chain 3C-like	1194	1.48E-95
XM_020078509.1	low-density lipoprotein receptor class A domain-containing protein 3 isoform X3	2582	3.10E-133
XM_020078510.1	protein phosphatase 1 regulatory subunit 3A-like isoform X3	4783	0
XM_020078511.1	cellular retinoic acid-binding protein 1	1006	3.33E-99
XM_020078512.1	exocyst complex component 3-like protein	3774	0
XM_020078513.1	PREDICTED: uncharacterized protein KIAA1644 homolog isoform X1	967	4.41E-148
XM_020078514.1	PREDICTED: uncharacterized protein KIAA1644 homolog isoform X2	4351	1.04E-134
XM_020078515.1	potassium voltage-gated channel subfamily D member 2-like isoform X1	2264	0
XM_020078516.1	prosaposin receptor GPR37	2987	0
XM_020078517.1	class E basic helix-loop-helix protein 41 isoform X1	2062	0
XM_020078518.1	class E basic helix-loop-helix protein 41 isoform X2	2059	0
XM_020078519.1	leucine-rich repeat and immunoglobulin-like domain-containing nogo receptor-interacting protein 2	3681	0
XM_020078520.1	neuronal acetylcholine receptor subunit alpha-3-like	2163	0
XM_020078521.1	ras association domain-containing protein 10-like	2398	0
XM_020078522.1	carbonic anhydrase 12 isoform X1	2465	0
XM_020078523.1	carbonic anhydrase 12 isoform X2	2854	0
XM_020078524.1	protein inscuteable homolog	2674	0
XM_020078525.1	transmembrane protein 117 isoform X1	2838	0
XM_020078526.1	transmembrane protein 117 isoform X1	2073	0
XM_020078527.1	alpha-1,3-mannosyl-glycoprotein 4-beta-N-acetylglucosaminyltransferase C	2654	0
XM_020078528.1	alpha-1,3-mannosyl-glycoprotein 4-beta-N-acetylglucosaminyltransferase C	2785	0
XM_020078529.1	lysyl oxidase homolog 4-like	2440	0
XM_020078530.1	leucine-rich repeat and transmembrane domain-containing protein 2-like	2010	0
XM_020078531.1	mitochondrial import receptor subunit TOM5 homolog	587	2.23E-18
XM_020078532.1	cyclic nucleotide-gated cation channel beta-3-like	4758	0
XM_020078533.1	nuclear pore complex protein Nup93	2199	0
XM_020078534.1	protein MAL2	1040	6.88E-121
XM_020078535.1	proprotein convertase subtilisin/kexin type 6	4315	0
XM_020078536.1	SH3 and multiple ankyrin repeat domains protein 3	10772	0
XM_020078537.1	arylsulfatase G	1290	0

XM_020078538.1	kelch-like protein 42	1978	0
XM_020078539.1	nucleus accumbens-associated protein 1-like isoform X3	1732	0
XM_020078540.1	sterol-4-alpha-carboxylate 3-dehydrogenase, decarboxylating	1740	0
XM_020078541.1	PREDICTED: uncharacterized protein LOC109624042	1129	0
XM_020078542.1	WW domain-containing oxidoreductase	2398	0
XM_020078543.1	genetic suppressor element 1-like	1022	2.89E-153
XM_020078544.1	serine/threonine-protein kinase WNK1-like	7950	0
XM_020078545.1	zona pellucida sperm-binding protein 4-like	1413	0
XM_020078546.1	inositol 1,4,5-trisphosphate receptor type 2	9405	0
XM_020078547.1	protein piccolo-like	19767	0
XM_020078548.1	voltage-dependent calcium channel subunit alpha-2/delta-1	5140	0
XM_020078549.1	inosine-5'-monophosphate dehydrogenase 1b-like	1186	0
XM_020078550.1	cadherin-related family member 5-like	2863	0
XM_020078551.1	synaptonemal complex central element protein 3	723	4.57E-65
XM_020078552.1	serine/threonine-protein phosphatase 6 regulatory subunit 2-like isoform X1	3233	0
XM_020078553.1	PREDICTED: ADM2-like	504	1.64E-83
XM_020078554.1	beta-1,4-N-acetylgalactosaminyltransferase 3-like	2325	0
XM_020078555.1	C2 domain-containing protein 5	4590	0
XM_020078556.1	beta-1,4-N-acetylgalactosaminyltransferase 3-like	3926	0
XM_020078557.1	phosphatidylinositol phosphatase PTPRQ	9929	0
XM_020078558.1	von Willebrand factor	8817	0

XM_020078559.1	neurofilament light polypeptide-like	4471	0
XM_020078560.1	myocyte-specific enhancer factor 2A	5726	0
XM_020078561.1	gamma-aminobutyric acid receptor subunit alpha-5	1910	0
XM_020078562.1	gonadotropin-releasing hormone II receptor-like	1188	0
XM_020078563.1	ras and EF-hand domain-containing protein-like	2837	0
XM_020078564.1	FERM domain-containing protein 5	5851	0
XM_020078565.1	mucosa-associated lymphoid tissue lymphoma translocation protein 1-like	2170	0
XM_020078566.1	cholesterol 25-hydroxylase-like protein 1, member 1	630	3.60E-156
XM_020078567.1	S phase cyclin A-associated protein in the endoplasmic reticulum	4573	0
XM_020078568.1	photoreceptor-specific nuclear receptor-like	1266	0
XM_020078569.1	zinc-binding protein A33-like	1431	0
XM_020078570.1	translation factor GUF1, mitochondrial-like	1098	0
XM_020078571.1	protein phosphatase 1 regulatory subunit 32-like	1207	0
XM_020078572.1	unconventional myosin-Id	2355	0
XM_020078573.1	PREDICTED: bestrophin-1-like	1803	0
XM_020078574.1	eosinophil peroxidase-like	2697	0
XM_020078575.1	intestinal mucin-like protein	1562	0
XM_020078576.1	PREDICTED: mucin-2-like, partial	3894	0
XM_020078577.1	PREDICTED: mucin-2-like, partial	966	1.78E-132
XM_020078578.1	pituitary homeobox 2 isoform X2	1461	8.67E-133
XM_020078579.1	olfactomedin-like protein 2A	1994	0
XM_020078580.1	dual oxidase maturation factor 1-like	1043	0
XM_020078581.1	poly [ADP-ribose] polymerase 6-like	1923	0
XM_020078582.1	neuronal acetylcholine receptor subunit non-alpha-2-like	1571	0
XM_020078583.1	early endosome antigen 1-like	2847	0
XM_020078584.1	enhancer of mRNA-decapping protein 3-like	2904	6.92E-125
XM_020078585.1	1-phosphatidylinositol 4,5-bisphosphate phosphodiesterase zeta-1-like	1596	0
XM_020078586.1	vertebrate ancient opsin-like	1153	0
XM_020078587.1	PREDICTED: uncharacterized protein C3orf20-like	943	0
XM_020078588.1	PREDICTED: uncharacterized protein C3orf20-like	654	2.93E-154
XM_020078589.1	cartilage intermediate layer protein 1-like	606	1.19E-149
XM_020078590.1	putative ATP-dependent RNA helicase TDRD12	5020	0
XM_020078591.1	ankyrin repeat domain-containing protein 50-like	5277	0
XM_020078592.1	PREDICTED: beta-synuclein	1030	3.10E-76
XM_020078593.1	transmembrane emp24 domain-containing protein 6-like	743	6.30E-165
XM_020078594.1	mixed lineage kinase domain-like protein	2026	0
XM_020078595.1	Golgi apparatus protein 1-like	4376	0
XM_020078596.1	copine-5-like isoform X2	1893	0
XM_020078597.1	leucine-rich repeat and IQ domain-containing protein 1	5945	0
XM_020078598.1	PREDICTED: beta-synuclein	988	2.70E-76
XM_020078599.1	eukaryotic translation initiation factor 4 gamma 2-like	4200	0
XM_020078600.1	solute carrier family 23 member 1-like	2333	0
XM_020078601.1	protein Wnt-2	884	0
XM_020078602.1	cystic fibrosis transmembrane conductance regulator	5736	0
XM_020078603.1	high choriolytic enzyme 1-like	941	0
XM_020078604.1	deformed epidermal autoregulatory factor 1 homolog	2521	0
XM_020078605.1	PREDICTED: putative uncharacterized protein DDB_G0290521	489	8.60E-33
XM_020078606.1	D(4) dopamine receptor-like	1326	0
XM_020078607.1	cadherin-related family member 5-like	2945	0
XM_020078608.1	kelch repeat and BTB domain-containing protein 13-like	1446	0
XM_020078609.1	gamma-aminobutyric acid receptor subunit alpha-6-like	1984	0
XM_020078610.1	protection of telomeres protein 1	2449	0
XM_020078611.1	hyaluronidase-1-like isoform X1	1554	0
XM_020078612.1	dynein heavy chain 12, axonemal-like	1725	0
XM_020078613.1	solute carrier family 13 member 1	2415	0
XM_020078614.1	receptor-type tyrosine-protein phosphatase zeta	6655	0
XM_020078615.1	voltage-dependent calcium channel subunit alpha-2/delta-4	3033	0
XM_020078616.1	forkhead box protein P2-like	2091	0

XM_020078617.1	PREDICTED: mucin-1-like	653	2.60E-73
XM_020078618.1	secretory carrier-associated membrane protein 2-like	1082	7.39E-180
XM_020078619.1	aggrecan core protein-like	6591	0
XM_020078620.1	cationic amino acid transporter 2-like	2566	0
XM_020078621.1	PREDICTED: uncharacterized protein LOC109624123, partial	1658	0
XM_020078622.1	ethanolamine kinase 1-like	1840	0
XM_020078623.1	protein RIC-3-like	2070	0
XM_020078624.1	tripartite motif-containing protein 66	930	0
XM_020078625.1	von Willebrand factor A domain-containing protein 3B	576	2.13E-121
XM_020078626.1	histone deacetylase 10	2510	0
XM_020078627.1	MANSC domain-containing protein 4	1218	0
XM_020078628.1	transcription intermediary factor 1-alpha-like	3286	0
XM_020078629.1	overexpressed in colon carcinoma 1 protein	219	2.21E-48
XM_020078630.1	NUAK family SNF1-like kinase 1	4957	0
XM_020078631.1	centromere protein U	1548	0
XM_020078632.1	RecName: Full=Prolactin; Short=PRL; Flags: Precursor	759	0
XM_020078633.1	protein kinase C-binding protein NELL2	3331	0
XM_020078634.1	non-muscle caldesmon-like isoform X1	2595	3.88E-110
XM_020078635.1	PREDICTED: spexin-like	255	2.32E-58
XM_020078636.1	unconventional myosin-Vc	6146	0
XM_020078637.1	AP-4 complex subunit epsilon-1 isoform X1	5471	0
XM_020078638.1	AP-4 complex subunit epsilon-1 isoform X1	5480	0
XM_020078639.1	anoctamin-4 isoform X3	6426	0
XM_020078640.1	anoctamin-5 isoform X1	4916	0
XM_020078641.1	anoctamin-5 isoform X2	2928	0
XM_020078642.1	xaa-Pro aminopeptidase 2	2364	0
XM_020078643.1	anoctamin-5 isoform X3	4732	0
XM_020078644.1	anoctamin-4 isoform X1	3035	0
XM_020078645.1	ATP-dependent Clp protease ATP-binding subunit clpX-like, mitochondrial isoform X1	3671	0
XM_020078646.1	ATP-dependent Clp protease ATP-binding subunit clpX-like, mitochondrial isoform X2	3629	0
XM_020078647.1	protein regulator of cytokinesis 1-like	2278	0
XM_020078648.1	vesicular glutamate transporter 2.1-like	3668	0
XM_020078649.1	PREDICTED: aromatase-like	2041	0
XM_020078650.1	pituitary homeobox 2 isoform X2	1459	1.40E-132
XM_020078651.1	ubiquitin-associated protein 1-like	1310	0
XM_020078652.1	P2Y purinoceptor 3-like	1307	0
XM_020078653.1	guanine nucleotide-binding protein G(I)/G(S)/G(T) subunit beta-1-like	2411	0
XM_020078654.1	V-type proton ATPase subunit e 1-like	752	5.21E-51
XM_020078655.1	Fanconi anemia group F protein	3782	0
XM_020078656.1	growth arrest-specific protein 2	1810	0
XM_020078657.1	growth arrest-specific protein 2	1810	0
XM_020078658.1	growth arrest-specific protein 2	1772	0
XM_020078659.1	transmembrane protein 178B-like	2018	0
XM_020078660.1	transcription factor PU.1	2012	3.84E-174
XM_020078661.1	fin bud initiation factor-like	1388	3.52E-143
XM_020078662.1	intestinal mucin-like protein	496	4.83E-80
XM_020078663.1	small VCP/p97-interacting protein	2221	3.75E-28
XM_020078664.1	sorting and assembly machinery component 50 homolog A isoform X1	1757	0
XM_020078665.1	sorting and assembly machinery component 50 homolog A isoform X2	1747	0
XM_020078666.1	GDNF family receptor alpha-4-like	2973	0
XM_020078667.1	protein SAAL1	1870	0
XM_020078668.1	isocitrate dehydrogenase [NAD] subunit alpha, mitochondrial isoform X1	1747	0
XM_020078669.1	isocitrate dehydrogenase [NAD] subunit alpha, mitochondrial isoform X2	1678	0
XM_020078670.1	DNA-directed RNA polymerase II subunit RPB3	1087	0
XM_020078671.1	cytochrome c oxidase subunit 5A, mitochondrial-like	955	1.28E-100
XM_020078672.1	alpha- and gamma-adaptin-binding protein p34 isoform X1	1696	0
XM_020078673.1	alpha- and gamma-adaptin-binding protein p34 isoform X2	1432	0
XM_020078674.1	plasma membrane calcium-transporting ATPase 1-like isoform X1	3768	0
XM_020078675.1	GDNF family receptor alpha-4-like	2862	0
XM_020078676.1	plasma membrane calcium-transporting ATPase 1-like isoform X2	3759	0
XM_020078677.1	plasma membrane calcium-transporting ATPase 1-like isoform X3	3732	0
XM_020078678.1	plasma membrane calcium-transporting ATPase 1-like isoform X3	3699	0
XM_020078679.1	NAD-dependent protein deacetylase sirtuin-3-like	1977	0
XM_020078680.1	NAD-dependent protein deacetylase sirtuin-3-like	2137	0
XM_020078681.1	---NA---	528	
XM_020078682.1	paired box protein Pax-6-like isoform X1	2378	0
XM_020078683.1	paired box protein Pax-6-like isoform X2	2399	0
XM_020078684.1	GDP-D-glucose phosphorylase 1 isoform X1	1802	0
XM_020078685.1	GDP-D-glucose phosphorylase 1 isoform X2	1799	0
XM_020078686.1	kxDL motif-containing protein 1	1422	2.79E-134
XM_020078687.1	phosphopantothenoylcysteine decarboxylase	798	1.17E-153
XM_020078688.1	homeobox protein Nkx-3.2	1943	5.15E-154
XM_020078689.1	DNA damage-binding protein 2 isoform X1	1672	0
XM_020078690.1	DNA damage-binding protein 2 isoform X2	1650	0
XM_020078691.1	protein FAM180A-like	723	9.10E-105
XM_020078692.1	POC1 centriolar protein homolog B	2385	0
XM_020078693.1	tryptophan 5-hydroxylase 1 isoform X1	2048	0
XM_020078694.1	tryptophan 5-hydroxylase 1 isoform X2	2009	0
XM_020078695.1	poly(U)-specific endoribonuclease-C-like	1529	0

XM_020078696.1	cytosolic 5'-nucleotidase 1B-like isoform X1	1611	7.06E-156
XM_020078697.1	cytosolic 5'-nucleotidase 1B-like isoform X2	1605	2.49E-153
XM_020078698.1	PREDICTED: netrin-4-like	1879	8.20E-63
XM_020078699.1	solute carrier organic anion transporter family member 1C1-like	3560	0
XM_020078700.1	soluble guanylate cyclase 88E-like isoform X1	3162	0
XM_020078701.1	solute carrier organic anion transporter family member 1C1-like	3302	0
XM_020078702.1	cholesterol side-chain cleavage enzyme, mitochondrial	1924	0
XM_020078703.1	transcription factor 12-like isoform X2	2542	0
XM_020078704.1	transcription factor 12-like isoform X2	2379	0
XM_020078705.1	zona pellucida sperm-binding protein 3-like	1641	0
XM_020078706.1	sorting nexin-24-like	1017	1.17E-141
XM_020078707.1	proprotein convertase subtilisin/kexin type 5-like	2622	0
XM_020078708.1	soluble guanylate cyclase 88E-like isoform X1	3148	0
XM_020078709.1	cGMP-inhibited 3',5'-cyclic phosphodiesterase A	4139	0
XM_020078710.1	PREDICTED: uncharacterized protein LOC109624190	1799	4.00E-168
XM_020078711.1	F-box and leucine-rich protein 22	1992	5.37E-137
XM_020078712.1	carboxypeptidase A2-like	1388	0
XM_020078713.1	telomere repeats-binding bouquet formation protein 1 isoform X1	2964	0
XM_020078714.1	telomere repeats-binding bouquet formation protein 1 isoform X2	2934	0
XM_020078715.1	telomere repeats-binding bouquet formation protein 1 isoform X3	2644	0
XM_020078716.1	soluble guanylate cyclase 88E-like isoform X1	3138	0
XM_020078717.1	protein KTI12 homolog	961	0
XM_020078718.1	apelin receptor B-like	2177	0
XM_020078719.1	PREDICTED: uncharacterized protein LOC109624196	1306	2.69E-161
XM_020078720.1	myelin regulatory factor isoform X1	5581	0
XM_020078721.1	myelin regulatory factor isoform X2	5021	0
XM_020078722.1	myelin regulatory factor isoform X3	5015	0

XM_020078723.1	myelin regulatory factor isoform X4	4952	0
XM_020078724.1	cartilage acidic protein 1-like isoform X1	1926	0
XM_020078725.1	cartilage acidic protein 1-like isoform X2	1998	0
XM_020078726.1	zinc finger and SCAN domain-containing protein 2-like isoform X1	2030	0
XM_020078727.1	zinc finger and BTB domain-containing protein 49-like isoform X2	2013	0
XM_020078728.1	myotubularin-related protein 4 isoform X2	1858	0
XM_020078729.1	peptidyl-prolyl cis-trans isomerase NIMA-interacting 4	746	5.01E-65
XM_020078730.1	coiled-coil domain-containing protein R3HCC1L	1734	0
XM_020078731.1	tectonic-1 isoform X2	2022	0
XM_020078732.1	pituitary homeobox 2 isoform X2	1174	3.64E-133
XM_020078733.1	golgin subfamily A member 7B-like	1585	6.94E-98
XM_020078734.1	zona pellucida sperm-binding protein 4-like	1442	0
XM_020078735.1	synaptotagmin-1-like isoform X1	2215	0
XM_020078736.1	synaptotagmin-1-like isoform X1	2276	0
XM_020078737.1	synaptotagmin-1-like isoform X1	2134	0
XM_020078738.1	sperm acrosome membrane-associated protein 4-like	887	3.15E-106
XM_020078739.1	sperm acrosome membrane-associated protein 4-like isoform X1	719	2.82E-87
XM_020078740.1	charged multivesicular body protein 1b-like isoform X1	1212	4.77E-121
XM_020078741.1	sperm acrosome membrane-associated protein 4-like isoform X2	719	6.64E-83
XM_020078742.1	sperm acrosome membrane-associated protein 4-like isoform X1	767	8.81E-80
XM_020078743.1	PREDICTED: olfactomedin-4-like	1963	0
XM_020078744.1	PREDICTED: olfactomedin-like	1658	0
XM_020078745.1	calcium and integrin-binding protein 1	880	1.17E-120
XM_020078746.1	PREDICTED: uncharacterized protein C11orf16 homolog	1243	0
XM_020078747.1	A-kinase-interacting protein 1	764	9.23E-123
XM_020078748.1	charged multivesicular body protein 1b-like isoform X2	1267	2.94E-118
XM_020078749.1	A-kinase-interacting protein 1	663	1.47E-123
XM_020078750.1	achaete-scute homolog 1b-like	1611	7.69E-115
XM_020078751.1	IQ domain-containing protein H	3612	0
XM_020078752.1	PREDICTED: uncharacterized protein C12orf73 homolog	613	4.02E-49
XM_020078753.1	transcription factor Maf-like	2555	2.47E-131
XM_020078754.1	IQ motif and SEC7 domain-containing protein 3-like	5647	0
XM_020078755.1	leucine-rich repeat-containing protein 4C-like	5089	0
XM_020078756.1	leucine-rich repeat-containing protein 4C-like	4453	0
XM_020078757.1	dnaJ homolog subfamily C member 24 isoform X1	681	6.22E-120
XM_020078758.1	dnaJ homolog subfamily C member 24 isoform X2	612	8.58E-102
XM_020078759.1	gamma-parvin isoform X1	2354	0
XM_020078760.1	cyclic nucleotide-gated cation channel-like	2102	0
XM_020078761.1	gamma-parvin isoform X2	2327	0
XM_020078762.1	sodium/nucleoside cotransporter 2-like	2090	0
XM_020078763.1	PREDICTED: leiomodin-2	1727	0
XM_020078764.1	bile acid receptor isoform X1	1758	0
XM_020078765.1	bile acid receptor isoform X1	2425	0
XM_020078766.1	bile acid receptor isoform X1	2398	0
XM_020078767.1	bile acid receptor isoform X1	1546	0
XM_020078768.1	potassium/sodium hyperpolarization-activated cyclic nucleotide-gated channel 3-like	3767	0
XM_020078769.1	homeobox protein DBX1 isoform X3	1513	4.65E-179
XM_020078770.1	ras and EF-hand domain-containing protein homolog	2196	0
XM_020078771.1	ras and EF-hand domain-containing protein homolog	2193	0
XM_020078772.1	fibrinogen-like protein 1	1425	0
XM_020078773.1	secreted frizzled-related protein 5-like	1406	0
XM_020078774.1	calcitonin gene-related peptide 2 isoform X1	937	2.69E-124

XM_020078775.1	calcitonin gene-related peptide 2 isoform X2	1015	3.05E-121
XM_020078776.1	calcitonin gene-related peptide 2 isoform X3	589	1.55E-119
XM_020078777.1	calcitonin gene-related peptide 2 isoform X4	586	9.90E-119
XM_020078778.1	calcitonin gene-related peptide 2 isoform X2	934	9.81E-99
XM_020078779.1	ETS homologous factor	1653	0
XM_020078780.1	fibulin-7-like isoform X2	2763	0
XM_020078781.1	fez family zinc finger protein 2	2664	0
XM_020078782.1	ras-related and estrogen-regulated growth inhibitor-like protein	1018	1.22E-155
XM_020078783.1	calnexin-like isoform X4	685	7.08E-59
XM_020078784.1	RNA-binding Raly-like protein isoform X1	2506	1.45E-111
XM_020078785.1	RNA-binding Raly-like protein isoform X1	2384	2.10E-101
XM_020078786.1	methionine aminopeptidase 2-like	1861	0
XM_020078787.1	PREDICTED: uncharacterized protein LOC109624241	1562	0
XM_020078788.1	high choriolytic enzyme 2-like	1026	0
XM_020078789.1	synaptotagmin-7-like isoform X1	1606	0
XM_020078790.1	synaptotagmin-7-like isoform X2	1565	0
XM_020078791.1	PREDICTED: uncharacterized protein LOC109624244 isoform X1	1132	1.94E-142
XM_020078792.1	PREDICTED: uncharacterized protein LOC109624244 isoform X2	1100	2.15E-136
XM_020078793.1	PREDICTED: uncharacterized protein LOC109624244 isoform X3	1021	1.15E-113
XM_020078794.1	CC chemokine, Paol-SCYA105	669	1.13E-65
XM_020078795.1	sodium-dependent phosphate transport protein 2B-like	2114	0
XM_020078796.1	vesicular glutamate transporter 3	1761	0
XM_020078797.1	anoctamin-4 isoform X1	4156	0
XM_020078798.1	carbohydrate sulfotransferase 1-like	2970	0
XM_020078799.1	sodium/potassium/calcium exchanger 2 isoform X1	2181	0
XM_020078800.1	interferon-induced transmembrane protein 5-like	1618	3.03E-83
XM_020078801.1	PREDICTED: uncharacterized protein LOC109624253	1097	0
XM_020078802.1	B-cell receptor-associated protein 29-like	1248	8.47E-174
XM_020078803.1	pro-neuregulin-4, membrane-bound isoform	904	1.29E-44
XM_020078804.1	pro-neuregulin-4, membrane-bound isoform	922	1.53E-44
XM_020078805.1	membrane-spanning 4-domains subfamily A member 4D-like	1652	4.49E-121
XM_020078806.1	cilia- and flagella-associated protein 161 isoform X1	1380	0
XM_020078807.1	cilia- and flagella-associated protein 161 isoform X2	1257	2.07E-173
XM_020078808.1	protein FAM19A5-like isoform X1	968	5.38E-81
XM_020078809.1	protein FAM19A5-like isoform X2	3256	1.06E-69
XM_020078810.1	pituitary homeobox 2 isoform X2	1118	1.00E-133
XM_020078811.1	PREDICTED: netrin-4-like	1021	1.03E-147
XM_020078812.1	proline-rich transmembrane protein 4-like	4241	0
XM_020078813.1	hyaluronidase PH-20-like	1867	0
XM_020078814.1	recombination activating protein 1	3319	0
XM_020078815.1	receptor-interacting serine/threonine-protein kinase 3-like isoform X1	1823	0
XM_020078816.1	receptor-interacting serine/threonine-protein kinase 3-like isoform X2	1799	0
XM_020078817.1	reverse transcriptase-like protein	1380	0
XM_020078818.1	X-linked interleukin-1 receptor accessory protein-like 2	4274	0
XM_020078819.1	C2 calcium-dependent domain-containing protein 4C-like	1482	0
XM_020078820.1	C2 calcium-dependent domain-containing protein 4C-like	1462	0
XM_020078821.1	hyaluronidase PH-20-like	1586	0
XM_020078822.1	myoD family inhibitor domain-containing protein-like	1178	6.61E-98
XM_020078823.1	lymphatic vessel endothelial hyaluronic acid receptor 1-like	1336	0
XM_020078824.1	3-oxo-5-beta-steroid 4-dehydrogenase-like	996	0
XM_020078825.1	EF-hand calcium-binding domain-containing protein 10-like	546	1.33E-98
XM_020078826.1	transmembrane protein 116-like	1461	0
XM_020078827.1	patatin-like phospholipase domain-containing protein 2 isoform X1	1680	0
XM_020078828.1	patatin-like phospholipase domain-containing protein 2 isoform X1	1582	0
XM_020078829.1	5-hydroxytryptamine receptor 4-like	1918	0
XM_020078830.1	PREDICTED: uncharacterized protein C3orf18-like	986	3.14E-60
XM_020078831.1	recombination activating protein 2	1602	0
XM_020078832.1	PREDICTED: ADM-like	1036	6.33E-112
XM_020078833.1	nuclear receptor-interacting protein 3 isoform X1	1414	1.21E-167
XM_020078834.1	nuclear receptor-interacting protein 3 isoform X2	1411	8.08E-167
XM_020078835.1	cysteine-rich motor neuron 1 protein-like	858	1.09E-139
XM_020078836.1	tissue factor pathway inhibitor-like isoform X1	1545	4.12E-100
XM_020078837.1	PREDICTED: uncharacterized protein LOC109624283	1058	1.01E-144
XM_020078838.1	oncoprotein-induced transcript 3 protein-like	1227	0
XM_020078839.1	dual oxidase 1	4584	0
XM_020078840.1	ubiquitin carboxyl-terminal hydrolase 37-like	1254	0
XM_020078841.1	ankyrin repeat domain-containing protein 34C	1843	0
XM_020078842.1	ras-related and estrogen-regulated growth inhibitor-like protein isoform X1	787	1.27E-152
XM_020078843.1	ras-related and estrogen-regulated growth inhibitor-like protein isoform X2	787	8.58E-153
XM_020078844.1	neurotensin/neuromedin N	992	1.09E-81
XM_020078845.1	PREDICTED: leptin-like	477	1.60E-95
XM_020078846.1	leucine-rich repeat-containing protein 10B-like	1303	3.83E-142
XM_020078847.1	reticulon-4 receptor-like 2	2940	0
XM_020078848.1	proprotein convertase subtilisin/kexin type 5-like	831	7.33E-120
XM_020078849.1	transmembrane emp24 domain-containing protein 6-like	794	3.37E-163
XM_020078850.1	cholesterol 25-hydroxylase-like protein 1, member 2	834	0
XM_020078851.1	nuclear factor of activated T-cells 5-like isoform X2	8127	0
XM_020078852.1	nuclear factor of activated T-cells 5-like isoform X2	7835	0
XM_020078853.1	genetic suppressor element 1-like isoform X1	5154	0

XM_020078854.1	genetic suppressor element 1-like isoform X2	5151	0
XM_020078855.1	26S proteasome non-ATPase regulatory subunit 7	1473	0
XM_020078856.1	peroxisomal coenzyme A diphosphatase NUDT7	901	6.54E-143
XM_020078857.1	hypoxanthine-guanine phosphoribosyltransferase-like isoform X1	1374	2.24E-155
XM_020078858.1	hypoxanthine-guanine phosphoribosyltransferase-like isoform X2	1356	3.50E-151
XM_020078859.1	zinc finger protein RFP-like	1709	0
XM_020078860.1	dynein light chain roadblock-type 2	808	8.49E-51
XM_020078861.1	gastrin-releasing peptide	678	2.26E-89
XM_020078862.1	liprin-beta-1 isoform X1	4174	0
XM_020078863.1	liprin-beta-1 isoform X2	4141	0
XM_020078864.1	liprin-beta-1 isoform X3	4129	0
XM_020078865.1	liprin-beta-1 isoform X4	4096	0
XM_020078866.1	aryl hydrocarbon receptor nuclear translocator-like protein 1 isoform X1	4885	0
XM_020078867.1	aryl hydrocarbon receptor nuclear translocator-like protein 1 isoform X2	4882	0
XM_020078868.1	aryl hydrocarbon receptor nuclear translocator-like protein 1 isoform X3	4792	0
XM_020078869.1	guanylyl cyclase inhibitory protein-like isoform X1	1302	4.40E-146
XM_020078870.1	guanylyl cyclase inhibitory protein-like isoform X2	1307	5.99E-127
XM_020078871.1	ES1 protein homolog, mitochondrial-like	872	2.45E-165
XM_020078872.1	suppression of tumorigenicity 5 protein	4412	0
XM_020078873.1	SRSF protein kinase 2-like isoform X1	4909	0
XM_020078874.1	SRSF protein kinase 2-like isoform X2	4750	0
XM_020078875.1	SRSF protein kinase 2-like isoform X3	2661	0
XM_020078876.1	uncharacterized aarF domain-containing protein kinase 2	2604	0
XM_020078877.1	PREDICTED: uncharacterized protein C15orf39 homolog	6894	0
XM_020078878.1	E3 ubiquitin-protein ligase ARIH2	3672	0
XM_020078879.1	sorting nexin-1-like	4823	0
XM_020078880.1	synaptic vesicle glycoprotein 2B-like	2325	0
XM_020078881.1	centrosomal protein of 290 kDa isoform X1	9079	0
XM_020078882.1	centrosomal protein of 290 kDa isoform X2	9058	0
XM_020078883.1	P2X purinoceptor 3-like	1746	0
XM_020078884.1	transmembrane and TPR repeat-containing protein 3	4939	0
XM_020078885.1	transmembrane and TPR repeat-containing protein 3	4898	0
XM_020078886.1	PREDICTED: uncharacterized protein C12orf29 homolog isoform X1	1841	0
XM_020078887.1	PREDICTED: uncharacterized protein C12orf29 homolog isoform X2	1839	0
XM_020078888.1	protein zwilch homolog	2575	0
XM_020078889.1	lactase-like protein isoform X1	1740	0
XM_020078890.1	lactase-like protein isoform X2	1719	0
XM_020078891.1	mothers against decapentaplegic homolog 6	2570	0
XM_020078892.1	dual specificity mitogen-activated protein kinase kinase 1	1548	0
XM_020078893.1	60S ribosomal protein L4	1265	0
XM_020078894.1	snRNA-activating protein complex subunit 5	1014	1.34E-36
XM_020078895.1	Bardet-Biedl syndrome 10 protein	2942	0
XM_020078896.1	synaptotagmin-1-like isoform X1	1756	0
XM_020078897.1	synaptotagmin-1-like isoform X1	1783	0
XM_020078898.1	CD9 antigen-like isoform X1	1695	1.77E-136
XM_020078899.1	CD9 antigen-like isoform X2	1634	6.13E-136
XM_020078900.1	PREDICTED: semaphorin-3A	3090	0
XM_020078901.1	transcription factor HES-7-like	1011	4.41E-142
XM_020078902.1	PREDICTED: semaphorin-3A	4466	0
XM_020078903.1	PREDICTED: semaphorin-3D	3189	0
XM_020078904.1	PREDICTED: semaphorin-3D	3184	0
XM_020078905.1	PREDICTED: semaphorin-3D	3274	0
XM_020078906.1	PREDICTED: semaphorin-3D	3081	0
XM_020078907.1	semaphorin-3E isoform X1	4819	0
XM_020078908.1	semaphorin-3E isoform X2	4816	0
XM_020078909.1	chromodomain-helicase-DNA-binding protein 2	9398	0
XM_020078910.1	DNA repair protein RAD52 homolog isoform X1	1691	0
XM_020078911.1	DNA repair protein RAD52 homolog isoform X2	1619	0
XM_020078912.1	proline-serine-threonine phosphatase-interacting protein 1-like	1728	0
XM_020078913.1	transcription factor HES-7.1-B-like	1071	1.46E-147
XM_020078914.1	proline-serine-threonine phosphatase-interacting protein 1-like	1444	0
XM_020078915.1	reticulocalbin-2 isoform X2	2729	0
XM_020078916.1	reticulocalbin-2 isoform X2	2764	0
XM_020078917.1	reticulocalbin-2 isoform X2	2793	0
XM_020078918.1	reticulocalbin-2 isoform X2	2730	0
XM_020078919.1	PREDICTED: tetraspanin-3	1639	5.97E-173
XM_020078920.1	protein C12orf4 homolog	3307	0
XM_020078921.1	protein C12orf4 homolog	3449	0
XM_020078922.1	protein C12orf4 homolog	3380	0
XM_020078923.1	protein C12orf4 homolog	3284	0
XM_020078924.1	transcription factor E2F5-like	3905	0
XM_020078925.1	GTPase HRas	3336	3.63E-113
XM_020078926.1	GTPase HRas	3199	2.50E-102
XM_020078927.1	vesicle transport protein SEC20	2141	1.37E-150
XM_020078928.1	GTPase KRas isoform X3	1903	5.29E-125
XM_020078929.1	F-box/LRR-repeat protein 13 isoform X1	3504	0
XM_020078930.1	F-box/LRR-repeat protein 13 isoform X2	3515	0
XM_020078931.1	F-box/LRR-repeat protein 13 isoform X2	3481	0
XM_020078932.1	leucine-rich repeat-containing protein 17-like	1929	0

XM_020078933.1	protein FAM185A	1538	0
XM_020078934.1	armadillo repeat-containing protein 10	2665	0
XM_020078935.1	vesicle transport protein SEC20	908	8.81E-157
XM_020078936.1	ATP-dependent DNA helicase DDX11-like isoform X1	3458	0
XM_020078937.1	ATP-dependent DNA helicase DDX11-like isoform X1	3435	0
XM_020078938.1	ATP-dependent DNA helicase DDX11-like isoform X1	3449	0
XM_020078939.1	ATP-dependent DNA helicase DDX11-like isoform X1	3446	0
XM_020078940.1	ATP-dependent DNA helicase DDX11-like isoform X1	2933	0
XM_020078941.1	ATP-dependent DNA helicase DDX11-like isoform X5	2921	0
XM_020078942.1	WAS protein family homolog 1	1679	0
XM_020078943.1	peptidyl-prolyl cis-trans isomerase FKBP5-like	2715	0
XM_020078944.1	CUGBP Elav-like family member 2 isoform X1	4364	0
XM_020078945.1	CUGBP Elav-like family member 2 isoform X1	5290	0
XM_020078946.1	CUGBP Elav-like family member 2 isoform X1	4362	0
XM_020078947.1	CUGBP Elav-like family member 2 isoform X1	4362	0
XM_020078948.1	CUGBP Elav-like family member 2 isoform X4	4361	0
XM_020078949.1	protein Wnt-8-like	1243	0
XM_020078950.1	CUGBP Elav-like family member 2 isoform X1	4348	0
XM_020078951.1	CUGBP Elav-like family member 2 isoform X6	4345	0
XM_020078952.1	CUGBP Elav-like family member 2 isoform X1	4099	0
XM_020078953.1	tetratricopeptide repeat protein 23-like	2989	0
XM_020078954.1	inactive serine protease PAMR1 isoform X1	4013	0
XM_020078955.1	inactive serine protease PAMR1 isoform X1	4015	0
XM_020078956.1	four-jointed box protein 1	2370	0
XM_020078957.1	tripartite motif-containing protein 44	2681	0
XM_020078958.1	tripartite motif-containing protein 44	2554	0
XM_020078959.1	tripartite motif-containing protein 44	2520	0
XM_020078960.1	protein MON2 homolog isoform X1	6089	0
XM_020078961.1	protein MON2 homolog isoform X2	5664	0
XM_020078962.1	protein MON2 homolog isoform X3	5655	0
XM_020078963.1	protein MON2 homolog isoform X2	5652	0
XM_020078964.1	protein MON2 homolog isoform X5	5649	0
XM_020078965.1	protein MON2 homolog isoform X6	5634	0
XM_020078966.1	hepatocyte growth factor	2962	0
XM_020078967.1	leucine-rich repeat-containing protein 4-like	3888	0
XM_020078968.1	gamma-tubulin complex component 6	7021	0
XM_020078969.1	gamma-tubulin complex component 6	6260	0
XM_020078970.1	gamma-tubulin complex component 6	7017	0
XM_020078971.1	DCC-interacting protein 13-beta	3054	0
XM_020078972.1	glucose-regulated protein 94	2771	0
XM_020078973.1	5'-nucleotidase domain-containing protein 3	2116	0
XM_020078974.1	Fanconi anemia group I protein	4641	0
XM_020078975.1	DNA polymerase subunit gamma-1 isoform X1	4576	0
XM_020078976.1	DNA polymerase subunit gamma-1 isoform X2	4573	0
XM_020078977.1	Golgi apparatus membrane protein TVP23 homolog A-like	941	1.31E-135
XM_020078978.1	tight junction protein ZO-1-like isoform X1	7412	0
XM_020078979.1	tight junction protein ZO-1-like isoform X2	7410	0
XM_020078980.1	NADH dehydrogenase [ubiquinone] 1 alpha subcomplex subunit 1	476	1.59E-47
XM_020078981.1	tight junction protein ZO-1-like isoform X3	7372	0
XM_020078982.1	tight junction protein ZO-1-like isoform X4	7352	0
XM_020078983.1	tight junction protein ZO-1-like isoform X5	7184	0
XM_020078984.1	tight junction protein ZO-1-like isoform X1	7624	0
XM_020078985.1	tight junction protein ZO-1-like isoform X7	7349	0
XM_020078986.1	dnaJ homolog subfamily A member 2-like	2719	0
XM_020078987.1	transmembrane protein 168-like	4364	0
XM_020078988.1	probable methyltransferase BMT2 homolog	3195	0
XM_020078989.1	kinesin-like protein KIF21A isoform X1	7499	0
XM_020078990.1	kinesin-like protein KIF21A isoform X2	7426	0
XM_020078991.1	kinesin-like protein KIF21A isoform X3	7424	0
XM_020078992.1	kinesin-like protein KIF21A isoform X4	7412	0
XM_020078993.1	kinesin-like protein KIF21A isoform X5	7297	0
XM_020078994.1	kinesin-like protein KIF21A isoform X6	7224	0
XM_020078995.1	dual specificity protein phosphatase 3-like	4335	0
XM_020078996.1	dual specificity protein phosphatase 3-like	4254	0
XM_020078997.1	troponin I, fast skeletal muscle-like	768	2.92E-107
XM_020078998.1	troponin I, fast skeletal muscle-like isoform X1	1428	1.31E-110
XM_020078999.1	troponin I, fast skeletal muscle-like isoform X2	1559	1.26E-109
XM_020079000.1	tropomyosin alpha-1 chain	1776	1.15E-135
XM_020079001.1	regulator of nonsense transcripts 1-like	3343	0
XM_020079002.1	chitinase domain-containing protein 1	1929	0
XM_020079003.1	CD151 antigen-like isoform X1	1919	1.63E-160
XM_020079004.1	CD151 antigen-like isoform X2	1913	7.81E-159
XM_020079005.1	Y+L amino acid transporter 2-like	2070	0
XM_020079006.1	protein eva-1 homolog C-like isoform X1	1942	0
XM_020079007.1	CD151 antigen-like isoform X3	1898	1.34E-154
XM_020079008.1	CD151 antigen-like isoform X1	1876	8.30E-139
XM_020079009.1	Parkinson disease 7 domain-containing protein 1	1155	2.42E-157
XM_020079010.1	calcium-dependent secretion activator 2 isoform X1	7782	0
XM_020079011.1	calcium-dependent secretion activator 2 isoform X2	7662	0

XM_020079012.1	calcium-dependent secretion activator 2 isoform X3	7632	0
XM_020079013.1	septin-7-like isoform X1	3879	0
XM_020079014.1	septin-7-like isoform X2	3877	0
XM_020079015.1	protein eva-1 homolog C-like isoform X2	1906	0
XM_020079016.1	phosphatidylinositol 4-phosphate 3-kinase C2 domain-containing subunit alpha	7258	0
XM_020079017.1	phosphatidylinositol 4-phosphate 3-kinase C2 domain-containing subunit alpha	7571	0
XM_020079018.1	cortactin-binding protein 2	6487	0
XM_020079019.1	transient receptor potential cation channel subfamily M member 1-like	5256	0
XM_020079020.1	ras-related protein Rab-8B	1850	1.10E-146
XM_020079021.1	40S ribosomal protein S27-like isoform X1	591	9.50E-58
XM_020079022.1	40S ribosomal protein S27-like isoform X2	764	5.81E-68
XM_020079023.1	mucin-3A-like isoform X1	3764	0
XM_020079024.1	protein eva-1 homolog C-like isoform X3	1861	0
XM_020079025.1	mucin-3A-like isoform X2	3737	0
XM_020079026.1	non-muscle caldesmon-like isoform X1	2034	8.17E-175
XM_020079027.1	non-muscle caldesmon-like isoform X2	2032	2.78E-174
XM_020079028.1	protein groucho-2 isoform X1	3970	0
XM_020079029.1	protein groucho-2 isoform X2	3970	0
XM_020079030.1	protein groucho-2 isoform X3	3942	0
XM_020079031.1	protein groucho-2 isoform X4	3942	0
XM_020079032.1	guanine nucleotide-binding protein G(i) subunit alpha-2	2553	0
XM_020079033.1	PHD and RING finger domain-containing protein 1 isoform X1	6239	0
XM_020079034.1	PHD and RING finger domain-containing protein 1 isoform X1	6271	0
XM_020079035.1	PHD and RING finger domain-containing protein 1 isoform X1	6230	0
XM_020079036.1	interferon regulatory factor 7	1995	0
XM_020079037.1	type 2 DNA topoisomerase 6 subunit B-like isoform X1	1802	0
XM_020079038.1	interferon regulatory factor 7	1184	0
XM_020079039.1	early endosome antigen 1 isoform X1	6071	0
XM_020079040.1	early endosome antigen 1 isoform X2	6056	0
XM_020079041.1	early endosome antigen 1 isoform X3	5680	0
XM_020079042.1	diphosphoinositol polyphosphate phosphohydrolase 2-like	1797	1.69E-87
XM_020079043.1	ELKS/Rab6-interacting/CAST family member 1 isoform X1	6852	0
XM_020079044.1	ELKS/Rab6-interacting/CAST family member 1 isoform X2	6839	0
XM_020079045.1	matrix metalloproteinase-16-like	4909	0
XM_020079046.1	type 2 DNA topoisomerase 6 subunit B-like isoform X2	1793	0
XM_020079047.1	tripartite motif-containing protein 16-like	2811	0
XM_020079048.1	very long-chain acyl-CoA synthetase-like	2872	0
XM_020079049.1	very long-chain acyl-CoA synthetase-like	3348	0
XM_020079050.1	troponin T, fast skeletal muscle isoforms-like isoform X7	1387	7.16E-77
XM_020079051.1	troponin T, fast skeletal muscle isoforms-like isoform X7	1378	6.57E-77
XM_020079052.1	troponin T, fast skeletal muscle isoforms-like isoform X7	1368	6.03E-77
XM_020079053.1	troponin T, fast skeletal muscle isoforms-like isoform X4	1359	5.42E-109
XM_020079054.1	troponin T, fast skeletal muscle isoforms-like isoform X5	1226	2.78E-117
XM_020079055.1	type 2 DNA topoisomerase 6 subunit B-like isoform X3	1790	0
XM_020079056.1	troponin T, fast skeletal muscle isoforms-like isoform X6	1207	2.11E-118
XM_020079057.1	troponin T, fast skeletal muscle isoforms-like isoform X7	1197	1.73E-129
XM_020079058.1	microtubule-associated protein futsch-like	16475	0
XM_020079059.1	unconventional myosin-IXa-like isoform X1	8207	0
XM_020079060.1	unconventional myosin-IXa-like isoform X2	8144	0
XM_020079061.1	A disintegrin and metalloproteinase with thrombospondin motifs 20	9325	0
XM_020079062.1	PREDICTED: plexin-B2	8020	0
XM_020079063.1	type 2 DNA topoisomerase 6 subunit B-like isoform X4	1718	0
XM_020079064.1	tyrosine-protein phosphatase non-receptor type 9 isoform X1	5462	0
XM_020079065.1	tyrosine-protein phosphatase non-receptor type 9 isoform X2	5373	0
XM_020079066.1	centrosomal protein of 152 kDa	6610	0
XM_020079067.1	N-acetylgalactosamine kinase isoform X1	1966	0
XM_020079068.1	N-acetylgalactosamine kinase isoform X2	1877	0
XM_020079069.1	C-Jun-amino-terminal kinase-interacting protein 2	7479	0
XM_020079070.1	pepsin A-like	1155	0
XM_020079071.1	potassium voltage-gated channel subfamily C member 1 isoform X1	3953	0
XM_020079072.1	potassium voltage-gated channel subfamily C member 1 isoform X2	2863	0
XM_020079073.1	potassium voltage-gated channel subfamily C member 1 isoform X3	2558	0
XM_020079074.1	potassium voltage-gated channel subfamily C member 1 isoform X4	2521	0
XM_020079075.1	secretion-regulating guanine nucleotide exchange factor	2131	0
XM_020079076.1	solute carrier family 25 member 53	1859	0
XM_020079077.1	CD81 antigen-like	1597	2.16E-171
XM_020079078.1	HMG box-containing protein 1	2694	0
XM_020079079.1	HMG box-containing protein 1	2759	0
XM_020079080.1	probable G-protein coupled receptor 22 isoform X1	3579	0
XM_020079081.1	cAMP-dependent protein kinase type II-alpha regulatory subunit	2818	0
XM_020079082.1	solute carrier family 25 member 53	1863	0
XM_020079083.1	DNA-binding protein RFX7-like	7392	0
XM_020079084.1	pseudouridine-metabolizing bifunctional protein C1861.05-like isoform X2	2472	0
XM_020079085.1	pseudouridine-metabolizing bifunctional protein C1861.05-like isoform X2	2442	0
XM_020079086.1	pseudouridine-metabolizing bifunctional protein C1861.05-like isoform X2	2365	0
XM_020079087.1	troponin I, slow skeletal muscle-like	1174	7.06E-103
XM_020079088.1	troponin I, slow skeletal muscle-like	905	1.71E-104
XM_020079089.1	troponin I, slow skeletal muscle-like	1149	6.70E-83
XM_020079090.1	troponin I, slow skeletal muscle-like	760	1.26E-92

XM_020079091.1	troponin I, slow skeletal muscle-like	756	2.49E-89
XM_020079092.1	troponin I, slow skeletal muscle-like	1064	4.56E-126
XM_020079093.1	spondin-2-like isoform X1	2050	0
XM_020079094.1	AFG3-like protein 1	4243	0
XM_020079095.1	filamin-C-like isoform X1	9073	0
XM_020079096.1	filamin-C-like isoform X2	8956	0
XM_020079097.1	dynamin-1-like protein isoform X1	2759	0
XM_020079098.1	dynamin-1-like protein isoform X2	2688	0
XM_020079099.1	troponin T, fast skeletal muscle-like isoform X3	1025	6.50E-92
XM_020079100.1	troponin T, fast skeletal muscle-like isoform X3	1015	5.87E-92
XM_020079101.1	RNA polymerase II elongation factor ELL-like	5507	0
XM_020079102.1	inactive peptidyl-prolyl cis-trans isomerase FKBP6	1743	0
XM_020079103.1	disintegrin and metalloproteinase domain-containing protein 10-like	5817	0
XM_020079104.1	neuronal cell adhesion molecule-like isoform X1	6195	0
XM_020079105.1	homeobox protein MSX-2-like	1920	8.81E-165
XM_020079106.1	neuronal cell adhesion molecule-like isoform X2	6183	0
XM_020079107.1	neuronal cell adhesion molecule-like isoform X3	6165	0
XM_020079108.1	neuronal cell adhesion molecule-like isoform X4	6159	0
XM_020079109.1	neuronal cell adhesion molecule-like isoform X5	6153	0
XM_020079110.1	neuronal cell adhesion molecule-like isoform X6	6138	0
XM_020079111.1	neuronal cell adhesion molecule-like isoform X7	6135	0
XM_020079112.1	neuronal cell adhesion molecule-like isoform X8	6099	0
XM_020079113.1	neuronal cell adhesion molecule-like isoform X9	6096	0
XM_020079114.1	neuronal cell adhesion molecule-like isoform X10	6093	0
XM_020079115.1	neuronal cell adhesion molecule-like isoform X11	6042	0
XM_020079116.1	neuronal cell adhesion molecule-like isoform X12	5982	0
XM_020079117.1	neuronal cell adhesion molecule-like isoform X13	5922	0
XM_020079118.1	neuronal cell adhesion molecule-like isoform X14	5817	0
XM_020079119.1	neuronal cell adhesion molecule-like isoform X15	5775	0
XM_020079120.1	neuronal cell adhesion molecule-like isoform X16	5760	0
XM_020079121.1	lactase-phlorizin hydrolase-like	3756	0
XM_020079122.1	neuronal cell adhesion molecule-like isoform X17	5640	0
XM_020079123.1	calumenin	2338	0
XM_020079124.1	calumenin	2333	0
XM_020079125.1	calumenin	2346	0
XM_020079126.1	calumenin	2335	0
XM_020079127.1	calumenin	2335	0
XM_020079128.1	calumenin	2308	0
XM_020079129.1	general transcription factor 3C polypeptide 6 isoform X1	1458	1.60E-90
XM_020079130.1	general transcription factor 3C polypeptide 6 isoform X2	1455	4.21E-75
XM_020079131.1	BCL2/adenovirus E1B 19 kDa protein-interacting protein 2 isoform X1	2469	0
XM_020079132.1	BCL2/adenovirus E1B 19 kDa protein-interacting protein 2 isoform X2	2433	0
XM_020079133.1	BCL2/adenovirus E1B 19 kDa protein-interacting protein 2 isoform X3	1792	0
XM_020079134.1	beta-1,3-galactosyl-O-glycosyl-glycoprotein beta-1,6-N-acetylglucosaminyltransferase 3	2056	0
XM_020079135.1	serine/threonine-protein phosphatase 2B catalytic subunit alpha isoform-like isoform X1	3465	0
XM_020079136.1	melanin-concentrating hormone receptor 2	1581	0
XM_020079137.1	troponin I, fast skeletal muscle-like	709	2.81E-108
XM_020079138.1	troponin I, fast skeletal muscle-like	605	7.15E-109
XM_020079139.1	troponin I, fast skeletal muscle-like	940	1.78E-120
XM_020079140.1	troponin I, fast skeletal muscle-like	861	6.46E-121
XM_020079141.1	troponin I, fast skeletal muscle-like	860	6.46E-121
XM_020079142.1	troponin I, fast skeletal muscle-like	962	3.75E-105
XM_020079143.1	carboxypeptidase A1	1415	0
XM_020079144.1	prickle-like protein 2 isoform X1	4334	0
XM_020079145.1	immunoglobulin superfamily containing leucine-rich repeat protein 2	3719	0
XM_020079146.1	stimulated by retinoic acid gene 6 protein homolog	3642	0
XM_020079147.1	stimulated by retinoic acid gene 6 protein homolog	3558	0
XM_020079148.1	stimulated by retinoic acid gene 6 protein homolog	3742	0
XM_020079149.1	fibroblast growth factor 20-like	1939	2.42E-145
XM_020079150.1	coiled-coil domain-containing protein 33	1673	0
XM_020079151.1	coiled-coil domain-containing protein 33	1387	0
XM_020079152.1	RING finger and SPRY domain-containing protein 1	3776	0
XM_020079153.1	ADP-ribosylation factor-like protein 2-binding protein	2199	2.86E-86
XM_020079154.1	inositol hexakisphosphate and diphosphoinositol-pentakisphosphate kinase 1-like isoform X1	6405	0
XM_020079155.1	inositol hexakisphosphate and diphosphoinositol-pentakisphosphate kinase 1-like isoform X2	6342	0
XM_020079156.1	inositol hexakisphosphate and diphosphoinositol-pentakisphosphate kinase 1-like isoform X3	4081	0
XM_020079157.1	fatty acyl-CoA hydrolase precursor, medium chain-like	2178	0
XM_020079158.1	F-box only protein 31	3529	0
XM_020079159.1	protein arginine N-methyltransferase 7	2423	0
XM_020079160.1	anamorsin isoform X2	1523	0
XM_020079161.1	cysteinyl leukotriene receptor 2-like	2141	0
XM_020079162.1	anamorsin isoform X2	2033	4.50E-170
XM_020079163.1	anamorsin isoform X2	1831	1.03E-170
XM_020079164.1	anamorsin isoform X2	1519	2.75E-172
XM_020079165.1	anamorsin isoform X2	1656	2.58E-171
XM_020079166.1	anamorsin isoform X2	1545	2.04E-172
XM_020079167.1	paired amphipathic helix protein Sin3a	6127	0
XM_020079168.1	paired amphipathic helix protein Sin3a	5605	0
XM_020079169.1	paired amphipathic helix protein Sin3a	5638	0

XM_020079170.1	paired amphipathic helix protein Sin3a	6260	0
XM_020079171.1	cysteinyl leukotriene receptor 2-like	2138	0
XM_020079172.1	protein TASOR isoform X4	9290	0
XM_020079173.1	protein TASOR isoform X4	9218	0
XM_020079174.1	protogenin B-like	5566	0
XM_020079175.1	probable ribosome biogenesis protein RLP24	931	3.82E-119
XM_020079176.1	iron-responsive element-binding protein 2	3656	0
XM_020079177.1	bromodomain-containing protein 1-like isoform X1	3750	0
XM_020079178.1	bromodomain-containing protein 1-like isoform X2	4944	0
XM_020079179.1	bromodomain-containing protein 1-like isoform X3	5041	0
XM_020079180.1	transmembrane protein 266	3707	0
XM_020079181.1	insulin gene enhancer protein ISL-2A isoform X1	1943	0
XM_020079182.1	insulin gene enhancer protein ISL-2B isoform X2	1938	0
XM_020079183.1	insulin gene enhancer protein ISL-2A isoform X1	1836	0
XM_020079184.1	platelet-derived growth factor receptor-like protein	1368	0
XM_020079185.1	electron transfer flavoprotein subunit alpha, mitochondrial	1500	0
XM_020079186.1	lamina-associated polypeptide 2-like	3062	0
XM_020079187.1	catenin delta-2-like isoform X4	2876	0
XM_020079188.1	single Ig IL-1-related receptor	2210	0
XM_020079189.1	twinfilin-2-like isoform X1	1207	0
XM_020079190.1	interleukin-1 receptor-associated kinase 4 isoform X1	1921	0
XM_020079191.1	interleukin-1 receptor-associated kinase 4 isoform X2	1903	0
XM_020079192.1	ubiquitin-conjugating enzyme E2 Q2-like isoform X1	3633	0
XM_020079193.1	ubiquitin-conjugating enzyme E2 Q2-like isoform X1	3632	0
XM_020079194.1	ubiquitin-conjugating enzyme E2 Q2-like isoform X1	1439	0
XM_020079195.1	ubiquitin-conjugating enzyme E2 Q2-like isoform X1	3631	0
XM_020079196.1	protein Wnt-8a-like	1412	0
XM_020079197.1	ceramide kinase-like	5847	0
XM_020079198.1	UDP-xylose and UDP-N-acetylglucosamine transporter	3450	0
XM_020079199.1	vesicle transport protein GOT1B	1136	1.86E-61
XM_020079200.1	phosphatidylserine synthase 1	4534	0
XM_020079201.1	dual specificity protein phosphatase 6	3165	0
XM_020079202.1	lactadherin-like isoform X1	4253	0
XM_020079203.1	EGF-like repeat and discoidin I-like domain-containing protein 3 isoform X2	4235	0
XM_020079204.1	EGF-like repeat and discoidin I-like domain-containing protein 3 isoform X3	4220	0
XM_020079205.1	EGF-like repeat and discoidin I-like domain-containing protein 3 isoform X4	4202	0
XM_020079206.1	potassium voltage-gated channel subfamily KQT member 1-like isoform X1	4322	0
XM_020079207.1	potassium voltage-gated channel subfamily KQT member 1-like isoform X1	4178	0
XM_020079208.1	potassium voltage-gated channel subfamily KQT member 1-like isoform X1	4333	0
XM_020079209.1	homeobox protein Nkx-2.5	1310	0
XM_020079210.1	potassium voltage-gated channel subfamily KQT member 1-like isoform X1	4240	0
XM_020079211.1	potassium voltage-gated channel subfamily KQT member 1-like isoform X1	2639	0
XM_020079212.1	28S ribosomal protein S35, mitochondrial	1362	0
XM_020079213.1	patatin-like phospholipase domain-containing protein 3	1661	0
XM_020079214.1	nucleobindin-2 isoform X1	2597	0
XM_020079215.1	nucleobindin-2 isoform X1	2550	0
XM_020079216.1	nucleobindin-2 isoform X1	2515	0
XM_020079217.1	UPF0676 protein C1494.01-like	1742	0
XM_020079218.1	CCR4-NOT transcription complex subunit 4 isoform X1	4405	0
XM_020079219.1	CCR4-NOT transcription complex subunit 4 isoform X2	4386	0
XM_020079220.1	zinc finger homeobox protein 3	11727	0
XM_020079221.1	serine/threonine-protein phosphatase 2B catalytic subunit alpha isoform-like isoform X2	3285	0
XM_020079222.1	protein atonal homolog 7-like	1239	8.53E-133
XM_020079223.1	putative cation exchanger C521.04c isoform X1	2894	0
XM_020079224.1	putative cation exchanger C521.04c isoform X2	2888	0
XM_020079225.1	kelch domain-containing protein 3	2519	0
XM_020079226.1	complement C1q tumor necrosis factor-related protein 4-like isoform X1	2042	0
XM_020079227.1	complement C1q tumor necrosis factor-related protein 4-like isoform X2	1952	0
XM_020079228.1	ubiquitin-conjugating enzyme E2 N-like	1890	1.96E-91
XM_020079229.1	pleckstrin homology domain-containing family A member 5-like isoform X1	4788	0
XM_020079230.1	pleckstrin homology domain-containing family A member 5-like isoform X2	4761	0
XM_020079231.1	pleckstrin homology domain-containing family A member 5-like isoform X3	4688	0
XM_020079232.1	pleckstrin homology domain-containing family A member 5-like isoform X4	4150	0
XM_020079233.1	pleckstrin homology domain-containing family A member 5-like isoform X5	939	7.85E-80
XM_020079234.1	pleckstrin homology domain-containing family A member 5-like isoform X6	4935	0
XM_020079235.1	selenoprotein H	484	1.73E-59
XM_020079236.1	von Willebrand factor A domain-containing protein 9	2207	0
XM_020079237.1	very-long-chain (3R)-3-hydroxyacyl-CoA dehydratase 3	1802	0
XM_020079238.1	FERM domain-containing protein 4A isoform X1	6501	0
XM_020079239.1	FERM domain-containing protein 4A isoform X1	6573	0
XM_020079240.1	FERM domain-containing protein 4A isoform X3	6706	0
XM_020079241.1	FERM domain-containing protein 4A isoform X4	6708	0
XM_020079242.1	FERM domain-containing protein 4A isoform X5	6701	0
XM_020079243.1	FERM domain-containing protein 4A isoform X6	6496	0
XM_020079244.1	FERM domain-containing protein 4A isoform X6	6902	0
XM_020079245.1	FERM domain-containing protein 4A isoform X8	6613	0
XM_020079246.1	FERM domain-containing protein 4A isoform X3	6383	0
XM_020079247.1	FERM domain-containing protein 4A isoform X8	6087	0
XM_020079248.1	alpha-2,8-sialyltransferase 8B isoform X1	4748	0

XM_020079249.1	ectonucleotide pyrophosphatase/phosphodiesterase family member 6-like	587	9.26E-107
XM_020079250.1	alpha-2,8-sialyltransferase 8B isoform X2	4745	0
XM_020079251.1	membrane protein FAM174B-like	2250	4.27E-81
XM_020079252.1	membrane protein FAM174B-like	2138	4.91E-81
XM_020079253.1	RNA cytidine acetyltransferase	3891	0
XM_020079254.1	RNA cytidine acetyltransferase	3850	0
XM_020079255.1	RNA cytidine acetyltransferase	3847	0
XM_020079256.1	adenosine deaminase-like protein	1265	0
XM_020079257.1	zinc finger protein 469	13824	0
XM_020079258.1	zinc finger protein 469	13685	0
XM_020079259.1	2-oxoglutarate dehydrogenase-like, mitochondrial	3762	0
XM_020079260.1	DNA topoisomerase I, mitochondrial-like	3295	0
XM_020079261.1	heat shock factor-binding protein 1	895	1.33E-36
XM_020079262.1	kelch repeat and BTB domain-containing protein 4	2267	0
XM_020079263.1	43 kDa receptor-associated protein of the synapse isoform X1	2858	0
XM_020079264.1	43 kDa receptor-associated protein of the synapse isoform X1	2350	0
XM_020079265.1	ankyrin repeat domain-containing protein 26-like isoform X1	8427	0
XM_020079266.1	ankyrin repeat domain-containing protein 26-like isoform X1	8025	0
XM_020079267.1	ankyrin repeat domain-containing protein 26-like isoform X1	8409	0
XM_020079268.1	platelet-derived growth factor receptor-like protein isoform X1	1639	0
XM_020079269.1	ankyrin repeat domain-containing protein 26-like isoform X1	8212	0
XM_020079270.1	ankyrin repeat domain-containing protein 26-like isoform X1	7532	0
XM_020079271.1	ornithine decarboxylase antizyme 2-like	2972	6.30E-151
XM_020079272.1	dihydrolipoyl dehydrogenase, mitochondrial	2842	0
XM_020079273.1	RGM domain family member B-like	4087	0
XM_020079274.1	phospholipid-transporting ATPase ID-like	3828	0
XM_020079275.1	platelet-derived growth factor receptor-like protein isoform X2	1624	0
XM_020079276.1	CTD small phosphatase-like protein 2	3246	0
XM_020079277.1	CTD small phosphatase-like protein 2	3243	0
XM_020079278.1	sulfide:quinone oxidoreductase, mitochondrial	1436	0
XM_020079279.1	isocitrate dehydrogenase [NADP], mitochondrial	1819	0
XM_020079280.1	PREDICTED: treslin	6642	0
XM_020079281.1	solute carrier family 45 member 3	4633	0
XM_020079282.1	solute carrier family 45 member 3	4648	0
XM_020079283.1	Krueppel-like factor 13	7867	1.64E-157
XM_020079284.1	pleckstrin homology domain-containing family A member 7-like isoform X1	4811	0
XM_020079285.1	pleckstrin homology domain-containing family A member 7-like isoform X2	4811	0
XM_020079286.1	pleckstrin homology domain-containing family A member 7-like isoform X3	4751	0
XM_020079287.1	pleckstrin homology domain-containing family A member 7-like isoform X2	4031	0
XM_020079288.1	pleckstrin homology domain-containing family A member 7-like isoform X5	4743	0
XM_020079289.1	pleckstrin homology domain-containing family A member 7-like isoform X6	4481	0
XM_020079290.1	pleckstrin homology domain-containing family A member 7-like isoform X7	4568	0
XM_020079291.1	periphilin-1-like isoform X5	1973	0
XM_020079292.1	periphilin-1-like isoform X5	1821	0
XM_020079293.1	periphilin-1-like isoform X5	1820	0
XM_020079294.1	YY1-associated factor 2	2329	1.45E-97
XM_020079295.1	ADP-ribosylation factor 4-like	1894	2.90E-126
XM_020079296.1	malate synthase, glyoxysomal-like	1923	0
XM_020079297.1	cyclin-dependent kinase 17-like	3291	0
XM_020079298.1	transcription initiation factor TFIID subunit 3	3145	0
XM_020079299.1	monoacylglycerol lipase ABHD2-A	4218	0
XM_020079300.1	retinaldehyde-binding protein 1	1576	0
XM_020079301.1	retinaldehyde-binding protein 1	1557	0
XM_020079302.1	peroxisome proliferator-activated receptor alpha-like	5853	0
XM_020079303.1	mitochondrial glutamate carrier 1-like	5101	0
XM_020079304.1	serine/threonine-protein phosphatase 2B catalytic subunit alpha isoform-like isoform X3	3418	0
XM_020079305.1	alpha-mannosidase 2C1 isoform X1	3334	0
XM_020079306.1	alpha-mannosidase 2C1 isoform X1	2884	0
XM_020079307.1	endonuclease 8-like 1 isoform X1	1557	0
XM_020079308.1	endonuclease 8-like 1 isoform X2	1555	0
XM_020079309.1	NADH dehydrogenase [ubiquinone] 1 subunit C1, mitochondrial	431	3.17E-45
XM_020079310.1	ADP-dependent glucokinase isoform X1	3301	0
XM_020079311.1	ADP-dependent glucokinase isoform X2	3298	0
XM_020079312.1	membrane-associated tyrosine- and threonine-specific cdc2-inhibitory kinase	3381	0
XM_020079313.1	insulin-like growth factor 1 receptor	5838	0
XM_020079314.1	PREDICTED: catalase	2884	0
XM_020079315.1	protein unc-45 homolog A	3062	0
XM_020079316.1	RCC1 domain-containing protein 1 isoform X1	1339	0
XM_020079317.1	RCC1 domain-containing protein 1 isoform X2	1310	0
XM_020079318.1	NADH dehydrogenase [ubiquinone] 1 subunit C1, mitochondrial	437	3.42E-45
XM_020079319.1	selenide, water dikinase 1	2594	0
XM_020079320.1	selenide, water dikinase 1	2792	0
XM_020079321.1	selenide, water dikinase 1	2382	0
XM_020079322.1	TBC1 domain family member 2B	5000	0
XM_020079323.1	SH2 domain-containing protein 7-like	1910	0
XM_020079324.1	SH2 domain-containing protein 7-like	1817	0
XM_020079325.1	zinc finger protein 592	5895	0
XM_020079326.1	zinc finger protein 592	5915	0
XM_020079327.1	zinc finger protein 592	5853	0

XM_020079328.1	protein FAM107B isoform X1	2009	1.46E-82
XM_020079329.1	protein FAM107B isoform X1	2040	2.78E-70
XM_020079330.1	caprin-1 isoform X1	2719	0
XM_020079331.1	ADP-ribosylation factor-like protein 3	1050	2.64E-112
XM_020079332.1	caprin-1 isoform X2	2716	0
XM_020079333.1	caprin-1 isoform X3	2704	0
XM_020079334.1	caprin-1 isoform X4	2701	0
XM_020079335.1	transmembrane and coiled-coil domains protein 3-like	3781	0
XM_020079336.1	ras association domain-containing protein 8-like isoform X1	3609	0
XM_020079337.1	ras association domain-containing protein 8-like isoform X2	3603	0
XM_020079338.1	PREDICTED: uncharacterized protein LOC109624569 isoform X1	5226	0
XM_020079339.1	PREDICTED: uncharacterized protein LOC109624569 isoform X2	5223	0
XM_020079340.1	PREDICTED: uncharacterized protein LOC109624569 isoform X3	5223	0
XM_020079341.1	PREDICTED: uncharacterized protein LOC109624569 isoform X4	5224	0
XM_020079342.1	PREDICTED: uncharacterized protein LOC109624569 isoform X5	5220	0
XM_020079343.1	PREDICTED: uncharacterized protein LOC109624569 isoform X6	5171	0
XM_020079344.1	anillin-like isoform X7	3860	0
XM_020079345.1	D(1) dopamine receptor-like	1134	0
XM_020079346.1	anillin-like isoform X8	3858	0
XM_020079347.1	AT-rich interactive domain-containing protein 3A-like	3212	0
XM_020079348.1	mannose-6-phosphate isomerase	4155	0
XM_020079349.1	inner centromere protein isoform X1	3138	0
XM_020079350.1	inner centromere protein isoform X2	3121	0
XM_020079351.1	synembryn-B isoform X1	3397	0
XM_020079352.1	synembryn-B isoform X2	1626	0
XM_020079353.1	suppressor of tumorigenicity 7 protein isoform X1	2312	0
XM_020079354.1	suppressor of tumorigenicity 7 protein isoform X2	2267	0
XM_020079355.1	suppressor of tumorigenicity 7 protein isoform X3	2265	0
XM_020079356.1	suppressor of tumorigenicity 7 protein isoform X4	2259	0
XM_020079357.1	suppressor of tumorigenicity 7 protein isoform X5	2252	0
XM_020079358.1	suppressor of tumorigenicity 7 protein isoform X6	2250	0
XM_020079359.1	LRRN4 C-terminal-like protein	1234	2.71E-156
XM_020079360.1	suppressor of tumorigenicity 7 protein isoform X1	2238	0
XM_020079361.1	pyruvate dehydrogenase (acetyl-transferring) kinase isozyme 2, mitochondrial-like	2770	0
XM_020079362.1	protein disulfide-isomerase A3	2207	0
XM_020079363.1	fibulin-1-like isoform X1	2901	0
XM_020079364.1	fibulin-1-like isoform X2	3159	0
XM_020079365.1	talin-2 isoform X1	9763	0
XM_020079366.1	talin-2 isoform X2	9760	0
XM_020079367.1	talin-2 isoform X3	9757	0
XM_020079368.1	LRRN4 C-terminal-like protein	1206	1.46E-156
XM_020079369.1	talin-2 isoform X4	9739	0
XM_020079370.1	talin-2 isoform X5	9733	0
XM_020079371.1	talin-2 isoform X6	9661	0
XM_020079372.1	ubiquitin-conjugating enzyme E2 H	3117	1.99E-105
XM_020079373.1	F-actin-capping protein subunit alpha-1-like	1951	0
XM_020079374.1	alpha-mannosidase 2x	5228	0
XM_020079375.1	alpha-mannosidase 2x	5238	0
XM_020079376.1	tumor protein p53-inducible protein 11	3241	1.80E-107
XM_020079377.1	tumor protein p53-inducible protein 11	3276	2.18E-107
XM_020079378.1	reelin isoform X1	11804	0
XM_020079379.1	reelin isoform X2	12066	0
XM_020079380.1	lipase maturation factor 2	3114	0
XM_020079381.1	putative methyltransferase NSUN7	2798	0
XM_020079382.1	inositol oxygenase	1658	0
XM_020079383.1	pancreatic secretory granule membrane major glycoprotein GP2-like	1477	0
XM_020079384.1	sorting nexin-18-like	5989	0
XM_020079385.1	prickle-like protein 1	2853	0
XM_020079386.1	prickle-like protein 1	2907	0
XM_020079387.1	prickle-like protein 1	3162	0
XM_020079388.1	C-myc promoter-binding protein isoform X1	7092	0
XM_020079389.1	C-myc promoter-binding protein isoform X2	6831	0
XM_020079390.1	contactin-2 isoform X1	5735	0
XM_020079391.1	contactin-2 isoform X1	5706	0
XM_020079392.1	contactin-2 isoform X1	5726	0
XM_020079393.1	NK1 transcription factor-related protein 1	2150	0
XM_020079394.1	kinesin-like protein KIF23 isoform X1	3550	0
XM_020079395.1	kinesin-like protein KIF23 isoform X2	3223	0
XM_020079396.1	ferritin, heavy subunit-like	1377	7.04E-129
XM_020079397.1	very-long-chain 3-oxoacyl-CoA reductase-A-like	2396	0
XM_020079398.1	potassium voltage-gated channel subfamily A member 1-like	4994	0
XM_020079399.1	shaker-related potassium channel tsha2-like	2294	0
XM_020079400.1	parathyroid hormone-related protein-like	1445	1.28E-122
XM_020079401.1	alpha-aminoadipic semialdehyde synthase, mitochondrial	3105	0
XM_020079402.1	alpha-aminoadipic semialdehyde synthase, mitochondrial	3136	0
XM_020079403.1	PDZ domain-containing RING finger protein 4-like	5050	0
XM_020079404.1	thiosulfate sulfurtransferase/rhodanese-like domain-containing protein 1	512	7.60E-86
XM_020079405.1	Bloom syndrome protein isoform X1	4699	0
XM_020079406.1	Bloom syndrome protein isoform X2	4663	0

XM_020079407.1	Bloom syndrome protein isoform X3	4609	0
XM_020079408.1	zinc finger protein 143	2044	0
XM_020079409.1	zinc finger protein 143	2040	0
XM_020079410.1	DNA-directed RNA polymerase III subunit RPC2	3767	0
XM_020079411.1	DENN domain-containing protein 5B-like	3956	0
XM_020079412.1	serine/threonine-protein kinase pim-3-like	3428	0
XM_020079413.1	peptidyl-prolyl cis-trans isomerase B isoform X1	1069	2.66E-143
XM_020079414.1	peptidyl-prolyl cis-trans isomerase B isoform X2	697	3.14E-123
XM_020079415.1	sodium- and chloride-dependent GABA transporter 2-like	2433	0
XM_020079416.1	ladderlectin-like isoform X1	1050	2.63E-129
XM_020079417.1	nuclear receptor subfamily 2 group C member 1-like isoform X1	2959	0
XM_020079418.1	nuclear receptor subfamily 2 group C member 1-like isoform X2	2927	0
XM_020079419.1	nuclear receptor ROR-alpha isoform X1	3880	0
XM_020079420.1	nuclear receptor ROR-alpha isoform X2	3599	0
XM_020079421.1	PREDICTED: caveolin-1	2641	3.88E-124
XM_020079422.1	neural Wiskott-Aldrich syndrome protein isoform X1	2679	0
XM_020079423.1	neural Wiskott-Aldrich syndrome protein isoform X2	2656	0
XM_020079424.1	apoptosis inhibitor 5 isoform X1	2371	0
XM_020079425.1	ladderlectin-like isoform X2	1047	1.72E-128
XM_020079426.1	apoptosis inhibitor 5 isoform X2	2335	0
XM_020079427.1	neuroepithelial cell-transforming gene 1 protein-like	2047	0
XM_020079428.1	PREDICTED: G2 and S phase-expressed protein 1 isoform X1	2652	0
XM_020079429.1	PREDICTED: G2 and S phase-expressed protein 1 isoform X1	2653	0
XM_020079430.1	PREDICTED: G2 and S phase-expressed protein 1 isoform X2	2649	0
XM_020079431.1	Hermansky-Pudlak syndrome 5 protein	3945	0
XM_020079432.1	Hermansky-Pudlak syndrome 5 protein	3938	0
XM_020079433.1	transcription factor RFX4 isoform X1	3619	0
XM_020079434.1	vascular endothelial growth factor receptor 3	4946	0
XM_020079435.1	transcription factor RFX4 isoform X2	3617	0
XM_020079436.1	transcription factor RFX4 isoform X3	3579	0
XM_020079437.1	transcription factor RFX4 isoform X4	3577	0
XM_020079438.1	metabotropic glutamate receptor 8-like	3277	0
XM_020079439.1	rod cGMP-specific 3',5'-cyclic phosphodiesterase subunit beta	2596	0
XM_020079440.1	engulfment and cell motility protein 3-like isoform X1	4022	0
XM_020079441.1	engulfment and cell motility protein 3-like isoform X2	2525	4.37E-152
XM_020079442.1	F-box/LRR-repeat protein 14-like	2682	0
XM_020079443.1	MICOS complex subunit MIC19 isoform X2	2875	1.03E-143
XM_020079444.1	1-aminocyclopropane-1-carboxylate synthase-like protein 1 isoform X1	3897	0
XM_020079445.1	1-aminocyclopropane-1-carboxylate synthase-like protein 1 isoform X1	3901	0
XM_020079446.1	1-aminocyclopropane-1-carboxylate synthase-like protein 1 isoform X1	3792	0
XM_020079447.1	1-aminocyclopropane-1-carboxylate synthase-like protein 1 isoform X1	3576	0
XM_020079448.1	E3 SUMO-protein ligase PIAS1 isoform X2	2207	0
XM_020079449.1	E3 SUMO-protein ligase PIAS1 isoform X2	2256	0
XM_020079450.1	E3 SUMO-protein ligase PIAS1 isoform X2	3083	0
XM_020079451.1	E3 SUMO-protein ligase PIAS1 isoform X2	2093	0
XM_020079452.1	E3 SUMO-protein ligase PIAS1 isoform X2	2079	0
XM_020079453.1	T-box transcription factor TBX22	2146	0
XM_020079454.1	pyruvate dehydrogenase protein X component, mitochondrial	2161	0
XM_020079455.1	proteasome subunit alpha type-1	1131	0
XM_020079456.1	metabotropic glutamate receptor 3-like	3744	0
XM_020079457.1	metabotropic glutamate receptor 3-like	3717	0
XM_020079458.1	UPF0577 protein KIAA1324-like homolog	3822	0
XM_020079459.1	probable fructose-2,6-bisphosphatase TIGAR A	1825	6.55E-171
XM_020079460.1	fibroblast growth factor 23-like	2558	1.31E-173

XM_020079461.1	fibroblast growth factor 6-like	2825	1.60E-132
XM_020079462.1	hepatocyte growth factor receptor	7655	0
XM_020079463.1	sodium-dependent neutral amino acid transporter SLC6A17 isoform X1	3516	0
XM_020079464.1	homeobox protein CDX-1	1963	3.57E-145
XM_020079465.1	G/T mismatch-specific thymine DNA glycosylase-like	2364	0
XM_020079466.1	G/T mismatch-specific thymine DNA glycosylase-like	2267	0
XM_020079467.1	cadherin-13 isoform X1	3839	0
XM_020079468.1	cadherin-13 isoform X1	3547	0
XM_020079469.1	proton myo-inositol cotransporter-like isoform X1	2020	0
XM_020079470.1	proton myo-inositol cotransporter-like isoform X2	2698	0
XM_020079471.1	proton myo-inositol cotransporter-like isoform X3	2566	0
XM_020079472.1	transformer-2 protein homolog beta isoform X4	2993	0
XM_020079473.1	transformer-2 protein homolog beta isoform X4	2991	0
XM_020079474.1	ubiquitin carboxyl-terminal hydrolase 3	4009	0
XM_020079475.1	cadherin-4-like isoform X2	3143	0
XM_020079476.1	pyruvate dehyrogenase phosphatase catalytic subunit 1	2582	0
XM_020079477.1	serine/threonine-protein kinase Nek4-like	1649	0
XM_020079478.1	pyruvate dehyrogenase phosphatase catalytic subunit 1	1852	0
XM_020079479.1	pyruvate dehyrogenase phosphatase catalytic subunit 1	1905	0
XM_020079480.1	secretory carrier-associated membrane protein 4-like isoform X2	3707	1.22E-129
XM_020079481.1	mothers against decapentaplegic homolog 3	3250	0
XM_020079482.1	SKI family transcriptional corepressor 1 homolog-B-like isoform X1	3681	0
XM_020079483.1	SKI family transcriptional corepressor 1 homolog-B-like isoform X1	3654	0
XM_020079484.1	SKI family transcriptional corepressor 1 homolog-B-like isoform X1	3578	0
XM_020079485.1	SKI family transcriptional corepressor 1 homolog-B-like isoform X1	3717	0

XM_020079486.1	SKI family transcriptional corepressor 1 homolog-B-like isoform X1	3685	0
XM_020079487.1	SKI family transcriptional corepressor 1 homolog-B-like isoform X1	3678	0
XM_020079488.1	SKI family transcriptional corepressor 1 homolog-B-like isoform X1	3623	0
XM_020079489.1	PREDICTED: uncharacterized protein C15orf61 homolog	1082	3.02E-113
XM_020079490.1	PREDICTED: uncharacterized protein C15orf61 homolog	744	4.79E-115
XM_020079491.1	E3 ubiquitin/ISG15 ligase TRIM25-like isoform X1	2760	0
XM_020079492.1	E3 ubiquitin/ISG15 ligase TRIM25-like isoform X2	2754	0
XM_020079493.1	circadian-associated transcriptional repressor-like	2548	0
XM_020079494.1	leucine-rich repeat serine/threonine-protein kinase 2 isoform X1	8503	0
XM_020079495.1	leucine-rich repeat serine/threonine-protein kinase 2 isoform X2	8500	0
XM_020079496.1	myoblast determination protein 1 homolog	2507	0
XM_020079497.1	ferritin, heavy subunit-like isoform X1	1291	4.97E-123
XM_020079498.1	ferritin, heavy subunit-like isoform X2	1288	3.60E-122
XM_020079499.1	coiled-coil domain-containing protein 136-like isoform X1	4011	0
XM_020079500.1	coiled-coil domain-containing protein 136-like isoform X2	3898	0
XM_020079501.1	coiled-coil domain-containing protein 136-like isoform X1	3940	0
XM_020079502.1	BEN domain-containing protein 4	2024	0
XM_020079503.1	GRAM domain-containing protein 4-like	4981	0
XM_020079504.1	AP-3 complex subunit beta-2 isoform X3	6942	0
XM_020079505.1	AP-3 complex subunit beta-2 isoform X5	6930	0
XM_020079506.1	AP-3 complex subunit beta-2 isoform X3	6906	0
XM_020079507.1	AP-3 complex subunit beta-2 isoform X4	7030	0
XM_020079508.1	AP-3 complex subunit beta-2 isoform X5	6894	0
XM_020079509.1	protein tyrosine phosphatase domain-containing protein 1-like	2191	0
XM_020079510.1	outer dense fiber protein 3-like protein 2	906	0
XM_020079511.1	rab-like protein 2B	1754	4.93E-163
XM_020079512.1	transcriptional enhancer factor TEF-3 isoform X1	2656	0
XM_020079513.1	transcriptional enhancer factor TEF-3 isoform X2	1900	0
XM_020079514.1	transcriptional enhancer factor TEF-3 isoform X3	1839	0
XM_020079515.1	transcriptional enhancer factor TEF-3 isoform X4	1830	0
XM_020079516.1	PREDICTED: uncharacterized protein LOC109624664	1280	2.60E-165
XM_020079517.1	transcriptional enhancer factor TEF-3 isoform X3	1836	0
XM_020079518.1	G/T mismatch-specific thymine DNA glycosylase-like isoform X1	1883	0
XM_020079519.1	G/T mismatch-specific thymine DNA glycosylase-like isoform X2	1746	0
XM_020079520.1	G/T mismatch-specific thymine DNA glycosylase-like isoform X3	1862	0
XM_020079521.1	pseudopodium-enriched atypical kinase 1	8716	0
XM_020079522.1	cyclin-dependent kinase inhibitor 1B	2525	8.25E-113
XM_020079523.1	cyclin-dependent kinase inhibitor 1B	1197	2.24E-118
XM_020079524.1	dnaJ homolog subfamily A member 1-like	1764	0
XM_020079525.1	cingulin-like protein 1 isoform X1	3565	0
XM_020079526.1	cingulin-like protein 1 isoform X2	3538	0
XM_020079527.1	PREDICTED: aquaporin-9-like	1356	5.33E-124
XM_020079528.1	creatine kinase U-type, mitochondrial-like	2181	0
XM_020079529.1	guanine nucleotide exchange factor for Rab-3A-like isoform X1	2866	0
XM_020079530.1	guanine nucleotide exchange factor for Rab-3A-like isoform X2	2746	1.85E-163
XM_020079531.1	choline/ethanolamine kinase	2561	0
XM_020079532.1	potassium voltage-gated channel subfamily D member 2-like isoform X1	3584	0
XM_020079533.1	potassium voltage-gated channel subfamily D member 2-like isoform X1	3301	0
XM_020079534.1	potassium voltage-gated channel subfamily D member 2-like isoform X1	3687	0
XM_020079535.1	5-hydroxytryptamine receptor 1-like	2928	0
XM_020079536.1	adenosylhomocysteinase 2-like isoform X1	2123	0
XM_020079537.1	adenosylhomocysteinase 2-like isoform X2	1770	0
XM_020079538.1	adenosylhomocysteinase 2-like isoform X3	1902	0
XM_020079539.1	muscarinic acetylcholine receptor M2-like	1636	0
XM_020079540.1	tumor necrosis factor ligand superfamily member 13B-like isoform X1	1240	3.59E-165
XM_020079541.1	IQ and ubiquitin-like domain-containing protein	2365	0
XM_020079542.1	lathosterol oxidase-like	2123	2.77E-51

XM_020079543.1	lathosterol oxidase-like	1289	0
XM_020079544.1	membrane-associated guanylate kinase, WW and PDZ domain-containing protein 2 isoform X1	6074	0
XM_020079545.1	tumor necrosis factor ligand superfamily member 13B-like isoform X2	1228	3.59E-162
XM_020079546.1	membrane-associated guanylate kinase, WW and PDZ domain-containing protein 2 isoform X2	4618	0
XM_020079547.1	histidine ammonia-lyase	2784	0
XM_020079548.1	protein BANP	2605	0
XM_020079549.1	hyaluronan and proteoglycan link protein 3	1258	0
XM_020079550.1	hyaluronan and proteoglycan link protein 3	2728	0
XM_020079551.1	PREDICTED: calphotin-like	1741	1.31E-85
XM_020079552.1	otogelin-like protein isoform X1	8532	0
XM_020079553.1	otogelin-like protein isoform X2	8529	0
XM_020079554.1	otogelin-like protein isoform X3	8499	0
XM_020079555.1	transmembrane protein 53-A-like isoform X3	4276	0
XM_020079556.1	nocturnin-like isoform X1	2066	0
XM_020079557.1	interleukin-12 subunit beta isoform X1	1143	0
XM_020079558.1	transmembrane protein 53-A-like isoform X3	4154	0
XM_020079559.1	transmembrane protein 53-A-like isoform X3	4314	0
XM_020079560.1	transmembrane protein 53-A-like isoform X3	4331	0
XM_020079561.1	transmembrane protein 53-A-like isoform X3	4182	0
XM_020079562.1	protein ALP1-like isoform X1	1395	0
XM_020079564.1	transcription factor Adf-1-like	1044	0
XM_020079565.1	interferon-stimulated 20 kDa exonuclease-like 2	1759	0

XM_020079566.1	interleukin-12 subunit beta isoform X2	1046	0
XM_020079567.1	protein phosphatase 1H-like	4261	0
XM_020079568.1	cAMP-regulated phosphoprotein 19-like	1515	8.31E-74
XM_020079569.1	epidermal growth factor receptor kinase substrate 8-like protein 2 isoform X1	3017	0
XM_020079570.1	epidermal growth factor receptor kinase substrate 8-like protein 2 isoform X2	3015	0
XM_020079571.1	epidermal growth factor receptor kinase substrate 8-like protein 2 isoform X3	3086	0
XM_020079572.1	epidermal growth factor receptor kinase substrate 8-like protein 2 isoform X4	2940	0
XM_020079573.1	epidermal growth factor receptor kinase substrate 8-like protein 2 isoform X5	2837	0
XM_020079574.1	PREDICTED: snurportin-1	1876	0
XM_020079575.1	bile acid-CoA:amino acid N-acyltransferase-like	1723	0
XM_020079576.1	bile acid-CoA:amino acid N-acyltransferase-like	1644	0
XM_020079577.1	pyruvate kinase PKM-like isoform X1	2197	0
XM_020079578.1	PREDICTED: uncharacterized protein LOC109624703	2652	0
XM_020079579.1	60S acidic ribosomal protein P2-like	489	2.23E-34
XM_020079580.1	embryonic polyadenylate-binding protein 2 isoform X1	1362	1.07E-159
XM_020079581.1	embryonic polyadenylate-binding protein 2 isoform X2	1339	1.44E-156
XM_020079582.1	trafficking protein particle complex subunit 2-like protein	589	7.36E-102
XM_020079583.1	adhesion G-protein coupled receptor G5-like isoform X1	3940	0
XM_020079584.1	adhesion G-protein coupled receptor G5-like isoform X2	3937	0
XM_020079585.1	ladderlectin-like isoform X1	785	1.75E-99
XM_020079586.1	phosphatidate phosphatase LPIN1-like isoform X1	5747	0
XM_020079587.1	phosphatidate phosphatase LPIN1-like isoform X2	5744	0
XM_020079588.1	phosphatidate phosphatase LPIN1-like isoform X3	5654	0
XM_020079589.1	phosphatidate phosphatase LPIN1-like isoform X4	2824	0
XM_020079590.1	C3a anaphylatoxin chemotactic receptor-like	2453	0
XM_020079591.1	C3a anaphylatoxin chemotactic receptor-like	2660	0
XM_020079592.1	F-box only protein 22	3175	0
XM_020079593.1	bromodomain-containing protein 7	1969	0
XM_020079594.1	synaptosomal-associated protein 25-A-like	2087	4.80E-140
XM_020079595.1	DNA damage-induced apoptosis suppressor protein	3176	0
XM_020079596.1	acyl-CoA synthetase family member 3, mitochondrial	3420	0
XM_020079597.1	probable G-protein coupled receptor 101	2063	0
XM_020079598.1	AP-3 complex subunit sigma-2	1708	5.44E-137
XM_020079599.1	AP-3 complex subunit sigma-2	1419	2.14E-138
XM_020079600.1	ribonuclease P protein subunit p29	1350	4.26E-163
XM_020079601.1	vitamin K-dependent protein S	3032	0
XM_020079602.1	PREDICTED: fumarylacetoacetase	2342	0
XM_020079603.1	leucine-rich repeat-containing protein 49 isoform X1	3264	0
XM_020079604.1	leucine-rich repeat-containing protein 49 isoform X2	3258	0
XM_020079605.1	lactoylglutathione lyase-like	1348	2.57E-131
XM_020079606.1	glucose-6-phosphate isomerase	2065	0
XM_020079607.1	two pore calcium channel protein 2 isoform X1	4136	0
XM_020079608.1	two pore calcium channel protein 2 isoform X2	4041	0
XM_020079609.1	two pore calcium channel protein 2 isoform X3	3897	0
XM_020079610.1	hepatocyte nuclear factor 6 isoform X1	5810	0
XM_020079611.1	hepatocyte nuclear factor 6 isoform X2	5795	0
XM_020079612.1	aldehyde dehydrogenase family 1 member A3	2817	0
XM_020079613.1	pirin isoform X1	3135	0
XM_020079614.1	pirin isoform X2	3087	0
XM_020079615.1	WD repeat-containing protein 76	2598	0
XM_020079616.1	U2 small nuclear ribonucleoprotein A'	1116	0
XM_020079617.1	BTB/POZ domain-containing protein KCTD12-like	1734	0
XM_020079618.1	growth arrest-specific protein 1-like	2993	4.85E-161
XM_020079619.1	ADP-ribosylation factor-like protein 13A	1894	0
XM_020079620.1	7-dehydrocholesterol reductase	1520	0
XM_020079621.1	7-dehydrocholesterol reductase	1499	0
XM_020079622.1	ras-specific guanine nucleotide-releasing factor 1-like isoform X1	5645	0
XM_020079623.1	ras-specific guanine nucleotide-releasing factor 1-like isoform X1	5754	0
XM_020079624.1	ras-specific guanine nucleotide-releasing factor 1-like isoform X1	4099	0
XM_020079625.1	LDLR chaperone MESD	1012	7.76E-116
XM_020079626.1	signal peptidase complex catalytic subunit SEC11A isoform X1	1104	1.41E-117
XM_020079627.1	signal peptidase complex catalytic subunit SEC11A isoform X2	1050	1.18E-151
XM_020079628.1	pyridoxal phosphate phosphatase PHOSPHO2	1515	5.53E-171
XM_020079629.1	pyridoxal phosphate phosphatase PHOSPHO2	1545	7.89E-171
XM_020079630.1	ATP synthase F(0) complex subunit C3, mitochondrial-like	813	8.12E-78
XM_020079631.1	ATP synthase F(0) complex subunit C3, mitochondrial-like	818	2.51E-77
XM_020079632.1	ATP synthase F(0) complex subunit C3, mitochondrial-like	767	1.14E-77
XM_020079633.1	nocturnin-like isoform X1	1990	0
XM_020079634.1	cilia- and flagella-associated protein 100	1869	0
XM_020079635.1	ATP synthase F(0) complex subunit C3, mitochondrial-like	783	1.12E-77
XM_020079636.1	5-formyltetrahydrofolate cyclo-ligase	1432	3.35E-136
XM_020079637.1	cadherin-8-like isoform X1	4332	0
XM_020079638.1	cadherin-8-like isoform X2	4326	0
XM_020079639.1	selenoprotein S	631	2.35E-42
XM_020079640.1	NT-3 growth factor receptor-like isoform X1	6354	0
XM_020079641.1	NT-3 growth factor receptor-like isoform X2	6345	0
XM_020079642.1	NT-3 growth factor receptor-like isoform X3	6339	0
XM_020079643.1	NT-3 growth factor receptor-like isoform X4	6291	0
XM_020079644.1	NT-3 growth factor receptor-like isoform X5	6276	0

XM_020079645.1	NT-3 growth factor receptor-like isoform X6	6192	0
XM_020079646.1	PREDICTED: insulin-like	349	1.36E-79
XM_020079647.1	NT-3 growth factor receptor-like isoform X7	6047	0
XM_020079648.1	PREDICTED: tetraspanin-3-like, partial	1782	4.64E-160
XM_020079649.1	protein C19orf12 homolog	1410	7.92E-83
XM_020079650.1	SS18-like protein 2	594	1.91E-44
XM_020079651.1	serine/threonine/tyrosine-interacting protein isoform X1	1095	0
XM_020079652.1	serine/threonine/tyrosine-interacting protein isoform X2	1066	0
XM_020079653.1	serine/threonine/tyrosine-interacting protein isoform X3	1413	8.91E-180
XM_020079654.1	poly [ADP-ribose] polymerase 6 isoform X1	4263	0
XM_020079655.1	poly [ADP-ribose] polymerase 6 isoform X2	4260	0
XM_020079656.1	poly [ADP-ribose] polymerase 6 isoform X3	4188	0
XM_020079657.1	poly [ADP-ribose] polymerase 6 isoform X4	4185	0
XM_020079658.1	putative solute carrier family 22 member 31	3266	0
XM_020079659.1	NADH dehydrogenase [ubiquinone] iron-sulfur protein 8, mitochondrial-like isoform X1	1084	2.78E-173
XM_020079660.1	signal peptide peptidase-like 2A	1068	5.46E-122
XM_020079661.1	small EDRK-rich factor 2	711	2.19E-21
XM_020079662.1	39S ribosomal protein L16, mitochondrial	1407	1.04E-159
XM_020079663.1	zinc finger protein 536 isoform X1	7339	0
XM_020079664.1	zinc finger protein 536 isoform X1	7311	0
XM_020079665.1	zinc finger protein 536 isoform X1	7349	0
XM_020079666.1	transmembrane protein 178B-like	4755	2.20E-178
XM_020079667.1	NADH dehydrogenase [ubiquinone] iron-sulfur protein 8, mitochondrial-like isoform X1	1153	6.70E-173
XM_020079668.1	nucleoside diphosphate-linked moiety X motif 19	2837	0
XM_020079669.1	myocyte-specific enhancer factor 2A-like	2923	0
XM_020079670.1	teashirt homolog 3	5898	0
XM_020079671.1	proprotein convertase subtilisin/kexin type 6	3199	0
XM_020079672.1	protein Lines homolog 1 isoform X1	2984	0
XM_020079673.1	protein Lines homolog 1 isoform X2	2982	0
XM_020079674.1	Nance-Horan syndrome protein-like	5222	0
XM_020079675.1	NADH dehydrogenase [ubiquinone] iron-sulfur protein 8, mitochondrial-like isoform X1	991	2.01E-148
XM_020079676.1	angiotensin-converting enzyme 2 isoform X1	2194	0
XM_020079677.1	angiotensin-converting enzyme 2 isoform X1	2302	0
XM_020079678.1	angiotensin-converting enzyme 2 isoform X1	2363	0
XM_020079679.1	glypican-6 isoform X2	6555	0
XM_020079680.1	glutamine-dependent NAD(+) synthetase	2439	0
XM_020079681.1	centrosomal protein of 89 kDa isoform X1	4281	0
XM_020079682.1	centrosomal protein of 89 kDa isoform X2	3500	0
XM_020079683.1	centrosomal protein of 89 kDa isoform X3	2468	0
XM_020079684.1	iroquois-class homeodomain protein IRX-5 isoform X1	2144	0
XM_020079685.1	iroquois-class homeodomain protein IRX-5 isoform X2	2128	0
XM_020079686.1	arylsulfatase G	2188	0
XM_020079687.1	PREDICTED: uncharacterized protein LOC109624777	1135	0
XM_020079688.1	immunoglobulin superfamily DCC subclass member 4-like	6514	0
XM_020079689.1	transcription initiation factor TFIID subunit 4-like	5436	0
XM_020079690.1	cytochrome P450 27C1	2390	0
XM_020079691.1	endophilin-A3-like isoform X1	3299	0
XM_020079692.1	endophilin-A3-like isoform X2	3266	0
XM_020079693.1	endophilin-A3-like isoform X3	3233	0
XM_020079694.1	ceramide synthase 2-like	3089	0
XM_020079695.1	ceramide synthase 2-like	3150	0
XM_020079696.1	PREDICTED: uncharacterized protein LOC109624777	1093	0
XM_020079697.1	low-density lipoprotein receptor-related protein 3 isoform X1	3389	0
XM_020079698.1	low-density lipoprotein receptor-related protein 3 isoform X2	3352	0
XM_020079699.1	dipeptidase 1	2049	0
XM_020079700.1	sulfotransferase family cytosolic 2B member 1-like	1234	0
XM_020079701.1	collagen and calcium-binding EGF domain-containing protein 1-like isoform X1	3024	0
XM_020079702.1	collagen and calcium-binding EGF domain-containing protein 1-like isoform X2	3174	0
XM_020079703.1	collagen and calcium-binding EGF domain-containing protein 1-like isoform X2	2907	9.07E-170
XM_020079704.1	thrombospondin type-1 domain-containing protein 4 isoform X1	4683	0
XM_020079705.1	thrombospondin type-1 domain-containing protein 4 isoform X2	3479	0
XM_020079706.1	cyclin-dependent kinase-like 5 isoform X2	6635	0
XM_020079707.1	cyclin-dependent kinase-like 5 isoform X2	6533	0
XM_020079708.1	titin homolog	6204	0
XM_020079709.1	PREDICTED: uncharacterized protein LOC109624790	1075	0
XM_020079710.1	UPF0258 protein KIAA1024-like	5622	0
XM_020079711.1	retinoblastoma-like protein 2 isoform X1	3287	0
XM_020079712.1	retinoblastoma-like protein 2 isoform X2	3268	0
XM_020079713.1	ankyrin repeat and death domain-containing protein 1A	3532	0
XM_020079714.1	hepatoma-derived growth factor-related protein 3 isoform X1	4454	2.97E-100
XM_020079715.1	hepatoma-derived growth factor-related protein 3 isoform X2	4451	2.66E-100
XM_020079716.1	PREDICTED: semaphorin-7A-like	2794	0
XM_020079717.1	mannan-binding lectin serine protease 1	1097	0
XM_020079718.1	membrane-spanning 4-domains subfamily A member 4D-like	1449	5.58E-140
XM_020079719.1	PREDICTED: complexin-3	3121	1.99E-85
XM_020079720.1	E3 ubiquitin-protein ligase RNF182-like	807	0
XM_020079721.1	PREDICTED: secretogranin-3	1640	0
XM_020079722.1	histidine decarboxylase-like	2189	0
XM_020079723.1	phosphorylase b kinase regulatory subunit alpha, skeletal muscle isoform-like	5225	0

XM_020079724.1	signal peptide, CUB and EGF-like domain-containing protein 1 isoform X1	1369	0
XM_020079725.1	polycomb protein SCMH1-like	3638	0
XM_020079726.1	arylsulfatase I	1716	0
XM_020079727.1	coiled-coil domain-containing protein 81	2326	0
XM_020079728.1	interleukin-18 receptor 1-like	1666	0
XM_020079729.1	interleukin-18 receptor accessory protein-like	2289	0
XM_020079730.1	PREDICTED: fibronectin-like	9288	0
XM_020079731.1	integrin beta-6	2040	0
XM_020079732.1	secretory phospholipase A2 receptor	3201	0
XM_020079733.1	protocadherin-11 X-linked-like	3034	0
XM_020079734.1	protein diaphanous homolog 3	4212	0
XM_020079735.1	lysosome-associated membrane glycoprotein 1	2082	0
XM_020079736.1	transmembrane protein 255B	1062	0
XM_020079737.1	TNFAIP3-interacting protein 1 isoform X1	1367	0
XM_020079738.1	DNA repair protein complementing XP-G cells	3747	0
XM_020079739.1	centromere protein Q	1121	2.64E-160
XM_020079740.1	glypican-5-like isoform X2	2852	0
XM_020079741.1	LIM domain only protein 7-like	3786	0
XM_020079742.1	tRNA (cytosine(34)-C(5))-methyltransferase, mitochondrial-like	843	4.40E-172
XM_020079743.1	carbonic anhydrase 7	1894	4.23E-152
XM_020079744.1	protein CBFA2T3-like	6621	0
XM_020079745.1	dihydroorotate dehydrogenase (quinone), mitochondrial	2160	0
XM_020079746.1	anoctamin-5 isoform X2	4747	0
XM_020079747.1	immunoglobulin superfamily DCC subclass member 3	2303	0
XM_020079748.1	transmembrane emp24 domain-containing protein 11-like	744	1.53E-153
XM_020079749.1	PREDICTED: consortin	5522	0
XM_020079750.1	nuclear receptor-binding protein-like	1106	0
XM_020079751.1	spermatogenesis-associated protein 17	1066	0
XM_020079752.1	zinc finger and BTB domain-containing protein 20-like	2007	0
XM_020079753.1	PREDICTED: uncharacterized protein LOC109624836	1812	0
XM_020079754.1	B(0,+)-type amino acid transporter 1	1985	0
XM_020079755.1	epithelial splicing regulatory protein 1-like	3114	0
XM_020079756.1	FERM domain-containing protein 5	1100	1.89E-125
XM_020079757.1	transmembrane emp24 domain-containing protein 3-like	1456	3.74E-156
XM_020079758.1	fibroblast growth factor 4-like	573	8.30E-121
XM_020079759.1	transient receptor potential cation channel subfamily M member 7	6701	0
XM_020079760.1	guanine nucleotide-binding protein subunit beta-4	1569	0
XM_020079761.1	unconventional myosin-Va	5727	0
XM_020079762.1	protein unc-13 homolog C-like	9225	0
XM_020079763.1	Wilms tumor protein 1-interacting protein	4390	1.59E-146
XM_020079764.1	A-kinase anchor protein 13-like	3722	0
XM_020079765.1	tyrosyl-DNA phosphodiesterase 1	1928	0
XM_020079766.1	adhesion G-protein coupled receptor G1-like	1373	0
XM_020079767.1	phosphorylase b kinase regulatory subunit beta	5373	0
XM_020079768.1	docking protein 3	1588	0
XM_020079769.1	serine/threonine-protein kinase pim-2-like	1605	0
XM_020079770.1	putative gonadotropin-releasing hormone II receptor	1251	0
XM_020079771.1	PREDICTED: neural-cadherin-like	8013	0
XM_020079772.1	AP-2 complex subunit alpha-2	3862	0
XM_020079773.1	CD81 antigen-like	2529	6.28E-145
XM_020079774.1	PREDICTED: mucin-5AC-like	5769	0
XM_020079775.1	PREDICTED: mucin-5B-like	3780	0
XM_020079776.1	PREDICTED: uncharacterized protein LOC109624861	618	7.69E-127
XM_020079777.1	cell migration-inducing and hyaluronan-binding protein	7516	0
XM_020079778.1	E3 ubiquitin-protein ligase MARCH3-like	767	0
XM_020079779.1	F-BAR and double SH3 domains protein 1-like	2296	0
XM_020079780.1	gap junction delta-2 protein-like	1322	0
XM_020079781.1	pleckstrin homology domain-containing family F member 1-like	964	1.41E-178
XM_020079782.1	anoctamin-4 isoform X1	4964	0
XM_020079783.1	pleckstrin homology domain-containing family O member 2	3735	0
XM_020079784.1	PREDICTED: oral cancer-overexpressed protein 1	800	7.66E-92
XM_020079785.1	protein crumbs homolog 2-like	864	5.19E-162
XM_020079786.1	NEDD8-activating enzyme E1 regulatory subunit	2016	0
XM_020079787.1	ubiquitin-like protein 7	1444	0
XM_020079788.1	ubiquitin-like protein 7	1409	0
XM_020079789.1	neuronal acetylcholine receptor subunit alpha-5	2152	0
XM_020079790.1	C-X-C motif chemokine 10-like	605	2.98E-59
XM_020079791.1	26S proteasome non-ATPase regulatory subunit 14	1182	0
XM_020079792.1	insulin gene enhancer protein ISL-3 isoform X1	1822	0
XM_020079793.1	insulin gene enhancer protein isl-2a isoform X2	1810	0
XM_020079794.1	insulin gene enhancer protein ISL-3 isoform X3	1153	0
XM_020079795.1	insulin gene enhancer protein ISL-3 isoform X4	1661	0
XM_020079796.1	chymotrypsin B-like	1078	0
XM_020079797.1	39S ribosomal protein L46, mitochondrial isoform X1	1070	0
XM_020079798.1	39S ribosomal protein L46, mitochondrial isoform X2	1069	0
XM_020079799.1	leucine-rich repeat-containing protein 28	1653	0
XM_020079800.1	cytochrome c oxidase subunit 5A, mitochondrial-like	695	3.51E-102
XM_020079801.1	methionyl-tRNA formyltransferase, mitochondrial isoform X1	1487	0
XM_020079802.1	methionyl-tRNA formyltransferase, mitochondrial isoform X1	1260	0

XM_020079803.1	transcription factor PU.1	1616	0
XM_020079804.1	lysM and putative peptidoglycan-binding domain-containing protein 2	806	2.08E-133
XM_020079805.1	kinesin-like protein KIF26A	8978	0
XM_020079806.1	histone-lysine N-methyltransferase SMYD3-like	800	1.43E-139
XM_020079807.1	histone-lysine N-methyltransferase SMYD3-like	845	7.94E-162
XM_020079808.1	transmembrane channel-like protein 3	3749	0
XM_020079809.1	POU domain, class 4, transcription factor 1	2468	0
XM_020079810.1	MAM domain-containing glycosylphosphatidylinositol anchor protein 2	4971	0
XM_020079811.1	synaptic vesicle glycoprotein 2B-like	2173	0
XM_020079812.1	28S ribosomal protein S11, mitochondrial	774	3.57E-145
XM_020079813.1	PREDICTED: synaptotagmin-5-like	3044	0
XM_020079814.1	PREDICTED: synaptotagmin-5-like	2365	0
XM_020079815.1	RWD domain-containing protein 3 isoform X1	1080	1.66E-177
XM_020079816.1	RWD domain-containing protein 3 isoform X2	1027	1.95E-176
XM_020079817.1	RWD domain-containing protein 3 isoform X3	1075	1.33E-137
XM_020079818.1	PREDICTED: interaptin-like	1032	0
XM_020079819.1	RWD domain-containing protein 3 isoform X4	1022	3.38E-136
XM_020079820.1	estrogen-related receptor gamma-like isoform X1	1588	0
XM_020079821.1	estrogen-related receptor gamma-like isoform X2	1425	0
XM_020079822.1	protein-lysine methyltransferase METTL21E-like isoform X1	1161	0
XM_020079823.1	protein-lysine methyltransferase METTL21E-like isoform X2	1158	0
XM_020079824.1	XK-related protein 5-like	3781	0
XM_020079825.1	PREDICTED: uncharacterized protein LOC109624901	2380	0
XM_020079826.1	serine/threonine-protein phosphatase with EF-hands 1-like	2517	0
XM_020079827.1	galanin receptor type 1-like	1182	0
XM_020079828.1	brain-derived neurotrophic factor isoform X2	1483	1.36E-167
XM_020079829.1	brain-derived neurotrophic factor isoform X2	1784	7.40E-164
XM_020079830.1	brain-derived neurotrophic factor isoform X2	1570	6.45E-165
XM_020079831.1	homeodomain-interacting protein kinase 1-like	1473	0
XM_020079832.1	brain-derived neurotrophic factor isoform X2	1559	5.60E-165
XM_020079833.1	brain-derived neurotrophic factor isoform X2	1496	5.77E-165
XM_020079834.1	brain-derived neurotrophic factor isoform X2	1485	2.31E-165
XM_020079835.1	brain-derived neurotrophic factor isoform X2	1418	1.50E-166
XM_020079836.1	protein ADP-ribosylarginine hydrolase-like protein 1	2560	0
XM_020079837.1	high-affinity choline transporter 1-like	2405	0
XM_020079838.1	high-affinity choline transporter 1-like	2405	0
XM_020079839.1	potassium/sodium hyperpolarization-activated cyclic nucleotide-gated channel 1-like	4346	0
XM_020079840.1	rhodopsin kinase-like	1692	0
XM_020079841.1	GTP-binding protein RAD	1033	0
XM_020079842.1	GTP-binding protein RAD	1614	0
XM_020079843.1	probable G-protein coupled receptor 151	1460	0
XM_020079844.1	UDP-glucuronosyltransferase 2C1-like isoform X3	1524	0
XM_020079845.1	growth arrest-specific protein 2	1737	0
XM_020079846.1	fibroblast growth factor 3	1822	6.37E-137
XM_020079847.1	proline-serine-threonine phosphatase-interacting protein 1-like	1467	0
XM_020079848.1	proline-serine-threonine phosphatase-interacting protein 1-like	1461	0
XM_020079849.1	transmembrane protein 266-like	3208	0
XM_020079850.1	melatonin receptor type 1A-like	590	1.12E-77
XM_020079851.1	carbohydrate sulfotransferase 8	3106	0
XM_020079852.1	nucleotide-binding oligomerization domain 1 protein	3183	0
XM_020079853.1	docking protein 4	1269	1.04E-149
XM_020079854.1	cadherin-4-like isoform X3	2957	0
XM_020079855.1	anoctamin-9-like isoform X1	1060	0
XM_020079856.1	anoctamin-9-like isoform X1	939	0
XM_020079857.1	anoctamin-9-like isoform X1	969	0
XM_020079858.1	copper transport protein ATOX1	1284	1.69E-40
XM_020079859.1	anoctamin-9-like isoform X1	979	0
XM_020079860.1	anoctamin-9-like isoform X1	834	0
XM_020079861.1	anoctamin-9-like isoform X1	740	1.02E-178
XM_020079862.1	anoctamin-9-like isoform X1	738	9.98E-172
XM_020079863.1	vascular endothelial growth factor D	1778	0
XM_020079864.1	PREDICTED: ephrin-B2a-like	2183	0
XM_020079865.1	transmembrane 6 superfamily member 1	1337	0
XM_020079866.1	telomere repeats-binding bouquet formation protein 2 isoform X1	1025	1.82E-137
XM_020079867.1	telomere repeats-binding bouquet formation protein 2 isoform X2	1003	4.93E-133
XM_020079868.1	pancreatic secretory granule membrane major glycoprotein GP2-like	1477	1.79E-105
XM_020079869.1	secretory carrier-associated membrane protein 4-like isoform X2	1104	4.46E-146
XM_020079870.1	fibroblast growth factor 4	620	3.61E-128
XM_020079871.1	coagulation factor V-like	1896	0

XM_020079872.1	mesoderm posterior protein 2-like	944	1.85E-162
XM_020079873.1	vascular endothelial growth factor receptor kdr-like isoform X1	6814	0
XM_020079874.1	trans-1,2-dihydrobenzene-1,2-diol dehydrogenase-like	1213	0
XM_020079875.1	interferon-induced protein 44-like	926	0
XM_020079876.1	chemokine-like factor	857	1.47E-68
XM_020079877.1	ubiquitin carboxyl-terminal hydrolase 8	1613	0
XM_020079878.1	vascular endothelial growth factor receptor kdr-like isoform X2	4151	0
XM_020079879.1	gastrin-releasing peptide receptor-like	1056	0
XM_020079880.1	interferon regulatory factor 1-like	792	2.17E-164
XM_020079881.1	interleukin 1 beta like 2	1213	0

XM_020079882.1	potassium-transporting ATPase subunit beta	876	0
XM_020079883.1	disks large homolog 3 isoform X1	4647	0
XM_020079884.1	beta-2 adrenergic receptor-like	1369	0
XM_020079885.1	gastrin-releasing peptide	635	1.40E-96
XM_020079886.1	regulator of G-protein signaling 9-binding protein	866	7.86E-159
XM_020079887.1	adhesion G-protein coupled receptor G2-like	641	1.91E-107
XM_020079888.1	protein eva-1 homolog A-like	585	1.98E-75
XM_020079889.1	ankyrin repeat domain-containing protein 11 isoform X1	10198	0
XM_020079890.1	ankyrin repeat domain-containing protein 11 isoform X2	10195	0
XM_020079891.1	disks large homolog 3 isoform X2	4643	0
XM_020079892.1	cadherin-4-like isoform X3	3285	0
XM_020079893.1	AFG3-like protein 2	3496	0
XM_020079894.1	golgin subfamily A member 4-like	603	2.84E-99
XM_020079895.1	cytoplasmic dynein 1 light intermediate chain 2 isoform X1	4177	0
XM_020079896.1	cytoplasmic dynein 1 light intermediate chain 2 isoform X2	4172	0
XM_020079897.1	disks large homolog 3 isoform X3	4629	0
XM_020079898.1	CKLF-like MARVEL transmembrane domain-containing protein 4	4476	4.30E-121
XM_020079899.1	CKLF-like MARVEL transmembrane domain-containing protein 3	2652	2.82E-83
XM_020079900.1	dmX-like protein 2 isoform X1	11348	0
XM_020079901.1	dmX-like protein 2 isoform X2	10945	0
XM_020079902.1	dmX-like protein 2 isoform X3	11282	0
XM_020079903.1	dmX-like protein 2 isoform X4	8835	0
XM_020079904.1	noelin-like isoform X2	3353	0
XM_020079905.1	P450 aromatase	2046	0
XM_020079906.1	disks large homolog 3 isoform X4	4589	0
XM_020079907.1	tumor necrosis factor alpha-induced protein 8-like protein 3	1591	1.30E-125
XM_020079908.1	protein FAM189A1 isoform X1	4298	0
XM_020079909.1	protein FAM189A1 isoform X2	4264	0
XM_020079910.1	protein CASC4 isoform X1	3182	0
XM_020079911.1	protein CASC4 isoform X2	3189	0
XM_020079912.1	protein CASC4 isoform X3	3013	0
XM_020079913.1	protein CASC4 isoform X4	3008	0
XM_020079914.1	CTD small phosphatase-like protein 2	4496	0
XM_020079915.1	disks large homolog 3 isoform X5	4736	0
XM_020079916.1	CTD small phosphatase-like protein 2	4374	0
XM_020079917.1	kinesin-like protein KIF28P	3475	0
XM_020079918.1	PREDICTED: frizzled-3-like	2925	0
XM_020079919.1	PREDICTED: frizzled-3-like	5174	0
XM_020079920.1	keratinocyte-associated protein 3 isoform X1	1296	1.05E-158
XM_020079921.1	keratinocyte-associated protein 3 isoform X1	3201	1.18E-146
XM_020079922.1	mitogen-activated protein kinase 6	4385	0
XM_020079923.1	disks large homolog 3 isoform X6	4520	0
XM_020079924.1	mitogen-activated protein kinase 6	4415	0
XM_020079925.1	RNA polymerase-associated protein LEO1	2216	0
XM_020079926.1	protein ELYS isoform X1	9131	0
XM_020079927.1	protein ELYS isoform X1	9305	0
XM_020079928.1	voltage-gated hydrogen channel 1	1620	6.02E-123
XM_020079929.1	protein ELYS isoform X1	9101	0
XM_020079930.1	PWWP domain-containing protein MUM1L1-like isoform X1	2141	0
XM_020079931.1	PWWP domain-containing protein MUM1L1-like isoform X1	2136	0
XM_020079932.1	PWWP domain-containing protein MUM1L1-like isoform X1	2099	0
XM_020079933.1	PWWP domain-containing protein MUM1L1-like isoform X1	2094	0
XM_020079934.1	disks large homolog 3 isoform X7	4515	0
XM_020079935.1	PWWP domain-containing protein MUM1L1-like isoform X1	2123	0
XM_020079936.1	PWWP domain-containing protein MUM1L1-like isoform X1	2102	0
XM_020079937.1	tight junction protein ZO-1-like isoform X1	7566	0
XM_020079938.1	tight junction protein ZO-1-like isoform X2	7562	0
XM_020079939.1	tight junction protein ZO-1-like isoform X3	7538	0
XM_020079940.1	tight junction protein ZO-1-like isoform X4	7534	0
XM_020079941.1	tight junction protein ZO-1-like isoform X5	7525	0
XM_020079942.1	tight junction protein ZO-1-like isoform X6	7505	0
XM_020079943.1	tight junction protein ZO-1-like isoform X5	7312	0
XM_020079944.1	disks large homolog 3 isoform X8	4502	0
XM_020079945.1	tight junction protein ZO-1-like isoform X8	7474	0
XM_020079946.1	gamma-taxilin-like	2986	0
XM_020079947.1	gamma-taxilin-like	2882	0
XM_020079948.1	gamma-taxilin-like	2876	0
XM_020079949.1	gamma-taxilin-like	3136	0
XM_020079950.1	gamma-taxilin-like	2886	0
XM_020079951.1	gamma-taxilin-like	3100	0
XM_020079952.1	gamma-taxilin-like	2933	0
XM_020079953.1	gamma-taxilin-like	3115	0
XM_020079954.1	disks large homolog 3 isoform X9	4488	0
XM_020079955.1	histone-binding protein RBBP7 isoform X1	2464	0
XM_020079956.1	histone-binding protein RBBP7 isoform X2	2461	0
XM_020079957.1	solute carrier family 12 member 4	3285	0
XM_020079958.1	CUGBP Elav-like family member 3-B	1485	0
XM_020079959.1	dipeptidase 2-like	2792	0
XM_020079960.1	ribonuclease P protein subunit p25-like protein	2120	0

XM_020079961.1	disks large homolog 3 isoform X10	4534	0
XM_020079962.1	chromodomain-helicase-DNA-binding protein 9 isoform X1	10070	0
XM_020079963.1	chromodomain-helicase-DNA-binding protein 9 isoform X1	10297	0
XM_020079964.1	chromodomain-helicase-DNA-binding protein 9 isoform X1	10048	0
XM_020079965.1	chromodomain-helicase-DNA-binding protein 9 isoform X3	10036	0
XM_020079966.1	chromodomain-helicase-DNA-binding protein 9 isoform X1	10018	0
XM_020079967.1	chromodomain-helicase-DNA-binding protein 9 isoform X1	10016	0
XM_020079968.1	chromodomain-helicase-DNA-binding protein 9 isoform X1	8910	0
XM_020079969.1	chromodomain-helicase-DNA-binding protein 9 isoform X1	7963	0
XM_020079970.1	chromodomain-helicase-DNA-binding protein 9 isoform X1	7956	0
XM_020079971.1	disks large homolog 3 isoform X11	4708	0
XM_020079972.1	intersectin-2-like isoform X2	7952	0
XM_020079973.1	GC-rich sequence DNA-binding factor 2-like	2828	0
XM_020079974.1	kinesin-like protein KIF13B isoform X1	11188	0
XM_020079975.1	kinesin-like protein KIF13B isoform X2	11464	0
XM_020079976.1	monocarboxylate transporter 8-like	5813	0
XM_020079977.1	kinesin-like protein KIF13B isoform X3	11434	0
XM_020079978.1	kinesin-like protein KIF13B isoform X4	11431	0
XM_020079979.1	proenkephalin-A-like isoform X1	1804	2.81E-134
XM_020079980.1	proenkephalin-A-like isoform X1	1783	6.39E-134
XM_020079981.1	disks large homolog 3 isoform X12	4458	0
XM_020079982.1	mitochondrial ribonuclease P protein 1	1968	0
XM_020079983.1	PREDICTED: golgin-45	2353	0
XM_020079984.1	thioredoxin-like protein 4B	1268	7.15E-104
XM_020079985.1	programmed cell death protein 7 isoform X1	2624	0
XM_020079986.1	programmed cell death protein 7 isoform X2	2595	0
XM_020079987.1	cytochrome b-c1 complex subunit Rieske, mitochondrial-like	1703	0
XM_020079988.1	E3 ubiquitin-protein ligase MARCH7 isoform X1	3201	0
XM_020079989.1	E3 ubiquitin-protein ligase MARCH7 isoform X2	3152	0
XM_020079990.1	disks large homolog 3 isoform X13	4624	0
XM_020079991.1	lymphocyte antigen 75-like	7769	0
XM_020079992.1	junction-mediating and -regulatory protein-like	3045	0
XM_020079993.1	fibronectin type III and SPRY domain-containing protein 2	2147	0
XM_020079994.1	fibronectin type III and SPRY domain-containing protein 2	2031	0
XM_020079995.1	fibronectin type III and SPRY domain-containing protein 2	2083	0
XM_020079996.1	fibronectin type III and SPRY domain-containing protein 2	1989	0
XM_020079997.1	fibronectin type III and SPRY domain-containing protein 2	2639	0
XM_020079998.1	UPF0469 protein KIAA0907-like	2771	0
XM_020079999.1	ubiquitin carboxyl-terminal hydrolase 8	5015	0
XM_020080000.1	ubiquitin carboxyl-terminal hydrolase 8	4792	0
XM_020080001.1	very long-chain acyl-CoA synthetase-like	2112	0
XM_020080002.1	testis-expressed sequence 11 protein	3016	0
XM_020080003.1	serine/threonine-protein kinase 16 isoform X1	1214	0
XM_020080004.1	serine/threonine-protein kinase 16 isoform X1	1105	0
XM_020080005.1	PEST proteolytic signal-containing nuclear protein isoform X1	1845	1.34E-90
XM_020080006.1	PEST proteolytic signal-containing nuclear protein isoform X1	927	1.99E-78
XM_020080007.1	PREDICTED: plexin-B3-like	6709	0
XM_020080008.1	SRSF protein kinase 3	3443	0
XM_020080009.1	pre-rRNA-processing protein TSR2 homolog	848	8.04E-100
XM_020080010.1	pyruvate kinase PKM-like isoform X1	3015	0
XM_020080011.1	pyruvate kinase PKM-like isoform X1	3016	0
XM_020080012.1	pyruvate kinase PKM-like isoform X1	2884	0
XM_020080013.1	trans-1,2-dihydrobenzene-1,2-diol dehydrogenase-like	1354	0
XM_020080014.1	bifunctional glutamate/proline--tRNA ligase isoform X1	6112	0
XM_020080015.1	bifunctional glutamate/proline--tRNA ligase isoform X2	6070	0
XM_020080016.1	bifunctional glutamate/proline--tRNA ligase isoform X3	6049	0
XM_020080017.1	bifunctional glutamate/proline--tRNA ligase isoform X4	5869	0
XM_020080018.1	bifunctional glutamate/proline--tRNA ligase isoform X5	5826	0
XM_020080019.1	bifunctional glutamate/proline--tRNA ligase isoform X6	5805	0
XM_020080020.1	nuclear factor 7, ovary-like	3352	0
XM_020080021.1	adhesion G protein-coupled receptor G3-like	1706	0
XM_020080022.1	Y+L amino acid transporter 2-like	3177	0
XM_020080023.1	probable RNA polymerase II nuclear localization protein SLC7A6OS	1856	0
XM_020080024.1	tripeptidyl-peptidase 2 isoform X1	7997	0
XM_020080025.1	tripeptidyl-peptidase 2 isoform X2	7957	0
XM_020080026.1	protein-lysine methyltransferase METTL21C-like	1905	0
XM_020080027.1	ADP-dependent glucokinase-like	3921	0
XM_020080028.1	cell growth regulator with EF hand domain protein 1	1762	5.75E-140
XM_020080029.1	EF-hand calcium-binding domain-containing protein 2 isoform X1	2100	4.80E-138
XM_020080030.1	rho guanine nucleotide exchange factor 6 isoform X1	5257	0
XM_020080031.1	EF-hand calcium-binding domain-containing protein 2 isoform X2	2227	1.01E-135
XM_020080032.1	E3 ubiquitin-protein ligase AMFR	3949	0
XM_020080033.1	leukotriene B4 receptor 1-like	1197	0
XM_020080034.1	glucosamine 6-phosphate N-acetyltransferase	1222	4.00E-108
XM_020080035.1	glucosamine 6-phosphate N-acetyltransferase	1095	2.11E-108
XM_020080036.1	leukotriene B4 receptor 1-like	1104	0
XM_020080037.1	ADP-ribosylation factor-like protein 6	2469	3.42E-129
XM_020080038.1	rho guanine nucleotide exchange factor 6 isoform X2	5231	0
XM_020080039.1	ephrin type-A receptor 6-like	3594	0

XM_020080040.1	isocitrate dehydrogenase [NADP], mitochondrial-like	1695	0
XM_020080041.1	retinol dehydrogenase 13-like	1915	0
XM_020080042.1	telomeric repeat-binding factor 2-like isoform X1	2148	0
XM_020080043.1	telomeric repeat-binding factor 2-like isoform X1	2052	0
XM_020080044.1	telomeric repeat-binding factor 2-like isoform X3	2028	0
XM_020080045.1	60S ribosome subunit biogenesis protein NIP7 homolog	922	2.01E-130
XM_020080046.1	CDC42 small effector protein 2	2654	3.66E-46
XM_020080047.1	rho guanine nucleotide exchange factor 6 isoform X1	4712	0
XM_020080048.1	cartilage intermediate layer protein 1	3912	0
XM_020080049.1	stereocilin-like isoform X1	2123	0
XM_020080050.1	stereocilin-like isoform X2	1499	0
XM_020080051.1	eukaryotic translation initiation factor 3 subunit J	2061	8.36E-81
XM_020080052.1	talin-2 isoform X1	10576	0
XM_020080053.1	talin-2 isoform X1	10611	0
XM_020080054.1	talin-2 isoform X1	10570	0
XM_020080055.1	bromodomain adjacent to zinc finger domain protein 2B-like isoform X1	8927	0
XM_020080056.1	bromodomain adjacent to zinc finger domain protein 2B-like isoform X2	8920	0
XM_020080057.1	bromodomain adjacent to zinc finger domain protein 2B-like isoform X3	8914	0
XM_020080058.1	bromodomain adjacent to zinc finger domain protein 2B-like isoform X4	8908	0
XM_020080059.1	bromodomain adjacent to zinc finger domain protein 2B-like isoform X5	8893	0
XM_020080060.1	transmembrane 9 superfamily member 2-like isoform X1	2806	0
XM_020080061.1	bromodomain adjacent to zinc finger domain protein 2B-like isoform X6	8881	0
XM_020080062.1	bromodomain adjacent to zinc finger domain protein 2B-like isoform X7	8861	0
XM_020080063.1	bromodomain adjacent to zinc finger domain protein 2B-like isoform X8	8848	0
XM_020080064.1	bromodomain adjacent to zinc finger domain protein 2B-like isoform X9	8842	0
XM_020080065.1	bromodomain adjacent to zinc finger domain protein 2B-like isoform X10	8824	0
XM_020080066.1	bromodomain adjacent to zinc finger domain protein 2B-like isoform X11	8822	0
XM_020080067.1	bromodomain adjacent to zinc finger domain protein 2B-like isoform X12	8811	0
XM_020080068.1	bromodomain adjacent to zinc finger domain protein 2B-like isoform X13	8805	0
XM_020080069.1	bromodomain adjacent to zinc finger domain protein 2B-like isoform X14	8778	0
XM_020080070.1	bromodomain adjacent to zinc finger domain protein 2B-like isoform X15	8776	0
XM_020080071.1	transmembrane 9 superfamily member 2-like isoform X2	2754	0
XM_020080072.1	bromodomain adjacent to zinc finger domain protein 2B-like isoform X16	8701	0
XM_020080073.1	bromodomain adjacent to zinc finger domain protein 2B-like isoform X17	8674	0
XM_020080074.1	bromodomain adjacent to zinc finger domain protein 2B-like isoform X18	8805	0
XM_020080075.1	G1/S-specific cyclin-E2-like	1963	0
XM_020080076.1	G1/S-specific cyclin-E2-like	2149	0
XM_020080077.1	PREDICTED: uncharacterized protein F13E9.13, mitochondrial-like	1338	0
XM_020080078.1	tumor protein p53-inducible nuclear protein 2-like	1538	1.51E-82
XM_020080079.1	aryl hydrocarbon receptor nuclear translocator 2 isoform X5	6057	0
XM_020080080.1	aryl hydrocarbon receptor nuclear translocator 2 isoform X5	5804	0
XM_020080081.1	aryl hydrocarbon receptor nuclear translocator 2 isoform X3	6008	0
XM_020080082.1	aryl hydrocarbon receptor nuclear translocator 2 isoform X4	5997	0
XM_020080083.1	aryl hydrocarbon receptor nuclear translocator 2 isoform X5	6410	0
XM_020080084.1	aryl hydrocarbon receptor nuclear translocator 2 isoform X5	6381	0
XM_020080085.1	transformer-2 protein homolog beta isoform X4	1815	2.06E-108
XM_020080086.1	aryl hydrocarbon receptor nuclear translocator 2 isoform X6	5755	0
XM_020080087.1	protein LSM14 homolog A isoform X1	4079	0
XM_020080088.1	protein LSM14 homolog A isoform X1	4076	0
XM_020080089.1	ubiquitin-conjugating enzyme E2 Q2-like isoform X1	4529	0
XM_020080090.1	type 1 insulin-like growth factor receptor	9913	0
XM_020080091.1	homer protein homolog 2 isoform X1	3121	0
XM_020080092.1	homer protein homolog 2 isoform X2	2816	0
XM_020080093.1	homer protein homolog 2 isoform X3	1479	0
XM_020080094.1	RNMT-activating mini protein	1105	4.22E-31
XM_020080095.1	RNMT-activating mini protein	1167	2.88E-30
XM_020080096.1	tropomyosin alpha-1 chain isoform X1	1204	2.16E-165
XM_020080097.1	tropomyosin alpha-1 chain isoform X2	976	9.03E-167
XM_020080098.1	tropomyosin alpha-1 chain isoform X3	1591	1.39E-163
XM_020080099.1	tropomyosin alpha-1 chain isoform X4	2324	1.08E-157
XM_020080100.1	tropomyosin alpha-1 chain isoform X5	1252	2.87E-156
XM_020080101.1	tropomyosin alpha-1 chain isoform X6	1121	1.61E-142
XM_020080102.1	tropomyosin alpha-1 chain isoform X7	893	4.93E-144
XM_020080103.1	tropomyosin alpha-4 chain isoform X8	1508	3.62E-140
XM_020080104.1	tropomyosin alpha-4 chain isoform X9	1059	4.50E-140
XM_020080105.1	AT-rich interactive domain-containing protein 3B isoform X1	6160	0
XM_020080106.1	AT-rich interactive domain-containing protein 3B isoform X1	1925	0
XM_020080107.1	sulfate transporter	4365	0
XM_020080108.1	A-kinase anchor protein 13 isoform X1	6178	0
XM_020080109.1	A-kinase anchor protein 13 isoform X2	6175	0
XM_020080110.1	dimethyladenosine transferase 1, mitochondrial	2398	0
XM_020080111.1	dimethyladenosine transferase 1, mitochondrial	2370	0
XM_020080112.1	saccharopine dehydrogenase-like oxidoreductase	2476	0
XM_020080113.1	transmembrane and coiled-coil domain-containing protein 3	4025	0
XM_020080114.1	transmembrane and coiled-coil domain-containing protein 3	3927	0
XM_020080115.1	transmembrane and coiled-coil domain-containing protein 3	3946	0
XM_020080116.1	neuropeptide Y receptor type 2-like	1222	0
XM_020080117.1	DCN1-like protein 2 isoform X3	3278	0
XM_020080118.1	DCN1-like protein 2 isoform X3	3269	0

XM_020080119.1	DCN1-like protein 2 isoform X3	3270	0
XM_020080120.1	DCN1-like protein 2 isoform X3	3460	0
XM_020080121.1	U2 small nuclear ribonucleoprotein auxiliary factor 35 kDa subunit-related protein 2	2829	0
XM_020080122.1	AP-1 complex subunit sigma-2-like	2094	7.86E-94
XM_020080123.1	AP-1 complex subunit sigma-2-like	1671	2.09E-95
XM_020080124.1	threonine--tRNA ligase, cytoplasmic-like isoform X1	2694	0
XM_020080125.1	threonine--tRNA ligase, cytoplasmic-like isoform X2	2699	0
XM_020080126.1	serine/threonine-protein kinase ULK3 isoform X1	2967	0
XM_020080127.1	serine/threonine-protein kinase ULK3 isoform X1	2929	0
XM_020080128.1	serine/threonine-protein kinase ULK3 isoform X1	2898	0
XM_020080129.1	serine/threonine-protein kinase ULK3 isoform X1	2902	0
XM_020080130.1	PRELI domain-containing protein 1, mitochondrial	1637	7.63E-136
XM_020080131.1	serine/threonine-protein kinase ULK3 isoform X1	2941	0
XM_020080132.1	serine/threonine-protein kinase ULK3 isoform X1	2812	0
XM_020080133.1	serine/threonine-protein kinase ULK3 isoform X1	2872	0
XM_020080134.1	serine/threonine-protein kinase ULK3 isoform X1	1780	0
XM_020080135.1	kinesin-like protein KIF18A isoform X1	3597	0
XM_020080136.1	kinesin-like protein KIF18A isoform X2	3420	0
XM_020080137.1	probable methyltransferase-like protein 15 isoform X1	2359	0
XM_020080138.1	probable methyltransferase-like protein 15 isoform X1	1423	0
XM_020080139.1	sal-like protein 1 isoform X2	5957	0
XM_020080140.1	PRELI domain-containing protein 1, mitochondrial	1752	2.68E-135
XM_020080141.1	sal-like protein 1 isoform X2	5322	0
XM_020080142.1	sal-like protein 1 isoform X2	5167	0
XM_020080143.1	protein FAM214B	4696	0
XM_020080144.1	TFIIH basal transcription factor complex helicase XPB subunit	2917	0
XM_020080145.1	GPALPP motifs-containing protein 1	1586	5.23E-180
XM_020080146.1	intestinal mucin-like protein	1375	0
XM_020080147.1	toll-interacting protein	2751	2.07E-163
XM_020080148.1	protein lin-7 homolog A isoform X1	4944	2.56E-119
XM_020080149.1	vacuolar protein sorting-associated protein 35	3904	0
XM_020080150.1	leucine-rich repeat-containing G-protein coupled receptor 4 isoform X1	5956	0
XM_020080151.1	leucine-rich repeat-containing G-protein coupled receptor 4 isoform X2	5953	0
XM_020080152.1	max dimerization protein 3-like	1662	3.46E-96
XM_020080153.1	PDZ domain-containing protein 2-like	4700	0
XM_020080154.1	stAR-related lipid transfer protein 5	3139	1.52E-146
XM_020080155.1	zinc finger protein 507 isoform X1	4858	0
XM_020080156.1	zinc finger protein 507 isoform X1	4947	0
XM_020080157.1	protein phosphatase 1G isoform X1	3735	0
XM_020080158.1	protein phosphatase 1G isoform X2	3732	0
XM_020080159.1	elongation factor-like GTPase 1	5267	0
XM_020080160.1	maspardin isoform X1	1308	0
XM_020080161.1	maspardin isoform X2	1227	0
XM_020080162.1	maspardin isoform X3	1331	0
XM_020080163.1	protein FAM193B	3435	0
XM_020080164.1	CUGBP Elav-like family member 1 isoform X1	3721	0
XM_020080165.1	CUGBP Elav-like family member 1 isoform X1	3718	0
XM_020080166.1	CUGBP Elav-like family member 1 isoform X2	3649	0
XM_020080167.1	CUGBP Elav-like family member 1 isoform X2	3646	0
XM_020080168.1	CUGBP Elav-like family member 1 isoform X2	3526	7.28E-156
XM_020080169.1	CUGBP Elav-like family member 1 isoform X2	3725	2.48E-173
XM_020080170.1	ubiquitin carboxyl-terminal hydrolase CYLD isoform X1	5391	0
XM_020080171.1	ubiquitin carboxyl-terminal hydrolase CYLD isoform X1	5042	0
XM_020080172.1	ubiquitin carboxyl-terminal hydrolase CYLD isoform X1	5274	0
XM_020080173.1	tropomodulin-1 isoform X2	1645	0
XM_020080174.1	semaphorin-4A-like isoform X1	4242	0
XM_020080175.1	protein ABHD17C	3657	0
XM_020080176.1	DEAD box protein 41	2287	0
XM_020080177.1	non-canonical poly(A) RNA polymerase PAPD5	4694	0
XM_020080178.1	PREDICTED: uncharacterized protein KIAA0355 homolog	4875	0
XM_020080179.1	PREDICTED: uncharacterized protein KIAA0355 homolog	5131	0
XM_020080180.1	PREDICTED: uncharacterized protein KIAA0355 homolog	5059	0
XM_020080181.1	IST1 homolog isoform X1	2873	0
XM_020080182.1	IST1 homolog isoform X1	1423	0
XM_020080183.1	IST1 homolog isoform X1	2843	0
XM_020080184.1	immunoglobulin-like domain-containing receptor 2 isoform X1	4283	0
XM_020080185.1	immunoglobulin-like domain-containing receptor 2 isoform X2	4226	0
XM_020080186.1	immunoglobulin-like domain-containing receptor 2 isoform X3	4154	0
XM_020080187.1	cell surface A33 antigen	1612	0
XM_020080188.1	protocadherin-17 isoform X1	8260	0
XM_020080189.1	protocadherin-17 isoform X2	8257	0
XM_020080190.1	ankyrin repeat and KH domain-containing protein 1-like isoform X1	8859	0
XM_020080191.1	protocadherin-17 isoform X3	5340	0
XM_020080192.1	protocadherin-17 isoform X4	4907	0
XM_020080193.1	protocadherin-17 isoform X5	5245	0
XM_020080194.1	pyruvate dehydrogenase phosphatase regulatory subunit, mitochondrial	5489	0
XM_020080195.1	pyruvate dehydrogenase phosphatase regulatory subunit, mitochondrial	3151	0
XM_020080196.1	amyloid beta A4 precursor protein-binding family A member 2-like isoform X1	5224	0
XM_020080197.1	amyloid beta A4 precursor protein-binding family A member 2-like isoform X1	5188	0
XM_020080198.1	sorbitol dehydrogenase-like	931	2.25E-106

XM_020080199.1	ankyrin repeat and KH domain-containing protein 1-like isoform X2	8464	0

XM_020080200.1	sorbitol dehydrogenase-like	1126	1.87E-70
XM_020080201.1	growth arrest-specific protein 6	4402	0
XM_020080202.1	leucine-rich repeat serine/threonine-protein kinase 1	8595	0
XM_020080203.1	transforming growth factor-beta receptor-associated protein 1	5733	0
XM_020080204.1	transforming growth factor-beta receptor-associated protein 1	5728	0
XM_020080205.1	transforming growth factor-beta receptor-associated protein 1	5737	0
XM_020080206.1	G1/S-specific cyclin-D1-like isoform X1	3874	3.79E-180
XM_020080207.1	ankyrin repeat and KH domain-containing protein 1-like isoform X3	8455	0
XM_020080208.1	high choriolytic enzyme 1-like	1249	0
XM_020080209.1	high choriolytic enzyme 1-like	968	0
XM_020080210.1	FAS-associated death domain protein	804	2.20E-136
XM_020080211.1	PREDICTED: plakophilin-3-like	4860	0
XM_020080212.1	NTPase KAP family P-loop domain-containing protein 1 isoform X1	4757	0
XM_020080213.1	NTPase KAP family P-loop domain-containing protein 1 isoform X1	4816	0
XM_020080214.1	NTPase KAP family P-loop domain-containing protein 1 isoform X1	4808	0
XM_020080215.1	NTPase KAP family P-loop domain-containing protein 1 isoform X1	4642	0
XM_020080216.1	ankyrin repeat and KH domain-containing protein 1-like isoform X4	8449	0
XM_020080217.1	NTPase KAP family P-loop domain-containing protein 1 isoform X1	4426	0
XM_020080218.1	LIM and senescent cell antigen-like-containing domain protein 1	2549	0
XM_020080219.1	ferritin, heavy subunit	1142	2.15E-130
XM_020080220.1	RNA-binding protein 26 isoform X1	4079	0
XM_020080221.1	RNA-binding protein 26 isoform X2	4077	0
XM_020080222.1	RNA-binding protein 26 isoform X3	3978	0
XM_020080223.1	zinc finger protein 710-like	4020	0
XM_020080224.1	neuronal acetylcholine receptor subunit alpha-3-like	3225	0
XM_020080225.1	neuronal acetylcholine receptor subunit beta-2-like isoform X2	1822	0
XM_020080226.1	PREDICTED: uncharacterized protein LOC109625123	1060	2.42E-155
XM_020080227.1	cullin-3 isoform X2	4128	0
XM_020080228.1	adenylate cyclase type 7 isoform X1	5030	0
XM_020080229.1	adenylate cyclase type 7 isoform X2	5091	0
XM_020080230.1	ras GTPase-activating protein 3	5160	0
XM_020080231.1	protein GREB1	6114	0
XM_020080232.1	cytochrome P450 1A1	2472	0
XM_020080233.1	AN1-type zinc finger protein 3	2992	4.64E-128
XM_020080234.1	dnaJ homolog subfamily A member 2	2263	0
XM_020080235.1	PHD finger protein 6 isoform X1	2119	0
XM_020080236.1	mesoderm development candidate 1	3324	0
XM_020080237.1	protein naked cuticle homolog 1	5314	0
XM_020080238.1	RNA-binding motif, single-stranded-interacting protein 1-like	3698	0
XM_020080239.1	matrix metalloproteinase-14-like	8840	0
XM_020080240.1	lysosome-associated membrane glycoprotein 2 isoform X1	2730	0
XM_020080241.1	SH2 domain-containing adapter protein F-like isoform X1	5031	0
XM_020080242.1	SH2 domain-containing adapter protein F-like isoform X2	4983	0
XM_020080243.1	SH2 domain-containing adapter protein F-like isoform X3	4884	0
XM_020080244.1	protocadherin-11 X-linked-like	4930	0
XM_020080245.1	apoptosis regulator BAX-like	3288	3.60E-126
XM_020080246.1	growth hormone-regulated TBC protein 1 isoform X1	2247	0
XM_020080247.1	growth hormone-regulated TBC protein 1 isoform X2	1674	0
XM_020080248.1	lysosome-associated membrane glycoprotein 2 isoform X2	2037	0
XM_020080249.1	cullin-3 isoform X2	3566	0
XM_020080250.1	chondroitin sulfate synthase 1	4391	0
XM_020080251.1	palmitoyltransferase ZDHHC7-like	4695	0
XM_020080252.1	palmitoyltransferase ZDHHC7-like	1459	0
XM_020080253.1	homeobox-containing protein 1-like isoform X1	7663	0
XM_020080254.1	homeobox-containing protein 1-like isoform X2	7660	0
XM_020080255.1	homeobox-containing protein 1-like isoform X3	7657	0
XM_020080256.1	U3 small nucleolar ribonucleoprotein protein MPP10	2245	0
XM_020080257.1	lysosome-associated membrane glycoprotein 2 isoform X3	4187	0
XM_020080258.1	methylmalonyl-CoA epimerase, mitochondrial	814	4.26E-125
XM_020080259.1	HEAT repeat-containing protein 3	3434	0
XM_020080260.1	cAMP-regulated phosphoprotein 19-A-like	2084	3.16E-74
XM_020080261.1	MTSS1-like protein isoform X1	11120	0
XM_020080262.1	MTSS1-like protein isoform X1	11111	0
XM_020080263.1	MTSS1-like protein isoform X3	10555	0
XM_020080264.1	lysophosphatidylcholine acyltransferase 1-like	2972	0
XM_020080265.1	high affinity cAMP-specific and IBMX-insensitive 3',5'-cyclic phosphodiesterase 8A-like	4708	0
XM_020080266.1	high affinity cAMP-specific and IBMX-insensitive 3',5'-cyclic phosphodiesterase 8A-like	4597	0
XM_020080267.1	RING finger protein 219	2631	0
XM_020080268.1	72 kDa type IV collagenase	2454	0
XM_020080269.1	V-type proton ATPase 116 kDa subunit a-like	3079	0
XM_020080270.1	RNA-binding protein MEX3B	3352	0
XM_020080271.1	neuropilin and tolloid-like protein 2	3823	0
XM_020080272.1	T-cell immunomodulatory protein	2862	0
XM_020080273.1	sodium-dependent noradrenaline transporter	3301	0
XM_020080274.1	sodium-dependent noradrenaline transporter	3314	0
XM_020080275.1	TOX high mobility group box family member 3	4324	0
XM_020080276.1	tyrosine-protein kinase CSK-like isoform X1	2686	0
XM_020080277.1	nuclear factor of activated T-cells, cytoplasmic 3 isoform X1	6515	0

XM_020080278.1	nuclear factor of activated T-cells, cytoplasmic 3 isoform X2	6125	0
XM_020080279.1	nuclear factor of activated T-cells, cytoplasmic 3 isoform X3	6106	0
XM_020080280.1	zinc finger and BTB domain-containing protein 5-like	2498	0
XM_020080281.1	neuroplastin-like isoform X1	2081	0
XM_020080282.1	neuroplastin-like isoform X2	1601	0
XM_020080283.1	neuroplastin-like isoform X3	1729	0
XM_020080284.1	AKT-interacting protein isoform X1	1911	0
XM_020080285.1	AKT-interacting protein isoform X1	1896	0
XM_020080286.1	AKT-interacting protein isoform X2	1908	0
XM_020080287.1	AKT-interacting protein isoform X2	1895	0
XM_020080288.1	CD276 antigen isoform X1	2153	3.98E-158
XM_020080289.1	CD276 antigen isoform X2	1323	0
XM_020080290.1	CD276 antigen isoform X3	1365	2.66E-173
XM_020080291.1	zinc finger protein basonuclin-1 isoform X1	4153	0
XM_020080292.1	zinc finger protein basonuclin-1 isoform X1	4299	0
XM_020080293.1	la-related protein 6	3141	0
XM_020080294.1	protein phosphatase Slingshot homolog 3-like	3435	0
XM_020080295.1	tudor domain-containing protein 3	2762	0
XM_020080296.1	protein TANC1-like	2307	0
XM_020080297.1	WD repeat, SAM and U-box domain-containing protein 1-like	3506	0
XM_020080298.1	PREDICTED: rhophilin-2	3969	0
XM_020080299.1	ankyrin repeat and SOCS box protein 7 isoform X1	3302	0
XM_020080300.1	ankyrin repeat and SOCS box protein 7 isoform X1	3434	0
XM_020080301.1	ankyrin repeat and SOCS box protein 7 isoform X1	3478	0
XM_020080302.1	protein phosphatase Slingshot homolog 3-like	3406	0
XM_020080303.1	BTB/POZ domain-containing protein KCTD3	7621	0
XM_020080304.1	probable Xaa-Pro aminopeptidase 3	1608	0
XM_020080305.1	PREDICTED: cryptochrome-1-like	2456	0
XM_020080306.1	serine protease 23-like	3989	0
XM_020080307.1	F-box/LRR-repeat protein 3-like	2099	0
XM_020080308.1	RNA polymerase II elongation factor ELL2-like	4307	0
XM_020080309.1	BTB/POZ domain-containing protein KCTD15 isoform X1	4189	0
XM_020080310.1	BTB/POZ domain-containing protein KCTD15 isoform X2	4481	0
XM_020080311.1	BTB/POZ domain-containing protein KCTD15 isoform X3	5060	0
XM_020080312.1	large neutral amino acids transporter small subunit 2-like isoform X1	2014	0
XM_020080313.1	glyoxylate reductase/hydroxypyruvate reductase-like isoform X1	1565	0
XM_020080314.1	cathepsin B-like	1822	0
XM_020080315.1	cathepsin B-like	1725	0
XM_020080316.1	protein-lysine 6-oxidase-like	5190	0
XM_020080317.1	UPF0583 protein C15orf59 homolog	2472	1.48E-162
XM_020080318.1	PHD finger protein 6 isoform X1	2125	0
XM_020080319.1	vesicular glutamate transporter 2.1	4474	0
XM_020080320.1	coiled-coil domain-containing protein 102A	4823	0
XM_020080321.1	glyoxylate reductase/hydroxypyruvate reductase-like isoform X1	1183	0
XM_020080322.1	glycine amidinotransferase, mitochondrial	2391	0
XM_020080323.1	multiple C2 and transmembrane domain-containing protein 2-like isoform X1	3777	0
XM_020080324.1	multiple C2 and transmembrane domain-containing protein 2-like isoform X1	3772	0
XM_020080325.1	multiple C2 and transmembrane domain-containing protein 2-like isoform X1	3773	0
XM_020080326.1	multiple C2 and transmembrane domain-containing protein 2-like isoform X1	3724	0
XM_020080327.1	multiple C2 and transmembrane domain-containing protein 2-like isoform X4	3721	0
XM_020080328.1	enhancer of mRNA-decapping protein 3-like	2660	0
XM_020080329.1	laminin subunit alpha-3-like	18586	0
XM_020080330.1	glyoxylate reductase/hydroxypyruvate reductase-like isoform X1	1505	0
XM_020080331.1	lamin-A-like isoform X1	2455	0
XM_020080332.1	protein MIS12 homolog	2518	5.69E-142
XM_020080333.1	transmembrane protein 39B	2646	0
XM_020080334.1	myosin-binding protein C, cardiac-type isoform X1	4257	0
XM_020080335.1	myosin-binding protein C, cardiac-type isoform X2	4254	0
XM_020080336.1	myosin-binding protein C, cardiac-type isoform X3	4233	0
XM_020080337.1	myosin-binding protein C, cardiac-type isoform X4	4230	0
XM_020080338.1	bifunctional purine biosynthesis protein PURH	2146	0
XM_020080339.1	glyoxylate reductase/hydroxypyruvate reductase-like isoform X3	1564	0
XM_020080340.1	iroquois-class homeodomain protein IRX-3	1759	0
XM_020080341.1	anti-apoptotic protein NR13-like	1529	2.72E-118
XM_020080342.1	very long-chain acyl-CoA synthetase-like	2928	0
XM_020080343.1	probable C-mannosyltransferase DPY19L3 isoform X1	6048	0
XM_020080344.1	probable C-mannosyltransferase DPY19L3 isoform X2	6044	0
XM_020080345.1	UPF0547 protein C16orf87 homolog	2239	5.19E-79
XM_020080346.1	delta-like protein 1	4548	0
XM_020080347.1	TM2 domain-containing protein 3	1902	8.47E-170
XM_020080348.1	probable ATP-dependent RNA helicase DDX28	2294	0
XM_020080349.1	alpha-ketoglutarate-dependent dioxygenase FTO	3631	0
XM_020080350.1	39S ribosomal protein L18, mitochondrial	905	1.37E-177
XM_020080351.1	synapse-associated protein 1	1861	0
XM_020080352.1	synapse-associated protein 1	1839	0
XM_020080353.1	putative short-chain dehydrogenase/reductase family 42E member 2	1785	0
XM_020080354.1	nuclear envelope phosphatase-regulatory subunit 1	777	1.33E-89
XM_020080355.1	general transcription factor IIF subunit 2 isoform X1	1475	0
XM_020080356.1	general transcription factor IIF subunit 2 isoform X2	1414	0

XM_020080357.1	protein FAM219B-like	2807	2.51E-109
XM_020080358.1	A disintegrin and metalloproteinase with thrombospondin motifs 18 isoform X1	4844	0
XM_020080359.1	A disintegrin and metalloproteinase with thrombospondin motifs 18 isoform X2	4071	0
XM_020080360.1	aminopeptidase Ey-like isoform X1	4064	0
XM_020080361.1	aminopeptidase Ey-like isoform X2	2966	0
XM_020080362.1	macrophage erythroblast attacher isoform X1	2988	0
XM_020080363.1	mesoderm posterior protein 2-like	909	7.09E-168

XM_020080364.1	ADAMTS-like protein 3 isoform X1	7547	0
XM_020080365.1	ADAMTS-like protein 3 isoform X2	7572	0
XM_020080366.1	ADAMTS-like protein 3 isoform X1	1950	0
XM_020080367.1	A disintegrin and metalloproteinase with thrombospondin motifs 17 isoform X1	4011	0
XM_020080368.1	A disintegrin and metalloproteinase with thrombospondin motifs 17 isoform X2	4008	0
XM_020080369.1	A disintegrin and metalloproteinase with thrombospondin motifs 17 isoform X3	3936	0
XM_020080370.1	aminopeptidase N-like isoform X1	3751	0
XM_020080371.1	macrophage erythroblast attacher isoform X1	3122	0
XM_020080372.1	aminopeptidase N-like isoform X1	2655	0
XM_020080373.1	BTB/POZ domain-containing protein 1 isoform X1	1883	0
XM_020080374.1	BTB/POZ domain-containing protein 1 isoform X2	1819	0
XM_020080375.1	immunoglobulin-like domain-containing receptor 1	2598	0
XM_020080376.1	immunoglobulin-like domain-containing receptor 1	2544	0
XM_020080377.1	zinc transporter ZIP13	2400	0
XM_020080378.1	vesicle-associated membrane protein 4 isoform X1	2184	4.20E-59
XM_020080379.1	vesicle-associated membrane protein 4 isoform X1	2045	2.51E-51
XM_020080380.1	heterogeneous nuclear ribonucleoprotein A0-like	1729	2.26E-128
XM_020080381.1	E3 ubiquitin-protein ligase LNX isoform X1	3470	0
XM_020080382.1	E3 ubiquitin-protein ligase LNX isoform X2	3464	0
XM_020080383.1	E3 ubiquitin-protein ligase LNX isoform X3	3454	0
XM_020080384.1	E3 ubiquitin-protein ligase LNX isoform X4	3447	0
XM_020080385.1	non-specific lipid-transfer protein-like	1140	4.22E-97
XM_020080386.1	leucine-rich repeat and immunoglobulin-like domain-containing nogo receptor-interacting protein 3	4838	0
XM_020080387.1	alcohol dehydrogenase [NADP(+)]	1333	0
XM_020080388.1	alcohol dehydrogenase [NADP(+)]	1387	0
XM_020080389.1	dimethylaniline monooxygenase [N-oxide-forming] 5-like isoform X1	3102	0
XM_020080390.1	dimethylaniline monooxygenase [N-oxide-forming] 5-like isoform X1	2751	0
XM_020080391.1	dimethylaniline monooxygenase [N-oxide-forming] 5-like isoform X1	3052	0
XM_020080392.1	phosphatidylinositol 3,4,5-trisphosphate 5-phosphatase 2B-like isoform X1	6118	0
XM_020080393.1	centrosomal protein of 63 kDa	2611	0
XM_020080394.1	transmembrane protein 205-like	2386	3.64E-106
XM_020080395.1	UDP-N-acetylglucosamine/UDP-glucose/GDP-mannose transporter-like	2318	0
XM_020080396.1	PHD finger protein 6 isoform X1	2115	0
XM_020080397.1	LIM domain transcription factor LMO4 isoform X1	1538	4.32E-117
XM_020080398.1	LIM domain transcription factor LMO4 isoform X2	950	1.89E-119
XM_020080399.1	SH2 domain-containing adapter protein D-like isoform X1	2430	0
XM_020080400.1	SH2 domain-containing adapter protein D-like isoform X2	2352	0
XM_020080401.1	phosphatidylinositol 3,4,5-trisphosphate 5-phosphatase 2B-like isoform X2	6115	0
XM_020080402.1	pre-B-cell leukemia transcription factor 1-like isoform X1	4577	0
XM_020080403.1	pre-B-cell leukemia transcription factor 1-like isoform X2	4464	0
XM_020080404.1	kelch-like protein 29	1964	0
XM_020080405.1	U6 snRNA-associated Sm-like protein LSm7 isoform X1	644	2.82E-71
XM_020080406.1	U6 snRNA-associated Sm-like protein LSm7 isoform X2	690	1.62E-69
XM_020080407.1	serine/threonine-protein kinase Kist	5079	0
XM_020080408.1	retinal rod rhodopsin-sensitive cGMP 3',5'-cyclic phosphodiesterase subunit delta	2323	6.19E-102
XM_020080409.1	choline transporter-like protein 5-A isoform X1	3682	0
XM_020080410.1	choline transporter-like protein 5-B isoform X2	4030	0
XM_020080411.1	CUGBP Elav-like family member 5	4880	0
XM_020080412.1	P2Y purinoceptor 2	2578	0
XM_020080413.1	coatomer subunit epsilon	1227	0
XM_020080414.1	neuroligin-4, X-linked	3587	0
XM_020080415.1	PREDICTED: uncharacterized protein C1orf21 homolog	2104	1.02E-70
XM_020080416.1	chromodomain-helicase-DNA-binding protein 1-like	3354	0
XM_020080417.1	PREDICTED: uncharacterized protein C2orf72 homolog isoform X1	2187	0
XM_020080418.1	PREDICTED: uncharacterized protein C2orf72 homolog isoform X2	2083	0
XM_020080419.1	vesicle transport protein USE1 isoform X1	2018	0
XM_020080420.1	vesicle transport protein USE1 isoform X2	1966	0
XM_020080421.1	vesicle transport protein USE1 isoform X1	2044	1.10E-160
XM_020080422.1	PREDICTED: uricase-like	1124	0
XM_020080423.1	beta-arrestin-2 isoform X2	1800	0
XM_020080424.1	sterile alpha motif domain-containing protein 13	1384	4.82E-74
XM_020080425.1	sterile alpha motif domain-containing protein 13	1407	5.99E-74
XM_020080426.1	sterile alpha motif domain-containing protein 13	1239	1.24E-74
XM_020080427.1	sterile alpha motif domain-containing protein 13	1220	1.04E-74
XM_020080428.1	oxysterol-binding protein-related protein 1-like isoform X1	3866	0
XM_020080429.1	oxysterol-binding protein-related protein 1-like isoform X2	3845	0
XM_020080430.1	oxysterol-binding protein-related protein 1-like isoform X3	3803	0
XM_020080431.1	delta(24)-sterol reductase	2486	0
XM_020080432.1	delta(24)-sterol reductase	2487	0
XM_020080433.1	sex comb on midleg-like protein 2 isoform X1	3973	0
XM_020080434.1	sex comb on midleg-like protein 2 isoform X2	3969	0
XM_020080435.1	coagulation factor V-like	768	8.65E-160

XM_020080436.1	AP-1 complex subunit sigma-2 isoform X1	2188	6.23E-116
XM_020080437.1	ras-related protein Rab-41 isoform X1	2686	6.99E-143
XM_020080438.1	AP-1 complex subunit sigma-2-like	2375	2.20E-106
XM_020080439.1	AP-1 complex subunit sigma-2-like	2366	3.24E-104
XM_020080440.1	AP-1 complex subunit sigma-2 isoform X4	606	9.57E-113
XM_020080441.1	AP-1 complex subunit sigma-2 isoform X5	2094	6.53E-104
XM_020080442.1	torsin-1A-like isoform X2	2435	0
XM_020080443.1	unconventional myosin-VIIa-like	7189	0
XM_020080444.1	cyclin-dependent kinase-like 5 isoform X1	3626	0
XM_020080445.1	cyclin-dependent kinase-like 5 isoform X2	3623	0

XM_020080446.1	ras-related protein Rab-41 isoform X2	2687	1.01E-142
XM_020080447.1	cyclin-dependent kinase-like 5 isoform X3	3521	0
XM_020080448.1	cyclin-dependent kinase-like 5 isoform X4	3503	0
XM_020080449.1	cyclin-dependent kinase-like 5 isoform X1	3339	0
XM_020080450.1	cyclin-dependent kinase-like 5 isoform X6	3131	0
XM_020080451.1	prothymosin alpha-A-like isoform X1	1382	1.21E-08
XM_020080452.1	prothymosin alpha-A-like isoform X2	1376	8.74E-09
XM_020080453.1	ubiquitin carboxyl-terminal hydrolase 46	2829	0
XM_020080454.1	solute carrier organic anion transporter family member 2A1	2542	0
XM_020080455.1	ras-related protein Rab-41 isoform X3	2689	1.85E-142
XM_020080456.1	high mobility group protein 20A isoform X1	1676	0
XM_020080457.1	high mobility group protein 20A isoform X1	1659	0
XM_020080458.1	SWI/SNF-related matrix-associated actin-dependent regulator of chromatin subfamily E member 1-related isoform X1	1673	3.83E-180
XM_020080459.1	PREDICTED: barttin	670	2.31E-78
XM_020080460.1	transcription factor HES-1	1355	2.59E-180
XM_020080461.1	fizzy-related protein homolog isoform X1	1974	0
XM_020080462.1	fizzy-related protein homolog isoform X2	1971	0
XM_020080463.1	ras-related protein Rab-41 isoform X4	2690	3.37E-142
XM_020080464.1	fizzy-related protein homolog isoform X3	1860	0
XM_020080465.1	xenotropic and polytropic retrovirus receptor 1 homolog	5862	0
XM_020080466.1	UV excision repair protein RAD23 homolog A-like	2218	0
XM_020080467.1	FYN-binding protein-like	1813	2.12E-166
XM_020080468.1	neurocan core protein	5641	0
XM_020080469.1	acyl-CoA synthetase family member 4	3546	0
XM_020080470.1	homeobox protein ESX1-like isoform X1	868	4.94E-93
XM_020080471.1	homeobox protein ESX1-like isoform X1	846	3.70E-93
XM_020080472.1	homeobox protein ESX1-like isoform X1	897	3.28E-93
XM_020080473.1	PREDICTED: beta-sarcoglycan	2364	0
XM_020080474.1	phosphatidylinositol 3,4,5-trisphosphate 5-phosphatase 1	4648	0
XM_020080475.1	E3 ubiquitin-protein ligase RNF126	1910	0
XM_020080476.1	mitochondrial import inner membrane translocase subunit TIM44	1984	0
XM_020080477.1	fatty-acid amide hydrolase 1 isoform X1	2204	0
XM_020080478.1	fatty-acid amide hydrolase 1 isoform X2	1881	0
XM_020080479.1	replication factor C subunit 4	1607	0
XM_020080480.1	DNA repair and recombination protein RAD54-like isoform X1	2549	0
XM_020080481.1	DNA repair and recombination protein RAD54-like isoform X1	2550	0
XM_020080482.1	protocadherin-11 X-linked-like	3229	0
XM_020080483.1	retinal homeobox protein Rx1-like	3651	0
XM_020080484.1	adenine DNA glycosylase isoform X1	2203	0
XM_020080485.1	adenine DNA glycosylase isoform X1	2053	0
XM_020080486.1	sterol O-acyltransferase 1	3408	0
XM_020080487.1	rho GTPase-activating protein 39-like isoform X1	5710	0
XM_020080488.1	ribonuclease P protein subunit p21-like	1672	7.10E-81
XM_020080489.1	PREDICTED: aquaporin-12-like	906	6.92E-178
XM_020080490.1	PREDICTED: cystinosin	1580	0
XM_020080491.1	PREDICTED: cystinosin	1571	0
XM_020080492.1	histone H2A deubiquitinase MYSM1	2792	0
XM_020080493.1	39S ribosomal protein L55, mitochondrial	1068	1.47E-57
XM_020080494.1	myelin proteolipid protein-like isoform X2	2120	0
XM_020080495.1	tubulin polyglutamylase TTLL7	3419	0
XM_020080496.1	PREDICTED: uncharacterized protein KIAA1211 homolog isoform X2	4891	0
XM_020080497.1	PREDICTED: uncharacterized protein KIAA1211 homolog isoform X1	4888	0
XM_020080498.1	PREDICTED: uncharacterized protein KIAA1211 homolog isoform X1	4876	0
XM_020080499.1	PREDICTED: uncharacterized protein KIAA1211 homolog isoform X1	4873	0
XM_020080500.1	PREDICTED: uncharacterized protein KIAA1211 homolog isoform X1	4881	0
XM_020080501.1	leucine-rich repeat-containing protein 40	2019	0
XM_020080502.1	myelin proteolipid protein-like isoform X2	3584	6.38E-142
XM_020080503.1	Krueppel-like factor 2	2617	0
XM_020080504.1	P protein	5501	0
XM_020080505.1	spindle assembly abnormal protein 6 homolog	2438	0
XM_020080506.1	type I iodothyronine deiodinase	1638	0
XM_020080507.1	nicotinamide riboside kinase 2-like isoform X1	984	2.56E-133
XM_020080508.1	nicotinamide riboside kinase 2-like isoform X1	770	1.22E-106
XM_020080509.1	PREDICTED: uncharacterized protein C19orf44 homolog	2681	0
XM_020080510.1	myelin proteolipid protein-like isoform X2	2159	4.44E-147
XM_020080511.1	serine protease I-1	1571	5.25E-178
XM_020080512.1	fibroblast growth factor 10-like	2915	1.07E-127
XM_020080513.1	forkhead box protein D2-like	2227	8.98E-171
XM_020080514.1	discoidin domain-containing receptor 2 isoform X1	4634	0

XM_020080515.1	discoidin domain-containing receptor 2 isoform X2	4583	0
XM_020080516.1	diencephalon/mesencephalon homeobox protein 1-B-like	2418	0
XM_020080517.1	caytaxin-like isoform X1	3940	0
XM_020080518.1	caytaxin-like isoform X1	3507	0
XM_020080519.1	polyribonucleotide 5'-hydroxyl-kinase Clp1	1636	0
XM_020080520.1	transmembrane protein 45A	2438	0
XM_020080521.1	transmembrane protein 45A	2435	0
XM_020080522.1	inactive N-acetylated-alpha-linked acidic dipeptidase-like protein 2	2795	0
XM_020080523.1	ras-related protein Rab-9A-like	4731	7.47E-138
XM_020080524.1	phosphatidylinositol N-acetylglucosaminyltransferase subunit C	1533	2.19E-178
XM_020080525.1	metalloprotease TIKI1	2730	0
XM_020080526.1	biogenesis of lysosome-related organelles complex 1 subunit 2	1400	6.04E-90
XM_020080527.1	ELAV-like protein 4 isoform X1	2344	0

XM_020080528.1	ELAV-like protein 4 isoform X2	2335	0
XM_020080529.1	ELAV-like protein 4 isoform X3	2305	0
XM_020080530.1	regulator of cell cycle RGCC-like isoform X1	1046	7.70E-62
XM_020080531.1	ELAV-like protein 4 isoform X4	2296	0
XM_020080532.1	ELAV-like protein 4 isoform X5	2275	0
XM_020080533.1	ELAV-like protein 4 isoform X6	2266	0
XM_020080534.1	ELAV-like protein 4 isoform X7	2725	0
XM_020080535.1	ELAV-like protein 4 isoform X8	2446	0
XM_020080536.1	ELAV-like protein 4 isoform X9	2227	0
XM_020080537.1	ELAV-like protein 4 isoform X10	2473	0
XM_020080538.1	ATP synthase subunit delta, mitochondrial	874	6.48E-111
XM_020080539.1	ras-like protein family member 11B	1499	5.73E-174
XM_020080540.1	regulator of cell cycle RGCC-like isoform X2	1012	1.00E-69
XM_020080541.1	60S ribosomal protein L36	462	2.74E-64
XM_020080542.1	methylmalonic aciduria and homocystinuria type C protein	2339	0
XM_020080543.1	C2 calcium-dependent domain-containing protein 4C-like	1168	0
XM_020080544.1	PREDICTED: uncharacterized protein LOC109625332	812	3.73E-117
XM_020080545.1	C2 calcium-dependent domain-containing protein 4C-like	6301	0
XM_020080546.1	PEX5-related protein	5512	0
XM_020080547.1	myocilin-like isoform X1	2144	0
XM_020080548.1	ceramide synthase 2-like	3000	0
XM_020080549.1	doublesex- and mab-3-related transcription factor A2	2590	0
XM_020080550.1	clathrin interactor 1	4755	0
XM_020080551.1	protein FAM43A	1719	0
XM_020080552.1	antihemorrhagic factor cHLP-B-like	1399	0
XM_020080553.1	dimethylaniline monooxygenase [N-oxide-forming] 5-like	2572	0
XM_020080554.1	protein IMPACT	1365	0
XM_020080555.1	hepatocyte nuclear factor 6-like isoform X1	3827	0
XM_020080556.1	one cut domain family member 2-like isoform X2	1693	0
XM_020080557.1	zinc finger protein 644-like	3973	0
XM_020080558.1	zinc finger protein 644-like	4020	0
XM_020080559.1	zinc finger FYVE domain-containing protein 9-like	3477	0
XM_020080560.1	U7 snRNA-associated Sm-like protein LSm11	1860	0
XM_020080561.1	prostaglandin E2 receptor EP1 subtype-like	1101	0
XM_020080562.1	sphingosine 1-phosphate receptor 3-like	3577	0
XM_020080563.1	capping protein, Arp2/3 and myosin-I linker protein 3-like	5323	0
XM_020080564.1	uracil nucleotide/cysteinyl leukotriene receptor	4231	0
XM_020080565.1	leucine-rich repeat-containing protein 24-like	5335	0
XM_020080566.1	PREDICTED: uncharacterized protein LOC109625354	3240	0
XM_020080567.1	beta-1,4-galactosyltransferase 2 isoform X1	3229	0
XM_020080568.1	protein SZT2	4150	0
XM_020080569.1	protein SZT2-like	5187	0
XM_020080570.1	CMP-N-acetylneuraminate-beta-1,4-galactoside alpha-2,3-sialyltransferase-like	2617	1.92E-178
XM_020080571.1	probable tRNA(His) guanylyltransferase	1854	0
XM_020080572.1	PREDICTED: artemin-like	795	4.45E-179
XM_020080573.1	lon protease homolog, mitochondrial	2930	0
XM_020080574.1	PREDICTED: uncharacterized protein LOC109625363	3533	0
XM_020080575.1	meprin A subunit alpha-like	2217	0
XM_020080576.1	carnitine O-palmitoyltransferase 2, mitochondrial-like	2443	0
XM_020080577.1	calcium load-activated calcium channel	947	3.49E-90
XM_020080578.1	protein wntless homolog	1605	0
XM_020080579.1	bcl-2-like protein 1 isoform X1	1288	2.84E-142
XM_020080580.1	tyrosine-protein kinase RYK	3387	0
XM_020080581.1	phospholipase D3-like isoform X1	4360	0
XM_020080582.1	zinc finger and BTB domain-containing protein 11	971	0
XM_020080583.1	probable E3 ubiquitin-protein ligase HERC1	12363	0
XM_020080584.1	coiled-coil and C2 domain-containing protein 2A	5260	0
XM_020080585.1	ralBP1-associated Eps domain-containing protein 2	1875	0
XM_020080586.1	leukemia NUP98 fusion partner 1	627	1.45E-147
XM_020080587.1	phospholipase D3-like isoform X2	4272	0
XM_020080588.1	coiled-coil and C2 domain-containing protein 2A-like	2359	0
XM_020080589.1	leucine-rich repeat-containing protein 66	2388	0
XM_020080590.1	pre-mRNA 3'-end-processing factor FIP1	1364	0
XM_020080591.1	transcription factor Maf-like	1278	1.97E-156
XM_020080592.1	laminin subunit alpha-3-like	5325	0
XM_020080593.1	cathepsin L1-like	1059	0

XM_020080594.1	inhibitor of growth protein 5	1043	3.32E-163
XM_020080595.1	metalloendopeptidase OMA1, mitochondrial	2384	0
XM_020080596.1	ankyrin-2 isoform X5	2331	0
XM_020080597.1	enoyl-CoA hydratase domain-containing protein 2, mitochondrial-like	2158	2.67E-159
XM_020080598.1	E3 ubiquitin-protein ligase synoviolin	3007	0
XM_020080599.1	PREDICTED: uncharacterized protein LOC109625389	1496	0
XM_020080600.1	interleukin-12 receptor subunit beta-2-like	2658	0
XM_020080601.1	PREDICTED: semaphorin-4E-like	3796	0
XM_020080602.1	signal-transducing adaptor protein 2-like	1596	0
XM_020080603.1	transmembrane 6 superfamily member 1-like	1146	0
XM_020080604.1	mitochondrial coenzyme A transporter SLC25A42-like	2262	0
XM_020080605.1	cartilage intermediate layer protein 1-like	6517	0
XM_020080606.1	PREDICTED: midnolin-like	2377	8.32E-128
XM_020080607.1	PREDICTED: midnolin-like	664	7.75E-111
XM_020080608.1	spindlin-W-like isoform X2	651	2.50E-130
XM_020080609.1	integral membrane protein GPR137B-like	3390	0

XM_020080610.1	cyclic AMP-responsive element-binding protein 3-like protein 4	2087	0
XM_020080611.1	histone-lysine N-methyltransferase, H3 lysine-79 specific-like	5843	0
XM_020080612.1	cytokine receptor-like factor 1	1260	0
XM_020080613.1	PREDICTED: perilipin-3-like	1736	0
XM_020080614.1	PREDICTED: semaphorin-6B	8290	0
XM_020080615.1	single-stranded DNA-binding protein 3-like	2574	2.34E-156
XM_020080616.1	glutamate-rich protein 3	5622	0
XM_020080617.1	cytochrome P450 2J2-like	3429	0
XM_020080618.1	neurogenic locus notch homolog protein 1-like	2482	0
XM_020080619.1	growth hormone-releasing hormone receptor-like	1502	0
XM_020080620.1	small integral membrane protein 19	842	5.10E-63
XM_020080621.1	transducin-like enhancer protein 1	2595	0
XM_020080622.1	transducin-like enhancer protein 4	1969	0
XM_020080623.1	protein farnesyltransferase/geranylgeranyltransferase type-1 subunit alpha	1834	0
XM_020080624.1	AP-3 complex subunit delta-1	4017	0
XM_020080625.1	RNA-binding protein 4.1-like	1687	0
XM_020080626.1	disintegrin and metalloproteinase domain-containing protein 10-like	2951	0
XM_020080627.1	SH2 domain-containing protein 7-like	1017	0
XM_020080628.1	cytosolic carboxypeptidase 6	1066	0
XM_020080629.1	tetratricopeptide repeat protein 39A	3546	0
XM_020080630.1	leucine-rich repeat, immunoglobulin-like domain and transmembrane domain-containing protein 3	1923	0
XM_020080631.1	epidermal growth factor receptor substrate 15	3615	0
XM_020080632.1	voltage-dependent P/Q-type calcium channel subunit alpha-1A-like	10192	0
XM_020080633.1	sulfhydryl oxidase 1	3554	0
XM_020080634.1	acyl-CoA-binding domain-containing protein 5 isoform X1	1224	0
XM_020080635.1	myeloid-derived growth factor	2378	3.02E-87
XM_020080636.1	latent-transforming growth factor beta-binding protein 2	10682	0
XM_020080637.1	E3 ubiquitin-protein ligase RNF220-like	1576	0
XM_020080638.1	obscurin isoform X9	16718	0
XM_020080639.1	fidgetin-like protein 1	2315	0
XM_020080640.1	protein FAM150-like	885	7.83E-92
XM_020080641.1	ankyrin repeat and SOCS box protein 5 isoform X1	1611	0
XM_020080642.1	leucine-rich repeat-containing protein 15-like	4726	0
XM_020080643.1	mitofusin 1	3025	0
XM_020080644.1	chloride channel CLIC-like protein 1	3339	0
XM_020080645.1	afadin- and alpha-actinin-binding protein-like	2174	0
XM_020080646.1	deoxyribonuclease-2-beta-like isoform X1	1077	0
XM_020080647.1	5-hydroxytryptamine receptor 3A-like	1370	0
XM_020080648.1	ankyrin repeat and SOCS box protein 5 isoform X2	1596	0
XM_020080649.1	5-hydroxytryptamine receptor 3A-like	1764	0
XM_020080650.1	neuromedin-U receptor 1-like	1299	0
XM_020080651.1	hydroxylysine kinase-like	2379	0
XM_020080652.1	protein transport protein Sec16A isoform X1	3823	0
XM_020080653.1	ecotropic viral integration site 5 protein homolog isoform X1	4405	0
XM_020080654.1	ecotropic viral integration site 5 protein homolog isoform X2	3986	0
XM_020080655.1	ecotropic viral integration site 5 protein homolog isoform X3	4222	0
XM_020080656.1	ankyrin repeat and SOCS box protein 5 isoform X3	1819	0
XM_020080657.1	adseverin-like isoform X1	2903	0
XM_020080658.1	7-alpha-hydroxycholest-4-en-3-one 12-alpha-hydroxylase-like	2250	0
XM_020080659.1	zinc finger protein 648-like	3986	0
XM_020080660.1	zinc finger protein 648-like	4087	0
XM_020080661.1	protein FAM69A-like	2050	0
XM_020080662.1	zinc finger protein Gfi-1-like	1733	0
XM_020080663.1	retinol dehydrogenase 8-like	954	0
XM_020080664.1	ankyrin repeat and SOCS box protein 5 isoform X4	1804	0
XM_020080665.1	60S ribosomal protein L5	1090	0
XM_020080666.1	PREDICTED: uncharacterized protein C1orf146 homolog	846	2.72E-125
XM_020080667.1	growth arrest and DNA damage-inducible protein GADD45 alpha-like	1304	1.82E-111
XM_020080668.1	myosin heavy chain, fast skeletal muscle-like	860	2.78E-76
XM_020080669.1	guanine nucleotide-binding protein G(I)/G(S)/G(O) subunit gamma-12-like	2334	1.85E-36
XM_020080670.1	guanine nucleotide-binding protein G(I)/G(S)/G(O) subunit gamma-12-like	1986	1.03E-36
XM_020080671.1	guanine nucleotide-binding protein G(I)/G(S)/G(O) subunit gamma-12-like	1994	1.84E-36
XM_020080672.1	natural killer enhancing factor	1002	2.72E-163

XM_020080673.1	phosphatidylinositol 4-phosphate 5-kinase type-1 gamma isoform X1	5483	0
XM_020080674.1	phosphatidylinositol 4-phosphate 5-kinase type-1 gamma isoform X2	5438	0
XM_020080675.1	phosphatidylinositol 4-phosphate 5-kinase type-1 gamma isoform X3	5393	0
XM_020080676.1	fibronectin type III and SPRY domain-containing protein 1	3204	0
XM_020080677.1	neuronal membrane glycoprotein M6-a	1315	0
XM_020080678.1	E3 ubiquitin-protein ligase MARCH2 isoform X1	5439	1.59E-155
XM_020080679.1	E3 ubiquitin-protein ligase MARCH2 isoform X2	5426	2.27E-153
XM_020080680.1	PREDICTED: uncharacterized protein LOC109625459 isoform X1	827	2.72E-154
XM_020080681.1	PREDICTED: uncharacterized protein LOC109625459 isoform X2	972	1.06E-155
XM_020080682.1	PREDICTED: uncharacterized protein LOC109625459 isoform X3	782	1.01E-142
XM_020080683.1	ras-related protein Rab-11B-like	1375	2.03E-160
XM_020080684.1	ras-related protein Rab-11B-like	2062	1.57E-157
XM_020080685.1	neuronal membrane glycoprotein M6-a	1222	0
XM_020080686.1	protein-glutamine gamma-glutamyltransferase K-like	2799	0
XM_020080687.1	PREDICTED: vitellogenin-like	4287	0
XM_020080688.1	zinc finger and BTB domain-containing protein 11	3755	0
XM_020080689.1	B-cell CLL/lymphoma 6 member B protein-like	3357	0
XM_020080690.1	tumor protein 63 isoform X1	3283	0
XM_020080691.1	tumor protein 63 isoform X2	3268	0
XM_020080692.1	tumor protein 63 isoform X3	3058	0
XM_020080693.1	tumor protein 63 isoform X4	3046	0
XM_020080694.1	lipoma-preferred partner homolog	7233	0
XM_020080695.1	lipoma-preferred partner homolog	7187	0
XM_020080696.1	lipoma-preferred partner homolog	6736	0
XM_020080697.1	lipoma-preferred partner homolog	6675	0
XM_020080698.1	elongation of very long chain fatty acids protein 6-like	2184	0
XM_020080699.1	tumor protein p63-regulated gene 1-like protein	3162	0
XM_020080700.1	tumor protein p63-regulated gene 1-like protein	3165	0
XM_020080701.1	MOB kinase activator 3C	1717	3.22E-160
XM_020080702.1	DNA-directed RNA polymerases I, II, and III subunit RPABC1 isoform X1	900	3.89E-142
XM_020080703.1	DNA-directed RNA polymerases I, II, and III subunit RPABC1 isoform X1	909	5.54E-140
XM_020080704.1	barH-like 2 homeobox protein	1473	0
XM_020080705.1	barH-like 2 homeobox protein	1387	0
XM_020080706.1	barH-like 2 homeobox protein	1381	0
XM_020080707.1	HAUS augmin-like complex subunit 8	1494	0
XM_020080708.1	yjeF N-terminal domain-containing protein 3 isoform X1	1034	0
XM_020080709.1	yjeF N-terminal domain-containing protein 3 isoform X2	808	3.97E-175
XM_020080710.1	tax1-binding protein 3	868	3.43E-104
XM_020080711.1	pro-epidermal growth factor	5383	0
XM_020080712.1	adapter protein CIKS	1951	0
XM_020080713.1	MPN domain-containing protein	2408	0
XM_020080714.1	tyrosine-protein kinase CSK-like isoform X1	3300	0
XM_020080715.1	acidic mammalian chitinase-like	1293	0
XM_020080716.1	cytochrome b-c1 complex subunit 6, mitochondrial	613	3.20E-48
XM_020080717.1	COMM domain-containing protein 2	1030	3.99E-144
XM_020080718.1	vesicle-trafficking protein SEC22b-B	983	5.26E-140
XM_020080719.1	PR domain zinc finger protein 5	3252	0
XM_020080720.1	sodium/glucose cotransporter 4	2672	0
XM_020080721.1	PREDICTED: uncharacterized protein C19orf60 homolog	949	7.33E-83
XM_020080722.1	muscleblind-like protein 3 isoform X1	3915	0
XM_020080723.1	follistatin-related protein 3	2136	1.31E-180
XM_020080724.1	GTP-binding protein REM 2-like isoform X1	1391	0
XM_020080725.1	GTP-binding protein REM 2-like isoform X1	1329	0
XM_020080726.1	atrial natriuretic peptide receptor 2-like	3238	0
XM_020080727.1	sodium-dependent phosphate transport protein 2B-like	2272	0
XM_020080728.1	PREDICTED: cadherin-24-like	4008	0
XM_020080729.1	excitatory amino acid transporter 5-like	1737	0
XM_020080730.1	muscleblind-like protein 3 isoform X1	3956	0
XM_020080731.1	PREDICTED: uncharacterized protein LOC109625495 isoform X1	1348	0
XM_020080732.1	PREDICTED: uncharacterized protein LOC109625495 isoform X2	783	1.72E-105
XM_020080733.1	U6 snRNA-associated Sm-like protein LSm2	804	2.15E-64
XM_020080734.1	kelch-like protein 21	2254	0
XM_020080735.1	sushi, nidogen and EGF-like domain-containing protein 1	5036	0
XM_020080736.1	magnesium transporter NIPA1	1949	5.37E-155
XM_020080737.1	NADH dehydrogenase [ubiquinone] 1 beta subcomplex subunit 5, mitochondrial	718	1.49E-118
XM_020080738.1	muscleblind-like protein 3 isoform X1	6669	0
XM_020080739.1	paxillin-like isoform X2	2586	0
XM_020080740.1	transcription factor Sox-14	2038	2.52E-155
XM_020080741.1	selenocysteine lyase	1688	0
XM_020080742.1	voltage-gated hydrogen channel 1	1308	1.93E-124
XM_020080743.1	tryptophan 2,3-dioxygenase	2482	0
XM_020080744.1	nuclear factor 1 C-type	3148	0
XM_020080745.1	tctex1 domain-containing protein 1-B-like	939	4.51E-85
XM_020080746.1	tctex1 domain-containing protein 1-B-like	873	2.10E-85
XM_020080747.1	mucosa-associated lymphoid tissue lymphoma translocation protein 1-like	2817	0
XM_020080748.1	NADH dehydrogenase [ubiquinone] 1 alpha subcomplex subunit 11	545	3.41E-96
XM_020080749.1	adhesion G protein-coupled receptor L4	2685	0
XM_020080750.1	muscleblind-like protein 3 isoform X1	6672	0
XM_020080751.1	PREDICTED: podocan	2611	0

XM_020080752.1	PREDICTED: podocan	2639	0
XM_020080753.1	PREDICTED: podocan	2706	0
XM_020080754.1	PREDICTED: podocan	2550	0
XM_020080755.1	hyaluronan and proteoglycan link protein 3	2136	0
XM_020080756.1	proprotein convertase subtilisin/kexin type 9	2740	0
XM_020080757.1	vesicle transport protein SFT2B-like	925	1.12E-113
XM_020080758.1	vesicle transport protein SFT2B-like	915	9.98E-114
XM_020080759.1	PREDICTED: rootletin-like	6610	0
XM_020080760.1	GS homeobox 2	1265	1.42E-177
XM_020080761.1	transmembrane protein 44 isoform X1	1482	0
XM_020080762.1	transmembrane protein 44 isoform X2	1458	0
XM_020080763.1	muscleblind-like protein 3 isoform X4	2080	0
XM_020080764.1	transmembrane protein 44 isoform X3	1398	0
XM_020080765.1	transmembrane protein 44 isoform X4	1386	0
XM_020080766.1	membrane protein FAM159B-like	2442	3.16E-94
XM_020080767.1	protein furry homolog-like isoform X1	11165	0
XM_020080768.1	protein furry homolog-like isoform X1	11146	0
XM_020080769.1	protein furry homolog-like isoform X10	11127	0
XM_020080770.1	protein furry homolog-like isoform X1	11126	0
XM_020080771.1	protein furry homolog-like isoform X9	11119	0
XM_020080772.1	protein furry homolog-like isoform X10	11107	0
XM_020080773.1	protein furry homolog-like isoform X9	11081	0
XM_020080774.1	protein furry homolog-like isoform X1	11611	0
XM_020080775.1	protein furry homolog-like isoform X9	11566	0
XM_020080776.1	protein furry homolog-like isoform X1	11351	0
XM_020080777.1	protein furry homolog-like isoform X10	11575	0
XM_020080778.1	ras-related protein Rap-2c	2389	3.75E-115
XM_020080779.1	protein furry homolog-like isoform X11	11511	0
XM_020080780.1	anaphase-promoting complex subunit 4 isoform X1	2824	0
XM_020080781.1	anaphase-promoting complex subunit 4 isoform X1	2892	0
XM_020080782.1	anaphase-promoting complex subunit 4 isoform X1	2807	0
XM_020080783.1	SLAIN motif-containing protein 2 isoform X1	2231	0
XM_020080784.1	SLAIN motif-containing protein 2 isoform X2	2228	0
XM_020080785.1	sodium/bile acid cotransporter	1799	0
XM_020080786.1	sodium/bile acid cotransporter 4 isoform X2	1224	2.19E-178
XM_020080787.1	zygote arrest protein 1	1224	0
XM_020080788.1	sphingosine 1-phosphate receptor 4	1899	0
XM_020080789.1	espin-like protein	3348	0
XM_020080790.1	putative bifunctional UDP-N-acetylglucosamine transferase and deubiquitinase ALG13	787	1.99E-118
XM_020080791.1	PREDICTED: uncharacterized protein LOC109625528	2364	2.94E-129
XM_020080792.1	LIM/homeobox protein Lhx8 isoform X1	1409	0
XM_020080793.1	LIM/homeobox protein Lhx8 isoform X2	1425	0
XM_020080794.1	angiotensin-converting enzyme 2 isoform X2	2492	2.16E-116
XM_020080795.1	one cut domain family member 2-like	1421	2.71E-179
XM_020080796.1	radial spoke head protein 4 homolog A-like	2028	0
XM_020080797.1	forkhead box protein E4-like	1848	0
XM_020080798.1	histamine H3 receptor-like	2353	0
XM_020080799.1	alpha-N-acetylgalactosaminide alpha-2,6-sialyltransferase 5-like	1700	0
XM_020080800.1	RNA polymerase II elongation factor ELL	1033	0
XM_020080801.1	TNFAIP3-interacting protein 1 isoform X1	3746	0
XM_020080802.1	hydroxysteroid 11-beta-dehydrogenase 1-like protein	1056	0
XM_020080803.1	potassium/sodium hyperpolarization-activated cyclic nucleotide-gated channel 2	3072	0
XM_020080804.1	E3 ubiquitin-protein ligase TRIM33-like	969	7.62E-107
XM_020080805.1	protein ANKUB1	2209	0
XM_020080806.1	low choriolytic enzyme-like	1124	8.10E-128
XM_020080807.1	receptor-transporting protein 3-like	769	2.41E-118
XM_020080808.1	TNFAIP3-interacting protein 1 isoform X1	3795	0
XM_020080809.1	epoxide hydrolase 4	1934	0
XM_020080810.1	coiled-coil domain-containing protein 181	2228	0
XM_020080811.1	cyclic nucleotide-gated cation channel alpha-3	1971	0
XM_020080812.1	ankycorbin isoform X1	3733	0
XM_020080813.1	LIM homeobox transcription factor 1-alpha-like	2364	0
XM_020080814.1	spermatogenesis-associated protein 1 isoform X1	2129	0
XM_020080815.1	spermatogenesis-associated protein 1 isoform X2	2114	0
XM_020080816.1	TNFAIP3-interacting protein 1 isoform X1	3713	0
XM_020080817.1	protein sel-1 homolog 3	3150	0
XM_020080818.1	OCIA domain-containing protein 2	1261	1.68E-117
XM_020080819.1	OCIA domain-containing protein 2	1197	7.87E-118
XM_020080820.1	OCIA domain-containing protein 2	964	4.99E-119
XM_020080821.1	deoxyribonuclease-2-beta-like isoform X1	1696	0
XM_020080822.1	deoxyribonuclease-2-beta-like isoform X2	1658	0
XM_020080823.1	complement C1q tumor necrosis factor-related protein 2	1201	9.15E-170
XM_020080824.1	complement C1q tumor necrosis factor-related protein 2	1261	5.70E-163
XM_020080825.1	TNFAIP3-interacting protein 1 isoform X1	3603	0
XM_020080826.1	Rieske domain-containing protein-like isoform X1	1169	1.94E-94
XM_020080827.1	excitatory amino acid transporter 1-like	1952	3.38E-112
XM_020080828.1	putative claudin-24	1244	9.79E-132
XM_020080829.1	C-C motif chemokine 17-like	1438	1.14E-71
XM_020080830.1	TNFAIP3-interacting protein 1 isoform X1	3686	0

XM_020080831.1	prostaglandin E2 receptor EP1 subtype-like	2039	0
XM_020080832.1	small integral membrane protein 24	1211	4.94E-48
XM_020080833.1	axonemal dynein light intermediate polypeptide 1-like	1056	3.53E-152
XM_020080834.1	relaxin-3 receptor 1-like	1212	0
XM_020080835.1	PREDICTED: claudin-18-like	873	1.55E-146
XM_020080836.1	TNFAIP3-interacting protein 1 isoform X1	3656	0
XM_020080837.1	tumor necrosis factor ligand superfamily member 6-like	804	0
XM_020080838.1	guanylate kinase-like	591	2.76E-145
XM_020080839.1	PREDICTED: uncharacterized protein C1orf228 homolog	1376	0
XM_020080840.1	leucine-rich repeat-containing protein 52-like	1110	0
XM_020080841.1	claudin-19 isoform X2	725	6.24E-122
XM_020080842.1	claudin-19 isoform X2	721	6.00E-122
XM_020080843.1	TNFAIP3-interacting protein 1 isoform X1	3530	0
XM_020080844.1	homeobox protein ESX1-like isoform X1	870	1.18E-151
XM_020080845.1	homeobox protein ESX1-like isoform X1	847	1.95E-108
XM_020080846.1	forkhead box protein D5-C-like	901	2.62E-168
XM_020080847.1	protein Jade-3	5034	0
XM_020080848.1	protein Jade-3	4678	0
XM_020080849.1	sodium/hydrogen exchanger 7 isoform X1	6855	0
XM_020080850.1	sodium/hydrogen exchanger 7 isoform X2	2623	0
XM_020080851.1	sodium/hydrogen exchanger 7 isoform X1	6380	0
XM_020080852.1	carbohydrate sulfotransferase 6-like	2668	0
XM_020080853.1	protein XRP2	2495	0
XM_020080854.1	PI-PLC X domain-containing protein 1-like isoform X1	2098	0
XM_020080855.1	dynactin subunit 4 isoform X1	2788	0
XM_020080856.1	PI-PLC X domain-containing protein 1-like isoform X1	2180	0
XM_020080857.1	PI-PLC X domain-containing protein 1-like isoform X1	2131	0
XM_020080858.1	PI-PLC X domain-containing protein 1-like isoform X1	2055	0
XM_020080859.1	PREDICTED: neuromedin-U	704	2.45E-71
XM_020080860.1	cathepsin F	1309	0
XM_020080861.1	ubiquitin-conjugating enzyme E2 R2-like	3457	5.40E-143
XM_020080862.1	dynactin subunit 4 isoform X2	2766	0
XM_020080863.1	neurogenic locus notch homolog protein 2-like	10052	0
XM_020080864.1	UDP-N-acetylglucosamine transporter	1621	0
XM_020080865.1	tropomyosin alpha-4 chain isoform X1	2294	2.37E-132
XM_020080866.1	tropomyosin alpha-4 chain isoform X2	1219	1.09E-136
XM_020080867.1	tropomyosin alpha-4 chain isoform X3	2406	1.85E-98
XM_020080868.1	tropomyosin alpha-4 chain isoform X4	1331	1.03E-102
XM_020080869.1	ras-related protein Rab-8A	2977	1.53E-142
XM_020080870.1	40S ribosomal protein S27-like	1448	2.33E-52
XM_020080871.1	basic immunoglobulin-like variable motif-containing protein	1888	0
XM_020080872.1	basic immunoglobulin-like variable motif-containing protein	1893	0
XM_020080873.1	nucleoside diphosphate kinase 7 isoform X1	1501	0
XM_020080874.1	glutathione peroxidase 3	1098	4.46E-155
XM_020080875.1	nucleoside diphosphate kinase 7 isoform X2	1489	0
XM_020080876.1	inosine-uridine preferring nucleoside hydrolase-like	1210	0
XM_020080877.1	methionine synthase	1144	0
XM_020080878.1	sodium/potassium-transporting ATPase subunit beta-1-like	2198	0
XM_020080879.1	chromatin assembly factor 1 subunit A	3773	0
XM_020080880.1	UBX domain-containing protein 6	3137	0
XM_020080881.1	secretory carrier-associated membrane protein 4-like isoform X2	2150	4.95E-165
XM_020080882.1	secretory carrier-associated membrane protein 4-like isoform X2	1027	1.98E-167
XM_020080883.1	secretory carrier-associated membrane protein 4-like isoform X2	947	7.23E-168
XM_020080884.1	olfactomedin-like protein 2B isoform X1	3570	0
XM_020080885.1	olfactomedin-like protein 2B isoform X2	3537	0
XM_020080886.1	BCL-6 corepressor-like protein 1 isoform X1	6377	0
XM_020080887.1	cyclic AMP-dependent transcription factor ATF-6 alpha	3691	0
XM_020080888.1	torsin-1A-interacting protein 2-like	2426	0
XM_020080889.1	torsin-1A-interacting protein 2-like	3102	0
XM_020080890.1	torsin-1A-interacting protein 2-like	3096	0
XM_020080891.1	chemokine XC receptor 1-like	1047	0
XM_020080892.1	protein PRRC2C isoform X1	9141	0
XM_020080893.1	BCL-6 corepressor-like protein 1 isoform X1	6373	0
XM_020080894.1	protein PRRC2C isoform X1	9147	0
XM_020080895.1	protein PRRC2C isoform X1	9135	0
XM_020080896.1	protein PRRC2C isoform X1	9132	0
XM_020080897.1	protein PRRC2C isoform X1	9126	0
XM_020080898.1	muellerian-inhibiting factor	2018	0
XM_020080899.1	ornithine decarboxylase antizyme 1	1285	1.32E-133
XM_020080900.1	nicalin isoform X1	3451	0
XM_020080901.1	BCL-6 corepressor-like protein 1 isoform X1	6662	0
XM_020080902.1	nicalin isoform X2	3449	0
XM_020080903.1	nicalin isoform X3	3404	0
XM_020080904.1	nicalin isoform X4	3402	0
XM_020080905.1	ceramide synthase 2-like	6273	0
XM_020080906.1	cortexin-1-like	4689	4.08E-43
XM_020080907.1	cortexin-1-like	3921	3.38E-43
XM_020080908.1	cortexin-1-like	4222	3.66E-43
XM_020080909.1	cortexin-1-like	3650	3.14E-43

XM_020080910.1	cortexin-1-like	5008	4.37E-43
XM_020080911.1	BCL-6 corepressor-like protein 1 isoform X1	6183	0
XM_020080912.1	cortexin-1-like	3542	3.04E-43
XM_020080913.1	cortexin-1-like	4729	4.12E-43
XM_020080914.1	cytochrome P450 2J2-like	2607	0
XM_020080915.1	protein Shroom2 isoform X2	6158	0
XM_020080916.1	protein Shroom2 isoform X2	6112	0
XM_020080917.1	protein Shroom2 isoform X2	5841	0
XM_020080918.1	protein Shroom2 isoform X2	6086	0
XM_020080919.1	protein Shroom2 isoform X2	5932	0
XM_020080920.1	BCL-6 corepressor-like protein 1 isoform X1	6067	0
XM_020080921.1	synapse-associated protein 1	2170	0
XM_020080922.1	synapse-associated protein 1	2459	0
XM_020080923.1	synapse-associated protein 1	2058	0
XM_020080924.1	synapse-associated protein 1	2065	0
XM_020080925.1	PREDICTED: claudin-34	1302	0
XM_020080926.1	alpha-aminoadipic semialdehyde dehydrogenase	2539	0
XM_020080927.1	actin, alpha cardiac	1965	0
XM_020080928.1	39S ribosomal protein L47, mitochondrial	1035	0
XM_020080929.1	BCL-6 corepressor-like protein 1 isoform X1	6319	0
XM_020080930.1	probable cation-transporting ATPase 13A3 isoform X1	9768	0
XM_020080931.1	probable cation-transporting ATPase 13A3 isoform X1	9923	0
XM_020080932.1	probable cation-transporting ATPase 13A3 isoform X1	9675	0
XM_020080933.1	dynamin-like 120 kDa protein, mitochondrial isoform X1	4882	0
XM_020080934.1	dynamin-like 120 kDa protein, mitochondrial isoform X2	4831	0
XM_020080935.1	dynamin-like 120 kDa protein, mitochondrial isoform X3	4726	0
XM_020080936.1	dynamin-like 120 kDa protein, mitochondrial isoform X4	4675	0
XM_020080937.1	paired amphipathic helix protein Sin3a	5931	0
XM_020080938.1	BCL-6 corepressor-like protein 1 isoform X1	6073	0
XM_020080939.1	HAUS augmin-like complex subunit 5	3042	0
XM_020080940.1	protein SZT2-like isoform X1	1997	0
XM_020080941.1	protein SZT2-like isoform X2	1988	0
XM_020080942.1	putative hydroxypyruvate isomerase	1087	0
XM_020080943.1	protein bicaudal C homolog 1 isoform X1	5368	0
XM_020080944.1	cytoplasmic FMR1-interacting protein 1	4576	0
XM_020080945.1	cytoplasmic FMR1-interacting protein 1	4589	0
XM_020080946.1	magnesium transporter NIPA2 isoform X1	1897	0
XM_020080947.1	BCL-6 corepressor-like protein 1 isoform X1	6373	0
XM_020080948.1	magnesium transporter NIPA2 isoform X1	2143	0
XM_020080949.1	fibronectin type III domain-containing protein 9	1002	4.80E-156
XM_020080950.1	serine/threonine-protein kinase TNNI3K	2779	0
XM_020080951.1	fucose-1-phosphate guanylyltransferase	2818	0
XM_020080952.1	acyl-coenzyme A thioesterase 11-like isoform X3	2366	0
XM_020080953.1	nuclear receptor ROR-beta-like	4884	0
XM_020080954.1	endophilin-A2-like isoform X1	3473	0
XM_020080955.1	ras-specific guanine nucleotide-releasing factor RalGPS2 isoform X1	5806	0
XM_020080956.1	ras-specific guanine nucleotide-releasing factor RalGPS2 isoform X1	5686	0
XM_020080957.1	ras-specific guanine nucleotide-releasing factor RalGPS2 isoform X1	5798	0
XM_020080958.1	NADPH oxidase 1	2326	0
XM_020080959.1	ras-specific guanine nucleotide-releasing factor RalGPS2 isoform X1	5728	0
XM_020080960.1	angiopoietin-related protein 1	2902	0
XM_020080961.1	angiopoietin-related protein 1	2891	0
XM_020080962.1	TBC1 domain family member 23 isoform X1	3849	0
XM_020080963.1	TBC1 domain family member 23 isoform X2	3804	0
XM_020080964.1	omega-amidase NIT2 isoform X1	1576	0
XM_020080965.1	omega-amidase NIT2 isoform X1	1225	0
XM_020080966.1	omega-amidase NIT2 isoform X1	1541	0
XM_020080967.1	ICOS ligand-like	1737	0
XM_020080968.1	interleukin-12 receptor subunit beta-2-like	4041	0
XM_020080969.1	plasminogen activator inhibitor 1 RNA-binding protein isoform X1	2632	2.95E-150
XM_020080970.1	plasminogen activator inhibitor 1 RNA-binding protein isoform X2	2627	5.49E-150
XM_020080971.1	cleavage stimulation factor subunit 2	1919	0
XM_020080972.1	plasminogen activator inhibitor 1 RNA-binding protein isoform X3	2630	2.93E-149
XM_020080973.1	plasminogen activator inhibitor 1 RNA-binding protein isoform X4	2625	2.10E-149
XM_020080974.1	3-keto-steroid reductase	1199	0
XM_020080975.1	elongation factor 2-like	2899	0
XM_020080976.1	ubiquitin carboxyl-terminal hydrolase 24 isoform X1	10815	0
XM_020080977.1	ubiquitin carboxyl-terminal hydrolase 24 isoform X2	10611	0
XM_020080978.1	ubiquitin carboxyl-terminal hydrolase 24 isoform X3	10797	0
XM_020080979.1	ubiquitin carboxyl-terminal hydrolase 24 isoform X4	10593	0
XM_020080980.1	ubiquitin carboxyl-terminal hydrolase 24 isoform X5	10722	0
XM_020080981.1	cleavage stimulation factor subunit 2	1948	0
XM_020080982.1	centrosome-associated protein 350 isoform X1	13093	0
XM_020080983.1	centrosome-associated protein 350 isoform X1	12919	0
XM_020080984.1	centrosome-associated protein 350 isoform X1	12970	0
XM_020080985.1	nexilin isoform X1	2814	0
XM_020080986.1	neuroligin-3-like isoform X1	3138	0
XM_020080987.1	nexilin isoform X1	2616	0
XM_020080988.1	nexilin isoform X1	3709	0

XM_020080989.1	nexilin isoform X1	2769	0
XM_020080990.1	probable ubiquitin carboxyl-terminal hydrolase FAM188B isoform X1	3786	0
XM_020080991.1	probable ubiquitin carboxyl-terminal hydrolase FAM188B isoform X2	3753	0
XM_020080992.1	cytochrome P450 2J2-like isoform X1	2092	0
XM_020080993.1	cytochrome P450 2J2-like isoform X2	2089	0
XM_020080994.1	cytochrome P450 2J5-like	2494	0
XM_020080995.1	protein FAM122B isoform X1	3471	5.93E-169
XM_020080996.1	cytochrome P450 2J2-like	1838	0
XM_020080997.1	FERM, RhoGEF and pleckstrin domain-containing protein 2 isoform X1	5030	0
XM_020080998.1	FERM, RhoGEF and pleckstrin domain-containing protein 2 isoform X2	4983	0
XM_020080999.1	TGF-beta-activated kinase 1 and MAP3K7-binding protein 2 isoform X1	2893	0
XM_020081000.1	TGF-beta-activated kinase 1 and MAP3K7-binding protein 2 isoform X1	2724	0
XM_020081001.1	transmembrane protein 47-like isoform X1	2950	6.55E-98
XM_020081002.1	protein zyg-11 homolog B	2623	0
XM_020081003.1	protein FAM122B isoform X2	3467	4.00E-168
XM_020081004.1	cytochrome c oxidase assembly factor 7	1159	1.60E-170
XM_020081005.1	macrophage colony-stimulating factor 1 receptor isoform X1	6430	0
XM_020081006.1	mast/stem cell growth factor receptor Kit isoform X1	5540	0
XM_020081007.1	mast/stem cell growth factor receptor Kit isoform X2	5519	0
XM_020081008.1	PREDICTED: lamin-B2	4754	0
XM_020081009.1	guanine nucleotide-binding protein G(I)/G(S)/G(T) subunit beta-1-like	5978	0
XM_020081010.1	guanine nucleotide-binding protein G(I)/G(S)/G(T) subunit beta-1-like	5896	0
XM_020081011.1	SUN domain-containing ossification factor isoform X1	7203	0
XM_020081012.1	SUN domain-containing ossification factor isoform X2	4460	0
XM_020081013.1	SUN domain-containing ossification factor isoform X3	4331	0
XM_020081014.1	guanine nucleotide-binding protein G(q) subunit alpha isoform X1	6554	0
XM_020081015.1	guanine nucleotide-binding protein subunit alpha-11 isoform X2	6554	0
XM_020081016.1	motile sperm domain-containing protein 1	1565	7.26E-158
XM_020081017.1	fumarate hydratase, mitochondrial	2221	0
XM_020081018.1	39S ribosomal protein L54, mitochondrial	1252	9.66E-90
XM_020081019.1	protein FAM32A	791	1.01E-100

XM_020081020.1	adhesion G protein-coupled receptor L2 isoform X1	7877	0
XM_020081021.1	adhesion G protein-coupled receptor L2 isoform X2	7353	0
XM_020081022.1	motile sperm domain-containing protein 1	1645	1.84E-157
XM_020081023.1	adhesion G protein-coupled receptor L2 isoform X3	7351	0
XM_020081024.1	adhesion G protein-coupled receptor L2 isoform X4	7201	0
XM_020081025.1	palladin isoform X1	6055	0
XM_020081026.1	palladin isoform X2	6052	0
XM_020081027.1	palladin isoform X1	6018	0
XM_020081028.1	palladin isoform X1	5955	0
XM_020081029.1	palladin isoform X2	4981	0
XM_020081030.1	palladin isoform X2	3799	0
XM_020081031.1	carbonyl reductase family member 4	3225	2.86E-165
XM_020081032.1	vacuolar protein sorting-associated protein 4B-like	2209	0
XM_020081033.1	ankyrin repeat domain-containing protein 29 isoform X1	1354	0
XM_020081034.1	ankyrin repeat domain-containing protein 29 isoform X2	1329	0
XM_020081035.1	ankyrin repeat domain-containing protein 29 isoform X3	1261	0
XM_020081036.1	hypoxanthine-guanine phosphoribosyltransferase	1526	3.95E-147
XM_020081037.1	ankyrin repeat domain-containing protein 29 isoform X4	1376	3.32E-173
XM_020081038.1	ankyrin repeat domain-containing protein 29 isoform X4	1396	4.14E-173
XM_020081039.1	pleckstrin homology domain-containing family B member 2	2118	4.64E-89
XM_020081040.1	amino-terminal enhancer of split isoform X1	5728	3.18E-102
XM_020081041.1	amino-terminal enhancer of split isoform X2	5725	9.24E-114
XM_020081042.1	amino-terminal enhancer of split isoform X3	5653	1.06E-81
XM_020081043.1	amino-terminal enhancer of split isoform X4	5650	4.61E-96
XM_020081044.1	transcriptional repressor p66-alpha-like isoform X1	4529	0
XM_020081045.1	transcriptional repressor p66-alpha-like isoform X2	4458	0
XM_020081046.1	serine/threonine-protein kinase PAK 2	3197	0
XM_020081047.1	serine/threonine-protein kinase PAK 2	3211	0
XM_020081048.1	serine/threonine-protein kinase PAK 2	3013	0
XM_020081049.1	phosphatidylinositol-glycan biosynthesis class X protein	1837	1.07E-165
XM_020081050.1	RUN and FYVE domain-containing protein 1-like	3900	0
XM_020081051.1	centrosomal protein of 19 kDa	892	1.34E-113
XM_020081052.1	unconventional myosin-IXb isoform X1	7660	0
XM_020081053.1	unconventional myosin-IXb isoform X2	7657	0
XM_020081054.1	unconventional myosin-IXb isoform X3	7609	0
XM_020081055.1	unconventional myosin-IXb isoform X4	7546	0
XM_020081056.1	unconventional myosin-IXb isoform X1	6925	0
XM_020081057.1	protein unc-13 homolog A-like isoform X1	6290	0
XM_020081058.1	protein unc-13 homolog A-like isoform X2	6284	0
XM_020081059.1	protein unc-13 homolog A-like isoform X3	6254	0
XM_020081060.1	protein unc-13 homolog A-like isoform X4	6233	0
XM_020081061.1	protein unc-13 homolog A-like isoform X5	5601	0
XM_020081062.1	protein unc-13 homolog A-like isoform X6	6197	0
XM_020081063.1	unconventional myosin-Va-like	7536	0
XM_020081064.1	heterogeneous nuclear ribonucleoprotein H isoform X1	2182	0
XM_020081065.1	methyl-CpG-binding domain protein 3-like isoform X1	1153	0
XM_020081066.1	methyl-CpG-binding domain protein 3-like isoform X1	1108	0
XM_020081067.1	methyl-CpG-binding domain protein 3-like isoform X2	927	0

XM_020081068.1	methyl-CpG-binding domain protein 3-like isoform X3	944	0
XM_020081069.1	methyl-CpG-binding domain protein 3-like isoform X4	930	0
XM_020081070.1	methyl-CpG-binding domain protein 3-like isoform X5	1059	0
XM_020081071.1	26S proteasome non-ATPase regulatory subunit 1	3276	0
XM_020081072.1	5-hydroxytryptamine receptor 2B	3384	0
XM_020081073.1	heterogeneous nuclear ribonucleoprotein H isoform X1	1978	0
XM_020081074.1	deoxynucleotidyltransferase terminal-interacting protein 2	3262	0
XM_020081075.1	spermatogenesis-associated protein 6-like isoform X1	3098	0
XM_020081076.1	spermatogenesis-associated protein 6-like isoform X2	2997	0
XM_020081077.1	spermatogenesis-associated protein 6-like isoform X3	1490	0
XM_020081078.1	spermatogenesis-associated protein 6-like isoform X4	1385	0
XM_020081079.1	Abelson tyrosine-protein kinase 2-like isoform X1	7617	0
XM_020081080.1	Abelson tyrosine-protein kinase 2-like isoform X2	7615	0
XM_020081081.1	profilin-2 isoform X1	2453	5.31E-93
XM_020081082.1	heterogeneous nuclear ribonucleoprotein H isoform X1	2179	0
XM_020081083.1	profilin-2 isoform X2	909	1.43E-98
XM_020081084.1	TSC22 domain family protein 2-like isoform X1	4250	0
XM_020081085.1	TSC22 domain family protein 2-like isoform X2	3215	0
XM_020081086.1	TSC22 domain family protein 2-like isoform X3	2586	0
XM_020081087.1	ER degradation-enhancing alpha-mannosidase-like protein 3 isoform X1	4869	0
XM_020081088.1	ER degradation-enhancing alpha-mannosidase-like protein 3 isoform X1	4781	0
XM_020081089.1	ER degradation-enhancing alpha-mannosidase-like protein 3 isoform X1	4917	0
XM_020081090.1	myocyte-specific enhancer factor 2B	1531	0
XM_020081091.1	heterogeneous nuclear ribonucleoprotein H isoform X1	2186	0
XM_020081092.1	transmembrane protein 221	1811	3.79E-159
XM_020081093.1	DNA-binding protein RFXANK	1048	1.34E-167
XM_020081094.1	DNA-binding protein RFXANK	1058	1.56E-167
XM_020081095.1	BLOC-1-related complex subunit 8 isoform X1	2018	2.89E-112
XM_020081096.1	BLOC-1-related complex subunit 8 isoform X2	2029	2.62E-111
XM_020081097.1	nuclear receptor 2C2-associated protein	853	2.37E-100
XM_020081098.1	protein groucho-1	1459	7.69E-68
XM_020081099.1	3-oxo-5-alpha-steroid 4-dehydrogenase 1	1657	0
XM_020081100.1	transmembrane protein 165	3082	2.51E-175
XM_020081101.1	AN1-type zinc finger protein 5-like isoform X1	4169	8.40E-111
XM_020081102.1	AN1-type zinc finger protein 5-like isoform X1	4443	1.51E-110
XM_020081103.1	AN1-type zinc finger protein 5-like isoform X1	1771	1.92E-105
XM_020081104.1	proheparin-binding EGF-like growth factor	1285	2.79E-108
XM_020081105.1	serine/arginine-rich splicing factor 11	2209	3.55E-134
XM_020081106.1	trafficking protein particle complex subunit 8 isoform X1	5785	0
XM_020081107.1	trafficking protein particle complex subunit 8 isoform X2	5701	0
XM_020081108.1	guanine nucleotide-binding protein subunit alpha-11-like	5894	0
XM_020081109.1	guanine nucleotide-binding protein subunit alpha-11-like	5646	0
XM_020081110.1	phosphoribosylformylglycinamidine synthase	4452	0
XM_020081111.1	phosphoribosylformylglycinamidine synthase	4349	0
XM_020081112.1	phosphoribosylformylglycinamidine synthase	4285	0
XM_020081113.1	E3 ubiquitin-protein ligase RNF170-like	1250	2.93E-137
XM_020081114.1	importin-13-like isoform X1	5164	0
XM_020081115.1	coagulation factor V-like	5367	0
XM_020081116.1	bifunctional heparan sulfate N-deacetylase/N-sulfotransferase 1	5333	0
XM_020081117.1	importin-13-like isoform X1	5091	0
XM_020081118.1	transmembrane protein 131 isoform X1	6292	0
XM_020081119.1	transmembrane protein 131 isoform X2	6282	0
XM_020081120.1	PREDICTED: uncharacterized protein C1orf112 homolog	3675	0
XM_020081121.1	PREDICTED: uncharacterized protein C1orf112 homolog	3680	0
XM_020081122.1	PREDICTED: uncharacterized protein C1orf112 homolog	3545	0
XM_020081123.1	protein-associating with the carboxyl-terminal domain of ezrin	3207	0
XM_020081124.1	protein-associating with the carboxyl-terminal domain of ezrin	3018	0
XM_020081125.1	F-box-like/WD repeat-containing protein TBL1XR1 isoform X1	5318	0
XM_020081126.1	F-box-like/WD repeat-containing protein TBL1XR1 isoform X1	5220	0
XM_020081127.1	4-trimethylaminobutyraldehyde dehydrogenase	2160	0
XM_020081128.1	mediator of RNA polymerase II transcription subunit 8 isoform X1	1260	8.53E-179
XM_020081129.1	pre-mRNA-splicing factor RBM22	1723	0
XM_020081130.1	mediator of RNA polymerase II transcription subunit 8 isoform X2	1239	4.61E-177
XM_020081131.1	rho guanine nucleotide exchange factor 18-like isoform X1	6854	0
XM_020081132.1	rho guanine nucleotide exchange factor 18-like isoform X2	5242	0
XM_020081133.1	rho guanine nucleotide exchange factor 18-like isoform X3	4977	0
XM_020081134.1	rho guanine nucleotide exchange factor 18-like isoform X4	5200	0
XM_020081135.1	rho guanine nucleotide exchange factor 18-like isoform X5	5197	0
XM_020081136.1	rho guanine nucleotide exchange factor 18-like isoform X6	6583	0
XM_020081137.1	outer dense fiber protein 3-like protein 2	921	0
XM_020081138.1	SHC-transforming protein 2 isoform X1	4450	0
XM_020081139.1	SHC-transforming protein 2 isoform X1	4434	0
XM_020081140.1	SHC-transforming protein 2 isoform X1	4485	0
XM_020081141.1	SHC-transforming protein 2 isoform X1	4531	0
XM_020081142.1	SHC-transforming protein 2 isoform X1	4470	0
XM_020081143.1	SHC-transforming protein 2 isoform X1	3892	0
XM_020081144.1	myozenin-2-like isoform X2	1477	0
XM_020081145.1	cyclin-dependent kinases regulatory subunit 1-like	532	3.82E-50
XM_020081146.1	protein strawberry notch homolog 2 isoform X1	6535	0

XM_020081147.1	protein strawberry notch homolog 2 isoform X2	6532	0
XM_020081148.1	protein strawberry notch homolog 2 isoform X3	6448	0
XM_020081149.1	protein strawberry notch homolog 2 isoform X4	6269	0
XM_020081150.1	gamma-aminobutyric acid receptor subunit beta-3 isoform X1	3683	0
XM_020081151.1	gamma-aminobutyric acid receptor subunit beta-3 isoform X2	1611	0
XM_020081152.1	gamma-aminobutyric acid receptor subunit beta-3 isoform X1	1725	0
XM_020081153.1	myozenin-2-like isoform X2	1405	1.19E-168
XM_020081154.1	gamma-aminobutyric acid receptor subunit alpha-5	3990	0
XM_020081155.1	gamma-aminobutyric acid receptor subunit alpha-5	1543	0
XM_020081156.1	gamma-aminobutyric acid receptor subunit gamma-3-like	1967	0
XM_020081157.1	PREDICTED: mucolipin-2	2986	0
XM_020081158.1	E3 SUMO-protein ligase PIAS4	4779	0
XM_020081159.1	heterogeneous nuclear ribonucleoprotein M-like isoform X1	2553	0
XM_020081160.1	heterogeneous nuclear ribonucleoprotein M-like isoform X2	2179	0
XM_020081161.1	arf-GAP with coiled-coil, ANK repeat and PH domain-containing protein 2 isoform X1	4616	0
XM_020081162.1	arf-GAP with coiled-coil, ANK repeat and PH domain-containing protein 2 isoform X2	4559	0
XM_020081163.1	arf-GAP with coiled-coil, ANK repeat and PH domain-containing protein 2 isoform X3	4547	0
XM_020081164.1	arf-GAP with coiled-coil, ANK repeat and PH domain-containing protein 2 isoform X4	4514	0
XM_020081165.1	suppressor of tumorigenicity 14 protein homolog	3052	0
XM_020081166.1	stress-70 protein, mitochondrial	3528	0
XM_020081167.1	mitochondrial import inner membrane translocase subunit Tim13	959	9.07E-53
XM_020081168.1	histone deacetylase complex subunit SAP130	4582	0
XM_020081169.1	histone deacetylase complex subunit SAP130	3292	0
XM_020081170.1	histone deacetylase complex subunit SAP130	3348	0
XM_020081171.1	endothelin-converting enzyme 2-like	2272	0
XM_020081172.1	inosine triphosphate pyrophosphatase	881	3.18E-153
XM_020081173.1	ran guanine nucleotide release factor	1034	1.93E-137
XM_020081174.1	tight junction protein ZO-3-like isoform X3	5040	0
XM_020081175.1	tight junction protein ZO-3-like isoform X3	4915	0
XM_020081176.1	tight junction protein ZO-3-like isoform X3	4743	0
XM_020081177.1	tight junction protein ZO-3-like isoform X3	4871	0
XM_020081178.1	mediator of RNA polymerase II transcription subunit 12 isoform X1	6949	0
XM_020081179.1	dual specificity mitogen-activated protein kinase kinase 2 isoform X1	3627	0
XM_020081180.1	dual specificity mitogen-activated protein kinase kinase 2 isoform X2	3616	0
XM_020081181.1	dimethylaniline monooxygenase [N-oxide-forming] 5-like	2379	0
XM_020081182.1	dimethylaniline monooxygenase [N-oxide-forming] 5-like	1996	0
XM_020081183.1	dimethylaniline monooxygenase [N-oxide-forming] 5-like	2016	0
XM_020081184.1	dimethylaniline monooxygenase [N-oxide-forming] 5-like	1967	0
XM_020081185.1	oxysterol-binding protein-related protein 9 isoform X1	4212	0
XM_020081186.1	oxysterol-binding protein-related protein 9 isoform X2	4174	0
XM_020081187.1	mediator of RNA polymerase II transcription subunit 12 isoform X2	6866	0
XM_020081188.1	oxysterol-binding protein-related protein 9 isoform X3	4091	0
XM_020081189.1	oxysterol-binding protein-related protein 9 isoform X4	4106	0
XM_020081190.1	oxysterol-binding protein-related protein 9 isoform X5	4067	0
XM_020081191.1	oxysterol-binding protein-related protein 9 isoform X6	4191	0
XM_020081192.1	oxysterol-binding protein-related protein 9 isoform X6	3982	0
XM_020081193.1	PREDICTED: vitellogenin-1-like	5021	0
XM_020081194.1	transmembrane protein 161A	3707	0
XM_020081195.1	run domain Beclin-1-interacting and cysteine-rich domain-containing protein	3309	0
XM_020081196.1	E3 ubiquitin-protein ligase SH3RF1	5474	0
XM_020081197.1	methionine--tRNA ligase, mitochondrial	2996	0
XM_020081198.1	insulin receptor-like	6150	0
XM_020081199.1	epidermal growth factor receptor substrate 15-like 1 isoform X1	7446	0
XM_020081200.1	epidermal growth factor receptor substrate 15-like 1 isoform X2	7431	0
XM_020081201.1	epidermal growth factor receptor substrate 15-like 1 isoform X3	7427	0
XM_020081202.1	epidermal growth factor receptor substrate 15-like 1 isoform X4	7413	0
XM_020081203.1	epidermal growth factor receptor substrate 15-like 1 isoform X5	7394	0
XM_020081204.1	epidermal growth factor receptor substrate 15-like 1 isoform X6	7380	0
XM_020081205.1	epidermal growth factor receptor substrate 15-like 1 isoform X7	7360	0
XM_020081206.1	PREDICTED: vitellogenin-2-like	5060	0
XM_020081207.1	disabled homolog 1 isoform X1	3634	0
XM_020081208.1	disabled homolog 1 isoform X2	3537	0
XM_020081209.1	disabled homolog 1 isoform X3	3517	0
XM_020081210.1	disabled homolog 1 isoform X1	3478	0
XM_020081211.1	synaptotagmin-like protein 4	2905	0
XM_020081212.1	zinc finger RNA-binding protein-like isoform X1	4873	0
XM_020081213.1	zinc finger RNA-binding protein-like isoform X2	4870	0
XM_020081214.1	zinc finger RNA-binding protein-like isoform X3	4870	0
XM_020081215.1	zinc finger RNA-binding protein-like isoform X4	4858	0
XM_020081216.1	zinc finger RNA-binding protein-like isoform X5	4855	0
XM_020081217.1	zinc finger RNA-binding protein-like isoform X1	4800	0
XM_020081218.1	zinc finger RNA-binding protein-like isoform X7	4797	0
XM_020081219.1	zinc finger RNA-binding protein-like isoform X7	4794	0
XM_020081220.1	protein mago nashi homolog	705	3.74E-106
XM_020081221.1	synaptotagmin-like protein 4	2654	0
XM_020081222.1	tetratricopeptide repeat protein 39C	4203	0
XM_020081223.1	tetratricopeptide repeat protein 39C	4320	0
XM_020081224.1	serine--pyruvate aminotransferase	1369	0
XM_020081225.1	dihydropyrimidinase-related protein 5 isoform X2	1282	0

XM_020081226.1	phospholipid phosphatase 2-like	2793	0
XM_020081227.1	scaffold attachment factor B2-like	1991	4.33E-99
XM_020081228.1	mitochondrial import receptor subunit TOM70	3302	0
XM_020081229.1	cysteine protease ATG4B isoform X1	1555	0
XM_020081230.1	cysteine protease ATG4B isoform X2	1540	0
XM_020081231.1	thymidylate kinase	1124	4.42E-158
XM_020081232.1	sushi repeat-containing protein SRPX2	2373	0
XM_020081233.1	receptor-type tyrosine-protein phosphatase F isoform X1	8196	0
XM_020081234.1	receptor-type tyrosine-protein phosphatase F isoform X2	7889	0
XM_020081235.1	receptor-type tyrosine-protein phosphatase F isoform X3	7877	0
XM_020081236.1	receptor-type tyrosine-protein phosphatase F isoform X4	7844	0
XM_020081237.1	receptor-type tyrosine-protein phosphatase F isoform X5	7865	0
XM_020081238.1	FYVE and coiled-coil domain-containing protein 1	6429	0
XM_020081239.1	FYVE and coiled-coil domain-containing protein 1	6432	0
XM_020081240.1	angiomotin-like protein 2 isoform X1	4071	0
XM_020081241.1	angiomotin-like protein 2 isoform X1	4148	0
XM_020081242.1	angiomotin-like protein 2 isoform X1	3985	0
XM_020081243.1	angiomotin-like protein 2 isoform X1	4068	0
XM_020081244.1	MAU2 chromatid cohesion factor homolog	3702	0
XM_020081245.1	protein AATF	2093	0
XM_020081246.1	zinc-binding protein A33-like	2340	0
XM_020081247.1	tRNA wybutosine-synthesizing protein 2 homolog	2448	0
XM_020081248.1	uridine-cytidine kinase 2-A-like	2010	0
XM_020081249.1	lysine-specific demethylase 4A isoform X1	3886	0
XM_020081250.1	lysine-specific demethylase 4A isoform X1	3883	0
XM_020081251.1	lysine-specific demethylase 4A isoform X1	3883	0
XM_020081252.1	serine/arginine repetitive matrix protein 1-like	2813	0
XM_020081253.1	protein-lysine 6-oxidase-like	2086	0
XM_020081254.1	glutamine synthetase-like	1776	0
XM_020081255.1	volume-regulated anion channel subunit LRRC8A	3163	0
XM_020081256.1	volume-regulated anion channel subunit LRRC8A	2719	0
XM_020081257.1	protein ABHD17A	4614	0
XM_020081258.1	kinesin-like protein KIF1A isoform X1	8308	0
XM_020081259.1	palmitoyltransferase ZDHHC9 isoform X1	4444	0
XM_020081260.1	kinesin-like protein KIF1A isoform X2	8305	0
XM_020081261.1	kinesin-like protein KIF1A isoform X3	8287	0
XM_020081262.1	kinesin-like protein KIF1A isoform X4	8284	0
XM_020081263.1	kinesin-like protein KIF1A isoform X5	8281	0
XM_020081264.1	kinesin-like protein KIF1A isoform X6	8257	0
XM_020081265.1	kinesin-like protein KIF1A isoform X7	8239	0
XM_020081266.1	kinesin-like protein KIF1A isoform X8	8218	0
XM_020081267.1	kinesin-like protein KIF1A isoform X9	8191	0
XM_020081268.1	kinesin-like protein KIF1A isoform X10	8152	0
XM_020081269.1	kinesin-like protein KIF1A isoform X11	8263	0
XM_020081270.1	palmitoyltransferase ZDHHC9 isoform X1	4909	0
XM_020081271.1	kinesin-like protein KIF1A isoform X12	8128	0
XM_020081272.1	kinesin-like protein KIF1A isoform X13	8521	0
XM_020081273.1	ubiquitin carboxyl-terminal hydrolase 13 isoform X1	5405	0
XM_020081274.1	ubiquitin carboxyl-terminal hydrolase 13 isoform X2	5392	0
XM_020081275.1	ubiquitin carboxyl-terminal hydrolase 13 isoform X3	5373	0
XM_020081276.1	protein ECT2 isoform X1	5143	0
XM_020081277.1	protein ECT2 isoform X2	5050	0
XM_020081278.1	protein ECT2 isoform X3	4969	0
XM_020081279.1	protein ECT2 isoform X4	4566	0
XM_020081280.1	palmitoyltransferase ZDHHC9 isoform X1	4423	0
XM_020081281.1	protein ECT2 isoform X5	4299	0
XM_020081282.1	minor histocompatibility protein HA-1	4659	0
XM_020081283.1	phospholipid phosphatase 3 isoform X1	3485	0
XM_020081284.1	phospholipid phosphatase 3 isoform X2	1515	0
XM_020081285.1	centrosomal protein of 135 kDa isoform X1	4662	0
XM_020081286.1	centrosomal protein of 135 kDa isoform X1	4659	0
XM_020081287.1	centrosomal protein of 135 kDa isoform X1	3792	0
XM_020081288.1	carbohydrate sulfotransferase 14	1749	0
XM_020081289.1	cystathionine gamma-lyase	1670	0
XM_020081290.1	transforming growth factor beta receptor type 3	5929	0
XM_020081291.1	caspase recruitment domain-containing protein 14-like	4667	0
XM_020081292.1	microtubule-associated tumor suppressor 1 isoform X1	5284	0
XM_020081293.1	integumentary mucin A.1-like isoform X1	3468	4.47E-121
XM_020081294.1	cell wall protein DAN4-like isoform X2	3234	3.87E-88
XM_020081295.1	glutamate--cysteine ligase regulatory subunit	2250	0
XM_020081296.1	glutamate--cysteine ligase regulatory subunit	2159	0
XM_020081297.1	C-C motif chemokine 25-like	1763	1.92E-57
XM_020081298.1	phospholipid-metabolizing enzyme A-C1-like	761	4.74E-132
XM_020081299.1	phospholipid-metabolizing enzyme A-C1-like	775	5.54E-132
XM_020081300.1	coiled-coil domain-containing protein 50 isoform X1	3610	0
XM_020081301.1	microtubule-associated tumor suppressor 1 isoform X1	5217	0
XM_020081302.1	coiled-coil domain-containing protein 50 isoform X2	3590	0
XM_020081303.1	coiled-coil domain-containing protein 50 isoform X3	2738	2.23E-159
XM_020081304.1	coiled-coil domain-containing protein 50 isoform X4	2719	3.68E-154

XM_020081305.1	Nance-Horan syndrome protein isoform X1	7416	0
XM_020081306.1	Nance-Horan syndrome protein isoform X2	7353	0
XM_020081307.1	Nance-Horan syndrome protein isoform X3	7036	0
XM_020081308.1	Nance-Horan syndrome protein isoform X1	6711	0
XM_020081309.1	ral guanine nucleotide dissociation stimulator-like 1 isoform X1	4021	0
XM_020081310.1	ral guanine nucleotide dissociation stimulator-like 1 isoform X2	2475	0
XM_020081311.1	microtubule-associated tumor suppressor 1 isoform X1	5044	0
XM_020081312.1	single-stranded DNA-binding protein 3 isoform X3	3506	7.83E-89
XM_020081313.1	single-stranded DNA-binding protein 3 isoform X2	3526	7.18E-84
XM_020081314.1	single-stranded DNA-binding protein 3 isoform X3	3701	6.88E-89
XM_020081315.1	protein WWC3 isoform X1	4722	0
XM_020081316.1	protein WWC3 isoform X2	4698	0
XM_020081317.1	protein WWC3 isoform X3	4637	0
XM_020081318.1	protein WWC3 isoform X4	4634	0
XM_020081319.1	calcium homeostasis endoplasmic reticulum protein isoform X1	3958	0
XM_020081320.1	microtubule-associated tumor suppressor 1 isoform X1	5336	0
XM_020081321.1	calcium homeostasis endoplasmic reticulum protein isoform X2	3945	0
XM_020081322.1	calcium homeostasis endoplasmic reticulum protein isoform X3	3919	0
XM_020081323.1	calcium homeostasis endoplasmic reticulum protein isoform X4	3906	0
XM_020081324.1	probable ATP-dependent RNA helicase DDX10	3764	0
XM_020081325.1	probable phospholipid-transporting ATPase VA	7620	0
XM_020081326.1	phospholipid-transporting ATPase ID-like	9342	0
XM_020081327.1	lactosylceramide 1,3-N-acetyl-beta-D-glucosaminyltransferase A-like	2178	0
XM_020081328.1	lactosylceramide 1,3-N-acetyl-beta-D-glucosaminyltransferase A-like	2571	0
XM_020081329.1	microtubule-associated tumor suppressor 1 isoform X1	2235	0
XM_020081330.1	guanine nucleotide exchange factor DBS-like	7391	0
XM_020081331.1	protein phosphatase inhibitor 2	3523	2.70E-108
XM_020081332.1	ankyrin repeat domain-containing protein 13C	3742	0
XM_020081333.1	carnitine O-palmitoyltransferase 2, mitochondrial	2381	0
XM_020081334.1	basigin isoform X1	1720	0
XM_020081335.1	basigin isoform X2	1709	0
XM_020081336.1	ectoderm-neural cortex protein 1-like	3490	0
XM_020081337.1	microtubule-associated tumor suppressor 1 isoform X1	2214	0
XM_020081338.1	cytoplasmic polyadenylation element-binding protein 2 isoform X1	5759	0
XM_020081339.1	cytoplasmic polyadenylation element-binding protein 2 isoform X2	2998	0
XM_020081340.1	cytoplasmic polyadenylation element-binding protein 2 isoform X1	1937	0
XM_020081341.1	phospholipid hydroperoxide glutathione peroxidase, mitochondrial-like isoform X1	1046	3.28E-134
XM_020081342.1	phospholipid hydroperoxide glutathione peroxidase, mitochondrial-like isoform X2	1049	2.36E-139
XM_020081343.1	phospholipid hydroperoxide glutathione peroxidase, mitochondrial-like isoform X3	1044	2.01E-135
XM_020081344.1	DNA-directed RNA polymerase, mitochondrial	4781	0
XM_020081345.1	PREDICTED: uncharacterized protein KIAA1107-like isoform X1	5225	0
XM_020081346.1	PREDICTED: uncharacterized protein KIAA1107-like isoform X1	5217	0
XM_020081347.1	PREDICTED: uncharacterized protein KIAA1107-like isoform X2	5198	0
XM_020081348.1	PREDICTED: uncharacterized protein KIAA1107-like isoform X3	4426	0
XM_020081349.1	PREDICTED: calreticulin-like	2054	0
XM_020081350.1	vesicle-associated membrane protein 7	1552	2.89E-146
XM_020081351.1	lipoprotein lipase	2739	0
XM_020081352.1	putative tRNA (cytidine(32)/guanosine(34)-2'-O)-methyltransferase	3120	0
XM_020081353.1	exocyst complex component 1 isoform X1	2993	0
XM_020081354.1	exocyst complex component 1 isoform X1	2805	0
XM_020081355.1	exocyst complex component 1 isoform X1	3137	0
XM_020081356.1	exocyst complex component 1 isoform X1	2787	0
XM_020081357.1	exocyst complex component 1 isoform X1	2760	0
XM_020081358.1	exocyst complex component 1 isoform X4	2742	0
XM_020081359.1	MAP kinase-interacting serine/threonine-protein kinase 1	3390	0
XM_020081360.1	vesicle-associated membrane protein 7	1469	1.10E-146
XM_020081361.1	PREDICTED: uncharacterized protein LOC109625834	2862	0
XM_020081362.1	elongation of very long chain fatty acids protein 1-like	1235	0
XM_020081363.1	elongation of very long chain fatty acids protein 1-like	2142	0
XM_020081364.1	BEN domain-containing protein 5 isoform X2	6236	0
XM_020081365.1	BEN domain-containing protein 5 isoform X2	6182	0
XM_020081366.1	BEN domain-containing protein 5 isoform X2	6451	0
XM_020081367.1	BEN domain-containing protein 5 isoform X2	5949	6.28E-178
XM_020081368.1	zinc finger protein GLIS1 isoform X1	4231	0
XM_020081369.1	zinc finger protein GLIS1 isoform X1	4217	0
XM_020081370.1	zinc finger protein GLIS1 isoform X1	4219	0
XM_020081371.1	elongation of very long chain fatty acids protein 4-like	1795	0
XM_020081372.1	ribosome production factor 1	1641	0
XM_020081373.1	DNA-directed RNA polymerases I and III subunit RPAC2-like	742	1.46E-81
XM_020081374.1	guanine nucleotide-binding protein G(I)/G(S)/G(O) subunit gamma-5-like	692	7.29E-43
XM_020081375.1	PC4 and SFRS1-interacting protein isoform X1	3316	3.05E-91
XM_020081376.1	transmembrane emp24 domain-containing protein 1-like	2388	6.42E-162
XM_020081377.1	C3 and PZP-like alpha-2-macroglobulin domain-containing protein 8	7523	0
XM_020081378.1	transcription factor AP-1-like	1985	0
XM_020081379.1	prohibitin-2 isoform X1	1677	0
XM_020081380.1	prohibitin-2 isoform X2	1659	5.31E-180
XM_020081381.1	HLA class II histocompatibility antigen gamma chain	783	7.78E-160
XM_020081382.1	voltage-dependent calcium channel beta subunit-associated regulatory protein	6759	0
XM_020081383.1	voltage-dependent calcium channel beta subunit-associated regulatory protein	6755	0

XM_020081384.1	serine/threonine-protein kinase Nek1 isoform X1	5333	0
XM_020081385.1	serine/threonine-protein kinase Nek1 isoform X2	5245	0
XM_020081386.1	ensconsin-like isoform X3	5564	0
XM_020081387.1	DCN1-like protein 4 isoform X2	2980	0
XM_020081388.1	DCN1-like protein 4 isoform X2	4166	7.45E-161
XM_020081389.1	DCN1-like protein 4 isoform X2	3933	1.58E-161
XM_020081390.1	DCN1-like protein 4 isoform X2	4052	3.55E-161
XM_020081391.1	DCN1-like protein 4 isoform X2	2994	1.02E-164
XM_020081392.1	DCN1-like protein 4 isoform X2	3890	0
XM_020081393.1	DCN1-like protein 4 isoform X2	3759	0
XM_020081394.1	DCN1-like protein 4 isoform X2	2925	0
XM_020081395.1	extracellular serine/threonine protein kinase FAM20C-like	2507	0
XM_020081396.1	ensconsin-like isoform X3	5555	0
XM_020081397.1	UDP-N-acetylhexosamine pyrophosphorylase isoform X1	1855	0
XM_020081398.1	UDP-N-acetylhexosamine pyrophosphorylase isoform X1	1827	0
XM_020081399.1	UDP-N-acetylhexosamine pyrophosphorylase isoform X1	1813	0
XM_020081400.1	F-box/LRR-repeat protein 5	3911	0
XM_020081401.1	peptidyl-prolyl cis-trans isomerase FKBP8	2830	0
XM_020081402.1	peptidyl-prolyl cis-trans isomerase FKBP8	2899	0
XM_020081403.1	phospholipid phosphatase 6	1412	5.91E-165
XM_020081404.1	phospholipid phosphatase 6	2034	6.79E-162
XM_020081405.1	ensconsin-like isoform X3	5552	0
XM_020081406.1	E3 ubiquitin-protein ligase RNF13	2882	0
XM_020081407.1	lysophosphatidic acid receptor 3	5258	0
XM_020081408.1	prolyl 3-hydroxylase 1 isoform X1	3801	0
XM_020081409.1	putative GTP-binding protein 6	2001	0
XM_020081410.1	PI-PLC X domain-containing protein 1-like isoform X1	1320	0
XM_020081411.1	ensconsin-like isoform X4	5540	0
XM_020081412.1	cAMP-dependent protein kinase catalytic subunit beta isoform X1	1862	0
XM_020081413.1	cAMP-dependent protein kinase catalytic subunit beta isoform X2	2006	0
XM_020081414.1	cAMP-dependent protein kinase catalytic subunit beta isoform X3	1997	0
XM_020081415.1	kynurenine--oxoglutarate transaminase 3	2632	0
XM_020081416.1	zinc finger Ran-binding domain-containing protein 2 isoform X1	1123	4.66E-96
XM_020081417.1	zinc finger Ran-binding domain-containing protein 2 isoform X1	2000	1.90E-92
XM_020081418.1	N-acetylneuraminate lyase	1941	0
XM_020081419.1	N-acetylneuraminate lyase	1896	0
XM_020081420.1	ensconsin-like isoform X5	5480	0
XM_020081421.1	N-acetylneuraminate lyase	1878	0
XM_020081422.1	heparan sulfate 2-O-sulfotransferase 1-like	2581	0
XM_020081423.1	PREDICTED: calreticulin-like	2538	0
XM_020081424.1	xyloside xylosyltransferase 1	2746	0
XM_020081425.1	retinoic acid receptor RXR-gamma-B isoform X1	2068	0
XM_020081426.1	retinoic acid receptor RXR-gamma-B isoform X1	1872	0
XM_020081427.1	retinoic acid receptor RXR-gamma-B isoform X1	1880	0
XM_020081428.1	ensconsin-like isoform X6	5438	0
XM_020081429.1	ras-related protein Rab-6B isoform X1	4065	1.74E-146
XM_020081430.1	ras-related protein Rab-6B isoform X2	4044	3.01E-141
XM_020081431.1	retinoic acid-induced protein 2	3006	0
XM_020081432.1	gamma-tubulin complex component 6	3746	0
XM_020081433.1	DNA methyltransferase 1-associated protein 1	2442	0
XM_020081434.1	FAS-associated factor 1	2958	0
XM_020081435.1	mesoderm induction early response protein 3 isoform X3	3418	0
XM_020081436.1	ensconsin-like isoform X7	5414	0
XM_020081437.1	protein Hook homolog 1	2703	0
XM_020081438.1	oxygen-dependent coproporphyrinogen-III oxidase, mitochondrial	2054	0
XM_020081439.1	RGM domain family member B-like	2192	0
XM_020081440.1	deoxyhypusine hydroxylase	1425	0
XM_020081441.1	deoxyhypusine hydroxylase	1467	0
XM_020081442.1	ubiquitin-protein ligase E3A-like	3790	0
XM_020081443.1	circadian locomoter output cycles protein kaput isoform X1	4483	0
XM_020081444.1	ensconsin-like isoform X8	5363	0
XM_020081445.1	circadian locomoter output cycles protein kaput isoform X1	3754	0
XM_020081446.1	circadian locomoter output cycles protein kaput isoform X1	3536	0
XM_020081447.1	circadian locomoter output cycles protein kaput isoform X1	3378	0
XM_020081448.1	circadian locomoter output cycles protein kaput isoform X1	3456	0
XM_020081449.1	circadian locomoter output cycles protein kaput isoform X1	4409	0
XM_020081450.1	serine/threonine-protein kinase NIM1-like isoform X1	2820	0
XM_020081451.1	serine/threonine-protein kinase NIM1-like isoform X1	2524	0
XM_020081452.1	serine/threonine-protein kinase NIM1-like isoform X1	2803	0
XM_020081453.1	serine/threonine-protein kinase NIM1-like isoform X1	2788	0
XM_020081454.1	serine/threonine-protein kinase NIM1-like isoform X1	2771	0
XM_020081455.1	ensconsin-like isoform X9	5339	0
XM_020081456.1	serine/threonine-protein kinase NIM1-like isoform X1	2813	0
XM_020081457.1	DNA polymerase epsilon subunit 4	780	5.30E-63
XM_020081458.1	tumor necrosis factor alpha-induced protein 8-like protein 1	3363	2.23E-126
XM_020081459.1	vascular endothelial growth factor receptor 2 isoform X1	4935	0
XM_020081460.1	vascular endothelial growth factor receptor 2 isoform X2	4933	0
XM_020081461.1	procollagen galactosyltransferase 2-like	2694	0
XM_020081462.1	axonemal dynein light intermediate polypeptide 1-like	939	1.62E-174

XM_020081463.1	ensconsin-like isoform X10	5279	0
XM_020081464.1	leucine-rich repeat-containing protein 41	4456	0
XM_020081465.1	5'-AMP-activated protein kinase catalytic subunit alpha-2	2364	0
XM_020081466.1	sec1 family domain-containing protein 2	2376	0
XM_020081467.1	large subunit GTPase 1 homolog	2574	0
XM_020081468.1	neuronal growth regulator 1 isoform X1	4502	0
XM_020081469.1	neuronal growth regulator 1 isoform X2	4455	0
XM_020081470.1	neuronal growth regulator 1 isoform X1	1799	0
XM_020081471.1	neuronal growth regulator 1 isoform X4	1693	0
XM_020081472.1	MAP7 domain-containing protein 2-like isoform X11	5236	4.41E-175
XM_020081473.1	leucine-rich repeat-containing protein 15-like	2620	0
XM_020081474.1	MICOS complex subunit MIC13	1571	2.92E-60
XM_020081475.1	bromodomain testis-specific protein-like isoform X1	4021	0
XM_020081476.1	bromodomain testis-specific protein-like isoform X2	4019	0
XM_020081477.1	bromodomain testis-specific protein-like isoform X3	4015	0
XM_020081478.1	lipoprotein lipase	2850	0
XM_020081479.1	geranylgeranyl transferase type-2 subunit alpha	2455	0
XM_020081480.1	ensconsin-like isoform X12	2579	0
XM_020081481.1	RING finger protein 11-like	2674	5.77E-93
XM_020081482.1	protein NDNF	2862	0
XM_020081483.1	germinal-center associated nuclear protein isoform X1	2139	0
XM_020081484.1	PREDICTED: E-selectin	2518	0
XM_020081485.1	PREDICTED: L-selectin-like	1139	0
XM_020081486.1	PREDICTED: E-selectin-like	1280	0
XM_020081487.1	PREDICTED: E-selectin-like	1247	0
XM_020081488.1	14 kDa phosphohistidine phosphatase-like	935	1.14E-70
XM_020081489.1	transmembrane protein 59-like isoform X1	2873	0
XM_020081490.1	transmembrane protein 59-like isoform X1	2769	0
XM_020081491.1	four and a half LIM domains protein 1-like	4097	0
XM_020081492.1	calsenilin-like isoform X1	1098	0
XM_020081493.1	calsenilin-like isoform X1	1110	0
XM_020081494.1	calsenilin-like isoform X1	1098	0
XM_020081495.1	calsenilin-like isoform X1	1103	0
XM_020081496.1	calsenilin-like isoform X4	1164	2.01E-150
XM_020081497.1	26S proteasome non-ATPase regulatory subunit 5	2438	0
XM_020081498.1	dolichyldiphosphatase 1	2164	3.03E-148
XM_020081499.1	proteasome subunit beta type-7-like	1005	0
XM_020081500.1	zinc finger protein 703-like	3699	0
XM_020081501.1	SKI family transcriptional corepressor 1 homolog-B-like isoform X1	4265	0
XM_020081502.1	dual specificity protein kinase CLK4-like isoform X1	2479	0
XM_020081503.1	SKI family transcriptional corepressor 2	4590	0
XM_020081504.1	SKI family transcriptional corepressor 2	4428	0
XM_020081505.1	SKI family transcriptional corepressor 1 homolog-B-like isoform X1	4253	0
XM_020081506.1	torsin-1A-like isoform X1	1679	0
XM_020081507.1	amyloid beta A4 precursor protein-binding family B member 2-like isoform X1	4668	0
XM_020081508.1	torsin-1A-like isoform X2	1149	0
XM_020081509.1	small conductance calcium-activated potassium channel protein 2 isoform X1	3115	0
XM_020081510.1	small conductance calcium-activated potassium channel protein 2 isoform X2	2169	0
XM_020081511.1	small conductance calcium-activated potassium channel protein 2 isoform X3	2225	0
XM_020081512.1	small conductance calcium-activated potassium channel protein 2 isoform X4	2102	0
XM_020081513.1	dual specificity protein kinase CLK4-like isoform X1	2068	0
XM_020081514.1	small conductance calcium-activated potassium channel protein 2 isoform X5	2266	0
XM_020081515.1	small conductance calcium-activated potassium channel protein 2 isoform X6	2264	0
XM_020081516.1	small conductance calcium-activated potassium channel protein 2 isoform X5	2005	0
XM_020081517.1	claudin-10-like isoform X2	1779	1.80E-157
XM_020081518.1	proteinase-activated receptor 3-like	2337	0
XM_020081519.1	epidermal growth factor-like protein 7	1727	0
XM_020081520.1	epidermal growth factor-like protein 7	1790	0
XM_020081521.1	epidermal growth factor-like protein 7	1765	0
XM_020081522.1	dual specificity protein kinase CLK4-like isoform X2	1703	0
XM_020081523.1	epidermal growth factor-like protein 7	1761	0
XM_020081524.1	junction-mediating and -regulatory protein-like	6109	0
XM_020081525.1	peptidyl-prolyl cis-trans isomerase CWC27 homolog isoform X1	2037	0
XM_020081526.1	peptidyl-prolyl cis-trans isomerase CWC27 homolog isoform X2	2034	0
XM_020081527.1	small glutamine-rich tetratricopeptide repeat-containing protein beta	1903	0
XM_020081528.1	small glutamine-rich tetratricopeptide repeat-containing protein beta	1843	0
XM_020081529.1	small glutamine-rich tetratricopeptide repeat-containing protein beta	1874	0
XM_020081530.1	thimet oligopeptidase-like	2487	0
XM_020081531.1	BRCA1-associated protein	3042	0
XM_020081532.1	beta-1,3-galactosyl-O-glycosyl-glycoprotein beta-1,6-N-acetylglucosaminyltransferase 4	3136	0
XM_020081533.1	receptor expression-enhancing protein 5-like	965	3.01E-116
XM_020081534.1	transmembrane protein 126A-like	888	4.58E-123
XM_020081535.1	protein FAM222A-like	4169	0
XM_020081536.1	protein FAM222A-like	3970	0
XM_020081537.1	protein FAM222A-like	4279	0
XM_020081538.1	protein FAM222A-like	4167	0
XM_020081539.1	protein FAM222A-like	3896	0
XM_020081540.1	60S ribosomal protein L37	437	4.83E-59
XM_020081541.1	alpha-2,8-sialyltransferase 8E	3636	0

XM_020081542.1	creatine kinase U-type, mitochondrial-like	1436	0
XM_020081543.1	metalloprotease TIKI1	3229	0
XM_020081544.1	metalloprotease TIKI1	2962	0
XM_020081545.1	protein SDA1 homolog	2350	0
XM_020081546.1	chondroitin sulfate proteoglycan 4-like	9130	0
XM_020081547.1	ATP-binding cassette sub-family B member 7, mitochondrial	3425	0
XM_020081548.1	RUN and SH3 domain-containing protein 1 isoform X2	4857	0
XM_020081549.1	RUN and SH3 domain-containing protein 1 isoform X2	4730	0
XM_020081550.1	28S ribosomal protein S2, mitochondrial	1532	0
XM_020081551.1	immediate early response 3-interacting protein 1	1247	4.39E-33
XM_020081552.1	TNF receptor-associated factor 2-like	3774	0
XM_020081553.1	TNF receptor-associated factor 2-like	3642	0
XM_020081554.1	septin-5-like isoform X1	1486	0
XM_020081555.1	septin-5-like isoform X2	1462	0
XM_020081556.1	septin-5-like isoform X2	1451	0
XM_020081557.1	alpha-N-acetyl-neuraminyl-2,3-beta-galactosyl-1,3-N-acetyl-galactosaminide alpha-2,6-sialyltransferase-like isoform X1	2469	0
XM_020081558.1	alpha-N-acetylgalactosaminide alpha-2,6-sialyltransferase 3-like isoform X2	2661	0
XM_020081559.1	tRNA pseudouridine synthase A, mitochondrial	1675	0
XM_020081560.1	probable palmitoyltransferase ZDHHC20	1987	0
XM_020081561.1	SWI/SNF-related matrix-associated actin-dependent regulator of chromatin subfamily B member 1-like isoform X1	1206	0
XM_020081562.1	SWI/SNF-related matrix-associated actin-dependent regulator of chromatin subfamily B member 1-like isoform X1	1851	0
XM_020081563.1	E3 ubiquitin-protein ligase RNF170-like	1517	0
XM_020081564.1	E3 ubiquitin-protein ligase RNF170-like	1590	0
XM_020081565.1	ribosome biogenesis protein NSA2 homolog	1151	5.13E-177
XM_020081566.1	V-type proton ATPase 116 kDa subunit a-like isoform X1	4541	0
XM_020081567.1	V-type proton ATPase 116 kDa subunit a-like isoform X2	4520	0
XM_020081568.1	LIM domain transcription factor LMO4.1-like	2584	2.32E-115
XM_020081569.1	LIM domain transcription factor LMO4.1-like	2724	6.62E-115
XM_020081570.1	versican core protein-like	7487	0
XM_020081571.1	adenosine receptor A2b	2610	0
XM_020081572.1	uracil phosphoribosyltransferase homolog	2060	1.03E-174
XM_020081573.1	small nuclear ribonucleoprotein Sm D3	707	1.77E-78
XM_020081574.1	phosphoglycerate mutase 2	1165	0
XM_020081575.1	eukaryotic translation initiation factor 4E-binding protein 1-like	1453	2.37E-79
XM_020081576.1	bone morphogenetic protein 1-like isoform X1	4730	0
XM_020081577.1	bone morphogenetic protein 1-like isoform X2	4727	0
XM_020081578.1	bone morphogenetic protein 1-like isoform X1	4023	0
XM_020081579.1	bone morphogenetic protein 1-like isoform X4	3548	0
XM_020081580.1	probable ATP-dependent RNA helicase DDX56	1948	0
XM_020081581.1	uracil phosphoribosyltransferase homolog	1858	7.53E-176
XM_020081582.1	WD repeat-containing protein 41	2187	0
XM_020081583.1	potassium/sodium hyperpolarization-activated cyclic nucleotide-gated channel 1-like isoform X2	5508	0
XM_020081584.1	potassium/sodium hyperpolarization-activated cyclic nucleotide-gated channel 1-like isoform X2	5946	0
XM_020081585.1	torsin-1A-like isoform X2	3044	0
XM_020081586.1	betaine--homocysteine S-methyltransferase 1-like	1744	0
XM_020081587.1	seizure 6-like protein isoform X1	5936	0
XM_020081588.1	seizure 6-like protein isoform X2	5933	0
XM_020081589.1	seizure 6-like protein isoform X3	5900	0
XM_020081590.1	seizure 6-like protein isoform X4	5897	0
XM_020081591.1	MORN repeat-containing protein 3-like	843	1.45E-176
XM_020081592.1	proteinase-activated receptor 2-like	2389	0
XM_020081593.1	voltage-gated hydrogen channel 1	1334	2.63E-124
XM_020081594.1	amyloid beta A4 precursor protein-binding family B member 2-like isoform X1	4682	0
XM_020081595.1	DNA-directed RNA polymerase I subunit RPA1	5527	0
XM_020081596.1	LIM/homeobox protein Lhx5	1675	0
XM_020081597.1	arrestin domain-containing protein 3-like isoform X1	1648	0
XM_020081598.1	arrestin domain-containing protein 3-like isoform X2	1621	0
XM_020081599.1	tetratricopeptide repeat protein 33	1786	0
XM_020081600.1	tetratricopeptide repeat protein 33	1809	0
XM_020081601.1	adenylate kinase isoenzyme 1	1281	2.50E-135
XM_020081602.1	cytochrome c oxidase subunit 5B, mitochondrial-like	883	3.65E-80
XM_020081603.1	dnaJ homolog subfamily B member 5-like	2024	0
XM_020081604.1	PREDICTED: uncharacterized protein LOC109625974 isoform X1	1586	0
XM_020081605.1	PREDICTED: uncharacterized protein LOC109625974 isoform X2	1583	0
XM_020081606.1	very long-chain acyl-CoA synthetase-like	2066	0
XM_020081607.1	pentatricopeptide repeat domain-containing protein 3, mitochondrial isoform X1	2319	0
XM_020081608.1	sulfate anion transporter 1-like isoform X1	3248	0
XM_020081609.1	sulfate anion transporter 1-like isoform X1	3218	0
XM_020081610.1	zinc-binding protein A33-like isoform X1	1912	0
XM_020081611.1	zinc-binding protein A33-like isoform X2	1909	0
XM_020081612.1	zinc-binding protein A33-like isoform X1	1762	0
XM_020081613.1	zinc-binding protein A33-like isoform X2	1759	0
XM_020081614.1	rabphilin-3A isoform X1	2671	0
XM_020081615.1	group 3 secretory phospholipase A2-like	2068	0
XM_020081616.1	pentatricopeptide repeat domain-containing protein 3, mitochondrial isoform X2	2316	0
XM_020081617.1	matrix metalloproteinase-17-like	4002	0
XM_020081618.1	small G protein signaling modulator 1-like	6125	0
XM_020081619.1	ras-specific guanine nucleotide-releasing factor 2-like isoform X1	6193	0
XM_020081620.1	ras-specific guanine nucleotide-releasing factor 2-like isoform X2	6100	0

XM_020081621.1	coiled-coil domain-containing protein 80-like isoform X1	3885	0
XM_020081622.1	coiled-coil domain-containing protein 80-like isoform X1	3889	0
XM_020081623.1	coiled-coil domain-containing protein 80-like isoform X1	3840	0
XM_020081624.1	protein AMBP-like isoform X1	1216	0
XM_020081625.1	protein AMBP-like isoform X2	1213	0
XM_020081626.1	protein AMBP-like isoform X3	1201	0
XM_020081627.1	protein AMBP-like isoform X4	1198	0
XM_020081628.1	collagen alpha-1(XXVII) chain A-like	6094	0
XM_020081629.1	lactosylceramide alpha-2,3-sialyltransferase	1902	0
XM_020081630.1	NF-kappa-B inhibitor-like protein 1	2139	0
XM_020081631.1	sodium/glucose cotransporter 2-like isoform X1	2661	0
XM_020081632.1	hyaluronan and proteoglycan link protein 3	2711	0
XM_020081633.1	high affinity cAMP-specific and IBMX-insensitive 3',5'-cyclic phosphodiesterase 8A-like	6175	0
XM_020081634.1	protein C9orf69 homolog	2011	1.76E-81
XM_020081635.1	cytochrome P450 3A27-like	2838	0
XM_020081636.1	cytochrome P450 4V2	1479	0
XM_020081637.1	spindle and kinetochore-associated protein 1	1185	8.66E-151
XM_020081638.1	kinesin-like protein KIF2A	3404	0
XM_020081639.1	tyrosine-protein kinase SYK isoform X1	2567	0
XM_020081640.1	FH2 domain-containing protein 1-like	3618	0
XM_020081641.1	tyrosine-protein kinase SYK isoform X1	2348	0
XM_020081642.1	tyrosine-protein kinase SYK isoform X1	2346	0
XM_020081643.1	tyrosine-protein kinase SYK isoform X1	2504	0
XM_020081644.1	fibrinogen C domain-containing protein 1 isoform X1	5460	0
XM_020081645.1	fibrinogen C domain-containing protein 1 isoform X2	5376	0
XM_020081646.1	DTW domain-containing protein 2	1565	0
XM_020081647.1	mitotic-spindle organizing protein 2-like isoform X1	1359	1.35E-111
XM_020081648.1	mitotic-spindle organizing protein 2-like isoform X2	1224	3.31E-101
XM_020081649.1	mitotic-spindle organizing protein 2-like isoform X3	1221	1.18E-100
XM_020081650.1	FH2 domain-containing protein 1-like	3697	0
XM_020081651.1	macrophage mannose receptor 1-like	4828	0
XM_020081652.1	chondroitin sulfate synthase 3	3787	0
XM_020081653.1	matrix metalloproteinase-17-like	3668	0
XM_020081654.1	prolactin receptor isoform X1	3151	0
XM_020081655.1	prolactin receptor isoform X2	3148	0
XM_020081656.1	GRAM domain-containing protein 3 isoform X1	2397	0
XM_020081657.1	GRAM domain-containing protein 3 isoform X2	2349	0
XM_020081658.1	GRAM domain-containing protein 3 isoform X3	2280	0
XM_020081659.1	GRAM domain-containing protein 3 isoform X4	2232	0
XM_020081660.1	PREDICTED: uncharacterized protein C2orf42 homolog isoform X1	2555	0
XM_020081661.1	PREDICTED: uncharacterized protein C2orf42 homolog isoform X2	2043	0
XM_020081662.1	rho-related GTP-binding protein RhoG-like	3062	1.19E-133
XM_020081663.1	FYN-binding protein isoform X1	3047	0
XM_020081664.1	FYN-binding protein isoform X2	3032	0
XM_020081665.1	FYN-binding protein isoform X3	2966	0
XM_020081666.1	myosin light chain 4-like	1123	4.72E-116
XM_020081667.1	dual specificity protein phosphatase 26	4879	1.35E-118
XM_020081668.1	plasminogen receptor (KT)	1293	4.14E-89
XM_020081669.1	plasminogen receptor (KT)	1171	4.30E-90
XM_020081670.1	E3 ubiquitin-protein ligase RNF34-like isoform X1	3119	0
XM_020081671.1	E3 ubiquitin-protein ligase RNF34-like isoform X2	3000	0
XM_020081672.1	E3 ubiquitin-protein ligase RNF34-like isoform X3	3077	0
XM_020081673.1	RING finger protein 208	2074	9.59E-174
XM_020081674.1	glypican-6 isoform X2	5597	0
XM_020081675.1	amyloid beta A4 precursor protein-binding family B member 2-like isoform X1	4828	0
XM_020081676.1	oxysterol-binding protein 2-like	4259	0
XM_020081677.1	protein FAM163B-like	4159	2.58E-84
XM_020081678.1	calcium-binding protein 1-like isoform X2	1995	0
XM_020081679.1	calcium-binding protein 1-like isoform X2	1825	0
XM_020081680.1	calcium-binding protein 1-like isoform X3	1660	2.37E-148
XM_020081681.1	SEC14-like protein 2 isoform X1	3180	0
XM_020081682.1	SEC14-like protein 2 isoform X1	3309	0
XM_020081683.1	leucine-rich repeat transmembrane neuronal protein 2	2749	0
XM_020081684.1	glutamate receptor ionotropic, NMDA 3A-like	5553	0
XM_020081685.1	calcium homeostasis modulator protein 3-like	846	0
XM_020081686.1	protein SCAI	2762	0
XM_020081687.1	protein crumbs homolog 2-like	5288	0
XM_020081688.1	alcohol dehydrogenase class-3-like	1529	0
XM_020081689.1	nuclear receptor subfamily 5 group A member 2-like	1014	0
XM_020081690.1	leucine-rich repeat-containing protein 20	2496	0
XM_020081691.1	DNA repair protein XRCC4-like	1849	0
XM_020081692.1	PREDICTED: alpha-tectorin-like	3799	0
XM_020081693.1	beta-adrenergic receptor kinase 2	8852	0
XM_020081694.1	electrogenic sodium bicarbonate cotransporter 4-like	3513	0
XM_020081695.1	G protein-coupled receptor kinase 5-like	3243	0
XM_020081696.1	ankyrin repeat domain-containing protein 34B-like	1542	0
XM_020081697.1	hydroxycarboxylic acid receptor 1-like	571	1.08E-112
XM_020081698.1	alcohol dehydrogenase class-3	1390	0
XM_020081699.1	membrane-associated phosphatidylinositol transfer protein 3 isoform X1	6336	0

XM_020081700.1	proteinase-activated receptor 1-like	2860	0
XM_020081701.1	catenin delta-2 isoform X6	321	3.41E-59
XM_020081702.1	arylsulfatase B	2879	0
XM_020081703.1	junction-mediating and -regulatory protein-like	5197	2.02E-156
XM_020081704.1	transmembrane protein 174	327	3.77E-75
XM_020081705.1	deoxycytidine kinase	968	2.04E-160
XM_020081706.1	neuropeptide FF receptor 2-like	1323	0
XM_020081707.1	KN motif and ankyrin repeat domain-containing protein 1-like	3777	0
XM_020081708.1	rhotekin isoform X5	1990	0
XM_020081709.1	eukaryotic translation initiation factor 4E-1A isoform X1	1551	4.21E-173
XM_020081710.1	AT-rich interactive domain-containing protein 5B	1674	0
XM_020081711.1	tumor necrosis factor receptor superfamily member 10B-like	2917	0
XM_020081712.1	trafficking protein particle complex subunit 13	1422	0
XM_020081713.1	interleukin-31 receptor subunit alpha-like	2161	0
XM_020081714.1	collagen alpha-1(V) chain-like	2229	0
XM_020081715.1	protein phosphatase 1 regulatory subunit 26	3719	0
XM_020081716.1	GTPase-activating Rap/Ran-GAP domain-like protein 3	5934	0
XM_020081717.1	eukaryotic translation initiation factor 4E-1A isoform X1	1463	1.90E-146
XM_020081718.1	alpha-N-acetylgalactosaminide alpha-2,6-sialyltransferase 6	1113	0
XM_020081719.1	mitoguardin 1	4210	0
XM_020081720.1	proteoglycan 4-like	2412	0
XM_020081721.1	proprotein convertase subtilisin/kexin type 5-like	5492	0
XM_020081722.1	protein prune homolog 2-like	3198	6.79E-160
XM_020081723.1	integrin alpha-1	6606	0
XM_020081724.1	serine/threonine-protein phosphatase with EF-hands 2	1824	0
XM_020081725.1	PREDICTED: alpha-synuclein-like	679	1.62E-55
XM_020081726.1	trimeric intracellular cation channel type B-A-like	1638	9.18E-168
XM_020081727.1	H/ACA ribonucleoprotein complex subunit 1	1244	1.17E-59
XM_020081728.1	PREDICTED: uncharacterized protein LOC109626067	1053	1.02E-138
XM_020081729.1	dynamin-2 isoform X1	2115	0
XM_020081730.1	surfeit locus protein 2	712	2.59E-77
XM_020081731.1	argininosuccinate synthase	1736	0
XM_020081732.1	probable palmitoyltransferase ZDHHC8	2227	0
XM_020081733.1	netrin receptor UNC5C-like	4656	0
XM_020081734.1	PDZ and LIM domain protein 5-like	1662	0
XM_020081735.1	chloride anion exchanger-like	453	3.27E-109
XM_020081736.1	annexin A4-like	890	0
XM_020081737.1	TBC1 domain family member 10B	3131	0
XM_020081738.1	UDP-N-acetylglucosamine--peptide N-acetylglucosaminyltransferase 110 kDa subunit isoform X1	4459	0
XM_020081739.1	proline-rich receptor-like protein kinase PERK8	1080	2.34E-71
XM_020081740.1	prenylcysteine oxidase 1	3957	0
XM_020081741.1	protein FAM166B-like	861	0
XM_020081742.1	amyloid beta A4 precursor protein-binding family B member 2-like isoform X1	4665	0
XM_020081743.1	lysosome membrane protein 2-like	2639	0
XM_020081744.1	zinc finger and BTB domain-containing protein 26-like isoform X3	1434	0
XM_020081745.1	pyruvate dehydrogenase phosphatase regulatory subunit, mitochondrial	4701	0
XM_020081746.1	exonuclease mut-7 homolog	3926	0
XM_020081747.1	UDP-N-acetylglucosamine--peptide N-acetylglucosaminyltransferase 110 kDa subunit isoform X2	4441	0
XM_020081748.1	splicing factor 3A subunit 1	3633	0
XM_020081749.1	laccase domain-containing protein 1	1685	0
XM_020081750.1	ankyrin-1 isoform X3	10131	0
XM_020081751.1	nodal homolog	1194	0
XM_020081752.1	low-density lipoprotein receptor-like	3972	0
XM_020081753.1	sorting nexin-30	4858	0
XM_020081754.1	UDP-N-acetylglucosamine--peptide N-acetylglucosaminyltransferase 110 kDa subunit isoform X3	4429	0
XM_020081755.1	PREDICTED: paralemmin-2-like	813	3.88E-116
XM_020081756.1	nucleolar complex protein 4 homolog	1870	0
XM_020081757.1	unconventional myosin-Ih-like	3516	0
XM_020081758.1	density-regulated protein	890	3.47E-113
XM_020081759.1	rod cGMP-specific 3',5'-cyclic phosphodiesterase subunit beta	4127	0
XM_020081760.1	rod cGMP-specific 3',5'-cyclic phosphodiesterase subunit beta	4059	0
XM_020081761.1	syntaxin-2-like	1350	0
XM_020081762.1	UDP-N-acetylglucosamine--peptide N-acetylglucosaminyltransferase 110 kDa subunit isoform X4	4423	0
XM_020081763.1	syntaxin-2-like	1393	0
XM_020081764.1	syntaxin-2-like	1390	0
XM_020081765.1	syntaxin-2-like	1352	0
XM_020081766.1	syntaxin-2-like	1344	0
XM_020081767.1	syntaxin-2-like	1309	0
XM_020081768.1	syntaxin-2-like	1314	0
XM_020081769.1	syntaxin-2-like	1279	0
XM_020081770.1	exosome complex component RRP4	1286	0
XM_020081771.1	keratin, type I cytoskeletal 18-like	1841	0
XM_020081772.1	UDP-N-acetylglucosamine--peptide N-acetylglucosaminyltransferase 110 kDa subunit isoform X5	4494	0
XM_020081773.1	PREDICTED: centrin-3	1198	2.92E-92
XM_020081774.1	E3 ubiquitin-protein ligase Midline-1-like	2141	0
XM_020081775.1	forkhead box protein D1	1916	0
XM_020081776.1	armadillo repeat protein deleted in velo-cardio-facial syndrome homolog isoform X1	3616	0
XM_020081777.1	armadillo repeat protein deleted in velo-cardio-facial syndrome homolog isoform X2	3544	0
XM_020081778.1	glycolipid transfer protein-like	1341	4.89E-154

XM_020081779.1	alpha-methylacyl-CoA racemase	1311	0
XM_020081780.1	UDP-N-acetylglucosamine--peptide N-acetylglucosaminyltransferase 110 kDa subunit isoform X6	4404	0
XM_020081781.1	calcineurin B homologous protein 3-like	2196	3.22E-154
XM_020081782.1	M17 homologue	980	1.01E-140
XM_020081783.1	tubulin-specific chaperone A	643	8.76E-73
XM_020081784.1	gamma-glutamyltransferase 5-like	2300	0
XM_020081785.1	GTP:AMP phosphotransferase AK3, mitochondrial	1815	1.73E-161
XM_020081786.1	anthrax toxin receptor 1-like	2752	0
XM_020081787.1	ubiquitin-related modifier 1	1192	1.16E-65
XM_020081788.1	UDP-N-acetylglucosamine--peptide N-acetylglucosaminyltransferase 110 kDa subunit isoform X7	4488	0
XM_020081789.1	PR domain zinc finger protein 12	1872	0
XM_020081790.1	A disintegrin and metalloproteinase with thrombospondin motifs 6	4085	0
XM_020081791.1	A disintegrin and metalloproteinase with thrombospondin motifs 6	3977	0
XM_020081792.1	potassium channel subfamily T member 2-like	4648	0
XM_020081793.1	coiled-coil domain-containing protein 74A-like isoform X1	1837	0
XM_020081794.1	coiled-coil domain-containing protein 74A-like isoform X1	1767	0
XM_020081795.1	UDP-N-acetylglucosamine--peptide N-acetylglucosaminyltransferase 110 kDa subunit isoform X8	4458	0
XM_020081796.1	coiled-coil domain-containing protein 74A-like isoform X1	1957	0
XM_020081797.1	pentatricopeptide repeat-containing protein 2, mitochondrial isoform X1	1461	0
XM_020081798.1	pentatricopeptide repeat-containing protein 2, mitochondrial isoform X2	1097	0
XM_020081799.1	prostaglandin E2 receptor EP4 subtype-like	2543	0
XM_020081800.1	transmembrane protein 132C-like	4025	0
XM_020081801.1	bone morphogenetic protein 5	1755	8.95E-141
XM_020081802.1	protein-lysine 6-oxidase-like	2032	0
XM_020081803.1	UDP-N-acetylglucosamine--peptide N-acetylglucosaminyltransferase 110 kDa subunit isoform X9	3746	0
XM_020081804.1	Krueppel-like factor 2	2537	0
XM_020081805.1	fibroblast growth factor 10-like	2251	1.51E-122
XM_020081806.1	substance-P receptor-like	3174	0
XM_020081807.1	SOSS complex subunit C-like	684	1.83E-48
XM_020081808.1	multiple epidermal growth factor-like domains protein 10	3639	0
XM_020081809.1	beta-crystallin B1-like	893	2.72E-145
XM_020081810.1	UDP-N-acetylglucosamine--peptide N-acetylglucosaminyltransferase 110 kDa subunit isoform X9	5519	0
XM_020081811.1	homeobox protein orthopedia isoform X1	1543	5.62E-180
XM_020081812.1	homeobox protein orthopedia isoform X2	1982	3.56E-174
XM_020081813.1	homeobox protein orthopedia isoform X3	1513	6.90E-170
XM_020081814.1	homeobox protein orthopedia isoform X4	1952	2.19E-164
XM_020081815.1	5-hydroxytryptamine receptor 4-like	1965	0
XM_020081816.1	prostaglandin E synthase	1782	3.20E-101
XM_020081817.1	transmembrane protein 119-like	1431	0
XM_020081818.1	transmembrane protein 119-like	1499	0
XM_020081819.1	protein FAM69B	2160	0
XM_020081820.1	homeobox protein vent1-like	1331	1.41E-168
XM_020081821.1	dopamine beta-hydroxylase	2580	0
XM_020081822.1	amyloid beta A4 precursor protein-binding family B member 2-like isoform X1	4662	0
XM_020081823.1	toll-like receptor 1	2943	0
XM_020081824.1	beta-crystallin A4	844	1.16E-139
XM_020081825.1	melanopsin-A-like isoform X2	1978	0
XM_020081826.1	protein CutA homolog isoform X2	1483	1.62E-106
XM_020081827.1	protein CutA homolog isoform X2	1425	1.24E-90
XM_020081828.1	corticotropin-releasing factor-binding protein	1569	0
XM_020081829.1	relaxin-3 receptor 1-like	2665	0
XM_020081830.1	DNL-type zinc finger protein	743	1.65E-147
XM_020081831.1	membrane-associated transporter protein	1990	0
XM_020081832.1	CUB and sushi domain-containing protein 1-like	5743	0
XM_020081833.1	LIM/homeobox protein Lhx6	1993	0
XM_020081834.1	integrator complex subunit 10 isoform X1	2832	0
XM_020081835.1	calcium-binding protein 7	2705	1.10E-150
XM_020081836.1	cyclin-dependent kinase 9	764	1.30E-171
XM_020081837.1	retinal homeobox protein Rx	1854	0
XM_020081838.1	PREDICTED: uncharacterized protein LOC109626150	1632	0
XM_020081839.1	protein S100-A1-like	418	1.44E-58
XM_020081840.1	cytosolic purine 5'-nucleotidase	1906	0
XM_020081841.1	cytosolic purine 5'-nucleotidase	1882	0
XM_020081842.1	integrator complex subunit 10 isoform X1	2824	0
XM_020081843.1	neutrophil gelatinase-associated lipocalin	878	1.33E-121
XM_020081844.1	heat shock protein 30-like	909	1.19E-127
XM_020081845.1	zinc finger protein GLIS1 isoform X1	3606	0
XM_020081846.1	zinc finger protein GLIS1 isoform X1	3603	0
XM_020081847.1	regulator of G-protein signaling 3 isoform X1	5961	0
XM_020081848.1	regulator of G-protein signaling 3 isoform X1	6070	0
XM_020081849.1	regulator of G-protein signaling 3 isoform X3	5950	0
XM_020081850.1	regulator of G-protein signaling 3 isoform X4	3869	0
XM_020081851.1	integrator complex subunit 10 isoform X1	2826	0
XM_020081852.1	regulator of G-protein signaling 3 isoform X5	3819	0
XM_020081853.1	regulator of G-protein signaling 3 isoform X3	3817	0
XM_020081854.1	regulator of G-protein signaling 3 isoform X7	2664	1.67E-112
XM_020081855.1	regulator of G-protein signaling 3 isoform X8	4197	1.05E-98
XM_020081856.1	regulator of G-protein signaling 3 isoform X5	2602	8.25E-66
XM_020081857.1	78 kDa glucose-regulated protein	2673	0

XM_020081858.1	rab9 effector protein with kelch motifs	1602	0
XM_020081859.1	rab9 effector protein with kelch motifs	1498	0
XM_020081860.1	rab9 effector protein with kelch motifs	1563	0
XM_020081861.1	integrator complex subunit 10 isoform X1	2761	0
XM_020081862.1	rab9 effector protein with kelch motifs	1380	0
XM_020081863.1	rab9 effector protein with kelch motifs	1201	0
XM_020081864.1	P-selectin glycoprotein ligand 1	2041	2.00E-175
XM_020081865.1	glycerol-3-phosphate acyltransferase 4	1798	0
XM_020081866.1	collagen and calcium-binding EGF domain-containing protein 1 isoform X1	1462	0
XM_020081867.1	collagen and calcium-binding EGF domain-containing protein 1 isoform X2	1459	0
XM_020081868.1	integrator complex subunit 10 isoform X1	2660	0
XM_020081869.1	CD276 antigen isoform X1	1863	0
XM_020081870.1	probable global transcription activator SNF2L2 isoform X1	1905	1.20E-158
XM_020081871.1	probable global transcription activator SNF2L2 isoform X1	1721	1.53E-159
XM_020081872.1	probable global transcription activator SNF2L2 isoform X1	1804	5.70E-149
XM_020081873.1	ATP synthase subunit e, mitochondrial	322	4.45E-28
XM_020081874.1	proline-serine-threonine phosphatase-interacting protein 2	1389	0
XM_020081875.1	cytochrome b ascorbate-dependent protein 3-like	899	1.31E-140
XM_020081876.1	bone morphogenetic protein 10-like	2003	0
XM_020081877.1	poly(ADP-ribose) glycohydrolase-like isoform X2	2213	0
XM_020081878.1	poly(ADP-ribose) glycohydrolase-like isoform X2	2210	0
XM_020081879.1	neuropeptide Y receptor type 4-like	2322	0
XM_020081880.1	solute carrier family 25 member 53	1908	0
XM_020081881.1	retinol-binding protein 4	670	5.77E-135
XM_020081882.1	interleukin 1 beta like 1	1593	4.12E-179
XM_020081883.1	T-box-containing protein TBX6L-like isoform X1	1471	0
XM_020081884.1	T-box-containing protein TBX6L-like isoform X2	1432	0
XM_020081885.1	carbonic anhydrase 4-like	1772	0
XM_020081886.1	neural proliferation differentiation and control protein 1-like	672	4.97E-62
XM_020081887.1	homeobox protein MSX-2-like	933	6.58E-142
XM_020081888.1	catenin delta-1 isoform X1	4768	0
XM_020081889.1	relaxin-3-like isoform X2	944	5.55E-129
XM_020081890.1	endothelial cell-specific molecule 1	1044	1.88E-110
XM_020081891.1	solute carrier family 2, facilitated glucose transporter member 11-like	1616	0
XM_020081892.1	histone-lysine N-methyltransferase SETD1B-A-like isoform X1	7843	0
XM_020081893.1	histone-lysine N-methyltransferase SETD1B-A-like isoform X1	5984	0
XM_020081894.1	lysine-specific demethylase 2B-like isoform X1	6127	0
XM_020081895.1	lysine-specific demethylase 2B-like isoform X2	4332	0
XM_020081896.1	catenin delta-1 isoform X1	4643	0
XM_020081897.1	lysine-specific demethylase 2B-like isoform X3	4215	0
XM_020081898.1	transmembrane protein 120B isoform X1	1852	0
XM_020081899.1	transmembrane protein 120B isoform X2	1700	0
XM_020081900.1	rho-related GTP-binding protein RhoF isoform X1	1220	4.80E-168
XM_020081901.1	rho-related GTP-binding protein RhoF isoform X2	1778	3.69E-140
XM_020081902.1	MORN repeat-containing protein 3	1198	3.35E-175
XM_020081903.1	calcium release-activated calcium channel protein 1	1467	2.68E-160
XM_020081904.1	catenin delta-1 isoform X1	4750	0
XM_020081905.1	protein pop-1-like	1157	0
XM_020081906.1	P2Y purinoceptor 1-like	1122	0
XM_020081907.1	solute carrier family 12 member 2 isoform X1	5755	0
XM_020081908.1	solute carrier family 12 member 2 isoform X2	5709	0
XM_020081909.1	PREDICTED: uncharacterized protein C9orf172 homolog	3392	0
XM_020081910.1	target of rapamycin complex 2 subunit MAPKAP1	2323	0
XM_020081911.1	multivesicular body subunit 12B	3921	0
XM_020081912.1	catenin delta-1 isoform X1	4663	0
XM_020081913.1	PREDICTED: complexin-4	538	1.95E-99
XM_020081914.1	putative UDP-GlcNAc:betaGal beta-1,3-N-acetylglucosaminyltransferase LOC100288842	1698	0
XM_020081915.1	placenta-specific gene 8 protein	446	2.51E-72
XM_020081916.1	cholesterol 25-hydroxylase-like protein 2	970	0
XM_020081917.1	cortexin-1-like	180	2.18E-27
XM_020081918.1	catenin delta-1 isoform X1	4596	0
XM_020081919.1	liver-expressed antimicrobial peptide 2-like	525	1.14E-51
XM_020081920.1	protein pop-1-like	697	1.83E-144
XM_020081921.1	homeodomain-interacting protein kinase 2-like	1098	0
XM_020081922.1	rap guanine nucleotide exchange factor 1	4474	0
XM_020081923.1	catenin delta-1 isoform X1	4478	0
XM_020081924.1	polypyrimidine tract-binding protein 1 isoform X1	3767	0
XM_020081925.1	polypyrimidine tract-binding protein 1 isoform X2	1793	0
XM_020081926.1	polypyrimidine tract-binding protein 1 isoform X3	1720	0
XM_020081927.1	polypyrimidine tract-binding protein 1 isoform X2	1786	0
XM_020081928.1	netrin-G1 isoform X2	2845	0
XM_020081929.1	uridine-cytidine kinase 1	2112	0
XM_020081930.1	roquin-2 isoform X1	7628	0
XM_020081931.1	roquin-2 isoform X1	7320	0
XM_020081932.1	roquin-2 isoform X1	7327	0
XM_020081933.1	catenin delta-1 isoform X1	4603	0
XM_020081934.1	roquin-2 isoform X1	7625	0
XM_020081935.1	spermatid perinuclear RNA-binding protein isoform X1	2581	0
XM_020081936.1	spermatid perinuclear RNA-binding protein isoform X1	4706	0

XM_020081937.1	spermatid perinuclear RNA-binding protein isoform X1	2807	0
XM_020081938.1	beta-2 adrenergic receptor-like	4811	0
XM_020081939.1	spermatid perinuclear RNA-binding protein isoform X1	3148	0
XM_020081940.1	probable G-protein coupled receptor 21	3039	0
XM_020081941.1	rab GTPase-activating protein 1-like isoform X1	5154	0
XM_020081942.1	guanine nucleotide-binding protein G(q) subunit alpha-like	2629	0
XM_020081943.1	guanine nucleotide-binding protein G(q) subunit alpha-like	2700	0
XM_020081944.1	guanine nucleotide-binding protein G(q) subunit alpha-like	2424	0
XM_020081945.1	protein CDKN2AIP homolog A-like	3615	0
XM_020081946.1	guanine nucleotide-binding protein G(q) subunit alpha-like	1815	0
XM_020081947.1	protein FAM196B-like isoform X1	2550	1.65E-144
XM_020081948.1	protein FAM196B-like isoform X2	2547	6.78E-144
XM_020081949.1	BCL2/adenovirus E1B 19 kDa protein-interacting protein 3-like	1379	4.96E-126
XM_020081950.1	ribosome-releasing factor 2, mitochondrial isoform X1	2726	0
XM_020081951.1	ribosome-releasing factor 2, mitochondrial isoform X2	2708	0
XM_020081952.1	ectoderm-neural cortex protein 1 isoform X1	4518	0
XM_020081953.1	ectoderm-neural cortex protein 1 isoform X1	4715	0
XM_020081954.1	protein CDKN2AIP homolog A-like	3204	0
XM_020081955.1	beta-hexosaminidase subunit beta isoform X1	2012	0
XM_020081956.1	beta-hexosaminidase subunit beta isoform X2	2005	0
XM_020081957.1	nuclear pore complex protein Nup214 isoform X4	7984	0
XM_020081958.1	nuclear pore complex protein Nup214 isoform X4	7981	0
XM_020081959.1	nuclear pore complex protein Nup214 isoform X4	7978	0
XM_020081960.1	nuclear pore complex protein Nup214 isoform X2	6956	0
XM_020081961.1	nuclear pore complex protein Nup214 isoform X5	6485	0
XM_020081962.1	protein FAM78A isoform X1	4304	0
XM_020081963.1	protein CDKN2AIP homolog A-like	3491	0
XM_020081964.1	protein FAM78A isoform X1	4322	0
XM_020081965.1	phospholipid phosphatase 6	4125	5.56E-157
XM_020081966.1	E3 ubiquitin-protein ligase KCMF1 isoform X1	1902	0
XM_020081967.1	E3 ubiquitin-protein ligase KCMF1 isoform X1	2183	0
XM_020081968.1	Golgi phosphoprotein 3-like	3292	5.53E-168
XM_020081969.1	PREDICTED: uncharacterized protein C18orf25 homolog isoform X1	3899	0
XM_020081970.1	PREDICTED: uncharacterized protein C18orf25 homolog isoform X1	3898	0
XM_020081971.1	PREDICTED: uncharacterized protein C18orf25 homolog isoform X2	3726	0
XM_020081972.1	PREDICTED: uncharacterized protein C18orf25 homolog isoform X3	3724	0
XM_020081973.1	E3 ubiquitin-protein ligase RNF165-like isoform X1	4851	0
XM_020081974.1	E3 ubiquitin-protein ligase RNF165-like isoform X2	4848	0
XM_020081975.1	E3 ubiquitin-protein ligase RNF165-like isoform X3	4848	0
XM_020081976.1	ubiquitin-conjugating enzyme E2 A	1648	5.41E-108
XM_020081977.1	protein PRRC2B isoform X1	9106	0
XM_020081978.1	protein PRRC2B isoform X2	9103	0
XM_020081979.1	protein PRRC2B isoform X3	9103	0
XM_020081980.1	protein PRRC2B isoform X4	9103	0
XM_020081981.1	protein PRRC2B isoform X1	9081	0
XM_020081982.1	protein PRRC2B isoform X6	7062	0
XM_020081983.1	protein PRRC2B isoform X7	9100	0
XM_020081984.1	fructose-1,6-bisphosphatase 1-like	1616	0
XM_020081985.1	fructose-1,6-bisphosphatase 1-like	1614	0
XM_020081986.1	growth arrest-specific protein 1-like	1986	1.67E-156
XM_020081987.1	disabled homolog 2 isoform X1	3159	0
XM_020081988.1	disabled homolog 2 isoform X2	3156	0
XM_020081989.1	disabled homolog 2 isoform X3	2553	0
XM_020081990.1	E3 ubiquitin-protein ligase BRE1A	3820	0
XM_020081991.1	complement component C9	2746	0
XM_020081992.1	nucleoporin NUP188 homolog	5913	0
XM_020081993.1	CDK5 regulatory subunit-associated protein 2 isoform X1	9460	0
XM_020081994.1	CDK5 regulatory subunit-associated protein 2 isoform X2	9405	0
XM_020081995.1	CDK5 regulatory subunit-associated protein 2 isoform X3	9402	0
XM_020081996.1	CDK5 regulatory subunit-associated protein 2 isoform X4	9253	0
XM_020081997.1	CDK5 regulatory subunit-associated protein 2 isoform X5	9250	0
XM_020081998.1	CDK5 regulatory subunit-associated protein 2 isoform X6	9247	0
XM_020081999.1	3-hydroxy-3-methylglutaryl-coenzyme A reductase-like isoform X1	3613	0
XM_020082000.1	3-hydroxy-3-methylglutaryl-coenzyme A reductase-like isoform X1	3508	0
XM_020082001.1	3-hydroxy-3-methylglutaryl-coenzyme A reductase-like isoform X1	3677	0
XM_020082002.1	3-hydroxy-3-methylglutaryl-coenzyme A reductase-like isoform X1	3610	0
XM_020082003.1	collagen type IV alpha-3-binding protein-like isoform X1	4203	0
XM_020082004.1	transmembrane protein 109-like isoform X1	1524	4.57E-132
XM_020082005.1	collagen type IV alpha-3-binding protein-like isoform X2	4191	0
XM_020082006.1	collagen type IV alpha-3-binding protein-like isoform X3	4124	0
XM_020082007.1	ubiquitin carboxyl-terminal hydrolase 20 isoform X1	8178	0
XM_020082008.1	ubiquitin carboxyl-terminal hydrolase 20 isoform X1	7988	0
XM_020082009.1	ubiquitin carboxyl-terminal hydrolase 20 isoform X1	8157	0
XM_020082010.1	surfeit locus protein 6	1250	3.67E-149
XM_020082011.1	surfeit locus protein 1	1332	0
XM_020082012.1	60S ribosomal protein L7a	908	1.01E-171
XM_020082013.1	transmembrane protein 109-like isoform X2	1461	1.75E-117
XM_020082014.1	metal transporter CNNM3 isoform X1	10710	0
XM_020082015.1	metal transporter CNNM3 isoform X2	10777	0

XM_020082016.1	coronin-1C-like isoform X1	2287	0
XM_020082017.1	coronin-1C-like isoform X1	2283	0
XM_020082018.1	coronin-1C-like isoform X1	2632	0
XM_020082019.1	coronin-1C-like isoform X1	2287	0
XM_020082020.1	protein phosphatase Slingshot homolog 1-like	7210	0
XM_020082021.1	synaptic vesicle 2-related protein-like	2138	0
XM_020082022.1	aryl hydrocarbon receptor-like	5660	0
XM_020082023.1	heterogeneous nuclear ribonucleoprotein D0-like	1568	8.77E-156
XM_020082024.1	palmitoyltransferase ZDHHC5-like isoform X1	5795	0
XM_020082025.1	heterogeneous nuclear ribonucleoprotein D0-like	1772	8.81E-155
XM_020082026.1	heterogeneous nuclear ribonucleoprotein D0-like	1458	2.40E-156
XM_020082027.1	heterogeneous nuclear ribonucleoprotein D0-like	1768	8.52E-155
XM_020082028.1	enolase-phosphatase E1	1884	0
XM_020082029.1	transmembrane protein 150C	2334	1.46E-117
XM_020082030.1	transmembrane protein 150C	2438	3.48E-117
XM_020082031.1	transmembrane protein 150C	2247	6.93E-118
XM_020082032.1	vesicle-associated membrane protein 8-like isoform X1	932	1.31E-49
XM_020082033.1	vesicle-associated membrane protein 8-like isoform X1	724	1.17E-49
XM_020082034.1	ATP-dependent RNA helicase DDX54	2938	0
XM_020082035.1	ATP-dependent RNA helicase DDX54	2968	0
XM_020082036.1	ras-related protein Rab-4A	2307	5.66E-156
XM_020082037.1	thioredoxin-related transmembrane protein 2	1515	0
XM_020082038.1	zinc finger protein 462	12390	0
XM_020082039.1	zinc finger protein 462	11727	0
XM_020082040.1	collagen alpha-1(V) chain	7060	0
XM_020082041.1	actin, cytoplasmic 1	3473	0
XM_020082042.1	integral membrane protein DGCR2/IDD isoform X1	4196	0
XM_020082043.1	integral membrane protein DGCR2/IDD isoform X2	4194	0
XM_020082044.1	integral membrane protein DGCR2/IDD isoform X3	4163	0
XM_020082045.1	protein DGCR14	3117	0
XM_020082046.1	helicase SKI2W-like	4325	0
XM_020082047.1	tetraspanin-5-like isoform X1	6497	3.36E-180
XM_020082048.1	phospholipid phosphatase 1	2418	0
XM_020082049.1	transportin-1 isoform X2	6097	0
XM_020082050.1	transportin-1 isoform X2	5902	0
XM_020082051.1	transportin-1 isoform X2	5903	0
XM_020082052.1	MOB kinase activator 1B	1190	1.72E-163
XM_020082053.1	sarcoplasmic/endoplasmic reticulum calcium ATPase 2	5230	0
XM_020082054.1	alpha-adducin isoform X1	2909	0
XM_020082055.1	alpha-adducin isoform X2	2891	0
XM_020082056.1	alpha-adducin isoform X3	2816	0
XM_020082057.1	alpha-adducin isoform X4	2804	0
XM_020082058.1	alpha-adducin isoform X5	2798	0
XM_020082059.1	alpha-adducin isoform X6	2711	0
XM_020082060.1	bromodomain-containing protein 8-like	3959	0
XM_020082061.1	alpha-adducin isoform X7	4602	0
XM_020082062.1	alpha-adducin isoform X8	4497	0
XM_020082063.1	alpha-adducin isoform X9	2541	0
XM_020082064.1	alpha-adducin isoform X10	2519	0
XM_020082065.1	alpha-adducin isoform X10	2481	0
XM_020082066.1	cohesin subunit SA-2-like	4734	0
XM_020082067.1	alpha-adducin isoform X7	2468	0
XM_020082068.1	A-kinase anchor protein 2-like	6305	0
XM_020082069.1	thymic stromal cotransporter homolog	2354	0
XM_020082070.1	F-box/WD repeat-containing protein 5	4426	0
XM_020082071.1	TNF receptor-associated factor 2-like	1782	0
XM_020082072.1	TNF receptor-associated factor 2-like	3384	0
XM_020082073.1	ubiquinone biosynthesis protein COQ4 homolog, mitochondrial	1912	0
XM_020082074.1	stimulator of interferon genes protein isoform X1	1617	0
XM_020082075.1	microtubule-associated serine/threonine-protein kinase 4 isoform X1	10212	0
XM_020082076.1	microtubule-associated serine/threonine-protein kinase 4 isoform X2	10194	0
XM_020082077.1	microtubule-associated serine/threonine-protein kinase 4 isoform X3	10131	0
XM_020082078.1	microtubule-associated serine/threonine-protein kinase 4 isoform X4	9401	0
XM_020082079.1	microtubule-associated serine/threonine-protein kinase 4 isoform X5	10243	0
XM_020082080.1	bone morphogenetic protein receptor type-1B-like isoform X1	3763	0
XM_020082081.1	bone morphogenetic protein receptor type-1B-like isoform X1	3448	0
XM_020082082.1	bone morphogenetic protein receptor type-1B-like isoform X1	2990	0
XM_020082083.1	stimulator of interferon genes protein isoform X1	1522	0
XM_020082084.1	transcription factor RFX3	6810	0
XM_020082085.1	mothers against decapentaplegic homolog 4	4445	0
XM_020082086.1	NAD(P) transhydrogenase, mitochondrial-like	5828	0
XM_020082087.1	chemokine-like receptor 1	1343	0
XM_020082088.1	PREDICTED: uncharacterized protein LOC109626292	1361	4.65E-172
XM_020082089.1	E3 ubiquitin-protein ligase RNF185	2429	1.05E-64
XM_020082090.1	homeobox protein goosecoid-2	888	1.45E-118
XM_020082091.1	apelin receptor B-like	1656	0
XM_020082092.1	protein phosphatase PTC7 homolog	2546	0
XM_020082093.1	DNA damage-inducible transcript 4-like protein	1068	1.82E-154
XM_020082094.1	protein transport protein Sec16A isoform X1	8713	0

XM_020082095.1	protein transport protein Sec16A isoform X1	8753	0
XM_020082096.1	protein transport protein Sec16A isoform X1	8653	0
XM_020082097.1	tyrosine-protein phosphatase non-receptor type 13 isoform X1	8632	0
XM_020082098.1	tyrosine-protein phosphatase non-receptor type 13 isoform X2	8617	0
XM_020082099.1	tyrosine-protein phosphatase non-receptor type 13 isoform X3	8500	0
XM_020082100.1	transcription factor IIIB 50 kDa subunit	1656	0
XM_020082101.1	prolactin-releasing peptide receptor-like	3097	0
XM_020082102.1	prolactin-releasing peptide receptor-like	3106	0
XM_020082103.1	rab11 family-interacting protein 1-like	4056	0
XM_020082104.1	astrotactin-2 isoform X1	5957	0
XM_020082105.1	astrotactin-2 isoform X2	5945	0
XM_020082106.1	E3 ubiquitin-protein ligase TRIM32	4326	0
XM_020082107.1	chromodomain-helicase-DNA-binding protein 1	7142	0
XM_020082108.1	tyrosine-protein kinase ABL1-like isoform X1	5923	0
XM_020082109.1	tyrosine-protein kinase ABL1-like isoform X2	5652	0
XM_020082110.1	tyrosine-protein kinase ABL1-like isoform X3	2841	0
XM_020082111.1	WW domain-binding protein 1-like	6033	4.71E-149
XM_020082112.1	ADP-ribosylation factor-like protein 3	2311	1.76E-127
XM_020082113.1	HMG domain-containing protein 3 isoform X1	6239	0
XM_020082114.1	ADP-ribosylation factor-like protein 3	2272	1.24E-127
XM_020082115.1	erbin isoform X1	8028	0
XM_020082116.1	erbin isoform X2	8025	0
XM_020082117.1	erbin isoform X3	5026	0
XM_020082118.1	erbin isoform X4	7884	0
XM_020082119.1	erbin isoform X5	4934	0
XM_020082120.1	sodium-dependent phosphate transporter 1-B-like	4312	0
XM_020082121.1	sodium-dependent phosphate transporter 1-B-like	4231	0
XM_020082122.1	HMG domain-containing protein 3 isoform X1	6239	0
XM_020082123.1	transcription factor TFIIIB component B'' homolog isoform X1	9519	0
XM_020082124.1	transcription factor TFIIIB component B'' homolog isoform X2	9516	0
XM_020082125.1	E3 ubiquitin-protein ligase NEDD4-like isoform X1	4851	0
XM_020082126.1	E3 ubiquitin-protein ligase NEDD4-like isoform X2	4848	0
XM_020082127.1	E3 ubiquitin-protein ligase NEDD4-like isoform X3	4790	0
XM_020082128.1	E3 ubiquitin-protein ligase NEDD4-like isoform X4	4654	0
XM_020082129.1	E3 ubiquitin-protein ligase NEDD4-like isoform X5	4282	0
XM_020082130.1	E3 ubiquitin-protein ligase NEDD4-like isoform X6	4246	0
XM_020082131.1	E3 ubiquitin-protein ligase NEDD4-like isoform X7	4243	0
XM_020082132.1	HMG domain-containing protein 3 isoform X1	6021	0
XM_020082133.1	mothers against decapentaplegic homolog 2 isoform X1	3198	0
XM_020082134.1	mothers against decapentaplegic homolog 2 isoform X2	3186	0
XM_020082135.1	N-acetyllactosaminide beta-1,3-N-acetylglucosaminyltransferase 2-like	1435	0
XM_020082136.1	heat shock protein 30-like	985	2.57E-128
XM_020082137.1	protein strawberry notch homolog 1 isoform X1	7331	0
XM_020082138.1	protein strawberry notch homolog 1 isoform X1	7227	0
XM_020082139.1	protein strawberry notch homolog 1 isoform X1	7071	0
XM_020082140.1	protein strawberry notch homolog 1 isoform X1	7259	0
XM_020082141.1	protein strawberry notch homolog 1 isoform X1	7056	0
XM_020082142.1	HMG domain-containing protein 3 isoform X1	6021	0
XM_020082143.1	protein strawberry notch homolog 1 isoform X1	7331	0
XM_020082144.1	prostaglandin G/H synthase 1-like isoform X1	2318	0
XM_020082145.1	phosducin-like protein isoform X2	1893	5.26E-168
XM_020082146.1	phosducin-like protein isoform X2	1874	4.30E-168
XM_020082147.1	prostaglandin G/H synthase 1-like isoform X3	3559	0
XM_020082148.1	hsp90 co-chaperone Cdc37-like 1	5095	0
XM_020082149.1	peptidylprolyl isomerase domain and WD repeat-containing protein 1	2176	0
XM_020082150.1	ADP-ribosylation factor 3	3505	0
XM_020082151.1	cohesin subunit SA-2-like	4731	0
XM_020082152.1	HMG domain-containing protein 3 isoform X1	6116	0
XM_020082153.1	zinc finger FYVE domain-containing protein 16-like	9415	0
XM_020082154.1	zinc finger FYVE domain-containing protein 16-like	9120	0
XM_020082155.1	vitamin K-dependent gamma-carboxylase isoform X1	3270	0
XM_020082156.1	vitamin K-dependent gamma-carboxylase isoform X2	3259	0
XM_020082157.1	sorting nexin-24	3451	1.88E-101
XM_020082158.1	protein transport protein Sec31A isoform X1	6179	0
XM_020082159.1	protein transport protein Sec31A isoform X2	6176	0
XM_020082160.1	protein transport protein Sec31A isoform X3	6173	0
XM_020082161.1	protein transport protein Sec31A isoform X4	6158	0
XM_020082162.1	protein transport protein Sec31A isoform X5	6122	0
XM_020082163.1	protein transport protein Sec31A isoform X6	6119	0
XM_020082164.1	protein transport protein Sec31A isoform X7	6116	0
XM_020082165.1	protein transport protein Sec31A isoform X8	6077	0
XM_020082166.1	kinesin-like protein KIN-4A isoform X1	2237	0
XM_020082167.1	protein transport protein Sec31A isoform X9	5744	0
XM_020082168.1	protein transport protein Sec31A isoform X10	5741	0
XM_020082169.1	protein transport protein Sec31A isoform X11	5738	0
XM_020082170.1	protein transport protein Sec31A isoform X12	5642	0
XM_020082171.1	protein transport protein Sec31A isoform X13	6155	0
XM_020082172.1	ephrin type-A receptor 5 isoform X1	4346	0
XM_020082173.1	ephrin type-A receptor 5 isoform X2	2188	0

XM_020082174.1	nipped-B-like protein isoform X1	8875	0
XM_020082175.1	nipped-B-like protein isoform X1	8358	0
XM_020082176.1	kinesin-like protein KIN-4A isoform X2	2234	0
XM_020082177.1	nipped-B-like protein isoform X1	8246	0
XM_020082178.1	proline dehydrogenase 1, mitochondrial-like isoform X1	2729	0
XM_020082180.1	serine/arginine-rich splicing factor 9-like	899	3.58E-121
XM_020082181.1	serine/arginine-rich splicing factor 9-like	929	3.79E-121
XM_020082182.1	serine/arginine-rich splicing factor 9-like	1128	1.43E-120
XM_020082183.1	serine/arginine-rich splicing factor 9-like	902	3.64E-121
XM_020082184.1	serine/arginine-rich splicing factor 9-like	1535	9.36E-118
XM_020082185.1	kinesin-like protein KIN-4A isoform X3	2222	0
XM_020082186.1	VIP36-like protein	2933	0
XM_020082187.1	mitochondrial fission process protein 1	3442	2.65E-90
XM_020082188.1	MARVEL domain-containing protein 2-like	2666	0
XM_020082189.1	MARVEL domain-containing protein 2-like	2699	0
XM_020082190.1	MARVEL domain-containing protein 2-like	3169	0
XM_020082191.1	MARVEL domain-containing protein 2-like	3136	0
XM_020082192.1	PREDICTED: occludin-like	2274	0
XM_020082193.1	PREDICTED: occludin-like	2191	0
XM_020082194.1	golgin subfamily A member 2 isoform X1	6373	0
XM_020082195.1	golgin subfamily A member 2 isoform X2	6220	0
XM_020082196.1	disabled homolog 2-interacting protein isoform X1	5993	0
XM_020082197.1	disabled homolog 2-interacting protein isoform X2	3552	0
XM_020082198.1	disabled homolog 2-interacting protein isoform X3	3703	0
XM_020082199.1	probable UDP-sugar transporter protein SLC35A4	1283	0
XM_020082200.1	disabled homolog 2-interacting protein isoform X4	5855	0
XM_020082201.1	disabled homolog 2-interacting protein isoform X3	5758	0
XM_020082202.1	disabled homolog 2-interacting protein isoform X3	5746	0
XM_020082203.1	disabled homolog 2-interacting protein isoform X5	5465	0
XM_020082204.1	disabled homolog 2-interacting protein isoform X5	5572	0
XM_020082205.1	GPN-loop GTPase 3 isoform X1	1450	0
XM_020082206.1	GPN-loop GTPase 3 isoform X1	1445	0
XM_020082207.1	actin-related protein 2/3 complex subunit 3	832	2.00E-133
XM_020082208.1	ubiquitin-conjugating enzyme E2 G1-like	2043	1.33E-119
XM_020082209.1	tankyrase-1-like isoform X1	5995	0
XM_020082210.1	tankyrase-1-like isoform X2	3954	0
XM_020082211.1	sushi domain-containing protein 2	2875	0
XM_020082212.1	serine/threonine-protein kinase TAO3-like isoform X1	4073	0
XM_020082213.1	serine/threonine-protein kinase TAO3-like isoform X1	4133	0
XM_020082214.1	serine/threonine-protein kinase TAO3-like isoform X1	3603	0
XM_020082215.1	serine/threonine-protein kinase TAO3-like isoform X1	3652	0
XM_020082216.1	serine/threonine-protein kinase TAO3-like isoform X1	3592	0
XM_020082217.1	serine/threonine-protein kinase TAO3-like isoform X1	4070	0
XM_020082218.1	heat shock protein beta-1-like	2500	1.41E-127
XM_020082219.1	PREDICTED: uncharacterized protein KIAA2026 homolog isoform X1	8359	0
XM_020082220.1	PREDICTED: uncharacterized protein KIAA2026 homolog isoform X2	8356	0
XM_020082221.1	PREDICTED: uncharacterized protein KIAA2026 homolog isoform X1	8079	0
XM_020082222.1	melanoma antigen recognized by T-cells 1 isoform X1	717	1.22E-57
XM_020082223.1	melanoma antigen recognized by T-cells 1 isoform X2	700	5.96E-52
XM_020082224.1	myocyte-specific enhancer factor 2C-like isoform X1	5965	0
XM_020082225.1	slit homolog 3 protein-like isoform X2	6196	0
XM_020082226.1	myocyte-specific enhancer factor 2C-like isoform X2	5962	0
XM_020082227.1	netrin receptor UNC5D-like isoform X1	3454	0
XM_020082228.1	netrin receptor UNC5D-like isoform X2	3936	0
XM_020082229.1	netrin receptor UNC5D-like isoform X1	3722	0
XM_020082230.1	cip1-interacting zinc finger protein	4006	0
XM_020082231.1	UPF0184 protein C9orf16 homolog	2221	2.21E-65
XM_020082232.1	tyrosine-protein phosphatase non-receptor type 11 isoform X1	5451	0
XM_020082233.1	slit homolog 3 protein-like isoform X2	5084	0
XM_020082234.1	tyrosine-protein phosphatase non-receptor type 11 isoform X2	5433	0
XM_020082235.1	patatin-like phospholipase domain-containing protein 7	4864	0
XM_020082236.1	NMDA receptor synaptonuclear signaling and neuronal migration factor	2158	0
XM_020082237.1	phosphatidylinositol transfer protein beta isoform-like	2093	0
XM_020082238.1	phosphatidylinositol transfer protein beta isoform-like	2066	0
XM_020082239.1	RAB6A-GEF complex partner protein 1	7924	0
XM_020082240.1	slit homolog 3 protein-like isoform X2	4936	0
XM_020082241.1	phosphatidylinositol 3-kinase regulatory subunit alpha isoform X1	5301	0
XM_020082242.1	phosphatidylinositol 3-kinase regulatory subunit alpha isoform X2	4206	0
XM_020082243.1	phosphatidylinositol 3-kinase regulatory subunit alpha isoform X1	4081	0
XM_020082244.1	ubiquitin-60S ribosomal protein L40	1177	2.53E-162
XM_020082245.1	tyrosine-protein kinase SgK223	5868	0
XM_020082246.1	galactose-1-phosphate uridylyltransferase	1553	0
XM_020082247.1	sigma non-opioid intracellular receptor 1	843	6.56E-148
XM_020082248.1	probable E3 ubiquitin-protein ligase HERC1	14911	0
XM_020082249.1	protein Hook homolog 3 isoform X1	3685	0
XM_020082250.1	protein Hook homolog 3 isoform X2	3819	0
XM_020082251.1	mediator of RNA polymerase II transcription subunit 13-like isoform X1	7687	0
XM_020082252.1	mediator of RNA polymerase II transcription subunit 13-like isoform X2	7685	0
XM_020082253.1	novel immune-type receptor	1144	0

XM_020082254.1	mediator of RNA polymerase II transcription subunit 13-like isoform X3	7669	0
XM_020082255.1	mediator of RNA polymerase II transcription subunit 13-like isoform X4	7669	0
XM_020082256.1	mediator of RNA polymerase II transcription subunit 13-like isoform X5	7651	0
XM_020082257.1	mediator of RNA polymerase II transcription subunit 13-like	3803	8.87E-68
XM_020082258.1	short-chain specific acyl-CoA dehydrogenase, mitochondrial	2199	0
XM_020082259.1	PREDICTED: claudin-5-like	1303	1.22E-120
XM_020082260.1	germ cell-less protein-like 1	3608	0
XM_020082261.1	germ cell-less protein-like 1	3613	0
XM_020082262.1	protein FAM136A	1130	2.38E-96
XM_020082263.1	M-phase phosphoprotein 9	4900	0
XM_020082264.1	electrogenic sodium bicarbonate cotransporter 1-like isoform X1	5366	0
XM_020082265.1	electrogenic sodium bicarbonate cotransporter 1-like isoform X1	5362	0
XM_020082266.1	electrogenic sodium bicarbonate cotransporter 1-like isoform X2	5265	0
XM_020082267.1	adapter molecule crk-like	3900	0
XM_020082268.1	AP-3 complex subunit beta-1	4293	0
XM_020082269.1	DNA-binding protein RFXANK	1949	0
XM_020082270.1	transcription factor BTF3	1065	1.11E-101
XM_020082271.1	transcription factor BTF3	1098	2.17E-101
XM_020082272.1	transcription factor BTF3	1203	7.09E-101
XM_020082273.1	elongation factor 1-gamma isoform X1	1573	0
XM_020082274.1	DNA-directed DNA/RNA polymerase mu isoform X1	2542	0
XM_020082275.1	DNA-directed DNA/RNA polymerase mu isoform X2	2539	0
XM_020082276.1	N-acetylglucosamine-6-sulfatase isoform X1	3490	0
XM_020082277.1	CAP-Gly domain-containing linker protein 1-like isoform X1	7139	0
XM_020082278.1	CAP-Gly domain-containing linker protein 1-like isoform X1	7123	0
XM_020082279.1	CAP-Gly domain-containing linker protein 1-like isoform X1	7035	0
XM_020082280.1	tenascin-like isoform X1	7811	0
XM_020082281.1	tenascin-like isoform X2	7134	0
XM_020082282.1	sorting nexin-25	3267	0
XM_020082283.1	elongation factor 1-gamma isoform X2	1613	0
XM_020082284.1	tenascin-like isoform X3	6857	0
XM_020082285.1	tenascin-like isoform X4	6583	0
XM_020082286.1	tenascin-like isoform X2	2112	1.42E-108
XM_020082287.1	S-adenosylmethionine synthase-like	4224	0
XM_020082288.1	nucleolysin TIA-1-like isoform X1	3234	0
XM_020082289.1	nucleolysin TIA-1-like isoform X2	3231	0
XM_020082290.1	nucleolysin TIA-1-like isoform X3	3221	0
XM_020082291.1	nucleolysin TIA-1-like isoform X4	3218	0
XM_020082292.1	lamin-A-like isoform X1	2944	0
XM_020082293.1	ARF GTPase-activating protein GIT2-like isoform X1	4957	0
XM_020082294.1	ARF GTPase-activating protein GIT2-like isoform X2	4954	0
XM_020082295.1	ARF GTPase-activating protein GIT2-like isoform X3	4950	0
XM_020082296.1	ARF GTPase-activating protein GIT2-like isoform X4	4948	0
XM_020082297.1	DNA-directed RNA polymerase II subunit RPB7	1286	0
XM_020082298.1	ARF GTPase-activating protein GIT2-like isoform X5	4864	0
XM_020082299.1	nudC domain-containing protein 3	3024	0
XM_020082300.1	28S ribosomal protein S24, mitochondrial	810	3.19E-114
XM_020082301.1	formin-binding protein 1 isoform X1	5284	0
XM_020082302.1	formin-binding protein 1 isoform X2	4598	0
XM_020082303.1	formin-binding protein 1 isoform X3	2180	0
XM_020082304.1	formin-binding protein 1 isoform X4	4520	0
XM_020082305.1	formin-binding protein 1 isoform X5	4517	0
XM_020082306.1	formin-binding protein 1 isoform X6	4514	0
XM_020082307.1	formin-binding protein 1 isoform X7	4505	0
XM_020082308.1	formin-binding protein 1 isoform X8	4481	0
XM_020082309.1	formin-binding protein 1 isoform X9	2087	0
XM_020082310.1	formin-binding protein 1 isoform X10	4424	0
XM_020082311.1	DNA-directed RNA polymerase II subunit RPB7	1109	2.01E-111
XM_020082312.1	formin-binding protein 1 isoform X11	4388	0
XM_020082313.1	formin-binding protein 1 isoform X12	4373	0
XM_020082314.1	formin-binding protein 1 isoform X13	1991	0
XM_020082315.1	formin-binding protein 1 isoform X14	4358	0
XM_020082316.1	formin-binding protein 1 isoform X15	1976	0
XM_020082317.1	dolichol kinase	3884	0
XM_020082318.1	phytanoyl-CoA dioxygenase domain-containing protein 1	2123	0
XM_020082319.1	far upstream element-binding protein 3 isoform X1	3037	0
XM_020082320.1	far upstream element-binding protein 3 isoform X2	3036	0
XM_020082321.1	far upstream element-binding protein 3 isoform X3	2980	0
XM_020082322.1	breakpoint cluster region protein-like isoform X1	5556	0
XM_020082323.1	breakpoint cluster region protein-like isoform X2	5514	0
XM_020082324.1	breakpoint cluster region protein-like	2679	0
XM_020082325.1	regulator of nonsense transcripts 3B	2366	1.70E-149
XM_020082326.1	B-cell CLL/lymphoma 7 protein family member A	3204	3.12E-93
XM_020082327.1	signal peptide peptidase-like 3 isoform X1	3758	0
XM_020082328.1	signal peptide peptidase-like 3 isoform X2	1751	0
XM_020082329.1	F-BAR domain only protein 2 isoform X1	6005	0
XM_020082330.1	F-BAR domain only protein 2 isoform X2	5944	0
XM_020082331.1	F-BAR domain only protein 2 isoform X3	5920	0
XM_020082332.1	F-BAR domain only protein 2 isoform X4	5920	0

XM_020082333.1	F-BAR domain only protein 2 isoform X5	5860	0
XM_020082334.1	F-BAR domain only protein 2 isoform X6	5407	0
XM_020082335.1	F-BAR domain only protein 2 isoform X7	5323	0
XM_020082336.1	zinc finger SWIM domain-containing protein 6-like	6362	0
XM_020082337.1	nuclear pore complex protein Nup155	4306	0
XM_020082338.1	ankyrin repeat domain-containing protein SOWAHD	1935	0
XM_020082339.1	rho GTPase-activating protein 24 isoform X1	3188	0
XM_020082340.1	rho GTPase-activating protein 24 isoform X2	2768	0
XM_020082341.1	rho GTPase-activating protein 24 isoform X3	2914	0
XM_020082342.1	rho GTPase-activating protein 24 isoform X4	2720	0
XM_020082343.1	rho GTPase-activating protein 24 isoform X5	2579	0
XM_020082344.1	rho GTPase-activating protein 24 isoform X3	2804	0
XM_020082345.1	protein PRRC1	4678	0
XM_020082346.1	protein PRRC1	4798	0
XM_020082347.1	serine/threonine-protein kinase ULK1 isoform X1	6334	0
XM_020082348.1	serine/threonine-protein kinase ULK1 isoform X2	6331	0
XM_020082349.1	serine/threonine-protein kinase ULK1 isoform X3	6331	0
XM_020082350.1	ribosome-recycling factor, mitochondrial	1637	4.06E-161
XM_020082351.1	probable RNA-binding protein 18 isoform X1	2461	9.90E-143
XM_020082352.1	PREDICTED: securin-like	745	1.76E-133
XM_020082353.1	probable RNA-binding protein 18 isoform X2	2431	2.14E-141
XM_020082354.1	capon-like protein isoform X3	4523	0
XM_020082355.1	capon-like protein isoform X3	4330	0
XM_020082356.1	capon-like protein isoform X3	4122	0
XM_020082357.1	capon-like protein isoform X3	4214	0
XM_020082358.1	carboxyl-terminal PDZ ligand of neuronal nitric oxide synthase protein-like isoform X4	3745	0
XM_020082359.1	T-box transcription factor TBX3 isoform X1	3594	0
XM_020082360.1	T-box transcription factor TBX3 isoform X2	2121	0
XM_020082361.1	T-box transcription factor TBX5-A-like isoform X1	4373	0
XM_020082362.1	T-box transcription factor TBX5-A-like isoform X1	4223	0
XM_020082363.1	cardiomyopathy-associated protein 5-like isoform X1	3697	0
XM_020082364.1	60S ribosomal protein L39	557	3.19E-65
XM_020082365.1	cardiomyopathy-associated protein 5-like isoform X1	3981	0
XM_020082366.1	homer protein homolog 1 isoform X1	2942	0
XM_020082367.1	homer protein homolog 1 isoform X1	2880	0
XM_020082368.1	homer protein homolog 1 isoform X1	1952	0
XM_020082369.1	phosphatidylinositol 4-kinase alpha-like isoform X1	8474	0
XM_020082370.1	phosphatidylinositol 4-kinase alpha-like isoform X2	8468	0
XM_020082371.1	phosphatidylinositol 4-kinase alpha-like isoform X3	8381	0
XM_020082372.1	CBP80/20-dependent translation initiation factor isoform X1	5648	0
XM_020082373.1	CBP80/20-dependent translation initiation factor isoform X2	5567	0
XM_020082374.1	tyrosine-protein kinase transmembrane receptor ROR2	4191	0
XM_020082375.1	stAR-related lipid transfer protein 7, mitochondrial	3663	0
XM_020082376.1	N-alpha-acetyltransferase 25, NatB auxiliary subunit	5074	0
XM_020082377.1	CCR4-NOT transcription complex subunit 6	6565	0
XM_020082378.1	set1/Ash2 histone methyltransferase complex subunit ASH2 isoform X1	1943	0
XM_020082379.1	set1/Ash2 histone methyltransferase complex subunit ASH2 isoform X2	1925	0
XM_020082380.1	volume-regulated anion channel subunit LRRC8A	7498	0
XM_020082381.1	G-protein-signaling modulator 1 isoform X1	3913	0
XM_020082382.1	G-protein-signaling modulator 1 isoform X2	3910	0
XM_020082383.1	G-protein-signaling modulator 1 isoform X1	3687	0
XM_020082384.1	LIM/homeobox protein Lhx3 isoform X1	2171	0
XM_020082385.1	CCR4-NOT transcription complex subunit 6	1796	0
XM_020082386.1	LIM/homeobox protein Lhx3 isoform X2	2168	0
XM_020082387.1	LIM/homeobox protein Lhx3 isoform X3	2377	0
XM_020082388.1	lysosome membrane protein 2-like isoform X1	4302	0
XM_020082389.1	lysosome membrane protein 2-like isoform X2	4260	0
XM_020082390.1	mitogen-activated protein kinase 8-like isoform X1	6185	0
XM_020082391.1	transcriptional activator protein Pur-alpha-like	2616	1.14E-177
XM_020082392.1	ankycorbin isoform X1	4930	0
XM_020082393.1	ankycorbin isoform X1	4887	0
XM_020082394.1	ankycorbin isoform X1	4843	0
XM_020082395.1	thrombospondin-4-B-like isoform X2	3745	0
XM_020082396.1	urea transporter 1-like	2244	0
XM_020082397.1	urea transporter 1-like	2247	0
XM_020082398.1	platelet-derived growth factor receptor beta	5194	0
XM_020082399.1	tricarboxylate transport protein, mitochondrial-like	2806	1.46E-167
XM_020082400.1	transmembrane emp24 domain-containing protein 2 isoform X1	2501	4.13E-134
XM_020082401.1	transmembrane emp24 domain-containing protein 2 isoform X2	2476	4.57E-129
XM_020082402.1	very long-chain acyl-CoA synthetase-like	2488	0
XM_020082403.1	zinc finger MIZ domain-containing protein 2 isoform X1	4511	0
XM_020082404.1	zinc finger MIZ domain-containing protein 2 isoform X2	4487	0
XM_020082405.1	zinc finger MIZ domain-containing protein 2 isoform X3	3534	0
XM_020082406.1	notch-regulated ankyrin repeat-containing protein	4198	3.94E-70
XM_020082407.1	voltage-dependent anion-selective channel protein 2-like isoform X1	2138	0
XM_020082408.1	voltage-dependent anion-selective channel protein 2-like isoform X2	2135	0
XM_020082409.1	RNA 3'-terminal phosphate cyclase-like protein isoform X2	1238	0
XM_020082410.1	macrophage colony-stimulating factor 1 receptor isoform X1	4539	0
XM_020082411.1	RNA 3'-terminal phosphate cyclase-like protein isoform X2	1104	0
XM_020082412.1	RNA 3'-terminal phosphate cyclase-like protein isoform X2	1031	0

XM_020082413.1	annexin A3	1539	0
XM_020082414.1	nucleus accumbens-associated protein 1-like isoform X1	7699	0

XM_020082415.1	transmembrane protein 35A	1677	3.58E-80
XM_020082416.1	voltage-gated hydrogen channel 1	1594	4.66E-123
XM_020082417.1	NF-kappa-B-activating protein	1738	7.29E-87
XM_020082418.1	nucleus accumbens-associated protein 1-like isoform X1	7321	0
XM_020082419.1	nucleus accumbens-associated protein 1-like isoform X1	7107	0
XM_020082420.1	microtubule-associated protein 1B	8320	0
XM_020082421.1	nucleolar protein 14	3136	0
XM_020082422.1	macrophage colony-stimulating factor 1 receptor isoform X1	4475	0
XM_020082423.1	PREDICTED: uncharacterized protein LOC109626453 isoform X1	5525	0
XM_020082424.1	PREDICTED: uncharacterized protein LOC109626453 isoform X2	5522	0
XM_020082425.1	ribosome biogenesis protein BRX1 homolog	1547	0
XM_020082426.1	cell cycle checkpoint protein RAD1	1681	0
XM_020082427.1	UPF0729 protein C18orf32 homolog	482	5.44E-44
XM_020082428.1	UPF0729 protein C18orf32 homolog	489	6.06E-44
XM_020082429.1	titin homolog	5792	0
XM_020082430.1	macrophage colony-stimulating factor 1 receptor isoform X1	4536	0
XM_020082431.1	titin homolog	3977	0
XM_020082432.1	zinc finger protein 687 isoform X1	6298	0
XM_020082433.1	zinc finger protein 687 isoform X1	5780	0
XM_020082434.1	gastrin-releasing peptide	1003	2.17E-93
XM_020082435.1	gastrin-releasing peptide	894	6.13E-94
XM_020082436.1	surfeit locus protein 4	2058	2.65E-160
XM_020082437.1	zinc finger protein 618 isoform X1	6321	0
XM_020082438.1	zinc finger protein 618 isoform X2	6449	0
XM_020082439.1	zinc finger protein 618 isoform X3	6425	0
XM_020082440.1	zinc finger protein 618 isoform X4	6425	0
XM_020082441.1	zinc finger protein 618 isoform X5	6413	0
XM_020082442.1	zinc finger protein 618 isoform X6	6359	0
XM_020082443.1	zinc finger protein 618 isoform X7	6347	0
XM_020082444.1	septin-6 isoform X3	2065	0
XM_020082445.1	ATP-dependent RNA helicase DHX29	4597	0
XM_020082446.1	dnaJ homolog subfamily C member 21 isoform X1	2338	0
XM_020082447.1	dnaJ homolog subfamily C member 21 isoform X2	2335	0
XM_020082448.1	dnaJ homolog subfamily C member 21 isoform X3	2336	0
XM_020082449.1	5-phosphohydroxy-L-lysine phospho-lyase isoform X1	2149	0
XM_020082450.1	dual specificity protein phosphatase 4	3696	0
XM_020082451.1	5'-AMP-activated protein kinase catalytic subunit alpha-1	2644	0
XM_020082452.1	septin-6 isoform X3	2821	0
XM_020082453.1	ER degradation-enhancing alpha-mannosidase-like protein 3 isoform X1	5329	0
XM_020082454.1	ER degradation-enhancing alpha-mannosidase-like protein 3 isoform X2	5442	0
XM_020082455.1	ER degradation-enhancing alpha-mannosidase-like protein 3 isoform X3	5415	0
XM_020082456.1	WD repeat-containing protein 70 isoform X1	2771	0
XM_020082457.1	WD repeat-containing protein 70 isoform X1	2832	0
XM_020082458.1	WD repeat-containing protein 70 isoform X1	2784	0
XM_020082459.1	N-lysine methyltransferase KMT5A isoform X1	1876	0
XM_020082460.1	N-lysine methyltransferase KMT5A isoform X2	1818	0
XM_020082461.1	dymeclin isoform X1	3954	0
XM_020082462.1	septin-6 isoform X3	2064	0
XM_020082463.1	dymeclin isoform X2	3948	0
XM_020082464.1	BDNF/NT-3 growth factors receptor-like	2974	0
XM_020082465.1	heterogeneous nuclear ribonucleoprotein K-like isoform X1	3660	0
XM_020082466.1	heterogeneous nuclear ribonucleoprotein K-like isoform X1	3540	0
XM_020082467.1	heterogeneous nuclear ribonucleoprotein K-like isoform X1	3627	0
XM_020082468.1	heterogeneous nuclear ribonucleoprotein K-like isoform X1	3547	0
XM_020082469.1	calcium channel flower homolog	3893	1.04E-100
XM_020082470.1	rho guanine nucleotide exchange factor 28-like	7656	0
XM_020082471.1	ATP-dependent RNA helicase DDX55	2332	0
XM_020082472.1	translation initiation factor eIF-2B subunit alpha	954	0
XM_020082473.1	ubiquitin-conjugating enzyme E2 L3-like	1724	5.01E-94
XM_020082474.1	septin-6 isoform X3	2059	0
XM_020082475.1	2-oxoglutarate dehydrogenase-like, mitochondrial	3403	0
XM_020082476.1	2-oxoglutarate dehydrogenase-like, mitochondrial	3357	0
XM_020082477.1	prion protein	3998	1.51E-132
XM_020082478.1	AF4/FMR2 family member 1-like isoform X1	6422	0
XM_020082479.1	AF4/FMR2 family member 1-like isoform X2	3626	0
XM_020082480.1	unconventional myosin-XVIIIb isoform X1	6881	0
XM_020082481.1	unconventional myosin-XVIIIb isoform X1	6904	0
XM_020082482.1	unconventional myosin-XVIIIb isoform X1	6839	0
XM_020082483.1	unconventional myosin-XVIIIb isoform X1	6868	0
XM_020082484.1	splicing regulatory glutamine/lysine-rich protein 1 isoform X2	4190	0
XM_020082485.1	splicing regulatory glutamine/lysine-rich protein 1 isoform X2	4368	3.57E-144
XM_020082486.1	splicing regulatory glutamine/lysine-rich protein 1 isoform X2	4607	1.60E-144
XM_020082487.1	septin-6 isoform X3	2058	0
XM_020082488.1	ornithine decarboxylase antizyme 2-like	1710	1.77E-159
XM_020082489.1	derlin-2 isoform X1	1237	0
XM_020082490.1	derlin-2 isoform X2	1129	1.60E-168
XM_020082491.1	derlin-2 isoform X3	1142	2.36E-157

XM_020082492.1	derlin-2 isoform X4	1037	7.31E-131
XM_020082493.1	nuclear factor interleukin-3-regulated protein	3597	0
XM_020082494.1	allograft inflammatory factor 1-like	2541	8.16E-96
XM_020082495.1	septin-6 isoform X3	2053	0
XM_020082496.1	condensin-2 complex subunit D3	4158	0
XM_020082497.1	calcium/calmodulin-dependent protein kinase type II subunit beta isoform X1	4630	0
XM_020082498.1	calcium/calmodulin-dependent protein kinase type II subunit beta isoform X2	4630	0
XM_020082499.1	calcium/calmodulin-dependent protein kinase type II subunit beta isoform X3	4627	0
XM_020082500.1	calcium/calmodulin-dependent protein kinase type II subunit beta isoform X4	4585	0
XM_020082501.1	calcium/calmodulin-dependent protein kinase type II subunit beta isoform X5	4581	0
XM_020082502.1	calcium/calmodulin-dependent protein kinase type II subunit beta isoform X6	4515	0
XM_020082503.1	calcium/calmodulin-dependent protein kinase type II subunit beta isoform X7	4512	0
XM_020082504.1	F-box/WD repeat-containing protein 2	3094	0
XM_020082505.1	AP2-associated protein kinase 1 isoform X1	5250	0
XM_020082506.1	AP2-associated protein kinase 1 isoform X2	5068	0
XM_020082507.1	RIMS-binding protein 2-like	4849	0
XM_020082508.1	kynurenine--oxoglutarate transaminase 1	1775	0
XM_020082509.1	synaptic functional regulator FMR1	6967	0
XM_020082510.1	putative methyltransferase C9orf114 homolog	1241	0
XM_020082511.1	lipoma HMGIC fusion partner-like 2 protein	2108	1.09E-157
XM_020082512.1	lipoma HMGIC fusion partner-like 2 protein	2334	1.10E-156
XM_020082513.1	lipoma HMGIC fusion partner-like 2 protein	2092	9.30E-158
XM_020082514.1	lipoma HMGIC fusion partner-like 2 protein	2251	4.74E-157
XM_020082515.1	tyrosine-protein kinase JAK2-like	5505	0
XM_020082516.1	laminin subunit gamma-3	6648	0
XM_020082517.1	laminin subunit gamma-3	6800	0
XM_020082518.1	epigen-like isoform X2	2172	5.85E-96
XM_020082519.1	nucleoredoxin-like protein 2	2404	2.38E-107
XM_020082520.1	guanine nucleotide-binding protein G(I)/G(S)/G(O) subunit gamma-10-like	681	2.25E-32
XM_020082521.1	calcium-binding protein 39-like	4662	0
XM_020082522.1	long-chain fatty acid transport protein 1-like	3034	0
XM_020082523.1	DNA repair protein SWI5 homolog	2110	4.18E-91
XM_020082524.1	synaptic vesicle glycoprotein 2C-like	6387	0
XM_020082525.1	methylglutaconyl-CoA hydratase, mitochondrial	2209	0
XM_020082526.1	ras-GEF domain-containing family member 1B-B-like isoform X1	2519	0
XM_020082527.1	ras-GEF domain-containing family member 1B-B-like isoform X1	2039	0
XM_020082528.1	ras-GEF domain-containing family member 1B-B-like isoform X1	2008	0
XM_020082529.1	anthrax toxin receptor 2-like isoform X1	1361	0
XM_020082530.1	anthrax toxin receptor 2-like isoform X2	1265	0
XM_020082531.1	peptidyl-prolyl cis-trans isomerase-like	1247	2.20E-116
XM_020082532.1	peptidyl-prolyl cis-trans isomerase-like	997	1.15E-117
XM_020082533.1	serine protease 23-like	2948	0
XM_020082534.1	galactosylceramide sulfotransferase-like	2306	0
XM_020082535.1	zinc finger matrin-type protein 5	1180	4.70E-110
XM_020082536.1	zinc finger matrin-type protein 5	1066	1.25E-110
XM_020082537.1	zinc finger matrin-type protein 5	1131	2.70E-110
XM_020082538.1	cytochrome b-c1 complex subunit 9	570	7.90E-37
XM_020082539.1	tetratricopeptide repeat protein 28-like isoform X1	8866	0
XM_020082540.1	tetratricopeptide repeat protein 28-like isoform X2	8842	0
XM_020082541.1	nucleoporin p54 isoform X1	2116	0
XM_020082542.1	nucleoporin p54 isoform X1	2006	0
XM_020082543.1	secretory carrier-associated membrane protein 1	2841	0
XM_020082544.1	endoplasmic reticulum metallopeptidase 1	6171	0
XM_020082545.1	endoplasmic reticulum metallopeptidase 1	6135	0
XM_020082546.1	fibroblast growth factor 13 isoform X1	2468	0
XM_020082547.1	PREDICTED: cofilin-2-like	1557	7.64E-102
XM_020082548.1	1-acyl-sn-glycerol-3-phosphate acyltransferase alpha-like	3729	2.39E-178
XM_020082549.1	gamma-glutamyltranspeptidase 1-like	2604	0
XM_020082550.1	gamma-glutamyltranspeptidase 1-like	2347	0
XM_020082551.1	gamma-glutamyltranspeptidase 1-like	2354	0
XM_020082552.1	leucine-rich repeat-containing protein 75B-like	3611	0
XM_020082553.1	scavenger receptor class B member 1 isoform X1	2432	0
XM_020082554.1	scavenger receptor class B member 1 isoform X2	2426	0
XM_020082555.1	scavenger receptor class B member 1 isoform X3	2308	0
XM_020082556.1	scavenger receptor class B member 1 isoform X4	2381	0
XM_020082557.1	scavenger receptor class B member 1 isoform X5	2375	0
XM_020082558.1	ubiquitin-60S ribosomal protein L40	6726	0
XM_020082559.1	neurofilament light polypeptide-like	2247	0
XM_020082560.1	zinc finger protein ZIC 1-like	2860	0
XM_020082561.1	eosinophil peroxidase-like isoform X1	3240	0
XM_020082562.1	eosinophil peroxidase-like isoform X2	2477	0
XM_020082563.1	ATP synthase subunit alpha, mitochondrial	1887	0
XM_020082564.1	guanine nucleotide-binding protein G(z) subunit alpha	1981	0
XM_020082565.1	lipoxygenase homology domain-containing protein 1 isoform X1	8297	0
XM_020082566.1	lipoxygenase homology domain-containing protein 1 isoform X1	8077	0
XM_020082567.1	lipoxygenase homology domain-containing protein 1 isoform X1	8175	0
XM_020082568.1	lipoxygenase homology domain-containing protein 1 isoform X1	7926	0
XM_020082569.1	lipoxygenase homology domain-containing protein 1 isoform X1	5707	0
XM_020082570.1	inositol polyphosphate 5-phosphatase K-like	3628	0

XM_020082571.1	smoothelin-like protein 1	1532	9.44E-165
XM_020082572.1	zinc finger protein ZIC 3	3962	0
XM_020082573.1	pyruvate dehydrogenase phosphatase regulatory subunit, mitochondrial	3370	0
XM_020082574.1	solute carrier family 35 member E4 isoform X1	4241	0
XM_020082575.1	solute carrier family 35 member E4 isoform X2	4195	0
XM_020082576.1	solute carrier family 35 member E4 isoform X1	4113	0
XM_020082577.1	protein JBTS17	10251	0
XM_020082578.1	BMP/retinoic acid-inducible neural-specific protein 1	6503	0
XM_020082579.1	BMP/retinoic acid-inducible neural-specific protein 1	6566	0
XM_020082580.1	low choriolytic enzyme-like	1089	0
XM_020082581.1	zinc finger protein ZIC 3	3826	0
XM_020082582.1	zinc finger CCHC domain-containing protein 9 isoform X2	1095	0
XM_020082583.1	zinc finger CCHC domain-containing protein 9 isoform X2	1007	3.04E-175
XM_020082584.1	zinc finger CCHC domain-containing protein 9 isoform X2	1086	2.46E-175
XM_020082585.1	multiple C2 and transmembrane domain-containing protein 1-like isoform X1	4728	0
XM_020082586.1	multiple C2 and transmembrane domain-containing protein 1-like isoform X2	4689	0
XM_020082587.1	multiple C2 and transmembrane domain-containing protein 1-like isoform X3	4686	0
XM_020082588.1	mothers against decapentaplegic homolog 7 isoform X1	2499	0
XM_020082589.1	mothers against decapentaplegic homolog 7 isoform X1	939	5.67E-138
XM_020082590.1	RILP-like protein 1 isoform X1	4109	0
XM_020082591.1	RILP-like protein 1 isoform X2	4197	0
XM_020082592.1	RILP-like protein 1 isoform X2	1630	0
XM_020082593.1	RILP-like protein 1 isoform X4	4058	0
XM_020082594.1	RILP-like protein 1 isoform X5	3884	0
XM_020082595.1	SH2 domain-containing protein 3C-like isoform X1	4709	0
XM_020082596.1	SH2 domain-containing protein 3C-like isoform X2	4628	0
XM_020082597.1	SH2 domain-containing protein 3C-like isoform X3	4337	0
XM_020082598.1	endothelial differentiation-related factor 1	1160	6.51E-103
XM_020082599.1	protein lin-54 homolog isoform X1	3817	0
XM_020082600.1	protein lin-54 homolog isoform X2	3790	0
XM_020082601.1	kelch-like protein 8 isoform X1	1968	0
XM_020082602.1	kelch-like protein 8 isoform X1	3517	0
XM_020082603.1	kelch-like protein 8 isoform X1	3469	0
XM_020082604.1	phospholipid phosphatase-related protein type 1 isoform X1	1553	0
XM_020082605.1	phospholipid phosphatase-related protein type 1 isoform X2	2657	0
XM_020082606.1	phospholipid phosphatase-related protein type 1 isoform X3	2844	0
XM_020082607.1	katanin p60 ATPase-containing subunit A-like 2 isoform X1	1870	0
XM_020082608.1	katanin p60 ATPase-containing subunit A-like 2 isoform X1	1761	0
XM_020082609.1	katanin p60 ATPase-containing subunit A-like 2 isoform X1	1651	0
XM_020082610.1	katanin p60 ATPase-containing subunit A-like 2 isoform X1	1603	0
XM_020082611.1	haloacid dehalogenase-like hydrolase domain-containing protein 2	1015	0
XM_020082612.1	mitogen-activated protein kinase 9-like	3468	0
XM_020082613.1	UV excision repair protein RAD23 homolog A-like	1728	0
XM_020082614.1	inactive carboxypeptidase-like protein X2	3812	0
XM_020082615.1	embryonic polyadenylate-binding protein A-like isoform X2	2164	3.61E-135
XM_020082616.1	embryonic polyadenylate-binding protein A-like isoform X2	2164	2.81E-134
XM_020082617.1	60S ribosomal protein L28	554	4.49E-78
XM_020082618.1	60S ribosomal protein L28	554	4.49E-78
XM_020082619.1	sorting nexin-18-like	4287	0
XM_020082620.1	sorting nexin-18-like	4268	0
XM_020082621.1	tubulin polyglutamylase TTLL11	3985	0
XM_020082622.1	calmodulin-regulated spectrin-associated protein 1-B-like isoform X1	6320	0
XM_020082623.1	calmodulin-regulated spectrin-associated protein 1-B-like isoform X2	4980	0
XM_020082624.1	rho GTPase-activating protein 24-like	1852	0
XM_020082625.1	serine palmitoyltransferase 1	2328	0
XM_020082626.1	noelin isoform X1	4113	0
XM_020082627.1	noelin isoform X2	3970	0
XM_020082628.1	calcium/calmodulin-dependent protein kinase kinase 2-like	4493	0
XM_020082629.1	cyclin-dependent kinase 2-associated protein 1 isoform X1	896	1.56E-82
XM_020082630.1	cyclin-dependent kinase 2-associated protein 1 isoform X2	930	6.61E-72
XM_020082631.1	coiled-coil domain-containing protein 62	2058	0
XM_020082632.1	fibroblast growth factor 13 isoform X2	2682	6.17E-169
XM_020082633.1	huntingtin-interacting protein 1-related protein-like	3260	0
XM_020082634.1	VIP36-like protein	1982	0
XM_020082635.1	folylpolyglutamate synthase, mitochondrial	2900	0
XM_020082636.1	probable inactive glycosyltransferase 25 family member 3	2930	0
XM_020082637.1	mothers against decapentaplegic homolog 5	3035	0
XM_020082638.1	chondroitin sulfate N-acetylgalactosaminyltransferase 2	4715	0
XM_020082639.1	COP9 signalosome complex subunit 4 isoform X1	1826	0
XM_020082640.1	COP9 signalosome complex subunit 4 isoform X2	1808	0
XM_020082641.1	MORN repeat-containing protein 5 isoform X1	900	2.31E-136
XM_020082642.1	MORN repeat-containing protein 5 isoform X2	813	1.29E-108
XM_020082643.1	NADH dehydrogenase [ubiquinone] 1 alpha subcomplex subunit 8	1076	6.22E-127
XM_020082644.1	mothers against decapentaplegic homolog 5	3534	0
XM_020082645.1	proteinase-activated receptor 2-like	1853	0
XM_020082646.1	progestin and adipoQ receptor family member 3-like	2985	0
XM_020082647.1	progestin and adipoQ receptor family member 3-like	3069	0
XM_020082648.1	progestin and adipoQ receptor family member 3-like	3121	0
XM_020082649.1	developmentally-regulated GTP-binding protein 2	1557	0

XM_020082650.1	mitotic checkpoint protein BUB3 isoform X1	1317	0
XM_020082651.1	mothers against decapentaplegic homolog 5	3336	0
XM_020082652.1	mitotic checkpoint protein BUB3 isoform X1	1339	0
XM_020082653.1	acyl-CoA desaturase-like	2193	0
XM_020082654.1	brain-specific angiogenesis inhibitor 1-associated protein 2-like protein 1	3650	0
XM_020082655.1	cytochrome P450 26A1	2025	0
XM_020082656.1	actin filament-associated protein 1-like 2	3428	0
XM_020082657.1	kelch repeat and BTB domain-containing protein 13-like	1599	0
XM_020082658.1	C-terminal-binding protein 2-like	2105	4.22E-48
XM_020082659.1	mothers against decapentaplegic homolog 5	2978	0
XM_020082660.1	delta-1-pyrroline-5-carboxylate synthase-like isoform X1	2926	0

XM_020082661.1	delta-1-pyrroline-5-carboxylate synthase-like isoform X1	2751	0
XM_020082662.1	delta-1-pyrroline-5-carboxylate synthase-like isoform X1	2756	0
XM_020082663.1	delta-1-pyrroline-5-carboxylate synthase-like isoform X1	2920	0
XM_020082664.1	inactive carboxypeptidase-like protein X2	3223	0
XM_020082665.1	carbohydrate sulfotransferase 15 isoform X1	2247	0
XM_020082666.1	carbohydrate sulfotransferase 15 isoform X2	1914	0
XM_020082667.1	tudor domain-containing protein 1	4034	0
XM_020082668.1	growth hormone-inducible transmembrane protein	1654	0
XM_020082669.1	growth hormone-inducible transmembrane protein	1663	0
XM_020082670.1	growth hormone-inducible transmembrane protein	1564	0
XM_020082671.1	desumoylating isopeptidase 1-like	2097	3.04E-113
XM_020082672.1	PDZ and LIM domain protein 7 isoform X1	2484	0
XM_020082673.1	X-ray repair cross-complementing protein 6	2248	0
XM_020082674.1	X-ray repair cross-complementing protein 6	2245	0
XM_020082675.1	V-type proton ATPase 116 kDa subunit a-like isoform X2	2997	0
XM_020082676.1	V-type proton ATPase 116 kDa subunit a-like isoform X2	2904	0
XM_020082677.1	urotensin-2 receptor-like isoform X2	4766	0
XM_020082678.1	urotensin-2 receptor-like isoform X2	2387	0
XM_020082679.1	urotensin-2 receptor-like isoform X2	4712	0
XM_020082680.1	urotensin-2 receptor-like isoform X2	4826	0
XM_020082681.1	PDZ and LIM domain protein 7 isoform X1	2427	0
XM_020082682.1	urotensin-2 receptor-like isoform X2	4617	0
XM_020082683.1	growth arrest-specific protein 7-like	3004	0
XM_020082684.1	neurogenic differentiation factor 2-like	1858	0
XM_020082685.1	protein phosphatase 1 regulatory subunit 1B-like	1482	4.37E-101
XM_020082686.1	heparan sulfate glucosamine 3-O-sulfotransferase 6-like	2498	0
XM_020082687.1	germ cell-specific gene 1-like protein	1036	0
XM_020082688.1	transcription factor MafF-like	1779	1.50E-122
XM_020082689.1	transcription factor MafF-like	2667	3.06E-97
XM_020082690.1	inactive rhomboid protein 1-like isoform X1	3829	0
XM_020082691.1	inactive rhomboid protein 1-like isoform X2	3709	0
XM_020082692.1	inactive rhomboid protein 1-like isoform X3	3682	0
XM_020082693.1	protein FAM199X	2252	0
XM_020082694.1	inactive rhomboid protein 1-like isoform X4	3782	0
XM_020082695.1	PREDICTED: alpha-N-acetylglucosaminidase	4124	0
XM_020082696.1	polycystic kidney disease protein 1-like 2	12557	0
XM_020082697.1	SH3 and cysteine-rich domain-containing protein 2-like	3924	0
XM_020082698.1	SH3 and cysteine-rich domain-containing protein 2-like	4004	0
XM_020082699.1	PREDICTED: formin-2	7367	0
XM_020082700.1	telomere length regulation protein TEL2 homolog isoform X1	3577	0
XM_020082701.1	telomere length regulation protein TEL2 homolog isoform X1	3451	0
XM_020082702.1	telomere length regulation protein TEL2 homolog isoform X1	3381	0
XM_020082703.1	telomere length regulation protein TEL2 homolog isoform X1	3567	0
XM_020082704.1	telomere length regulation protein TEL2 homolog isoform X1	3371	0
XM_020082705.1	leucine-rich repeat-containing protein 4B-like	6593	0
XM_020082706.1	COMM domain-containing protein 5-like	1412	1.82E-147
XM_020082707.1	GTP-binding protein SAR1b	1068	2.92E-144
XM_020082708.1	short/branched chain specific acyl-CoA dehydrogenase, mitochondrial	1955	0
XM_020082709.1	A disintegrin and metalloproteinase with thrombospondin motifs 14	4578	0
XM_020082710.1	PREDICTED: uncharacterized protein LOC109626618 isoform X1	1954	0
XM_020082711.1	PREDICTED: uncharacterized protein LOC109626618 isoform X2	1947	0
XM_020082712.1	ras-related protein Rab-26-like	909	1.42E-91
XM_020082713.1	fibroblast growth factor 13 isoform X3	2300	1.31E-141
XM_020082714.1	probable tRNA pseudouridine synthase 1 isoform X1	1052	0
XM_020082715.1	probable tRNA pseudouridine synthase 1 isoform X2	1051	0
XM_020082716.1	probable tRNA pseudouridine synthase 1 isoform X3	1031	2.30E-174
XM_020082717.1	GTPase IMAP family member 1-like	2141	0
XM_020082718.1	sodium/hydrogen exchanger 6	4868	0
XM_020082719.1	homeobox protein DLX-3	1630	0
XM_020082720.1	extracellular serine/threonine protein kinase FAM20C-like	2791	0
XM_020082721.1	pro-neuregulin-3, membrane-bound isoform-like	3120	0
XM_020082722.1	GDNF family receptor alpha-1	3112	0
XM_020082723.1	NHL repeat-containing protein 3	3721	0
XM_020082724.1	DNA damage-inducible transcript 4 protein	1493	4.78E-120
XM_020082725.1	DNA damage-inducible transcript 4 protein	1453	3.72E-120
XM_020082726.1	6-pyruvoyl tetrahydrobiopterin synthase	1464	8.04E-105
XM_020082727.1	ankyrin repeat and SAM domain-containing protein 3 isoform X1	3748	0
XM_020082728.1	ankyrin repeat and SAM domain-containing protein 3 isoform X1	3755	0

XM_020082729.1	ankyrin repeat and SAM domain-containing protein 3 isoform X1	3741	0
XM_020082730.1	transcription initiation factor TFIID subunit 7-like	1639	0
XM_020082731.1	testican-2-like isoform X1	3686	0
XM_020082732.1	testican-2-like isoform X1	3786	0
XM_020082733.1	phosphatidylethanolamine N-methyltransferase	2419	2.15E-165
XM_020082734.1	myocardin isoform X1	4657	0
XM_020082735.1	myocardin isoform X2	4705	0
XM_020082736.1	myocardin isoform X3	4627	0
XM_020082737.1	myocardin isoform X4	4579	0
XM_020082738.1	leucine carboxyl methyltransferase 1	1946	0
XM_020082739.1	NHP2-like protein 1	663	8.82E-79
XM_020082740.1	potassium voltage-gated channel subfamily H member 4-like	5309	0
XM_020082741.1	lipase maturation factor 1	2033	0
XM_020082742.1	protein FAM13B isoform X1	7793	0
XM_020082743.1	ubiquitin carboxyl-terminal hydrolase 31-like	6481	0
XM_020082744.1	potassium voltage-gated channel subfamily A member 7-like	2653	0
XM_020082745.1	potassium voltage-gated channel subfamily A member 7-like	2603	0
XM_020082746.1	potassium voltage-gated channel subfamily A member 7-like	2618	0
XM_020082747.1	heparan sulfate glucosamine 3-O-sulfotransferase 6-like	2392	0
XM_020082748.1	dexamethasone-induced Ras-related protein 1-like	2411	0
XM_020082749.1	melanopsin-A-like isoform X1	3592	0
XM_020082750.1	melanopsin-A-like isoform X2	1711	0
XM_020082751.1	protein FAM13B isoform X1	7788	0
XM_020082752.1	homeobox protein SIX3	1646	0
XM_020082753.1	G-protein coupled receptor 26-like	1504	0
XM_020082754.1	PREDICTED: uncharacterized protein LOC109626648	2740	0
XM_020082755.1	PREDICTED: uncharacterized protein LOC109626648	2603	0
XM_020082756.1	PREDICTED: uncharacterized protein LOC109626648	2687	0
XM_020082757.1	protein FRA10AC1	1214	6.12E-175
XM_020082758.1	protein FAM13B isoform X1	7517	0
XM_020082759.1	protein shisa-6 homolog isoform X1	4290	0
XM_020082760.1	protein shisa-6 homolog isoform X2	4287	0
XM_020082761.1	protein shisa-6 homolog isoform X3	4269	0
XM_020082762.1	protein shisa-6 homolog isoform X4	4251	0
XM_020082763.1	protein shisa-6 homolog isoform X5	4230	0
XM_020082764.1	protein shisa-6 homolog isoform X6	4137	0
XM_020082765.1	protein shisa-6 homolog isoform X7	4116	0
XM_020082766.1	actin-binding LIM protein 1-like isoform X1	2299	0
XM_020082767.1	actin-binding LIM protein 1-like isoform X2	2283	0
XM_020082768.1	protein FAM13B isoform X1	7790	0
XM_020082769.1	actin-binding LIM protein 1-like isoform X3	2235	0
XM_020082770.1	actin-binding LIM protein 1-like isoform X4	2208	0
XM_020082771.1	F-box/LRR-repeat protein 15	2180	0
XM_020082772.1	beta-1 adrenergic receptor	2129	0
XM_020082773.1	MSS51 homolog, mitochondrial	1708	0
XM_020082774.1	myeloid-associated differentiation marker-like protein 2	1138	6.19E-159
XM_020082775.1	protein kinase C and casein kinase substrate in neurons protein 2-like	2138	0
XM_020082776.1	protein FAM13B isoform X1	7748	0
XM_020082777.1	protein kinase C and casein kinase substrate in neurons protein 2-like	2115	0
XM_020082778.1	protein kinase C and casein kinase substrate in neurons protein 2-like	1953	0
XM_020082779.1	protein kinase C and casein kinase substrate in neurons protein 2-like	1910	0
XM_020082780.1	circularly permutated Ras protein 1-like isoform X1	3599	0
XM_020082781.1	circularly permutated Ras protein 1-like isoform X1	3604	0
XM_020082782.1	circularly permutated Ras protein 1-like isoform X1	3597	0
XM_020082783.1	MSS51 homolog, mitochondrial	2511	0
XM_020082784.1	protein FAM13B isoform X1	7721	0
XM_020082785.1	E3 SUMO-protein ligase EGR2	2165	0
XM_020082786.1	chromobox protein homolog 7-like	4192	8.81E-138
XM_020082787.1	arf-GAP with dual PH domain-containing protein 1-like	2371	0
XM_020082788.1	V-set and transmembrane domain-containing protein 4-like	3923	0
XM_020082789.1	forkhead box protein J1-B-like	3438	0
XM_020082790.1	tumor necrosis factor receptor superfamily member 12A	2153	8.16E-56
XM_020082791.1	mucin-5AC-like isoform X1	4481	0
XM_020082792.1	protein FAM13B isoform X5	7718	0
XM_020082793.1	mucin-5AC-like isoform X2	4478	0
XM_020082794.1	dehydrogenase/reductase SDR family member 7C-B-like	1696	0
XM_020082795.1	adhesion G protein-coupled receptor A1-like	2741	0
XM_020082796.1	fibroblast growth factor 13 isoform X4	2005	3.67E-138
XM_020082797.1	coiled-coil domain-containing protein 57 isoform X1	2171	0
XM_020082798.1	coiled-coil domain-containing protein 57 isoform X2	1758	0
XM_020082799.1	choline O-acetyltransferase-like	4026	0
XM_020082800.1	myb-like protein I	1703	2.06E-161
XM_020082801.1	protein FAM13B isoform X1	7607	0
XM_020082802.1	ras-related C3 botulinum toxin substrate 1-like	3155	2.06E-68
XM_020082803.1	fatty acid synthase-like	3247	0
XM_020082804.1	rab3 GTPase-activating protein catalytic subunit	3274	0
XM_020082805.1	myosin XVB	10478	0
XM_020082806.1	ectonucleotide pyrophosphatase/phosphodiesterase family member 7-like	1416	0
XM_020082807.1	ubiquitin-conjugating enzyme E2 D4-like	563	2.68E-87

XM_020082808.1	lymphocyte antigen 75-like	995	0
XM_020082809.1	clathrin light chain B isoform X1	1899	7.10E-128
XM_020082810.1	golgin subfamily A member 4-like	806	9.02E-150
XM_020082811.1	methionine synthase	4746	0
XM_020082812.1	muscarinic acetylcholine receptor M3-like	1803	0
XM_020082813.1	PREDICTED: uncharacterized protein C10orf107 homolog	479	1.21E-105
XM_020082814.1	rho GTPase-activating protein 23-like	6237	0
XM_020082815.1	PREDICTED: caskin-2-like	1545	0
XM_020082816.1	dynein beta chain, ciliary-like	862	0
XM_020082817.1	clathrin light chain B isoform X2	1876	6.00E-122
XM_020082818.1	dual specificity mitogen-activated protein kinase kinase 4-like	5038	0
XM_020082819.1	glucagon-like peptide 2 receptor	1596	0
XM_020082820.1	PREDICTED: uncharacterized protein LOC109626689	8545	0
XM_020082821.1	cerebellar degeneration-related protein 2-like	5183	6.76E-153
XM_020082822.1	PREDICTED: uncharacterized protein LOC109626691	826	0
XM_020082823.1	RING finger protein unkempt homolog	3824	0
XM_020082824.1	prolyl 3-hydroxylase 1 isoform X1	996	0
XM_020082825.1	clathrin light chain B isoform X3	1843	1.73E-113
XM_020082826.1	voltage-dependent T-type calcium channel subunit alpha-1G-like	2221	0
XM_020082827.1	COP9 signalosome complex subunit 3	1005	0
XM_020082828.1	FERM and PDZ domain-containing protein 2	3453	0
XM_020082829.1	retinol-binding protein 3	2772	0
XM_020082830.1	poly(A) polymerase alpha-like	917	3.34E-146
XM_020082831.1	poly(A) polymerase alpha-like	4025	0
XM_020082832.1	clathrin light chain B isoform X4	2357	1.22E-105
XM_020082833.1	trinucleotide repeat-containing gene 6B protein-like	3698	0
XM_020082834.1	sorting nexin-29	6087	0
XM_020082835.1	disheveled-associated activator of morphogenesis 1-like isoform X2	4975	0
XM_020082836.1	SUN domain-containing protein 2	1128	0
XM_020082837.1	cytohesin-1 isoform X1	2678	0
XM_020082838.1	BAH and coiled-coil domain-containing protein 1	6847	0
XM_020082839.1	gap junction beta-3 protein-like	711	5.80E-178
XM_020082840.1	PREDICTED: uncharacterized protein C7orf50 homolog	682	1.33E-77
XM_020082841.1	coiled-coil domain-containing protein 78	804	0
XM_020082842.1	HIG1 domain family member 2A, mitochondrial	1245	2.61E-68
XM_020082843.1	STIP1 homology and U box-containing protein 1-like	1221	0
XM_020082844.1	PREDICTED: uncharacterized protein LOC109626711	5666	0
XM_020082845.1	myeloid-associated differentiation marker-like protein 2	897	0
XM_020082846.1	testicular acid phosphatase homolog	1305	0
XM_020082847.1	stonustoxin subunit beta-like	720	3.85E-165
XM_020082848.1	liprin-alpha-3-like isoform X1	4429	0
XM_020082849.1	fibroblast growth factor 21	609	3.77E-131
XM_020082850.1	PREDICTED: neurotrypsin-like	5212	0
XM_020082851.1	GTPase IMAP family member 8-like	1821	0
XM_020082852.1	hypoxia-inducible factor 1-alpha-like	663	3.43E-150
XM_020082853.1	phospholipid-transporting ATPase IA isoform X1	6274	0
XM_020082854.1	homeodomain-interacting protein kinase 2-like	466	1.57E-89
XM_020082855.1	sphingomyelin synthase-related protein 1	2517	3.58E-161
XM_020082856.1	myosin light chain kinase, smooth muscle-like	779	7.04E-170
XM_020082857.1	FERM domain-containing protein 6-like	1814	0
XM_020082858.1	mitotic spindle-associated MMXD complex subunit MIP18-like	321	3.03E-60
XM_020082859.1	breast cancer type 1 susceptibility protein	4650	0
XM_020082860.1	hyaluronan-binding protein 2	877	4.91E-178
XM_020082861.1	phospholipid-transporting ATPase IA isoform X2	6275	0
XM_020082862.1	pleckstrin homology domain-containing family S member 1	1503	0
XM_020082863.1	attractin-like protein 1	6864	0
XM_020082864.1	rod cGMP-specific 3',5'-cyclic phosphodiesterase subunit beta	2556	0
XM_020082865.1	protocadherin Fat 1 isoform X4	5896	0
XM_020082866.1	low-density lipoprotein receptor-related protein 2-like	8490	0
XM_020082867.1	transmembrane protein 94 isoform X1	6437	0
XM_020082868.1	transmembrane protein 94 isoform X2	6425	0
XM_020082869.1	phospholipid-transporting ATPase IA isoform X3	6267	0
XM_020082870.1	transmembrane protein 94 isoform X3	6380	0
XM_020082871.1	zinc finger protein 12-like isoform X1	3144	0
XM_020082872.1	zinc finger protein 595-like isoform X2	3114	0
XM_020082873.1	1-phosphatidylinositol 4,5-bisphosphate phosphodiesterase delta-3-A-like	2835	0
XM_020082874.1	MBT domain-containing protein 1 isoform X1	4435	0
XM_020082875.1	MBT domain-containing protein 1 isoform X2	4280	0
XM_020082876.1	tudor and KH domain-containing protein isoform X2	2668	0
XM_020082877.1	tudor and KH domain-containing protein isoform X2	2289	0
XM_020082878.1	phospholipid-transporting ATPase IA isoform X4	6267	0
XM_020082879.1	tudor and KH domain-containing protein isoform X2	2311	0
XM_020082880.1	tudor and KH domain-containing protein isoform X2	2316	0
XM_020082881.1	tudor and KH domain-containing protein isoform X2	2352	0
XM_020082882.1	crossover junction endonuclease EME1	2435	0
XM_020082883.1	crossover junction endonuclease EME1	2400	0
XM_020082884.1	zinc finger protein 569-like	3816	0
XM_020082885.1	acyl-CoA-binding domain-containing protein 5 isoform X1	2524	0
XM_020082886.1	Na(+)/H(+) exchange regulatory cofactor NHE-RF1	1550	0

XM_020082887.1	phospholipid-transporting ATPase IA isoform X5	6228	0
XM_020082888.1	carbonic anhydrase-related protein 10	2499	0
XM_020082889.1	ras-related protein Rab-37-like	3453	3.41E-161
XM_020082890.1	PREDICTED: uncharacterized protein LOC109626745	1302	1.18E-144
XM_020082891.1	nucleoside diphosphate kinase A-like isoform X1	875	7.28E-111
XM_020082892.1	39S ribosomal protein L27, mitochondrial	763	2.25E-105
XM_020082893.1	39S ribosomal protein L27, mitochondrial	721	1.32E-105
XM_020082894.1	phospholipid-transporting ATPase IA isoform X6	6222	0
XM_020082895.1	zinc finger CCCH domain-containing protein 7B-like isoform X1	8130	0
XM_020082896.1	zinc finger CCCH domain-containing protein 7B-like isoform X1	8042	0
XM_020082897.1	zinc finger CCCH domain-containing protein 7B-like isoform X1	8050	0
XM_020082898.1	zinc finger CCCH domain-containing protein 7B-like isoform X1	8017	0
XM_020082899.1	lethal(3)malignant brain tumor-like protein 2 isoform X1	3651	0
XM_020082900.1	lethal(3)malignant brain tumor-like protein 2 isoform X2	3585	0
XM_020082901.1	lethal(3)malignant brain tumor-like protein 2 isoform X3	3290	0
XM_020082902.1	chondroadherin-like protein	2924	0
XM_020082903.1	ran GTPase-activating protein 1-like	3050	0
XM_020082904.1	ran GTPase-activating protein 1-like	3064	0
XM_020082905.1	thyrotroph embryonic factor-like isoform X2	3071	0
XM_020082906.1	thyrotroph embryonic factor-like isoform X2	2970	0

XM_020082907.1	glutaredoxin domain-containing cysteine-rich protein 2 isoform X1	998	0
XM_020082908.1	PHD finger-like domain-containing protein 5A	553	5.00E-75
XM_020082909.1	disintegrin and metalloproteinase domain-containing protein 9-like	2880	0
XM_020082910.1	60S ribosomal protein L19-like	729	2.38E-85
XM_020082911.1	gap junction alpha-3 protein-like	2599	0
XM_020082912.1	vacuolar fusion protein CCZ1 homolog isoform X1	1871	0
XM_020082913.1	vacuolar fusion protein CCZ1 homolog isoform X1	1795	0
XM_020082914.1	parvalbumin, thymic CPV3-like	835	2.23E-74
XM_020082915.1	ribosomal L1 domain-containing protein 1	1285	0
XM_020082916.1	hemoglobin subunit beta-A-like	536	1.21E-109
XM_020082917.1	gamma-crystallin M2-like isoform X1	538	5.27E-133
XM_020082918.1	gamma-crystallin M2-like isoform X2	534	2.93E-134
XM_020082919.1	39S ribosomal protein L38, mitochondrial	1184	0
XM_020082920.1	cytoplasmic polyadenylation element-binding protein 4-like isoform X1	2444	0
XM_020082921.1	RPE-retinal G protein-coupled receptor-like	1480	0
XM_020082922.1	4-hydroxyphenylpyruvate dioxygenase	1741	0
XM_020082923.1	vacuolar-sorting protein SNF8	1098	1.21E-165
XM_020082924.1	homeobox protein Dlx4b-like	1424	0
XM_020082925.1	von Willebrand factor A domain-containing protein 2	2841	0
XM_020082926.1	von Willebrand factor A domain-containing protein 2	2648	0
XM_020082927.1	hemoglobin subunit alpha-like	485	4.97E-105
XM_020082928.1	cytoplasmic polyadenylation element-binding protein 4-like isoform X1	2890	0
XM_020082929.1	pepsin A-like	1418	0
XM_020082930.1	forkhead box protein I2	1637	0
XM_020082931.1	abl interactor 1-like isoform X1	2069	5.01E-159
XM_020082932.1	abl interactor 1-like isoform X2	2036	6.41E-151
XM_020082933.1	NADH dehydrogenase [ubiquinone] 1 beta subcomplex subunit 8, mitochondrial	689	8.95E-130
XM_020082934.1	gamma-crystallin M2-like	692	9.80E-138
XM_020082935.1	cytoplasmic polyadenylation element-binding protein 4-like isoform X1	2887	0
XM_020082936.1	transmembrane protein 130	2226	0
XM_020082937.1	L-xylulose reductase	1050	6.21E-163
XM_020082938.1	L-xylulose reductase	1054	6.45E-163
XM_020082939.1	WD repeat-containing protein on Y chromosome-like isoform X1	4307	0
XM_020082940.1	WD repeat-containing protein on Y chromosome-like isoform X2	4157	0
XM_020082941.1	cytoplasmic polyadenylation element-binding protein 4-like isoform X1	2441	0
XM_020082942.1	WD repeat-containing protein on Y chromosome-like isoform X1	2846	0
XM_020082943.1	glutathione-specific gamma-glutamylcyclotransferase 1-like	1305	1.15E-120
XM_020082944.1	dnaJ homolog subfamily B member 12	2326	0
XM_020082945.1	transcription factor Sox-8-like	1748	0
XM_020082946.1	heparan sulfate glucosamine 3-O-sulfotransferase 2-like	1410	0
XM_020082947.1	cytoplasmic polyadenylation element-binding protein 4-like isoform X1	2420	0
XM_020082948.1	carbohydrate sulfotransferase 3	2632	0
XM_020082949.1	PREDICTED: uncharacterized protein C16orf52 homolog B-like	2200	2.21E-113
XM_020082950.1	vesicular glutamate transporter 1-like	2936	0
XM_020082951.1	alpha-1,6-mannosylglycoprotein 6-beta-N-acetylglucosaminyltransferase B-like isoform X1	3295	0
XM_020082952.1	alpha-1,6-mannosylglycoprotein 6-beta-N-acetylglucosaminyltransferase B-like isoform X2	3139	0
XM_020082953.1	tripartite motif-containing protein 14-like	2272	0
XM_020082954.1	synaptotagmin-2-like isoform X2	1786	0
XM_020082955.1	cytoplasmic polyadenylation element-binding protein 4-like isoform X4	2214	0
XM_020082956.1	protein NLRC3	4247	0
XM_020082957.1	growth/differentiation factor 10	2731	0
XM_020082958.1	peptidyl-tRNA hydrolase ICT1, mitochondrial	731	2.44E-144
XM_020082959.1	NADPH oxidase organizer 1-like	1994	0
XM_020082960.1	hemoglobin subunit alpha-D-like	1430	1.21E-97
XM_020082961.1	cytochrome c oxidase assembly protein COX19	654	2.33E-64
XM_020082962.1	PI-PLC X domain-containing protein 1-like isoform X1	1622	0
XM_020082963.1	PI-PLC X domain-containing protein 1-like isoform X1	1604	0
XM_020082964.1	zinc finger protein 518A-like	3903	0
XM_020082965.1	phenylethanolamine N-methyltransferase	1181	0

XM_020082966.1	C1q-related factor-like	1602	4.89E-137
XM_020082967.1	granulocyte colony-stimulating factor	1803	3.98E-145
XM_020082968.1	granulocyte colony-stimulating factor	955	4.09E-145
XM_020082969.1	granulocyte colony-stimulating factor	846	1.02E-145
XM_020082970.1	hematopoietically-expressed homeobox protein HHEX-like	726	3.35E-117
XM_020082971.1	steroidogenic acute regulatory protein, mitochondrial-like	1315	0
XM_020082972.1	zinc finger protein 518A-like	3903	0
XM_020082973.1	leucine-rich repeat-containing protein 3B-like	1492	2.18E-174
XM_020082974.1	PREDICTED: aquaporin-8-like	1030	1.93E-162
XM_020082975.1	fascin-2-like isoform X2	2280	0
XM_020082976.1	tubby protein-like	2020	0
XM_020082977.1	transmembrane protein 100-like	2541	9.62E-90
XM_020082978.1	radial spoke head 10 homolog B2	2333	0
XM_020082979.1	synaptic vesicular amine transporter	2316	0
XM_020082980.1	PREDICTED: aquaporin-8-like	971	0
XM_020082981.1	rhomboid-related protein 1	1794	0
XM_020082982.1	cadherin-related family member 1-like isoform X1	3128	0
XM_020082983.1	gap junction alpha-3 protein-like	2358	0
XM_020082984.1	zinc finger protein 518A-like	5193	0
XM_020082985.1	cadherin-related family member 1-like isoform X2	3040	0
XM_020082986.1	meiosis-specific with OB domain-containing protein	1624	0
XM_020082987.1	glutathione peroxidase 6-like	779	7.39E-92
XM_020082988.1	MKL/myocardin-like protein 2 isoform X6	5659	0

XM_020082989.1	MKL/myocardin-like protein 2 isoform X6	5656	0
XM_020082990.1	MKL/myocardin-like protein 2 isoform X3	5626	0
XM_020082991.1	MKL/myocardin-like protein 2 isoform X4	5408	0
XM_020082992.1	MKL/myocardin-like protein 2 isoform X4	5329	0
XM_020082993.1	MKL/myocardin-like protein 2 isoform X6	5566	0
XM_020082994.1	MKL/myocardin-like protein 2 isoform X6	5563	0
XM_020082995.1	MKL/myocardin-like protein 2 isoform X5	5554	0
XM_020082996.1	MKL/myocardin-like protein 2 isoform X4	5125	0
XM_020082997.1	MKL/myocardin-like protein 2 isoform X5	5458	0
XM_020082998.1	large neutral amino acids transporter small subunit 3	3986	0
XM_020082999.1	MKL/myocardin-like protein 2 isoform X4	5051	0
XM_020083000.1	MKL/myocardin-like protein 2 isoform X6	4972	0
XM_020083001.1	MKL/myocardin-like protein 2 isoform X4	5133	0
XM_020083002.1	UPF0505 protein C16orf62 homolog isoform X1	3743	0
XM_020083003.1	UPF0505 protein C16orf62 homolog isoform X2	3742	0
XM_020083004.1	UPF0505 protein C16orf62 homolog isoform X3	3756	0
XM_020083005.1	UPF0505 protein C16orf62 homolog isoform X4	3753	0
XM_020083006.1	UPF0505 protein C16orf62 homolog isoform X5	3585	0
XM_020083007.1	UPF0505 protein C16orf62 homolog isoform X3	3734	0
XM_020083008.1	probable palmitoyltransferase ZDHHC16 isoform X2	2609	0
XM_020083009.1	probable palmitoyltransferase ZDHHC16 isoform X2	2674	0
XM_020083010.1	probable palmitoyltransferase ZDHHC16 isoform X2	2632	0
XM_020083011.1	Golgi to ER traffic protein 4 homolog isoform X1	2426	0
XM_020083012.1	Golgi to ER traffic protein 4 homolog isoform X2	2469	0
XM_020083013.1	mediator of RNA polymerase II transcription subunit 19	1234	1.03E-123
XM_020083014.1	homeobox protein HMX3-B-like	1099	0
XM_020083015.1	neuronal pentraxin-2-like	2199	0
XM_020083016.1	urotensin-2 receptor-like isoform X2	1218	0
XM_020083017.1	WAP, Kazal, immunoglobulin, Kunitz and NTR domain-containing protein 2-like	1740	0
XM_020083018.1	forkhead box protein L1	1114	0
XM_020083019.1	glycylpeptide N-tetradecanoyltransferase 1-like	1578	0
XM_020083020.1	gap junction delta-3 protein	1143	3.88E-171
XM_020083021.1	pyroglutamylated RFamide peptide receptor	2553	0
XM_020083022.1	glucagon receptor-like	1436	0
XM_020083023.1	lysine-specific demethylase 2A	5543	0
XM_020083024.1	serine/threonine-protein kinase LMTK1-like isoform X1	2289	0
XM_020083025.1	proline-rich protein 35	2328	0
XM_020083026.1	transmembrane protein 235-like	961	3.80E-125
XM_020083027.1	leucine-rich repeat-containing protein 18-like	879	1.22E-110
XM_020083028.1	transmembrane protein 26-like	1067	5.71E-170
XM_020083029.1	leucine-rich repeat, immunoglobulin-like domain and transmembrane domain-containing protein 1	1961	0
XM_020083030.1	retinol-binding protein 3-like	1854	0
XM_020083031.1	leucine-rich repeat, immunoglobulin-like domain and transmembrane domain-containing protein 2	1620	0
XM_020083032.1	protein TBATA	1403	0
XM_020083033.1	cathepsin F	2189	0
XM_020083034.1	growth/differentiation factor 2-like	1239	0
XM_020083035.1	PREDICTED: uncharacterized protein LOC109626849	1033	3.49E-112
XM_020083036.1	protein shisa-9B-like	1463	0
XM_020083037.1	stonustoxin subunit beta-like	903	1.17E-163
XM_020083038.1	stonustoxin subunit beta-like	828	2.41E-175
XM_020083039.1	ATP-citrate synthase	3593	0
XM_020083040.1	disintegrin and metalloproteinase domain-containing protein 11-like isoform X1	3001	0
XM_020083041.1	disintegrin and metalloproteinase domain-containing protein 11-like isoform X2	2814	0
XM_020083042.1	glycerol-3-phosphate acyltransferase 4	1845	0
XM_020083043.1	disintegrin and metalloproteinase domain-containing protein 11-like isoform X3	2910	0
XM_020083044.1	kelch-like protein 11	3675	0

XM_020083045.1	peptidyl-prolyl cis-trans isomerase FKBP10-like	2135	0
XM_020083046.1	7-methylguanosine phosphate-specific 5'-nucleotidase-like isoform X1	2222	0
XM_020083047.1	7-methylguanosine phosphate-specific 5'-nucleotidase-like isoform X2	2212	0
XM_020083048.1	AP-4 complex accessory subunit tepsin	3598	0
XM_020083049.1	monocarboxylate transporter 7-like	2777	0
XM_020083050.1	arylsulfatase G	2945	0
XM_020083051.1	arylsulfatase G	2863	0
XM_020083052.1	WD repeat domain phosphoinositide-interacting protein 1	2908	0
XM_020083053.1	PREDICTED: archaemetzincin-2	1760	0
XM_020083054.1	protein ABHD18	2693	0
XM_020083055.1	protein FAM104A	2135	5.88E-64
XM_020083056.1	zinc finger protein 503-like	2126	0
XM_020083057.1	voltage-dependent anion-selective channel protein 2-like	1716	0
XM_020083058.1	catechol O-methyltransferase domain-containing protein 1-like isoform X1	1064	0
XM_020083059.1	catechol O-methyltransferase domain-containing protein 1-like isoform X2	1266	7.01E-178
XM_020083060.1	catechol O-methyltransferase domain-containing protein 1-like isoform X1	994	1.73E-159
XM_020083061.1	protein ABHD18	2711	0
XM_020083062.1	catechol O-methyltransferase domain-containing protein 1-like isoform X2	1077	0
XM_020083063.1	catechol O-methyltransferase domain-containing protein 1-like isoform X2	1064	1.83E-162
XM_020083064.1	calcium-activated potassium channel subunit alpha-1-like	4704	0
XM_020083065.1	rho GTPase-activating protein 22-like isoform X1	4635	0
XM_020083066.1	rho GTPase-activating protein 22-like isoform X1	4125	0
XM_020083067.1	rho GTPase-activating protein 22-like isoform X3	4163	0
XM_020083068.1	protein ABHD18	2707	0
XM_020083069.1	mitogen-activated protein kinase 8 isoform X1	4443	0
XM_020083070.1	mitogen-activated protein kinase 8 isoform X2	4486	0

XM_020083071.1	mitogen-activated protein kinase 8 isoform X3	1707	0
XM_020083072.1	mitogen-activated protein kinase 8 isoform X1	4443	0
XM_020083073.1	vinculin isoform X1	5038	0
XM_020083074.1	vinculin isoform X2	5024	0
XM_020083075.1	adenosine kinase-like isoform X1	1803	0
XM_020083076.1	adenosine kinase-like isoform X2	1771	0
XM_020083077.1	protein ABHD18	2725	0
XM_020083078.1	3-phosphoinositide-dependent protein kinase 1 isoform X1	3497	0
XM_020083079.1	3-phosphoinositide-dependent protein kinase 1 isoform X2	3602	0
XM_020083080.1	3-phosphoinositide-dependent protein kinase 1 isoform X2	3544	0
XM_020083081.1	BTB/POZ domain-containing protein KCTD5-like isoform X2	2516	0
XM_020083082.1	BTB/POZ domain-containing protein KCTD5-like isoform X2	937	3.34E-170
XM_020083083.1	histone acetyltransferase KAT6B isoform X1	7730	0
XM_020083084.1	histone acetyltransferase KAT6B isoform X2	7082	0
XM_020083085.1	dual specificity phosphatase DUPD1-like	1101	6.51E-153
XM_020083086.1	dual specificity phosphatase DUPD1-like	1058	1.82E-152
XM_020083087.1	dual specificity phosphatase DUPD1-like	1040	9.53E-151
XM_020083088.1	dual specificity protein phosphatase 13-like	1370	1.11E-118
XM_020083089.1	dual specificity protein phosphatase 13-like	1274	1.82E-92
XM_020083090.1	immunoglobulin-binding protein 1	1299	2.57E-131
XM_020083091.1	dual specificity phosphatase DUPD1-like	998	9.67E-135
XM_020083092.1	transformation/transcription domain-associated protein isoform X1	11733	0
XM_020083093.1	transformation/transcription domain-associated protein isoform X1	12529	0
XM_020083094.1	transformation/transcription domain-associated protein isoform X1	11709	0
XM_020083095.1	transformation/transcription domain-associated protein isoform X1	11553	0
XM_020083096.1	transformation/transcription domain-associated protein isoform X4	11532	0
XM_020083097.1	zinc finger protein 646-like isoform X1	7413	0
XM_020083098.1	zinc finger protein 646-like isoform X1	7570	0
XM_020083099.1	zinc finger protein 646-like isoform X1	7467	0
XM_020083100.1	zinc finger protein 646-like isoform X1	4286	0
XM_020083101.1	zinc finger protein 668-like isoform X1	2447	0
XM_020083102.1	zinc finger protein 668-like isoform X1	2454	0
XM_020083103.1	heparan sulfate glucosamine 3-O-sulfotransferase 1-like	1843	0
XM_020083104.1	E3 ubiquitin-protein ligase RNF38-like	3637	0
XM_020083105.1	heat-stable enterotoxin receptor-like	3825	0
XM_020083106.1	heat-stable enterotoxin receptor-like	3821	0
XM_020083107.1	heat-stable enterotoxin receptor-like	3905	0
XM_020083108.1	heat-stable enterotoxin receptor-like	3879	0
XM_020083109.1	heat-stable enterotoxin receptor-like	3745	0
XM_020083110.1	phospholipase B-like 1	1819	0
XM_020083111.1	BTB/POZ domain-containing protein KCTD5-like isoform X1	7702	6.33E-156
XM_020083112.1	BTB/POZ domain-containing protein KCTD5-like isoform X1	7603	4.78E-159
XM_020083113.1	BTB/POZ domain-containing protein KCTD5-like isoform X1	7699	3.58E-155
XM_020083114.1	E3 ubiquitin-protein ligase RNF38-like	2977	0
XM_020083115.1	BTB/POZ domain-containing protein KCTD5-like isoform X1	7654	1.11E-144
XM_020083116.1	BTB/POZ domain-containing protein KCTD5-like isoform X4	7651	4.30E-144
XM_020083117.1	protein chibby homolog 1 isoform X1	576	1.60E-80
XM_020083118.1	protein chibby homolog 1 isoform X1	660	1.07E-78
XM_020083119.1	myoferlin-like isoform X1	7049	0
XM_020083120.1	myoferlin-like isoform X2	7010	0
XM_020083121.1	myoferlin-like isoform X3	6992	0
XM_020083122.1	myoferlin-like isoform X4	6980	0
XM_020083123.1	myoferlin-like isoform X5	6860	0

XM_020083124.1	E3 ubiquitin-protein ligase RNF38-like	2946	0
XM_020083125.1	alanyl-tRNA editing protein Aarsd1	1555	0
XM_020083126.1	prostaglandin E synthase 3 isoform X1	817	9.43E-96
XM_020083127.1	methyltransferase-like protein 9 isoform X1	2986	0
XM_020083128.1	methyltransferase-like protein 9 isoform X1	2841	0
XM_020083129.1	methyltransferase-like protein 9 isoform X1	2808	0
XM_020083130.1	ER lumen protein-retaining receptor 2	2272	2.17E-138
XM_020083131.1	DNA cross-link repair 1A protein	3426	0
XM_020083132.1	NHL repeat-containing protein 2	4599	0
XM_020083133.1	histone acetyltransferase p300-like isoform X1	9919	0
XM_020083134.1	histone acetyltransferase p300-like isoform X2	9913	0
XM_020083135.1	histone acetyltransferase p300-like isoform X3	8520	0
XM_020083136.1	zinc finger CCHC domain-containing protein 7-like isoform X1	2257	0
XM_020083137.1	histone acetyltransferase p300-like isoform X4	10328	0
XM_020083138.1	trinucleotide repeat-containing gene 6B protein	8545	0
XM_020083139.1	trinucleotide repeat-containing gene 6B protein	6665	0
XM_020083140.1	canalicular multispecific organic anion transporter 2-like isoform X1	5743	0
XM_020083141.1	canalicular multispecific organic anion transporter 2-like isoform X2	5739	0
XM_020083142.1	canalicular multispecific organic anion transporter 2-like isoform X3	4139	0
XM_020083143.1	glutamate dehydrogenase, mitochondrial	2673	0
XM_020083144.1	zinc finger CCHC domain-containing protein 7-like isoform X1	2044	0
XM_020083145.1	proliferation marker protein Ki-67 isoform X1	5881	0
XM_020083146.1	proliferation marker protein Ki-67 isoform X2	5840	0
XM_020083147.1	receptor-type tyrosine-protein phosphatase H-like isoform X1	2770	0
XM_020083148.1	receptor-type tyrosine-protein phosphatase H-like isoform X1	2257	0
XM_020083149.1	interferon-stimulated 20 kDa exonuclease-like 2	2011	0
XM_020083150.1	interferon-stimulated 20 kDa exonuclease-like 2	2004	0
XM_020083151.1	putative nuclease HARBI1	1541	0
XM_020083152.1	phosphatidylinositol N-acetylglucosaminyltransferase subunit P	1214	1.67E-80
XM_020083153.1	zinc finger CCHC domain-containing protein 7-like isoform X1	1752	0
XM_020083154.1	rab11 family-interacting protein 3 isoform X1	5226	0
XM_020083155.1	rab11 family-interacting protein 3 isoform X2	3845	0
XM_020083156.1	rab11 family-interacting protein 3 isoform X3	3047	0
XM_020083157.1	peroxisomal 2,4-dienoyl-CoA reductase isoform X1	1358	0
XM_020083158.1	peroxisomal 2,4-dienoyl-CoA reductase isoform X2	1422	0
XM_020083159.1	centrosome-associated protein CEP250-like isoform X1	8907	0
XM_020083160.1	centrosome-associated protein CEP250-like isoform X2	8876	0
XM_020083161.1	centrosome-associated protein CEP250-like isoform X3	8841	0
XM_020083162.1	centrosome-associated protein CEP250-like isoform X4	8786	0
XM_020083163.1	centrosome-associated protein CEP250-like isoform X5	8777	0
XM_020083164.1	carnitine O-palmitoyltransferase 1, liver isoform-like isoform X1	3964	0
XM_020083165.1	carnitine O-palmitoyltransferase 1, liver isoform-like isoform X2	3961	0
XM_020083166.1	mitogen-activated protein kinase kinase kinase kinase 4-like	1308	9.17E-178
XM_020083167.1	dynein assembly factor 3, axonemal	1607	0
XM_020083168.1	troponin T, slow skeletal muscle	765	1.79E-120
XM_020083169.1	rho GTPase-activating protein 17 isoform X1	5837	0
XM_020083170.1	rho GTPase-activating protein 17 isoform X2	5804	0
XM_020083171.1	rho GTPase-activating protein 17 isoform X1	5861	0
XM_020083172.1	rho GTPase-activating protein 17 isoform X4	5555	0
XM_020083173.1	protein phosphatase 1B-like isoform X1	3886	0
XM_020083174.1	protein phosphatase 1B-like isoform X1	3566	0
XM_020083175.1	protein phosphatase 1B-like isoform X1	3858	0
XM_020083176.1	protein phosphatase 1B-like isoform X1	2911	0
XM_020083177.1	disks large homolog 5-like isoform X1	7486	0
XM_020083178.1	disks large homolog 5-like isoform X2	7471	0
XM_020083179.1	ligand of Numb protein X 2-like	3886	0
XM_020083180.1	ras-associating and dilute domain-containing protein-like	4717	0
XM_020083181.1	sulfotransferase family cytosolic 2B member 1-like	1023	0
XM_020083182.1	sulfotransferase family cytosolic 2B member 1-like	1141	0
XM_020083183.1	hexosaminidase D	1868	0
XM_020083184.1	hexosaminidase D	1866	0
XM_020083185.1	nuclear prelamin A recognition factor	2474	0
XM_020083186.1	PREDICTED: uncharacterized protein C17orf62 homolog	1498	2.36E-115
XM_020083187.1	ligand of Numb protein X 2-like	3920	0
XM_020083188.1	ketimine reductase mu-crystallin	1522	0
XM_020083189.1	ras-related C3 botulinum toxin substrate 1-like	2277	1.02E-124
XM_020083190.1	F-box only protein 11-like isoform X1	4294	0
XM_020083191.1	F-box only protein 11-like isoform X2	4291	0
XM_020083192.1	F-box only protein 11-like isoform X3	4273	0
XM_020083193.1	F-box only protein 11-like isoform X4	4270	0
XM_020083194.1	DNA mismatch repair protein Msh2-like	475	2.37E-48
XM_020083195.1	ligand of Numb protein X 2-like	3826	0
XM_020083196.1	serine/threonine-protein kinase tousled-like 2 isoform X2	3321	0
XM_020083197.1	serine/threonine-protein kinase tousled-like 2 isoform X2	3508	0
XM_020083198.1	serine/threonine-protein kinase tousled-like 2 isoform X2	3050	0
XM_020083199.1	serine/threonine-protein kinase tousled-like 2 isoform X2	3226	0
XM_020083200.1	PREDICTED: beclin-1	2162	0
XM_020083201.1	dynamin-binding protein-like isoform X1	6407	0
XM_020083202.1	dynamin-binding protein-like isoform X2	6389	0

XM_020083203.1	dynamin-binding protein-like isoform X3	5367	0
XM_020083204.1	SPRY domain-containing SOCS box protein 3-like isoform X1	1916	0
XM_020083205.1	ligand of Numb protein X 2-like	3796	0
XM_020083206.1	SPRY domain-containing SOCS box protein 3-like isoform X2	1913	0
XM_020083207.1	SPRY domain-containing SOCS box protein 3-like isoform X1	1893	0
XM_020083208.1	cytosolic Fe-S cluster assembly factor NUBP2 isoform X1	2025	1.61E-180
XM_020083209.1	cytosolic Fe-S cluster assembly factor NUBP2 isoform X1	2003	1.25E-175
XM_020083210.1	V-type proton ATPase 16 kDa proteolipid subunit-like	1509	1.29E-80
XM_020083211.1	zinc finger protein 385C isoform X1	2911	0
XM_020083212.1	dnaJ homolog subfamily C member 7-like isoform X1	2968	0
XM_020083213.1	dnaJ homolog subfamily C member 7-like isoform X2	2950	0
XM_020083214.1	dnaJ homolog subfamily C member 7-like isoform X3	2884	0
XM_020083215.1	dnaJ homolog subfamily C member 7-like isoform X4	2866	0
XM_020083216.1	angiotensin-converting enzyme	4126	0
XM_020083217.1	integrin alpha-3	4457	0
XM_020083218.1	solute carrier family 25 member 43	1629	0
XM_020083219.1	proteasome activator complex subunit 4B-like	6317	0
XM_020083220.1	thrombospondin-2 isoform X2	2862	0
XM_020083221.1	ectonucleoside triphosphate diphosphohydrolase 1 isoform X1	1954	0
XM_020083222.1	ectonucleoside triphosphate diphosphohydrolase 1 isoform X2	2069	0
XM_020083223.1	ectonucleoside triphosphate diphosphohydrolase 1 isoform X2	2023	0
XM_020083224.1	potassium channel subfamily K member 1-like	2000	0
XM_020083225.1	aconitate hydratase, mitochondrial	3901	0
XM_020083226.1	myosin phosphatase Rho-interacting protein-like isoform X1	3770	0
XM_020083227.1	myosin phosphatase Rho-interacting protein-like isoform X2	3710	0
XM_020083228.1	mediator of RNA polymerase II transcription subunit 1	6193	0
XM_020083229.1	palmitoyltransferase ZDHHC6 isoform X1	2255	0
XM_020083230.1	palmitoyltransferase ZDHHC6 isoform X2	2252	0
XM_020083231.1	vesicle transport through interaction with t-SNAREs homolog 1A isoform X1	2700	2.37E-136
XM_020083232.1	vesicle transport through interaction with t-SNAREs homolog 1A isoform X2	2677	4.19E-132
XM_020083233.1	vesicle transport through interaction with t-SNAREs homolog 1A isoform X3	2711	4.95E-129
XM_020083234.1	glypican-5-like isoform X2	3597	0
XM_020083235.1	vesicle transport through interaction with t-SNAREs homolog 1A isoform X4	2688	8.07E-125
XM_020083236.1	ubiquitin carboxyl-terminal hydrolase 42 isoform X1	5052	0
XM_020083237.1	ubiquitin carboxyl-terminal hydrolase 42 isoform X1	5636	0
XM_020083238.1	ubiquitin carboxyl-terminal hydrolase 42 isoform X1	5051	0
XM_020083239.1	myosin light chain kinase, smooth muscle-like	2414	0
XM_020083240.1	bifunctional coenzyme A synthase	3465	0
XM_020083241.1	UPF0488 protein C8orf33 homolog	1547	0
XM_020083242.1	histone H3.3	821	5.28E-94
XM_020083243.1	protein diaphanous homolog 1	5654	0
XM_020083244.1	protein CCSMST1	814	6.16E-96
XM_020083245.1	putative pre-mRNA-splicing factor ATP-dependent RNA helicase DHX32	2893	0
XM_020083246.1	matrix metalloproteinase-21	2209	0
XM_020083247.1	BRCA2 and CDKN1A-interacting protein	1480	3.35E-177
XM_020083248.1	uroporphyrinogen-III synthase	1255	0
XM_020083249.1	uroporphyrinogen-III synthase	1428	0
XM_020083250.1	C-Jun-amino-terminal kinase-interacting protein 4-like isoform X1	7130	0
XM_020083251.1	C-Jun-amino-terminal kinase-interacting protein 4-like isoform X2	7127	0
XM_020083252.1	C-Jun-amino-terminal kinase-interacting protein 4-like isoform X3	7057	0
XM_020083253.1	C-Jun-amino-terminal kinase-interacting protein 4-like isoform X1	6415	0
XM_020083254.1	C-Jun-amino-terminal kinase-interacting protein 4-like isoform X3	6342	0
XM_020083255.1	protein diaphanous homolog 2 isoform X2	7480	0
XM_020083256.1	mismatch repair endonuclease PMS2	2980	0
XM_020083257.1	eukaryotic translation initiation factor 2-alpha kinase 1	2388	0
XM_020083258.1	ankyrin repeat domain-containing protein 61 isoform X1	1741	0
XM_020083259.1	ankyrin repeat domain-containing protein 61 isoform X2	1699	0
XM_020083260.1	dedicator of cytokinesis protein 1	6584	0
XM_020083261.1	dedicator of cytokinesis protein 1	6456	0
XM_020083262.1	dedicator of cytokinesis protein 1	6391	0
XM_020083263.1	protein FAM196A	2073	0
XM_020083264.1	protein diaphanous homolog 2 isoform X2	7429	0
XM_020083265.1	titin homolog	7720	0
XM_020083266.1	titin homolog	7625	0
XM_020083267.1	transcription factor Sox-9-A-like	3358	0
XM_020083268.1	bone morphogenetic protein 2-like	1310	0
XM_020083269.1	DNA-directed RNA polymerase III subunit RPC4-like isoform X1	2760	0
XM_020083270.1	DNA-directed RNA polymerase III subunit RPC4-like isoform X2	2757	0
XM_020083271.1	protein diaphanous homolog 2 isoform X2	3884	0
XM_020083272.1	DNA-directed RNA polymerase III subunit RPC4-like isoform X3	2757	0
XM_020083273.1	SWI/SNF-related matrix-associated actin-dependent regulator of chromatin subfamily E member 1-related isoform X1	1163	2.98E-161
XM_020083274.1	leucine-rich repeat-containing protein 45 isoform X1	2915	0
XM_020083275.1	leucine-rich repeat-containing protein 45 isoform X2	2825	1.57E-176
XM_020083276.1	leucine-rich repeat-containing protein 45 isoform X3	2822	5.87E-176
XM_020083277.1	leucine-rich repeat-containing protein 45 isoform X1	2809	4.23E-171
XM_020083278.1	leucine-rich repeat-containing protein 45-like	1234	0
XM_020083279.1	leucine-rich repeat-containing protein 45-like	1100	0
XM_020083280.1	ubiquitin domain-containing protein 1-like	3537	1.52E-141
XM_020083281.1	protein transport protein Sec31A-like	5414	0

XM_020083282.1	protein transport protein Sec31A-like	5434	0
XM_020083283.1	putative sodium-coupled neutral amino acid transporter 10 isoform X1	4005	0
XM_020083284.1	FACT complex subunit SSRP1 isoform X1	2472	0
XM_020083285.1	putative sodium-coupled neutral amino acid transporter 10 isoform X2	4005	0
XM_020083286.1	putative sodium-coupled neutral amino acid transporter 10 isoform X3	4002	0
XM_020083287.1	putative sodium-coupled neutral amino acid transporter 10 isoform X4	3984	0
XM_020083288.1	NADH dehydrogenase [ubiquinone] 1 alpha subcomplex assembly factor 8	737	2.83E-43
XM_020083289.1	E3 ubiquitin-protein ligase BRE1B isoform X1	3352	0
XM_020083290.1	E3 ubiquitin-protein ligase BRE1B isoform X2	3349	0
XM_020083291.1	annexin A4-like	2947	0
XM_020083292.1	paladin isoform X1	4380	0
XM_020083293.1	FACT complex subunit SSRP1 isoform X1	2391	0
XM_020083294.1	paladin isoform X1	4567	0
XM_020083295.1	nodal homolog	1681	0
XM_020083296.1	leucine-rich repeat-containing protein 20	1115	2.48E-125
XM_020083297.1	leucine-rich repeat-containing protein 20	888	4.65E-127
XM_020083298.1	myosin-16-like isoform X2	4811	0
XM_020083299.1	myosin-16-like isoform X2	4805	0
XM_020083300.1	myosin-16-like isoform X4	4788	0
XM_020083301.1	myosin-16-like isoform X4	4630	0
XM_020083302.1	FACT complex subunit SSRP1 isoform X1	2391	0
XM_020083303.1	putative RNA-binding protein Luc7-like 1 isoform X1	2307	2.01E-155
XM_020083304.1	putative RNA-binding protein Luc7-like 1 isoform X1	2381	1.01E-138
XM_020083305.1	epidermal growth factor receptor kinase substrate 8-like protein 1	2286	0
XM_020083306.1	probable JmjC domain-containing histone demethylation protein 2C isoform X1	10583	0
XM_020083307.1	probable JmjC domain-containing histone demethylation protein 2C isoform X1	10248	0
XM_020083308.1	TOM1-like protein 2 isoform X1	3155	0
XM_020083309.1	TOM1-like protein 2 isoform X2	3146	0
XM_020083310.1	TOM1-like protein 2 isoform X3	3095	0
XM_020083311.1	FACT complex subunit SSRP1 isoform X1	2466	0
XM_020083312.1	TOM1-like protein 2 isoform X1	3281	0
XM_020083313.1	ryanodine receptor 2	19371	0
XM_020083314.1	tripartite motif-containing protein 65	1611	0
XM_020083315.1	WW domain-binding protein 2	3601	3.69E-131
XM_020083316.1	solute carrier family 35 member G1	3811	0
XM_020083317.1	leucine-rich glioma-inactivated protein 1-like	1891	0
XM_020083318.1	U4/U6 small nuclear ribonucleoprotein Prp4 isoform X2	1595	0
XM_020083319.1	glycerol-3-phosphate phosphatase	2258	0
XM_020083320.1	BRICHOS domain-containing protein 5-like isoform X1	1371	3.18E-151
XM_020083321.1	BRICHOS domain-containing protein 5-like isoform X2	1502	6.72E-150
XM_020083322.1	zinc finger MIZ domain-containing protein 1-like isoform X2	6734	0
XM_020083323.1	cardiotrophin-like cytokine factor 1	3983	0
XM_020083324.1	zinc finger MIZ domain-containing protein 1-like isoform X1	6135	0
XM_020083325.1	DNA topoisomerase 2-alpha	5152	0
XM_020083326.1	tectonin beta-propeller repeat-containing protein 1-like isoform X2	5255	0
XM_020083327.1	tectonin beta-propeller repeat-containing protein 1-like isoform X2	5407	0
XM_020083328.1	tectonin beta-propeller repeat-containing protein 1-like isoform X2	5411	0
XM_020083329.1	class A basic helix-loop-helix protein 15	1474	3.05E-75
XM_020083330.1	class A basic helix-loop-helix protein 15	1582	7.79E-75
XM_020083331.1	class A basic helix-loop-helix protein 15	1531	5.04E-75
XM_020083332.1	protein kinase C epsilon type-like	4797	0
XM_020083333.1	integrin beta-3 isoform X1	2945	0
XM_020083334.1	integrin beta-3 isoform X1	2250	0
XM_020083335.1	carboxypeptidase N catalytic chain	1982	0
XM_020083336.1	neutral alpha-glucosidase AB isoform X3	4260	0
XM_020083337.1	PREDICTED: calpain-15-like	6888	0
XM_020083338.1	transmembrane protein 238-like	2197	3.40E-69
XM_020083339.1	signal transducer and activator of transcription 5B-like isoform X1	5564	0
XM_020083340.1	signal transducer and activator of transcription 5B-like isoform X1	5455	0
XM_020083341.1	signal transducer and activator of transcription 5B-like isoform X1	5561	0
XM_020083342.1	myosin-binding protein C, fast-type-like isoform X3	3582	0
XM_020083343.1	myosin-binding protein C, fast-type-like isoform X2	3662	0
XM_020083344.1	myosin-binding protein C, fast-type-like isoform X3	3622	0
XM_020083345.1	neutral alpha-glucosidase AB isoform X3	4199	0
XM_020083346.1	DNA repair protein RAD51 homolog 1 isoform X1	1290	0
XM_020083347.1	eukaryotic peptide chain release factor GTP-binding subunit ERF3A	2655	0
XM_020083348.1	zinc finger protein 518A-like	5808	0
XM_020083349.1	zinc finger protein 518A-like	5441	0
XM_020083350.1	zinc finger protein 518A-like	5412	0
XM_020083351.1	zinc finger protein 135-like	3261	0
XM_020083352.1	transcription factor 7-like 2 isoform X1	4042	0
XM_020083353.1	neutral alpha-glucosidase AB isoform X3	4265	0
XM_020083354.1	transcription factor 7-like 2 isoform X2	4027	0
XM_020083355.1	transcription factor 7-like 2 isoform X3	4027	0
XM_020083356.1	transcription factor 7-like 2 isoform X4	4012	0
XM_020083357.1	transcription factor 7-like 2 isoform X5	3991	0
XM_020083358.1	transcription factor 7-like 2 isoform X6	3991	0
XM_020083359.1	transcription factor 7-like 2 isoform X7	3976	0
XM_020083360.1	transcription factor 7-like 2 isoform X8	3976	0

XM_020083361.1	transcription factor 7-like 2 isoform X9	1894	0
XM_020083362.1	transcription factor 7-like 2 isoform X10	1918	0
XM_020083363.1	transcription factor 7-like 2 isoform X9	1810	0
XM_020083364.1	ras-related protein Rab-28	1090	3.00E-144
XM_020083365.1	neutral alpha-glucosidase AB isoform X3	4237	0
XM_020083366.1	transcription factor 7-like 2 isoform X11	1903	0
XM_020083367.1	transcription factor 7-like 2 isoform X12	1888	0
XM_020083368.1	transcription factor 7-like 2 isoform X9	1929	0
XM_020083369.1	transcription factor 7-like 2 isoform X2	1914	0
XM_020083370.1	transcription factor 7-like 2 isoform X15	1867	0
XM_020083371.1	inositol hexakisphosphate kinase 1	7486	0
XM_020083372.1	ornithine aminotransferase, mitochondrial	2624	0
XM_020083373.1	ornithine aminotransferase, mitochondrial	2745	0
XM_020083374.1	phospholysine phosphohistidine inorganic pyrophosphate phosphatase	1134	0
XM_020083375.1	neutral alpha-glucosidase AB isoform X4	4194	0
XM_020083376.1	pentraxin fusion protein-like	1478	3.87E-166
XM_020083377.1	beta-1,3-N-acetylglucosaminyltransferase radical fringe-like	4166	0
XM_020083378.1	calcium/calmodulin-dependent protein kinase type II subunit gamma-like	3233	3.97E-109
XM_020083379.1	serine/threonine-protein phosphatase CPPED1	4593	0
XM_020083380.1	1-phosphatidylinositol 4,5-bisphosphate phosphodiesterase epsilon-1 isoform X1	11282	0
XM_020083381.1	1-phosphatidylinositol 4,5-bisphosphate phosphodiesterase epsilon-1 isoform X2	11240	0
XM_020083382.1	MAGUK p55 subfamily member 3-like isoform X1	2800	0
XM_020083383.1	MAGUK p55 subfamily member 3-like isoform X2	2730	0
XM_020083384.1	26S protease regulatory subunit 8 isoform X1	1489	0
XM_020083385.1	26S protease regulatory subunit 8 isoform X2	1382	0
XM_020083386.1	tyrosine-protein kinase BTK	2348	0
XM_020083387.1	neurexin-1a-like isoform X1	4782	0
XM_020083388.1	neurexin-1a-like isoform X2	4758	0
XM_020083389.1	neurexin-1a-like isoform X3	4755	0
XM_020083390.1	neurexin-1a-like isoform X4	4752	0
XM_020083391.1	neurexin-1a-like isoform X5	4734	0
XM_020083392.1	neurexin-1a-like isoform X6	4728	0
XM_020083393.1	neurexin-1a-like isoform X7	4725	0
XM_020083394.1	neurexin-1a-like isoform X8	4701	0
XM_020083395.1	neurexin-1a-like isoform X9	4653	0
XM_020083396.1	tyrosine-protein kinase BTK	2275	0
XM_020083397.1	neurexin-3-beta isoform X7	3302	9.82E-74
XM_020083398.1	mediator of RNA polymerase II transcription subunit 24 isoform X1	3301	0
XM_020083399.1	mediator of RNA polymerase II transcription subunit 24 isoform X1	3287	0
XM_020083400.1	mediator of RNA polymerase II transcription subunit 24 isoform X1	3289	0
XM_020083401.1	ATP-sensitive inward rectifier potassium channel 12	1302	0
XM_020083402.1	ubiquitin thioesterase Zranb1-like	3279	0
XM_020083403.1	ubiquitin thioesterase Zranb1-like	3121	0
XM_020083404.1	ATP synthase mitochondrial F1 complex assembly factor 2	2057	0
XM_020083405.1	glucose-induced degradation protein 4 homolog	2532	1.26E-140
XM_020083406.1	cAMP-dependent protein kinase type I-alpha regulatory subunit	3047	0
XM_020083407.1	lethal(2) giant larvae protein homolog 2 isoform X1	4770	0
XM_020083408.1	lethal(2) giant larvae protein homolog 2 isoform X2	4820	0
XM_020083409.1	lethal(2) giant larvae protein homolog 2 isoform X3	3330	0
XM_020083410.1	PREDICTED: uncharacterized protein LOC109627051 isoform X1	1352	0
XM_020083411.1	PREDICTED: uncharacterized protein LOC109627051 isoform X1	1410	0
XM_020083412.1	PREDICTED: uncharacterized protein LOC109627051 isoform X2	1247	0
XM_020083413.1	alpha-galactosidase A	1966	0
XM_020083414.1	glycerol-3-phosphate acyltransferase 2, mitochondrial-like	5679	0
XM_020083415.1	parvalbumin, thymic-like	738	2.93E-76
XM_020083416.1	parvalbumin beta-like	895	2.14E-69
XM_020083417.1	ribosome biogenesis protein BMS1 homolog	3860	0
XM_020083418.1	coiled-coil domain-containing protein 186 isoform X1	4514	0
XM_020083419.1	coiled-coil domain-containing protein 186 isoform X2	4496	0
XM_020083420.1	mitochondrial Rho GTPase 2-like	3161	0
XM_020083421.1	SH3 domain-binding protein 1-like	3182	0
XM_020083422.1	cdc42 effector protein 1-like	3068	0
XM_020083423.1	histone-lysine N-methyltransferase 2D-like	2465	0
XM_020083424.1	60S ribosomal protein L36a	483	9.73E-63
XM_020083425.1	gap junction gamma-1 protein-like	2521	0
XM_020083426.1	gap junction gamma-1 protein-like	2517	0
XM_020083427.1	gap junction gamma-1 protein-like	2366	0
XM_020083428.1	exocyst complex component 6 isoform X1	2633	0
XM_020083429.1	exocyst complex component 6 isoform X2	2629	0
XM_020083430.1	exocyst complex component 6 isoform X3	2617	0
XM_020083431.1	exocyst complex component 6 isoform X4	2599	0
XM_020083432.1	exocyst complex component 6 isoform X5	2587	0
XM_020083433.1	exocyst complex component 6 isoform X1	2597	0
XM_020083434.1	exocyst complex component 6 isoform X1	2585	0
XM_020083435.1	ankyrin repeat and SOCS box protein 12-like	1801	0
XM_020083436.1	zinc transporter ZIP11 isoform X1	1985	1.35E-169
XM_020083437.1	zinc transporter ZIP11 isoform X2	1964	4.37E-164
XM_020083438.1	elongator complex protein 1	4187	0
XM_020083439.1	protein Tob1-like	2299	0

XM_020083440.1	protein Tob1-like	2430	0
XM_020083441.1	dephospho-CoA kinase domain-containing protein	2327	1.75E-149
XM_020083442.1	protein FAM234A	2824	0
XM_020083443.1	protein FAM234A	2828	0
XM_020083444.1	protein FAM234A	2820	0
XM_020083445.1	protein FAM234A	2594	0
XM_020083446.1	protein FAM234A	2560	0
XM_020083447.1	cytochrome c oxidase subunit 6B1-like	734	2.53E-61
XM_020083448.1	metastasis-associated protein MTA3-like	3571	0
XM_020083449.1	MKL/myocardin-like protein 2 isoform X2	7600	0
XM_020083450.1	MKL/myocardin-like protein 2 isoform X2	7564	0
XM_020083451.1	F-box only protein 38-like	1756	2.48E-62
XM_020083452.1	DCN1-like protein 3	3241	0
XM_020083453.1	DCN1-like protein 3	2885	0
XM_020083454.1	integrin beta-4 isoform X2	7952	0
XM_020083455.1	integrin beta-4 isoform X2	6182	0
XM_020083456.1	integrin beta-4 isoform X2	6072	0
XM_020083457.1	integrin beta-4 isoform X2	6031	0
XM_020083458.1	integrin beta-4 isoform X2	5913	0
XM_020083459.1	forkhead box protein K1	2471	0
XM_020083460.1	serine/threonine-protein kinase SBK1-like	4010	0
XM_020083461.1	retinol dehydrogenase 13	1425	0
XM_020083462.1	MAGUK p55 subfamily member 2 isoform X2	3569	0
XM_020083463.1	MAGUK p55 subfamily member 2 isoform X2	3375	0
XM_020083464.1	MAGUK p55 subfamily member 2 isoform X2	3372	0
XM_020083465.1	highly divergent homeobox isoform X1	2841	0
XM_020083466.1	peptide Y	617	2.41E-52
XM_020083467.1	synaptic vesicle membrane protein VAT-1 homolog	2256	0
XM_020083468.1	rho-related GTP-binding protein RhoN	1635	1.14E-172
XM_020083469.1	major facilitator superfamily domain-containing protein 1-like	2413	0
XM_020083470.1	crossover junction endonuclease EME1	1613	0
XM_020083471.1	crossover junction endonuclease EME1	1649	0
XM_020083472.1	metalloproteinase inhibitor 2-like	2773	1.15E-158
XM_020083473.1	highly divergent homeobox isoform X2	2838	0
XM_020083474.1	WD repeat-containing protein 24	3039	0
XM_020083475.1	WD repeat-containing protein 24	3090	0
XM_020083476.1	WD repeat-containing protein 24	3032	0
XM_020083477.1	PREDICTED: uncharacterized protein LOC109627089	1070	3.16E-159
XM_020083478.1	PREDICTED: uncharacterized protein LOC109627089	949	6.54E-160
XM_020083479.1	receptor-type tyrosine-protein phosphatase epsilon-like isoform X1	4137	0
XM_020083480.1	receptor-type tyrosine-protein phosphatase epsilon-like isoform X2	4130	0
XM_020083481.1	receptor-type tyrosine-protein phosphatase epsilon-like isoform X3	4090	0
XM_020083482.1	receptor-type tyrosine-protein phosphatase epsilon-like isoform X4	2667	0
XM_020083483.1	receptor-type tyrosine-protein phosphatase epsilon-like isoform X3	2073	0
XM_020083484.1	zinc finger CCHC domain-containing protein 24-like	2709	1.62E-166
XM_020083485.1	peptidyl-prolyl cis-trans isomerase-like	978	4.48E-142
XM_020083486.1	transcription factor Sox-3	1870	0
XM_020083487.1	DNA-directed RNA polymerase III subunit RPC1	4937	0
XM_020083488.1	MMS19 nucleotide excision repair protein homolog isoform X1	3868	0
XM_020083489.1	MMS19 nucleotide excision repair protein homolog isoform X2	3865	0
XM_020083490.1	testis-expressed sequence 2 protein-like	3699	0
XM_020083491.1	neuralized-like protein 2	1627	0
XM_020083492.1	methionine-R-sulfoxide reductase B1-A-like	1144	7.87E-74
XM_020083493.1	glucosidase 2 subunit beta	2284	0
XM_020083494.1	neuronal acetylcholine receptor subunit alpha-7-like	3728	0
XM_020083495.1	ribosome biogenesis protein TSR3 homolog	1200	2.62E-176
XM_020083496.1	phosphoethanolamine/phosphocholine phosphatase isoform X1	4087	0
XM_020083497.1	PREDICTED: uncharacterized protein LOC109627103	4117	0
XM_020083498.1	activating signal cointegrator 1 complex subunit 1	1435	0
XM_020083499.1	activating signal cointegrator 1 complex subunit 1	1211	0
XM_020083500.1	anaphase-promoting complex subunit 16	1036	3.82E-54
XM_020083501.1	28 kDa heat- and acid-stable phosphoprotein-like	2016	5.93E-54
XM_020083502.1	interleukin-21 receptor-like	1433	0
XM_020083503.1	SUMO-conjugating enzyme UBC9	1236	2.82E-114
XM_020083504.1	SUMO-conjugating enzyme UBC9	1552	9.52E-113
XM_020083505.1	WD repeat domain phosphoinositide-interacting protein 3	2084	0
XM_020083506.1	protocadherin Fat 2	15703	0
XM_020083507.1	paired box protein Pax-2a-like isoform X1	3649	0
XM_020083508.1	paired box protein Pax-2a-like isoform X2	3639	0
XM_020083509.1	paired box protein Pax-2a-like isoform X3	3627	0
XM_020083510.1	paired box protein Pax-2a-like isoform X4	3618	0
XM_020083511.1	paired box protein Pax-2a-like isoform X5	3612	0
XM_020083512.1	paired box protein Pax-2a-like isoform X6	3602	0
XM_020083513.1	disks large-associated protein 5 isoform X1	3145	0
XM_020083514.1	disks large-associated protein 5 isoform X2	2950	0
XM_020083515.1	intraflagellar transport protein 140 homolog	4533	0
XM_020083516.1	intraflagellar transport protein 140 homolog	5202	0
XM_020083517.1	glycoprotein-N-acetylgalactosamine 3-beta-galactosyltransferase 1-B-like	2700	0
XM_020083518.1	transmembrane protein 204	2466	9.80E-122

XM_020083519.1	potassium voltage-gated channel subfamily C member 1-like isoform X1	2349	0
XM_020083520.1	potassium voltage-gated channel subfamily C member 1-like isoform X1	2402	0
XM_020083521.1	conserved oligomeric Golgi complex subunit 7	3303	0
XM_020083522.1	protein KIAA0556 homolog isoform X1	6584	0
XM_020083523.1	protein KIAA0556 homolog isoform X1	6973	0
XM_020083524.1	protein KIAA0556 homolog isoform X1	6557	0
XM_020083525.1	protein KIAA0556 homolog isoform X1	6482	0
XM_020083526.1	PREDICTED: josephin-1	3858	2.11E-131
XM_020083527.1	voltage-dependent T-type calcium channel subunit alpha-1G-like	5707	0
XM_020083528.1	protein unc-13 homolog D isoform X1	3429	0
XM_020083529.1	protein unc-13 homolog D isoform X2	3364	0
XM_020083530.1	malignant T-cell-amplified sequence 1	915	1.25E-133
XM_020083531.1	zinc finger protein Pegasus-like	2357	0
XM_020083532.1	zinc finger protein Pegasus-like	2411	0
XM_020083533.1	zinc finger protein Pegasus-like	2277	0
XM_020083534.1	L-seryl-tRNA(Sec) kinase	2194	0
XM_020083535.1	retinol-binding protein 4	1358	2.74E-149
XM_020083536.1	meteorin-like protein	2616	0
XM_020083537.1	sn1-specific diacylglycerol lipase beta	3629	0
XM_020083538.1	spermatogenesis-associated protein 20 isoform X1	4632	0
XM_020083539.1	spermatogenesis-associated protein 20 isoform X2	2517	0
XM_020083540.1	spermatogenesis-associated protein 20 isoform X3	2521	0
XM_020083541.1	GH3 domain-containing protein	3094	0
XM_020083542.1	glutamate receptor 1-like	6304	0
XM_020083543.1	5'(3')-deoxyribonucleotidase, mitochondrial isoform X1	1646	2.78E-174
XM_020083544.1	5'(3')-deoxyribonucleotidase, mitochondrial isoform X1	1398	5.96E-159
XM_020083545.1	ras-related protein Rab-5C-like	1812	3.59E-138
XM_020083546.1	ras-related protein Rab-5C-like	1715	6.36E-139
XM_020083547.1	polymerase I and transcript release factor-like	4296	0
XM_020083548.1	cytohesin-1 isoform X1	1919	0
XM_020083549.1	phosphorylase b kinase gamma catalytic chain, liver/testis isoform	2163	0
XM_020083550.1	coiled-coil domain-containing protein 189	1029	0
XM_020083551.1	cold shock domain-containing protein C2	3255	4.05E-82
XM_020083552.1	SH3 and multiple ankyrin repeat domains protein 1-like	9121	0
XM_020083553.1	WD repeat-containing protein 90	5985	0
XM_020083554.1	protein FAM53B	2304	0
XM_020083555.1	disintegrin and metalloproteinase domain-containing protein 12-like	5034	0
XM_020083556.1	matrix metalloproteinase-17-like	3085	0
XM_020083557.1	matrix metalloproteinase-17-like	3106	0
XM_020083558.1	carbonic anhydrase 4-like	1803	0
XM_020083559.1	2-aminoethanethiol dioxygenase	2788	1.18E-173
XM_020083560.1	terminal uridylyltransferase 7	2937	0
XM_020083561.1	zinc finger protein ubi-d4 isoform X1	2848	0
XM_020083562.1	LIM domain-binding protein 3-like isoform X1	1791	0
XM_020083563.1	LIM domain-binding protein 3-like isoform X1	1718	0
XM_020083564.1	LIM domain-binding protein 3-like isoform X1	1767	0
XM_020083565.1	extracellular serine/threonine protein kinase FAM20C-like	4163	0
XM_020083566.1	RNA-binding protein with serine-rich domain 1-like	1911	5.46E-82
XM_020083567.1	UBA-like domain-containing protein 1 isoform X1	2933	1.73E-67
XM_020083568.1	zinc finger protein ubi-d4 isoform X2	2845	0
XM_020083569.1	UBA-like domain-containing protein 1 isoform X2	2893	1.37E-52
XM_020083570.1	histone H1.0-B	1419	5.60E-50
XM_020083571.1	TBC1 domain family member 24	2176	0
XM_020083572.1	NADPH:adrenodoxin oxidoreductase, mitochondrial	2150	0
XM_020083573.1	PREDICTED: uncharacterized protein LOC109627158 isoform X1	889	8.71E-165
XM_020083574.1	PREDICTED: uncharacterized protein LOC109627158 isoform X2	889	8.07E-166
XM_020083575.1	PREDICTED: uncharacterized protein LOC109627158 isoform X3	862	5.07E-158
XM_020083576.1	PREDICTED: uncharacterized protein LOC109627158 isoform X4	859	8.23E-159
XM_020083577.1	zinc finger protein ubi-d4 isoform X3	2713	0
XM_020083578.1	PREDICTED: uncharacterized protein LOC109627158 isoform X5	859	5.92E-158
XM_020083579.1	PREDICTED: uncharacterized protein LOC109627158 isoform X6	859	2.00E-158
XM_020083580.1	PREDICTED: uncharacterized protein LOC109627158 isoform X7	829	1.28E-151
XM_020083581.1	PREDICTED: uncharacterized protein LOC109627158 isoform X8	829	1.70E-150
XM_020083582.1	PREDICTED: uncharacterized protein LOC109627158 isoform X9	799	1.61E-144
XM_020083583.1	PREDICTED: uncharacterized protein LOC109627158 isoform X10	776	3.77E-137
XM_020083584.1	centrosomal protein of 55 kDa-like isoform X1	3291	0
XM_020083585.1	centrosomal protein of 55 kDa-like isoform X2	3288	0
XM_020083586.1	centrosomal protein of 55 kDa-like isoform X3	3285	0
XM_020083587.1	centrosomal protein of 55 kDa-like isoform X4	3282	0
XM_020083588.1	dynein heavy chain 9	13736	0
XM_020083589.1	serine/threonine-protein kinase BRSK1-like isoform X1	6177	0
XM_020083590.1	serine/threonine-protein kinase BRSK2-like isoform X2	6162	0
XM_020083591.1	serine/threonine-protein kinase BRSK1-like isoform X3	6066	0
XM_020083592.1	tetratricopeptide repeat protein 9A-like	1187	3.85E-178
XM_020083593.1	serine/threonine-protein kinase BRSK1-like isoform X4	3886	0
XM_020083594.1	serine/threonine-protein kinase BRSK1-like isoform X5	3871	0
XM_020083595.1	serine/threonine-protein kinase BRSK2-like isoform X6	5829	0
XM_020083596.1	serine/threonine-protein kinase BRSK2-like isoform X7	5814	0
XM_020083597.1	serine/threonine-protein kinase BRSK2-like isoform X8	5718	0

XM_020083598.1	serine/threonine-protein kinase BRSK1-like isoform X1	6091	0
XM_020083599.1	serine/threonine-protein kinase BRSK1-like isoform X10	6051	0
XM_020083600.1	lutropin-choriogonadotropic hormone receptor-like	2796	0
XM_020083601.1	SAP30-binding protein isoform X1	1640	1.60E-157
XM_020083602.1	SAP30-binding protein isoform X2	1588	2.81E-143
XM_020083603.1	SAP30-binding protein isoform X3	1094	4.06E-171
XM_020083604.1	SAP30-binding protein isoform X4	1042	6.05E-157
XM_020083605.1	sodium-dependent neutral amino acid transporter B(0)AT2-like	3386	0
XM_020083606.1	pro-neuregulin-2, membrane-bound isoform-like	2466	0
XM_020083607.1	sodium-dependent neutral amino acid transporter B(0)AT2-like	3314	0
XM_020083608.1	sodium-dependent neutral amino acid transporter B(0)AT2-like	3276	0
XM_020083609.1	RNA binding protein fox-1 homolog 3-like	1352	0
XM_020083610.1	PREDICTED: midasin-like	1323	1.42E-70
XM_020083611.1	ferritin, middle subunit-like	1274	1.65E-126
XM_020083612.1	ferritin, middle subunit-like	1265	1.49E-126
XM_020083613.1	ferritin, middle subunit-like	1026	4.03E-128
XM_020083614.1	anthrax toxin receptor 1-like	5066	0
XM_020083615.1	cytochrome b-c1 complex subunit 2, mitochondrial isoform X1	1717	0
XM_020083616.1	cytochrome b-c1 complex subunit 2, mitochondrial isoform X2	1707	0
XM_020083617.1	DNA repair endonuclease XPF	3131	0
XM_020083618.1	histone acetyltransferase KAT7-like isoform X1	2744	0
XM_020083619.1	E3 ubiquitin-protein ligase TM129	1347	0
XM_020083620.1	transcriptional activator protein Pur-alpha-like	1440	0
XM_020083621.1	histone acetyltransferase KAT7-like isoform X1	2662	0
XM_020083622.1	pleckstrin homology domain-containing family A member 4 isoform X1	4658	0
XM_020083623.1	pleckstrin homology domain-containing family A member 4 isoform X1	4560	0
XM_020083624.1	pleckstrin homology domain-containing family A member 4 isoform X3	4508	0
XM_020083625.1	pleckstrin homology domain-containing family A member 4 isoform X4	3306	0
XM_020083626.1	mitochondrial import receptor subunit TOM22 homolog	1577	1.40E-45
XM_020083627.1	Fanconi anemia core complex-associated protein 100	3012	0
XM_020083628.1	baculoviral IAP repeat-containing protein 5.2-like	682	9.88E-89
XM_020083629.1	poly(ADP-ribose) glycohydrolase-like isoform X2	3488	0
XM_020083630.1	poly(ADP-ribose) glycohydrolase-like isoform X2	3344	0
XM_020083631.1	nuclear receptor-binding factor 2	1410	0
XM_020083632.1	probable serine/threonine-protein kinase kinX isoform X3	5507	0
XM_020083633.1	probable serine/threonine-protein kinase kinX isoform X3	5504	0
XM_020083634.1	probable serine/threonine-protein kinase kinX isoform X3	5495	0
XM_020083635.1	glutaryl-CoA dehydrogenase, mitochondrial isoform X1	2006	0
XM_020083636.1	glutaryl-CoA dehydrogenase, mitochondrial isoform X1	1949	0
XM_020083637.1	glutaryl-CoA dehydrogenase, mitochondrial isoform X1	1968	0
XM_020083638.1	glutaryl-CoA dehydrogenase, mitochondrial isoform X1	2005	0
XM_020083639.1	glutaryl-CoA dehydrogenase, mitochondrial isoform X1	1947	0
XM_020083640.1	PREDICTED: syntaxin-8	1476	4.46E-127
XM_020083641.1	PREDICTED: mucolipin-1-like	2444	0
XM_020083642.1	E3 ubiquitin-protein ligase NEURL1-like	4950	0
XM_020083643.1	probable serine/threonine-protein kinase kinX isoform X3	6007	0
XM_020083644.1	protein unc-93 homolog B1	3423	0
XM_020083645.1	protein unc-93 homolog B1	3410	0
XM_020083646.1	glutamate receptor 2-like	4302	0
XM_020083647.1	small ubiquitin-related modifier 2	849	1.66E-66
XM_020083648.1	endoplasmic reticulum-Golgi intermediate compartment protein 2-like	2275	0
XM_020083649.1	E3 SUMO-protein ligase CBX4	3098	0
XM_020083650.1	sialic acid synthase-like	1687	0
XM_020083651.1	phosphoinositide 3-kinase regulatory subunit 5 isoform X1	3777	0
XM_020083652.1	phosphoinositide 3-kinase regulatory subunit 5 isoform X2	3755	0
XM_020083653.1	probable serine/threonine-protein kinase kinX isoform X3	5963	0
XM_020083654.1	glutathione S-transferase theta-3-like	1375	4.51E-175
XM_020083655.1	guanine nucleotide-binding protein G(I)/G(S)/G(O) subunit gamma-10-like	1365	1.22E-34
XM_020083656.1	max-interacting protein 1 isoform X1	2175	0
XM_020083657.1	max-interacting protein 1 isoform X2	2285	7.73E-144
XM_020083658.1	transcription factor Sp8 isoform X2	3557	0
XM_020083659.1	tether containing UBX domain for GLUT4 isoform X1	2181	0
XM_020083660.1	tether containing UBX domain for GLUT4 isoform X2	2125	0
XM_020083661.1	nocturnin-like isoform X1	2064	0
XM_020083662.1	nocturnin-like isoform X1	1857	0
XM_020083663.1	F-box only protein 8	2733	0
XM_020083664.1	F-box only protein 8	2707	0
XM_020083665.1	flocculation protein FLO11-like isoform X1	4762	0
XM_020083666.1	NAD-dependent protein deacetylase sirtuin-7	2360	0
XM_020083667.1	protein O-GlcNAcase-like isoform X1	2438	0
XM_020083668.1	protein O-GlcNAcase-like isoform X2	2435	0
XM_020083669.1	hematological and neurological expressed 1 protein-like	2015	1.36E-80
XM_020083670.1	insulin-like growth factor-binding protein 4	4635	1.97E-171
XM_020083671.1	phosphomannomutase 2 isoform X1	1044	0
XM_020083672.1	phosphomannomutase 2 isoform X2	985	4.87E-172
XM_020083673.1	flocculation protein FLO11-like isoform X2	4645	0
XM_020083674.1	PREDICTED: uncharacterized protein LOC109627226 isoform X1	2655	0
XM_020083675.1	PREDICTED: uncharacterized protein LOC109627226 isoform X2	4032	0
XM_020083676.1	deoxycytidylate deaminase isoform X1	1426	2.54E-153

XM_020083677.1	deoxycytidylate deaminase isoform X2	1383	1.07E-141
XM_020083678.1	40S ribosomal protein S15a	460	2.04E-91
XM_020083679.1	40S ribosomal protein S15a	512	1.93E-91
XM_020083680.1	40S ribosomal protein S15a	522	7.58E-92
XM_020083681.1	40S ribosomal protein S15a	461	1.03E-91
XM_020083682.1	40S ribosomal protein S15a	460	7.94E-92
XM_020083683.1	sphingosine kinase 1-like	5173	0
XM_020083684.1	intraflagellar transport protein 56	2113	0
XM_020083685.1	multidrug and toxin extrusion protein 1 isoform X1	2707	0
XM_020083686.1	EH domain-containing protein 1-like	2190	0
XM_020083687.1	multidrug and toxin extrusion protein 1 isoform X2	2680	0
XM_020083688.1	high-affinity choline transporter 1-like	1970	0
XM_020083689.1	importin subunit alpha-1-like	1693	0
XM_020083690.1	neuronal acetylcholine receptor subunit beta-2-like isoform X2	1751	0
XM_020083691.1	platelet endothelial cell adhesion molecule-like isoform X1	2485	0
XM_020083692.1	platelet endothelial cell adhesion molecule-like isoform X2	2482	0
XM_020083693.1	platelet endothelial cell adhesion molecule-like isoform X3	2428	0
XM_020083694.1	platelet endothelial cell adhesion molecule-like isoform X4	2425	0
XM_020083695.1	platelet endothelial cell adhesion molecule-like isoform X5	2557	0
XM_020083696.1	platelet endothelial cell adhesion molecule-like isoform X6	2588	0
XM_020083697.1	PREDICTED: secernin-2	1909	0
XM_020083698.1	GPI ethanolamine phosphate transferase 3	7903	0
XM_020083699.1	solute carrier family 2, facilitated glucose transporter member 5-like isoform X1	2300	0
XM_020083700.1	solute carrier family 2, facilitated glucose transporter member 5-like isoform X2	2298	0
XM_020083701.1	sodium/potassium/calcium exchanger 3-like	4697	0
XM_020083702.1	transitional endoplasmic reticulum ATPase-like	4128	0
XM_020083703.1	multivesicular body subunit 12A	2057	0
XM_020083704.1	methylthioribose-1-phosphate isomerase	2215	0
XM_020083705.1	zona pellucida sperm-binding protein 4-like	3736	0
XM_020083706.1	zona pellucida sperm-binding protein 4-like	3634	0
XM_020083707.1	carboxypeptidase Z-like isoform X1	2807	0
XM_020083708.1	carboxypeptidase Z-like isoform X2	2768	0
XM_020083709.1	rhomboid-related protein 3 isoform X1	4532	0
XM_020083710.1	synaptopodin isoform X1	6424	0
XM_020083711.1	rhomboid-related protein 3 isoform X2	2030	0
XM_020083712.1	rhomboid-related protein 3 isoform X3	1136	0
XM_020083713.1	rhomboid-related protein 3 isoform X4	1871	0
XM_020083714.1	phosphoinositide 3-kinase regulatory subunit 6 isoform X1	3859	0
XM_020083715.1	phosphoinositide 3-kinase regulatory subunit 6 isoform X2	3856	0
XM_020083716.1	phosphoinositide 3-kinase regulatory subunit 6 isoform X3	3853	0
XM_020083717.1	phosphoinositide 3-kinase regulatory subunit 6 isoform X4	3850	0
XM_020083718.1	protein N-terminal asparagine amidohydrolase	2253	0
XM_020083719.1	synaptopodin isoform X1	6282	0
XM_020083720.1	DENN domain-containing protein 1B-like isoform X1	4261	0
XM_020083721.1	DENN domain-containing protein 1B-like isoform X2	4195	0
XM_020083722.1	basement membrane-specific heparan sulfate proteoglycan core protein-like	1718	0
XM_020083723.1	tripartite motif-containing protein 16-like	2042	0
XM_020083724.1	tumor suppressor candidate 3	1281	0
XM_020083725.1	chromobox protein homolog 8 isoform X1	2206	0
XM_020083726.1	chromobox protein homolog 8 isoform X2	1919	0
XM_020083727.1	sphingosine 1-phosphate receptor 1-like	2756	0
XM_020083728.1	synaptopodin isoform X1	5126	0
XM_020083729.1	von Willebrand factor A domain-containing protein 7-like	3771	0
XM_020083730.1	von Willebrand factor A domain-containing protein 7-like	3789	0
XM_020083731.1	von Willebrand factor A domain-containing protein 7-like	3250	0
XM_020083732.1	leucine-rich repeat and immunoglobulin-like domain-containing nogo receptor-interacting protein 2	3499	0
XM_020083733.1	prostaglandin D2 receptor 2-like	1457	0
XM_020083734.1	calcium uptake protein 3, mitochondrial isoform X1	2076	0
XM_020083735.1	calcium uptake protein 3, mitochondrial isoform X2	2070	0
XM_020083736.1	calcium uptake protein 3, mitochondrial isoform X3	2061	0
XM_020083737.1	calcium uptake protein 3, mitochondrial isoform X4	2055	0
XM_020083738.1	synaptopodin isoform X1	5075	0
XM_020083739.1	fibroblast growth factor 20-like	1573	8.21E-147
XM_020083740.1	transcription factor 15-like	2412	8.63E-121
XM_020083741.1	dead end protein 1-like	1291	0
XM_020083742.1	DNA repair protein complementing XP-A cells	1788	1.38E-165
XM_020083743.1	ecto-ADP-ribosyltransferase 5-like	853	0
XM_020083744.1	glutamate receptor ionotropic, NMDA 2B-like isoform X1	6220	0
XM_020083745.1	glutamate receptor ionotropic, NMDA 2B-like isoform X2	4630	0
XM_020083746.1	group XIIB secretory phospholipase A2-like protein	1168	5.44E-130
XM_020083747.1	metal transporter CNNM2	5312	0
XM_020083748.1	homeobox protein MSH-C-like	1804	2.36E-177
XM_020083749.1	E3 ubiquitin-protein ligase RNF38-like	2890	0
XM_020083750.1	PREDICTED: netrin-1	3092	0
XM_020083751.1	PREDICTED: netrin-1	3089	0
XM_020083752.1	protein Daple-like	6087	0
XM_020083753.1	neuronal pentraxin-1-like	2414	0
XM_020083754.1	serine hydrolase-like protein	581	4.02E-57
XM_020083755.1	reticulon-4 receptor-like 2	2394	0

XM_020083756.1	beta-crystallin B1-like	1093	3.53E-167
XM_020083757.1	meteorin-like protein	1678	0
XM_020083758.1	transcription factor MafG-like	3998	1.09E-85
XM_020083759.1	transcription factor MafG-like	4014	9.41E-86
XM_020083760.1	epsin-3-like isoform X1	3399	0
XM_020083761.1	fibrous sheath CABYR-binding protein-like isoform X1	3438	0
XM_020083762.1	epsin-3-like isoform X2	3396	0
XM_020083763.1	epsin-3-like isoform X3	3315	0
XM_020083764.1	epsin-3-like isoform X1	3130	0
XM_020083765.1	inositol 1,4,5-trisphosphate receptor-interacting protein-like	2053	0
XM_020083766.1	zona pellucida sperm-binding protein 3-like	1550	0
XM_020083767.1	D-dopachrome decarboxylase-like	683	1.52E-82
XM_020083768.1	mpv17-like protein	2475	3.50E-149
XM_020083769.1	CD276 antigen homolog isoform X1	2411	0
XM_020083770.1	CD276 antigen homolog isoform X2	2378	0
XM_020083771.1	arsenite methyltransferase	1516	0
XM_020083772.1	coiled-coil domain-containing protein 85A-like isoform X1	2687	0
XM_020083773.1	alpha-(1,3)-fucosyltransferase 11	2268	0
XM_020083774.1	coiled-coil domain-containing protein 85A-like isoform X2	2745	0
XM_020083775.1	coiled-coil domain-containing protein 85A-like isoform X3	3102	0
XM_020083776.1	atherin-like isoform X1	4190	0
XM_020083777.1	zinc finger protein OZF-like isoform X1	1946	0
XM_020083778.1	zinc finger protein OZF-like isoform X1	1965	0
XM_020083779.1	max-binding protein MNT-like isoform X1	3274	0
XM_020083780.1	max-binding protein MNT-like isoform X2	3271	0
XM_020083781.1	protocadherin-18-like isoform X1	2082	0
XM_020083782.1	FERM and PDZ domain-containing protein 1-like	8339	0
XM_020083783.1	scavenger receptor cysteine-rich domain-containing group B protein-like	3490	0
XM_020083784.1	N-acetylmuramoyl-L-alanine amidase isoform X1	2482	0
XM_020083785.1	N-acetylmuramoyl-L-alanine amidase isoform X1	2469	0
XM_020083786.1	inositol polyphosphate 5-phosphatase OCRL-1 isoform X1	5428	0
XM_020083787.1	neuron-specific protein family member 1-like isoform X1	2644	4.69E-106
XM_020083788.1	neuron-specific protein family member 1-like isoform X2	2641	3.51E-105
XM_020083789.1	nuclear factor interleukin-3-regulated protein-like	2102	0
XM_020083790.1	tensin-3-like isoform X2	3881	0
XM_020083791.1	WD repeat-containing protein 17	2293	0
XM_020083792.1	microtubule-associated tumor suppressor 1 isoform X1	4300	0
XM_020083793.1	inositol polyphosphate 5-phosphatase OCRL-1 isoform X2	5404	0
XM_020083794.1	protein PAT1 homolog 1-like	2758	0
XM_020083795.1	zinc finger protein 436-like	5724	0
XM_020083796.1	glycine receptor subunit beta-like	1980	0
XM_020083797.1	PREDICTED: teneurin-3-like	9746	0
XM_020083798.1	PREDICTED: teneurin-3-like	800	8.55E-131
XM_020083799.1	teleost multiple tissue opsin-3a	1014	0
XM_020083800.1	VPS10 domain-containing receptor SorCS1-like	6983	0
XM_020083801.1	inositol polyphosphate 5-phosphatase OCRL-1 isoform X3	5287	0
XM_020083802.1	collagen alpha-1(XVII) chain-like	4862	0
XM_020083803.1	rho GDP-dissociation inhibitor 1-like	2484	8.42E-127
XM_020083804.1	anaphase-promoting complex subunit 11	370	9.51E-30
XM_020083805.1	regulatory-associated protein of mTOR	6901	0
XM_020083806.1	coiled-coil domain-containing protein 40-like	2767	0
XM_020083807.1	PREDICTED: uncharacterized protein LOC109627315	651	3.66E-160
XM_020083808.1	inositol polyphosphate 5-phosphatase OCRL-1 isoform X1	5159	0

XM_020083809.1	skin secretory protein xP2-like	2235	5.20E-156
XM_020083810.1	solute carrier family 43 member 3-like	1579	0
XM_020083811.1	C-type lectin domain family 4 member F-like	2898	0
XM_020083812.1	zinc finger protein 2 homolog isoform X2	4262	0
XM_020083813.1	leucine-rich repeat and immunoglobulin-like domain-containing nogo receptor-interacting protein 2	3293	0
XM_020083814.1	dynein heavy chain 6, axonemal	11977	0
XM_020083815.1	alpha-2-macroglobulin receptor-associated protein	1861	0
XM_020083816.1	regulator of G-protein signaling 12-like	2784	0
XM_020083817.1	synaptonemal complex central element protein 2	599	1.44E-116
XM_020083818.1	xylosyltransferase 1	3152	0
XM_020083819.1	coiled-coil domain-containing protein 85A-like	1778	0
XM_020083820.1	ras GTPase-activating protein-binding protein 1	2642	0
XM_020083821.1	B-cell lymphoma/leukemia 11A-like	3291	0
XM_020083822.1	cep170-like protein	2666	0
XM_020083823.1	transcription factor LBX1-like	1467	8.61E-90
XM_020083824.1	N-sulphoglucosamine sulphohydrolase	1518	0
XM_020083825.1	endonuclease III-like protein 1	575	4.42E-104
XM_020083826.1	PREDICTED: tuberin-like	2606	0
XM_020083827.1	somatostatin receptor type 5-like	2120	0
XM_020083828.1	lysyl oxidase homolog 4-like	1906	0
XM_020083829.1	PREDICTED: uncharacterized protein LOC109627337	1069	3.03E-165
XM_020083830.1	eukaryotic peptide chain release factor subunit 1	2183	0
XM_020083831.1	polymeric immunoglobulin receptor	2434	0
XM_020083832.1	dynamin-2 isoform X1	3256	0
XM_020083833.1	adhesion G protein-coupled receptor L1-like	1114	1.71E-98
XM_020083834.1	nucleoredoxin-like protein 1	660	1.19E-113

XM_020083835.1	heme oxygenase 2 isoform X2	951	0
XM_020083836.1	PREDICTED: uncharacterized protein LOC109627343	3029	0
XM_020083837.1	growth arrest-specific protein 8-like	2484	0
XM_020083838.1	stimulated by retinoic acid gene 6 protein-like	1746	0
XM_020083839.1	galactosylceramide sulfotransferase-like	1474	0
XM_020083840.1	PREDICTED: plastin-3-like	4103	0
XM_020083841.1	ecto-ADP-ribosyltransferase 5-like	870	0
XM_020083842.1	E3 ubiquitin-protein ligase SMURF2	4037	0
XM_020083843.1	(E3-independent) E2 ubiquitin-conjugating enzyme UBE2O-like	1114	0
XM_020083844.1	(E3-independent) E2 ubiquitin-conjugating enzyme-like	3861	0
XM_020083845.1	serine/threonine-protein kinase N1-like	3688	0
XM_020083846.1	flocculation protein FLO11-like	1223	1.02E-92
XM_020083847.1	zinc finger protein Gfi-1-like	732	4.23E-171
XM_020083848.1	U3 small nucleolar RNA-associated protein 6 homolog	2296	0
XM_020083849.1	PREDICTED: uncharacterized protein LOC109627358	1578	0
XM_020083850.1	PREDICTED: uncharacterized protein MISP3	3505	0
XM_020083851.1	iduronate 2-sulfatase	3813	0
XM_020083852.1	PREDICTED: paralemmin-3	2796	0
XM_020083853.1	ras-related protein Rab-3A-like	708	6.13E-151
XM_020083854.1	GTPase IMAP family member 4-like	1379	0
XM_020083855.1	zinc finger protein 638-like isoform X1	5859	0
XM_020083856.1	zinc finger protein 638-like isoform X2	5847	0
XM_020083857.1	zinc finger protein 638-like isoform X1	5543	0
XM_020083858.1	lysine-specific demethylase 2A-like isoform X1	5155	0
XM_020083859.1	lysine-specific demethylase 2A-like isoform X2	5152	0
XM_020083860.1	lysine-specific demethylase 2A-like isoform X3	4602	0
XM_020083861.1	lysine-specific demethylase 2A-like isoform X4	5402	0
XM_020083862.1	sarcoplasmic/endoplasmic reticulum calcium ATPase 2-like	6841	0
XM_020083863.1	m7GpppX diphosphatase	1225	0
XM_020083864.1	palmitoyltransferase ZDHHC5-like isoform X1	6403	0
XM_020083865.1	palmitoyltransferase ZDHHC5-like isoform X1	4680	0
XM_020083866.1	palmitoyltransferase ZDHHC5-like isoform X1	5055	0
XM_020083867.1	E3 ubiquitin-protein ligase DTX1-like isoform X1	6657	0
XM_020083868.1	E3 ubiquitin-protein ligase DTX1-like isoform X1	6495	0
XM_020083869.1	N-acetylgalactosaminyltransferase 7 isoform X1	5000	0
XM_020083870.1	N-acetylgalactosaminyltransferase 7 isoform X2	5016	0
XM_020083871.1	N-acetylgalactosaminyltransferase 7 isoform X1	5002	0
XM_020083872.1	E3 ubiquitin-protein ligase TRIM39-like	3216	0
XM_020083873.1	large neutral amino acids transporter small subunit 4-like isoform X1	5306	0
XM_020083874.1	ribosomal protein S6 kinase beta-2-like	3211	0
XM_020083875.1	cleavage and polyadenylation specificity factor subunit 6-like	3801	0
XM_020083876.1	F-box/WD repeat-containing protein 11 isoform X1	3057	0
XM_020083877.1	vascular endothelial growth factor C	1848	0
XM_020083878.1	H/ACA ribonucleoprotein complex non-core subunit NAF1	2649	2.81E-172
XM_020083879.1	zinc finger protein 239-like	2480	0
XM_020083880.1	store-operated calcium entry-associated regulatory factor	1251	0
XM_020083881.1	vacuolar protein sorting-associated protein 37C	4302	3.08E-112
XM_020083882.1	vacuolar protein sorting-associated protein 37C	3922	2.13E-111
XM_020083883.1	ankyrin repeat and SOCS box protein 5-like	2158	0
XM_020083884.1	F-box/WD repeat-containing protein 11 isoform X1	3190	0
XM_020083885.1	thioredoxin-related transmembrane protein 2-B	2618	0
XM_020083886.1	syntaxin-3-like isoform X1	912	0
XM_020083887.1	PREDICTED: uncharacterized protein LOC109627383	1236	6.41E-166
XM_020083888.1	transmembrane protein 187	2186	0
XM_020083889.1	PREDICTED: uncharacterized protein LOC109627385 isoform X1	2183	2.77E-153
XM_020083890.1	PREDICTED: uncharacterized protein LOC109627385 isoform X2	2066	8.60E-123
XM_020083891.1	transmembrane protein 109-like	4028	2.82E-133
XM_020083892.1	signal peptidase complex subunit 3	841	6.50E-123
XM_020083893.1	UPF0687 protein C20orf27 homolog isoform X1	2453	2.71E-107
XM_020083894.1	UPF0687 protein C20orf27 homolog isoform X1	2578	6.88E-107
XM_020083895.1	PREDICTED: teneurin-3-like	10338	0
XM_020083896.1	UPF0687 protein C20orf27 homolog isoform X1	2524	4.65E-107
XM_020083897.1	UPF0687 protein C20orf27 homolog isoform X1	2450	1.66E-106
XM_020083898.1	DNA polymerase delta subunit 4	832	1.62E-60
XM_020083899.1	envoplakin-like protein	6518	0
XM_020083900.1	(E3-independent) E2 ubiquitin-conjugating enzyme	3407	0
XM_020083901.1	inactive rhomboid protein 2	5026	0
XM_020083902.1	inactive rhomboid protein 2	4756	0
XM_020083903.1	peroxisomal acyl-coenzyme A oxidase 1 isoform X1	2529	0
XM_020083904.1	peroxisomal acyl-coenzyme A oxidase 1 isoform X2	2529	0
XM_020083905.1	DEAD (Asp-Glu-Ala-Asp) box helicase 5-1a	2447	0
XM_020083906.1	histone-lysine N-methyltransferase SETD7 isoform X1	2335	0
XM_020083907.1	signal recognition particle subunit SRP68	3877	0
XM_020083908.1	DNA polymerase subunit gamma-2, mitochondrial	1898	0
XM_020083909.1	DNA polymerase subunit gamma-2, mitochondrial	1828	0
XM_020083910.1	serotonin N-acetyltransferase-like	1511	7.00E-145
XM_020083911.1	CST complex subunit TEN1	756	1.18E-94
XM_020083912.1	pre-mRNA-splicing factor CWC25 homolog	1778	1.01E-180
XM_020083913.1	histone-lysine N-methyltransferase SETD7 isoform X2	2486	0

XM_020083914.1	ribonuclease H2 subunit A isoform X1	1236	0
XM_020083915.1	ribonuclease H2 subunit A isoform X2	1246	0
XM_020083916.1	ribonuclease H2 subunit A isoform X1	1243	0
XM_020083917.1	ATP-sensitive inward rectifier potassium channel 12-like	2525	0
XM_020083918.1	tartrate-resistant acid phosphatase type 5	1273	0
XM_020083919.1	5-demethoxyubiquinone hydroxylase, mitochondrial	1845	3.36E-161
XM_020083920.1	PREDICTED: uncharacterized protein LOC109627407	1835	5.09E-116
XM_020083921.1	ATP synthase subunit d, mitochondrial	650	4.38E-117
XM_020083922.1	lisH domain-containing protein FOPNL	873	2.40E-101
XM_020083923.1	UDP-GlcNAc:betaGal beta-1,3-N-acetylglucosaminyltransferase-like protein 1	2211	0
XM_020083924.1	centrosomal protein of 44 kDa isoform X1	1513	0
XM_020083925.1	centrosomal protein of 44 kDa isoform X2	1438	0
XM_020083926.1	coiled-coil-helix-coiled-coil-helix domain-containing protein 10, mitochondrial	831	9.69E-26
XM_020083927.1	N-acetylaspartate synthetase-like isoform X1	1878	0
XM_020083928.1	short coiled-coil protein B-like	1353	1.49E-38
XM_020083929.1	N-acetylaspartate synthetase-like isoform X2	1687	5.97E-141
XM_020083930.1	goose-type lysozyme	759	1.68E-144
XM_020083931.1	ecto-ADP-ribosyltransferase 5-like isoform X2	1224	0
XM_020083932.1	ecto-ADP-ribosyltransferase 5-like isoform X2	1192	0
XM_020083933.1	ecto-ADP-ribosyltransferase 5-like isoform X2	1157	0
XM_020083934.1	VPS10 domain-containing receptor SorCS3-like isoform X1	4348	0
XM_020083935.1	VPS10 domain-containing receptor SorCS3-like isoform X2	4248	0
XM_020083936.1	alpha-2A adrenergic receptor	2096	0
XM_020083937.1	short coiled-coil protein B-like	1298	2.11E-43
XM_020083938.1	germ cell-specific gene 1-like protein	1638	0
XM_020083939.1	volume-regulated anion channel subunit LRRC8A	2998	0
XM_020083940.1	volume-regulated anion channel subunit LRRC8A	2948	0
XM_020083941.1	NADH dehydrogenase [ubiquinone] 1 beta subcomplex subunit 7	618	9.36E-72
XM_020083942.1	fibroblast growth factor 8	1817	1.94E-141
XM_020083943.1	cilia- and flagella-associated protein 52	2184	0
XM_020083944.1	non-structural maintenance of chromosomes element 1 homolog isoform X1	945	4.34E-175
XM_020083945.1	non-structural maintenance of chromosomes element 1 homolog isoform X1	1201	8.19E-173
XM_020083946.1	beta-2-glycoprotein 1-like	1918	0
XM_020083947.1	TBC1 domain family member 24-like	3185	0
XM_020083948.1	CREB-binding protein-like isoform X1	8638	0
XM_020083949.1	ubiquitin-conjugating enzyme E2 D2	2033	1.54E-102
XM_020083950.1	CREB-binding protein-like isoform X2	9472	0
XM_020083951.1	CREB-binding protein-like isoform X3	9168	0
XM_020083952.1	adenylate cyclase type 9-like	7679	0
XM_020083953.1	PREDICTED: uncharacterized protein LOC109627433	1763	0
XM_020083954.1	phospholipid phosphatase 3-like	1978	1.84E-174
XM_020083955.1	serine protease HTRA3-like isoform X1	1778	0
XM_020083956.1	serine protease HTRA3-like isoform X2	1775	0
XM_020083957.1	serine protease HTRA3-like isoform X3	1754	0
XM_020083958.1	serine protease HTRA3-like isoform X4	1751	0
XM_020083959.1	caspase recruitment domain-containing protein 14	4277	0
XM_020083960.1	histamine H2 receptor-like	2069	0
XM_020083961.1	sialic acid synthase-like	2090	0
XM_020083962.1	tektin-4 isoform X1	1635	0
XM_020083963.1	tektin-4 isoform X2	1632	0
XM_020083964.1	regulator of G-protein signaling 14-like isoform X1	2687	0
XM_020083965.1	glucagon receptor-like	3312	0
XM_020083966.1	tubulin polymerization-promoting protein family member 3-like	1452	2.69E-105
XM_020083967.1	prostaglandin E2 receptor EP1 subtype-like	2057	0
XM_020083968.1	MYCBP-associated protein	2488	0
XM_020083969.1	transmembrane channel-like protein 5	3376	0
XM_020083970.1	voltage-dependent calcium channel gamma-4 subunit-like	2090	0
XM_020083971.1	T-cell leukemia homeobox protein 3	2628	6.89E-153
XM_020083972.1	PREDICTED: uncharacterized protein LOC109627448	1645	6.56E-145
XM_020083973.1	neuropeptide B	918	4.88E-91
XM_020083974.1	beta-crystallin A1-like	946	5.09E-147
XM_020083975.1	endonuclease V isoform X1	991	0
XM_020083976.1	endonuclease V isoform X2	987	0
XM_020083977.1	beta-2-glycoprotein 1-like	1366	0
XM_020083978.1	DAN related protein	1288	0
XM_020083979.1	PREDICTED: uncharacterized protein C17orf67 homolog	734	2.61E-52
XM_020083980.1	Kv channel-interacting protein 1 isoform X1	1304	1.89E-172
XM_020083981.1	Krueppel-like factor 1	1323	0
XM_020083982.1	retinal cone rhodopsin-sensitive cGMP 3',5'-cyclic phosphodiesterase subunit gamma-like	749	1.51E-35
XM_020083983.1	retinal cone rhodopsin-sensitive cGMP 3',5'-cyclic phosphodiesterase subunit gamma-like	340	1.10E-37
XM_020083984.1	PREDICTED: uncharacterized protein LOC109627459	1986	2.54E-164
XM_020083985.1	GTP-binding protein Rhes-like	1539	6.25E-137
XM_020083986.1	UPF0450 protein C17orf58 homolog	2481	0
XM_020083987.1	ecto-ADP-ribosyltransferase 5-like isoform X2	1440	0
XM_020083988.1	Kv channel-interacting protein 1 isoform X2	1068	7.42E-174
XM_020083989.1	forkhead box protein D1-like	1187	0
XM_020083990.1	PREDICTED: tetraspanin-1-like	976	8.20E-124
XM_020083991.1	PREDICTED: uncharacterized protein LOC109627465 isoform X1	1244	7.75E-152
XM_020083992.1	PREDICTED: uncharacterized protein LOC109627465 isoform X2	1232	5.87E-149

XM_020083993.1	PREDICTED: uncharacterized protein LOC109627465 isoform X3	1150	4.76E-115
XM_020083994.1	osteocalcin 2-like	1244	1.10E-20
XM_020083995.1	solute carrier family 43 member 3-like	2192	0
XM_020083996.1	Kv channel-interacting protein 1 isoform X3	928	3.30E-163
XM_020083997.1	calcium-binding protein 2-like	839	4.75E-118
XM_020083998.1	keratin, type I cytoskeletal 18-like	2150	0
XM_020083999.1	protein phosphatase 1 regulatory subunit 27	1609	3.43E-119
XM_020084000.1	voltage-dependent calcium channel gamma-5 subunit	1915	4.92E-177
XM_020084001.1	PREDICTED: zeta-sarcoglycan-like	1935	0
XM_020084002.1	PREDICTED: uncharacterized protein C16orf45 homolog	1522	1.56E-133
XM_020084003.1	UPF0575 protein C19orf67 homolog	1427	0
XM_020084004.1	Kv channel-interacting protein 1 isoform X4	1213	3.19E-161
XM_020084005.1	sodium/potassium/calcium exchanger 2 isoform X1	1875	0
XM_020084006.1	run domain Beclin-1-interacting and cysteine-rich domain-containing protein	2235	0
XM_020084007.1	inactive phospholipase D5-like	1818	0
XM_020084008.1	trace amine-associated receptor 13c-like	996	0
XM_020084009.1	dexamethasone-induced Ras-related protein 1-like	1299	0
XM_020084010.1	myeloid-associated differentiation marker-like protein 2	1226	0
XM_020084011.1	G-protein coupled receptor 26-like	1757	0
XM_020084012.1	carboxylesterase notum2-like	1497	0
XM_020084013.1	PREDICTED: uncharacterized protein LOC109627484 isoform X1	853	3.42E-165
XM_020084014.1	PREDICTED: uncharacterized protein LOC109627484 isoform X2	786	7.74E-155
XM_020084015.1	trace amine-associated receptor 13c-like	984	0
XM_020084016.1	myeloid-associated differentiation marker homolog	1420	0
XM_020084017.1	ecto-ADP-ribosyltransferase 5-like	955	0
XM_020084018.1	endoplasmic reticulum resident protein 27	871	4.84E-180
XM_020084019.1	cysteine-rich protein 1-like	349	1.57E-44
XM_020084020.1	PREDICTED: ependymin-like	1070	5.89E-149
XM_020084021.1	N-alpha-acetyltransferase 15, NatA auxiliary subunit-like	3310	0
XM_020084022.1	stromal membrane-associated protein 1-like	2074	0
XM_020084023.1	keratin, type I cytoskeletal 13-like	1888	0
XM_020084024.1	keratin, type I cytoskeletal 13-like	1471	0
XM_020084025.1	keratin, type I cytoskeletal 13-like	1523	0
XM_020084026.1	keratin, type I cytoskeletal 13-like	1293	0
XM_020084027.1	centromere protein K	1378	4.04E-158
XM_020084028.1	complement C1q-like protein 2	826	6.56E-172
XM_020084029.1	melanin-concentrating hormone receptor 1	1740	0
XM_020084030.1	glutamate receptor 3 isoform X1	4687	0
XM_020084031.1	gastrin/cholecystokinin-like peptide	496	1.87E-57
XM_020084032.1	calcium homeostasis modulator protein 1-like	1059	0
XM_020084033.1	regulator of G-protein signaling 14-like isoform X2	2666	0
XM_020084034.1	transcription factor Sp2-like	3094	0
XM_020084035.1	zinc finger protein 260-like isoform X1	2170	0
XM_020084036.1	zinc finger protein 180-like isoform X2	1972	0
XM_020084037.1	protein RRP5 homolog	5943	0
XM_020084038.1	glutamate receptor 3 isoform X2	4687	0
XM_020084039.1	transcription initiation factor TFIID subunit 5	2670	0
XM_020084040.1	RING finger protein 122-like isoform X2	2234	3.09E-96
XM_020084041.1	up-regulated during skeletal muscle growth protein 5	427	3.01E-36
XM_020084042.1	up-regulated during skeletal muscle growth protein 5	421	2.80E-36
XM_020084043.1	RAS protein activator like-3-like isoform X1	3917	0
XM_020084044.1	disabled homolog 2-interacting protein isoform X5	3043	0
XM_020084045.1	E3 ubiquitin/ISG15 ligase TRIM25 isoform X1	2421	0
XM_020084046.1	E3 ubiquitin/ISG15 ligase TRIM25 isoform X1	2243	0
XM_020084047.1	E3 ubiquitin/ISG15 ligase TRIM25 isoform X1	2333	0
XM_020084048.1	E3 ubiquitin/ISG15 ligase TRIM25 isoform X1	2286	0
XM_020084049.1	E3 ubiquitin/ISG15 ligase TRIM25 isoform X4	2222	0
XM_020084050.1	diacylglycerol kinase epsilon	5856	0
XM_020084051.1	proline-rich basic protein 1	8992	0
XM_020084052.1	E3 ubiquitin/ISG15 ligase TRIM25-like	2825	0
XM_020084053.1	myosin-9-like isoform X1	6878	0
XM_020084054.1	myosin-9-like isoform X2	6857	0
XM_020084055.1	dual specificity mitogen-activated protein kinase kinase 7 isoform X1	8601	0
XM_020084056.1	dual specificity mitogen-activated protein kinase kinase 7 isoform X2	8553	0
XM_020084057.1	peroxisome proliferator-activated receptor gamma coactivator-related protein 1-like isoform X1	3857	0
XM_020084058.1	peroxisome proliferator-activated receptor gamma coactivator-related protein 1-like isoform X1	3854	0
XM_020084059.1	LIM domain-binding protein 1 isoform X2	3376	0
XM_020084060.1	proline-rich basic protein 1	9028	0
XM_020084061.1	LIM domain-binding protein 1 isoform X2	3454	0
XM_020084062.1	LIM domain-binding protein 1 isoform X2	3414	0
XM_020084063.1	LIM domain-binding protein 1 isoform X2	3766	0
XM_020084064.1	LIM domain-binding protein 1 isoform X2	3239	0
XM_020084065.1	LIM domain-binding protein 1 isoform X2	3347	0
XM_020084066.1	meiosis arrest female protein 1 isoform X1	8339	0
XM_020084067.1	meiosis arrest female protein 1 isoform X2	8336	0
XM_020084068.1	meiosis arrest female protein 1 isoform X3	7820	0
XM_020084069.1	meiosis arrest female protein 1 isoform X4	7664	0
XM_020084070.1	meiosis arrest female protein 1 isoform X4	7170	0
XM_020084071.1	pannexin-1 isoform X1	3023	0

XM_020084072.1	pannexin-1 isoform X1	2879	0
XM_020084073.1	pannexin-1 isoform X1	3023	0
XM_020084074.1	fibroblast growth factor receptor-like 1	6100	0
XM_020084075.1	PREDICTED: uncharacterized protein C16orf45 homolog	2012	1.49E-116
XM_020084076.1	far upstream element-binding protein 2-like	2486	0
XM_020084077.1	DDB1- and CUL4-associated factor 15	2779	0
XM_020084078.1	MHC class II regulatory factor RFX1 isoform X1	4330	0
XM_020084079.1	MHC class II regulatory factor RFX1 isoform X1	4268	0
XM_020084080.1	MHC class II regulatory factor RFX1 isoform X1	4230	0
XM_020084081.1	MHC class II regulatory factor RFX1 isoform X1	4327	0
XM_020084082.1	MHC class II regulatory factor RFX1 isoform X1	4327	0
XM_020084083.1	MHC class II regulatory factor RFX1 isoform X1	4237	0
XM_020084084.1	relaxin-3-like isoform X1	686	5.24E-110
XM_020084085.1	ATP-binding cassette sub-family A member 1 isoform X1	9550	0
XM_020084086.1	ATP-binding cassette sub-family A member 1 isoform X2	9541	0
XM_020084087.1	ras-related protein Rab-33B	1300	3.84E-165
XM_020084088.1	probable pancreatic secretory proteinase inhibitor	521	4.08E-51
XM_020084089.1	myosin-11 isoform X2	5513	0
XM_020084090.1	myosin-11 isoform X2	8233	0
XM_020084091.1	myosin-11 isoform X2	8206	0
XM_020084092.1	nuclear distribution protein nudE homolog 1-like	2633	0
XM_020084093.1	leucine-rich repeat protein SHOC-2	4141	0
XM_020084094.1	programmed cell death protein 4	2231	0
XM_020084095.1	N-alpha-acetyltransferase 15, NatA auxiliary subunit	4270	0
XM_020084096.1	ras-related protein Rab-33B-like	2134	2.82E-163
XM_020084097.1	WW domain binding protein 1-like isoform X1	4378	0
XM_020084098.1	WW domain binding protein 1-like isoform X2	4193	0
XM_020084099.1	PREDICTED: uncharacterized protein LOC109627538 isoform X1	1328	6.80E-151
XM_020084100.1	DNA polymerase lambda	2657	0
XM_020084101.1	protein DPCD	1674	1.31E-159
XM_020084102.1	coiled-coil and C2 domain-containing protein 1A	5118	0
XM_020084103.1	PREDICTED: uncharacterized protein LOC109627545 isoform X1	2776	0
XM_020084104.1	PREDICTED: uncharacterized protein LOC109627545 isoform X1	2738	0
XM_020084105.1	PREDICTED: uncharacterized protein LOC109627545 isoform X1	2777	0
XM_020084106.1	PREDICTED: uncharacterized protein LOC109627545 isoform X2	2746	0
XM_020084107.1	PREDICTED: uncharacterized protein LOC109627545 isoform X3	2734	0
XM_020084108.1	PREDICTED: uncharacterized protein LOC109627538 isoform X2	1202	3.21E-116
XM_020084109.1	afadin- and alpha-actinin-binding protein-like isoform X1	1574	0
XM_020084110.1	afadin- and alpha-actinin-binding protein-like isoform X1	1557	0
XM_020084111.1	afadin- and alpha-actinin-binding protein-like isoform X1	1512	0
XM_020084112.1	nanos homolog 3	1682	3.07E-151
XM_020084113.1	nucleosome-remodeling factor subunit BPTF isoform X1	11226	0
XM_020084114.1	nucleosome-remodeling factor subunit BPTF isoform X2	11220	0
XM_020084115.1	nucleosome-remodeling factor subunit BPTF isoform X3	11202	0
XM_020084116.1	nucleosome-remodeling factor subunit BPTF isoform X4	9982	0
XM_020084117.1	nucleosome-remodeling factor subunit BPTF isoform X5	11196	0
XM_020084118.1	nucleosome-remodeling factor subunit BPTF isoform X6	11175	0
XM_020084119.1	nucleosome-remodeling factor subunit BPTF isoform X7	11046	0
XM_020084120.1	nucleosome-remodeling factor subunit BPTF isoform X8	11043	0
XM_020084121.1	huntingtin isoform X1	12471	0
XM_020084122.1	regulator of G-protein signaling 14-like isoform X3	2591	0
XM_020084123.1	rho guanine nucleotide exchange factor 37	5967	0
XM_020084124.1	huntingtin isoform X2	12469	0
XM_020084125.1	huntingtin isoform X3	12459	0
XM_020084126.1	huntingtin isoform X4	12423	0
XM_020084127.1	huntingtin isoform X5	12075	0
XM_020084128.1	huntingtin isoform X6	12361	0
XM_020084129.1	huntingtin isoform X7	12358	0
XM_020084130.1	huntingtin isoform X8	12457	0
XM_020084131.1	huntingtin isoform X9	12346	0
XM_020084132.1	gamma-adducin-like isoform X1	5010	0
XM_020084133.1	rho guanine nucleotide exchange factor 37	5887	0
XM_020084134.1	gamma-adducin-like isoform X1	5095	0
XM_020084135.1	gamma-adducin-like isoform X1	5190	0
XM_020084136.1	gamma-adducin-like isoform X1	4972	0
XM_020084137.1	gamma-adducin-like isoform X1	4986	0
XM_020084138.1	gamma-adducin-like isoform X1	4917	0
XM_020084139.1	gamma-adducin-like isoform X4	4893	0
XM_020084140.1	GTPase IMAP family member 4-like isoform X1	2096	2.90E-176
XM_020084141.1	GTPase IMAP family member 4-like isoform X2	2081	1.29E-172
XM_020084142.1	claudin-6-like isoform X3	1859	2.00E-91
XM_020084143.1	rho guanine nucleotide exchange factor 37	5834	0
XM_020084144.1	mitochondrial dicarboxylate carrier	2947	0
XM_020084145.1	GTPase IMAP family member 4-like	1005	5.78E-175
XM_020084146.1	polycystic kidney disease protein 1-like 2	17261	0
XM_020084147.1	integrin alpha-M-like	5774	0
XM_020084148.1	protein phosphatase 1 regulatory subunit 12C isoform X1	3445	0
XM_020084149.1	protein phosphatase 1 regulatory subunit 12C isoform X2	3436	0
XM_020084150.1	nicotinate-nucleotide pyrophosphorylase [carboxylating]	1029	0

XM_020084151.1	nicotinate-nucleotide pyrophosphorylase [carboxylating]	1008	0
XM_020084152.1	PREDICTED: uncharacterized protein LOC109627559 isoform X1	3385	0
XM_020084153.1	PREDICTED: uncharacterized protein LOC109627559 isoform X2	3361	0
XM_020084154.1	PREDICTED: uncharacterized protein LOC109627559 isoform X3	3250	0
XM_020084155.1	CD97 antigen-like	4741	0
XM_020084156.1	rho guanine nucleotide exchange factor 37	5964	0
XM_020084157.1	long-chain fatty acid transport protein 1-like	2396	0
XM_020084158.1	procollagen galactosyltransferase 1	3341	0
XM_020084159.1	nucleoredoxin-like protein 1	702	4.07E-138
XM_020084160.1	myosin-9-like isoform X1	9133	0
XM_020084161.1	tubulin beta-1 chain-like	1881	0
XM_020084162.1	ADP-ribosylation factor-binding protein GGA1-like isoform X2	2840	0
XM_020084163.1	probable ATP-dependent RNA helicase DDX17	4623	0
XM_020084164.1	nodal modulator 1-like	7636	0
XM_020084165.1	myoneurin-like isoform X3	5782	0
XM_020084166.1	myoneurin-like isoform X3	5719	0
XM_020084167.1	proton-coupled amino acid transporter 1-like	3562	0
XM_020084168.1	myoneurin-like isoform X3	2857	0
XM_020084169.1	PREDICTED: uncharacterized protein LOC109627570 isoform X4	851	1.31E-131
XM_020084170.1	high affinity choline transporter 1-like	4678	0
XM_020084171.1	THO complex subunit 4-like	1312	6.13E-134
XM_020084172.1	phosphatidylinositol 5-phosphate 4-kinase type-2 beta	2805	0
XM_020084173.1	proteasome subunit beta type-3	825	1.25E-153
XM_020084174.1	nuclear factor 1 X-type isoform X1	6732	0
XM_020084175.1	nuclear factor 1 X-type isoform X2	6688	0
XM_020084176.1	nuclear factor 1 X-type isoform X1	6564	0
XM_020084177.1	nuclear factor 1 X-type isoform X4	6737	0
XM_020084178.1	nuclear factor 1 X-type isoform X5	6622	0
XM_020084179.1	nuclear factor 1 X-type isoform X6	6630	0
XM_020084180.1	nuclear factor 1 X-type isoform X7	6564	0
XM_020084181.1	PH and SEC7 domain-containing protein 3-like	4625	0
XM_020084182.1	nuclear factor 1 X-type isoform X8	6576	0
XM_020084183.1	nuclear factor 1 X-type isoform X9	6487	0
XM_020084184.1	nuclear factor 1 X-type isoform X10	6447	0
XM_020084185.1	nuclear factor 1 X-type isoform X11	6411	0
XM_020084186.1	nuclear factor 1 X-type isoform X12	6540	0
XM_020084187.1	structure-specific endonuclease subunit SLX4	6364	0
XM_020084188.1	structure-specific endonuclease subunit SLX4	6290	0
XM_020084189.1	MAPK regulated corepressor interacting protein 2-like	1322	8.48E-102
XM_020084190.1	PH and SEC7 domain-containing protein 3-like	1971	0
XM_020084191.1	serine/threonine-protein kinase 10-like	4979	0
XM_020084192.1	CST complex subunit STN1	1574	0
XM_020084193.1	activating transcription factor 7-interacting protein 1-like isoform X1	4041	0
XM_020084194.1	activating transcription factor 7-interacting protein 1-like isoform X1	4052	0
XM_020084195.1	activating transcription factor 7-interacting protein 1-like isoform X1	3390	0
XM_020084196.1	activating transcription factor 7-interacting protein 1-like isoform X1	4064	0
XM_020084197.1	activating transcription factor 7-interacting protein 1-like isoform X1	3851	0
XM_020084198.1	trinucleotide repeat-containing gene 6C protein-like isoform X1	12296	0
XM_020084199.1	trinucleotide repeat-containing gene 6C protein-like isoform X2	12158	0
XM_020084200.1	trinucleotide repeat-containing gene 6C protein-like isoform X1	11903	0
XM_020084201.1	NK-tumor recognition protein isoform X4	4852	0
XM_020084202.1	NK-tumor recognition protein isoform X4	4832	0
XM_020084203.1	NK-tumor recognition protein isoform X4	4828	0
XM_020084204.1	long-chain-fatty-acid--CoA ligase 1-like isoform X1	3565	0
XM_020084205.1	NK-tumor recognition protein isoform X4	4615	0
XM_020084206.1	vesicle-trafficking protein SEC22c	1820	0
XM_020084207.1	PREDICTED: cytoglobin-2	756	1.90E-132
XM_020084208.1	structural maintenance of chromosomes protein 3	4005	0
XM_020084209.1	ral GTPase-activating protein subunit alpha-2 isoform X2	2867	0
XM_020084210.1	testis-expressed sequence 2 protein-like isoform X1	5354	0
XM_020084211.1	testis-expressed sequence 2 protein-like isoform X1	5351	0
XM_020084212.1	testis-expressed sequence 2 protein-like isoform X1	5348	0
XM_020084213.1	regulator of G-protein signaling 14-like isoform X4	2524	0
XM_020084214.1	long-chain-fatty-acid--CoA ligase 1-like isoform X1	2388	0
XM_020084215.1	regulating synaptic membrane exocytosis protein 2-like isoform X1	4832	0
XM_020084216.1	regulating synaptic membrane exocytosis protein 1-like isoform X2	4769	0
XM_020084217.1	regulating synaptic membrane exocytosis protein 2-like isoform X3	4679	0
XM_020084218.1	regulating synaptic membrane exocytosis protein 1-like isoform X4	8007	0
XM_020084219.1	keratin, type I cytoskeletal 13-like	2232	0
XM_020084220.1	SEC14-like protein 1 isoform X1	5341	0
XM_020084221.1	SEC14-like protein 1 isoform X2	5329	0
XM_020084222.1	SEC14-like protein 1 isoform X3	5314	0
XM_020084223.1	long-chain-fatty-acid--CoA ligase 1-like isoform X1	3565	0
XM_020084224.1	SEC14-like protein 1 isoform X4	5300	0
XM_020084225.1	protein disulfide-isomerase	3226	0
XM_020084226.1	RNA binding protein fox-1 homolog 2-like isoform X1	5767	0
XM_020084227.1	RNA binding protein fox-1 homolog 2-like isoform X2	5764	0
XM_020084228.1	RNA binding protein fox-1 homolog 2-like isoform X3	5758	0
XM_020084229.1	RNA binding protein fox-1 homolog 2-like isoform X4	2574	0

XM_020084230.1	RNA binding protein fox-1 homolog 2-like isoform X5	4395	2.88E-143
XM_020084231.1	neuronal-specific septin-3-like isoform X1	3812	0
XM_020084232.1	long-chain-fatty-acid--CoA ligase 1-like isoform X1	2427	0
XM_020084233.1	neuronal-specific septin-3-like isoform X2	3696	0
XM_020084234.1	neuronal-specific septin-3-like isoform X3	3931	0
XM_020084235.1	neuronal-specific septin-3-like isoform X2	3599	0
XM_020084236.1	neuronal-specific septin-3-like isoform X3	3106	0
XM_020084237.1	neuronal-specific septin-3-like isoform X3	2932	0
XM_020084238.1	transcription activator BRG1-like isoform X1	7163	0
XM_020084239.1	transcription activator BRG1-like isoform X2	7160	0
XM_020084240.1	transcription activator BRG1-like isoform X3	7130	0
XM_020084241.1	transcription activator BRG1-like isoform X4	7127	0
XM_020084242.1	transcription activator BRG1-like isoform X1	4439	0
XM_020084243.1	cysteine protease ATG4D	2930	0
XM_020084244.1	cysteine protease ATG4D	2925	0
XM_020084245.1	complement C3-like	5270	0
XM_020084246.1	actin filament-associated protein 1-like 1 isoform X1	3300	0
XM_020084247.1	transcription factor jun-B-like	1482	0
XM_020084248.1	transcription factor jun-B-like	1374	0
XM_020084249.1	natural killer enhancing factor	1023	2.92E-145
XM_020084250.1	protein kinase C alpha type isoform X1	6645	0
XM_020084251.1	protein kinase C alpha type isoform X2	6624	0
XM_020084252.1	protein kinase C alpha type isoform X3	6615	0
XM_020084253.1	protein kinase C alpha type isoform X4	6607	0
XM_020084254.1	NACHT, LRR and PYD domains-containing protein 14-like isoform X1	3311	0
XM_020084255.1	NACHT, LRR and PYD domains-containing protein 14-like isoform X2	3143	0
XM_020084256.1	actin filament-associated protein 1-like 1 isoform X2	3372	0
XM_020084257.1	NACHT, LRR and PYD domains-containing protein 14-like isoform X3	3285	0
XM_020084258.1	NACHT, LRR and PYD domains-containing protein 14-like isoform X2	3321	0
XM_020084259.1	elongation factor 1-alpha 1	2161	0
XM_020084260.1	cytosolic purine 5'-nucleotidase	2206	0
XM_020084261.1	cytosolic purine 5'-nucleotidase	1946	0
XM_020084262.1	MICAL-like protein 1 isoform X1	5648	0
XM_020084263.1	MICAL-like protein 1 isoform X2	5639	0
XM_020084264.1	MICAL-like protein 1 isoform X3	5606	0
XM_020084265.1	actin filament-associated protein 1-like 1 isoform X2	3146	0
XM_020084266.1	MICAL-like protein 1 isoform X4	5597	0
XM_020084267.1	UPF0193 protein EVG1	775	8.18E-148
XM_020084268.1	UPF0193 protein EVG1	826	1.28E-146
XM_020084269.1	clathrin light chain A isoform X1	1800	2.02E-167
XM_020084270.1	clathrin light chain A isoform X2	1763	8.18E-156
XM_020084271.1	clathrin light chain A isoform X3	1701	4.07E-141
XM_020084272.1	inner centromere protein-like	5584	0
XM_020084273.1	G protein-coupled receptor kinase 4 isoform X1	5299	0
XM_020084274.1	G protein-coupled receptor kinase 4 isoform X2	5345	0
XM_020084275.1	protein Dok-7-like isoform X1	3086	0
XM_020084276.1	protein Dok-7-like isoform X2	3083	0
XM_020084277.1	protein Dok-7-like isoform X1	2958	0
XM_020084278.1	gamma-aminobutyric acid receptor subunit pi-like isoform X1	2527	0
XM_020084279.1	choline transporter-like protein 2 isoform X1	3605	0
XM_020084280.1	choline transporter-like protein 2 isoform X2	3561	0
XM_020084281.1	choline transporter-like protein 2 isoform X3	2927	0
XM_020084282.1	coiled-coil domain-containing protein 151 isoform X1	2051	0
XM_020084283.1	coiled-coil domain-containing protein 151 isoform X2	1727	0
XM_020084284.1	kelch-like ECH-associated protein 1	4771	0
XM_020084285.1	protein transport protein Sec23B	3422	0
XM_020084286.1	ubinuclein-2-like isoform X1	7094	0
XM_020084287.1	ubinuclein-2-like isoform X2	6997	0
XM_020084288.1	catenin delta-1-like isoform X3	3092	0
XM_020084289.1	catenin delta-1-like isoform X3	3134	0
XM_020084290.1	catenin delta-1-like isoform X3	3074	0
XM_020084291.1	XK-related protein 2	2905	0
XM_020084292.1	catenin delta-1-like isoform X3	2942	0
XM_020084293.1	catenin delta-1-like isoform X3	2986	0
XM_020084294.1	catenin delta-1-like isoform X3	3118	0
XM_020084295.1	BUB3-interacting and GLEBS motif-containing protein ZNF207-like	2584	4.55E-73
XM_020084296.1	regulator of G-protein signaling 12-like isoform X1	3867	0
XM_020084297.1	regulator of G-protein signaling 12-like isoform X2	3867	0
XM_020084298.1	regulator of G-protein signaling 12-like isoform X3	3745	0
XM_020084299.1	regulator of G-protein signaling 12-like isoform X4	1890	0
XM_020084300.1	regulator of G-protein signaling 12-like isoform X5	1890	0

XM_020084301.1	axin-1 isoform X1	4400	0
XM_020084302.1	axin-1 isoform X1	4352	0
XM_020084303.1	neuropathy target esterase isoform X1	5901	0
XM_020084304.1	neuropathy target esterase isoform X2	5784	0
XM_020084305.1	regulator of G-protein signaling 14-like isoform X4	2552	0
XM_020084306.1	BTB/POZ domain-containing protein 18 isoform X2	4510	0
XM_020084307.1	neuropathy target esterase isoform X3	5784	0
XM_020084308.1	BTB/POZ domain-containing protein 18 isoform X2	4402	0

XM_020084309.1	mitochondrial import inner membrane translocase subunit Tim10	907	3.91E-58
XM_020084310.1	phospholipid phosphatase-related protein type 5-like isoform X2	2182	0
XM_020084311.1	phospholipid phosphatase-related protein type 5-like isoform X2	1284	0
XM_020084312.1	phospholipid phosphatase-related protein type 5-like isoform X2	1280	0
XM_020084313.1	phospholipid phosphatase-related protein type 5-like isoform X2	1462	0
XM_020084314.1	phospholipid phosphatase-related protein type 5-like isoform X2	2198	0
XM_020084315.1	BTB/POZ domain-containing protein 18 isoform X2	4440	0
XM_020084316.1	interleukin enhancer-binding factor 3-like isoform X1	3458	0
XM_020084317.1	interleukin enhancer-binding factor 3-like isoform X2	3428	0
XM_020084318.1	interleukin enhancer-binding factor 3-like isoform X3	2905	0
XM_020084319.1	ATP-dependent RNA helicase DDX39A	1653	0
XM_020084320.1	protein CYR61-like	2543	0
XM_020084321.1	neuronal-specific septin-3-like isoform X2	3453	0
XM_020084322.1	neuronal-specific septin-3-like isoform X2	3598	0
XM_020084323.1	neuronal-specific septin-3-like isoform X2	3526	0
XM_020084324.1	BTB/POZ domain-containing protein 18 isoform X2	4340	0
XM_020084325.1	E3 ubiquitin-protein ligase RNF38-like	4798	0
XM_020084326.1	low-density lipoprotein receptor-like isoform X1	4192	0
XM_020084327.1	low-density lipoprotein receptor-like isoform X2	4189	0
XM_020084328.1	AP-1 complex subunit mu-2	1811	0
XM_020084329.1	cyclin-dependent kinase 4 inhibitor D	1108	2.54E-115
XM_020084330.1	calcium-binding mitochondrial carrier protein SCaMC-1-like isoform X1	2772	0
XM_020084331.1	calcium-binding mitochondrial carrier protein SCaMC-1-like isoform X1	2748	0
XM_020084332.1	calcium-binding mitochondrial carrier protein SCaMC-1-like isoform X1	2395	0
XM_020084333.1	calcium-binding mitochondrial carrier protein SCaMC-1-like isoform X1	2212	0
XM_020084334.1	regulator of G-protein signaling 5-like	1082	1.50E-117
XM_020084335.1	regulator of G-protein signaling 5-like	1043	9.38E-118
XM_020084336.1	leydig cell tumor 10 kDa protein homolog	1184	2.31E-49
XM_020084337.1	calcium-binding protein 2-like isoform X1	1026	9.27E-142
XM_020084338.1	cAMP-dependent protein kinase catalytic subunit alpha	5140	0
XM_020084339.1	factor VIII intron 22 protein-like	1598	0
XM_020084340.1	epithelial membrane protein 2-like	2397	3.48E-91
XM_020084341.1	SH3 and PX domain-containing protein 2A-like isoform X1	11947	0
XM_020084342.1	SH3 and PX domain-containing protein 2A-like isoform X2	11932	0
XM_020084343.1	mitochondrial cardiolipin hydrolase	1758	6.52E-135
XM_020084344.1	transcription elongation factor 1 homolog	1775	6.81E-43
XM_020084345.1	calcium-binding protein 2-like isoform X2	768	1.89E-103
XM_020084346.1	high mobility group-T protein-like	1233	9.13E-99
XM_020084347.1	ATPase ASNA1	2601	0
XM_020084348.1	ATPase family AAA domain-containing protein 5-like isoform X1	3462	0
XM_020084349.1	ATPase family AAA domain-containing protein 5-like isoform X2	3467	0
XM_020084350.1	ATPase family AAA domain-containing protein 5-like isoform X3	3459	0
XM_020084351.1	arf-GAP with dual PH domain-containing protein 2-like	1791	0
XM_020084352.1	transcription elongation factor, mitochondrial	1706	0
XM_020084353.1	mastermind-like protein 3 isoform X1	5764	0
XM_020084354.1	mastermind-like protein 3 isoform X2	5691	0
XM_020084355.1	protein AF-17 isoform X1	6574	0
XM_020084356.1	protein AF-17 isoform X1	6405	0
XM_020084357.1	beta-crystallin B1-like	1187	1.12E-163
XM_020084358.1	calnexin-like isoform X3	3498	0
XM_020084359.1	calnexin-like isoform X3	3495	0
XM_020084360.1	calnexin-like isoform X3	3492	0
XM_020084361.1	calnexin-like isoform X3	3490	0
XM_020084362.1	polycomb group RING finger protein 2	1692	0
XM_020084363.1	CDGSH iron-sulfur domain-containing protein 3, mitochondrial	1614	2.27E-89
XM_020084364.1	rab11 family-interacting protein 4 isoform X1	4504	0
XM_020084365.1	rab11 family-interacting protein 4 isoform X2	4423	0
XM_020084366.1	rab11 family-interacting protein 4 isoform X1	4540	0
XM_020084367.1	tropomodulin-1 isoform X1	4034	0
XM_020084368.1	tropomodulin-1 isoform X1	3841	0
XM_020084369.1	tropomodulin-1 isoform X1	3512	0
XM_020084370.1	PREDICTED: complexin-1-like	2022	9.45E-48
XM_020084371.1	beta-crystallin A1-like	1179	6.92E-156
XM_020084372.1	transcription factor AP-4-like	4235	4.00E-171
XM_020084373.1	EVI5-like protein isoform X1	5061	0
XM_020084374.1	EVI5-like protein isoform X2	5028	0
XM_020084375.1	ubiquitin carboxyl-terminal hydrolase 43	8325	0
XM_020084376.1	potassium voltage-gated channel subfamily KQT member 5-like isoform X1	7355	0
XM_020084377.1	potassium voltage-gated channel subfamily KQT member 5-like isoform X2	7352	0
XM_020084378.1	potassium voltage-gated channel subfamily KQT member 5-like isoform X3	7246	0
XM_020084379.1	potassium voltage-gated channel subfamily KQT member 5-like isoform X4	7242	0
XM_020084380.1	GATA-type zinc finger protein 1	2930	0
XM_020084381.1	GATA-type zinc finger protein 1	2767	0
XM_020084382.1	GATA-type zinc finger protein 1	3107	0

XM_020084383.1	ETS-related transcription factor Elf-2-like isoform X1	2981	0
XM_020084384.1	ferredoxin-2, mitochondrial	1609	1.05E-140
XM_020084385.1	short coiled-coil protein B-like	2763	5.86E-74
XM_020084386.1	tumor necrosis factor ligand superfamily member 14-like	1837	0
XM_020084387.1	tumor necrosis factor ligand superfamily member 14-like	1388	0

XM_020084388.1	tumor necrosis factor ligand superfamily member 14-like	1164	0
XM_020084389.1	transmembrane emp24 domain-containing protein 1-like	1759	2.03E-145
XM_020084390.1	RNA helicase Mov10l1 isoform X1	6353	0
XM_020084391.1	regulator of G-protein signaling 14-like isoform X1	2491	0
XM_020084392.1	ETS-related transcription factor Elf-2-like isoform X2	2979	0
XM_020084393.1	ETS-related transcription factor Elf-2-like isoform X1	3426	0
XM_020084394.1	ETS-related transcription factor Elf-2-like isoform X2	3424	0
XM_020084395.1	ETS-related transcription factor Elf-2-like isoform X1	3498	0
XM_020084396.1	ETS-related transcription factor Elf-2-like isoform X2	3465	0
XM_020084397.1	bifunctional UDP-N-acetylglucosamine 2-epimerase/N-acetylmannosamine kinase isoform X1	3248	0
XM_020084398.1	bifunctional UDP-N-acetylglucosamine 2-epimerase/N-acetylmannosamine kinase isoform X2	2389	0
XM_020084399.1	bifunctional UDP-N-acetylglucosamine 2-epimerase/N-acetylmannosamine kinase isoform X1	2397	0
XM_020084400.1	pyrroline-5-carboxylate reductase 1, mitochondrial-like	2357	0
XM_020084401.1	stromal cell-derived factor 1-like	3758	4.89E-42
XM_020084402.1	ETS-related transcription factor Elf-2-like isoform X1	2950	0
XM_020084403.1	neuronal acetylcholine receptor subunit alpha-3-like	2199	0
XM_020084404.1	neuronal acetylcholine receptor subunit alpha-7-like	2206	0
XM_020084405.1	target of Myb protein 1-like isoform X1	1731	0
XM_020084406.1	target of Myb protein 1-like isoform X2	1728	0
XM_020084407.1	target of Myb protein 1-like isoform X3	1836	0
XM_020084408.1	PREDICTED: noggin-like	1981	0
XM_020084409.1	cytoplasmic phosphatidylinositol transfer protein 1-like isoform X1	4609	0
XM_020084410.1	cytoplasmic phosphatidylinositol transfer protein 1-like isoform X1	1258	0
XM_020084411.1	trafficking kinesin-binding protein 1-like	3686	0
XM_020084412.1	transcription factor SOX-10-like isoform X1	3237	0
XM_020084413.1	fibrinogen-like protein 1	2915	0
XM_020084414.1	rab11 family-interacting protein 1-like	5539	0
XM_020084415.1	sodium channel and clathrin linker 1	3407	0
XM_020084416.1	UPF0462 protein C4orf33 homolog isoform X2	891	2.88E-166
XM_020084417.1	UPF0462 protein C4orf33 homolog isoform X2	864	2.02E-166
XM_020084418.1	UPF0462 protein C4orf33 homolog isoform X2	1549	1.00E-162
XM_020084419.1	UPF0462 protein C4orf33 homolog isoform X2	811	1.48E-148
XM_020084420.1	UPF0462 protein C4orf33 homolog isoform X2	837	2.03E-148
XM_020084421.1	UPF0462 protein C4orf33 homolog isoform X2	778	1.57E-149
XM_020084422.1	UPF0462 protein C4orf33 homolog isoform X2	1069	4.04E-147
XM_020084423.1	nuclear factor interleukin-3-regulated protein-like	3441	0
XM_020084424.1	nucleolar protein 11	2792	0
XM_020084425.1	ral guanine nucleotide dissociation stimulator-like 1 isoform X1	4257	0
XM_020084426.1	ral guanine nucleotide dissociation stimulator-like 1 isoform X1	4217	0
XM_020084427.1	sideroflexin-5-like isoform X1	3405	0
XM_020084428.1	ral guanine nucleotide dissociation stimulator-like 1 isoform X1	4187	0
XM_020084429.1	ral guanine nucleotide dissociation stimulator-like 1 isoform X1	3893	0
XM_020084430.1	phosphoribosyl pyrophosphate synthase-associated protein 1-like isoform X1	2012	0
XM_020084431.1	phosphoribosyl pyrophosphate synthase-associated protein 1-like isoform X2	1966	0
XM_020084432.1	ethanolamine-phosphate cytidylyltransferase-like isoform X1	2445	0
XM_020084433.1	ethanolamine-phosphate cytidylyltransferase-like isoform X1	2425	0
XM_020084434.1	transportin-2-like isoform X1	3060	0
XM_020084435.1	low molecular weight neuronal intermediate filament-like isoform X2	3938	0
XM_020084436.1	sideroflexin-5-like isoform X1	3184	0
XM_020084437.1	ras-related protein Rab-40C-like	1794	0
XM_020084438.1	ras-related protein Rab-40C-like	1718	0
XM_020084439.1	protein Wiz-like isoform X1	3517	0
XM_020084440.1	protein Wiz-like isoform X1	3511	0
XM_020084441.1	protein Wiz-like isoform X1	3643	0
XM_020084442.1	protein Wiz-like isoform X1	3586	0
XM_020084443.1	phosphatidylinositol 4-kinase type 2-beta	2849	0
XM_020084444.1	microtubule-associated serine/threonine-protein kinase 1-like isoform X1	8937	0
XM_020084445.1	microtubule-associated serine/threonine-protein kinase 1-like isoform X2	8934	0
XM_020084446.1	heme oxygenase 2 isoform X2	2762	0
XM_020084447.1	RING finger protein 11-like	1280	3.76E-86
XM_020084448.1	polycomb protein SUZ12 isoform X1	3713	0
XM_020084449.1	forkhead box protein N3	2396	1.35E-169
XM_020084450.1	polycomb protein SUZ12 isoform X2	3996	0
XM_020084451.1	multidrug resistance-associated protein 1-like isoform X1	6182	0
XM_020084452.1	multidrug resistance-associated protein 1-like isoform X1	6178	0
XM_020084453.1	ras and Rab interactor 2 isoform X1	5255	0
XM_020084454.1	ras and Rab interactor 2 isoform X2	3146	0
XM_020084455.1	importin subunit alpha-1	1852	0
XM_020084456.1	importin subunit alpha-1	1831	0
XM_020084457.1	RNA-binding protein 20 isoform X3	4093	0
XM_020084458.1	RNA-binding protein 20 isoform X3	4484	0
XM_020084459.1	RNA-binding protein 20 isoform X3	4064	0
XM_020084460.1	RNA-binding protein 20 isoform X3	4097	0
XM_020084461.1	RNA-binding protein 20 isoform X3	4044	0
XM_020084462.1	RNA-binding protein 20 isoform X3	4129	0
XM_020084463.1	fos-related antigen 1 isoform X1	1505	8.90E-139
XM_020084464.1	sodium-independent sulfate anion transporter	2632	0
XM_020084465.1	glycerophosphodiester phosphodiesterase 1-like	924	0
XM_020084466.1	transmembrane protein 186	1217	3.31E-146

XM_020084467.1	UPF0668 protein C10orf76 homolog	3290	0
XM_020084468.1	xaa-Pro aminopeptidase 1 isoform X1	2098	0
XM_020084469.1	xaa-Pro aminopeptidase 1 isoform X1	2179	0
XM_020084470.1	fos-related antigen 1 isoform X1	1326	6.76E-125
XM_020084471.1	PREDICTED: uncharacterized protein LOC109627714	2013	0
XM_020084472.1	PREDICTED: uncharacterized protein LOC109627714	2107	0
XM_020084473.1	PREDICTED: uncharacterized protein LOC109627714	1968	0
XM_020084474.1	Na(+)/H(+) exchange regulatory cofactor NHE-RF2-like	2951	0
XM_020084475.1	protocadherin-10-like isoform X1	4013	0
XM_020084476.1	protocadherin-10-like isoform X2	5721	0
XM_020084477.1	protocadherin-10-like isoform X3	5498	0
XM_020084478.1	centriolar coiled-coil protein of 110 kDa	4113	0
XM_020084479.1	centriolar coiled-coil protein of 110 kDa	4110	0
XM_020084480.1	guanine nucleotide-binding protein G(o) subunit alpha-like	2424	0
XM_020084481.1	dnaJ homolog subfamily B member 1-like	1673	0
XM_020084482.1	protein YIF1A	2063	0
XM_020084483.1	mitochondrial Rho GTPase 1-A-like isoform X1	3345	0
XM_020084484.1	mitochondrial Rho GTPase 1-A-like isoform X2	2300	0
XM_020084485.1	tudor domain-containing protein 7A-like	4576	0
XM_020084486.1	tudor domain-containing protein 7A-like	4534	0
XM_020084487.1	tudor domain-containing protein 7A-like	4257	0
XM_020084488.1	alpha-1,6-mannosylglycoprotein 6-beta-N-acetylglucosaminyltransferase B-like isoform X1	4817	0
XM_020084489.1	alpha-1,6-mannosylglycoprotein 6-beta-N-acetylglucosaminyltransferase B-like isoform X1	4770	0
XM_020084490.1	alpha-1,6-mannosylglycoprotein 6-beta-N-acetylglucosaminyltransferase B-like isoform X1	4814	0
XM_020084491.1	protein YIF1A	2062	0
XM_020084492.1	alpha-1,6-mannosylglycoprotein 6-beta-N-acetylglucosaminyltransferase B-like isoform X1	4811	0
XM_020084493.1	PREDICTED: uncharacterized protein LOC109627727	3299	7.30E-175
XM_020084494.1	zinc finger CCCH domain-containing protein 6	4227	0
XM_020084495.1	very-long-chain enoyl-CoA reductase isoform X1	1761	0
XM_020084496.1	very-long-chain enoyl-CoA reductase isoform X2	1545	0
XM_020084497.1	very-long-chain enoyl-CoA reductase isoform X3	1539	0
XM_020084498.1	26S proteasome non-ATPase regulatory subunit 12	1765	0
XM_020084499.1	histone chaperone asf1b-B-like	1676	4.68E-143
XM_020084500.1	protein YIF1A	2088	0
XM_020084501.1	myotubularin-related protein 7-like isoform X1	6420	0
XM_020084502.1	myotubularin-related protein 7-like isoform X2	6469	0
XM_020084503.1	SWI/SNF-related matrix-associated actin-dependent regulator of chromatin subfamily E member 1 isoform X1	2688	0
XM_020084504.1	SWI/SNF-related matrix-associated actin-dependent regulator of chromatin subfamily E member 1 isoform X2	2559	0
XM_020084505.1	C-C chemokine receptor type 7	1461	0
XM_020084506.1	exosome complex component RRP45	2729	0
XM_020084507.1	solute carrier family 25 member 38-B-like	3028	0
XM_020084508.1	soluble calcium-activated nucleotidase 1 isoform X2	2781	0
XM_020084509.1	WAP, Kazal, immunoglobulin, Kunitz and NTR domain-containing protein 2-like	3081	0
XM_020084510.1	UPF0585 protein C16orf13 homolog	980	1.87E-153
XM_020084511.1	SLAIN motif-containing protein-like	3202	0
XM_020084512.1	PR domain-containing protein 11	5566	0
XM_020084513.1	PR domain-containing protein 11	5241	0
XM_020084514.1	D-tyrosyl-tRNA(Tyr) deacylase 1	2115	4.58E-131
XM_020084515.1	D-tyrosyl-tRNA(Tyr) deacylase 1	2106	4.20E-131
XM_020084516.1	vacuolar protein sorting-associated protein 37A	2766	0
XM_020084517.1	voltage-dependent calcium channel gamma-1 subunit	3503	5.07E-129
XM_020084518.1	dual specificity protein phosphatase 5	1695	0
XM_020084519.1	cationic amino acid transporter 2 isoform X1	3146	0
XM_020084520.1	ADP-ribosylation factor-binding protein GGA3-like	3602	0
XM_020084521.1	PREDICTED: uncharacterized protein LOC109627749	2771	0
XM_020084522.1	PREDICTED: uncharacterized protein LOC109627749	2891	0
XM_020084523.1	alpha-2C adrenergic receptor-like	3054	0
XM_020084524.1	trafficking protein particle complex subunit 11	4043	0
XM_020084525.1	homeobox protein HMX1-like	1538	0
XM_020084526.1	homeobox protein HMX1-like	1743	3.10E-169
XM_020084527.1	decaprenyl-diphosphate synthase subunit 2 isoform X1	3026	0
XM_020084528.1	decaprenyl-diphosphate synthase subunit 2 isoform X2	2724	0
XM_020084529.1	heterogeneous nuclear ribonucleoprotein A0-like	1639	1.99E-141
XM_020084530.1	fibroblast growth factor receptor-like 1	2987	0
XM_020084531.1	protein FAM161B	3604	0
XM_020084532.1	ubiquinone biosynthesis monooxygenase COQ6, mitochondrial	1744	0
XM_020084533.1	ammonium transporter Rh type A-like	1863	0
XM_020084534.1	transmembrane protein 50A	1227	4.50E-99
XM_020084535.1	transmembrane protein 50A	1410	3.14E-98
XM_020084536.1	BRCA1-A complex subunit BRE	1871	0
XM_020084537.1	protein C9orf72 homolog	2732	0
XM_020084538.1	protein C9orf72 homolog	2542	0
XM_020084539.1	PREDICTED: uncharacterized protein C2orf71 homolog	3475	0
XM_020084540.1	cytoplasmic FMR1-interacting protein 1	5245	0
XM_020084541.1	tRNA (adenine(58)-N(1))-methyltransferase, mitochondrial	1462	0
XM_020084542.1	speedy protein A isoform X1	1447	0
XM_020084543.1	speedy protein A isoform X2	1451	0
XM_020084544.1	speedy protein A isoform X3	1451	0
XM_020084545.1	speedy protein A isoform X4	1571	0
XM_020084546.1	speedy protein A isoform X4	1428	0

XM_020084547.1	connector enhancer of kinase suppressor of ras 1-like	3313	0
XM_020084548.1	PREDICTED: claudin-23-like	2086	1.57E-160
XM_020084549.1	PREDICTED: claudin-23-like	1970	4.66E-161
XM_020084550.1	WD repeat-containing protein 25	1752	0
XM_020084551.1	39S ribosomal protein L33, mitochondrial	747	2.46E-35
XM_020084552.1	ribose-phosphate pyrophosphokinase 1 isoform X1	2147	0
XM_020084553.1	cytospin-A isoform X2	4014	0
XM_020084554.1	PREDICTED: vasohibin-1	4528	0
XM_020084555.1	apoptosis-stimulating of p53 protein 1-like isoform X1	3420	0
XM_020084556.1	apoptosis-stimulating of p53 protein 1-like isoform X2	3314	0
XM_020084557.1	apoptosis-stimulating of p53 protein 1-like isoform X3	3228	0
XM_020084558.1	apoptosis-stimulating of p53 protein 1-like isoform X4	3118	0
XM_020084559.1	type II iodothyronine deiodinase	1598	2.70E-177
XM_020084560.1	ribose-phosphate pyrophosphokinase 1 isoform X2	2140	0
XM_020084561.1	hepatocyte nuclear factor 3-beta	1998	0
XM_020084562.1	mitochondrial peptide methionine sulfoxide reductase-like isoform X1	1663	2.74E-158
XM_020084563.1	mitochondrial peptide methionine sulfoxide reductase-like isoform X1	1182	9.08E-161
XM_020084564.1	mitochondrial peptide methionine sulfoxide reductase-like isoform X1	1537	1.66E-139
XM_020084565.1	calcipressin-3 isoform X1	3985	1.27E-156
XM_020084566.1	calcipressin-3 isoform X1	3984	1.27E-156
XM_020084567.1	calcipressin-3 isoform X1	3722	3.03E-134
XM_020084568.1	charged multivesicular body protein 3	1682	2.32E-116
XM_020084569.1	ribose-phosphate pyrophosphokinase 1 isoform X3	1847	3.47E-155
XM_020084570.1	uncharacterized aarF domain-containing protein kinase 5 isoform X2	2990	0
XM_020084571.1	ras and Rab interactor 3-like isoform X1	4025	0
XM_020084572.1	ras and Rab interactor 3-like isoform X2	4022	0
XM_020084573.1	origin recognition complex subunit 3 isoform X1	2391	0
XM_020084574.1	origin recognition complex subunit 3 isoform X2	2388	0
XM_020084575.1	protein max-like isoform X1	1733	6.17E-91
XM_020084576.1	protein max-like isoform X2	1726	2.45E-87
XM_020084577.1	RAS guanyl-releasing protein 1 isoform X1	3878	0
XM_020084578.1	RAS guanyl-releasing protein 1 isoform X2	3875	0
XM_020084579.1	gap junction beta-3 protein-like	2445	0
XM_020084580.1	gap junction beta-3 protein-like	2399	0
XM_020084581.1	fibrinogen-like protein 1-like protein	1924	0
XM_020084582.1	SCY1-like protein 2	3723	0
XM_020084583.1	fibrinogen-like protein 1-like protein	1929	0
XM_020084584.1	fibrinogen-like protein 1-like protein	1919	0
XM_020084585.1	PREDICTED: uncharacterized protein C14orf80 homolog isoform X1	1443	0
XM_020084586.1	PREDICTED: uncharacterized protein C14orf80 homolog isoform X2	1383	0
XM_020084587.1	tuberoinfundibular peptide of 39 residues isoform X1	961	1.04E-120
XM_020084588.1	tuberoinfundibular peptide of 39 residues isoform X2	898	1.73E-120
XM_020084589.1	tuberoinfundibular peptide of 39 residues isoform X3	922	2.20E-111
XM_020084590.1	tuberoinfundibular peptide of 39 residues isoform X4	859	4.27E-111
XM_020084591.1	tuberoinfundibular peptide of 39 residues isoform X5	919	1.80E-110
XM_020084592.1	mitochondrial ribonuclease P protein 3	2316	0
XM_020084593.1	DNA-binding protein inhibitor ID-4-like	585	1.51E-81
XM_020084594.1	cationic amino acid transporter 2 isoform X2	2216	0
XM_020084595.1	PREDICTED: atlastin-3-like	3541	0
XM_020084596.1	gap junction beta-4 protein-like	2598	7.44E-178
XM_020084597.1	acid phosphatase type 7	1853	0
XM_020084598.1	acid phosphatase type 7	1858	0
XM_020084599.1	acid phosphatase type 7	1736	0
XM_020084600.1	KATNB1-like protein 1 isoform X1	1468	0
XM_020084601.1	KATNB1-like protein 1 isoform X2	1378	0
XM_020084602.1	gamma-aminobutyric acid receptor subunit beta-1-like isoform X2	2798	0
XM_020084603.1	gamma-aminobutyric acid receptor subunit beta-1-like isoform X2	2850	0
XM_020084604.1	gamma-aminobutyric acid receptor subunit beta-1-like isoform X2	2762	0
XM_020084605.1	gamma-aminobutyric acid receptor subunit beta-1-like isoform X2	2674	0
XM_020084606.1	protein FAM177A1	1882	1.02E-129
XM_020084607.1	homeobox protein OTX2	2168	0
XM_020084608.1	protocadherin-19 isoform X1	5546	0
XM_020084609.1	retinol dehydrogenase 14-like	1312	0
XM_020084610.1	CLOCK-interacting pacemaker-like isoform X1	4685	0
XM_020084611.1	CLOCK-interacting pacemaker-like isoform X2	4682	0
XM_020084612.1	multiple epidermal growth factor-like domains protein 11 isoform X1	1902	0
XM_020084613.1	proprotein convertase subtilisin/kexin type 5-like isoform X2	1752	0
XM_020084614.1	39S ribosomal protein L35, mitochondrial	690	1.64E-85
XM_020084615.1	glucosamine-6-phosphate isomerase 2	1356	0
XM_020084616.1	glucosamine-6-phosphate isomerase 2	1566	0
XM_020084617.1	protocadherin-19 isoform X2	5534	0
XM_020084618.1	glucosamine-6-phosphate isomerase 2	1437	0
XM_020084619.1	leucine-rich repeat-containing protein 9-like	6763	0
XM_020084620.1	serine/threonine-protein kinase D3 isoform X1	3463	0
XM_020084621.1	serine/threonine-protein kinase D3 isoform X2	3424	0
XM_020084622.1	receptor expression-enhancing protein 1-like	2128	0
XM_020084623.1	gap junction beta-4 protein-like	1566	0
XM_020084624.1	mutS protein homolog 4	3068	0
XM_020084625.1	protocadherin-19 isoform X3	5528	0
XM_020084626.1	delta-like protein 4	3852	0

XM_020084627.1	protein unc-79 homolog isoform X1	8493	0
XM_020084628.1	protein unc-79 homolog isoform X2	8091	0

XM_020084629.1	pre-mRNA-splicing factor SYF2	992	1.52E-162
XM_020084630.1	ragulator complex protein LAMTOR3 isoform X1	841	1.45E-86
XM_020084631.1	ragulator complex protein LAMTOR3 isoform X1	978	7.20E-86
XM_020084632.1	ragulator complex protein LAMTOR3 isoform X1	738	4.21E-87
XM_020084633.1	ragulator complex protein LAMTOR3 isoform X1	899	4.33E-82
XM_020084634.1	protocadherin-19 isoform X4	5516	0
XM_020084635.1	unconventional myosin-VI-like	5559	0
XM_020084636.1	grainyhead-like protein 3 homolog	2743	0
XM_020084637.1	COMM domain-containing protein 8	1267	2.81E-107
XM_020084638.1	transmembrane protein 234	1032	1.38E-87
XM_020084639.1	protocadherin-19 isoform X5	5333	0
XM_020084640.1	gap junction alpha-10 protein-like	1679	0
XM_020084641.1	glutaminyl-peptide cyclotransferase	2136	0
XM_020084642.1	glutamyl aminopeptidase	3685	0
XM_020084643.1	cdc42 effector protein 3-like	2579	7.05E-135
XM_020084644.1	centrosomal protein kizuna isoform X1	2523	0
XM_020084645.1	centrosomal protein kizuna isoform X1	2321	0
XM_020084646.1	centrosomal protein kizuna isoform X3	2436	0
XM_020084647.1	protocadherin-19 isoform X6	5321	0
XM_020084648.1	serine/threonine-protein kinase PAK 6	3875	0
XM_020084649.1	serine/threonine-protein kinase PAK 6	2559	0
XM_020084650.1	cytochrome c oxidase subunit 8A, mitochondrial	597	1.23E-44
XM_020084651.1	proto-oncogene c-Fos-like	2438	0
XM_020084652.1	inositol-trisphosphate 3-kinase A	2651	0
XM_020084653.1	ribosomal protein S6 kinase-like 1	2801	0
XM_020084654.1	homeobox protein Nkx-2.2 isoform X1	1794	2.02E-175
XM_020084655.1	homeobox protein Nkx-2.2 isoform X1	1844	9.94E-175
XM_020084656.1	protocadherin-19 isoform X7	5303	0
XM_020084657.1	homeobox protein Nkx-2.2 isoform X1	1949	8.51E-169
XM_020084658.1	kelch repeat and BTB domain-containing protein 11	2775	0
XM_020084659.1	rho GTPase-activating protein 20-like	3250	0
XM_020084660.1	sine oculis-binding protein homolog	3750	0
XM_020084661.1	probable G-protein coupled receptor 135	4914	0
XM_020084662.1	SH3 and cysteine-rich domain-containing protein isoform X1	2955	0
XM_020084663.1	SH3 and cysteine-rich domain-containing protein isoform X2	2922	0
XM_020084664.1	SH3 and cysteine-rich domain-containing protein isoform X3	2922	0
XM_020084665.1	SH3 and cysteine-rich domain-containing protein isoform X4	2889	0
XM_020084666.1	complement component C1q receptor-like	3016	0
XM_020084667.1	fibronectin type III domain-containing protein 5-like	5096	1.74E-143
XM_020084668.1	endothelin B receptor-like	3831	0
XM_020084669.1	TMF-regulated nuclear protein 1	4355	1.10E-73
XM_020084670.1	ankyrin repeat domain-containing protein 6 isoform X1	1910	0
XM_020084671.1	transcription regulator protein BACH2-like	2499	0
XM_020084672.1	disheveled-associated activator of morphogenesis 1-like isoform X1	4569	0
XM_020084673.1	probable phospholipid-transporting ATPase VD	4156	0
XM_020084674.1	tripartite motif-containing protein 16-like	1041	0
XM_020084675.1	endothelin B receptor-like	3835	0
XM_020084676.1	apolipoprotein B-100-like	474	3.80E-87
XM_020084677.1	PREDICTED: gephyrin-like	3394	0
XM_020084678.1	synaptotagmin-14-like isoform X4	2525	0
XM_020084679.1	calpain-1 catalytic subunit-like	2061	0
XM_020084680.1	PREDICTED: uncharacterized protein LOC109627859, partial	3453	0
XM_020084681.1	PREDICTED: gamma-2-syntrophin	3097	0
XM_020084682.1	PREDICTED: uncharacterized protein KIAA1109 homolog	16594	0
XM_020084683.1	eukaryotic translation initiation factor 4E-like	702	3.09E-151
XM_020084684.1	alcohol dehydrogenase class-3 chain L	1083	0
XM_020084685.1	cilia- and flagella-associated protein 99-like	985	0
XM_020084686.1	ubiquitin-conjugating enzyme E2 K	2073	1.08E-128
XM_020084687.1	myb-like protein X	2007	0
XM_020084688.1	melanoma inhibitory activity protein 3	6271	0
XM_020084689.1	ral GTPase-activating protein subunit alpha-1	9618	0
XM_020084690.1	proteasome subunit alpha type-6-like	1613	2.29E-148
XM_020084691.1	tetratricopeptide repeat protein 7B	4326	0
XM_020084692.1	tubulin beta-4B chain-like	1851	0
XM_020084693.1	PREDICTED: ribokinase	1324	0
XM_020084694.1	proto-oncogene c-Fos-like	2883	5.36E-84
XM_020084695.1	WD repeat-containing protein 35	4464	0
XM_020084696.1	matrilin-4 isoform X1	2988	0
XM_020084697.1	BTB/POZ domain-containing protein KCTD12-like	3466	0
XM_020084698.1	kinesin-like protein KIF6	2554	0
XM_020084699.1	absent in melanoma 1 protein-like	9933	0
XM_020084700.1	myelin transcription factor 1-like protein	4577	0
XM_020084701.1	macoilin-1-like isoform X3	4430	0
XM_020084702.1	runt-related transcription factor 3	634	6.37E-125
XM_020084703.1	homeobox protein Nkx-2.4	554	3.12E-104
XM_020084704.1	serine/threonine-protein kinase D3-like	2024	0
XM_020084705.1	CD109 antigen-like	4397	0

XM_020084706.1	AT-rich interactive domain-containing protein 1B-like	2038	0
XM_020084707.1	AT-rich interactive domain-containing protein 1A-like	6368	0
XM_020084708.1	biorientation of chromosomes in cell division protein 1-like 1	6052	0
XM_020084709.1	PREDICTED: uncharacterized protein KIAA1522 homolog	5596	0
XM_020084710.1	C-1-tetrahydrofolate synthase, cytoplasmic-like	1967	0
XM_020084711.1	1-phosphatidylinositol 4,5-bisphosphate phosphodiesterase beta-2-like	3813	0
XM_020084712.1	pleckstrin homology domain-containing family G member 3-like	3678	0
XM_020084713.1	transmembrane protein 121-like	3552	0
XM_020084714.1	dnaJ homolog subfamily C member 5-like isoform X1	1098	0
XM_020084715.1	PREDICTED: formin-1-like	5454	0
XM_020084716.1	ryanodine receptor 3-like	15490	0
XM_020084717.1	thrombospondin-2 isoform X1	5477	0
XM_020084718.1	regulator of G-protein signaling 7	1723	0
XM_020084719.1	protein lin-52 homolog	455	1.56E-78
XM_020084720.1	N-acetyllactosaminide beta-1,3-N-acetylglucosaminyltransferase 2-like	2473	0
XM_020084721.1	latent-transforming growth factor beta-binding protein 2	7760	0
XM_020084722.1	tetratricopeptide repeat protein 39B-like	335	1.22E-77
XM_020084723.1	protein angel homolog 1	3462	0
XM_020084724.1	leucine-rich repeat-containing protein 74A	1482	0
XM_020084725.1	PREDICTED: olfactomedin-4-like	1422	0
XM_020084726.1	PREDICTED: olfactomedin-4-like	1374	0
XM_020084727.1	radixin-like isoform X1	1198	0
XM_020084728.1	N-acetyllactosaminide beta-1,3-N-acetylglucosaminyltransferase 2-like	2517	0
XM_020084729.1	echinoderm microtubule-associated protein-like 5	6939	0
XM_020084730.1	homeodomain-interacting protein kinase 2-like	1192	0
XM_020084731.1	60 kDa lysophospholipase	2877	0
XM_020084732.1	kinesin-like protein KIF26A	8503	0
XM_020084733.1	inositol-tetrakisphosphate 1-kinase	826	5.81E-106
XM_020084734.1	cilia- and flagella-associated protein 99-like	985	1.23E-177
XM_020084735.1	probable sodium-coupled neutral amino acid transporter 6	556	8.95E-92
XM_020084736.1	sodium-coupled neutral amino acid transporter 3-like	983	1.77E-71
XM_020084737.1	prolactin-releasing peptide receptor-like	1215	0
XM_020084738.1	probable RNA-binding protein EIF1AD	1923	6.02E-91
XM_020084739.1	kinase D-interacting substrate of 220 kDa-like	3217	0
XM_020084740.1	doublecortin domain-containing protein 2-like	1684	0
XM_020084741.1	tonsoku-like protein	4359	0
XM_020084742.1	CAAX prenyl protease 1 homolog	2526	0
XM_020084743.1	Fanconi anemia group M protein-like	5322	0
XM_020084744.1	doublecortin domain-containing protein 2-like	823	6.20E-175
XM_020084745.1	tyrosine-protein kinase Lck	2158	0
XM_020084746.1	probable RNA-binding protein EIF1AD	1919	4.94E-91
XM_020084747.1	tyrosine-protein kinase Lck	2095	0
XM_020084748.1	hydroxymethylglutaryl-CoA lyase, mitochondrial	1597	0
XM_020084749.1	ras-related protein Rab-15-like	1348	6.00E-156
XM_020084750.1	serine/threonine-protein phosphatase 2A regulatory subunit B'' subunit gamma	1736	0
XM_020084751.1	gamma-aminobutyric acid receptor subunit alpha-1-like	1427	0
XM_020084752.1	adenylyltransferase and sulfurtransferase MOCS3 isoform X1	1563	0
XM_020084753.1	adenylyltransferase and sulfurtransferase MOCS3 isoform X2	1531	0
XM_020084754.1	probable RNA-binding protein EIF1AD	1859	8.53E-92
XM_020084755.1	homeobox protein SIX6	1468	1.56E-162
XM_020084756.1	apoptosis regulatory protein Siva	800	8.73E-108
XM_020084757.1	ALK tyrosine kinase receptor	7100	0
XM_020084758.1	E3 ubiquitin-protein ligase UBR1 isoform X1	6534	0
XM_020084759.1	E3 ubiquitin-protein ligase UBR1 isoform X2	6519	0
XM_020084760.1	ATP-dependent RNA helicase DDX24	2966	0
XM_020084761.1	ankyrin repeat and SOCS box protein 2 isoform X2	2069	0
XM_020084762.1	ankyrin repeat and SOCS box protein 2 isoform X2	2229	0
XM_020084763.1	ankyrin repeat and SOCS box protein 2 isoform X2	2187	0
XM_020084764.1	ankyrin repeat and SOCS box protein 2 isoform X2	2111	0
XM_020084765.1	protein Z-dependent protease inhibitor-like	1543	0
XM_020084766.1	zinc finger protein 711	4057	0
XM_020084767.1	coiled-coil domain-containing protein 42 homolog isoform X1	1463	0
XM_020084768.1	coiled-coil domain-containing protein 42 like-2-like isoform X2	1454	0
XM_020084769.1	PREDICTED: ataxin-3-like	3086	0
XM_020084770.1	protein FAM181A	1370	3.65E-165
XM_020084771.1	protein FAM181A	1398	5.06E-165
XM_020084772.1	proline-rich membrane anchor 1	1495	3.56E-149
XM_020084773.1	ubiquitin thioesterase OTUB2	969	1.83E-169
XM_020084774.1	zinc finger protein 711	3908	0
XM_020084775.1	S100P-binding protein isoform X1	1714	0
XM_020084776.1	S100P-binding protein isoform X2	2114	0
XM_020084777.1	S100P-binding protein isoform X1	1580	0
XM_020084778.1	glycoprotein hormones alpha chain	691	4.16E-72
XM_020084779.1	gem-associated protein 2	1000	2.98E-103
XM_020084780.1	glutamate-rich protein 1 isoform X1	1766	0
XM_020084781.1	glutamate-rich protein 1 isoform X2	1697	0
XM_020084782.1	iron-sulfur cluster assembly 2 homolog, mitochondrial	984	4.98E-111
XM_020084783.1	eosinophil peroxidase-like	2707	0
XM_020084784.1	oligodendrocyte transcription factor 3-like	1302	8.40E-136

XM_020084785.1	piRNA biogenesis protein EXD1 isoform X1	2209	0
XM_020084786.1	E3 ubiquitin-protein ligase NEURL1B isoform X1	2520	0
XM_020084787.1	piRNA biogenesis protein EXD1 isoform X2	2188	0
XM_020084788.1	GTP cyclohydrolase 1 feedback regulatory protein	914	1.99E-58
XM_020084789.1	disks large-associated protein 2	3365	0
XM_020084790.1	gamma-aminobutyric acid receptor subunit gamma-1	2390	0
XM_020084791.1	microsomal glutathione S-transferase 3-like	770	3.58E-98
XM_020084792.1	dynein light chain 1, axonemal	1470	1.68E-97
XM_020084793.1	5-hydroxytryptamine receptor 1D	2788	0
XM_020084794.1	E3 ubiquitin-protein ligase NEURL1B isoform X2	2797	0
XM_020084795.1	5-hydroxytryptamine receptor 1D	2924	0
XM_020084796.1	ATPase inhibitor B, mitochondrial-like	684	2.21E-48
XM_020084797.1	serine dehydratase-like	1683	0
XM_020084798.1	protein FAM167B	1712	1.50E-136
XM_020084799.1	protein-lysine methyltransferase METTL21D	957	1.07E-166
XM_020084800.1	cytosolic 5'-nucleotidase 1A-like	3002	0
XM_020084801.1	radial spoke head protein 3 homolog	1240	0
XM_020084802.1	rho-related GTP-binding protein RhoV-like	990	2.62E-156
XM_020084803.1	putative transmembrane protein INAFM2	1834	1.44E-105
XM_020084804.1	melatonin receptor type 1B-like	1561	0
XM_020084805.1	prospero homeobox protein 1-like	2824	0
XM_020084806.1	prospero homeobox protein 1-like	2796	0
XM_020084807.1	PREDICTED: uncharacterized protein C2orf50 homolog	1526	7.78E-106
XM_020084808.1	paired box protein Pax-1	1808	0
XM_020084809.1	zinc-binding protein A33-like	1720	0
XM_020084810.1	PREDICTED: neuroglobin	3167	1.53E-106
XM_020084811.1	PREDICTED: neuroglobin	3095	1.02E-106
XM_020084812.1	phospholipase D4	1786	0
XM_020084813.1	thyroxine 5-deiodinase	1332	0
XM_020084814.1	intraflagellar transport protein 43 homolog	938	1.12E-129
XM_020084815.1	ornithine decarboxylase-like	1323	0
XM_020084816.1	PREDICTED: uncharacterized protein LOC109627973 isoform X1	2484	0
XM_020084817.1	glutathione-specific gamma-glutamylcyclotransferase 1-like	1243	1.46E-154
XM_020084818.1	calpain-1 catalytic subunit-like	3160	0
XM_020084819.1	muscarinic acetylcholine receptor M3-like	1588	0
XM_020084820.1	protein odd-skipped-related 1-like	1553	0
XM_020084821.1	protein odd-skipped-related 1-like	1550	0
XM_020084822.1	5-hydroxytryptamine receptor 1B-like	1514	0
XM_020084823.1	leucine-rich repeat and fibronectin type III domain-containing protein 1	2544	0
XM_020084824.1	PREDICTED: uncharacterized protein LOC109627973 isoform X2	2481	0
XM_020084825.1	ovarian cancer G-protein coupled receptor 1-like	1534	0
XM_020084826.1	potassium voltage-gated channel subfamily S member 3	2080	0
XM_020084827.1	palmitoyltransferase ZDHHC21 isoform X2	1888	0
XM_020084828.1	39S ribosomal protein L11, mitochondrial	869	8.07E-115
XM_020084829.1	vascular endothelial growth factor A-like	1897	7.94E-96
XM_020084830.1	brain-enriched guanylate kinase-associated protein-like	2588	0
XM_020084831.1	protein delta homolog 1	2969	0
XM_020084832.1	synapse differentiation-inducing gene protein 1-like	1844	8.46E-164
XM_020084833.1	PREDICTED: uncharacterized protein LOC109627973 isoform X3	2478	0
XM_020084834.1	synapse differentiation-inducing gene protein 1-like	1871	3.01E-163
XM_020084835.1	interphotoreceptor matrix proteoglycan 1	3468	0
XM_020084836.1	myotubularin-related protein 9-like isoform X1	1993	0
XM_020084837.1	myotubularin-related protein 9-like isoform X2	1936	0
XM_020084838.1	visual system homeobox 2 isoform X1	1815	0
XM_020084839.1	visual system homeobox 2 isoform X2	1752	0
XM_020084840.1	ankyrin repeat domain-containing protein 63	1952	0
XM_020084841.1	PREDICTED: uncharacterized protein LOC109627973 isoform X4	2474	0
XM_020084842.1	solute carrier family 35 member F4 isoform X1	1869	0
XM_020084843.1	solute carrier family 35 member F4 isoform X2	1325	0
XM_020084844.1	solute carrier family 35 member F4 isoform X3	1352	0
XM_020084845.1	complement component C1q receptor-like	1416	0
XM_020084846.1	potassium voltage-gated channel subfamily F member 1-like	2201	0
XM_020084847.1	insulinoma-associated protein 2	3093	0
XM_020084848.1	sodium/bile acid cotransporter	1516	0
XM_020084849.1	sodium/bile acid cotransporter	1490	0
XM_020084850.1	cannabinoid receptor type 1B-like	1866	0
XM_020084851.1	V-type proton ATPase subunit C 1-A	1231	0
XM_020084852.1	protein YIPF7-like	1136	8.92E-147
XM_020084853.1	PCTP-like protein	2507	0
XM_020084854.1	leucine-rich repeat and fibronectin type III domain-containing protein 1	4720	0
XM_020084855.1	leucine-rich repeat and immunoglobulin-like domain-containing nogo receptor-interacting protein 2	3435	0
XM_020084856.1	serine palmitoyltransferase 2-like	5505	0
XM_020084857.1	methionine aminopeptidase 1	3113	0
XM_020084858.1	alcohol dehydrogenase 1-like	1431	0
XM_020084859.1	disintegrin and metalloproteinase domain-containing protein 17-like	3414	0
XM_020084860.1	cleavage and polyadenylation specificity factor subunit 3 isoform X1	2805	0
XM_020084861.1	cleavage and polyadenylation specificity factor subunit 3 isoform X2	1880	0
XM_020084862.1	14-3-3 protein beta/alpha-1	2031	0
XM_020084863.1	14-3-3 protein beta/alpha-1	1978	0

XM_020084864.1	ETS-related transcription factor Elf-1-like isoform X1	4674	0
XM_020084865.1	dnaJ homolog subfamily C member 30-like	2356	0
XM_020084866.1	isoamyl acetate-hydrolyzing esterase 1 homolog	1017	7.12E-153
XM_020084867.1	integrin beta-1-binding protein 1	1633	3.60E-101
XM_020084868.1	integrin beta-1-binding protein 1	1632	3.50E-101
XM_020084869.1	protein atonal homolog 8 isoform X1	2897	0
XM_020084870.1	integrin beta-1-binding protein 1	1812	1.91E-100
XM_020084871.1	photoreceptor outer segment membrane glycoprotein 2-like	1146	0
XM_020084872.1	ETS-related transcription factor Elf-1-like isoform X1	4994	0
XM_020084873.1	noncompact myelin-associated protein	1397	3.83E-57
XM_020084874.1	noncompact myelin-associated protein	1272	1.38E-57

XM_020084875.1	RNA polymerase-associated protein RTF1 homolog	1196	0
XM_020084876.1	visual pigment-like receptor peropsin	1110	0
XM_020084877.1	potassium channel subfamily K member 16-like	928	0
XM_020084878.1	potassium channel subfamily K member 17-like	912	0
XM_020084879.1	ETS-related transcription factor Elf-1-like isoform X1	5097	0
XM_020084880.1	PREDICTED: prosaposin-like	459	7.00E-75
XM_020084881.1	zinc finger MYM-type protein 4-like isoform X1	6825	0
XM_020084882.1	zinc finger MYM-type protein 4-like isoform X2	6796	0
XM_020084883.1	ETS-related transcription factor Elf-1-like isoform X1	4421	0
XM_020084884.1	zinc finger MYM-type protein 4-like isoform X3	6712	0
XM_020084885.1	zinc finger MYM-type protein 4-like isoform X4	6657	0
XM_020084886.1	zinc finger MYM-type protein 4-like isoform X5	6682	0
XM_020084887.1	zinc finger MYM-type protein 4-like isoform X4	6694	0
XM_020084888.1	digestive organ expansion factor homolog	2784	0
XM_020084889.1	Y+L amino acid transporter 2-like	2059	0
XM_020084890.1	poly(ADP-ribose) glycohydrolase ARH3	2154	0
XM_020084891.1	autophagy-related protein 2 homolog B isoform X1	7608	0
XM_020084892.1	ETS-related transcription factor Elf-1-like isoform X1	4617	0
XM_020084893.1	autophagy-related protein 2 homolog B isoform X1	7591	0
XM_020084894.1	autophagy-related protein 2 homolog B isoform X1	7582	0
XM_020084895.1	autophagy-related protein 2 homolog B isoform X1	7549	0
XM_020084896.1	autophagy-related protein 2 homolog B isoform X1	6347	0
XM_020084897.1	PREDICTED: uncharacterized protein LOC109628035 isoform X1	2066	0
XM_020084898.1	PREDICTED: uncharacterized protein LOC109628035 isoform X2	2060	0
XM_020084899.1	PREDICTED: uncharacterized protein LOC109628035 isoform X3	2045	0
XM_020084900.1	PREDICTED: uncharacterized protein LOC109628035 isoform X4	2027	0
XM_020084901.1	rab9 effector protein with kelch motifs-like isoform X5	1955	0
XM_020084902.1	rab9 effector protein with kelch motifs-like isoform X6	1916	0
XM_020084903.1	rab9 effector protein with kelch motifs-like isoform X7	1709	0
XM_020084904.1	NADP-dependent oxidoreductase domain-containing protein 1 isoform X1	1763	0
XM_020084905.1	NADP-dependent oxidoreductase domain-containing protein 1 isoform X2	1719	0
XM_020084906.1	pre-mRNA-processing factor 39-like	2658	0
XM_020084907.1	protein TMED8	1754	1.00E-152
XM_020084908.1	protein TMED8	2030	1.94E-151
XM_020084909.1	epididymal secretory protein E1	1099	2.35E-104
XM_020084910.1	GSK3-beta interaction protein	1616	3.23E-90
XM_020084911.1	rho GTPase-activating protein 11A-like	3980	0
XM_020084912.1	rho GTPase-activating protein 11A-like	3709	0
XM_020084913.1	actin, alpha cardiac	1357	0
XM_020084914.1	T-lymphoma invasion and metastasis-inducing protein 1-like	6984	0
XM_020084915.1	T-lymphoma invasion and metastasis-inducing protein 1-like	6972	0
XM_020084916.1	protein SCAF8 isoform X1	5381	0
XM_020084917.1	protein SCAF8 isoform X1	5377	0
XM_020084918.1	forkhead box protein O1-A-like	7154	0
XM_020084919.1	protein SCAF8 isoform X1	4813	0
XM_020084920.1	homeobox protein SIX4	3896	0
XM_020084921.1	CDK-activating kinase assembly factor MAT1 isoform X1	1301	0
XM_020084922.1	CDK-activating kinase assembly factor MAT1 isoform X1	1337	0
XM_020084923.1	homeobox protein SIX1	1586	0
XM_020084924.1	tRNA (guanine(37)-N1)-methyltransferase isoform X1	1764	0
XM_020084925.1	tRNA (guanine(37)-N1)-methyltransferase isoform X2	1752	0
XM_020084926.1	zona pellucida sperm-binding protein 3-like	2061	0
XM_020084927.1	polyadenylate-binding protein 4	2565	0
XM_020084928.1	probable histone deacetylase 1-B	2546	0
XM_020084929.1	peptidyl-prolyl cis-trans isomerase E	1154	0
XM_020084930.1	magnesium transporter NIPA2-like	2051	0
XM_020084931.1	keratinocyte-associated protein 3-like	1128	5.37E-140
XM_020084932.1	pecanex-like protein 4	4612	0
XM_020084933.1	protein phosphatase 1A isoform X1	3814	0
XM_020084934.1	protein phosphatase 1A isoform X2	6543	0
XM_020084935.1	protein phosphatase 1A isoform X3	5443	0
XM_020084936.1	dehydrogenase/reductase SDR family member 7C-B-like	1364	0
XM_020084937.1	hypoxia-inducible factor 1-alpha	3668	0
XM_020084938.1	serine protease HTRA2, mitochondrial-like	2949	0
XM_020084939.1	zinc finger and BTB domain-containing protein 18.2-like isoform X1	6348	0
XM_020084940.1	zinc finger and BTB domain-containing protein 18.2-like isoform X1	5864	0
XM_020084941.1	zinc finger and BTB domain-containing protein 18.2-like isoform X3	2992	0
XM_020084942.1	mitochondrial import inner membrane translocase subunit Tim8 A	1104	2.95E-59

XM_020084943.1	RAC-alpha serine/threonine-protein kinase isoform X1	3749	0
XM_020084944.1	RAC-alpha serine/threonine-protein kinase isoform X1	3812	0
XM_020084945.1	RAC-alpha serine/threonine-protein kinase isoform X1	3746	0
XM_020084946.1	sorting nexin-14 isoform X1	3659	0
XM_020084947.1	cysteine-rich hydrophobic domain-containing protein 2-like	1904	4.07E-113
XM_020084948.1	sorting nexin-14 isoform X2	3632	0
XM_020084949.1	sorting nexin-14 isoform X3	3511	0
XM_020084950.1	sorting nexin-14 isoform X4	3483	0
XM_020084951.1	heterogeneous nuclear ribonucleoprotein Q-like isoform X1	3172	0
XM_020084952.1	heterogeneous nuclear ribonucleoprotein Q-like isoform X1	3296	0
XM_020084953.1	heterogeneous nuclear ribonucleoprotein Q-like isoform X1	3480	0
XM_020084954.1	heterogeneous nuclear ribonucleoprotein Q-like isoform X1	3282	0
XM_020084955.1	heterogeneous nuclear ribonucleoprotein Q-like isoform X1	3072	0
XM_020084956.1	heterogeneous nuclear ribonucleoprotein Q-like isoform X1	3329	0
XM_020084957.1	VIP36-like protein	2239	0
XM_020084958.1	zinc finger and BTB domain-containing protein 49	6393	0
XM_020084959.1	putative E3 ubiquitin-protein ligase UBR7	2352	0
XM_020084960.1	zona pellucida sperm-binding protein 3-like	2094	0
XM_020084961.1	sodium-dependent lysophosphatidylcholine symporter 1-B-like	3670	0
XM_020084962.1	protein NRDE2 homolog	3838	0
XM_020084963.1	trafficking protein particle complex subunit 12	3960	0
XM_020084964.1	trafficking protein particle complex subunit 12	3955	0
XM_020084965.1	protein TSSC1	1569	0
XM_020084966.1	1,2-dihydroxy-3-keto-5-methylthiopentene dioxygenase	2058	1.42E-124
XM_020084967.1	F-box only protein 33	2323	0
XM_020084968.1	serine/threonine-protein kinase PLK4	3241	0
XM_020084969.1	protein RMD5 homolog B	2163	0
XM_020084970.1	heat shock 70 kDa protein 4L	2718	0
XM_020084971.1	heat shock 70 kDa protein 4L	3668	0
XM_020084972.1	rab3 GTPase-activating protein non-catalytic subunit	5134	0
XM_020084973.1	valine--tRNA ligase, mitochondrial	3477	0
XM_020084974.1	valine--tRNA ligase, mitochondrial	3395	0
XM_020084975.1	nuclear receptor coactivator 7-like isoform X1	4833	0
XM_020084976.1	nuclear receptor coactivator 7-like isoform X1	4963	0
XM_020084977.1	nuclear receptor coactivator 7-like isoform X1	4830	0
XM_020084978.1	selenoprotein N	3717	0
XM_020084979.1	selenoprotein N	3721	0
XM_020084980.1	selenoprotein N	3690	0
XM_020084981.1	selenoprotein N	3686	0
XM_020084982.1	PQ-loop repeat-containing protein 3	1541	4.40E-105
XM_020084983.1	rho-associated protein kinase 2	6414	0
XM_020084984.1	melanoma differentiation-associated protein 5	1579	0
XM_020084985.1	heme-binding protein 2-like	2674	0
XM_020084986.1	IQ domain-containing protein C	896	3.31E-165
XM_020084987.1	serine/arginine repetitive matrix protein 1 isoform X4	3436	1.08E-103
XM_020084988.1	serine/arginine repetitive matrix protein 1 isoform X3	3433	1.28E-103
XM_020084989.1	LON peptidase N-terminal domain and RING finger protein 3-like	6205	0
XM_020084990.1	serine/arginine repetitive matrix protein 1 isoform X4	3349	2.38E-103
XM_020084991.1	serine/arginine repetitive matrix protein 1 isoform X4	3432	1.22E-103
XM_020084992.1	L-threonine 3-dehydrogenase, mitochondrial-like isoform X1	1774	0
XM_020084993.1	L-threonine 3-dehydrogenase, mitochondrial-like isoform X1	2556	0
XM_020084994.1	L-threonine 3-dehydrogenase, mitochondrial-like isoform X1	1806	0
XM_020084995.1	zinc finger protein Rlf	6361	0
XM_020084996.1	zinc finger protein 593	900	3.41E-82
XM_020084997.1	C-C chemokine receptor type 6-like	2016	0
XM_020084998.1	centrosomal protein of 170 kDa protein B isoform X1	6966	0
XM_020084999.1	centrosomal protein of 170 kDa protein B isoform X2	6927	0
XM_020085000.1	centrosomal protein of 170 kDa protein B isoform X3	6862	0
XM_020085001.1	centrosomal protein of 170 kDa protein B isoform X4	6807	0
XM_020085002.1	multidrug resistance-associated protein 5	5408	0
XM_020085003.1	kinectin isoform X1	5023	0
XM_020085004.1	kinectin isoform X2	5020	0
XM_020085005.1	kinectin isoform X3	5020	0
XM_020085006.1	kinectin isoform X4	5017	0
XM_020085007.1	kinectin isoform X5	5014	0
XM_020085008.1	kinectin isoform X6	4939	0
XM_020085009.1	kinectin isoform X7	4936	0
XM_020085010.1	protein dispatched homolog 1	5970	0
XM_020085011.1	RNA pseudouridylate synthase domain-containing protein 2 isoform X1	2508	0
XM_020085012.1	RNA pseudouridylate synthase domain-containing protein 2 isoform X2	2400	0
XM_020085013.1	RNA pseudouridylate synthase domain-containing protein 2 isoform X3	2366	0
XM_020085014.1	protein-L-isoaspartate(D-aspartate) O-methyltransferase-like isoform X1	1387	0
XM_020085015.1	protein-L-isoaspartate(D-aspartate) O-methyltransferase-like isoform X1	1572	0
XM_020085016.1	protein-L-isoaspartate(D-aspartate) O-methyltransferase-like isoform X1	1377	0
XM_020085017.1	neuronal acetylcholine receptor subunit beta-2-like isoform X2	3962	0
XM_020085018.1	protein-L-isoaspartate(D-aspartate) O-methyltransferase-like isoform X1	1423	2.69E-179
XM_020085019.1	PREDICTED: uncharacterized protein C15orf57 homolog	1271	4.65E-130
XM_020085020.1	E3 ubiquitin-protein ligase pellino homolog 2 isoform X1	3427	0
XM_020085021.1	E3 ubiquitin-protein ligase pellino homolog 2 isoform X2	3422	0

XM_020085022.1	collagen alpha-1(XII) chain isoform X1	10278	0
XM_020085023.1	collagen alpha-1(XII) chain isoform X2	10045	0
XM_020085024.1	collagen alpha-1(XII) chain isoform X1	10957	0
XM_020085025.1	collagen alpha-1(XII) chain isoform X1	6783	0
XM_020085026.1	neuronal acetylcholine receptor subunit beta-2-like isoform X2	4186	0
XM_020085027.1	serine/threonine-protein phosphatase 2A 56 kDa regulatory subunit gamma isoform isoform X1	4179	0
XM_020085028.1	serine/threonine-protein phosphatase 2A 56 kDa regulatory subunit gamma isoform isoform X2	4062	0
XM_020085029.1	serine/threonine-protein phosphatase 2A 56 kDa regulatory subunit gamma isoform isoform X3	4144	0
XM_020085030.1	serine/threonine-protein phosphatase 2A 56 kDa regulatory subunit gamma isoform isoform X4	1491	0
XM_020085031.1	tripeptidyl-peptidase 1 isoform X1	2971	0
XM_020085032.1	tripeptidyl-peptidase 1 isoform X2	2971	0
XM_020085033.1	transcriptional repressor protein YY1-like	1857	0
XM_020085034.1	signal-induced proliferation-associated 1-like protein 1	7455	0
XM_020085035.1	neuronal acetylcholine receptor subunit beta-2-like isoform X2	4182	0
XM_020085036.1	signal-induced proliferation-associated 1-like protein 1	7370	0
XM_020085037.1	glutathione peroxidase 1-like	982	4.21E-120
XM_020085038.1	troponin C, skeletal muscle	1861	2.19E-100
XM_020085039.1	serine/threonine-protein kinase Nek9	5303	0
XM_020085040.1	PREDICTED: trichohyalin-like	2141	0
XM_020085041.1	CD209 antigen-like	1289	0
XM_020085042.1	acylphosphatase-1 isoform X1	618	4.35E-66
XM_020085043.1	neuronal acetylcholine receptor subunit beta-2-like isoform X2	4097	0
XM_020085044.1	acylphosphatase-1 isoform X2	576	1.56E-67
XM_020085045.1	exocyst complex component 5	3382	0
XM_020085046.1	AP-5 complex subunit mu-1	2902	0
XM_020085047.1	AP-5 complex subunit mu-1	2630	0
XM_020085048.1	apoptosis-resistant E3 ubiquitin protein ligase 1	5577	0
XM_020085049.1	apoptosis-resistant E3 ubiquitin protein ligase 1	5561	0
XM_020085050.1	rRNA-processing protein FCF1 homolog isoform X1	894	1.37E-136
XM_020085051.1	rRNA-processing protein FCF1 homolog isoform X2	929	1.11E-131
XM_020085052.1	neuronal acetylcholine receptor subunit beta-2-like isoform X2	4176	0
XM_020085053.1	protein Daple-like	6705	0
XM_020085054.1	FH2 domain-containing protein 1-like	3463	0
XM_020085055.1	adenylosuccinate synthetase isozyme 1	1733	0
XM_020085056.1	inverted formin-2-like	934	1.50E-156
XM_020085057.1	filamin-A-interacting protein 1-like	4400	0
XM_020085058.1	filamin-A-interacting protein 1-like	4379	0
XM_020085059.1	cell cycle control protein 50A-like	2343	0
XM_020085060.1	neuronal acetylcholine receptor subunit beta-2-like isoform X2	4101	0
XM_020085061.1	neurexin-3a isoform X1	7324	0
XM_020085062.1	neurexin-3a isoform X1	7304	0
XM_020085063.1	neurexin-3a isoform X1	7315	0
XM_020085064.1	neurexin-3a isoform X1	6688	0
XM_020085065.1	neurexin-3 isoform X4	6679	0
XM_020085066.1	neurexin-3a isoform X1	5946	0
XM_020085067.1	neurexin-3a isoform X1	4448	0
XM_020085068.1	neurexin-3-beta isoform X7	3803	0
XM_020085069.1	neuronal acetylcholine receptor subunit beta-2-like isoform X2	4253	0
XM_020085070.1	neurexin-3a-beta isoform X8	4439	0
XM_020085071.1	neurexin-3a-beta isoform X9	3812	0
XM_020085072.1	zinc finger protein 292	7602	0
XM_020085073.1	peroxidasin homolog isoform X1	7682	0
XM_020085074.1	peroxidasin homolog isoform X2	7610	0
XM_020085075.1	probable palmitoyltransferase ZDHHC14 isoform X1	2750	0
XM_020085076.1	probable palmitoyltransferase ZDHHC14 isoform X2	2705	0
XM_020085077.1	probable palmitoyltransferase ZDHHC14 isoform X1	2853	0
XM_020085078.1	peroxisome proliferator-activated receptor gamma coactivator 1-beta-like	5724	0
XM_020085079.1	RNA polymerase II-associated protein 1	4905	0
XM_020085080.1	RNA polymerase II-associated protein 1	4900	0
XM_020085081.1	RNA polymerase II-associated protein 1	4829	0
XM_020085082.1	transmembrane protein 151B-like	2244	0
XM_020085083.1	FAS-associated factor 2	2452	0
XM_020085084.1	eIF-2-alpha kinase GCN2	5678	0
XM_020085085.1	connector enhancer of kinase suppressor of ras 3	2831	0
XM_020085086.1	interactor protein for cytohesin exchange factors 1 isoform X1	2546	0
XM_020085087.1	interactor protein for cytohesin exchange factors 1 isoform X2	2485	0
XM_020085088.1	interactor protein for cytohesin exchange factors 1 isoform X3	2454	0
XM_020085089.1	interactor protein for cytohesin exchange factors 1 isoform X2	2669	0
XM_020085090.1	ADP-ribosylation factor 6	2432	5.14E-122
XM_020085091.1	ena/VASP-like protein isoform X1	4533	0
XM_020085092.1	ena/VASP-like protein isoform X2	4667	0
XM_020085093.1	ena/VASP-like protein isoform X3	4772	0
XM_020085094.1	ena/VASP-like protein isoform X4	4278	0
XM_020085095.1	ena/VASP-like protein isoform X5	3600	0
XM_020085096.1	sphingolipid delta(4)-desaturase/C4-monooxygenase DES2	2123	0
XM_020085097.1	SPARC-like protein 1	1736	0
XM_020085098.1	ribosomal protein S6 kinase alpha-1 isoform X1	4300	0
XM_020085099.1	ribosomal protein S6 kinase 2 alpha isoform X2	4565	0
XM_020085100.1	ribosomal protein S6 kinase alpha-1 isoform X1	3880	0

XM_020085101.1	ornithine decarboxylase-like	3030	0
XM_020085102.1	arf-GAP with SH3 domain, ANK repeat and PH domain-containing protein 2 isoform X1	5151	0
XM_020085103.1	arf-GAP with SH3 domain, ANK repeat and PH domain-containing protein 2 isoform X2	5148	0
XM_020085104.1	disheveled-associated activator of morphogenesis 2 isoform X1	3655	0
XM_020085105.1	disheveled-associated activator of morphogenesis 2 isoform X2	3589	0
XM_020085106.1	SPARC-like protein 1	1277	0
XM_020085107.1	disheveled-associated activator of morphogenesis 2 isoform X2	3559	0
XM_020085108.1	abscission/NoCut checkpoint regulator	3372	0
XM_020085109.1	mitotic checkpoint serine/threonine-protein kinase BUB1 beta	2241	0
XM_020085110.1	protein phosphatase 1 regulatory subunit 14B-like	2013	2.15E-55
XM_020085111.1	mitochondrial fission regulator 1-like	3427	2.01E-178
XM_020085112.1	mitochondrial fission regulator 1-like	3462	2.72E-178
XM_020085113.1	reticulon-1 isoform X1	3405	0
XM_020085114.1	reticulon-1 isoform X2	1059	4.17E-125
XM_020085115.1	reticulon-1 isoform X3	980	1.86E-112
XM_020085116.1	arf-GAP with SH3 domain, ANK repeat and PH domain-containing protein 1-like isoform X1	4685	0
XM_020085117.1	arf-GAP with SH3 domain, ANK repeat and PH domain-containing protein 3-like isoform X2	4682	0
XM_020085118.1	arf-GAP with SH3 domain, ANK repeat and PH domain-containing protein 1-like isoform X3	4588	0
XM_020085119.1	WD40 repeat-containing protein SMU1	2558	0
XM_020085120.1	tubulin polyglutamylase TTLL5 isoform X1	6745	0

XM_020085121.1	tubulin polyglutamylase TTLL5 isoform X2	6703	0
XM_020085122.1	transforming growth factor beta-3	3766	0
XM_020085123.1	WD repeat-containing protein 43	2910	0
XM_020085124.1	transcription factor E2F2	3493	0
XM_020085125.1	DNA-binding protein inhibitor ID-3	1235	3.92E-79
XM_020085126.1	mitogen-activated protein kinase kinase kinase 7-like isoform X1	3142	0
XM_020085127.1	mitogen-activated protein kinase kinase kinase 7-like isoform X2	3140	0
XM_020085128.1	dual adapter for phosphotyrosine and 3-phosphotyrosine and 3-phosphoinositide	2691	0
XM_020085129.1	zinc finger FYVE domain-containing protein 1-like	5294	0
XM_020085130.1	ADP/ATP translocase 3	1270	0
XM_020085131.1	chloride intracellular channel protein 4	3100	2.69E-178
XM_020085132.1	transcription factor SOX-11	3282	0
XM_020085133.1	glycine N-methyltransferase	3552	0
XM_020085134.1	rho guanine nucleotide exchange factor 10 isoform X1	5489	0
XM_020085135.1	rho guanine nucleotide exchange factor 10 isoform X2	5372	0
XM_020085136.1	NF-kappa-B inhibitor alpha-like	1623	0
XM_020085137.1	zinc finger protein DPF3-like	1893	0
XM_020085138.1	leucine-rich repeat, immunoglobulin-like domain and transmembrane domain-containing protein 3 isoform X1	1785	0
XM_020085139.1	leucine-rich repeat, immunoglobulin-like domain and transmembrane domain-containing protein 3 isoform X2	1776	0
XM_020085140.1	leucine-rich repeat, immunoglobulin-like domain and transmembrane domain-containing protein 3 isoform X3	1431	0
XM_020085141.1	JNK1/MAPK8-associated membrane protein	2357	0
XM_020085142.1	MICOS complex subunit MIC60	2814	0
XM_020085143.1	PREDICTED: uncharacterized protein C4orf32 homolog	3998	1.92E-61
XM_020085144.1	apolipoprotein B-100-like	3669	0
XM_020085145.1	tonsoku-like protein	2814	0
XM_020085146.1	glutamyl-tRNA(Gln) amidotransferase subunit A, mitochondrial	3411	0
XM_020085147.1	reticulon-4-interacting protein 1, mitochondrial	1535	0
XM_020085148.1	solute carrier family 25 member 45-like	3175	0
XM_020085149.1	MAGUK p55 subfamily member 5 isoform X1	2534	0
XM_020085150.1	nutritionally-regulated adipose and cardiac enriched protein homolog isoform X1	3297	0
XM_020085151.1	nutritionally-regulated adipose and cardiac enriched protein homolog isoform X1	3324	0
XM_020085152.1	nutritionally-regulated adipose and cardiac enriched protein homolog isoform X1	3214	0
XM_020085153.1	nutritionally-regulated adipose and cardiac enriched protein homolog isoform X1	3180	0
XM_020085154.1	RING finger protein 17	2295	0
XM_020085155.1	nutritionally-regulated adipose and cardiac enriched protein homolog isoform X1	2940	0
XM_020085156.1	nutritionally-regulated adipose and cardiac enriched protein homolog isoform X1	2758	0
XM_020085157.1	nutritionally-regulated adipose and cardiac enriched protein homolog isoform X1	3270	0
XM_020085158.1	transmembrane protein 179	1559	1.21E-156
XM_020085159.1	BSD domain-containing protein 1 isoform X1	2878	0
XM_020085160.1	BSD domain-containing protein 1 isoform X1	2550	0
XM_020085161.1	leucine-rich repeat-containing protein 32-like isoform X2	2904	0
XM_020085162.1	PREDICTED: pinin	2623	0
XM_020085163.1	galectin-8-like isoform X1	2464	0
XM_020085164.1	galectin-8-like isoform X2	2344	0
XM_020085165.1	interferon alpha-inducible protein 27-like protein 2	569	7.65E-21
XM_020085166.1	E3 ubiquitin-protein ligase RNF144B	5105	0
XM_020085167.1	PREDICTED: gremlin-1-like	818	1.23E-108
XM_020085168.1	leucine-rich repeat-containing protein 32-like isoform X2	2898	0
XM_020085169.1	low density lipoprotein receptor adapter protein 1	2290	0
XM_020085170.1	arginine/serine-rich protein 1-like	1391	1.68E-110
XM_020085171.1	transforming acidic coiled-coil-containing protein 3	2841	0
XM_020085172.1	MARCKS-related protein-like	1725	1.16E-39
XM_020085173.1	lateral signaling target protein 2 homolog isoform X1	5198	0
XM_020085174.1	lateral signaling target protein 2 homolog isoform X2	5195	0
XM_020085175.1	lateral signaling target protein 2 homolog isoform X3	5102	0
XM_020085176.1	ral GTPase-activating protein subunit alpha-2 isoform X1	6141	0
XM_020085177.1	ral GTPase-activating protein subunit alpha-2 isoform X2	6081	0
XM_020085178.1	molybdenum cofactor biosynthesis protein 1 isoform X1	3842	0
XM_020085179.1	molybdenum cofactor biosynthesis protein 1 isoform X1	3733	0

XM_020085180.1	molybdenum cofactor biosynthesis protein 1 isoform X1	1599	0
XM_020085181.1	gap junction beta-1 protein-like	3074	0
XM_020085182.1	protein O-mannosyl-transferase 2	3178	0
XM_020085183.1	protein CLN8	2582	0
XM_020085184.1	echinoderm microtubule-associated protein-like 1 isoform X1	4859	0
XM_020085185.1	echinoderm microtubule-associated protein-like 1 isoform X2	4832	0
XM_020085186.1	echinoderm microtubule-associated protein-like 1 isoform X3	4413	0
XM_020085187.1	echinoderm microtubule-associated protein-like 1 isoform X4	4802	0
XM_020085188.1	echinoderm microtubule-associated protein-like 1 isoform X1	4381	0
XM_020085189.1	echinoderm microtubule-associated protein-like 1 isoform X6	4775	0
XM_020085190.1	echinoderm microtubule-associated protein-like 1 isoform X8	4108	0
XM_020085191.1	gap junction beta-1 protein-like	3068	0
XM_020085192.1	echinoderm microtubule-associated protein-like 1 isoform X8	4913	0
XM_020085193.1	protein kinase C eta type	2547	0
XM_020085194.1	protein kinase C eta type	2446	0
XM_020085195.1	lysophospholipid acyltransferase 2	3547	0
XM_020085196.1	protein L-Myc-1b-like	2148	0
XM_020085197.1	dimethyladenosine transferase 1, mitochondrial	1709	0
XM_020085198.1	dimethyladenosine transferase 1, mitochondrial	1757	0
XM_020085199.1	dimethyladenosine transferase 1, mitochondrial	1681	0
XM_020085200.1	homeobox protein CDX-4	1641	0
XM_020085201.1	PREDICTED: claudin-20	4661	2.33E-133
XM_020085202.1	PREDICTED: claudin-20	2567	4.30E-139
XM_020085203.1	kunitz-type protease inhibitor 1	2742	0
XM_020085204.1	histidine--tRNA ligase, cytoplasmic isoform X1	2522	0
XM_020085205.1	G patch domain-containing protein 2-like isoform X1	4712	0
XM_020085206.1	G patch domain-containing protein 2-like isoform X2	4708	0
XM_020085207.1	feline leukemia virus subgroup C receptor-related protein 2 isoform X1	4151	0
XM_020085208.1	feline leukemia virus subgroup C receptor-related protein 2 isoform X1	3948	0
XM_020085209.1	feline leukemia virus subgroup C receptor-related protein 2 isoform X1	3964	0
XM_020085210.1	feline leukemia virus subgroup C receptor-related protein 2 isoform X1	4040	0
XM_020085211.1	probable ergosterol biosynthetic protein 28	1504	2.96E-97
XM_020085212.1	isovaleryl-CoA dehydrogenase, mitochondrial	2128	0
XM_020085213.1	histidine--tRNA ligase, cytoplasmic isoform X2	2167	0
XM_020085214.1	gamma-aminobutyric acid receptor subunit rho-1-like isoform X1	2271	0
XM_020085215.1	gamma-aminobutyric acid receptor subunit rho-1-like isoform X2	2568	0
XM_020085216.1	peptidase M20 domain-containing protein 2	3021	0
XM_020085217.1	dihydrolipoyllysine-residue succinyltransferase component of 2-oxoglutarate dehydrogenase complex, mitochondrial-like	2117	0
XM_020085218.1	grainyhead-like protein 1 homolog isoform X1	3299	0
XM_020085219.1	grainyhead-like protein 1 homolog isoform X2	3293	0
XM_020085220.1	protein tyrosine phosphatase type IVA 2-like	3513	8.83E-114
XM_020085221.1	protein tyrosine phosphatase type IVA 2-like	3754	2.79E-113
XM_020085222.1	protein tyrosine phosphatase type IVA 2-like	3511	8.70E-114
XM_020085223.1	kinesin-like protein KIF15	4783	0
XM_020085224.1	sushi domain-containing protein 4-like isoform X1	3424	0
XM_020085225.1	sushi domain-containing protein 4-like isoform X2	3417	0
XM_020085226.1	serine/threonine-protein phosphatase 2A 55 kDa regulatory subunit B beta isoform isoform X1	3464	0
XM_020085227.1	fibrous sheath-interacting protein 1	1408	0
XM_020085228.1	potassium channel subfamily K member 5	2472	0
XM_020085229.1	extracellular tyrosine-protein kinase PKDCC	2449	0
XM_020085230.1	BEN domain-containing protein 3	4153	0
XM_020085231.1	BEN domain-containing protein 3	4403	0
XM_020085232.1	PREDICTED: uncharacterized protein C6orf203 homolog isoform X1	1217	3.77E-141
XM_020085233.1	PREDICTED: uncharacterized protein C6orf203 homolog isoform X1	1150	8.26E-141
XM_020085234.1	serine/threonine-protein phosphatase 2A 55 kDa regulatory subunit B beta isoform isoform X2	3869	0
XM_020085235.1	PREDICTED: uncharacterized protein C6orf203 homolog isoform X1	1094	4.10E-141
XM_020085236.1	bromo adjacent homology domain-containing 1 protein	4155	0
XM_020085237.1	bromo adjacent homology domain-containing 1 protein	3781	0
XM_020085238.1	leukocyte tyrosine kinase receptor-like isoform X1	6196	0
XM_020085239.1	leukocyte tyrosine kinase receptor-like isoform X2	6193	0
XM_020085240.1	mis18-binding protein 1	3867	0
XM_020085241.1	DNA mismatch repair protein Mlh3 isoform X1	4181	0
XM_020085242.1	DNA mismatch repair protein Mlh3 isoform X2	4151	0
XM_020085243.1	DNA mismatch repair protein Mlh3 isoform X3	4049	0
XM_020085244.1	calcium permeable stress-gated cation channel 1	6868	0
XM_020085245.1	60S ribosomal protein L13a isoform X1	715	1.80E-119
XM_020085246.1	60S ribosomal protein L13a isoform X1	750	4.82E-116
XM_020085247.1	probable E3 ubiquitin-protein ligase MID2 isoform X1	6188	0
XM_020085248.1	putative ATP-dependent RNA helicase TDRD9 isoform X1	4425	0
XM_020085249.1	putative ATP-dependent RNA helicase TDRD9 isoform X2	4422	0
XM_020085250.1	protein RD3-like	1384	1.55E-150
XM_020085251.1	UPF0317 protein C14orf159 homolog, mitochondrial isoform X1	3228	0
XM_020085252.1	UPF0317 protein C14orf159 homolog, mitochondrial isoform X2	3320	0
XM_020085253.1	nucleolar protein 10	3500	0
XM_020085254.1	tetratricopeptide repeat protein 8	1731	0
XM_020085255.1	probable E3 ubiquitin-protein ligase MID2 isoform X1	5772	0
XM_020085256.1	C-type lectin domain family 4 member M-like	1284	0
XM_020085257.1	C-type lectin domain family 4 member M-like	1129	0
XM_020085258.1	C-type lectin domain family 4 member M-like	1134	0

XM_020085259.1	CD209 antigen-like protein E	1179	0
XM_020085260.1	transmembrane protein 260	4065	0
XM_020085261.1	PREDICTED: akirin-2	1396	3.26E-113
XM_020085262.1	SERTA domain-containing protein 4-like	4251	0
XM_020085263.1	probable E3 ubiquitin-protein ligase MID2 isoform X1	6127	0
XM_020085264.1	collectin-11 isoform X1	1470	1.35E-180
XM_020085265.1	collectin-11 isoform X2	967	1.28E-178
XM_020085266.1	PREDICTED: neuroglobin-like	1213	2.91E-119
XM_020085267.1	signal recognition particle 14 kDa protein	1479	1.66E-61
XM_020085268.1	nucleic acid dioxygenase ALKBH1	1849	0
XM_020085269.1	heterogeneous nuclear ribonucleoprotein D0-like	641	7.90E-70
XM_020085270.1	tryptophan--tRNA ligase, cytoplasmic	2022	0
XM_020085271.1	probable E3 ubiquitin-protein ligase MID2 isoform X1	5632	0
XM_020085272.1	PREDICTED: uncharacterized protein C15orf52 homolog isoform X1	2070	0
XM_020085273.1	PREDICTED: uncharacterized protein C15orf52 homolog isoform X2	2067	0
XM_020085274.1	PREDICTED: uncharacterized protein C15orf52 homolog isoform X3	2240	0
XM_020085275.1	PREDICTED: uncharacterized protein C15orf52 homolog isoform X4	2237	0
XM_020085276.1	PREDICTED: kininogen-1-like	489	9.05E-42
XM_020085277.1	CAP-Gly domain-containing linker protein 3-like	3891	0
XM_020085278.1	probable E3 ubiquitin-protein ligase MID2 isoform X1	6122	0
XM_020085279.1	NIPA-like protein 3	2226	0
XM_020085280.1	complement component 1 Q subcomponent-binding protein, mitochondrial	1626	0
XM_020085281.1	RING finger protein 121	1471	0
XM_020085282.1	roundabout homolog 4 isoform X1	4612	0
XM_020085283.1	roundabout homolog 4 isoform X2	4308	0
XM_020085284.1	putative monooxygenase p33MONOX isoform X1	2318	0
XM_020085285.1	coiled-coil domain-containing protein 61 isoform X1	2963	0
XM_020085286.1	coiled-coil domain-containing protein 61 isoform X2	2943	0
XM_020085287.1	acetyl-CoA acetyltransferase, mitochondrial	1661	0
XM_020085288.1	disks large homolog 2-like	1937	0
XM_020085289.1	disks large homolog 1-like	1900	6.07E-175
XM_020085290.1	zinc finger protein-like 1	1895	0
XM_020085291.1	pleckstrin homology-like domain family B member 2 isoform X1	5206	0
XM_020085292.1	putative monooxygenase p33MONOX isoform X1	2348	0
XM_020085293.1	pleckstrin homology-like domain family B member 2 isoform X2	5203	0
XM_020085294.1	GTPase Era, mitochondrial	1880	0
XM_020085295.1	PREDICTED: uncharacterized protein CXorf57 homolog isoform X1	3260	0
XM_020085296.1	PREDICTED: uncharacterized protein CXorf57 homolog isoform X2	3150	0
XM_020085297.1	neuroligin-3-like isoform X1	3919	0
XM_020085298.1	neuroligin-3-like isoform X2	3859	0
XM_020085299.1	PREDICTED: neuroligin-2-like	5172	0
XM_020085300.1	GDNF family receptor alpha-4-like isoform X1	5324	0
XM_020085301.1	putative monooxygenase p33MONOX isoform X1	2363	0
XM_020085302.1	GDNF family receptor alpha-4-like isoform X1	4189	0
XM_020085303.1	ras-related GTP-binding protein A	1754	0
XM_020085304.1	ras-related GTP-binding protein A	1622	0
XM_020085305.1	SH2 domain-containing protein 4A-like	2514	0
XM_020085306.1	SH2 domain-containing protein 4A-like	2552	0
XM_020085307.1	dehydrogenase/reductase SDR family member 11	1521	2.41E-131
XM_020085308.1	endothelial cell-selective adhesion molecule	4021	0
XM_020085309.1	putative monooxygenase p33MONOX isoform X1	2159	4.15E-179
XM_020085310.1	spectrin beta chain, non-erythrocytic 4-like	9021	0
XM_020085311.1	sodium/calcium exchanger 1-like	5094	0
XM_020085312.1	pituitary homeobox 1 isoform X1	1879	0
XM_020085313.1	pituitary homeobox 1 isoform X1	1769	0
XM_020085314.1	pituitary homeobox 1 isoform X3	1725	2.10E-158
XM_020085315.1	roundabout homolog 2-like	5201	0
XM_020085316.1	alkaline ceramidase 3	3109	3.77E-155
XM_020085317.1	transcription cofactor vestigial-like protein 1	1950	1.25E-139
XM_020085318.1	putative monooxygenase p33MONOX isoform X1	1921	3.03E-180
XM_020085319.1	sodium/calcium exchanger 2-like isoform X1	4430	0
XM_020085320.1	sodium/calcium exchanger 2-like isoform X2	4412	0
XM_020085321.1	serine/threonine-protein kinase Chk1	2032	0
XM_020085322.1	PREDICTED: kaptin	1928	0
XM_020085323.1	phosphatidylinositol 3,4,5-trisphosphate 3-phosphatase TPTE2-like isoform X1	2097	0
XM_020085324.1	phosphatidylinositol 3,4,5-trisphosphate 3-phosphatase TPTE2-like isoform X1	2052	0
XM_020085325.1	phosphatidylinositol 3,4,5-trisphosphate 3-phosphatase TPTE2-like isoform X1	1652	0
XM_020085326.1	phosphatidylinositol 3,4,5-trisphosphate 3-phosphatase TPTE2-like isoform X1	1933	0
XM_020085327.1	fibrinogen-like protein 1	1531	0
XM_020085328.1	fibrinogen-like protein 1	1526	0
XM_020085329.1	fibrinogen-like protein 1	1377	0
XM_020085330.1	DEAD box protein 41	2170	0
XM_020085331.1	AP-1 complex-associated regulatory protein isoform X1	2191	0
XM_020085332.1	PREDICTED: sedoheptulokinase	3266	0
XM_020085333.1	BTB/POZ domain-containing protein KCTD7	5072	0
XM_020085334.1	PREDICTED: trichohyalin-like	4012	0
XM_020085335.1	galactosylgalactosylxylosylprotein 3-beta-glucuronosyltransferase 1 isoform X2	3293	0
XM_020085336.1	beta-galactosidase-1-like protein 2	1918	0
XM_020085337.1	pleckstrin homology-like domain family B member 1 isoform X2	5019	0

XM_020085338.1	AP-1 complex-associated regulatory protein isoform X2	2095	0
XM_020085339.1	pleckstrin homology-like domain family B member 1 isoform X2	5141	0
XM_020085340.1	pleckstrin homology-like domain family B member 1 isoform X2	5066	0
XM_020085341.1	pleckstrin homology-like domain family B member 1 isoform X3	4896	0
XM_020085342.1	pleckstrin homology-like domain family B member 1 isoform X2	4890	0
XM_020085343.1	pleckstrin homology-like domain family B member 1 isoform X2	4881	0
XM_020085344.1	pleckstrin homology-like domain family B member 1 isoform X2	4832	0
XM_020085345.1	pleckstrin homology-like domain family B member 1 isoform X7	4458	0
XM_020085346.1	protein FAM46C-like	3867	0
XM_020085347.1	protein FAM46C-like	4200	0
XM_020085348.1	procollagen C-endopeptidase enhancer 2-like	1837	0
XM_020085349.1	coiled-coil alpha-helical rod protein 1	3727	0
XM_020085350.1	microtubule-associated protein 6 homolog isoform X1	2200	0
XM_020085351.1	microtubule-associated protein 6 homolog isoform X2	2088	0
XM_020085352.1	glutamine--fructose-6-phosphate aminotransferase [isomerizing] 2	3249	0
XM_020085353.1	microtubule-associated protein 6 homolog isoform X3	2051	0
XM_020085354.1	heat shock protein beta-1	1465	4.82E-116
XM_020085355.1	heat shock protein beta-1	1373	1.10E-115
XM_020085356.1	CXXC-type zinc finger protein 5-like isoform X1	2734	1.59E-176
XM_020085357.1	CXXC-type zinc finger protein 5-like isoform X1	2594	4.10E-177
XM_020085358.1	CXXC-type zinc finger protein 5-like isoform X1	2521	1.94E-177
XM_020085359.1	CXXC-type zinc finger protein 5-like isoform X1	2536	2.25E-177
XM_020085360.1	CXXC-type zinc finger protein 5-like isoform X1	2514	1.82E-177
XM_020085361.1	CXXC-type zinc finger protein 5-like isoform X1	2706	1.22E-176
XM_020085362.1	CXXC-type zinc finger protein 5-like isoform X1	2529	2.12E-177
XM_020085363.1	CXXC-type zinc finger protein 5-like isoform X1	2727	9.08E-176
XM_020085364.1	rho GTPase-activating protein 32 isoform X1	5974	0
XM_020085365.1	voltage-gated potassium channel subunit beta-3-like	4440	0
XM_020085366.1	FERM and PDZ domain-containing protein 1-like isoform X1	7613	0

XM_020085367.1	ATP-binding cassette sub-family G member 4	3222	0
XM_020085368.1	transcobalamin-1-like isoform X1	660	2.52E-101
XM_020085369.1	transcobalamin-1-like isoform X1	642	1.39E-82
XM_020085370.1	PREDICTED: uncharacterized protein LOC109628317 isoform X1	1761	2.87E-108
XM_020085371.1	PREDICTED: uncharacterized protein LOC109628317 isoform X1	1800	2.89E-97
XM_020085372.1	probable ribonuclease ZC3H12B	4585	0
XM_020085373.1	probable ribonuclease ZC3H12B	5010	0
XM_020085374.1	dixin isoform X1	3472	0
XM_020085375.1	FERM and PDZ domain-containing protein 1-like isoform X2	7559	0
XM_020085376.1	dixin isoform X2	3454	0
XM_020085377.1	dixin isoform X3	3448	0
XM_020085378.1	PREDICTED: uncharacterized protein C11orf87 homolog	2888	3.80E-79
XM_020085379.1	gamma-butyrobetaine dioxygenase	1868	0
XM_020085380.1	glucose-6-phosphate exchanger SLC37A2	2291	0
XM_020085381.1	mannose-P-dolichol utilization defect 1 protein-like	2078	5.16E-139
XM_020085382.1	MAP7 domain-containing protein 2-like isoform X1	5150	0
XM_020085383.1	MAP7 domain-containing protein 2-like isoform X2	5151	0
XM_020085384.1	MAP7 domain-containing protein 2-like isoform X3	5142	0
XM_020085385.1	MAP7 domain-containing protein 2-like isoform X4	5125	0
XM_020085386.1	MAP7 domain-containing protein 2-like isoform X4	5061	0
XM_020085387.1	DNA-(apurinic or apyrimidinic site) lyase 2	2208	0
XM_020085388.1	myb/SANT-like DNA-binding domain-containing protein 2 isoform X1	3444	0
XM_020085389.1	myb/SANT-like DNA-binding domain-containing protein 2 isoform X2	3408	0
XM_020085390.1	protein Jade-1	5215	0
XM_020085391.1	histone H4 transcription factor-like	2078	0
XM_020085392.1	collagenase 3-like	1648	0
XM_020085393.1	intraflagellar transport protein 57 homolog	1790	0
XM_020085394.1	solute carrier family 22 member 6	2192	0
XM_020085395.1	solute carrier family 22 member 6	2116	0
XM_020085396.1	solute carrier family 22 member 6	2873	0
XM_020085397.1	protein NLRC3-like	3067	0
XM_020085398.1	E3 ubiquitin-protein ligase RNF167-like	2099	0
XM_020085399.1	isoaspartyl peptidase/L-asparaginase-like	1361	0
XM_020085400.1	transcription elongation factor SPT4 isoform X1	673	1.24E-84
XM_020085402.1	serine/arginine repetitive matrix protein 3 isoform X1	3976	0
XM_020085403.1	serine/arginine repetitive matrix protein 3 isoform X2	3925	0
XM_020085404.1	serine/arginine repetitive matrix protein 3 isoform X1	3873	0
XM_020085405.1	transcription cofactor vestigial-like protein 1	1861	4.92E-140
XM_020085406.1	protein phosphatase 1 regulatory subunit 14A-like	1867	6.31E-117
XM_020085407.1	protein phosphatase 1 regulatory subunit 14A-like	1864	7.53E-117
XM_020085408.1	seizure protein 6 homolog	3350	0
XM_020085409.1	carbohydrate-responsive element-binding protein-like isoform X1	2876	0
XM_020085410.1	carbohydrate-responsive element-binding protein-like isoform X2	2873	0
XM_020085411.1	interferon regulatory factor 1-like isoform X1	1730	0
XM_020085412.1	interferon regulatory factor 1-like isoform X2	1718	0
XM_020085413.1	GTP-binding nuclear protein Ran	1121	3.98E-156
XM_020085414.1	probable palmitoyltransferase ZDHHC20	2517	0
XM_020085415.1	protein BTG3-like	1163	1.49E-172
XM_020085416.1	protein BTG3-like	1076	4.91E-173
XM_020085417.1	integrin-linked protein kinase-like	1879	0

XM_020085418.1	glycerophosphodiester phosphodiesterase domain-containing protein 5-like isoform X1	3397	0
XM_020085419.1	glycerophosphodiester phosphodiesterase domain-containing protein 5-like isoform X2	3388	0
XM_020085420.1	glycerophosphodiester phosphodiesterase domain-containing protein 5-like isoform X3	3381	0
XM_020085421.1	GTP-binding nuclear protein Ran	1101	3.06E-156
XM_020085422.1	diacylglycerol O-acyltransferase 2-like	1614	0
XM_020085423.1	G protein-activated inward rectifier potassium channel 3-like	2351	0
XM_020085424.1	sodium/potassium/calcium exchanger 3-like	4509	0
XM_020085425.1	complement factor B-like	2692	0
XM_020085426.1	tricarboxylate transport protein, mitochondrial-like	2895	0
XM_020085427.1	2-acylglycerol O-acyltransferase 2	1333	0
XM_020085428.1	coiled-coil domain-containing protein 15 isoform X1	1960	0
XM_020085429.1	coiled-coil domain-containing protein 15 isoform X2	1831	0
XM_020085430.1	mitochondrial uncoupling protein 2-like	1901	0
XM_020085431.1	gap junction beta-1 protein-like	2338	5.21E-163
XM_020085432.1	gap junction beta-1 protein-like	2369	1.79E-167
XM_020085433.1	protein shisa-3 homolog	1737	5.40E-136
XM_020085434.1	zinc finger SWIM domain-containing protein 7	2990	8.14E-90
XM_020085435.1	opioid-binding protein/cell adhesion molecule-like isoform X1	4353	0
XM_020085436.1	opioid-binding protein/cell adhesion molecule homolog isoform X2	4130	0
XM_020085437.1	homeobox protein PKNOX2 isoform X1	3391	0
XM_020085438.1	homeobox protein PKNOX2 isoform X1	3267	0
XM_020085439.1	homeobox protein PKNOX2 isoform X1	3376	0
XM_020085440.1	homeobox protein PKNOX2 isoform X1	3244	0
XM_020085441.1	homeobox protein PKNOX2 isoform X1	3232	0
XM_020085442.1	neuronal tyrosine-phosphorylated phosphoinositide-3-kinase adapter 2 isoform X1	5440	0
XM_020085443.1	heat shock protein beta-1	1427	7.63E-115
XM_020085444.1	fructose-bisphosphate aldolase B	1472	0
XM_020085445.1	melanoma-derived growth regulatory protein	1142	2.17E-81
XM_020085446.1	beta,beta-carotene 9',10'-oxygenase-like	3110	0
XM_020085447.1	carbonic anhydrase 4-like	2629	0
XM_020085448.1	gap junction delta-2 protein-like	3738	0
XM_020085449.1	reticulon-2-like isoform X3	2779	1.83E-106

XM_020085450.1	Purkinje cell protein 4-like isoform X1	1220	2.91E-33
XM_020085451.1	Purkinje cell protein 4-like isoform X2	1187	2.67E-25
XM_020085452.1	cyclic AMP-dependent transcription factor ATF-4-like	3593	0
XM_020085453.1	tumor necrosis factor alpha-induced protein 2-like	3487	0
XM_020085454.1	protein cornichon homolog 2 isoform X1	4007	2.84E-90
XM_020085455.1	probable palmitoyltransferase ZDHHC20	2589	0
XM_020085456.1	protein cornichon homolog 2 isoform X2	4500	5.50E-79
XM_020085457.1	oligodendrocyte-myelin glycoprotein-like	2660	0
XM_020085458.1	homeodomain-interacting protein kinase 4-like	1443	0
XM_020085459.1	15-hydroxyprostaglandin dehydrogenase [NAD(+)]-like	496	7.50E-103
XM_020085460.1	POU domain, class 2, transcription factor 3	2908	0
XM_020085461.1	nuclear envelope pore membrane protein POM 121-like	2003	0
XM_020085462.1	G protein-activated inward rectifier potassium channel 3-like	1601	0
XM_020085463.1	probable palmitoyltransferase ZDHHC20	2544	0
XM_020085464.1	inositol polyphosphate 5-phosphatase K-like	1625	0
XM_020085465.1	PREDICTED: uncharacterized protein LOC109628382	738	3.35E-150
XM_020085466.1	multidrug and toxin extrusion protein 1-like	3046	0
XM_020085467.1	short transient receptor potential channel 3-like	3097	0
XM_020085468.1	cilia- and flagella-associated protein 54	8266	0
XM_020085469.1	adhesion G-protein coupled receptor G2-like	4115	0
XM_020085470.1	APC membrane recruitment protein 2-like	4488	0
XM_020085471.1	latent-transforming growth factor beta-binding protein 3	4752	0
XM_020085472.1	signal-induced proliferation-associated 1-like protein 1	3214	0
XM_020085473.1	CCR4-NOT transcription complex subunit 7	2423	0
XM_020085474.1	histone acetyltransferase KAT5	1908	0
XM_020085475.1	sorting nexin-19-like	2400	0
XM_020085476.1	ubiquitin-associated and SH3 domain-containing protein B-like	3664	0
XM_020085477.1	PREDICTED: uncharacterized protein LOC109628394	2534	1.01E-141
XM_020085478.1	retinal guanylyl cyclase 2-like	3606	0
XM_020085479.1	olfactory receptor 52D1-like	942	0
XM_020085480.1	transcription cofactor vestigial-like protein 1	1959	1.37E-139
XM_020085481.1	sodium-dependent noradrenaline transporter	2627	0
XM_020085482.1	SLAIN motif-containing protein-like	1410	0
XM_020085483.1	neuroligin-3-like isoform X1	811	3.04E-122
XM_020085484.1	glutamate receptor 3-like	2077	3.43E-145
XM_020085485.1	magnesium transporter protein 1	1989	0
XM_020085486.1	PREDICTED: uncharacterized protein LOC109628402	711	1.37E-153
XM_020085487.1	Down syndrome cell adhesion molecule-like protein 1 homolog	6051	0
XM_020085488.1	protein phosphatase methylesterase 1	1339	0
XM_020085489.1	olfactory receptor-like protein OLF4	924	0
XM_020085490.1	glycine receptor subunit alpha-2-like	1434	0
XM_020085491.1	T-box transcription factor TBX2b-like	1911	0
XM_020085492.1	protocadherin beta-15-like isoform X1	2811	0
XM_020085493.1	Kv channel-interacting protein 1-like	790	2.00E-169
XM_020085494.1	V-set and immunoglobulin domain-containing protein 1-like	924	0
XM_020085495.1	vacuolar protein sorting-associated protein 51 homolog	2471	0
XM_020085496.1	trichohyalin-like isoform X1	6349	0

XM_020085497.1	sodium- and chloride-dependent GABA transporter ine-like	1863	0
XM_020085498.1	putative ATP-dependent RNA helicase DHX33	1322	0
XM_020085499.1	protein spinster homolog 3-like	1932	0
XM_020085500.1	vacuole membrane protein 1-like	4387	0
XM_020085501.1	janus kinase and microtubule-interacting protein 1-like isoform X2	2814	0
XM_020085502.1	PR domain zinc finger protein 15	3208	0
XM_020085503.1	uromodulin-like 1	3628	0
XM_020085504.1	protein Wnt-11-like	676	9.34E-66
XM_020085505.1	immunoglobulin superfamily member 5-like	1368	0
XM_020085506.1	SAM domain-containing protein SAMSN-1-like	1738	0
XM_020085507.1	WSC domain-containing protein 1-like	2799	0
XM_020085508.1	trichohyalin-like isoform X3	7573	0
XM_020085509.1	cilia- and flagella-associated protein 58	2615	0
XM_020085510.1	cytotoxic and regulatory T-cell molecule-like	1151	0
XM_020085511.1	zona pellucida-like domain-containing protein 1	1264	0
XM_020085512.1	CD166 antigen	3731	0
XM_020085513.1	heat shock cognate 70 kDa protein	2317	0
XM_020085514.1	histone H2A	1841	1.39E-65
XM_020085515.1	histone H2B 5	527	8.72E-59
XM_020085516.1	heat shock cognate 71 kDa protein	1997	0
XM_020085517.1	proteasome assembly chaperone 1	1033	0
XM_020085518.1	myotubularin-related protein 7	3770	0
XM_020085519.1	collagenase 3-like	1526	0
XM_020085520.1	peroxiredoxin-5, mitochondrial	965	6.35E-130
XM_020085521.1	mitochondrial import inner membrane translocase subunit Tim22	1068	2.04E-117
XM_020085522.1	diamine acetyltransferase 2-like	1388	1.19E-127
XM_020085523.1	mitochondrial uncoupling protein 2-like isoform X1	934	9.53E-159
XM_020085524.1	mitochondrial uncoupling protein 2-like isoform X2	1135	0
XM_020085525.1	dnaJ homolog subfamily B member 13-like	971	0
XM_020085526.1	Meckel syndrome type 1 protein isoform X1	2382	0
XM_020085527.1	Meckel syndrome type 1 protein isoform X2	2379	0
XM_020085528.1	spliceosome-associated protein CWC15 homolog	1024	6.89E-106
XM_020085529.1	spliceosome-associated protein CWC15 homolog	997	4.06E-106
XM_020085530.1	mid1-interacting protein 1-B-like	1445	1.59E-116
XM_020085531.1	spliceosome-associated protein CWC15 homolog	1017	6.21E-106

XM_020085532.1	transmembrane protein 88-like	1277	3.75E-99
XM_020085533.1	transmembrane protein 88-like	1161	9.92E-100
XM_020085534.1	ester hydrolase C11orf54 homolog	1251	0
XM_020085535.1	thioredoxin domain-containing protein 15 isoform X1	1316	0
XM_020085536.1	thioredoxin domain-containing protein 15 isoform X2	1306	0
XM_020085537.1	class A basic helix-loop-helix protein 15	1711	5.46E-153
XM_020085538.1	LIM/homeobox protein Lhx1-like	2348	0
XM_020085539.1	erythrocyte band 7 integral membrane protein-like	1263	0
XM_020085540.1	pterin-4-alpha-carbinolamine dehydratase 2 isoform X1	622	2.61E-92
XM_020085541.1	pterin-4-alpha-carbinolamine dehydratase 2 isoform X2	726	6.80E-82
XM_020085542.1	membrane magnesium transporter 1	1153	4.79E-81
XM_020085543.1	transmembrane protease serine 2	1860	0
XM_020085544.1	transmembrane protease serine 2	1824	0
XM_020085545.1	transmembrane protease serine 2	1752	0
XM_020085546.1	very-long-chain 3-oxoacyl-CoA reductase-like	2118	0
XM_020085547.1	adenosine receptor A2b	1912	0
XM_020085548.1	adenosine receptor A2b	1938	0
XM_020085549.1	zinc finger protein 346 isoform X1	1532	0
XM_020085550.1	zinc finger protein 346 isoform X2	1529	0
XM_020085551.1	fibroblast growth factor 11-like isoform X1	814	0
XM_020085552.1	fibroblast growth factor 11-like isoform X1	576	3.72E-139
XM_020085553.1	transmembrane protein 218	1003	1.41E-34
XM_020085554.1	transcription cofactor vestigial-like protein 1	1904	7.86E-140
XM_020085555.1	prefoldin subunit 6	905	2.39E-73
XM_020085556.1	ATP synthase subunit g, mitochondrial	525	8.28E-72
XM_020085557.1	rhodopsin kinase-like	1828	0
XM_020085558.1	delta(14)-sterol reductase	1864	0
XM_020085560.1	mitochondrial uncoupling protein 2-like isoform X2	889	0
XM_020085561.1	GRB2-associated-binding protein 3	2165	0
XM_020085562.1	Na(+)/H(+) exchange regulatory cofactor NHE-RF3-like isoform X1	1832	0
XM_020085563.1	Na(+)/H(+) exchange regulatory cofactor NHE-RF3-like isoform X2	1829	0
XM_020085564.1	Na(+)/H(+) exchange regulatory cofactor NHE-RF3-like isoform X1	1791	0
XM_020085565.1	GS homeobox 1	1322	7.86E-162
XM_020085566.1	cytochrome c oxidase subunit 7B, mitochondrial-like	534	2.68E-53
XM_020085567.1	histone deacetylase 3	2422	0
XM_020085568.1	ependymin-1-like isoform X1	937	1.04E-161
XM_020085569.1	ependymin-1-like isoform X2	928	3.73E-159
XM_020085570.1	sodium channel subunit beta-4	1992	1.37E-156
XM_020085571.1	potassium channel subfamily K member 4-like isoform X1	2583	0
XM_020085572.1	potassium channel subfamily K member 4-like isoform X1	2571	0
XM_020085573.1	thrombospondin type-1 domain-containing protein 1	3338	0
XM_020085574.1	thrombospondin type-1 domain-containing protein 1	3267	0
XM_020085575.1	homeobox protein otx5-like	1758	2.76E-132
XM_020085576.1	transmembrane 4 L6 family member 1-like isoform X1	987	1.51E-115

XM_020085577.1	transmembrane 4 L6 family member 1-like isoform X2	862	8.22E-116
XM_020085578.1	protocadherin-1-like isoform X2	4010	0
XM_020085579.1	protein phosphatase 1B-like isoform X1	1718	0
XM_020085580.1	zinc transporter 9	3489	0
XM_020085581.1	protein phosphatase 1B-like isoform X2	1715	0
XM_020085582.1	chordin-like protein 2	2675	0
XM_020085583.1	leucine-rich repeat transmembrane protein FLRT1-like	3037	0
XM_020085584.1	dnaJ homolog subfamily B member 13-like	1010	0
XM_020085585.1	insulin receptor substrate 1-B-like	4075	0
XM_020085586.1	claudin-15-like isoform X1	1947	7.04E-153
XM_020085587.1	claudin-15-like isoform X2	1876	4.38E-161
XM_020085588.1	PREDICTED: nectin-1-like	2462	0
XM_020085589.1	synaptotagmin-like protein 2	4911	0
XM_020085590.1	pannexin-3 isoform X1	1787	0
XM_020085591.1	sodium bicarbonate transporter-like protein 11 isoform X1	3338	0
XM_020085592.1	pannexin-3 isoform X2	2026	0
XM_020085593.1	neurosecretory protein VGF-like	2380	0
XM_020085594.1	BICD family-like cargo adapter 2 isoform X1	1772	0
XM_020085595.1	BICD family-like cargo adapter 2 isoform X1	1791	0
XM_020085596.1	BICD family-like cargo adapter 2 isoform X1	1625	0
XM_020085597.1	uncharacterized serine/threonine-protein kinase SgK494	1801	0
XM_020085598.1	protein turtle homolog B-like	4454	0
XM_020085599.1	sodium bicarbonate transporter-like protein 11 isoform X2	3415	0
XM_020085600.1	PREDICTED: trichohyalin-like	2644	0
XM_020085601.1	jouberin isoform X1	4094	0
XM_020085602.1	U2 snRNP-associated SURP motif-containing protein-like	3166	0
XM_020085603.1	U2 snRNP-associated SURP motif-containing protein-like	3464	0
XM_020085604.1	U2 snRNP-associated SURP motif-containing protein-like	3086	0
XM_020085605.1	leucine-rich repeat and calponin homology domain-containing protein 2	4048	0
XM_020085606.1	kelch-like protein 13 isoform X1	2595	0
XM_020085607.1	kelch-like protein 13 isoform X2	2059	0
XM_020085608.1	hyaluronan mediated motility receptor	3678	0
XM_020085609.1	zinc finger protein OZF-like isoform X2	3563	0
XM_020085610.1	interleukin-13 receptor subunit alpha-2-like	1557	0
XM_020085611.1	LRRN4 C-terminal-like protein	2744	2.18E-152
XM_020085612.1	follistatin-related protein 4-like	2674	0
XM_020085613.1	PREDICTED: uncharacterized protein C11orf63 homolog	2490	0
XM_020085614.1	PREDICTED: uncharacterized protein C11orf63 homolog	2516	0

XM_020085615.1	histamine H2 receptor-like	1474	0
XM_020085616.1	macrophage metalloelastase-like	1562	0
XM_020085617.1	PREDICTED: uncharacterized protein LOC109628505	1903	0
XM_020085618.1	short transient receptor potential channel 2-like	3365	0
XM_020085619.1	15-hydroxyprostaglandin dehydrogenase [NAD(+)]-like	1044	0
XM_020085620.1	protein phosphatase 1E isoform X1	2091	0
XM_020085621.1	protein phosphatase 1E isoform X1	2001	0
XM_020085622.1	protein phosphatase 1E isoform X1	1947	0
XM_020085623.1	PREDICTED: lipocalin-like	759	5.08E-136
XM_020085624.1	cyclic nucleotide-gated olfactory channel	2174	0
XM_020085625.1	wiskott-Aldrich syndrome protein family member 3-like	1500	0
XM_020085626.1	lens fiber membrane intrinsic protein-like	774	2.28E-120
XM_020085627.1	lens fiber membrane intrinsic protein-like	730	1.33E-120
XM_020085628.1	testican-2-like isoform X1	2563	0
XM_020085629.1	inositol-trisphosphate 3-kinase A-like	792	5.49E-64
XM_020085630.1	PREDICTED: peripherin-2-like	1302	0
XM_020085631.1	palmitoyltransferase ZDHHC23-like	1386	0
XM_020085632.1	gap junction delta-2 protein-like	1095	0
XM_020085633.1	dual specificity protein phosphatase 10	2027	4.94E-158
XM_020085634.1	zona pellucida-like domain-containing protein 1	1242	0
XM_020085635.1	testican-2-like isoform X1	2551	0
XM_020085636.1	scavenger receptor cysteine-rich domain-containing group B protein-like	1868	0
XM_020085637.1	organic solute transporter subunit alpha-like	1138	0
XM_020085638.1	endonuclease domain-containing 1 protein-like	1128	0
XM_020085639.1	brain-specific homeobox protein homolog	860	1.35E-145
XM_020085640.1	interleukin-1 receptor accessory protein-like	1336	1.00E-85
XM_020085641.1	testican-2-like isoform X1	2498	0
XM_020085642.1	potassium channel subfamily K member 13-like	1407	0
XM_020085643.1	PREDICTED: uncharacterized protein LOC109628531	1212	0
XM_020085644.1	immunoglobulin superfamily member 11-like	976	0
XM_020085645.1	GPI transamidase component PIG-S isoform X1	2372	0
XM_020085646.1	GPI transamidase component PIG-S isoform X2	1772	0
XM_020085647.1	testican-2-like isoform X1	2465	0
XM_020085648.1	fructose-bisphosphate aldolase C	2339	0
XM_020085649.1	serine/arginine-rich splicing factor 9-like	2481	7.34E-104
XM_020085650.1	serine/arginine-rich splicing factor 9-like	2078	2.90E-105
XM_020085651.1	serine/arginine-rich splicing factor 9-like	1643	5.08E-107
XM_020085652.1	dynein light chain 2, cytoplasmic-like	1084	1.27E-59
XM_020085653.1	dynein light chain 2, cytoplasmic-like	1056	9.56E-60
XM_020085654.1	clathrin heavy chain 1 isoform X1	6475	0
XM_020085655.1	clathrin heavy chain 1 isoform X2	6472	0

XM_020085656.1	peptidyl-tRNA hydrolase 2, mitochondrial isoform X1	3120	1.79E-94
XM_020085657.1	peptidyl-tRNA hydrolase 2, mitochondrial isoform X2	3161	3.39E-93
XM_020085658.1	peptidyl-tRNA hydrolase 2, mitochondrial isoform X1	3249	4.56E-92
XM_020085659.1	U4/U6 small nuclear ribonucleoprotein Prp4 isoform X2	2512	0
XM_020085660.1	zinc finger ZZ-type and EF-hand domain-containing protein 1 isoform X1	11525	0
XM_020085661.1	zinc finger ZZ-type and EF-hand domain-containing protein 1 isoform X2	11522	0
XM_020085662.1	zinc finger ZZ-type and EF-hand domain-containing protein 1 isoform X3	11371	0
XM_020085663.1	rabankyrin-5 isoform X1	4171	0
XM_020085664.1	protein atonal homolog 8 isoform X2	2901	0
XM_020085665.1	probable palmitoyltransferase ZDHHC20	2462	4.40E-165
XM_020085666.1	rabankyrin-5 isoform X1	4361	0
XM_020085667.1	membrane-associated progesterone receptor component 1	969	1.51E-173
XM_020085668.1	nuclear receptor corepressor 1 isoform X1	9657	0
XM_020085669.1	nuclear receptor corepressor 1 isoform X1	9594	0
XM_020085670.1	nuclear receptor corepressor 1 isoform X1	9651	0
XM_020085671.1	nuclear receptor corepressor 1 isoform X1	9631	0
XM_020085672.1	nuclear receptor corepressor 1 isoform X1	9618	0
XM_020085673.1	nuclear receptor corepressor 1 isoform X1	9508	0
XM_020085674.1	nuclear receptor corepressor 1 isoform X1	7086	0
XM_020085675.1	PREDICTED: tetraspanin-6	1708	2.78E-158
XM_020085676.1	nuclear receptor corepressor 1 isoform X1	8214	0
XM_020085677.1	N-acetylglucosaminyl-phosphatidylinositol de-N-acetylase	1303	7.79E-180
XM_020085678.1	centromere protein V	777	4.33E-108
XM_020085679.1	centromere protein V	743	1.09E-108
XM_020085680.1	tripartite motif-containing protein 16-like	3022	0
XM_020085681.1	tripartite motif-containing protein 16-like	3048	0
XM_020085682.1	tripartite motif-containing protein 16-like	2971	0
XM_020085683.1	tripartite motif-containing protein 16-like	2070	0
XM_020085684.1	PREDICTED: tetraspanin-6	1717	2.98E-158
XM_020085685.1	ruvB-like 2 isoform X1	1817	0
XM_020085686.1	ruvB-like 2 isoform X2	1814	0
XM_020085687.1	E3 ubiquitin-protein ligase listerin isoform X1	6067	0
XM_020085688.1	E3 ubiquitin-protein ligase listerin isoform X2	6064	0
XM_020085689.1	hephaestin-like protein 1 isoform X1	4545	0
XM_020085690.1	hephaestin-like protein 1 isoform X2	4530	0
XM_020085691.1	hephaestin-like protein 1 isoform X1	3389	0
XM_020085692.1	transmembrane protein 135	3385	0
XM_020085693.1	la-related protein 1 isoform X1	6716	0
XM_020085694.1	la-related protein 1 isoform X2	6701	0
XM_020085695.1	fatty acid hydroxylase domain-containing protein 2	5123	0
XM_020085696.1	ribosomal protein S6 kinase alpha-6 isoform X1	3735	0
XM_020085697.1	spectrin beta chain, non-erythrocytic 1-like isoform X1	11027	0
XM_020085698.1	spectrin beta chain, non-erythrocytic 1-like isoform X3	10889	0
XM_020085699.1	spectrin beta chain, non-erythrocytic 1-like isoform X3	11148	0
XM_020085700.1	MAP/microtubule affinity-regulating kinase 4-like	2251	0
XM_020085701.1	creatine kinase M-type	1689	0
XM_020085702.1	MAP/microtubule affinity-regulating kinase 4-like isoform X1	1285	7.99E-90
XM_020085703.1	MAP/microtubule affinity-regulating kinase 4-like isoform X2	568	2.27E-81
XM_020085704.1	sarcoplasmic/endoplasmic reticulum calcium ATPase 3	6767	0
XM_020085705.1	ribosomal protein S6 kinase alpha-6 isoform X1	3714	0
XM_020085706.1	gamma-aminobutyric acid receptor subunit beta-4-like isoform X1	2235	0
XM_020085707.1	gamma-aminobutyric acid receptor subunit beta-4-like isoform X2	2121	0
XM_020085708.1	transmembrane protein 164 isoform X1	2717	0
XM_020085709.1	transmembrane protein 164 isoform X1	2525	0
XM_020085710.1	transmembrane protein 164 isoform X2	2140	6.01E-114
XM_020085711.1	gamma-aminobutyric acid receptor subunit alpha-5	2827	0
XM_020085712.1	52 kDa repressor of the inhibitor of the protein kinase-like	8345	0
XM_020085713.1	ribosomal protein S6 kinase alpha-6 isoform X1	3614	0
XM_020085714.1	rho GTPase-activating protein 5	5618	0
XM_020085715.1	unconventional myosin-Ic-like	3782	0
XM_020085716.1	unconventional myosin-Ic-like	3744	0
XM_020085717.1	flotillin-2 isoform X1	4677	0
XM_020085718.1	flotillin-2 isoform X2	4679	0
XM_020085719.1	AMME syndrome candidate gene 1 protein	4494	2.23E-171
XM_020085720.1	THO complex subunit 2	5704	0
XM_020085721.1	THO complex subunit 2	5701	0
XM_020085722.1	ribosomal protein S6 kinase alpha-6 isoform X1	3384	0
XM_020085723.1	acetylcholine receptor subunit gamma-like	2444	0
XM_020085724.1	ceramide-1-phosphate transfer protein-like isoform X1	4253	5.03E-174
XM_020085725.1	ceramide-1-phosphate transfer protein-like isoform X2	4245	1.88E-171
XM_020085726.1	sodium/potassium-transporting ATPase subunit beta-2	4642	0
XM_020085727.1	TBC1 domain family member 8B isoform X1	6728	0
XM_020085728.1	TBC1 domain family member 8B isoform X2	6634	0
XM_020085729.1	gap junction alpha-3 protein-like	1905	0
XM_020085730.1	dolichyl-diphosphooligosaccharide--protein glycosyltransferase subunit STT3A	2447	0
XM_020085731.1	dolichyl-diphosphooligosaccharide--protein glycosyltransferase subunit STT3A	2256	0
XM_020085732.1	dolichyl-diphosphooligosaccharide--protein glycosyltransferase subunit STT3A	2313	0
XM_020085733.1	cytoskeleton-associated protein 2-like	2117	0
XM_020085734.1	protocadherin-18-like isoform X1	4613	0

XM_020085735.1	acetyl-CoA carboxylase 1 isoform X1	9127	0
XM_020085736.1	acetyl-CoA carboxylase 1 isoform X2	9109	0
XM_020085737.1	acetyl-CoA carboxylase 1 isoform X3	8807	0
XM_020085738.1	acetyl-CoA carboxylase 1 isoform X4	8702	0
XM_020085739.1	acetyl-CoA carboxylase 1 isoform X5	8684	0
XM_020085740.1	acetyl-CoA carboxylase 1 isoform X6	8953	0
XM_020085741.1	acetyl-CoA carboxylase 1 isoform X7	8660	0
XM_020085742.1	acetyl-CoA carboxylase 1 isoform X8	8657	0
XM_020085743.1	acetyl-CoA carboxylase 1 isoform X9	8639	0
XM_020085744.1	acetyl-CoA carboxylase 1 isoform X10	8908	0
XM_020085745.1	protocadherin-18-like isoform X2	4580	0
XM_020085746.1	ribosomal protein S6 kinase beta-1 isoform X1	4771	0
XM_020085747.1	ribosomal protein S6 kinase beta-1 isoform X1	4520	0
XM_020085748.1	E3 ubiquitin-protein ligase NRDP1	2314	0
XM_020085749.1	gamma-aminobutyric acid receptor subunit rho-3	1688	0
XM_020085750.1	spermine synthase	2375	0
XM_020085751.1	PREDICTED: uncharacterized protein LOC109628586	2138	0
XM_020085752.1	folliculin-interacting protein 1	6626	0
XM_020085753.1	protocadherin-18-like isoform X3	4561	0
XM_020085754.1	mediator of RNA polymerase II transcription subunit 13-like	8072	0
XM_020085755.1	rho GTPase-activating protein 32 isoform X1	8500	0
XM_020085756.1	rho GTPase-activating protein 32 isoform X1	8496	0
XM_020085757.1	rho GTPase-activating protein 32 isoform X1	8310	0
XM_020085758.1	rho GTPase-activating protein 32 isoform X1	8145	0
XM_020085759.1	rho GTPase-activating protein 32 isoform X1	8535	0
XM_020085760.1	rho GTPase-activating protein 32 isoform X1	6670	0
XM_020085761.1	nuclear apoptosis-inducing factor 1-like	2425	0
XM_020085762.1	nuclear apoptosis-inducing factor 1-like	2421	0
XM_020085763.1	nuclear apoptosis-inducing factor 1-like	2328	0
XM_020085764.1	calpain small subunit 1-like	2471	7.34E-147
XM_020085765.1	centrosomal protein of 128 kDa-like	1853	0
XM_020085766.1	centromere protein I isoform X1	3636	0
XM_020085767.1	pyruvate dehydrogenase E1 component subunit alpha, mitochondrial-like	1998	0
XM_020085768.1	natural killer enhancing factor	978	0
XM_020085769.1	calpain-5 isoform X1	2681	0
XM_020085770.1	calpain-5-like isoform X2	2738	0
XM_020085771.1	serine/threonine-protein kinase PAK 3 isoform X1	5987	0
XM_020085772.1	serine/threonine-protein kinase PAK 3 isoform X2	5960	0
XM_020085773.1	serine/threonine-protein kinase PAK 3 isoform X3	5897	0
XM_020085774.1	zinc finger protein 711	4832	0
XM_020085775.1	centromere protein I isoform X2	3635	0
XM_020085776.1	zinc finger protein 711	4465	0
XM_020085777.1	diamine acetyltransferase 1-like	1911	7.61E-116
XM_020085778.1	apoptosis-inducing factor 1, mitochondrial isoform X1	2881	0
XM_020085779.1	apoptosis-inducing factor 1, mitochondrial isoform X1	2880	0
XM_020085780.1	apoptosis-inducing factor 1, mitochondrial isoform X3	2514	0
XM_020085781.1	nuclear fragile X mental retardation-interacting protein 2	3025	0
XM_020085782.1	misshapen-like kinase 1 isoform X1	5402	0
XM_020085783.1	misshapen-like kinase 1 isoform X2	5396	0
XM_020085784.1	misshapen-like kinase 1 isoform X3	5378	0
XM_020085785.1	misshapen-like kinase 1 isoform X4	5315	0
XM_020085786.1	misshapen-like kinase 1 isoform X5	5294	0
XM_020085787.1	misshapen-like kinase 1 isoform X6	5201	0
XM_020085788.1	misshapen-like kinase 1 isoform X7	5135	0
XM_020085789.1	ubiquitin-like protein 3	2155	1.85E-77
XM_020085790.1	misshapen-like kinase 1 isoform X8	5102	0
XM_020085791.1	misshapen-like kinase 1 isoform X9	4901	0
XM_020085792.1	misshapen-like kinase 1 isoform X10	5291	0
XM_020085793.1	myelin protein zero-like protein 2	1943	4.50E-133
XM_020085794.1	platelet-activating factor acetylhydrolase IB subunit gamma	3431	2.44E-159
XM_020085795.1	platelet-activating factor acetylhydrolase IB subunit gamma	3040	1.01E-160
XM_020085796.1	interferon regulatory factor 2-binding protein 1-like	3202	0
XM_020085797.1	calcium uptake protein 2, mitochondrial	1794	0
XM_020085798.1	protocadherin-10-like isoform X3	1850	1.08E-65
XM_020085799.1	pecanex-like protein 3 isoform X1	8885	0
XM_020085800.1	pecanex-like protein 3 isoform X1	7584	0
XM_020085801.1	pecanex-like protein 3 isoform X1	8882	0
XM_020085802.1	pecanex-like protein 3 isoform X1	8855	0
XM_020085803.1	syntaxin-3-like isoform X1	2949	0
XM_020085804.1	C2 domain-containing protein 2-like	3924	0
XM_020085805.1	C2 domain-containing protein 2-like	3936	0
XM_020085806.1	UDP-N-acetylglucosamine--dolichyl-phosphate N-acetylglucosaminephosphotransferase	2664	0
XM_020085807.1	histone H2AX	651	3.37E-66
XM_020085808.1	ras-related protein Rab-5A-like	2858	4.44E-145
XM_020085809.1	ras-related protein Rab-5A-like	2850	2.78E-144
XM_020085810.1	membrane-associated progesterone receptor component 1	1818	3.80E-105
XM_020085811.1	syntaxin-3-like isoform X1	1362	0
XM_020085812.1	ephrin type-B receptor 4	6152	0
XM_020085813.1	junctional adhesion molecule C-like	2236	0

XM_020085814.1	C2 domain-containing protein 2-like	4358	0
XM_020085815.1	autophagy-related protein 101	1288	1.97E-161
XM_020085816.1	autophagy-related protein 101	1211	1.17E-161
XM_020085817.1	histone-lysine N-methyltransferase, H3 lysine-36 and H4 lysine-20 specific-like	9650	0
XM_020085818.1	histone-lysine N-methyltransferase, H3 lysine-36 and H4 lysine-20 specific-like	9342	0
XM_020085819.1	histone-lysine N-methyltransferase, H3 lysine-36 and H4 lysine-20 specific-like	9618	0
XM_020085820.1	syntaxin-3-like isoform X1	1271	0
XM_020085821.1	histone-lysine N-methyltransferase, H3 lysine-36 and H4 lysine-20 specific-like	9418	0
XM_020085822.1	histone-lysine N-methyltransferase, H3 lysine-36 and H4 lysine-20 specific-like	9520	0
XM_020085823.1	histone-lysine N-methyltransferase, H3 lysine-36 and H4 lysine-20 specific-like	9682	0
XM_020085824.1	kinesin-like protein KIF20A isoform X1	3653	0
XM_020085825.1	kinesin-like protein KIF20A isoform X1	2710	0
XM_020085826.1	cationic amino acid transporter 2-like	1836	0
XM_020085827.1	cell division cycle protein 23 homolog	2289	0
XM_020085828.1	stress-induced-phosphoprotein 1	2016	0
XM_020085829.1	spermatogenesis-associated protein 22 isoform X1	1488	0
XM_020085830.1	spermatogenesis-associated protein 22 isoform X1	1439	0
XM_020085831.1	spermatogenesis-associated protein 22 isoform X1	1485	0
XM_020085832.1	transmembrane protein 211-like	3156	4.43E-139
XM_020085833.1	kelch-like protein 3	3405	0
XM_020085834.1	vitamin K epoxide reductase complex subunit 1-like protein 1	5300	2.39E-103
XM_020085835.1	histone H3.3	2032	8.87E-89
XM_020085836.1	fibroblast growth factor receptor 4	6367	0
XM_020085837.1	myotubularin-related protein 4 isoform X1	5332	0
XM_020085838.1	myotubularin-related protein 4 isoform X3	5304	0
XM_020085839.1	myotubularin-related protein 4 isoform X3	5382	0
XM_020085840.1	protein transport protein Sec24A-like	5235	0
XM_020085841.1	nuclear factor related to kappa-B-binding protein	4431	0
XM_020085842.1	transmembrane protein 45A	1155	0
XM_020085843.1	transmembrane protein 45A	1156	0
XM_020085844.1	RING finger protein 214	5518	0
XM_020085845.1	TSC22 domain family protein 3-like isoform X3	2656	1.41E-148
XM_020085846.1	Down syndrome critical region protein 3	1046	0
XM_020085847.1	Down syndrome critical region protein 3	1226	0
XM_020085848.1	Down syndrome critical region protein 3	1053	0
XM_020085849.1	probable ATP-dependent RNA helicase DDX46	3969	0
XM_020085850.1	UPF0461 protein C5orf24 homolog	3879	4.16E-121
XM_020085851.1	UPF0461 protein C5orf24 homolog	3882	4.22E-121
XM_020085852.1	protocadherin alpha-C2-like isoform X1	7001	0
XM_020085853.1	protocadherin alpha-C2-like isoform X2	3530	0
XM_020085854.1	TSC22 domain family protein 1-like isoform X2	2067	2.70E-114
XM_020085855.1	protocadherin alpha-C2-like isoform X1	5560	0
XM_020085856.1	protocadherin alpha-C2-like isoform X2	5557	0
XM_020085857.1	protocadherin alpha-C2-like isoform X3	5597	0
XM_020085858.1	bromodomain and WD repeat-containing protein 3-like	7562	0
XM_020085859.1	transcriptional regulator ATRX-like isoform X1	6284	0
XM_020085860.1	transcriptional regulator ATRX-like isoform X2	6156	0

XM_020085861.1	TSC22 domain family protein 3-like isoform X3	2154	9.35E-86
XM_020085862.1	estrogen-related receptor gamma-like isoform X1	4512	0
XM_020085863.1	estrogen-related receptor gamma-like isoform X1	4489	0
XM_020085864.1	estrogen-related receptor gamma-like isoform X1	4300	0
XM_020085865.1	estrogen-related receptor gamma-like isoform X1	4479	0
XM_020085866.1	heat shock 70 kDa protein 4L	4693	0
XM_020085867.1	zona pellucida-like domain-containing protein 1	3149	0
XM_020085868.1	rap guanine nucleotide exchange factor 6 isoform X1	9343	0
XM_020085869.1	rap guanine nucleotide exchange factor 6 isoform X2	9325	0
XM_020085870.1	rap guanine nucleotide exchange factor 6 isoform X3	9319	0
XM_020085871.1	rap guanine nucleotide exchange factor 6 isoform X4	9301	0
XM_020085872.1	ADP/ATP translocase 2	1799	0
XM_020085873.1	phosphatidylinositol-binding clathrin assembly protein-like isoform X1	3826	0
XM_020085874.1	phosphatidylinositol-binding clathrin assembly protein-like isoform X2	3823	0
XM_020085875.1	leucine-rich repeat transmembrane protein FLRT1-like	5615	0
XM_020085876.1	phosphatidylinositol-binding clathrin assembly protein-like isoform X3	3820	0
XM_020085877.1	phosphatidylinositol-binding clathrin assembly protein-like isoform X4	3817	0
XM_020085878.1	phosphatidylinositol-binding clathrin assembly protein-like isoform X5	3796	0
XM_020085879.1	phosphatidylinositol-binding clathrin assembly protein-like isoform X6	3784	0
XM_020085880.1	phosphatidylinositol-binding clathrin assembly protein-like isoform X7	3742	0
XM_020085881.1	phosphatidylinositol-binding clathrin assembly protein-like isoform X8	3712	0
XM_020085882.1	phosphatidylinositol-binding clathrin assembly protein-like isoform X9	3589	0
XM_020085883.1	phosphatidylinositol-binding clathrin assembly protein-like isoform X10	3559	0
XM_020085884.1	phosphatidylinositol-binding clathrin assembly protein-like isoform X11	3514	0
XM_020085885.1	phosphatidylinositol-binding clathrin assembly protein-like isoform X12	3511	0
XM_020085886.1	phosphatidylinositol-binding clathrin assembly protein-like isoform X13	3508	0
XM_020085887.1	phosphatidylinositol-binding clathrin assembly protein-like isoform X14	3484	0
XM_020085888.1	phosphatidylinositol-binding clathrin assembly protein-like isoform X15	3472	0
XM_020085889.1	phosphatidylinositol-binding clathrin assembly protein-like isoform X16	3442	0
XM_020085890.1	phosphatidylinositol-binding clathrin assembly protein-like isoform X17	3700	0
XM_020085891.1	O-acetyl-ADP-ribose deacetylase MACROD1 isoform X1	1658	0
XM_020085892.1	moesin isoform X1	4449	0

XM_020085893.1	moesin isoform X2	3579	0
XM_020085894.1	serine/arginine repetitive matrix protein 1-like	2562	0
XM_020085895.1	proline and serine-rich protein 1 isoform X1	3977	0
XM_020085896.1	proline and serine-rich protein 1 isoform X2	3884	0
XM_020085897.1	vacuolar protein-sorting-associated protein 36	1566	0
XM_020085898.1	roundabout homolog 3 isoform X1	5603	0
XM_020085899.1	O-acetyl-ADP-ribose deacetylase MACROD1 isoform X1	1688	0
XM_020085900.1	roundabout homolog 3 isoform X2	5600	0
XM_020085901.1	roundabout homolog 3 isoform X3	5601	0
XM_020085902.1	roundabout homolog 3 isoform X4	5591	0
XM_020085903.1	roundabout homolog 3 isoform X5	5588	0
XM_020085904.1	barrier-to-autointegration factor	1084	7.90E-58
XM_020085905.1	guanine nucleotide-binding protein G(I)/G(S)/G(O) subunit gamma-4	499	1.66E-50
XM_020085906.1	nuclear receptor-interacting protein 1	6292	0
XM_020085907.1	nuclear receptor-interacting protein 1	6374	0
XM_020085908.1	AP-1 complex subunit beta-1	4955	0
XM_020085909.1	AP-1 complex subunit beta-1	4950	0
XM_020085910.1	AP-1 complex subunit beta-1	4849	0
XM_020085911.1	ubiquitin carboxyl-terminal hydrolase 25 isoform X1	4408	0
XM_020085912.1	ubiquitin carboxyl-terminal hydrolase 25 isoform X2	4138	0
XM_020085913.1	dedicator of cytokinesis protein 1	5809	0
XM_020085914.1	solute carrier family 12 member 9-like	3564	0
XM_020085915.1	guanine nucleotide-binding protein G(I)/G(S)/G(T) subunit beta-1-like	2249	0
XM_020085916.1	high affinity cationic amino acid transporter 1-like isoform X1	5294	0
XM_020085917.1	high affinity cationic amino acid transporter 1-like isoform X2	2387	0
XM_020085918.1	coxsackievirus and adenovirus receptor homolog	1564	0
XM_020085919.1	unconventional myosin-XIX	4411	0
XM_020085920.1	phosphatidylinositol-glycan biosynthesis class W protein	2627	0
XM_020085921.1	phosphatidylinositol-glycan biosynthesis class W protein	2627	0
XM_020085922.1	zinc finger HIT domain-containing protein 3 isoform X1	695	1.85E-100
XM_020085923.1	zinc finger HIT domain-containing protein 3 isoform X2	674	9.87E-95
XM_020085924.1	7SK snRNA methylphosphate capping enzyme-like isoform X1	6798	0
XM_020085925.1	protein FAM196B-like	2545	0
XM_020085926.1	7SK snRNA methylphosphate capping enzyme-like isoform X1	6652	0
XM_020085927.1	7SK snRNA methylphosphate capping enzyme-like isoform X1	6713	0
XM_020085928.1	7SK snRNA methylphosphate capping enzyme-like isoform X1	6717	0
XM_020085929.1	calcium/calmodulin-dependent protein kinase kinase 1 isoform X1	4718	0
XM_020085930.1	SUMO-interacting motif-containing protein 1 isoform X1	2790	0
XM_020085931.1	calcium/calmodulin-dependent protein kinase kinase 1 isoform X2	5466	0
XM_020085932.1	P2X purinoceptor 1 isoform X1	2481	0
XM_020085933.1	P2X purinoceptor 1 isoform X2	2469	0
XM_020085934.1	P2X purinoceptor 1 isoform X3	2436	0
XM_020085935.1	pro-neuregulin-2, membrane-bound isoform-like isoform X1	2257	0
XM_020085936.1	pro-neuregulin-2, membrane-bound isoform-like isoform X2	2091	0
XM_020085937.1	pro-neuregulin-2, membrane-bound isoform-like isoform X2	2085	0
XM_020085938.1	pro-neuregulin-2, membrane-bound isoform-like isoform X4	2284	0
XM_020085939.1	transcriptional activator protein Pur-alpha-like	4354	0
XM_020085941.1	transcriptional activator protein Pur-alpha-like	4405	0
XM_020085942.1	transcriptional activator protein Pur-alpha-like	4271	0
XM_020085943.1	transient receptor potential cation channel subfamily V member 2	2962	0
XM_020085944.1	protein FAM222B	3979	0
XM_020085945.1	baculoviral IAP repeat-containing protein 2-like isoform X1	2949	0
XM_020085946.1	baculoviral IAP repeat-containing protein 2-like isoform X1	2927	0
XM_020085947.1	baculoviral IAP repeat-containing protein 2-like isoform X1	2955	0
XM_020085948.1	60S ribosomal protein L23a	577	9.71E-97
XM_020085949.1	autism susceptibility gene 2 protein isoform X1	5848	0
XM_020085950.1	autism susceptibility gene 2 protein isoform X2	5814	0
XM_020085951.1	autism susceptibility gene 2 protein isoform X3	4774	0
XM_020085952.1	glypican-6-like isoform X1	5204	0
XM_020085953.1	serine/threonine-protein phosphatase 2B catalytic subunit alpha isoform-like isoform X1	4471	0
XM_020085954.1	serine/threonine-protein phosphatase 2B catalytic subunit alpha isoform-like isoform X2	4420	0
XM_020085955.1	dnaJ homolog subfamily C member 18	2444	0
XM_020085956.1	protein CDKN2AIP homolog A-like	613	4.86E-76
XM_020085957.1	ubiquitin-conjugating enzyme E2 B	2212	3.47E-104
XM_020085958.1	TAF6-like RNA polymerase II p300/CBP-associated factor-associated factor 65 kDa subunit 6L	2435	0
XM_020085959.1	glypican-6-like isoform X2	4455	0
XM_020085960.1	28S ribosomal protein S31, mitochondrial	1814	0
XM_020085961.1	disks large homolog 4 isoform X1	5668	0
XM_020085962.1	disks large homolog 4 isoform X2	5745	0
XM_020085963.1	disks large homolog 4 isoform X3	5628	0
XM_020085964.1	disks large homolog 4 isoform X4	5742	0
XM_020085965.1	disks large homolog 4 isoform X5	5685	0
XM_020085966.1	disks large homolog 4 isoform X6	5429	0
XM_020085967.1	zinc finger and BTB domain-containing protein 20	2833	0
XM_020085968.1	zinc finger and BTB domain-containing protein 20	2998	0
XM_020085969.1	protein SMG8	3409	0
XM_020085970.1	ATP-sensitive inward rectifier potassium channel 10 isoform X1	1353	0
XM_020085971.1	fibrous sheath CABYR-binding protein-like isoform X1	1870	0
XM_020085972.1	fibrous sheath CABYR-binding protein-like isoform X1	1857	0

XM_020085973.1	sorting nexin-25	4631	0
XM_020085974.1	fibrous sheath CABYR-binding protein-like isoform X1	1861	0
XM_020085975.1	fibrous sheath CABYR-binding protein-like isoform X1	1813	0
XM_020085976.1	tigger transposable element-derived protein 6	1837	0
XM_020085977.1	tigger transposable element-derived protein 6	1835	0
XM_020085978.1	tigger transposable element-derived protein 6	1621	0
XM_020085979.1	myelin proteolipid protein-like isoform X1	1756	0
XM_020085980.1	myelin proteolipid protein-like isoform X2	1665	3.98E-178
XM_020085981.1	myelin proteolipid protein-like isoform X2	1652	1.20E-177
XM_020085982.1	sorting nexin-25	4554	0
XM_020085983.1	myb-binding protein 1A	4172	0
XM_020085984.1	retinoblastoma-associated protein isoform X1	4052	0
XM_020085985.1	retinoblastoma-associated protein isoform X1	3857	0
XM_020085986.1	retinoblastoma-associated protein isoform X1	3723	0
XM_020085987.1	lysophosphatidic acid receptor 6-like	1821	0
XM_020085988.1	lysophosphatidic acid receptor 6-like	1443	0
XM_020085989.1	ras-related protein Rab-6A isoform X1	2388	5.54E-147
XM_020085990.1	ras-related protein Rab-6A isoform X2	2394	4.15E-147
XM_020085991.1	sorting nexin-25	4533	0
XM_020085992.1	HMG box transcription factor BBX isoform X1	8603	0
XM_020085993.1	HMG box transcription factor BBX isoform X1	7924	0
XM_020085994.1	HMG box transcription factor BBX isoform X1	8054	0
XM_020085995.1	HMG box transcription factor BBX isoform X1	8602	0
XM_020085996.1	HMG box transcription factor BBX isoform X1	3994	0
XM_020085997.1	HMG box transcription factor BBX isoform X1	8521	0
XM_020085998.1	transcription initiation factor TFIID subunit 1-like isoform X1	6598	0
XM_020085999.1	transcription initiation factor TFIID subunit 1-like isoform X2	6592	0
XM_020086000.1	transcription initiation factor TFIID subunit 1-like isoform X3	6561	0
XM_020086001.1	sorting nexin-25	4456	0
XM_020086002.1	transcription initiation factor TFIID subunit 1-like isoform X4	6556	0
XM_020086003.1	transcription initiation factor TFIID subunit 1-like isoform X5	6453	0
XM_020086004.1	transcription initiation factor TFIID subunit 1-like isoform X6	6447	0
XM_020086005.1	cohesin subunit SA-2-like isoform X1	6116	0
XM_020086006.1	cohesin subunit SA-2-like isoform X1	6028	0
XM_020086007.1	cohesin subunit SA-2-like isoform X1	6113	0
XM_020086008.1	cohesin subunit SA-2-like isoform X1	6110	0
XM_020086009.1	cohesin subunit SA-2-like isoform X4	5808	0
XM_020086010.1	peripheral-type benzodiazepine receptor-associated protein 1	10174	0
XM_020086011.1	ribosomal protein S6 kinase alpha-4-like	5622	0
XM_020086012.1	adenylosuccinate synthetase isozyme 2-like	2388	0
XM_020086013.1	vacuolar protein sorting-associated protein 26B-like	3843	0
XM_020086014.1	glutathione reductase, mitochondrial isoform X1	2394	0
XM_020086015.1	vacuolar protein sorting-associated protein 26B-like	2823	0
XM_020086016.1	phospholipase D2-like	5324	0
XM_020086017.1	APC membrane recruitment protein 1-like	6517	0
XM_020086018.1	neuronal migration protein doublecortin	3889	0
XM_020086019.1	SUMO-interacting motif-containing protein 1 isoform X2	2347	0
XM_020086020.1	serine/threonine-protein phosphatase 2A catalytic subunit alpha isoform	1722	0
XM_020086021.1	rho GTPase-activating protein 42-like	4891	0
XM_020086022.1	GTP-binding protein SAR1b	2428	2.58E-139
XM_020086023.1	glutathione reductase, mitochondrial isoform X2	2345	0
XM_020086024.1	serine/threonine-protein kinase 36 isoform X1	4193	0
XM_020086025.1	serine/threonine-protein kinase 36 isoform X1	4200	0
XM_020086026.1	serine/threonine-protein kinase 36 isoform X1	4064	0
XM_020086027.1	serine/threonine-protein kinase 36 isoform X1	4166	0
XM_020086028.1	A disintegrin and metalloproteinase with thrombospondin motifs 15-like	4835	0
XM_020086029.1	ryanodine receptor 1-like isoform X1	15606	0
XM_020086030.1	ryanodine receptor 1-like isoform X2	15624	0
XM_020086031.1	cell adhesion molecule 2-like isoform X1	7216	0
XM_020086032.1	cell adhesion molecule 2-like isoform X2	7189	0
XM_020086033.1	cell adhesion molecule 2-like isoform X3	7102	0
XM_020086034.1	androgen receptor-like isoform X1	5785	0
XM_020086035.1	androgen receptor-like isoform X2	3413	0
XM_020086036.1	tyrosine-protein kinase BTK	2198	0
XM_020086037.1	55 kDa erythrocyte membrane protein	2345	0
XM_020086038.1	protein phosphatase 1D isoform X1	4104	0
XM_020086039.1	protein phosphatase 1D isoform X2	4003	0
XM_020086040.1	14-3-3 protein beta/alpha-1	2563	4.44E-177
XM_020086041.1	E3 ubiquitin-protein ligase TRIM37 isoform X1	5291	0
XM_020086042.1	E3 ubiquitin-protein ligase TRIM37 isoform X2	5288	0
XM_020086043.1	E3 ubiquitin-protein ligase TRIM37 isoform X3	5282	0
XM_020086044.1	E3 ubiquitin-protein ligase TRIM37 isoform X4	5248	0
XM_020086045.1	E3 ubiquitin-protein ligase TRIM37 isoform X5	4093	0
XM_020086046.1	E3 ubiquitin-protein ligase TRIM37 isoform X6	3359	0
XM_020086047.1	diacylglycerol O-acyltransferase 2	2861	0
XM_020086048.1	nectin-3-like protein isoform X1	5561	0
XM_020086049.1	nectin-3-like protein isoform X1	3443	0
XM_020086050.1	mediator of RNA polymerase II transcription subunit 7	1800	5.57E-161
XM_020086051.1	E3 ubiquitin-protein ligase RNF43 isoform X1	5287	0

XM_020086052.1	E3 ubiquitin-protein ligase RNF43 isoform X2	5266	0
XM_020086053.1	4-hydroxyphenylpyruvate dioxygenase	3329	0
XM_020086054.1	PREDICTED: uncharacterized protein LOC109628735	1906	1.41E-146
XM_020086055.1	FERM domain-containing protein 8	4006	0
XM_020086056.1	FERM domain-containing protein 8	4001	0
XM_020086057.1	integrator complex subunit 2	5191	0
XM_020086058.1	cytospin-B isoform X1	4542	0
XM_020086059.1	cytospin-B isoform X1	4644	0
XM_020086060.1	cytospin-B isoform X1	4642	0
XM_020086061.1	cytospin-B isoform X1	4552	0
XM_020086062.1	RING finger protein 44 isoform X1	4057	0
XM_020086063.1	vesicle-associated membrane protein 2	2773	2.06E-42
XM_020086064.1	kinase suppressor of Ras 1 isoform X1	5021	0
XM_020086065.1	kinase suppressor of Ras 1 isoform X2	4980	0
XM_020086066.1	PAX3- and PAX7-binding protein 1	3408	0
XM_020086067.1	chromosome-associated kinesin KIF4A	4663	0
XM_020086068.1	E3 ubiquitin-protein ligase CBL-B	7178	0
XM_020086069.1	bromodomain-containing protein 8-like isoform X1	5635	0
XM_020086070.1	bromodomain-containing protein 8-like isoform X2	5614	0
XM_020086071.1	RING finger protein 44 isoform X1	3741	0
XM_020086072.1	bromodomain-containing protein 8-like isoform X3	5590	0
XM_020086073.1	bromodomain-containing protein 8-like isoform X4	5642	0
XM_020086074.1	guanine nucleotide-binding protein G(I)/G(S)/G(O) subunit gamma-4	485	2.03E-45
XM_020086075.1	phosphoglycerate kinase 1	2147	0
XM_020086076.1	X-ray radiation resistance-associated protein 1	3250	0
XM_020086077.1	PREDICTED: sialidase-3	2899	0
XM_020086078.1	unconventional myosin-XVIIIa-like isoform X1	11217	0
XM_020086079.1	RING finger protein 44 isoform X1	4009	0
XM_020086080.1	unconventional myosin-XVIIIa-like isoform X1	11161	0
XM_020086081.1	unconventional myosin-XVIIIa-like isoform X3	11172	0
XM_020086082.1	unconventional myosin-XVIIIa-like isoform X4	11145	0
XM_020086083.1	unconventional myosin-XVIIIa-like isoform X5	10965	0
XM_020086084.1	unconventional myosin-XVIIIa-like isoform X1	11150	0
XM_020086085.1	unconventional myosin-XVIIIa-like isoform X7	9810	0
XM_020086086.1	unconventional myosin-XVIIIa-like isoform X8	9765	0
XM_020086087.1	unconventional myosin-XVIIIa-like isoform X9	10920	0
XM_020086088.1	transcriptional coactivator YAP1-like isoform X1	4384	0
XM_020086089.1	transcriptional coactivator YAP1-like isoform X2	1367	0
XM_020086090.1	angiopoietin-related protein 5-like	1356	0
XM_020086091.1	UV radiation resistance-associated gene protein	4056	0
XM_020086092.1	vacuolar protein sorting-associated protein 11 homolog	3002	0
XM_020086093.1	T-complex protein 1 subunit theta isoform X1	2127	0
XM_020086094.1	T-complex protein 1 subunit theta isoform X1	2141	0
XM_020086095.1	T-complex protein 1 subunit theta isoform X1	2123	0
XM_020086096.1	T-complex protein 1 subunit theta isoform X1	1993	0
XM_020086097.1	nucleophosmin-like isoform X1	2066	4.28E-173
XM_020086098.1	nucleophosmin-like isoform X2	2068	2.16E-172
XM_020086099.1	lysine-specific demethylase 6B-like	6447	0
XM_020086100.1	ubiquitin-conjugating enzyme E2 G1-like	1985	7.01E-120
XM_020086101.1	vascular endothelial zinc finger 1-like	3231	0
XM_020086102.1	opioid-binding protein/cell adhesion molecule-like isoform X1	7687	0
XM_020086103.1	opioid-binding protein/cell adhesion molecule homolog isoform X2	7160	0
XM_020086104.1	opioid-binding protein/cell adhesion molecule-like isoform X3	7651	0
XM_020086105.1	opioid-binding protein/cell adhesion molecule homolog isoform X2	7351	0
XM_020086106.1	protein FAM76B isoform X1	2535	0
XM_020086107.1	protein FAM76B isoform X2	2533	0
XM_020086108.1	PREDICTED: apelin	4666	3.19E-34
XM_020086109.1	core histone macro-H2A.2	1796	0
XM_020086110.1	core histone macro-H2A.2	1787	0
XM_020086111.1	core histone macro-H2A.1 isoform X3	1238	4.94E-133
XM_020086112.1	BUD13 homolog isoform X1	1777	0
XM_020086113.1	BUD13 homolog isoform X2	1739	0
XM_020086114.1	U11/U12 small nuclear ribonucleoprotein 35 kDa protein	2044	1.01E-113
XM_020086115.1	junctional adhesion molecule C-like	3863	0
XM_020086116.1	PREDICTED: beta-glucuronidase	2791	0
XM_020086117.1	myotubularin-related protein 2 isoform X1	3274	0
XM_020086118.1	myotubularin-related protein 2 isoform X2	3271	0
XM_020086119.1	rho GTPase-activating protein 42-like	4054	0
XM_020086120.1	wolframin-like isoform X1	3940	0
XM_020086121.1	V-type proton ATPase catalytic subunit A	2127	0
XM_020086122.1	protein spinster homolog 2 isoform X1	5004	0
XM_020086123.1	protein spinster homolog 2 isoform X1	5014	0
XM_020086124.1	calcium signal-modulating cyclophilin ligand isoform X1	2409	0
XM_020086125.1	calcium signal-modulating cyclophilin ligand isoform X2	2404	0
XM_020086126.1	serrate RNA effector molecule homolog isoform X1	2968	0
XM_020086127.1	serrate RNA effector molecule homolog isoform X2	2965	0
XM_020086128.1	serrate RNA effector molecule homolog isoform X6	2965	0
XM_020086129.1	serrate RNA effector molecule homolog isoform X6	2962	0
XM_020086130.1	serrate RNA effector molecule homolog isoform X6	2959	0

XM_020086131.1	serrate RNA effector molecule homolog isoform X6	2956	0
XM_020086132.1	histone RNA hairpin-binding protein-like isoform X1	1656	2.19E-178
XM_020086133.1	serrate RNA effector molecule homolog isoform X1	2982	0
XM_020086134.1	serrate RNA effector molecule homolog isoform X8	2976	0
XM_020086135.1	serrate RNA effector molecule homolog isoform X9	2881	0
XM_020086136.1	serrate RNA effector molecule homolog isoform X9	2875	0
XM_020086137.1	serrate RNA effector molecule homolog isoform X11	2869	0
XM_020086138.1	zinc finger and BTB domain-containing protein 14	5201	0
XM_020086139.1	gametogenetin-binding protein 2	2242	0
XM_020086140.1	A-kinase anchor protein 10, mitochondrial isoform X1	3904	0
XM_020086141.1	A-kinase anchor protein 10, mitochondrial isoform X2	3312	0
XM_020086142.1	histone RNA hairpin-binding protein-like isoform X2	1651	1.24E-176
XM_020086143.1	transmembrane protein 47-like	1864	1.64E-108
XM_020086144.1	PREDICTED: plastin-3-like	2438	0
XM_020086145.1	PREDICTED: plastin-3-like	2470	0
XM_020086146.1	kelch-like protein 4 isoform X1	3724	0
XM_020086147.1	non-histone chromosomal protein HMG-17-like	1582	4.08E-10
XM_020086148.1	RNA-binding protein Nova-1 isoform X1	6171	0
XM_020086149.1	RNA-binding protein Nova-1 isoform X2	5849	0
XM_020086150.1	52 kDa repressor of the inhibitor of the protein kinase-like	2728	0
XM_020086151.1	neuronal PAS domain-containing protein 2-like isoform X1	3140	0
XM_020086152.1	neuronal PAS domain-containing protein 2-like isoform X1	5401	0
XM_020086153.1	neuronal PAS domain-containing protein 2-like isoform X1	5212	0
XM_020086154.1	neurexin-2-like isoform X1	7371	0
XM_020086155.1	CXXC-type zinc finger protein 5-like isoform X1	1799	2.62E-159
XM_020086156.1	neurexin-2-like isoform X2	7344	0
XM_020086157.1	neurexin-2-like isoform X3	7281	0
XM_020086158.1	neurexin-2-like isoform X4	7254	0
XM_020086159.1	neurexin-1-beta-like isoform X5	4298	0
XM_020086160.1	zinc finger protein ubi-d4-like isoform X1	3623	0
XM_020086161.1	zinc finger protein ubi-d4-like isoform X2	3581	0
XM_020086162.1	ferredoxin-fold anticodon-binding domain-containing protein 1	2512	0
XM_020086163.1	mitochondrial import receptor subunit TOM40B-like	2483	0
XM_020086164.1	mitochondrial import receptor subunit TOM40B-like	2489	0
XM_020086165.1	CXXC-type zinc finger protein 5-like isoform X1	1710	9.57E-160
XM_020086166.1	mitochondrial import receptor subunit TOM40B-like	2304	0
XM_020086167.1	large neutral amino acids transporter small subunit 4-like isoform X1	3338	0
XM_020086168.1	large neutral amino acids transporter small subunit 4-like isoform X2	3320	0
XM_020086169.1	heterogeneous nuclear ribonucleoprotein L-like isoform X1	1893	0
XM_020086170.1	heterogeneous nuclear ribonucleoprotein L-like isoform X2	1832	0
XM_020086171.1	microtubule-associated tumor suppressor candidate 2-like isoform X1	6539	0
XM_020086172.1	microtubule-associated tumor suppressor candidate 2-like isoform X1	6603	0
XM_020086173.1	microtubule-associated tumor suppressor candidate 2-like isoform X2	6536	0
XM_020086174.1	long-chain-fatty-acid--CoA ligase 6	4048	0
XM_020086175.1	multidrug and toxin extrusion protein 1-like isoform X1	2592	0
XM_020086176.1	multidrug and toxin extrusion protein 1-like isoform X2	2573	0
XM_020086177.1	transmembrane and immunoglobulin domain-containing protein 1 isoform X1	1513	2.88E-136
XM_020086178.1	cell cycle checkpoint control protein RAD9A	2786	0
XM_020086179.1	transmembrane and immunoglobulin domain-containing protein 1 isoform X1	1567	1.58E-135
XM_020086180.1	transmembrane and immunoglobulin domain-containing protein 1 isoform X1	1334	2.08E-119
XM_020086181.1	transmembrane and immunoglobulin domain-containing protein 1 isoform X1	1330	2.62E-119
XM_020086182.1	putative ATP-dependent RNA helicase DHX33	3577	0
XM_020086183.1	P2X purinoceptor 5	2378	0
XM_020086184.1	ER membrane protein complex subunit 6	1025	6.13E-42
XM_020086185.1	ER membrane protein complex subunit 6	1022	5.98E-42
XM_020086186.1	delta-like protein B	2962	0
XM_020086187.1	cell cycle checkpoint control protein RAD9A	2624	0
XM_020086188.1	rab5 GDP/GTP exchange factor-like	3847	0
XM_020086189.1	PREDICTED: uncharacterized protein LOC109628803 isoform X1	1800	0
XM_020086190.1	PREDICTED: uncharacterized protein LOC109628803 isoform X1	1712	0
XM_020086191.1	PREDICTED: uncharacterized protein LOC109628803 isoform X1	1684	0
XM_020086192.1	PREDICTED: uncharacterized protein LOC109628803 isoform X1	1692	0
XM_020086193.1	roundabout homolog 1-like	5257	0
XM_020086194.1	claudin-7-A-like isoform X1	1786	1.12E-153
XM_020086195.1	claudin-7-B-like isoform X2	1690	1.64E-130
XM_020086196.1	sphingomyelin phosphodiesterase	2941	0
XM_020086197.1	active breakpoint cluster region-related protein isoform X1	2722	0
XM_020086198.1	active breakpoint cluster region-related protein isoform X2	2586	0
XM_020086199.1	active breakpoint cluster region-related protein isoform X3	1297	0
XM_020086200.1	cell cycle checkpoint control protein RAD9A	1758	0
XM_020086201.1	notchless protein homolog 1	2857	0
XM_020086202.1	glucose-6-phosphate exchanger SLC37A4-like	2120	0
XM_020086203.1	glucose-6-phosphate exchanger SLC37A4-like	1865	0
XM_020086204.1	glucose-6-phosphate exchanger SLC37A4-like	2067	0
XM_020086205.1	glucose-6-phosphate exchanger SLC37A4-like	1941	0
XM_020086206.1	glucose-6-phosphate exchanger SLC37A4-like	2128	0
XM_020086207.1	glucose-6-phosphate exchanger SLC37A4-like	2166	0
XM_020086208.1	glucose-6-phosphate exchanger SLC37A4-like	1913	0
XM_020086209.1	glucose-6-phosphate exchanger SLC37A4-like	3057	0

XM_020086210.1	layilin isoform X1	2385	0
XM_020086211.1	layilin isoform X2	2364	0
XM_020086212.1	alpha-1,2-mannosyltransferase ALG9	2369	0
XM_020086213.1	solute carrier family 43 member 3-like	2798	0
XM_020086214.1	transgelin isoform X1	1875	2.13E-147
XM_020086215.1	receptor-interacting serine/threonine-protein kinase 4	3103	0
XM_020086216.1	centromere/kinetochore protein zw10 homolog	3183	0
XM_020086217.1	extracellular calcium-sensing receptor-like	2983	0
XM_020086218.1	beta-1,4-galactosyltransferase 4	2479	0
XM_020086219.1	beta-1,4-galactosyltransferase 4	2247	0
XM_020086220.1	RNA-binding protein Musashi homolog 2 isoform X1	3485	0
XM_020086221.1	RNA-binding protein Musashi homolog 2 isoform X1	3488	0
XM_020086222.1	RNA-binding protein Musashi homolog 2 isoform X1	3414	0
XM_020086223.1	RNA-binding protein Musashi homolog 2 isoform X1	3428	0
XM_020086224.1	RNA-binding protein Musashi homolog 2 isoform X1	3365	0
XM_020086225.1	RNA-binding protein Musashi homolog 2 isoform X5	3304	0
XM_020086226.1	RNA-binding protein Musashi homolog 2 isoform X6	3248	0
XM_020086227.1	PREDICTED: uncharacterized protein LOC109628820	1092	0
XM_020086228.1	RNA-binding protein Musashi homolog 2 isoform X7	3184	0
XM_020086229.1	RNA-binding protein Musashi homolog 2 isoform X8	3114	0
XM_020086230.1	RNA-binding protein Musashi homolog 2 isoform X1	1093	0
XM_020086231.1	serpin H1-like isoform X2	2068	0
XM_020086232.1	protein EURL homolog	3148	0
XM_020086233.1	sperm-associated antigen 7	1763	2.86E-169
XM_020086234.1	HEAT repeat-containing protein 6	4013	0
XM_020086235.1	kelch-like protein 4 isoform X1	2364	0
XM_020086236.1	ribonucleoside-diphosphate reductase large subunit-like	2818	0
XM_020086237.1	solute carrier family 25 member 34	3221	0
XM_020086238.1	solute carrier family 25 member 45-like	2365	0
XM_020086239.1	dihydrolipoyllysine-residue acetyltransferase component of pyruvate dehydrogenase complex, mitochondrial	2479	0
XM_020086240.1	PREDICTED: uncharacterized protein DDB_G0286299-like	2586	0
XM_020086241.1	forkhead box protein O1-A-like	3942	0
XM_020086242.1	forkhead box protein O1-A-like	3849	0
XM_020086243.1	connector enhancer of kinase suppressor of ras 2-like isoform X1	4359	0
XM_020086244.1	connector enhancer of kinase suppressor of ras 2-like isoform X2	4350	0
XM_020086245.1	connector enhancer of kinase suppressor of ras 2-like isoform X3	4341	0
XM_020086246.1	connector enhancer of kinase suppressor of ras 2-like isoform X4	3527	0
XM_020086247.1	connector enhancer of kinase suppressor of ras 2-like isoform X5	4332	0
XM_020086248.1	etoposide-induced protein 2.4 homolog	2208	0
XM_020086249.1	glycogen phosphorylase, muscle form-like	2939	0
XM_020086250.1	hepatocyte cell adhesion molecule	1731	0
XM_020086251.1	mitochondrial import inner membrane translocase subunit Tim10 B	1691	2.65E-131
XM_020086252.1	ankyrin repeat domain-containing protein 13D	3159	0
XM_020086253.1	F-box only protein 50 isoform X1	1135	9.74E-173
XM_020086254.1	F-box only protein 50 isoform X2	1130	1.21E-170
XM_020086255.1	F-box only protein 50 isoform X3	1006	2.11E-143
XM_020086256.1	fasciculation and elongation protein zeta-1 isoform X1	2478	0
XM_020086257.1	fasciculation and elongation protein zeta-1 isoform X2	2392	0
XM_020086258.1	Down syndrome cell adhesion molecule homolog	8109	0
XM_020086259.1	lipase member H	2433	0
XM_020086260.1	lipase member H	1692	0
XM_020086261.1	lipase member H	1563	0
XM_020086262.1	choline-phosphate cytidylyltransferase B-like	1340	0
XM_020086263.1	SH3 domain-containing kinase-binding protein 1-like isoform X2	1414	4.97E-105
XM_020086264.1	SH3 domain-containing kinase-binding protein 1-like isoform X2	965	4.02E-94
XM_020086265.1	SH3 domain-containing kinase-binding protein 1-like isoform X2	979	4.75E-97
XM_020086266.1	signal-transducing adaptor protein 1	1273	0
XM_020086267.1	SH3 domain-containing kinase-binding protein 1-like isoform X2	879	7.13E-95
XM_020086268.1	charged multivesicular body protein 1b	1390	2.24E-121
XM_020086269.1	PREDICTED: uncharacterized protein C1orf106-like	3189	0
XM_020086270.1	PREDICTED: uncharacterized protein C1orf106-like	2947	0
XM_020086271.1	PREDICTED: uncharacterized protein C1orf106-like	3202	0
XM_020086272.1	guanine nucleotide-binding protein-like 3	2102	0
XM_020086273.1	C-X-C motif chemokine 14	2244	6.06E-57
XM_020086274.1	integrin alpha-5-like	3997	0
XM_020086275.1	protein snail homolog Sna-like	2341	2.77E-147
XM_020086276.1	dynein heavy chain 1, axonemal	13049	0
XM_020086277.1	protein tyrosine phosphatase domain-containing protein 1-like	3271	0
XM_020086278.1	receptor tyrosine-protein kinase erbB-3-like isoform X1	3668	0
XM_020086279.1	receptor tyrosine-protein kinase erbB-3-like isoform X1	3650	0
XM_020086280.1	probable nuclear hormone receptor HR38 isoform X1	1991	0
XM_020086281.1	probable nuclear hormone receptor HR38 isoform X2	3324	0
XM_020086282.1	Krueppel-like factor 15 isoform X1	3808	0
XM_020086283.1	Krueppel-like factor 15 isoform X1	3832	0
XM_020086284.1	Krueppel-like factor 15 isoform X1	3724	0
XM_020086285.1	transcription factor SOX-6-like isoform X2	5052	0
XM_020086286.1	annexin A6 isoform X1	2593	0
XM_020086287.1	transcription factor SOX-6-like isoform X2	5049	0
XM_020086288.1	transcription factor 15-like	1797	2.49E-115

XM_020086289.1	coiled-coil domain-containing protein 174 isoform X1	2281	0
XM_020086290.1	coiled-coil domain-containing protein 174 isoform X2	2278	0
XM_020086291.1	26S proteasome non-ATPase regulatory subunit 6	1382	0
XM_020086292.1	ataxin-7-like protein 1	3105	0
XM_020086293.1	ataxin-7-like protein 1	3814	0
XM_020086294.1	annexin A6 isoform X2	2578	0
XM_020086295.1	phosphatase and actin regulator 3-like	2492	0
XM_020086296.1	THUMP domain-containing protein 3	2175	0
XM_020086297.1	THUMP domain-containing protein 3	2093	0
XM_020086298.1	melanocyte protein PMEL-like	2952	0
XM_020086299.1	rho guanine nucleotide exchange factor 25-like isoform X1	3105	0
XM_020086300.1	rho guanine nucleotide exchange factor 25-like isoform X2	3102	0
XM_020086301.1	rho guanine nucleotide exchange factor 25-like isoform X3	3084	0
XM_020086302.1	rho guanine nucleotide exchange factor 25-like isoform X4	3007	0
XM_020086303.1	liprin-alpha-4 isoform X1	5398	0
XM_020086304.1	liprin-alpha-4 isoform X2	5300	0
XM_020086305.1	liprin-alpha-4 isoform X3	5371	0
XM_020086306.1	liprin-alpha-4 isoform X4	5273	0
XM_020086307.1	high mobility group nucleosome-binding domain-containing protein 5	1421	5.66E-08
XM_020086308.1	liprin-alpha-4 isoform X5	5335	0
XM_020086309.1	dnaJ homolog subfamily C member 11-like isoform X1	3955	0
XM_020086310.1	dnaJ homolog subfamily C member 11-like isoform X1	3925	0
XM_020086311.1	solute carrier family 2, facilitated glucose transporter member 9	1816	0
XM_020086312.1	solute carrier family 2, facilitated glucose transporter member 9	1816	0
XM_020086313.1	protein-glutamine gamma-glutamyltransferase 2-like	2494	0
XM_020086314.1	neuroendocrine convertase 1	4674	0
XM_020086315.1	kelch-like protein 4 isoform X1	1897	0
XM_020086316.1	CXXC-type zinc finger protein 1-like	2133	0
XM_020086317.1	CXXC-type zinc finger protein 1-like	2127	0
XM_020086318.1	cadherin-4-like isoform X3	4602	0
XM_020086319.1	cadherin-4-like isoform X3	4593	0
XM_020086320.1	integral membrane protein 2A	1972	1.07E-180
XM_020086321.1	cadherin-4-like isoform X3	4401	0
XM_020086322.1	cadherin-4-like isoform X3	4460	0
XM_020086323.1	microspherule protein 1	1961	0
XM_020086324.1	serine/threonine-protein kinase Nek4 isoform X1	3482	0
XM_020086325.1	serine/threonine-protein kinase Nek4 isoform X2	3473	0
XM_020086326.1	serine/threonine-protein kinase Nek4 isoform X3	3443	0
XM_020086327.1	EF-hand and coiled-coil domain-containing protein 1-like isoform X1	3373	0
XM_020086328.1	EF-hand and coiled-coil domain-containing protein 1-like isoform X2	3318	0
XM_020086329.1	homeobox protein MSX-1	1860	0
XM_020086330.1	protein canopy homolog 2	537	1.79E-68
XM_020086331.1	sodium-coupled neutral amino acid transporter 3-like isoform X1	4656	0
XM_020086332.1	sodium-coupled neutral amino acid transporter 3-like isoform X1	4623	0
XM_020086333.1	disintegrin and metalloproteinase domain-containing protein 19	4433	0
XM_020086334.1	ETS domain-containing protein Elk-3-like	4031	0
XM_020086335.1	protein kish-B	1673	1.31E-43
XM_020086336.1	tRNA (guanine-N(7)-)-methyltransferase	1186	0
XM_020086337.1	CUGBP Elav-like family member 4	3033	0
XM_020086338.1	protein FAM83D	2290	0
XM_020086339.1	RING1 and YY1-binding protein	1780	1.36E-108
XM_020086340.1	PREDICTED: contactin-3-like	6932	0
XM_020086341.1	NEDD8-activating enzyme E1 catalytic subunit isoform X2	1840	0
XM_020086342.1	NEDD8-activating enzyme E1 catalytic subunit isoform X2	2094	0
XM_020086343.1	NEDD8-activating enzyme E1 catalytic subunit isoform X2	1962	0
XM_020086344.1	dystrophin-related protein 2	3122	0
XM_020086345.1	von Hippel-Lindau disease tumor suppressor	1764	3.86E-117
XM_020086346.1	PREDICTED: sortilin-like	3874	0
XM_020086347.1	myogenic factor 6 isoform X1	1677	4.63E-175
XM_020086348.1	protein FAM72A	3158	6.13E-86
XM_020086349.1	RNA-binding protein 5-like isoform X1	3067	0
XM_020086350.1	RNA-binding protein 5-like isoform X2	2926	0
XM_020086351.1	dystrophin-related protein 2	2901	0
XM_020086352.1	RNA-binding protein 5-like isoform X1	3349	0
XM_020086353.1	PREDICTED: uncharacterized protein LOC109628903	2580	0
XM_020086354.1	MAP kinase-activated protein kinase 2-like	2710	0
XM_020086355.1	heme transporter hrg1-A-like	1711	4.17E-83
XM_020086356.1	inositol hexakisphosphate kinase 1	1971	0
XM_020086357.1	zinc finger matrin-type protein 1-like	2155	0
XM_020086358.1	complement C1q-like protein 3	4206	9.69E-119
XM_020086359.1	guanine nucleotide exchange factor MSS4	724	1.46E-93
XM_020086360.1	small nuclear ribonucleoprotein E	582	7.87E-53
XM_020086361.1	ras-GEF domain-containing family member 1C isoform X1	1975	0
XM_020086362.1	single-strand selective monofunctional uracil DNA glycosylase	1639	0
XM_020086363.1	V-set and transmembrane domain-containing protein 2-like protein	1058	2.62E-159
XM_020086364.1	transmembrane protein 233	696	4.85E-61
XM_020086365.1	protein hinderin	1975	0
XM_020086366.1	FYVE, RhoGEF and PH domain-containing protein 5-like isoform X1	5575	0
XM_020086367.1	FYVE, RhoGEF and PH domain-containing protein 5-like isoform X2	5548	0

XM_020086368.1	ras-GEF domain-containing family member 1C isoform X1	1862	0
XM_020086369.1	PAK4-inhibitor inka2-like	1284	0
XM_020086370.1	PREDICTED: caveolin-3	1326	7.15E-107
XM_020086371.1	PREDICTED: contactin-4-like	5145	0
XM_020086372.1	EF-hand and coiled-coil domain-containing protein 1	2782	0
XM_020086373.1	small vasohibin-binding protein	901	4.17E-40
XM_020086374.1	small vasohibin-binding protein	902	4.29E-40
XM_020086375.1	small vasohibin-binding protein	1099	2.04E-39
XM_020086376.1	ras-GEF domain-containing family member 1C isoform X1	1898	0
XM_020086377.1	sodium-dependent neutral amino acid transporter SLC6A17-like	2474	0
XM_020086378.1	sodium-dependent neutral amino acid transporter SLC6A17-like	4173	0
XM_020086379.1	E3 ubiquitin-protein ligase Midline-1-like	1715	0
XM_020086380.1	opioid growth factor receptor-like protein 1	1941	0
XM_020086381.1	potassium channel subfamily K member 9-like isoform X1	1441	0
XM_020086382.1	potassium channel subfamily K member 9-like isoform X1	1255	0
XM_020086383.1	potassium channel subfamily K member 3-like isoform X3	1159	0
XM_020086384.1	D(1B) dopamine receptor	3433	0
XM_020086385.1	ras-GEF domain-containing family member 1C isoform X1	1973	0
XM_020086386.1	cas scaffolding protein family member 4	3169	0
XM_020086387.1	arf-GAP with GTPase, ANK repeat and PH domain-containing protein 2	4139	0
XM_020086388.1	peptidyl-prolyl cis-trans isomerase FKBP11	584	1.10E-75
XM_020086389.1	ellis-van Creveld syndrome protein	3271	0
XM_020086390.1	metabotropic glutamate receptor 2-like isoform X1	3479	0
XM_020086391.1	metabotropic glutamate receptor 2-like isoform X1	3452	0
XM_020086392.1	trans-acting T-cell-specific transcription factor GATA-3 isoform X1	2371	0
XM_020086393.1	kelch-like protein 4 isoform X1	1805	0
XM_020086394.1	apolipoprotein F	3092	0
XM_020086395.1	zinc finger protein 385A-like isoform X1	3074	0
XM_020086396.1	zinc finger protein 385A-like isoform X2	3091	0
XM_020086397.1	DNA polymerase alpha subunit B	2411	0
XM_020086398.1	myoD family inhibitor domain-containing protein isoform X1	5014	1.66E-100
XM_020086399.1	probable G-protein coupled receptor	5474	0
XM_020086400.1	histone deacetylase 11	3687	0
XM_020086401.1	25-hydroxyvitamin D-1 alpha hydroxylase, mitochondrial	1799	0
XM_020086402.1	major facilitator superfamily domain-containing protein 4A-like	2853	0
XM_020086403.1	amphoterin-induced protein 1-like	4652	0
XM_020086404.1	probable G-protein coupled receptor 22	4620	0
XM_020086405.1	DNA polymerase alpha subunit B	2293	0
XM_020086406.1	F-box only protein 2 isoform X1	2057	1.04E-125
XM_020086407.1	F-box only protein 2 isoform X1	2045	9.27E-126
XM_020086408.1	F-box only protein 2 isoform X1	2051	2.97E-124
XM_020086409.1	PREDICTED: prolargin	1944	0
XM_020086410.1	probable G-protein coupled receptor 173	2508	0
XM_020086411.1	probable G-protein coupled receptor 22	3969	0
XM_020086412.1	protein FAM19A1-like isoform X1	2659	1.05E-92
XM_020086413.1	protein FAM19A1-like isoform X1	1905	4.61E-95
XM_020086414.1	protein FAM19A1-like isoform X1	2567	1.12E-89
XM_020086415.1	protein phosphatase 1 regulatory subunit 3D-like	3488	1.76E-179
XM_020086416.1	kinesin-like protein KIF21B	3287	0
XM_020086417.1	sestrin-1 isoform X2	1150	1.00E-122
XM_020086418.1	ras association domain-containing protein 5	2761	5.41E-167
XM_020086419.1	sodium- and chloride-dependent neutral and basic amino acid transporter B(0+)-like	2815	0
XM_020086420.1	bile acid-CoA:amino acid N-acyltransferase-like	1494	0
XM_020086421.1	SPRY domain-containing protein 3-like	3707	0
XM_020086422.1	protein lifeguard 2-like	768	0
XM_020086423.1	low-density lipoprotein receptor-related protein 1-like	6495	0
XM_020086424.1	extended synaptotagmin-1-like	6358	0
XM_020086425.1	inositol 1,4,5-trisphosphate receptor type 1	9475	0
XM_020086426.1	protein PXR1-like	796	5.76E-96
XM_020086427.1	MICOS complex subunit MIC27	2262	0
XM_020086428.1	ATP-dependent 6-phosphofructokinase, muscle type-like	7830	0
XM_020086429.1	SWI/SNF complex subunit SMARCC1-like	3184	0
XM_020086430.1	receptor tyrosine-protein kinase erbB-4-like	544	2.31E-131
XM_020086431.1	vasopressin V2 receptor-like	1041	0
XM_020086432.1	appetite-regulating hormone	318	7.96E-62
XM_020086433.1	PREDICTED: uncharacterized protein C6orf132-like	3338	0
XM_020086434.1	protein FAM107B-like	525	1.37E-105
XM_020086435.1	transforming acidic coiled-coil-containing protein 1-like	4602	0
XM_020086436.1	titin homolog isoform X3	4950	0
XM_020086437.1	death ligand signal enhancer	2821	0
XM_020086438.1	dynein intermediate chain 1, axonemal	3833	0
XM_020086439.1	DDB1- and CUL4-associated factor 12	3155	0
XM_020086440.1	PREDICTED: otopetrin-1	1404	0
XM_020086441.1	A disintegrin and metalloproteinase with thrombospondin motifs 9	6778	0
XM_020086442.1	membrane-associated guanylate kinase, WW and PDZ domain-containing protein 1-like	3161	0
XM_020086443.1	mitochondrial intermembrane space import and assembly protein 40	1257	2.78E-86
XM_020086444.1	zinc finger CCCH domain-containing protein 13-like	3168	0
XM_020086445.1	cadherin-like protein 26	2854	0
XM_020086446.1	death-inducer obliterator 1-like	7743	0

XM_020086447.1	protein kinase C-binding protein 1-like	3222	0
XM_020086448.1	UV-stimulated scaffold protein A isoform X1	2837	0
XM_020086449.1	nuclear receptor coactivator 3	5001	0
XM_020086450.1	gamma-glutamyltransferase 7-like	2952	0
XM_020086451.1	blue-sensitive opsin-like	795	9.08E-159
XM_020086452.1	guanine nucleotide-binding protein-like 3-like protein	2059	0
XM_020086453.1	histone-lysine N-methyltransferase 2A isoform X1	2045	0
XM_020086454.1	SRSF protein kinase 1-like	2109	0
XM_020086455.1	inositol 1,4,5-trisphosphate receptor type 1-like	9923	0
XM_020086456.1	UV-stimulated scaffold protein A isoform X2	2834	0
XM_020086457.1	protein SHQ1 homolog	1507	0
XM_020086458.1	intraflagellar transport protein 52 homolog	1422	0
XM_020086459.1	cyclin-dependent kinase 18	1618	0
XM_020086460.1	AP-5 complex subunit beta-1-like	2127	0
XM_020086461.1	kinetochore protein Nuf2-like	843	0
XM_020086462.1	UV-stimulated scaffold protein A isoform X3	2831	0
XM_020086463.1	PREDICTED: uncharacterized protein KIAA1257 homolog	3109	0
XM_020086464.1	fibulin-2-like isoform X1	4917	0
XM_020086465.1	constitutive coactivator of peroxisome proliferator-activated receptor gamma isoform X1	5974	0
XM_020086466.1	human immunodeficiency virus type I enhancer-binding protein 2 homolog	6040	0
XM_020086467.1	copine-5-like isoform X2	3195	0
XM_020086468.1	transcriptional repressor scratch 2-like	1017	1.11E-180
XM_020086469.1	PREDICTED: plexin-B1-like	9665	0
XM_020086470.1	UV-stimulated scaffold protein A isoform X4	2828	0
XM_020086471.1	PREDICTED: plexin-B1-like	9660	0
XM_020086472.1	PREDICTED: plexin-B1-like	9576	0
XM_020086473.1	SET domain-containing protein 5 isoform X1	8218	0
XM_020086474.1	SET domain-containing protein 5 isoform X2	8189	0
XM_020086475.1	SET domain-containing protein 5 isoform X3	8123	0
XM_020086476.1	SET domain-containing protein 5 isoform X4	8094	0
XM_020086477.1	zinc finger protein ZXDC-like	4550	0
XM_020086478.1	cytosolic 10-formyltetrahydrofolate dehydrogenase-like	3214	0
XM_020086479.1	myotubularin-related protein 14	4007	0
XM_020086480.1	PREDICTED: uncharacterized protein LOC109629011	2062	0
XM_020086481.1	Krueppel-like factor 15	3480	0
XM_020086482.1	gastrula zinc finger protein XlCGF48.2-like isoform X1	1738	0
XM_020086483.1	palladin isoform X6	5315	0
XM_020086484.1	zinc finger protein 2 homolog isoform X2	1792	0
XM_020086485.1	lipoma HMGIC fusion partner-like 4 protein	1482	7.88E-163
XM_020086486.1	lipoma HMGIC fusion partner-like 4 protein	1190	7.73E-164
XM_020086487.1	PREDICTED: endothelin-3-like	3582	3.21E-116
XM_020086488.1	probable peptidyl-tRNA hydrolase 2	2077	7.30E-116
XM_020086489.1	probable peptidyl-tRNA hydrolase 2	2063	6.55E-116
XM_020086490.1	ubiquitin-conjugating enzyme E2 variant 1-like	576	1.01E-89
XM_020086491.1	ubiquitin-conjugating enzyme E2 variant 1-like	554	7.67E-90
XM_020086492.1	p53 and DNA damage-regulated protein 1	607	1.68E-90
XM_020086493.1	microphthalmia-associated transcription factor-like isoform X1	1799	0
XM_020086494.1	microphthalmia-associated transcription factor-like isoform X2	1775	0
XM_020086495.1	dead end protein 1-like isoform X1	4223	0
XM_020086496.1	poly [ADP-ribose] polymerase 3	2253	0
XM_020086497.1	proteasome subunit alpha type-4	1129	3.20E-180
XM_020086498.1	proteasome subunit alpha type-4	1198	7.71E-180
XM_020086499.1	proteasome subunit alpha type-4	1081	1.73E-180
XM_020086500.1	ubiquitin carboxyl-terminal hydrolase CYLD isoform X1	2666	0
XM_020086501.1	cyclin-dependent kinase inhibitor 1-like	1841	7.79E-112
XM_020086502.1	E3 ubiquitin-protein ligase RAD18 isoform X1	1711	0
XM_020086503.1	E3 ubiquitin-protein ligase RAD18 isoform X2	1766	0
XM_020086504.1	dead end protein 1-like isoform X2	4070	0
XM_020086505.1	E3 ubiquitin-protein ligase RAD18 isoform X3	1666	0
XM_020086506.1	myosin regulatory light chain 2, ventricular/cardiac muscle isoform	879	3.01E-121
XM_020086507.1	putative deoxyribonuclease TATDN2	2610	0
XM_020086508.1	catechol O-methyltransferase domain-containing protein 1-like	2617	0
XM_020086509.1	MORC family CW-type zinc finger protein 3-like	3474	0
XM_020086510.1	N-acylethanolamine-hydrolyzing acid amidase	1306	0
XM_020086511.1	MICOS complex subunit MIC25	1117	1.21E-128
XM_020086512.1	protein RFT1 homolog	2187	0
XM_020086513.1	E3 ubiquitin-protein ligase NRDP1	1202	0
XM_020086514.1	E3 ubiquitin-protein ligase NRDP1	1115	0
XM_020086515.1	prefoldin subunit 5	684	6.27E-99
XM_020086516.1	protein Wnt-7a	2227	0
XM_020086517.1	twist-related protein 2-like	2049	1.84E-120

XM_020086518.1	villin-1-like isoform X1	2488	0
XM_020086519.1	acyl-coenzyme A thioesterase 8	1243	0
XM_020086520.1	acidic mammalian chitinase isoform X1	1618	0
XM_020086521.1	acidic mammalian chitinase isoform X2	1478	0
XM_020086522.1	copine-5-like isoform X2	3294	0
XM_020086523.1	oxytocin receptor	1562	0
XM_020086524.1	histone-lysine N-methyltransferase SETMAR	1101	0
XM_020086525.1	prostacyclin synthase	1643	0

XM_020086526.1	contactin-4-like isoform X1	4268	0
XM_020086527.1	contactin-4-like isoform X2	3090	0
XM_020086528.1	contactin-4-like isoform X3	4161	0
XM_020086529.1	PREDICTED: uncharacterized protein LOC109629048	1155	3.36E-141
XM_020086530.1	PREDICTED: uncharacterized protein LOC109629048	1202	6.02E-141
XM_020086531.1	coiled-coil domain-containing protein 120-like	2569	0
XM_020086532.1	cilia- and flagella-associated protein 57	4019	0
XM_020086533.1	matrix-remodeling-associated protein 8-like	2177	0
XM_020086534.1	protein unc-119 homolog B-like	2220	1.55E-169
XM_020086535.1	MAP kinase-activated protein kinase 2-like	1807	0
XM_020086536.1	PREDICTED: trypsin-like	762	6.73E-162
XM_020086537.1	vasopressin V2 receptor-like	1101	0
XM_020086538.1	adenosine receptor A1-like	3150	0
XM_020086539.1	transmembrane protein 128	1072	3.91E-119
XM_020086540.1	chicken-type gonadotropin-releasing hormone 2 precursor	584	5.20E-46
XM_020086541.1	matrix metalloproteinase-19-like	1986	0
XM_020086542.1	PREDICTED: uncharacterized protein LOC109629062	1701	0
XM_020086543.1	PREDICTED: netrin-4-like	2794	0
XM_020086544.1	olfactomedin-like protein 1	3691	0
XM_020086545.1	calcium/calmodulin-dependent protein kinase II inhibitor 2-like	1283	5.90E-52
XM_020086546.1	coiled-coil domain-containing protein 3-like	2268	0
XM_020086547.1	trans-acting T-cell-specific transcription factor GATA-3 isoform X1	1740	0
XM_020086548.1	cytokine-like protein 1	1363	3.77E-86
XM_020086549.1	dysbindin-like isoform X2	1217	2.38E-107
XM_020086550.1	globoside alpha-1,3-N-acetylgalactosaminyltransferase 1-like isoform X1	2151	0
XM_020086551.1	globoside alpha-1,3-N-acetylgalactosaminyltransferase 1-like isoform X2	2088	0
XM_020086552.1	CD8 alpha chain	1737	6.63E-127
XM_020086553.1	calcium-binding and coiled-coil domain-containing protein 1-like	2734	0
XM_020086554.1	neurogenic differentiation factor 4-like	1663	0
XM_020086555.1	insulin-like growth factor-binding protein 5	1139	6.39E-129
XM_020086556.1	potassium voltage-gated channel subfamily B member 1-like	2415	0
XM_020086557.1	PREDICTED: lumican-like	2005	0
XM_020086558.1	interleukin-10 precursor	1026	1.35E-121
XM_020086559.1	beta-crystallin B3-like	987	0
XM_020086560.1	E3 ubiquitin-protein ligase MARCH9-like	1842	0
XM_020086561.1	phosphatidylinositol 5-phosphate 4-kinase type-2 gamma-like	1221	0
XM_020086562.1	mucin-5AC-like isoform X1	1949	0
XM_020086563.1	tripartite motif-containing protein 16-like protein	1857	0
XM_020086564.1	kelch domain-containing protein 8A	1786	0
XM_020086565.1	guanylyl cyclase-activating protein 1-like	1077	3.63E-139
XM_020086566.1	retinol dehydrogenase 10-like	948	0
XM_020086567.1	homeobox protein BarH-like 1	1441	5.45E-159
XM_020086568.1	ammonium transporter Rh type B-like	1546	0
XM_020086569.1	trypsinogen 3	1156	0
XM_020086570.1	probable tubulin polyglutamylase TTLL1	2814	0
XM_020086571.1	transcription factor 7-like 2	1665	0
XM_020086572.1	transcription factor 7-like 2	1506	0
XM_020086573.1	transcription factor 7-like 2	1648	0
XM_020086574.1	nicalin isoform X3	1734	0
XM_020086575.1	probable G-protein coupled receptor 61	1404	0
XM_020086576.1	glucagon family neuropeptides-like	834	1.79E-101
XM_020086577.1	glucagon family neuropeptides-like	782	9.48E-102
XM_020086578.1	toxin MIT1-like	616	4.33E-91
XM_020086579.1	T-cell surface glycoprotein CD8 beta chain	1127	5.06E-117
XM_020086580.1	solute carrier family 2, facilitated glucose transporter member 11-like	1662	0
XM_020086581.1	serine/threonine/tyrosine-interacting-like protein 1	917	0
XM_020086582.1	PREDICTED: urocortin-3-like	1000	9.33E-81
XM_020086583.1	ankyrin repeat domain-containing protein 33B-like	1377	0
XM_020086584.1	intraflagellar transport protein 52 homolog	340	1.01E-73
XM_020086585.1	DEP domain-containing mTOR-interacting protein-like	407	9.73E-87
XM_020086586.1	myosin-7-like isoform X1	6157	0
XM_020086587.1	myosin-7-like isoform X2	7798	0
XM_020086588.1	myosin-7-like isoform X1	5454	0
XM_020086589.1	short transient receptor potential channel 4-associated protein	3482	0
XM_020086590.1	src-like-adapter 2	1290	4.73E-180
XM_020086591.1	src-like-adapter 2	1460	2.32E-178
XM_020086592.1	Fanconi anemia group D2 protein	4963	0
XM_020086593.1	Fanconi anemia group D2 protein	5042	0
XM_020086594.1	ubiquitin carboxyl-terminal hydrolase 4-like	3539	0
XM_020086595.1	ER membrane protein complex subunit 3	1775	0
XM_020086596.1	ER membrane protein complex subunit 3	1807	0
XM_020086597.1	ER membrane protein complex subunit 3	1517	0
XM_020086598.1	glutathione peroxidase 1-like	994	2.05E-134
XM_020086599.1	dnaJ homolog subfamily C member 14	5422	0
XM_020086600.1	NGFI-A-binding protein 1	3264	0
XM_020086601.1	inositol polyphosphate 1-phosphatase	1558	0
XM_020086602.1	inositol polyphosphate 1-phosphatase	1464	0
XM_020086603.1	protein SYS1 homolog	2765	3.48E-104
XM_020086604.1	neuritin-like protein	3803	3.36E-93

XM_020086605.1	protein S100-B-like	933	1.39E-65
XM_020086606.1	leucine-rich repeat neuronal protein 1-like	3461	0
XM_020086607.1	leucine-rich repeat neuronal protein 1-like	3368	0
XM_020086608.1	leucine-rich repeat neuronal protein 1-like	3359	0
XM_020086609.1	leucine-rich repeat neuronal protein 1-like	3544	0
XM_020086610.1	ras-related protein Rab-33A	3657	0
XM_020086611.1	class E basic helix-loop-helix protein 40-like	2603	0
XM_020086612.1	sulfatase-modifying factor 1	1744	0
XM_020086613.1	blue-sensitive opsin-like	1056	0
XM_020086614.1	inter-alpha-trypsin inhibitor heavy chain H3-like	3840	0
XM_020086615.1	inter-alpha-trypsin inhibitor heavy chain H3	2860	0
XM_020086616.1	scm-like with four MBT domains protein 1 isoform X1	4147	0
XM_020086617.1	scm-like with four MBT domains protein 1 isoform X2	4144	0
XM_020086618.1	musculoskeletal embryonic nuclear protein 1-like isoform X1	1031	1.30E-50
XM_020086619.1	store-operated calcium entry regulator STIMATE-like isoform X2	2691	0
XM_020086620.1	lysine-specific demethylase 5B-like	5954	0
XM_020086621.1	negative elongation factor C/D	2206	0
XM_020086622.1	cathepsin Z-like	1702	0
XM_020086623.1	disco-interacting protein 2 homolog B-A isoform X1	7902	0
XM_020086624.1	disco-interacting protein 2 homolog B-A isoform X2	7878	0
XM_020086625.1	cyclic AMP-dependent transcription factor ATF-7-like isoform X1	3339	0
XM_020086626.1	cyclic AMP-dependent transcription factor ATF-7-like isoform X1	3343	0
XM_020086627.1	cyclic AMP-dependent transcription factor ATF-7-like isoform X1	3326	0
XM_020086628.1	cyclic AMP-dependent transcription factor ATF-7-like isoform X1	3324	0
XM_020086629.1	cyclic AMP-dependent transcription factor ATF-7-like isoform X1	3313	0
XM_020086630.1	cyclic AMP-dependent transcription factor ATF-7-like isoform X1	1653	0
XM_020086631.1	cyclic AMP-dependent transcription factor ATF-1-like isoform X1	1798	1.21E-149
XM_020086632.1	cyclic AMP-dependent transcription factor ATF-1-like isoform X2	1797	4.13E-150
XM_020086633.1	cyclic AMP-dependent transcription factor ATF-1-like isoform X3	1698	1.45E-127
XM_020086634.1	methyltransferase-like protein 7A	1159	2.38E-158
XM_020086635.1	host cell factor 1 isoform X1	6619	0
XM_020086636.1	host cell factor 1 isoform X1	5431	0
XM_020086637.1	host cell factor 1 isoform X1	5427	0
XM_020086638.1	host cell factor 1 isoform X1	5306	0
XM_020086639.1	host cell factor 1 isoform X1	6616	0
XM_020086640.1	ras-related protein rab7-like	2614	2.64E-146
XM_020086641.1	eukaryotic translation initiation factor 4B isoform X4	2398	1.24E-124
XM_020086642.1	eukaryotic translation initiation factor 4B isoform X4	2305	2.41E-131
XM_020086643.1	vesicle transport protein SFT2B-like	1324	9.98E-112
XM_020086644.1	eukaryotic translation initiation factor 4B isoform X4	2178	1.38E-131
XM_020086645.1	eukaryotic translation initiation factor 4B isoform X4	2111	2.29E-131
XM_020086646.1	keratin, type I cytoskeletal 18-like	1622	0
XM_020086647.1	ATP-dependent RNA helicase DDX19B	3823	0
XM_020086648.1	tRNA pseudouridine synthase-like 1 isoform X1	1203	0
XM_020086649.1	tRNA pseudouridine synthase-like 1 isoform X1	1137	0
XM_020086650.1	tRNA pseudouridine synthase-like 1 isoform X1	1136	0
XM_020086651.1	tRNA pseudouridine synthase-like 1 isoform X1	1164	0
XM_020086652.1	tRNA pseudouridine synthase-like 1 isoform X1	1093	0
XM_020086653.1	arf-GAP with coiled-coil, ANK repeat and PH domain-containing protein 3	3607	0
XM_020086654.1	leucine-rich repeats and immunoglobulin-like domains protein 1	5837	0
XM_020086655.1	S-adenosylmethionine mitochondrial carrier protein	2146	3.06E-175
XM_020086656.1	cGMP-dependent protein kinase 2-like	2816	0
XM_020086657.1	dnaJ homolog subfamily C member 16	4010	0
XM_020086658.1	dnaJ homolog subfamily C member 16	3864	0
XM_020086659.1	beta-1,3-galactosyltransferase 6 isoform X1	3323	0
XM_020086660.1	beta-1,3-galactosyltransferase 6 isoform X1	2538	0
XM_020086661.1	beta-1,3-galactosyltransferase 6 isoform X1	3626	0
XM_020086662.1	beta-1,3-galactosyltransferase 6 isoform X1	3664	0
XM_020086663.1	cytidine deaminase isoform X1	1056	2.00E-130
XM_020086664.1	cytidine deaminase isoform X2	1014	3.44E-120
XM_020086665.1	ATP-dependent 6-phosphofructokinase, muscle type-like	2589	0
XM_020086666.1	ATP-dependent 6-phosphofructokinase, muscle type-like	1078	2.07E-93
XM_020086667.1	blue-sensitive opsin-like	1062	0
XM_020086668.1	PREDICTED: uncharacterized protein LOC109629162	3343	0
XM_020086669.1	protein OS-9 isoform X1	3596	0
XM_020086670.1	protein OS-9 isoform X2	3593	0
XM_020086671.1	glycoprotein-N-acetylgalactosamine 3-beta-galactosyltransferase 1-B-like	4880	0
XM_020086672.1	glycoprotein-N-acetylgalactosamine 3-beta-galactosyltransferase 1-B-like	4240	0
XM_020086673.1	membrane-associated guanylate kinase, WW and PDZ domain-containing protein 1-like isoform X1	3234	0
XM_020086674.1	membrane-associated guanylate kinase, WW and PDZ domain-containing protein 1-like isoform X2	3215	0
XM_020086675.1	dual specificity tyrosine-phosphorylation-regulated kinase 2-like	5290	0
XM_020086676.1	cell division control protein 42 homolog isoform X1	2057	1.07E-133
XM_020086677.1	cell division control protein 42 homolog isoform X1	2759	6.29E-132
XM_020086678.1	kinesin-like protein KIF21B isoform X1	9806	0
XM_020086679.1	kinesin-like protein KIF21B isoform X2	9767	0
XM_020086680.1	polyribonucleotide nucleotidyltransferase 1, mitochondrial	2979	0
XM_020086681.1	kinesin-like protein KIF21B isoform X3	9668	0
XM_020086682.1	lipoma HMGIC fusion partner-like 3 protein isoform X1	1070	8.63E-147
XM_020086683.1	gastrula zinc finger protein XlCGF57.1-like isoform X1	5333	0

XM_020086684.1	gastrula zinc finger protein XlCGF57.1-like isoform X1	5215	0
XM_020086685.1	gastrula zinc finger protein XlCGF57.1-like isoform X1	5301	0
XM_020086686.1	gastrula zinc finger protein XlCGF57.1-like isoform X1	5302	0
XM_020086687.1	gastrula zinc finger protein XlCGF57.1-like isoform X1	5248	0
XM_020086688.1	gastrula zinc finger protein XlCGF57.1-like isoform X1	5249	0
XM_020086689.1	zinc finger protein 2-like isoform X6	5249	0
XM_020086690.1	zinc finger protein 2-like isoform X7	5249	0
XM_020086691.1	gastrula zinc finger protein XlCGF57.1-like isoform X1	5249	0
XM_020086692.1	gastrula zinc finger protein XlCGF57.1-like isoform X1	5250	0
XM_020086693.1	gastrula zinc finger protein XlCGF57.1-like isoform X1	5250	0
XM_020086694.1	zinc finger protein 2-like isoform X11	5216	0
XM_020086695.1	transcription factor COE3 isoform X1	4426	0
XM_020086696.1	zinc finger protein 2-like isoform X12	2231	0
XM_020086697.1	gastrula zinc finger protein XlCGF57.1-like isoform X13	5217	0
XM_020086698.1	zinc finger protein 2 homolog isoform X14	5218	0
XM_020086699.1	zinc finger protein 2 homolog isoform X15	5219	0
XM_020086700.1	gastrula zinc finger protein XlCGF57.1-like isoform X16	5164	0
XM_020086701.1	gastrula zinc finger protein XlCGF57.1-like isoform X17	5164	0
XM_020086702.1	gastrula zinc finger protein XlCGF57.1-like isoform X18	5166	0
XM_020086703.1	gastrula zinc finger protein XlCGF57.1-like isoform X19	5132	0
XM_020086704.1	gastrula zinc finger protein XlCGF57.1-like isoform X20	5133	0
XM_020086705.1	gastrula zinc finger protein XlCGF57.1-like isoform X21	5134	0
XM_020086706.1	transcription factor COE3 isoform X2	4405	0
XM_020086707.1	gastrula zinc finger protein XlCGF57.1-like isoform X22	5101	0
XM_020086708.1	zinc finger protein 260-like isoform X23	5079	0
XM_020086709.1	zinc finger protein 260-like isoform X24	5079	0
XM_020086710.1	gastrula zinc finger protein XlCGF57.1-like isoform X25	5082	0
XM_020086711.1	gastrula zinc finger protein XlCGF57.1-like isoform X26	5048	0
XM_020086712.1	gastrula zinc finger protein XlCGF57.1-like isoform X27	4992	0
XM_020086713.1	gastrula zinc finger protein XlCGF57.1-like isoform X28	4964	0
XM_020086714.1	lysyl oxidase homolog 2A-like isoform X1	2522	0
XM_020086715.1	lysyl oxidase homolog 2A-like isoform X1	2219	0
XM_020086716.1	transcription factor COE3 isoform X3	4402	0
XM_020086717.1	R3H and coiled-coil domain-containing protein 1	2884	0
XM_020086718.1	golgin subfamily A member 7-like	2098	1.60E-97
XM_020086719.1	ubiquitin-conjugating enzyme E2 R2	7272	1.08E-143
XM_020086720.1	Rieske domain-containing protein-like isoform X1	849	8.51E-117
XM_020086721.1	bis(5'-nucleosyl)-tetraphosphatase [asymmetrical]	892	4.68E-106
XM_020086722.1	rho GTPase-activating protein 39-like isoform X1	4989	0
XM_020086723.1	rho GTPase-activating protein 39-like isoform X1	4959	0
XM_020086724.1	transcription factor COE3 isoform X4	4348	0
XM_020086725.1	rho GTPase-activating protein 39-like isoform X1	4965	0
XM_020086726.1	plexin-A1 isoform X1	8221	0
XM_020086727.1	plexin-A1 isoform X1	8199	0
XM_020086728.1	plexin-A1 isoform X1	8215	0
XM_020086729.1	paxillin isoform X1	4155	0
XM_020086730.1	paxillin isoform X2	3998	0
XM_020086731.1	protein polybromo-1-like isoform X1	4362	0
XM_020086732.1	protein polybromo-1-like isoform X2	3884	0
XM_020086733.1	transcription factor COE3 isoform X5	4327	0
XM_020086734.1	methylosome protein 50	1635	0
XM_020086735.1	sodium channel protein type 8 subunit alpha-like isoform X1	8983	0
XM_020086736.1	sodium channel protein type 8 subunit alpha-like isoform X2	8950	0
XM_020086737.1	embryonic stem cell-specific 5-hydroxymethylcytosine-binding protein	2183	0
XM_020086738.1	embryonic stem cell-specific 5-hydroxymethylcytosine-binding protein	2024	0
XM_020086739.1	ras-related protein rab7	2506	2.39E-144
XM_020086740.1	angiogenic factor with G patch and FHA domains 1 isoform X1	4689	0
XM_020086741.1	angiogenic factor with G patch and FHA domains 1 isoform X2	2079	0
XM_020086742.1	protein bicaudal C homolog 1-like	3305	0
XM_020086743.1	transcription factor COE3 isoform X6	4324	0
XM_020086744.1	RNA polymerase II elongation factor ELL2	4657	0
XM_020086745.1	nucleolar protein 56	2025	0
XM_020086746.1	retinol dehydrogenase 12-like	1460	0
XM_020086747.1	protein timeless homolog isoform X1	4155	0
XM_020086748.1	protein timeless homolog isoform X2	4143	0
XM_020086749.1	protein timeless homolog isoform X1	4313	0
XM_020086750.1	peripherin isoform X1	2255	0
XM_020086751.1	peripherin isoform X1	2047	0
XM_020086752.1	CCA tRNA nucleotidyltransferase 1, mitochondrial	1913	0
XM_020086753.1	CCA tRNA nucleotidyltransferase 1, mitochondrial	2019	0
XM_020086754.1	homeobox protein Nkx-6.1	2699	0
XM_020086755.1	PRA1 family protein 3-like	1211	1.93E-134
XM_020086756.1	homeodomain-interacting protein kinase 1-like isoform X1	4661	0
XM_020086757.1	homeodomain-interacting protein kinase 1-like isoform X2	4658	0
XM_020086758.1	homeodomain-interacting protein kinase 1-like isoform X3	4525	0
XM_020086759.1	homeodomain-interacting protein kinase 1-like isoform X4	4522	0
XM_020086760.1	calpastatin isoform X3	4975	0
XM_020086761.1	calpastatin isoform X3	4945	0
XM_020086762.1	calpastatin isoform X1	5023	0
XM_020086763.1	calpastatin isoform X3	4960	0

XM_020086764.1	calpastatin isoform X4	4888	0
XM_020086765.1	calpastatin isoform X3	4934	0
XM_020086766.1	calpastatin isoform X6	4864	0
XM_020086767.1	calpastatin isoform X1	4897	0
XM_020086768.1	cardiolipin synthase (CMP-forming)	1679	0
XM_020086769.1	calpastatin isoform X8	4756	0
XM_020086770.1	calpastatin isoform X9	4750	0
XM_020086771.1	calpastatin isoform X10	4663	0
XM_020086772.1	calpastatin isoform X11	4642	0
XM_020086773.1	calpastatin isoform X12	4555	0
XM_020086774.1	calpastatin isoform X13	4677	0
XM_020086775.1	calpastatin isoform X14	4671	0
XM_020086776.1	calpastatin isoform X15	4456	0
XM_020086777.1	calpastatin isoform X16	4563	0
XM_020086778.1	calpastatin isoform X17	4380	0
XM_020086779.1	calpastatin isoform X17	4256	0
XM_020086780.1	ubiquitin carboxyl-terminal hydrolase 49	4089	0
XM_020086781.1	transmembrane and coiled-coil domains protein 1-like	2566	0
XM_020086782.1	dolichyl-diphosphooligosaccharide--protein glycosyltransferase subunit 2 isoform X1	2458	0
XM_020086783.1	poly(rC)-binding protein 3-like isoform X1	2876	0
XM_020086784.1	dolichyl-diphosphooligosaccharide--protein glycosyltransferase subunit 2 isoform X2	1993	0
XM_020086785.1	myb-related protein B isoform X1	3003	0
XM_020086786.1	myb-related protein B isoform X2	2997	0
XM_020086787.1	nuclear ubiquitous casein and cyclin-dependent kinase substrate 1	1756	1.92E-53
XM_020086788.1	growth/differentiation factor 11-like isoform X1	2137	0
XM_020086789.1	growth/differentiation factor 11-like isoform X2	2134	0
XM_020086790.1	leucine-rich repeat flightless-interacting protein 2-like	3445	0
XM_020086791.1	poly(rC)-binding protein 3-like isoform X1	2917	0
XM_020086792.1	transcription factor E3-like isoform X1	3484	0
XM_020086793.1	transcription factor E3-like isoform X2	3466	0
XM_020086794.1	MAP kinase-activated protein kinase 2-like	3378	0
XM_020086795.1	6-phosphofructo-2-kinase/fructose-2,6-bisphosphatase 4-like isoform X1	2775	0
XM_020086796.1	6-phosphofructo-2-kinase/fructose-2,6-bisphosphatase 4-like isoform X2	2684	0
XM_020086797.1	6-phosphofructo-2-kinase/fructose-2,6-bisphosphatase 4-like isoform X3	3074	0
XM_020086798.1	6-phosphofructo-2-kinase/fructose-2,6-bisphosphatase 4-like isoform X4	2985	0
XM_020086799.1	actin-related protein 2/3 complex subunit 4	1109	7.15E-119
XM_020086800.1	poly(rC)-binding protein 3-like isoform X1	1919	0
XM_020086801.1	AMP deaminase 2-like isoform X1	6722	0
XM_020086802.1	AMP deaminase 2-like isoform X2	2733	0
XM_020086803.1	epidermal growth factor receptor kinase substrate 8-like protein 3 isoform X1	2164	0
XM_020086804.1	epidermal growth factor receptor kinase substrate 8-like protein 3 isoform X2	2161	0
XM_020086805.1	epidermal growth factor receptor kinase substrate 8-like protein 3 isoform X3	1962	0
XM_020086806.1	selenocysteine-specific elongation factor	2989	0
XM_020086807.1	ruvB-like 1	1729	0
XM_020086808.1	musculoskeletal embryonic nuclear protein 1-like	1675	5.70E-46
XM_020086809.1	ubiquitin-associated protein 1 isoform X1	4090	0
XM_020086810.1	ubiquitin-associated protein 1 isoform X1	1468	0
XM_020086811.1	inositol hexakisphosphate kinase 1	2181	0
XM_020086812.1	ubiquitin-associated protein 2 isoform X3	4842	0
XM_020086813.1	cytochrome P450 1B1 isoform X1	4104	0
XM_020086814.1	ubiquitin-associated protein 2 isoform X3	4834	0
XM_020086815.1	ubiquitin-associated protein 2 isoform X3	4578	0
XM_020086816.1	ubiquitin-associated protein 2 isoform X3	4962	0
XM_020086817.1	forkhead box protein P1-B-like isoform X1	7165	0
XM_020086818.1	forkhead box protein P1-B-like isoform X2	7163	0
XM_020086819.1	forkhead box protein P1-B-like isoform X3	7220	0
XM_020086820.1	zinc finger protein Eos isoform X1	6276	0
XM_020086821.1	zinc finger protein Eos isoform X1	6316	0
XM_020086822.1	zinc finger protein Eos isoform X1	6159	0
XM_020086823.1	cytochrome P450 1B1 isoform X1	3890	0
XM_020086824.1	zinc finger protein Eos isoform X1	6144	0
XM_020086825.1	zinc finger protein Eos isoform X4	6027	0
XM_020086826.1	40S ribosomal protein S26	488	1.33E-50
XM_020086827.1	zinc finger CCCH domain-containing protein 10	2353	0
XM_020086828.1	zinc finger CCCH domain-containing protein 10	2456	0
XM_020086829.1	sorting nexin-32-like	1915	0
XM_020086830.1	sorting nexin-32-like	1955	0
XM_020086831.1	NUAK family SNF1-like kinase 1	3987	0
XM_020086832.1	G1/S-specific cyclin-D2-like	1582	1.62E-176
XM_020086833.1	SRSF protein kinase 1-like isoform X1	3420	0
XM_020086834.1	SRSF protein kinase 1-like isoform X2	3398	0
XM_020086835.1	SRSF protein kinase 3-like isoform X3	3295	0
XM_020086836.1	lysyl oxidase homolog 4-like	5115	0
XM_020086837.1	protein transport protein Sec61 subunit alpha-like 1	2557	0
XM_020086838.1	citron Rho-interacting kinase-like isoform X1	8522	0
XM_020086839.1	citron Rho-interacting kinase-like isoform X1	8517	0
XM_020086840.1	citron Rho-interacting kinase-like isoform X1	8361	0
XM_020086841.1	citron Rho-interacting kinase-like isoform X1	6209	0
XM_020086842.1	citron Rho-interacting kinase-like isoform X4	6206	0
XM_020086843.1	PHD finger protein 20-like isoform X3	4527	0

XM_020086844.1	PHD finger protein 20-like isoform X3	4504	0
XM_020086845.1	PHD finger protein 20-like isoform X3	4578	0
XM_020086846.1	PHD finger protein 20-like isoform X3	4345	0
XM_020086847.1	PHD finger protein 20-like isoform X4	4371	0
XM_020086848.1	PHD finger protein 20-like isoform X3	4334	0
XM_020086849.1	carbohydrate sulfotransferase 11-like	2111	0
XM_020086850.1	cartilage acidic protein 1-like	2222	0
XM_020086851.1	ADP-ribosylation factor GTPase-activating protein 1 isoform X1	4906	0
XM_020086852.1	ADP-ribosylation factor GTPase-activating protein 1 isoform X1	4912	0
XM_020086853.1	ADP-ribosylation factor GTPase-activating protein 1 isoform X1	4850	0
XM_020086854.1	activin receptor type-1B-like isoform X1	4657	0
XM_020086855.1	activin receptor type-1B-like isoform X2	4726	0
XM_020086856.1	activin receptor type-1B-like isoform X3	4568	0
XM_020086857.1	dolichyl-diphosphooligosaccharide--protein glycosyltransferase subunit 1	2831	0
XM_020086858.1	band 4.1-like protein 1 isoform X1	7310	0
XM_020086859.1	band 4.1-like protein 1 isoform X1	7307	0
XM_020086860.1	band 4.1-like protein 1 isoform X1	7265	0
XM_020086861.1	band 4.1-like protein 1 isoform X1	7229	0
XM_020086862.1	band 4.1-like protein 1 isoform X1	6950	0
XM_020086863.1	band 4.1-like protein 1 isoform X5	5897	0
XM_020086864.1	band 4.1-like protein 1 isoform X6	5810	0
XM_020086865.1	band 4.1-like protein 1 isoform X7	5765	0
XM_020086866.1	period circadian protein homolog 2-like isoform X1	5854	0
XM_020086867.1	thioredoxin reductase 1, cytoplasmic-like	3154	0
XM_020086868.1	guanine nucleotide-binding protein G(i) subunit alpha-2	2063	0
XM_020086869.1	protein Mdm4 isoform X1	3719	0
XM_020086870.1	probable serine/threonine-protein kinase samkC	1304	4.26E-106
XM_020086871.1	protein Mdm4 isoform X2	3704	0
XM_020086872.1	protein Mdm4 isoform X1	3516	0
XM_020086873.1	DNA topoisomerase 1	3510	0
XM_020086874.1	mitogen-activated protein kinase 14A	3474	0
XM_020086875.1	forkhead box protein I1	2210	0
XM_020086876.1	signal transducer and activator of transcription 1-alpha/beta-like isoform X1	3137	0
XM_020086877.1	signal transducer and activator of transcription 1-alpha/beta-like isoform X1	3132	0
XM_020086878.1	signal transducer and activator of transcription 1-alpha/beta-like isoform X1	2536	0
XM_020086879.1	G-protein coupled receptor 84	1659	0
XM_020086880.1	probable serine/threonine-protein kinase samkC	1137	8.07E-107
XM_020086881.1	WD repeat-containing protein 1	3042	0
XM_020086882.1	transcriptional adapter 3	1702	0
XM_020086883.1	transcriptional adapter 3	1833	0
XM_020086884.1	transcriptional adapter 3	1852	0
XM_020086885.1	actin-related protein 2/3 complex subunit 4-like isoform X1	1276	3.92E-122
XM_020086886.1	actin-related protein 2/3 complex subunit 4-like isoform X2	1245	1.35E-118
XM_020086887.1	long wavelength sensitive opsin	1074	0
XM_020086888.1	SLIT-ROBO Rho GTPase-activating protein 3 isoform X1	7157	0
XM_020086889.1	probable serine/threonine-protein kinase samkC	1090	5.17E-107
XM_020086890.1	SLIT-ROBO Rho GTPase-activating protein 3 isoform X2	7085	0
XM_020086891.1	la-related protein 4-like isoform X1	4523	0
XM_020086892.1	la-related protein 4-like isoform X2	5551	0
XM_020086893.1	la-related protein 4-like isoform X1	5800	0
XM_020086894.1	transcription initiation factor TFIID subunit 4-like	3888	0
XM_020086895.1	transcription initiation factor TFIID subunit 4-like	4324	0
XM_020086896.1	protein LSM14 homolog B-like	1572	0
XM_020086897.1	casein kinase II subunit alpha-like	3008	0
XM_020086898.1	natural resistance-associated macrophage protein 2-like isoform X1	3040	0
XM_020086899.1	natural resistance-associated macrophage protein 2-like isoform X2	1923	0
XM_020086900.1	keratin, type II cytoskeletal 8-like	2167	0
XM_020086901.1	protein phosphatase 1 regulatory subunit 1C	542	8.04E-60
XM_020086902.1	serine/threonine-protein phosphatase 6 regulatory ankyrin repeat subunit C-like	5536	0
XM_020086903.1	calcium/calmodulin-dependent protein kinase type 1-like	4216	0
XM_020086904.1	protein transport protein Sec61 subunit alpha-like	2541	0
XM_020086905.1	semaphorin-3F-like isoform X1	5286	0
XM_020086906.1	semaphorin-3F-like isoform X2	5271	0
XM_020086907.1	semaphorin-3F-like isoform X3	5196	0
XM_020086908.1	semaphorin-3F-like isoform X4	5181	0
XM_020086909.1	isocitrate dehydrogenase [NAD] subunit beta, mitochondrial isoform X1	2280	0
XM_020086910.1	isocitrate dehydrogenase [NAD] subunit beta, mitochondrial isoform X2	1711	0
XM_020086911.1	transcription factor EB isoform X3	4411	0
XM_020086912.1	transcription factor EB isoform X3	4401	0
XM_020086913.1	transcription factor EB isoform X3	4434	0
XM_020086914.1	transcription factor EB isoform X3	4565	0
XM_020086915.1	transcription factor EB isoform X3	4515	0
XM_020086916.1	transcription factor EB isoform X3	4542	0
XM_020086917.1	transcription factor EB isoform X3	4384	0
XM_020086918.1	centrosome and spindle pole-associated protein 1	3716	0
XM_020086919.1	6-phosphofructo-2-kinase/fructose-2,6-bisphosphatase 4-like isoform X1	2339	0
XM_020086920.1	6-phosphofructo-2-kinase/fructose-2,6-bisphosphatase 4-like isoform X2	2400	0
XM_020086921.1	6-phosphofructo-2-kinase/fructose-2,6-bisphosphatase 4-like isoform X3	2114	0
XM_020086922.1	6-phosphofructo-2-kinase/fructose-2,6-bisphosphatase 4-like isoform X4	2174	0

XM_020086923.1	cAMP and cAMP-inhibited cGMP 3',5'-cyclic phosphodiesterase 10A isoform X1	5446	0
XM_020086924.1	leucine-rich repeat neuronal protein 2-like	3457	0
XM_020086925.1	leucine-rich repeat neuronal protein 2-like	3388	0
XM_020086926.1	engulfment and cell motility protein 2 isoform X1	3400	0
XM_020086927.1	engulfment and cell motility protein 2 isoform X1	3468	0
XM_020086928.1	engulfment and cell motility protein 2 isoform X1	3394	0
XM_020086929.1	engulfment and cell motility protein 2 isoform X1	3364	0
XM_020086930.1	engulfment and cell motility protein 2 isoform X4	3358	0
XM_020086931.1	protein NDRG3 isoform X1	2174	0
XM_020086932.1	protein NDRG3 isoform X2	2135	0
XM_020086933.1	cAMP and cAMP-inhibited cGMP 3',5'-cyclic phosphodiesterase 10A isoform X1	5332	0
XM_020086934.1	protein NDRG3 isoform X3	2262	0
XM_020086935.1	RNA-binding motif, single-stranded-interacting protein 2-like isoform X1	6276	0
XM_020086936.1	RNA-binding motif, single-stranded-interacting protein 2-like isoform X1	6273	0
XM_020086937.1	RNA-binding motif, single-stranded-interacting protein 2-like isoform X1	6273	0
XM_020086938.1	PREDICTED: bystin	1676	0
XM_020086939.1	mediator of RNA polymerase II transcription subunit 20 isoform X1	1122	4.33E-176
XM_020086940.1	mediator of RNA polymerase II transcription subunit 20 isoform X2	1060	3.87E-157
XM_020086941.1	serine/arginine-rich splicing factor 3 isoform X1	1277	1.19E-59
XM_020086942.1	cAMP and cAMP-inhibited cGMP 3',5'-cyclic phosphodiesterase 10A isoform X1	5444	0
XM_020086943.1	serine/arginine-rich splicing factor 3 isoform X2	1180	3.20E-90
XM_020086944.1	striatin-interacting protein 1 homolog	3816	0
XM_020086945.1	ubiquitin-conjugating enzyme E2 J2	2787	4.88E-162
XM_020086946.1	E3 ubiquitin-protein ligase TRIM33-like	1519	4.24E-88
XM_020086947.1	prolyl 3-hydroxylase 1 isoform X1	3349	0
XM_020086948.1	prolyl 3-hydroxylase 1 isoform X1	3123	0
XM_020086949.1	prolyl 3-hydroxylase 1 isoform X1	3140	0
XM_020086950.1	prolyl 3-hydroxylase 1 isoform X1	3266	0
XM_020086951.1	TATA element modulatory factor	4506	0
XM_020086952.1	zinc finger and BTB domain-containing protein 49	2811	0
XM_020086953.1	zinc finger and BTB domain-containing protein 49	2882	0
XM_020086954.1	zinc finger and BTB domain-containing protein 49	2824	0
XM_020086955.1	mitochondrial ribosome-associated GTPase 1	1536	0
XM_020086956.1	cell growth-regulating nucleolar protein	1658	0
XM_020086957.1	UPF0258 protein KIAA1024-like	4444	0
XM_020086958.1	UPF0258 protein KIAA1024-like	4765	0
XM_020086959.1	rac GTPase-activating protein 1-like	3491	0
XM_020086960.1	rac GTPase-activating protein 1-like	3486	0
XM_020086961.1	cleavage stimulation factor subunit 1	2666	0
XM_020086962.1	cleavage stimulation factor subunit 1	2721	0
XM_020086963.1	cleavage stimulation factor subunit 1	2663	0
XM_020086964.1	cleavage stimulation factor subunit 1	2768	0
XM_020086965.1	cleavage stimulation factor subunit 1	2582	0
XM_020086966.1	cleavage stimulation factor subunit 1	2765	0
XM_020086967.1	monocarboxylate transporter 2-like	3612	0
XM_020086968.1	shadow of prion protein	1639	1.31E-35
XM_020086969.1	prickle-like protein 2 isoform X1	3553	0
XM_020086970.1	prickle-like protein 2 isoform X1	3731	0
XM_020086971.1	protein kinase C delta type-like	3632	0
XM_020086972.1	RNA-binding protein Musashi homolog 1 isoform X1	1456	0
XM_020086973.1	synaptoporin isoform X1	2197	0
XM_020086974.1	synaptoporin isoform X2	2226	0
XM_020086975.1	interferon-induced GTP-binding protein Mx1	2638	0
XM_020086976.1	THO complex subunit 7 homolog	802	3.10E-140
XM_020086977.1	protein kinase C delta type	3101	0
XM_020086978.1	sodium- and chloride-dependent neutral and basic amino acid transporter B(0+)	2634	0
XM_020086979.1	PREDICTED: fibromodulin-like	1481	0
XM_020086980.1	RNA-binding protein 45	2039	0
XM_020086981.1	syntaxin-18 isoform X1	1453	0
XM_020086982.1	syntaxin-18 isoform X2	1450	0
XM_020086983.1	myb/SANT-like DNA-binding domain-containing protein 4	2307	0
XM_020086984.1	myb/SANT-like DNA-binding domain-containing protein 4	2121	0
XM_020086985.1	neuron-specific protein family member 1	1997	1.55E-118
XM_020086986.1	ras-related protein rab7-like	1503	1.88E-151
XM_020086987.1	neural cell adhesion molecule L1-like protein isoform X1	6119	0
XM_020086988.1	neural cell adhesion molecule L1-like protein isoform X2	6109	0
XM_020086989.1	neural cell adhesion molecule L1-like protein isoform X3	6101	0
XM_020086990.1	neural cell adhesion molecule L1-like protein isoform X4	6091	0
XM_020086991.1	neural cell adhesion molecule L1-like protein isoform X5	6083	0
XM_020086992.1	neural cell adhesion molecule L1-like protein isoform X6	6064	0
XM_020086993.1	ras association domain-containing protein 6-like	926	2.08E-179
XM_020086994.1	neural cell adhesion molecule L1-like protein isoform X7	6016	0
XM_020086995.1	phosphatidate phosphatase LPIN2-like	4524	0
XM_020086996.1	vesicle-associated membrane protein 3	2539	3.11E-38
XM_020086997.1	LIM domain and actin-binding protein 1-like	2532	0
XM_020086998.1	forkhead box protein P4	7051	0
XM_020086999.1	myosin regulatory light polypeptide 9	1395	2.40E-117
XM_020087000.1	disks large-associated protein 4-like isoform X1	7166	0
XM_020087001.1	disks large-associated protein 4-like isoform X1	7034	0

XM_020087002.1	disks large-associated protein 4-like isoform X1	6745	0
XM_020087003.1	disks large-associated protein 4-like isoform X1	7032	0
XM_020087004.1	disks large-associated protein 4-like isoform X1	6613	0
XM_020087005.1	disks large-associated protein 4-like isoform X1	6516	0
XM_020087006.1	glycine receptor subunit alpha-4-like isoform X1	2600	0
XM_020087007.1	transmembrane protein 72	701	3.43E-116
XM_020087008.1	disks large-associated protein 4-like isoform X1	7064	0
XM_020087009.1	disks large-associated protein 4-like isoform X1	6911	0
XM_020087010.1	disks large-associated protein 4-like isoform X4	3769	0
XM_020087011.1	retinoic acid receptor gamma isoform X1	3658	0
XM_020087012.1	retinoic acid receptor gamma isoform X1	3650	0
XM_020087013.1	retinoic acid receptor gamma-A-like isoform X3	3555	0
XM_020087014.1	retinoic acid receptor gamma-A-like isoform X4	3549	0
XM_020087015.1	kelch repeat and BTB domain-containing protein 2-like	3041	0
XM_020087016.1	metabotropic glutamate receptor 6-like	2936	0
XM_020087017.1	E3 ubiquitin-protein ligase TRAIP	2003	0
XM_020087018.1	transmembrane O-methyltransferase-like	1357	0
XM_020087019.1	PREDICTED: aquaporin-7-like	1076	0
XM_020087020.1	tubulin polyglutamylase complex subunit 2 isoform X1	2491	0
XM_020087021.1	tubulin polyglutamylase complex subunit 2 isoform X2	2537	0
XM_020087022.1	SLIT-ROBO Rho GTPase-activating protein 2-like isoform X1	4144	0
XM_020087023.1	SLIT-ROBO Rho GTPase-activating protein 2-like isoform X2	4127	0
XM_020087024.1	SLIT-ROBO Rho GTPase-activating protein 2-like isoform X1	2130	0
XM_020087025.1	AP-5 complex subunit beta-1-like	3245	0
XM_020087026.1	flocculation protein FLO11-like	3652	0
XM_020087027.1	tripartite motif-containing protein 16-like	1928	0
XM_020087028.1	ADP-ribosylation factor 3	2634	2.14E-124
XM_020087029.1	ADP-ribosylation factor 3	2983	2.99E-123
XM_020087030.1	pleckstrin homology domain-containing family G member 4B isoform X4	6894	0
XM_020087031.1	C-X-C motif chemokine 10-like	960	3.66E-73
XM_020087032.1	ras-related protein Rap-1A-like	2536	4.11E-128
XM_020087033.1	FERM domain-containing protein 4B-like	4178	0
XM_020087034.1	glutaredoxin 2 isoform X2	1413	1.72E-68
XM_020087035.1	rho-related BTB domain-containing protein 2-like	2311	0
XM_020087036.1	adenosylhomocysteinase 2-like	5159	0
XM_020087037.1	nuclear receptor subfamily 1 group D member 1-like	3520	0
XM_020087038.1	protein cereblon	3424	0
XM_020087039.1	claudin-19 isoform X1	2105	1.07E-141
XM_020087040.1	claudin-19 isoform X2	2623	1.01E-135
XM_020087041.1	cyclin-dependent kinase 2	2125	0
XM_020087042.1	diacylglycerol kinase alpha-like isoform X2	2941	0
XM_020087043.1	PREDICTED: uncharacterized protein LOC109629342 isoform X1	2266	0
XM_020087044.1	diacylglycerol kinase alpha-like isoform X2	2767	0
XM_020087045.1	syntaxin-16 isoform X1	2721	0
XM_020087046.1	syntaxin-16 isoform X2	2706	0
XM_020087047.1	syntaxin-16 isoform X3	2688	0
XM_020087048.1	syntaxin-16 isoform X4	2676	0
XM_020087049.1	syntaxin-16 isoform X5	2655	0
XM_020087050.1	syntaxin-16 isoform X6	2643	0
XM_020087051.1	syntaxin-16 isoform X6	2212	0
XM_020087052.1	PREDICTED: uncharacterized protein LOC109629342 isoform X1	2225	0
XM_020087053.1	glycosyltransferase 8 domain-containing protein 2 isoform X2	2504	0
XM_020087054.1	signal peptidase complex subunit 1	582	6.85E-66
XM_020087055.1	PREDICTED: uncharacterized protein C1orf50 homolog	1086	5.04E-140
XM_020087056.1	PREDICTED: exportin-2	3633	0
XM_020087057.1	protein Wnt-4 isoform X2	3171	0
XM_020087058.1	protein Wnt-4 isoform X2	2771	0
XM_020087059.1	protein Wnt-4 isoform X2	2573	0
XM_020087060.1	PREDICTED: uncharacterized protein LOC109629342 isoform X1	2219	0
XM_020087061.1	cyclin-dependent kinase 4	2071	0
XM_020087062.1	ADP-ribosylation factor-like protein 8B	2533	1.83E-126
XM_020087063.1	adiponectin receptor protein 1	2893	0
XM_020087064.1	adiponectin receptor protein 1	3037	0
XM_020087065.1	proliferation-associated protein 2G4 isoform X1	2453	0
XM_020087066.1	proliferation-associated protein 2G4 isoform X1	2628	0
XM_020087067.1	extracellular sulfatase Sulf-1-like isoform X1	4570	0
XM_020087068.1	extracellular sulfatase Sulf-1-like isoform X1	4439	0
XM_020087069.1	PREDICTED: uncharacterized protein LOC109629342 isoform X2	2263	0
XM_020087070.1	extracellular sulfatase Sulf-1-like isoform X1	4546	0
XM_020087071.1	class E basic helix-loop-helix protein 40-like	2442	0
XM_020087072.1	CDK5 regulatory subunit-associated protein 1	2224	0
XM_020087073.1	bactericidal permeability-increasing protein-like	2893	0
XM_020087074.1	succinate--CoA ligase [GDP-forming] subunit beta, mitochondrial	2290	0
XM_020087075.1	putative fidgetin-like protein 2	4549	0
XM_020087076.1	collagen alpha-1(VII) chain	12007	0
XM_020087077.1	probable aminopeptidase NPEPL1	1772	0
XM_020087078.1	interferon regulatory factor 6	1983	0
XM_020087079.1	lysine-specific demethylase PHF2 isoform X1	6242	0
XM_020087080.1	zinc finger MIZ domain-containing protein 1-like isoform X1	4205	0

XM_020087081.1	lysine-specific demethylase PHF2 isoform X2	6230	0
XM_020087082.1	lysine-specific demethylase PHF2 isoform X3	6221	0
XM_020087083.1	lysine-specific demethylase PHF2 isoform X4	6237	0
XM_020087084.1	60S acidic ribosomal protein P0 isoform X1	1177	0
XM_020087085.1	60S acidic ribosomal protein P0 isoform X1	1106	0
XM_020087086.1	solute carrier family 41 member 1-like	6338	0
XM_020087087.1	PHD finger protein 13	3474	3.87E-158
XM_020087088.1	protein FAM219A-like isoform X1	4076	2.76E-82
XM_020087089.1	glycine receptor subunit alpha-4-like isoform X2	2485	0
XM_020087090.1	zinc finger MIZ domain-containing protein 1-like isoform X1	3977	0
XM_020087091.1	protein FAM219A-like isoform X2	4054	5.26E-88
XM_020087092.1	zinc fingers and homeoboxes protein 3-like	4693	0
XM_020087093.1	zinc fingers and homeoboxes protein 3-like	4519	0
XM_020087094.1	5'-AMP-activated protein kinase subunit gamma-1 isoform X2	2713	0
XM_020087095.1	5'-AMP-activated protein kinase subunit gamma-1 isoform X2	2805	0
XM_020087096.1	5'-AMP-activated protein kinase subunit gamma-1 isoform X2	2619	0
XM_020087097.1	serine/arginine-rich splicing factor 6-like	1576	1.29E-97
XM_020087098.1	PREDICTED: uncharacterized protein LOC109629376	6283	0
XM_020087099.1	protein-lysine methyltransferase METTL21B	995	2.09E-142
XM_020087100.1	vitamin D3 receptor B-like	2487	0
XM_020087101.1	collagen alpha-1(XIX) chain-like isoform X1	5006	4.71E-59
XM_020087102.1	translocon-associated protein subunit alpha-like	1765	8.59E-146
XM_020087103.1	signaling threshold-regulating transmembrane adapter 1-like	1196	5.41E-125
XM_020087104.1	IQ motif and SEC7 domain-containing protein 1-like	7487	0
XM_020087105.1	histone deacetylase 7-like isoform X2	3586	0
XM_020087106.1	histone deacetylase 7-like isoform X2	3684	0
XM_020087107.1	PREDICTED: limbin	4650	0
XM_020087108.1	E3 ubiquitin-protein ligase DTX3L-like	2307	0
XM_020087109.1	collagen alpha-1(XIX) chain-like isoform X2	2325	2.81E-55
XM_020087110.1	low-density lipoprotein receptor-related protein 1-like	2065	9.04E-62
XM_020087111.1	transmembrane protein 183B-like	2285	0
XM_020087112.1	cell death activator CIDE-3 isoform X1	1809	1.25E-161
XM_020087113.1	cell death activator CIDE-3 isoform X1	1662	1.20E-151
XM_020087114.1	transmembrane protein 198-like isoform X1	2585	0
XM_020087115.1	transmembrane protein 198-like isoform X2	2526	0
XM_020087116.1	transmembrane protein 198-like isoform X3	2514	0
XM_020087117.1	zinc finger protein with KRAB and SCAN domains 1-like isoform X1	2815	0
XM_020087118.1	zinc finger protein with KRAB and SCAN domains 1-like isoform X1	2620	0
XM_020087119.1	electroneutral sodium bicarbonate exchanger 1-like	7094	0
XM_020087120.1	keratin, type II cytoskeletal 8-like	2337	0
XM_020087121.1	bridging integrator 2-like	2499	0
XM_020087122.1	cadherin-8-like isoform X1	5501	0
XM_020087123.1	cytochrome b-c1 complex subunit 1, mitochondrial	1997	0
XM_020087124.1	transmembrane protein adipocyte-associated 1	3284	0
XM_020087125.1	receptor-type tyrosine-protein phosphatase epsilon-like isoform X1	3091	0
XM_020087126.1	ethanolamine kinase 2	3783	0
XM_020087127.1	transcription factor MafB-like	1881	0
XM_020087128.1	eukaryotic translation initiation factor 4E type 3-like	2073	1.46E-146
XM_020087129.1	methyltransferase-like protein 7A	2272	2.20E-161
XM_020087130.1	tensin-2-like isoform X1	5766	0
XM_020087131.1	tensin-2-like isoform X2	5736	0
XM_020087132.1	tensin-2-like isoform X1	5159	0
XM_020087133.1	zinc finger protein 511	1789	0
XM_020087134.1	calmodulin-binding transcription activator 1-like	9352	0
XM_020087135.1	uncharacterized family 31 glucosidase KIAA1161 homolog	4539	0
XM_020087136.1	uncharacterized family 31 glucosidase KIAA1161 homolog	4505	0
XM_020087137.1	macrophage-stimulating protein receptor	5141	0
XM_020087138.1	caM kinase-like vesicle-associated protein	2498	0
XM_020087139.1	ephrin type-A receptor 8 isoform X1	4546	0
XM_020087140.1	ephrin type-A receptor 8 isoform X2	4540	0
XM_020087141.1	FYVE, RhoGEF and PH domain-containing protein 5-like isoform X1	6162	0
XM_020087142.1	FYVE, RhoGEF and PH domain-containing protein 5-like isoform X2	6135	0
XM_020087143.1	acyl-CoA dehydrogenase family member 9, mitochondrial	2174	0
XM_020087144.1	testis-expressed sequence 264 protein	2503	6.50E-139
XM_020087145.1	homeobox protein MSX-2-like	1056	1.43E-163
XM_020087146.1	EGF domain-specific O-linked N-acetylglucosamine transferase	2987	0
XM_020087147.1	protein FAM19A4-like	1640	3.89E-92
XM_020087148.1	protein FAM19A4-like	1640	1.34E-92
XM_020087149.1	beta-1,4 N-acetylgalactosaminyltransferase 1 isoform X1	3560	0
XM_020087150.1	beta-1,4 N-acetylgalactosaminyltransferase 1 isoform X1	3425	0
XM_020087151.1	beta-1,4 N-acetylgalactosaminyltransferase 1 isoform X1	3391	0
XM_020087152.1	beta-1,4 N-acetylgalactosaminyltransferase 1 isoform X1	3338	0
XM_020087153.1	beta-1,4 N-acetylgalactosaminyltransferase 1 isoform X1	3557	0
XM_020087154.1	beta-1,4 N-acetylgalactosaminyltransferase 1 isoform X1	3383	0
XM_020087155.1	beta-1,4 N-acetylgalactosaminyltransferase 1 isoform X1	2962	0
XM_020087156.1	vacuolar fusion protein MON1 homolog A	1904	0
XM_020087157.1	splicing factor 3B subunit 5	890	4.49E-60
XM_020087158.1	leucine-rich repeat neuronal protein 1-like	3289	0
XM_020087159.1	urocanate hydratase	2473	0

XM_020087160.1	epithelial cell adhesion molecule	1576	0
XM_020087161.1	protein phosphatase 1 regulatory inhibitor subunit 16B	4418	0
XM_020087162.1	adenosine 3'-phospho 5'-phosphosulfate transporter 2	2251	0
XM_020087163.1	guanine nucleotide exchange factor VAV3	6301	0
XM_020087164.1	leucine-rich repeat-containing protein 1	2414	0
XM_020087165.1	NADH dehydrogenase [ubiquinone] 1 beta subcomplex subunit 4	745	6.98E-98
XM_020087166.1	sperm-associated antigen 16 protein-like	724	1.73E-70
XM_020087167.1	transmembrane protein 108	4294	0
XM_020087168.1	transmembrane protein 108	3955	0
XM_020087169.1	vasoactive intestinal polypeptide receptor 1 isoform X1	3878	0
XM_020087170.1	vasoactive intestinal polypeptide receptor 1 isoform X1	3806	0
XM_020087171.1	ubiquitin-conjugating enzyme E2 W isoform X1	1734	3.00E-112
XM_020087172.1	ubiquitin-conjugating enzyme E2 W isoform X2	1570	2.52E-108
XM_020087173.1	intraflagellar transport protein 74 homolog	2156	0
XM_020087174.1	GRB2-associated and regulator of MAPK protein 1	5914	0
XM_020087175.1	F-box only protein 5	1964	0
XM_020087176.1	kinesin-1 heavy chain isoform X1	3869	0
XM_020087177.1	kinesin-1 heavy chain isoform X2	3854	0
XM_020087178.1	retinol dehydrogenase 11-like	1193	0
XM_020087179.1	retinol dehydrogenase 12-like	2072	0
XM_020087180.1	telomeric repeat-binding factor 1	1546	0
XM_020087181.1	somatomedin-B and thrombospondin type-1 domain-containing protein isoform X1	1533	0
XM_020087182.1	somatomedin-B and thrombospondin type-1 domain-containing protein isoform X2	1526	0
XM_020087183.1	trichohyalin-like isoform X1	4610	0
XM_020087184.1	trichohyalin-like isoform X2	4607	0
XM_020087185.1	trichohyalin-like isoform X2	4018	0
XM_020087186.1	forkhead box protein H1-like	3004	0
XM_020087187.1	forkhead box protein H1-like	3119	0
XM_020087188.1	probable lysosomal cobalamin transporter	3060	0
XM_020087189.1	FAST kinase domain-containing protein 1, mitochondrial	2266	0
XM_020087190.1	beta-1,4-galactosyltransferase 2 isoform X1	2330	0
XM_020087191.1	ADP-ribosyl cyclase/cyclic ADP-ribose hydrolase 1-like	2019	0
XM_020087192.1	ADP-ribosyl cyclase/cyclic ADP-ribose hydrolase 1-like	1670	0
XM_020087193.1	protocadherin-11 X-linked-like	3398	0
XM_020087194.1	TNFAIP3-interacting protein 1 isoform X1	1573	0
XM_020087195.1	ADP-ribosyl cyclase/cyclic ADP-ribose hydrolase 1-like	915	0
XM_020087196.1	glucose-fructose oxidoreductase domain-containing protein 1	4669	0
XM_020087197.1	COP9 signalosome complex subunit 5	1326	0
XM_020087198.1	extended synaptotagmin-2 isoform X1	3856	0
XM_020087199.1	extended synaptotagmin-2 isoform X2	3853	0
XM_020087200.1	type I inositol 1,4,5-trisphosphate 5-phosphatase isoform X1	3444	0
XM_020087201.1	extended synaptotagmin-2 isoform X3	3804	0
XM_020087202.1	extended synaptotagmin-2 isoform X4	3744	0
XM_020087203.1	extended synaptotagmin-2 isoform X5	3741	0
XM_020087204.1	sonic hedgehog protein	2597	0
XM_020087205.1	receptor activity-modifying protein 1-like	3534	2.59E-132
XM_020087206.1	class E basic helix-loop-helix protein 22	1943	1.57E-155
XM_020087207.1	type I inositol 1,4,5-trisphosphate 5-phosphatase isoform X2	3495	0
XM_020087208.1	pituitary adenylate cyclase-activating polypeptide isoform X1	1734	1.39E-163
XM_020087209.1	pituitary adenylate cyclase-activating polypeptide isoform X2	1085	3.50E-132
XM_020087210.1	pituitary adenylate cyclase-activating polypeptide isoform X3	1111	2.45E-147
XM_020087211.1	pituitary adenylate cyclase-activating polypeptide isoform X4	1629	5.02E-135
XM_020087212.1	pituitary adenylate cyclase-activating polypeptide isoform X5	2146	8.41E-92
XM_020087213.1	DNA endonuclease RBBP8 isoform X1	2407	0
XM_020087214.1	DNA endonuclease RBBP8 isoform X2	2382	0
XM_020087215.1	DNA endonuclease RBBP8 isoform X3	2369	0
XM_020087216.1	MCM domain-containing protein 2	2166	0
XM_020087217.1	transcription factor 24	1234	2.58E-74
XM_020087218.1	WD repeat-containing protein 60	2343	0
XM_020087219.1	5-hydroxytryptamine receptor 1D-like isoform X1	1904	0
XM_020087220.1	PREDICTED: neuroguidin	1159	0
XM_020087221.1	CCAAT/enhancer-binding protein gamma-like	845	9.80E-128
XM_020087222.1	PREDICTED: thiamine-triphosphatase	1910	3.25E-157
XM_020087223.1	PREDICTED: thiamine-triphosphatase	1618	1.27E-158
XM_020087224.1	PREDICTED: thiamine-triphosphatase	1474	2.39E-159
XM_020087225.1	PREDICTED: thiamine-triphosphatase	1396	9.46E-160
XM_020087226.1	amiloride-sensitive amine oxidase [copper-containing]	3286	0
XM_020087227.1	cystatin B	637	2.45E-69
XM_020087228.1	5-hydroxytryptamine receptor 1D-like isoform X1	1680	0
XM_020087229.1	potassium voltage-gated channel subfamily B member 2	6427	0
XM_020087230.1	potassium voltage-gated channel subfamily B member 2	6422	0
XM_020087231.1	APC membrane recruitment protein 2	4748	0
XM_020087232.1	homeobox protein DBX1-like	1839	0
XM_020087233.1	homeobox protein Mohawk	3961	0
XM_020087234.1	kinesin-like protein KIF20A isoform X1	2755	0
XM_020087235.1	kinesin-like protein KIF20A isoform X1	2324	0
XM_020087236.1	kinesin-like protein KIF20A isoform X3	2404	0
XM_020087237.1	gap junction delta-4 protein	4231	0
XM_020087238.1	GPI mannosyltransferase 1 isoform X1	2513	0

XM_020087239.1	GPI mannosyltransferase 1 isoform X2	2422	0
XM_020087240.1	putative thiamine transporter SLC35F3	1415	0
XM_020087241.1	leucine-rich repeat-containing protein 32-like isoform X2	3113	0
XM_020087242.1	rab effector MyRIP-like isoform X2	3165	0
XM_020087243.1	rab effector MyRIP-like isoform X2	3229	0
XM_020087244.1	armadillo repeat-containing protein 4 isoform X1	4628	0
XM_020087245.1	armadillo repeat-containing protein 4 isoform X1	4746	0
XM_020087246.1	armadillo repeat-containing protein 4 isoform X1	3730	0
XM_020087247.1	docking protein 6 isoform X1	3425	0
XM_020087248.1	docking protein 6 isoform X2	3396	0
XM_020087249.1	homogentisate 1,2-dioxygenase	1818	0
XM_020087250.1	G-protein coupled receptor 12-like	4891	4.43E-160
XM_020087251.1	transcription factor Sox-17-alpha-like	1722	0
XM_020087252.1	mitogen-activated protein kinase kinase kinase 8	2208	0
XM_020087253.1	solute carrier family 35 member G2	2100	0
XM_020087254.1	double-stranded RNA-specific editase B2	4774	0
XM_020087255.1	zinc transporter ZIP12	4613	0

XM_020087256.1	PREDICTED: cerebellin-2	2295	6.82E-143
XM_020087257.1	protein kinase C and casein kinase substrate in neurons protein 1-like	1883	0
XM_020087258.1	crystallin J1B-like	2571	0
XM_020087259.1	tumor necrosis factor receptor superfamily member 11A	2637	0
XM_020087260.1	zinc finger CCHC domain-containing protein 2-like	2177	5.24E-163
XM_020087261.1	microtubule-associated protein 4-like	5532	1.51E-133
XM_020087262.1	PREDICTED: SCO-spondin-like	3207	0
XM_020087263.1	neuropilin-2 isoform X3	6328	0
XM_020087264.1	copine-3-like isoform X2	1944	0
XM_020087265.1	agouti-signaling protein-like	686	6.74E-53
XM_020087266.1	RNA-binding protein 40	1534	0
XM_020087267.1	28S ribosomal protein S5, mitochondrial	1625	0
XM_020087268.1	protein FAN	3169	0
XM_020087269.1	cyclic nucleotide-gated cation channel beta-3-like	2063	0
XM_020087270.1	cyclic nucleotide-gated cation channel beta-3-like	2543	0
XM_020087271.1	matrix metalloproteinase-16-like	3297	0
XM_020087272.1	PREDICTED: LOW QUALITY PROTEIN: uncharacterized protein C11orf65-like	822	0
XM_020087273.1	PREDICTED: uncharacterized protein LOC109629529	1148	5.39E-166
XM_020087274.1	PREDICTED: uncharacterized protein LOC109629530	3691	0
XM_020087275.1	zinc finger C2HC domain-containing protein 1A	1851	1.15E-120
XM_020087276.1	ropporin-1-like protein	793	9.97E-142
XM_020087277.1	GPI ethanolamine phosphate transferase 1	3166	0
XM_020087278.1	nuclear nucleic acid-binding protein C1D	1254	1.72E-88
XM_020087279.1	protein AF-10	3317	0
XM_020087280.1	polycystic kidney disease protein 1-like 2	7458	0
XM_020087281.1	DNA polymerase alpha catalytic subunit	4479	0
XM_020087282.1	F-box only protein 15	1530	0
XM_020087283.1	PREDICTED: rotatin	7248	0
XM_020087284.1	PREDICTED: junctophilin-1	3400	9.40E-138
XM_020087285.1	vicilin-like seed storage protein At2g18540	826	1.38E-142
XM_020087286.1	transmembrane protease serine 7	7184	0
XM_020087287.1	phospholipid-transporting ATPase IB-like	3362	0
XM_020087288.1	cell adhesion molecule-related/down-regulated by oncogenes-like	3702	0
XM_020087289.1	inhibitor of nuclear factor kappa-B kinase subunit alpha-like	3418	0
XM_020087290.1	coiled-coil domain-containing protein 191	2569	0
XM_020087291.1	thiosulfate sulfurtransferase/rhodanese-like domain-containing protein 1	358	8.48E-85
XM_020087292.1	PREDICTED: uncharacterized protein LOC109629548	945	0
XM_020087293.1	thrombospondin type-1 domain-containing protein 7A-like	2725	0
XM_020087294.1	CTD small phosphatase-like protein	1836	0
XM_020087295.1	deleted in lung and esophageal cancer protein 1	3909	0
XM_020087296.1	inhibitor of nuclear factor kappa-B kinase subunit alpha-like	3439	0
XM_020087297.1	mitogen-activated protein kinase kinase kinase 3-like	1818	0
XM_020087298.1	solute carrier family 22 member 13-like	3973	0
XM_020087299.1	PREDICTED: uncharacterized protein LOC109629554	1688	0
XM_020087300.1	RNA-binding protein 25-like	1286	1.51E-45
XM_020087301.1	receptor-type tyrosine-protein phosphatase N2-like	1939	0
XM_020087302.1	insulin-induced gene 1 protein	1099	0
XM_020087303.1	GDNF family receptor alpha-4-like	2813	0
XM_020087304.1	inhibitor of nuclear factor kappa-B kinase subunit alpha-like	3391	0
XM_020087305.1	disks large-associated protein 1-like	2699	0
XM_020087306.1	macrophage mannose receptor 1-like	4221	0
XM_020087307.1	structural maintenance of chromosomes flexible hinge domain-containing protein 1	5799	0
XM_020087308.1	oxygen-regulated protein 1	4743	0
XM_020087309.1	anion exchange protein 2-like	2758	5.23E-146
XM_020087310.1	protein scribble homolog	3062	0
XM_020087311.1	transcriptional activator GLI3-like	950	5.10E-99
XM_020087312.1	transcriptional activator GLI3-like	5360	0
XM_020087313.1	PREDICTED: cadherin-18-like	1117	0
XM_020087314.1	cadherin-8-like isoform X2	2317	0
XM_020087315.1	PREDICTED: uncharacterized protein LOC109629565 isoform X1	4866	0
XM_020087316.1	F-box/LRR-repeat protein 7	2361	0
XM_020087317.1	charged multivesicular body protein 5-like	968	8.03E-142

XM_020087318.1	coiled-coil domain-containing protein 58 isoform X1	749	3.24E-107
XM_020087319.1	coiled-coil domain-containing protein 58 isoform X2	1300	8.86E-101
XM_020087320.1	coiled-coil domain-containing protein 58 isoform X3	454	2.14E-89
XM_020087321.1	cryptochrome DASH-like	2068	0
XM_020087322.1	flap endonuclease 1	1114	0
XM_020087323.1	transcription elongation factor B polypeptide 1	782	2.54E-79
XM_020087324.1	PREDICTED: uncharacterized protein LOC109629565 isoform X2	4845	0
XM_020087325.1	39S ribosomal protein L15, mitochondrial	1134	0
XM_020087326.1	D(3) dopamine receptor	2429	0
XM_020087327.1	organic solute transporter subunit alpha-like	1288	0
XM_020087328.1	proteasome maturation protein	840	9.25E-90
XM_020087329.1	proteasome maturation protein	864	1.23E-89
XM_020087330.1	charged multivesicular body protein 4b-like	1346	2.41E-113
XM_020087331.1	peroxisome biogenesis factor 2 isoform X1	1956	5.32E-173
XM_020087332.1	PREDICTED: uncharacterized protein LOC109629565 isoform X3	4594	0
XM_020087333.1	peroxisome biogenesis factor 2 isoform X2	1952	4.01E-172
XM_020087334.1	putative methyltransferase NSUN6 isoform X1	2419	0
XM_020087335.1	putative methyltransferase NSUN6 isoform X1	2379	0
XM_020087336.1	putative methyltransferase NSUN6 isoform X1	2415	0
XM_020087337.1	ubiquitin carboxyl-terminal hydrolase 22-like	1570	0

XM_020087338.1	yae1 domain-containing protein 1	945	1.58E-150
XM_020087339.1	proteasome activator complex subunit 1	878	5.08E-137
XM_020087340.1	PREDICTED: uncharacterized protein LOC109629565 isoform X3	4506	0
XM_020087341.1	carboxypeptidase A6 isoform X1	2571	0
XM_020087342.1	carboxypeptidase A6 isoform X2	2569	0
XM_020087343.1	type-1 angiotensin II receptor	1964	0
XM_020087344.1	noelin-3 isoform X2	1882	0
XM_020087345.1	mitochondrial import inner membrane translocase subunit Tim21	1184	9.49E-148
XM_020087346.1	cysteine-rich secretory protein LCCL domain-containing 1 isoform X1	2836	0
XM_020087347.1	cysteine-rich secretory protein LCCL domain-containing 1 isoform X2	2812	0
XM_020087348.1	delta-type opioid receptor-like isoform X1	1191	0
XM_020087349.1	delta-type opioid receptor-like isoform X1	978	0
XM_020087350.1	delta-type opioid receptor-like isoform X1	963	0
XM_020087351.1	uracil nucleotide/cysteinyl leukotriene receptor-like	1453	0
XM_020087352.1	ribosomal protein 63, mitochondrial	1323	1.12E-68
XM_020087353.1	inositol monophosphatase 1-like	1244	0
XM_020087354.1	armadillo repeat-containing protein 3	2550	0
XM_020087355.1	transient receptor potential cation channel subfamily A member 1	4249	0
XM_020087356.1	AN1-type zinc finger protein 1	940	1.47E-173
XM_020087357.1	PR domain zinc finger protein 14	2623	0
XM_020087358.1	potassium voltage-gated channel subfamily H member 2-like isoform X2	2075	0
XM_020087359.1	potassium voltage-gated channel subfamily H member 2-like isoform X2	1439	0
XM_020087360.1	potassium voltage-gated channel subfamily H member 2-like isoform X2	1394	0
XM_020087361.1	nephrocystin-3 isoform X1	5083	0
XM_020087362.1	nephrocystin-3 isoform X2	5080	0
XM_020087363.1	U2 snRNP-associated SURP motif-containing protein isoform X1	3354	0
XM_020087364.1	enoyl-CoA hydratase, mitochondrial	1394	0
XM_020087365.1	U2 snRNP-associated SURP motif-containing protein isoform X2	3318	0
XM_020087366.1	U2 snRNP-associated SURP motif-containing protein isoform X3	2560	0
XM_020087367.1	short transient receptor potential channel 1	3018	0
XM_020087368.1	acyl-CoA dehydrogenase family member 11	2768	0
XM_020087369.1	carbohydrate sulfotransferase 6-like	2870	0
XM_020087370.1	procollagen C-endopeptidase enhancer 2	2058	0
XM_020087371.1	procollagen C-endopeptidase enhancer 2	2894	0
XM_020087372.1	ubiquitin-like modifier-activating enzyme 6	1792	0
XM_020087373.1	C-C chemokine receptor type 9-like	2560	0
XM_020087374.1	CD226 antigen isoform X1	1344	0
XM_020087375.1	CD226 antigen isoform X1	1806	1.97E-167
XM_020087376.1	protein-cysteine N-palmitoyltransferase HHAT	2727	0
XM_020087377.1	neuropilin and tolloid-like protein 1 isoform X1	2298	0
XM_020087378.1	neuropilin and tolloid-like protein 1 isoform X1	2333	0
XM_020087379.1	neuropilin and tolloid-like protein 1 isoform X1	2223	0
XM_020087380.1	complement C1q and tumor necrosis factor-related protein 9A-like	1798	2.87E-171
XM_020087381.1	transmembrane protein 14C-like	465	1.19E-57
XM_020087382.1	PREDICTED: uncharacterized protein C8orf34 homolog	2620	0
XM_020087383.1	transmembrane protein 241	1036	0
XM_020087384.1	protein phosphatase 1 regulatory subunit 42	1464	0
XM_020087385.1	protein phosphatase 1 regulatory subunit 42	1505	0
XM_020087386.1	protein phosphatase 1 regulatory subunit 42	1488	0
XM_020087387.1	cilia- and flagella-associated protein 36 isoform X1	2390	0
XM_020087388.1	serine/threonine-protein kinase MAK	2313	0
XM_020087389.1	aryl hydrocarbon receptor-like	2229	0
XM_020087390.1	C-C chemokine receptor type 9-like	1371	0
XM_020087391.1	transcription factor Sox-17-alpha-like	1142	0
XM_020087392.1	meiotic recombination protein REC8 homolog	2050	0
XM_020087393.1	protein phosphatase 1 regulatory subunit 17	674	4.47E-64
XM_020087394.1	cilia- and flagella-associated protein 36 isoform X2	2350	0
XM_020087395.1	XK-related protein 9	1617	0
XM_020087396.1	ankyrin repeat domain-containing protein 33B-like	2499	0

XM_020087397.1	cAMP-responsive element modulator	1354	2.80E-44
XM_020087398.1	jouberin isoform X2	1721	3.68E-113
XM_020087399.1	cilia- and flagella-associated protein 36 isoform X3	2294	0
XM_020087400.1	methionine-R-sulfoxide reductase B2, mitochondrial	1101	4.45E-126
XM_020087401.1	mitochondrial calcium uniporter regulator 1-like isoform X1	848	1.28E-159
XM_020087402.1	mitochondrial calcium uniporter regulator 1-like isoform X2	818	5.25E-152
XM_020087403.1	mitochondrial calcium uniporter regulator 1-like isoform X3	731	2.75E-128
XM_020087404.1	cystatin B	360	1.32E-69
XM_020087405.1	nanos homolog 1-like	744	3.58E-106
XM_020087406.1	PREDICTED: uncharacterized protein LOC109629637	1080	0
XM_020087407.1	PI-PLC X domain-containing protein 1-like isoform X1	1108	0
XM_020087408.1	homeodomain-interacting protein kinase 1-like isoform X1	1835	0
XM_020087409.1	homeodomain-interacting protein kinase 1-like isoform X2	1823	0
XM_020087410.1	adhesion G protein-coupled receptor A1	7445	0
XM_020087411.1	regulator of G-protein signaling 9-binding protein B-like	1195	1.58E-163
XM_020087412.1	transcription factor 21	1079	4.56E-92
XM_020087413.1	5-hydroxytryptamine receptor 5A	1483	0
XM_020087414.1	cyclin-dependent kinase 13	5923	0
XM_020087415.1	mediator of RNA polymerase II transcription subunit 1-like isoform X1	2518	0
XM_020087416.1	mediator of RNA polymerase II transcription subunit 1-like isoform X2	2489	0
XM_020087417.1	smoothelin-like protein 2 isoform X1	3151	0
XM_020087418.1	mediator of RNA polymerase II transcription subunit 1-like isoform X1	2558	0
XM_020087419.1	mediator of RNA polymerase II transcription subunit 1-like isoform X4	1962	0

XM_020087420.1	ras-related protein Ral-A	4294	4.56E-121
XM_020087421.1	desmin-like isoform X2	2207	0
XM_020087422.1	pleckstrin homology-like domain family B member 2 isoform X1	6200	0
XM_020087423.1	pleckstrin homology-like domain family B member 1 isoform X2	6071	0
XM_020087424.1	pleckstrin homology-like domain family B member 2 isoform X3	6053	0
XM_020087425.1	pleckstrin homology-like domain family B member 2 isoform X1	5764	0
XM_020087426.1	beta,beta-carotene 15,15'-dioxygenase-like	2145	0
XM_020087427.1	transmembrane protein 56-B-like	4593	5.76E-172
XM_020087428.1	cAMP-dependent protein kinase inhibitor alpha-like	939	1.16E-45
XM_020087429.1	bone morphogenetic protein 6	4813	0
XM_020087430.1	thioredoxin domain-containing protein 5	3477	0
XM_020087431.1	eukaryotic translation elongation factor 1 epsilon-1	2239	2.50E-106
XM_020087432.1	neuroendocrine convertase 2	2841	0
XM_020087433.1	biogenesis of lysosome-related organelles complex 1 subunit 5	2290	8.23E-104
XM_020087434.1	contactin-associated protein-like 2 isoform X1	6353	0
XM_020087435.1	contactin-associated protein-like 2 isoform X2	6323	0
XM_020087436.1	thiamin pyrophosphokinase 1	1690	0
XM_020087437.1	thiamin pyrophosphokinase 1	1631	0
XM_020087438.1	PREDICTED: lipocalin-like	991	9.68E-171
XM_020087439.1	complement component C8 gamma chain	1042	3.62E-152
XM_020087440.1	PREDICTED: lipocalin-like	1344	2.28E-149
XM_020087441.1	protein transport protein Sec61 subunit gamma	500	5.92E-30
XM_020087442.1	D-beta-hydroxybutyrate dehydrogenase, mitochondrial-like	1955	0
XM_020087443.1	MAD2L1-binding protein	2509	0
XM_020087444.1	cytoplasmic protein NCK1 isoform X1	5201	0
XM_020087445.1	cytoplasmic protein NCK1 isoform X2	4699	0
XM_020087446.1	glycogenin-1-like isoform X1	1815	0
XM_020087447.1	glycogenin-1-like isoform X2	1776	0
XM_020087448.1	carboxypeptidase B	1459	0
XM_020087449.1	copine-3-like isoform X1	3095	0
XM_020087450.1	copine-3-like isoform X2	1991	0
XM_020087451.1	WD repeat-containing protein 37-like	4228	0
XM_020087452.1	WD repeat-containing protein 37-like	4225	0
XM_020087453.1	WD repeat-containing protein 37-like	4098	0
XM_020087454.1	isopentenyl-diphosphate Delta-isomerase 1 isoform X2	1483	0
XM_020087455.1	isopentenyl-diphosphate Delta-isomerase 1 isoform X2	1501	0
XM_020087456.1	isopentenyl-diphosphate Delta-isomerase 1 isoform X2	1505	0
XM_020087457.1	ATP synthase-coupling factor 6, mitochondrial isoform X2	2884	0
XM_020087458.1	isopentenyl-diphosphate Delta-isomerase 1 isoform X2	1483	0
XM_020087459.1	isopentenyl-diphosphate Delta-isomerase 1 isoform X2	1338	3.35E-178
XM_020087460.1	isopentenyl-diphosphate Delta-isomerase 1 isoform X2	1326	6.63E-180
XM_020087461.1	isopentenyl-diphosphate Delta-isomerase 1 isoform X2	1282	2.30E-180
XM_020087462.1	YTH domain-containing family protein 3 isoform X1	3369	1.24E-148
XM_020087463.1	YTH domain-containing family protein 3 isoform X2	2963	4.03E-146
XM_020087464.1	mycophenolic acid acyl-glucuronide esterase, mitochondrial	1397	0
XM_020087465.1	transgelin isoform X1	1866	1.31E-143
XM_020087466.1	transgelin isoform X2	1866	9.45E-144
XM_020087467.1	transgelin isoform X2	1646	1.38E-144
XM_020087468.1	sickle tail protein homolog isoform X1	7470	0
XM_020087469.1	sickle tail protein homolog isoform X2	7467	0
XM_020087470.1	sickle tail protein homolog isoform X3	7364	0
XM_020087471.1	lactosylceramide alpha-2,3-sialyltransferase-like isoform X1	1564	0
XM_020087472.1	sickle tail protein homolog isoform X4	7344	0
XM_020087473.1	sickle tail protein homolog isoform X5	7332	0
XM_020087474.1	sickle tail protein homolog isoform X6	7093	0
XM_020087475.1	sickle tail protein homolog isoform X6	6861	0

XM_020087476.1	sickle tail protein homolog isoform X1	7101	0
XM_020087477.1	sickle tail protein homolog isoform X6	6643	0
XM_020087478.1	sickle tail protein homolog isoform X10	5918	0
XM_020087479.1	sickle tail protein homolog isoform X11	5734	0
XM_020087480.1	sickle tail protein homolog isoform X12	5612	0
XM_020087481.1	lactosylceramide alpha-2,3-sialyltransferase-like isoform X1	1621	0
XM_020087482.1	OTU domain-containing protein 1	2227	0
XM_020087483.1	nucleolar MIF4G domain-containing protein 1	2860	0
XM_020087484.1	limb region 1 protein homolog	4631	0
XM_020087485.1	RING finger protein 32	1292	0
XM_020087486.1	RING finger protein 32	2989	0
XM_020087487.1	motor neuron and pancreas homeobox protein 1	3551	0
XM_020087488.1	arf-GAP with SH3 domain, ANK repeat and PH domain-containing protein 1 isoform X1	5269	0
XM_020087489.1	arf-GAP with SH3 domain, ANK repeat and PH domain-containing protein 1 isoform X2	5260	0
XM_020087490.1	arf-GAP with SH3 domain, ANK repeat and PH domain-containing protein 1 isoform X3	5176	0
XM_020087491.1	arf-GAP with SH3 domain, ANK repeat and PH domain-containing protein 1 isoform X4	5107	0
XM_020087492.1	arf-GAP with SH3 domain, ANK repeat and PH domain-containing protein 1 isoform X5	5098	0
XM_020087493.1	elongation factor 1-alpha 1	1925	0
XM_020087494.1	40S ribosomal protein S24	569	7.37E-85
XM_020087495.1	myosin heavy chain, fast skeletal muscle	6779	0
XM_020087496.1	repressor of RNA polymerase III transcription MAF1 homolog	2400	3.32E-150
XM_020087497.1	ADP-ribosylation factor-like protein 5B	2596	3.12E-125
XM_020087498.1	zinc finger homeobox protein 3	14194	0
XM_020087499.1	partitioning defective 3 homolog	6854	0
XM_020087500.1	serine/threonine-protein kinase OSR1-like isoform X1	5456	0
XM_020087501.1	serine/threonine-protein kinase OSR1-like isoform X1	5451	0
XM_020087502.1	myeloid differentiation primary response protein MyD88	1636	0
XM_020087503.1	protein disulfide-isomerase A4	2410	0
XM_020087504.1	alanine aminotransferase 2-like	2399	0
XM_020087505.1	monocarboxylate transporter 12-B-like	3794	0
XM_020087506.1	solute carrier organic anion transporter family member 5A1	6454	0
XM_020087507.1	solute carrier organic anion transporter family member 5A1	6401	0
XM_020087508.1	extracellular sulfatase Sulf-1-like isoform X1	2078	1.24E-163
XM_020087509.1	leucine zipper transcription factor-like protein 1	2099	0
XM_020087510.1	dnaJ homolog subfamily C member 16	7607	0
XM_020087511.1	spermatogenesis-associated protein 13 isoform X1	4558	0
XM_020087512.1	spermatogenesis-associated protein 13 isoform X2	2641	0
XM_020087513.1	spermatogenesis-associated protein 13 isoform X1	1923	0
XM_020087514.1	spermatogenesis-associated protein 13 isoform X1	1711	0
XM_020087515.1	spermatogenesis-associated protein 13 isoform X1	1658	0
XM_020087516.1	spermatogenesis-associated protein 13 isoform X1	1661	0
XM_020087517.1	nucleophosmin-like isoform X1	1198	9.30E-73
XM_020087518.1	spermatogenesis-associated protein 13 isoform X1	1658	0
XM_020087519.1	spermatogenesis-associated protein 13 isoform X1	1660	0
XM_020087520.1	copine-3-like isoform X1	3454	0
XM_020087521.1	copine-3-like isoform X2	3576	0
XM_020087522.1	regulator of microtubule dynamics protein 2-like	1491	0
XM_020087523.1	netrin-G1 isoform X1	3917	0
XM_020087524.1	netrin-G1 isoform X1	3388	0
XM_020087525.1	netrin-G1 isoform X1	4143	0
XM_020087526.1	protein arginine N-methyltransferase 6	1700	0
XM_020087527.1	nucleoporin p58/p45 isoform X1	3514	0
XM_020087528.1	peptide chain release factor 1-like, mitochondrial	2547	0
XM_020087529.1	nucleoporin p58/p45 isoform X1	3428	0
XM_020087530.1	nucleoporin p58/p45 isoform X3	3324	0
XM_020087531.1	mucin-17-like isoform X1	9448	0
XM_020087532.1	mucin-17-like isoform X1	8556	0
XM_020087533.1	mucin-17-like isoform X1	9445	0
XM_020087534.1	mucin-17-like isoform X3	9094	0
XM_020087535.1	mucin-17-like isoform X3	9302	0
XM_020087536.1	nuclear receptor coactivator 2 isoform X1	8321	0
XM_020087537.1	nuclear receptor coactivator 2 isoform X1	8534	0
XM_020087538.1	nuclear receptor coactivator 2 isoform X1	8223	0
XM_020087539.1	nuclear receptor coactivator 2 isoform X1	8166	0
XM_020087540.1	nuclear receptor coactivator 2 isoform X1	8238	0
XM_020087541.1	nuclear receptor coactivator 2 isoform X1	8140	0
XM_020087542.1	nuclear receptor coactivator 2 isoform X1	8451	0
XM_020087543.1	ectodysplasin-A isoform X1	2283	0
XM_020087544.1	isthmin-1 isoform X1	3045	0
XM_020087545.1	nuclear receptor coactivator 2 isoform X1	8081	0
XM_020087546.1	EMILIN-2 isoform X1	4112	0
XM_020087547.1	EMILIN-2 isoform X2	4109	0
XM_020087548.1	methyltransferase-like protein 4	1538	0
XM_020087549.1	phosphatidate phosphatase LPIN2	3250	0
XM_020087550.1	supervillin isoform X1	7931	0
XM_020087551.1	supervillin isoform X2	7895	0
XM_020087552.1	supervillin isoform X3	7727	0
XM_020087553.1	isthmin-1 isoform X2	3042	0
XM_020087554.1	supervillin isoform X4	7661	0

XM_020087555.1	supervillin isoform X5	7568	0
XM_020087556.1	mitochondrial fission regulator 1	2536	5.67E-169
XM_020087557.1	mitochondrial fission regulator 1	2522	4.89E-169
XM_020087558.1	armadillo repeat-containing protein 1	2946	0
XM_020087559.1	armadillo repeat-containing protein 1	2944	0
XM_020087560.1	WW domain-containing adapter protein with coiled-coil isoform X1	4032	0
XM_020087561.1	WW domain-containing adapter protein with coiled-coil isoform X1	4029	0
XM_020087562.1	ras-related protein Rab-18	2629	2.74E-142
XM_020087563.1	methionine synthase reductase isoform X1	3832	0
XM_020087564.1	methionine synthase reductase isoform X1	3706	0
XM_020087565.1	T-complex protein 1 subunit epsilon	1978	0
XM_020087566.1	GDNF family receptor alpha-1-like isoform X1	4662	0
XM_020087567.1	carboxymethylenebutenolidase homolog	1317	0
XM_020087568.1	carboxymethylenebutenolidase homolog	1340	0
XM_020087569.1	carboxymethylenebutenolidase homolog	1308	0
XM_020087570.1	protein AF-10-like isoform X1	3041	1.63E-80
XM_020087571.1	protein AF-10-like isoform X2	3038	1.43E-81
XM_020087572.1	protein AF-10-like isoform X1	3038	2.84E-46
XM_020087573.1	protein AF-10-like isoform X4	3017	8.61E-85
XM_020087574.1	ribosome biogenesis regulatory protein homolog	1627	7.79E-178
XM_020087575.1	GDNF family receptor alpha-1-like isoform X2	4506	0
XM_020087576.1	tripartite motif-containing protein 55 isoform X1	3468	0
XM_020087577.1	tripartite motif-containing protein 55 isoform X2	3465	0
XM_020087578.1	corticotropin releasing hormone	2613	1.09E-89
XM_020087579.1	corticotropin releasing hormone	2587	9.27E-90
XM_020087580.1	corticotropin releasing hormone	2565	8.17E-90
XM_020087581.1	scavenger receptor class A member 3	4702	0
XM_020087582.1	transmembrane protein 236	2068	0
XM_020087583.1	very-long-chain (3R)-3-hydroxyacyl-CoA dehydratase 1	962	0

XM_020087584.1	solute carrier family 12 member 7-like isoform X1	3415	0
XM_020087585.1	solute carrier family 12 member 7-like isoform X2	3432	0
XM_020087586.1	solute carrier family 12 member 7-like isoform X3	3298	0
XM_020087587.1	zinc finger protein ZIC 4-like	2642	0
XM_020087588.1	arginine vasopressin-induced protein 1	2173	1.06E-105
XM_020087589.1	zinc finger protein ZIC 1 isoform X1	2316	0
XM_020087590.1	zinc finger protein ZIC 1 isoform X2	2311	0
XM_020087591.1	phosphoenolpyruvate carboxykinase [GTP], mitochondrial	3241	0
XM_020087592.1	RNA-binding protein 33 isoform X1	5909	0
XM_020087593.1	RNA-binding protein 33 isoform X2	5906	0
XM_020087594.1	RNA-binding protein 33 isoform X3	5897	0
XM_020087595.1	myomesin-1 isoform X5	5595	0
XM_020087596.1	myomesin-1 isoform X2	5123	0
XM_020087597.1	myomesin-1 isoform X3	5108	0
XM_020087598.1	myomesin-1 isoform X4	5989	0
XM_020087599.1	translin-associated protein X	1504	0
XM_020087600.1	myomesin-1 isoform X5	4633	0
XM_020087601.1	myomesin-1 isoform X5	4621	0
XM_020087602.1	myomesin-1 isoform X6	5973	0
XM_020087603.1	protein disulfide-isomerase TMX3	3670	0
XM_020087604.1	brefeldin A-inhibited guanine nucleotide-exchange protein 1 isoform X1	7199	0
XM_020087605.1	brefeldin A-inhibited guanine nucleotide-exchange protein 1 isoform X2	7253	0
XM_020087606.1	rho GTPase-activating protein 12 isoform X1	4289	0
XM_020087607.1	rho GTPase-activating protein 12 isoform X2	4286	0
XM_020087608.1	rho GTPase-activating protein 12 isoform X3	4214	0
XM_020087609.1	rho GTPase-activating protein 12 isoform X4	4211	0
XM_020087610.1	phosphatidylinositol 5-phosphate 4-kinase type-2 alpha isoform X1	2759	0
XM_020087611.1	phosphatidylinositol 5-phosphate 4-kinase type-2 alpha isoform X2	2750	0
XM_020087612.1	phosphatidylinositol 5-phosphate 4-kinase type-2 alpha isoform X3	2657	0
XM_020087613.1	pleckstrin homology domain-containing family H member 2	6375	0
XM_020087614.1	phosphatidylinositol 5-phosphate 4-kinase type-2 alpha isoform X2	2642	0
XM_020087615.1	high affinity cAMP-specific 3',5'-cyclic phosphodiesterase 7A isoform X1	4069	0
XM_020087616.1	high affinity cAMP-specific 3',5'-cyclic phosphodiesterase 7A isoform X2	4067	0
XM_020087617.1	high affinity cAMP-specific 3',5'-cyclic phosphodiesterase 7A isoform X3	3980	0
XM_020087618.1	polyadenylate-binding protein 2 isoform X1	1682	8.02E-147
XM_020087619.1	polyadenylate-binding protein 2 isoform X2	1679	5.03E-146
XM_020087620.1	polyadenylate-binding protein 2 isoform X3	1222	8.23E-116
XM_020087621.1	double-stranded RNA-binding protein Staufen homolog 2	2235	0
XM_020087622.1	cysteine/serine-rich nuclear protein 1-like	4304	0
XM_020087623.1	cysteine/serine-rich nuclear protein 1-like	4102	0
XM_020087624.1	choline-phosphate cytidylyltransferase B isoform X1	2929	0
XM_020087625.1	DNA mismatch repair protein Mlh1	2478	0
XM_020087626.1	choline-phosphate cytidylyltransferase B isoform X2	2923	0
XM_020087627.1	choline-phosphate cytidylyltransferase B isoform X3	2720	0
XM_020087628.1	pyruvate dehydrogenase kinase, isozyme 3	2588	0
XM_020087629.1	ectodysplasin-A isoform X2	2278	0
XM_020087630.1	NEDD4-like E3 ubiquitin-protein ligase WWP1 isoform X1	4476	0
XM_020087631.1	NEDD4-like E3 ubiquitin-protein ligase WWP1 isoform X1	4609	0
XM_020087632.1	NEDD4-like E3 ubiquitin-protein ligase WWP1 isoform X1	4474	0
XM_020087633.1	poly(U)-binding-splicing factor PUF60 isoform X2	1993	0

XM_020087634.1	poly(U)-binding-splicing factor PUF60 isoform X2	1781	0
XM_020087635.1	poly(U)-binding-splicing factor PUF60 isoform X2	1789	0
XM_020087636.1	poly(U)-binding-splicing factor PUF60 isoform X2	1785	0
XM_020087637.1	poly(U)-binding-splicing factor PUF60 isoform X2	1777	0
XM_020087638.1	poly(U)-binding-splicing factor PUF60 isoform X2	1714	0
XM_020087639.1	receptor-type tyrosine-protein phosphatase epsilon-like isoform X1	3071	0
XM_020087640.1	poly(U)-binding-splicing factor PUF60 isoform X3	1647	0
XM_020087641.1	zinc finger E-box-binding homeobox 2 isoform X1	4747	0
XM_020087642.1	MAM and LDL-receptor class A domain-containing protein 1 isoform X1	5768	0
XM_020087643.1	MAM and LDL-receptor class A domain-containing protein 1 isoform X2	5765	0
XM_020087644.1	leucine-rich repeat-containing protein 14-like	2692	0
XM_020087645.1	ATP-dependent 6-phosphofructokinase, platelet type-like isoform X1	3507	0
XM_020087646.1	ATP-dependent 6-phosphofructokinase, platelet type-like isoform X2	3516	0
XM_020087647.1	receptor-type tyrosine-protein phosphatase epsilon-like isoform X1	2628	0
XM_020087648.1	ATP-dependent 6-phosphofructokinase, platelet type-like isoform X3	3509	0
XM_020087649.1	ATP-dependent 6-phosphofructokinase, platelet type-like isoform X4	3527	0
XM_020087650.1	ATP-dependent 6-phosphofructokinase, platelet type-like isoform X5	3503	0
XM_020087651.1	ATP-dependent 6-phosphofructokinase, platelet type-like isoform X6	3523	0
XM_020087652.1	ATP-dependent 6-phosphofructokinase, platelet type-like isoform X7	3509	0
XM_020087653.1	ATP-dependent 6-phosphofructokinase, platelet type-like isoform X8	3532	0
XM_020087654.1	importin subunit alpha-5	3382	0
XM_020087655.1	protein FAM162A	1250	1.90E-114
XM_020087656.1	receptor-type tyrosine-protein phosphatase epsilon-like isoform X1	2433	0
XM_020087657.1	DNA-dependent protein kinase catalytic subunit	12672	0
XM_020087658.1	zinc finger and BTB domain-containing protein 47 isoform X1	9252	0
XM_020087659.1	zinc finger and BTB domain-containing protein 47 isoform X1	9345	0
XM_020087660.1	kelch-like protein 40	2076	0
XM_020087661.1	deleted in autism protein 1	3959	0
XM_020087662.1	deleted in autism protein 1	3704	0
XM_020087663.1	E3 ubiquitin-protein ligase RLIM	3329	0
XM_020087664.1	serine/threonine-protein kinase PRP4 homolog	4127	0
XM_020087665.1	procollagen-lysine,2-oxoglutarate 5-dioxygenase 2 isoform X1	3377	0

XM_020087666.1	procollagen-lysine,2-oxoglutarate 5-dioxygenase 2 isoform X2	3315	0
XM_020087667.1	phospholipid scramblase 2-like isoform X2	4178	0
XM_020087668.1	phospholipid scramblase 2-like isoform X2	2745	0
XM_020087669.1	elongation of very long chain fatty acids protein 5	3755	0
XM_020087670.1	phospholipid scramblase 2-like isoform X2	3430	5.16E-160
XM_020087671.1	phospholipid scramblase 2-like isoform X2	4076	5.12E-158
XM_020087672.1	phospholipid scramblase 2-like isoform X2	3444	5.79E-160
XM_020087673.1	nuclear receptor-binding protein 2-like	4502	0
XM_020087674.1	PX domain-containing protein 1	3317	1.40E-161
XM_020087675.1	basic helix-loop-helix domain-containing protein USF3	7673	0
XM_020087676.1	elongation of very long chain fatty acids protein 5	3686	0
XM_020087677.1	SPRY domain-containing SOCS box protein 4-like	1875	0
XM_020087678.1	myelin-associated neurite-outgrowth inhibitor	1755	2.05E-117
XM_020087679.1	protein CDV3 homolog isoform X1	2353	1.03E-94
XM_020087680.1	protein CDV3 homolog isoform X2	2350	1.89E-106
XM_020087681.1	SWI/SNF-related matrix-associated actin-dependent regulator of chromatin subfamily D member 3 isoform X1	4536	0
XM_020087682.1	SWI/SNF-related matrix-associated actin-dependent regulator of chromatin subfamily D member 3 isoform X2	4534	0
XM_020087683.1	SWI/SNF-related matrix-associated actin-dependent regulator of chromatin subfamily D member 3 isoform X3	4445	0
XM_020087684.1	SWI/SNF-related matrix-associated actin-dependent regulator of chromatin subfamily D member 3 isoform X4	4443	0
XM_020087685.1	leukocyte elastase inhibitor-like	1757	0
XM_020087686.1	leukocyte elastase inhibitor-like	1753	0
XM_020087687.1	leukocyte elastase inhibitor-like	1423	0
XM_020087688.1	leukocyte elastase inhibitor-like	1454	0
XM_020087689.1	PREDICTED: cofilin-1-A-like	1148	1.67E-114
XM_020087690.1	leukocyte elastase inhibitor-like	1496	0
XM_020087691.1	extracellular sulfatase Sulf-1-like isoform X1	5167	0
XM_020087692.1	extracellular sulfatase Sulf-1-like isoform X1	5095	0
XM_020087693.1	extracellular sulfatase Sulf-1-like isoform X1	4754	0
XM_020087694.1	extracellular sulfatase Sulf-1-like isoform X1	5095	0
XM_020087695.1	extracellular sulfatase Sulf-1-like isoform X1	3326	0
XM_020087696.1	xin actin-binding repeat-containing protein 1-like isoform X1	6058	0
XM_020087697.1	xin actin-binding repeat-containing protein 1-like isoform X1	5774	0
XM_020087698.1	xin actin-binding repeat-containing protein 1-like isoform X1	5755	0
XM_020087699.1	solute carrier family 22 member 13-like	1993	0
XM_020087700.1	ubiquitin-protein ligase E3B	4482	0
XM_020087701.1	RPE-retinal G protein-coupled receptor-like	1381	0
XM_020087702.1	ubiquitin-protein ligase E3B	3608	0
XM_020087703.1	polycomb complex protein BMI-1	2777	0
XM_020087704.1	COMM domain-containing protein 3	937	1.95E-143
XM_020087705.1	voltage-dependent L-type calcium channel subunit beta-2 isoform X1	3020	0
XM_020087706.1	voltage-dependent L-type calcium channel subunit beta-2 isoform X2	2569	0
XM_020087707.1	voltage-dependent L-type calcium channel subunit beta-2 isoform X3	2570	0
XM_020087708.1	voltage-dependent L-type calcium channel subunit beta-2 isoform X4	2905	0
XM_020087709.1	voltage-dependent L-type calcium channel subunit beta-2 isoform X5	2453	0
XM_020087710.1	voltage-dependent L-type calcium channel subunit beta-2 isoform X1	2486	0
XM_020087711.1	glycine--tRNA ligase	2659	0
XM_020087712.1	probable palmitoyltransferase ZDHHC20	4179	0

XM_020087713.1	probable palmitoyltransferase ZDHHC20	4210	0
XM_020087714.1	1-acyl-sn-glycerol-3-phosphate acyltransferase delta	2888	0
XM_020087715.1	translocating chain-associated membrane protein 1	2563	0
XM_020087716.1	integrin beta-1 isoform X1	3980	0
XM_020087717.1	integrin beta-1 isoform X2	3902	0
XM_020087718.1	PREDICTED: importin-4	3900	0
XM_020087719.1	collagen alpha-1(XI) chain-like isoform X1	6159	0
XM_020087720.1	collagen alpha-1(XI) chain-like isoform X2	6156	0
XM_020087721.1	collagen alpha-1(XI) chain-like isoform X3	6156	0
XM_020087722.1	collagen alpha-1(XI) chain-like isoform X4	6153	0
XM_020087723.1	collagen alpha-1(XI) chain-like isoform X5	6066	0
XM_020087724.1	collagen alpha-1(XI) chain-like isoform X6	5775	0
XM_020087725.1	collagen alpha-1(XI) chain-like isoform X7	5681	0
XM_020087726.1	ankyrin repeat and SAM domain-containing protein 6 isoform X1	4522	0
XM_020087727.1	ankyrin repeat and SAM domain-containing protein 6 isoform X2	4142	0
XM_020087728.1	zinc finger HIT domain-containing protein 2	1875	0
XM_020087729.1	40S ribosomal protein S20	569	6.02E-83
XM_020087730.1	myosin regulatory light chain 2, smooth muscle minor isoform	1033	8.50E-124
XM_020087731.1	suppressor of cytokine signaling 6	4940	0
XM_020087732.1	glycine--tRNA ligase-like	2508	0
XM_020087733.1	protein-cysteine N-palmitoyltransferase HHAT-like protein	2095	0
XM_020087734.1	cyclin-Y isoform X1	3292	0
XM_020087735.1	cyclin-Y isoform X2	3271	0
XM_020087736.1	cyclin-Y isoform X3	3268	0
XM_020087737.1	cyclin-Y isoform X4	3247	0
XM_020087738.1	vacuolar protein sorting-associated protein 41 homolog	3039	0
XM_020087739.1	POU domain, class 6, transcription factor 2 isoform X1	3492	0
XM_020087740.1	Krueppel-like factor 5	4195	0
XM_020087741.1	POU domain, class 6, transcription factor 2 isoform X1	3463	0
XM_020087742.1	POU domain, class 6, transcription factor 2 isoform X1	3487	0
XM_020087743.1	POU domain, class 6, transcription factor 2 isoform X1	3478	0
XM_020087744.1	POU domain, class 6, transcription factor 2 isoform X1	3574	0
XM_020087745.1	POU domain, class 6, transcription factor 2 isoform X1	2281	0
XM_020087746.1	POU domain, class 6, transcription factor 2 isoform X1	3794	0
XM_020087747.1	POU domain, class 6, transcription factor 2 isoform X1	3712	0
XM_020087748.1	POU domain, class 6, transcription factor 2 isoform X1	3384	0
XM_020087749.1	POU domain, class 6, transcription factor 2 isoform X6	3375	0
XM_020087750.1	E3 ubiquitin-protein ligase MARCH6	3811	0
XM_020087751.1	arylamine N-acetyltransferase 1-like	1296	0
XM_020087752.1	rho GTPase-activating protein 29-like isoform X1	5294	0
XM_020087753.1	rho GTPase-activating protein 29-like isoform X2	5291	0
XM_020087754.1	transmembrane protein 41A-B-like	3563	2.70E-180
XM_020087755.1	villin-1-like isoform X1	2972	0
XM_020087756.1	villin-1-like isoform X2	2969	0
XM_020087757.1	villin-1-like isoform X3	2903	0
XM_020087758.1	1-phosphatidylinositol 4,5-bisphosphate phosphodiesterase delta-1-like isoform X1	3084	0
XM_020087759.1	1-phosphatidylinositol 4,5-bisphosphate phosphodiesterase delta-1-like isoform X2	3216	0
XM_020087760.1	transmembrane protein 182-like isoform X1	1516	1.20E-149
XM_020087761.1	zinc finger protein 438	4965	0
XM_020087762.1	zinc finger protein 438	5041	0
XM_020087763.1	zinc finger protein 438	4961	0
XM_020087764.1	transcription factor AP-2-alpha isoform X1	2876	0
XM_020087765.1	transcription factor AP-2-alpha isoform X2	2918	0
XM_020087766.1	transcription factor AP-2-alpha isoform X3	3372	0
XM_020087767.1	eyes absent homolog 1 isoform X1	4328	0
XM_020087768.1	eyes absent homolog 1 isoform X2	4341	0
XM_020087769.1	lutropin-choriogonadotropic hormone receptor-like	2624	0
XM_020087770.1	eyes absent homolog 1 isoform X3	4321	0
XM_020087771.1	eyes absent homolog 1 isoform X4	4306	0
XM_020087772.1	eyes absent homolog 1 isoform X5	4276	0
XM_020087773.1	eyes absent homolog 1 isoform X6	4273	0
XM_020087774.1	eyes absent homolog 1 isoform X7	4259	0
XM_020087775.1	eyes absent homolog 1 isoform X8	4255	0
XM_020087776.1	eyes absent homolog 1 isoform X9	4239	0
XM_020087777.1	eyes absent homolog 1 isoform X10	4237	0
XM_020087778.1	serine/threonine-protein kinase Sgk3-like	3854	0
XM_020087779.1	serine/threonine-protein kinase Sgk3-like	3897	0
XM_020087780.1	retinal-specific ATP-binding cassette transporter-like	7686	0
XM_020087781.1	very-long-chain enoyl-CoA reductase-like	1774	0
XM_020087782.1	lisH domain and HEAT repeat-containing protein KIAA1468 homolog isoform X1	4621	0
XM_020087783.1	proto-oncogene c-Rel	3377	0
XM_020087784.1	lisH domain and HEAT repeat-containing protein KIAA1468 homolog isoform X2	4028	0
XM_020087785.1	lisH domain and HEAT repeat-containing protein KIAA1468 homolog isoform X3	4023	0
XM_020087786.1	lisH domain and HEAT repeat-containing protein KIAA1468 homolog isoform X4	4021	0
XM_020087787.1	lisH domain and HEAT repeat-containing protein KIAA1468 homolog isoform X5	4618	0
XM_020087788.1	chondroitin sulfate glucuronyltransferase	3802	0
XM_020087789.1	cyclin-dependent kinase 19-like	2770	0
XM_020087790.1	death-associated protein 1	1441	8.75E-60
XM_020087791.1	plexin domain-containing protein 2-like isoform X1	5327	0

XM_020087792.1	plexin domain-containing protein 2 isoform X1	4999	0
XM_020087793.1	semaphorin-5A isoform X1	4914	0
XM_020087794.1	semaphorin-5A isoform X1	4647	0
XM_020087795.1	semaphorin-5A isoform X1	4913	0
XM_020087796.1	GTP-binding protein SAR1b	1727	3.66E-142
XM_020087797.1	transcriptional repressor NF-X1	3792	0
XM_020087798.1	endophilin-B2 isoform X6	2204	0
XM_020087799.1	PREDICTED: uncharacterized protein LOC109629837	1614	1.94E-166
XM_020087800.1	60S ribosomal protein L7	881	2.21E-169
XM_020087801.1	mitogen-activated protein kinase kinase kinase 3-like isoform X1	4335	0
XM_020087802.1	mitogen-activated protein kinase kinase kinase 3-like isoform X2	4326	0
XM_020087803.1	ranBP-type and C3HC4-type zinc finger-containing protein 1 isoform X1	3623	0
XM_020087804.1	ranBP-type and C3HC4-type zinc finger-containing protein 1 isoform X2	3620	0
XM_020087805.1	prolactin-releasing peptide	1185	9.85E-62
XM_020087806.1	suppressor protein SRP40-like isoform X2	4155	0
XM_020087807.1	suppressor protein SRP40-like isoform X2	2569	0
XM_020087808.1	disintegrin and metalloproteinase domain-containing protein 8-like	3572	0
XM_020087809.1	GRAM domain-containing protein 1C isoform X1	3286	0
XM_020087810.1	GRAM domain-containing protein 1C isoform X2	3244	0
XM_020087811.1	DNA topoisomerase 2-binding protein 1	5106	0
XM_020087812.1	N-alpha-acetyltransferase 50 isoform X1	2023	3.81E-119
XM_020087813.1	N-alpha-acetyltransferase 50 isoform X2	2022	2.78E-118
XM_020087814.1	retinol dehydrogenase 10-like	2901	0
XM_020087815.1	dnaJ homolog subfamily B member 6-like	2104	4.67E-112
XM_020087816.1	dnaJ homolog subfamily B member 6-like	1821	4.44E-78
XM_020087817.1	kinesin-like protein KIF9	2879	0
XM_020087818.1	solute carrier family 22 member 17	3990	0
XM_020087819.1	BMP and activin membrane-bound inhibitor homolog	1962	5.94E-177
XM_020087820.1	amine sulfotransferase-like	1274	0
XM_020087821.1	Golgi reassembly-stacking protein 2-like	2128	0
XM_020087822.1	chymotrypsin-like elastase family member 2A	866	0
XM_020087823.1	GTPase IMAP family member 4-like isoform X1	899	4.54E-148
XM_020087824.1	GTPase IMAP family member 4-like isoform X2	3160	9.82E-138
XM_020087825.1	queuine tRNA-ribosyltransferase accessory subunit 2 isoform X1	2245	0
XM_020087826.1	queuine tRNA-ribosyltransferase accessory subunit 2 isoform X1	2021	0
XM_020087827.1	queuine tRNA-ribosyltransferase accessory subunit 2 isoform X1	2012	0
XM_020087828.1	queuine tRNA-ribosyltransferase accessory subunit 2 isoform X1	1987	0
XM_020087829.1	queuine tRNA-ribosyltransferase accessory subunit 2 isoform X1	2226	0

XM_020087830.1	hepatocyte nuclear factor 4-gamma-like	1489	0
XM_020087831.1	cysteinyl leukotriene receptor 1	1572	0
XM_020087832.1	amine sulfotransferase-like	638	9.03E-92
XM_020087833.1	PREDICTED: 5-oxoprolinase	4570	0
XM_020087834.1	acyl-protein thioesterase 1	1390	5.71E-173
XM_020087835.1	regulator of G-protein signaling 20-like isoform X1	2266	1.35E-126
XM_020087836.1	myotubularin-related protein 6	2466	0
XM_020087837.1	V-type proton ATPase catalytic subunit A	2634	0
XM_020087838.1	V-type proton ATPase catalytic subunit A	2507	0
XM_020087839.1	neutrophil cytosol factor 2	1934	0
XM_020087840.1	diphthamide biosynthesis protein 2	1690	0
XM_020087841.1	diphthamide biosynthesis protein 2	1612	0
XM_020087842.1	homeobox protein engrailed-1-B-like	1931	4.03E-174
XM_020087843.1	transmembrane protein 182-like isoform X2	1505	5.35E-136
XM_020087844.1	EGF-containing fibulin-like extracellular matrix protein 1 isoform X2	3188	0
XM_020087845.1	catenin delta-2-like isoform X1	5034	0
XM_020087846.1	catenin delta-2-like isoform X2	4925	0
XM_020087847.1	EGF-containing fibulin-like extracellular matrix protein 1 isoform X2	3370	0
XM_020087848.1	catenin delta-2-like isoform X3	3951	0
XM_020087849.1	catenin delta-2-like isoform X4	3969	0
XM_020087850.1	phosphatidylinositol 3,4,5-trisphosphate-dependent Rac exchanger 2 protein	6213	0
XM_020087851.1	junctional protein associated with coronary artery disease	7330	0
XM_020087852.1	junctional protein associated with coronary artery disease	7422	0
XM_020087853.1	junctional protein associated with coronary artery disease	7428	0
XM_020087854.1	poly(A) RNA polymerase, mitochondrial	2684	0
XM_020087855.1	DDB1- and CUL4-associated factor 11	2847	0
XM_020087856.1	EGF-containing fibulin-like extracellular matrix protein 1 isoform X2	3360	0
XM_020087857.1	cullin-2 isoform X1	2879	0
XM_020087858.1	cullin-2 isoform X2	2218	0
XM_020087859.1	wiskott-Aldrich syndrome protein family member 3-like	3026	0
XM_020087860.1	clusterin-like protein 1	1493	0
XM_020087861.1	thymidylate synthase	1377	0
XM_020087862.1	mitochondrial enolase superfamily member 1	1170	0
XM_020087863.1	homeobox protein AKR-like	1944	1.88E-173
XM_020087864.1	MAGUK p55 subfamily member 7	4219	0
XM_020087865.1	MAGUK p55 subfamily member 7	4057	0
XM_020087866.1	receptor-type tyrosine-protein phosphatase-like N	2421	0
XM_020087867.1	peroxisomal leader peptide-processing protease	2151	0
XM_020087868.1	transmembrane 9 superfamily member 1	2440	0
XM_020087869.1	myc proto-oncogene protein	1955	0
XM_020087870.1	myc proto-oncogene protein	1965	0

XM_020087871.1	programmed cell death protein 6 isoform X1	1845	2.40E-135
XM_020087872.1	programmed cell death protein 6 isoform X2	1829	3.65E-132
XM_020087873.1	myb-related protein A isoform X1	5530	0
XM_020087874.1	myb-related protein A isoform X2	5512	0
XM_020087875.1	myb-related protein A isoform X3	5419	0
XM_020087876.1	nucleolar and coiled-body phosphoprotein 1-like isoform X1	3982	0
XM_020087877.1	nucleolar and coiled-body phosphoprotein 1-like isoform X2	3812	0
XM_020087878.1	inositol monophosphatase 1-like	1973	3.31E-179
XM_020087879.1	inositol monophosphatase 1-like	2026	1.89E-178
XM_020087880.1	2-oxoglutarate dehydrogenase-like, mitochondrial	4451	0
XM_020087881.1	serine/threonine-protein kinase Sgk3-like	2350	0
XM_020087882.1	serine/threonine-protein kinase Sgk3-like	2400	0
XM_020087883.1	condensin-2 complex subunit G2	3779	0
XM_020087884.1	SKI/DACH domain-containing protein 1	4613	0
XM_020087885.1	sperm-associated antigen 6-like isoform X1	1728	0
XM_020087886.1	sperm-associated antigen 6-like isoform X1	1907	0
XM_020087887.1	sperm-associated antigen 6-like	1871	0
XM_020087888.1	2-oxoglutarate dehydrogenase-like, mitochondrial	4437	0
XM_020087889.1	dermatan-sulfate epimerase-like protein	6510	0
XM_020087890.1	progressive ankylosis protein homolog	2835	0
XM_020087891.1	protein FAM49B	1935	0
XM_020087892.1	protein FAM49B	1930	0
XM_020087893.1	PREDICTED: mucin-17-like	6101	0
XM_020087894.1	delta-type opioid receptor-like	3921	0
XM_020087895.1	BTB/POZ domain-containing protein 3	2867	0
XM_020087896.1	EH domain-containing protein 2 isoform X1	3043	0
XM_020087897.1	EH domain-containing protein 2 isoform X2	3024	0
XM_020087898.1	Golgi SNAP receptor complex member 1	1320	2.86E-166
XM_020087899.1	cGMP-inhibited 3',5'-cyclic phosphodiesterase A-like	4252	0
XM_020087900.1	protein ABHD15	2425	0
XM_020087901.1	28S ribosomal protein S6, mitochondrial	838	4.88E-76
XM_020087902.1	tetratricopeptide repeat protein 9A-like	5797	8.12E-147
XM_020087903.1	general transcription factor II-I repeat domain-containing protein 2-like	3302	0
XM_020087904.1	gem-associated protein 7	1142	9.51E-92
XM_020087905.1	hypermethylated in cancer 1 protein isoform X3	6931	0
XM_020087906.1	hypermethylated in cancer 1 protein isoform X3	6905	0
XM_020087907.1	hypermethylated in cancer 1 protein isoform X3	7012	0
XM_020087908.1	hypermethylated in cancer 1 protein isoform X3	7028	0
XM_020087909.1	F-BAR and double SH3 domains protein 2 isoform X1	3236	0
XM_020087910.1	F-BAR and double SH3 domains protein 2 isoform X2	3173	0
XM_020087911.1	F-BAR and double SH3 domains protein 2 isoform X3	3161	0

XM_020087912.1	conserved oligomeric Golgi complex subunit 6 isoform X1	2399	0
XM_020087913.1	ras guanyl-releasing protein 3	4376	0
XM_020087914.1	conserved oligomeric Golgi complex subunit 6 isoform X2	2291	0
XM_020087915.1	cell adhesion molecule 1-like	4981	0
XM_020087916.1	intraflagellar transport protein 80 homolog isoform X1	2728	0
XM_020087917.1	intraflagellar transport protein 80 homolog isoform X2	2945	0
XM_020087918.1	retinitis pigmentosa 1-like 1 protein isoform X1	3332	0
XM_020087919.1	retinitis pigmentosa 1-like 1 protein isoform X1	3262	0
XM_020087920.1	retinitis pigmentosa 1-like 1 protein isoform X1	3285	0
XM_020087921.1	retinitis pigmentosa 1-like 1 protein isoform X1	1391	3.52E-58
XM_020087922.1	ras guanyl-releasing protein 3	4206	0
XM_020087923.1	retinitis pigmentosa 1-like 1 protein isoform X1	1341	6.24E-32
XM_020087924.1	60S ribosomal export protein NMD3	2013	0
XM_020087925.1	60S ribosomal export protein NMD3	2014	0
XM_020087926.1	filamin-interacting protein FAM101B	2507	7.91E-127
XM_020087927.1	PREDICTED: claudin-8-like	2103	0
XM_020087928.1	arfaptin-2-like isoform X1	3010	0
XM_020087929.1	arfaptin-2-like isoform X2	2947	0
XM_020087930.1	arfaptin-2-like isoform X3	2860	0
XM_020087931.1	progesterone receptor	2884	0
XM_020087932.1	immunoglobulin superfamily member 10	8034	0
XM_020087933.1	probable tRNA methyltransferase 9-like protein isoform X1	2428	0
XM_020087934.1	cathepsin F	3506	3.20E-83
XM_020087935.1	mastermind-like protein 2	3310	0
XM_020087936.1	layilin isoform X2	4022	0
XM_020087937.1	periostin-like isoform X2	2631	0
XM_020087938.1	transmembrane and coiled-coil domain-containing protein 6 isoform X1	2291	0
XM_020087939.1	periostin-like isoform X2	2532	0
XM_020087940.1	intraflagellar transport protein 20 homolog	1780	6.91E-76
XM_020087941.1	intraflagellar transport protein 20 homolog	1862	1.27E-75
XM_020087942.1	serine/threonine-protein kinase Nek8	4065	0
XM_020087943.1	isobutyryl-CoA dehydrogenase, mitochondrial	2683	0
XM_020087944.1	microtubule-associated tumor suppressor candidate 2-like isoform X1	4149	0
XM_020087945.1	microtubule-associated tumor suppressor candidate 2-like isoform X2	4146	0
XM_020087946.1	microtubule-associated tumor suppressor candidate 2-like isoform X3	1555	1.72E-179
XM_020087947.1	SPARC-related modular calcium-binding protein 2 isoform X1	2565	0
XM_020087948.1	erythrocyte band 7 integral membrane protein-like	936	0
XM_020087949.1	transmembrane protease serine 2	2810	0

XM_020087950.1	transmembrane protease serine 2	2804	0
XM_020087951.1	PREDICTED: uncharacterized protein C11orf57 homolog	2291	1.66E-116
XM_020087952.1	bone morphogenetic protein 4	3079	0
XM_020087953.1	ras-related protein Rab-34 isoform X1	1597	0
XM_020087954.1	ras-related protein Rab-34 isoform X2	1593	0
XM_020087955.1	SPARC-related modular calcium-binding protein 2 isoform X2	2556	0
XM_020087956.1	adenine phosphoribosyltransferase	789	2.37E-144
XM_020087957.1	transcriptional adapter 2-alpha	1629	0
XM_020087958.1	beta-1,3-glucosyltransferase-like isoform X2	2154	0
XM_020087959.1	coiled-coil-helix-coiled-coil-helix domain-containing protein 2	1055	5.94E-36
XM_020087960.1	reticulon-4 receptor-like 1	3884	0
XM_020087961.1	28S ribosomal protein S23, mitochondrial isoform X1	1086	1.36E-139
XM_020087962.1	28S ribosomal protein S23, mitochondrial isoform X2	1083	7.88E-139
XM_020087963.1	phosphorylase b kinase gamma catalytic chain, skeletal muscle/heart isoform	2364	0
XM_020087964.1	RILP-like protein 1 isoform X1	2541	0
XM_020087965.1	RILP-like protein 1 isoform X2	2643	0
XM_020087966.1	disrupted in schizophrenia 1 protein	5323	0
XM_020087967.1	UPF0692 protein C19orf54 homolog	1884	0
XM_020087968.1	UPF0692 protein C19orf54 homolog	1861	0
XM_020087969.1	myeloid leukemia factor 1 isoform X1	2383	0
XM_020087970.1	myeloid leukemia factor 1 isoform X2	1032	0
XM_020087971.1	myeloid leukemia factor 1 isoform X3	2339	4.89E-173
XM_020087972.1	serine/Arginine-related protein 53	1475	4.59E-90
XM_020087973.1	serine/Arginine-related protein 53	1506	6.28E-90
XM_020087974.1	fukutin-related protein	2250	0
XM_020087975.1	THAP domain-containing protein 5-like	1900	0
XM_020087976.1	interferon-induced very large GTPase 1-like isoform X2	5601	0
XM_020087977.1	interferon-induced very large GTPase 1-like isoform X2	5843	0
XM_020087978.1	interferon-induced very large GTPase 1-like isoform X2	5458	0
XM_020087979.1	cdc42 effector protein 1-like	2333	0
XM_020087980.1	interferon-induced very large GTPase 1-like isoform X2	5026	0
XM_020087981.1	short transient receptor potential channel 4-like	4092	0
XM_020087982.1	glutamate receptor ionotropic, kainate 4	3388	0
XM_020087983.1	TPA-induced transmembrane protein	2483	2.24E-147
XM_020087984.1	HHIP-like protein 1	4658	0
XM_020087985.1	HHIP-like protein 1	4783	0
XM_020087986.1	contactin-5 isoform X1	4446	0
XM_020087987.1	contactin-5 isoform X2	4434	0
XM_020087988.1	lipoma HMGIC fusion partner-like 3 protein isoform X1	1418	5.03E-113
XM_020087989.1	probable E3 ubiquitin-protein ligase HERC6	3673	0
XM_020087990.1	rhomboid-related protein 4 isoform X1	1418	0
XM_020087991.1	non-structural maintenance of chromosomes element 4 homolog A-like	1435	0
XM_020087992.1	rhomboid-related protein 4 isoform X2	1409	0
XM_020087993.1	synaptotagmin-like protein 2 isoform X1	3241	0
XM_020087994.1	synaptotagmin-like protein 2 isoform X2	2728	0
XM_020087995.1	synaptotagmin-like protein 2 isoform X3	3127	0
XM_020087996.1	RNA-binding protein Musashi homolog 2-like isoform X1	4551	0
XM_020087997.1	RNA-binding protein Musashi homolog 2-like isoform X2	4603	0
XM_020087998.1	RNA-binding protein Musashi homolog 2-like isoform X2	1028	0
XM_020087999.1	non-structural maintenance of chromosomes element 4 homolog A-like	1452	0
XM_020088000.1	neutral cholesterol ester hydrolase 1-like	3824	0
XM_020088001.1	P2Y purinoceptor 6	3921	0
XM_020088002.1	P2Y purinoceptor 6	3995	0
XM_020088003.1	group 3 secretory phospholipase A2-like	3742	0
XM_020088004.1	rho guanine nucleotide exchange factor 19	4051	0
XM_020088005.1	PREDICTED: uncharacterized protein LOC109630006	1430	0
XM_020088006.1	P2Y purinoceptor 2	2622	0
XM_020088007.1	ankyrin repeat and protein kinase domain-containing protein 1	2768	0
XM_020088008.1	prostacyclin receptor isoform X1	2751	0
XM_020088009.1	prostacyclin receptor isoform X2	1286	1.58E-179
XM_020088010.1	prostacyclin receptor isoform X3	1160	0
XM_020088011.1	molybdenum cofactor sulfurase	4018	0
XM_020088012.1	endothelial cell-selective adhesion molecule-like	2575	0
XM_020088013.1	G-protein coupled receptor 4	4472	0
XM_020088014.1	cell adhesion molecule 2-like isoform X1	3245	0
XM_020088015.1	cell adhesion molecule 2-like isoform X2	3218	0
XM_020088016.1	calcium-binding protein 8 isoform X1	4265	0
XM_020088017.1	calcium-binding protein 8 isoform X1	4257	6.99E-131
XM_020088018.1	transmembrane protein 136	2765	5.21E-144
XM_020088019.1	transmembrane protein 136	2483	4.26E-145
XM_020088020.1	transmembrane and coiled-coil domain-containing protein 6 isoform X2	2287	0
XM_020088021.1	sodium channel subunit beta-3 isoform X1	1720	6.23E-163
XM_020088022.1	sodium channel subunit beta-3 isoform X2	1333	1.82E-137
XM_020088023.1	dual specificity protein phosphatase 14	2124	1.31E-138
XM_020088024.1	centrosomal protein of 126 kDa	3416	0
XM_020088025.1	serine protease HTRA1	1935	0
XM_020088026.1	centrosomal protein of 126 kDa	3295	0
XM_020088027.1	FH2 domain-containing protein 1-like	2986	0
XM_020088028.1	FH2 domain-containing protein 1-like	2873	0

XM_020088029.1	oligodendrocyte-myelin glycoprotein-like	1186	0
XM_020088030.1	Na(+)/H(+) exchange regulatory cofactor NHE-RF3-like	1246	0
XM_020088031.1	protocadherin Fat 3	12367	0
XM_020088032.1	small G protein signaling modulator 2-like	7323	0
XM_020088033.1	isoaspartyl peptidase/L-asparaginase	2669	0
XM_020088034.1	melatonin receptor type 1B-like	1065	0
XM_020088035.1	lysosomal acid phosphatase-like	3300	0
XM_020088036.1	neuroblast differentiation-associated protein AHNAK-like	4809	0
XM_020088037.1	PREDICTED: protocadherin-16-like	10172	0
XM_020088038.1	PREDICTED: protocadherin-16-like	2245	0
XM_020088039.1	guanylyl cyclase-activating protein 1-like	570	2.96E-138
XM_020088040.1	asparagine--tRNA ligase, cytoplasmic isoform X1	1478	0
XM_020088041.1	isoaspartyl peptidase/L-asparaginase	2655	0
XM_020088042.1	rho GTPase-activating protein 42	5158	0
XM_020088043.1	PREDICTED: nectin-1-like	1642	0
XM_020088044.1	cell adhesion molecule-related/down-regulated by oncogenes-like	568	3.55E-95
XM_020088045.1	suppressor of tumorigenicity 14 protein homolog	3240	0
XM_020088046.1	ryanodine receptor 1-like	16565	0
XM_020088047.1	cytochrome P450 2G1-like	3921	0
XM_020088048.1	neuronal acetylcholine receptor subunit alpha-9-like	1599	0
XM_020088049.1	olfactory receptor 52N5-like	936	0
XM_020088050.1	olfactory receptor 52B2-like	936	0
XM_020088051.1	olfactory receptor 52B2-like	942	0
XM_020088052.1	E3 ubiquitin-protein ligase MARCH8 isoform X1	1804	0
XM_020088053.1	olfactory receptor 2AT4-like	966	0
XM_020088054.1	olfactory receptor 2AT4-like	975	0
XM_020088055.1	protocadherin Fat 4-like	2150	0
XM_020088056.1	WD repeat-containing protein 49-like	3017	0
XM_020088057.1	neurobeachin-like isoform X11	9038	0
XM_020088058.1	nectin-3-like protein isoform X1	3067	0
XM_020088059.1	E3 ubiquitin-protein ligase MARCH8 isoform X2	1783	0
XM_020088060.1	synergin gamma	4943	0
XM_020088061.1	multidrug and toxin extrusion protein 1-like	453	6.56E-107
XM_020088062.1	multidrug and toxin extrusion protein 1-like	1777	0
XM_020088063.1	multidrug and toxin extrusion protein 1-like	1467	0
XM_020088064.1	neural cell adhesion molecule 1-like	5255	0
XM_020088065.1	protein turtle homolog B-like	7034	0
XM_020088066.1	zona pellucida-like domain-containing protein 1	1368	0
XM_020088067.1	E3 ubiquitin-protein ligase MARCH8 isoform X3	1390	0
XM_020088068.1	synaptotagmin-like protein 2	2149	0
XM_020088069.1	zona pellucida-like domain-containing protein 1	1065	0
XM_020088070.1	mediator of RNA polymerase II transcription subunit 13-like	5736	0
XM_020088071.1	extracellular calcium-sensing receptor-like	2613	0
XM_020088072.1	extracellular calcium-sensing receptor-like	2664	0
XM_020088073.1	extracellular calcium-sensing receptor-like	4035	0
XM_020088074.1	extracellular calcium-sensing receptor-like	2463	0
XM_020088075.1	extracellular calcium-sensing receptor-like	2505	0
XM_020088076.1	extracellular calcium-sensing receptor-like	2547	0
XM_020088077.1	rho guanine nucleotide exchange factor 16	3973	0
XM_020088078.1	homeobox protein HMX3-A	2247	0
XM_020088079.1	2-oxoglutarate receptor 1-like	1164	0
XM_020088080.1	P2Y purinoceptor 13-like	1431	0
XM_020088081.1	P2Y purinoceptor 6	993	0
XM_020088082.1	structural maintenance of chromosomes protein 4	4219	0
XM_020088083.1	SH2B adapter protein 3	1199	0
XM_020088084.1	PREDICTED: sialidase-4-like	1565	0
XM_020088085.1	signal-induced proliferation-associated 1-like protein 3	6768	0
XM_020088086.1	calpain-1 catalytic subunit-like	2277	0
XM_020088087.1	protein jagged-1b-like	3648	0
XM_020088088.1	cleft lip and palate transmembrane protein 1-like protein	1704	0
XM_020088089.1	homeobox protein HMX2	1929	2.85E-158
XM_020088090.1	protein phosphatase 1 regulatory subunit 37-like	2211	0
XM_020088091.1	SH3 domain-binding glutamic acid-rich-like protein	1468	1.03E-77
XM_020088092.1	PREDICTED: calmodulin, partial	1929	3.20E-57
XM_020088093.1	neuronal PAS domain-containing protein 1	3397	0
XM_020088094.1	transmembrane protein 160-like	401	1.20E-45
XM_020088095.1	Down syndrome cell adhesion molecule-like protein 1	9867	0
XM_020088096.1	centrosomal protein of 57 kDa-like	1248	0
XM_020088097.1	zona pellucida-like domain-containing protein 1	1648	0
XM_020088098.1	tetratricopeptide repeat protein 12	1707	0
XM_020088099.1	beta-soluble NSF attachment protein isoform X1	1904	0
XM_020088100.1	hepatocyte cell adhesion molecule-like	1608	0
XM_020088101.1	LIM domain kinase 1-like	5131	0
XM_020088102.1	PREDICTED: uncharacterized protein LOC109630091	964	0
XM_020088103.1	forkhead box protein N1	1734	0
XM_020088104.1	solute carrier organic anion transporter family member 1C1-like	5009	0
XM_020088105.1	tRNA-dihydrouridine(20a/20b) synthase [NAD(P)+]-like	828	0
XM_020088106.1	vacuolar protein sorting-associated protein 53 homolog	4072	0
XM_020088107.1	beta-soluble NSF attachment protein isoform X2	1513	1.01E-153

XM_020088108.1	NACHT and WD repeat domain-containing protein 2-like	5073	0
XM_020088109.1	tumor necrosis factor receptor superfamily member 19L	2928	0
XM_020088110.1	L-aminoadipate-semialdehyde dehydrogenase-phosphopantetheinyl transferase	976	0
XM_020088111.1	glutamate receptor ionotropic, kainate 1-like	2614	0
XM_020088112.1	BICD family-like cargo adapter 1	2309	0
XM_020088113.1	alpha-(1,3)-fucosyltransferase 4-like	1705	0
XM_020088114.1	PREDICTED: claudin-4-like	1062	3.49E-115
XM_020088115.1	optic atrophy 3 protein	1127	1.05E-94
XM_020088116.1	extracellular calcium-sensing receptor-like	2661	0
XM_020088117.1	extracellular calcium-sensing receptor-like	2568	0
XM_020088118.1	transmembrane protein 254	1129	1.23E-76
XM_020088119.1	extracellular calcium-sensing receptor-like	2565	0
XM_020088120.1	extracellular calcium-sensing receptor-like	2448	0
XM_020088121.1	extracellular calcium-sensing receptor-like	2355	0
XM_020088122.1	extracellular calcium-sensing receptor-like	2484	0
XM_020088123.1	extracellular calcium-sensing receptor-like	2454	0
XM_020088124.1	serine/threonine-protein kinase Nek3	1927	0
XM_020088125.1	glyoxalase domain-containing protein 4	1987	0
XM_020088126.1	fructose-bisphosphate aldolase C-A-like	1887	0
XM_020088127.1	thioredoxin domain-containing protein 17	737	1.78E-89
XM_020088128.1	capZ-interacting protein-like isoform X1	1739	8.84E-137
XM_020088129.1	lysophosphatidic acid receptor 4	2136	0
XM_020088130.1	PREDICTED: cyclin-J	1908	0
XM_020088131.1	capZ-interacting protein-like isoform X1	1715	6.83E-137
XM_020088132.1	capZ-interacting protein-like isoform X1	1621	2.40E-137
XM_020088133.1	capZ-interacting protein-like isoform X1	1736	4.91E-136
XM_020088134.1	high affinity cGMP-specific 3',5'-cyclic phosphodiesterase 9A-like	2510	0
XM_020088135.1	mitochondrial ornithine transporter 1-like	1677	0
XM_020088136.1	histone H2A-like	610	6.82E-98
XM_020088137.1	flavin reductase (NADPH)-like	1233	7.58E-159
XM_020088138.1	mediator of RNA polymerase II transcription subunit 29	886	1.20E-104
XM_020088139.1	prolyl 4-hydroxylase subunit alpha-3 isoform X1	2211	0
XM_020088140.1	prolyl 4-hydroxylase subunit alpha-3 isoform X2	2260	0
XM_020088141.1	prolyl 4-hydroxylase subunit alpha-3 isoform X3	2206	0
XM_020088142.1	collagen alpha-1(XXI) chain-like isoform X1	3790	0
XM_020088143.1	D(2) dopamine receptor	2188	0
XM_020088144.1	protein FAM181A	1786	0
XM_020088145.1	protein FAM98B-like	1413	0
XM_020088146.1	growth hormone secretagogue receptor type 1-like	2383	0
XM_020088147.1	PREDICTED: uncharacterized protein LOC109630129	1210	0
XM_020088148.1	leucine-rich repeat and fibronectin type III domain-containing protein 1	2103	0
XM_020088149.1	collagen alpha-1(XXI) chain-like isoform X1	3804	0
XM_020088150.1	serine/threonine-protein kinase DCLK1-like	4250	0
XM_020088151.1	spartin-like isoform X1	2951	0
XM_020088152.1	spartin-like isoform X2	2998	0
XM_020088153.1	nectin-3-like protein isoform X1	5251	0
XM_020088154.1	nectin-3-like protein isoform X1	5248	0
XM_020088155.1	ribosomal RNA-processing protein 8	2469	0
XM_020088156.1	protein mab-21-like 1	2331	0
XM_020088157.1	diablo homolog, mitochondrial-like	1481	6.50E-172

XM_020088158.1	olfactory receptor 52B2-like	1031	0
XM_020088159.1	putative polypeptide N-acetylgalactosaminyltransferase-like protein 3	1905	0
XM_020088160.1	putative polypeptide N-acetylgalactosaminyltransferase-like protein 3	1429	2.83E-84
XM_020088161.1	beta-crystallin B1-like	1462	8.86E-144
XM_020088162.1	kelch repeat and BTB domain-containing protein 3 isoform X1	2985	0
XM_020088163.1	kelch repeat and BTB domain-containing protein 3 isoform X1	2636	0
XM_020088164.1	PREDICTED: claudin-8-like	916	3.67E-132
XM_020088165.1	protein FAM83H-like	4085	0
XM_020088166.1	protein fosB isoform X1	3130	0
XM_020088167.1	protein fosB isoform X2	3100	0
XM_020088168.1	protein fosB isoform X3	2976	3.39E-161
XM_020088169.1	short stature homeobox protein 2	2416	8.41E-168
XM_020088170.1	short transient receptor potential channel 6	2846	0
XM_020088171.1	thymocyte nuclear protein 1	956	4.51E-163
XM_020088172.1	PREDICTED: claudin-8-like	1991	0
XM_020088173.1	P2Y purinoceptor 13-like	1672	0
XM_020088174.1	protein sel-1 homolog 3-like	3359	0
XM_020088175.1	LYR motif-containing protein 9	865	4.78E-39
XM_020088176.1	gap junction alpha-5 protein-like	2837	0
XM_020088177.1	P2Y purinoceptor 13-like	1554	0
XM_020088178.1	P2Y purinoceptor 13-like	1545	0
XM_020088179.1	protein unc-119 homolog A	1613	2.01E-152
XM_020088180.1	beta-crystallin A1-1	591	1.28E-149
XM_020088181.1	probable G-protein coupled receptor 149	3568	0
XM_020088182.1	---NA---	924	
XM_020088183.1	lens fiber membrane intrinsic protein-like	704	1.63E-125
XM_020088184.1	lens fiber membrane intrinsic protein-like	699	5.07E-126
XM_020088185.1	prominin-1 isoform X4	3546	0
XM_020088186.1	guanylate cyclase soluble subunit alpha-2	3296	0

XM_020088187.1	interferon alpha/beta receptor 2-like	2763	0
XM_020088188.1	interferon alpha/beta receptor 2-like	2472	0
XM_020088189.1	olfactory receptor 2AT4-like	1266	0
XM_020088190.1	G-protein coupled receptor 4-like	1853	0
XM_020088191.1	gap junction gamma-1 protein-like	1107	0
XM_020088192.1	nuclear apoptosis-inducing factor 1-like	1946	0
XM_020088193.1	post-GPI attachment to proteins factor 2-like	1367	1.62E-161
XM_020088194.1	FXYD domain-containing ion transport regulator 6-like isoform X2	940	2.52E-54
XM_020088195.1	PREDICTED: uncharacterized protein LOC109630173	1868	0
XM_020088196.1	PREDICTED: uncharacterized protein KIAA1841 homolog	3156	0
XM_020088197.1	sprouty-related, EVH1 domain-containing protein 1-like	1406	0
XM_020088198.1	carbonic anhydrase 4-like	1519	0
XM_020088199.1	inner ear-specific collagen-like	1464	0
XM_020088200.1	uncharacterized membrane protein C3orf80 homolog	877	2.77E-124
XM_020088201.1	PREDICTED: claudin-17-like	898	3.05E-161
XM_020088202.1	lysophosphatidic acid receptor 4	2286	0
XM_020088203.1	prolactin-releasing peptide receptor-like	1474	0
XM_020088204.1	zona pellucida-like domain-containing protein 1	1275	0
XM_020088205.1	threonine aspartase 1 isoform X1	2704	0
XM_020088206.1	N-acetyllactosaminide beta-1,3-N-acetylglucosaminyltransferase 2-like	1493	0
XM_020088207.1	RNA polymerase II-associated protein 3	993	0
XM_020088208.1	serine palmitoyltransferase small subunit B	322	1.26E-53
XM_020088209.1	PREDICTED: uncharacterized protein LOC109630189	1783	0
XM_020088210.1	threonine aspartase 1 isoform X1	2726	0
XM_020088211.1	tripartite motif-containing protein 59	2652	0
XM_020088212.1	tripartite motif-containing protein 59	2623	0
XM_020088213.1	tripartite motif-containing protein 59	2404	0
XM_020088214.1	importin subunit alpha-3	2754	0
XM_020088215.1	rho GTPase-activating protein 5	8444	0
XM_020088216.1	AP-2 complex subunit sigma isoform X1	2433	6.76E-104
XM_020088217.1	AP-2 complex subunit sigma isoform X2	2400	4.93E-95
XM_020088218.1	threonine aspartase 1 isoform X1	1625	0
XM_020088219.1	AP-2 complex subunit sigma isoform X1	2399	1.15E-94
XM_020088220.1	AP-2 complex subunit sigma isoform X1	2468	2.28E-93
XM_020088221.1	PREDICTED: claudin-4-like	1298	2.71E-118
XM_020088222.1	PREDICTED: claudin-4-like	1107	4.98E-148
XM_020088223.1	PREDICTED: claudin-4-like	1273	3.29E-127
XM_020088224.1	PREDICTED: claudin-4-like	1236	1.69E-139
XM_020088225.1	PREDICTED: claudin-4-like	1214	6.90E-124
XM_020088226.1	PREDICTED: claudin-4-like	1142	6.66E-117
XM_020088227.1	WD repeat-containing protein 81	7672	0
XM_020088228.1	replication protein A 70 kDa DNA-binding subunit	2661	0
XM_020088229.1	plasma protease C1 inhibitor	1882	0
XM_020088230.1	homeobox protein SIX2	1939	4.34E-166
XM_020088231.1	plasma protease C1 inhibitor	2165	0
XM_020088232.1	zinc finger and BTB domain-containing protein 16-A-like	4372	0
XM_020088233.1	dixin-A-like isoform X1	2185	0
XM_020088234.1	dixin-A-like isoform X2	2182	0
XM_020088235.1	transmembrane protease serine 5 isoform X1	1635	0
XM_020088236.1	transmembrane protease serine 5 isoform X2	1505	0
XM_020088237.1	transmembrane protease serine 5 isoform X3	1515	0
XM_020088238.1	PIH1 domain-containing protein 2 isoform X1	1345	0
XM_020088239.1	PIH1 domain-containing protein 2 isoform X2	1333	0

XM_020088240.1	E3 ubiquitin-protein ligase CBL	6962	0
XM_020088241.1	cell surface glycoprotein MUC18 isoform X1	3597	0
XM_020088242.1	cell surface glycoprotein MUC18 isoform X2	3537	0
XM_020088243.1	inactive phospholipase D5-like isoform X1	4658	0
XM_020088244.1	cell surface glycoprotein MUC18 isoform X3	3495	0
XM_020088245.1	coiled-coil domain-containing protein 153	1305	8.28E-114
XM_020088246.1	serine/threonine-protein kinase D2	4834	0
XM_020088247.1	striatin isoform X2	2635	0
XM_020088248.1	protein Smaug homolog 2	4907	0
XM_020088249.1	suppressor of cytokine signaling 5	5073	0
XM_020088250.1	pre-mRNA-processing-splicing factor 8	7728	0
XM_020088251.1	inactive phospholipase D5-like isoform X2	4552	0
XM_020088252.1	neurofibromin isoform X1	12312	0
XM_020088253.1	neurofibromin isoform X2	12309	0
XM_020088254.1	neurofibromin isoform X3	12309	0
XM_020088255.1	neurofibromin isoform X4	12309	0
XM_020088256.1	neurofibromin isoform X5	12306	0
XM_020088257.1	neurofibromin isoform X6	12249	0
XM_020088258.1	neurofibromin isoform X7	5409	0
XM_020088259.1	neurofibromin isoform X8	4095	0
XM_020088260.1	neurofibromin isoform X1	2662	0
XM_020088261.1	neurofibromin isoform X1	2507	0
XM_020088262.1	neurofibromin isoform X1	2508	0
XM_020088263.1	neurofibromin isoform X11	12246	0
XM_020088264.1	cytochrome P450 2G1-like	1878	0
XM_020088265.1	cyclin-dependent kinase-like 1	1556	0

XM_020088266.1	protein CASP isoform X1	7849	0
XM_020088267.1	protein CASP isoform X2	7952	0
XM_020088268.1	stAR-related lipid transfer protein 13-like isoform X1	4780	0
XM_020088269.1	stAR-related lipid transfer protein 13-like isoform X1	4830	0
XM_020088270.1	stAR-related lipid transfer protein 13-like isoform X1	5681	0
XM_020088271.1	stAR-related lipid transfer protein 13-like isoform X1	4564	0
XM_020088272.1	stAR-related lipid transfer protein 13-like isoform X1	3412	0
XM_020088273.1	stAR-related lipid transfer protein 13-like isoform X1	3449	0
XM_020088274.1	homeobox protein SIX3 isoform X1	1939	0
XM_020088275.1	stAR-related lipid transfer protein 13-like isoform X1	3332	0
XM_020088276.1	stAR-related lipid transfer protein 13-like isoform X1	3466	0
XM_020088277.1	probable tRNA methyltransferase 9-like protein isoform X1	3489	0
XM_020088278.1	probable tRNA methyltransferase 9-like protein isoform X1	3586	0
XM_020088279.1	probable tRNA methyltransferase 9-like protein isoform X1	3413	0
XM_020088280.1	probable tRNA methyltransferase 9-like protein isoform X1	2813	0
XM_020088281.1	histone deacetylase 11	1581	0
XM_020088282.1	histone deacetylase 11	1540	0
XM_020088283.1	NLR family member X1 isoform X1	3658	0
XM_020088284.1	homeobox protein SIX3 isoform X2	1774	2.15E-162
XM_020088285.1	NLR family member X1 isoform X2	3571	0
XM_020088286.1	heterogeneous nuclear ribonucleoprotein U-like protein 1	3821	0
XM_020088287.1	RAS guanyl-releasing protein 1-like	4106	0
XM_020088288.1	protein FAM168A isoform X1	2033	6.38E-126
XM_020088289.1	protein FAM168A isoform X2	1985	6.30E-112
XM_020088290.1	protein FAM168A isoform X1	1925	1.33E-109
XM_020088291.1	protein FAM168A isoform X2	1877	2.46E-95
XM_020088292.1	histone-lysine N-methyltransferase 2A isoform X1	17903	0
XM_020088293.1	histone-lysine N-methyltransferase 2A isoform X2	17927	0
XM_020088294.1	complement C1q tumor necrosis factor-related protein 2	1234	1.19E-144
XM_020088295.1	MARVEL domain-containing protein 1	1920	1.78E-74
XM_020088296.1	platelet-activating factor acetylhydrolase IB subunit alpha	5400	0
XM_020088297.1	platelet-activating factor acetylhydrolase IB subunit alpha	5260	0
XM_020088298.1	platelet-activating factor acetylhydrolase IB subunit alpha	5286	0
XM_020088299.1	RNA-binding protein Nova-1-like isoform X1	2384	0
XM_020088300.1	RNA-binding protein Nova-1-like isoform X2	11080	0
XM_020088301.1	RNA-binding protein Nova-1-like isoform X3	2315	0
XM_020088302.1	RNA-binding protein Nova-1-like isoform X4	11011	0
XM_020088303.1	clustered mitochondria protein homolog isoform X1	6811	0
XM_020088304.1	clustered mitochondria protein homolog isoform X2	6724	0
XM_020088305.1	ubiquitin carboxyl-terminal hydrolase 16 isoform X1	4053	0
XM_020088306.1	ubiquitin carboxyl-terminal hydrolase 16 isoform X2	4050	0
XM_020088307.1	pituitary homeobox 3	2013	6.70E-180
XM_020088308.1	transcription regulator protein BACH1-like	2941	0
XM_020088309.1	RWD domain-containing protein 2B	1810	0
XM_020088310.1	rab5 GDP/GTP exchange factor-like	3530	0
XM_020088311.1	rab5 GDP/GTP exchange factor-like	3407	0
XM_020088312.1	low affinity immunoglobulin gamma Fc region receptor III-like	1289	0
XM_020088313.1	syntaxin-1A-like isoform X1	1404	0
XM_020088314.1	syntaxin-1A-like isoform X2	1405	0
XM_020088315.1	syntaxin-1A-like isoform X3	2053	0
XM_020088316.1	syntaxin-1A-like isoform X5	1301	3.00E-172
XM_020088317.1	syntaxin-1A-like isoform X5	1337	2.58E-178
XM_020088318.1	syntaxin-1A-like isoform X6	1178	8.64E-154
XM_020088319.1	caspase-1-like isoform X1	979	0
XM_020088320.1	PREDICTED: shootin-1	3008	0
XM_020088321.1	caspase-1-like isoform X1	968	0
XM_020088322.1	serine/threonine-protein kinase TAO1-like	4398	0
XM_020088323.1	serine/threonine-protein kinase TAO1-like	4538	0
XM_020088324.1	serine/threonine-protein kinase TAO1-like	4584	0
XM_020088325.1	protein KIAA2022 homolog	6962	0
XM_020088326.1	dual specificity tyrosine-phosphorylation-regulated kinase 1A isoform X1	6234	0
XM_020088327.1	dual specificity tyrosine-phosphorylation-regulated kinase 1A isoform X1	5931	0
XM_020088328.1	dual specificity tyrosine-phosphorylation-regulated kinase 1A isoform X1	5390	0
XM_020088329.1	dual specificity tyrosine-phosphorylation-regulated kinase 1A isoform X1	5389	0
XM_020088330.1	dual specificity tyrosine-phosphorylation-regulated kinase 1A isoform X1	6164	0
XM_020088331.1	PREDICTED: shootin-1	3021	0
XM_020088332.1	carboxypeptidase D	6482	0
XM_020088333.1	PREDICTED: uncharacterized protein C22orf31 homolog	1744	5.75E-121
XM_020088334.1	probable ATP-dependent RNA helicase DDX6	5231	0
XM_020088335.1	probable ATP-dependent RNA helicase DDX6	5216	0
XM_020088336.1	PREDICTED: trehalase	2144	0
XM_020088337.1	breast cancer type 2 susceptibility protein	9078	0
XM_020088338.1	2',3'-cyclic-nucleotide 3'-phosphodiesterase	1731	0
XM_020088339.1	collagen alpha-1(XXVI) chain-like isoform X1	3101	0
XM_020088340.1	collagen alpha-1(XXVI) chain-like isoform X2	3095	0
XM_020088341.1	collagen alpha-1(XXVI) chain-like isoform X3	3239	0
XM_020088342.1	collagen alpha-1(XXVI) chain-like isoform X4	3233	0
XM_020088343.1	WD repeat-containing protein 53	3024	0
XM_020088344.1	signal recognition particle receptor subunit beta	1881	0

XM_020088345.1	max-binding protein MNT-like	4159	0
XM_020088346.1	septin-5-like isoform X1	3417	0
XM_020088347.1	septin-4-like isoform X2	2951	0
XM_020088348.1	septin-4-like isoform X3	3007	0
XM_020088349.1	glioma tumor suppressor candidate region gene 1 protein isoform X1	7001	0
XM_020088350.1	glioma tumor suppressor candidate region gene 1 protein isoform X2	5822	0
XM_020088351.1	glioma tumor suppressor candidate region gene 1 protein isoform X3	6982	0
XM_020088352.1	glioma tumor suppressor candidate region gene 1 protein isoform X4	6701	0
XM_020088353.1	glioma tumor suppressor candidate region gene 1 protein isoform X4	6792	0
XM_020088354.1	sterile alpha and TIR motif-containing protein 1	3937	0
XM_020088355.1	DEAD (Asp-Glu-Ala-Asp) box helicase 5-2c	2442	0
XM_020088356.1	solute carrier family 13 member 2-like	2543	0
XM_020088357.1	proton-coupled folate transporter isoform X1	3280	0
XM_020088358.1	proton-coupled folate transporter isoform X1	1490	0
XM_020088359.1	protein C3orf33 homolog isoform X1	1928	0
XM_020088360.1	protein C3orf33 homolog isoform X2	1868	0
XM_020088361.1	protein C3orf33 homolog isoform X3	1814	0
XM_020088362.1	protein C3orf33 homolog isoform X4	1747	0
XM_020088363.1	protein C3orf33 homolog isoform X5	1451	1.78E-152
XM_020088364.1	nuclear pore complex protein Nup88	2505	0
XM_020088365.1	26S proteasome non-ATPase regulatory subunit 8	1546	3.09E-177
XM_020088366.1	rho guanine nucleotide exchange factor 17	11788	0
XM_020088367.1	amyloid beta A4 precursor protein-binding family B member 1 isoform X1	2471	0
XM_020088368.1	DNA-binding protein Ikaros isoform X1	2616	0
XM_020088369.1	amyloid beta A4 precursor protein-binding family B member 1 isoform X1	2359	0
XM_020088370.1	amyloid beta A4 precursor protein-binding family B member 1 isoform X1	4744	0
XM_020088371.1	amyloid beta A4 precursor protein-binding family B member 1 isoform X1	2357	0
XM_020088372.1	fibronectin type-III domain-containing protein 3A	6093	0
XM_020088373.1	interferon regulatory factor 2-binding protein 1-like	5053	0
XM_020088374.1	DNA-directed RNA polymerase I subunit RPA34-like	2233	2.43E-170
XM_020088375.1	rho guanine nucleotide exchange factor 12-like isoform X1	7066	0
XM_020088376.1	rho guanine nucleotide exchange factor 12-like isoform X2	7064	0
XM_020088377.1	DNA-binding protein Ikaros isoform X2	2613	0
XM_020088378.1	rho guanine nucleotide exchange factor 12-like isoform X3	7000	0
XM_020088379.1	rho guanine nucleotide exchange factor 12-like isoform X4	6963	0
XM_020088380.1	rho guanine nucleotide exchange factor 12-like isoform X5	6897	0
XM_020088381.1	rho guanine nucleotide exchange factor 12-like isoform X6	5533	0
XM_020088382.1	tyrosine-protein kinase receptor UFO	6049	0
XM_020088383.1	galectin-4-like isoform X5	1471	1.77E-103
XM_020088384.1	galectin-4-like isoform X5	1459	1.56E-103
XM_020088385.1	galectin-6-like isoform X3	1435	0
XM_020088386.1	galectin-4-like isoform X4	1408	0
XM_020088387.1	DNA-binding protein Ikaros isoform X3	2481	0
XM_020088388.1	galectin-4-like isoform X5	1381	0
XM_020088389.1	ribonucleoside-diphosphate reductase large subunit	2881	0
XM_020088390.1	stromal interaction molecule 1 isoform X1	4801	0
XM_020088391.1	stromal interaction molecule 1 isoform X2	2282	0
XM_020088392.1	stromal interaction molecule 1 isoform X3	2076	0
XM_020088393.1	B-cell CLL/lymphoma 9 protein-like	5029	0
XM_020088394.1	B-cell CLL/lymphoma 9 protein-like	5216	0
XM_020088395.1	C-X-C chemokine receptor type 5	1210	0
XM_020088396.1	DNA-binding protein Ikaros isoform X4	2346	0
XM_020088397.1	serine/threonine-protein phosphatase 2A 65 kDa regulatory subunit A beta isoform	3322	0
XM_020088398.1	coiled-coil domain-containing protein 92	4267	1.81E-151
XM_020088399.1	TLC domain-containing protein 2	3454	1.39E-166
XM_020088400.1	FRAS1-related extracellular matrix protein 2-like	11250	0
XM_020088401.1	S-adenosyl-L-methionine-dependent tRNA 4-demethylwyosine synthase	3509	0
XM_020088402.1	ribosome maturation protein SBDS	1215	2.36E-168
XM_020088403.1	DNA-binding protein Ikaros isoform X5	2343	0

XM_020088404.1	PREDICTED: claudin-4-like	2089	2.29E-133
XM_020088405.1	T-lymphoma invasion and metastasis-inducing protein 1-like	6346	0
XM_020088406.1	T-lymphoma invasion and metastasis-inducing protein 1-like	6236	0
XM_020088407.1	rho GTPase-activating protein 32-like isoform X1	6427	0
XM_020088408.1	rho GTPase-activating protein 32-like isoform X2	6423	0
XM_020088409.1	4F2 cell-surface antigen heavy chain-like	1767	0
XM_020088410.1	DNA-binding protein Ikaros isoform X6	2679	0
XM_020088411.1	reticulon-2-like isoform X1	2528	0
XM_020088412.1	reticulon-2-like isoform X2	2525	0
XM_020088413.1	reticulon-2-like isoform X3	1516	6.86E-120
XM_020088414.1	reticulon-2-like isoform X3	1403	5.85E-117
XM_020088415.1	protein phosphatase 1A-like	1215	0
XM_020088416.1	neutral amino acid transporter B(0)	2633	0
XM_020088417.1	rRNA methyltransferase 3, mitochondrial	1964	0
XM_020088418.1	DNA-binding protein Ikaros isoform X7	2409	0
XM_020088419.1	nucleoredoxin-like protein 2	2123	0
XM_020088420.1	phosphatidylinositol-binding clathrin assembly protein-like isoform X1	4182	0
XM_020088421.1	phosphatidylinositol-binding clathrin assembly protein-like isoform X2	4133	0
XM_020088422.1	phosphatidylinositol-binding clathrin assembly protein-like isoform X3	4074	0
XM_020088423.1	phosphatidylinositol-binding clathrin assembly protein-like isoform X4	4096	0

XM_020088424.1	phosphatidylinositol-binding clathrin assembly protein-like isoform X5	4026	0
XM_020088425.1	phosphatidylinositol-binding clathrin assembly protein-like isoform X6	4026	0
XM_020088426.1	phosphatidylinositol-binding clathrin assembly protein-like isoform X7	3940	0
XM_020088427.1	phosphatidylinositol-binding clathrin assembly protein-like isoform X8	3844	0
XM_020088428.1	transcriptional coactivator YAP1	4568	0
XM_020088429.1	PREDICTED: teneurin-4	11211	0
XM_020088430.1	vacuolar protein sorting-associated protein 26B-like	2403	0
XM_020088431.1	kynurenine 3-monooxygenase	1731	0
XM_020088432.1	junctional adhesion molecule C-like	1716	0
XM_020088433.1	nucleolar pre-ribosomal-associated protein 1 isoform X1	7249	0
XM_020088434.1	nucleolar pre-ribosomal-associated protein 1 isoform X2	7243	0
XM_020088435.1	protein NipSnap homolog 2	1897	0
XM_020088436.1	phosphoserine phosphatase isoform X1	1239	2.03E-170
XM_020088437.1	phosphoserine phosphatase isoform X2	1696	4.57E-162
XM_020088438.1	roundabout homolog 2 isoform X1	5407	0
XM_020088439.1	roundabout homolog 2 isoform X2	5380	0
XM_020088440.1	roundabout homolog 2 isoform X3	5352	0
XM_020088441.1	roundabout homolog 2 isoform X4	5973	0
XM_020088442.1	roundabout homolog 2 isoform X5	5281	0
XM_020088443.1	roundabout homolog 2 isoform X6	5224	0
XM_020088444.1	synaptic vesicular amine transporter	2665	0
XM_020088445.1	roundabout homolog 2 isoform X7	5254	0
XM_020088446.1	rho-related GTP-binding protein RhoU-like	2505	1.88E-138
XM_020088447.1	C2 domain-containing protein 3	6944	0
XM_020088448.1	glutaminyl-peptide cyclotransferase-like protein	2504	0
XM_020088449.1	U1 small nuclear ribonucleoprotein A	1312	6.41E-120
XM_020088450.1	double-strand break repair protein MRE11A	2645	0
XM_020088451.1	transmembrane protease serine 4-like isoform X2	2987	0
XM_020088452.1	transmembrane protease serine 4-like isoform X2	3073	0
XM_020088453.1	transmembrane protease serine 4-like isoform X2	2899	0
XM_020088454.1	transmembrane protease serine 4-like isoform X2	2835	0
XM_020088455.1	proline-rich protein 18	1850	5.14E-156
XM_020088456.1	transmembrane protease serine 4-like isoform X2	2879	0
XM_020088457.1	protein KIAA0100 homolog isoform X1	8060	0
XM_020088458.1	protein KIAA0100 homolog isoform X1	8057	0
XM_020088459.1	protein KIAA0100 homolog isoform X1	8024	0
XM_020088460.1	protein furry homolog isoform X1	10538	0
XM_020088461.1	protein furry homolog isoform X2	10523	0
XM_020088462.1	protein furry homolog isoform X3	9300	0
XM_020088463.1	protein furry homolog isoform X4	10393	0
XM_020088464.1	protein furry homolog isoform X5	10456	0
XM_020088465.1	protein furry homolog isoform X6	10229	0
XM_020088466.1	protein furry homolog isoform X7	10363	0
XM_020088467.1	dedicator of cytokinesis protein 10	7223	0
XM_020088468.1	tripartite motif-containing protein 3-like isoform X1	4406	0
XM_020088469.1	homeobox protein EMX2 isoform X1	2113	7.45E-139
XM_020088470.1	tripartite motif-containing protein 3-like isoform X1	4443	0
XM_020088471.1	tripartite motif-containing protein 3-like isoform X2	2508	0
XM_020088472.1	ras-related protein Rab-19-like	2159	1.52E-144
XM_020088473.1	mid1-interacting protein 1-B-like	728	6.39E-69
XM_020088474.1	NADH dehydrogenase [ubiquinone] 1 subunit C2-like	572	6.83E-80
XM_020088475.1	elongation factor G, mitochondrial	2773	0
XM_020088476.1	latexin isoform X1	1638	0
XM_020088477.1	latexin isoform X1	974	0
XM_020088478.1	homeobox protein EMX2 isoform X2	1928	9.49E-100
XM_020088479.1	mRNA decay activator protein ZFP36L1-like	3365	0
XM_020088480.1	egl nine homolog 1-like	3940	0
XM_020088481.1	egl nine homolog 1-like	3936	0
XM_020088482.1	roundabout homolog 1 isoform X1	6726	0
XM_020088483.1	roundabout homolog 1 isoform X2	6948	0
XM_020088484.1	roundabout homolog 1 isoform X1	6150	0
XM_020088485.1	solute carrier family 13 member 2-like	3084	0
XM_020088486.1	multidrug and toxin extrusion protein 1-like	2256	0
XM_020088487.1	homeobox protein SIX5-like	5945	0
XM_020088488.1	PREDICTED: nectin-1-like	5129	0
XM_020088489.1	amyloid-like protein 2 isoform X1	2946	0
XM_020088490.1	opioid growth factor receptor-like protein 1	4126	0
XM_020088491.1	amyloid-like protein 2 isoform X2	2943	0
XM_020088492.1	amyloid-like protein 2 isoform X3	2909	0
XM_020088493.1	amyloid-like protein 2 isoform X4	2907	0
XM_020088494.1	amyloid-like protein 2 isoform X5	2868	0
XM_020088495.1	amyloid-like protein 2 isoform X6	2832	0
XM_020088496.1	glycine receptor subunit alphaZ1-like	3301	2.60E-70
XM_020088497.1	amyloid-like protein 2 isoform X7	2778	0
XM_020088498.1	testis-expressed sequence 26 protein isoform X1	1222	0
XM_020088499.1	testis-expressed sequence 26 protein isoform X2	1385	0
XM_020088500.1	high mobility group-T protein-like	1150	4.90E-106
XM_020088501.1	TNF receptor-associated factor 2-like	4324	0
XM_020088502.1	CD166 antigen homolog A-like isoform X1	1780	0

XM_020088503.1	CD166 antigen homolog A-like isoform X2	1749	0
XM_020088504.1	insulin-like growth factor binding protein-2b	1175	4.60E-137
XM_020088505.1	vascular endothelial zinc finger 1 isoform X1	4365	0
XM_020088506.1	vascular endothelial zinc finger 1 isoform X2	4299	0
XM_020088507.1	vascular endothelial zinc finger 1 isoform X3	4257	0
XM_020088508.1	B-cell receptor-associated protein 29	2107	4.23E-144
XM_020088509.1	spectrin beta chain, non-erythrocytic 4	11749	0
XM_020088510.1	serine/threonine-protein kinase SIK2 isoform X1	4954	0
XM_020088511.1	serine/threonine-protein kinase SIK2 isoform X2	4951	0
XM_020088512.1	insulin-like growth factor-binding protein 2-A	1131	5.86E-137
XM_020088513.1	relA-associated inhibitor isoform X2	4551	0
XM_020088514.1	relA-associated inhibitor isoform X2	4706	0
XM_020088515.1	relA-associated inhibitor isoform X2	4578	0
XM_020088516.1	relA-associated inhibitor isoform X2	4744	0
XM_020088517.1	PR domain zinc finger protein 10	4532	0
XM_020088518.1	PR domain zinc finger protein 10	4654	0
XM_020088519.1	PR domain zinc finger protein 10	4911	0
XM_020088520.1	echinoderm microtubule-associated protein-like 2	4121	0
XM_020088521.1	ras-related protein Rap-2b	2037	1.89E-117
XM_020088522.1	integrin-linked protein kinase	3052	0
XM_020088523.1	14-3-3 protein beta/alpha-1	3611	1.15E-173
XM_020088524.1	52 kDa repressor of the inhibitor of the protein kinase-like	3703	0
XM_020088525.1	growth/differentiation factor 10-like	2752	0
XM_020088526.1	schwannomin-interacting protein 1 isoform X1	2647	0
XM_020088527.1	schwannomin-interacting protein 1 isoform X2	2283	0
XM_020088528.1	schwannomin-interacting protein 1 isoform X3	2546	0
XM_020088529.1	schwannomin-interacting protein 1 isoform X4	1518	2.52E-129
XM_020088530.1	schwannomin-interacting protein 1 isoform X5	1531	1.04E-114
XM_020088531.1	ras-related protein Rab-4A	3525	3.44E-148
XM_020088532.1	ras-related protein Rab-4A	3647	4.92E-147
XM_020088533.1	inositol-trisphosphate 3-kinase C-like	3634	0
XM_020088534.1	RCC1 and BTB domain-containing protein 2	3225	0
XM_020088535.1	RCC1 and BTB domain-containing protein 2	3248	0
XM_020088536.1	RCC1 and BTB domain-containing protein 2	2989	0
XM_020088537.1	carbohydrate sulfotransferase 15-like	4320	0
XM_020088538.1	ubiquitin conjugation factor E4 A	5317	0
XM_020088539.1	serine/threonine-protein phosphatase 5	2604	0
XM_020088540.1	TLC domain-containing protein 2	1927	0
XM_020088541.1	1-phosphatidylinositol 4,5-bisphosphate phosphodiesterase eta-1 isoform X3	6905	0
XM_020088542.1	1-phosphatidylinositol 4,5-bisphosphate phosphodiesterase eta-1 isoform X2	5999	0
XM_020088543.1	1-phosphatidylinositol 4,5-bisphosphate phosphodiesterase eta-1 isoform X3	6884	0
XM_020088544.1	1-phosphatidylinositol 4,5-bisphosphate phosphodiesterase eta-1 isoform X4	7074	0
XM_020088545.1	1-phosphatidylinositol 4,5-bisphosphate phosphodiesterase eta-1 isoform X2	3461	0
XM_020088546.1	neprilysin isoform X1	5384	0
XM_020088547.1	neprilysin isoform X1	5099	0
XM_020088548.1	biogenesis of lysosome-related organelles complex 1 subunit 3	1146	2.52E-122
XM_020088549.1	trafficking protein particle complex subunit 6B-like	1880	6.43E-115
XM_020088550.1	protein very KIND	7540	0
XM_020088551.1	trafficking protein particle complex subunit 6B-like	1683	1.25E-115
XM_020088552.1	trafficking protein particle complex subunit 6B-like	1783	1.51E-115
XM_020088553.1	trafficking protein particle complex subunit 6B-like	1826	3.82E-115
XM_020088554.1	trafficking protein particle complex subunit 6B-like	1668	8.10E-116
XM_020088555.1	pre-rRNA-processing protein TSR1 homolog	2725	0
XM_020088556.1	mRNA decay activator protein ZFP36L1-like	2434	0
XM_020088557.1	forkhead box protein A1-like	2747	0
XM_020088558.1	PREDICTED: uncharacterized protein LOC109630378 isoform X1	3895	0
XM_020088559.1	PREDICTED: uncharacterized protein LOC109630378 isoform X2	3892	0
XM_020088560.1	interleukin-12 p35	1255	1.08E-138
XM_020088561.1	apoptosis-inducing factor 3-like	2566	0
XM_020088562.1	phosphomethylethanolamine N-methyltransferase-like isoform X2	1595	0
XM_020088563.1	transmembrane protein 121-like	1162	0
XM_020088564.1	out at first protein homolog	2857	0
XM_020088565.1	high affinity cationic amino acid transporter 1-like	3581	0
XM_020088566.1	phospholipase D3	1966	0
XM_020088567.1	GRB2-associated-binding protein 2-like	1157	5.07E-150
XM_020088568.1	GRB2-associated-binding protein 2-like isoform X1	2678	2.96E-83
XM_020088569.1	GRB2-associated-binding protein 2-like isoform X2	2675	1.49E-82
XM_020088570.1	alpha-soluble NSF attachment protein	2185	0
XM_020088571.1	methyltransferase-like protein 16 isoform X2	2171	0
XM_020088572.1	methyltransferase-like protein 16 isoform X2	2194	0
XM_020088573.1	methyltransferase-like protein 16 isoform X2	2163	0
XM_020088574.1	methyltransferase-like protein 16 isoform X2	2174	0
XM_020088575.1	methyltransferase-like protein 16 isoform X2	2245	0
XM_020088576.1	forkhead box protein O1-A-like	4475	0
XM_020088577.1	forkhead box protein O1-A-like	4296	0
XM_020088578.1	protein phosphatase 1L	1816	0
XM_020088579.1	ADP-ribosylation factor-like protein 14	1020	1.13E-152
XM_020088580.1	roundabout homolog 2-like isoform X1	5141	0
XM_020088581.1	roundabout homolog 2-like isoform X2	5129	0

XM_020088582.1	roundabout homolog 2-like isoform X3	5126	0
XM_020088583.1	roundabout homolog 2-like isoform X4	5123	0
XM_020088584.1	roundabout homolog 2-like isoform X5	5114	0
XM_020088585.1	roundabout homolog 2-like isoform X6	5108	0
XM_020088586.1	roundabout homolog 2-like isoform X7	5111	0
XM_020088587.1	potassium channel subfamily K member 18	3608	0
XM_020088588.1	P2Y purinoceptor 2	3430	0
XM_020088589.1	P2Y purinoceptor 2	3418	0
XM_020088590.1	protein-tyrosine sulfotransferase 1 isoform X1	2030	0
XM_020088591.1	protein-tyrosine sulfotransferase 1 isoform X1	2025	0
XM_020088592.1	protein-tyrosine sulfotransferase 1 isoform X2	1975	0
XM_020088593.1	protein-tyrosine sulfotransferase 1 isoform X2	1970	0
XM_020088594.1	RING finger protein 26-like	3271	0
XM_020088595.1	tubulin-folding cofactor B	2844	1.95E-177
XM_020088596.1	diphthamide biosynthesis protein 2	2958	0
XM_020088597.1	2-oxoisovalerate dehydrogenase subunit alpha, mitochondrial	2034	0
XM_020088598.1	calcium/calmodulin-dependent protein kinase kinase 1-like	1341	0
XM_020088599.1	E3 ubiquitin-protein ligase NEURL1-like	4385	0
XM_020088600.1	mitochondrial uncoupling protein 2-like	2835	0
XM_020088601.1	leucine-rich repeat and fibronectin type III domain-containing protein 1	5644	0
XM_020088602.1	leucine-rich repeat and fibronectin type III domain-containing protein 1	5464	0
XM_020088603.1	leucine-rich repeat and fibronectin type III domain-containing protein 1	5711	0
XM_020088604.1	glutamate receptor 4 isoform X1	5700	0
XM_020088605.1	glutamate receptor 4 isoform X1	5700	0
XM_020088606.1	muscleblind-like protein 1	2231	1.93E-171
XM_020088607.1	muscleblind-like protein 1	2097	4.60E-172
XM_020088608.1	splicing factor, arginine/serine-rich 15-like isoform X2	3052	0
XM_020088609.1	splicing factor, arginine/serine-rich 15-like isoform X1	3049	0
XM_020088610.1	potassium channel subfamily K member 6-like	2358	0
XM_020088611.1	muscular LMNA-interacting protein isoform X1	3786	0
XM_020088612.1	transforming growth factor beta-1-like	4048	0
XM_020088613.1	synapse differentiation-inducing gene protein 1-like	3731	2.34E-140
XM_020088614.1	exosome complex component RRP46	930	1.95E-164
XM_020088615.1	ATP-dependent RNA helicase DHX36	3838	0
XM_020088616.1	probable ATP-dependent RNA helicase DHX34	4088	0
XM_020088617.1	BTB/POZ domain-containing adapter for CUL3-mediated RhoA degradation protein 2	2104	0
XM_020088618.1	BTB/POZ domain-containing adapter for CUL3-mediated RhoA degradation protein 2	2111	0
XM_020088619.1	mitogen-activated protein kinase kinase kinase 10	6837	0
XM_020088620.1	protein moonraker isoform X1	3781	0
XM_020088621.1	protein moonraker isoform X2	3777	0
XM_020088622.1	protein moonraker isoform X3	3778	0
XM_020088623.1	muscular LMNA-interacting protein isoform X2	2358	0
XM_020088624.1	protein moonraker isoform X4	3779	0
XM_020088625.1	lysosomal Pro-X carboxypeptidase	2484	0
XM_020088626.1	membrane-associated phosphatidylinositol transfer protein 3 isoform X1	6800	0
XM_020088627.1	membrane-associated phosphatidylinositol transfer protein 3 isoform X1	5622	0
XM_020088628.1	membrane-associated phosphatidylinositol transfer protein 3 isoform X1	6797	0
XM_020088629.1	membrane-associated phosphatidylinositol transfer protein 3 isoform X1	5771	0
XM_020088630.1	PREDICTED: uncharacterized protein C19orf47 homolog isoform X1	2563	0
XM_020088631.1	PREDICTED: uncharacterized protein C19orf47 homolog isoform X2	2586	0
XM_020088632.1	PREDICTED: uncharacterized protein C19orf47 homolog isoform X3	2486	0
XM_020088633.1	PREDICTED: claudin-3-like	1068	2.16E-150
XM_020088634.1	1,4-alpha-glucan-branching enzyme	3140	0
XM_020088635.1	lysophosphatidic acid receptor 6-like	2448	0
XM_020088636.1	neuronal PAS domain-containing protein 4-like	3148	0
XM_020088637.1	major facilitator superfamily domain-containing protein 1 isoform X1	2111	0
XM_020088638.1	major facilitator superfamily domain-containing protein 1 isoform X1	1885	0
XM_020088639.1	major facilitator superfamily domain-containing protein 1 isoform X3	1998	0
XM_020088640.1	F-box only protein 40	2143	0
XM_020088641.1	tetratricopeptide repeat protein 36	1114	2.63E-107
XM_020088642.1	SERTA domain-containing protein 2-like isoform X1	3497	0
XM_020088643.1	SERTA domain-containing protein 2-like isoform X1	3352	0
XM_020088644.1	SERTA domain-containing protein 2-like isoform X1	3385	5.54E-180
XM_020088645.1	SERTA domain-containing protein 2-like isoform X1	3382	2.81E-179
XM_020088646.1	myb-like protein X	4975	0
XM_020088647.1	elastin-like isoform X4	5123	1.99E-38
XM_020088648.1	elastin-like isoform X4	5119	1.98E-38
XM_020088649.1	leucine-rich repeat-containing protein 18	904	2.63E-176
XM_020088650.1	elastin-like isoform X4	5120	2.44E-38
XM_020088651.1	elastin-like isoform X4	5093	1.42E-38
XM_020088652.1	elastin-like isoform X4	5087	2.43E-38
XM_020088653.1	elastin-like isoform X4	5084	2.85E-38
XM_020088654.1	elastin-like isoform X7	5078	6.91E-36
XM_020088655.1	elastin-like isoform X9	5072	3.44E-27
XM_020088656.1	elastin-like isoform X9	5042	3.38E-28
XM_020088657.1	elastin-like isoform X10	5027	1.25E-25
XM_020088658.1	elastin-like isoform X4	5024	1.92E-38
XM_020088659.1	elastin-like isoform X4	4970	1.89E-38
XM_020088660.1	elastin-like isoform X4	4871	1.82E-38

XM_020088661.1	elastin-like isoform X5	4712	1.71E-38
XM_020088662.1	elastin-like isoform X4	4418	7.19E-39
XM_020088663.1	RNA polymerase II-associated factor 1 homolog isoform X1	2110	0
XM_020088664.1	latent-transforming growth factor beta-binding protein 1	6405	0
XM_020088665.1	RNA polymerase II-associated factor 1 homolog isoform X2	1834	0
XM_020088666.1	protein spinster homolog 1-like	2116	0
XM_020088667.1	PREDICTED: claudin-3-like	1301	1.53E-118
XM_020088668.1	hepatocyte nuclear factor 1-beta isoform X1	2221	0
XM_020088669.1	hepatocyte nuclear factor 1-beta isoform X2	1916	0
XM_020088670.1	CWF19-like protein 2	3119	0
XM_020088671.1	protein YIF1A	2157	0
XM_020088672.1	protein YIF1A	2148	0
XM_020088673.1	meiosis-specific nuclear structural protein 1-like	867	4.19E-168
XM_020088674.1	coiled-coil domain-containing protein 85C-like	1449	0
XM_020088675.1	nephrocystin-4 isoform X1	4634	0
XM_020088676.1	nephrocystin-4 isoform X2	4628	0
XM_020088677.1	band 4.1-like protein 1 isoform X1	5633	0
XM_020088678.1	band 4.1-like protein 1 isoform X2	5630	0
XM_020088679.1	AMP deaminase 1 isoform X1	2645	0
XM_020088680.1	AMP deaminase 1 isoform X2	2622	0
XM_020088681.1	rho GTPase-activating protein 4	3278	0
XM_020088682.1	rho GTPase-activating protein 4	3204	0
XM_020088683.1	rho GTPase-activating protein 4	3303	0
XM_020088684.1	peptidyl-prolyl cis-trans isomerase FKBP1A-like	1882	1.71E-71
XM_020088685.1	actin-related protein 8	2443	0
XM_020088686.1	sodium-dependent neutral amino acid transporter SLC6A17 isoform X1	6303	0
XM_020088687.1	intraflagellar transport protein 80 homolog	1947	0
XM_020088688.1	sodium-dependent neutral amino acid transporter SLC6A17 isoform X1	6584	0
XM_020088689.1	sodium-dependent neutral amino acid transporter SLC6A17 isoform X1	1202	0
XM_020088690.1	ATP-dependent 6-phosphofructokinase, muscle type-like	2605	0
XM_020088691.1	acylamino-acid-releasing enzyme-like	2488	0
XM_020088692.1	E3 ubiquitin-protein ligase Midline-1-like	4010	0
XM_020088693.1	E3 ubiquitin-protein ligase Midline-1-like	4004	0
XM_020088694.1	E3 ubiquitin-protein ligase Midline-1-like	4007	0
XM_020088695.1	E3 ubiquitin-protein ligase Midline-1-like	3934	0
XM_020088696.1	twist-related protein 2-like	1011	1.47E-131
XM_020088697.1	suppressor of IKBKE 1	1878	3.14E-152
XM_020088698.1	zinc finger protein Eos-like isoform X2	3953	0
XM_020088699.1	zinc finger protein Eos-like isoform X2	3882	0
XM_020088700.1	B-cell lymphoma/leukemia 11A-like	6203	0
XM_020088701.1	zinc finger protein Eos-like isoform X2	3845	0
XM_020088702.1	dnaJ homolog subfamily C member 16	1407	0
XM_020088703.1	kinesin-like protein KIF3B	2033	2.06E-35
XM_020088704.1	transcription factor GATA-5-like	2702	0
XM_020088705.1	transcription factor GATA-5-like	2697	0
XM_020088706.1	transcription factor GATA-5-like	2479	0
XM_020088707.1	transcription factor GATA-5-like	2509	0
XM_020088708.1	zinc finger and BTB domain-containing protein 17	3437	0
XM_020088709.1	zinc finger protein 385A-like	4016	5.39E-97
XM_020088710.1	TOX high mobility group box family member 2-like isoform X1	3387	0
XM_020088711.1	TOX high mobility group box family member 2-like isoform X2	1469	0
XM_020088712.1	thymocyte selection-associated high mobility group box protein TOX-like isoform X3	2774	0
XM_020088713.1	low molecular weight neuronal intermediate filament-like isoform X2	1263	0
XM_020088714.1	AP-1 complex subunit beta-1	3067	0
XM_020088715.1	tastin isoform X1	2660	0
XM_020088716.1	tastin isoform X2	2763	0
XM_020088717.1	tastin isoform X3	2534	0
XM_020088718.1	gamma-aminobutyric acid receptor subunit delta isoform X1	2728	0
XM_020088719.1	gamma-aminobutyric acid receptor subunit delta isoform X2	2643	0
XM_020088720.1	translocon-associated protein subunit delta	1109	7.64E-121
XM_020088721.1	probable G-protein coupled receptor 153	4735	0
XM_020088722.1	ubiA prenyltransferase domain-containing protein 1	1750	0
XM_020088723.1	60S ribosomal protein L22	539	1.13E-52
XM_020088724.1	N-alpha-acetyltransferase 10	1211	7.25E-149
XM_020088725.1	homeobox protein OTX2-B-like	1157	0
XM_020088726.1	pre-mRNA-splicing factor SPF27	1373	3.91E-152
XM_020088727.1	charged multivesicular body protein 4b-like	985	4.82E-104
XM_020088728.1	PREDICTED: uncharacterized protein LOC109630499	3099	0
XM_020088729.1	PREDICTED: uncharacterized protein LOC109630499	3027	0
XM_020088730.1	transmembrane protein 9	2014	9.93E-122
XM_020088731.1	transmembrane protein 9	2037	3.15E-121

XM_020088732.1	succinate dehydrogenase [ubiquinone] iron-sulfur subunit, mitochondrial	1355	0
XM_020088733.1	phosphomethylethanolamine N-methyltransferase-like isoform X2	1674	0
XM_020088734.1	dynein heavy chain 12, axonemal	13031	0
XM_020088735.1	prostaglandin E synthase 3-like	1627	5.33E-103
XM_020088736.1	targeting protein for Xklp2 isoform X1	906	5.58E-95
XM_020088737.1	targeting protein for Xklp2 isoform X1	888	7.49E-91
XM_020088738.1	serine/threonine-protein kinase 32C	6020	0
XM_020088739.1	Krueppel-like factor 15 isoform X1	5062	4.30E-172

XM_020088740.1	Krueppel-like factor 15 isoform X2	2003	6.96E-161
XM_020088741.1	Krueppel-like factor 15 isoform X3	2505	8.43E-142
XM_020088742.1	PREDICTED: contactin-4-like	5082	0
XM_020088743.1	UPF0577 protein KIAA1324 homolog	3471	0
XM_020088744.1	phosphatidylinositol 4,5-bisphosphate 3-kinase catalytic subunit delta isoform isoform X1	5662	0
XM_020088745.1	phosphatidylinositol 4,5-bisphosphate 3-kinase catalytic subunit delta isoform isoform X1	5657	0
XM_020088746.1	phosphatidylinositol 4,5-bisphosphate 3-kinase catalytic subunit delta isoform isoform X1	5659	0
XM_020088747.1	mitotic spindle assembly checkpoint protein MAD2B	1799	9.75E-151
XM_020088748.1	actin-related protein 5	2257	0
XM_020088749.1	rho guanine nucleotide exchange factor 5-like isoform X3	3889	0
XM_020088750.1	cilia- and flagella-associated protein 46	7641	0
XM_020088751.1	pleckstrin homology domain-containing family N member 1 isoform X2	3558	0
XM_020088752.1	pleckstrin homology domain-containing family N member 1 isoform X2	3492	0
XM_020088753.1	protein bassoon-like	14631	0
XM_020088754.1	protein spinster homolog 3-like	781	1.82E-137
XM_020088755.1	transcription initiation factor TFIID subunit 10	1080	7.26E-115
XM_020088756.1	potassium voltage-gated channel subfamily KQT member 2-like isoform X1	4489	0
XM_020088757.1	potassium voltage-gated channel subfamily KQT member 2-like isoform X2	4471	0
XM_020088758.1	potassium voltage-gated channel subfamily KQT member 2-like isoform X3	4459	0
XM_020088759.1	potassium voltage-gated channel subfamily KQT member 2-like isoform X4	4435	0
XM_020088760.1	potassium voltage-gated channel subfamily KQT member 2-like isoform X5	4417	0
XM_020088761.1	potassium voltage-gated channel subfamily KQT member 2-like isoform X6	4405	0
XM_020088762.1	potassium voltage-gated channel subfamily KQT member 2-like isoform X7	4399	0
XM_020088763.1	potassium voltage-gated channel subfamily KQT member 2-like isoform X8	4387	0
XM_020088764.1	zinc finger MYND domain-containing protein 10-like	885	0
XM_020088765.1	potassium voltage-gated channel subfamily KQT member 2-like isoform X9	4381	0
XM_020088766.1	potassium voltage-gated channel subfamily KQT member 2-like isoform X10	4345	0
XM_020088767.1	potassium voltage-gated channel subfamily KQT member 2-like isoform X11	4327	0
XM_020088768.1	potassium voltage-gated channel subfamily KQT member 2-like isoform X12	4414	0
XM_020088769.1	tumor protein p63-regulated gene 1-like protein	2827	0
XM_020088770.1	piezo-type mechanosensitive ion channel component 2-like	8450	0
XM_020088771.1	ATP synthase subunit epsilon, mitochondrial	471	9.18E-30
XM_020088772.1	probable E3 ubiquitin-protein ligase makorin-2 isoform X1	1926	0
XM_020088773.1	E3 ubiquitin-protein ligase makorin-1-like isoform X2	1923	0
XM_020088774.1	vasopressin V2 receptor-like isoform X1	2710	0
XM_020088775.1	vasopressin V2 receptor-like isoform X2	2464	0
XM_020088776.1	5-aminolevulinate synthase, erythroid-specific, mitochondrial-like	2392	0
XM_020088777.1	DNA excision repair protein ERCC-6	5193	0
XM_020088778.1	carbohydrate sulfotransferase 11-like isoform X1	3636	0
XM_020088779.1	carbohydrate sulfotransferase 11-like isoform X2	3633	0
XM_020088780.1	ataxin-7-like protein 2	2500	0
XM_020088781.1	transcription factor Sp5-like	2167	0
XM_020088782.1	lethal(3)malignant brain tumor-like protein 1 isoform X1	3942	0
XM_020088783.1	lethal(3)malignant brain tumor-like protein 1 isoform X2	3483	0
XM_020088784.1	synaptonemal complex protein 2 isoform X1	5209	0
XM_020088785.1	synaptonemal complex protein 2 isoform X2	5206	0
XM_020088786.1	synaptonemal complex protein 2 isoform X3	5206	0
XM_020088787.1	cytochrome c oxidase subunit 4 isoform 1, mitochondrial	1070	1.60E-104
XM_020088788.1	beta-1,4 N-acetylgalactosaminyltransferase 1-like isoform X1	2712	0
XM_020088789.1	beta-1,4 N-acetylgalactosaminyltransferase 1-like isoform X2	2697	0
XM_020088790.1	beta-1,4 N-acetylgalactosaminyltransferase 1-like isoform X3	2694	0
XM_020088791.1	N-terminal EF-hand calcium-binding protein 1-like isoform X1	2420	0
XM_020088792.1	N-terminal EF-hand calcium-binding protein 1-like isoform X1	2290	0
XM_020088793.1	differentially expressed in FDCP 6 homolog isoform X1	3136	0
XM_020088794.1	differentially expressed in FDCP 6 homolog isoform X2	1860	0
XM_020088795.1	gap junction gamma-1 protein-like	3327	0
XM_020088796.1	gap junction gamma-1 protein-like	3359	0
XM_020088797.1	N-acetyltransferase 6-like	2064	0
XM_020088798.1	vesicular inhibitory amino acid transporter-like	2919	0
XM_020088799.1	protein FAM19A5-like isoform X1	3004	2.00E-69
XM_020088800.1	protein FAM19A3-like isoform X2	1116	5.27E-71
XM_020088801.1	polycystin-2 isoform X1	2340	0
XM_020088802.1	adherens junction-associated protein 1	7063	0
XM_020088803.1	RBBP8 N-terminal-like protein isoform X1	4294	0
XM_020088804.1	RBBP8 N-terminal-like protein isoform X2	4291	0
XM_020088805.1	RBBP8 N-terminal-like protein isoform X3	4291	0
XM_020088806.1	RBBP8 N-terminal-like protein isoform X4	4177	0
XM_020088807.1	lipoma HMGIC fusion partner-like 4 protein	6470	5.10E-151
XM_020088808.1	zinc finger protein PLAGL2-like	4410	0
XM_020088809.1	nuclear factor 7, ovary-like	2341	0
XM_020088810.1	transmembrane protein 240	2421	3.49E-94
XM_020088811.1	ADP-ribosylation factor 3	509	4.55E-74
XM_020088812.1	voltage-dependent L-type calcium channel subunit alpha-1S	5457	0
XM_020088813.1	protocadherin Fat 1 isoform X3	11693	0
XM_020088814.1	neural cell adhesion molecule L1	6114	0
XM_020088815.1	serine/threonine-protein kinase WNK1-like	6526	0
XM_020088816.1	signal transducer and activator of transcription 5B-like	3542	0
XM_020088817.1	protein FAM107B-like	697	1.23E-93
XM_020088818.1	phosphomethylethanolamine N-methyltransferase-like isoform X2	1652	0

XM_020088819.1	ragulator complex protein LAMTOR5	552	2.05E-39
XM_020088820.1	glucocorticoid modulatory element-binding protein 1-like	975	0
XM_020088821.1	nucleolar complex protein 2 homolog	2208	0
XM_020088822.1	nicotinamide/nicotinic acid mononucleotide adenylyltransferase 1	1876	0
XM_020088823.1	GPI transamidase component PIG-T	2630	0
XM_020088824.1	fibroblast growth factor receptor 2-like	4546	0
XM_020088825.1	large neutral amino acids transporter small subunit 1-like	2875	0
XM_020088826.1	tetratricopeptide repeat protein 34	3412	0
XM_020088827.1	chymotrypsin-like elastase family member 2A	965	1.20E-178
XM_020088828.1	taste receptor type 1 member 1-like	4053	0
XM_020088829.1	PREDICTED: uncharacterized protein LOC109630565	1687	0
XM_020088830.1	E3 ubiquitin-protein ligase UBR4	16637	0
XM_020088831.1	taste receptor type 1 member 1-like	2628	0
XM_020088832.1	serine/threonine-protein kinase Sgk2	1186	0
XM_020088833.1	transketolase-like isoform X1	2006	0
XM_020088834.1	voltage-dependent L-type calcium channel subunit alpha-1D-like	969	2.00E-129
XM_020088835.1	probable E3 ubiquitin-protein ligase TRIML1	1116	0
XM_020088836.1	protein NLRC3-like	3364	0
XM_020088837.1	aminomethyltransferase, mitochondrial	2020	0
XM_020088838.1	hyaluronidase-1-like isoform X1	1089	0
XM_020088839.1	DALR anticodon-binding domain-containing protein 3	1669	0
XM_020088840.1	PREDICTED: hyaluronidase-2-like	1701	0
XM_020088841.1	PREDICTED: semaphorin-3A-like	7438	0
XM_020088842.1	transmembrane protein 26-like	1236	0
XM_020088843.1	PREDICTED: uncharacterized protein LOC109630580	2248	0
XM_020088844.1	chloride channel protein ClC-Ka	1998	0
XM_020088845.1	serine/threonine-protein kinase mTOR	4483	0
XM_020088846.1	spectrin alpha chain, non-erythrocytic 1-like	2700	0
XM_020088847.1	tyrosine-protein phosphatase non-receptor type 11-like	4829	0
XM_020088848.1	suppression of tumorigenicity 5 protein	3795	0
XM_020088849.1	neuronal acetylcholine receptor subunit alpha-2-like	1854	0
XM_020088850.1	V-set and transmembrane domain-containing protein 2-like protein	3897	3.64E-85
XM_020088851.1	beta-catenin-like protein 1	1147	7.16E-127
XM_020088852.1	protein-glutamine gamma-glutamyltransferase 5-like	3171	0
XM_020088853.1	transcription factor E2F1-like	840	0
XM_020088854.1	zinc finger protein 341-like	2714	0
XM_020088855.1	mitogen-activated protein kinase 14B-like	1695	0
XM_020088856.1	serum response factor-like	1209	1.97E-115
XM_020088857.1	protein FAM83A-like	1540	0
XM_020088858.1	olfactory receptor 2G3-like	1167	0
XM_020088859.1	solute carrier organic anion transporter family member 5A1	4121	0
XM_020088860.1	nuclear factor 7, ovary-like	1542	0
XM_020088861.1	transmembrane 9 superfamily member 4	4708	0
XM_020088862.1	kinesin-like protein KIF3B	2456	0
XM_020088863.1	dual specificity tyrosine-phosphorylation-regulated kinase 4-like	2136	0
XM_020088864.1	alpha-1,3-mannosyl-glycoprotein 4-beta-N-acetylglucosaminyltransferase C	1779	0
XM_020088865.1	protein Flattop	789	4.73E-147
XM_020088866.1	PREDICTED: LOW QUALITY PROTEIN: uncharacterized protein LOC109630604	1458	0
XM_020088867.1	lysosomal-trafficking regulator	10100	0
XM_020088868.1	atrial natriuretic peptide receptor 1-like	2034	0
XM_020088869.1	regulator of telomere elongation helicase 1	3345	0
XM_020088870.1	voltage-gated potassium channel subunit beta-1-like	1034	0
XM_020088871.1	protein FAM107B-like	420	1.37E-77
XM_020088872.1	organic solute transporter subunit alpha-like	957	0
XM_020088873.1	putative beta-lactamase-like 1	1674	0
XM_020088874.1	ephrin type-B receptor 2-like	1037	0
XM_020088875.1	PR domain zinc finger protein 2-like	5192	0
XM_020088876.1	PR domain zinc finger protein 16	7290	0
XM_020088877.1	interferon-induced, double-stranded RNA-activated protein kinase-like	518	2.21E-80
XM_020088878.1	coiled-coil domain-containing protein 30 isoform X1	5761	0
XM_020088879.1	coiled-coil domain-containing protein 30 isoform X1	5677	0
XM_020088880.1	coiled-coil domain-containing protein 30 isoform X1	6020	0
XM_020088881.1	coiled-coil domain-containing protein 30 isoform X1	6005	0
XM_020088882.1	coiled-coil domain-containing protein 30 isoform X1	6002	0
XM_020088883.1	coiled-coil domain-containing protein 30 isoform X1	5951	0
XM_020088884.1	coiled-coil domain-containing protein 30 isoform X1	5945	0
XM_020088885.1	coiled-coil domain-containing protein 30 isoform X7	5873	0
XM_020088886.1	kazrin isoform X1	4409	0
XM_020088887.1	kazrin isoform X2	4407	0
XM_020088888.1	kazrin isoform X3	4397	0
XM_020088889.1	kazrin isoform X4	4323	0
XM_020088890.1	kazrin isoform X5	4322	0
XM_020088891.1	kazrin isoform X6	2765	0
XM_020088892.1	kazrin isoform X7	3806	0
XM_020088893.1	phosphomethylethanolamine N-methyltransferase-like isoform X2	1743	0
XM_020088894.1	macrophage mannose receptor 1-like	1195	7.86E-179
XM_020088895.1	kazrin isoform X8	4171	0
XM_020088896.1	forkhead box protein J3-like isoform X1	2606	0
XM_020088897.1	forkhead box protein J3-like isoform X1	2561	0

XM_020088898.1	forkhead box protein J3-like isoform X1	4316	0
XM_020088899.1	forkhead box protein J3-like isoform X1	2474	0
XM_020088900.1	N-acetylaspartylglutamate synthase A isoform X1	2138	0
XM_020088901.1	N-acetylaspartylglutamate synthase A isoform X2	2156	0
XM_020088902.1	N-acetylaspartylglutamate synthase A isoform X3	2252	0
XM_020088903.1	N-acetylaspartylglutamate synthase A isoform X4	1864	0
XM_020088904.1	zinc finger protein 362-like isoform X1	4628	0
XM_020088905.1	zinc finger protein 362-like isoform X1	4602	0
XM_020088906.1	tumor necrosis factor receptor superfamily member 1A-like isoform X1	2964	0
XM_020088907.1	tumor necrosis factor receptor superfamily member 1A-like isoform X1	2881	0
XM_020088908.1	fidgetin-like protein 1	1608	0
XM_020088909.1	zinc finger MYND domain-containing protein 12	1220	0
XM_020088910.1	transmembrane protein 51-like	3773	5.13E-122
XM_020088911.1	chymotrypsin-like elastase family member 2A	888	0
XM_020088912.1	chymotrypsin-like elastase family member 2A	905	0
XM_020088913.1	PREDICTED: uncharacterized protein LOC109630629	2700	5.01E-41
XM_020088914.1	epithelial membrane protein 3-like	1015	2.42E-97
XM_020088915.1	epithelial membrane protein 3-like	1018	2.50E-97
XM_020088916.1	ATP synthase F(0) complex subunit B1, mitochondrial	1051	3.12E-160
XM_020088917.1	E3 ubiquitin-protein ligase HUWE1 isoform X1	14825	0
XM_020088918.1	E3 ubiquitin-protein ligase HUWE1 isoform X1	14850	0
XM_020088919.1	E3 ubiquitin-protein ligase HUWE1 isoform X1	14798	0
XM_020088920.1	protein jagged-1	5338	0
XM_020088921.1	E3 ubiquitin-protein ligase HUWE1 isoform X1	14695	0
XM_020088922.1	E3 ubiquitin-protein ligase HUWE1 isoform X1	14979	0
XM_020088923.1	E3 ubiquitin-protein ligase HUWE1 isoform X1	14674	0
XM_020088924.1	E3 ubiquitin-protein ligase HUWE1 isoform X1	14822	0
XM_020088925.1	E3 ubiquitin-protein ligase HUWE1 isoform X1	14813	0
XM_020088926.1	E3 ubiquitin-protein ligase HUWE1 isoform X1	14813	0
XM_020088927.1	E3 ubiquitin-protein ligase HUWE1 isoform X1	14807	0
XM_020088928.1	E3 ubiquitin-protein ligase HUWE1 isoform X1	14804	0
XM_020088929.1	E3 ubiquitin-protein ligase HUWE1 isoform X1	14804	0
XM_020088930.1	E3 ubiquitin-protein ligase HUWE1 isoform X1	14804	0
XM_020088931.1	E3 ubiquitin-protein ligase HUWE1 isoform X9	14804	0
XM_020088932.1	E3 ubiquitin-protein ligase HUWE1 isoform X1	14792	0
XM_020088933.1	E3 ubiquitin-protein ligase HUWE1 isoform X1	14775	0
XM_020088934.1	E3 ubiquitin-protein ligase HUWE1 isoform X12	14738	0
XM_020088935.1	E3 ubiquitin-protein ligase HUWE1 isoform X13	14762	0
XM_020088936.1	ribosome-binding protein 1	3857	0
XM_020088937.1	histone lysine demethylase PHF8	5900	0
XM_020088938.1	small nuclear ribonucleoprotein G	556	3.20E-49
XM_020088939.1	neurogenic differentiation factor 4-like	1997	1.85E-171
XM_020088940.1	transcription factor HES-5-like	1286	1.34E-97
XM_020088941.1	cytosolic acyl coenzyme A thioester hydrolase isoform X1	1707	0
XM_020088942.1	cytosolic acyl coenzyme A thioester hydrolase isoform X2	1691	0
XM_020088943.1	Fanconi anemia group E protein	1852	0
XM_020088944.1	selenoprotein K	879	1.18E-34
XM_020088945.1	troponin C, slow skeletal and cardiac muscles-like	807	2.90E-83
XM_020088946.1	emerin-like isoform X1	1260	2.44E-89
XM_020088947.1	calpain-1 catalytic subunit-like	2079	0
XM_020088948.1	synaptophysin-like protein 1	1189	7.47E-163
XM_020088949.1	OTU domain-containing protein 3 isoform X1	1539	0
XM_020088950.1	OTU domain-containing protein 3 isoform X2	1503	0
XM_020088951.1	probable E3 ubiquitin-protein ligase DTX3 isoform X1	1132	3.33E-139
XM_020088952.1	lipopolysaccharide-binding protein-like isoform X2	1703	0
XM_020088953.1	lipopolysaccharide-binding protein-like isoform X2	1781	0
XM_020088954.1	MICOS complex subunit mic25a-like isoform X1	1498	9.73E-153
XM_020088955.1	MICOS complex subunit mic25a-like isoform X2	1474	1.64E-128
XM_020088956.1	transcription factor HES-5-like	1087	1.31E-97
XM_020088957.1	NADH dehydrogenase [ubiquinone] 1 beta subcomplex subunit 11, mitochondrial	591	4.80E-88
XM_020088958.1	zinc finger protein 385A-like isoform X1	2265	0
XM_020088959.1	netrin receptor UNC5B-b-like	2657	1.27E-120
XM_020088960.1	zinc finger protein 385A-like isoform X2	2347	0
XM_020088961.1	zinc finger protein 385A-like isoform X3	2162	0
XM_020088962.1	PREDICTED: uncharacterized protein C1orf106 homolog isoform X1	2249	0
XM_020088963.1	PREDICTED: uncharacterized protein C1orf106 homolog isoform X1	2205	0
XM_020088964.1	serine hydroxymethyltransferase, mitochondrial-like	2686	0
XM_020088965.1	cilia- and flagella-associated protein 74	5137	0
XM_020088966.1	lens fiber major intrinsic protein-like	1397	0
XM_020088967.1	SH3 and cysteine-rich domain-containing protein 3-like	2043	0
XM_020088968.1	SH3 and cysteine-rich domain-containing protein 3-like	2065	0
XM_020088969.1	cilia- and flagella-associated protein 206	2237	0
XM_020088970.1	anoctamin-4 isoform X1	4743	0
XM_020088971.1	calcium uniporter regulatory subunit MCUb, mitochondrial-like	5324	0
XM_020088972.1	MICOS complex subunit Mic10-like	696	1.28E-49
XM_020088973.1	ceramide kinase-like	2346	0
XM_020088974.1	heat shock protein beta-7-like	2623	1.06E-99
XM_020088975.1	solute carrier family 26 member 10-like	2477	0
XM_020088976.1	mannan-binding lectin serine protease 2 isoform X1	1749	0
XM_020088977.1	mannan-binding lectin serine protease 2 isoform X1	1753	0

XM_020088978.1	mannan-binding lectin serine protease 2 isoform X1	1704	0
XM_020088979.1	PREDICTED: contactin-3-like	3081	0
XM_020088980.1	phosphomethylethanolamine N-methyltransferase-like isoform X2	1609	0
XM_020088981.1	polyhomeotic-like protein 2	1188	2.05E-160
XM_020088982.1	glyoxalase domain-containing protein 5 isoform X1	888	1.17E-130
XM_020088983.1	glyoxalase domain-containing protein 5 isoform X2	861	5.10E-124
XM_020088984.1	syntaphilin-like isoform X1	4507	0
XM_020088985.1	syntaphilin-like isoform X2	4327	0
XM_020088986.1	homeobox protein aristaless-like 3 isoform X1	1527	0
XM_020088987.1	homeobox protein aristaless-like 3 isoform X2	1464	0
XM_020088988.1	sodium-coupled neutral amino acid transporter 3-like	1576	0
XM_020088989.1	baculoviral IAP repeat-containing protein 5.1-A-like	693	1.72E-109
XM_020088990.1	phosphatidylinositol 4,5-bisphosphate 3-kinase catalytic subunit delta isoform isoform X1	3682	0
XM_020088991.1	kelch domain-containing protein 8B	2809	0
XM_020088992.1	SRSF protein kinase 3-like	2463	0
XM_020088993.1	probable tubulin polyglutamylase TTLL1	2377	0
XM_020088994.1	epidermal growth factor receptor kinase substrate 8-like isoform X1	2351	0
XM_020088995.1	golgin subfamily A member 4	6195	0
XM_020088996.1	epidermal growth factor receptor kinase substrate 8-like isoform X1	2329	0
XM_020088997.1	epidermal growth factor receptor kinase substrate 8-like protein 3 isoform X2	2348	0
XM_020088998.1	PREDICTED: caveolin-2-like	2125	1.07E-113
XM_020088999.1	preprosomatostatin I	1064	2.24E-63
XM_020089000.1	transcription factor HES-3	871	3.67E-160
XM_020089001.1	forkhead box protein L2-like	1189	5.82E-164
XM_020089002.1	TRAF3-interacting JNK-activating modulator-like	2023	0
XM_020089003.1	TRAF3-interacting JNK-activating modulator-like	2042	0
XM_020089004.1	nucleolin 2-like	2391	0
XM_020089005.1	homeodomain-interacting protein kinase 1-like	1511	0
XM_020089006.1	PREDICTED: uncharacterized protein C1orf127 homolog	2640	0
XM_020089007.1	cis-aconitate decarboxylase-like	1546	0
XM_020089008.1	deoxyribonuclease gamma	906	0
XM_020089009.1	transcription factor HES-2	1135	4.31E-121
XM_020089010.1	NADH dehydrogenase [ubiquinone] 1 alpha subcomplex subunit 4-like 2	944	3.10E-54
XM_020089011.1	neurexophilin-4 isoform X2	1018	0
XM_020089012.1	forkhead box protein P1-like	1149	0
XM_020089013.1	serine/threonine-protein kinase SBK1-like	1173	0
XM_020089014.1	taste receptor type 1 member 1-like	2466	0
XM_020089015.1	neurexin-1a-like isoform X4	3484	0
XM_020089016.1	PREDICTED: uncharacterized protein C1orf194 homolog isoform X1	468	1.93E-100
XM_020089017.1	PREDICTED: uncharacterized protein C1orf194 homolog isoform X2	569	1.00E-91
XM_020089018.1	protein FAM131C isoform X1	1240	2.33E-164
XM_020089019.1	protein FAM131C isoform X2	1237	1.30E-163
XM_020089020.1	transcription factor HES-2-like	677	2.29E-126
XM_020089021.1	C-type natriuretic peptide 3-like	552	1.16E-66
XM_020089022.1	neurotensin receptor type 1	1317	0
XM_020089023.1	ankyrin repeat domain-containing protein 33B-like	1350	0
XM_020089024.1	endoplasmic reticulum lectin 1	1349	0
XM_020089025.1	receptor tyrosine-protein kinase erbB-4-like	901	0
XM_020089026.1	ras association domain-containing protein 8-like	1428	0
XM_020089027.1	D(1)-like dopamine receptor	1404	0
XM_020089028.1	AMP deaminase 2 isoform X2	4612	0
XM_020089029.1	AMP deaminase 2 isoform X2	4589	0
XM_020089030.1	AMP deaminase 2 isoform X2	4520	0
XM_020089031.1	amphoterin-induced protein 1-like	6366	0
XM_020089032.1	amphoterin-induced protein 1-like	6071	0
XM_020089033.1	amphoterin-induced protein 1-like	6286	0
XM_020089034.1	amphoterin-induced protein 1-like	6151	0
XM_020089035.1	probable G-protein coupled receptor 61	3699	0
XM_020089036.1	collagen alpha-1(XIX) chain-like	1978	0
XM_020089037.1	guanine nucleotide-binding protein G(k) subunit alpha-like	2748	0
XM_020089038.1	guanine nucleotide-binding protein G(t) subunit alpha-2-like	1952	0
XM_020089039.1	centrosomal protein of 104 kDa isoform X1	4917	0
XM_020089040.1	centrosomal protein of 104 kDa isoform X1	4927	0
XM_020089041.1	centrosomal protein of 104 kDa isoform X1	4829	0
XM_020089042.1	dnaJ homolog subfamily C member 16	3463	0
XM_020089043.1	dnaJ homolog subfamily C member 16	3443	0
XM_020089044.1	dnaJ homolog subfamily C member 16	3250	0
XM_020089045.1	leucine-rich repeat-containing protein 47	1881	0
XM_020089046.1	PREDICTED: caspase-9	1952	0
XM_020089047.1	PREDICTED: caspase-9	1813	0
XM_020089048.1	GDNF family receptor alpha-like	1222	0
XM_020089049.1	PREDICTED: caspase-9	1772	0
XM_020089050.1	UDP-GlcNAc:betaGal beta-1,3-N-acetylglucosaminyltransferase 7-like	2143	0
XM_020089051.1	L-rhamnose-binding lectin SML-like	771	3.47E-166
XM_020089052.1	kinesin-like protein KIF1B isoform X1	9103	0
XM_020089053.1	kinesin-like protein KIF1B isoform X2	9085	0
XM_020089054.1	kinesin-like protein KIF1B isoform X3	9025	0
XM_020089055.1	kinesin-like protein KIF1B isoform X4	8983	0
XM_020089056.1	kinesin-like protein KIF1B isoform X5	8965	0
XM_020089057.1	kinesin-like protein KIF1B isoform X6	8851	0

XM_020089058.1	kinesin-like protein KIF1B isoform X7	8713	0
XM_020089059.1	kinesin-like protein KIF1B isoform X8	6184	0
XM_020089060.1	kinesin-like protein KIF1B isoform X9	6140	0
XM_020089061.1	6-phosphogluconate dehydrogenase, decarboxylating	1973	0
XM_020089062.1	AP-3 complex subunit mu-1-like	1140	0
XM_020089063.1	filamin-A-like isoform X3	7754	0
XM_020089064.1	filamin-A-like isoform X4	7730	0
XM_020089065.1	filamin-A-like isoform X3	9560	0
XM_020089066.1	filamin-A-like isoform X4	9536	0
XM_020089067.1	voltage-dependent L-type calcium channel subunit alpha-1D-like	8753	0
XM_020089068.1	choline dehydrogenase, mitochondrial	3556	0
XM_020089069.1	choline dehydrogenase, mitochondrial	3517	0
XM_020089070.1	mRNA-decapping enzyme 1A	3973	0
XM_020089071.1	interleukin-17 receptor B	2381	0
XM_020089072.1	troponin C, skeletal muscle	1150	2.15E-97
XM_020089073.1	transcription initiation factor TFIID subunit 13	596	4.09E-67
XM_020089074.1	transcription initiation factor TFIID subunit 13	602	5.66E-67
XM_020089075.1	stress response protein NST1-like	3323	0
XM_020089076.1	troponin C, skeletal muscle-like	538	2.35E-81
XM_020089077.1	collagen alpha-1(XX) chain-like	6730	0
XM_020089078.1	cadherin EGF LAG seven-pass G-type receptor 3 isoform X1	13514	0
XM_020089079.1	cadherin EGF LAG seven-pass G-type receptor 3 isoform X1	10852	0
XM_020089080.1	WD repeat-containing protein 6	5055	0
XM_020089081.1	WD repeat-containing protein 6	4944	0
XM_020089082.1	solute carrier family 26 member 6-like	2166	0
XM_020089083.1	PREDICTED: uncharacterized protein LOC109630745 isoform X1	1547	6.37E-153
XM_020089084.1	PREDICTED: uncharacterized protein LOC109630745 isoform X2	1544	2.43E-152
XM_020089085.1	solute carrier family 26 member 6-like	4418	0
XM_020089086.1	histone-lysine N-methyltransferase 2D isoform X1	18439	0
XM_020089087.1	WD repeat and FYVE domain-containing protein 3-like	9961	0
XM_020089088.1	histone-lysine N-methyltransferase 2D isoform X2	18436	0
XM_020089089.1	NF-kappa-B essential modulator isoform X1	2196	0
XM_020089090.1	NF-kappa-B essential modulator isoform X3	2154	0
XM_020089091.1	NF-kappa-B essential modulator isoform X3	2067	0
XM_020089092.1	NF-kappa-B essential modulator isoform X1	2352	0
XM_020089093.1	proline synthase co-transcribed bacterial homolog protein	1890	0
XM_020089094.1	transmembrane emp24 domain-containing protein 4	2874	4.58E-112
XM_020089095.1	calsyntenin-1 isoform X1	7632	0
XM_020089096.1	phospholipid-metabolizing enzyme A-C1-like	1766	1.83E-114
XM_020089097.1	calsyntenin-1 isoform X2	7089	0
XM_020089098.1	calsyntenin-1 isoform X3	7581	0
XM_020089099.1	calsyntenin-1 isoform X4	7038	0
XM_020089100.1	beta-catenin-interacting protein 1	1333	8.54E-97
XM_020089101.1	canalicular multispecific organic anion transporter 1	4223	0
XM_020089102.1	serine/threonine-protein kinase mTOR	3707	0
XM_020089103.1	angiopoietin-related protein 7	1800	0
XM_020089104.1	PREDICTED: uncharacterized protein KIAA2013 homolog	6533	0
XM_020089105.1	cold shock domain-containing protein E1 isoform X1	4759	0
XM_020089106.1	cold shock domain-containing protein E1 isoform X2	4729	0
XM_020089107.1	cold shock domain-containing protein E1 isoform X3	4822	0
XM_020089108.1	cold shock domain-containing protein E1 isoform X4	4702	0
XM_020089109.1	cold shock domain-containing protein E1 isoform X5	4672	0
XM_020089110.1	cold shock domain-containing protein E1 isoform X1	4570	0
XM_020089111.1	cold shock domain-containing protein E1 isoform X7	4590	0
XM_020089112.1	bromodomain and PHD finger-containing protein 3-like isoform X1	5653	0
XM_020089113.1	bromodomain and PHD finger-containing protein 3-like isoform X1	5650	0
XM_020089114.1	neuromedin-U receptor 1-like	1260	0
XM_020089115.1	bile acid receptor-like isoform X2	2654	0
XM_020089116.1	bile acid receptor-like isoform X2	2739	0
XM_020089117.1	bile acid receptor-like isoform X2	2423	0
XM_020089118.1	bile acid receptor-like isoform X2	2573	0
XM_020089119.1	lysosomal amino acid transporter 1 homolog isoform X2	1888	0
XM_020089120.1	lysosomal amino acid transporter 1 homolog isoform X2	1963	0
XM_020089121.1	lysosomal amino acid transporter 1 homolog isoform X2	1890	0
XM_020089122.1	arginine-glutamic acid dipeptide repeats protein isoform X1	5192	0
XM_020089123.1	arginine-glutamic acid dipeptide repeats protein isoform X1	5132	0
XM_020089124.1	arginine-glutamic acid dipeptide repeats protein isoform X1	5841	0
XM_020089125.1	arginine-glutamic acid dipeptide repeats protein isoform X1	5103	0
XM_020089126.1	arginine-glutamic acid dipeptide repeats protein isoform X1	5056	0
XM_020089127.1	arginine-glutamic acid dipeptide repeats protein isoform X1	5162	0
XM_020089128.1	rho guanine nucleotide exchange factor 16	3485	0
XM_020089129.1	Hermansky-Pudlak syndrome 1 protein	2561	0
XM_020089130.1	rho guanine nucleotide exchange factor 16	3399	0
XM_020089131.1	nuclease-sensitive element-binding protein 1 isoform X1	1536	1.51E-106
XM_020089132.1	nuclease-sensitive element-binding protein 1 isoform X2	1533	5.26E-109
XM_020089133.1	nuclease-sensitive element-binding protein 1 isoform X3	1524	1.91E-105
XM_020089134.1	nuclease-sensitive element-binding protein 1 isoform X4	1521	9.30E-110
XM_020089135.1	nuclease-sensitive element-binding protein 1 isoform X5	1473	1.18E-98
XM_020089136.1	nuclease-sensitive element-binding protein 1 isoform X6	1470	3.55E-101

XM_020089137.1	nuclease-sensitive element-binding protein 1 isoform X7	1460	1.38E-97
XM_020089138.1	nuclease-sensitive element-binding protein 1 isoform X8	1457	6.29E-102
XM_020089139.1	espin-like protein	4698	0
XM_020089140.1	ubiquitin carboxyl-terminal hydrolase 19 isoform X1	7810	0
XM_020089141.1	ubiquitin carboxyl-terminal hydrolase 19 isoform X2	7805	0
XM_020089142.1	ubiquitin carboxyl-terminal hydrolase 19 isoform X3	6123	0
XM_020089143.1	adenylate cyclase type 6-like	3853	0
XM_020089144.1	adenylate cyclase type 6-like	5435	0
XM_020089145.1	adenylate cyclase type 6-like	3808	0
XM_020089146.1	polypeptide N-acetylgalactosaminyltransferase 6-like	2828	0
XM_020089147.1	polypeptide N-acetylgalactosaminyltransferase 6-like	2910	0
XM_020089148.1	polypeptide N-acetylgalactosaminyltransferase 6-like	2971	0
XM_020089149.1	polypeptide N-acetylgalactosaminyltransferase 6-like	2806	0
XM_020089150.1	polypeptide N-acetylgalactosaminyltransferase 6-like	2920	0
XM_020089151.1	polypeptide N-acetylgalactosaminyltransferase 6-like	2824	0
XM_020089152.1	glucose-6-phosphate 1-dehydrogenase isoform X2	2058	0
XM_020089153.1	protein FAM110A-like	3188	0
XM_020089154.1	protein FAM110A-like	2843	0
XM_020089155.1	phosphoglycerate mutase 1-like	1909	1.36E-178
XM_020089156.1	protein FAM110A-like	2329	0
XM_020089157.1	msx2-interacting protein isoform X2	12983	0
XM_020089158.1	msx2-interacting protein isoform X2	12723	0
XM_020089159.1	transmembrane protein 82	1902	0
XM_020089160.1	protein NLRC3-like	3047	0
XM_020089161.1	transforming protein RhoA	3192	1.07E-133
XM_020089162.1	TELO2-interacting protein 1 homolog	4030	0
XM_020089163.1	TELO2-interacting protein 1 homolog	3953	0
XM_020089164.1	tuftelin-interacting protein 11 isoform X1	3597	0
XM_020089165.1	brefeldin A-inhibited guanine nucleotide-exchange protein 2-like isoform X1	7556	0
XM_020089166.1	brefeldin A-inhibited guanine nucleotide-exchange protein 2-like isoform X2	7552	0
XM_020089167.1	PDZ domain-containing protein 7-like	3959	0
XM_020089168.1	brefeldin A-inhibited guanine nucleotide-exchange protein 2-like isoform X3	7549	0
XM_020089169.1	brefeldin A-inhibited guanine nucleotide-exchange protein 2-like isoform X4	7546	0
XM_020089170.1	protein-L-isoaspartate O-methyltransferase domain-containing protein 1	3406	0
XM_020089171.1	inosine-5'-monophosphate dehydrogenase 2 isoform X1	2328	0
XM_020089172.1	inosine-5'-monophosphate dehydrogenase 2 isoform X2	2255	0
XM_020089173.1	MICAL C-terminal-like protein	3088	5.51E-157
XM_020089174.1	vacuolar protein sorting-associated protein 13D isoform X1	14458	0
XM_020089175.1	vacuolar protein sorting-associated protein 13D isoform X2	14437	0
XM_020089176.1	EF-hand domain-containing protein D2	5283	1.37E-124
XM_020089177.1	heat shock 70 kDa protein	2853	0
XM_020089178.1	potassium voltage-gated channel subfamily C member 4	4262	0
XM_020089179.1	EGF-containing fibulin-like extracellular matrix protein 1 isoform X2	759	2.31E-116
XM_020089180.1	ER membrane protein complex subunit 1 isoform X1	4007	0
XM_020089181.1	ER membrane protein complex subunit 1 isoform X2	3998	0
XM_020089182.1	ERBB receptor feedback inhibitor 1-like isoform X1	3772	0
XM_020089183.1	ERBB receptor feedback inhibitor 1-like isoform X2	3636	0
XM_020089184.1	cyclin-dependent kinase 11B-like isoform X1	3147	0
XM_020089185.1	cyclin-dependent kinase 11B-like isoform X1	3833	0
XM_020089186.1	cyclin-dependent kinase 11B-like isoform X1	3108	0
XM_020089187.1	matrix metalloproteinase-23	1753	0
XM_020089188.1	agrin isoform X2	9307	0
XM_020089189.1	agrin isoform X1	9295	0
XM_020089190.1	agrin isoform X2	9281	0
XM_020089191.1	agrin isoform X6	9264	0
XM_020089192.1	agrin isoform X5	9238	0
XM_020089193.1	leucine-rich glioma-inactivated protein 1-like	3195	0
XM_020089194.1	agrin isoform X5	9226	0
XM_020089195.1	agrin isoform X7	10645	0
XM_020089196.1	agrin isoform X8	8880	0
XM_020089197.1	agrin isoform X9	8837	0
XM_020089198.1	agrin isoform X10	10567	0
XM_020089199.1	guanine nucleotide-binding protein G(I)/G(S)/G(T) subunit beta-1-like	3942	0
XM_020089200.1	PREDICTED: sortilin-like	5051	0
XM_020089201.1	PREDICTED: neugrin	1269	0
XM_020089202.1	TAR DNA-binding protein 43 isoform X2	2250	0
XM_020089203.1	TAR DNA-binding protein 43 isoform X2	2383	0
XM_020089204.1	TAR DNA-binding protein 43 isoform X2	1922	0
XM_020089205.1	TAR DNA-binding protein 43 isoform X3	2222	0
XM_020089206.1	somatostatin receptor type 5-like	1059	0
XM_020089207.1	TAR DNA-binding protein 43 isoform X2	2356	0
XM_020089208.1	TAR DNA-binding protein 43 isoform X2	2199	0
XM_020089209.1	activity-dependent neuroprotector homeobox protein	4401	0
XM_020089210.1	dolichol-phosphate mannosyltransferase subunit 1	1206	3.85E-173
XM_020089211.1	protein-glutamine gamma-glutamyltransferase 2-like	2470	0
XM_020089212.1	regulation of nuclear pre-mRNA domain-containing protein 1B	3508	0
XM_020089213.1	serine/arginine-rich splicing factor 5-like	2719	6.59E-100
XM_020089214.1	peroxisome proliferator-activated receptor delta	3783	0
XM_020089215.1	myelin transcription factor 1 isoform X1	6743	0

XM_020089216.1	myelin transcription factor 1 isoform X2	6740	0
XM_020089217.1	choline O-acetyltransferase	2367	0
XM_020089218.1	myelin transcription factor 1 isoform X3	6680	0
XM_020089219.1	myelin transcription factor 1 isoform X4	6674	0
XM_020089220.1	myelin transcription factor 1 isoform X5	6662	0
XM_020089221.1	myelin transcription factor 1 isoform X1	6830	0
XM_020089222.1	myelin transcription factor 1 isoform X7	6593	0
XM_020089223.1	putative Polycomb group protein ASXL1	5471	0
XM_020089224.1	matrix metalloproteinase-17-like isoform X1	2815	0
XM_020089225.1	keratin, type II cytoskeletal 8-like	2259	0
XM_020089226.1	la-related protein 4-like isoform X2	5454	0
XM_020089227.1	la-related protein 4-like isoform X1	4928	0
XM_020089228.1	sodium channel protein type 8 subunit alpha-like isoform X1	10129	0
XM_020089229.1	leucine-rich repeat, immunoglobulin-like domain and transmembrane domain-containing protein 3	591	1.85E-120
XM_020089230.1	sodium channel protein type 8 subunit alpha-like isoform X2	5871	0
XM_020089231.1	sodium channel protein type 8 subunit alpha-like isoform X1	5424	0
XM_020089232.1	ubiquitin conjugation factor E4 B isoform X1	4076	0
XM_020089233.1	ubiquitin conjugation factor E4 B isoform X2	5528	0
XM_020089234.1	ral GTPase-activating protein subunit beta isoform X1	7016	0
XM_020089235.1	ral GTPase-activating protein subunit beta isoform X2	7013	0
XM_020089236.1	ral GTPase-activating protein subunit beta isoform X3	7006	0
XM_020089237.1	ral GTPase-activating protein subunit beta isoform X4	6995	0
XM_020089238.1	ral GTPase-activating protein subunit beta isoform X5	6950	0
XM_020089239.1	ral GTPase-activating protein subunit beta isoform X6	6947	0
XM_020089240.1	ral GTPase-activating protein subunit beta isoform X7	6937	0
XM_020089241.1	transmembrane protein 201	3838	0
XM_020089242.1	mitogen-activated protein kinase 14A-like isoform X1	2564	0
XM_020089243.1	disks large homolog 5-like	741	1.07E-166
XM_020089244.1	mitogen-activated protein kinase 14A-like isoform X2	2566	0
XM_020089245.1	E3 ubiquitin-protein ligase ARIH2	3983	0
XM_020089246.1	E3 ubiquitin-protein ligase ARIH2	3960	0
XM_020089247.1	structural maintenance of chromosomes protein 1A	4828	0
XM_020089248.1	PREDICTED: semaphorin-3B	3226	0
XM_020089249.1	PREDICTED: semaphorin-3B	3470	0
XM_020089250.1	PREDICTED: semaphorin-3B	5062	0
XM_020089251.1	zinc finger protein 335 isoform X1	5724	0
XM_020089252.1	zinc finger protein 335 isoform X2	5716	0
XM_020089253.1	zinc finger protein 335 isoform X3	5544	0
XM_020089254.1	PREDICTED: uncharacterized protein LOC109630828 isoform X1	1021	7.73E-163
XM_020089255.1	PREDICTED: uncharacterized protein LOC109630828 isoform X2	943	1.10E-160
XM_020089256.1	disks large homolog 5-like	7300	0
XM_020089257.1	toxin MIT1-like	699	7.35E-65
XM_020089258.1	pepsin A-like	1269	0
XM_020089259.1	helicase ARIP4-like isoform X1	7755	0
XM_020089260.1	helicase ARIP4-like isoform X1	4615	0
XM_020089261.1	helicase ARIP4-like isoform X1	4643	0
XM_020089262.1	helicase ARIP4-like isoform X1	4668	0
XM_020089263.1	disco-interacting protein 2 homolog B-A-like	2782	0
XM_020089264.1	disco-interacting protein 2 homolog B-A-like	3951	0
XM_020089265.1	isocitrate dehydrogenase [NAD] subunit gamma, mitochondrial isoform X1	2033	0
XM_020089266.1	isocitrate dehydrogenase [NAD] subunit gamma, mitochondrial isoform X2	2021	0
XM_020089267.1	protein FAM3A	2139	2.86E-149
XM_020089268.1	protein FAM3A	2290	1.31E-148
XM_020089269.1	cilia- and flagella-associated protein 43	3869	0
XM_020089270.1	phosphatase and actin regulator 3-like isoform X1	2340	0
XM_020089271.1	phosphatase and actin regulator 3-like isoform X2	2337	0
XM_020089272.1	phosphatase and actin regulator 3-like isoform X3	2337	0
XM_020089273.1	whey acidic protein-like	1056	4.55E-85
XM_020089274.1	protein Wnt-10b	2294	0
XM_020089275.1	proto-oncogene Wnt-1 isoform X1	3490	0
XM_020089276.1	proto-oncogene Wnt-1 isoform X2	1910	0
XM_020089277.1	activin receptor type-1B-like	3953	0
XM_020089278.1	PREDICTED: plexin-B1-like	9496	0
XM_020089279.1	kelch-like protein 17 isoform X1	3447	0
XM_020089280.1	kelch-like protein 17 isoform X1	3336	0
XM_020089281.1	kelch-like protein 17 isoform X1	3437	0
XM_020089282.1	serine/threonine-protein kinase 10-like	3000	0
XM_020089283.1	kelch-like protein 17 isoform X1	3071	0
XM_020089284.1	deleted in malignant brain tumors 1 protein-like isoform X2	2831	0
XM_020089285.1	deleted in malignant brain tumors 1 protein-like isoform X2	2835	0
XM_020089286.1	emerin-like isoform X1	2116	9.11E-159
XM_020089287.1	emerin-like isoform X1	1979	9.59E-135
XM_020089288.1	tensin-2-like isoform X1	7620	0
XM_020089289.1	tensin-2-like isoform X2	7617	0
XM_020089290.1	tensin-2-like isoform X3	7590	0
XM_020089291.1	tensin-2-like isoform X4	7578	0
XM_020089292.1	tensin-2-like isoform X5	6987	0
XM_020089293.1	tensin-2-like isoform X5	6894	0
XM_020089294.1	myosin light polypeptide 6 isoform X2	819	4.39E-107

XM_020089295.1	VPS10 domain-containing receptor SorCS3-like	4323	0
XM_020089296.1	myosin light polypeptide 6 isoform X2	855	1.01E-107
XM_020089297.1	myosin light polypeptide 6 isoform X2	868	1.17E-107
XM_020089298.1	ephrin type-A receptor 2-like isoform X1	5247	0
XM_020089299.1	ephrin type-A receptor 2-like isoform X2	5085	0
XM_020089300.1	proline-rich protein 36-like	7264	0
XM_020089301.1	proline-rich protein 36-like	7358	0
XM_020089302.1	proline-rich protein 36-like	7315	0
XM_020089303.1	myelin transcription factor 1-like isoform X1	4306	0
XM_020089304.1	myelin transcription factor 1-like isoform X1	4288	0
XM_020089305.1	myelin transcription factor 1-like isoform X1	4303	0

XM_020089306.1	myelin transcription factor 1-like isoform X1	4303	0
XM_020089307.1	myelin transcription factor 1-like isoform X1	4279	0
XM_020089308.1	myelin transcription factor 1-like isoform X5	4276	0
XM_020089309.1	myelin transcription factor 1-like isoform X1	4237	0
XM_020089310.1	gamma-adducin-like isoform X2	1989	0
XM_020089311.1	myelin transcription factor 1-like isoform X7	4210	0
XM_020089312.1	myelin transcription factor 1-like isoform X1	4192	0
XM_020089313.1	myelin transcription factor 1-like isoform X1	4174	0
XM_020089314.1	myelin transcription factor 1-like isoform X10	4165	0
XM_020089315.1	myelin transcription factor 1-like isoform X11	4162	0
XM_020089316.1	C-terminal-binding protein 1	3983	0
XM_020089317.1	matrix metalloproteinase-17-like isoform X1	2623	0
XM_020089318.1	protein-serine O-palmitoleoyltransferase porcupine	2181	0
XM_020089319.1	keratin, type I cytoskeletal 18-like	1774	0
XM_020089320.1	keratin, type I cytoskeletal 18-like	1407	0
XM_020089321.1	putative RNA-binding protein 15B	6706	0
XM_020089322.1	bcl-2-like protein 1 isoform X1	2306	1.06E-168
XM_020089323.1	bcl-2-like protein 1 isoform X1	2485	6.50E-168
XM_020089324.1	bcl-2-like protein 1 isoform X1	2156	2.22E-169
XM_020089325.1	disintegrin and metalloproteinase domain-containing protein 12-like	3069	0
XM_020089326.1	bcl-2-like protein 1 isoform X1	2164	2.44E-169
XM_020089327.1	bcl-2-like protein 1 isoform X1	2368	2.01E-168
XM_020089328.1	bcl-2-like protein 1 isoform X1	1321	2.48E-135
XM_020089329.1	keratin, type II cytoskeletal 8-like	2550	0
XM_020089330.1	putative fidgetin-like protein 2	4907	0
XM_020089331.1	putative fidgetin-like protein 2	4997	0
XM_020089332.1	putative fidgetin-like protein 2	5024	0
XM_020089333.1	putative fidgetin-like protein 2	4948	0
XM_020089334.1	putative fidgetin-like protein 2	4988	0
XM_020089335.1	E3 ubiquitin-protein ligase RLIM	4065	0
XM_020089336.1	E3 ubiquitin-protein ligase RLIM	4062	0
XM_020089337.1	E3 ubiquitin-protein ligase TRIM33 isoform X1	4773	0
XM_020089338.1	E3 ubiquitin-protein ligase TRIM33 isoform X2	4722	0
XM_020089339.1	G-protein coupled receptor 26-like	417	2.23E-87
XM_020089340.1	E3 ubiquitin-protein ligase TRIM33 isoform X3	4715	0
XM_020089341.1	E3 ubiquitin-protein ligase TRIM33 isoform X4	4664	0
XM_020089342.1	interferon-related developmental regulator 2-like isoform X1	3370	0
XM_020089343.1	interferon-related developmental regulator 2-like isoform X1	3312	0
XM_020089344.1	transmembrane protein 115	6376	3.24E-152
XM_020089345.1	spermatogenesis-associated protein 13 isoform X4	7371	0
XM_020089346.1	laminin subunit beta-2-like	6683	0
XM_020089347.1	laminin subunit beta-2-like	6803	0
XM_020089348.1	dynein heavy chain 12, axonemal-like	1937	0
XM_020089349.1	dynein heavy chain 12, axonemal-like	1934	0
XM_020089350.1	E3 ubiquitin-protein ligase Itchy-like	5362	0
XM_020089351.1	vesicle-associated membrane protein-associated protein B/C-like isoform X1	2307	1.40E-148
XM_020089352.1	dachshund homolog 1-like	1796	0
XM_020089353.1	vesicle-associated membrane protein-associated protein B/C-like isoform X2	2256	5.62E-150
XM_020089354.1	laminin subunit beta-2-like	7199	0
XM_020089355.1	mitofusin 2	4751	0
XM_020089356.1	eukaryotic translation initiation factor 2 subunit 2	2255	2.34E-172
XM_020089357.1	alpha-enolase isoform X1	1786	0
XM_020089358.1	alpha-enolase isoform X2	1849	0
XM_020089359.1	alpha-enolase isoform X2	1845	0
XM_020089360.1	protein scribble homolog	2684	0
XM_020089361.1	tumor protein p73 isoform X1	3655	0
XM_020089362.1	tumor protein p73 isoform X2	3571	0
XM_020089363.1	WD repeat-containing protein WRAP73	1996	0
XM_020089364.1	WD repeat-containing protein WRAP73	1894	0
XM_020089365.1	solute carrier family 35 member E2B	4222	0
XM_020089366.1	solute carrier family 35 member E2B	4107	0
XM_020089367.1	solute carrier family 25 member 45-like	2470	0
XM_020089368.1	solute carrier family 25 member 45-like	2451	0
XM_020089369.1	solute carrier family 25 member 45-like	2354	0
XM_020089370.1	solute carrier family 25 member 45-like	2387	0
XM_020089371.1	death effector domain-containing protein-like	5045	0
XM_020089372.1	death effector domain-containing protein-like	5085	0
XM_020089373.1	SUZ domain-containing protein 1-like	2502	1.90E-106

XM_020089374.1	PREDICTED: uncharacterized protein LOC109630885	3524	0
XM_020089375.1	probable tubulin polyglutamylase TTLL1	2215	0
XM_020089376.1	pecanex-like protein 3 isoform X1	3610	0
XM_020089377.1	protein phosphatase 1 regulatory subunit 3D-like	2171	0
XM_020089378.1	protein phosphatase 1 regulatory subunit 3D-like	1921	0
XM_020089379.1	protein phosphatase 1 regulatory subunit 3D-like	2185	0
XM_020089380.1	protein phosphatase 1 regulatory subunit 3D-like	1901	0
XM_020089381.1	PRELI domain containing protein 3B-like	3103	9.55E-134
XM_020089382.1	protein LMBR1L	3267	0
XM_020089383.1	desert hedgehog protein	2104	0
XM_020089384.1	desert hedgehog protein	1989	0
XM_020089385.1	procollagen-lysine,2-oxoglutarate 5-dioxygenase 1	3235	0
XM_020089386.1	protein RCC2	2806	0
XM_020089387.1	protein RCC2	2740	0

XM_020089388.1	SRSF protein kinase 1 isoform X1	3003	0
XM_020089389.1	mitogen-activated protein kinase kinase kinase MLK4	2799	0
XM_020089390.1	SRSF protein kinase 1 isoform X2	3272	0
XM_020089391.1	SRSF protein kinase 1 isoform X3	3003	0
XM_020089392.1	lipoma HMGIC fusion partner-like 3 protein isoform X1	893	5.45E-135
XM_020089393.1	serine--tRNA ligase, cytoplasmic	2501	0
XM_020089394.1	PTB domain-containing engulfment adapter protein 1-like	770	6.50E-118
XM_020089395.1	guanine nucleotide-binding protein G(s) subunit alpha	3188	0
XM_020089396.1	pleckstrin homology domain-containing family M member 2 isoform X1	6338	0
XM_020089397.1	pleckstrin homology domain-containing family M member 2 isoform X2	6278	0
XM_020089398.1	5' exonuclease Apollo	2684	0
XM_020089399.1	adenosine receptor A1-like	4247	0
XM_020089400.1	serine/threonine-protein kinase WNK3-like isoform X2	6918	0
XM_020089401.1	matrix metalloproteinase-17-like isoform X3	2489	0
XM_020089402.1	interferon gamma receptor 1 precursor	3165	0
XM_020089403.1	serine/threonine-protein kinase WNK3-like isoform X2	6902	0
XM_020089404.1	serine/threonine-protein kinase WNK2-like isoform X3	6777	0
XM_020089405.1	serine/threonine-protein kinase WNK3-like isoform X4	5113	0
XM_020089406.1	zinc finger protein PLAGL2-like	6712	0
XM_020089407.1	protein RER1 isoform X1	2679	7.24E-137
XM_020089408.1	protein RER1 isoform X1	2680	4.94E-137
XM_020089409.1	protein RER1 isoform X1	2659	6.22E-137
XM_020089410.1	protein RER1 isoform X1	1248	7.43E-137
XM_020089411.1	monoglyceride lipase	7505	0
XM_020089412.1	TBC1 domain family member 25	4615	0
XM_020089413.1	NSFL1 cofactor p47	2256	0
XM_020089414.1	transmembrane and coiled-coil domain-containing protein 4	3037	0
XM_020089415.1	RAC-alpha serine/threonine-protein kinase isoform X1	6724	0
XM_020089416.1	von Willebrand factor A domain-containing protein 1-like	2793	0
XM_020089417.1	plasma membrane calcium-transporting ATPase 3-like isoform X1	4796	0
XM_020089418.1	plasma membrane calcium-transporting ATPase 3-like isoform X1	4804	0
XM_020089419.1	plasma membrane calcium-transporting ATPase 3-like isoform X1	7133	0
XM_020089420.1	plasma membrane calcium-transporting ATPase 3-like isoform X1	7933	0
XM_020089421.1	plasma membrane calcium-transporting ATPase 3-like isoform X1	7915	0
XM_020089422.1	plasma membrane calcium-transporting ATPase 3-like isoform X1	3856	0
XM_020089423.1	plasma membrane calcium-transporting ATPase 3-like isoform X5	4638	0
XM_020089424.1	U8 snoRNA-decapping enzyme-like	994	3.38E-126
XM_020089425.1	rho GTPase-activating protein 4	5408	0
XM_020089426.1	zinc finger protein castor homolog 1 isoform X1	7137	0
XM_020089427.1	zinc finger protein castor homolog 1 isoform X2	7131	0
XM_020089428.1	cytoplasmic dynein 2 light intermediate chain 1	1138	0
XM_020089429.1	zinc finger protein castor homolog 1 isoform X3	7376	0
XM_020089430.1	zinc finger protein castor homolog 1 isoform X4	6795	0
XM_020089431.1	zinc finger protein castor homolog 1 isoform X5	6789	0
XM_020089432.1	cAMP-dependent protein kinase type II-alpha regulatory subunit	3064	0
XM_020089433.1	cAMP-dependent protein kinase type II-alpha regulatory subunit	2824	0
XM_020089434.1	cAMP-dependent protein kinase type II-alpha regulatory subunit	3062	0
XM_020089435.1	E3 ubiquitin-protein ligase MIB2 isoform X1	2585	0
XM_020089436.1	E3 ubiquitin-protein ligase MIB2 isoform X2	2608	0
XM_020089437.1	E3 ubiquitin-protein ligase MIB2 isoform X2	3137	0
XM_020089438.1	E3 ubiquitin-protein ligase MIB2 isoform X1	2149	0
XM_020089439.1	histone deacetylase 7-like isoform X3	6708	0
XM_020089440.1	histone deacetylase 7-like isoform X3	6690	0
XM_020089441.1	histone deacetylase 7-like isoform X3	6712	0
XM_020089442.1	histone deacetylase 7-like isoform X3	6643	0
XM_020089443.1	leucine-rich PPR motif-containing protein, mitochondrial	2454	0
XM_020089444.1	SPRY domain-containing SOCS box protein 4-like	2620	0
XM_020089445.1	SPRY domain-containing SOCS box protein 4-like	2548	0
XM_020089446.1	zinc finger protein 271-like	3304	0
XM_020089447.1	E3 ubiquitin-protein ligase TRIM33-like	1789	4.71E-146
XM_020089448.1	oxysterol-binding protein-related protein 2-like	4523	0
XM_020089449.1	peroxisomal membrane protein PEX14 isoform X1	2171	0
XM_020089450.1	peroxisomal membrane protein PEX14 isoform X2	2109	0
XM_020089451.1	peroxisomal membrane protein PEX14 isoform X3	1958	0
XM_020089452.1	partitioning defective 6 homolog beta	4878	0

XM_020089453.1	beta-catenin-interacting protein 1	3329	1.55E-49
XM_020089454.1	beta-catenin-interacting protein 1	3392	2.20E-49
XM_020089455.1	neutral and basic amino acid transport protein rBAT	1810	0
XM_020089456.1	serine/threonine-protein kinase pim-1-like	2792	0
XM_020089457.1	transmembrane protein 106B-like	2815	5.96E-126
XM_020089458.1	kinesin heavy chain-like isoform X1	6874	0
XM_020089459.1	kinesin heavy chain-like isoform X2	6874	0
XM_020089460.1	NAD kinase isoform X1	3588	0
XM_020089461.1	NAD kinase-like isoform X2	3583	0
XM_020089462.1	tyrosine-protein kinase Lyn-like	4664	0
XM_020089463.1	dentin sialophosphoprotein-like isoform X1	4902	0
XM_020089464.1	dentin sialophosphoprotein-like isoform X1	4831	0
XM_020089465.1	pleckstrin homology domain-containing family N member 1 isoform X1	2864	0
XM_020089466.1	pleckstrin homology domain-containing family N member 1 isoform X2	2861	0
XM_020089467.1	S1 RNA-binding domain-containing protein 1	3634	0
XM_020089468.1	selenide, water dikinase 1	2197	0
XM_020089469.1	voltage-dependent L-type calcium channel subunit beta-4-like	4261	0

XM_020089470.1	monocarboxylate transporter 5	5307	0
XM_020089471.1	spermidine synthase	2778	0
XM_020089472.1	adenosylhomocysteinase 2 isoform X1	3104	0
XM_020089473.1	adenosylhomocysteinase 2 isoform X2	1943	0
XM_020089474.1	adenosylhomocysteinase 2 isoform X3	2726	0
XM_020089475.1	nuclear transcription factor Y subunit alpha isoform X1	2126	1.16E-105
XM_020089476.1	nuclear transcription factor Y subunit alpha isoform X2	2124	5.17E-107
XM_020089477.1	nuclear transcription factor Y subunit alpha isoform X3	2111	1.33E-78
XM_020089478.1	nuclear transcription factor Y subunit alpha isoform X3	2201	2.12E-78
XM_020089479.1	protein Daple-like	3308	0
XM_020089480.1	cyclin-related protein FAM58A	2576	0
XM_020089481.1	DNA fragmentation factor subunit beta	2086	0
XM_020089482.1	UPF0688 protein C1orf174 homolog	1758	2.00E-91
XM_020089483.1	semaphorin-3G isoform X1	6315	0
XM_020089484.1	semaphorin-3G isoform X2	6312	0
XM_020089485.1	probable G-protein coupled receptor 157	2721	0
XM_020089486.1	rho guanine nucleotide exchange factor 25-like isoform X1	4465	0
XM_020089487.1	rho guanine nucleotide exchange factor 25-like isoform X2	4462	0
XM_020089488.1	rho guanine nucleotide exchange factor 25-like isoform X3	4257	0
XM_020089489.1	vitamin D3 receptor A	8822	0
XM_020089490.1	copine-5-like isoform X1	3960	0
XM_020089491.1	copine-5-like isoform X2	3944	0
XM_020089492.1	carbonic anhydrase 6	1883	0
XM_020089493.1	solute carrier family 2, facilitated glucose transporter member 9	1817	0
XM_020089494.1	glutathione synthetase	2826	0
XM_020089495.1	methylenetetrahydrofolate reductase-like	1251	0
XM_020089496.1	methylenetetrahydrofolate reductase-like	1347	0
XM_020089497.1	methylenetetrahydrofolate reductase-like	1403	0
XM_020089498.1	methylenetetrahydrofolate reductase-like	1318	0
XM_020089499.1	RIB43A-like with coiled-coils protein 1	1248	0
XM_020089500.1	coiled-coil domain-containing protein 22	2865	0
XM_020089501.1	PREDICTED: uncharacterized protein LOC109630962, partial	9735	0
XM_020089502.1	homeodomain-interacting protein kinase 1-like	1510	0
XM_020089503.1	cyclic AMP-dependent transcription factor ATF-7-like isoform X2	1790	0
XM_020089504.1	cyclic AMP-dependent transcription factor ATF-7-like isoform X2	1650	0
XM_020089505.1	interleukin-17 receptor D-like isoform X1	2476	0
XM_020089506.1	interleukin-17 receptor D-like isoform X2	2027	0
XM_020089507.1	protein FAM50A	1447	4.14E-156
XM_020089508.1	copine-5-like isoform X2	4490	0
XM_020089509.1	probable cation-transporting ATPase 13A3 isoform X1	3775	0
XM_020089510.1	probable cation-transporting ATPase 13A2 isoform X1	3787	0
XM_020089511.1	protein-S-isoprenylcysteine O-methyltransferase	3648	0
XM_020089512.1	solute carrier family 41 member 1-like	4390	0
XM_020089513.1	solute carrier family 41 member 1-like	4336	0
XM_020089514.1	E3 ubiquitin-protein ligase pellino homolog 1-like	3739	0
XM_020089515.1	solute carrier family 41 member 1-like	4320	0
XM_020089516.1	solute carrier family 41 member 1-like	4469	0
XM_020089517.1	DNA repair protein complementing XP-C cells	2972	0
XM_020089518.1	structural maintenance of chromosomes protein 6	3565	0
XM_020089519.1	protein SOGA1 isoform X1	6414	0
XM_020089520.1	protein SOGA1 isoform X2	6411	0
XM_020089521.1	protein SOGA1 isoform X3	6348	0
XM_020089522.1	BLOC-1-related complex subunit 6 isoform X1	4447	0
XM_020089523.1	BLOC-1-related complex subunit 6 isoform X2	4369	0
XM_020089524.1	ankyrin repeat and SOCS box protein 8	2588	0
XM_020089525.1	ankyrin repeat and SOCS box protein 8	2434	0
XM_020089526.1	ankyrin repeat and SOCS box protein 8	2295	0
XM_020089527.1	PREDICTED: caltractin-like	1542	5.63E-107
XM_020089528.1	UTP--glucose-1-phosphate uridylyltransferase-like	3019	0
XM_020089529.1	matrix metalloproteinase-9	2787	0
XM_020089530.1	solute carrier family 25 member 33	2366	0
XM_020089531.1	60S ribosomal protein L10a	793	4.36E-144

XM_020089532.1	calcitonin gene-related peptide type 1 receptor	4390	0
XM_020089533.1	exosome component 10 isoform X1	2971	0
XM_020089534.1	exosome component 10 isoform X2	2829	0
XM_020089535.1	dolichyl-diphosphooligosaccharide--protein glycosyltransferase 48 kDa subunit	1940	0
XM_020089536.1	glycoprotein-N-acetylgalactosamine 3-beta-galactosyltransferase 1-B-like	2272	0
XM_020089537.1	glycoprotein-N-acetylgalactosamine 3-beta-galactosyltransferase 1-B-like	2348	0
XM_020089538.1	PREDICTED: tachykinin-3	850	6.33E-73
XM_020089539.1	probable E3 ubiquitin-protein ligase DTX3 isoform X1	3425	0
XM_020089540.1	WD repeat-containing and planar cell polarity effector protein fritz homolog	4016	0
XM_020089541.1	probable E3 ubiquitin-protein ligase DTX3 isoform X1	3215	0
XM_020089542.1	probable E3 ubiquitin-protein ligase DTX3 isoform X1	3174	0
XM_020089543.1	TBC1 domain family member 22B isoform X1	3965	0
XM_020089544.1	TBC1 domain family member 22B isoform X2	3965	0
XM_020089545.1	TBC1 domain family member 22B isoform X3	3904	0
XM_020089546.1	activin receptor type-1-like isoform X1	3003	0
XM_020089547.1	neural cell adhesion molecule L1-like protein isoform X1	4636	0
XM_020089548.1	neural cell adhesion molecule L1-like protein isoform X1	4610	0
XM_020089549.1	neural cell adhesion molecule L1-like protein isoform X1	4556	0
XM_020089550.1	neural cell adhesion molecule L1-like protein isoform X1	4588	0
XM_020089551.1	neural cell adhesion molecule L1-like protein isoform X1	4573	0
XM_020089552.1	neural cell adhesion molecule L1-like protein isoform X1	4459	0
XM_020089553.1	neural cell adhesion molecule L1-like protein isoform X5	4321	0
XM_020089554.1	neural cell adhesion molecule L1-like protein isoform X6	4258	0
XM_020089555.1	contactin-associated protein-like 4	2270	0
XM_020089556.1	neural cell adhesion molecule L1-like protein isoform X7	4210	0
XM_020089557.1	probable G-protein coupled receptor 160	1678	0
XM_020089558.1	peptidyl-prolyl cis-trans isomerase-like	1054	4.81E-127
XM_020089559.1	mitogen-activated protein kinase 13-like	1516	0
XM_020089560.1	rho-related GTP-binding protein Rho6	3531	7.33E-161
XM_020089561.1	paired box protein Pax-7-like isoform X1	2360	0
XM_020089562.1	paired box protein Pax-7-like isoform X2	2345	0
XM_020089563.1	paired box protein Pax-7-like isoform X3	1750	0
XM_020089564.1	plexin-A1 isoform X1	7315	0
XM_020089565.1	histone-lysine N-methyltransferase SUV39H1 isoform X1	1747	0
XM_020089566.1	mitotic spindle-associated MMXD complex subunit MIP18-like	986	3.33E-134
XM_020089567.1	histone-lysine N-methyltransferase SUV39H1 isoform X1	2071	0
XM_020089568.1	DNA fragmentation factor subunit alpha	3390	0
XM_020089569.1	60S ribosomal protein L10	757	9.26E-162
XM_020089570.1	runt-related transcription factor 2-like	1940	0
XM_020089571.1	carboxy-terminal domain RNA polymerase II polypeptide A small phosphatase 1-like isoform X3	3892	0
XM_020089572.1	carboxy-terminal domain RNA polymerase II polypeptide A small phosphatase 1-like isoform X2	2118	1.74E-163
XM_020089573.1	carboxy-terminal domain RNA polymerase II polypeptide A small phosphatase 1-like isoform X3	2111	5.07E-171
XM_020089574.1	cyclic AMP-dependent transcription factor ATF-2	3299	0
XM_020089575.1	protein lunapark-B-like isoform X1	2157	0
XM_020089576.1	ribulose-phosphate 3-epimerase	1321	2.80E-168
XM_020089577.1	TRAF3-interacting protein 1 isoform X1	3716	0
XM_020089578.1	TRAF3-interacting protein 1 isoform X2	3695	0
XM_020089579.1	CD302 antigen isoform X1	1891	6.51E-152
XM_020089580.1	CD302 antigen isoform X2	1888	4.04E-151
XM_020089581.1	attractin-like protein 1	534	2.55E-132
XM_020089582.1	CD302 antigen isoform X3	1885	2.19E-150
XM_020089583.1	thrombospondin type-1 domain-containing protein 7B	7559	0
XM_020089584.1	caspase 10	2277	0
XM_020089585.1	caspase 10	2374	0
XM_020089586.1	interferon-inducible double-stranded RNA-dependent protein kinase activator A isoform X1	1989	0
XM_020089587.1	interferon-inducible double-stranded RNA-dependent protein kinase activator A isoform X1	2093	0
XM_020089588.1	GDP-fucose protein O-fucosyltransferase 2	2302	0
XM_020089589.1	claudin-10-like isoform X2	1284	1.10E-176
XM_020089590.1	PREDICTED: claudin-10-like	1226	2.50E-164
XM_020089591.1	claudin-10-like isoform X2	1502	8.62E-166
XM_020089592.1	claudin-10-like isoform X2	1371	1.57E-154
XM_020089593.1	coiled-coil domain-containing protein 172	681	4.79E-128
XM_020089594.1	FAST kinase domain-containing protein 3, mitochondrial-like	3992	0
XM_020089595.1	myc box-dependent-interacting protein 1-like	4576	0
XM_020089596.1	low-density lipoprotein receptor-related protein 1B	14986	0
XM_020089597.1	UPF0524 protein C3orf70 homolog	3183	2.44E-125
XM_020089598.1	matrix-remodeling-associated protein 5-like	9683	0
XM_020089599.1	C-X-C chemokine receptor type 4	1739	0
XM_020089600.1	putative methyltransferase DDB_G0268948	1563	0
XM_020089601.1	putative methyltransferase DDB_G0268948	986	0
XM_020089602.1	putative methyltransferase DDB_G0268948	1559	0
XM_020089603.1	protocadherin-8-like isoform X2	3178	0
XM_020089604.1	pro-neuregulin-3, membrane-bound isoform-like	257	1.94E-54
XM_020089605.1	FAST kinase domain-containing protein 1, mitochondrial	3178	0
XM_020089606.1	FAST kinase domain-containing protein 1, mitochondrial	3155	0
XM_020089607.1	FAST kinase domain-containing protein 1, mitochondrial	3161	0
XM_020089608.1	FAST kinase domain-containing protein 1, mitochondrial	3096	0
XM_020089609.1	FAST kinase domain-containing protein 1, mitochondrial	3078	0
XM_020089610.1	glutamate decarboxylase 1-like	2872	0

XM_020089611.1	nuclear receptor subfamily 4 group A member 2	2533	0
XM_020089612.1	glycerol-3-phosphate dehydrogenase, mitochondrial	1756	4.82E-65
XM_020089613.1	hydroxyacylglutathione hydrolase-like protein	3023	0
XM_020089614.1	dehydrogenase/reductase SDR family member on chromosome X	5278	0
XM_020089615.1	dehydrogenase/reductase SDR family member on chromosome X	5644	0
XM_020089616.1	activin receptor type-1C isoform X1	3577	0
XM_020089617.1	intersectin-2-like isoform X1	2237	0
XM_020089618.1	activin receptor type-1C isoform X2	3529	0
XM_020089619.1	putative hexokinase HKDC1 isoform X2	2501	0
XM_020089620.1	ornithine carbamoyltransferase, mitochondrial	2016	0
XM_020089621.1	chondroitin sulfate synthase 2	3251	0
XM_020089622.1	NXPE family member 3	3854	0
XM_020089623.1	syntaxin-binding protein 5-like isoform X1	5708	0
XM_020089624.1	syntaxin-binding protein 5-like isoform X2	4841	0
XM_020089625.1	syntaxin-binding protein 5-like isoform X3	4736	0
XM_020089626.1	syntaxin-binding protein 5-like isoform X4	4682	0
XM_020089627.1	syntaxin-binding protein 5-like isoform X5	4649	0
XM_020089628.1	syntaxin-binding protein 5-like isoform X6	4580	0
XM_020089629.1	syntaxin-binding protein 5-like isoform X7	4505	0
XM_020089630.1	pyridoxal kinase-like	5921	1.68E-165
XM_020089631.1	syntaxin-binding protein 5-like isoform X8	4472	0
XM_020089632.1	syntaxin-binding protein 5-like isoform X9	4403	0
XM_020089633.1	pituitary-specific positive transcription factor 1 isoform X1	2329	0
XM_020089634.1	pituitary-specific positive transcription factor 1 isoform X1	2163	0
XM_020089635.1	pituitary-specific positive transcription factor 1 isoform X1	2343	0
XM_020089636.1	pituitary-specific positive transcription factor 1 isoform X1	2558	0
XM_020089637.1	pituitary-specific positive transcription factor 1 isoform X1	2785	0
XM_020089638.1	pituitary-specific positive transcription factor 1 isoform X1	2345	0
XM_020089639.1	pituitary-specific positive transcription factor 1 isoform X1	2310	0
XM_020089640.1	pituitary-specific positive transcription factor 1 isoform X1	2320	0
XM_020089641.1	pituitary-specific positive transcription factor 1 isoform X1	3807	0
XM_020089642.1	pituitary-specific positive transcription factor 1 isoform X4	1968	0
XM_020089643.1	G protein-activated inward rectifier potassium channel 1-like	4049	0
XM_020089644.1	nucleolus and neural progenitor protein	2484	0
XM_020089645.1	SH2 domain-containing protein 4A-like	1877	4.76E-162
XM_020089646.1	solute carrier family 22 member 7-like	450	8.21E-110
XM_020089647.1	neuroligin-4, X-linked-like	5769	0
XM_020089648.1	receptor-type tyrosine-protein phosphatase-like N	4109	0
XM_020089649.1	rho GTPase-activating protein 6 isoform X2	3391	0
XM_020089650.1	transcription cofactor vestigial-like protein 3	1976	0
XM_020089651.1	transcription cofactor vestigial-like protein 3	1807	0
XM_020089652.1	coiled-coil domain-containing protein 138 isoform X1	2433	0
XM_020089653.1	coiled-coil domain-containing protein 138 isoform X2	2421	0
XM_020089654.1	coiled-coil domain-containing protein 138 isoform X3	2364	0
XM_020089655.1	melanoma differentiation-associated protein 5	3312	0
XM_020089656.1	gamma-aminobutyric acid type B receptor subunit 2 isoform X2	3909	0
XM_020089657.1	E3 ubiquitin-protein ligase TRIM68-like	2108	0
XM_020089658.1	DNA primase large subunit	2101	0
XM_020089659.1	toll-like receptor 8	3296	0
XM_020089660.1	toll-like receptor 8	3547	0
XM_020089661.1	atypical chemokine receptor 3	2960	0
XM_020089662.1	fas apoptotic inhibitory molecule 1	3110	4.74E-121
XM_020089663.1	fas apoptotic inhibitory molecule 1	3107	6.45E-121
XM_020089664.1	fas apoptotic inhibitory molecule 1	3003	2.17E-121
XM_020089665.1	fas apoptotic inhibitory molecule 1	3004	2.62E-121
XM_020089666.1	FAST kinase domain-containing protein 1, mitochondrial	3023	0
XM_020089667.1	beta-crystallin A2 isoform X1	967	8.08E-173
XM_020089668.1	beta-crystallin A2 isoform X1	935	3.89E-158
XM_020089669.1	protein Wnt-10a	3574	0
XM_020089670.1	inorganic pyrophosphatase	1355	0
XM_020089671.1	BTB/POZ domain-containing protein KCTD6	1874	0
XM_020089672.1	general transcription factor IIF subunit 2-like	1227	3.67E-158
XM_020089673.1	NAD(P)H dehydrogenase [quinone] 1-like	982	0
XM_020089674.1	gap junction alpha-5 protein	2704	0
XM_020089675.1	beta-chimaerin isoform X3	2421	0
XM_020089676.1	G-protein coupled receptor 143	2489	0
XM_020089677.1	poly [ADP-ribose] polymerase 15-like	3968	0
XM_020089678.1	protein FEV isoform X2	2877	1.03E-146
XM_020089679.1	protein FEV isoform X2	2879	6.63E-123
XM_020089680.1	protein FEV isoform X2	2827	2.07E-123
XM_020089681.1	PREDICTED: uncharacterized protein C3orf38 homolog	1364	0
XM_020089682.1	sushi repeat-containing protein SRPX isoform X1	1394	0
XM_020089683.1	sushi repeat-containing protein SRPX isoform X2	1341	0
XM_020089684.1	cilia- and flagella-associated protein 65	5423	0
XM_020089685.1	PREDICTED: tomoregulin-2	2290	0
XM_020089686.1	transient receptor potential cation channel subfamily M member 2	4541	0
XM_020089687.1	transcription factor Sp5	1738	0
XM_020089688.1	cyclin-dependent kinase 5 activator 1	3179	0
XM_020089689.1	indian hedgehog B protein-like	3325	0

XM_020089690.1	PREDICTED: uncharacterized protein LOC109631093	1418	0
XM_020089691.1	CASP8 and FADD-like apoptosis regulator isoform X1	2528	0
XM_020089692.1	CASP8 and FADD-like apoptosis regulator isoform X1	2668	0
XM_020089693.1	CASP8 and FADD-like apoptosis regulator isoform X1	2670	0
XM_020089694.1	CASP8 and FADD-like apoptosis regulator isoform X1	2357	0
XM_020089695.1	protein Wnt-6	3485	0
XM_020089696.1	immunoglobulin superfamily member 3-like	4431	0
XM_020089697.1	tumor necrosis factor receptor superfamily member EDAR	3899	0
XM_020089698.1	protocadherin-17 isoform X2	4687	0
XM_020089699.1	protocadherin-8-like isoform X1	3408	0
XM_020089700.1	protocadherin-8-like isoform X2	3405	0
XM_020089701.1	serine/threonine-protein kinase pim-2-like	764	2.66E-180
XM_020089702.1	CAVP-target protein	780	3.95E-169
XM_020089703.1	VPS10 domain-containing receptor SorCS1-like	5595	0
XM_020089704.1	obscurin-like protein 1	8816	0
XM_020089705.1	serine/threonine-protein kinase pim-2-like	1035	0
XM_020089706.1	dynein heavy chain 7, axonemal	11668	0
XM_020089707.1	poly [ADP-ribose] polymerase 14-like	5377	0
XM_020089708.1	lactase-phlorizin hydrolase-like	5745	0
XM_020089709.1	ankyrin repeat and SOCS box protein 12-like	2271	0
XM_020089710.1	olfactory receptor 11H6-like	933	0
XM_020089711.1	olfactory receptor 11H6-like	774	1.90E-156
XM_020089712.1	olfactory receptor 11H6-like	945	1.61E-179
XM_020089713.1	olfactory receptor 5AC1-like	984	0
XM_020089714.1	pyridine nucleotide-disulfide oxidoreductase domain-containing protein 2	2108	0
XM_020089715.1	olfactory receptor 51I2-like	948	0
XM_020089716.1	mitogen-activated protein kinase kinase kinase 19-like	4171	0
XM_020089717.1	polycystic kidney disease protein 1-like 2	6846	0
XM_020089718.1	serine/threonine-protein kinase pim-2-like	1610	0
XM_020089719.1	serine/threonine-protein kinase pim-2-like	1119	0
XM_020089720.1	uridine 5'-monophosphate synthase	2636	0
XM_020089721.1	olfactory receptor 1D5-like	594	6.46E-119
XM_020089722.1	olfactory receptor 13-like	948	0
XM_020089723.1	ADP-ribosylation factor-like protein 6	612	4.13E-152
XM_020089724.1	ephrin type-A receptor 3	6128	0
XM_020089725.1	adapter molecule crk-like	2427	0
XM_020089726.1	N-acetylserotonin O-methyltransferase-like protein isoform X3	1220	0
XM_020089727.1	solute carrier family 12 member 8-like	1409	0
XM_020089728.1	protein HEG homolog 1	3761	0
XM_020089729.1	KAT8 regulatory NSL complex subunit 1-like protein	3435	0
XM_020089730.1	microtubule-associated protein 2-like	7528	0
XM_020089731.1	carbamoyl-phosphate synthase [ammonia], mitochondrial	4566	0
XM_020089732.1	receptor tyrosine-protein kinase erbB-4-like	402	1.21E-94
XM_020089733.1	ATP-binding cassette sub-family A member 12	7609	0
XM_020089734.1	tubulin alpha chain-like	1086	0
XM_020089735.1	dnaJ homolog subfamily B member 6-like	1860	1.80E-143
XM_020089736.1	DNA helicase MCM8	3368	0
XM_020089737.1	PREDICTED: uncharacterized protein LOC109631140	1012	0
XM_020089738.1	insulin-like growth factor 2 mRNA-binding protein 2	1887	0
XM_020089739.1	catenin delta-2-like isoform X1	4417	0
XM_020089740.1	protein TANC1	6982	0
XM_020089741.1	bromodomain adjacent to zinc finger domain protein 2B-like	7844	0
XM_020089742.1	DNA helicase MCM8	3376	0
XM_020089743.1	dipeptidyl peptidase 4-like	2446	0
XM_020089744.1	STE20/SPS1-related proline-alanine-rich protein kinase	1571	0
XM_020089745.1	low-density lipoprotein receptor-related protein 2	7155	0
XM_020089746.1	methyltransferase-like protein 5	715	3.10E-126
XM_020089747.1	unconventional myosin-VIIa-like	3441	0
XM_020089748.1	rap guanine nucleotide exchange factor 4-like	3456	0
XM_020089749.1	acetylcholine receptor subunit alpha	1261	0
XM_020089750.1	dnaJ homolog subfamily C member 3	2024	0
XM_020089751.1	conserved oligomeric Golgi complex subunit 3	2258	0
XM_020089752.1	FERM and PDZ domain-containing protein 4-like	723	1.50E-163
XM_020089753.1	bone morphogenetic protein 5	2871	0
XM_020089754.1	lymphocyte function-associated antigen 3-like	920	6.93E-158
XM_020089755.1	immunoglobulin superfamily member 3-like	2603	0
XM_020089756.1	vang-like protein 2	2543	0
XM_020089757.1	nectin-3-like protein isoform X2	1179	0
XM_020089758.1	POU domain, class 3, transcription factor 1	2444	0
XM_020089759.1	follistatin-related protein 1	2117	2.06E-171
XM_020089760.1	collagen alpha-1(IV) chain	6465	0
XM_020089761.1	X-linked retinitis pigmentosa GTPase regulator-like	5740	0
XM_020089762.1	bone morphogenetic protein 5	2781	0
XM_020089763.1	protein-lysine methyltransferase METTL21E-like	792	0
XM_020089764.1	junctional adhesion molecule B-like	1021	4.15E-96
XM_020089765.1	teleost multiple tissue opsin-1b	951	0
XM_020089766.1	KDEL motif-containing protein 1	1895	0
XM_020089767.1	transmembrane protein 177	1228	0
XM_020089768.1	bile salt export pump	4069	0

XM_020089769.1	calsequestrin-1-like isoform X5	1649	0
XM_020089770.1	potassium voltage-gated channel subfamily E member 1	538	3.44E-86
XM_020089771.1	olfactory receptor 10A6-like	975	0
XM_020089772.1	olfactory receptor 11H6-like	930	0
XM_020089773.1	tumor necrosis factor receptor superfamily member 6-like isoform X1	1130	0
XM_020089774.1	solute carrier family 15 member 2-like	2409	0
XM_020089775.1	dynein light chain Tctex-type 3	816	4.34E-79
XM_020089776.1	collagen alpha-2(IV) chain isoform X1	6179	0
XM_020089777.1	collagen alpha-2(IV) chain isoform X2	5258	0
XM_020089778.1	ATP-dependent (S)-NAD(P)H-hydrate dehydratase isoform X1	2582	0
XM_020089779.1	ATP-dependent (S)-NAD(P)H-hydrate dehydratase isoform X2	1685	0
XM_020089780.1	ATP-dependent (S)-NAD(P)H-hydrate dehydratase isoform X3	1387	0
XM_020089781.1	ATP-dependent (S)-NAD(P)H-hydrate dehydratase isoform X4	1553	0
XM_020089782.1	tumor necrosis factor receptor superfamily member 6-like isoform X2	1173	0
XM_020089783.1	ATP-dependent (S)-NAD(P)H-hydrate dehydratase isoform X1	1573	0
XM_020089784.1	inhibitor of growth protein 1	1387	0
XM_020089785.1	inhibitor of growth protein 1	1341	0
XM_020089786.1	ras-related protein Rab-5A-like	1542	4.36E-173
XM_020089787.1	ubiquitin-conjugating enzyme E2 A-like	1326	5.97E-108
XM_020089788.1	ankyrin repeat and SOCS box protein 9-like isoform X1	1469	0
XM_020089789.1	ankyrin repeat and SOCS box protein 9-like isoform X2	1473	0
XM_020089790.1	ankyrin repeat and SOCS box protein 9-like isoform X3	1366	2.32E-179
XM_020089791.1	tumor necrosis factor receptor superfamily member 26-like isoform X3	1204	0
XM_020089792.1	homeobox protein DLX-2	1720	0
XM_020089793.1	potassium voltage-gated channel subfamily H member 7	4415	0
XM_020089794.1	death-associated protein-like 1	822	8.01E-69
XM_020089795.1	arylsulfatase G	2384	0
XM_020089796.1	transmembrane protein 41A	1443	2.32E-155
XM_020089797.1	TRAF family member-associated NF-kappa-B activator	1519	0
XM_020089798.1	glycine receptor subunit alpha-2 isoform X1	3116	0
XM_020089799.1	glycine receptor subunit alpha-2 isoform X2	2702	0
XM_020089800.1	E3 ubiquitin-protein ligase Midline-1-like	1948	0
XM_020089801.1	E3 ubiquitin-protein ligase Midline-1-like	1799	0
XM_020089802.1	E3 ubiquitin-protein ligase Midline-1-like	1776	0
XM_020089803.1	GTP cyclohydrolase 1-like	1410	2.06E-166
XM_020089804.1	E3 ubiquitin-protein ligase Midline-1-like	1781	0
XM_020089805.1	membrane transport protein XK	914	1.43E-121
XM_020089806.1	homeobox protein Hox-D12	1855	1.41E-179
XM_020089807.1	kelch-like protein 41	2384	0
XM_020089808.1	histamine N-methyltransferase-like	1620	0
XM_020089809.1	histamine N-methyltransferase-like	1535	0
XM_020089810.1	rhodopsin kinase-like	2350	0
XM_020089811.1	homeobox protein DLX-1	1879	3.20E-141
XM_020089812.1	eomesodermin homolog isoform X3	2715	0
XM_020089813.1	kelch-like protein 6	3117	0
XM_020089814.1	kelch-like protein 6	3040	0
XM_020089815.1	teleost multiple tissue opsin-1b	1667	0
XM_020089816.1	DNA excision repair protein ERCC-1 isoform X1	1354	0
XM_020089817.1	DNA excision repair protein ERCC-1 isoform X2	1285	0
XM_020089818.1	cytochrome b561	1226	0
XM_020089819.1	zinc finger protein Helios-like	2051	0
XM_020089820.1	histamine N-methyltransferase-like	1088	0
XM_020089821.1	sorting nexin-4-like	1123	0
XM_020089822.1	alpha-1,6-mannosylglycoprotein 6-beta-N-acetylglucosaminyltransferase A-like isoform X1	2741	0
XM_020089823.1	alpha-1,6-mannosylglycoprotein 6-beta-N-acetylglucosaminyltransferase A-like isoform X1	2620	0
XM_020089824.1	cholecystokinin receptor-like	3255	0
XM_020089825.1	testis-expressed sequence 30 protein isoform X1	1619	1.22E-169
XM_020089826.1	testis-expressed sequence 30 protein isoform X2	1514	1.52E-153
XM_020089827.1	oligodendrocyte transcription factor 3	1775	2.08E-119
XM_020089828.1	testis-expressed sequence 30 protein isoform X3	1426	1.35E-106
XM_020089829.1	NEDD8-conjugating enzyme UBE2F-like	1884	3.07E-83
XM_020089830.1	NEDD8-conjugating enzyme UBE2F-like	2043	1.01E-82
XM_020089831.1	NEDD8-conjugating enzyme UBE2F-like	1701	6.87E-84
XM_020089832.1	acid-sensing ion channel 4 isoform X1	2292	0
XM_020089833.1	acid-sensing ion channel 4 isoform X2	2283	0
XM_020089834.1	zinc finger protein 888-like	1641	1.74E-177
XM_020089835.1	putative fidgetin-like protein 2	2787	0
XM_020089836.1	putative fidgetin-like protein 2	2907	0
XM_020089837.1	transmembrane protein 163-like	2366	2.18E-164
XM_020089838.1	pancreas transcription factor 1 subunit alpha-like isoform X1	2076	3.36E-136
XM_020089839.1	BAG family molecular chaperone regulator 2	1398	5.26E-147
XM_020089840.1	helix-loop-helix protein 13-like isoform X2	1783	1.84E-120
XM_020089841.1	E3 ubiquitin-protein ligase MARCH9-like	1917	0
XM_020089842.1	high-affinity choline transporter 1-like	3031	0
XM_020089843.1	PREDICTED: neurexophilin-2	2256	7.06E-176
XM_020089844.1	Bardet-Biedl syndrome 5 protein	1108	0
XM_020089845.1	twist-related protein 2-like	948	9.11E-78
XM_020089846.1	2-amino-3-carboxymuconate-6-semialdehyde decarboxylase isoform X1	1491	0
XM_020089847.1	2-amino-3-carboxymuconate-6-semialdehyde decarboxylase isoform X2	1834	0

XM_020089848.1	tumor necrosis factor ligand superfamily member 13B	1864	5.44E-165
XM_020089849.1	endothelin B receptor-like	1281	0
XM_020089850.1	motor neuron and pancreas homeobox protein 1-like	1951	0
XM_020089851.1	transcription elongation factor B polypeptide 3-like isoform X1	2310	0
XM_020089852.1	PREDICTED: claudin-14	1485	2.89E-113
XM_020089853.1	PREDICTED: claudin-14	1638	1.43E-112
XM_020089854.1	PREDICTED: claudin-14	1299	3.77E-114
XM_020089855.1	beta-1,3-galactosyltransferase 2-like	2513	0
XM_020089856.1	leukocyte cell-derived chemotaxin 1-like	1523	0
XM_020089857.1	oral-facial-digital syndrome 1 protein isoform X1	3495	0
XM_020089858.1	oral-facial-digital syndrome 1 protein isoform X2	3342	0
XM_020089859.1	transcription elongation factor B polypeptide 3-like isoform X2	2253	0
XM_020089860.1	epidermal growth factor-like protein 6 isoform X1	2530	0
XM_020089861.1	epidermal growth factor-like protein 6 isoform X2	2466	0
XM_020089862.1	epidermal growth factor-like protein 6 isoform X3	2342	0
XM_020089863.1	neuronal membrane glycoprotein M6-a	3046	0
XM_020089864.1	myelin proteolipid protein-like isoform X2	2818	3.45E-171
XM_020089865.1	ras-related protein Rab-9A-like	2017	2.60E-134
XM_020089866.1	trafficking protein particle complex subunit 2	650	3.08E-102
XM_020089867.1	thymosin beta-4	608	7.64E-09
XM_020089868.1	glucose-6-phosphatase 2 isoform X1	1262	0
XM_020089869.1	glucose-6-phosphatase 2 isoform X2	1259	0
XM_020089870.1	glucose-6-phosphatase 2 isoform X3	1247	0
XM_020089871.1	coiled-coil domain-containing protein 89	1336	0
XM_020089872.1	3-hydroxyanthranilate 3,4-dioxygenase	1352	0
XM_020089873.1	F-box only protein 47	1693	0
XM_020089874.1	F-box only protein 47	1749	0
XM_020089875.1	anterior gradient protein 3-like	777	5.03E-120
XM_020089876.1	5-hydroxytryptamine receptor 1D	1323	0
XM_020089877.1	transmembrane protein FAM155A	2087	0
XM_020089878.1	protein lifeguard 3-like isoform X1	1340	0
XM_020089879.1	protein lifeguard 3-like isoform X2	1214	9.91E-175
XM_020089880.1	PREDICTED: uncharacterized protein LOC109631217	2384	0
XM_020089881.1	coiled-coil domain-containing protein 173-like	2219	0
XM_020089882.1	PREDICTED: uncharacterized protein PF11_0207-like	1395	0
XM_020089883.1	cytohesin-interacting protein	1407	0
XM_020089884.1	putative pre-mRNA-splicing factor ATP-dependent RNA helicase DHX32	3071	0
XM_020089885.1	protein reprimo	807	2.61E-68
XM_020089886.1	putative sodium-coupled neutral amino acid transporter 11	1672	0
XM_020089887.1	stress-associated endoplasmic reticulum protein 2	429	9.09E-43
XM_020089888.1	cell surface A33 antigen-like	1688	0
XM_020089889.1	homeobox even-skipped homolog protein 2	1378	0
XM_020089890.1	zinc transporter ZIP9-like	1780	5.17E-166
XM_020089891.1	PREDICTED: melanoregulin-like	657	1.75E-148
XM_020089892.1	phosphatidylinositol N-acetylglucosaminyltransferase subunit A isoform X1	2068	0
XM_020089893.1	phosphatidylinositol N-acetylglucosaminyltransferase subunit A isoform X2	1808	0
XM_020089894.1	GA-binding protein alpha chain	2029	0
XM_020089895.1	GA-binding protein alpha chain	2007	0
XM_020089896.1	gastrin-releasing peptide receptor-like	1083	0
XM_020089897.1	ankyrin repeat and SOCS box protein 11-like isoform X1	1053	0
XM_020089898.1	ankyrin repeat and SOCS box protein 11-like isoform X2	1054	0
XM_020089899.1	splicing factor U2AF 35 kDa subunit	1135	5.37E-112
XM_020089900.1	amyloid beta A4 protein	4386	0
XM_020089901.1	ectodysplasin-A receptor-associated adapter protein isoform X1	2008	2.82E-145
XM_020089902.1	protein dopey-2	8232	0
XM_020089903.1	MORC family CW-type zinc finger protein 3	3397	0
XM_020089904.1	chromatin assembly factor 1 subunit B	2486	0
XM_020089905.1	sodium channel protein type 1 subunit alpha-like	7332	0
XM_020089906.1	tetratricopeptide repeat protein 21B	4049	0
XM_020089907.1	polypeptide N-acetylgalactosaminyltransferase 6-like	3991	0
XM_020089908.1	E3 SUMO-protein ligase RanBP2-like isoform X1	9614	0
XM_020089909.1	ectodysplasin-A receptor-associated adapter protein isoform X2	2005	2.70E-144
XM_020089910.1	E3 SUMO-protein ligase RanBP2-like isoform X2	9587	0
XM_020089911.1	unconventional myosin-Ib isoform X1	5925	0
XM_020089912.1	unconventional myosin-Ib isoform X2	5838	0
XM_020089913.1	unconventional myosin-Ib isoform X3	5750	0
XM_020089914.1	unconventional myosin-Ib isoform X4	5663	0
XM_020089915.1	basic leucine zipper and W2 domain-containing protein 1-A-like	2050	0
XM_020089916.1	homeobox protein Hox-A3	1736	0
XM_020089917.1	homeobox protein Hox-A10	2401	0
XM_020089918.1	ectodysplasin-A receptor-associated adapter protein isoform X3	1903	4.11E-118
XM_020089919.1	homeobox protein Hox-D9	1503	4.57E-154
XM_020089920.1	homeobox protein Hox-D4	3448	0
XM_020089921.1	homeobox protein Hox-D11a-like	1404	1.05E-176
XM_020089922.1	neurabin-1-like isoform X1	4850	0
XM_020089923.1	neurabin-1-like isoform X1	4887	0
XM_020089924.1	neurabin-1-like isoform X1	5477	0
XM_020089925.1	neurabin-1-like isoform X1	4385	0
XM_020089926.1	neurabin-1-like isoform X1	4268	0

XM_020089927.1	pyruvate dehydrogenase (acetyl-transferring) kinase isozyme 1, mitochondrial	3645	0
XM_020089928.1	E3 ubiquitin-protein ligase UBR3 isoform X1	8097	0
XM_020089929.1	E3 ubiquitin-protein ligase UBR3 isoform X2	7815	0
XM_020089930.1	myelin and lymphocyte protein-like	896	6.75E-110
XM_020089931.1	E3 ubiquitin-protein ligase UBR3 isoform X3	7785	0
XM_020089932.1	kelch-like protein 23	2636	0
XM_020089933.1	lupus La protein	1806	0
XM_020089934.1	SH3 domain-containing RING finger protein 3 isoform X1	6654	0
XM_020089935.1	SH3 domain-containing RING finger protein 3 isoform X2	6585	0
XM_020089936.1	ankyrin repeat domain-containing protein SOWAHC	2223	0
XM_020089937.1	septin-6 isoform X3	1272	0
XM_020089938.1	serine/threonine-protein kinase tousled-like 1	3551	0
XM_020089939.1	DDB1- and CUL4-associated factor 17	2208	0
XM_020089940.1	DDB1- and CUL4-associated factor 17	2240	0
XM_020089941.1	DDB1- and CUL4-associated factor 17	2259	0
XM_020089942.1	myelin and lymphocyte protein-like	860	4.33E-97
XM_020089943.1	Golgi reassembly-stacking protein 2	2005	0
XM_020089944.1	methyltransferase-like protein 8 isoform X1	1692	0
XM_020089945.1	methyltransferase-like protein 8 isoform X2	1559	0
XM_020089946.1	methyltransferase-like protein 8 isoform X3	1600	0
XM_020089947.1	INO80 complex subunit D isoform X1	4668	0
XM_020089948.1	INO80 complex subunit D isoform X1	3519	0
XM_020089949.1	NADH-ubiquinone oxidoreductase 75 kDa subunit, mitochondrial-like	2640	0
XM_020089950.1	G-protein coupled receptor 1	1922	0
XM_020089951.1	B-cell CLL/lymphoma 9 protein-like	5248	0
XM_020089952.1	mucin-5AC-like isoform X1	2354	0
XM_020089953.1	B-cell CLL/lymphoma 9 protein-like	5165	0
XM_020089954.1	B-cell CLL/lymphoma 9 protein-like	5244	0
XM_020089955.1	B-cell CLL/lymphoma 9 protein-like	5092	0
XM_020089956.1	B-cell CLL/lymphoma 9 protein-like	4865	0
XM_020089957.1	B-cell CLL/lymphoma 9 protein-like	5362	0
XM_020089958.1	B-cell CLL/lymphoma 9 protein-like	5851	0
XM_020089959.1	UDP-glucuronosyltransferase 2A2-like	2175	0
XM_020089960.1	general transcription factor IIE subunit 1	2532	0
XM_020089961.1	general transcription factor IIE subunit 1	2525	0

XM_020089962.1	mucin-5AC-like isoform X1	2174	0
XM_020089963.1	granzyme B(G,H)-like	565	7.17E-91
XM_020089964.1	rab-like protein 3	1541	3.32E-161
XM_020089965.1	coiled-coil domain-containing protein 148	2478	0
XM_020089966.1	coiled-coil domain-containing protein 148	2505	0
XM_020089967.1	coiled-coil domain-containing protein 148	2492	0
XM_020089968.1	uridine phosphorylase 2	1836	0
XM_020089969.1	PREDICTED: uncharacterized protein C7orf57 homolog	1135	1.51E-139
XM_020089970.1	serine/arginine repetitive matrix protein 1-like	5973	4.11E-125
XM_020089971.1	UDP-glucose:glycoprotein glucosyltransferase 2 isoform X1	6305	0
XM_020089972.1	UDP-glucose:glycoprotein glucosyltransferase 2 isoform X2	6188	0
XM_020089973.1	heparan-sulfate 6-O-sulfotransferase 3	1651	0
XM_020089974.1	PREDICTED: frizzled-7-A-like	4555	0
XM_020089975.1	dedicator of cytokinesis protein 9 isoform X1	7708	0
XM_020089976.1	dedicator of cytokinesis protein 9 isoform X2	7702	0
XM_020089977.1	dedicator of cytokinesis protein 9 isoform X3	7454	0
XM_020089978.1	protein Wnt-8b	2044	0
XM_020089979.1	dedicator of cytokinesis protein 9 isoform X4	7678	0
XM_020089980.1	dedicator of cytokinesis protein 9 isoform X5	7672	0
XM_020089981.1	dedicator of cytokinesis protein 9 isoform X6	7660	0
XM_020089982.1	dedicator of cytokinesis protein 9 isoform X7	7406	0
XM_020089983.1	dedicator of cytokinesis protein 9 isoform X8	7269	0
XM_020089984.1	dedicator of cytokinesis protein 9 isoform X9	7624	0
XM_020089985.1	dedicator of cytokinesis protein 9 isoform X10	7370	0
XM_020089986.1	integrin alpha-6 isoform X1	4019	0
XM_020089987.1	integrin alpha-6 isoform X1	3491	0
XM_020089988.1	nuclear pore glycoprotein p62	1941	6.71E-169
XM_020089989.1	gap junction gamma-1 protein-like	2765	0
XM_020089990.1	gap junction gamma-1 protein-like	2866	0
XM_020089991.1	lactase-phlorizin hydrolase-like	5984	0
XM_020089992.1	protein FAM204A isoform X1	2230	4.89E-140
XM_020089993.1	glycogen synthase kinase-3 beta isoform X1	5900	0
XM_020089994.1	glycogen synthase kinase-3 beta isoform X2	5887	0
XM_020089995.1	glycogen synthase kinase-3 beta isoform X3	5875	0
XM_020089996.1	glycogen synthase kinase-3 beta isoform X4	5863	0
XM_020089997.1	protein Daple-like	5230	0
XM_020089998.1	TSC22 domain family protein 1-like isoform X1	4778	0
XM_020089999.1	TSC22 domain family protein 1-like isoform X2	2078	1.37E-70
XM_020090000.1	TSC22 domain family protein 1-like isoform X3	2044	1.28E-76
XM_020090001.1	dual 3',5'-cyclic-AMP and -GMP phosphodiesterase 11A	3841	0
XM_020090002.1	protein FAM204A isoform X2	2222	2.23E-139
XM_020090003.1	alkyldihydroxyacetonephosphate synthase, peroxisomal	2508	0
XM_020090004.1	cytochrome c-a-like	874	1.29E-72
XM_020090005.1	cyclin-T2-like isoform X2	4002	0

XM_020090006.1	cyclin-T2-like isoform X2	3848	0
XM_020090007.1	POU domain, class 2, transcription factor 1 isoform X1	8452	0
XM_020090008.1	POU domain, class 2, transcription factor 1 isoform X2	8449	0
XM_020090009.1	POU domain, class 2, transcription factor 1 isoform X3	8443	0
XM_020090010.1	POU domain, class 2, transcription factor 1 isoform X4	8440	0
XM_020090011.1	protein FAM204A isoform X3	2229	1.29E-138
XM_020090012.1	POU domain, class 2, transcription factor 1 isoform X5	8798	0
XM_020090013.1	POU domain, class 2, transcription factor 1 isoform X6	8437	0
XM_020090014.1	POU domain, class 2, transcription factor 1 isoform X7	8425	0
XM_020090015.1	POU domain, class 2, transcription factor 1 isoform X8	8386	0
XM_020090016.1	transmembrane protein 223	1341	4.50E-165
XM_020090017.1	GRIP and coiled-coil domain-containing protein 2	6694	0
XM_020090018.1	1-acyl-sn-glycerol-3-phosphate acyltransferase gamma-like	5027	0
XM_020090019.1	myosin light chain 1, skeletal muscle isoform	1179	2.86E-111
XM_020090020.1	protein FAM204A isoform X4	2223	6.42E-138
XM_020090021.1	calcium-binding mitochondrial carrier protein Aralar1 isoform X1	2525	0
XM_020090022.1	calcium-binding mitochondrial carrier protein Aralar1 isoform X1	2640	0
XM_020090023.1	calcium-binding mitochondrial carrier protein Aralar1 isoform X3	2363	0
XM_020090024.1	cytoplasmic dynein 1 intermediate chain 2-like isoform X2	2792	0
XM_020090025.1	cytoplasmic dynein 1 intermediate chain 2-like isoform X2	2732	0
XM_020090026.1	tenascin-like isoform X4	8738	0
XM_020090027.1	WD repeat, SAM and U-box domain-containing protein 1-like isoform X1	2368	0
XM_020090028.1	WD repeat, SAM and U-box domain-containing protein 1-like isoform X2	2388	0
XM_020090029.1	WD repeat, SAM and U-box domain-containing protein 1-like isoform X3	2361	0
XM_020090030.1	gap junction alpha-3 protein-like	3005	0
XM_020090031.1	NK1 transcription factor-related protein 2	1497	0
XM_020090032.1	extended synaptotagmin-3-like	4532	0
XM_020090033.1	desmin-like isoform X1	2266	0
XM_020090034.1	desmin-like isoform X2	2226	0
XM_020090035.1	transmembrane protein 198-B-like	4600	6.72E-136
XM_020090036.1	transmembrane protein 198-B-like	4596	6.63E-136
XM_020090037.1	transmembrane protein 198-B-like	4243	1.25E-136
XM_020090038.1	beta-galactoside alpha-2,6-sialyltransferase 2	5883	0
XM_020090039.1	beta-galactoside alpha-2,6-sialyltransferase 2	5581	0
XM_020090040.1	band 4.1-like protein 5 isoform X1	4544	0
XM_020090041.1	band 4.1-like protein 5 isoform X2	4493	0
XM_020090042.1	band 4.1-like protein 5 isoform X3	4490	0
XM_020090043.1	phosphoinositide 3-kinase adapter protein 1 isoform X1	3058	0

XM_020090044.1	formin-like protein 2	5440	0
XM_020090045.1	formin-like protein 2	3244	0
XM_020090046.1	peptidyl-prolyl cis-trans isomerase G	3926	0
XM_020090047.1	peptidyl-prolyl cis-trans isomerase G	3851	0
XM_020090048.1	coiled-coil domain-containing protein 173-like	3097	0
XM_020090049.1	activin receptor type-1-like isoform X1	3349	0
XM_020090050.1	mitogen-activated protein kinase kinase kinase MLT isoform X1	2879	0
XM_020090051.1	mitogen-activated protein kinase kinase kinase MLT isoform X2	2904	0
XM_020090052.1	phosphoinositide 3-kinase adapter protein 1 isoform X2	3052	0
XM_020090053.1	cell division cycle-associated protein 7	3220	0
XM_020090054.1	PREDICTED: sacsin-like	13724	0
XM_020090055.1	tyrosine-protein phosphatase non-receptor type 4	6900	0
XM_020090056.1	ATP-dependent 6-phosphofructokinase, liver type-like isoform X1	3141	0
XM_020090057.1	ATP-dependent 6-phosphofructokinase, liver type-like isoform X2	3123	0
XM_020090058.1	transmembrane protein 169	2576	0
XM_020090059.1	transmembrane protein 169	2624	0
XM_020090060.1	phosphoinositide 3-kinase adapter protein 1 isoform X3	2631	0
XM_020090061.1	collagen alpha-1(XVIII) chain-like isoform X1	5931	0
XM_020090062.1	collagen alpha-1(XVIII) chain-like isoform X1	5889	0
XM_020090063.1	collagen alpha-1(XVIII) chain-like isoform X1	5703	0
XM_020090064.1	hyccin isoform X1	5759	0
XM_020090065.1	lysosome-associated membrane glycoprotein 1-like	2948	2.10E-142
XM_020090066.1	F-box-like/WD repeat-containing protein TBL1XR1 isoform X1	3663	0
XM_020090067.1	disco-interacting protein 2 homolog A-like isoform X1	7605	0
XM_020090068.1	disco-interacting protein 2 homolog A-like isoform X2	7596	0
XM_020090069.1	disco-interacting protein 2 homolog A-like isoform X3	7575	0
XM_020090070.1	disco-interacting protein 2 homolog A-like isoform X4	7566	0
XM_020090071.1	disco-interacting protein 2 homolog A-like isoform X5	7563	0
XM_020090072.1	disco-interacting protein 2 homolog A-like isoform X6	7533	0
XM_020090073.1	androgen-induced gene 1 protein-like	903	8.30E-146
XM_020090074.1	corepressor interacting with RBPJ 1	2034	1.93E-141
XM_020090075.1	transcription factor Sp8 isoform X2	1908	0
XM_020090076.1	obg-like ATPase 1	1698	0
XM_020090077.1	partitioning defective 3 homolog B isoform X1	8834	0
XM_020090078.1	partitioning defective 3 homolog B isoform X2	8831	0
XM_020090079.1	partitioning defective 3 homolog B isoform X3	8807	0
XM_020090080.1	partitioning defective 3 homolog B isoform X4	4467	0
XM_020090081.1	PREDICTED: striated muscle preferentially expressed protein kinase	15191	0
XM_020090082.1	CXC receptor 2	1337	0
XM_020090083.1	serum deprivation-response protein-like	5156	0
XM_020090084.1	collagen alpha-2(VI) chain-like isoform X1	3442	0

XM_020090085.1	collagen alpha-2(VI) chain-like isoform X2	3448	0
XM_020090086.1	collagen alpha-2(VI) chain-like isoform X3	3442	0
XM_020090087.1	X-ray repair cross-complementing protein 5	2877	0
XM_020090088.1	motile sperm domain-containing protein 2	2197	0
XM_020090089.1	PREDICTED: uncharacterized protein LOC109631375	2109	0
XM_020090090.1	ankyrin repeat and zinc finger domain-containing protein 1 isoform X1	2953	0
XM_020090091.1	ankyrin repeat and zinc finger domain-containing protein 1 isoform X2	2950	0
XM_020090092.1	ankyrin repeat and zinc finger domain-containing protein 1 isoform X1	2737	0
XM_020090093.1	ankyrin repeat and zinc finger domain-containing protein 1 isoform X4	2540	0
XM_020090094.1	ankyrin repeat and zinc finger domain-containing protein 1 isoform X5	2515	0
XM_020090095.1	ankyrin repeat and zinc finger domain-containing protein 1 isoform X1	2440	0
XM_020090096.1	beta-galactosidase-1-like protein 2	2286	0
XM_020090097.1	brachyury protein isoform X1	1797	0
XM_020090098.1	low choriolytic enzyme-like	1275	0
XM_020090099.1	high choriolytic enzyme 2-like	928	0
XM_020090100.1	xin actin-binding repeat-containing protein 2 isoform X1	10489	0
XM_020090101.1	xin actin-binding repeat-containing protein 2 isoform X2	10465	0
XM_020090102.1	serine/threonine-protein kinase 24-like isoform X1	4710	0
XM_020090103.1	serine/threonine-protein kinase 24-like isoform X2	1782	0
XM_020090104.1	serine/threonine-protein kinase 24-like isoform X3	4648	0
XM_020090105.1	polypeptide N-acetylgalactosaminyltransferase 5	3632	0
XM_020090106.1	brachyury protein isoform X2	1736	0
XM_020090107.1	PREDICTED: ermin	1396	0
XM_020090108.1	ras-related protein Ral-B	2379	3.06E-134
XM_020090109.1	mitogen-activated protein kinase kinase kinase 13	6386	0
XM_020090110.1	mitogen-activated protein kinase kinase kinase 13	6301	0
XM_020090111.1	double-stranded RNA-specific editase 1 isoform X1	3220	0
XM_020090112.1	double-stranded RNA-specific editase 1 isoform X2	3210	0
XM_020090113.1	unconventional myosin-X-like isoform X1	8093	0
XM_020090114.1	unconventional myosin-X-like isoform X2	8090	0
XM_020090115.1	ephrin type-A receptor 6	3394	0
XM_020090116.1	PREDICTED: syntaxin-19	2212	0
XM_020090117.1	PREDICTED: syntaxin-19	2344	0
XM_020090118.1	PREDICTED: uncharacterized protein LOC109631389	884	6.50E-179
XM_020090119.1	NAD-dependent malic enzyme, mitochondrial	3547	0
XM_020090120.1	NAD-dependent malic enzyme, mitochondrial	3507	0
XM_020090121.1	Na(+)/H(+) exchange regulatory cofactor NHE-RF3	1991	0
XM_020090122.1	Golgi pH regulator	2042	0
XM_020090123.1	SLIT and NTRK-like protein 4	4965	0
XM_020090124.1	OX-2 membrane glycoprotein-like	3346	0
XM_020090125.1	cell surface glycoprotein CD200 receptor 1-like	1597	0
XM_020090126.1	histone deacetylase 4-like isoform X1	8297	0
XM_020090127.1	histone deacetylase 4-like isoform X2	8294	0
XM_020090128.1	histone deacetylase 4-like isoform X3	8234	0
XM_020090129.1	histone deacetylase 4-like isoform X4	8251	0
XM_020090130.1	fucose mutarotase	677	7.96E-96
XM_020090131.1	poly [ADP-ribose] polymerase 9 isoform X1	3334	0
XM_020090132.1	poly [ADP-ribose] polymerase 9 isoform X1	3266	0
XM_020090133.1	poly [ADP-ribose] polymerase 9 isoform X1	2245	0
XM_020090134.1	ATP-binding cassette sub-family A member 12	8512	0
XM_020090135.1	A-kinase anchor protein 17A isoform X2	4338	0
XM_020090136.1	A-kinase anchor protein 17A isoform X2	4542	0
XM_020090137.1	A-kinase anchor protein 17A isoform X2	4361	0
XM_020090138.1	ankyrin repeat domain-containing protein 10-like isoform X2	1567	0
XM_020090139.1	ankyrin repeat domain-containing protein 10-like isoform X2	1561	0
XM_020090140.1	leucine-rich repeat-containing protein 58	2618	0
XM_020090141.1	LIM and senescent cell antigen-like-containing domain protein 1 isoform X1	3240	0
XM_020090142.1	LIM and senescent cell antigen-like-containing domain protein 1 isoform X2	3046	0
XM_020090143.1	homeobox protein vent1-like	1169	1.34E-149
XM_020090144.1	LIM and senescent cell antigen-like-containing domain protein 1 isoform X3	3223	0
XM_020090145.1	LIM and senescent cell antigen-like-containing domain protein 1 isoform X4	1650	0
XM_020090146.1	LIM and senescent cell antigen-like-containing domain protein 1 isoform X5	3046	0
XM_020090147.1	LIM and senescent cell antigen-like-containing domain protein 1 isoform X3	2943	0
XM_020090148.1	neuropilin-2 isoform X1	6296	0
XM_020090149.1	neuropilin-2 isoform X2	5749	0
XM_020090150.1	neuropilin-2 isoform X3	6263	0
XM_020090151.1	receptor tyrosine-protein kinase erbB-4 isoform X1	7990	0
XM_020090152.1	receptor tyrosine-protein kinase erbB-4 isoform X1	7977	0
XM_020090153.1	receptor tyrosine-protein kinase erbB-4 isoform X1	8010	0
XM_020090154.1	receptor tyrosine-protein kinase erbB-4 isoform X1	7987	0
XM_020090155.1	integral membrane protein GPR155	3734	0
XM_020090156.1	integral membrane protein GPR155	3653	0
XM_020090157.1	homeobox protein vent1-like	602	1.76E-100
XM_020090158.1	PREDICTED: secernin-3	2452	0
XM_020090159.1	PREDICTED: secernin-3	2455	0
XM_020090160.1	collagen alpha-1(XXVIII) chain-like isoform X1	4613	0
XM_020090161.1	collagen alpha-1(XXVIII) chain-like isoform X2	4595	0
XM_020090162.1	transcription factor Dp-1 isoform X1	2613	0
XM_020090163.1	transcription factor Dp-1 isoform X1	2615	0

XM_020090164.1	transcription factor Dp-1 isoform X1	2449	0
XM_020090165.1	transcription factor Dp-1 isoform X1	2408	0
XM_020090166.1	transcription factor Dp-1 isoform X1	2585	0
XM_020090167.1	transcription factor Dp-1 isoform X1	2587	0
XM_020090168.1	transcription factor Dp-1 isoform X1	2425	0
XM_020090169.1	SOSS complex subunit B2-like	3098	1.98E-149
XM_020090170.1	insulin receptor substrate 2	7412	0
XM_020090171.1	sprouty-related, EVH1 domain-containing protein 2	1044	7.63E-117
XM_020090172.1	growth hormone-regulated TBC protein 1-A-like	1723	0
XM_020090173.1	DCN1-like protein 1 isoform X1	1124	0
XM_020090174.1	DCN1-like protein 1 isoform X2	920	0
XM_020090175.1	protein ABHD12B	4201	0
XM_020090176.1	mannose-1-phosphate guanyltransferase alpha isoform X1	3066	0
XM_020090177.1	mannose-1-phosphate guanyltransferase alpha isoform X2	3049	0
XM_020090178.1	zinc finger protein 148 isoform X1	2647	0
XM_020090179.1	zinc finger protein 148 isoform X1	2541	0
XM_020090180.1	zinc finger protein 148 isoform X1	6814	0
XM_020090181.1	zinc finger protein 148 isoform X1	2643	0
XM_020090182.1	FERM, RhoGEF and pleckstrin domain-containing protein 1-like	4815	0
XM_020090183.1	FERM, RhoGEF and pleckstrin domain-containing protein 1-like	4819	0
XM_020090184.1	dnaJ homolog subfamily C member 5-like isoform X1	1010	1.49E-122
XM_020090185.1	RNA-binding motif, single-stranded-interacting protein 2-like isoform X1	2434	0
XM_020090186.1	SWI/SNF-related matrix-associated actin-dependent regulator of chromatin subfamily A-like protein 1	3539	0
XM_020090187.1	thioredoxin domain-containing protein 3 homolog isoform X1	2150	0
XM_020090188.1	thioredoxin domain-containing protein 3 homolog isoform X2	2147	0
XM_020090189.1	thioredoxin domain-containing protein 3 homolog isoform X3	2122	0
XM_020090190.1	BRCA1-associated RING domain protein 1-like isoform X1	2674	0
XM_020090191.1	BRCA1-associated RING domain protein 1-like isoform X2	2528	0
XM_020090192.1	von Willebrand factor C domain-containing protein 2-like isoform X1	5670	1.20E-143
XM_020090193.1	von Willebrand factor C domain-containing protein 2-like isoform X2	5769	1.27E-168
XM_020090194.1	zinc finger protein 654	5738	0
XM_020090195.1	thioredoxin reductase 1, cytoplasmic-like	3657	0
XM_020090196.1	NFU1 iron-sulfur cluster scaffold homolog, mitochondrial-like	858	7.66E-176
XM_020090197.1	thioredoxin reductase 1, cytoplasmic-like	3655	0
XM_020090198.1	thioredoxin reductase 1, cytoplasmic-like	3309	0
XM_020090199.1	zinc transporter ZIP10-like	3400	0
XM_020090200.1	glypican-6 isoform X1	3944	0
XM_020090201.1	glypican-6 isoform X2	3914	0
XM_020090202.1	glypican-6 isoform X3	2136	0
XM_020090203.1	glypican-6 isoform X1	3737	0
XM_020090204.1	L-dopachrome tautomerase	1981	0
XM_020090205.1	muscleblind-like protein 2a isoform X1	4572	0
XM_020090206.1	muscleblind-like protein 2a isoform X1	4545	0
XM_020090207.1	muscleblind-like protein 2a isoform X1	4667	0
XM_020090208.1	muscleblind-like protein 2a isoform X1	4518	0
XM_020090209.1	muscleblind-like protein 2a isoform X4	4625	0
XM_020090210.1	long wavelength sensitive opsin	1208	0
XM_020090211.1	muscleblind-like protein 2a isoform X5	4613	0
XM_020090212.1	translationally-controlled tumor protein	1071	1.64E-122
XM_020090213.1	PREDICTED: augurin	699	1.02E-105
XM_020090214.1	UDP-glucuronic acid decarboxylase 1	2556	0
XM_020090215.1	asparagine--tRNA ligase, cytoplasmic isoform X1	2233	0
XM_020090216.1	multidrug resistance-associated protein 4 isoform X1	5288	0
XM_020090217.1	multidrug resistance-associated protein 4 isoform X1	5733	0
XM_020090218.1	multidrug resistance-associated protein 4 isoform X1	5333	0
XM_020090219.1	multidrug resistance-associated protein 4 isoform X1	5177	0
XM_020090220.1	multidrug resistance-associated protein 4 isoform X1	5125	0
XM_020090221.1	multidrug resistance-associated protein 4 isoform X1	5195	0
XM_020090222.1	multidrug resistance-associated protein 4 isoform X1	4787	0
XM_020090223.1	multidrug resistance-associated protein 4 isoform X1	4893	0
XM_020090224.1	glutamate receptor ionotropic, delta-1	3488	0
XM_020090225.1	multidrug resistance-associated protein 4 isoform X1	4752	0
XM_020090226.1	multidrug resistance-associated protein 4 isoform X1	4705	0
XM_020090227.1	multidrug resistance-associated protein 4 isoform X1	5077	0
XM_020090228.1	pyridoxal kinase	2470	0
XM_020090229.1	D-3-phosphoglycerate dehydrogenase	1983	0
XM_020090230.1	CD99 antigen-like isoform X1	4326	8.29E-87
XM_020090231.1	nuclear factor erythroid 2-related factor 1	2390	0
XM_020090232.1	oxysterol-binding protein-related protein 6 isoform X1	5994	0
XM_020090233.1	oxysterol-binding protein-related protein 6 isoform X2	5901	0
XM_020090234.1	G-protein coupled receptor 161	3114	0
XM_020090235.1	transcription factor Sp3	3806	0
XM_020090236.1	immunoglobulin-like domain-containing receptor 2 isoform X1	2586	0
XM_020090237.1	immunoglobulin-like domain-containing receptor 2 isoform X2	2530	0
XM_020090238.1	cordon-bleu protein-like 1 isoform X2	4303	0
XM_020090239.1	cordon-bleu protein-like 1 isoform X2	3486	0
XM_020090240.1	cordon-bleu protein-like 1 isoform X2	3476	0
XM_020090241.1	cordon-bleu protein-like 1 isoform X2	3404	0
XM_020090242.1	PREDICTED: glucagon	558	2.70E-87

XM_020090243.1	sodium-driven chloride bicarbonate exchanger	2823	0
XM_020090244.1	heterogeneous nuclear ribonucleoprotein A3 isoform X1	1935	1.65E-135
XM_020090245.1	heterogeneous nuclear ribonucleoprotein A3 isoform X1	1923	1.46E-135
XM_020090246.1	sodium/myo-inositol cotransporter-like	4658	0
XM_020090247.1	ventral anterior homeobox 1	3014	0
XM_020090248.1	sodium/myo-inositol cotransporter-like	4603	0
XM_020090249.1	sodium/myo-inositol cotransporter-like	4590	0
XM_020090250.1	dehydrogenase/reductase SDR family member 9	1359	0
XM_020090251.1	ras-related protein Rap-2a	2561	1.87E-106
XM_020090252.1	SLIT and NTRK-like protein 5	5870	0
XM_020090253.1	SLIT and NTRK-like protein 5	5939	0
XM_020090254.1	SLIT and NTRK-like protein 5	5957	0
XM_020090255.1	SLIT and NTRK-like protein 5	5732	0
XM_020090256.1	pre-mRNA-processing factor 40 homolog A isoform X2	3048	0
XM_020090257.1	pre-mRNA-processing factor 40 homolog A isoform X2	2966	0
XM_020090258.1	speckle-type POZ protein-like	2879	0
XM_020090259.1	disintegrin and metalloproteinase domain-containing protein 23 isoform X1	4657	0
XM_020090260.1	fibulin-7-like isoform X2	2644	0
XM_020090261.1	disintegrin and metalloproteinase domain-containing protein 23 isoform X2	4419	0
XM_020090262.1	disintegrin and metalloproteinase domain-containing protein 23 isoform X3	4419	0
XM_020090263.1	disintegrin and metalloproteinase domain-containing protein 23 isoform X3	4510	0
XM_020090264.1	disintegrin and metalloproteinase domain-containing protein 23 isoform X5	2563	0
XM_020090265.1	disintegrin and metalloproteinase domain-containing protein 23 isoform X5	2526	0
XM_020090266.1	disintegrin and metalloproteinase domain-containing protein 23 isoform X6	4328	0
XM_020090267.1	ATP synthase F(0) complex subunit C3, mitochondrial-like	793	9.11E-78
XM_020090268.1	ATP synthase F(0) complex subunit C3, mitochondrial-like	774	7.36E-78
XM_020090269.1	growth factor receptor-bound protein 14 isoform X1	2869	0
XM_020090270.1	growth factor receptor-bound protein 14 isoform X1	2892	0
XM_020090271.1	growth factor receptor-bound protein 14 isoform X1	2900	0
XM_020090272.1	growth factor receptor-bound protein 14 isoform X1	2918	0
XM_020090273.1	growth factor receptor-bound protein 14 isoform X1	2982	0
XM_020090274.1	growth factor receptor-bound protein 14 isoform X1	2687	0
XM_020090275.1	synaptic vesicular amine transporter	1694	0
XM_020090276.1	growth factor receptor-bound protein 14 isoform X1	2598	0
XM_020090277.1	synaptotagmin-like protein 5 isoform X1	3572	0
XM_020090278.1	synaptotagmin-like protein 5 isoform X2	3566	0
XM_020090279.1	synaptotagmin-like protein 5 isoform X3	3563	0
XM_020090280.1	guanine nucleotide exchange factor VAV3-like isoform X1	1884	0
XM_020090281.1	kinetochore protein Spc25	1984	6.64E-149
XM_020090282.1	kinetochore protein Spc25	1974	2.95E-149
XM_020090283.1	peroxisomal bifunctional enzyme	3007	0
XM_020090284.1	synaptic vesicular amine transporter	1675	0
XM_020090285.1	charged multivesicular body protein 2b	2332	6.32E-147
XM_020090286.1	ceramide synthase 6 isoform X1	2267	0
XM_020090287.1	ceramide synthase 6 isoform X2	2247	0
XM_020090288.1	transcription factor SOX-21	2067	4.68E-134
XM_020090289.1	transformer-2 protein homolog beta isoform X3	1246	9.94E-69

XM_020090290.1	transformer-2 protein homolog beta isoform X3	1244	9.66E-69
XM_020090291.1	transformer-2 protein homolog beta isoform X3	1461	6.80E-68
XM_020090292.1	transformer-2 protein homolog beta isoform X3	1123	1.34E-69
XM_020090293.1	transformer-2 protein homolog beta isoform X3	1123	5.27E-69
XM_020090294.1	serine/threonine-protein kinase SIK2-like	4903	0
XM_020090295.1	polypeptide N-acetylgalactosaminyltransferase 13	3518	0
XM_020090296.1	unconventional myosin-XVI	8823	0
XM_020090297.1	inactive heparanase-2	2328	0
XM_020090298.1	PREDICTED: alsin-like	6871	0
XM_020090299.1	histone acetyltransferase type B catalytic subunit	1852	0
XM_020090300.1	immunoglobulin-like domain-containing receptor 1	2712	0
XM_020090301.1	tRNA selenocysteine 1-associated protein 1 isoform X1	1593	5.65E-174
XM_020090302.1	tRNA selenocysteine 1-associated protein 1 isoform X2	1587	0
XM_020090303.1	tRNA selenocysteine 1-associated protein 1 isoform X3	1578	3.56E-170
XM_020090304.1	syntaxin-12-like	1495	6.16E-167
XM_020090305.1	syntaxin-12-like	1357	1.17E-167
XM_020090306.1	transmembrane protein 26-like	2527	2.52E-173
XM_020090307.1	syntaxin-12-like	1362	1.26E-167
XM_020090308.1	PREDICTED: uncharacterized protein LOC109631493 isoform X1	3243	0
XM_020090309.1	PREDICTED: uncharacterized protein LOC109631493 isoform X1	3291	0
XM_020090310.1	PREDICTED: uncharacterized protein LOC109631493 isoform X1	3071	0
XM_020090311.1	PREDICTED: uncharacterized protein LOC109631493 isoform X2	3219	0
XM_020090312.1	PREDICTED: uncharacterized protein LOC109631493 isoform X3	2145	0
XM_020090313.1	SNF-related serine/threonine-protein kinase-like	4274	0
XM_020090314.1	SNF-related serine/threonine-protein kinase-like	4143	0
XM_020090315.1	zinc finger and BTB domain-containing protein 8B	2201	0
XM_020090316.1	splicing factor 3A subunit 3	1884	0
XM_020090317.1	fatty acid-binding protein, heart-like	754	7.79E-95
XM_020090318.1	trophoblast glycoprotein-like	3684	0
XM_020090319.1	arachidonate 5-lipoxygenase	2304	0
XM_020090320.1	glutathione S-transferase A-like	949	1.29E-170
XM_020090321.1	PREDICTED: uncharacterized protein LOC109631501 isoform X1	2832	0

XM_020090322.1	PREDICTED: uncharacterized protein LOC109631501 isoform X1	2803	0
XM_020090323.1	mitogen-activated protein kinase 15	2390	0
XM_020090324.1	copine-3-like isoform X2	3302	0
XM_020090325.1	peroxisome biogenesis factor 1 isoform X1	3780	0
XM_020090326.1	peroxisome biogenesis factor 1 isoform X2	3777	0
XM_020090327.1	3-oxo-5-alpha-steroid 4-dehydrogenase 1	2451	3.00E-180
XM_020090328.1	3-oxo-5-alpha-steroid 4-dehydrogenase 1	1523	0
XM_020090329.1	3-oxo-5-alpha-steroid 4-dehydrogenase 1	1969	0
XM_020090330.1	3-oxo-5-alpha-steroid 4-dehydrogenase 1	1918	0
XM_020090331.1	3-oxo-5-alpha-steroid 4-dehydrogenase 1	1378	0
XM_020090332.1	methylated-DNA--protein-cysteine methyltransferase isoform X1	1328	4.21E-140
XM_020090333.1	adenylate cyclase type 2 isoform X1	4617	0
XM_020090334.1	adenylate cyclase type 2 isoform X2	4151	0
XM_020090335.1	transcription factor Sp8-like	2188	0
XM_020090336.1	probable G-protein coupled receptor 174	1892	0
XM_020090337.1	RNA-binding protein 24	3037	9.57E-53
XM_020090338.1	tissue alpha-L-fucosidase-like	2017	0
XM_020090339.1	triple QxxK/R motif-containing protein	2125	1.96E-50
XM_020090340.1	E3 ubiquitin-protein ligase ZNRF2-like	1864	2.99E-127
XM_020090341.1	methylated-DNA--protein-cysteine methyltransferase isoform X2	1325	2.77E-139
XM_020090342.1	visinin-like protein 1	2272	1.61E-135
XM_020090343.1	tRNA dimethylallyltransferase, mitochondrial	2157	0
XM_020090344.1	glucocorticoid-induced transcript 1 protein	2367	0
XM_020090345.1	protein FAM8A1-like	3041	1.05E-171
XM_020090346.1	protein FAM8A1-like	2939	8.30E-172
XM_020090347.1	trafficking protein particle complex subunit 3	1378	2.78E-130
XM_020090348.1	cAMP-dependent protein kinase inhibitor beta	2487	1.61E-40
XM_020090349.1	cAMP-dependent protein kinase inhibitor beta	2060	1.11E-40
XM_020090350.1	protein CBFA2T1 isoform X1	4068	0
XM_020090351.1	protein CBFA2T1 isoform X2	4596	0
XM_020090352.1	switch-associated protein 70-like	1565	0
XM_020090353.1	protein CBFA2T1 isoform X1	3567	0
XM_020090354.1	39S ribosomal protein L3, mitochondrial	1296	0
XM_020090355.1	protein FAM133B	1593	9.00E-25
XM_020090356.1	cytosolic 5'-nucleotidase 1A-like	4165	0
XM_020090357.1	protein lin-28 homolog A-like	4432	1.90E-100
XM_020090358.1	mediator of RNA polymerase II transcription subunit 10	1306	1.87E-92
XM_020090359.1	OTU domain-containing protein 6B	1543	0
XM_020090360.1	retinitis pigmentosa 9 protein	1617	1.96E-104
XM_020090361.1	iroquois-class homeodomain protein irx-1-A-like	1948	0
XM_020090362.1	retinoic acid receptor beta isoform X1	1658	0
XM_020090363.1	enolase 4 isoform X1	2230	0
XM_020090364.1	retinoic acid receptor beta isoform X2	1680	0
XM_020090365.1	retinoic acid receptor beta isoform X3	1683	0
XM_020090366.1	retinoic acid receptor beta isoform X4	1542	0
XM_020090367.1	NADH dehydrogenase [ubiquinone] iron-sulfur protein 6, mitochondrial	513	6.99E-93
XM_020090368.1	UPF0711 protein C18orf21 homolog	1904	8.83E-129
XM_020090369.1	AT-hook DNA-binding motif-containing protein 1	6663	0
XM_020090370.1	small integral membrane protein 12	1176	2.87E-44
XM_020090371.1	twist-related protein 2-like	1471	2.38E-93
XM_020090372.1	enolase 4 isoform X2	2161	0
XM_020090373.1	protein FAM92A1 isoform X1	1295	0
XM_020090374.1	protein FAM92A1 isoform X2	1175	2.24E-167
XM_020090375.1	low density lipoprotein receptor adapter protein 1-B-like	1895	0
XM_020090376.1	nucleoporin-like protein 2	2950	1.84E-140
XM_020090377.1	small nuclear ribonucleoprotein Sm D1	628	1.50E-64
XM_020090378.1	asparagine synthetase [glutamine-hydrolyzing] isoform X1	2058	0
XM_020090379.1	asparagine synthetase [glutamine-hydrolyzing] isoform X2	1803	0
XM_020090380.1	asparagine synthetase [glutamine-hydrolyzing] isoform X3	1709	0
XM_020090381.1	chondroitin sulfate proteoglycan 5-like isoform X1	3155	0
XM_020090382.1	chondroitin sulfate proteoglycan 5-like isoform X2	3071	0
XM_020090383.1	thioredoxin-like protein 4A	903	2.51E-101
XM_020090384.1	dorsal root ganglia homeobox protein	2427	0
XM_020090385.1	gap junction alpha-4 protein-like	2309	0
XM_020090386.1	gap junction alpha-4 protein-like	2306	0
XM_020090387.1	leucine-rich repeat-containing protein 15-like	3655	0
XM_020090388.1	integrin beta-8	3246	0
XM_020090389.1	synaptotagmin-like protein 1 isoform X1	2614	0
XM_020090390.1	synaptotagmin-like protein 1 isoform X1	2609	0
XM_020090391.1	synaptotagmin-like protein 1 isoform X1	2622	0
XM_020090392.1	synaptotagmin-like protein 1 isoform X1	2617	0
XM_020090393.1	stromal membrane-associated protein 2-like isoform X1	4625	0
XM_020090394.1	stromal membrane-associated protein 2-like isoform X2	1665	0
XM_020090395.1	glycoprotein endo-alpha-1,2-mannosidase-like protein	2293	0
XM_020090396.1	PREDICTED: uncharacterized protein KIAA0895 homolog	3591	0
XM_020090397.1	von Willebrand factor C domain-containing protein 2-like isoform X1	2424	0
XM_020090398.1	potassium voltage-gated channel subfamily G member 2-like	4358	0
XM_020090399.1	PREDICTED: neurexophilin-1	2214	0
XM_020090400.1	transmembrane protein 200A-like	3443	0

XM_020090401.1	transmembrane protein 200A-like	3365	0
XM_020090402.1	protein naked cuticle homolog 2-like	1141	0
XM_020090403.1	ras-related protein Rab-25-like	1982	1.01E-164
XM_020090404.1	potassium voltage-gated channel subfamily KQT member 4	4814	0
XM_020090405.1	alpha-globin transcription factor CP2 isoform X1	4468	0
XM_020090406.1	programmed cell death 6-interacting protein	3302	0
XM_020090407.1	cAMP-regulated phosphoprotein 21-like	2085	0
XM_020090408.1	sGnRH precursor	503	2.53E-47
XM_020090409.1	serine/threonine-protein kinase DCLK3-like	2610	0
XM_020090410.1	tyrosine-protein kinase yes	1230	0
XM_020090411.1	methyltransferase-like protein 24	1575	0
XM_020090412.1	EF-hand calcium-binding domain-containing protein 1-like	702	1.69E-92
XM_020090413.1	RNA-binding protein 48	932	1.27E-169
XM_020090414.1	kelch-like protein 7	2306	0
XM_020090415.1	probable G-protein coupled receptor 174	1853	0
XM_020090416.1	lactose-binding lectin l-2-like	563	1.41E-118
XM_020090417.1	protein NLRC3-like	1245	0
XM_020090418.1	calcitonin gene-related peptide type 1 receptor	4575	0
XM_020090419.1	protein NDNF-like	2367	0
XM_020090420.1	PREDICTED: neurabin-1	5941	0
XM_020090421.1	ankyrin repeat and SOCS box protein 4	1386	0
XM_020090422.1	cytoplasmic dynein 1 intermediate chain 1	1685	0
XM_020090423.1	calcium-binding mitochondrial carrier protein Aralar2	1867	0
XM_020090424.1	MAGUK p55 subfamily member 6-like	1163	0
XM_020090425.1	inactive phospholipase C-like protein 2	3687	0
XM_020090426.1	WAS/WASL-interacting protein family member 2-like	1995	5.55E-99
XM_020090427.1	juxtaposed with another zinc finger protein 1-like	3128	5.61E-97
XM_020090428.1	non-syndromic hearing impairment protein 5-like	2192	0
XM_020090429.1	adhesion G protein-coupled receptor B2-like	2855	0
XM_020090430.1	phospholipase A1 member A isoform X3	1493	0
XM_020090431.1	collagen alpha-2(IX) chain-like	653	1.33E-09
XM_020090432.1	homeodomain-interacting protein kinase 2-like	1611	0
XM_020090433.1	PREDICTED: uncharacterized protein LOC109631586	729	3.11E-152
XM_020090434.1	PREDICTED: uncharacterized protein LOC109631587, partial	861	5.66E-153
XM_020090435.1	sodium-dependent neutral amino acid transporter B(0)AT1-like	2997	0
XM_020090436.1	non-canonical poly(A) RNA polymerase PAPD7	2638	0
XM_020090437.1	lysophosphatidylcholine acyltransferase 1-like	1297	0
XM_020090438.1	eukaryotic translation initiation factor 3 subunit I	1290	0
XM_020090439.1	poly [ADP-ribose] polymerase 10	3836	0
XM_020090440.1	3-hydroxymethyl-3-methylglutaryl-CoA lyase, cytoplasmic	2198	0
XM_020090441.1	collagen alpha-3(IX) chain-like	3837	0
XM_020090442.1	collagen alpha-1(XXII) chain	7943	0
XM_020090443.1	glucocorticoid modulatory element-binding protein 1-like	1318	0
XM_020090444.1	endonuclease/exonuclease/phosphatase family domain-containing protein 1	2129	0
XM_020090445.1	collagen alpha-1(XXVIII) chain-like	3519	0
XM_020090446.1	connective tissue growth factor-like	3634	0
XM_020090447.1	immunoglobulin superfamily DCC subclass member 3-like	2169	0
XM_020090448.1	transcription factor HIVEP3-like isoform X1	6968	0
XM_020090449.1	redox-regulatory protein FAM213A-like	1837	1.02E-143
XM_020090450.1	transcription factor HIVEP3-like isoform X1	6875	0
XM_020090451.1	transcription factor HIVEP3-like isoform X1	6823	0
XM_020090452.1	transcription factor HIVEP3-like isoform X1	7038	0
XM_020090453.1	transcription factor HIVEP3-like isoform X1	6931	0
XM_020090454.1	transcription factor HIVEP3-like isoform X1	6872	0
XM_020090455.1	phosphatase and actin regulator 4A-like isoform X2	4461	0
XM_020090456.1	phosphatase and actin regulator 4A-like isoform X2	4653	0
XM_020090457.1	phosphatase and actin regulator 4A-like isoform X2	4660	0
XM_020090458.1	AT-rich interactive domain-containing protein 1A-like	2752	3.08E-162
XM_020090459.1	palmitoyltransferase ZDHHC18-like	3349	0
XM_020090460.1	brachyury protein homolog A	2300	0
XM_020090461.1	protein FAM46A-like	2146	0
XM_020090462.1	DNA nucleotidylexotransferase isoform X1	2466	0
XM_020090463.1	delta-type opioid receptor-like	1965	0
XM_020090464.1	keratinocyte differentiation factor 1	2439	0
XM_020090465.1	nuclear migration protein nudC	1403	0
XM_020090466.1	nuclear receptor subfamily 0 group B member 2-like	3326	0
XM_020090467.1	PREDICTED: endothelin-2-like	2459	3.44E-106
XM_020090468.1	PREDICTED: endothelin-2-like	2385	1.94E-106
XM_020090469.1	PREDICTED: endothelin-2-like	2151	2.93E-107
XM_020090470.1	PREDICTED: endothelin-2-like	2455	3.28E-106
XM_020090471.1	DNA nucleotidylexotransferase isoform X2	1632	0
XM_020090472.1	PREDICTED: endothelin-2-like	2330	1.27E-106
XM_020090473.1	gastrula zinc finger protein XlCGF57.1-like	995	0
XM_020090474.1	homeobox protein Hox-A3	2624	0
XM_020090475.1	homeobox protein Hox-A3	2486	0
XM_020090476.1	homeobox protein Hox-A3	3149	0
XM_020090477.1	homeobox protein Hox-A3	2377	0
XM_020090478.1	homeobox protein Hox-A3	2466	0
XM_020090479.1	homeobox protein Hox-A10	2210	0

XM_020090480.1	homeobox protein Hox-A10	2352	0
XM_020090481.1	homeobox protein Hox-A10	2099	0
XM_020090482.1	homeobox even-skipped homolog protein 1	2483	0
XM_020090483.1	galectin-related protein isoform X1	1019	6.18E-132
XM_020090484.1	homeobox protein Hox-A2	2023	0
XM_020090485.1	homeobox protein Hox-A1	2415	0
XM_020090486.1	homeobox protein Hox-A13	1811	0
XM_020090487.1	homeobox protein Hox-B5a-like	1936	0
XM_020090488.1	homeobox protein Hox-A11	2208	0
XM_020090489.1	homeobox protein Hox-A9	2000	0
XM_020090490.1	homeobox protein Hox-A4	2805	7.60E-158
XM_020090491.1	galectin-related protein isoform X2	1010	8.25E-130
XM_020090492.1	gap junction beta-4 protein-like	1613	1.11E-180
XM_020090493.1	sodium-dependent neutral amino acid transporter B(0)AT1-like	2777	0
XM_020090494.1	PREDICTED: gamma-glutamylcyclotransferase-like	1073	3.21E-152
XM_020090495.1	1-acylglycerol-3-phosphate O-acyltransferase ABHD5-like	1335	0
XM_020090496.1	protein 4.1 isoform X7	3131	0
XM_020090497.1	protein 4.1 isoform X2	3083	0
XM_020090498.1	protein 4.1 isoform X7	3077	0
XM_020090499.1	protein 4.1 isoform X4	3068	0
XM_020090500.1	protein 4.1 isoform X8	3056	0
XM_020090501.1	protein 4.1 isoform X6	3014	0
XM_020090502.1	synaptonemal complex protein 3	1297	1.13E-146
XM_020090503.1	protein 4.1 isoform X7	3008	0
XM_020090504.1	protein 4.1 isoform X8	2945	0
XM_020090505.1	protein 4.1 isoform X7	3077	0
XM_020090506.1	sorting nexin-10A-like isoform X2	1325	1.62E-154
XM_020090507.1	sorting nexin-10A-like isoform X2	1281	1.24E-126
XM_020090508.1	sorting nexin-10A-like isoform X2	1067	1.23E-126
XM_020090509.1	sorting nexin-10A-like isoform X2	708	2.52E-126
XM_020090510.1	gonadotropin-releasing hormone II receptor-like	2568	0
XM_020090511.1	SOSS complex subunit C-like	729	7.99E-41
XM_020090512.1	mitochondrial import receptor subunit TOM7 homolog	676	2.53E-33
XM_020090513.1	proline-rich protein 15-like	1661	7.30E-72
XM_020090514.1	ubiquitin domain-containing protein 1-like isoform X1	1667	3.05E-132
XM_020090515.1	pro-neuropeptide Y	664	2.54E-47
XM_020090516.1	NF-kappa-B inhibitor-interacting Ras-like protein 1	1246	2.28E-138
XM_020090517.1	NF-kappa-B inhibitor-interacting Ras-like protein 1	1107	3.02E-139
XM_020090518.1	succinate dehydrogenase assembly factor 3, mitochondrial	1078	2.06E-85
XM_020090519.1	protein Simiate	918	3.24E-133
XM_020090520.1	protachykinin-like isoform X1	1140	3.92E-82
XM_020090521.1	cytochrome b-c1 complex subunit 7	498	2.91E-69
XM_020090522.1	ubiquitin domain-containing protein 1-like isoform X2	1572	1.40E-131
XM_020090523.1	cytochrome c-a-like	1201	5.35E-70
XM_020090524.1	cytochrome c-a-like	1156	3.76E-70
XM_020090525.1	KH domain-containing, RNA-binding, signal transduction-associated protein 3	2411	7.89E-154
XM_020090526.1	leucine-rich repeat-containing protein 3B	1238	0
XM_020090527.1	src-like-adapter 2	2180	0
XM_020090528.1	caspase 3	1719	0
XM_020090529.1	probable G-protein coupled receptor 174	1515	0
XM_020090530.1	G-protein coupled receptor 12-like	1905	0
XM_020090531.1	forkhead box protein H1-like	1361	0
XM_020090532.1	apoptosis regulator BAX-like	870	3.21E-141
XM_020090533.1	preprovasoactive intestinal peptide	1293	5.32E-107
XM_020090534.1	HEPACAM family member 2	2246	0
XM_020090535.1	protein FAM65B isoform X1	1224	0
XM_020090536.1	protein FAM65B isoform X2	1044	0
XM_020090537.1	protein FAM65B isoform X1	1218	0
XM_020090538.1	glycoprotein-N-acetylgalactosamine 3-beta-galactosyltransferase 1-B-like	1089	0
XM_020090539.1	glycoprotein-N-acetylgalactosamine 3-beta-galactosyltransferase 1-B-like	1084	0
XM_020090540.1	brain and acute leukemia cytoplasmic protein-like	1785	8.10E-87
XM_020090541.1	zinc finger protein 704	2277	0
XM_020090542.1	receptor-transporting protein 3-like	1045	1.08E-120
XM_020090543.1	RecName: Full=Cholecystokinin; Short=CCK; Contains: RecName: Full=Cholecystokinin-8; Short=CCK8; Flags: Precursor	760	9.50E-81
XM_020090544.1	39S ribosomal protein L53, mitochondrial	478	1.55E-72
XM_020090545.1	arylsulfatase I-like	1815	0
XM_020090546.1	gap junction beta-4 protein-like	949	0
XM_020090547.1	leucine-rich repeat-containing protein 14B-like	2034	0
XM_020090548.1	CMP-N-acetylneuraminate-beta-galactosamide-alpha-2,3-sialyltransferase 2-like	1818	0
XM_020090549.1	cyclic AMP-responsive element-binding protein 5-like isoform X2	2245	0
XM_020090550.1	cyclic AMP-responsive element-binding protein 5-like isoform X2	2148	0
XM_020090551.1	cyclic AMP-responsive element-binding protein 5-like isoform X2	2202	0
XM_020090552.1	gap junction beta-3 protein-like	902	1.03E-166
XM_020090553.1	homeobox protein MOX-2-like	1615	0
XM_020090554.1	N-terminal EF-hand calcium-binding protein 1	2023	0
XM_020090555.1	tissue-type plasminogen activator-like	1502	0
XM_020090556.1	homeodomain-interacting protein kinase 1-like	1287	0
XM_020090557.1	replication protein A 32 kDa subunit-like	1229	0
XM_020090558.1	tyrosyl-DNA phosphodiesterase 2	1268	0

XM_020090559.1	transmembrane protein 64	4745	0
XM_020090560.1	PREDICTED: uncharacterized protein C6orf62 homolog	3469	1.80E-153
XM_020090561.1	PREDICTED: geminin	1527	3.06E-144
XM_020090562.1	acyl-coenzyme A thioesterase 13 isoform X1	1590	7.94E-86
XM_020090563.1	acyl-coenzyme A thioesterase 13 isoform X2	1472	6.67E-73
XM_020090564.1	iroquois-class homeodomain protein irx-4-A-like	1874	0
XM_020090565.1	thrombospondin type-1 domain-containing protein 7A-like	2341	0
XM_020090566.1	heparan sulfate glucosamine 3-O-sulfotransferase 1-like	1943	0
XM_020090567.1	collagen alpha-1(XVI) chain isoform X2	6891	0
XM_020090568.1	collagen alpha-1(XVI) chain isoform X1	6854	0
XM_020090569.1	collagen alpha-1(VIII) chain isoform X2	6032	2.99E-98
XM_020090570.1	collagen alpha-1(VIII) chain isoform X2	5951	3.15E-98
XM_020090571.1	angiopoietin-1 isoform X1	2549	0
XM_020090572.1	collagen alpha-2(IX) chain-like	2104	2.04E-115
XM_020090573.1	protein tyrosine phosphatase type IVA 2-like	2174	4.77E-123
XM_020090574.1	protein tyrosine phosphatase type IVA 2-like	2026	1.18E-123
XM_020090575.1	fibronectin type III domain-containing protein 5	1255	3.77E-134
XM_020090576.1	vinculin isoform X1	1221	0
XM_020090577.1	PREDICTED: uncharacterized protein C11orf70 homolog	1006	0
XM_020090578.1	transmembrane protein 196	1024	8.67E-82
XM_020090579.1	kelch-like protein 38	2064	0
XM_020090580.1	potassium channel subfamily K member 9	1499	0
XM_020090581.1	synaptonemal complex central element protein 1 isoform X1	1020	3.07E-138
XM_020090582.1	synaptonemal complex central element protein 1 isoform X2	1014	1.54E-136
XM_020090583.1	CD164 sialomucin-like 2 protein	1500	5.24E-80
XM_020090584.1	V-set and transmembrane domain-containing protein 4-like isoform X1	1570	0
XM_020090585.1	PREDICTED: uncharacterized protein C5orf49 homolog	544	4.28E-101
XM_020090586.1	protein FAM221A	958	0
XM_020090587.1	volume-regulated anion channel subunit LRRC8A	1465	0
XM_020090588.1	pro-FMRFamide-related neuropeptide VF-like	618	6.38E-132
XM_020090589.1	RecName: Full=Interleukin-6; Short=IL-6; Flags: Precursor	693	1.15E-157
XM_020090590.1	V-set and transmembrane domain-containing protein 4-like isoform X1	1503	0
XM_020090591.1	SWI/SNF complex subunit SMARCC1-like	3228	0
XM_020090592.1	protein asteroid homolog 1	2882	0
XM_020090593.1	protein asteroid homolog 1	2882	0
XM_020090594.1	protein asteroid homolog 1	2936	0
XM_020090595.1	protein asteroid homolog 1	2919	0
XM_020090596.1	hyccin isoform X1	1744	0
XM_020090597.1	hyccin isoform X1	1654	0
XM_020090598.1	probable G-protein coupled receptor 174	1402	0
XM_020090599.1	hyccin isoform X1	2606	0
XM_020090600.1	PREDICTED: uncharacterized protein LOC109631721	1905	0
XM_020090601.1	PREDICTED: uncharacterized protein LOC109631721	1921	0
XM_020090602.1	protein argonaute-2	8996	0
XM_020090603.1	dickkopf-related protein 1-like	1521	0
XM_020090604.1	homeobox protein DLX-5	1303	0
XM_020090605.1	homeobox protein DLX-6	1763	2.35E-153
XM_020090606.1	valine--tRNA ligase, mitochondrial	3605	0
XM_020090607.1	valine--tRNA ligase, mitochondrial	3606	0
XM_020090608.1	alpha-1,3-mannosyl-glycoprotein 2-beta-N-acetylglucosaminyltransferase-like	5439	0
XM_020090609.1	alpha-1,3-mannosyl-glycoprotein 2-beta-N-acetylglucosaminyltransferase-like	5469	0
XM_020090610.1	alpha-1,3-mannosyl-glycoprotein 2-beta-N-acetylglucosaminyltransferase-like	4847	0
XM_020090611.1	protein phosphatase 1 regulatory subunit 11	1682	9.13E-39
XM_020090612.1	DNA topoisomerase 2-beta isoform X3	5625	0
XM_020090613.1	DNA topoisomerase 2-beta isoform X3	5622	0
XM_020090614.1	DNA topoisomerase 2-beta isoform X3	4594	0
XM_020090615.1	storkhead-box protein 1	6650	0
XM_020090616.1	DNA topoisomerase 2-beta isoform X3	4547	0
XM_020090617.1	peptide-N(4)-(N-acetyl-beta-glucosaminyl)asparagine amidase	2768	0
XM_020090618.1	3-oxoacyl-[acyl-carrier-protein] synthase, mitochondrial	2647	0
XM_020090619.1	3-oxoacyl-[acyl-carrier-protein] synthase, mitochondrial	2566	0
XM_020090620.1	3-oxoacyl-[acyl-carrier-protein] synthase, mitochondrial	2579	0
XM_020090621.1	E3 ubiquitin-protein ligase TRIM71	9172	0
XM_020090622.1	CLIP-associating protein 2 isoform X1	6600	0
XM_020090623.1	CLIP-associating protein 2 isoform X2	6597	0
XM_020090624.1	storkhead-box protein 1	6566	0
XM_020090625.1	CLIP-associating protein 2 isoform X3	6582	0
XM_020090626.1	CLIP-associating protein 2 isoform X4	6575	0
XM_020090627.1	CLIP-associating protein 2 isoform X5	6576	0
XM_020090628.1	CLIP-associating protein 2 isoform X6	6572	0
XM_020090629.1	CLIP-associating protein 2 isoform X7	6573	0
XM_020090630.1	CLIP-associating protein 2 isoform X8	6557	0
XM_020090631.1	CLIP-associating protein 2 isoform X9	6554	0
XM_020090632.1	CLIP-associating protein 2 isoform X10	6548	0
XM_020090633.1	CLIP-associating protein 2 isoform X11	6545	0
XM_020090634.1	CLIP-associating protein 2 isoform X12	6539	0
XM_020090635.1	storkhead-box protein 1	6569	0
XM_020090636.1	CLIP-associating protein 2 isoform X13	6536	0
XM_020090637.1	CLIP-associating protein 2 isoform X14	6536	0

XM_020090638.1	CLIP-associating protein 2 isoform X15	6537	0
XM_020090639.1	CLIP-associating protein 2 isoform X16	6527	0
XM_020090640.1	CLIP-associating protein 2 isoform X17	6521	0
XM_020090641.1	CLIP-associating protein 2 isoform X18	6514	0
XM_020090642.1	CLIP-associating protein 2 isoform X19	6512	0
XM_020090643.1	CLIP-associating protein 2 isoform X20	6509	0
XM_020090644.1	CLIP-associating protein 2 isoform X21	6502	0
XM_020090645.1	CLIP-associating protein 2 isoform X22	6476	0
XM_020090646.1	storkhead-box protein 1	6285	0
XM_020090647.1	CLIP-associating protein 2 isoform X23	6469	0
XM_020090648.1	CLIP-associating protein 2 isoform X24	6451	0
XM_020090649.1	CLIP-associating protein 2 isoform X25	6446	0
XM_020090650.1	CLIP-associating protein 2 isoform X26	6428	0
XM_020090651.1	CLIP-associating protein 2 isoform X27	6428	0
XM_020090652.1	CLIP-associating protein 2 isoform X28	6403	0
XM_020090653.1	CLIP-associating protein 2 isoform X29	6401	0
XM_020090654.1	CLIP-associating protein 2 isoform X30	6373	0
XM_020090655.1	CLIP-associating protein 2 isoform X31	5348	0
XM_020090656.1	CLIP-associating protein 2 isoform X1	5495	0
XM_020090657.1	storkhead-box protein 1	6288	0
XM_020090658.1	CLIP-associating protein 2 isoform X32	5336	0
XM_020090659.1	nuclear factor erythroid 2-related factor 1	2611	0
XM_020090660.1	chromobox protein homolog 3 isoform X1	1784	3.54E-116
XM_020090661.1	chromobox protein homolog 3 isoform X2	1622	6.94E-114
XM_020090662.1	chromobox protein homolog 3 isoform X1	1617	5.19E-114
XM_020090663.1	collagen alpha-2(I) chain	5484	0
XM_020090664.1	tax1-binding protein 1 homolog A-like isoform X1	3001	0
XM_020090665.1	tax1-binding protein 1 homolog A-like isoform X1	4151	0
XM_020090666.1	tax1-binding protein 1 homolog A-like isoform X1	4279	0
XM_020090667.1	3-hydroxyisobutyrate dehydrogenase, mitochondrial-like	2250	0
XM_020090668.1	catenin beta-1-like isoform X1	3538	0
XM_020090669.1	zinc finger protein 883-like	3811	0
XM_020090670.1	catenin beta-1-like isoform X1	3427	0
XM_020090671.1	catenin beta-1-like isoform X2	2570	0
XM_020090672.1	catenin beta-1-like isoform X2	2530	0
XM_020090673.1	protein NDRG1	2579	0
XM_020090674.1	WNT1-inducible-signaling pathway protein 1	1804	0
XM_020090675.1	DNA-binding protein SATB1-like	4071	0
XM_020090676.1	TBC1 domain family member 5 isoform X1	4055	0
XM_020090677.1	TBC1 domain family member 5 isoform X1	4079	0
XM_020090678.1	zinc finger protein 883-like	3820	0
XM_020090679.1	TBC1 domain family member 5 isoform X1	3991	0
XM_020090680.1	TBC1 domain family member 5 isoform X1	2778	0
XM_020090681.1	trafficking kinesin-binding protein 1-like isoform X1	4726	0
XM_020090682.1	trafficking kinesin-binding protein 1-like isoform X1	4826	0
XM_020090683.1	trafficking kinesin-binding protein 1-like isoform X1	4835	0
XM_020090684.1	trafficking kinesin-binding protein 1-like isoform X1	4723	0
XM_020090685.1	trafficking kinesin-binding protein 1-like isoform X1	4047	0
XM_020090686.1	digestive cysteine proteinase 1-like	2556	0
XM_020090687.1	solute carrier family 22 member 13-like	3486	0
XM_020090688.1	zinc finger protein 883-like	3779	0
XM_020090689.1	transcription factor E2F3-like isoform X1	3472	0
XM_020090690.1	transcription factor E2F3-like isoform X1	2723	0
XM_020090691.1	transcription factor SOX-4-like	3585	0
XM_020090692.1	FH1/FH2 domain-containing protein 3-like isoform X1	6467	0
XM_020090693.1	FH1/FH2 domain-containing protein 3-like isoform X2	6440	0
XM_020090694.1	FH1/FH2 domain-containing protein 3-like isoform X3	6038	0
XM_020090695.1	glutathione S-transferase A-like	720	3.27E-170
XM_020090696.1	glutathione S-transferase A-like	1159	2.51E-167
XM_020090697.1	CAS1 domain-containing protein 1	5447	0
XM_020090698.1	epsilon-sarcoglycan isoform X1	1997	0
XM_020090699.1	epsilon-sarcoglycan isoform X2	1965	0
XM_020090700.1	tripartite motif-containing protein 16-like	1917	0
XM_020090701.1	epsilon-sarcoglycan isoform X3	1954	0
XM_020090702.1	epsilon-sarcoglycan isoform X4	1905	0
XM_020090703.1	macoilin-1-like isoform X1	4173	0
XM_020090704.1	macoilin-1-like isoform X2	4176	0
XM_020090705.1	macoilin-1-like isoform X3	3983	0
XM_020090706.1	serum response factor-binding protein 1 isoform X1	3276	0
XM_020090707.1	serum response factor-binding protein 1 isoform X2	3247	0
XM_020090708.1	nuclear pore complex protein Nup153 isoform X1	5718	0
XM_020090709.1	nuclear pore complex protein Nup153 isoform X2	5715	0
XM_020090710.1	stathmin domain-containing protein 1	1805	1.06E-178
XM_020090711.1	forkhead box protein O6-like	4636	0
XM_020090712.1	cbp/p300-interacting transactivator 3-like	3344	1.32E-116
XM_020090713.1	transmembrane protein 150A-like	4390	1.06E-177
XM_020090714.1	importin subunit alpha-7 isoform X1	6541	0
XM_020090715.1	importin subunit alpha-7 isoform X1	6565	0
XM_020090716.1	cleavage and polyadenylation specificity factor subunit 1	4661	0

XM_020090717.1	cleavage and polyadenylation specificity factor subunit 1	4601	0
XM_020090718.1	uncharacterized aarF domain-containing protein kinase 5 isoform X2	2282	0
XM_020090719.1	uncharacterized aarF domain-containing protein kinase 5 isoform X2	2243	0
XM_020090720.1	uncharacterized aarF domain-containing protein kinase 5 isoform X2	2310	0
XM_020090721.1	adenylyl cyclase-associated protein 2 isoform X1	2311	0
XM_020090722.1	adenylyl cyclase-associated protein 2 isoform X2	2273	0
XM_020090723.1	aurora kinase A and ninein-interacting protein	1851	0
XM_020090724.1	vacuolar protein sorting-associated protein 28 homolog	1121	2.34E-166
XM_020090725.1	vacuolar protein sorting-associated protein 28 homolog	1807	8.26E-163
XM_020090726.1	PREDICTED: uncharacterized protein KIAA1522 homolog isoform X1	5968	0
XM_020090727.1	PREDICTED: uncharacterized protein KIAA1522 homolog isoform X1	5969	0
XM_020090728.1	PREDICTED: uncharacterized protein KIAA1522 homolog isoform X2	5887	0
XM_020090729.1	PREDICTED: uncharacterized protein KIAA1522 homolog isoform X2	5854	0
XM_020090730.1	PREDICTED: uncharacterized protein KIAA1522 homolog isoform X3	5860	0
XM_020090731.1	PREDICTED: uncharacterized protein KIAA1522 homolog isoform X4	5576	0
XM_020090732.1	tyrosine--tRNA ligase, cytoplasmic	2354	0
XM_020090733.1	triple functional domain protein isoform X1	6362	0
XM_020090734.1	PREDICTED: uncharacterized protein LOC109631751 isoform X1	2246	0
XM_020090735.1	triple functional domain protein isoform X2	6333	0
XM_020090736.1	telomerase reverse transcriptase	4230	0
XM_020090737.1	sodium-dependent neutral amino acid transporter B(0)AT3-like	2022	0
XM_020090738.1	wiskott-Aldrich syndrome protein family member 2	3002	0
XM_020090739.1	PREDICTED: ataxin-1	9856	0
XM_020090740.1	PREDICTED: ataxin-1	9749	0
XM_020090741.1	PREDICTED: ataxin-1	9473	0
XM_020090742.1	GMP reductase 2-like	1425	0
XM_020090743.1	pumilio homolog 1 isoform X1	5338	0
XM_020090744.1	pumilio homolog 1 isoform X2	5335	0
XM_020090745.1	pumilio homolog 1 isoform X3	5298	0
XM_020090746.1	pumilio homolog 1 isoform X4	3735	0
XM_020090747.1	pumilio homolog 1 isoform X5	5295	0
XM_020090748.1	PREDICTED: syndecan-3	4540	3.50E-53
XM_020090749.1	transmembrane protein 54-like isoform X2	1869	0
XM_020090750.1	transmembrane protein 54-like isoform X2	1796	0
XM_020090751.1	neurocalcin-delta A	3246	4.21E-133
XM_020090752.1	anillin isoform X1	4974	0
XM_020090753.1	anillin isoform X2	4971	0
XM_020090754.1	anillin isoform X3	4972	0
XM_020090755.1	anillin isoform X4	4969	0
XM_020090756.1	anillin isoform X5	4948	0
XM_020090757.1	anillin isoform X6	4945	0
XM_020090758.1	anillin isoform X7	4920	0
XM_020090759.1	PREDICTED: uncharacterized protein LOC109631786	2525	2.36E-159
XM_020090760.1	phosphatidylinositide phosphatase SAC1-B-like	4955	0
XM_020090761.1	cysteine and histidine-rich protein 1	4302	0
XM_020090762.1	cysteine and histidine-rich protein 1	4192	0
XM_020090763.1	antizyme inhibitor 1	3214	0
XM_020090764.1	ankyrin repeat and IBR domain-containing protein 1-like	4976	0
XM_020090765.1	ankyrin repeat and IBR domain-containing protein 1-like	6472	0
XM_020090766.1	Y+L amino acid transporter 2-like	3833	0
XM_020090767.1	Y+L amino acid transporter 2-like	3740	0
XM_020090768.1	PHD finger protein 14 isoform X1	3681	0
XM_020090769.1	PHD finger protein 14 isoform X2	5008	0
XM_020090770.1	septin-7-like isoform X1	2313	0
XM_020090771.1	leucine-rich repeat-containing protein 27	1630	0
XM_020090772.1	septin-7-like isoform X2	2292	0
XM_020090773.1	transcription factor Sp4 isoform X1	3713	0
XM_020090774.1	transcription factor Sp4 isoform X1	2520	0
XM_020090775.1	cell division cycle-associated 7-like protein	1755	0
XM_020090776.1	growth factor receptor-bound protein 10-like isoform X1	6814	0
XM_020090777.1	growth factor receptor-bound protein 10-like isoform X2	6129	0
XM_020090778.1	growth factor receptor-bound protein 10-like isoform X3	6678	0
XM_020090779.1	receptor-type tyrosine-protein phosphatase U-like	5906	0
XM_020090780.1	leucine-rich repeat-containing protein 27	1568	0
XM_020090781.1	ras-related protein Rab-5A-like	2369	5.48E-155
XM_020090782.1	ras-related protein Rab-5A-like	2379	1.33E-155
XM_020090783.1	PREDICTED: syncoilin-like	2531	0
XM_020090784.1	histone-binding protein RBBP4	2126	0
XM_020090785.1	thyroid hormone receptor beta isoform X1	7629	0
XM_020090786.1	thyroid hormone receptor beta isoform X2	7569	0
XM_020090787.1	gamma-aminobutyric acid type B receptor subunit 2 isoform X2	7035	0
XM_020090788.1	gamma-aminobutyric acid type B receptor subunit 2 isoform X2	6416	0
XM_020090789.1	gamma-aminobutyric acid type B receptor subunit 2 isoform X2	6428	0
XM_020090790.1	gamma-aminobutyric acid type B receptor subunit 2 isoform X2	6276	0
XM_020090791.1	gamma-aminobutyric acid type B receptor subunit 2 isoform X2	6288	0
XM_020090792.1	nuclear transcription factor Y subunit gamma isoform X1	2360	3.74E-129
XM_020090793.1	coiled-coil domain-containing protein 177-like	2414	0
XM_020090794.1	nuclear transcription factor Y subunit gamma isoform X1	2329	2.85E-129
XM_020090795.1	nuclear transcription factor Y subunit gamma isoform X1	2387	4.76E-129

XM_020090796.1	nuclear transcription factor Y subunit gamma isoform X1	2341	3.18E-129
XM_020090797.1	nuclear transcription factor Y subunit gamma isoform X1	2488	1.17E-128
XM_020090798.1	nuclear transcription factor Y subunit gamma isoform X1	2357	1.55E-128
XM_020090799.1	nuclear transcription factor Y subunit gamma isoform X1	2337	1.28E-128
XM_020090800.1	nuclear transcription factor Y subunit gamma isoform X1	2486	4.82E-128
XM_020090801.1	nuclear transcription factor Y subunit gamma isoform X1	2291	1.01E-129
XM_020090802.1	nuclear transcription factor Y subunit gamma isoform X2	2288	6.24E-129
XM_020090803.1	histone acetyltransferase KAT2B-like	4203	0
XM_020090804.1	shugoshin 1 isoform X1	2647	0
XM_020090805.1	shugoshin 1 isoform X2	2579	0
XM_020090806.1	zinc finger protein 385D-like isoform X1	1776	0
XM_020090807.1	PREDICTED: uncharacterized protein C6orf118 homolog isoform X1	1477	0
XM_020090808.1	zinc finger protein 385D-like isoform X2	1668	0
XM_020090809.1	zinc finger protein 385D-like isoform X3	1660	0
XM_020090810.1	gamma-taxilin-like	3951	0
XM_020090811.1	CTD small phosphatase-like protein 2	4000	0
XM_020090812.1	protein spire homolog 1-like isoform X1	4297	0
XM_020090813.1	protein spire homolog 1-like isoform X2	4288	0
XM_020090814.1	protein spire homolog 1-like isoform X3	4273	0
XM_020090815.1	protein spire homolog 1-like isoform X4	4267	0
XM_020090816.1	PREDICTED: uncharacterized protein C6orf118 homolog isoform X2	1429	0
XM_020090817.1	protein spire homolog 1-like isoform X5	4255	0
XM_020090818.1	protein spire homolog 1-like isoform X6	4066	0
XM_020090819.1	protein spire homolog 1-like isoform X7	4243	0
XM_020090820.1	protein spire homolog 1-like isoform X8	4222	0
XM_020090821.1	protein spire homolog 1-like isoform X9	4234	0
XM_020090822.1	PREDICTED: uncharacterized protein LOC109631751 isoform X2	2197	0
XM_020090823.1	UBAP1-MVB12-associated (UMA)-domain containing protein 1 isoform X1	3679	6.25E-130
XM_020090824.1	UBAP1-MVB12-associated (UMA)-domain containing protein 1 isoform X2	3667	1.74E-126
XM_020090825.1	replication protein A 14 kDa subunit	635	2.00E-87
XM_020090826.1	engulfment and cell motility protein 1 isoform X1	3771	0
XM_020090827.1	engulfment and cell motility protein 1 isoform X2	2472	1.07E-162
XM_020090828.1	SH3 domain-binding protein 5-like	2538	0
XM_020090829.1	PREDICTED: maturin	2342	1.34E-79
XM_020090830.1	WD repeat-containing protein 27-like	2079	0
XM_020090831.1	PQ-loop repeat-containing protein 1 isoform X1	3255	0
XM_020090832.1	PQ-loop repeat-containing protein 1 isoform X2	4153	1.14E-167
XM_020090833.1	heat shock factor-binding protein 1-like	462	6.89E-49
XM_020090834.1	PREDICTED: uncharacterized protein LOC109631822 isoform X1	1335	1.15E-163
XM_020090835.1	PREDICTED: uncharacterized protein LOC109631822 isoform X2	1328	6.29E-163
XM_020090836.1	PREDICTED: uncharacterized protein LOC109631822 isoform X3	1321	4.72E-133
XM_020090837.1	GATA zinc finger domain-containing protein 1	1281	1.78E-157
XM_020090838.1	chromatin accessibility complex protein 1	1353	3.61E-73
XM_020090839.1	cathepsin B-like	4230	0
XM_020090840.1	cathepsin B-like	4107	0
XM_020090841.1	CTP synthase 1	2446	0
XM_020090842.1	centrosomal protein of 55 kDa-like isoform X1	1400	0
XM_020090843.1	protein lifeguard 1-like	2899	4.25E-167
XM_020090844.1	ubiquilin-1 isoform X2	2231	0
XM_020090845.1	lens fiber membrane intrinsic protein-like	603	5.00E-125
XM_020090846.1	protein L-Myc-1b-like	4113	0
XM_020090847.1	rap guanine nucleotide exchange factor 5-like isoform X1	6120	0
XM_020090848.1	rap guanine nucleotide exchange factor 5-like isoform X2	6118	0
XM_020090849.1	protein FAM83H-like	5647	0
XM_020090850.1	centrosomal protein of 55 kDa-like isoform X1	1325	0
XM_020090851.1	regulating synaptic membrane exocytosis protein 3	6677	1.35E-175
XM_020090852.1	deleted in azoospermia-like protein 1	2438	2.57E-158
XM_020090853.1	deleted in azoospermia-like protein 1	2225	6.19E-146
XM_020090854.1	deleted in azoospermia-like isoform X3	2385	7.09E-144
XM_020090855.1	deleted in azoospermia-like isoform X4	1886	5.58E-133
XM_020090856.1	dynein heavy chain 3, axonemal	13281	0
XM_020090857.1	phosphatidylserine synthase 1	3301	0
XM_020090858.1	transcription termination factor 3, mitochondrial	1704	0
XM_020090859.1	SH3 domain-binding glutamic acid-rich-like protein 3	2900	2.78E-56
XM_020090860.1	PREDICTED: syndetin	4394	0
XM_020090861.1	ADNP homeobox protein 2-like	4371	0
XM_020090862.1	protein FAM196A-like	2148	0
XM_020090863.1	U5 small nuclear ribonucleoprotein 40 kDa protein	1429	0
XM_020090864.1	nucleolar protein of 40 kDa	1667	7.73E-125
XM_020090865.1	polypeptide N-acetylgalactosaminyltransferase 4-like	3532	0
XM_020090866.1	trafficking protein particle complex subunit 9 isoform X1	4770	0
XM_020090867.1	trafficking protein particle complex subunit 9 isoform X2	4743	0
XM_020090868.1	ubiquitin-conjugating enzyme E2 E1-like	1458	2.40E-105
XM_020090869.1	ubiquitin-conjugating enzyme E2 E1-like	1831	1.98E-103
XM_020090870.1	ubiquitin-conjugating enzyme E2 E1-like	1415	1.80E-105
XM_020090871.1	cytoplasmic dynein 1 light intermediate chain 1-like isoform X1	1681	0
XM_020090872.1	cytoplasmic dynein 1 light intermediate chain 1-like isoform X2	2957	0
XM_020090873.1	nuclear receptor subfamily 1 group D member 2	3320	0
XM_020090874.1	probable histone deacetylase 1-B isoform X1	2375	0

XM_020090875.1	cyclic GMP-AMP synthase	1788	0
XM_020090876.1	probable histone deacetylase 1-B isoform X1	2411	0
XM_020090877.1	alpha-(1,3)-fucosyltransferase 9-like isoform X1	2928	0
XM_020090878.1	alpha-(1,3)-fucosyltransferase 9-like isoform X2	2937	0
XM_020090879.1	sodium/potassium-transporting ATPase subunit beta-1-interacting protein 1	2758	4.98E-144
XM_020090880.1	serine/threonine-protein kinase 35-like	4195	0
XM_020090881.1	tRNA (cytosine(34)-C(5))-methyltransferase	2884	0
XM_020090882.1	testis- and ovary-specific PAZ domain-containing protein 1 isoform X1	5809	0
XM_020090883.1	testis- and ovary-specific PAZ domain-containing protein 1 isoform X1	4781	0
XM_020090884.1	testis- and ovary-specific PAZ domain-containing protein 1 isoform X1	5643	0
XM_020090885.1	catenin alpha-2	3665	0
XM_020090886.1	PREDICTED: uncharacterized protein LOC109631857	820	1.01E-150
XM_020090887.1	pyruvate dehydrogenase kinase, isozyme 4	3610	0
XM_020090888.1	peptidyl-prolyl cis-trans isomerase FKBP10-like	2626	0
XM_020090889.1	transmembrane protein 222-like	4344	2.59E-125
XM_020090890.1	serine/threonine-protein phosphatase 6 regulatory ankyrin repeat subunit A-like isoform X1	4631	0
XM_020090891.1	serine/threonine-protein phosphatase 6 regulatory ankyrin repeat subunit A-like isoform X2	4582	0
XM_020090892.1	serine/threonine-protein phosphatase 6 regulatory ankyrin repeat subunit A-like isoform X3	4117	0
XM_020090893.1	CUB and sushi domain-containing protein 2	12597	0
XM_020090894.1	oxysterol-binding protein-related protein 3-like	3339	0
XM_020090895.1	sodium-dependent lysophosphatidylcholine symporter 1	2021	0
XM_020090896.1	ADP-ribosylation factor-like protein 4A	1149	2.25E-137
XM_020090897.1	fatty acid-binding protein, heart-like	925	4.03E-91
XM_020090898.1	KH domain-containing, RNA-binding, signal transduction-associated protein 2	1438	0
XM_020090899.1	PREDICTED: stathmin-2	2537	8.97E-121
XM_020090900.1	PREDICTED: uncharacterized protein LOC109631871 isoform X1	2230	0
XM_020090901.1	PREDICTED: uncharacterized protein LOC109631871 isoform X2	2227	0
XM_020090902.1	PREDICTED: uncharacterized protein LOC109631871 isoform X3	2227	0
XM_020090903.1	PREDICTED: uncharacterized protein LOC109631751 isoform X3	2144	0
XM_020090904.1	PREDICTED: uncharacterized protein LOC109631871 isoform X4	2227	0
XM_020090905.1	progranulin type I	1752	0
XM_020090906.1	plasma kallikrein-like	1237	0
XM_020090907.1	hairy/enhancer-of-split related with YRPW motif-like protein	3604	1.05E-140
XM_020090908.1	PREDICTED: stathmin-like	1382	1.63E-90
XM_020090909.1	PREDICTED: stathmin-like	1265	4.91E-91
XM_020090910.1	PREDICTED: stathmin-like	1225	3.25E-91
XM_020090911.1	transmembrane protein 106B-like	3155	6.79E-155
XM_020090912.1	ubiquitin-conjugating enzyme E2 Q2-like isoform X1	4430	0
XM_020090913.1	serine incorporator 1-like	2208	0
XM_020090914.1	probable acyl-CoA dehydrogenase 6	1656	0
XM_020090915.1	BET1 homolog	1118	3.36E-62
XM_020090916.1	homeobox protein NOBOX	2688	0
XM_020090917.1	cartilage matrix protein	3322	0
XM_020090918.1	placenta-specific protein 9	649	9.06E-39
XM_020090919.1	ubiquitin-conjugating enzyme E2 E1-like	1491	3.47E-138
XM_020090920.1	protein FAM135B	4863	0
XM_020090921.1	protein FAM135B	4813	0
XM_020090922.1	protein FAM135B	4838	0
XM_020090923.1	transformer-2 protein homolog alpha isoform X1	1689	1.66E-113
XM_020090924.1	transformer-2 protein homolog alpha isoform X1	1683	1.56E-113
XM_020090925.1	zinc finger and BTB domain-containing protein 46	3392	0
XM_020090926.1	zinc finger and BTB domain-containing protein 46	3327	0
XM_020090927.1	tissue factor pathway inhibitor 2	890	3.37E-153
XM_020090928.1	guanine nucleotide-binding protein G(T) subunit gamma-T1-like	880	1.51E-44
XM_020090929.1	guanine nucleotide-binding protein G(T) subunit gamma-T1-like	812	7.99E-45
XM_020090930.1	acetylcholinesterase collagenic tail peptide-like isoform X1	2921	0
XM_020090931.1	ankyrin repeat domain-containing protein 2-like	1034	0
XM_020090932.1	acetylcholinesterase collagenic tail peptide-like isoform X1	2905	0
XM_020090933.1	acetylcholinesterase collagenic tail peptide-like isoform X1	2812	0
XM_020090934.1	acetylcholinesterase collagenic tail peptide-like isoform X1	2901	0
XM_020090935.1	acetylcholinesterase collagenic tail peptide-like isoform X1	2900	0
XM_020090936.1	peptidyl-prolyl cis-trans isomerase FKBP14-like	1641	2.44E-142
XM_020090937.1	PREDICTED: uncharacterized protein LOC109631894 isoform X1	1439	0
XM_020090938.1	PREDICTED: uncharacterized protein LOC109631894 isoform X1	1433	0
XM_020090939.1	membrane progestin receptor alpha-B-like isoform X2	3192	0
XM_020090940.1	membrane progestin receptor alpha-B-like isoform X2	1723	0
XM_020090941.1	phosphoprotein associated with glycosphingolipid-enriched microdomains 1	2641	0
XM_020090942.1	phosphoprotein associated with glycosphingolipid-enriched microdomains 1	2644	0
XM_020090943.1	E3 ubiquitin-protein ligase MYLIP	2767	0
XM_020090944.1	MARCKS-related protein-like	1722	2.53E-19
XM_020090945.1	protein FAM49A-like isoform X1	2412	0
XM_020090946.1	protein FAM49A-like isoform X1	2359	0
XM_020090947.1	protein FAM49A-like isoform X1	2568	0
XM_020090948.1	G patch domain-containing protein 1	3043	0
XM_020090949.1	PREDICTED: neurensin-1-like	1319	4.22E-111
XM_020090950.1	PREDICTED: neurensin-1-like	1236	1.19E-111
XM_020090951.1	polypeptide N-acetylgalactosaminyltransferase 6-like	2697	0
XM_020090952.1	zinc finger and BTB domain-containing protein 18 isoform X1	5427	0
XM_020090953.1	DNA-directed RNA polymerase I subunit RPA43	1876	0

XM_020090954.1	F-box-like/WD repeat-containing protein TBL1XR1 isoform X1	2551	0
XM_020090955.1	ras-related protein Rab-39B-like	4461	1.08E-139
XM_020090956.1	cyclin-dependent kinase 6	3293	0
XM_020090957.1	PREDICTED: uncharacterized protein C6orf47 homolog	3878	1.36E-169
XM_020090958.1	programmed cell death protein 6 isoform X2	1798	3.58E-124
XM_020090959.1	KH domain-containing, RNA-binding, signal transduction-associated protein 1-like	2053	3.44E-97
XM_020090960.1	scinderin-like protein	3234	0
XM_020090961.1	type 2 phosphatidylinositol 4,5-bisphosphate 4-phosphatase	2242	0
XM_020090962.1	tumor protein D52 isoform X1	1542	1.86E-110
XM_020090963.1	zinc finger and BTB domain-containing protein 18 isoform X2	5402	0
XM_020090964.1	tumor protein D52 isoform X1	591	2.18E-70
XM_020090965.1	sestrin-2-like isoform X1	2687	0
XM_020090966.1	sestrin-2-like isoform X1	1940	0
XM_020090967.1	coiled-coil domain-containing protein 126	2706	2.46E-72
XM_020090968.1	coiled-coil domain-containing protein 126	2575	1.43E-72
XM_020090969.1	insulin-like growth factor 2 mRNA-binding protein 3	1941	0
XM_020090970.1	hairy/enhancer-of-split related with YRPW motif protein 1	2174	1.10E-132
XM_020090971.1	zinc finger and BTB domain-containing protein 18 isoform X3	5190	0
XM_020090972.1	V-type proton ATPase subunit C 1-A	2136	0
XM_020090973.1	V-type proton ATPase subunit C 1-A	2031	0
XM_020090974.1	regulation of nuclear pre-mRNA domain-containing protein 1A isoform X1	2226	0
XM_020090975.1	regulation of nuclear pre-mRNA domain-containing protein 1A isoform X2	1239	0
XM_020090976.1	60S ribosomal protein L14	566	7.80E-91
XM_020090977.1	nuclear receptor coactivator 7	2020	0
XM_020090978.1	DNA-binding protein inhibitor ID-4-like	2071	1.22E-93
XM_020090979.1	zinc finger and BTB domain-containing protein 18 isoform X4	5273	0
XM_020090980.1	DNA-binding protein inhibitor ID-4-like	1287	8.41E-97
XM_020090981.1	palmitoyltransferase ZDHHC3-like isoform X1	1502	0
XM_020090982.1	palmitoyltransferase ZDHHC3-like isoform X2	3830	0
XM_020090983.1	palmitoyltransferase ZDHHC3-like isoform X3	1371	0
XM_020090984.1	clathrin coat assembly protein AP180 isoform X1	5226	0
XM_020090985.1	clathrin coat assembly protein AP180 isoform X1	5194	0
XM_020090986.1	clathrin coat assembly protein AP180 isoform X1	3419	0
XM_020090987.1	clathrin coat assembly protein AP180 isoform X1	3407	0
XM_020090988.1	clathrin coat assembly protein AP180 isoform X1	3401	0
XM_020090989.1	zinc finger and BTB domain-containing protein 18 isoform X4	6005	0
XM_020090990.1	clathrin coat assembly protein AP180 isoform X1	3353	0
XM_020090991.1	clathrin coat assembly protein AP180 isoform X1	3338	0
XM_020090992.1	clathrin coat assembly protein AP180 isoform X7	3221	0
XM_020090993.1	clathrin coat assembly protein AP180 isoform X1	3200	0
XM_020090994.1	clathrin coat assembly protein AP180 isoform X9	3164	0
XM_020090995.1	clathrin coat assembly protein AP180 isoform X10	3080	0
XM_020090996.1	syntabulin isoform X1	2413	0
XM_020090997.1	syntabulin isoform X2	2687	0
XM_020090998.1	protein FAM84B-like	1931	0
XM_020090999.1	zinc finger and BTB domain-containing protein 18 isoform X4	5158	0
XM_020091000.1	protein FAM84B-like	1753	0
XM_020091001.1	cytosolic non-specific dipeptidase-like	1823	0
XM_020091002.1	cysteine-rich and transmembrane domain-containing protein 1-like	1658	2.84E-83
XM_020091003.1	protein O-linked-mannose beta-1,4-N-acetylglucosaminyltransferase 2	3830	0
XM_020091004.1	protein O-linked-mannose beta-1,4-N-acetylglucosaminyltransferase 2	3833	0
XM_020091005.1	centrosomal protein of 162 kDa isoform X1	3976	0
XM_020091006.1	centrosomal protein of 162 kDa isoform X2	3974	0
XM_020091007.1	keratinocyte differentiation factor 1	2167	0
XM_020091008.1	keratinocyte differentiation factor 1	2183	0
XM_020091009.1	protein MAL2	2116	2.58E-101
XM_020091010.1	FXYD domain-containing ion transport regulator 3-like	1157	1.40E-57
XM_020091011.1	adenylosuccinate synthetase isozyme 2	3995	0
XM_020091012.1	A disintegrin and metalloproteinase with thrombospondin motifs 16	5914	0
XM_020091013.1	TBC1 domain family member 31	3864	0
XM_020091014.1	zinc fingers and homeoboxes protein 2-like	7696	0
XM_020091015.1	ER membrane protein complex subunit 2	1443	0
XM_020091016.1	E3 ubiquitin-protein ligase ZNRF2-like	5195	6.30E-106
XM_020091017.1	serine protease hepsin	3587	0
XM_020091018.1	serine protease hepsin	3420	0
XM_020091019.1	B-cell receptor CD22-like	1411	0
XM_020091020.1	B-cell receptor CD22-like	1930	0
XM_020091021.1	fasciculation and elongation protein zeta-2-like	2267	0
XM_020091022.1	C-type lectin domain family 3 member A	1570	7.72E-129
XM_020091023.1	zinc finger protein 184-like isoform X1	2075	0
XM_020091024.1	regulating synaptic membrane exocytosis protein 2 isoform X1	5281	0
XM_020091025.1	regulating synaptic membrane exocytosis protein 2 isoform X2	5263	0
XM_020091026.1	regulating synaptic membrane exocytosis protein 2 isoform X3	1479	0
XM_020091027.1	cytochrome b5 reductase 4	2348	0
XM_020091028.1	protein ATP1B4	1235	0
XM_020091029.1	trophoblast glycoprotein-like	1322	0
XM_020091030.1	transmembrane protein 222-like	1718	6.19E-132
XM_020091031.1	DNA-directed RNA polymerase I subunit RPA12	1124	5.70E-82
XM_020091032.1	zinc finger protein 184-like isoform X1	638	4.04E-89

XM_020091033.1	DNA-directed RNA polymerase I subunit RPA12	1188	1.03E-81
XM_020091034.1	DNA-directed RNA polymerase I subunit RPA12	1120	5.18E-82
XM_020091035.1	DNA-directed RNA polymerase I subunit RPA12	1100	7.17E-83
XM_020091036.1	TGF-beta receptor type-2-like isoform X1	2848	0
XM_020091037.1	TGF-beta receptor type-2-like isoform X2	1876	0
XM_020091038.1	mdm2-binding protein	3344	0
XM_020091039.1	iroquois-class homeodomain protein irx-1-like	1790	0
XM_020091040.1	RWD domain-containing protein 1	1420	1.38E-122
XM_020091041.1	PREDICTED: uncharacterized protein LOC109631958	1354	0
XM_020091042.1	nuclear factor of activated T-cells, cytoplasmic 1 isoform X1	5178	0
XM_020091043.1	nuclear factor of activated T-cells, cytoplasmic 1 isoform X2	4512	0
XM_020091044.1	transmembrane protein 42-like	2628	6.02E-69
XM_020091045.1	transmembrane protein 147	1375	4.41E-163
XM_020091046.1	pituitary tumor-transforming gene 1 protein-interacting protein-like	1161	3.44E-103
XM_020091047.1	NAD-dependent malic enzyme, mitochondrial	2467	0
XM_020091048.1	E3 ubiquitin-protein ligase RNF144B	2165	0
XM_020091049.1	E3 ubiquitin-protein ligase RNF144B	2129	0
XM_020091050.1	E3 ubiquitin-protein ligase RNF144B	1948	0
XM_020091051.1	GPN-loop GTPase 2	1372	0
XM_020091052.1	orexin receptor type 2	1427	0
XM_020091053.1	protein FAM46A-like	4287	0
XM_020091054.1	thiosulfate sulfurtransferase/rhodanese-like domain-containing protein 1	1518	1.48E-126
XM_020091055.1	transforming growth factor beta-2-like	3417	0
XM_020091056.1	R-spondin 1	2859	5.39E-164
XM_020091057.1	PREDICTED: uncharacterized protein C8orf76-like	1879	0
XM_020091058.1	blood vessel epicardial substance	3404	0
XM_020091059.1	lysophospholipase-like protein 1	1068	0
XM_020091060.1	CUB domain-containing protein 1-like	3548	0
XM_020091061.1	t-SNARE domain-containing protein 1	2208	0
XM_020091062.1	growth/differentiation factor 6 isoform X1	3063	0
XM_020091063.1	protein BEAN1	1174	1.11E-168
XM_020091064.1	growth/differentiation factor 6 isoform X2	3000	0
XM_020091065.1	RecName: Full=Cholecystokinin; Short=CCK; Contains: RecName: Full=Cholecystokinin-8; Short=CCK8; Flags: Precursor	550	2.10E-85
XM_020091066.1	popeye domain-containing protein 3	3843	0
XM_020091067.1	COX assembly mitochondrial protein homolog	594	2.16E-74
XM_020091068.1	protein FAM26E-like	2347	0
XM_020091069.1	PREDICTED: neurensin-1	2648	7.39E-140
XM_020091070.1	PREDICTED: neurensin-1	2636	6.66E-140
XM_020091071.1	serine protease 23-like	2756	0
XM_020091072.1	squalene monooxygenase	2613	0
XM_020091073.1	failed axon connections homolog	2511	0
XM_020091074.1	melanocortin receptor 5-like	1371	0
XM_020091075.1	sulfotransferase family cytosolic 2B member 1-like	1546	0
XM_020091076.1	oxysterol-binding protein-related protein 10-like isoform X1	3572	0
XM_020091077.1	oxysterol-binding protein-related protein 10-like isoform X2	3725	0
XM_020091078.1	PREDICTED: gamma-glutamylcyclotransferase-like	755	3.45E-127
XM_020091079.1	low-density lipoprotein receptor class A domain-containing protein 4 isoform X1	4282	2.36E-120
XM_020091080.1	low-density lipoprotein receptor class A domain-containing protein 4 isoform X2	4317	4.93E-125
XM_020091081.1	low-density lipoprotein receptor class A domain-containing protein 4 isoform X3	4404	5.90E-146
XM_020091082.1	thyrotropin-releasing hormone receptor-like	2610	0
XM_020091083.1	transmembrane protein 158	2372	2.81E-125
XM_020091084.1	E3 ubiquitin-protein ligase TRIM39-like	2494	0
XM_020091085.1	iroquois-class homeodomain protein IRX-2	3630	0
XM_020091086.1	hyaluronan synthase 2	4618	0
XM_020091087.1	nucleotide-binding oligomerization domain 1 protein	3599	0
XM_020091088.1	kelch-like protein 32	2964	0
XM_020091089.1	transmembrane protein 229B-like	1263	1.21E-117
XM_020091090.1	kelch-like protein 32	2956	0
XM_020091091.1	kelch-like protein 32	2874	0
XM_020091092.1	amine sulfotransferase-like isoform X1	3195	0
XM_020091093.1	amine sulfotransferase-like isoform X1	3144	0
XM_020091094.1	desmin-like isoform X1	2505	0
XM_020091095.1	desmin-like isoform X2	1773	0
XM_020091096.1	zinc finger and BTB domain-containing protein 49	3980	0
XM_020091097.1	protein FAM26F-like	2100	0
XM_020091098.1	neural cell adhesion molecule 1-like isoform X1	3390	0
XM_020091099.1	neural cell adhesion molecule 1-like isoform X2	3313	0
XM_020091100.1	neural cell adhesion molecule 1-like isoform X3	3264	0
XM_020091101.1	40S ribosomal protein S10	1098	0
XM_020091102.1	neuropeptide Y receptor type 4-like	1356	0
XM_020091103.1	protein ATP1B4	1763	0
XM_020091104.1	centrosomal protein of 192 kDa	8406	0
XM_020091105.1	centrosomal protein of 76 kDa	3369	0
XM_020091106.1	general transcription factor II-I repeat domain-containing protein 2-like	1551	0
XM_020091107.1	reverse transcriptase-like protein	741	7.84E-178
XM_020091108.1	regulator of G-protein signaling 22	2952	0
XM_020091109.1	vacuolar protein sorting-associated protein 13B	12938	0
XM_020091110.1	threo-3-hydroxyaspartate ammonia-lyase-like	2007	0
XM_020091111.1	ribonucleases P/MRP protein subunit POP1 isoform X2	639	7.36E-132

XM_020091112.1	neuroendocrine convertase 1-like	2413	0
XM_020091113.1	speckle-type POZ protein	5718	0
XM_020091114.1	matrix metalloproteinase-24-like	615	2.88E-157
XM_020091115.1	2-oxoisovalerate dehydrogenase subunit beta, mitochondrial	1144	0
XM_020091116.1	eukaryotic translation initiation factor 4 gamma 1-like	1317	0
XM_020091117.1	myelin basic protein-like	433	8.25E-59
XM_020091118.1	zinc finger protein 236-like	871	0
XM_020091119.1	PREDICTED: uncharacterized protein C8orf76-like	605	1.46E-95
XM_020091120.1	brain-specific angiogenesis inhibitor 1-like	4239	0
XM_020091121.1	PREDICTED: uncharacterized protein LOC109632024	1238	1.67E-152
XM_020091122.1	brain-specific angiogenesis inhibitor 1-like	1574	0
XM_020091123.1	coiled-coil domain-containing protein 65-like	1396	0
XM_020091124.1	CD209 antigen-like protein E	1040	5.72E-178
XM_020091125.1	ectonucleotide pyrophosphatase/phosphodiesterase family member 2	2286	0
XM_020091126.1	protein FAM198A	2808	0
XM_020091127.1	IgGFc-binding protein-like	8409	0
XM_020091128.1	dysbindin-like isoform X2	2140	1.10E-177
XM_020091129.1	homeodomain-interacting protein kinase 3-like	819	4.51E-116
XM_020091130.1	adenylate cyclase type 3	6232	0
XM_020091131.1	histidine triad nucleotide-binding protein 3	1290	9.15E-101
XM_020091132.1	coiled-coil domain-containing protein 42 homolog	1268	0
XM_020091133.1	elongation of very long chain fatty acids protein 4-like	3471	3.67E-165
XM_020091134.1	CUB and sushi domain-containing protein 3-like	12588	0
XM_020091135.1	C-C chemokine receptor type 8-like	1280	0
XM_020091136.1	adenylate cyclase type 2-like	4572	0
XM_020091137.1	lysophosphatidylcholine acyltransferase 1-like	5347	0
XM_020091138.1	transcription factor 7-like	795	2.34E-170
XM_020091139.1	lysophospholipid acyltransferase 1	1664	0
XM_020091140.1	tumor necrosis factor receptor superfamily member 6B-like	1303	0
XM_020091141.1	alpha-1-syntrophin isoform X1	4007	0
XM_020091142.1	collagen alpha-1(XIV) chain	7406	0
XM_020091143.1	microtubule-actin cross-linking factor 1-like	18222	0
XM_020091144.1	E3 ubiquitin-protein ligase UBR5 isoform X1	8580	0
XM_020091145.1	E3 ubiquitin-protein ligase UBR5 isoform X2	8888	0
XM_020091146.1	E3 ubiquitin-protein ligase UBR5 isoform X3	8864	0
XM_020091147.1	E3 ubiquitin-protein ligase UBR5 isoform X4	8838	0
XM_020091148.1	E3 ubiquitin-protein ligase UBR5 isoform X5	8856	0
XM_020091149.1	E3 ubiquitin-protein ligase UBR5 isoform X6	8538	0
XM_020091150.1	E3 ubiquitin-protein ligase UBR5 isoform X7	8481	0
XM_020091151.1	ribonucleases P/MRP protein subunit POP1 isoform X1	3719	0
XM_020091152.1	ribonucleases P/MRP protein subunit POP1 isoform X2	3701	0
XM_020091153.1	protein spire homolog 1-like isoform X1	5118	0
XM_020091154.1	protein spire homolog 1-like isoform X2	5109	0
XM_020091155.1	protein spire homolog 1-like isoform X3	5067	0
XM_020091156.1	matrilin-4 isoform X1	2353	0
XM_020091157.1	E3 SUMO-protein ligase ZNF451	2741	0
XM_020091158.1	skin secretory protein xP2-like isoform X4	3114	8.52E-57
XM_020091159.1	skin secretory protein xP2-like isoform X4	3078	8.08E-57
XM_020091160.1	skin secretory protein xP2-like isoform X4	3048	7.50E-57
XM_020091161.1	skin secretory protein xP2-like isoform X4	3024	5.00E-57
XM_020091162.1	protein LYRIC-like	2896	0
XM_020091163.1	Krueppel-like factor 11	1979	0
XM_020091164.1	antizyme inhibitor 1	2875	0
XM_020091165.1	antizyme inhibitor 1	2825	0
XM_020091166.1	E3 SUMO-protein ligase ZNF451	2739	0
XM_020091167.1	V-type proton ATPase subunit C 1-A	1955	0
XM_020091168.1	IQ domain-containing protein G-like	1047	4.07E-141
XM_020091169.1	lysosomal-associated transmembrane protein 4B isoform X1	1940	1.56E-139
XM_020091170.1	lysosomal-associated transmembrane protein 4B isoform X2	906	1.36E-146
XM_020091171.1	ribonuclease UK114-like	1507	5.60E-92
XM_020091172.1	E3 SUMO-protein ligase ZNF451	2749	0
XM_020091173.1	zinc finger protein 236 isoform X1	5506	0
XM_020091174.1	zinc finger protein 236 isoform X2	5503	0
XM_020091175.1	zinc finger protein 236 isoform X3	5563	0
XM_020091176.1	sal-like protein 3	6228	0
XM_020091177.1	teashirt homolog 1-like isoform X1	4835	0
XM_020091178.1	zinc finger protein 516	8053	0
XM_020091179.1	zinc finger protein 516	8020	0
XM_020091180.1	zinc finger protein 407-like	4055	0
XM_020091181.1	E3 SUMO-protein ligase ZNF451	2763	0
XM_020091182.1	DEP domain-containing protein 1B	3530	0
XM_020091183.1	galanin receptor type 1-like	1823	0
XM_020091184.1	myelin basic protein-like isoform X1	2049	2.09E-59
XM_020091185.1	myelin basic protein-like isoform X1	2067	3.72E-53
XM_020091186.1	myelin basic protein-like isoform X3	2009	9.62E-44
XM_020091187.1	myelin basic protein-like isoform X3	2027	1.17E-37
XM_020091188.1	E3 SUMO-protein ligase ZNF451	2745	0
XM_020091189.1	E3 ubiquitin-protein ligase E3D	1867	0
XM_020091190.1	ubiquinone biosynthesis O-methyltransferase, mitochondrial isoform X1	1536	0
XM_020091191.1	ubiquinone biosynthesis O-methyltransferase, mitochondrial isoform X2	1466	0

XM_020091192.1	ethylmalonyl-CoA decarboxylase	1385	0
XM_020091193.1	39S ribosomal protein L32, mitochondrial	944	5.45E-104
XM_020091194.1	sodium-dependent noradrenaline transporter	1954	0
XM_020091195.1	sodium- and chloride-dependent transporter XTRP3	3032	0
XM_020091196.1	tuftelin-interacting protein 11 isoform X1	1593	0
XM_020091197.1	tRNA (guanine(10)-N2)-methyltransferase homolog	1620	0
XM_020091198.1	RNA-binding motif, single-stranded-interacting protein 2-like isoform X1	2406	0
XM_020091199.1	collagen alpha-1(IX) chain-like	2706	0
XM_020091200.1	RNA-binding motif, single-stranded-interacting protein 2-like isoform X1	2819	0
XM_020091201.1	xylose isomerase-like	1834	0
XM_020091202.1	WD and tetratricopeptide repeats protein 1	2031	0
XM_020091203.1	WD and tetratricopeptide repeats protein 1	1944	0
XM_020091204.1	protein odd-skipped-related 1-like	1384	0
XM_020091205.1	protein odd-skipped-related 1-like	1302	0
XM_020091206.1	PREDICTED: secretagogin-like	1365	0
XM_020091207.1	POU domain, class 3, transcription factor 2	2067	0
XM_020091208.1	aromatic-L-amino-acid decarboxylase	2867	0
XM_020091209.1	aromatic-L-amino-acid decarboxylase	2827	0
XM_020091210.1	NIPA-like protein 2	1459	0
XM_020091211.1	PREDICTED: beta-microseminoprotein	515	3.68E-64
XM_020091212.1	transcription and mRNA export factor ENY2-2	735	1.21E-54
XM_020091213.1	transcription and mRNA export factor ENY2-2	587	2.17E-55
XM_020091214.1	FXYD domain-containing ion transport regulator 11-like	878	1.73E-47
XM_020091215.1	PREDICTED: R-spondin-3-like	2743	0
XM_020091216.1	protein NOV homolog	2201	0
XM_020091217.1	PR domain zinc finger protein 13	2553	0
XM_020091218.1	PREDICTED: uncharacterized protein LOC109632095	1072	2.20E-109
XM_020091219.1	PREDICTED: uncharacterized protein LOC109632095	989	9.90E-110
XM_020091220.1	stanniocalcin 2	1170	2.46E-162
XM_020091221.1	actin-binding Rho-activating protein-like	789	6.13E-134
XM_020091222.1	actin-binding Rho-activating protein-like	787	3.64E-134
XM_020091223.1	cleft lip and palate transmembrane protein 1-like protein	2877	0
XM_020091224.1	meiosis-specific nuclear structural protein 1-like	1136	2.68E-150
XM_020091225.1	ectonucleoside triphosphate diphosphohydrolase 2-like	2594	0
XM_020091226.1	syntaxin-12-like	2093	6.22E-131
XM_020091227.1	syntaxin-12-like	2082	5.54E-131
XM_020091228.1	syntaxin-12-like	2066	4.79E-131
XM_020091229.1	eomesodermin-like isoform X1	2570	0
XM_020091230.1	eomesodermin-like isoform X2	2558	0
XM_020091231.1	hepcidin 1	470	1.91E-38
XM_020091232.1	kelch-like protein 38	1982	0
XM_020091233.1	kelch-like protein 38	2457	0
XM_020091234.1	potassium voltage-gated channel subfamily S member 3	2206	0
XM_020091235.1	iroquois-class homeodomain protein irx-4-A-like isoform X1	2009	0
XM_020091236.1	iroquois-class homeodomain protein irx-4-A-like isoform X2	1757	0
XM_020091237.1	otoraplin isoform X1	763	9.97E-93
XM_020091238.1	collectin-11 isoform X2	2930	1.68E-148
XM_020091239.1	tumor necrosis factor receptor superfamily member 11B	2136	0
XM_020091240.1	macrophage mannose receptor 1-like	1604	0
XM_020091241.1	nuclear receptor subfamily 0 group B member 2-like	1122	4.55E-178
XM_020091242.1	basic helix-loop-helix transcription factor scleraxis	1762	9.47E-101
XM_020091243.1	UBX domain-containing protein 11	1834	0
XM_020091244.1	otoraplin isoform X2	712	9.97E-79
XM_020091245.1	transmembrane protein 74-like	1431	1.98E-166
XM_020091246.1	zymogen granule membrane protein 16	815	1.27E-99
XM_020091247.1	B-cell receptor CD22-like	2982	0
XM_020091248.1	single-minded homolog 1	1334	0
XM_020091249.1	C-C chemokine receptor type 1-like	1220	0
XM_020091250.1	CKLF-like MARVEL transmembrane domain-containing protein 8	1437	1.36E-120
XM_020091251.1	doublecortin domain-containing protein 2-like	2058	0
XM_020091252.1	potassium channel subfamily K member 13-like	1233	0
XM_020091253.1	adrenocorticotropic hormone receptor	1127	0
XM_020091254.1	C-C chemokine receptor type 2-like	1362	0
XM_020091255.1	homeodomain-interacting protein kinase 1-like	398	5.40E-95
XM_020091256.1	prostate stem cell antigen-like	756	2.31E-10
XM_020091257.1	plectin-like isoform X1	17540	0
XM_020091258.1	plectin-like isoform X2	17504	0
XM_020091259.1	plectin-like isoform X3	17498	0
XM_020091260.1	homeobox protein prophet of Pit-1-like	806	9.46E-174
XM_020091261.1	plectin-like isoform X4	17486	0
XM_020091262.1	microtubule-actin cross-linking factor 1-like	8434	0
XM_020091263.1	microtubule-actin cross-linking factor 1-like	8230	0
XM_020091264.1	dendritic cell-specific transmembrane protein	1447	0
XM_020091265.1	sperm-associated antigen 1	4726	0
XM_020091266.1	E3 ubiquitin-protein ligase RNF19A	5215	0
XM_020091267.1	F-box only protein 43 isoform X1	2642	0
XM_020091268.1	F-box only protein 43 isoform X2	2634	0
XM_020091269.1	dual specificity tyrosine-phosphorylation-regulated kinase 1A isoform X1	5800	0
XM_020091270.1	dual specificity tyrosine-phosphorylation-regulated kinase 1A isoform X1	4224	0
XM_020091271.1	rRNA 2'-O-methyltransferase fibrillarin	1622	3.75E-171

XM_020091272.1	zinc fingers and homeoboxes protein 2-like	4794	0
XM_020091273.1	zinc fingers and homeoboxes protein 2-like	4556	0

XM_020091274.1	derlin-2 isoform X3	1298	1.40E-164
XM_020091275.1	E3 ubiquitin-protein ligase rnf146-like	1487	0
XM_020091276.1	malate dehydrogenase, mitochondrial	1554	0
XM_020091277.1	succinate dehydrogenase [ubiquinone] flavoprotein subunit, mitochondrial	2925	0
XM_020091278.1	coiled-coil domain-containing protein 127 isoform X2	1892	1.19E-146
XM_020091279.1	protein atonal homolog 7-like	902	3.88E-77
XM_020091280.1	coiled-coil domain-containing protein 127 isoform X2	1750	1.13E-141
XM_020091281.1	coiled-coil domain-containing protein 127 isoform X2	1738	1.07E-140
XM_020091282.1	PREDICTED: telethonin-like	1519	2.07E-114
XM_020091283.1	PREDICTED: frizzled-1-like	4452	0
XM_020091284.1	cyclin-dependent kinase 14 isoform X1	6270	0
XM_020091285.1	cyclin-dependent kinase 14 isoform X1	5853	0
XM_020091286.1	cyclin-dependent kinase 14 isoform X3	1670	0
XM_020091287.1	sister chromatid cohesion protein DCC1	1558	0
XM_020091288.1	39S ribosomal protein L13, mitochondrial	1291	1.28E-132
XM_020091289.1	focal adhesion kinase 1-like	5670	0
XM_020091290.1	catenin beta-1-like isoform X2	3345	0
XM_020091291.1	interleukin-22 receptor subunit alpha-2-like	757	1.56E-124
XM_020091292.1	metastasis suppressor protein 1-like isoform X3	6299	0
XM_020091293.1	metastasis suppressor protein 1-like isoform X2	6296	0
XM_020091294.1	metastasis suppressor protein 1-like isoform X3	6287	0
XM_020091295.1	metastasis suppressor protein 1-like isoform X4	6188	0
XM_020091296.1	metastasis suppressor protein 1-like isoform X6	6164	0
XM_020091297.1	metastasis suppressor protein 1-like isoform X6	6152	0
XM_020091298.1	metastasis suppressor protein 1-like isoform X7	3771	0
XM_020091299.1	metastasis suppressor protein 1-like isoform X10	6053	0
XM_020091300.1	metastasis suppressor protein 1-like isoform X9	6053	0
XM_020091301.1	metastasis suppressor protein 1-like isoform X10	6041	0
XM_020091302.1	transmembrane protein 65-like	3320	1.24E-147
XM_020091303.1	oxidation resistance protein 1 isoform X1	4647	0
XM_020091304.1	oxidation resistance protein 1 isoform X2	4527	0
XM_020091305.1	oxidation resistance protein 1 isoform X3	4566	0
XM_020091306.1	39S ribosomal protein L2, mitochondrial-like	394	4.64E-59
XM_020091307.1	14-3-3 protein beta/alpha-1	1801	1.87E-162
XM_020091308.1	14-3-3 protein beta/alpha-1	1763	1.25E-162
XM_020091309.1	transcription factor E2F3	5106	0
XM_020091310.1	polyadenylate-binding protein 1-like	2976	0
XM_020091311.1	triple functional domain protein	10709	0
XM_020091312.1	protein dopey-1 isoform X1	8555	0
XM_020091313.1	protein dopey-1 isoform X2	8525	0
XM_020091314.1	CCR4-NOT transcription complex subunit 10 isoform X1	2648	0
XM_020091315.1	CCR4-NOT transcription complex subunit 10 isoform X2	2645	0
XM_020091316.1	CCR4-NOT transcription complex subunit 10 isoform X1	2507	0
XM_020091317.1	T-cell activation inhibitor, mitochondrial isoform X1	3692	0
XM_020091318.1	urokinase plasminogen activator surface receptor-like	1389	1.32E-55
XM_020091319.1	T-cell activation inhibitor, mitochondrial isoform X1	1591	0
XM_020091320.1	ankyrin repeat and IBR domain-containing protein 1	5870	0
XM_020091321.1	ankyrin repeat and IBR domain-containing protein 1	5815	0
XM_020091322.1	protein lifeguard 1-like	1331	0
XM_020091323.1	protein lifeguard 1-like	1275	0
XM_020091324.1	probable ATP-dependent DNA helicase HFM1 isoform X1	8248	0
XM_020091325.1	probable ATP-dependent DNA helicase HFM1 isoform X1	8238	0
XM_020091326.1	zinc finger with UFM1-specific peptidase domain protein isoform X3	2055	0
XM_020091327.1	zinc finger with UFM1-specific peptidase domain protein isoform X3	1946	0
XM_020091328.1	zinc finger with UFM1-specific peptidase domain protein isoform X3	2086	0
XM_020091329.1	zinc finger with UFM1-specific peptidase domain protein isoform X3	2059	0
XM_020091330.1	zinc finger with UFM1-specific peptidase domain protein isoform X3	2058	0
XM_020091331.1	zinc finger with UFM1-specific peptidase domain protein isoform X3	2042	0
XM_020091332.1	prokineticin receptor 1-like	1174	0
XM_020091333.1	importin subunit alpha-6 isoform X1	2911	0
XM_020091334.1	importin subunit alpha-6 isoform X2	1732	0
XM_020091335.1	PREDICTED: exostosin-1	5178	0
XM_020091336.1	G protein-regulated inducer of neurite outgrowth 1	3941	0
XM_020091337.1	protein NDRG1-like isoform X1	1628	0
XM_020091338.1	protein NDRG1-like isoform X1	1539	0
XM_020091339.1	protein tyrosine phosphatase type IVA 3 isoform X1	2898	3.95E-117
XM_020091340.1	protein tyrosine phosphatase type IVA 3 isoform X1	2668	7.41E-118
XM_020091341.1	protein tyrosine phosphatase type IVA 3 isoform X1	2716	1.06E-117
XM_020091342.1	protein tyrosine phosphatase type IVA 3 isoform X1	2825	6.74E-98
XM_020091343.1	anoctamin-8-like isoform X2	3217	0
XM_020091344.1	1-acylglycerol-3-phosphate O-acyltransferase ABHD5	3438	0
XM_020091345.1	probable rhodanese domain-containing dual specificity protein phosphatase	835	6.63E-142
XM_020091346.1	transcription factor SOX-4-like	4508	3.57E-168
XM_020091347.1	arginine/serine-rich protein PNISR isoform X1	2974	0
XM_020091348.1	arginine/serine-rich protein PNISR isoform X1	3220	0
XM_020091349.1	arginine/serine-rich protein PNISR isoform X1	1371	0
XM_020091350.1	transcription initiation factor TFIID subunit 2	5185	0

XM_020091351.1	centrosomal protein of 85 kDa	4425	0
XM_020091352.1	centrosomal protein of 85 kDa	4289	0
XM_020091353.1	carboxypeptidase Q	2154	0
XM_020091354.1	trafficking kinesin-binding protein 1-like isoform X1	6510	0
XM_020091355.1	visual pigment-like receptor peropsin	1239	0
XM_020091356.1	trafficking kinesin-binding protein 1-like isoform X1	6990	0
XM_020091357.1	trafficking kinesin-binding protein 1-like isoform X3	2758	0
XM_020091358.1	double-strand-break repair protein rad21 homolog	2877	0
XM_020091359.1	tyrosine-protein phosphatase non-receptor type 2-like	2273	0
XM_020091360.1	ubiquitin carboxyl-terminal hydrolase 45 isoform X1	5701	0
XM_020091361.1	ubiquitin carboxyl-terminal hydrolase 45 isoform X2	5698	0
XM_020091362.1	F-actin-uncapping protein LRRC16A	4997	0
XM_020091363.1	neurocalcin-delta A	3221	3.01E-133
XM_020091364.1	neurocalcin-delta A	3300	5.27E-133
XM_020091365.1	neurocalcin-delta A	3195	2.54E-133
XM_020091366.1	PRELI domain containing protein 3A isoform X1	2565	2.57E-119
XM_020091367.1	PRELI domain containing protein 3A isoform X2	2490	7.70E-117
XM_020091368.1	maestro heat-like repeat-containing protein family member 1 isoform X1	6712	0
XM_020091369.1	maestro heat-like repeat-containing protein family member 1 isoform X2	6700	0
XM_020091370.1	maestro heat-like repeat-containing protein family member 1 isoform X3	6673	0
XM_020091371.1	non-canonical poly(A) RNA polymerase PAPD7-like isoform X1	4742	0
XM_020091372.1	non-canonical poly(A) RNA polymerase PAPD7-like isoform X2	4720	0
XM_020091373.1	non-canonical poly(A) RNA polymerase PAPD7-like isoform X3	4688	0
XM_020091374.1	threonylcarbamoyladenosine tRNA methylthiotransferase	2695	0
XM_020091375.1	magnesium transporter MRS2 homolog, mitochondrial isoform X1	3322	0
XM_020091376.1	magnesium transporter MRS2 homolog, mitochondrial isoform X2	2638	0
XM_020091377.1	integrin beta-1-like isoform X1	1604	0
XM_020091378.1	integrin beta-1-like isoform X2	1431	0
XM_020091379.1	glycerol-3-phosphate dehydrogenase 1-like protein	3186	0
XM_020091380.1	glycerol-3-phosphate dehydrogenase 1-like protein	2371	0
XM_020091381.1	glycerol-3-phosphate dehydrogenase 1-like protein	1883	0
XM_020091382.1	protoporphyrinogen oxidase	2531	0
XM_020091383.1	SPARC-like protein 1	936	0
XM_020091384.1	RNA-binding protein 42	1703	0
XM_020091385.1	nucleoporin SEH1 isoform X2	1467	0
XM_020091386.1	nucleoporin SEH1 isoform X2	1619	0
XM_020091387.1	nucleoporin SEH1 isoform X2	1552	0
XM_020091388.1	microtubule cross-linking factor 1-like	4253	0
XM_020091389.1	SNF-related serine/threonine-protein kinase-like	5646	0
XM_020091390.1	ATPase family AAA domain-containing protein 2 isoform X1	4517	0
XM_020091391.1	ATPase family AAA domain-containing protein 2 isoform X2	4502	0
XM_020091392.1	syndecan-4-like isoform X1	3268	9.57E-133
XM_020091393.1	glyceraldehyde-3-phosphate dehydrogenase	1333	0
XM_020091394.1	centrosomal protein of 170 kDa isoform X1	4982	0
XM_020091395.1	PREDICTED: frizzled-6	2579	0
XM_020091396.1	protein cordon-bleu isoform X1	6652	0
XM_020091397.1	protein cordon-bleu isoform X1	6593	0
XM_020091398.1	protein cordon-bleu isoform X1	6607	0
XM_020091399.1	protein cordon-bleu isoform X1	6580	0
XM_020091400.1	protein cordon-bleu isoform X4	6535	0
XM_020091401.1	protein cordon-bleu isoform X1	6490	0
XM_020091402.1	phosphatidylinositide phosphatase SAC1-A	3043	0
XM_020091403.1	centrosomal protein of 170 kDa isoform X2	7466	0
XM_020091404.1	growth factor receptor-bound protein 10-like isoform X1	6218	0
XM_020091405.1	growth factor receptor-bound protein 10-like isoform X2	6215	0
XM_020091406.1	growth factor receptor-bound protein 10-like isoform X3	6216	0
XM_020091407.1	growth factor receptor-bound protein 10-like isoform X4	5605	0
XM_020091408.1	DEP domain-containing mTOR-interacting protein isoform X1	3688	0
XM_020091409.1	DEP domain-containing mTOR-interacting protein isoform X2	3685	0
XM_020091410.1	G-protein coupled receptor family C group 6 member A	4379	0
XM_020091411.1	protein cornichon homolog 4	1621	3.67E-95
XM_020091412.1	centrosomal protein of 170 kDa isoform X3	7445	0
XM_020091413.1	PREDICTED: uncharacterized protein LOC109632225 isoform X1	1977	0
XM_020091414.1	PREDICTED: uncharacterized protein LOC109632225 isoform X2	1933	0
XM_020091415.1	RRP15-like protein	1222	5.58E-118
XM_020091416.1	WD repeat-containing protein 26-like	3549	0
XM_020091417.1	glutamate receptor ionotropic, kainate 2 isoform X1	5016	0
XM_020091418.1	glutamate receptor ionotropic, kainate 2 isoform X2	4988	0
XM_020091419.1	protein Jumonji-like	6078	0
XM_020091420.1	WASH complex subunit strumpellin	4022	0
XM_020091421.1	centrosomal protein of 170 kDa isoform X4	7445	0
XM_020091422.1	WASH complex subunit strumpellin	3993	0
XM_020091423.1	E3 SUMO-protein ligase NSE2	1211	5.50E-171
XM_020091424.1	F-box/LRR-repeat protein 4 isoform X1	4881	0
XM_020091425.1	F-box/LRR-repeat protein 4 isoform X1	4986	0
XM_020091426.1	eukaryotic translation initiation factor 3 subunit H	1329	0
XM_020091427.1	dihydropyrimidinase-related protein 3 isoform X2	1841	0
XM_020091428.1	centrosomal protein of 170 kDa isoform X5	7394	0
XM_020091429.1	aldehyde dehydrogenase, mitochondrial	2062	0

XM_020091430.1	little elongation complex subunit 1	5266	0
XM_020091431.1	proteasome subunit alpha type-2	1583	5.82E-165
XM_020091432.1	protein phosphatase 1 regulatory subunit 35-like	934	3.51E-177
XM_020091433.1	E3 ubiquitin-protein ligase HACE1 isoform X1	5126	0
XM_020091434.1	E3 ubiquitin-protein ligase HACE1 isoform X2	5090	0
XM_020091435.1	protein MMS22-like	4126	0
XM_020091436.1	centrosomal protein of 170 kDa isoform X1	6556	0
XM_020091437.1	receptor-binding cancer antigen expressed on SiSo cells	1405	6.16E-142

XM_020091438.1	dynein heavy chain 5, axonemal	14490	0
XM_020091439.1	DDB1- and CUL4-associated factor 13	1868	0
XM_020091440.1	LIM domain-containing protein 1	3920	0
XM_020091441.1	prolactin regulatory element-binding protein isoform X1	2262	0
XM_020091442.1	prolactin regulatory element-binding protein isoform X2	2262	0
XM_020091443.1	trophoblast glycoprotein-like	2239	0
XM_020091444.1	F-box only protein 32	2424	0
XM_020091445.1	mitochondrial folate transporter/carrier	1737	0
XM_020091446.1	collagen triple helix repeat-containing protein 1 isoform X1	1101	5.19E-154
XM_020091447.1	collagen triple helix repeat-containing protein 1 isoform X2	1081	1.09E-142
XM_020091448.1	rhotekin-2-like isoform X1	4680	0
XM_020091449.1	dual specificity protein kinase TTK isoform X1	3431	0
XM_020091450.1	dual specificity protein kinase TTK isoform X2	3428	0
XM_020091451.1	serine/threonine-protein kinase ULK4 isoform X1	4499	0
XM_020091452.1	serine/threonine-protein kinase ULK4 isoform X1	4295	0
XM_020091453.1	PREDICTED: uncharacterized protein KIAA1143 homolog	2192	7.62E-84
XM_020091454.1	5-azacytidine-induced protein 2 isoform X1	3698	0
XM_020091455.1	5-azacytidine-induced protein 2 isoform X1	3866	0
XM_020091456.1	5-azacytidine-induced protein 2 isoform X1	3692	0
XM_020091457.1	rhotekin-2-like isoform X2	4578	0
XM_020091458.1	eukaryotic translation initiation factor 3 subunit E-A	1540	0
XM_020091459.1	riboflavin transporter 2-like	2866	0
XM_020091460.1	riboflavin transporter 2-like	2745	0
XM_020091461.1	probable leucine--tRNA ligase, mitochondrial	3684	0
XM_020091462.1	grainyhead-like protein 2 homolog isoform X1	3166	0
XM_020091463.1	grainyhead-like protein 2 homolog isoform X2	3085	0
XM_020091464.1	grainyhead-like protein 2 homolog isoform X3	3064	0
XM_020091465.1	actin-related protein 10	2148	0
XM_020091466.1	ATP-binding cassette sub-family G member 8	3321	0
XM_020091467.1	visinin-like protein 1	1151	5.66E-140
XM_020091468.1	rho GTPase-activating protein 18 isoform X1	3706	0
XM_020091469.1	rho GTPase-activating protein 18 isoform X2	3704	0
XM_020091470.1	radial spoke head protein 9 homolog isoform X1	1115	0
XM_020091471.1	radial spoke head protein 9 homolog isoform X2	1110	0
XM_020091472.1	radial spoke head protein 9 homolog isoform X3	1063	4.98E-169
XM_020091473.1	28S ribosomal protein S18a, mitochondrial	1049	4.25E-153
XM_020091474.1	peptidyl-prolyl cis-trans isomerase-like 4	1690	0
XM_020091475.1	NF-kappa-B inhibitor alpha-like	1398	0
XM_020091476.1	alpha-1,6-mannosyl-glycoprotein 2-beta-N-acetylglucosaminyltransferase-like	1994	0
XM_020091477.1	alpha-1,6-mannosyl-glycoprotein 2-beta-N-acetylglucosaminyltransferase-like	2004	0
XM_020091478.1	ATP-binding cassette sub-family G member 5	2351	0
XM_020091479.1	NADH dehydrogenase [ubiquinone] 1 alpha subcomplex assembly factor 4	2245	7.78E-100
XM_020091480.1	40S ribosomal protein S12	525	1.02E-90
XM_020091481.1	SH3 domain-binding glutamic acid-rich-like protein 2	2411	4.75E-70
XM_020091482.1	proteasome subunit alpha type-6-like	1017	0
XM_020091483.1	katanin p60 ATPase-containing subunit A1	2247	0
XM_020091484.1	katanin p60 ATPase-containing subunit A1	2245	0
XM_020091485.1	katanin p60 ATPase-containing subunit A1	2455	0
XM_020091486.1	dermatan-sulfate epimerase isoform X1	4392	0
XM_020091487.1	dermatan-sulfate epimerase isoform X1	4007	0
XM_020091488.1	inositol-trisphosphate 3-kinase B isoform X1	3120	0
XM_020091489.1	inositol-trisphosphate 3-kinase B isoform X2	3117	0
XM_020091490.1	inositol-trisphosphate 3-kinase B isoform X3	3108	0
XM_020091491.1	complex III assembly factor LYRM7	800	5.13E-98
XM_020091492.1	CSC1-like protein 1	4126	0
XM_020091493.1	protein FAM177A1-like isoform X1	1804	5.11E-106
XM_020091494.1	protein FAM177A1-like isoform X2	1802	1.40E-104
XM_020091495.1	zinc finger protein 395-like	3436	0
XM_020091496.1	zinc finger protein 395-like	3423	0
XM_020091497.1	zinc finger protein 395-like	3322	0
XM_020091498.1	homeobox protein OTX2-like	1957	1.30E-154
XM_020091499.1	ribosome production factor 2 homolog	1337	0
XM_020091500.1	PREDICTED: stathmin-4	998	1.92E-118
XM_020091501.1	thyroxine 5-deiodinase-like	1702	0
XM_020091502.1	histidine triad nucleotide-binding protein 1	562	1.95E-90
XM_020091503.1	homeobox protein Meis2-like	2263	0
XM_020091504.1	signal recognition particle 9 kDa protein	1173	5.68E-106
XM_020091505.1	cAMP-specific 3',5'-cyclic phosphodiesterase 7B isoform X1	5010	0
XM_020091506.1	cAMP-specific 3',5'-cyclic phosphodiesterase 7B isoform X1	4840	0
XM_020091507.1	cAMP-specific 3',5'-cyclic phosphodiesterase 7B isoform X1	4734	0
XM_020091508.1	cAMP-specific 3',5'-cyclic phosphodiesterase 7B isoform X1	3906	0

XM_020091509.1	POU domain, class 3, transcription factor 1	3305	0
XM_020091510.1	autophagy-related protein 11-like	3856	0
XM_020091511.1	autophagy-related protein 11-like	3812	0
XM_020091512.1	sodium/potassium-transporting ATPase subunit beta-1-interacting protein 2	5796	8.83E-140
XM_020091513.1	phospholipase DDHD1-like	4329	0
XM_020091514.1	solute carrier family 22 member 13-like	3120	0
XM_020091515.1	CSC1-like protein 2 isoform X1	4689	0
XM_020091516.1	WNT1-inducible-signaling pathway protein 3	1350	0
XM_020091517.1	centrosomal protein of 85 kDa-like isoform X1	2638	0
XM_020091518.1	centrosomal protein of 85 kDa-like isoform X2	2635	0
XM_020091519.1	centrosomal protein of 85 kDa-like isoform X3	2629	0
XM_020091520.1	centrosomal protein of 85 kDa-like isoform X4	2587	0
XM_020091521.1	centrosomal protein of 85 kDa-like isoform X5	2470	0
XM_020091522.1	PREDICTED: vertnin	3448	0
XM_020091523.1	saccharopine dehydrogenase-like oxidoreductase	1846	0
XM_020091524.1	CSC1-like protein 2 isoform X2	4648	0
XM_020091525.1	epoxide hydrolase 1	1826	0
XM_020091526.1	epoxide hydrolase 1	1806	0
XM_020091527.1	epoxide hydrolase 1	1810	0
XM_020091528.1	cholesterol 24-hydroxylase	2324	0
XM_020091529.1	failed axon connections homolog	4492	0
XM_020091530.1	protein FAM84B-like	1884	0
XM_020091531.1	transcription regulator protein BACH2-like	2793	0
XM_020091532.1	ankyrin repeat and EF-hand domain-containing protein 1 isoform X2	2421	0
XM_020091533.1	ankyrin repeat and EF-hand domain-containing protein 1 isoform X2	3667	0
XM_020091534.1	dihydroxyacetone phosphate acyltransferase-like	2634	0
XM_020091535.1	glycerophosphoinositol inositolphosphodiesterase GDPD2 isoform X1	3361	0
XM_020091536.1	calpain-1 catalytic subunit-like	3186	0
XM_020091537.1	UBX domain-containing protein 2A	1614	0
XM_020091538.1	syntaxin-binding protein 6-like	2021	0
XM_020091539.1	leucine-rich repeat and fibronectin type III domain-containing protein 1	4116	0
XM_020091540.1	calpain-1 catalytic subunit-like	3181	0
XM_020091541.1	PREDICTED: calmin	4647	0
XM_020091542.1	PREDICTED: uncharacterized protein LOC109632318	1348	0
XM_020091543.1	patched domain-containing protein 4	4920	0
XM_020091544.1	isthmin-1 isoform X2	5111	0
XM_020091545.1	PREDICTED: chromogranin-A	2163	0
XM_020091546.1	transmembrane protein 200A	2350	0
XM_020091547.1	transmembrane protein 200A	3539	0
XM_020091548.1	synapse differentiation-inducing gene protein 1-like	4068	5.68E-114
XM_020091549.1	RAD51-associated protein 2	4448	0
XM_020091550.1	RAD51-associated protein 2	4462	0
XM_020091551.1	tyrosine-protein kinase Lyn-like	1272	0
XM_020091552.1	calpain-1 catalytic subunit-like	2302	0
XM_020091553.1	complement C1q-like protein 2	1044	0
XM_020091554.1	transcriptional activator Myb	3486	0
XM_020091555.1	neuroblastoma-amplified sequence	7651	0
XM_020091556.1	estrogen-related receptor gamma-like	3048	0
XM_020091557.1	dynein regulatory complex subunit 3-like	1696	0
XM_020091558.1	adenylate cyclase type 3-like	3938	0
XM_020091559.1	CSC1-like protein 1	2568	0
XM_020091560.1	PREDICTED: uncharacterized protein LOC109632335	2117	0
XM_020091561.1	interleukin-20 receptor subunit alpha-like	2144	0
XM_020091562.1	delta-like protein 4	1088	8.68E-177
XM_020091563.1	39S ribosomal protein L14, mitochondrial	709	1.71E-104
XM_020091564.1	PREDICTED: uncharacterized protein LOC109632338	942	0
XM_020091565.1	solute carrier family 25 member 45-like	2269	0
XM_020091566.1	ena/VASP-like protein	2596	0
XM_020091567.1	gonadotropin-releasing hormone receptor-like	933	0
XM_020091568.1	MAX gene-associated protein-like	5650	0
XM_020091569.1	lysosome-associated membrane glycoprotein 5	1569	0
XM_020091570.1	39S ribosomal protein L14, mitochondrial	582	3.31E-105
XM_020091571.1	major facilitator superfamily domain-containing protein 2B-like	1241	0
XM_020091572.1	PREDICTED: opsin-5-like	1071	0
XM_020091573.1	runt-related transcription factor 2-like	1216	0
XM_020091574.1	ectonucleotide pyrophosphatase/phosphodiesterase family member 5-like	3452	1.62E-124
XM_020091575.1	PH-interacting protein	9278	0
XM_020091576.1	B-cell lymphoma/leukemia 11B-like	3339	0
XM_020091577.1	carcinoembryonic antigen-related cell adhesion molecule 5-like	3657	0
XM_020091578.1	homeobox-containing protein 1	5144	0
XM_020091579.1	intraflagellar transport protein 20 homolog	441	1.20E-77
XM_020091580.1	adhesion G protein-coupled receptor F5-like	1428	0
XM_020091581.1	actin-binding LIM protein 1 isoform X1	4037	0
XM_020091582.1	trace amine-associated receptor 1-like	1077	0
XM_020091583.1	trace amine-associated receptor 1-like	936	0
XM_020091584.1	adhesion G-protein coupled receptor F1-like	2727	0
XM_020091585.1	transcription factor 24-like	812	4.33E-175
XM_020091586.1	ubiquitin-conjugating enzyme E2 J1	3547	2.95E-124
XM_020091587.1	gamma-aminobutyric acid receptor subunit rho-2-like	1852	0

XM_020091588.1	PREDICTED: midasin-like	5767	0
XM_020091589.1	actin-binding LIM protein 1 isoform X2	3988	0
XM_020091590.1	PREDICTED: midasin-like	8716	0
XM_020091591.1	ephrin type-A receptor 7	6457	0
XM_020091592.1	laminin subunit alpha-4	5789	0
XM_020091593.1	tubulin epsilon chain	1696	0
XM_020091594.1	DNA polymerase zeta catalytic subunit	1490	0
XM_020091595.1	monocarboxylate transporter 10	1530	0
XM_020091596.1	actin-binding LIM protein 1 isoform X3	3756	0
XM_020091597.1	cyclin-dependent kinase 19-like	436	1.77E-82
XM_020091598.1	B2 bradykinin receptor-like	1206	0
XM_020091599.1	regulator of microtubule dynamics protein 2-like	2007	0
XM_020091600.1	actin-binding LIM protein 1 isoform X4	3965	0
XM_020091601.1	centromere-associated protein E	6288	0
XM_020091602.1	zinc finger protein 292-like	1435	0
XM_020091603.1	mitogen-activated protein kinase-binding protein 1	5929	0
XM_020091604.1	1-phosphatidylinositol 4,5-bisphosphate phosphodiesterase beta-1	4035	0
XM_020091605.1	glycerophosphoinositol inositolphosphodiesterase GDPD2 isoform X2	3358	0
XM_020091606.1	1-phosphatidylinositol 4,5-bisphosphate phosphodiesterase beta-4 isoform X1	4744	0
XM_020091607.1	1-phosphatidylinositol 4,5-bisphosphate phosphodiesterase beta-4 isoform X1	4722	0
XM_020091608.1	1-phosphatidylinositol 4,5-bisphosphate phosphodiesterase beta-4 isoform X1	4432	0
XM_020091609.1	1-phosphatidylinositol 4,5-bisphosphate phosphodiesterase beta-4 isoform X1	4495	0
XM_020091610.1	actin-binding LIM protein 1 isoform X5	3829	0
XM_020091611.1	bromo adjacent homology domain-containing 1 protein-like	3970	0
XM_020091612.1	bromo adjacent homology domain-containing 1 protein-like	3482	0
XM_020091613.1	kelch-like protein 21	4609	0
XM_020091614.1	kelch-like protein 21	4615	0
XM_020091615.1	kelch-like protein 21	4541	0
XM_020091616.1	GDNF-inducible zinc finger protein 1-like	3086	0
XM_020091617.1	serine/threonine-protein kinase PAK 5 isoform X1	4292	0
XM_020091618.1	serine/threonine-protein kinase PAK 5 isoform X2	4083	0
XM_020091619.1	actin-binding LIM protein 1 isoform X6	3709	0
XM_020091620.1	serine/threonine-protein kinase PAK 5 isoform X3	4246	0
XM_020091621.1	serine/threonine-protein kinase PAK 5 isoform X2	4208	0
XM_020091622.1	serine/threonine-protein kinase PAK 5 isoform X5	3919	0
XM_020091623.1	serine/threonine-protein kinase PAK 5 isoform X6	3861	0
XM_020091624.1	glycerophosphocholine phosphodiesterase GPCPD1 isoform X1	3743	0
XM_020091625.1	glycerophosphocholine phosphodiesterase GPCPD1 isoform X1	3651	0
XM_020091626.1	fermitin family homolog 1	2617	0
XM_020091627.1	solute carrier family 23 member 1-like	1979	0
XM_020091628.1	actin-binding LIM protein 1 isoform X7	3890	0
XM_020091629.1	PREDICTED: secretogranin-1	2042	0
XM_020091630.1	tRNA (adenine(58)-N(1))-methyltransferase non-catalytic subunit TRM6	2131	0
XM_020091631.1	bone morphogenetic protein 2	2008	0
XM_020091632.1	transcriptional repressor protein YY1-like isoform X1	2269	0
XM_020091633.1	transcriptional repressor protein YY1-like isoform X2	1400	0
XM_020091634.1	tribbles homolog 2-like	2545	0
XM_020091635.1	galectin-3-like isoform X1	2232	0
XM_020091636.1	galectin-3-like isoform X2	1059	1.33E-179
XM_020091637.1	actin-binding LIM protein 1 isoform X8	3806	0
XM_020091638.1	echinoderm microtubule-associated protein-like 1	1294	9.94E-163
XM_020091639.1	beta-soluble NSF attachment protein-like isoform X1	2202	0
XM_020091640.1	beta-soluble NSF attachment protein-like isoform X2	2156	5.61E-151
XM_020091641.1	splicing factor 3B subunit 6	926	1.75E-77
XM_020091642.1	thyrotropin receptor	2410	0
XM_020091643.1	gap junction alpha-10 protein	1553	0
XM_020091644.1	actin-binding LIM protein 1 isoform X9	3784	0
XM_020091645.1	gamma-crystallin M3-like	724	7.11E-116
XM_020091646.1	meprin A subunit alpha-like	1833	0
XM_020091647.1	triadin isoform X1	1107	3.03E-111
XM_020091648.1	triadin isoform X2	1081	4.57E-103
XM_020091649.1	triadin isoform X3	1065	1.29E-100
XM_020091650.1	triadin isoform X4	1066	1.45E-98
XM_020091651.1	triadin isoform X5	1039	2.54E-92
XM_020091652.1	cyclin-dependent kinase 19-like	1555	0
XM_020091653.1	probable ribonuclease ZC3H12D	2385	0
XM_020091654.1	probable ribonuclease ZC3H12D	2308	0
XM_020091655.1	meprin A subunit alpha-like	2016	0
XM_020091656.1	adapter protein CIKS	1930	0
XM_020091657.1	adapter protein CIKS	1824	0
XM_020091658.1	proenkephalin-A-like isoform X1	1417	1.22E-171
XM_020091659.1	proenkephalin-A-like isoform X2	1276	3.32E-143
XM_020091660.1	protein cornichon homolog 3 isoform X1	1279	3.45E-102
XM_020091661.1	actin-binding LIM protein 1 isoform X10	3643	0
XM_020091662.1	protein cornichon homolog 3 isoform X2	1335	1.04E-101
XM_020091663.1	protein cornichon homolog 3 isoform X3	1276	1.58E-101
XM_020091664.1	solute carrier family 35 member F1-like isoform X1	2873	0
XM_020091665.1	solute carrier family 35 member F1-like isoform X2	2870	0
XM_020091666.1	transcription factor 21	1048	1.82E-106

XM_020091667.1	phosphatidate phosphatase LPIN1	2090	0
XM_020091668.1	fibronectin type III domain-containing protein 4	2355	2.24E-146
XM_020091669.1	gamma-aminobutyric acid receptor subunit rho-1-like	1587	0
XM_020091670.1	leucine-rich repeat-containing protein 15-like	1826	0
XM_020091671.1	leucine-rich repeat-containing protein 15-like	1597	0
XM_020091672.1	hyaluronan-binding protein 2-like	2170	0
XM_020091673.1	inactive peptidyl-prolyl cis-trans isomerase FKBP6	1347	0
XM_020091674.1	inactive peptidyl-prolyl cis-trans isomerase FKBP6	1346	0
XM_020091675.1	inactive peptidyl-prolyl cis-trans isomerase FKBP6	1238	0
XM_020091676.1	potassium channel subfamily K member 10-like	2716	0
XM_020091677.1	protein FAM177B	792	2.77E-119
XM_020091678.1	SH3 domain-binding glutamic acid-rich-like protein 3	584	2.24E-53
XM_020091679.1	PREDICTED: teneurin-1-like	2865	0
XM_020091680.1	PREDICTED: opsin-5	1736	0
XM_020091681.1	heparan sulfate glucosamine 3-O-sulfotransferase 5	1692	0
XM_020091682.1	sex comb on midleg-like protein 4	2384	1.16E-176
XM_020091683.1	protein FAM160B1 isoform X2	5120	0

XM_020091684.1	vascular endothelial growth factor A-like isoform X1	842	6.61E-113
XM_020091685.1	vascular endothelial growth factor A-like isoform X2	795	1.84E-112
XM_020091686.1	vascular endothelial growth factor A-like isoform X3	1415	3.51E-109
XM_020091687.1	vascular endothelial growth factor A-like isoform X4	796	1.30E-111
XM_020091688.1	glycerophosphoinositol inositolphosphodiesterase GDPD2 isoform X3	3332	0
XM_020091689.1	PREDICTED: uncharacterized protein C14orf37 homolog isoform X1	3114	0
XM_020091690.1	PREDICTED: uncharacterized protein C14orf37 homolog isoform X2	3099	0
XM_020091691.1	protein FAM167A	2082	3.86E-145
XM_020091692.1	protein FAM160B1 isoform X2	5485	0
XM_020091693.1	hormonally up-regulated neu tumor-associated kinase homolog	2740	0
XM_020091694.1	dynein intermediate chain 1, axonemal-like	2133	0
XM_020091695.1	SH2 domain-containing protein 1A-like	1021	1.05E-101
XM_020091696.1	prostaglandin E2 receptor EP2 subtype-like	1223	0
XM_020091697.1	PREDICTED: interleukin-17C-like	1227	5.33E-74
XM_020091698.1	potassium channel subfamily K member 13-like	1436	0
XM_020091699.1	gap junction delta-2 protein-like	1043	0
XM_020091700.1	E3 ubiquitin-protein ligase TRIM39-like	2815	0
XM_020091701.1	protein stum homolog	1444	2.63E-76
XM_020091702.1	sterile alpha motif domain-containing protein 15	1139	1.96E-92
XM_020091703.1	phospholipase B1, membrane-associated-like	1696	0
XM_020091704.1	transmembrane protein 244	702	1.65E-127
XM_020091705.1	probable G-protein coupled receptor 139	1089	0
XM_020091706.1	kinesin-like protein KIF13B isoform X3	7190	0
XM_020091707.1	kinesin-like protein KIF13B isoform X3	7187	0
XM_020091708.1	kinesin-like protein KIF13B isoform X3	7507	0
XM_020091709.1	kinesin-like protein KIF13B isoform X3	7357	0
XM_020091710.1	kinesin-like protein KIF13B isoform X4	7154	0
XM_020091711.1	serum response factor-like	1709	5.78E-136
XM_020091712.1	kinesin-like protein KIF13B isoform X5	7133	0
XM_020091713.1	kinesin-like protein KIF13B isoform X6	7097	0
XM_020091714.1	elongator complex protein 3	2679	0
XM_020091715.1	protein enabled homolog isoform X5	3977	2.04E-89
XM_020091716.1	protein enabled homolog isoform X2	4020	4.94E-88
XM_020091717.1	protein enabled homolog isoform X5	4328	4.40E-86
XM_020091718.1	protein enabled homolog isoform X5	3326	1.54E-90
XM_020091719.1	protein enabled homolog isoform X5	3173	3.41E-91
XM_020091720.1	lamin-B receptor isoform X1	4133	0
XM_020091721.1	lamin-B receptor isoform X1	4168	0
XM_020091722.1	lamin-B receptor isoform X1	4109	0
XM_020091723.1	lamin-B receptor isoform X1	4131	0
XM_020091724.1	lamin-B receptor isoform X1	4115	0
XM_020091725.1	lamin-B receptor isoform X1	4113	0
XM_020091726.1	aurora kinase A isoform X1	1535	0
XM_020091727.1	baculoviral IAP repeat-containing protein 6 isoform X1	16209	0
XM_020091728.1	lamin-B receptor isoform X1	4100	0
XM_020091729.1	CASP8-associated protein 2	7027	0
XM_020091730.1	band 4.1-like protein 2 isoform X1	6762	0
XM_020091731.1	band 4.1-like protein 2 isoform X1	6777	0
XM_020091732.1	band 4.1-like protein 2 isoform X1	6734	0
XM_020091733.1	band 4.1-like protein 2 isoform X1	6710	0
XM_020091734.1	band 4.1-like protein 2 isoform X4	6683	0
XM_020091735.1	band 4.1-like protein 2 isoform X1	6648	0
XM_020091736.1	band 4.1-like protein 2 isoform X6	6649	0
XM_020091737.1	baculoviral IAP repeat-containing protein 6 isoform X2	16206	0
XM_020091738.1	band 4.1-like protein 2 isoform X7	3268	0
XM_020091739.1	band 4.1-like protein 2 isoform X8	6480	0
XM_020091740.1	band 4.1-like protein 2 isoform X9	6411	0
XM_020091741.1	left-right determination factor 2-like	1486	0
XM_020091742.1	heterogeneous nuclear ribonucleoprotein U	4686	0
XM_020091743.1	kinesin-like protein KIF26A	6883	0
XM_020091744.1	nutritionally-regulated adipose and cardiac enriched protein homolog isoform X2	4242	0
XM_020091745.1	baculoviral IAP repeat-containing protein 6 isoform X3	16179	0

XM_020091746.1	T-box transcription factor TBX18-like	2911	0
XM_020091747.1	interleukin-1 receptor-associated kinase 1-binding protein 1	938	2.78E-165
XM_020091748.1	PREDICTED: uncharacterized protein LOC109632461	1357	5.11E-151
XM_020091749.1	PREDICTED: uncharacterized protein LOC109632461	1355	5.81E-151
XM_020091750.1	tumor necrosis factor receptor superfamily member 21 isoform X1	4883	0
XM_020091751.1	tumor necrosis factor receptor superfamily member 21 isoform X2	5246	0
XM_020091752.1	baculoviral IAP repeat-containing protein 6 isoform X4	16119	0
XM_020091753.1	CD2-associated protein isoform X1	5741	0
XM_020091754.1	CD2-associated protein isoform X2	5738	0
XM_020091755.1	CD2-associated protein isoform X3	5642	0
XM_020091756.1	HBS1-like protein isoform X1	3177	0
XM_020091757.1	HBS1-like protein isoform X2	3199	0
XM_020091758.1	aldehyde dehydrogenase family 8 member A1	2853	0
XM_020091759.1	protein SOGA3-like isoform X1	2451	0
XM_020091760.1	protein SOGA3-like isoform X1	2431	0
XM_020091761.1	baculoviral IAP repeat-containing protein 6 isoform X5	16089	0
XM_020091762.1	protein SOGA3-like isoform X1	2865	0
XM_020091763.1	protein SOGA3-like isoform X1	2655	0
XM_020091764.1	PREDICTED: uncharacterized protein LOC109632467 isoform X2	3032	0
XM_020091765.1	PREDICTED: uncharacterized protein LOC109632467 isoform X2	2454	0

XM_020091766.1	E3 ubiquitin-protein ligase rnf146-like	2532	0
XM_020091767.1	E3 ubiquitin-protein ligase rnf146-like	2500	0
XM_020091768.1	E3 ubiquitin-protein ligase rnf146-like	2529	0
XM_020091769.1	elongation of very long chain fatty acids protein 4-like	2180	0
XM_020091770.1	baculoviral IAP repeat-containing protein 6 isoform X6	16047	0
XM_020091771.1	PREDICTED: R-spondin-3-like	1285	7.50E-136
XM_020091772.1	DEAD box protein 41	2807	0
XM_020091773.1	alpha-galactosidase A	1639	0
XM_020091774.1	alpha-galactosidase A	1756	0
XM_020091775.1	vascular endothelial growth factor A isoform X3	3709	2.07E-117
XM_020091776.1	vascular endothelial growth factor A isoform X3	1277	3.70E-121
XM_020091777.1	vascular endothelial growth factor A isoform X3	3634	1.59E-127
XM_020091778.1	dnaJ homolog subfamily C member 16-like	3942	0
XM_020091779.1	iodotyrosine deiodinase 1	986	0
XM_020091780.1	protein phosphatase 1 regulatory subunit 14C	2206	1.59E-75
XM_020091781.1	zinc finger FYVE domain-containing protein 1-like	4532	0
XM_020091782.1	neurofilament medium polypeptide-like	3034	0
XM_020091783.1	DDB1- and CUL4-associated factor 4 isoform X1	2429	0
XM_020091784.1	DDB1- and CUL4-associated factor 4 isoform X2	2407	0
XM_020091785.1	phosphatidylinositol N-acetylglucosaminyltransferase subunit H	1357	1.35E-127
XM_020091786.1	WD repeat-containing protein 26	3859	0
XM_020091787.1	ensconsin isoform X1	4264	0
XM_020091788.1	ensconsin isoform X2	4239	0
XM_020091789.1	ensconsin isoform X3	4167	0
XM_020091790.1	mitochondrial fission regulator 1	2506	0
XM_020091791.1	armadillo repeat-containing protein 1-like	1390	0
XM_020091792.1	solute carrier family 2, facilitated glucose transporter member 12	3311	0
XM_020091793.1	zinc finger protein 503	2582	0
XM_020091794.1	TATA box-binding protein-like protein 1	1099	9.56E-136
XM_020091795.1	TATA box-binding protein-like protein 1	1291	9.55E-135
XM_020091796.1	TATA box-binding protein-like protein 1	952	1.54E-136
XM_020091797.1	S-adenosylmethionine decarboxylase proenzyme	4030	0
XM_020091798.1	PRKR-interacting protein 1	1022	2.37E-66
XM_020091799.1	glutaredoxin-related protein 5, mitochondrial	2423	9.40E-107
XM_020091800.1	troponin C, skeletal muscle	1615	5.58E-100
XM_020091801.1	zinc finger protein DPF3 isoform X1	3831	0
XM_020091802.1	zinc finger protein DPF3 isoform X2	3789	0
XM_020091803.1	protein LEG1 homolog	1415	0
XM_020091804.1	syntaxin-12-like	1939	4.66E-173
XM_020091805.1	voltage-dependent anion-selective channel protein 2	1854	0
XM_020091806.1	serine/threonine-protein phosphatase 2A 56 kDa regulatory subunit gamma isoform-like isoform X1	5537	0
XM_020091807.1	serine/threonine-protein phosphatase 2A 56 kDa regulatory subunit gamma isoform-like isoform X2	1934	0
XM_020091808.1	serine/threonine-protein phosphatase 2A 56 kDa regulatory subunit gamma isoform-like isoform X3	5608	0
XM_020091809.1	serine/threonine-protein phosphatase 2A 56 kDa regulatory subunit gamma isoform-like isoform X4	1797	0
XM_020091810.1	cyclin-K isoform X1	2685	0
XM_020091811.1	cyclin-K isoform X2	2682	0
XM_020091812.1	ATPase family AAA domain-containing protein 2B isoform X1	6442	0
XM_020091813.1	ATPase family AAA domain-containing protein 2B isoform X2	4974	0
XM_020091814.1	serine/threonine-protein kinase MRCK alpha isoform X1	7093	0
XM_020091815.1	serine/threonine-protein kinase MRCK alpha isoform X2	7078	0
XM_020091816.1	serine/threonine-protein kinase MRCK alpha isoform X3	7053	0
XM_020091817.1	serine/threonine-protein kinase MRCK alpha isoform X4	7016	0
XM_020091818.1	catechol O-methyltransferase domain-containing protein 1-like	894	0
XM_020091819.1	ethanolaminephosphotransferase 1	4186	0
XM_020091820.1	dolichyl-diphosphooligosaccharide--protein glycosyltransferase subunit 4	379	1.86E-19
XM_020091821.1	ankyrin repeat domain-containing protein 6 isoform X1	4404	0
XM_020091822.1	ankyrin repeat domain-containing protein 6 isoform X1	4454	0
XM_020091823.1	ankyrin repeat domain-containing protein 6 isoform X1	4408	0
XM_020091824.1	ankyrin repeat domain-containing protein 6 isoform X1	4310	0

XM_020091825.1	ankyrin repeat domain-containing protein 6 isoform X1	4401	0
XM_020091826.1	LYR motif-containing protein 2	1162	7.04E-50
XM_020091827.1	toll-like receptor 5 membrane form	2995	0
XM_020091828.1	PREDICTED: thrombomodulin-like	3803	0
XM_020091829.1	complement component C1q receptor-like	2124	0
XM_020091830.1	leucine-rich repeat-containing protein C10orf11 homolog	2899	1.32E-133
XM_020091831.1	neurexin-3a isoform X1	6147	0
XM_020091832.1	heat shock protein HSP 90-alpha 1	2730	0
XM_020091833.1	E3 ubiquitin-protein ligase pellino homolog 2-like	5694	0
XM_020091834.1	ADP-ribosylation factor 6-like	2271	1.19E-122
XM_020091835.1	mitogen-activated protein kinase kinase kinase 7 isoform X1	3714	0
XM_020091836.1	mitogen-activated protein kinase kinase kinase 7 isoform X2	3633	0
XM_020091837.1	leucine-rich repeat transmembrane protein FLRT2	7334	0
XM_020091838.1	tyrosine-protein kinase FRK	3466	0
XM_020091839.1	N-alpha-acetyltransferase 30	3858	0
XM_020091840.1	PREDICTED: microcephalin	2244	0
XM_020091841.1	pre-mRNA-processing factor 39 isoform X1	3105	0
XM_020091842.1	pre-mRNA-processing factor 39 isoform X2	3096	0
XM_020091843.1	HD domain-containing protein 2	1161	3.72E-170
XM_020091844.1	tumor protein D53 isoform X1	2864	1.47E-123
XM_020091845.1	tumor protein D53 isoform X2	2858	2.41E-121
XM_020091846.1	tumor protein D53 isoform X3	2825	1.18E-112
XM_020091847.1	tumor protein D53 isoform X4	2819	1.70E-110
XM_020091848.1	centromere protein W	567	6.91E-34
XM_020091849.1	myristoylated alanine-rich C-kinase substrate	2351	2.89E-15
XM_020091850.1	tudor domain-containing protein 6-like	7178	0
XM_020091851.1	coiled-coil domain-containing protein 85C isoform X1	3935	0
XM_020091852.1	coiled-coil domain-containing protein 85C isoform X2	3892	0
XM_020091853.1	angiopoietin-1 isoform X1	1913	0
XM_020091854.1	coiled-coil domain-containing protein 85C isoform X3	3889	0
XM_020091855.1	fasciculation and elongation protein zeta-2-like isoform X1	4108	0
XM_020091856.1	fasciculation and elongation protein zeta-2-like isoform X2	4136	0
XM_020091857.1	fasciculation and elongation protein zeta-2-like isoform X3	4027	1.33E-171
XM_020091858.1	fasciculation and elongation protein zeta-2-like isoform X4	4055	2.51E-169
XM_020091859.1	tyrosine-protein kinase Fyn isoform X1	3474	0
XM_020091860.1	tyrosine-protein kinase Fyn isoform X1	3406	0
XM_020091861.1	tyrosine-protein kinase Fyn isoform X1	3316	0
XM_020091862.1	tyrosine-protein kinase Fyn isoform X1	3309	0
XM_020091863.1	tyrosine-protein kinase Fyn isoform X4	3152	0
XM_020091864.1	26S protease regulatory subunit 10B	1460	0
XM_020091865.1	baculoviral IAP repeat-containing protein 2-like isoform X1	1733	0
XM_020091866.1	1-acyl-sn-glycerol-3-phosphate acyltransferase epsilon	3334	0
XM_020091867.1	transcription factor SOX-7	2190	0
XM_020091868.1	PIN2/TERF1-interacting telomerase inhibitor 1	2055	0
XM_020091869.1	DNA repair protein RAD51 homolog 1 isoform X1	1981	0
XM_020091870.1	DNA repair protein RAD51 homolog 1 isoform X1	1359	0
XM_020091871.1	DNA repair protein RAD51 homolog 1 isoform X1	1359	0
XM_020091872.1	DNA repair protein RAD51 homolog 1 isoform X1	1889	0
XM_020091873.1	heat shock protein HSP 90-alpha	2897	0
XM_020091874.1	intersectin-2-like isoform X1	6686	0
XM_020091875.1	intersectin-2-like isoform X2	6641	0
XM_020091876.1	TGF-beta-activated kinase 1 and MAP3K7-binding protein 2 isoform X1	4729	0
XM_020091877.1	TGF-beta-activated kinase 1 and MAP3K7-binding protein 2 isoform X1	4573	0
XM_020091878.1	TGF-beta-activated kinase 1 and MAP3K7-binding protein 2 isoform X1	4715	0
XM_020091879.1	glutathione S-transferase A4-like	1067	2.16E-146
XM_020091880.1	MAM domain-containing glycosylphosphatidylinositol anchor protein 2	6945	0
XM_020091881.1	MAM domain-containing glycosylphosphatidylinositol anchor protein 2	7161	0
XM_020091882.1	low choriolytic enzyme-like	933	0
XM_020091883.1	serine palmitoyltransferase 2-like	3939	0
XM_020091884.1	protein dispatched homolog 1	5738	0
XM_020091885.1	protein dispatched homolog 1	5822	0
XM_020091886.1	protein dispatched homolog 1	5705	0
XM_020091887.1	vacuolar protein sorting-associated protein 18 homolog	3878	0
XM_020091888.1	DNA helicase MCM9 isoform X1	6104	0
XM_020091889.1	DNA helicase MCM9 isoform X1	6076	0
XM_020091890.1	DNA helicase MCM9 isoform X1	6042	0
XM_020091891.1	DNA helicase MCM9 isoform X1	6035	0
XM_020091892.1	DNA helicase MCM9 isoform X1	6032	0
XM_020091893.1	TBC1 domain family member 10B	2268	0
XM_020091894.1	transmembrane protein 14C-like	1004	6.33E-52
XM_020091895.1	DNA helicase MCM9 isoform X1	5857	0
XM_020091896.1	ATP-dependent RNA helicase DDX51 isoform X1	2742	0
XM_020091897.1	ATP-dependent RNA helicase DDX51 isoform X2	2581	0
XM_020091898.1	small integral membrane protein 8	501	1.36E-67
XM_020091899.1	serine incorporator 1	2449	0
XM_020091900.1	protein FAM49A isoform X1	3152	0
XM_020091901.1	protein FAM49A isoform X1	1316	0
XM_020091902.1	protein FAM49A isoform X1	1315	0
XM_020091903.1	chloride intracellular channel protein 5-like	3445	0

XM_020091904.1	protein sel-1 homolog 1	4305	0
XM_020091905.1	transcription initiation factor IIA subunit 1	2887	4.08E-108
XM_020091906.1	glycoprotein integral membrane protein 1	4394	0
XM_020091907.1	alpha-1,6-mannosyl-glycoprotein 2-beta-N-acetylglucosaminyltransferase	1990	0
XM_020091908.1	alpha-1,6-mannosyl-glycoprotein 2-beta-N-acetylglucosaminyltransferase	1912	0
XM_020091909.1	40S ribosomal protein S29	383	9.10E-37
XM_020091910.1	atypical kinase COQ8A, mitochondrial-like isoform X1	3849	0
XM_020091911.1	atypical kinase COQ8A, mitochondrial-like isoform X2	3815	0
XM_020091912.1	peroxisome proliferator-activated receptor gamma coactivator-related protein 1	4449	0
XM_020091913.1	integrator complex subunit 9	2369	0
XM_020091914.1	platelet-activating factor acetylhydrolase isoform X2	2171	0
XM_020091915.1	platelet-activating factor acetylhydrolase isoform X2	2024	0
XM_020091916.1	platelet-activating factor acetylhydrolase isoform X2	2072	0
XM_020091917.1	gap junction alpha-1 protein	4210	0
XM_020091918.1	gap junction alpha-1 protein	4161	0
XM_020091919.1	serine/threonine-protein kinase Sgk1 isoform X1	2685	0
XM_020091920.1	serine/threonine-protein kinase Sgk1 isoform X2	2534	0
XM_020091921.1	serine/threonine-protein kinase Sgk1 isoform X1	2493	0
XM_020091922.1	serine/threonine-protein kinase Sgk1 isoform X4	2460	0
XM_020091923.1	serine/threonine-protein kinase Sgk1 isoform X5	2561	0
XM_020091924.1	serine/threonine-protein kinase Sgk1 isoform X6	2468	0
XM_020091925.1	zinc finger protein 143	2437	0
XM_020091926.1	LIM domain-binding protein 1 isoform X2	1876	0
XM_020091927.1	ectonucleoside triphosphate diphosphohydrolase 5-like	2160	0
XM_020091928.1	sodium-dependent multivitamin transporter-like	2719	0
XM_020091929.1	sodium-dependent multivitamin transporter-like	2723	0

XM_020091930.1	EH domain-containing protein 3	4416	0
XM_020091931.1	methylmalonate-semialdehyde dehydrogenase [acylating], mitochondrial-like	2765	0
XM_020091932.1	CLOCK-interacting pacemaker-like	3818	0
XM_020091933.1	cytosolic carboxypeptidase-like protein 5 isoform X1	3866	0
XM_020091934.1	LIM domain-binding protein 1 isoform X2	1221	0
XM_020091935.1	cytosolic carboxypeptidase-like protein 5 isoform X1	3857	0
XM_020091936.1	cytosolic carboxypeptidase-like protein 5 isoform X1	3701	0
XM_020091937.1	cytosolic carboxypeptidase-like protein 5 isoform X1	3845	0
XM_020091938.1	centrosomal protein of 128 kDa isoform X1	6181	0
XM_020091939.1	centrosomal protein of 128 kDa isoform X2	6154	0
XM_020091940.1	mediator of RNA polymerase II transcription subunit 23	4694	0
XM_020091941.1	solute carrier family 25 member 45-like	3547	0
XM_020091942.1	DNA replication licensing factor MCM3	3109	0
XM_020091943.1	LIM domain-binding protein 1-like isoform X1	1597	0
XM_020091944.1	probable E3 ubiquitin-protein ligase RNF217 isoform X1	7676	0
XM_020091945.1	probable E3 ubiquitin-protein ligase RNF217 isoform X2	7606	0
XM_020091946.1	EMILIN-1-like isoform X1	4535	0
XM_020091947.1	EMILIN-1-like isoform X2	4517	0
XM_020091948.1	E3 UFM1-protein ligase 1	2812	0
XM_020091949.1	synaptosomal-associated protein 25	2406	4.65E-134
XM_020091950.1	LIM domain-binding protein 1-like isoform X4	2692	0
XM_020091951.1	26S protease regulatory subunit 6B isoform X2	1582	0
XM_020091952.1	heat shock factor protein 2	5084	0
XM_020091953.1	histone deacetylase 2	1739	0
XM_020091954.1	BRO1 domain-containing protein BROX	3155	0
XM_020091955.1	L-threonine 3-dehydrogenase, mitochondrial-like	1297	0
XM_020091956.1	L-threonine 3-dehydrogenase, mitochondrial-like	2229	0
XM_020091957.1	A-kinase anchor protein 7 isoform X1	5020	0
XM_020091958.1	A-kinase anchor protein 7 isoform X2	4982	0
XM_020091959.1	A-kinase anchor protein 7 isoform X3	2612	0
XM_020091960.1	probable G-protein coupled receptor 45	4707	0
XM_020091961.1	myotubularin-related protein 9	3999	0
XM_020091962.1	thyroid adenoma-associated protein isoform X1	6119	0
XM_020091963.1	clathrin coat assembly protein AP180-like isoform X1	4190	0
XM_020091964.1	clathrin coat assembly protein AP180-like isoform X1	4187	0
XM_020091965.1	clathrin coat assembly protein AP180-like isoform X1	4187	0
XM_020091966.1	clathrin coat assembly protein AP180-like isoform X1	4184	0
XM_020091967.1	clathrin coat assembly protein AP180-like isoform X1	4175	0
XM_020091968.1	clathrin coat assembly protein AP180-like isoform X1	4160	0
XM_020091969.1	clathrin coat assembly protein AP180-like isoform X6	4145	0
XM_020091970.1	clathrin coat assembly protein AP180-like isoform X7	4019	0
XM_020091971.1	clathrin coat assembly protein AP180-like isoform X8	3931	0
XM_020091972.1	clathrin coat assembly protein AP180-like isoform X9	3847	0
XM_020091973.1	thyroid adenoma-associated protein isoform X1	6341	0
XM_020091974.1	clathrin coat assembly protein AP180-like isoform X10	3832	0
XM_020091975.1	clathrin coat assembly protein AP180-like isoform X11	3802	0
XM_020091976.1	clathrin coat assembly protein AP180-like isoform X12	3781	0
XM_020091977.1	clathrin coat assembly protein AP180-like isoform X13	3760	0
XM_020091978.1	clathrin coat assembly protein AP180-like isoform X14	3751	0
XM_020091979.1	clathrin coat assembly protein AP180-like isoform X15	3676	0
XM_020091980.1	clathrin coat assembly protein AP180-like isoform X16	3661	0
XM_020091981.1	clathrin coat assembly protein AP180-like isoform X17	3610	0
XM_020091982.1	serine protease 23-like	1768	0

XM_020091983.1	thyroid adenoma-associated protein isoform X1	6113	0
XM_020091984.1	forkhead box protein N3	5172	0
XM_020091985.1	forkhead box protein N3	4997	0
XM_020091986.1	forkhead box protein N3	5178	0
XM_020091987.1	forkhead box protein N3	4922	0
XM_020091988.1	homeodomain-interacting protein kinase 2-like	1936	0
XM_020091989.1	protein FAM46A-like	4537	0
XM_020091990.1	protein FAM46A-like	4151	0
XM_020091991.1	SNW domain-containing protein 1	2160	0
XM_020091992.1	phosphoacetylglucosamine mutase	2416	0
XM_020091993.1	phosphoacetylglucosamine mutase	2440	0
XM_020091994.1	glial fibrillary acidic protein-like isoform X1	1918	0
XM_020091995.1	glial fibrillary acidic protein-like isoform X2	1915	0
XM_020091996.1	mRNA decay activator protein ZFP36L2 isoform X1	3593	0
XM_020091997.1	glial fibrillary acidic protein-like isoform X3	1722	0
XM_020091998.1	XK-related protein 6	1803	0
XM_020091999.1	cerebral cavernous malformations 2 protein	2721	0
XM_020092000.1	collagen alpha-1(X) chain	2669	2.55E-105
XM_020092001.1	5'-nucleotidase domain-containing protein 1	1669	0
XM_020092002.1	serine/threonine-protein kinase 35-like	2550	0
XM_020092003.1	serine/threonine-protein kinase 35-like	2546	0
XM_020092004.1	mRNA decay activator protein ZFP36L2 isoform X2	3592	0
XM_020092005.1	peptidyl-prolyl cis-trans isomerase FKBP1A-like	914	1.57E-148
XM_020092006.1	ubiquitin-like protein fubi and ribosomal protein S30	613	2.47E-83
XM_020092007.1	ubiquitin-like protein fubi and ribosomal protein S30	634	3.22E-83
XM_020092008.1	alpha-1-antitrypsin homolog	1763	0
XM_020092009.1	connective tissue growth factor isoform X1	1581	0
XM_020092010.1	connective tissue growth factor isoform X2	1468	0
XM_020092011.1	dehydrodolichyl diphosphate synthase complex subunit nus1-like	1282	1.40E-121

XM_020092012.1	cyclin-related protein FAM58A	2527	0
XM_020092013.1	eyes absent homolog 4 isoform X1	3117	0
XM_020092014.1	eyes absent homolog 4 isoform X2	3117	0
XM_020092015.1	eyes absent homolog 4 isoform X3	3033	0
XM_020092016.1	PREDICTED: formin-2-like	5903	0
XM_020092017.1	eyes absent homolog 4 isoform X1	3014	0
XM_020092018.1	eyes absent homolog 4 isoform X5	2955	0
XM_020092019.1	eyes absent homolog 4 isoform X6	2955	0
XM_020092020.1	mRNA decay activator protein ZFP36L1-like	3753	0
XM_020092021.1	alpha-2Db adrenergic receptor-like isoform X1	2275	0
XM_020092022.1	Golgi-associated PDZ and coiled-coil motif-containing protein isoform X1	2038	0
XM_020092023.1	Golgi-associated PDZ and coiled-coil motif-containing protein isoform X2	2014	0
XM_020092024.1	zinc-binding protein A33-like	2581	0
XM_020092025.1	60S ribosomal protein L7-like 1	1362	4.82E-165
XM_020092026.1	breast cancer metastasis-suppressor 1-like protein	3323	0
XM_020092027.1	deleted in malignant brain tumors 1 protein-like isoform X2	4305	0
XM_020092028.1	deleted in malignant brain tumors 1 protein-like isoform X2	4443	0
XM_020092029.1	deleted in malignant brain tumors 1 protein-like isoform X2	4392	0
XM_020092030.1	muscarinic acetylcholine receptor M3-like	4127	0
XM_020092031.1	rho-related GTP-binding protein RhoV-like	2266	1.88E-136
XM_020092032.1	high mobility group nucleosome-binding domain-containing protein 3 isoform X1	1307	6.43E-35
XM_020092033.1	high mobility group nucleosome-binding domain-containing protein 3 isoform X2	1201	4.96E-22
XM_020092034.1	muscarinic acetylcholine receptor M3-like	4234	0
XM_020092035.1	protein phosphatase 1 regulatory subunit 3A-like isoform X2	6474	0
XM_020092036.1	periodic tryptophan protein 1 homolog	2089	0
XM_020092037.1	glucoside xylosyltransferase 1-like	3146	0
XM_020092038.1	membrane-associated guanylate kinase, WW and PDZ domain-containing protein 2-like isoform X1	3206	0
XM_020092039.1	membrane-associated guanylate kinase, WW and PDZ domain-containing protein 2-like isoform X2	3201	0
XM_020092040.1	membrane-associated guanylate kinase, WW and PDZ domain-containing protein 2-like isoform X3	3183	0
XM_020092041.1	PREDICTED: gremlin-2	2226	1.00E-119
XM_020092042.1	membrane-associated guanylate kinase, WW and PDZ domain-containing protein 2-like isoform X4	2723	0
XM_020092043.1	membrane-associated guanylate kinase, WW and PDZ domain-containing protein 2-like isoform X1	2704	0
XM_020092044.1	membrane-associated guanylate kinase, WW and PDZ domain-containing protein 2-like isoform X6	3540	0
XM_020092045.1	membrane-associated guanylate kinase, WW and PDZ domain-containing protein 2-like isoform X7	2700	0
XM_020092046.1	PDZ domain-containing RING finger protein 4-like	4626	0
XM_020092047.1	calcium/calmodulin-dependent protein kinase type 1D	3658	0
XM_020092048.1	mRNA-decapping enzyme 1B-like	3728	0
XM_020092049.1	metabotropic glutamate receptor 8-like	2943	0
XM_020092050.1	aldose reductase-like	1719	0
XM_020092051.1	PREDICTED: plakophilin-2	3190	0
XM_020092052.1	contactin-2 isoform X1	5017	0
XM_020092053.1	ryanodine receptor 2-like	15152	0
XM_020092054.1	contactin-2 isoform X1	5005	0
XM_020092055.1	synaptophysin-like protein 1	4088	3.41E-165
XM_020092056.1	tetraspanin-11-like isoform X1	1001	2.42E-167
XM_020092057.1	tetraspanin-11-like isoform X1	1000	2.42E-167
XM_020092058.1	tetraspanin-11-like isoform X1	2198	6.80E-158
XM_020092059.1	tetraspanin-11-like isoform X1	1090	2.35E-155
XM_020092060.1	pseudouridylate synthase 7 homolog-like protein	2835	0
XM_020092061.1	pseudouridylate synthase 7 homolog-like protein	2874	0

XM_020092062.1	solute carrier organic anion transporter family member 1C1-like	3154	0
XM_020092063.1	solute carrier organic anion transporter family member 1C1-like	2883	0
XM_020092064.1	mitochondrial tRNA-specific 2-thiouridylase 1 isoform X1	1803	0
XM_020092065.1	mitochondrial tRNA-specific 2-thiouridylase 1 isoform X2	1886	0
XM_020092066.1	DNA mismatch repair protein Msh6 isoform X1	4390	0
XM_020092067.1	mitochondrial tRNA-specific 2-thiouridylase 1 isoform X3	1721	0
XM_020092068.1	serine dehydratase-like	1195	0
XM_020092069.1	leucine-rich repeat and transmembrane domain-containing protein 2	1445	3.09E-162
XM_020092070.1	leucine-rich repeats and immunoglobulin-like domains protein 3	2342	0
XM_020092071.1	ras and EF-hand domain-containing protein homolog	3551	0
XM_020092072.1	ras and EF-hand domain-containing protein homolog	3458	0
XM_020092073.1	protein arginine N-methyltransferase 8-B	1618	0
XM_020092074.1	DNA mismatch repair protein Msh6 isoform X2	4388	0
XM_020092075.1	troponin I, slow skeletal muscle-like	958	1.69E-122
XM_020092076.1	protein FAM19A5 isoform X1	2207	2.64E-75
XM_020092077.1	protein FAM19A5 isoform X2	2696	1.14E-78
XM_020092078.1	pyridine nucleotide-disulfide oxidoreductase domain-containing protein 1 isoform X1	2652	0
XM_020092079.1	pyridine nucleotide-disulfide oxidoreductase domain-containing protein 1 isoform X2	2754	0
XM_020092080.1	PREDICTED: sortilin-like	2986	0
XM_020092081.1	proline-rich protein 5-like	1865	0
XM_020092082.1	proline-rich protein 5-like	1879	0
XM_020092083.1	proline-rich protein 5-like	1764	0
XM_020092084.1	troponin T, fast skeletal muscle-like isoform X1	1355	0
XM_020092085.1	troponin T, fast skeletal muscle-like isoform X2	1038	1.31E-177
XM_020092086.1	F-box only protein 11 isoform X1	4202	0
XM_020092087.1	troponin T, fast skeletal muscle-like isoform X3	1474	1.46E-152
XM_020092088.1	troponin T, fast skeletal muscle-like isoform X3	1561	3.99E-152
XM_020092089.1	troponin T, fast skeletal muscle-like isoform X4	1000	6.05E-159
XM_020092090.1	phytanoyl-CoA dioxygenase, peroxisomal	2004	2.92E-180
XM_020092091.1	fibroblast growth factor 6-like	4261	5.84E-141
XM_020092092.1	ninjurin-2-like isoform X3	2648	6.65E-89
XM_020092093.1	ninjurin-2-like isoform X3	1579	3.99E-91

XM_020092094.1	F-box only protein 11 isoform X2	4199	0
XM_020092095.1	ninjurin-2-like isoform X3	1741	1.38E-103
XM_020092096.1	ninjurin-2-like isoform X3	801	4.46E-108
XM_020092097.1	transmembrane and TPR repeat-containing protein 3	4412	0
XM_020092098.1	proton myo-inositol cotransporter-like	5874	0
XM_020092099.1	tubby-related protein 3 isoform X1	1256	2.77E-95
XM_020092100.1	tubby-related protein 3 isoform X2	1250	2.07E-94
XM_020092101.1	tubby-related protein 3 isoform X3	928	1.32E-90
XM_020092102.1	tubby-related protein 3 isoform X4	1384	7.22E-86
XM_020092103.1	tetraspanin-8-like isoform X2	1310	5.79E-155
XM_020092104.1	tetraspanin-8-like isoform X2	1277	4.49E-172
XM_020092105.1	tetraspanin-8-like isoform X1	2564	0
XM_020092106.1	tetraspanin-8-like isoform X1	2561	0
XM_020092107.1	tetratricopeptide repeat protein 7A	4094	0
XM_020092108.1	PREDICTED: liprin-alpha-2-like	1179	0
XM_020092109.1	protein FAM180A-like	809	1.27E-125
XM_020092110.1	semaphorin-3F-like isoform X3	1626	0
XM_020092111.1	fatty acyl-CoA reductase 1-like isoform X1	646	1.82E-105
XM_020092112.1	G1/S-specific cyclin-D2-like	1311	0
XM_020092113.1	probable polypeptide N-acetylgalactosaminyltransferase 8	1807	0
XM_020092114.1	N-acetylglucosamine-1-phosphotransferase subunits alpha/beta-like	1036	0
XM_020092115.1	PREDICTED: tetraspanin-7-like	1089	0
XM_020092116.1	apoptotic protease-activating factor 1	3626	0
XM_020092117.1	alpha-(1,3)-fucosyltransferase 9-like	586	4.55E-148
XM_020092118.1	PREDICTED: uncharacterized protein C2orf61 homolog	788	6.80E-168
XM_020092119.1	DEP domain-containing protein 4	2531	0
XM_020092120.1	voltage-dependent calcium channel subunit alpha-2/delta-1-like	4594	0
XM_020092121.1	PREDICTED: semaphorin-3aa-like	4347	0
XM_020092122.1	ubiquitin-conjugating enzyme E2 H	795	6.17E-118
XM_020092123.1	voltage-dependent calcium channel subunit alpha-2/delta-4-like	4323	0
XM_020092124.1	tetraspanin-8-like isoform X1	1281	0
XM_020092125.1	kit ligand	828	2.03E-180
XM_020092126.1	macrophage mannose receptor 1-like	984	1.37E-179
XM_020092127.1	zinc finger protein 800-like	1621	0
XM_020092128.1	nicotinamide phosphoribosyltransferase	3037	0
XM_020092129.1	multiple coagulation factor deficiency protein 2 isoform X2	2000	3.12E-61
XM_020092130.1	forkhead box protein P2-like	636	3.99E-98
XM_020092131.1	dedicator of cytokinesis protein 4	5542	0
XM_020092132.1	striatin-interacting protein 2	2634	0
XM_020092133.1	achaete-scute homolog 4	519	2.71E-112
XM_020092134.1	suppressor APC domain-containing protein 1	1481	1.50E-127
XM_020092135.1	PREDICTED: mucin-2-like	6430	0
XM_020092136.1	transcription factor SOX-6-like isoform X2	4814	0
XM_020092137.1	lymphoid-restricted membrane protein-like	5731	0
XM_020092138.1	mitogen-activated protein kinase 14-like	4144	0
XM_020092139.1	mitogen-activated protein kinase 12-like	953	2.08E-178
XM_020092140.1	multiple coagulation factor deficiency protein 2 isoform X2	2105	5.00E-64

XM_020092141.1	centrosomal protein of 83 kDa	2088	0
XM_020092142.1	anoctamin-4 isoform X1	5256	0
XM_020092143.1	phosphatidylinositol 4,5-bisphosphate 3-kinase catalytic subunit gamma isoform	4433	0
XM_020092144.1	receptor-type tyrosine-protein phosphatase zeta-like	8522	0
XM_020092145.1	transcription factor 7-like 2	632	1.61E-109
XM_020092146.1	YEATS domain-containing protein 2 isoform X1	1073	5.71E-147
XM_020092147.1	copine-5-like isoform X2	1870	0
XM_020092148.1	multiple coagulation factor deficiency protein 2 isoform X2	2054	1.60E-60
XM_020092149.1	putative cation exchanger C521.04c isoform X1	2678	0
XM_020092150.1	low affinity vacuolar monovalent cation/H(+) antiporter-like isoform X2	2597	0
XM_020092151.1	putative cation exchanger C521.04c isoform X3	2596	0
XM_020092152.1	putative cation exchanger C521.04c isoform X4	2555	0
XM_020092153.1	src substrate cortactin-like	1949	0
XM_020092154.1	suppression of tumorigenicity 5 protein	4050	0
XM_020092155.1	transmembrane protein 53-A-like isoform X3	1743	0
XM_020092156.1	protein Wnt-7b-like	1126	0
XM_020092157.1	small subunit processome component 20 homolog isoform X1	9071	0
XM_020092158.1	small subunit processome component 20 homolog isoform X2	9068	0
XM_020092159.1	FYVE, RhoGEF and PH domain-containing protein 4-like isoform X1	3239	0
XM_020092160.1	troponin C, skeletal muscle	1896	7.33E-99
XM_020092161.1	FYVE, RhoGEF and PH domain-containing protein 4-like isoform X2	3190	0
XM_020092162.1	PREDICTED: uncharacterized protein LOC109632732	4733	0
XM_020092163.1	PREDICTED: uncharacterized protein LOC109632732	4740	0
XM_020092164.1	protein LCHN-like	3898	0
XM_020092165.1	ADP-ribosylation factor 3	1845	1.17E-115
XM_020092166.1	cyclin-dependent kinase inhibitor 1B	1758	2.07E-128
XM_020092167.1	leucine-rich repeat and guanylate kinase domain-containing protein isoform X1	3359	0
XM_020092168.1	leucine-rich repeat and guanylate kinase domain-containing protein isoform X2	3356	0
XM_020092169.1	leucine-rich repeat and guanylate kinase domain-containing protein isoform X3	3356	0
XM_020092170.1	bcl-2-like protein 13 isoform X1	1456	1.60E-50
XM_020092171.1	sodium-coupled monocarboxylate transporter 1-like	1870	0
XM_020092172.1	cGMP-dependent protein kinase 1 isoform X1	4876	0
XM_020092173.1	branched-chain-amino-acid aminotransferase, cytosolic-like	2237	0
XM_020092174.1	hepatocyte growth factor-like isoform X1	4744	0
XM_020092175.1	hepatocyte growth factor-like isoform X2	4726	0
XM_020092176.1	E3 ubiquitin-protein ligase TRIM39-like	2100	0
XM_020092177.1	probable G protein-coupled receptor 85	2373	0
XM_020092178.1	probable G protein-coupled receptor 85	2296	0
XM_020092179.1	39S ribosomal protein L42, mitochondrial isoform X1	604	4.71E-106
XM_020092180.1	cGMP-dependent protein kinase 1 isoform X2	4892	0
XM_020092181.1	39S ribosomal protein L42, mitochondrial isoform X2	540	2.55E-88
XM_020092182.1	unique cartilage matrix-associated protein	1190	7.33E-53
XM_020092183.1	coiled-coil domain-containing protein 3	775	3.29E-80
XM_020092184.1	paired box protein Pax-4-like	1508	0
XM_020092185.1	PREDICTED: uncharacterized protein LOC109632751	2128	0
XM_020092186.1	putative interleukin-17 receptor E-like	1977	0
XM_020092187.1	peroxisome proliferator-activated receptor alpha-like	794	1.21E-76
XM_020092188.1	golgin subfamily B member 1-like	10791	0
XM_020092189.1	adenosine deaminase CECR1	1529	0
XM_020092190.1	multiple inositol polyphosphate phosphatase 1-like isoform X1	1651	0
XM_020092191.1	RAD51-associated protein 1 isoform X1	1478	3.02E-141
XM_020092192.1	RAD51-associated protein 1 isoform X2	2211	6.89E-138
XM_020092193.1	V-type proton ATPase subunit E 1-like	1510	6.96E-128
XM_020092194.1	PREDICTED: leptin-B-like	622	9.94E-85
XM_020092195.1	multiple inositol polyphosphate phosphatase 1-like isoform X2	1060	0
XM_020092196.1	fibroblast growth factor 23	1083	8.87E-176
XM_020092197.1	islet amyloid polypeptide	563	4.82E-62
XM_020092198.1	F-box DNA helicase 1 isoform X1	3780	0
XM_020092199.1	F-box DNA helicase 1 isoform X2	4312	0
XM_020092200.1	splicing factor 45	1509	0
XM_020092201.1	splicing factor 45	1478	0
XM_020092202.1	cell division cycle protein 123 homolog	2007	0
XM_020092203.1	interleukin-15 receptor subunit alpha isoform X1	2394	3.47E-147
XM_020092204.1	type-2 angiotensin II receptor-like	2116	0
XM_020092205.1	phosphatidylcholine:ceramide cholinephosphotransferase 2	3874	0
XM_020092206.1	interleukin-15 receptor subunit alpha isoform X2	2283	7.95E-120
XM_020092207.1	ADP-sugar pyrophosphatase	867	1.86E-163
XM_020092208.1	ADP-sugar pyrophosphatase	1834	1.23E-158
XM_020092209.1	PREDICTED: uncharacterized protein LOC109632776 isoform X1	3863	0
XM_020092210.1	PREDICTED: uncharacterized protein LOC109632776 isoform X1	3714	0
XM_020092211.1	epiphycan isoform X1	2334	0
XM_020092212.1	epiphycan isoform X1	2139	0
XM_020092213.1	phosphatidylcholine:ceramide cholinephosphotransferase 2	1733	0
XM_020092214.1	epiphycan isoform X1	3003	0
XM_020092215.1	PREDICTED: decorin-like	2095	0
XM_020092216.1	PREDICTED: decorin-like	2252	0
XM_020092217.1	zinc finger CCCH domain-containing protein 4	1743	0
XM_020092218.1	PREDICTED: keratocan	1889	0
XM_020092219.1	PREDICTED: lumican	2459	0

XM_020092220.1	PREDICTED: uncharacterized protein LOC109632782	1935	0
XM_020092221.1	differentially expressed in FDCP 6 homolog	2310	0
XM_020092222.1	malonyl-CoA-acyl carrier protein transacylase, mitochondrial	1482	0
XM_020092223.1	DNA damage-regulated autophagy modulator protein 2	2015	2.51E-169
XM_020092224.1	WASH complex subunit CCDC53	2100	1.82E-118
XM_020092225.1	probable tubulin polyglutamylase TTLL1	2471	0
XM_020092226.1	probable tubulin polyglutamylase TTLL1	1853	0
XM_020092227.1	probable tubulin polyglutamylase TTLL1	2242	0
XM_020092228.1	protein Tob1-like	4814	3.76E-100
XM_020092229.1	kinesin-like protein KIF21A isoform X2	7926	0
XM_020092230.1	kinesin-like protein KIF21A isoform X2	7877	0
XM_020092231.1	ATP-binding cassette sub-family D member 2-like	6798	0
XM_020092232.1	serine/threonine-protein kinase WNK1-like	13987	0
XM_020092233.1	zinc finger BED domain-containing protein 4	5027	0
XM_020092234.1	dol-P-Man:Man(7)GlcNAc(2)-PP-Dol alpha-1,6-mannosyltransferase	1986	0
XM_020092235.1	cysteine-rich with EGF-like domain protein 2	1104	0
XM_020092236.1	leucine-rich repeat-containing protein 4-like	5162	0
XM_020092237.1	staphylococcal nuclease domain-containing protein 1	3792	0
XM_020092238.1	F-box/LRR-repeat protein 14	2611	0
XM_020092239.1	protein Wnt-5b	3399	0
XM_020092240.1	prickle-like protein 1	4040	0
XM_020092241.1	prickle-like protein 1	3963	0
XM_020092242.1	prickle-like protein 1	3959	0
XM_020092243.1	periphilin-1-like isoform X1	1845	0
XM_020092244.1	periphilin-1-like isoform X1	1849	0
XM_020092245.1	periphilin-1-like isoform X1	1838	0
XM_020092246.1	periphilin-1-like isoform X1	1839	0
XM_020092247.1	periphilin-1-like isoform X1	1809	0
XM_020092248.1	periphilin-1-like isoform X4	1803	0
XM_020092249.1	periphilin-1-like isoform X5	1722	0
XM_020092250.1	periphilin-1-like isoform X6	1716	0
XM_020092251.1	zinc finger CCHC-type and RNA-binding motif-containing protein 1	1709	3.09E-123
XM_020092252.1	proline-rich transmembrane protein 4	4939	0
XM_020092253.1	inosine-5'-monophosphate dehydrogenase 1b isoform X1	3876	0
XM_020092254.1	inosine-5'-monophosphate dehydrogenase 1b isoform X2	3873	0
XM_020092255.1	V-type immunoglobulin domain-containing suppressor of T-cell activation	1947	0
XM_020092256.1	inosine-5'-monophosphate dehydrogenase 1b isoform X3	3792	0
XM_020092257.1	ethanolamine kinase 1	3926	0
XM_020092258.1	poly [ADP-ribose] polymerase 12-like isoform X1	4013	0
XM_020092259.1	poly [ADP-ribose] polymerase 12-like isoform X2	3977	0
XM_020092260.1	transcription factor ETV6 isoform X1	2196	0
XM_020092261.1	transcription factor ETV6 isoform X2	2193	0
XM_020092262.1	transcription factor ETV6 isoform X3	2311	0
XM_020092263.1	transcription factor ETV6 isoform X1	2065	0
XM_020092264.1	protein FAM19A2-like isoform X1	2073	1.89E-81
XM_020092265.1	protein FAM19A2-like isoform X2	2070	1.32E-80
XM_020092266.1	neuronal cell adhesion molecule-like isoform X1	8608	0
XM_020092267.1	phospholipid phosphatase 5-like	4219	0
XM_020092268.1	neuronal cell adhesion molecule-like isoform X2	8725	0
XM_020092269.1	neuronal cell adhesion molecule-like isoform X3	8719	0
XM_020092270.1	neuronal cell adhesion molecule-like isoform X4	8707	0
XM_020092271.1	neuronal cell adhesion molecule-like isoform X5	8689	0
XM_020092272.1	neuronal cell adhesion molecule-like isoform X6	8672	0
XM_020092273.1	neuronal cell adhesion molecule-like isoform X7	8658	0
XM_020092274.1	neuronal cell adhesion molecule-like isoform X2	4051	0
XM_020092275.1	neuronal cell adhesion molecule-like isoform X9	3670	0
XM_020092276.1	neuronal cell adhesion molecule-like isoform X10	8314	0
XM_020092277.1	neuronal cell adhesion molecule-like isoform X11	8706	0
XM_020092278.1	neuronal cell adhesion molecule-like isoform X12	8609	0
XM_020092279.1	neuronal cell adhesion molecule-like isoform X13	8294	0
XM_020092280.1	sodium-coupled monocarboxylate transporter 1-like	2234	0
XM_020092281.1	PREDICTED: uncharacterized protein C10orf105-like	1444	3.50E-70
XM_020092282.1	LYR motif-containing protein 5A isoform X2	841	2.53E-68
XM_020092283.1	LYR motif-containing protein 5A isoform X2	792	2.93E-61
XM_020092284.1	LYR motif-containing protein 5A isoform X2	790	2.17E-60
XM_020092285.1	protein CASC1	2340	0
XM_020092286.1	cyclin-dependent kinase 17	4218	0
XM_020092287.1	ETS domain-containing protein Elk-3-like	4046	0
XM_020092288.1	GRAM domain-containing protein 4 isoform X1	6797	0
XM_020092289.1	GRAM domain-containing protein 4 isoform X2	6734	0
XM_020092290.1	GRAM domain-containing protein 4 isoform X3	6716	0
XM_020092291.1	GRAM domain-containing protein 4 isoform X4	6145	0
XM_020092292.1	GRAM domain-containing protein 4 isoform X4	6200	0
XM_020092293.1	tyrosine-protein phosphatase non-receptor type 12-like isoform X1	5691	0
XM_020092294.1	tyrosine-protein phosphatase non-receptor type 12-like isoform X2	2871	0
XM_020092295.1	eukaryotic translation initiation factor 1	919	1.51E-65
XM_020092296.1	tyrosine-protein phosphatase non-receptor type 12-like isoform X3	5854	0
XM_020092297.1	cleavage and polyadenylation specificity factor subunit 6 isoform X2	2548	1.51E-125
XM_020092298.1	cleavage and polyadenylation specificity factor subunit 6 isoform X2	2545	1.47E-125

XM_020092299.1	cleavage and polyadenylation specificity factor subunit 6 isoform X3	2494	1.03E-116
XM_020092300.1	cleavage and polyadenylation specificity factor subunit 6 isoform X4	2440	2.02E-114
XM_020092301.1	calcium-independent phospholipase A2-gamma-like isoform X2	3114	0
XM_020092302.1	calcium-independent phospholipase A2-gamma-like isoform X2	2481	0
XM_020092303.1	calcium-independent phospholipase A2-gamma-like isoform X2	2291	0
XM_020092304.1	THAP domain-containing protein 5 isoform X1	1790	0
XM_020092305.1	THAP domain-containing protein 5 isoform X2	1790	0
XM_020092306.1	dnaJ homolog subfamily B member 9-like	1864	2.49E-118
XM_020092307.1	A-kinase anchor protein 14	1259	2.12E-143
XM_020092308.1	SEC23-interacting protein isoform X1	3672	0
XM_020092309.1	forkhead box protein M1	3688	0
XM_020092310.1	forkhead box protein M1	3790	0
XM_020092311.1	cystine/glutamate transporter-like	2359	0
XM_020092312.1	RAD9, HUS1, RAD1-interacting nuclear orphan protein 1	1860	0
XM_020092313.1	3-oxo-5-beta-steroid 4-dehydrogenase	1224	0
XM_020092314.1	ubiquitin carboxyl-terminal hydrolase 15-like	3867	2.66E-121
XM_020092315.1	ubiquitin-conjugating enzyme E2 N	2657	1.64E-103
XM_020092316.1	SEC23-interacting protein isoform X2	3669	0
XM_020092317.1	plexin-A4 isoform X1	6514	0
XM_020092318.1	plexin-A4 isoform X1	6271	0
XM_020092319.1	plexin-A4 isoform X1	4937	0
XM_020092320.1	leucine-rich repeat neuronal protein 1-like	4370	0
XM_020092321.1	non-muscle caldesmon-like isoform X1	3652	3.11E-103
XM_020092322.1	non-muscle caldesmon-like isoform X1	3638	7.55E-103
XM_020092323.1	non-muscle caldesmon-like isoform X1	3635	3.68E-103
XM_020092324.1	non-muscle caldesmon-like isoform X1	3541	5.17E-103
XM_020092325.1	non-muscle caldesmon-like isoform X1	3508	7.37E-103
XM_020092326.1	PREDICTED: synapsin-3	7031	0
XM_020092327.1	FERM and PDZ domain-containing protein 3	6408	0
XM_020092328.1	mini-chromosome maintenance complex-binding protein	2133	0
XM_020092329.1	metalloproteinase inhibitor 4	1941	0
XM_020092330.1	putative homeodomain transcription factor 2 isoform X1	4639	0
XM_020092331.1	putative homeodomain transcription factor 2 isoform X2	4597	0
XM_020092332.1	putative homeodomain transcription factor 2 isoform X3	4448	0
XM_020092333.1	transmembrane protein 60-like	1913	2.20E-75
XM_020092334.1	transmembrane protein 60-like	1919	4.98E-76
XM_020092335.1	transmembrane protein 60-like	2053	2.23E-75
XM_020092336.1	dynamin-1-like protein isoform X1	3026	0
XM_020092337.1	mini-chromosome maintenance complex-binding protein	2096	0
XM_020092338.1	dynamin-1-like protein isoform X2	2861	0
XM_020092339.1	PREDICTED: uncharacterized protein C7orf73 homolog isoform X1	943	8.12E-56
XM_020092340.1	PREDICTED: uncharacterized protein C7orf73 homolog isoform X1	730	3.82E-28
XM_020092341.1	small lysine-rich protein 1	444	1.40E-22
XM_020092342.1	leucine-rich repeat-containing protein 17-like	2180	0
XM_020092343.1	ras-related protein Rab-19-like	3548	3.32E-157
XM_020092344.1	muscarinic acetylcholine receptor M2	3807	0
XM_020092345.1	sodium- and chloride-dependent GABA transporter 2-like	2697	0
XM_020092346.1	poly [ADP-ribose] polymerase 11 isoform X1	1979	0
XM_020092347.1	poly [ADP-ribose] polymerase 11 isoform X1	1859	0
XM_020092348.1	poly [ADP-ribose] polymerase 11 isoform X1	1971	0
XM_020092349.1	chondroitin sulfate N-acetylgalactosaminyltransferase 2	3419	0
XM_020092350.1	coatomer subunit gamma-2	2969	0
XM_020092351.1	mesoderm-specific transcript homolog protein	1681	0
XM_020092352.1	mesoderm-specific transcript homolog protein	1493	0
XM_020092353.1	mesoderm-specific transcript homolog protein	1688	0
XM_020092354.1	protein kinase C and casein kinase substrate in neurons protein 2 isoform X1	3397	0
XM_020092355.1	protein kinase C and casein kinase substrate in neurons protein 2 isoform X2	3349	0
XM_020092356.1	protein kinase C and casein kinase substrate in neurons protein 2 isoform X3	3256	0
XM_020092357.1	protein kinase C and casein kinase substrate in neurons protein 2 isoform X4	3208	0
XM_020092358.1	chondroitin sulfate N-acetylgalactosaminyltransferase 2	3359	0
XM_020092359.1	diphosphoinositol polyphosphate phosphohydrolase 3-beta-like	3885	6.03E-83
XM_020092360.1	high affinity choline transporter 1-like	4744	0
XM_020092361.1	PREDICTED: uncharacterized protein LOC109632858 isoform X1	4622	0
XM_020092362.1	high affinity choline transporter 1-like	4648	0
XM_020092363.1	protein asunder homolog	2894	0
XM_020092364.1	protein asunder homolog	2903	0
XM_020092365.1	protein asunder homolog	2882	0
XM_020092366.1	protein asunder homolog	2877	0
XM_020092367.1	high affinity choline transporter 1-like	1829	0
XM_020092368.1	chondroitin sulfate N-acetylgalactosaminyltransferase 2	3302	0
XM_020092369.1	exocyst complex component 4	5596	0
XM_020092370.1	voltage-dependent L-type calcium channel subunit alpha-1C-like	7494	0
XM_020092371.1	PREDICTED: plexin-C1	4717	0
XM_020092372.1	ras-related and estrogen-regulated growth inhibitor	2202	1.41E-130
XM_020092373.1	ELKS/Rab6-interacting/CAST family member 1-like isoform X1	6334	0
XM_020092374.1	ELKS/Rab6-interacting/CAST family member 1-like isoform X2	6241	0
XM_020092375.1	ELKS/Rab6-interacting/CAST family member 1-like isoform X3	6223	0
XM_020092376.1	ELKS/Rab6-interacting/CAST family member 1-like isoform X4	6211	0
XM_020092377.1	ELKS/Rab6-interacting/CAST family member 1-like isoform X5	6130	0

XM_020092378.1	ELKS/Rab6-interacting/CAST family member 1-like isoform X6	6118	0
XM_020092379.1	atlastin-1 isoform X2	3565	0
XM_020092380.1	cadherin EGF LAG seven-pass G-type receptor 1 isoform X1	11152	0
XM_020092381.1	cadherin EGF LAG seven-pass G-type receptor 1 isoform X2	11071	0
XM_020092382.1	ankyrin repeat and sterile alpha motif domain-containing protein 1B isoform X1	5229	0
XM_020092383.1	ankyrin repeat and sterile alpha motif domain-containing protein 1B isoform X2	5226	0
XM_020092384.1	ankyrin repeat and sterile alpha motif domain-containing protein 1B isoform X3	6343	0
XM_020092385.1	ankyrin repeat and sterile alpha motif domain-containing protein 1B isoform X4	1617	0
XM_020092386.1	TBC1 domain family member 22A isoform X1	5462	0
XM_020092387.1	TBC1 domain family member 22A isoform X2	5399	0
XM_020092388.1	TBC1 domain family member 22A isoform X3	5139	0
XM_020092389.1	ceramide kinase-like	3867	0
XM_020092390.1	RNA polymerase II-associated protein 3	2203	0
XM_020092391.1	RNA polymerase II-associated protein 3	2262	0
XM_020092392.1	equilibrative nucleoside transporter 1	2918	0
XM_020092393.1	mitochondrial inner membrane protease ATP23 homolog	1291	1.28E-174
XM_020092394.1	IQ motif and SEC7 domain-containing protein 3	5745	0
XM_020092395.1	sodium- and chloride-dependent GABA transporter 1-like isoform X1	2048	0
XM_020092396.1	transcription intermediary factor 1-alpha-like isoform X1	4665	0
XM_020092397.1	transcription intermediary factor 1-alpha-like isoform X2	4663	0
XM_020092398.1	transcription intermediary factor 1-alpha-like isoform X3	4644	0
XM_020092399.1	transcription factor EC isoform X1	2276	0
XM_020092400.1	equilibrative nucleoside transporter 1	2857	0
XM_020092401.1	transcription factor EC isoform X2	2200	0
XM_020092402.1	transcription factor EC isoform X3	2178	0
XM_020092403.1	transcription factor EC isoform X4	2201	0
XM_020092404.1	aldose reductase-like	1680	0
XM_020092405.1	SCY1-like protein 2	4801	0
XM_020092406.1	serine/threonine-protein kinase 36 isoform X1	5052	0
XM_020092407.1	collagen alpha-1(VII) chain-like	9485	0
XM_020092408.1	6-phosphofructo-2-kinase/fructose-2,6-bisphosphatase 3 isoform X1	2650	0
XM_020092409.1	equilibrative nucleoside transporter 1	2831	0
XM_020092410.1	6-phosphofructo-2-kinase/fructose-2,6-bisphosphatase 3 isoform X2	2673	0
XM_020092411.1	6-phosphofructo-2-kinase/fructose-2,6-bisphosphatase 3 isoform X3	2417	0
XM_020092412.1	neurotrophin 3	1909	1.58E-177
XM_020092413.1	protein piccolo-like	15610	0
XM_020092414.1	alpha-N-acetylneuraminide alpha-2,8-sialyltransferase	5579	0
XM_020092415.1	zinc finger protein interacting with ribonucleoprotein K-like isoform X2	2182	0
XM_020092416.1	equilibrative nucleoside transporter 1	2871	0
XM_020092417.1	zinc finger protein interacting with ribonucleoprotein K-like isoform X2	2127	0
XM_020092418.1	zinc finger protein interacting with ribonucleoprotein K-like isoform X1	2168	0
XM_020092419.1	tetraspanin-33 isoform X1	2508	0
XM_020092420.1	tetraspanin-33 isoform X2	2348	0
XM_020092421.1	transmembrane protein 168-A-like	3315	0
XM_020092422.1	adiponectin receptor protein 1	1701	0
XM_020092423.1	MORN repeat-containing protein 2	1319	1.75E-90
XM_020092424.1	MORN repeat-containing protein 2	1506	1.11E-89
XM_020092425.1	MORN repeat-containing protein 2	1502	1.07E-89
XM_020092426.1	MORN repeat-containing protein 2	1333	1.97E-90
XM_020092427.1	MORN repeat-containing protein 2	1385	1.63E-90
XM_020092428.1	ADP-ribosylation factor GTPase-activating protein 3 isoform X1	1804	0
XM_020092429.1	ADP-ribosylation factor GTPase-activating protein 3 isoform X2	4516	0
XM_020092430.1	ADP-ribosylation factor GTPase-activating protein 3 isoform X3	1778	0
XM_020092431.1	ADP-ribosylation factor GTPase-activating protein 3 isoform X4	1637	0
XM_020092432.1	plasma membrane calcium-transporting ATPase 1 isoform X1	3995	0
XM_020092433.1	plasma membrane calcium-transporting ATPase 1 isoform X1	3384	0
XM_020092434.1	sorting nexin-5	2542	0
XM_020092435.1	zinc finger protein 135-like	3406	0
XM_020092436.1	ubiquitin carboxyl-terminal hydrolase 48	2294	0
XM_020092437.1	peroxisome assembly protein 26	1719	0
XM_020092438.1	protein LLP homolog	929	4.91E-68
XM_020092439.1	protein LLP homolog	748	6.50E-69
XM_020092440.1	serine/threonine-protein kinase pim-3	2483	0
XM_020092441.1	methyltransferase-like protein 25	2544	0
XM_020092442.1	thyroid transcription factor 1-associated protein 26 isoform X2	1322	0
XM_020092443.1	thyroid transcription factor 1-associated protein 26 isoform X2	1193	7.11E-151
XM_020092444.1	thyroid transcription factor 1-associated protein 26 isoform X2	1246	0
XM_020092445.1	forkhead box protein P2-like isoform X1	2324	0
XM_020092446.1	mitochondrial genome maintenance exonuclease 1 isoform X1	2377	0
XM_020092447.1	forkhead box protein P2-like isoform X2	2307	0
XM_020092448.1	forkhead box protein P2-like isoform X3	2280	0
XM_020092449.1	monocarboxylate transporter 2	5087	0
XM_020092450.1	cyclin-dependent kinase inhibitor 1-like	2855	3.17E-108
XM_020092451.1	coiled-coil domain-containing protein 146	3225	0
XM_020092452.1	mucin-5AC-like isoform X2	2262	0
XM_020092453.1	mucin-5AC-like isoform X2	2695	0
XM_020092454.1	N-acyl-phosphatidylethanolamine-hydrolyzing phospholipase D isoform X2	3042	0
XM_020092455.1	tenomodulin isoform X1	1402	0
XM_020092456.1	mitochondrial genome maintenance exonuclease 1 isoform X1	2266	0

XM_020092457.1	N-acyl-phosphatidylethanolamine-hydrolyzing phospholipase D isoform X2	1512	0
XM_020092458.1	N-acyl-phosphatidylethanolamine-hydrolyzing phospholipase D isoform X2	1364	0
XM_020092459.1	round spermatid basic protein 1-like	6718	0
XM_020092460.1	protein phosphatase 1H-like	5553	0
XM_020092461.1	YY1-associated factor 2-like	3966	1.14E-73
XM_020092462.1	MICOS complex subunit MIC19 isoform X1	2392	0
XM_020092463.1	MICOS complex subunit MIC19 isoform X1	2169	0
XM_020092464.1	mitochondrial genome maintenance exonuclease 1 isoform X1	2330	0
XM_020092465.1	MICOS complex subunit MIC19 isoform X2	2353	0
XM_020092466.1	MICOS complex subunit MIC19 isoform X2	2131	0
XM_020092467.1	MICOS complex subunit MIC19 isoform X1	1923	2.31E-132
XM_020092468.1	kinase D-interacting substrate of 220 kDa-like	1917	1.88E-76
XM_020092469.1	putative Dol-P-Glc:Glc(2)Man(9)GlcNAc(2)-PP-Dol alpha-1,2-glucosyltransferase	3133	0
XM_020092470.1	EKC/KEOPS complex subunit TPRKB	736	6.61E-115
XM_020092471.1	putative ATP-dependent RNA helicase DHX57	5677	0
XM_020092472.1	mitochondrial genome maintenance exonuclease 1 isoform X1	2417	0
XM_020092473.1	myoD family inhibitor domain-containing protein isoform X1	3520	5.69E-136
XM_020092474.1	myoD family inhibitor domain-containing protein isoform X2	1281	2.27E-124
XM_020092475.1	ras association domain-containing protein 8-like	4497	0
XM_020092476.1	gamma-secretase-activating protein	4620	0
XM_020092477.1	putative adenosylhomocysteinase 3	2926	0
XM_020092478.1	anoctamin-5 isoform X2	4372	0
XM_020092479.1	mitochondrial genome maintenance exonuclease 1 isoform X1	2278	0
XM_020092480.1	nuclear respiratory factor 1	2675	0
XM_020092481.1	cytoskeleton-associated protein 4	2001	0
XM_020092482.1	smoothened homolog	3848	0
XM_020092483.1	ataxin-7-like protein 1	6514	0
XM_020092484.1	ataxin-7-like protein 1	6318	0
XM_020092485.1	fibulin-1-like isoform X1	2778	0
XM_020092486.1	fibulin-1-like isoform X2	2095	0
XM_020092487.1	nuclear-interacting partner of ALK	2384	0
XM_020092488.1	T-complex protein 11 homolog isoform X2	3112	0
XM_020092489.1	glycine N-acyltransferase-like protein 3 isoform X1	1926	0
XM_020092490.1	PREDICTED: ataxin-10	1694	0
XM_020092491.1	interferon-related developmental regulator 1	2059	0
XM_020092492.1	sugar phosphate exchanger 3	3360	0
XM_020092493.1	PR domain zinc finger protein 4	3531	0
XM_020092494.1	PR domain zinc finger protein 4	3519	0
XM_020092495.1	PR domain zinc finger protein 4	3502	0
XM_020092496.1	PR domain zinc finger protein 4	3514	0
XM_020092497.1	PR domain zinc finger protein 4	3334	0
XM_020092498.1	glycine N-acyltransferase-like protein 3 isoform X2	1827	1.23E-177
XM_020092499.1	NF-kappa-B essential modulator isoform X3	2554	0
XM_020092500.1	protein FAM3A	1458	2.50E-153
XM_020092501.1	solute carrier organic anion transporter family member 1C1-like	2291	0
XM_020092502.1	solute carrier organic anion transporter family member 1C1-like	3899	0
XM_020092503.1	inhibitor of nuclear factor kappa-B kinase-interacting protein	1032	0
XM_020092504.1	transmembrane protein 209	2493	0
XM_020092505.1	transmembrane protein 209	2409	0
XM_020092506.1	fructose-2,6-bisphosphatase TIGAR B-like isoform X1	1643	9.93E-169
XM_020092507.1	fructose-2,6-bisphosphatase TIGAR B-like isoform X2	1522	4.32E-129
XM_020092508.1	kelch domain-containing protein 10	2480	0
XM_020092509.1	host cell factor 1 isoform X1	2421	0
XM_020092510.1	cystatin C	771	1.78E-77
XM_020092511.1	HMG box-containing protein 1-like isoform X1	4802	0
XM_020092512.1	HMG box-containing protein 1-like isoform X2	4799	0
XM_020092513.1	HMG box-containing protein 1-like isoform X3	4285	0
XM_020092514.1	HMG box-containing protein 1-like isoform X4	4564	0
XM_020092515.1	HMG box-containing protein 1-like isoform X4	4340	0
XM_020092516.1	HMG box-containing protein 1-like isoform X2	4337	0
XM_020092517.1	zinc finger protein 135-like	5055	0
XM_020092518.1	zinc finger protein 135-like	4992	0
XM_020092519.1	protein MCM10 homolog	3152	0
XM_020092520.1	RRP12-like protein	4231	0
XM_020092521.1	ubiquitin carboxyl-terminal hydrolase FAM188A	2536	0
XM_020092522.1	farnesyl pyrophosphate synthase isoform X1	1859	0
XM_020092523.1	farnesyl pyrophosphate synthase isoform X1	1899	0
XM_020092524.1	farnesyl pyrophosphate synthase isoform X1	1904	0
XM_020092525.1	tenomodulin isoform X2	1399	0
XM_020092526.1	anthrax toxin receptor 1-like	3702	0
XM_020092527.1	farnesyl pyrophosphate synthase isoform X1	1749	0
XM_020092528.1	tumor necrosis factor alpha-induced protein 8-like protein 2	2062	7.70E-131
XM_020092529.1	FAD synthase	2166	0
XM_020092530.1	FAD synthase	2175	0
XM_020092531.1	FAD synthase	2084	0
XM_020092532.1	PREDICTED: stathmin-2-like	2061	5.41E-105
XM_020092533.1	rhomboid-related protein 2	2146	0
XM_020092534.1	rhomboid-related protein 2	2064	0
XM_020092535.1	TPA: 14 kDa-apolipoprotein	871	1.29E-102

XM_020092536.1	neuronal acetylcholine receptor subunit beta-2-like isoform X2	4220	0
XM_020092537.1	E3 ubiquitin-protein ligase CBL-like isoform X1	2638	0
XM_020092538.1	E3 ubiquitin-protein ligase CBL-like isoform X2	2605	0
XM_020092539.1	B-cell lymphoma 3 protein	2103	0
XM_020092540.1	transcription elongation factor A protein 3 isoform X1	2794	0
XM_020092541.1	transcription elongation factor A protein 3 isoform X2	2791	0
XM_020092542.1	transcription elongation factor A protein 3 isoform X3	2656	0
XM_020092543.1	PR domain zinc finger protein 8-like isoform X1	2665	0
XM_020092544.1	transcription elongation factor A protein 3 isoform X4	1999	0
XM_020092545.1	transcription elongation factor A protein 3 isoform X5	1818	0
XM_020092546.1	transcription elongation factor A protein 3 isoform X6	1758	0
XM_020092547.1	transcription elongation factor A protein 3 isoform X7	1746	0
XM_020092548.1	transcription elongation factor A protein 3 isoform X8	1686	0
XM_020092549.1	transcription elongation factor A protein 3 isoform X9	1548	0
XM_020092550.1	ephrin-A1-like isoform X2	1921	4.39E-150
XM_020092551.1	mitochondrial import receptor subunit TOM40B-like	1527	0
XM_020092552.1	PR domain zinc finger protein 8-like isoform X1	2601	0
XM_020092553.1	tuftelin-like isoform X1	2321	0
XM_020092554.1	integrin alpha-5-like	4416	0
XM_020092555.1	SH3 domain-binding protein 4	4202	0
XM_020092556.1	protein S100-A1-like	830	6.04E-60
XM_020092557.1	protein FAM110A-like	2674	0
XM_020092558.1	dynactin subunit 3	1243	3.41E-134
XM_020092559.1	juxtaposed with another zinc finger protein 1-like	2214	1.44E-124
XM_020092560.1	PR domain zinc finger protein 8-like isoform X1	2652	0
XM_020092561.1	DENN domain-containing protein 3 isoform X1	4143	0
XM_020092562.1	DENN domain-containing protein 3 isoform X2	4137	0
XM_020092563.1	ribonuclease ZC3H12A-like	3058	0
XM_020092564.1	dehydrodolichyl diphosphate synthase complex subunit DHDDS	1646	0
XM_020092565.1	dehydrodolichyl diphosphate synthase complex subunit DHDDS	1661	0
XM_020092566.1	RNA-binding protein 12B-A-like	2230	0
XM_020092567.1	acetylcholinesterase collagenic tail peptide-like isoform X1	3061	0
XM_020092568.1	acetylcholinesterase collagenic tail peptide-like isoform X2	3046	0
XM_020092569.1	PR domain zinc finger protein 8-like isoform X1	2331	0
XM_020092570.1	acetylcholinesterase collagenic tail peptide-like isoform X3	2947	0
XM_020092571.1	protein BTG3-like	1889	0
XM_020092572.1	homeobox protein Hox-A10	1874	0
XM_020092573.1	homeobox protein Hox-A10	1558	1.56E-173
XM_020092574.1	protein naked cuticle homolog 2 isoform X1	2312	0
XM_020092575.1	protein naked cuticle homolog 2 isoform X2	2309	0
XM_020092576.1	ephrin-A4-like isoform X1	2865	6.29E-123
XM_020092577.1	ephrin-A4-like isoform X2	2838	1.51E-118
XM_020092578.1	tropomodulin-1 isoform X1	1565	0
XM_020092579.1	homeobox protein Hox-A11b-like	2838	0
XM_020092580.1	protein OSCP1	2644	0
XM_020092581.1	rho GTPase-activating protein 22-like	3613	0
XM_020092582.1	protein FAM189B	3941	0
XM_020092583.1	protein eva-1 homolog B-like	967	2.93E-86
XM_020092584.1	probable U3 small nucleolar RNA-associated protein 11	1110	1.48E-168
XM_020092585.1	sodium/potassium-transporting ATPase subunit gamma-like isoform X1	1265	2.77E-46
XM_020092586.1	sodium/potassium-transporting ATPase subunit gamma-like isoform X1	1276	3.43E-46
XM_020092587.1	FXYD domain-containing ion transport regulator 6-like isoform X2	1235	1.94E-26
XM_020092588.1	FXYD domain-containing ion transport regulator 6-like isoform X2	1241	2.09E-26
XM_020092589.1	macrophage mannose receptor 1-like isoform X1	2521	0
XM_020092590.1	macrophage mannose receptor 1-like isoform X1	2620	0
XM_020092591.1	granulocyte colony-stimulating factor receptor	3100	0
XM_020092592.1	mitogen-activated protein kinase 8-like isoform X1	3517	0
XM_020092593.1	interleukin-6 receptor subunit alpha-like	2768	0
XM_020092594.1	forkhead box protein O6-like	4657	0
XM_020092595.1	PREDICTED: uncharacterized protein LOC109633012	1298	0
XM_020092596.1	PREDICTED: uncharacterized protein LOC109633013	2559	0
XM_020092597.1	PREDICTED: uncharacterized protein LOC109633014 isoform X1	1251	0
XM_020092598.1	PREDICTED: uncharacterized protein LOC109633014 isoform X1	1249	0
XM_020092599.1	chemokine-like receptor 1	2614	0
XM_020092600.1	mitogen-activated protein kinase 8-like isoform X1	3517	0
XM_020092601.1	sodium channel subunit beta-1-like	3680	1.52E-143
XM_020092602.1	ETS translocation variant 3-like protein isoform X1	2668	0
XM_020092603.1	ETS translocation variant 3-like protein isoform X1	2576	0
XM_020092604.1	ETS translocation variant 3-like protein isoform X1	2903	0
XM_020092605.1	FXYD domain-containing ion transport regulator 6-like isoform X2	1870	1.63E-129
XM_020092606.1	zinc finger protein 696-like	10737	0
XM_020092607.1	sodium/potassium-transporting ATPase subunit alpha-3 isoform X1	3084	0
XM_020092608.1	mitogen-activated protein kinase 8-like isoform X1	3563	0
XM_020092609.1	cyclic AMP-responsive element-binding protein 5-like isoform X2	997	0
XM_020092610.1	probable serine carboxypeptidase CPVL	586	1.32E-73
XM_020092611.1	solute carrier family 40 member 1-like	1731	0
XM_020092612.1	sodium-dependent neutral amino acid transporter B(0)AT3-like	1848	0
XM_020092613.1	sodium-dependent neutral amino acid transporter B(0)AT1-like	2859	0
XM_020092614.1	tenascin-like isoform X3	3015	0

XM_020092615.1	mitogen-activated protein kinase 8-like isoform X1	3421	0
XM_020092616.1	NF-kappa-B inhibitor delta	1368	0
XM_020092617.1	FXYD domain-containing ion transport regulator 6-like isoform X2	461	1.85E-62
XM_020092618.1	kin of IRRE-like protein 1	7361	0
XM_020092619.1	suppressor of tumorigenicity 14 protein homolog	3967	0
XM_020092620.1	zinc finger and BTB domain-containing protein 16-A-like	2429	0
XM_020092621.1	serine/threonine-protein kinase Sgk1-like	2433	0
XM_020092622.1	mitogen-activated protein kinase 8-like isoform X1	3527	0
XM_020092623.1	DNA repair and recombination protein RAD54B-like	1846	0
XM_020092624.1	nuclear receptor subfamily 4 group A member 3	1199	0
XM_020092625.1	G-protein coupled receptor 20-like	1104	0
XM_020092626.1	rano class II histocompatibility antigen, A beta chain-like	791	1.46E-154
XM_020092627.1	multidrug resistance protein 1	3074	0
XM_020092628.1	disintegrin and metalloproteinase domain-containing protein 22	5423	0
XM_020092629.1	acyl-coenzyme A thioesterase THEM4	861	3.43E-148
XM_020092630.1	serum amyloid P-component-like	642	1.29E-159
XM_020092631.1	cingulin-like protein 1	3490	0
XM_020092632.1	small conductance calcium-activated potassium channel protein 2-like	5042	0
XM_020092633.1	tyrosine-protein phosphatase non-receptor type 13-like	9153	0
XM_020092634.1	PREDICTED: glucosylceramidase, partial	1621	0
XM_020092635.1	homeodomain-interacting protein kinase 3-like	985	0
XM_020092636.1	meiotic recombination protein REC8 homolog	1901	0
XM_020092637.1	cytochrome P450 11B, mitochondrial-like	2523	0
XM_020092638.1	pyruvate kinase PKM-like isoform X1	1181	0
XM_020092639.1	potassium/sodium hyperpolarization-activated cyclic nucleotide-gated channel 3-like	5224	0
XM_020092640.1	NADH dehydrogenase [ubiquinone] flavoprotein 1, mitochondrial-like	1511	0
XM_020092641.1	TCF3 fusion partner	820	1.33E-89
XM_020092642.1	serine/threonine-protein kinase LMTK3	9469	0
XM_020092643.1	protocadherin beta-4-like	1212	0
XM_020092644.1	annexin A11-like isoform X1	2752	0
XM_020092645.1	RNA binding protein fox-1 homolog 1-like	1451	0
XM_020092646.1	protein kinase C gamma type-like	3427	0
XM_020092647.1	single-stranded DNA cytosine deaminase	813	2.40E-157
XM_020092648.1	intermediate filament family orphan 1	2024	0
XM_020092649.1	CD27 antigen	2081	0
XM_020092650.1	CD27 antigen	667	4.59E-126
XM_020092651.1	protein turtle homolog B-like	1429	0
XM_020092652.1	annexin A11-like isoform X2	2605	0
XM_020092653.1	tapasin-related protein	1889	0
XM_020092654.1	complement C1r subcomponent-like isoform X1	4348	0
XM_020092655.1	arginine-glutamic acid dipeptide repeats protein-like isoform X2	5772	0
XM_020092656.1	calsyntenin-1 isoform X4	2874	0
XM_020092657.1	PREDICTED: uncharacterized protein LOC109633066	1598	0
XM_020092658.1	protein capicua homolog	6873	0
XM_020092659.1	carcinoembryonic antigen-related cell adhesion molecule 5-like	2215	0
XM_020092660.1	carcinoembryonic antigen-related cell adhesion molecule 5-like	4062	0
XM_020092661.1	trypsinogen 1	1691	0
XM_020092662.1	zinc finger CCHC domain-containing protein 24-like isoform X1	5882	1.54E-139
XM_020092663.1	meprin A subunit beta-like	1793	0
XM_020092664.1	apolipoprotein Eb-like	2500	0
XM_020092665.1	forkhead box protein O6-like	4126	0
XM_020092666.1	glutamate receptor ionotropic, kainate 3	2756	0
XM_020092667.1	mitogen-activated protein kinase kinase kinase 5-like	4332	0
XM_020092668.1	gap junction alpha-9 protein-like	1491	0
XM_020092669.1	zinc finger CCHC domain-containing protein 24-like isoform X2	3104	1.98E-131
XM_020092670.1	PREDICTED: uncharacterized protein LOC109633078	1773	0
XM_020092671.1	myelin-associated glycoprotein-like	5360	0
XM_020092672.1	B-cell receptor CD22-like	2857	0
XM_020092673.1	B-cell receptor CD22-like	2220	0
XM_020092674.1	transmembrane protease serine 6-like	1431	0
XM_020092675.1	myelin-associated glycoprotein-like isoform X1	1821	0
XM_020092676.1	free fatty acid receptor 2-like	915	0
XM_020092677.1	free fatty acid receptor 2-like	942	0
XM_020092678.1	igLON family member 5-like	1069	3.60E-136
XM_020092679.1	soluble guanylate cyclase 88E-like isoform X1	3027	0
XM_020092680.1	zinc finger protein 668-like isoform X2	9410	0
XM_020092681.1	transcription factor HIVEP3-like isoform X1	6840	0
XM_020092682.1	peptidyl-prolyl cis-trans isomerase F, mitochondrial-like	730	2.55E-104
XM_020092683.1	transcription factor HIVEP3-like isoform X1	6671	0
XM_020092684.1	transcription factor HIVEP3-like isoform X1	6635	0
XM_020092685.1	receptor-type tyrosine-protein phosphatase U isoform X1	7073	0
XM_020092686.1	receptor-type tyrosine-protein phosphatase U isoform X2	7049	0
XM_020092687.1	receptor-type tyrosine-protein phosphatase U isoform X3	4404	0
XM_020092688.1	zinc finger protein 728-like isoform X1	4877	0
XM_020092689.1	zinc finger protein 728-like isoform X1	5082	0
XM_020092690.1	putative uncharacterized zinc finger protein 814	3657	0
XM_020092691.1	zinc finger protein 157-like	3250	0
XM_020092692.1	discoidin, CUB and LCCL domain-containing protein 1-like isoform X1	2899	0
XM_020092693.1	discoidin, CUB and LCCL domain-containing protein 1-like isoform X2	2896	0

XM_020092694.1	protein eyes shut homolog	9406	0
XM_020092695.1	zinc finger protein 251-like isoform X1	2898	0
XM_020092696.1	zinc finger protein 251-like isoform X2	2827	0
XM_020092697.1	PREDICTED: uncharacterized protein LOC109633098	2602	0
XM_020092698.1	glucocorticoid modulatory element-binding protein 2-like	3023	0
XM_020092699.1	glucocorticoid modulatory element-binding protein 2-like	2649	0
XM_020092700.1	serine incorporator 2-like	1605	0
XM_020092701.1	zinc finger protein 696-like	2490	0
XM_020092702.1	zinc finger protein 696-like	2804	0
XM_020092703.1	protein eyes shut homolog	9493	0
XM_020092704.1	membrane progestin receptor alpha	3766	0
XM_020092705.1	trypsinogen 2 precursor	822	2.96E-167
XM_020092706.1	PREDICTED: stathmin-like	899	3.79E-72
XM_020092707.1	histone-lysine N-methyltransferase 2B isoform X1	13896	0
XM_020092708.1	histone-lysine N-methyltransferase 2B isoform X2	13872	0
XM_020092709.1	histone-lysine N-methyltransferase 2B isoform X3	13734	0
XM_020092710.1	amyloid-like protein 1 isoform X1	2369	0
XM_020092711.1	amyloid-like protein 1 isoform X2	2366	0
XM_020092712.1	proline and serine-rich protein 3 isoform X1	2482	0
XM_020092713.1	proline and serine-rich protein 3 isoform X1	2361	0
XM_020092714.1	proline and serine-rich protein 3 isoform X1	2566	0
XM_020092715.1	proline and serine-rich protein 3 isoform X1	2191	0
XM_020092716.1	PHD finger protein 3 isoform X3	6037	0
XM_020092717.1	heat shock cognate 71 kDa protein-like	2450	0
XM_020092718.1	TBC1 domain family member 15-like	3260	0
XM_020092719.1	IGF-like family receptor 1 isoform X1	1123	0
XM_020092720.1	IGF-like family receptor 1 isoform X2	1110	0
XM_020092721.1	chymotrypsin-like protease CTRL-1	1496	0
XM_020092722.1	protein lin-37 homolog	1411	5.84E-178
XM_020092723.1	heat shock protein beta-6 isoform X1	971	4.67E-104
XM_020092724.1	PHD finger protein 3 isoform X3	6083	0
XM_020092725.1	heat shock protein beta-6 isoform X1	862	2.17E-104
XM_020092726.1	heat shock protein beta-6 isoform X1	726	1.41E-104
XM_020092727.1	gamma-secretase subunit PEN-2	891	2.82E-68
XM_020092728.1	gamma-secretase subunit PEN-2	888	3.82E-68
XM_020092729.1	gamma-secretase subunit PEN-2	909	7.68E-68
XM_020092730.1	sugar transporter SWEET1	988	3.14E-157
XM_020092731.1	2-hydroxyacyl-CoA lyase 1 isoform X2	1962	0
XM_020092732.1	2-hydroxyacyl-CoA lyase 1 isoform X2	2016	0
XM_020092733.1	PHD finger protein 3 isoform X3	6045	0
XM_020092734.1	PREDICTED: meckelin	3157	0
XM_020092735.1	protein S100-A1-like	401	6.73E-79
XM_020092736.1	protein S100-A1-like	522	1.68E-78
XM_020092737.1	protein S100-A1-like	604	2.18E-77
XM_020092738.1	protein S100-A1-like	522	5.48E-78
XM_020092739.1	V-set and immunoglobulin domain-containing protein 10-like	2604	0
XM_020092740.1	hairy/enhancer-of-split related with YRPW motif protein 1-like	1499	3.35E-123
XM_020092741.1	glycylpeptide N-tetradecanoyltransferase 2	2238	0
XM_020092742.1	PHD finger protein 3 isoform X3	5932	0
XM_020092743.1	twist-related protein 2-like	1007	1.13E-84
XM_020092744.1	28S ribosomal protein S15, mitochondrial	1081	7.63E-165
XM_020092745.1	INO80 complex subunit C	1263	2.27E-93
XM_020092746.1	probable assembly chaperone of rpl4	1237	0
XM_020092747.1	myomesin-1 isoform X6	5042	0
XM_020092748.1	protein THEMIS2	3264	0
XM_020092749.1	protein THEMIS2	3145	0
XM_020092750.1	protein THEMIS2	3226	0
XM_020092751.1	cytochrome P450 4B1	3226	0
XM_020092752.1	acid sphingomyelinase-like phosphodiesterase 3b isoform X1	1462	0
XM_020092753.1	acid sphingomyelinase-like phosphodiesterase 3b isoform X2	1508	0
XM_020092754.1	isoaspartyl peptidase/L-asparaginase	1697	0
XM_020092755.1	quinone oxidoreductase PIG3	1549	0
XM_020092756.1	nuclear inhibitor of protein phosphatase 1 isoform X2	1541	0
XM_020092757.1	nuclear inhibitor of protein phosphatase 1 isoform X2	1505	0
XM_020092758.1	nuclear inhibitor of protein phosphatase 1 isoform X2	1617	0
XM_020092759.1	replication protein A 32 kDa subunit isoform X1	1192	0
XM_020092760.1	replication protein A 32 kDa subunit isoform X2	1343	0
XM_020092761.1	protein lin-28 homolog A isoform X1	4544	3.29E-119
XM_020092762.1	protein lin-28 homolog A isoform X2	4656	5.16E-124
XM_020092763.1	protocadherin beta-3-like	3228	0
XM_020092764.1	mediator of RNA polymerase II transcription subunit 18	1140	5.21E-144
XM_020092765.1	SH2 domain-containing adapter protein E-like	1853	0
XM_020092766.1	ephrin-A3-like isoform X2	1554	2.99E-142
XM_020092767.1	ankyrin-3-like isoform X1	8876	0
XM_020092768.1	sodium channel modifier 1 isoform X1	1306	2.69E-177
XM_020092769.1	sodium channel modifier 1 isoform X2	1288	1.91E-172
XM_020092770.1	delta-type opioid receptor-like	3100	0
XM_020092771.1	hepcidin 2	619	7.46E-45
XM_020092772.1	protein S100-A1-like isoform X2	761	3.53E-74

XM_020092773.1	mutS protein homolog 5	2783	0
XM_020092774.1	serum amyloid P-component-like	1636	0
XM_020092775.1	ankyrin-3-like isoform X2	8813	0
XM_020092776.1	RIIa domain-containing protein 1	1232	1.49E-59
XM_020092777.1	hydroxyacylglutathione hydrolase-like protein	1072	3.67E-118
XM_020092778.1	transcriptional regulator Erg-like isoform X1	1620	0
XM_020092779.1	transcriptional regulator Erg-like isoform X2	1617	0
XM_020092780.1	transcriptional regulator Erg-like isoform X3	1587	0
XM_020092781.1	PREDICTED: uncharacterized protein LOC109633150 isoform X1	1087	1.31E-151
XM_020092782.1	PREDICTED: uncharacterized protein LOC109633150 isoform X2	1054	5.74E-160
XM_020092783.1	PREDICTED: uncharacterized protein LOC109633150 isoform X3	1039	1.77E-139
XM_020092784.1	PREDICTED: uncharacterized protein LOC109633150 isoform X4	1006	8.92E-148
XM_020092785.1	ankyrin-3-like isoform X3	8780	0
XM_020092786.1	TYRO protein tyrosine kinase-binding protein	987	6.35E-75
XM_020092787.1	lysM and putative peptidoglycan-binding domain-containing protein 1	867	2.77E-114
XM_020092788.1	myelin-associated glycoprotein-like isoform X1	2535	0
XM_020092789.1	myelin-associated glycoprotein-like isoform X1	2531	0
XM_020092790.1	myelin-associated glycoprotein-like isoform X1	2532	0
XM_020092791.1	dual 3',5'-cyclic-AMP and -GMP phosphodiesterase 11A-like	3305	0
XM_020092792.1	apolipoprotein C-I	629	1.19E-41
XM_020092793.1	ankyrin-3-like isoform X4	8753	0
XM_020092794.1	homeobox protein Hox-A9b-like	1319	0
XM_020092795.1	D(2) dopamine receptor A-like	2471	0
XM_020092796.1	R-spondin 1	2235	3.22E-163
XM_020092797.1	R-spondin 1	2361	1.16E-162
XM_020092798.1	R-spondin 1	2358	1.12E-162
XM_020092799.1	steroid 21-hydroxylase-like isoform X1	1918	0
XM_020092800.1	ankyrin-3-like isoform X5	8750	0
XM_020092801.1	steroid 21-hydroxylase-like isoform X2	1807	0
XM_020092802.1	homeobox protein Hox-A13b	1831	0
XM_020092803.1	free fatty acid receptor 2	2098	0
XM_020092804.1	free fatty acid receptor 2	2095	0
XM_020092805.1	peptidyl-prolyl cis-trans isomerase FKBP14	1019	1.65E-146
XM_020092806.1	PREDICTED: uncharacterized protein LOC109633165	1332	0
XM_020092807.1	peptide YY-like	695	8.36E-69
XM_020092808.1	meprin A subunit beta-like	2231	0
XM_020092809.1	GTP-binding protein RAD	1911	0
XM_020092810.1	transmembrane protein 200B-like	1685	9.24E-132
XM_020092811.1	apoptosis-associated speck-like protein containing a CARD isoform X2	1226	5.63E-178
XM_020092812.1	transmembrane 9 superfamily member 3	3619	0
XM_020092813.1	protein S100-G-like	631	2.34E-54
XM_020092814.1	trypsinogen-like protein 3	1702	5.83E-175
XM_020092815.1	protein S100-G-like	585	1.88E-54
XM_020092816.1	sperm acrosome membrane-associated protein 6 isoform X1	1261	0
XM_020092817.1	sperm acrosome membrane-associated protein 6 isoform X2	1238	0
XM_020092818.1	hyaluronan synthase 1-like	1807	0
XM_020092819.1	G patch domain-containing protein 8-like	5014	0
XM_020092820.1	transcriptional repressor scratch 1-like	1680	0
XM_020092821.1	chemokine-like receptor 1 isoform X1	1507	0
XM_020092822.1	chemokine-like receptor 1 isoform X1	1465	0
XM_020092823.1	monoacylglycerol lipase ABHD12-like	1821	0
XM_020092824.1	lens fiber membrane intrinsic protein-like	702	6.33E-125
XM_020092825.1	myelin-associated glycoprotein-like isoform X1	2121	0
XM_020092826.1	myelin-associated glycoprotein-like isoform X1	1999	1.92E-180
XM_020092827.1	probable palmitoyltransferase ZDHHC1	1651	0
XM_020092828.1	fatty acid-binding protein 10-A, liver basic-like	581	2.12E-89
XM_020092829.1	E3 ubiquitin-protein ligase NHLRC1	2004	0
XM_020092830.1	adenosine receptor A1-like	1690	0
XM_020092831.1	metalloreductase STEAP2	1446	0

XM_020092832.1	thioredoxin-dependent peroxide reductase, mitochondrial	929	2.86E-174
XM_020092833.1	prostate-associated microseminoprotein-like	672	2.09E-79
XM_020092834.1	prostate-associated microseminoprotein-like	629	7.88E-80
XM_020092835.1	interferon-inducible GTPase 5-like	1140	0
XM_020092836.1	PREDICTED: uncharacterized protein C7orf31 homolog isoform X1	1578	0
XM_020092837.1	PREDICTED: uncharacterized protein C7orf31 homolog isoform X1	1249	0
XM_020092838.1	cannabinoid receptor 2-like	1556	0
XM_020092839.1	lens epithelial cell protein LEP503	754	5.92E-79
XM_020092840.1	PREDICTED: uncharacterized protein LOC109633199	1224	0
XM_020092841.1	calcium/calmodulin-dependent protein kinase type II subunit gamma isoform X1	5388	0
XM_020092842.1	FH1/FH2 domain-containing protein 3-like	7635	0
XM_020092843.1	rap guanine nucleotide exchange factor 5-like isoform X1	3576	0
XM_020092844.1	rap guanine nucleotide exchange factor 5-like isoform X2	3752	0
XM_020092845.1	rap guanine nucleotide exchange factor 5-like isoform X1	3534	0
XM_020092846.1	rap guanine nucleotide exchange factor 5-like isoform X4	3375	0
XM_020092847.1	retinoic acid receptor RXR-beta-A-like isoform X1	3130	0
XM_020092848.1	retinoic acid receptor RXR-beta-A-like isoform X1	1771	0
XM_020092849.1	retinoic acid receptor RXR-beta-A-like isoform X1	1796	0
XM_020092850.1	retinoic acid receptor RXR-beta-A-like isoform X3	1756	0
XM_020092851.1	calcium/calmodulin-dependent protein kinase type II subunit gamma isoform X2	5379	0

XM_020092852.1	retinoic acid receptor RXR-beta-A-like isoform X1	1740	0
XM_020092853.1	SPRY domain-containing SOCS box protein 1	930	1.42E-176
XM_020092854.1	testis development-related protein-like	734	6.22E-81
XM_020092855.1	anionic trypsin-1-like	1075	0
XM_020092856.1	trypsin-like isoform X1	989	0
XM_020092857.1	trypsin-like isoform X2	967	5.73E-176
XM_020092858.1	trypsinogen 2 precursor	1055	0
XM_020092859.1	calcium/calmodulin-dependent protein kinase type II subunit gamma isoform X1	5343	0
XM_020092860.1	39S ribosomal protein L17, mitochondrial	671	7.57E-120
XM_020092861.1	protein argonaute-1 isoform X1	4812	0
XM_020092862.1	protein argonaute-1 isoform X2	4778	0
XM_020092863.1	protein argonaute-3 isoform X1	8936	0
XM_020092864.1	protein argonaute-3 isoform X2	8915	0
XM_020092865.1	histone-lysine N-methyltransferase SETD2 isoform X1	12001	0
XM_020092866.1	histone-lysine N-methyltransferase SETD2 isoform X1	11832	0
XM_020092867.1	histone-lysine N-methyltransferase SETD2 isoform X1	12123	0
XM_020092868.1	calcium/calmodulin-dependent protein kinase type II subunit gamma isoform X2	5334	0
XM_020092869.1	RNA-binding protein 12B-A-like	2498	0
XM_020092870.1	RNA-binding protein 12B-A-like	2397	0
XM_020092871.1	RNA-binding protein 12B-A-like	2591	0
XM_020092872.1	uridine phosphorylase 1-like isoform X1	1578	0
XM_020092873.1	uridine phosphorylase 1-like isoform X1	1438	0
XM_020092874.1	PREDICTED: uncharacterized protein C8orf88 homolog	922	2.23E-91
XM_020092875.1	PREDICTED: uncharacterized protein C8orf88 homolog	885	2.51E-91
XM_020092876.1	attractin-like protein 1	10751	0
XM_020092877.1	calcium/calmodulin-dependent protein kinase type II subunit gamma isoform X5	5328	0
XM_020092878.1	mediator of DNA damage checkpoint protein 1	7757	0
XM_020092879.1	mediator of DNA damage checkpoint protein 1	7729	0
XM_020092880.1	mediator of DNA damage checkpoint protein 1	7747	0
XM_020092881.1	myosin-10-like isoform X1	6415	0
XM_020092882.1	probable carboxypeptidase X1	2674	0
XM_020092883.1	myosin-10-like isoform X1	9385	0
XM_020092884.1	myosin-10-like isoform X1	9596	0
XM_020092885.1	myosin-10-like isoform X1	9361	0
XM_020092886.1	myosin-10-like isoform X1	9560	0
XM_020092887.1	myosin-10-like isoform X5	9548	0
XM_020092888.1	calcium/calmodulin-dependent protein kinase type II subunit gamma isoform X6	5307	0
XM_020092889.1	myosin-10-like isoform X6	9479	0
XM_020092890.1	rho guanine nucleotide exchange factor 5-like isoform X3	6534	0
XM_020092891.1	rho guanine nucleotide exchange factor 5-like isoform X3	5835	0
XM_020092892.1	rho guanine nucleotide exchange factor 5-like isoform X3	3860	0
XM_020092893.1	rho guanine nucleotide exchange factor 5-like isoform X3	3361	0
XM_020092894.1	zinc finger protein 865-like isoform X1	7226	0
XM_020092895.1	zinc finger protein 865-like isoform X1	6982	0
XM_020092896.1	zinc finger protein 865-like isoform X1	5126	0
XM_020092897.1	condensin complex subunit 1	4844	0
XM_020092898.1	calcium/calmodulin-dependent protein kinase type II subunit gamma isoform X7	5295	0
XM_020092899.1	chromodomain-helicase-DNA-binding protein 4-like	5051	0
XM_020092900.1	valine--tRNA ligase, mitochondrial	4065	0
XM_020092901.1	histone-lysine N-methyltransferase SETDB1-B-like isoform X2	4261	0
XM_020092902.1	histone-lysine N-methyltransferase SETDB1-B-like isoform X2	4085	0
XM_020092903.1	histone-lysine N-methyltransferase SETDB1-B-like isoform X2	4166	0
XM_020092904.1	histone-lysine N-methyltransferase SETDB1-B-like isoform X2	4185	0
XM_020092905.1	histone-lysine N-methyltransferase SETDB1-B-like isoform X2	4037	0
XM_020092906.1	histone-lysine N-methyltransferase SETDB1-B-like isoform X2	4243	0
XM_020092907.1	calcium/calmodulin-dependent protein kinase type II subunit gamma isoform X8	5280	0
XM_020092908.1	thrombospondin type-1 domain-containing protein 4-like isoform X1	6102	0
XM_020092909.1	thrombospondin type-1 domain-containing protein 4-like isoform X1	6099	0
XM_020092910.1	glutamate receptor ionotropic, kainate 5-like isoform X1	5938	0
XM_020092911.1	glutamate receptor ionotropic, kainate 5-like isoform X2	5893	0
XM_020092912.1	pleckstrin homology domain-containing family G member 6	3557	0
XM_020092913.1	ephrin type-B receptor 5-like isoform X1	4314	0
XM_020092914.1	ephrin type-B receptor 5-like isoform X2	3030	0
XM_020092915.1	zinc finger protein 585A-like isoform X1	3497	0
XM_020092916.1	calcium/calmodulin-dependent protein kinase type II subunit gamma isoform X9	5271	0
XM_020092917.1	zinc finger protein 585A-like isoform X2	3494	0
XM_020092918.1	sodium/potassium-transporting ATPase subunit alpha-3 isoform X1	3611	0
XM_020092919.1	sodium/potassium-transporting ATPase subunit alpha-3 isoform X1	3652	0
XM_020092920.1	sodium/potassium-transporting ATPase subunit alpha-3 isoform X2	3572	0
XM_020092921.1	sodium/potassium-transporting ATPase subunit alpha-3 isoform X2	3613	0
XM_020092922.1	epithelial discoidin domain-containing receptor 1 isoform X1	5309	0
XM_020092923.1	epithelial discoidin domain-containing receptor 1 isoform X2	5267	0
XM_020092924.1	epithelial discoidin domain-containing receptor 1 isoform X3	5252	0
XM_020092925.1	epithelial discoidin domain-containing receptor 1 isoform X4	5189	0
XM_020092926.1	calcium/calmodulin-dependent protein kinase type II delta chain isoform X10	5262	0
XM_020092927.1	ras-interacting protein 1	3858	0
XM_020092928.1	C-Jun-amino-terminal kinase-interacting protein 4-like isoform X1	4371	0
XM_020092929.1	C-Jun-amino-terminal kinase-interacting protein 4-like isoform X2	4368	0
XM_020092930.1	UBX domain-containing protein 1	1569	1.92E-176

XM_020092931.1	chloride channel protein 1	4628	0
XM_020092932.1	toll-like receptor 13	3705	0
XM_020092933.1	calsyntenin-1 isoform X2	4237	0
XM_020092934.1	CCR4-NOT transcription complex subunit 3 isoform X1	3743	0
XM_020092935.1	calcium/calmodulin-dependent protein kinase type II subunit gamma isoform X11	5253	0
XM_020092936.1	CCR4-NOT transcription complex subunit 3 isoform X2	3735	0
XM_020092937.1	CCR4-NOT transcription complex subunit 3 isoform X3	3735	0
XM_020092938.1	CCR4-NOT transcription complex subunit 3 isoform X4	3727	0
XM_020092939.1	zinc finger protein 236-like	4254	0
XM_020092940.1	zinc finger protein 236-like	3544	0
XM_020092941.1	sphingosine kinase 2-like	5370	0
XM_020092942.1	sphingosine kinase 2-like	5273	0
XM_020092943.1	ubiquitin carboxyl-terminal hydrolase 5 isoform X1	2830	0
XM_020092944.1	calcium/calmodulin-dependent protein kinase type II delta chain isoform X12	5247	0
XM_020092945.1	ubiquitin carboxyl-terminal hydrolase 5 isoform X2	2829	0
XM_020092946.1	ubiquitin carboxyl-terminal hydrolase 5 isoform X3	2772	0
XM_020092947.1	ubiquitin carboxyl-terminal hydrolase 5 isoform X4	2607	0
XM_020092948.1	myocardin-like isoform X1	5433	0
XM_020092949.1	myocardin-like isoform X2	5430	0
XM_020092950.1	myocardin-like isoform X3	5430	0
XM_020092951.1	myocardin-like isoform X4	5397	0
XM_020092952.1	myocardin-like isoform X5	5292	0
XM_020092953.1	leukocyte receptor cluster member 8	5473	0
XM_020092954.1	calcium/calmodulin-dependent protein kinase type II subunit delta isoform X13	5235	0
XM_020092955.1	polyhomeotic-like protein 1	3381	0
XM_020092956.1	kell blood group glycoprotein-like	2617	0
XM_020092957.1	aryl hydrocarbon receptor nuclear translocator-like isoform X1	4950	0
XM_020092958.1	aryl hydrocarbon receptor nuclear translocator-like isoform X2	4527	0
XM_020092959.1	aryl hydrocarbon receptor nuclear translocator-like isoform X3	4826	0
XM_020092960.1	putative methyltransferase NSUN6 isoform X1	2756	0
XM_020092961.1	prolyl 3-hydroxylase 1 isoform X1	2544	0
XM_020092962.1	calcium/calmodulin-dependent protein kinase type II subunit gamma isoform X8	5235	0
XM_020092963.1	POU domain, class 2, transcription factor 2-like	4867	0
XM_020092964.1	ETS domain-containing transcription factor ERF-like isoform X1	3618	0
XM_020092965.1	ETS domain-containing transcription factor ERF-like isoform X2	3603	0
XM_020092966.1	transient receptor potential cation channel subfamily V member 6-like	2277	0
XM_020092967.1	protein shisa-7-like	2590	0
XM_020092968.1	transmembrane channel-like protein 4	2361	0
XM_020092969.1	brain-specific angiogenesis inhibitor 1-associated protein 2-like isoform X1	4386	0
XM_020092970.1	brain-specific angiogenesis inhibitor 1-associated protein 2-like isoform X1	4383	0
XM_020092971.1	calcium/calmodulin-dependent protein kinase type II subunit gamma isoform X9	5226	0
XM_020092972.1	brain-specific angiogenesis inhibitor 1-associated protein 2-like isoform X1	4895	0
XM_020092973.1	PREDICTED: uncharacterized protein LOC109633254 isoform X1	2565	0
XM_020092974.1	PREDICTED: uncharacterized protein LOC109633254 isoform X1	2736	0
XM_020092975.1	PREDICTED: uncharacterized protein LOC109633254 isoform X1	2588	0
XM_020092976.1	PREDICTED: uncharacterized protein LOC109633254 isoform X3	2179	0
XM_020092977.1	PREDICTED: uncharacterized protein LOC109633254 isoform X4	2153	0
XM_020092978.1	epsin-1-like isoform X1	3811	0
XM_020092979.1	epsin-1-like isoform X1	2397	0
XM_020092980.1	epsin-1-like isoform X1	2169	0
XM_020092981.1	calcium/calmodulin-dependent protein kinase type II subunit gamma isoform X16	5220	0
XM_020092982.1	peroxisomal biogenesis factor 5 isoform X1	3217	0
XM_020092983.1	peroxisomal biogenesis factor 5 isoform X2	3093	0
XM_020092984.1	lipoma-preferred partner homolog	2957	0
XM_020092985.1	lipoma-preferred partner homolog	2948	0
XM_020092986.1	lipoma-preferred partner homolog	2746	0
XM_020092987.1	lipoma-preferred partner homolog	2704	0
XM_020092988.1	PREDICTED: uncharacterized protein LOC109633259	4474	0
XM_020092989.1	forkhead box protein J2-like isoform X1	2850	0
XM_020092990.1	calcium/calmodulin-dependent protein kinase type II subunit gamma isoform X17	5211	0
XM_020092991.1	forkhead box protein J2-like isoform X2	2829	0
XM_020092992.1	forkhead box protein J2-like isoform X3	2826	0
XM_020092993.1	PREDICTED: uncharacterized protein LOC109633261 isoform X1	2457	0
XM_020092994.1	PREDICTED: uncharacterized protein LOC109633261 isoform X2	2380	0
XM_020092995.1	zinc finger protein 2-like	2549	0
XM_020092996.1	guanine nucleotide-binding protein-like 1	4311	0
XM_020092997.1	UPF0469 protein KIAA0907 homolog isoform X1	2446	0
XM_020092998.1	UPF0469 protein KIAA0907 homolog isoform X2	1930	0
XM_020092999.1	calcium/calmodulin-dependent protein kinase type II delta chain isoform X18	5202	0
XM_020093000.1	UPF0469 protein KIAA0907 homolog isoform X3	1824	0
XM_020093001.1	protein tweety homolog 1 isoform X1	3119	0
XM_020093002.1	protein tweety homolog 1 isoform X2	2951	0
XM_020093003.1	protein tweety homolog 1 isoform X3	3012	0
XM_020093004.1	neuronal pentraxin-2-like	2832	0
XM_020093005.1	tyrosine-protein phosphatase non-receptor type 6 isoform X1	1932	0
XM_020093006.1	tyrosine-protein phosphatase non-receptor type 6 isoform X2	2173	0
XM_020093007.1	calcium/calmodulin-dependent protein kinase type II subunit gamma isoform X19	5199	0
XM_020093008.1	rap1 GTPase-activating protein 2-like	2039	0
XM_020093009.1	zinc finger and BTB domain-containing protein 7B-like	4700	0

XM_020093010.1	zinc finger and BTB domain-containing protein 7B-like	4603	0
XM_020093011.1	folliculin isoform X1	2794	0
XM_020093012.1	folliculin isoform X2	2781	0
XM_020093013.1	leukocyte receptor cluster member 9	2568	0
XM_020093014.1	zinc finger protein 384-like isoform X1	2836	0
XM_020093015.1	zinc finger protein 384-like isoform X2	2610	0
XM_020093016.1	calcium/calmodulin-dependent protein kinase type II subunit gamma isoform X20	5190	0
XM_020093017.1	inward rectifier potassium channel 2-like	6234	0
XM_020093018.1	inward rectifier potassium channel 2-like	6060	0
XM_020093019.1	inward rectifier potassium channel 2-like	5924	0
XM_020093020.1	inward rectifier potassium channel 2-like	6113	0
XM_020093021.1	tumor suppressor p53-binding protein 1-like	1521	0
XM_020093022.1	nectin-2 isoform X1	2699	0
XM_020093023.1	nectin-2 isoform X2	3232	0
XM_020093024.1	nectin-2 isoform X3	3229	0
XM_020093025.1	protein ABHD16A isoform X1	2183	0
XM_020093026.1	protein ABHD16A isoform X2	2221	0
XM_020093027.1	calcium/calmodulin-dependent protein kinase type II subunit gamma isoform X21	5187	0
XM_020093028.1	solute carrier family 2, facilitated glucose transporter member 1-like	3706	0
XM_020093029.1	U4/U6 small nuclear ribonucleoprotein Prp31	1782	0
XM_020093030.1	splicing factor U2AF 65 kDa subunit isoform X1	2128	0
XM_020093031.1	splicing factor U2AF 65 kDa subunit isoform X2	2110	0
XM_020093032.1	splicing factor U2AF 65 kDa subunit isoform X3	2092	0
XM_020093033.1	splicing factor U2AF 65 kDa subunit isoform X4	2074	0
XM_020093034.1	lysophospholipid acyltransferase 7	3590	0
XM_020093035.1	lysophospholipid acyltransferase 7	3541	0
XM_020093036.1	calcium/calmodulin-dependent protein kinase type II subunit gamma isoform X22	5178	0
XM_020093037.1	zinc-binding protein A33-like	3218	0
XM_020093038.1	lysophospholipid acyltransferase 5 isoform X1	2977	0
XM_020093039.1	lysophospholipid acyltransferase 5 isoform X2	2974	0
XM_020093040.1	caspase-2 isoform X1	3103	0
XM_020093041.1	caspase-2 isoform X2	3091	0
XM_020093042.1	caspase-2 isoform X3	3028	0
XM_020093043.1	CUGBP Elav-like family member 3 isoform X1	2327	0
XM_020093044.1	CUGBP Elav-like family member 3 isoform X2	2321	0
XM_020093045.1	calcium/calmodulin-dependent protein kinase type II subunit gamma isoform X23	5154	0
XM_020093046.1	CUGBP Elav-like family member 3 isoform X3	2315	0
XM_020093047.1	CUGBP Elav-like family member 3 isoform X4	2300	0
XM_020093048.1	CUGBP Elav-like family member 3 isoform X5	2267	0
XM_020093049.1	glutamate-rich WD repeat-containing protein 1	1591	0
XM_020093050.1	tyrosine-protein kinase STYK1-like	2268	0
XM_020093051.1	cell division cycle-associated protein 3 isoform X1	1620	0
XM_020093052.1	cell division cycle-associated protein 3 isoform X2	1617	0
XM_020093053.1	AF4/FMR2 family member 4-like	2891	0
XM_020093054.1	calcium/calmodulin-dependent protein kinase type II subunit gamma isoform X24	5130	0
XM_020093055.1	glycogen synthase kinase-3 beta-like	4125	0
XM_020093056.1	flotillin-2 isoform X1	2885	0
XM_020093057.1	cytohesin-1 isoform X1	4432	0
XM_020093058.1	voltage-dependent calcium channel gamma-4 subunit-like	3430	0
XM_020093059.1	voltage-dependent calcium channel gamma-4 subunit-like	3584	0
XM_020093060.1	coiled-coil domain-containing protein 106-like isoform X1	3224	0
XM_020093061.1	coiled-coil domain-containing protein 106-like isoform X2	3218	0
XM_020093062.1	calcium/calmodulin-dependent protein kinase type II subunit gamma isoform X25	5127	0
XM_020093063.1	tumor necrosis factor receptor superfamily member 1A	3076	0
XM_020093064.1	dual specificity protein kinase CLK2-like	2249	0
XM_020093065.1	C-X-C chemokine receptor type 3-like	1439	0
XM_020093066.1	death effector domain-containing protein-like	1907	0
XM_020093067.1	ceramide synthase 2-like	3193	0
XM_020093068.1	urotensin-2 receptor-like isoform X2	2378	1.47E-168
XM_020093069.1	C-X-C chemokine receptor type 3-2-like	1817	0
XM_020093070.1	calcium/calmodulin-dependent protein kinase type II subunit gamma isoform X26	5118	0
XM_020093071.1	tubulin beta chain isoform X2	1657	0
XM_020093072.1	tubulin beta chain isoform X2	1620	0
XM_020093073.1	complement C1r subcomponent-like isoform X1	1284	0
XM_020093074.1	complement C1r subcomponent-like isoform X2	1162	0
XM_020093075.1	hepatic leukemia factor-like	4528	1.32E-173
XM_020093076.1	C-X-C chemokine receptor type 3-like	1529	0
XM_020093077.1	C-X-C chemokine receptor type 3-like	1526	0
XM_020093078.1	C-X-C chemokine receptor type 3-like	1497	0
XM_020093079.1	calcium/calmodulin-dependent protein kinase type II subunit gamma isoform X27	5094	0
XM_020093080.1	C-X-C chemokine receptor type 3-like	1414	0
XM_020093081.1	zinc-binding protein A33-like	1315	0
XM_020093082.1	germ cell-specific gene 1-like protein	2377	0
XM_020093083.1	germ cell-specific gene 1-like protein	3629	0
XM_020093084.1	guanine nucleotide-binding protein G(I)/G(S)/G(T) subunit beta-3	2239	0
XM_020093085.1	guanine nucleotide-binding protein G(I)/G(S)/G(T) subunit beta-3	2270	0
XM_020093086.1	guanine nucleotide-binding protein G(I)/G(S)/G(T) subunit beta-3	2245	0
XM_020093087.1	guanine nucleotide-binding protein G(I)/G(S)/G(T) subunit beta-3	2223	0
XM_020093088.1	calcium/calmodulin-dependent protein kinase type II subunit gamma isoform X28	5085	0

XM_020093089.1	guanine nucleotide-binding protein G(I)/G(S)/G(T) subunit beta-3	2187	0
XM_020093090.1	voltage-dependent calcium channel gamma-7 subunit-like	4238	0
XM_020093091.1	glyceraldehyde-3-phosphate dehydrogenase	1539	0
XM_020093092.1	cdc42 effector protein 4-like	2145	1.01E-168
XM_020093093.1	tumor necrosis factor receptor superfamily member 5-like	2406	0
XM_020093094.1	cytoplasmic phosphatidylinositol transfer protein 1-like isoform X1	3344	0
XM_020093095.1	T-cell surface glycoprotein CD4-2	1519	0
XM_020093096.1	cation-dependent mannose-6-phosphate receptor	2500	0
XM_020093097.1	zinc finger protein 205-like	1257	1.23E-180
XM_020093098.1	leukocyte receptor cluster member 1	2095	1.76E-87
XM_020093099.1	conserved oligomeric Golgi complex subunit 2	3172	0
XM_020093100.1	COP9 signalosome complex subunit 7a isoform X1	1537	2.88E-172
XM_020093101.1	COP9 signalosome complex subunit 7a isoform X2	1545	6.29E-167
XM_020093102.1	voltage-dependent calcium channel gamma-6 subunit	1259	2.80E-123
XM_020093103.1	voltage-dependent calcium channel gamma-6 subunit	3124	4.95E-116
XM_020093104.1	inhibitor of growth protein 1	1511	0
XM_020093105.1	adaptin ear-binding coat-associated protein 1-like isoform X2	2385	1.84E-138
XM_020093106.1	adaptin ear-binding coat-associated protein 1-like isoform X1	2377	4.53E-130
XM_020093107.1	INO80 complex subunit E isoform X1	1471	3.52E-92
XM_020093108.1	INO80 complex subunit E isoform X2	1380	1.35E-66
XM_020093109.1	triosephosphate isomerase	1472	2.25E-175
XM_020093110.1	ribosomal RNA small subunit methyltransferase NEP1	1205	3.36E-176
XM_020093111.1	ubiquitin-conjugating enzyme E2 S	2159	1.71E-136
XM_020093112.1	deleted in malignant brain tumors 1 protein-like isoform X1	1862	0
XM_020093113.1	early activation antigen CD69-like	2575	1.11E-151
XM_020093114.1	PREDICTED: uncharacterized protein LOC109633328 isoform X1	1650	6.19E-142
XM_020093115.1	PREDICTED: uncharacterized protein LOC109633328 isoform X2	1623	8.35E-141
XM_020093116.1	glutathione S-transferase kappa 1	1107	2.44E-169
XM_020093117.1	glutathione S-transferase kappa 1	1104	2.46E-169
XM_020093118.1	platelet-activating factor acetylhydrolase IB subunit gamma	1980	2.36E-161
XM_020093119.1	platelet-activating factor acetylhydrolase IB subunit gamma	1983	2.44E-161
XM_020093120.1	platelet-activating factor acetylhydrolase IB subunit gamma	1866	6.84E-162
XM_020093121.1	deleted in malignant brain tumors 1 protein-like isoform X2	1701	0
XM_020093122.1	PILR alpha-associated neural protein	1350	1.36E-116
XM_020093123.1	C-type lectin domain family 4 member E-like	906	1.12E-167
XM_020093124.1	protein lev-9-like	752	3.92E-149
XM_020093125.1	isochorismatase domain-containing protein 2	1026	3.13E-144
XM_020093126.1	isochorismatase domain-containing protein 2	888	5.39E-145
XM_020093127.1	40S ribosomal protein S9	771	5.31E-129
XM_020093128.1	PREDICTED: visinin-like	1178	8.63E-138
XM_020093129.1	60S ribosomal protein L18	758	6.40E-117
XM_020093130.1	PREDICTED: josephin-2	2051	9.99E-120
XM_020093131.1	PREDICTED: josephin-2	2053	9.99E-120
XM_020093132.1	protein Z-dependent protease inhibitor-like	2438	0
XM_020093133.1	mpv17-like protein	1189	5.72E-119
XM_020093134.1	retinol-binding protein 5	821	1.75E-101
XM_020093135.1	C-type natriuretic peptide 1	1619	3.59E-63
XM_020093136.1	microfibrillar-associated protein 5	900	1.89E-92
XM_020093137.1	39S ribosomal protein L51, mitochondrial	813	1.97E-89
XM_020093138.1	vesicle-associated membrane protein 2-like	2968	2.46E-43
XM_020093139.1	gamma-aminobutyric acid receptor-associated protein-like 1	1029	2.67E-63
XM_020093140.1	serine/threonine-protein kinase SBK1-like	933	2.08E-83
XM_020093141.1	ubiquitin-like protein ATG12	543	7.31E-69
XM_020093142.1	PLAC8-like protein 1	862	3.65E-68
XM_020093143.1	zinc finger protein Pegasus-like	2221	0
XM_020093144.1	transmembrane protein 238-like	658	3.18E-44
XM_020093145.1	zinc finger protein castor homolog 1-like	690	1.21E-43
XM_020093146.1	cytochrome c oxidase subunit 6B1-like	893	5.45E-60
XM_020093147.1	cytochrome c oxidase subunit 6B1-like	900	5.81E-60
XM_020093148.1	glycine receptor subunit alphaZ1-like	766	3.21E-167
XM_020093149.1	glutamate receptor ionotropic, NMDA 2D-like	8888	0
XM_020093150.1	T-cell surface glycoprotein CD4-1	1721	0
XM_020093151.1	carcinoembryonic antigen-related cell adhesion molecule 5-like	2884	0
XM_020093152.1	zinc finger protein Pegasus-like	2248	0
XM_020093153.1	protein 4.1-like isoform X1	5655	0
XM_020093154.1	protein 4.1-like isoform X2	5592	0
XM_020093155.1	protein 4.1-like isoform X3	5586	0
XM_020093156.1	protein 4.1-like isoform X1	5546	0
XM_020093157.1	protein 4.1-like isoform X5	5541	0
XM_020093158.1	protein 4.1-like isoform X6	5522	0
XM_020093159.1	protein 4.1-like isoform X7	5477	0
XM_020093160.1	protein 4.1-like isoform X8	5296	0
XM_020093161.1	protein 4.1-like isoform X9	5232	0
XM_020093162.1	protein 4.1-like isoform X10	5172	0
XM_020093163.1	protein 4.1-like isoform X11	4103	0
XM_020093164.1	homeobox protein Hox-D4b-like	4145	0
XM_020093165.1	casein kinase II subunit beta-like	1222	1.24E-145
XM_020093166.1	casein kinase II subunit beta-like	1219	1.87E-148
XM_020093167.1	serine/arginine-rich splicing factor 10-like isoform X1	1558	1.45E-74

XM_020093168.1	serine/arginine-rich splicing factor 10-like isoform X2	1758	2.60E-37
XM_020093169.1	proline-rich nuclear receptor coactivator 2-like	3255	1.13E-77
XM_020093170.1	apolipoprotein A-I-like	1159	0
XM_020093171.1	serine/threonine-protein phosphatase 6 regulatory ankyrin repeat subunit A	4102	0
XM_020093172.1	histone deacetylase 9 isoform X1	4656	0
XM_020093173.1	histone deacetylase 9 isoform X1	2250	0
XM_020093174.1	histone deacetylase 9 isoform X1	2918	0
XM_020093175.1	histone deacetylase 9 isoform X3	3829	0
XM_020093176.1	histone deacetylase 9 isoform X3	4104	0
XM_020093177.1	homeobox protein Hox-D9b-like	4462	2.92E-168
XM_020093178.1	histone deacetylase 9 isoform X1	3786	0
XM_020093179.1	GRAM domain-containing protein 1A isoform X1	4752	0
XM_020093180.1	GRAM domain-containing protein 1A isoform X2	4738	0
XM_020093181.1	GRAM domain-containing protein 1A isoform X3	4497	0
XM_020093182.1	GRAM domain-containing protein 1A isoform X4	4617	0
XM_020093183.1	dnaJ homolog subfamily C member 28	1563	0
XM_020093184.1	sodium channel subunit beta-1-like	1318	9.03E-118
XM_020093185.1	apoptosis-associated speck-like protein containing a CARD isoform X1	1012	1.16E-134
XM_020093186.1	apoptosis-associated speck-like protein containing a CARD isoform X2	1006	1.84E-133
XM_020093187.1	apoptosis-associated speck-like protein containing a CARD isoform X3	946	1.10E-116
XM_020093188.1	potassium voltage-gated channel subfamily E member 1-like	643	1.02E-101
XM_020093189.1	homeobox protein Hox-D11b-like isoform X1	3021	0
XM_020093190.1	histone-lysine N-methyltransferase ASH1L-like isoform X2	8278	0
XM_020093191.1	histone-lysine N-methyltransferase ASH1L-like isoform X2	5576	0
XM_020093192.1	RUN and SH3 domain-containing protein 1 isoform X1	8356	0
XM_020093193.1	RUN and SH3 domain-containing protein 1 isoform X2	8338	0
XM_020093194.1	RUN and SH3 domain-containing protein 1 isoform X3	7486	0
XM_020093195.1	RUN and SH3 domain-containing protein 1 isoform X4	7468	0
XM_020093196.1	transmembrane protein 245	4492	0
XM_020093197.1	homeobox protein Hox-D11b-like isoform X2	3018	0
XM_020093198.1	polypeptide N-acetylgalactosaminyltransferase 1	4219	0
XM_020093199.1	DOMON domain-containing protein FRRS1L	2216	6.10E-178
XM_020093200.1	phospholipid-transporting ATPase ID-like	5410	0
XM_020093201.1	protein numb homolog isoform X1	3204	0
XM_020093202.1	protein numb homolog isoform X1	3390	0
XM_020093203.1	protein numb homolog isoform X1	3171	0
XM_020093204.1	protein numb homolog isoform X1	3070	0
XM_020093205.1	protein numb homolog isoform X4	3037	0
XM_020093206.1	homeobox protein Hox-D11b-like isoform X1	2165	0
XM_020093207.1	polypeptide N-acetylgalactosaminyltransferase 16	2600	0
XM_020093208.1	regulation of nuclear pre-mRNA domain-containing protein 2-like isoform X1	6089	0
XM_020093209.1	regulation of nuclear pre-mRNA domain-containing protein 2-like isoform X2	6086	0
XM_020093210.1	threonine--tRNA ligase, cytoplasmic-like isoform X3	3346	0
XM_020093211.1	threonine--tRNA ligase, cytoplasmic-like isoform X4	3341	0
XM_020093212.1	testis-expressed sequence 10 protein	3185	0
XM_020093213.1	testis-expressed sequence 10 protein	3120	0
XM_020093214.1	testis-expressed sequence 10 protein	3523	0
XM_020093215.1	inversin isoform X1	3141	0
XM_020093216.1	inversin isoform X2	3141	0
XM_020093217.1	PREDICTED: uncharacterized protein LOC109633389 isoform X1	1608	0
XM_020093218.1	PREDICTED: uncharacterized protein LOC109633389 isoform X1	1595	0
XM_020093219.1	PREDICTED: uncharacterized protein LOC109633389 isoform X1	1576	0
XM_020093220.1	metaxin-3 isoform X2	1508	0
XM_020093221.1	rho guanine nucleotide exchange factor 1 isoform X1	6759	0
XM_020093222.1	rho guanine nucleotide exchange factor 1 isoform X1	6779	0
XM_020093223.1	rho guanine nucleotide exchange factor 1 isoform X1	6519	0
XM_020093224.1	rho guanine nucleotide exchange factor 1 isoform X1	6510	0
XM_020093225.1	rho guanine nucleotide exchange factor 1 isoform X1	6348	0
XM_020093226.1	rho guanine nucleotide exchange factor 1 isoform X1	6547	0
XM_020093227.1	rho guanine nucleotide exchange factor 1 isoform X1	6240	0
XM_020093228.1	B-cell antigen receptor complex-associated protein alpha chain	1624	2.17E-158
XM_020093229.1	IgGFc-binding protein-like	16767	0
XM_020093230.1	PREDICTED: claudin-12	2500	4.74E-179
XM_020093231.1	GTP-binding protein 10	1305	0
XM_020093232.1	tax1-binding protein 1 homolog B-like	3548	0
XM_020093233.1	alpha-2A adrenergic receptor-like	1434	0
XM_020093234.1	tax1-binding protein 1 homolog B-like	3552	0
XM_020093235.1	3-hydroxyisobutyrate dehydrogenase, mitochondrial-like	1643	0
XM_020093236.1	circadian-associated transcriptional repressor	2616	0
XM_020093237.1	circadian-associated transcriptional repressor	2575	0
XM_020093238.1	carbonic anhydrase 14 isoform X1	1901	0
XM_020093239.1	carbonic anhydrase 14 isoform X2	1877	0
XM_020093240.1	carbonic anhydrase 14 isoform X3	1847	0
XM_020093241.1	carbonic anhydrase 14 isoform X4	1823	0
XM_020093242.1	carbonic anhydrase 14 isoform X5	1616	0
XM_020093243.1	cellular retinoic acid-binding protein 2-like	1361	7.25E-84
XM_020093244.1	kinesin-like protein KIFC3	4440	0
XM_020093245.1	max-interacting protein 1-like	2681	2.82E-158
XM_020093246.1	centrosomal protein of 72 kDa isoform X1	5521	0

XM_020093247.1	centrosomal protein of 72 kDa isoform X2	1818	0
XM_020093248.1	tubulin polymerization-promoting protein isoform X1	1331	0
XM_020093249.1	tubulin polymerization-promoting protein isoform X2	1175	2.16E-174
XM_020093250.1	coiled-coil domain-containing protein 12	823	3.26E-94
XM_020093251.1	metal regulatory transcription factor 1	2518	0
XM_020093252.1	metal regulatory transcription factor 1	5436	0
XM_020093253.1	glycoprotein endo-alpha-1,2-mannosidase-like protein	5149	0
XM_020093254.1	yrdC domain-containing protein, mitochondrial	1244	1.54E-180
XM_020093255.1	survival of motor neuron-related-splicing factor 30	1312	7.35E-170
XM_020093256.1	ras-related protein Rab-13	2575	3.67E-128
XM_020093257.1	40S ribosomal protein S27-like isoform X1	1209	1.73E-54
XM_020093258.1	40S ribosomal protein S27-like isoform X2	1210	9.37E-54
XM_020093259.1	probable ATP-dependent RNA helicase DDX6	4354	0
XM_020093260.1	probable ATP-dependent RNA helicase DDX6	4821	0
XM_020093261.1	protein FAM171A1 isoform X1	5179	0
XM_020093262.1	protein FAM171A1 isoform X2	5086	0
XM_020093263.1	H-2 class II histocompatibility antigen, A-U alpha chain-like	1485	8.38E-172
XM_020093264.1	spermatogenesis-associated protein 4	777	3.90E-178
XM_020093265.1	toll like receptor 14	3672	0
XM_020093266.1	semaphorin-4A-like isoform X1	5871	0
XM_020093267.1	leucine-rich repeat and immunoglobulin-like domain-containing nogo receptor-interacting protein 1	3112	0
XM_020093268.1	leucine-rich repeat and immunoglobulin-like domain-containing nogo receptor-interacting protein 1	3362	0
XM_020093269.1	zona pellucida sperm-binding protein 3-like	1575	0
XM_020093270.1	Friend leukemia integration 1 transcription factor-like isoform X1	2360	0
XM_020093271.1	Friend leukemia integration 1 transcription factor-like isoform X1	2683	0
XM_020093272.1	Friend leukemia integration 1 transcription factor-like isoform X3	1374	0
XM_020093273.1	coiled-coil domain-containing protein 105	1993	0
XM_020093274.1	sperm acrosome membrane-associated protein 4-like	802	2.79E-61
XM_020093275.1	HORMA domain-containing protein 1-like	1799	0
XM_020093276.1	alpha-endosulfine isoform X1	3519	1.64E-56
XM_020093277.1	alpha-endosulfine isoform X2	692	1.01E-42
XM_020093278.1	ligand-dependent corepressor-like isoform X2	7263	0
XM_020093279.1	double-stranded RNA-specific adenosine deaminase	5100	0
XM_020093280.1	double-stranded RNA-specific adenosine deaminase	5642	0
XM_020093281.1	neuronal acetylcholine receptor subunit alpha-7-like isoform X1	2144	0
XM_020093282.1	neuronal acetylcholine receptor subunit alpha-7-like isoform X1	2164	0
XM_020093283.1	endoplasmic reticulum resident protein 44	3394	0
XM_020093284.1	PREDICTED: syntaxin-17	3737	1.02E-151
XM_020093285.1	beta-1,4-galactosyltransferase 3-like	4593	0
XM_020093286.1	beta-1,4-galactosyltransferase 3-like	4572	0
XM_020093287.1	ligand-dependent corepressor-like isoform X2	2037	0
XM_020093288.1	protein SMG9	2289	0
XM_020093289.1	zinc finger protein 91-like	2309	0
XM_020093290.1	zinc finger protein 91-like	2230	0
XM_020093291.1	zinc finger protein 91-like	2062	0
XM_020093292.1	urokinase plasminogen activator surface receptor-like	850	1.67E-145
XM_020093293.1	intermediate conductance calcium-activated potassium channel protein 4	1773	0
XM_020093294.1	FAST kinase domain-containing protein 1, mitochondrial	2529	0
XM_020093295.1	alpha-1,3/1,6-mannosyltransferase ALG2	2667	0
XM_020093296.1	phospholipid-metabolizing enzyme A-C1-like	1271	5.20E-135
XM_020093297.1	protein transport protein Sec61 subunit beta	933	6.38E-35
XM_020093298.1	MORN repeat-containing protein 2	2615	1.59E-125
XM_020093299.1	serine/threonine-protein kinase 40	4989	0
XM_020093300.1	phosphatidylinositol 4-phosphate 5-kinase type-1 alpha-like isoform X1	4918	0
XM_020093301.1	phosphatidylinositol 4-phosphate 5-kinase type-1 alpha-like isoform X1	4859	0
XM_020093302.1	26S proteasome non-ATPase regulatory subunit 4-like	1508	0
XM_020093303.1	PDZ domain-containing protein 2-like	5175	0
XM_020093304.1	PDZ domain-containing protein 2-like	4844	0
XM_020093305.1	PREDICTED: aquaporin-10-like	1562	0
XM_020093306.1	PREDICTED: uncharacterized protein C1orf43 homolog isoform X1	1782	7.69E-147
XM_020093307.1	PREDICTED: uncharacterized protein C1orf43 homolog isoform X1	1706	6.70E-141
XM_020093308.1	protein DBF4 homolog A	3123	0
XM_020093309.1	RUN domain-containing protein 3B	2677	0
XM_020093310.1	protein FAM135A isoform X1	5552	0
XM_020093311.1	solute carrier family 25 member 40	2275	0
XM_020093312.1	cell surface glycoprotein MUC18-like isoform X1	3960	0
XM_020093313.1	cell surface glycoprotein MUC18-like isoform X2	3927	0
XM_020093314.1	SHC-transforming protein 1 isoform X1	4619	0
XM_020093315.1	SHC-transforming protein 1 isoform X1	4163	0
XM_020093316.1	ras-related GTP-binding protein A	3137	0
XM_020093317.1	C-Myc-binding protein-like isoform X1	750	7.18E-67
XM_020093318.1	C-Myc-binding protein-like isoform X2	842	2.84E-63
XM_020093319.1	protein FAM135A isoform X2	5549	0
XM_020093320.1	peroxisomal carnitine O-octanoyltransferase	3006	0
XM_020093321.1	phosphatidylcholine translocator ABCB4-like	1836	6.02E-116
XM_020093322.1	acyl-CoA-binding domain-containing protein 7	883	5.47E-58
XM_020093323.1	lethal(3)malignant brain tumor-like protein 3 isoform X1	3999	0
XM_020093324.1	lethal(3)malignant brain tumor-like protein 3 isoform X2	3972	0
XM_020093325.1	lethal(3)malignant brain tumor-like protein 3 isoform X3	3972	0

XM_020093326.1	lethal(3)malignant brain tumor-like protein 3 isoform X4	3945	0
XM_020093327.1	lethal(3)malignant brain tumor-like protein 3 isoform X5	5106	0
XM_020093328.1	protein FAM135A isoform X3	5549	0
XM_020093329.1	lethal(3)malignant brain tumor-like protein 3 isoform X5	4120	0
XM_020093330.1	lethal(3)malignant brain tumor-like protein 3 isoform X5	3947	0
XM_020093331.1	lethal(3)malignant brain tumor-like protein 3 isoform X5	3985	0
XM_020093332.1	lethal(3)malignant brain tumor-like protein 3 isoform X5	4106	0
XM_020093334.1	integrin alpha-10	3421	0
XM_020093335.1	DNA-directed RNA polymerase III subunit RPC3	1967	0
XM_020093336.1	E3 ubiquitin-protein ligase RNF115	2169	6.43E-166
XM_020093337.1	protein FAM135A isoform X4	5501	0
XM_020093338.1	pleckstrin homology domain-containing family G member 4B isoform X1	5831	0
XM_020093339.1	pleckstrin homology domain-containing family G member 4B isoform X2	5768	0
XM_020093340.1	pleckstrin homology domain-containing family G member 4B isoform X3	5127	0
XM_020093341.1	pleckstrin homology domain-containing family G member 4B isoform X1	5699	0
XM_020093342.1	hormone-sensitive lipase isoform X1	2721	0
XM_020093343.1	hormone-sensitive lipase isoform X1	6376	0
XM_020093344.1	neurobeachin-like protein 2 isoform X1	11750	0
XM_020093345.1	protein FAM135A isoform X5	5498	0
XM_020093346.1	neurobeachin-like protein 2 isoform X2	11663	0
XM_020093347.1	MAGUK p55 subfamily member 6-like	2873	0
XM_020093348.1	MAGUK p55 subfamily member 6-like	3096	0
XM_020093349.1	MAGUK p55 subfamily member 6-like	2788	0
XM_020093350.1	MAGUK p55 subfamily member 6-like	3011	0
XM_020093351.1	non-syndromic hearing impairment protein 5 isoform X1	2233	0
XM_020093352.1	non-syndromic hearing impairment protein 5 isoform X1	2061	0
XM_020093353.1	kin of IRRE-like protein 1	1753	0
XM_020093354.1	putative Ras-related protein Rab-42	2739	1.71E-158
XM_020093355.1	transcription initiation factor TFIID subunit 12	1025	6.73E-94
XM_020093356.1	cytosolic 5'-nucleotidase 3A isoform X2	2793	0
XM_020093357.1	cytosolic 5'-nucleotidase 3A isoform X2	2411	0
XM_020093358.1	collagen alpha-1(IX) chain isoform X1	3808	0
XM_020093359.1	cytosolic 5'-nucleotidase 3A isoform X2	2434	0
XM_020093360.1	nucleolar GTP-binding protein 2	3297	0
XM_020093361.1	axonemal dynein light intermediate polypeptide 1-like	1054	1.11E-171
XM_020093362.1	thyroid hormone receptor-associated protein 3 isoform X1	4192	0
XM_020093363.1	thyroid hormone receptor-associated protein 3 isoform X1	4191	0
XM_020093364.1	thyroid hormone receptor-associated protein 3 isoform X1	4146	0
XM_020093365.1	thyroid hormone receptor-associated protein 3 isoform X1	4097	0
XM_020093366.1	thyroid hormone receptor-associated protein 3 isoform X1	4001	0
XM_020093367.1	collagen alpha-1(IX) chain isoform X2	2978	1.85E-174
XM_020093368.1	thyroid hormone receptor-associated protein 3 isoform X1	3688	0
XM_020093369.1	SH3 domain-containing protein 21 isoform X1	1856	0
XM_020093370.1	SH3 domain-containing protein 21 isoform X2	1853	0
XM_020093371.1	SH3 domain-containing protein 21 isoform X3	1841	0
XM_020093372.1	mannosyl-oligosaccharide 1,2-alpha-mannosidase IA-like isoform X1	4958	0
XM_020093373.1	mannosyl-oligosaccharide 1,2-alpha-mannosidase IA-like isoform X2	3240	0
XM_020093374.1	nascent polypeptide-associated complex subunit alpha isoform X4	6990	0
XM_020093375.1	Golgi-associated plant pathogenesis-related protein 1	1642	1.17E-111
XM_020093376.1	upstream stimulatory factor 2-like isoform X1	3424	0
XM_020093377.1	upstream stimulatory factor 2-like isoform X2	3386	0
XM_020093378.1	kinesin-like protein KIF13A isoform X1	7696	0
XM_020093379.1	kinesin-like protein KIF13A isoform X2	7693	0
XM_020093380.1	serine/threonine-protein kinase MARK1 isoform X1	4107	0
XM_020093381.1	kinesin-like protein KIF13A isoform X3	7693	0
XM_020093382.1	kinesin-like protein KIF13A isoform X4	7687	0
XM_020093383.1	kinesin-like protein KIF13A isoform X5	7684	0
XM_020093384.1	kinesin-like protein KIF13A isoform X6	7663	0
XM_020093385.1	kinesin-like protein KIF13A isoform X7	7525	0
XM_020093386.1	kinesin-like protein KIF13A isoform X8	7522	0
XM_020093387.1	kinesin-like protein KIF13A isoform X9	7519	0
XM_020093388.1	kinesin-like protein KIF13A isoform X10	7486	0
XM_020093389.1	induced myeloid leukemia cell differentiation protein Mcl-1 homolog	1892	0
XM_020093390.1	RING finger protein 212B	983	0
XM_020093391.1	serine/threonine-protein kinase MARK1 isoform X2	2798	0
XM_020093392.1	pre-B-cell leukemia transcription factor-interacting protein 1-like isoform X1	4076	0
XM_020093393.1	pre-B-cell leukemia transcription factor-interacting protein 1-like isoform X2	4073	0
XM_020093394.1	pre-B-cell leukemia transcription factor-interacting protein 1-like isoform X3	4070	0
XM_020093395.1	trichohyalin-like isoform X4	4052	0
XM_020093396.1	pre-B-cell leukemia transcription factor-interacting protein 1-like isoform X5	3932	0
XM_020093397.1	pre-B-cell leukemia transcription factor-interacting protein 1-like isoform X6	3929	0
XM_020093398.1	neurofilament heavy polypeptide-like	1811	3.54E-111
XM_020093399.1	PREDICTED: uncharacterized protein LOC109633489	1209	2.50E-92
XM_020093400.1	pyruvate dehyrogenase phosphatase catalytic subunit 1	3504	0
XM_020093401.1	pyruvate dehyrogenase phosphatase catalytic subunit 1	3461	0
XM_020093402.1	pyruvate dehyrogenase phosphatase catalytic subunit 1	3319	0
XM_020093403.1	pyruvate dehyrogenase phosphatase catalytic subunit 1	3594	0
XM_020093404.1	Na(+)/H(+) exchanger beta-like isoform X1	4346	0
XM_020093405.1	rod cGMP-specific 3',5'-cyclic phosphodiesterase subunit beta	2951	0
XM_020093406.1	Na(+)/H(+) exchanger beta-like isoform X2	4288	0

XM_020093407.1	26S protease regulatory subunit 6B isoform X2	1626	0
XM_020093408.1	26S protease regulatory subunit 6B isoform X2	1673	0
XM_020093409.1	26S protease regulatory subunit 6B isoform X2	1584	0
XM_020093410.1	PREDICTED: uncharacterized protein LOC109633494	2895	0
XM_020093411.1	CTP synthase 1-like	3310	0
XM_020093412.1	phosphatidylserine synthase 1	3098	0
XM_020093413.1	bromodomain-containing protein 9 isoform X1	2287	0
XM_020093414.1	bromodomain-containing protein 9 isoform X2	2224	0
XM_020093415.1	pachytene checkpoint protein 2 homolog	2224	0
XM_020093416.1	phosphatase and actin regulator 4A-like isoform X2	4016	0
XM_020093417.1	phosphatase and actin regulator 4A-like isoform X2	4025	0
XM_020093418.1	PREDICTED: sideroflexin-1-like	4241	0
XM_020093419.1	nuclear receptor ROR-beta-like	2204	0
XM_020093420.1	extracellular matrix protein 1-like isoform X1	2246	0
XM_020093421.1	extracellular matrix protein 1-like isoform X2	2078	0
XM_020093422.1	PREDICTED: akirin-1	1399	3.48E-111
XM_020093423.1	casein kinase II subunit alpha-like	2273	0
XM_020093424.1	casein kinase II subunit alpha-like	2221	0
XM_020093425.1	casein kinase II subunit alpha-like	2148	0
XM_020093426.1	casein kinase II subunit alpha-like	2385	0
XM_020093427.1	protein lifeguard 1-like	2090	4.98E-178
XM_020093428.1	protein lifeguard 1-like	2171	1.20E-177
XM_020093429.1	speriolin-like protein	1059	5.56E-174
XM_020093430.1	redox-regulatory protein FAM213A-like isoform X1	1656	0
XM_020093431.1	ribonuclease P protein subunit p38	2491	8.63E-174
XM_020093432.1	cathepsin S-like	2223	0
XM_020093433.1	dynein heavy chain 11, axonemal	14114	0
XM_020093434.1	polycomb protein SCMH1 isoform X1	3382	0
XM_020093435.1	polycomb protein SCMH1 isoform X1	3374	0
XM_020093436.1	polycomb protein SCMH1 isoform X1	3291	0
XM_020093437.1	polycomb protein SCMH1 isoform X1	3380	0
XM_020093438.1	polycomb protein SCMH1 isoform X1	3380	0
XM_020093439.1	polycomb protein SCMH1 isoform X1	2951	0
XM_020093440.1	redox-regulatory protein FAM213A-like isoform X1	1752	0
XM_020093441.1	MAP7 domain-containing protein 1 isoform X2	3655	0
XM_020093442.1	MAP7 domain-containing protein 1 isoform X1	3653	0
XM_020093443.1	MAP7 domain-containing protein 1 isoform X3	3634	0
XM_020093444.1	MAP7 domain-containing protein 1 isoform X4	3550	0
XM_020093445.1	MAP7 domain-containing protein 1 isoform X5	3529	0
XM_020093446.1	tyrosine-protein phosphatase non-receptor type 21 isoform X1	3465	0
XM_020093447.1	cbp/p300-interacting transactivator 3-like	1982	2.38E-127
XM_020093448.1	redox-regulatory protein FAM213A-like isoform X1	1503	2.63E-145
XM_020093449.1	kin of IRRE-like protein 2 isoform X1	4618	0
XM_020093450.1	kin of IRRE-like protein 2 isoform X2	2808	0
XM_020093451.1	transcription factor Sp8 isoform X2	2537	0
XM_020093452.1	transcription factor Sp8 isoform X2	2714	0
XM_020093453.1	transcription factor Sp8 isoform X2	2599	0
XM_020093454.1	potassium channel subfamily K member 5-like	2206	0
XM_020093455.1	serum paraoxonase/arylesterase 2-like	1455	0
XM_020093456.1	redox-regulatory protein FAM213A-like isoform X1	1447	7.16E-145
XM_020093457.1	beta-chimaerin isoform X2	2668	0
XM_020093458.1	beta-chimaerin isoform X2	2229	0
XM_020093459.1	beta-chimaerin isoform X2	2182	0
XM_020093460.1	beta-chimaerin isoform X2	1984	0
XM_020093461.1	U4/U6 small nuclear ribonucleoprotein Prp3	2954	0
XM_020093462.1	carcinoembryonic antigen-related cell adhesion molecule 1-like	5318	0
XM_020093463.1	cell adhesion molecule 4 isoform X1	4879	0
XM_020093464.1	cell adhesion molecule 4 isoform X2	3005	0
XM_020093465.1	cell adhesion molecule 4 isoform X3	4811	0
XM_020093466.1	cell adhesion molecule 4 isoform X4	4961	0
XM_020093467.1	cell adhesion molecule 4 isoform X5	4892	0
XM_020093468.1	cathepsin K-like isoform X1	1745	0
XM_020093469.1	cathepsin K-like isoform X1	1646	0
XM_020093470.1	intersectin-1 isoform X2	5030	0
XM_020093471.1	type II inositol 1,4,5-trisphosphate 5-phosphatase isoform X1	3287	0
XM_020093472.1	type II inositol 1,4,5-trisphosphate 5-phosphatase isoform X1	3019	0
XM_020093473.1	type II inositol 1,4,5-trisphosphate 5-phosphatase isoform X3	2912	0
XM_020093474.1	type II inositol 1,4,5-trisphosphate 5-phosphatase isoform X3	2779	0
XM_020093475.1	type II inositol 1,4,5-trisphosphate 5-phosphatase isoform X5	2671	0
XM_020093476.1	PR domain zinc finger protein 1-like	4345	0
XM_020093477.1	solute carrier family 45 member 4-like isoform X1	5010	0
XM_020093478.1	solute carrier family 45 member 4-like isoform X2	5007	0
XM_020093479.1	solute carrier family 45 member 4-like isoform X3	5556	0
XM_020093480.1	intersectin-1 isoform X2	5027	0
XM_020093481.1	regulator of chromosome condensation	1950	0
XM_020093482.1	regulator of chromosome condensation	1973	0
XM_020093483.1	nuclear inhibitor of protein phosphatase 1 isoform X2	1859	1.51E-143
XM_020093484.1	pleckstrin homology domain-containing family A member 8	3558	0
XM_020093485.1	lipolysis-stimulated lipoprotein receptor-like isoform X1	2689	0
XM_020093486.1	lipolysis-stimulated lipoprotein receptor-like isoform X2	2628	0

XM_020093487.1	RNA-binding protein 24-like	2167	8.16E-114
XM_020093488.1	diacylglycerol O-acyltransferase 1-like	2757	0

XM_020093489.1	autophagy protein 5 isoform X1	2048	0
XM_020093490.1	autophagy protein 5 isoform X2	1253	2.85E-179
XM_020093491.1	pleckstrin homology-like domain family B member 3 isoform X1	2818	0
XM_020093492.1	DNA polymerase eta	2406	0
XM_020093493.1	pleckstrin homology-like domain family B member 3 isoform X1	3259	0
XM_020093494.1	pleckstrin homology-like domain family B member 3 isoform X1	2771	0
XM_020093495.1	zinc finger protein 687 isoform X1	5286	0
XM_020093496.1	zinc finger protein 687 isoform X1	5360	0
XM_020093497.1	hepatocyte nuclear factor 6-like	6885	0
XM_020093498.1	DNA repair protein XRCC1	3153	0
XM_020093499.1	sorting nexin-27-like isoform X1	4702	0
XM_020093500.1	sorting nexin-27-like isoform X2	4716	0
XM_020093501.1	oxysterol-binding protein-related protein 3 isoform X1	4098	0
XM_020093502.1	oxysterol-binding protein-related protein 3 isoform X2	4092	0
XM_020093503.1	oxysterol-binding protein-related protein 3 isoform X3	3882	0
XM_020093504.1	GTP-binding protein 2	4199	0
XM_020093505.1	oxysterol-binding protein-related protein 3 isoform X4	3996	0
XM_020093506.1	oxysterol-binding protein-related protein 3 isoform X5	3990	0
XM_020093507.1	sphingomyelin phosphodiesterase 5-like isoform X1	2362	0
XM_020093508.1	sphingomyelin phosphodiesterase 5-like isoform X1	2291	0
XM_020093509.1	sphingomyelin phosphodiesterase 5-like isoform X1	1928	0
XM_020093510.1	mitochondrial assembly of ribosomal large subunit protein 1	1031	1.59E-178
XM_020093511.1	myelin-associated glycoprotein isoform X1	4790	0
XM_020093512.1	myelin-associated glycoprotein isoform X2	4736	0
XM_020093513.1	myelin-associated glycoprotein isoform X3	4639	0
XM_020093514.1	myelin-associated glycoprotein isoform X4	4693	0
XM_020093515.1	electron transfer flavoprotein subunit beta	1557	6.32E-147
XM_020093516.1	tumor necrosis factor receptor superfamily member 16-like isoform X1	2849	0
XM_020093517.1	tumor necrosis factor receptor superfamily member 16-like isoform X2	2846	0
XM_020093518.1	PREDICTED: uncharacterized protein LOC109633549	1633	6.11E-142
XM_020093519.1	transmembrane protein 145 isoform X1	2994	0
XM_020093520.1	transmembrane protein 145 isoform X2	2992	0
XM_020093521.1	nibrin isoform X1	2933	0
XM_020093522.1	nibrin isoform X2	2921	0
XM_020093523.1	novel immune-type receptor	3180	0
XM_020093524.1	oxidative stress-induced growth inhibitor 2	2603	0
XM_020093525.1	PREDICTED: uncharacterized protein LOC109633555	3325	0
XM_020093526.1	metalloreductase STEAP3	2185	0
XM_020093527.1	four and a half LIM domains protein 3-like isoform X2	2284	0
XM_020093528.1	PREDICTED: ataxin-1-like	7072	0
XM_020093529.1	40S ribosomal protein S19	532	1.10E-105
XM_020093530.1	Golgi-specific brefeldin A-resistance guanine nucleotide exchange factor 1 isoform X1	6298	0
XM_020093531.1	complement C4-A-like	5432	0
XM_020093532.1	zinc finger protein 883-like	2315	0
XM_020093533.1	cadherin-4-like isoform X3	2887	0
XM_020093534.1	POU domain, class 3, transcription factor 1	4201	0
XM_020093535.1	POU domain, class 3, transcription factor 1	4056	0
XM_020093536.1	transmembrane protein 150A-like	2376	0
XM_020093537.1	Golgi-specific brefeldin A-resistance guanine nucleotide exchange factor 1 isoform X2	6295	0
XM_020093538.1	PREDICTED: occludin-like	2161	0
XM_020093539.1	PREDICTED: occludin-like	2114	0
XM_020093540.1	MARVEL domain-containing protein 2-like	2871	0
XM_020093541.1	zinc finger and BTB domain-containing protein 26-like isoform X3	2693	0
XM_020093542.1	zinc finger and BTB domain-containing protein 26-like isoform X3	2687	0
XM_020093543.1	zinc finger and BTB domain-containing protein 26-like isoform X3	2664	0
XM_020093544.1	zinc finger and BTB domain-containing protein 26-like isoform X3	2615	0
XM_020093545.1	zinc finger and BTB domain-containing protein 26-like isoform X3	2639	0
XM_020093546.1	Golgi-specific brefeldin A-resistance guanine nucleotide exchange factor 1 isoform X3	6286	0
XM_020093547.1	zinc finger and BTB domain-containing protein 26-like isoform X3	2523	0
XM_020093548.1	condensin complex subunit 2	2519	0
XM_020093549.1	lysyl oxidase homolog 2 isoform X1	3195	0
XM_020093550.1	lysyl oxidase homolog 2 isoform X2	1975	0
XM_020093551.1	lysyl oxidase homolog 2 isoform X2	1968	0
XM_020093552.1	lysyl oxidase homolog 2 isoform X2	2197	0
XM_020093553.1	arrestin domain-containing protein 3-like	2297	0
XM_020093554.1	serine/threonine-protein kinase ULK1-like isoform X1	4363	0
XM_020093555.1	serine/threonine-protein kinase ULK1-like isoform X2	4360	0
XM_020093556.1	serine/threonine-protein kinase ULK1-like isoform X3	4360	0
XM_020093557.1	beta-1,3-galactosyltransferase 2-like	2892	0
XM_020093558.1	transport and Golgi organization protein 2 homolog isoform X1	1864	0
XM_020093559.1	mitogen-activated protein kinase kinase kinase kinase 3 isoform X1	3653	0
XM_020093560.1	transport and Golgi organization protein 2 homolog isoform X2	1861	0
XM_020093561.1	vacuolar protein sorting-associated protein 33A	2515	0
XM_020093562.1	cytoplasmic tRNA 2-thiolation protein 1	1722	0
XM_020093563.1	cationic amino acid transporter 4	4152	0
XM_020093564.1	substance-P receptor-like	8915	0
XM_020093565.1	gamma-glutamyltransferase 5-like isoform X1	2390	0

XM_020093566.1	gamma-glutamyltransferase 5-like isoform X2	2310	0
XM_020093567.1	mitogen-activated protein kinase kinase kinase kinase 3 isoform X2	7405	0
XM_020093568.1	transmembrane protein 230	1476	3.51E-57
XM_020093569.1	ATP-binding cassette sub-family B member 9	3097	0
XM_020093570.1	zinc finger matrin-type protein 4 isoform X1	1938	0
XM_020093571.1	zinc finger matrin-type protein 4 isoform X2	1935	0
XM_020093572.1	zinc finger matrin-type protein 4 isoform X3	1609	0
XM_020093573.1	zinc finger matrin-type protein 4 isoform X4	5129	0
XM_020093574.1	zinc finger matrin-type protein 4 isoform X5	1424	0
XM_020093575.1	zinc finger matrin-type protein 4 isoform X6	1782	0
XM_020093576.1	zinc finger matrin-type protein 4 isoform X7	5032	0
XM_020093577.1	mitogen-activated protein kinase kinase kinase kinase 3 isoform X3	3590	0
XM_020093578.1	vesicle-associated membrane protein 5-like	1314	7.57E-32
XM_020093579.1	tetraspanin-33 isoform X2	2135	0
XM_020093580.1	PDZ and LIM domain protein 3	2432	0
XM_020093581.1	RING finger and CHY zinc finger domain-containing protein 1	2107	2.52E-152
XM_020093582.1	SMC5-SMC6 complex localization factor protein 1 isoform X1	3235	0
XM_020093583.1	SMC5-SMC6 complex localization factor protein 1 isoform X2	3130	0
XM_020093584.1	SMC5-SMC6 complex localization factor protein 1 isoform X3	3195	0
XM_020093585.1	mitogen-activated protein kinase kinase kinase kinase 3 isoform X4	3473	0
XM_020093586.1	intraflagellar transport protein 81 homolog	2646	0
XM_020093587.1	PREDICTED: uncharacterized protein C9orf40 homolog	1455	1.05E-103
XM_020093588.1	cytochrome b ascorbate-dependent protein 3-like isoform X1	2456	0
XM_020093589.1	cytochrome b ascorbate-dependent protein 3-like isoform X1	2496	0
XM_020093590.1	PREDICTED: uncharacterized protein LOC109633594	3033	0
XM_020093591.1	WD repeat-containing protein 54	1448	0
XM_020093592.1	WD repeat-containing protein 54	1443	0
XM_020093593.1	phosphatidylinositol 4-phosphate 5-kinase-like protein 1	2243	0
XM_020093594.1	mitogen-activated protein kinase kinase kinase kinase 3 isoform X5	7228	0
XM_020093595.1	phosphatidylethanolamine-binding protein 4	2945	1.62E-127
XM_020093596.1	adhesion G protein-coupled receptor A2	5383	0
XM_020093597.1	PREDICTED: uncharacterized protein C9orf172 homolog	5342	0
XM_020093598.1	PREDICTED: uncharacterized protein KIAA0825 homolog	5146	0
XM_020093599.1	U3 small nucleolar ribonucleoprotein protein IMP4	1264	0
XM_020093600.1	beta-1 adrenergic receptor-like	3192	0
XM_020093601.1	mitogen-activated protein kinase kinase kinase kinase 3 isoform X6	7165	0
XM_020093602.1	rho-related BTB domain-containing protein 2 isoform X1	5702	0
XM_020093603.1	rho-related BTB domain-containing protein 2 isoform X2	5687	0
XM_020093604.1	rho-related BTB domain-containing protein 2 isoform X1	5499	0
XM_020093605.1	rho-related BTB domain-containing protein 2 isoform X4	2279	0
XM_020093606.1	rho-related BTB domain-containing protein 2 isoform X5	2132	0
XM_020093607.1	carnosine N-methyltransferase isoform X1	1733	0
XM_020093608.1	carnosine N-methyltransferase isoform X2	1730	0
XM_020093609.1	PREDICTED: uncharacterized protein LOC109633606	3409	0
XM_020093610.1	PREDICTED: uncharacterized protein LOC109633606	3404	0
XM_020093611.1	PREDICTED: uncharacterized protein LOC109633608	2607	0
XM_020093612.1	calcium-binding protein 1 isoform X1	2940	0
XM_020093613.1	calcium-binding protein 1 isoform X2	2338	1.01E-112
XM_020093614.1	probable E3 ubiquitin-protein ligase HERC1	2700	0
XM_020093615.1	THUMP domain-containing protein 3	1932	0
XM_020093616.1	T-box-containing protein TBX6L-like	1990	0
XM_020093617.1	filamin-interacting protein FAM101A-like	3467	1.72E-131
XM_020093618.1	neurofilament light polypeptide-like	2327	0
XM_020093619.1	2-oxoglutarate and iron-dependent oxygenase domain-containing protein 2	2375	0
XM_020093620.1	coxsackievirus and adenovirus receptor homolog	2926	0
XM_020093621.1	polypeptide N-acetylgalactosaminyltransferase 9-like	1572	7.09E-167
XM_020093622.1	polypeptide N-acetylgalactosaminyltransferase 9-like	1529	8.36E-178
XM_020093623.1	citron Rho-interacting kinase-like isoform X1	6313	0
XM_020093624.1	citron Rho-interacting kinase-like isoform X2	6301	0
XM_020093625.1	citron Rho-interacting kinase-like isoform X3	6289	0
XM_020093626.1	eukaryotic translation initiation factor 3 subunit A	4610	0
XM_020093627.1	citron Rho-interacting kinase-like isoform X4	6371	0
XM_020093628.1	citron Rho-interacting kinase-like isoform X5	5186	0
XM_020093629.1	citron Rho-interacting kinase-like isoform X6	6359	0
XM_020093630.1	adhesion G-protein coupled receptor D1 isoform X1	4994	0
XM_020093631.1	adhesion G-protein coupled receptor D1 isoform X2	4895	0
XM_020093632.1	dual specificity testis-specific protein kinase 1-like isoform X1	3142	0
XM_020093633.1	dual specificity testis-specific protein kinase 1-like isoform X1	3069	0
XM_020093634.1	neuropeptide Y receptor type 4-like	2055	0
XM_020093635.1	guanine deaminase	2637	0
XM_020093636.1	GDNF family receptor alpha-2-like isoform X1	3436	0
XM_020093637.1	GDNF family receptor alpha-2-like isoform X2	3295	0
XM_020093638.1	adenosine receptor A2b	1950	0
XM_020093639.1	sphingosine-1-phosphate lyase 1 isoform X1	4408	0
XM_020093640.1	protein FAM219A	2303	1.30E-89
XM_020093641.1	leucine-rich repeat-containing protein 75B-like	3100	0
XM_020093642.1	myocyte-specific enhancer factor 2C	2623	0
XM_020093643.1	PREDICTED: uncharacterized protein C2orf81 homolog	627	2.72E-111
XM_020093644.1	arrestin domain-containing protein 3-like	987	0

XM_020093645.1	COUP transcription factor 1	1895	0
XM_020093646.1	integrin alpha-6-like	3305	0
XM_020093647.1	sphingosine-1-phosphate lyase 1 isoform X2	4178	0
XM_020093648.1	protein FAM172A	1485	0
XM_020093649.1	multiple C2 and transmembrane domain-containing protein 1	1626	0
XM_020093650.1	multiple C2 and transmembrane domain-containing protein 1-like	708	4.04E-118
XM_020093651.1	protein FAM81B	1113	0
XM_020093652.1	glutamine--fructose-6-phosphate aminotransferase [isomerizing] 1-like	3848	0
XM_020093653.1	aspartate aminotransferase, cytoplasmic-like	1329	0
XM_020093654.1	type I inositol 1,4,5-trisphosphate 5-phosphatase-like	3504	0
XM_020093655.1	PREDICTED: uncharacterized protein LOC109633642	963	5.79E-177
XM_020093656.1	tetratricopeptide repeat protein 28-like	7640	0
XM_020093657.1	group 3 secretory phospholipase A2-like	1686	0
XM_020093658.1	pterin-4-alpha-carbinolamine dehydratase	902	1.40E-72
XM_020093659.1	serine/threonine-protein kinase pim-2-like	960	0
XM_020093660.1	transmembrane channel-like protein 1	2910	0
XM_020093661.1	transmembrane channel-like protein 2	2799	0
XM_020093662.1	cytochrome P450 1A1-like	1761	0
XM_020093663.1	major facilitator superfamily domain-containing protein 3	3159	0
XM_020093664.1	pre-B-cell leukemia transcription factor 3-like	2243	2.79E-118
XM_020093665.1	PREDICTED: uncharacterized protein LOC109633651	975	0
XM_020093666.1	potassium voltage-gated channel subfamily V member 2-like	1602	0
XM_020093667.1	cell division cycle-associated protein 2	4322	0
XM_020093668.1	kinesin light chain 1-like isoform X1	3245	0
XM_020093669.1	putative hexokinase HKDC1 isoform X1	3787	0
XM_020093670.1	mothers against decapentaplegic homolog 2-like	1606	0
XM_020093671.1	protein FAM216A	399	3.16E-89
XM_020093672.1	BICD family-like cargo adapter 1	4204	0
XM_020093673.1	eIF-2-alpha kinase activator GCN1	8213	0
XM_020093674.1	zinc metalloproteinase-disintegrin-like crotastatin	2136	0
XM_020093675.1	kinesin light chain 1-like isoform X2	3242	0
XM_020093676.1	serine protease HTRA1-like	1401	0
XM_020093677.1	T-box transcription factor TBX3-like	825	3.69E-160
XM_020093678.1	tenascin-like isoform X3	6584	0
XM_020093679.1	RAF proto-oncogene serine/threonine-protein kinase isoform X1	6649	0
XM_020093680.1	patatin-like phospholipase domain-containing protein 7	1471	1.08E-153
XM_020093681.1	retinal dehydrogenase 1	2510	0
XM_020093682.1	electrogenic sodium bicarbonate cotransporter 4-like isoform X1	3554	0
XM_020093683.1	kinesin light chain 1-like isoform X3	3236	0
XM_020093684.1	electrogenic sodium bicarbonate cotransporter 4-like isoform X1	3542	0
XM_020093685.1	DNA-directed RNA polymerase III subunit RPC4	1655	0
XM_020093686.1	general transcription factor IIH subunit 2-like isoform X1	1640	0
XM_020093687.1	general transcription factor IIH subunit 2-like isoform X1	1763	0
XM_020093688.1	general transcription factor IIH subunit 2-like isoform X1	1677	0
XM_020093689.1	general transcription factor IIH subunit 2-like isoform X1	1665	0
XM_020093690.1	cytochrome c oxidase subunit 6A, mitochondrial	628	5.81E-69
XM_020093691.1	PREDICTED: aquaporin-3-like	1830	0
XM_020093692.1	kinesin light chain 1-like isoform X4	3233	0
XM_020093693.1	indoleamine 2,3-dioxygenase 2-like	1439	0
XM_020093694.1	LIM homeobox transcription factor 1-alpha-like	1917	2.50E-135
XM_020093695.1	PREDICTED: alpha-L-iduronidase	2416	0
XM_020093696.1	immediate early response gene 5-like protein	1078	0
XM_020093697.1	syntaxin-2-like isoform X1	1158	0
XM_020093698.1	syntaxin-2-like isoform X1	1212	0
XM_020093699.1	syntaxin-2-like isoform X1	1680	0
XM_020093700.1	kinesin light chain 1-like isoform X5	4275	0
XM_020093701.1	dihydrofolate reductase	782	1.63E-142
XM_020093702.1	PREDICTED: uncharacterized protein LOC109633679 isoform X1	1190	0
XM_020093703.1	PREDICTED: uncharacterized protein LOC109633679 isoform X1	1170	0
XM_020093704.1	E3 ubiquitin-protein ligase RNF181	918	6.48E-114
XM_020093705.1	E3 ubiquitin-protein ligase RNF181	707	4.54E-115
XM_020093706.1	sodium-dependent phosphate transporter 1-B-like	2430	0
XM_020093707.1	cytochrome c oxidase subunit 7C, mitochondrial	446	1.42E-40
XM_020093708.1	kinesin light chain 1-like isoform X1	4248	0
XM_020093709.1	multivesicular body subunit 12B-like isoform X1	987	3.86E-169
XM_020093710.1	multivesicular body subunit 12B-like isoform X2	986	4.94E-170
XM_020093711.1	leucine zipper putative tumor suppressor 2 homolog	2898	0
XM_020093712.1	PREDICTED: aquaporin-9-like	998	0
XM_020093713.1	protein dispatched homolog 3 isoform X1	5298	0
XM_020093714.1	protein dispatched homolog 3 isoform X2	5295	0
XM_020093715.1	PREDICTED: uncharacterized protein LOC109633687	1998	0
XM_020093716.1	5-hydroxytryptamine receptor 7-like	2779	0
XM_020093717.1	immediate early response 3-interacting protein 1-like	558	9.65E-44
XM_020093718.1	rho GTPase-activating protein 25	2149	0
XM_020093719.1	cGMP-dependent protein kinase 1-like	2179	0
XM_020093720.1	serine/threonine-protein phosphatase 4 regulatory subunit 3B isoform X1	4694	0
XM_020093721.1	homeobox protein Nkx-3.1-like	884	3.61E-128
XM_020093722.1	stanniocalcin 2	834	0
XM_020093723.1	leukocyte surface antigen CD53-like	2199	5.31E-151

XM_020093724.1	transmembrane protein 132D-like	3869	0
XM_020093725.1	sushi domain-containing protein 1-like isoform X1	2036	0
XM_020093726.1	sushi domain-containing protein 1-like isoform X2	2939	0
XM_020093727.1	prolactin-releasing peptide receptor-like	1668	0
XM_020093728.1	serine/threonine-protein phosphatase 4 regulatory subunit 3B isoform X2	4604	0
XM_020093729.1	galactose-specific cell agglutination protein gsf2-like	1665	4.81E-125
XM_020093730.1	leucine-rich repeat transmembrane neuronal protein 4-like	2586	0
XM_020093731.1	gamma-glutamyltranspeptidase 1-like	1891	0
XM_020093732.1	E3 ubiquitin-protein ligase NEURL1-like	1110	0
XM_020093733.1	homeobox protein Nkx-6.1	2029	6.81E-172
XM_020093734.1	P2X purinoceptor 4	1274	0

XM_020093735.1	retinol-binding protein 4	754	2.99E-145
XM_020093736.1	collagen alpha-1(XVII) chain-like	6829	0
XM_020093737.1	early growth response protein 3	2024	0
XM_020093738.1	NMDA receptor synaptonuclear signaling and neuronal migration factor-like	2312	0
XM_020093739.1	AT-rich interactive domain-containing protein 5B	2033	0
XM_020093740.1	alpha-1A adrenergic receptor-like	1591	0
XM_020093741.1	neuropeptide FF receptor 1-like	1446	0
XM_020093742.1	radial spoke head protein 4 homolog A-like	1732	0
XM_020093743.1	PREDICTED: uncharacterized protein LOC109633717	1620	0
XM_020093744.1	collagen alpha-1(XVII) chain-like	6443	0
XM_020093745.1	ankyrin repeat domain-containing protein 34B-like	1871	0
XM_020093746.1	cholesterol 25-hydroxylase-like protein	1389	2.97E-178
XM_020093747.1	homeodomain-interacting protein kinase 1-like	1959	0
XM_020093748.1	PREDICTED: uncharacterized protein LOC109633721	777	3.50E-135
XM_020093749.1	bone morphogenetic protein 10	1461	0
XM_020093750.1	PREDICTED: uncharacterized protein C22orf15 homolog	816	2.85E-108
XM_020093751.1	cell cycle checkpoint control protein RAD9B	2442	0
XM_020093752.1	serine/threonine-protein phosphatase alpha-2 isoform-like	1948	0
XM_020093753.1	protein phosphatase PTC7 homolog	3417	0
XM_020093754.1	serine/threonine-protein kinase 10-like	2712	0
XM_020093755.1	ubiquitin-conjugating enzyme E2 G1-like	896	4.74E-124
XM_020093756.1	myosin regulatory light chain 2, ventricular/cardiac muscle isoform	715	1.78E-120
XM_020093757.1	probable ATP-dependent DNA helicase HFM1 isoform X1	7224	0
XM_020093758.1	leucine-rich repeat extensin-like protein 1	1233	1.84E-145
XM_020093759.1	probable cytosolic iron-sulfur protein assembly protein CIAO1	1434	0
XM_020093760.1	transmembrane protein 127	2391	3.11E-152
XM_020093761.1	transmembrane protein 127	2028	8.06E-154
XM_020093762.1	ES1 protein homolog, mitochondrial-like	878	0
XM_020093763.1	titin homolog isoform X1	6126	0
XM_020093764.1	titin homolog isoform X1	6054	0
XM_020093765.1	titin homolog isoform X1	5990	0
XM_020093766.1	titin homolog isoform X1	6040	0
XM_020093767.1	titin homolog isoform X1	5969	0
XM_020093768.1	ubiquitin carboxyl-terminal hydrolase 44-like	3940	0
XM_020093769.1	titin homolog isoform X1	5935	0
XM_020093770.1	titin homolog isoform X1	6124	0
XM_020093771.1	titin homolog isoform X1	6001	0
XM_020093772.1	RING finger protein 10 isoform X1	3836	0
XM_020093773.1	RING finger protein 10 isoform X2	3689	0
XM_020093774.1	beta-crystallin B3-like	1229	3.03E-155
XM_020093775.1	beta-crystallin B3-like	1254	3.48E-155
XM_020093776.1	beta-crystallin B1-like	772	1.07E-145
XM_020093777.1	anthrax toxin receptor 1-like	3532	0
XM_020093778.1	PREDICTED: uncharacterized protein C9orf78 homolog	1332	0
XM_020093779.1	NFU1 iron-sulfur cluster scaffold homolog, mitochondrial	1101	3.81E-177
XM_020093780.1	methionine aminopeptidase 2-like	2306	0
XM_020093781.1	mediator of RNA polymerase II transcription subunit 22 isoform X1	1056	2.56E-152
XM_020093782.1	mediator of RNA polymerase II transcription subunit 22 isoform X1	1042	9.95E-144
XM_020093783.1	mediator of RNA polymerase II transcription subunit 22 isoform X3	1626	7.14E-96
XM_020093784.1	usherin-like isoform X2	2648	6.69E-99
XM_020093785.1	usherin-like isoform X2	2509	4.94E-82
XM_020093786.1	sarcoplasmic/endoplasmic reticulum calcium ATPase 2-like isoform X1	6125	0
XM_020093787.1	sarcoplasmic/endoplasmic reticulum calcium ATPase 2-like isoform X2	4179	0
XM_020093788.1	anaphase-promoting complex subunit 7	3400	0
XM_020093789.1	golgin subfamily A member 3 isoform X1	6473	0
XM_020093790.1	golgin subfamily A member 3 isoform X1	6912	0
XM_020093791.1	golgin subfamily A member 3 isoform X1	6471	0
XM_020093792.1	golgin subfamily A member 3 isoform X1	6347	0
XM_020093793.1	ankyrin repeat and LEM domain-containing protein 2	3760	0
XM_020093794.1	ankyrin repeat and LEM domain-containing protein 2	3816	0
XM_020093795.1	ankyrin repeat and LEM domain-containing protein 2	3680	0
XM_020093796.1	serine/threonine-protein phosphatase PGAM5, mitochondrial isoform X1	1615	0
XM_020093797.1	serine/threonine-protein phosphatase PGAM5, mitochondrial isoform X2	1612	0
XM_020093798.1	serine/threonine-protein phosphatase PGAM5, mitochondrial isoform X3	1609	0
XM_020093799.1	serine/threonine-protein phosphatase PGAM5, mitochondrial isoform X4	1606	0
XM_020093800.1	transmembrane protein 2	5982	0
XM_020093801.1	protein ABHD17B	2359	0
XM_020093802.1	serine/threonine-protein phosphatase 2A 55 kDa regulatory subunit B delta isoform	2759	0

XM_020093803.1	PREDICTED: uncharacterized protein C9orf85 homolog	1167	4.97E-103
XM_020093804.1	rhotekin isoform X1	4568	0
XM_020093805.1	rhotekin isoform X2	2452	0
XM_020093806.1	rhotekin isoform X3	2637	0
XM_020093807.1	rhotekin isoform X4	2008	0
XM_020093808.1	rhotekin isoform X5	1973	0
XM_020093809.1	lysM and putative peptidoglycan-binding domain-containing protein 3	1843	0
XM_020093810.1	metallo-beta-lactamase domain-containing protein 2	1558	0
XM_020093811.1	DNA-directed RNA polymerase III subunit RPC7	2029	5.70E-120
XM_020093812.1	DNA-directed RNA polymerase III subunit RPC7	2009	4.68E-120
XM_020093813.1	DNA-directed RNA polymerase III subunit RPC7	1774	4.76E-121
XM_020093814.1	armadillo repeat protein deleted in velo-cardio-facial syndrome isoform X1	6119	0
XM_020093815.1	BCL2/adenovirus E1B 19 kDa protein-interacting protein 3-like	1299	1.33E-128
XM_020093816.1	armadillo repeat protein deleted in velo-cardio-facial syndrome isoform X2	6101	0
XM_020093817.1	armadillo repeat protein deleted in velo-cardio-facial syndrome isoform X3	6097	0
XM_020093818.1	armadillo repeat protein deleted in velo-cardio-facial syndrome isoform X4	6035	0
XM_020093819.1	armadillo repeat protein deleted in velo-cardio-facial syndrome isoform X5	6064	0
XM_020093820.1	armadillo repeat protein deleted in velo-cardio-facial syndrome isoform X6	6006	0
XM_020093821.1	armadillo repeat protein deleted in velo-cardio-facial syndrome isoform X7	5951	0
XM_020093822.1	armadillo repeat protein deleted in velo-cardio-facial syndrome isoform X8	6103	0
XM_020093823.1	armadillo repeat protein deleted in velo-cardio-facial syndrome isoform X1	5962	0
XM_020093824.1	thioredoxin reductase 2, mitochondrial	3835	0
XM_020093825.1	hepatocyte nuclear factor 1-alpha isoform X1	2727	0
XM_020093826.1	hepatocyte nuclear factor 1-alpha isoform X2	1683	0
XM_020093827.1	RNA-binding protein Musashi homolog 1 isoform X1	2138	0
XM_020093828.1	prosaposin isoform X1	2444	0
XM_020093829.1	RNA-binding protein Musashi homolog 1 isoform X1	2137	0
XM_020093830.1	RNA-binding protein Musashi homolog 1 isoform X1	2121	0
XM_020093831.1	5'-AMP-activated protein kinase subunit beta-1-like	1627	2.57E-175
XM_020093832.1	5'-AMP-activated protein kinase subunit beta-1-like	1851	3.44E-174
XM_020093833.1	hepatocyte nuclear factor 1-alpha isoform X2	1967	4.07E-108
XM_020093834.1	phospholipase A2	479	3.72E-103
XM_020093835.1	nuclear receptor corepressor 2 isoform X1	9900	0
XM_020093836.1	nuclear receptor corepressor 2 isoform X2	9897	0
XM_020093837.1	prosaposin isoform X2	2438	0
XM_020093838.1	nuclear receptor corepressor 2 isoform X3	9897	0
XM_020093839.1	nuclear receptor corepressor 2 isoform X4	9852	0
XM_020093840.1	nuclear receptor corepressor 2 isoform X5	9849	0
XM_020093841.1	serine/threonine-protein phosphatase 2B catalytic subunit gamma isoform-like isoform X1	4390	0
XM_020093842.1	serine/threonine-protein phosphatase 2B catalytic subunit gamma isoform-like isoform X2	4365	0
XM_020093843.1	vacuolar protein sorting-associated protein 37C	3257	2.06E-134
XM_020093844.1	angiopoietin-related protein 1	2983	0
XM_020093845.1	neurocalcin-delta A	2234	2.17E-131
XM_020093846.1	neurocalcin-delta A	2228	3.69E-132
XM_020093847.1	PREDICTED: hemicentin-1-like	13229	0
XM_020093848.1	equilibrative nucleoside transporter 1	2665	0
XM_020093849.1	leukocyte surface antigen CD53-like	7071	2.76E-125
XM_020093850.1	probable palmitoyltransferase ZDHHC8 isoform X1	6482	0
XM_020093851.1	probable palmitoyltransferase ZDHHC8 isoform X2	2567	0
XM_020093852.1	ran-specific GTPase-activating protein	1893	1.25E-130
XM_020093853.1	microprocessor complex subunit DGCR8	4753	0
XM_020093854.1	tRNA (uracil-5-)-methyltransferase homolog A	3365	0
XM_020093855.1	tRNA (uracil-5-)-methyltransferase homolog A	3362	0
XM_020093856.1	E1A-binding protein p400 isoform X1	9889	0
XM_020093857.1	E1A-binding protein p400 isoform X2	9542	0
XM_020093858.1	E1A-binding protein p400 isoform X3	9880	0
XM_020093859.1	E1A-binding protein p400 isoform X4	9874	0
XM_020093860.1	E1A-binding protein p400 isoform X5	9589	0
XM_020093861.1	E1A-binding protein p400 isoform X6	9580	0
XM_020093862.1	fibroblast growth factor receptor 2-like isoform X1	2625	0
XM_020093863.1	low molecular weight neuronal intermediate filament-like isoform X1	3394	0
XM_020093864.1	low molecular weight neuronal intermediate filament-like isoform X1	3262	0
XM_020093865.1	serine/threonine-protein kinase pim-2-like	1299	0
XM_020093866.1	max dimerization protein 1	5660	3.08E-102
XM_020093867.1	dynamin-1-like protein isoform X1	3800	0
XM_020093868.1	dynamin-1-like protein isoform X1	3798	0
XM_020093869.1	dynamin-1-like protein isoform X1	3524	0
XM_020093870.1	dynamin-1-like protein isoform X1	3760	0
XM_020093871.1	fibroblast growth factor receptor 2-like isoform X2	2383	0
XM_020093872.1	ubiquitin-60S ribosomal protein L40	1239	1.32E-99
XM_020093873.1	transient receptor potential cation channel subfamily M member 3-like isoform X1	9106	0
XM_020093874.1	transient receptor potential cation channel subfamily M member 3-like isoform X2	8977	0
XM_020093875.1	myotubularin-related protein 3 isoform X1	7966	0
XM_020093876.1	myotubularin-related protein 3 isoform X2	7939	0
XM_020093877.1	myotubularin-related protein 3 isoform X3	7850	0
XM_020093878.1	ras GTPase-activating protein 1	5400	0
XM_020093879.1	PREDICTED: cyclin-H	1597	0
XM_020093880.1	serine/threonine-protein phosphatase 2A 55 kDa regulatory subunit B alpha isoform	3855	0
XM_020093881.1	calcineurin-binding protein cabin-1 isoform X1	8813	0

XM_020093882.1	calcineurin-binding protein cabin-1 isoform X1	9052	0
XM_020093883.1	adenosine kinase-like isoform X1	2280	0
XM_020093884.1	calcineurin-binding protein cabin-1 isoform X1	8747	0
XM_020093885.1	calcineurin-binding protein cabin-1 isoform X1	8699	0
XM_020093886.1	calcineurin-binding protein cabin-1 isoform X4	8633	0
XM_020093887.1	nucleolysin TIA-1-like	2707	0
XM_020093888.1	cytokine-dependent hematopoietic cell linker	1273	0
XM_020093889.1	methylcytosine dioxygenase TET3 isoform X3	10996	0
XM_020093890.1	methylcytosine dioxygenase TET3 isoform X3	10936	0
XM_020093891.1	methylcytosine dioxygenase TET3 isoform X3	11028	0
XM_020093892.1	methylcytosine dioxygenase TET3 isoform X3	10993	0
XM_020093893.1	methylcytosine dioxygenase TET3 isoform X3	10696	0
XM_020093894.1	adenosine kinase-like isoform X2	2204	0
XM_020093895.1	probable E3 ubiquitin-protein ligase HECTD4 isoform X1	15102	0
XM_020093896.1	probable E3 ubiquitin-protein ligase HECTD4 isoform X2	15096	0
XM_020093897.1	rab5 GDP/GTP exchange factor-like	7498	0
XM_020093898.1	AP2-associated protein kinase 1-like isoform X1	5831	0
XM_020093899.1	AP2-associated protein kinase 1-like isoform X2	5740	0
XM_020093900.1	AP2-associated protein kinase 1-like isoform X3	5740	0
XM_020093901.1	AP2-associated protein kinase 1-like isoform X4	5638	0
XM_020093902.1	AP2-associated protein kinase 1-like isoform X1	4905	0
XM_020093903.1	AP2-associated protein kinase 1-like isoform X6	5572	0
XM_020093904.1	AP2-associated protein kinase 1-like isoform X7	5278	0
XM_020093905.1	AP2-associated protein kinase 1-like isoform X8	7092	0
XM_020093906.1	very low-density lipoprotein receptor-like isoform X1	4287	0
XM_020093907.1	very low-density lipoprotein receptor-like isoform X2	4278	0
XM_020093908.1	dual specificity phosphatase DUPD1	1696	3.93E-155
XM_020093909.1	very low-density lipoprotein receptor-like isoform X3	4278	0
XM_020093910.1	CUB and sushi domain-containing protein 3-like	3337	0
XM_020093911.1	E3 ubiquitin-protein ligase CHFR	5392	0
XM_020093912.1	E3 ubiquitin-protein ligase CHFR	5312	0
XM_020093913.1	cytospin-A isoform X1	6258	0
XM_020093914.1	cytospin-A isoform X1	6263	0
XM_020093915.1	cytospin-A isoform X1	6146	0
XM_020093916.1	cytospin-A isoform X1	6085	0
XM_020093917.1	cytospin-A isoform X1	6222	0
XM_020093918.1	dual specificity phosphatase DUPD1	1693	3.80E-155
XM_020093919.1	PREDICTED: malectin	4686	4.09E-164
XM_020093920.1	rho-related BTB domain-containing protein 2-like	5271	0
XM_020093921.1	rho-related BTB domain-containing protein 2-like	5406	0
XM_020093922.1	rho-related BTB domain-containing protein 2-like	5163	0
XM_020093923.1	ubiquitin-conjugating enzyme E2 D4	3448	2.93E-98
XM_020093924.1	bone morphogenetic protein 1-like isoform X2	4937	0
XM_020093925.1	bone morphogenetic protein 1-like isoform X2	4901	0
XM_020093926.1	solute carrier family 15 member 4	2105	0
XM_020093927.1	glycosyltransferase 1 domain-containing protein 1	1624	0
XM_020093928.1	tetratricopeptide repeat protein 37	5183	0
XM_020093929.1	arylsulfatase K	1737	0
XM_020093930.1	dual specificity phosphatase DUPD1-like	681	1.63E-126
XM_020093931.1	paxillin-like isoform X2	5751	0
XM_020093932.1	paxillin-like isoform X2	3715	0
XM_020093933.1	fibrosin-1-like protein isoform X1	4932	0
XM_020093934.1	fibrosin-1-like protein isoform X2	6047	0
XM_020093935.1	trans-Golgi network integral membrane protein 2	3331	0
XM_020093936.1	dual specificity protein phosphatase 18-like	810	1.14E-132
XM_020093937.1	protein unc-119 homolog B	3755	1.70E-174
XM_020093938.1	ribonuclease P/MRP protein subunit POP5	1300	2.31E-93
XM_020093939.1	G-protein coupled receptor 98	21153	0
XM_020093940.1	ras-related protein Rab-35	3207	7.26E-133
XM_020093941.1	dual specificity phosphatase DUPD1-like	854	8.00E-119
XM_020093942.1	nucleolar protein 6	4069	0
XM_020093943.1	zinc finger BED domain-containing protein 1-like isoform X1	2981	0
XM_020093944.1	zinc finger BED domain-containing protein 1-like isoform X1	1960	0
XM_020093945.1	tankyrase-1 isoform X1	4707	0
XM_020093946.1	tankyrase-1 isoform X2	4013	0
XM_020093947.1	AN1-type zinc finger protein 5 isoform X2	2496	2.34E-119
XM_020093948.1	AN1-type zinc finger protein 5 isoform X2	915	1.08E-122
XM_020093949.1	AN1-type zinc finger protein 5 isoform X2	992	1.12E-121
XM_020093950.1	AN1-type zinc finger protein 5 isoform X2	649	1.42E-123
XM_020093951.1	nuclear receptor ROR-beta	2790	0
XM_020093952.1	transient receptor potential cation channel subfamily M member 6-like	7246	0
XM_020093953.1	fidgetin-like protein 1	3438	0
XM_020093954.1	dedicator of cytokinesis protein 1	7463	0
XM_020093955.1	rab11 family-interacting protein 1-like isoform X1	5941	0
XM_020093956.1	rab11 family-interacting protein 1-like isoform X1	4060	0
XM_020093957.1	dihydropyrimidinase-related protein 2-like	4147	0
XM_020093958.1	phosphatidate cytidylyltransferase 2-like	4669	0
XM_020093959.1	VIP36-like protein	4186	0
XM_020093960.1	T-box transcription factor TBX1 isoform X1	2612	0

XM_020093961.1	fidgetin-like protein 1	3200	0
XM_020093962.1	T-box transcription factor TBX1 isoform X2	2561	0
XM_020093963.1	guanine nucleotide-binding protein subunit beta-like protein 1	3393	0
XM_020093964.1	guanine nucleotide-binding protein subunit beta-like protein 1	3447	0
XM_020093965.1	guanine nucleotide-binding protein subunit beta-like protein 1	3425	0
XM_020093966.1	guanine nucleotide-binding protein subunit beta-like protein 1	3366	0
XM_020093967.1	guanine nucleotide-binding protein subunit beta-like protein 1	3385	0
XM_020093968.1	netrin receptor UNC5D isoform X1	4184	0
XM_020093969.1	netrin receptor UNC5D isoform X1	4060	0
XM_020093970.1	netrin receptor UNC5D isoform X1	4151	0
XM_020093971.1	RIMS-binding protein 2 isoform X1	6677	0
XM_020093972.1	RIMS-binding protein 2 isoform X2	6671	0
XM_020093973.1	RIMS-binding protein 2 isoform X3	4734	0
XM_020093974.1	RIMS-binding protein 2 isoform X4	4524	0
XM_020093975.1	RIMS-binding protein 2 isoform X5	4398	0
XM_020093976.1	tubulin--tyrosine ligase	5093	0
XM_020093977.1	RIMS-binding protein 2 isoform X6	3898	0
XM_020093978.1	RIMS-binding protein 2 isoform X6	4050	0
XM_020093979.1	RIMS-binding protein 2 isoform X8	4377	0
XM_020093980.1	RIMS-binding protein 2 isoform X9	4356	0
XM_020093981.1	RIMS-binding protein 2 isoform X10	4305	0
XM_020093982.1	RIMS-binding protein 2 isoform X11	4503	0
XM_020093983.1	beta-adducin isoform X1	2817	0
XM_020093984.1	beta-adducin isoform X2	2751	0
XM_020093985.1	beta-adducin isoform X3	2733	0
XM_020093986.1	factor in the germline alpha	887	6.34E-151
XM_020093987.1	U4/U6.U5 small nuclear ribonucleoprotein 27 kDa protein	898	5.32E-45
XM_020093988.1	breakpoint cluster region protein	5777	0
XM_020093989.1	insulin-degrading enzyme isoform X1	5121	0
XM_020093990.1	PREDICTED: erlin-2	1338	0
XM_020093991.1	rab-like protein 6 isoform X1	6062	4.52E-177
XM_020093992.1	rab-like protein 6 isoform X2	6032	1.01E-169
XM_020093993.1	arrestin domain-containing protein 3	3055	0
XM_020093994.1	E3 ubiquitin-protein ligase DTX1 isoform X1	6910	0
XM_020093995.1	E3 ubiquitin-protein ligase DTX1 isoform X2	6901	0
XM_020093996.1	E3 ubiquitin-protein ligase DTX1 isoform X3	6880	0
XM_020093997.1	glycerol-3-phosphate acyltransferase 4	3223	0
XM_020093998.1	insulin-degrading enzyme isoform X2	5121	0
XM_020093999.1	glycerol-3-phosphate acyltransferase 4	3173	0
XM_020094000.1	beta,beta-carotene 9',10'-oxygenase-like isoform X1	4218	0
XM_020094001.1	beta,beta-carotene 9',10'-oxygenase-like isoform X1	4173	0
XM_020094002.1	elastase-like serine protease	999	0
XM_020094003.1	protein turtle homolog A-like isoform X1	8030	0
XM_020094004.1	protein turtle homolog A-like isoform X1	7818	0
XM_020094005.1	KAT8 regulatory NSL complex subunit 3 isoform X1	3404	0
XM_020094006.1	KAT8 regulatory NSL complex subunit 3 isoform X2	2695	0
XM_020094007.1	KAT8 regulatory NSL complex subunit 3 isoform X3	3589	0
XM_020094008.1	zinc finger and BTB domain-containing protein 34-like isoform X1	6876	0
XM_020094009.1	zinc finger and BTB domain-containing protein 34-like isoform X1	6800	0
XM_020094010.1	drebrin-like protein isoform X1	2719	0
XM_020094011.1	protein lunapark-B-like isoform X1	2976	0
XM_020094012.1	drebrin-like protein isoform X2	2602	0
XM_020094013.1	phytanoyl-CoA hydroxylase-interacting protein-like	1732	0
XM_020094014.1	protein phosphatase 1 regulatory subunit 3C-like isoform X1	2222	0
XM_020094015.1	protein phosphatase 1 regulatory subunit 3C-like isoform X1	1655	0
XM_020094016.1	protein phosphatase 1 regulatory subunit 3C-like isoform X1	1551	0
XM_020094017.1	DNA replication complex GINS protein SLD5	1887	3.80E-159
XM_020094018.1	A disintegrin and metalloproteinase with thrombospondin motifs 13 isoform X1	4262	0
XM_020094019.1	A disintegrin and metalloproteinase with thrombospondin motifs 13 isoform X1	4258	0
XM_020094020.1	N-lysine methyltransferase SMYD2-A-like	3105	0
XM_020094021.1	protein lunapark-B-like isoform X1	2979	0
XM_020094022.1	carnitine O-acetyltransferase-like	2315	0
XM_020094023.1	rasGAP-activating-like protein 1	4204	0
XM_020094024.1	protein DGCR6-like	1716	6.81E-123
XM_020094025.1	transmembrane protein 161B	4268	0
XM_020094026.1	stromal cell-derived factor 2-like protein 1	2051	2.95E-137
XM_020094027.1	PREDICTED: beta-ureidopropionase	1479	0
XM_020094028.1	protein lunapark-B-like isoform X1	2958	0
XM_020094029.1	protein GUCD1	2380	1.37E-163
XM_020094030.1	BCL2/adenovirus E1B 19 kDa protein-interacting protein 3-like	1546	7.78E-176
XM_020094031.1	long-chain-fatty-acid--CoA ligase 1-like isoform X3	3030	0
XM_020094032.1	vesicle-associated membrane protein 8-like	2392	3.35E-51
XM_020094033.1	bridging integrator 3	2369	7.35E-180
XM_020094034.1	ectonucleoside triphosphate diphosphohydrolase 4 isoform X1	3173	0
XM_020094035.1	ectonucleoside triphosphate diphosphohydrolase 4 isoform X2	3153	0
XM_020094036.1	ectonucleoside triphosphate diphosphohydrolase 4 isoform X3	3150	0
XM_020094037.1	protein lunapark-B-like isoform X1	2910	0
XM_020094038.1	ectonucleoside triphosphate diphosphohydrolase 4 isoform X4	3134	0
XM_020094039.1	aspartate beta-hydroxylase domain-containing protein 2	4217	0

XM_020094040.1	aspartate beta-hydroxylase domain-containing protein 2	4022	0
XM_020094041.1	aspartate beta-hydroxylase domain-containing protein 2	4213	0
XM_020094042.1	aspartate beta-hydroxylase domain-containing protein 2	3938	0
XM_020094043.1	aspartate beta-hydroxylase domain-containing protein 2	4068	0
XM_020094044.1	transcription factor 7-like 1-A isoform X1	2725	0
XM_020094045.1	transcription factor 7-like 1-A isoform X2	2722	0
XM_020094046.1	protein lunapark-B-like isoform X1	2886	0
XM_020094047.1	AP-3 complex subunit mu-2	3103	0
XM_020094048.1	zinc finger protein 703-like	2944	0
XM_020094049.1	anaphase-promoting complex subunit 5	3595	0
XM_020094050.1	kelch-like protein 22 isoform X1	4513	0
XM_020094051.1	kelch-like protein 22 isoform X2	4501	0
XM_020094052.1	TM2 domain-containing protein 2	3687	1.05E-130
XM_020094053.1	PREDICTED: mitoferrin-1-like	3604	0
XM_020094054.1	drebrin-like protein	3476	0
XM_020094055.1	multiple C2 and transmembrane domain-containing protein 1-like	4573	2.52E-76
XM_020094056.1	semaphorin-4G-like isoform X1	6138	0
XM_020094057.1	secreted frizzled-related protein 1	2306	0
XM_020094058.1	neuronal acetylcholine receptor subunit beta-2-like isoform X2	2055	2.32E-169
XM_020094059.1	annexin A2-A-like	1575	0
XM_020094060.1	ATPase family AAA domain-containing protein 1-A-like	3948	0
XM_020094061.1	homeobox protein cut-like 2 isoform X1	5341	0
XM_020094062.1	homeobox protein cut-like 2 isoform X2	5338	0
XM_020094063.1	homeobox protein cut-like 2 isoform X3	5320	0
XM_020094064.1	homeobox protein cut-like 2 isoform X4	5327	0
XM_020094065.1	homeobox protein cut-like 2 isoform X5	5341	0
XM_020094066.1	protein FAM151A isoform X1	2719	0
XM_020094067.1	golgin subfamily A member 7	2404	2.48E-96
XM_020094068.1	survival motor neuron protein 1-like	1101	1.02E-138
XM_020094069.1	protein O-GlcNAcase isoform X1	5781	0
XM_020094070.1	intraflagellar transport protein 25 homolog	503	6.65E-96
XM_020094071.1	synapsin-1-like isoform X1	2600	0
XM_020094072.1	synapsin-1-like isoform X1	5416	0
XM_020094073.1	rabphilin-3A isoform X1	4451	0
XM_020094074.1	rabphilin-3A isoform X1	4524	0
XM_020094075.1	rabphilin-3A isoform X1	4440	0
XM_020094076.1	Hermansky-Pudlak syndrome 4 protein isoform X1	3556	0
XM_020094077.1	Hermansky-Pudlak syndrome 4 protein isoform X2	3439	0
XM_020094078.1	protein O-GlcNAcase isoform X2	5727	0
XM_020094079.1	PREDICTED: uncharacterized protein LOC109633903	1601	0
XM_020094080.1	vinexin-like isoform X1	3693	0
XM_020094081.1	vinexin-like isoform X2	3660	0
XM_020094082.1	vinexin-like isoform X3	3657	0
XM_020094083.1	sorbin and SH3 domain-containing protein 2-like isoform X4	3584	0
XM_020094084.1	sorbin and SH3 domain-containing protein 2-like isoform X5	3549	0
XM_020094085.1	sorbin and SH3 domain-containing protein 2-like isoform X6	3547	0
XM_020094086.1	ankyrin repeat and SOCS box protein 6	2502	0
XM_020094087.1	ankyrin repeat and SOCS box protein 6	2509	0
XM_020094088.1	ankyrin repeat and SOCS box protein 6	2510	0
XM_020094089.1	ankyrin repeat and SOCS box protein 6	2511	0
XM_020094090.1	piwi-like protein 1	3364	0
XM_020094091.1	piwi-like protein 1	3391	0
XM_020094092.1	cytochrome c oxidase assembly protein COX15 homolog	2063	0
XM_020094093.1	piwi-like protein 1	3158	0
XM_020094094.1	piwi-like protein 1	4012	0
XM_020094095.1	transmembrane protein 132C isoform X1	4151	0
XM_020094096.1	transmembrane protein 132C isoform X2	4205	0
XM_020094097.1	transmembrane protein 132C isoform X3	4280	0
XM_020094098.1	transmembrane protein 132C isoform X4	4280	0
XM_020094099.1	transmembrane protein 132C isoform X5	3943	0
XM_020094100.1	transmembrane protein 132C isoform X6	4268	0
XM_020094101.1	transmembrane protein 132C isoform X7	4265	0
XM_020094102.1	tumor necrosis factor receptor superfamily member 10B-like isoform X1	2730	0
XM_020094103.1	tumor necrosis factor receptor superfamily member 22-like isoform X2	1670	0
XM_020094104.1	TNF receptor-associated factor 2-like isoform X1	3904	0
XM_020094105.1	TNF receptor-associated factor 2-like isoform X2	3829	0
XM_020094106.1	copper homeostasis protein cutC homolog	1309	0
XM_020094107.1	TNF receptor-associated factor 2-like isoform X3	3754	0
XM_020094108.1	acid-sensing ion channel 2 isoform X2	4824	0
XM_020094109.1	acid-sensing ion channel 2 isoform X2	3529	0
XM_020094110.1	Kv channel-interacting protein 2 isoform X1	2994	9.25E-162
XM_020094111.1	acid-sensing ion channel 2 isoform X2	3655	0
XM_020094112.1	acid-sensing ion channel 2 isoform X2	3343	0
XM_020094113.1	cytochrome c oxidase subunit NDUFA4	614	3.38E-56
XM_020094114.1	phospholipid phosphatase 3-like	2758	0
XM_020094115.1	migration and invasion enhancer 1-like	1398	8.36E-61
XM_020094116.1	coiled-coil domain-containing protein 137	1996	1.00E-127
XM_020094117.1	CD2 antigen cytoplasmic tail-binding protein 2	1445	0
XM_020094118.1	Kv channel-interacting protein 2 isoform X2	2767	2.92E-161

XM_020094119.1	sphingosine 1-phosphate receptor 2-like	3836	0
XM_020094120.1	heparan sulfate glucosamine 3-O-sulfotransferase 3B1-like	2599	0
XM_020094121.1	ER membrane protein complex subunit 10 isoform X1	1028	2.13E-180
XM_020094122.1	ER membrane protein complex subunit 10 isoform X2	1126	0
XM_020094123.1	PREDICTED: synaptotagmin-3	2290	0
XM_020094124.1	PREDICTED: synaptotagmin-3	2228	0
XM_020094125.1	zinc phosphodiesterase ELAC protein 2	2479	0
XM_020094126.1	ras-interacting protein 1-like isoform X1	5435	0
XM_020094127.1	Kv channel-interacting protein 2 isoform X3	2896	4.21E-155
XM_020094128.1	ras-associating and dilute domain-containing protein-like isoform X2	5321	0
XM_020094129.1	band 3 anion exchange protein-like	3947	0
XM_020094130.1	band 3 anion exchange protein-like	3928	0
XM_020094131.1	peripheral myelin protein 22	1573	3.96E-89
XM_020094132.1	mitogen-activated protein kinase kinase kinase 14-like isoform X1	3026	0
XM_020094133.1	mitogen-activated protein kinase kinase kinase 14-like isoform X1	2931	0
XM_020094134.1	ubiquitin domain-containing protein UBFD1 isoform X1	1449	0
XM_020094135.1	ubiquitin domain-containing protein UBFD1 isoform X1	1388	0
XM_020094136.1	Kv channel-interacting protein 2 isoform X4	2999	0
XM_020094137.1	ubiquitin domain-containing protein UBFD1 isoform X1	1334	0
XM_020094138.1	ubiquitin domain-containing protein UBFD1 isoform X1	1292	1.99E-180
XM_020094139.1	ubiquitin domain-containing protein UBFD1 isoform X1	1407	8.13E-180
XM_020094140.1	ubiquitin domain-containing protein UBFD1 isoform X1	1184	0
XM_020094141.1	transmembrane protein 255A	837	4.49E-55
XM_020094142.1	myosin light chain kinase, smooth muscle-like isoform X1	3721	0
XM_020094143.1	myosin light chain kinase, smooth muscle-like isoform X1	2811	0
XM_020094144.1	39S ribosomal protein L12, mitochondrial	1232	6.27E-96
XM_020094145.1	MAGUK p55 subfamily member 3 isoform X1	3952	0
XM_020094146.1	MAGUK p55 subfamily member 3 isoform X2	3910	0
XM_020094147.1	MAGUK p55 subfamily member 3 isoform X3	3886	0
XM_020094148.1	MAGUK p55 subfamily member 3 isoform X4	3844	0
XM_020094149.1	metalloproteinase inhibitor 2-like	2160	5.84E-158
XM_020094150.1	suppressor of cytokine signaling 3	1944	1.21E-148
XM_020094151.1	excitatory amino acid transporter 2-like	4297	0
XM_020094152.1	protein tweety homolog 2-like	3443	0
XM_020094153.1	protein crumbs homolog 3	2098	2.08E-60
XM_020094154.1	PREDICTED: neurexophilin-1-like	5803	6.61E-179
XM_020094155.1	WAS/WASL-interacting protein family member 3-like isoform X1	964	1.83E-20
XM_020094156.1	cytochrome P450 3A56-like	1820	0
XM_020094157.1	PREDICTED: uncharacterized protein LOC109633955 isoform X2	815	1.03E-67
XM_020094158.1	polymerase I and transcript release factor-like	3865	0
XM_020094159.1	growth arrest-specific protein 7-like isoform X1	2566	0
XM_020094160.1	growth arrest-specific protein 7-like isoform X2	2245	0
XM_020094161.1	growth arrest-specific protein 7-like isoform X3	2300	0
XM_020094162.1	zinc-binding protein A33-like	2628	0
XM_020094163.1	myosin phosphatase Rho-interacting protein-like isoform X1	5178	0
XM_020094164.1	myosin phosphatase Rho-interacting protein-like isoform X1	5175	0
XM_020094165.1	germ cell-specific gene 1-like protein	1714	0
XM_020094166.1	putative tRNA (cytidine(32)/guanosine(34)-2'-O)-methyltransferase	2700	2.79E-159
XM_020094167.1	desumoylating isopeptidase 2	5458	1.40E-107
XM_020094168.1	THAP domain-containing protein 5 isoform X1	2762	0
XM_020094169.1	THAP domain-containing protein 5 isoform X1	2737	0
XM_020094170.1	glucagon receptor-like isoform X1	2447	0
XM_020094171.1	glucagon receptor-like isoform X2	2405	0
XM_020094172.1	glucagon receptor-like isoform X3	2375	0
XM_020094173.1	protein HID1-like	3147	0
XM_020094174.1	protein sidekick-2 isoform X1	7973	0
XM_020094175.1	protein sidekick-2 isoform X2	7949	0
XM_020094176.1	ubiquitin carboxyl-terminal hydrolase 31	5525	0
XM_020094177.1	PREDICTED: perforin-1-like	1984	0
XM_020094178.1	AN1-type zinc finger protein 2A isoform X1	1356	2.26E-165
XM_020094179.1	AN1-type zinc finger protein 2A isoform X2	1412	1.26E-166
XM_020094180.1	acidic repeat-containing protein-like isoform X1	2240	0
XM_020094181.1	integral membrane protein GPR155	5170	0
XM_020094182.1	tripartite motif-containing protein 16-like isoform X1	1753	0
XM_020094183.1	tripartite motif-containing protein 16-like isoform X2	1750	0
XM_020094184.1	protein amnionless	2000	0
XM_020094185.1	speckle-type POZ protein-like	3189	0
XM_020094186.1	speckle-type POZ protein-like	3054	0
XM_020094187.1	speckle-type POZ protein-like	3053	0
XM_020094188.1	speckle-type POZ protein-like	2917	0
XM_020094189.1	voltage-dependent calcium channel gamma-2 subunit	3901	0
XM_020094190.1	integral membrane protein GPR155	2722	0
XM_020094191.1	rho GTPase-activating protein 44-like	3245	0
XM_020094192.1	C1q-related factor-like	2846	4.21E-131
XM_020094193.1	glutamate receptor ionotropic, NMDA 2C-like	5880	0
XM_020094194.1	tumor necrosis factor receptor superfamily member 16-like	3781	0
XM_020094195.1	receptor activity-modifying protein 2 isoform X1	2189	8.38E-116
XM_020094196.1	receptor activity-modifying protein 2 isoform X2	2187	4.45E-115
XM_020094197.1	receptor activity-modifying protein 2 isoform X3	2286	8.70E-111

XM_020094198.1	serine/threonine-protein kinase SBK1-like	2700	0
XM_020094199.1	protein ELFN1-like	2544	0
XM_020094200.1	WAS/WASL-interacting protein family member 2-like	3837	1.27E-56
XM_020094201.1	tetratricopeptide repeat protein 30A-like	1402	0
XM_020094202.1	5-hydroxytryptamine receptor 3A-like	1329	0
XM_020094203.1	non-structural maintenance of chromosomes element 4 homolog A-like	1029	0
XM_020094204.1	dual specificity mitogen-activated protein kinase kinase 4-like	1449	0
XM_020094205.1	myosin heavy chain, fast skeletal muscle-like	5817	0
XM_020094206.1	splicing factor U2AF 65 kDa subunit-like	1812	0
XM_020094207.1	G1/S-specific cyclin-D3-like	644	8.36E-118
XM_020094208.1	PREDICTED: spidroin-1-like	2366	0
XM_020094209.1	mitogen-activated protein kinase kinase kinase 3	5483	0
XM_020094210.1	GTPase IMAP family member 8-like	2927	0
XM_020094211.1	homeobox protein Meis1	3891	0
XM_020094212.1	protein HEG-like	769	2.18E-120
XM_020094213.1	cell wall protein DAN4-like	1084	1.78E-20
XM_020094214.1	putative all-trans-retinol 13,14-reductase	2511	0
XM_020094215.1	synaptic vesicle membrane protein VAT-1 homolog	2905	2.68E-151
XM_020094216.1	rho-related GTP-binding protein RhoN-like	1652	4.90E-129
XM_020094217.1	C-type mannose receptor 2	2036	0
XM_020094218.1	transmembrane protein 184A	2943	0
XM_020094219.1	homeobox protein Meis1	3895	0
XM_020094220.1	RING finger protein unkempt homolog	2728	0
XM_020094221.1	nitrogen permease regulator 3-like protein	1910	0
XM_020094222.1	hemoglobin subunit beta-like	342	1.18E-80
XM_020094223.1	hemoglobin embryonic subunit alpha-like	715	1.70E-105
XM_020094224.1	intercellular adhesion molecule 1	4856	0
XM_020094225.1	syntaxin-binding protein 1-like	2216	0
XM_020094226.1	GEM-interacting protein-like	3781	0

XM_020094227.1	zinc finger protein 436-like	3101	0
XM_020094228.1	prostaglandin E2 receptor EP1 subtype-like	1080	0
XM_020094229.1	PREDICTED: uncharacterized protein LOC109634015	1120	0
XM_020094230.1	potassium channel subfamily T member 2-like	3012	0
XM_020094231.1	G-protein coupled receptor 183-like	972	0
XM_020094232.1	GTPase IMAP family member 5-like	1355	0
XM_020094233.1	5-hydroxytryptamine receptor 3A-like	1230	0
XM_020094234.1	rho GTPase-activating protein 23 isoform X1	8538	0
XM_020094235.1	rho GTPase-activating protein 23 isoform X2	5502	0
XM_020094236.1	phosphatidylinositide phosphatase SAC2 isoform X1	6393	0
XM_020094237.1	rho GTPase-activating protein 23 isoform X3	8499	0
XM_020094238.1	rho GTPase-activating protein 23 isoform X4	8382	0
XM_020094239.1	rho GTPase-activating protein 23 isoform X5	8079	0
XM_020094240.1	rho GTPase-activating protein 23 isoform X6	8343	0
XM_020094241.1	rho GTPase-activating protein 23 isoform X7	7898	0
XM_020094242.1	rho GTPase-activating protein 23 isoform X8	8256	0
XM_020094243.1	rho GTPase-activating protein 23 isoform X8	7524	0
XM_020094244.1	supervillin-like isoform X1	5372	0
XM_020094245.1	supervillin-like isoform X2	5309	0
XM_020094246.1	supervillin-like isoform X3	5216	0
XM_020094247.1	phosphatidylinositide phosphatase SAC2 isoform X2	6375	0
XM_020094248.1	cyclin-dependent kinase 12 isoform X1	6219	0
XM_020094249.1	cyclin-dependent kinase 12 isoform X2	6207	0
XM_020094250.1	cyclin-dependent kinase 12 isoform X3	5796	0
XM_020094251.1	cyclin-dependent kinase 12 isoform X4	5784	0
XM_020094252.1	potassium voltage-gated channel subfamily H member 4-like	3910	0
XM_020094253.1	E3 ubiquitin-protein ligase DTX3L-like	3718	0
XM_020094254.1	ATP-citrate synthase	4860	0
XM_020094255.1	ATP-citrate synthase	3419	0
XM_020094256.1	116 kDa U5 small nuclear ribonucleoprotein component	3492	0
XM_020094257.1	zinc finger protein 281-like isoform X1	4970	0
XM_020094258.1	zinc finger protein 281-like isoform X1	5039	0
XM_020094259.1	chloride intracellular channel protein 2-like	1164	7.45E-121
XM_020094260.1	BAG family molecular chaperone regulator 3	3224	0
XM_020094261.1	disintegrin and metalloproteinase domain-containing protein 11-like isoform X1	5059	0
XM_020094262.1	disintegrin and metalloproteinase domain-containing protein 11-like isoform X2	3207	0
XM_020094263.1	disintegrin and metalloproteinase domain-containing protein 11-like isoform X3	5038	0
XM_020094264.1	disintegrin and metalloproteinase domain-containing protein 11-like isoform X4	5150	0
XM_020094265.1	disintegrin and metalloproteinase domain-containing protein 11-like isoform X5	5129	0
XM_020094266.1	disintegrin and metalloproteinase domain-containing protein 11-like isoform X6	3283	0
XM_020094267.1	integrin beta-3-like	4040	0
XM_020094268.1	histone acetyltransferase KAT2A	3296	0
XM_020094269.1	1-phosphatidylinositol 4,5-bisphosphate phosphodiesterase delta-3	3840	0
XM_020094270.1	2',3'-cyclic-nucleotide 3'-phosphodiesterase	3263	0
XM_020094271.1	2',3'-cyclic-nucleotide 3'-phosphodiesterase	3460	0
XM_020094272.1	kelch-like protein 11	3624	0
XM_020094273.1	putative ZDHHC-type palmitoyltransferase 6	2289	0
XM_020094274.1	serine/threonine-protein kinase tousled-like 2 isoform X1	3922	0
XM_020094275.1	serine/threonine-protein kinase tousled-like 2 isoform X2	3822	0
XM_020094276.1	zinc finger protein 652	3523	0

XM_020094277.1	zinc finger protein 652	3295	0
XM_020094278.1	zinc finger protein 652	3321	0
XM_020094279.1	zinc finger protein 652	3301	0
XM_020094280.1	probable ATP-dependent RNA helicase DHX58	3332	0
XM_020094281.1	voltage-dependent L-type calcium channel subunit beta-1 isoform X1	3757	0
XM_020094282.1	voltage-dependent L-type calcium channel subunit beta-1 isoform X2	3748	0
XM_020094283.1	voltage-dependent L-type calcium channel subunit beta-1 isoform X3	3625	0
XM_020094284.1	voltage-dependent L-type calcium channel subunit beta-1 isoform X4	3616	0
XM_020094285.1	voltage-dependent L-type calcium channel subunit beta-1 isoform X5	3771	0
XM_020094286.1	cadherin-4-like isoform X2	4166	0
XM_020094287.1	zinc finger protein 385C isoform X1	5088	0
XM_020094288.1	zinc finger protein 385C isoform X1	5181	0
XM_020094289.1	zinc finger protein 385C isoform X1	5075	0
XM_020094290.1	zinc finger protein 385C isoform X1	5058	0
XM_020094291.1	zinc finger protein 385C isoform X1	4989	0
XM_020094292.1	zinc finger protein 385C isoform X1	4989	0
XM_020094293.1	zinc finger protein 385C isoform X1	4852	0
XM_020094294.1	peptidyl-prolyl cis-trans isomerase FKBP10-like	2310	0
XM_020094295.1	G protein-activated inward rectifier potassium channel 1-like	2220	0
XM_020094296.1	dnaJ homolog subfamily C member 7	2818	0
XM_020094297.1	tetratricopeptide repeat protein 25 isoform X1	2776	0
XM_020094298.1	tetratricopeptide repeat protein 25 isoform X2	2773	0
XM_020094299.1	plexin domain-containing protein 1-like isoform X1	1969	0
XM_020094300.1	leucine-rich repeat transmembrane protein FLRT1-like	4945	0
XM_020094301.1	plexin domain-containing protein 1-like isoform X1	1938	0
XM_020094302.1	glycylpeptide N-tetradecanoyltransferase 1	3793	0
XM_020094303.1	gap junction gamma-1 protein-like	1925	0
XM_020094304.1	gap junction gamma-1 protein-like	1922	0
XM_020094305.1	gap junction gamma-1 protein-like	1959	0
XM_020094306.1	gap junction gamma-1 protein-like	1780	0
XM_020094307.1	stAR-related lipid transfer protein 3 isoform X1	3688	0
XM_020094308.1	stAR-related lipid transfer protein 3 isoform X1	3888	0

XM_020094309.1	stAR-related lipid transfer protein 3 isoform X1	3556	0
XM_020094310.1	F-box/LRR-repeat protein 20	4380	0
XM_020094311.1	synaptonemal complex protein SC65	1769	0
XM_020094312.1	O-acetyl-ADP-ribose deacetylase MACROD2 isoform X1	1504	0
XM_020094313.1	P43 5S RNA-binding protein-like isoform X1	1506	0
XM_020094314.1	P43 5S RNA-binding protein-like isoform X2	1474	0
XM_020094315.1	P43 5S RNA-binding protein-like isoform X3	1410	0
XM_020094316.1	methyltransferase-like protein 2-A	1630	0
XM_020094317.1	protein HEXIM1	1921	0
XM_020094318.1	alpha-2,8-sialyltransferase 8F-like isoform X1	1813	0
XM_020094319.1	alpha-2,8-sialyltransferase 8F-like isoform X2	2388	0
XM_020094320.1	SH3 and cysteine-rich domain-containing protein 2-like	2787	4.78E-179
XM_020094321.1	O-acetyl-ADP-ribose deacetylase MACROD2 isoform X2	2722	0
XM_020094322.1	SH3 and cysteine-rich domain-containing protein 2-like	2972	0
XM_020094323.1	cyclin-dependent kinase 4-like	1663	0
XM_020094324.1	prohibitin isoform X1	1682	0
XM_020094325.1	prohibitin isoform X1	1664	3.63E-177
XM_020094326.1	cytosolic 5'-nucleotidase 3A isoform X2	2201	0
XM_020094327.1	phosphoethanolamine/phosphocholine phosphatase isoform X1	1842	0
XM_020094328.1	phosphoethanolamine/phosphocholine phosphatase isoform X1	1863	0
XM_020094329.1	phosphoethanolamine/phosphocholine phosphatase isoform X1	1696	0
XM_020094330.1	speriolin-like protein	1251	2.78E-173
XM_020094331.1	PREDICTED: melanoregulin-like	1808	1.19E-145
XM_020094332.1	LIM and SH3 domain protein 1	3930	7.80E-131
XM_020094333.1	signal-induced proliferation-associated 1-like protein 2 isoform X1	7385	0
XM_020094334.1	transmembrane protein 98	2707	2.65E-144
XM_020094335.1	ras-related protein Rab-5C	1217	2.40E-152
XM_020094336.1	ras-related protein Rab-5C	1220	2.49E-152
XM_020094337.1	60S ribosomal protein L19 isoform X1	762	5.16E-91
XM_020094338.1	60S ribosomal protein L19 isoform X1	677	2.60E-90
XM_020094339.1	NF-kappa-B inhibitor-interacting Ras-like protein 2	2252	1.17E-136
XM_020094340.1	NF-kappa-B inhibitor-interacting Ras-like protein 2	2244	9.33E-137
XM_020094341.1	protein phosphatase 1 regulatory subunit 1B isoform X1	2851	7.84E-89
XM_020094342.1	signal-induced proliferation-associated 1-like protein 2 isoform X1	6789	0
XM_020094343.1	protein phosphatase 1 regulatory subunit 1B isoform X2	2845	1.45E-87
XM_020094344.1	PREDICTED: telethonin	1646	1.19E-127
XM_020094345.1	tetratricopeptide repeat protein 25-like	863	6.51E-104
XM_020094346.1	reprimo-like protein	1492	3.01E-58
XM_020094347.1	ADP-ribosylation factor-like protein 5C	1183	3.34E-136
XM_020094348.1	signal-induced proliferation-associated 1-like protein 2 isoform X3	7121	0
XM_020094349.1	neurogenic locus notch homolog protein 2-like	7943	0
XM_020094350.1	myosin heavy chain, fast skeletal muscle-like	5891	0
XM_020094351.1	myosin heavy chain, fast skeletal muscle-like	5910	0
XM_020094352.1	bromodomain-containing protein 4-like isoform X1	5193	0
XM_020094353.1	bromodomain-containing protein 4-like isoform X2	6835	0
XM_020094354.1	bromodomain-containing protein 4-like isoform X1	7294	0
XM_020094355.1	adhesion G protein-coupled receptor L1	10306	0

XM_020094356.1	calmodulin-regulated spectrin-associated protein 3 isoform X1	7030	0
XM_020094357.1	signal-induced proliferation-associated 1-like protein 2 isoform X4	4758	0
XM_020094358.1	calmodulin-regulated spectrin-associated protein 3 isoform X2	6995	0
XM_020094359.1	manganese-transporting ATPase 13A1	4403	0
XM_020094360.1	pre-mRNA-splicing factor SYF1 isoform X1	3368	0
XM_020094361.1	pre-mRNA-splicing factor SYF1 isoform X2	3494	0
XM_020094362.1	protein VAC14 homolog	3733	0
XM_020094363.1	adhesion G protein-coupled receptor E1-like isoform X1	3489	0
XM_020094364.1	adhesion G protein-coupled receptor E1-like isoform X2	3465	0
XM_020094365.1	long-chain fatty acid transport protein 1-like	3262	0
XM_020094366.1	long-chain fatty acid transport protein 1-like	2918	0
XM_020094367.1	long-chain fatty acid transport protein 1-like	3277	0
XM_020094368.1	long-chain fatty acid transport protein 1-like	3134	0
XM_020094369.1	procollagen galactosyltransferase 1-like	3585	0
XM_020094370.1	NAD-dependent protein deacetylase sirtuin-3-like	3566	0
XM_020094371.1	nuclear receptor subfamily 5 group A member 2-like	2195	0
XM_020094372.1	zinc-binding protein A33-like	2914	0
XM_020094373.1	zinc finger protein 287-like isoform X2	1807	0
XM_020094374.1	zinc finger protein 287-like isoform X2	1606	0
XM_020094375.1	zinc finger protein 287-like isoform X2	1465	0
XM_020094376.1	lysophosphatidic acid receptor 1-A-like isoform X1	1658	0
XM_020094377.1	lysophosphatidic acid receptor 1-A-like isoform X2	2874	0
XM_020094378.1	lysophosphatidic acid receptor 1-A-like isoform X3	1628	0
XM_020094379.1	lysophosphatidic acid receptor 1-A-like isoform X4	2844	0
XM_020094380.1	LIM homeobox transcription factor 1-alpha-like	2862	0
XM_020094381.1	pre-B-cell leukemia transcription factor 1-like isoform X1	5409	0
XM_020094382.1	pre-B-cell leukemia transcription factor 1-like isoform X2	5282	0
XM_020094383.1	CDK2-associated and cullin domain-containing protein 1 isoform X1	2269	0
XM_020094384.1	PREDICTED: uncharacterized protein LOC109634100	1628	0
XM_020094385.1	GDH/6PGL endoplasmic bifunctional protein	985	4.27E-159
XM_020094386.1	microfibril-associated glycoprotein 4-like isoform X1	1596	1.95E-165
XM_020094387.1	desmin-like isoform X2	1177	0
XM_020094388.1	microfibril-associated glycoprotein 4-like isoform X2	1522	2.23E-160
XM_020094389.1	microfibril-associated glycoprotein 4-like isoform X2	1585	7.57E-159
XM_020094390.1	microfibril-associated glycoprotein 4-like isoform X1	1528	1.00E-161
XM_020094391.1	heme-binding protein 2-like	946	2.61E-139
XM_020094392.1	CDK2-associated and cullin domain-containing protein 1 isoform X2	2155	0
XM_020094393.1	heme-binding protein 2-like	852	9.85E-159
XM_020094394.1	coiled-coil-helix-coiled-coil-helix domain-containing protein 5	1127	2.54E-73
XM_020094395.1	coiled-coil-helix-coiled-coil-helix domain-containing protein 5	999	6.59E-74
XM_020094396.1	protein PET100 homolog, mitochondrial	1557	2.80E-43
XM_020094397.1	myosin heavy chain, fast skeletal muscle-like	5999	0
XM_020094398.1	retinol dehydrogenase 8-like	1117	0
XM_020094399.1	myosin-11 isoform X2	5706	0
XM_020094400.1	myosin heavy chain, fast skeletal muscle-like	5888	0
XM_020094401.1	myosin-8-like isoform X1	5820	0
XM_020094402.1	myosin-8-like isoform X2	5805	0
XM_020094403.1	myosin heavy chain, skeletal muscle, adult-like isoform X3	5796	0
XM_020094404.1	myosin-8-like isoform X4	5787	0
XM_020094405.1	pantothenate kinase 1 isoform X1	3480	0
XM_020094406.1	nucleobindin-2 isoform X1	1370	0
XM_020094407.1	eukaryotic translation initiation factor 3 subunit G	1078	0
XM_020094408.1	tRNA-splicing endonuclease subunit Sen54	1705	0
XM_020094409.1	methyltransferase-like protein 22	2123	0
XM_020094410.1	PREDICTED: otopetrin-2-like	2043	0
XM_020094411.1	RUS1 family protein C16orf58 homolog	1474	0
XM_020094412.1	pantothenate kinase 1 isoform X2	2607	0
XM_020094413.1	protein FAM83F-like	2333	0
XM_020094414.1	proteasome assembly chaperone 3	1038	1.33E-84
XM_020094415.1	protein Wnt-9b-like	2585	0
XM_020094416.1	UNC93-like protein MFSD11	2108	0
XM_020094417.1	inward rectifier potassium channel 2-like	1637	0
XM_020094418.1	pentraxin fusion protein-like	932	2.78E-164
XM_020094419.1	charged multivesicular body protein 6-like	1105	1.44E-86
XM_020094420.1	cytohesin-1 isoform X1	1851	0
XM_020094421.1	cx9C motif-containing protein 4	1081	4.50E-46
XM_020094422.1	cx9C motif-containing protein 4	1077	4.39E-46
XM_020094423.1	BAI1-associated protein 3	4499	0
XM_020094424.1	probable E3 ubiquitin-protein ligase TRIM8	2633	0
XM_020094425.1	homeobox protein unc-4 homolog	2254	0
XM_020094426.1	PREDICTED: DELTA-sagatoxin-Srs1a-like	1084	1.20E-123
XM_020094427.1	coenzyme Q-binding protein COQ10 homolog, mitochondrial-like	1249	2.43E-162
XM_020094428.1	transcription factor 20-like	2579	0
XM_020094429.1	dehydrogenase/reductase SDR family member 7C-B-like	1360	0
XM_020094430.1	dehydrogenase/reductase SDR family member 7C-B-like	1511	0
XM_020094431.1	ankyrin repeat and SOCS box protein 16	2246	0
XM_020094432.1	transmembrane protein 100-like	1423	1.39E-58
XM_020094433.1	interferon-induced 35 kDa protein	2241	0
XM_020094434.1	suppressor of fused homolog isoform X1	3300	0

XM_020094435.1	tumor necrosis factor ligand superfamily member 14-like	2099	1.20E-157
XM_020094436.1	tumor necrosis factor ligand superfamily member 14-like	2127	8.68E-163
XM_020094437.1	intercellular adhesion molecule 5	2955	0
XM_020094438.1	ectonucleotide pyrophosphatase/phosphodiesterase family member 7-like	2011	0
XM_020094439.1	adiponectin receptor protein 1	1797	0
XM_020094440.1	GRB2-related adapter protein 2	1524	0
XM_020094441.1	apolipoprotein L2-like	2062	0
XM_020094442.1	suppressor of fused homolog isoform X2	3300	0
XM_020094443.1	complement C1q-like protein 2	1092	1.73E-158
XM_020094444.1	heparan sulfate glucosamine 3-O-sulfotransferase 2	1378	0
XM_020094445.1	retinol dehydrogenase 8-like	1247	0
XM_020094446.1	cytochrome c oxidase subunit 6A, mitochondrial-like	649	1.47E-55
XM_020094447.1	PREDICTED: uncharacterized protein LOC109634150	1888	1.10E-121
XM_020094448.1	tetraspanin-33-like isoform X3	1864	0
XM_020094449.1	PREDICTED: bryoporin-like	897	3.30E-132
XM_020094450.1	heparan sulfate glucosamine 3-O-sulfotransferase 4-like	1417	0
XM_020094451.1	protein shisa-6 homolog isoform X1	2453	0
XM_020094452.1	protein shisa-6 homolog isoform X2	2432	0
XM_020094453.1	BRISC complex subunit Abro1 isoform X1	4159	0
XM_020094454.1	transmembrane protein 235-like isoform X1	1493	4.15E-137
XM_020094455.1	transmembrane protein 235-like isoform X2	1415	8.06E-118
XM_020094456.1	BTB/POZ domain-containing protein 17-like	1902	0
XM_020094457.1	PREDICTED: recoverin	612	1.23E-143
XM_020094458.1	galanin receptor type 2-like	2469	0
XM_020094459.1	inward rectifier potassium channel 16-like	1354	0
XM_020094460.1	BRISC complex subunit Abro1 isoform X2	4156	0
XM_020094461.1	PREDICTED: sesquipedalian-1-like	1416	0
XM_020094462.1	mpv17-like protein	1105	1.03E-136
XM_020094463.1	retinal rod rhodopsin-sensitive cGMP 3',5'-cyclic phosphodiesterase subunit gamma-like	273	1.21E-63
XM_020094464.1	PREDICTED: orexin	864	1.65E-72
XM_020094465.1	myosin-binding protein C, slow-type-like isoform X1	1155	0
XM_020094466.1	myosin-binding protein C, slow-type-like isoform X2	1140	0
XM_020094467.1	myosin-binding protein C, cardiac-type-like isoform X3	1119	0
XM_020094468.1	PREDICTED: uncharacterized protein LOC109634168 isoform X1	1225	0
XM_020094469.1	PREDICTED: uncharacterized protein LOC109634168 isoform X2	1219	0
XM_020094470.1	phosphoinositide-interacting protein-like	1066	3.17E-93
XM_020094471.1	tripartite motif-containing protein 16-like	1053	6.01E-153
XM_020094472.1	protein-lysine N-methyltransferase METTL10	2091	5.87E-169
XM_020094473.1	G-protein coupled receptor family C group 5 member C-like	1646	0
XM_020094474.1	UPF0722 protein C11orf88 homolog	497	8.28E-58
XM_020094475.1	germ cell-specific gene 1-like protein	921	0
XM_020094476.1	group 10 secretory phospholipase A2-like	526	7.56E-77
XM_020094477.1	UPF0585 protein C16orf13 homolog B-like	752	9.77E-145
XM_020094478.1	microtubule-associated serine/threonine-protein kinase 3-like	1019	2.18E-179
XM_020094479.1	dynamin-2 isoform X1	5300	0
XM_020094480.1	sorbin and SH3 domain-containing protein 1-like isoform X1	6259	0
XM_020094481.1	dynamin-2 isoform X2	5288	0
XM_020094482.1	dynamin-2 isoform X1	3482	0
XM_020094483.1	queuine tRNA-ribosyltransferase accessory subunit 2 isoform X1	1711	0
XM_020094484.1	transmembrane emp24 domain-containing protein 1-like	1639	2.28E-172
XM_020094485.1	G patch domain-containing protein 8 isoform X3	5833	0
XM_020094486.1	G patch domain-containing protein 8 isoform X3	5691	0
XM_020094487.1	G patch domain-containing protein 8 isoform X3	5732	0
XM_020094488.1	G patch domain-containing protein 8 isoform X3	5591	0
XM_020094489.1	sorbin and SH3 domain-containing protein 1-like isoform X2	6229	0
XM_020094490.1	Golgi SNAP receptor complex member 2	2001	1.69E-129
XM_020094491.1	SRC kinase signaling inhibitor 1	9174	0
XM_020094492.1	formin-like protein 1	4344	0
XM_020094493.1	ADP-ribosylation factor 1-like	2729	1.02E-101
XM_020094494.1	SH3 and multiple ankyrin repeat domains protein 1 isoform X1	10821	0
XM_020094495.1	SH3 and multiple ankyrin repeat domains protein 1 isoform X1	10904	0
XM_020094496.1	sorbin and SH3 domain-containing protein 1-like isoform X3	6184	0
XM_020094497.1	histone-lysine N-methyltransferase KMT5B	7649	0
XM_020094498.1	histone-lysine N-methyltransferase KMT5B	2818	0
XM_020094499.1	tubulin-specific chaperone D isoform X1	3879	0
XM_020094500.1	tubulin-specific chaperone D isoform X1	3837	0
XM_020094501.1	SAM and SH3 domain-containing protein 3	498	9.11E-60
XM_020094502.1	tubulin-specific chaperone D isoform X1	3715	0
XM_020094503.1	epsin-3 isoform X1	3571	0
XM_020094504.1	epsin-3 isoform X1	3574	0
XM_020094505.1	epsin-3 isoform X1	3569	0
XM_020094506.1	epsin-3 isoform X1	3493	0
XM_020094507.1	sorbin and SH3 domain-containing protein 1-like isoform X4	6175	0
XM_020094508.1	zinc finger protein 750	2395	0
XM_020094509.1	PREDICTED: ketosamine-3-kinase-like	1417	0
XM_020094510.1	ADP-ribosylation factor-like protein 16 isoform X1	1482	3.32E-134
XM_020094511.1	ADP-ribosylation factor-like protein 16 isoform X1	1467	4.14E-102
XM_020094512.1	signal transducer and activator of transcription 5B-like	4729	0
XM_020094513.1	signal transducer and activator of transcription 5B-like	4678	0

XM_020094514.1	signal transducer and activator of transcription 1-alpha/beta-like isoform X1	3467	0
XM_020094515.1	sorbin and SH3 domain-containing protein 1-like isoform X5	7699	0
XM_020094516.1	signal transducer and activator of transcription 1-alpha/beta-like isoform X1	3407	0
XM_020094517.1	hemoglobin subunit beta-A-like	598	1.02E-109
XM_020094518.1	hemoglobin subunit beta-A-like	643	8.96E-110
XM_020094519.1	hemoglobin subunit beta-A-like	576	3.74E-110
XM_020094520.1	hemoglobin subunit beta-1-like	714	8.11E-107
XM_020094521.1	hemoglobin subunit alpha-like	685	9.91E-94
XM_020094522.1	hemoglobin subunit alpha-like	643	9.67E-104
XM_020094523.1	hepatocyte growth factor-regulated tyrosine kinase substrate isoform X1	3693	0
XM_020094524.1	hepatocyte growth factor-regulated tyrosine kinase substrate isoform X2	3690	0
XM_020094525.1	hepatocyte growth factor-regulated tyrosine kinase substrate isoform X3	3687	0
XM_020094526.1	radical S-adenosyl methionine domain-containing protein 1, mitochondrial isoform X1	2051	0
XM_020094527.1	probable methyltransferase TARBP1	5593	0
XM_020094528.1	radical S-adenosyl methionine domain-containing protein 1, mitochondrial isoform X1	1729	0
XM_020094529.1	E3 ubiquitin-protein ligase TRIM39-like	2250	0
XM_020094530.1	claudin-6-like isoform X3	969	1.07E-112
XM_020094531.1	insulin-like growth factor-binding protein complex acid labile subunit	2134	0
XM_020094532.1	G-protein coupled estrogen receptor 1	1115	0
XM_020094533.1	probable G-protein coupled receptor 146	4601	0
XM_020094534.1	ATP-dependent RNA helicase DDX42	3637	0
XM_020094535.1	STE20-related kinase adapter protein alpha isoform X2	2184	0
XM_020094536.1	STE20-related kinase adapter protein alpha isoform X2	2184	0
XM_020094537.1	STE20-related kinase adapter protein alpha isoform X2	2069	0
XM_020094538.1	STE20-related kinase adapter protein alpha isoform X2	2218	0
XM_020094539.1	FERM domain-containing protein 6-like	3068	0
XM_020094540.1	RING finger protein 113A	1095	0
XM_020094541.1	potassium voltage-gated channel subfamily A member 7-like	2164	0
XM_020094542.1	branched-chain-amino-acid aminotransferase, cytosolic-like isoform X1	3897	0
XM_020094543.1	branched-chain-amino-acid aminotransferase, cytosolic-like isoform X2	4251	0
XM_020094544.1	chromobox protein homolog 2	3872	0
XM_020094545.1	PREDICTED: noggin-like	1393	1.78E-137
XM_020094546.1	phosphatidylcholine transfer protein	2165	2.65E-154
XM_020094547.1	FERM domain-containing protein 6-like	2991	0
XM_020094548.1	cAMP-dependent protein kinase catalytic subunit alpha isoform X1	4306	0
XM_020094549.1	cAMP-dependent protein kinase catalytic subunit alpha isoform X2	4753	0
XM_020094550.1	histone chaperone asf1b-B	1521	4.00E-142
XM_020094551.1	protein tweety homolog 3	4151	0
XM_020094552.1	sorting nexin-8	2868	0
XM_020094553.1	sorting nexin-8	2829	0
XM_020094554.1	7,8-dihydro-8-oxoguanine triphosphatase	1361	2.15E-88
XM_020094555.1	FERM domain-containing protein 6-like	2964	0
XM_020094556.1	7,8-dihydro-8-oxoguanine triphosphatase	1379	2.24E-88
XM_020094557.1	7,8-dihydro-8-oxoguanine triphosphatase	1294	4.27E-89
XM_020094558.1	7,8-dihydro-8-oxoguanine triphosphatase	1290	4.14E-89
XM_020094559.1	serine/threonine-protein kinase LMTK1-like isoform X1	7880	0
XM_020094560.1	serine/threonine-protein kinase LMTK1-like isoform X2	7837	0
XM_020094561.1	serine/threonine-protein kinase LMTK1-like isoform X3	7511	0
XM_020094562.1	serine/threonine-protein kinase LMTK1-like isoform X4	6092	0
XM_020094563.1	brain-specific angiogenesis inhibitor 1-associated protein 2 isoform X1	3093	0
XM_020094564.1	brain-specific angiogenesis inhibitor 1-associated protein 2 isoform X2	3090	0
XM_020094565.1	brain-specific angiogenesis inhibitor 1-associated protein 2 isoform X3	3172	0
XM_020094566.1	brain-specific angiogenesis inhibitor 1-associated protein 2 isoform X4	2088	0
XM_020094567.1	brain-specific angiogenesis inhibitor 1-associated protein 2 isoform X5	3217	0
XM_020094568.1	potassium voltage-gated channel subfamily H member 6 isoform X1	5457	0
XM_020094569.1	potassium voltage-gated channel subfamily H member 6 isoform X2	5454	0
XM_020094570.1	leucine zipper putative tumor suppressor 2 homolog	6160	0
XM_020094571.1	potassium voltage-gated channel subfamily H member 6 isoform X3	5448	0
XM_020094572.1	potassium voltage-gated channel subfamily H member 6 isoform X4	5445	0
XM_020094573.1	potassium voltage-gated channel subfamily H member 6 isoform X1	5320	0
XM_020094574.1	potassium voltage-gated channel subfamily H member 6 isoform X6	5415	0
XM_020094575.1	potassium voltage-gated channel subfamily H member 6 isoform X1	4893	0
XM_020094576.1	DDB1- and CUL4-associated factor 7	2336	0
XM_020094577.1	protein SCO1 homolog, mitochondrial	1793	0
XM_020094578.1	histone-lysine N-methyltransferase SETD1A	8519	0
XM_020094579.1	leucine zipper putative tumor suppressor 2 homolog	2796	0
XM_020094580.1	dedicator of cytokinesis protein 7-like isoform X1	7423	0
XM_020094581.1	dedicator of cytokinesis protein 7-like isoform X2	7420	0
XM_020094582.1	dedicator of cytokinesis protein 7-like isoform X3	7408	0
XM_020094583.1	dedicator of cytokinesis protein 7-like isoform X4	7405	0
XM_020094584.1	dedicator of cytokinesis protein 7-like isoform X5	7321	0
XM_020094585.1	dedicator of cytokinesis protein 7-like isoform X6	7318	0
XM_020094586.1	nucleolar transcription factor 1 isoform X1	5536	0
XM_020094587.1	nucleolar transcription factor 1 isoform X1	5305	0
XM_020094588.1	F-box/LRR-repeat protein 12	3448	0
XM_020094589.1	leucine zipper putative tumor suppressor 2 homolog	2632	0
XM_020094590.1	nucleolar transcription factor 1 isoform X1	5105	0
XM_020094591.1	nucleolar transcription factor 1 isoform X1	5005	0
XM_020094592.1	nucleolar transcription factor 1 isoform X1	4968	0

XM_020094593.1	armadillo repeat-containing protein 5	4379	0
XM_020094594.1	protein ANTAGONIST OF LIKE HETEROCHROMATIN PROTEIN 1-like	2026	0
XM_020094595.1	PREDICTED: uncharacterized protein LOC109634240 isoform X1	1741	1.32E-155
XM_020094596.1	PREDICTED: uncharacterized protein LOC109634240 isoform X2	1421	1.71E-146
XM_020094597.1	apoptosis regulator BAX-like	2488	5.72E-135
XM_020094598.1	40S ribosomal protein S11	639	7.15E-109
XM_020094599.1	serine/threonine-protein phosphatase alpha-2 isoform-like	3190	0
XM_020094600.1	serine/threonine-protein phosphatase alpha-2 isoform-like	3168	0
XM_020094601.1	SERTA domain-containing protein 2	6037	3.84E-172
XM_020094602.1	cAMP-specific 3',5'-cyclic phosphodiesterase 4C-like isoform X1	5738	0
XM_020094603.1	cAMP-specific 3',5'-cyclic phosphodiesterase 4B-like isoform X2	5620	0
XM_020094604.1	cAMP-specific 3',5'-cyclic phosphodiesterase 4B-like isoform X2	5196	0
XM_020094605.1	cAMP-specific 3',5'-cyclic phosphodiesterase 4B-like isoform X2	5169	0
XM_020094606.1	glucosidase 2 subunit beta	1972	0
XM_020094607.1	glucosidase 2 subunit beta	1976	0
XM_020094608.1	forkhead box protein K1	2752	0
XM_020094609.1	chitobiosyldiphosphodolichol beta-mannosyltransferase	1817	0
XM_020094610.1	SERTA domain-containing protein 2	5936	1.61E-171
XM_020094611.1	ras-related protein Rab-40C-like	3646	0
XM_020094612.1	ubiquitin carboxyl-terminal hydrolase 36 isoform X1	4390	0
XM_020094613.1	ubiquitin carboxyl-terminal hydrolase 36 isoform X1	4177	0
XM_020094614.1	ubiquitin carboxyl-terminal hydrolase 36 isoform X1	4233	0
XM_020094615.1	cytohesin-1 isoform X1	1251	0
XM_020094616.1	cytohesin-1 isoform X2	3339	0
XM_020094617.1	cytohesin-1 isoform X3	3293	0
XM_020094618.1	cytohesin-1 isoform X2	3357	0
XM_020094619.1	DNA (cytosine-5)-methyltransferase 1	4917	0
XM_020094620.1	E3 ubiquitin-protein ligase TRIM39-like	2621	0
XM_020094621.1	PDZ domain-containing protein GIPC1-like	3037	0
XM_020094622.1	eukaryotic translation initiation factor 3 subunit D isoform X1	1886	0
XM_020094623.1	polycomb group RING finger protein 6	1691	0
XM_020094624.1	eukaryotic translation initiation factor 3 subunit D isoform X2	1880	0
XM_020094625.1	somatostatin receptor type 5-like	2631	0
XM_020094626.1	amine sulfotransferase-like isoform X1	1053	0
XM_020094627.1	amine sulfotransferase-like isoform X2	1048	0
XM_020094628.1	amine sulfotransferase-like isoform X3	873	0
XM_020094629.1	glial fibrillary acidic protein-like	3536	0
XM_020094630.1	ataxin-7-like protein 3 isoform X1	1535	0
XM_020094631.1	ataxin-7-like protein 3 isoform X1	1598	0
XM_020094632.1	adhesion G-protein coupled receptor G4-like	2409	0
XM_020094633.1	ataxin-7-like protein 3 isoform X1	2912	0
XM_020094634.1	ataxin-7-like protein 3 isoform X1	2909	0
XM_020094635.1	ataxin-7-like protein 3 isoform X1	1464	0
XM_020094636.1	transmembrane and ubiquitin-like domain-containing protein 2	1918	1.37E-136

XM_020094637.1	calcium uptake protein 1, mitochondrial isoform X1	1797	0
XM_020094638.1	inactive phospholipase C-like protein 2	5530	0
XM_020094639.1	TBC1 domain family member 10B	6161	0
XM_020094640.1	TBC1 domain family member 10B	6158	0
XM_020094641.1	cytokine receptor-like factor 3	2861	0
XM_020094642.1	cytokine receptor-like factor 3	2856	0
XM_020094643.1	calcium uptake protein 1, mitochondrial isoform X2	1785	0
XM_020094644.1	E3 ubiquitin-protein ligase MGRN1 isoform X1	3694	0
XM_020094645.1	E3 ubiquitin-protein ligase MGRN1 isoform X2	3741	0
XM_020094646.1	serotonin N-acetyltransferase-like isoform X1	1138	1.53E-153
XM_020094647.1	serotonin N-acetyltransferase-like isoform X2	1135	1.15E-152
XM_020094648.1	collagen alpha-1(XI) chain-like isoform X1	7603	0
XM_020094649.1	collagen alpha-1(XI) chain-like isoform X2	7600	0
XM_020094650.1	collagen alpha-1(XI) chain-like isoform X3	7195	0
XM_020094651.1	PREDICTED: otopetrin-2	2135	0
XM_020094652.1	calcium uptake protein 1, mitochondrial isoform X3	1743	0
XM_020094653.1	Usher syndrome type-1G protein isoform X1	3367	0
XM_020094654.1	Usher syndrome type-1G protein isoform X1	3356	0
XM_020094655.1	Usher syndrome type-1G protein isoform X1	3271	0
XM_020094656.1	Golgi apparatus membrane protein TVP23 homolog B-like	1477	3.97E-141
XM_020094657.1	LIM domain-containing protein 2	2059	2.00E-77
XM_020094658.1	cell death-inducing p53-target protein 1 homolog	2896	2.60E-78
XM_020094659.1	U11/U12 small nuclear ribonucleoprotein 25 kDa protein	1032	1.51E-102
XM_020094660.1	calcium uptake protein 1, mitochondrial isoform X4	1740	0
XM_020094661.1	DNA-directed RNA polymerase III subunit RPC10	655	1.08E-78
XM_020094662.1	cdc42-interacting protein 4	3508	0
XM_020094663.1	noelin-2-like isoform X1	2314	0
XM_020094664.1	noelin-2-like isoform X2	1780	0
XM_020094665.1	noelin-2-like isoform X3	1454	0
XM_020094666.1	tubulin beta-4B chain-like	1781	0
XM_020094667.1	hepatic leukemia factor isoform X1	5007	4.29E-159
XM_020094668.1	hepatic leukemia factor isoform X2	5007	3.80E-158
XM_020094669.1	calcium uptake protein 1, mitochondrial isoform X5	1734	0
XM_020094670.1	PREDICTED: uncharacterized protein LOC109634280 isoform X1	5905	0
XM_020094671.1	PREDICTED: uncharacterized protein LOC109634280 isoform X2	5875	0

XM_020094672.1	protein FAM117A isoform X1	2857	0
XM_020094673.1	protein FAM117A isoform X2	2890	1.05E-144
XM_020094674.1	solute carrier family 35 member B1	2112	0
XM_020094675.1	transcription factor MafG	5442	2.09E-96
XM_020094676.1	zinc finger CCCH domain-containing protein 7A-like isoform X2	3659	0
XM_020094677.1	calcium uptake protein 1, mitochondrial isoform X6	1728	0
XM_020094678.1	zinc finger CCCH domain-containing protein 7A-like isoform X2	3722	0
XM_020094679.1	zinc finger CCCH domain-containing protein 7A-like isoform X2	3624	0
XM_020094680.1	recQ-mediated genome instability protein 2	917	7.29E-102
XM_020094681.1	contactin-associated protein 1	4203	0
XM_020094682.1	histone-lysine N-methyltransferase EZH1 isoform X1	2319	0
XM_020094683.1	cdc42 effector protein 1-like	3450	0
XM_020094684.1	ELAV-like protein 3 isoform X1	5779	0
XM_020094685.1	calcium uptake protein 1, mitochondrial isoform X7	1722	0
XM_020094686.1	ELAV-like protein 3 isoform X2	5776	0
XM_020094687.1	ELAV-like protein 3 isoform X3	5776	0
XM_020094688.1	ELAV-like protein 3 isoform X4	5758	0
XM_020094689.1	ELAV-like protein 3 isoform X5	5725	0
XM_020094690.1	ELAV-like protein 3 isoform X6	5722	0
XM_020094691.1	ELAV-like protein 3 isoform X7	5722	0
XM_020094692.1	ELAV-like protein 3 isoform X8	5719	0
XM_020094693.1	ELAV-like protein 3 isoform X9	5704	0
XM_020094694.1	ELAV-like protein 3 isoform X10	5701	0
XM_020094695.1	ELAV-like protein 3 isoform X11	5701	0
XM_020094696.1	ELAV-like protein 3 isoform X12	5686	0
XM_020094697.1	ELAV-like protein 3 isoform X13	5665	0
XM_020094698.1	ELAV-like protein 3 isoform X14	5662	0
XM_020094699.1	ELAV-like protein 3 isoform X15	5659	0
XM_020094700.1	RUN domain-containing protein 3A-like isoform X1	2175	0
XM_020094701.1	PDZ domain-containing protein 8	7083	0
XM_020094702.1	RUN domain-containing protein 3A-like isoform X2	2163	0
XM_020094703.1	non-receptor tyrosine-protein kinase TYK2	4158	0
XM_020094704.1	integrator complex subunit 1 isoform X1	7770	0
XM_020094705.1	integrator complex subunit 1 isoform X2	7767	0
XM_020094706.1	integrator complex subunit 1 isoform X3	7764	0
XM_020094707.1	integrator complex subunit 1 isoform X4	7761	0
XM_020094708.1	integrator complex subunit 1 isoform X5	7758	0
XM_020094709.1	KN motif and ankyrin repeat domain-containing protein 2-like	4801	0
XM_020094710.1	polycomb protein suz12-B-like	4913	0
XM_020094711.1	ribonucleoprotein PTB-binding 1 isoform X1	4076	0
XM_020094712.1	ribonucleoprotein PTB-binding 1 isoform X2	4073	0
XM_020094713.1	calcium-activated potassium channel subunit alpha-1-like isoform X1	5609	0
XM_020094714.1	ribonucleoprotein PTB-binding 1 isoform X3	4147	0
XM_020094715.1	pleckstrin homology domain-containing family H member 3	4868	0
XM_020094716.1	atherin-like isoform X1	2660	0
XM_020094717.1	atherin-like isoform X2	2683	0
XM_020094718.1	atherin-like isoform X3	2680	0
XM_020094719.1	very-long-chain enoyl-CoA reductase-like	1571	0
XM_020094720.1	V-type proton ATPase 116 kDa subunit a-like isoform X1	3665	0
XM_020094721.1	V-type proton ATPase 116 kDa subunit a-like isoform X2	3638	0
XM_020094722.1	calcium-activated potassium channel subunit alpha-1-like isoform X2	5597	0
XM_020094723.1	V-type proton ATPase 116 kDa subunit a-like isoform X3	3620	0
XM_020094724.1	coronin-1C-like isoform X1	1698	0
XM_020094725.1	PREDICTED: claudin-4-like	1501	8.08E-98
XM_020094726.1	60S ribosomal protein L3	1367	0
XM_020094727.1	liprin-alpha-3-like isoform X1	7065	0
XM_020094728.1	liprin-alpha-3-like isoform X2	5877	0
XM_020094729.1	progranulin type I	2527	0
XM_020094730.1	calcium-activated potassium channel subunit alpha-1-like isoform X3	5594	0
XM_020094731.1	60S ribosomal protein L27	484	1.24E-86
XM_020094732.1	transmembrane protein 104	2587	0
XM_020094733.1	suppressor of SWI4 1 homolog	2068	0
XM_020094734.1	angiopoietin-related protein 6	1689	0
XM_020094735.1	P2Y purinoceptor 11	1148	0
XM_020094736.1	hsp90 co-chaperone Cdc37	2800	0
XM_020094737.1	protein FAM134C	3198	0
XM_020094738.1	calcium-activated potassium channel subunit alpha-1-like isoform X4	4749	0
XM_020094739.1	dual specificity mitogen-activated protein kinase kinase 6	2936	0
XM_020094740.1	max-like protein X	2713	1.14E-155
XM_020094741.1	homologous-pairing protein 2 homolog isoform X1	1095	6.06E-155
XM_020094742.1	homologous-pairing protein 2 homolog isoform X1	1089	2.29E-149
XM_020094743.1	PREDICTED: uncharacterized protein C17orf53 homolog isoform X1	2326	0
XM_020094744.1	PREDICTED: uncharacterized protein C17orf53 homolog isoform X2	2213	0
XM_020094745.1	calcium-activated potassium channel subunit alpha-1-like isoform X5	4740	0
XM_020094746.1	protein kinase C and casein kinase substrate in neurons protein 2-like	2423	0
XM_020094747.1	protein kinase C and casein kinase substrate in neurons protein 2-like	2524	0
XM_020094748.1	BAH and coiled-coil domain-containing protein 1-like isoform X1	5035	0
XM_020094749.1	BAH and coiled-coil domain-containing protein 1-like isoform X2	5032	0
XM_020094750.1	BAH and coiled-coil domain-containing protein 1-like isoform X3	5032	0

XM_020094751.1	fascin-2-like isoform X1	1544	0
XM_020094752.1	fascin-2-like isoform X2	1484	0
XM_020094753.1	nuclear distribution protein nudE homolog 1-like	4299	0
XM_020094754.1	calcium-activated potassium channel subunit alpha-1-like isoform X6	4668	0
XM_020094755.1	nuclear distribution protein nudE homolog 1-like	4173	0
XM_020094756.1	transcription elongation factor B polypeptide 2-like	1847	2.98E-79
XM_020094757.1	MKL/myocardin-like protein 2 isoform X1	5472	0
XM_020094758.1	MKL/myocardin-like protein 2 isoform X1	5236	0
XM_020094759.1	teleost multiple tissue opsin-2b	1056	0
XM_020094760.1	MKL/myocardin-like protein 2 isoform X1	5515	0
XM_020094761.1	MKL/myocardin-like protein 2 isoform X2	5457	0
XM_020094762.1	MKL/myocardin-like protein 2 isoform X1	5237	0
XM_020094763.1	protein shisa-9A-like	1472	0
XM_020094764.1	calcium-activated potassium channel subunit alpha-1-like isoform X7	4659	0
XM_020094765.1	solute carrier family 25 member 39-like	2501	0
XM_020094766.1	RNA binding protein fox-1 homolog 3-like isoform X1	5076	0
XM_020094767.1	RNA binding protein fox-1 homolog 3-like isoform X2	5303	0
XM_020094768.1	RNA binding protein fox-1 homolog 3-like isoform X3	1651	0
XM_020094769.1	RNA binding protein fox-1 homolog 3-like isoform X4	1337	0
XM_020094770.1	RNA binding protein fox-1 homolog 3-like isoform X5	5025	0
XM_020094771.1	RNA binding protein fox-1 homolog 3-like isoform X6	5039	0
XM_020094772.1	RNA binding protein fox-1 homolog 3-like isoform X7	1600	0
XM_020094773.1	RNA binding protein fox-1 homolog 3-like isoform X8	5088	0
XM_020094774.1	RNA binding protein fox-1 homolog 3-like isoform X1	5140	0
XM_020094775.1	calcium-activated potassium channel subunit alpha-1-like isoform X8	5483	0
XM_020094776.1	RNA binding protein fox-1 homolog 3-like isoform X10	1878	0
XM_020094777.1	histone-lysine N-methyltransferase EZH1 isoform X1	4022	0
XM_020094778.1	histone-lysine N-methyltransferase EZH1 isoform X1	3763	0
XM_020094779.1	histone-lysine N-methyltransferase EZH1 isoform X1	4018	0
XM_020094780.1	histone-lysine N-methyltransferase EZH1 isoform X1	4065	0
XM_020094781.1	histone-lysine N-methyltransferase EZH1 isoform X1	4061	0
XM_020094782.1	dnaJ homolog subfamily B member 1-like	3680	0
XM_020094783.1	monocyte to macrophage differentiation factor 2-like	2600	2.29E-172
XM_020094784.1	calcium-activated potassium channel subunit alpha-1-like isoform X9	5474	0
XM_020094785.1	tubulin gamma-1 chain	2442	0
XM_020094786.1	mediator of RNA polymerase II transcription subunit 25 isoform X1	2553	0
XM_020094787.1	mediator of RNA polymerase II transcription subunit 25 isoform X2	2550	0
XM_020094788.1	mediator of RNA polymerase II transcription subunit 25 isoform X3	2550	0
XM_020094789.1	mediator of RNA polymerase II transcription subunit 25 isoform X4	2481	0
XM_020094790.1	mediator of RNA polymerase II transcription subunit 25 isoform X5	2478	0
XM_020094791.1	proline-rich AKT1 substrate 1	2321	0
XM_020094792.1	myosin regulatory light chain 2, skeletal muscle isoform	1514	3.19E-116
XM_020094793.1	calcium-activated potassium channel subunit alpha-1-like isoform X10	4753	0
XM_020094794.1	axin-1 isoform X1	6143	0
XM_020094795.1	axin-1 isoform X1	6467	0
XM_020094796.1	axin-1 isoform X1	6269	0
XM_020094797.1	axin-1 isoform X1	6140	0
XM_020094798.1	axin-1 isoform X1	6277	0
XM_020094799.1	axin-1 isoform X1	6035	0
XM_020094800.1	serine/threonine-protein kinase 17A-like	2852	0
XM_020094801.1	phosphatidylinositol N-acetylglucosaminyltransferase subunit Q	3420	0
XM_020094802.1	phosphatidylinositol N-acetylglucosaminyltransferase subunit Q	3434	0
XM_020094803.1	calcium-activated potassium channel subunit alpha-1-like isoform X11	4630	0
XM_020094804.1	TBC1 domain family member 17	2562	0
XM_020094805.1	RUN domain-containing protein 1	3450	0
XM_020094806.1	UPF0764 protein C16orf89 homolog	1538	0
XM_020094807.1	coiled-coil domain-containing protein 47	2016	0
XM_020094808.1	3 beta-hydroxysteroid dehydrogenase type 7	2418	0
XM_020094809.1	inactive rhomboid protein 1 isoform X1	3617	0
XM_020094810.1	calcium-activated potassium channel subunit alpha-1-like isoform X12	5454	0
XM_020094811.1	inactive rhomboid protein 1 isoform X2	3507	0
XM_020094812.1	retinol dehydrogenase 8-like	2007	0
XM_020094813.1	MICAL-like protein 2 isoform X1	4033	0
XM_020094814.1	MICAL-like protein 2 isoform X2	3971	0
XM_020094815.1	MICAL-like protein 2 isoform X3	3606	0
XM_020094816.1	calcium-activated potassium channel subunit alpha-1-like isoform X13	5445	0
XM_020094817.1	calcium-activated potassium channel subunit alpha-1-like isoform X14	5567	0
XM_020094818.1	myelin protein zero-like protein 2	4184	4.06E-137
XM_020094819.1	small nuclear ribonucleoprotein Sm D2 isoform X1	777	4.98E-69
XM_020094820.1	small nuclear ribonucleoprotein Sm D2 isoform X2	780	1.36E-59
XM_020094821.1	transcription cofactor HES-6-like	1912	1.37E-157
XM_020094822.1	transcription cofactor HES-6-like	1747	2.10E-159
XM_020094823.1	phosphatidylinositol transfer protein beta isoform-like	1850	0
XM_020094824.1	calcium-activated potassium channel subunit alpha-1-like isoform X15	5541	0
XM_020094825.1	smoothelin-like protein 2	2758	0
XM_020094826.1	smoothelin-like protein 2	2766	0
XM_020094827.1	upstream stimulatory factor 1	1805	0
XM_020094828.1	glia-derived nexin isoform X1	2006	0
XM_020094829.1	glia-derived nexin isoform X2	2008	0

XM_020094830.1	limbic system-associated membrane protein	2511	0
XM_020094831.1	probable ATP-dependent RNA helicase DHX40	2426	0
XM_020094832.1	calcium-activated potassium channel subunit alpha-1-like isoform X16	5538	0
XM_020094833.1	long-chain-fatty-acid--CoA ligase 4-like	3691	0
XM_020094834.1	PREDICTED: neuromodulin	2203	2.54E-30
XM_020094835.1	protein yippee-like 2	902	2.83E-81
XM_020094836.1	protein yippee-like 2	708	9.53E-82
XM_020094837.1	leucine-rich repeat-containing protein 75B-like	7474	0
XM_020094838.1	GRAM domain-containing protein 1B isoform X1	4761	0
XM_020094839.1	GRAM domain-containing protein 1B isoform X2	3411	0
XM_020094840.1	cyclic AMP-dependent transcription factor ATF-4-like	3845	0
XM_020094841.1	adapter molecule crk-like	1944	0
XM_020094842.1	macrophage metalloelastase-like isoform X1	1749	0
XM_020094843.1	inositol 1,4,5-trisphosphate receptor-interacting protein	2304	0
XM_020094844.1	macrophage metalloelastase-like isoform X2	1657	0
XM_020094845.1	matrix metalloproteinase-20-like	1239	0
XM_020094846.1	platelet-derived growth factor D isoform X1	3930	0
XM_020094847.1	platelet-derived growth factor D isoform X2	3927	0
XM_020094848.1	platelet-derived growth factor D isoform X3	3909	0
XM_020094849.1	platelet-derived growth factor D isoform X4	3906	0
XM_020094850.1	G protein-activated inward rectifier potassium channel 3-like	3425	0
XM_020094851.1	forkhead box protein L2	1911	0
XM_020094852.1	lysosomal amino acid transporter 1 homolog isoform X2	1456	0
XM_020094853.1	lysosomal amino acid transporter 1 homolog isoform X2	1397	0
XM_020094854.1	glutathione S-transferase omega-1	1087	2.11E-168
XM_020094855.1	lysosomal amino acid transporter 1 homolog isoform X2	1360	0
XM_020094856.1	lysosomal amino acid transporter 1 homolog isoform X2	3274	0
XM_020094857.1	RING-box protein 2	768	5.11E-78
XM_020094858.1	thymus-specific serine protease	2136	0
XM_020094859.1	calsyntenin-1 isoform X4	6204	0
XM_020094860.1	ventricular zone-expressed PH domain-containing protein homolog 1	2468	0
XM_020094861.1	pentraxin-related protein PTX3	2955	0
XM_020094862.1	transmembrane protein 199	1307	5.61E-105
XM_020094863.1	sodium-dependent noradrenaline transporter	4418	0
XM_020094864.1	programmed cell death protein 10-like	1529	1.40E-139
XM_020094865.1	tumor necrosis factor receptor superfamily member 19 isoform X1	2004	0
XM_020094866.1	swi5-dependent recombination DNA repair protein 1 homolog	1098	5.67E-143
XM_020094867.1	tumor necrosis factor receptor superfamily member 19 isoform X2	2005	0
XM_020094868.1	sodium channel subunit beta-2	1398	9.52E-106
XM_020094869.1	DNA ligase 1-like	3320	9.98E-128
XM_020094870.1	inositol-tetrakisphosphate 1-kinase-like	1188	0
XM_020094871.1	integral membrane protein 2C-like	1983	2.37E-175
XM_020094872.1	galactosylgalactosylxylosylprotein 3-beta-glucuronosyltransferase 1 isoform X1	4431	0
XM_020094873.1	galactosylgalactosylxylosylprotein 3-beta-glucuronosyltransferase 1 isoform X1	4427	0
XM_020094874.1	proto-oncogene DBL-like	2659	0
XM_020094875.1	galactosylgalactosylxylosylprotein 3-beta-glucuronosyltransferase 1 isoform X2	4359	0
XM_020094876.1	galactosylgalactosylxylosylprotein 3-beta-glucuronosyltransferase 1 isoform X2	4355	0
XM_020094877.1	leukocyte elastase inhibitor-like	2274	0
XM_020094878.1	leukocyte elastase inhibitor-like	2224	0
XM_020094879.1	leukocyte elastase inhibitor-like	2285	0
XM_020094880.1	PREDICTED: uncharacterized protein LOC109634405 isoform X1	11567	0
XM_020094881.1	neurogenic locus notch homolog protein 2-like	3587	0
XM_020094882.1	zinc finger B-box domain-containing protein 1	3957	0

XM_020094883.1	ATP-sensitive inward rectifier potassium channel 1 isoform X1	2468	0
XM_020094884.1	ATP-sensitive inward rectifier potassium channel 1 isoform X2	2366	0
XM_020094885.1	secreted frizzled-related protein 2-like	2414	0
XM_020094886.1	paired mesoderm homeobox protein 2A	2645	2.62E-169
XM_020094887.1	endothelin-converting enzyme-like 1	3703	0
XM_020094888.1	PREDICTED: uncharacterized protein LOC109634405 isoform X2	11560	0
XM_020094889.1	immunoglobulin superfamily member 5-like isoform X1	1429	0
XM_020094890.1	immunoglobulin superfamily member 5-like isoform X2	1400	0
XM_020094891.1	immunoglobulin superfamily member 5-like isoform X3	1242	0
XM_020094892.1	T-lymphocyte activation antigen CD80-like	943	0
XM_020094893.1	nuclear mitotic apparatus protein 1	5502	0
XM_020094894.1	caseinolytic peptidase B protein homolog	1092	0
XM_020094895.1	PREDICTED: uncharacterized protein LOC109634405 isoform X3	11474	0
XM_020094896.1	transmembrane protein 120A	969	0
XM_020094897.1	ras GTPase-activating protein 2-like	3843	0
XM_020094898.1	sodium channel subunit beta-4-like	1167	1.93E-106
XM_020094899.1	lysosomal alpha-glucosidase-like isoform X1	5939	0
XM_020094900.1	PREDICTED: uncharacterized protein LOC109634421	1005	1.67E-173
XM_020094901.1	leucine-, glutamate- and lysine-rich protein 1	2005	0
XM_020094902.1	ribosomal protein S6 kinase beta-1-like	479	2.62E-78
XM_020094903.1	PREDICTED: uncharacterized protein LOC109634405 isoform X4	11327	0
XM_020094904.1	dapper homolog 1-like	3672	0
XM_020094905.1	kin of IRRE-like protein 3	6328	0
XM_020094906.1	matrix metalloproteinase-20-like	1482	0
XM_020094907.1	von Willebrand factor A domain-containing protein 5A-like	1279	0
XM_020094908.1	P2X purinoceptor 5-like	874	0

XM_020094909.1	PREDICTED: uncharacterized protein LOC109634429	993	0
XM_020094910.1	PREDICTED: uncharacterized protein LOC109634405 isoform X5	11523	0
XM_020094911.1	von Willebrand factor A domain-containing protein 5A-like	2891	0
XM_020094912.1	myelin protein zero-like protein 2	918	1.08E-165
XM_020094913.1	transmembrane protein 25	2354	0
XM_020094914.1	complement C1q-like protein 2	700	2.87E-168
XM_020094915.1	TRPM8 channel-associated factor homolog	546	5.87E-137
XM_020094916.1	TRPM8 channel-associated factor homolog	2373	0
XM_020094917.1	zinc finger protein neuro-d4	5411	0
XM_020094918.1	homeobox protein meis3-B-like	1606	0
XM_020094919.1	phosphatidylinositol 4,5-bisphosphate 3-kinase catalytic subunit delta isoform isoform X1	4833	0
XM_020094920.1	ephrin type-A receptor 4-like	3595	0
XM_020094921.1	exportin-1 isoform X1	5001	0
XM_020094922.1	sphingosine-1-phosphate phosphatase 2	818	6.81E-148
XM_020094923.1	dehydrogenase/reductase SDR family member 12-like isoform X2	1525	3.55E-149
XM_020094924.1	solute carrier family 12 member 9-like	4910	0
XM_020094925.1	endothelin-converting enzyme-like 1	2033	0
XM_020094926.1	homeobox protein SEBOX-like	1011	0
XM_020094927.1	glucuronokinase 1-like	2055	0
XM_020094928.1	exportin-1 isoform X1	4752	0
XM_020094929.1	ankycorbin isoform X1	1377	0
XM_020094930.1	tubulin delta chain	2108	0
XM_020094931.1	collagenase 3-like	2002	0
XM_020094932.1	lysophosphatidic acid receptor 6-like	999	0
XM_020094933.1	lysophosphatidic acid receptor 6-like	1040	0
XM_020094934.1	retinol-binding protein 2	634	4.12E-98
XM_020094935.1	transcription cofactor HES-6-like	1218	6.64E-138
XM_020094936.1	exportin-1 isoform X1	4990	0
XM_020094937.1	ras-related protein Rab-38	1788	4.39E-156
XM_020094938.1	PREDICTED: uncharacterized protein C11orf53 homolog isoform X1	1589	2.15E-128
XM_020094939.1	PREDICTED: uncharacterized protein C11orf53 homolog isoform X2	1570	5.52E-124
XM_020094940.1	B-cell CLL/lymphoma 7 protein family member B isoform X1	1383	5.11E-137
XM_020094941.1	B-cell CLL/lymphoma 7 protein family member B isoform X2	1324	2.87E-114
XM_020094942.1	retinal guanylyl cyclase 2-like	3396	0
XM_020094943.1	G2/mitotic-specific cyclin-B2-like isoform X1	1737	0
XM_020094944.1	G2/mitotic-specific cyclin-B-like isoform X2	1725	0
XM_020094945.1	Purkinje cell protein 4-like	1013	1.86E-25
XM_020094946.1	SAM domain-containing protein SAMSN-1-like	1752	0
XM_020094947.1	proteasome subunit alpha type-6-like	1208	9.63E-175
XM_020094948.1	heat shock 70 kDa protein 12A isoform X1	6224	0
XM_020094949.1	alkaline phosphatase	1979	0
XM_020094950.1	transcription elongation factor B polypeptide 3-like	678	6.38E-06
XM_020094951.1	transcription cofactor HES-6-like	1213	6.40E-138
XM_020094952.1	somatolactin precursor	1729	4.50E-157
XM_020094953.1	ras and Rab interactor 3-like	3289	0
XM_020094954.1	peptidoglycan-recognition protein SB1-like	710	2.37E-124
XM_020094955.1	heat shock protein beta-7-like	1314	5.33E-114
XM_020094956.1	heat shock 70 kDa protein 12A isoform X2	6221	0
XM_020094957.1	C5a anaphylatoxin chemotactic receptor 1-like	1204	0
XM_020094958.1	C5a anaphylatoxin chemotactic receptor 1-like	1188	0
XM_020094959.1	rhodopsin kinase 2-like	1632	0
XM_020094960.1	PREDICTED: putative uncharacterized protein DDB_G0282499	1131	2.50E-102
XM_020094961.1	beta-galactosidase-1-like protein 2	1884	0
XM_020094962.1	zona pellucida sperm-binding protein 4-like isoform X1	1559	0
XM_020094963.1	zona pellucida sperm-binding protein 4-like isoform X2	1320	0
XM_020094964.1	heat shock 70 kDa protein 12A isoform X3	6209	0

XM_020094965.1	homeobox protein otx5-like	959	6.04E-169
XM_020094966.1	DNA-directed RNA polymerase II subunit RPB9	485	1.05E-76
XM_020094967.1	CD3 epsilon	1947	4.30E-104
XM_020094968.1	XIAP-associated factor 1 isoform X1	1748	1.26E-161
XM_020094969.1	XIAP-associated factor 1 isoform X2	1685	5.67E-146
XM_020094970.1	heat shock 70 kDa protein 12A isoform X4	6206	0
XM_020094971.1	protein FAM131A-like	1294	0
XM_020094972.1	T-cell surface glycoprotein CD3 gamma chain-like isoform X1	1005	4.78E-122
XM_020094973.1	T-cell surface glycoprotein CD3 gamma chain-like isoform X1	991	3.98E-122
XM_020094974.1	T-cell surface glycoprotein CD3 gamma chain-like isoform X1	968	1.56E-118
XM_020094975.1	homeobox protein BarH-like 2	2097	8.20E-168
XM_020094976.1	gap junction delta-2 protein-like	1069	0
XM_020094977.1	complement C1q-like protein 2	1145	0
XM_020094978.1	heat shock 70 kDa protein 12A isoform X5	6137	0
XM_020094979.1	POU domain class 2-associating factor 1	1460	0
XM_020094980.1	PREDICTED: tektin-1-like	1462	0
XM_020094981.1	zinc-binding protein A33-like isoform X1	2147	0
XM_020094982.1	autophagy-related protein 11-like	1540	0
XM_020094983.1	PREDICTED: uncharacterized protein LOC109634491	1220	0
XM_020094984.1	forkhead box protein G1-like	1483	0
XM_020094985.1	heat shock 70 kDa protein 12A isoform X6	6122	0
XM_020094986.1	transmembrane protein 255B-like	1221	8.72E-180
XM_020094987.1	transmembrane O-methyltransferase-like	788	2.27E-175

XM_020094988.1	acidic repeat-containing protein-like isoform X2	2003	0
XM_020094989.1	probable phospholipid-transporting ATPase IH isoform X4	6311	0
XM_020094990.1	pleckstrin homology-like domain family B member 1 isoform X1	8123	0
XM_020094991.1	pleckstrin homology-like domain family B member 1 isoform X2	8135	0
XM_020094992.1	pleckstrin homology-like domain family B member 1 isoform X3	7954	0
XM_020094993.1	pleckstrin homology-like domain family B member 1 isoform X4	7929	0
XM_020094994.1	alpha-2-macroglobulin-like isoform X1	5347	0
XM_020094995.1	alpha-2-macroglobulin-like isoform X2	5317	0
XM_020094996.1	alpha-2-macroglobulin-like isoform X3	5287	0
XM_020094997.1	clathrin heavy chain 1-like isoform X1	7239	0
XM_020094998.1	clathrin heavy chain 1-like isoform X2	7235	0
XM_020094999.1	mitogen-activated protein kinase kinase kinase 4 isoform X1	5361	0
XM_020095000.1	clathrin heavy chain 1-like isoform X3	7215	0
XM_020095001.1	clathrin heavy chain 1-like isoform X4	7211	0
XM_020095002.1	vasodilator-stimulated phosphoprotein isoform X2	3219	1.27E-90
XM_020095003.1	vasodilator-stimulated phosphoprotein isoform X2	3163	5.67E-90
XM_020095004.1	gem-associated protein 4	3848	0
XM_020095005.1	ubiquitin carboxyl-terminal hydrolase 2-like isoform X1	3554	0
XM_020095006.1	ubiquitin carboxyl-terminal hydrolase 2-like isoform X1	3422	0
XM_020095007.1	ubiquitin carboxyl-terminal hydrolase 2-like isoform X1	3828	0
XM_020095008.1	mitogen-activated protein kinase kinase kinase 4 isoform X2	5259	0
XM_020095009.1	ubiquitin carboxyl-terminal hydrolase 2-like isoform X1	3832	0
XM_020095010.1	ubiquitin carboxyl-terminal hydrolase 2-like isoform X1	3552	0
XM_020095011.1	ubiquitin carboxyl-terminal hydrolase 2-like isoform X1	2969	0
XM_020095012.1	ubiquitin carboxyl-terminal hydrolase 2-like isoform X4	2967	0
XM_020095013.1	tapasin-related protein-like isoform X1	1976	0
XM_020095014.1	tapasin-related protein-like isoform X1	1918	0
XM_020095015.1	protein FAM57A	1046	7.39E-163
XM_020095016.1	unconventional myosin-Ic isoform X1	5897	0
XM_020095017.1	mitogen-activated protein kinase kinase kinase 4 isoform X3	5239	0
XM_020095018.1	unconventional myosin-Ic isoform X2	5967	0
XM_020095019.1	unconventional myosin-Ic isoform X1	6267	0
XM_020095020.1	large neutral amino acids transporter small subunit 4-like isoform X1	4639	0
XM_020095021.1	large neutral amino acids transporter small subunit 4-like isoform X1	4636	0
XM_020095022.1	large neutral amino acids transporter small subunit 4-like isoform X2	4621	0
XM_020095023.1	period circadian protein homolog 2-like isoform X1	7205	0
XM_020095024.1	period circadian protein homolog 2-like isoform X1	7209	0
XM_020095025.1	period circadian protein homolog 2-like isoform X1	7202	0
XM_020095026.1	glioma tumor suppressor candidate region gene 2 protein	1570	0
XM_020095027.1	histone H3.3	2025	8.28E-89
XM_020095028.1	histone H3.3	1870	2.37E-89
XM_020095029.1	histone H3.3	1922	3.69E-89
XM_020095030.1	tyrosine-protein kinase BAZ1B isoform X1	8088	0
XM_020095031.1	prolyl 4-hydroxylase subunit alpha-1 isoform X1	3245	0
XM_020095032.1	tyrosine-protein kinase BAZ1B isoform X2	8058	0
XM_020095033.1	E3 ubiquitin-protein ligase TRIP12 isoform X1	7522	0
XM_020095034.1	E3 ubiquitin-protein ligase TRIP12 isoform X1	6365	0
XM_020095035.1	E3 ubiquitin-protein ligase TRIP12 isoform X1	6385	0
XM_020095036.1	E3 ubiquitin-protein ligase TRIP12 isoform X1	6533	0
XM_020095037.1	E3 ubiquitin-protein ligase TRIP12 isoform X1	6561	0
XM_020095038.1	E3 ubiquitin-protein ligase TRIP12 isoform X1	6177	0
XM_020095039.1	E3 ubiquitin-protein ligase TRIP12 isoform X1	7596	0
XM_020095040.1	E3 ubiquitin-protein ligase TRIP12 isoform X1	7593	0
XM_020095041.1	prolyl 4-hydroxylase subunit alpha-1 isoform X1	3154	0
XM_020095042.1	E3 ubiquitin-protein ligase TRIP12 isoform X5	7563	0
XM_020095043.1	alpha-2-macroglobulin-like isoform X1	4542	0
XM_020095044.1	alpha-2-macroglobulin-like isoform X1	4533	0
XM_020095045.1	tubulin-specific chaperone cofactor E-like protein isoform X1	7148	0
XM_020095046.1	tubulin-specific chaperone cofactor E-like protein isoform X1	7213	0
XM_020095047.1	tubulin-specific chaperone cofactor E-like protein isoform X1	7096	0
XM_020095048.1	tubulin-specific chaperone cofactor E-like protein isoform X1	7013	0
XM_020095049.1	apolipoprotein A-IV	1261	8.81E-172
XM_020095050.1	prolyl 4-hydroxylase subunit alpha-1 isoform X1	3245	0
XM_020095051.1	centrosomal protein of 164 kDa	5862	0
XM_020095052.1	matrix metalloproteinase-28-like	2199	0
XM_020095053.1	RNA-binding protein FUS-like isoform X3	1802	5.13E-99
XM_020095054.1	RNA-binding protein FUS-like isoform X3	1767	1.43E-99
XM_020095055.1	RNA-binding protein FUS-like isoform X3	1768	0
XM_020095056.1	RNA-binding protein FUS-like isoform X4	1732	0
XM_020095057.1	protein FAM124B-like	3076	0
XM_020095058.1	signal recognition particle receptor subunit alpha isoform X1	3685	0
XM_020095059.1	signal recognition particle receptor subunit alpha isoform X2	3682	0
XM_020095060.1	protein FAM118B	2328	0
XM_020095061.1	toll/interleukin-1 receptor domain-containing adapter protein isoform X1	1398	0
XM_020095062.1	toll/interleukin-1 receptor domain-containing adapter protein isoform X2	1387	1.82E-173
XM_020095063.1	apoptosis-stimulating of p53 protein 2 isoform X1	4746	0
XM_020095064.1	leucine-rich repeat-containing protein 51-like	951	3.71E-122
XM_020095065.1	ragulator complex protein LAMTOR1	1344	1.11E-103
XM_020095066.1	NADPH--cytochrome P450 reductase	3654	0

XM_020095067.1	glycerophosphodiester phosphodiesterase domain-containing protein 1	2416	0
XM_020095068.1	cyclin-K isoform X1	3416	0
XM_020095069.1	coatomer subunit beta'	4734	0
XM_020095070.1	apoptosis-stimulating of p53 protein 2 isoform X1	4678	0
XM_020095071.1	28S ribosomal protein S22, mitochondrial	1379	0
XM_020095072.1	bromodomain and WD repeat-containing protein 3-like isoform X1	8232	0
XM_020095073.1	bromodomain and WD repeat-containing protein 3-like isoform X2	8214	0
XM_020095074.1	bromodomain and WD repeat-containing protein 3-like isoform X3	8211	0
XM_020095075.1	SH3 domain-binding glutamic acid-rich protein-like isoform X1	1604	3.23E-99
XM_020095076.1	SH3 domain-binding glutamic acid-rich protein-like isoform X1	1580	3.13E-99
XM_020095077.1	SH3 domain-binding glutamic acid-rich protein-like isoform X1	1553	2.40E-99
XM_020095078.1	SH3 domain-binding glutamic acid-rich protein-like isoform X1	1532	1.95E-99
XM_020095079.1	SH3 domain-binding glutamic acid-rich-like protein isoform X5	1520	7.44E-78
XM_020095080.1	apoptosis-stimulating of p53 protein 2 isoform X1	4562	0
XM_020095081.1	SH3 domain-binding glutamic acid-rich protein-like isoform X1	1505	1.48E-99
XM_020095082.1	SH3 domain-binding glutamic acid-rich-like protein isoform X5	1448	4.36E-78
XM_020095083.1	SH3 domain-binding glutamic acid-rich-like protein isoform X5	1421	3.39E-78
XM_020095084.1	SH3 domain-binding glutamic acid-rich-like protein isoform X9	1316	1.43E-73
XM_020095085.1	SH3 domain-binding glutamic acid-rich-like protein isoform X9	1289	1.11E-73
XM_020095086.1	SH3 domain-binding glutamic acid-rich-like protein isoform X9	1268	9.05E-74
XM_020095087.1	SH3 domain-binding glutamic acid-rich-like protein isoform X5	1244	6.63E-73
XM_020095088.1	SH3 domain-binding glutamic acid-rich-like protein isoform X9	1241	1.37E-74
XM_020095089.1	tail-anchored protein insertion receptor WRB-like	1032	9.13E-118
XM_020095090.1	tail-anchored protein insertion receptor WRB-like	869	1.24E-118
XM_020095091.1	apoptosis-stimulating of p53 protein 2 isoform X1	4707	0
XM_020095092.1	non-histone chromosomal protein HMG-14-like	1275	5.23E-11
XM_020095093.1	PREDICTED: secretogranin-2	2158	0
XM_020095094.1	WD repeat and FYVE domain-containing protein 1	2851	0
XM_020095095.1	AP-1 complex subunit sigma-3	1205	2.97E-108
XM_020095096.1	eukaryotic translation initiation factor 4 gamma 1-like isoform X7	6757	0
XM_020095097.1	eukaryotic translation initiation factor 4 gamma 1-like isoform X7	6755	0
XM_020095098.1	eukaryotic translation initiation factor 4 gamma 1-like isoform X7	6707	0
XM_020095099.1	apoptosis-stimulating of p53 protein 2 isoform X1	4659	0
XM_020095100.1	eukaryotic translation initiation factor 4 gamma 1-like isoform X7	6706	0
XM_020095101.1	eukaryotic translation initiation factor 4 gamma 1-like isoform X7	6748	0
XM_020095102.1	eukaryotic translation initiation factor 4 gamma 1-like isoform X5	6736	0
XM_020095103.1	eukaryotic translation initiation factor 4 gamma 1-like isoform X5	6703	0
XM_020095104.1	eukaryotic translation initiation factor 4 gamma 1-like isoform X7	6218	0
XM_020095105.1	eukaryotic translation initiation factor 4 gamma 1-like isoform X7	6163	0
XM_020095106.1	eukaryotic translation initiation factor 4 gamma 1-like isoform X5	6270	0
XM_020095107.1	serine/threonine-protein kinase SIK3 isoform X1	5764	0
XM_020095108.1	serine/threonine-protein kinase SIK3 isoform X1	5741	0
XM_020095109.1	serine/threonine-protein kinase SIK3 isoform X1	5761	0
XM_020095110.1	serine/threonine-protein kinase SIK3 isoform X1	5629	0
XM_020095111.1	ARF GTPase-activating protein GIT1 isoform X1	3745	0
XM_020095112.1	ARF GTPase-activating protein GIT1 isoform X2	3718	0
XM_020095113.1	ARF GTPase-activating protein GIT1 isoform X1	3592	0
XM_020095114.1	slit homolog 1 protein isoform X1	7494	0
XM_020095115.1	huntingtin-interacting protein 1 isoform X1	6866	0
XM_020095116.1	huntingtin-interacting protein 1 isoform X2	7076	0
XM_020095117.1	sodium/glucose cotransporter 2-like isoform X1	2302	0
XM_020095118.1	sodium/glucose cotransporter 2-like isoform X1	2287	0
XM_020095119.1	sodium/glucose cotransporter 2-like isoform X1	2185	0
XM_020095120.1	kelch-like protein 20	3115	0
XM_020095121.1	sodium/glucose cotransporter 2-like isoform X1	2086	0
XM_020095122.1	serine--tRNA ligase, mitochondrial	2209	0
XM_020095123.1	slit homolog 1 protein isoform X2	7491	0
XM_020095124.1	cilia- and flagella-associated protein 45	1858	0
XM_020095125.1	glucose-6-phosphate exchanger SLC37A4-like	2378	0
XM_020095126.1	trafficking protein particle complex subunit 4	989	1.28E-160
XM_020095127.1	40S ribosomal protein S25	533	5.47E-50
XM_020095128.1	PDZ domain-containing protein 11	538	8.35E-110
XM_020095129.1	phosphatidylinositol 3,4,5-trisphosphate 5-phosphatase 2	6480	0
XM_020095130.1	folate receptor beta	1415	2.10E-168
XM_020095131.1	anaphase-promoting complex subunit 15	1193	3.76E-42
XM_020095132.1	slit homolog 1 protein isoform X3	5026	0
XM_020095133.1	14-3-3 protein epsilon isoform X1	1838	1.30E-156
XM_020095134.1	14-3-3 protein epsilon isoform X2	1890	3.32E-144
XM_020095135.1	F-box only protein 46	4710	0
XM_020095136.1	TCDD-inducible poly [ADP-ribose] polymerase isoform X2	3707	0
XM_020095137.1	TCDD-inducible poly [ADP-ribose] polymerase isoform X2	3328	0
XM_020095138.1	TCDD-inducible poly [ADP-ribose] polymerase isoform X2	3222	0
XM_020095139.1	TCDD-inducible poly [ADP-ribose] polymerase isoform X2	3110	0
XM_020095140.1	adapter molecule crk-like	3956	5.03E-111
XM_020095141.1	slit homolog 1 protein isoform X4	7431	0
XM_020095142.1	SLIT and NTRK-like protein 3	5588	0
XM_020095143.1	neuroligin-4, X-linked-like	1995	0
XM_020095144.1	multidrug resistance-associated protein 4-like	3982	0
XM_020095145.1	WD repeat-containing protein 49	3216	0

XM_020095146.1	complex I assembly factor TIMMDC1, mitochondrial	2364	0
XM_020095147.1	tumor protein p53-inducible protein 13	2935	0
XM_020095148.1	PREDICTED: cylicin-2-like	1787	2.26E-37
XM_020095149.1	polymerase delta-interacting protein 2 isoform X1	3177	0
XM_020095150.1	polymerase delta-interacting protein 2 isoform X2	3171	0
XM_020095151.1	chloride channel protein 2 isoform X1	6714	0
XM_020095152.1	EH domain-binding protein 1 isoform X1	4440	0
XM_020095153.1	chloride channel protein 2 isoform X2	6663	0
XM_020095154.1	chloride channel protein 2 isoform X3	6630	0
XM_020095155.1	chloride channel protein 2 isoform X4	6606	0
XM_020095156.1	coatomer subunit delta isoform X1	2059	0
XM_020095157.1	coatomer subunit delta isoform X1	2126	0
XM_020095158.1	EH domain-containing protein 2-like	2004	0
XM_020095159.1	mitochondrial fission factor isoform X1	1595	0
XM_020095160.1	mitochondrial fission factor isoform X2	1427	0
XM_020095161.1	EH domain-binding protein 1 isoform X2	4437	0
XM_020095162.1	mitochondrial fission factor isoform X3	1366	4.02E-154
XM_020095163.1	multiple epidermal growth factor-like domains protein 6	6338	0
XM_020095164.1	26S proteasome non-ATPase regulatory subunit 2	3395	0
XM_020095165.1	voltage-gated potassium channel subunit beta-1 isoform X1	4794	0
XM_020095166.1	voltage-gated potassium channel subunit beta-1 isoform X2	4791	0
XM_020095167.1	voltage-gated potassium channel subunit beta-1-like isoform X1	5262	0
XM_020095168.1	voltage-gated potassium channel subunit beta-1 isoform X4	4613	0
XM_020095169.1	voltage-gated potassium channel subunit beta-1 isoform X3	4575	0
XM_020095170.1	EH domain-binding protein 1 isoform X3	4330	0
XM_020095171.1	voltage-gated potassium channel subunit beta-1 isoform X3	4770	0
XM_020095172.1	translocon-associated protein subunit gamma	927	3.63E-119
XM_020095173.1	cullin-3 isoform X1	3563	0
XM_020095174.1	cullin-3 isoform X2	3560	0
XM_020095175.1	CXADR-like membrane protein isoform X1	6332	0
XM_020095176.1	CXADR-like membrane protein isoform X1	6065	0
XM_020095177.1	RNA-binding protein 7	1501	1.14E-165
XM_020095178.1	EH domain-binding protein 1 isoform X4	4227	0
XM_020095179.1	phenylalanine--tRNA ligase beta subunit isoform X1	2816	0
XM_020095180.1	phenylalanine--tRNA ligase beta subunit isoform X2	2821	0
XM_020095181.1	arf-GAP with Rho-GAP domain, ANK repeat and PH domain-containing protein 1 isoform X1	2518	0
XM_020095182.1	arf-GAP with Rho-GAP domain, ANK repeat and PH domain-containing protein 1 isoform X2	4362	0
XM_020095183.1	protein NLRC3-like	3466	0
XM_020095184.1	putative N-acetylated-alpha-linked acidic dipeptidase	2739	0
XM_020095185.1	porphobilinogen deaminase isoform X1	3838	0
XM_020095186.1	porphobilinogen deaminase isoform X2	3921	0
XM_020095187.1	EH domain-binding protein 1 isoform X5	4179	0
XM_020095188.1	cytoplasmic dynein 2 heavy chain 1	13504	0
XM_020095189.1	zinc finger and BTB domain-containing protein 38	5228	0
XM_020095190.1	zinc finger and BTB domain-containing protein 38	4886	0
XM_020095191.1	PREDICTED: frizzled-4	2399	0
XM_020095192.1	PREDICTED: frizzled-4	2646	0
XM_020095193.1	serine-protein kinase ATM	9981	0
XM_020095194.1	serine-protein kinase ATM	9734	0
XM_020095195.1	transmembrane protease serine 2	1620	0
XM_020095196.1	transmembrane protease serine 2	1617	0
XM_020095197.1	rap1 GTPase-activating protein 2 isoform X1	5522	0
XM_020095198.1	rap1 GTPase-activating protein 2 isoform X2	5489	0
XM_020095199.1	rap1 GTPase-activating protein 2 isoform X3	4915	0
XM_020095200.1	rap1 GTPase-activating protein 2 isoform X3	4848	0
XM_020095201.1	rap1 GTPase-activating protein 2 isoform X5	5453	0
XM_020095202.1	rap1 GTPase-activating protein 2 isoform X6	4882	0
XM_020095203.1	rap1 GTPase-activating protein 2 isoform X2	4720	0
XM_020095204.1	rap1 GTPase-activating protein 2 isoform X2	4676	0
XM_020095205.1	mitochondrial intermediate peptidase	3898	0
XM_020095206.1	mitochondrial intermediate peptidase	3732	0
XM_020095207.1	solute carrier family 25 member 36-A-like	4018	0
XM_020095208.1	cell division cycle and apoptosis regulator protein 1	4989	0
XM_020095209.1	PREDICTED: sacsin	14057	0
XM_020095210.1	PREDICTED: gamma-sarcoglycan	2281	0
XM_020095211.1	PREDICTED: gamma-sarcoglycan	2278	0
XM_020095212.1	protein NPAT isoform X1	5271	0
XM_020095213.1	protein NPAT isoform X2	5250	0
XM_020095214.1	arf-GAP domain and FG repeat-containing protein 1 isoform X1	2851	0
XM_020095215.1	arf-GAP domain and FG repeat-containing protein 1 isoform X2	2851	0
XM_020095216.1	arf-GAP domain and FG repeat-containing protein 1 isoform X3	2811	0
XM_020095217.1	arf-GAP domain and FG repeat-containing protein 1 isoform X4	2777	0
XM_020095218.1	cell division cycle and apoptosis regulator protein 1	4852	0
XM_020095219.1	arf-GAP domain and FG repeat-containing protein 1 isoform X5	2777	0
XM_020095220.1	arf-GAP domain and FG repeat-containing protein 1 isoform X6	2630	0
XM_020095221.1	arf-GAP domain and FG repeat-containing protein 1 isoform X7	2590	0
XM_020095222.1	arf-GAP domain and FG repeat-containing protein 1 isoform X8	2552	0
XM_020095223.1	arf-GAP domain and FG repeat-containing protein 1 isoform X9	2553	0
XM_020095224.1	arf-GAP domain and FG repeat-containing protein 1 isoform X10	2439	0

XM_020095225.1	thiamine transporter 1	2630	0
XM_020095226.1	beta-secretase 2-like	4007	0
XM_020095227.1	RAC-beta serine/threonine-protein kinase-like	3041	0
XM_020095228.1	NADPH oxidase 4	3450	0
XM_020095229.1	PREDICTED: tyrosinase	3227	0
XM_020095230.1	PREDICTED: tyrosinase	3064	0
XM_020095231.1	probable E3 ubiquitin-protein ligase HERC4 isoform X1	3483	0
XM_020095232.1	PREDICTED: tyrosinase	3076	0
XM_020095233.1	glycerophosphodiester phosphodiesterase domain-containing protein 5	3034	0
XM_020095234.1	inositol polyphosphate 5-phosphatase K isoform X1	4285	0
XM_020095235.1	inositol polyphosphate 5-phosphatase K isoform X2	4258	0
XM_020095236.1	inositol polyphosphate 5-phosphatase K isoform X3	4228	0
XM_020095237.1	endonuclease domain-containing 1 protein-like	1393	0
XM_020095238.1	CLOCK-interacting pacemaker-like isoform X2	3312	0
XM_020095239.1	CLOCK-interacting pacemaker-like isoform X2	3455	0
XM_020095240.1	probable E3 ubiquitin-protein ligase HERC4 isoform X2	3406	0
XM_020095241.1	CLOCK-interacting pacemaker-like isoform X2	3515	0
XM_020095242.1	CLOCK-interacting pacemaker-like isoform X2	3240	0
XM_020095243.1	A-kinase anchor protein SPHKAP isoform X1	5813	0
XM_020095244.1	A-kinase anchor protein SPHKAP isoform X1	6044	0
XM_020095245.1	A-kinase anchor protein SPHKAP isoform X1	5655	0
XM_020095246.1	A-kinase anchor protein SPHKAP isoform X1	5771	0
XM_020095247.1	A-kinase anchor protein SPHKAP isoform X1	5649	0
XM_020095248.1	dynein assembly factor with WDR repeat domains 1	2690	0
XM_020095249.1	Golgi integral membrane protein 4 isoform X1	3547	0
XM_020095250.1	Golgi integral membrane protein 4 isoform X2	3505	0
XM_020095251.1	protein C-ets-1 isoform X1	4496	0
XM_020095252.1	protein C-ets-1 isoform X2	4448	0
XM_020095253.1	protein C-ets-1 isoform X3	4537	0
XM_020095254.1	cytochrome c oxidase subunit 5B, mitochondrial-like	1176	1.41E-90
XM_020095255.1	protein C-ets-1 isoform X3	4580	0
XM_020095256.1	dipeptidyl peptidase 1	2783	0
XM_020095257.1	dipeptidyl peptidase 1	2668	0
XM_020095258.1	SID1 transmembrane family member 2 isoform X1	3323	0
XM_020095259.1	collagen alpha-1(XXIII) chain	5134	0
XM_020095260.1	SID1 transmembrane family member 2 isoform X2	3109	0
XM_020095261.1	vacuole membrane protein 1-like	2266	0
XM_020095262.1	vacuole membrane protein 1-like	2247	0
XM_020095263.1	NAD-dependent protein deacetylase sirtuin-3-like	2793	0
XM_020095264.1	ATP synthase subunit s-like protein	1575	2.15E-157
XM_020095265.1	Friend leukemia integration 1 transcription factor isoform X1	4294	0
XM_020095266.1	Friend leukemia integration 1 transcription factor isoform X1	4320	0
XM_020095267.1	sodium/calcium exchanger 1 isoform X1	8652	0
XM_020095268.1	Friend leukemia integration 1 transcription factor isoform X3	4317	0
XM_020095269.1	Friend leukemia integration 1 transcription factor isoform X4	4293	0
XM_020095270.1	Friend leukemia integration 1 transcription factor isoform X5	4290	0
XM_020095271.1	Friend leukemia integration 1 transcription factor isoform X1	4320	0
XM_020095272.1	Friend leukemia integration 1 transcription factor isoform X1	4447	0
XM_020095273.1	Friend leukemia integration 1 transcription factor isoform X1	4446	0
XM_020095274.1	proprotein convertase subtilisin/kexin type 7	3456	0
XM_020095275.1	numb-like protein isoform X1	4494	0
XM_020095276.1	numb-like protein isoform X1	4750	0
XM_020095277.1	sodium/calcium exchanger 1 isoform X1	8887	0
XM_020095278.1	transforming growth factor beta regulator 1 isoform X1	2420	0
XM_020095279.1	transforming growth factor beta regulator 1 isoform X1	2455	0
XM_020095280.1	transforming growth factor beta regulator 1 isoform X1	2128	0
XM_020095281.1	transforming growth factor beta regulator 1 isoform X1	2357	0
XM_020095282.1	sialate O-acetylesterase	1876	0
XM_020095283.1	von Willebrand factor A domain-containing protein 7-like isoform X1	3599	0
XM_020095284.1	von Willebrand factor A domain-containing protein 7-like isoform X1	3340	0
XM_020095285.1	von Willebrand factor A domain-containing protein 7-like isoform X3	2879	0
XM_020095286.1	sodium/calcium exchanger 1 isoform X1	8555	0
XM_020095287.1	sodium- and chloride-dependent GABA transporter 2-like	1981	0
XM_020095288.1	tsukushin isoform X1	2672	0
XM_020095289.1	tsukushin isoform X1	2602	0
XM_020095290.1	zinc finger FYVE domain-containing protein 1-like	2694	0
XM_020095291.1	calcium-binding protein 39-like	3392	0
XM_020095292.1	cAMP and cAMP-inhibited cGMP 3',5'-cyclic phosphodiesterase 10A isoform X1	5232	0
XM_020095293.1	immunoglobulin superfamily member 11	4648	0
XM_020095294.1	sodium/calcium exchanger 1 isoform X1	8628	0
XM_020095295.1	MDS1 and EVI1 complex locus protein EVI1 isoform X1	5239	0
XM_020095296.1	MDS1 and EVI1 complex locus protein EVI1 isoform X2	5236	0
XM_020095297.1	MDS1 and EVI1 complex locus protein EVI1 isoform X3	5212	0
XM_020095298.1	MDS1 and EVI1 complex locus protein EVI1 isoform X4	5396	0
XM_020095299.1	MDS1 and EVI1 complex locus protein EVI1 isoform X5	5366	0
XM_020095300.1	MDS1 and EVI1 complex locus protein EVI1 isoform X6	5212	0
XM_020095301.1	MDS1 and EVI1 complex locus protein MDS1-like	610	2.04E-91
XM_020095302.1	beta-galactosidase-1-like protein 2	3584	0
XM_020095303.1	PTB-containing, cubilin and LRP1-interacting protein	3007	5.31E-151

XM_020095304.1	sodium/calcium exchanger 1 isoform X1	8649	0
XM_020095305.1	heat shock 70 kDa protein 13	3017	0
XM_020095306.1	Down syndrome cell adhesion molecule-like	7837	0
XM_020095307.1	bleomycin hydrolase	1789	0
XM_020095308.1	cell adhesion molecule 3 isoform 1	1649	0
XM_020095309.1	cell adhesion molecule 3 isoform 1	1735	0
XM_020095310.1	cell adhesion molecule 3 isoform 1	1579	0
XM_020095311.1	dynein assembly factor 1, axonemal	1974	0
XM_020095312.1	39S ribosomal protein L44, mitochondrial	1523	0
XM_020095313.1	PREDICTED: frizzled-9	3049	0
XM_020095314.1	von Willebrand factor A domain-containing protein 5A-like	3074	0
XM_020095315.1	poly(A) polymerase gamma isoform X1	5195	0
XM_020095316.1	transgelin isoform X1	1371	3.46E-147
XM_020095317.1	transgelin isoform X1	1350	1.03E-144
XM_020095318.1	beta-secretase 1	3272	0
XM_020095319.1	T-box transcription factor TBX1-A-like isoform X1	1327	0
XM_020095320.1	T-box transcription factor TBX1-A-like isoform X2	1145	0
XM_020095321.1	SPRY domain-containing SOCS box protein 4-like	2365	0
XM_020095322.1	serpin H1-like isoform X2	2021	0
XM_020095323.1	poly(A) polymerase gamma isoform X2	2354	0
XM_020095324.1	serpin H1-like isoform X2	2030	0
XM_020095325.1	serpin H1-like isoform X2	2039	0
XM_020095326.1	acetolactate synthase-like protein	2411	0
XM_020095327.1	ubiquitin-associated and SH3 domain-containing protein B isoform X1	5337	0
XM_020095328.1	ubiquitin-associated and SH3 domain-containing protein B isoform X2	5334	0
XM_020095329.1	metabotropic glutamate receptor 5-like isoform X1	3537	0
XM_020095330.1	metabotropic glutamate receptor 5-like isoform X2	3495	0
XM_020095331.1	metabotropic glutamate receptor 5-like	1090	1.07E-162
XM_020095332.1	paired box protein Pax-3	3095	0
XM_020095333.1	atypical kinase COQ8B, mitochondrial-like isoform X1	2334	0
XM_020095334.1	trithorax group protein osa-like isoform X1	5102	0
XM_020095335.1	atypical kinase COQ8B, mitochondrial-like isoform X2	2328	0
XM_020095336.1	2-phosphoxylose phosphatase 1 isoform X1	4456	0
XM_020095337.1	2-phosphoxylose phosphatase 1 isoform X1	3402	0
XM_020095338.1	2-phosphoxylose phosphatase 1 isoform X1	3009	0
XM_020095339.1	CMP-N-acetylneuraminate-beta-galactosamide-alpha-2,3-sialyltransferase 4	2431	0
XM_020095340.1	CMP-N-acetylneuraminate-beta-galactosamide-alpha-2,3-sialyltransferase 4	2498	0
XM_020095341.1	CMP-N-acetylneuraminate-beta-galactosamide-alpha-2,3-sialyltransferase 4	2646	0
XM_020095342.1	C-terminal-binding protein 2-like isoform X2	1899	0
XM_020095343.1	poly [ADP-ribose] polymerase 14-like	4088	0
XM_020095344.1	coagulation factor XIII A chain	2608	0
XM_020095345.1	coagulation factor XIII A chain	2978	0
XM_020095346.1	zinc fingers and homeoboxes protein 2-like	1686	0
XM_020095347.1	cell death activator CIDE-B	1258	8.00E-144
XM_020095348.1	C-terminal-binding protein 2-like isoform X3	2072	0
XM_020095349.1	UPF0687 protein C20orf27 homolog isoform X1	2603	4.85E-111
XM_020095350.1	UPF0687 protein C20orf27 homolog isoform X1	2693	9.31E-111
XM_020095351.1	5,6-dihydroxyindole-2-carboxylic acid oxidase	2478	0
XM_020095352.1	peroxisome proliferator-activated receptor gamma coactivator 1-alpha isoform X1	4262	0
XM_020095353.1	peroxisome proliferator-activated receptor gamma coactivator 1-alpha isoform X2	3967	0
XM_020095354.1	peroxisome proliferator-activated receptor gamma coactivator 1-alpha isoform X3	3964	0
XM_020095355.1	peroxisome proliferator-activated receptor gamma coactivator 1-alpha isoform X4	3964	0
XM_020095356.1	peroxisome proliferator-activated receptor gamma coactivator 1-alpha isoform X5	5258	0
XM_020095357.1	peroxisome proliferator-activated receptor gamma coactivator 1-alpha isoform X6	3961	0
XM_020095358.1	probable tRNA (uracil-O(2)-)-methyltransferase	2303	0
XM_020095359.1	testis-expressed sequence 36 protein	668	9.09E-129
XM_020095360.1	oligosaccharyltransferase complex subunit OSTC	1159	2.26E-103
XM_020095361.1	ventral anterior homeobox 2-like	3812	1.48E-93
XM_020095362.1	RING finger protein B-like isoform X1	2441	0
XM_020095363.1	RING finger protein B-like isoform X2	2282	0
XM_020095364.1	caspase activity and apoptosis inhibitor 1	1393	0
XM_020095365.1	la-related protein 7 isoform X1	2166	0
XM_020095366.1	la-related protein 7 isoform X2	2163	0
XM_020095367.1	active breakpoint cluster region-related protein-like isoform X1	3221	0
XM_020095368.1	active breakpoint cluster region-related protein-like isoform X2	3220	0
XM_020095369.1	active breakpoint cluster region-related protein-like isoform X3	2513	0
XM_020095370.1	active breakpoint cluster region-related protein-like isoform X4	2925	0
XM_020095371.1	transmembrane protein 179B	1655	7.42E-143
XM_020095372.1	tRNA methyltransferase 10 homolog B	1432	0
XM_020095373.1	interleukin-8-like isoform X1	719	4.00E-74
XM_020095374.1	interleukin-8-like isoform X1	796	1.44E-59
XM_020095375.1	coiled-coil domain-containing protein 149-like	2433	0
XM_020095376.1	macrophage-capping protein	1495	0
XM_020095377.1	DNA damage-inducible transcript 4-like protein	1657	4.72E-136
XM_020095378.1	A disintegrin and metalloproteinase with thrombospondin motifs 3-like	985	0
XM_020095379.1	protein FAM83H-like	5534	0
XM_020095380.1	docking protein 2	3145	0
XM_020095381.1	mitochondrial fission 1 protein	927	7.18E-81
XM_020095382.1	homeobox protein HMX1	1361	4.81E-171

XM_020095383.1	homeobox protein HMX2-like	1293	7.81E-168
XM_020095384.1	synaptotagmin-7-like isoform X2	2628	0
XM_020095385.1	protein FAM83H-like	5652	0
XM_020095386.1	PREDICTED: uncharacterized protein LOC109634698	2845	0
XM_020095387.1	glucosidase 2 subunit beta	2096	0
XM_020095388.1	PREDICTED: zeta-sarcoglycan-like	2655	0
XM_020095389.1	carboxypeptidase Z-like	2361	0
XM_020095390.1	tudor domain-containing protein 7A-like	5458	0
XM_020095391.1	protein FAM83H-like	5682	0
XM_020095392.1	tudor domain-containing protein 7A-like	5178	0
XM_020095393.1	myozenin-2-like isoform X1	1359	0
XM_020095394.1	myozenin-2-like isoform X1	1408	0
XM_020095395.1	myozenin-2-like isoform X2	1323	0
XM_020095396.1	myozenin-2-like isoform X2	1371	0
XM_020095397.1	ubiquitin carboxyl-terminal hydrolase 48	2468	0
XM_020095398.1	ubiquitin carboxyl-terminal hydrolase 48	1524	0
XM_020095399.1	PREDICTED: uncharacterized protein LOC109634707	1602	0
XM_020095400.1	leucine-rich repeat LGI family member 2-like	3081	0
XM_020095401.1	carbohydrate sulfotransferase 12-like	1418	0
XM_020095402.1	fatty acid-binding protein, intestinal-like	1513	4.69E-90
XM_020095403.1	nucleolar and coiled-body phosphoprotein 1 isoform X2	3993	1.09E-56
XM_020095404.1	PREDICTED: uncharacterized protein LOC109634713	2660	3.70E-108
XM_020095405.1	cholecystokinin receptor-like	3463	0
XM_020095406.1	PREDICTED: netrin-1-like	3551	0
XM_020095407.1	calcium-binding protein 2-like	771	2.39E-131
XM_020095408.1	heat shock 70 kDa protein 12B-like	2865	0
XM_020095409.1	nucleolar and coiled-body phosphoprotein 1 isoform X2	3993	1.09E-56
XM_020095410.1	serine/threonine-protein kinase MARK2	4838	0
XM_020095411.1	1-phosphatidylinositol 4,5-bisphosphate phosphodiesterase beta-3-like	5258	0
XM_020095412.1	prospero homeobox protein 1-like	4869	0
XM_020095413.1	B-cell scaffold protein with ankyrin repeats-like	1872	0
XM_020095414.1	O-phosphoseryl-tRNA(Sec) selenium transferase-like	1874	0
XM_020095415.1	inactive ubiquitin carboxyl-terminal hydrolase 53-like	4695	0
XM_020095416.1	E3 ubiquitin-protein ligase DTX4-like	2394	0
XM_020095417.1	tissue-type plasminogen activator isoform X1	1869	0
XM_020095418.1	arf-GAP domain and FG repeat-containing protein 2-like	2192	0
XM_020095419.1	secreted frizzled-related protein 5	2161	0
XM_020095420.1	protein canopy 4-like	959	5.17E-116
XM_020095421.1	arf-GAP with Rho-GAP domain, ANK repeat and PH domain-containing protein 2	6740	0
XM_020095422.1	DEAD-box ATP-dependent RNA helicase 42-like	2059	0
XM_020095423.1	sushi, von Willebrand factor type A, EGF and pentraxin domain-containing protein 1	4000	0
XM_020095424.1	sushi, von Willebrand factor type A, EGF and pentraxin domain-containing protein 1-like	1274	1.06E-162
XM_020095425.1	dual specificity testis-specific protein kinase 1	2051	0
XM_020095426.1	GTPase IMAP family member 8-like	6589	0
XM_020095427.1	zinc finger protein OZF-like	1415	0
XM_020095428.1	lecithin retinol acyltransferase-like	443	6.24E-89
XM_020095429.1	zinc finger matrin-type protein 4-like	3496	2.83E-131
XM_020095430.1	tetratricopeptide repeat protein 39B-like	4213	0
XM_020095431.1	complement factor I	1951	0
XM_020095432.1	calcium uniporter regulatory subunit MCUb, mitochondrial-like	1011	0
XM_020095433.1	aminoacyl tRNA synthase complex-interacting multifunctional protein 1-like	501	1.46E-88
XM_020095434.1	F-box only protein 41-like	3694	0
XM_020095435.1	nephronectin isoform X2	2232	3.46E-136
XM_020095436.1	zinc finger protein 827 isoform X2	2029	0
XM_020095437.1	flocculation protein FLO11-like	2010	0
XM_020095438.1	golgin subfamily A member 7B	4412	1.28E-91
XM_020095439.1	zinc finger protein 638-like	5677	0
XM_020095440.1	myoferlin-like isoform X3	2181	0
XM_020095441.1	myoferlin-like isoform X1	3887	3.88E-126
XM_020095442.1	PC4 and SFRS1-interacting protein isoform X1	3347	0
XM_020095443.1	proline-, glutamic acid- and leucine-rich protein 1-like isoform X1	5752	0
XM_020095444.1	fibrinogen alpha chain	2468	0
XM_020095445.1	probable RNA-binding protein 46	3072	0
XM_020095446.1	steroid 17-alpha-hydroxylase/17,20 lyase-like	1720	0
XM_020095447.1	early growth response protein 1-like	2472	0
XM_020095448.1	neuropeptide Y receptor type 2-like	5962	0
XM_020095449.1	phosphatidylcholine:ceramide cholinephosphotransferase 2	2871	0
XM_020095450.1	dickkopf-related protein 2-like	1495	1.37E-115
XM_020095451.1	lecithin retinol acyltransferase-like	2221	6.94E-146
XM_020095452.1	GTPase IMAP family member GIMD1-like	724	1.11E-166
XM_020095453.1	ADP-ribosylation factor-like protein 2	945	5.65E-134
XM_020095454.1	5-phosphohydroxy-L-lysine phospho-lyase isoform X1	1893	0
XM_020095455.1	5-phosphohydroxy-L-lysine phospho-lyase isoform X1	1803	0
XM_020095456.1	N-acetyl-D-glucosamine kinase	2018	0
XM_020095457.1	sorting nexin-15	1684	0
XM_020095458.1	anaphase-promoting complex subunit 1 isoform X1	6658	0
XM_020095459.1	doublesex- and mab-3-related transcription factor A1	1474	0
XM_020095460.1	PC-esterase domain-containing protein 1A-like isoform X1	1743	0
XM_020095461.1	PC-esterase domain-containing protein 1A-like isoform X2	1730	0

XM_020095462.1	NF-X1-type zinc finger protein NFXL1	1622	5.56E-76
XM_020095463.1	alcohol dehydrogenase [NADP(+)] A-like	1118	0
XM_020095464.1	alcohol dehydrogenase [NADP(+)] A-like	1146	0
XM_020095465.1	membrane-anchored junction protein	1072	4.13E-169
XM_020095466.1	glycoprotein hormone alpha-2	411	1.39E-82
XM_020095467.1	anaphase-promoting complex subunit 1 isoform X2	6172	0
XM_020095468.1	cytosolic beta-glucosidase-like	1614	0
XM_020095469.1	probable E3 ubiquitin-protein ligase ARI5	1092	0
XM_020095470.1	basic leucine zipper transcriptional factor ATF-like 2	2800	0
XM_020095471.1	transmembrane 4 L6 family member 4	967	7.69E-120
XM_020095472.1	protein Dok-7-like isoform X2	2681	0
XM_020095473.1	protein Dok-7-like isoform X2	2469	0
XM_020095474.1	anaphase-promoting complex subunit 1 isoform X3	6163	0
XM_020095475.1	claudin-15-like isoform X1	1308	7.70E-161
XM_020095476.1	protein fantom-like	4134	0
XM_020095477.1	dynactin subunit 6-like	1003	5.84E-141
XM_020095478.1	olfactory receptor 52B2-like isoform X1	1830	0
XM_020095479.1	olfactory receptor 52B2-like isoform X2	1815	0
XM_020095480.1	ELM2 and SANT domain-containing protein 1-like	3555	0
XM_020095481.1	neuronal acetylcholine receptor subunit alpha-9-II-like	2104	0
XM_020095482.1	ELM2 and SANT domain-containing protein 1-like	3381	0
XM_020095483.1	E3 ubiquitin-protein ligase TRIM33-like	1171	9.92E-125
XM_020095484.1	E3 ubiquitin-protein ligase TRIM33-like	1135	6.47E-125
XM_020095485.1	RING finger protein 222-like	1002	1.17E-108
XM_020095486.1	tumor necrosis factor alpha-induced protein 3	3789	0
XM_020095487.1	atrial natriuretic peptide receptor 1-like	3031	0
XM_020095488.1	placenta-specific gene 8 protein-like	949	1.71E-75
XM_020095489.1	leishmanolysin-like peptidase	2134	0
XM_020095490.1	dynein intermediate chain 2, axonemal	1763	0
XM_020095491.1	G-protein coupled receptor 26-like	1904	0
XM_020095492.1	GRB2-associated-binding protein 1-like	880	1.74E-59
XM_020095493.1	spore wall protein 2-like	1805	0
XM_020095494.1	tumor necrosis factor alpha-induced protein 3	3537	0
XM_020095495.1	potential E3 ubiquitin-protein ligase ariadne-2-like	1341	0
XM_020095496.1	protein NLRC3-like	2807	0
XM_020095497.1	fibrinogen alpha chain-like	974	2.62E-131
XM_020095498.1	glycoprotein hormone beta-5-like	534	5.20E-82
XM_020095499.1	tumor necrosis factor alpha-induced protein 3	3507	0
XM_020095500.1	PREDICTED: uncharacterized protein LOC109634805	963	3.65E-99
XM_020095501.1	zinc finger protein 638-like	9410	0
XM_020095502.1	zinc finger protein 638-like	4089	0
XM_020095503.1	CD209 antigen-like protein E	592	3.48E-116
XM_020095504.1	DENN domain-containing protein 4C isoform X1	7776	0
XM_020095505.1	DENN domain-containing protein 4C isoform X1	7808	0
XM_020095506.1	DENN domain-containing protein 4C isoform X1	7768	0
XM_020095507.1	DENN domain-containing protein 4C isoform X1	7737	0
XM_020095508.1	HAUS augmin-like complex subunit 6	3048	0
XM_020095509.1	perilipin-2-like isoform X1	1475	0
XM_020095510.1	phosphatidylinositol 3,4,5-trisphosphate 3-phosphatase and dual-specificity protein phosphatase PTEN isoform X1	3138	0
XM_020095511.1	PREDICTED: borealin-2-like	930	1.42E-162
XM_020095512.1	sal-like protein 2 isoform X1	4683	0
XM_020095513.1	N6-adenosine-methyltransferase 70 kDa subunit	2952	0
XM_020095514.1	tetratricopeptide repeat protein 5	1957	0
XM_020095515.1	E3 ubiquitin-protein ligase CCNB1IP1	1508	0
XM_020095516.1	E3 ubiquitin-protein ligase CCNB1IP1	1456	0
XM_020095517.1	E3 ubiquitin-protein ligase CCNB1IP1	1432	0
XM_020095518.1	phosphatidylinositol 3,4,5-trisphosphate 3-phosphatase and dual-specificity protein phosphatase PTEN isoform X2	3070	0
XM_020095519.1	E3 ubiquitin-protein ligase CCNB1IP1	1407	0
XM_020095520.1	E3 ubiquitin-protein ligase CCNB1IP1	1389	0
XM_020095521.1	synaptopodin-2-like isoform X1	6444	0
XM_020095522.1	synaptopodin-2-like isoform X1	6717	0
XM_020095523.1	protein transport protein Sec24D	4929	0
XM_020095524.1	Kv channel-interacting protein 4-like isoform X1	2208	0
XM_020095525.1	Kv channel-interacting protein 4-like isoform X1	2215	0
XM_020095526.1	Kv channel-interacting protein 4-like isoform X1	2084	6.42E-179
XM_020095527.1	Kv channel-interacting protein 4-like isoform X1	2569	1.98E-161
XM_020095528.1	Kv channel-interacting protein 4-like isoform X1	2112	4.41E-158
XM_020095529.1	girdin-like isoform X1	4779	0
XM_020095530.1	large neutral amino acids transporter small subunit 2-like isoform X1	4042	0
XM_020095531.1	large neutral amino acids transporter small subunit 2-like isoform X1	4026	0
XM_020095532.1	large neutral amino acids transporter small subunit 2-like isoform X1	3826	0
XM_020095533.1	large neutral amino acids transporter small subunit 2-like isoform X1	3715	0
XM_020095534.1	large neutral amino acids transporter small subunit 2-like isoform X1	3626	0
XM_020095535.1	GMP reductase 2-like	2701	0
XM_020095536.1	GMP reductase 2-like	2731	0
XM_020095537.1	ubiquitin-60S ribosomal protein L40	1712	2.68E-45
XM_020095538.1	girdin-like isoform X2	4776	0
XM_020095539.1	phosphatidylinositol 4,5-bisphosphate 3-kinase catalytic subunit delta isoform isoform X1	3031	0
XM_020095540.1	non-lysosomal glucosylceramidase	4117	0

XM_020095541.1	non-lysosomal glucosylceramidase	4119	0
XM_020095542.1	RAB6A-GEF complex partner protein 2 isoform X1	1944	0
XM_020095543.1	RAB6A-GEF complex partner protein 2 isoform X1	1838	0
XM_020095544.1	RAB6A-GEF complex partner protein 2 isoform X1	1892	0
XM_020095545.1	prostate-associated microseminoprotein-like	1513	2.11E-89
XM_020095546.1	period circadian protein homolog 1-like isoform X1	6127	0
XM_020095547.1	girdin-like isoform X3	4686	0
XM_020095548.1	period circadian protein homolog 1-like isoform X2	5989	0
XM_020095549.1	mucin-17-like isoform X1	1043	1.79E-133
XM_020095550.1	REST corepressor 2	1910	0
XM_020095551.1	REST corepressor 2	1689	0
XM_020095552.1	pyruvate carboxylase, mitochondrial isoform X1	6268	0
XM_020095553.1	pyruvate carboxylase, mitochondrial isoform X2	6830	0
XM_020095554.1	pyruvate carboxylase, mitochondrial isoform X1	6324	0
XM_020095555.1	leucine-rich repeat and fibronectin type III domain-containing protein 1	4912	0
XM_020095556.1	girdin-like isoform X4	4683	0
XM_020095557.1	galactosylgalactosylxylosylprotein 3-beta-glucuronosyltransferase 1 isoform X2	2622	0
XM_020095558.1	galactosylgalactosylxylosylprotein 3-beta-glucuronosyltransferase 1 isoform X2	2061	0
XM_020095559.1	galactosylgalactosylxylosylprotein 3-beta-glucuronosyltransferase 1 isoform X2	1233	0
XM_020095560.1	N-alpha-acetyltransferase 40	4024	8.10E-145
XM_020095561.1	DNA-directed RNA polymerase II subunit RPB1	6064	0
XM_020095562.1	solute carrier family 2, facilitated glucose transporter member 4	3741	0
XM_020095563.1	G protein pathway suppressor 2	1811	0
XM_020095564.1	girdin-like isoform X5	3120	0
XM_020095565.1	G protein pathway suppressor 2	1637	0
XM_020095566.1	Y-box-binding protein 2 isoform X1	1841	7.23E-155
XM_020095567.1	Y-box-binding protein 2 isoform X1	1431	6.92E-157
XM_020095568.1	Y-box-binding protein 2 isoform X2	1839	4.58E-154
XM_020095569.1	Y-box-binding protein 2 isoform X2	1429	4.40E-156
XM_020095570.1	eukaryotic translation initiation factor 5A-1-like	1756	1.21E-100
XM_020095571.1	embryonal Fyn-associated substrate-like	4355	0
XM_020095572.1	PREDICTED: uncharacterized protein LOC109634848	2728	0
XM_020095573.1	dehydrogenase/reductase SDR family member 4-like	1348	0
XM_020095574.1	protein inturned isoform X1	3569	0
XM_020095575.1	protein inturned isoform X2	3566	0
XM_020095576.1	protein inturned isoform X3	3607	0
XM_020095577.1	protein inturned isoform X4	3603	0
XM_020095578.1	kynurenine/alpha-aminoadipate aminotransferase, mitochondrial	1832	0
XM_020095579.1	kynurenine/alpha-aminoadipate aminotransferase, mitochondrial	2081	0
XM_020095580.1	INO80 complex subunit B isoform X1	1441	2.32E-153
XM_020095581.1	pleckstrin homology domain-containing family A member 1 isoform X1	1641	0
XM_020095582.1	INO80 complex subunit B isoform X2	1405	3.26E-144
XM_020095583.1	PREDICTED: uncharacterized protein LOC109634854	2716	0
XM_020095584.1	low density lipoprotein receptor adapter protein 1-like	2518	6.04E-154
XM_020095585.1	low density lipoprotein receptor adapter protein 1-like	1442	6.44E-159
XM_020095586.1	low density lipoprotein receptor adapter protein 1-like	1374	2.83E-159
XM_020095587.1	low density lipoprotein receptor adapter protein 1-like	1297	1.10E-159
XM_020095588.1	diamine acetyltransferase 2-like	2421	6.21E-120
XM_020095589.1	pleckstrin homology domain-containing family A member 1 isoform X2	1642	0
XM_020095590.1	palmitoyltransferase ZDHHC21 isoform X1	3953	0
XM_020095591.1	palmitoyltransferase ZDHHC21 isoform X1	3770	0
XM_020095592.1	palmitoyltransferase ZDHHC21 isoform X1	3551	0
XM_020095593.1	palmitoyltransferase ZDHHC21 isoform X1	3550	0
XM_020095594.1	breast cancer metastasis-suppressor 1 isoform X1	1473	3.72E-116
XM_020095595.1	breast cancer metastasis-suppressor 1 isoform X1	1460	5.88E-114
XM_020095596.1	protein phosphatase 1 regulatory subunit 14B	2760	7.69E-71
XM_020095597.1	receptor-type tyrosine-protein phosphatase delta isoform X1	7208	0
XM_020095598.1	pleckstrin homology domain-containing family A member 1 isoform X3	1715	0
XM_020095599.1	receptor-type tyrosine-protein phosphatase delta isoform X1	7164	0
XM_020095600.1	receptor-type tyrosine-protein phosphatase delta isoform X1	7184	0
XM_020095601.1	receptor-type tyrosine-protein phosphatase delta isoform X1	6972	0
XM_020095602.1	receptor-type tyrosine-protein phosphatase delta isoform X1	6561	0
XM_020095603.1	receptor-type tyrosine-protein phosphatase delta isoform X1	7205	0
XM_020095604.1	receptor-type tyrosine-protein phosphatase delta isoform X1	7205	0
XM_020095605.1	receptor-type tyrosine-protein phosphatase delta isoform X4	7202	0
XM_020095606.1	receptor-type tyrosine-protein phosphatase delta isoform X1	7200	0
XM_020095607.1	receptor-type tyrosine-protein phosphatase delta isoform X6	7197	0
XM_020095608.1	receptor-type tyrosine-protein phosphatase delta isoform X1	7197	0
XM_020095609.1	receptor-type tyrosine-protein phosphatase delta isoform X1	7197	0
XM_020095610.1	receptor-type tyrosine-protein phosphatase delta isoform X9	7194	0
XM_020095611.1	receptor-type tyrosine-protein phosphatase delta isoform X10	7191	0
XM_020095612.1	receptor-type tyrosine-protein phosphatase delta isoform X11	7185	0
XM_020095613.1	receptor-type tyrosine-protein phosphatase delta isoform X12	7182	0
XM_020095614.1	receptor-type tyrosine-protein phosphatase delta isoform X13	7178	0
XM_020095615.1	receptor-type tyrosine-protein phosphatase delta isoform X14	7170	0
XM_020095616.1	receptor-type tyrosine-protein phosphatase delta isoform X15	7166	0
XM_020095617.1	receptor-type tyrosine-protein phosphatase delta isoform X16	7163	0
XM_020095618.1	receptor-type tyrosine-protein phosphatase delta isoform X17	6901	0
XM_020095619.1	receptor-type tyrosine-protein phosphatase delta isoform X18	6898	0
XM_020095620.1	receptor-type tyrosine-protein phosphatase delta isoform X19	6889	0

XM_020095621.1	receptor-type tyrosine-protein phosphatase delta isoform X20	6877	0
XM_020095622.1	receptor-type tyrosine-protein phosphatase delta isoform X21	6859	0
XM_020095623.1	receptor-type tyrosine-protein phosphatase delta isoform X22	5969	0
XM_020095624.1	integral membrane protein GPR137B-like	2715	0
XM_020095625.1	receptor-type tyrosine-protein phosphatase delta isoform X23	5965	0
XM_020095626.1	receptor-type tyrosine-protein phosphatase delta isoform X24	5962	0
XM_020095627.1	receptor-type tyrosine-protein phosphatase delta isoform X25	5958	0
XM_020095628.1	receptor-type tyrosine-protein phosphatase delta isoform X26	5954	0
XM_020095629.1	receptor-type tyrosine-protein phosphatase delta isoform X27	5951	0
XM_020095630.1	receptor-type tyrosine-protein phosphatase delta isoform X28	5943	0
XM_020095631.1	receptor-type tyrosine-protein phosphatase delta isoform X29	5935	0
XM_020095632.1	receptor-type tyrosine-protein phosphatase delta isoform X30	5923	0
XM_020095633.1	receptor-type tyrosine-protein phosphatase delta isoform X31	7152	0
XM_020095634.1	receptor-type tyrosine-protein phosphatase delta isoform X32	7155	0
XM_020095635.1	receptor-type tyrosine-protein phosphatase delta isoform X33	7193	0
XM_020095636.1	receptor-type tyrosine-protein phosphatase delta isoform X34	6848	0
XM_020095637.1	receptor-type tyrosine-protein phosphatase delta isoform X35	6845	0
XM_020095638.1	receptor-type tyrosine-protein phosphatase delta isoform X36	6874	0
XM_020095639.1	receptor-type tyrosine-protein phosphatase delta isoform X37	5955	0
XM_020095640.1	egl nine homolog 1-like	1965	0
XM_020095641.1	receptor-type tyrosine-protein phosphatase delta isoform X38	5939	0
XM_020095642.1	PREDICTED: uncharacterized protein LOC109634866	3856	0
XM_020095643.1	major facilitator superfamily domain-containing protein 8	2516	0
XM_020095644.1	E3 ubiquitin-protein ligase TRIM39-like	2212	0
XM_020095645.1	non-receptor tyrosine-protein kinase TNK1	2798	0
XM_020095646.1	non-receptor tyrosine-protein kinase TNK1	2950	0
XM_020095647.1	non-receptor tyrosine-protein kinase TNK1	2835	0
XM_020095648.1	non-receptor tyrosine-protein kinase TNK1	2887	0
XM_020095649.1	phospholipid scramblase 2-like isoform X2	3098	0
XM_020095650.1	sodium/calcium exchanger 1-like isoform X1	4525	0
XM_020095651.1	gamma-aminobutyric acid receptor subunit pi-like isoform X1	5199	0
XM_020095652.1	sodium/calcium exchanger 1-like isoform X2	4489	0
XM_020095653.1	bcl2-associated agonist of cell death	3347	1.25E-94
XM_020095654.1	bcl2-associated agonist of cell death	3368	1.43E-94
XM_020095655.1	polyadenylate-binding protein-interacting protein 2B isoform X1	2565	1.96E-33
XM_020095656.1	polyadenylate-binding protein-interacting protein 2B isoform X1	2522	1.93E-31
XM_020095657.1	regulator of G-protein signaling 12-like isoform X1	5330	0
XM_020095658.1	regulator of G-protein signaling 12-like isoform X2	3511	0
XM_020095659.1	regulator of G-protein signaling 12-like isoform X3	3660	0
XM_020095660.1	gamma-aminobutyric acid receptor subunit pi-like isoform X1	5244	0
XM_020095661.1	myb/SANT-like DNA-binding domain-containing protein 1	1888	4.62E-180
XM_020095662.1	serine/threonine-protein phosphatase 2B catalytic subunit alpha isoform isoform X1	2695	0
XM_020095663.1	serine/threonine-protein phosphatase 2B catalytic subunit alpha isoform isoform X2	2686	0
XM_020095664.1	serine/threonine-protein phosphatase 2B catalytic subunit alpha isoform isoform X3	2668	0
XM_020095665.1	serine/threonine-protein phosphatase 2B catalytic subunit alpha isoform isoform X4	2659	0
XM_020095666.1	serine/threonine-protein phosphatase 2B catalytic subunit alpha isoform isoform X5	2655	0
XM_020095667.1	serine/threonine-protein phosphatase 2B catalytic subunit alpha isoform isoform X6	2628	0
XM_020095668.1	ATP-binding cassette sub-family A member 1-like isoform X1	8831	0
XM_020095669.1	gamma-aminobutyric acid receptor subunit pi-like isoform X1	4317	0
XM_020095670.1	ATP-binding cassette sub-family A member 1-like isoform X1	8911	0
XM_020095671.1	ATP-binding cassette sub-family A member 1-like isoform X1	8868	0
XM_020095672.1	ATP-binding cassette sub-family A member 1-like isoform X1	8831	0
XM_020095673.1	ATP-binding cassette sub-family A member 1-like isoform X1	6239	0
XM_020095674.1	insulin receptor substrate 1-B-like isoform X1	4641	0
XM_020095675.1	insulin receptor substrate 1-B-like isoform X2	5088	0
XM_020095676.1	insulin receptor substrate 1-B-like isoform X3	4572	0
XM_020095677.1	insulin receptor substrate 1-B-like isoform X4	7938	0
XM_020095678.1	insulin receptor substrate 1-B-like isoform X2	4793	0
XM_020095679.1	stromal interaction molecule 2 isoform X1	4991	0
XM_020095680.1	stromal interaction molecule 2 isoform X1	4025	0
XM_020095681.1	stromal interaction molecule 2 isoform X1	5110	0
XM_020095682.1	TBC1 domain family member 19	2761	0
XM_020095683.1	TBC1 domain family member 19	1834	0
XM_020095684.1	mitochondrial inner membrane protein OXA1L isoform X1	1744	0
XM_020095685.1	mitochondrial inner membrane protein OXA1L isoform X2	1628	0
XM_020095686.1	mitochondrial inner membrane protein OXA1L isoform X3	1612	0
XM_020095687.1	Y+L amino acid transporter 2-like	1965	0
XM_020095688.1	very long-chain specific acyl-CoA dehydrogenase, mitochondrial	3952	0
XM_020095689.1	HAUS augmin-like complex subunit 3 isoform X1	2474	0
XM_020095690.1	HAUS augmin-like complex subunit 3 isoform X1	2490	0
XM_020095691.1	HAUS augmin-like complex subunit 3 isoform X1	2423	0
XM_020095692.1	prostaglandin reductase 1	1992	0
XM_020095693.1	prostaglandin reductase 1	1887	0
XM_020095694.1	protein O-GlcNAcase-like	2870	0
XM_020095695.1	inactive tyrosine-protein kinase 7	5453	0
XM_020095696.1	E3 ubiquitin-protein ligase TRIM39-like	2022	0
XM_020095697.1	macrophage mannose receptor 1-like isoform X1	1875	0
XM_020095698.1	macrophage mannose receptor 1-like isoform X1	1762	0
XM_020095699.1	macrophage mannose receptor 1-like isoform X1	1704	0
XM_020095700.1	macrophage mannose receptor 1-like isoform X1	1617	0

XM_020095701.1	urea transporter 1-like isoform X1	2799	0
XM_020095702.1	urea transporter 1-like isoform X2	1919	0
XM_020095703.1	urea transporter 1-like isoform X3	2481	0
XM_020095704.1	multiple PDZ domain protein isoform X1	7584	0
XM_020095705.1	multiple PDZ domain protein isoform X2	7512	0
XM_020095706.1	multiple PDZ domain protein isoform X3	7494	0
XM_020095707.1	multiple PDZ domain protein isoform X4	7422	0
XM_020095708.1	protein MEMO1	3705	0
XM_020095709.1	receptor-type tyrosine-protein phosphatase eta-like	2303	0
XM_020095710.1	cellular tumor antigen p53-like isoform X1	2915	0
XM_020095711.1	cellular tumor antigen p53-like isoform X1	3011	0
XM_020095712.1	cellular tumor antigen p53-like isoform X1	1273	0
XM_020095713.1	pre-mRNA-splicing factor ATP-dependent RNA helicase DHX15-like	2935	0
XM_020095714.1	segment polarity protein dishevelled homolog DVL-2	3435	0
XM_020095715.1	dnaJ homolog subfamily C member 3-like	3402	0
XM_020095716.1	N-alpha-acetyltransferase 38, NatC auxiliary subunit	719	1.01E-73
XM_020095717.1	collagen alpha-1(XXV) chain isoform X1	5917	0
XM_020095718.1	collagen alpha-1(XXV) chain isoform X2	5526	0
XM_020095719.1	collagen alpha-1(XXV) chain isoform X3	5905	0
XM_020095720.1	suppressor of cytokine signaling 5	9468	0
XM_020095721.1	collagen alpha-1(XXV) chain isoform X4	5881	0
XM_020095722.1	collagen alpha-1(XXV) chain isoform X5	5872	0
XM_020095723.1	collagen alpha-1(XXV) chain isoform X6	5881	0
XM_020095724.1	sodium/potassium/calcium exchanger 2 isoform X1	5891	0
XM_020095725.1	sodium/potassium/calcium exchanger 2 isoform X1	5307	0
XM_020095726.1	sodium/potassium/calcium exchanger 2 isoform X1	5822	0
XM_020095727.1	60S ribosomal protein L34	477	6.63E-81
XM_020095728.1	RNA-binding protein with multiple splicing-like	3774	1.56E-118
XM_020095729.1	RNA-binding protein with multiple splicing-like	3768	1.52E-118
XM_020095730.1	RNA-binding protein with multiple splicing-like	3770	1.54E-118
XM_020095731.1	RNA-binding protein with multiple splicing-like	3397	2.09E-119
XM_020095732.1	RNA-binding protein with multiple splicing-like	3764	1.49E-118
XM_020095733.1	adhesion G protein-coupled receptor A3	5232	0
XM_020095734.1	interferon regulatory factor 2-binding protein 2-B-like	2316	0
XM_020095735.1	adhesion G protein-coupled receptor L3-like isoform X1	7490	0
XM_020095736.1	adhesion G protein-coupled receptor L3-like isoform X2	5889	0
XM_020095737.1	adhesion G protein-coupled receptor L3-like isoform X3	7472	0
XM_020095738.1	adhesion G protein-coupled receptor L3-like isoform X4	5874	0
XM_020095739.1	adhesion G protein-coupled receptor L3-like isoform X5	7451	0
XM_020095740.1	protocadherin alpha-8-like	1707	0
XM_020095741.1	adhesion G protein-coupled receptor L3-like isoform X6	7364	0
XM_020095742.1	adhesion G protein-coupled receptor L3-like isoform X7	7345	0
XM_020095743.1	adhesion G protein-coupled receptor L3-like isoform X8	7305	0
XM_020095744.1	adhesion G protein-coupled receptor L3-like isoform X9	5272	0
XM_020095745.1	adhesion G protein-coupled receptor L3-like isoform X10	5146	0
XM_020095746.1	adhesion G protein-coupled receptor L3-like isoform X11	7463	0
XM_020095747.1	adhesion G protein-coupled receptor L3-like isoform X12	7354	0
XM_020095748.1	adhesion G protein-coupled receptor L3-like isoform X13	7296	0
XM_020095749.1	adhesion G protein-coupled receptor L3-like isoform X14	7336	0
XM_020095750.1	adhesion G protein-coupled receptor L3-like isoform X15	5263	0
XM_020095751.1	V-type proton ATPase subunit B, brain isoform	2769	0
XM_020095752.1	adhesion G protein-coupled receptor L3-like isoform X16	5137	0
XM_020095753.1	adhesion G protein-coupled receptor L3-like isoform X17	7321	0
XM_020095754.1	adhesion G protein-coupled receptor L3-like isoform X18	7281	0
XM_020095755.1	dynactin subunit 1 isoform X1	4412	0
XM_020095756.1	dynactin subunit 1 isoform X2	4409	0
XM_020095757.1	dynactin subunit 1 isoform X3	4388	0
XM_020095758.1	dynactin subunit 1 isoform X1	4455	0
XM_020095759.1	dynactin subunit 1 isoform X5	4328	0
XM_020095760.1	dynactin subunit 1 isoform X6	4324	0
XM_020095761.1	dynactin subunit 1 isoform X1	4304	0
XM_020095762.1	dynactin subunit 1 isoform X8	4304	0
XM_020095763.1	dynactin subunit 1 isoform X1	4283	0
XM_020095764.1	dynactin subunit 1 isoform X3	4280	0
XM_020095765.1	dynactin subunit 1 isoform X11	4259	0
XM_020095766.1	DNA-directed RNA polymerase I subunit RPA2	3726	0
XM_020095767.1	rho guanine nucleotide exchange factor 15-like isoform X1	4636	0
XM_020095768.1	rho guanine nucleotide exchange factor 15-like isoform X1	4650	0
XM_020095769.1	rho guanine nucleotide exchange factor 15-like isoform X1	4633	0
XM_020095770.1	rho guanine nucleotide exchange factor 15-like isoform X1	4630	0
XM_020095771.1	PREDICTED: uncharacterized protein LOC109634917	1138	1.60E-160
XM_020095772.1	PREDICTED: uncharacterized protein LOC109634918	2586	0
XM_020095773.1	PREDICTED: LOW QUALITY PROTEIN: uncharacterized protein LOC109634919	2518	0
XM_020095774.1	PREDICTED: LOW QUALITY PROTEIN: uncharacterized protein LOC109634920	2509	0
XM_020095775.1	PREDICTED: uncharacterized protein LOC109634922	1348	0
XM_020095776.1	tetratricopeptide repeat protein 29 isoform X1	2135	0
XM_020095777.1	tetratricopeptide repeat protein 29 isoform X2	2132	0
XM_020095778.1	tetratricopeptide repeat protein 29 isoform X3	2129	0
XM_020095779.1	N-acylneuraminate-9-phosphatase isoform X1	1238	1.52E-179

XM_020095780.1	sodium/bile acid cotransporter 7 isoform X1	2553	0
XM_020095781.1	sodium/bile acid cotransporter 7 isoform X2	2599	0
XM_020095782.1	serine/threonine-protein phosphatase 2A 56 kDa regulatory subunit beta isoform	7074	0
XM_020095783.1	T-complex protein 1 subunit eta isoform X1	2304	0
XM_020095784.1	T-complex protein 1 subunit eta isoform X2	2363	0
XM_020095785.1	NACHT and WD repeat domain-containing protein 2	5674	0
XM_020095786.1	death domain-containing protein 1	1964	0
XM_020095787.1	N-acylneuraminate-9-phosphatase isoform X2	1382	2.95E-162
XM_020095788.1	homeodomain-interacting protein kinase 1-like	1482	0
XM_020095789.1	homeodomain-interacting protein kinase 1-like	1473	0
XM_020095790.1	PREDICTED: uncharacterized protein LOC109634930	2347	1.45E-165
XM_020095791.1	PREDICTED: uncharacterized protein LOC109634931	731	3.22E-155
XM_020095792.1	microfibrillar-associated protein 3-like	2659	0
XM_020095793.1	low-density lipoprotein receptor-related protein 10	3852	0
XM_020095794.1	4F2 cell-surface antigen heavy chain-like	2305	0
XM_020095795.1	recombining binding protein suppressor of hairless isoform X2	3955	0
XM_020095796.1	coiled-coil domain-containing protein 6	2627	0
XM_020095797.1	recombining binding protein suppressor of hairless isoform X2	3787	0
XM_020095798.1	recombining binding protein suppressor of hairless isoform X2	3792	0
XM_020095799.1	transmembrane protein 151B-like	6978	0
XM_020095800.1	ELAV-like protein 2 isoform X1	3592	0
XM_020095801.1	ELAV-like protein 2 isoform X2	3556	0
XM_020095802.1	ELAV-like protein 2 isoform X1	3811	0
XM_020095803.1	gamma-aminobutyric acid receptor-associated protein	1401	7.28E-83
XM_020095804.1	platelet-activating factor acetylhydrolase IB subunit alpha	2768	0
XM_020095805.1	cytochrome P450 26B1	5815	0
XM_020095806.1	nuclear factor 1 B-type isoform X3	7013	0
XM_020095807.1	nuclear factor 1 B-type isoform X3	7010	0
XM_020095808.1	calcineurin subunit B type 1	3048	1.28E-111
XM_020095809.1	nuclear factor 1 B-type isoform X3	6849	0
XM_020095810.1	nuclear factor 1 B-type isoform X3	6763	0
XM_020095811.1	nuclear factor 1 B-type isoform X4	6924	0
XM_020095812.1	nuclear factor 1 B-type isoform X5	6921	0
XM_020095813.1	nuclear factor 1 B-type isoform X6	6889	0
XM_020095814.1	PREDICTED: semaphorin-4F-like	4211	0
XM_020095815.1	meiosis 1 arrest protein	2165	0
XM_020095816.1	meiosis 1 arrest protein	2379	0
XM_020095817.1	protocadherin-1-like isoform X2	4103	0
XM_020095818.1	sperm-associated antigen 17 isoform X1	6530	0
XM_020095819.1	sperm-associated antigen 17 isoform X2	6527	0
XM_020095820.1	sperm-associated antigen 17 isoform X3	6527	0
XM_020095821.1	sperm-associated antigen 17 isoform X4	6503	0
XM_020095822.1	nucleolysin TIAR isoform X1	3791	0
XM_020095823.1	alpha-mannosidase 2x	3260	0
XM_020095824.1	tetratricopeptide repeat protein 31-like isoform X1	2678	0
XM_020095825.1	tetratricopeptide repeat protein 31-like isoform X2	2675	0
XM_020095826.1	tetratricopeptide repeat protein 31-like isoform X1	2522	0
XM_020095827.1	CTD nuclear envelope phosphatase 1	2967	9.50E-161
XM_020095828.1	ADAMTS-like protein 1 isoform X1	6514	0
XM_020095829.1	ADAMTS-like protein 1 isoform X2	6511	0
XM_020095830.1	ADAMTS-like protein 1 isoform X3	6252	0
XM_020095831.1	nucleolysin TIAR isoform X2	3708	9.13E-171
XM_020095832.1	ADAMTS-like protein 1 isoform X1	1920	0
XM_020095833.1	thioredoxin-like protein 1	728	6.45E-64
XM_020095834.1	leucine rich adaptor protein 1-like	2224	2.56E-116
XM_020095835.1	E3 ubiquitin-protein ligase ZFP91-like isoform X1	2502	0
XM_020095836.1	zinc finger protein weckle-like isoform X2	2421	0
XM_020095837.1	E3 ubiquitin-protein ligase ZFP91-like isoform X1	1912	0
XM_020095838.1	nucleolysin TIAR isoform X2	3500	1.79E-171
XM_020095839.1	wings apart-like protein homolog	4117	0
XM_020095840.1	zinc finger protein 135-like	2568	0
XM_020095841.1	forkhead box protein P1-B-like isoform X1	2525	0
XM_020095842.1	forkhead box protein P1-B-like isoform X1	2540	0
XM_020095843.1	forkhead box protein P1-B-like isoform X1	2518	0
XM_020095844.1	forkhead box protein P1-B-like isoform X1	2436	0
XM_020095845.1	extended synaptotagmin-1-like	4835	0
XM_020095846.1	PRA1 family protein 3	1916	1.83E-122
XM_020095847.1	poly(rC)-binding protein 2-like	1152	0
XM_020095848.1	NEDD8-activating enzyme E1 catalytic subunit-like isoform X1	1648	0
XM_020095849.1	NEDD8-activating enzyme E1 catalytic subunit-like isoform X1	1602	0
XM_020095850.1	transmembrane and coiled-coil domains protein 1 isoform X1	2810	0
XM_020095851.1	AP-1 complex subunit mu-2	3491	0
XM_020095852.1	transmembrane and coiled-coil domains protein 1 isoform X1	2634	0
XM_020095853.1	transmembrane and coiled-coil domains protein 1 isoform X1	2813	0
XM_020095854.1	transmembrane and coiled-coil domains protein 1 isoform X1	2724	0
XM_020095855.1	transmembrane and coiled-coil domains protein 1 isoform X1	2168	0
XM_020095856.1	transmembrane and coiled-coil domains protein 1 isoform X1	2261	0
XM_020095857.1	transmembrane and coiled-coil domains protein 1 isoform X1	2121	0
XM_020095858.1	transmembrane and coiled-coil domains protein 1 isoform X1	1889	0

XM_020095859.1	transmembrane and coiled-coil domains protein 1 isoform X1	2225	0
XM_020095860.1	prickle-like protein 3 isoform X1	3715	0
XM_020095861.1	prickle-like protein 3 isoform X2	3712	0
XM_020095862.1	coatomer subunit zeta-1	1186	6.93E-127
XM_020095863.1	solute carrier family 17 member 9	3603	0
XM_020095864.1	E3 ubiquitin-protein ligase RNF114	2820	3.10E-163
XM_020095865.1	TFIIA-alpha and beta-like factor	1769	0
XM_020095866.1	ATP-dependent RNA helicase DDX39A	2018	0
XM_020095867.1	protein FAM110A-like	1977	0
XM_020095868.1	protein FAM110A-like	2081	0
XM_020095869.1	sodium/potassium-transporting ATPase subunit beta-1-interacting protein 4-like isoform X1	3520	7.38E-144
XM_020095870.1	sodium/potassium-transporting ATPase subunit beta-1-interacting protein 4-like isoform X1	1046	9.02E-153
XM_020095871.1	sodium/potassium-transporting ATPase subunit beta-1-interacting protein 3-like isoform X3	3436	1.20E-122
XM_020095872.1	specifically androgen-regulated gene protein-like	3475	0
XM_020095873.1	nucleolar protein 4-like	6956	0
XM_020095874.1	protein Wnt-2b-A	2625	0
XM_020095875.1	PDZ domain-containing protein 4-like isoform X1	4673	0
XM_020095876.1	PDZ domain-containing protein 4-like isoform X2	4625	0
XM_020095877.1	eukaryotic translation initiation factor 4E-like	1803	2.33E-143
XM_020095878.1	protein Wnt-7a-like	2519	0
XM_020095879.1	protein phosphatase 1 regulatory subunit 12A-like isoform X1	1665	1.74E-126
XM_020095880.1	protein phosphatase 1 regulatory subunit 12A-like isoform X1	1669	1.85E-126
XM_020095881.1	protein phosphatase 1 regulatory subunit 12A-like isoform X1	1405	3.95E-110
XM_020095882.1	protein phosphatase 1 regulatory subunit 12A-like isoform X2	1059	8.35E-107
XM_020095883.1	protein phosphatase 1 regulatory subunit 12A-like isoform X2	1056	6.00E-107
XM_020095884.1	synapsin-1-like isoform X2	3556	0
XM_020095885.1	F-box/WD repeat-containing protein 12	1918	0
XM_020095886.1	cysteine-rich with EGF-like domain protein 1	2557	0
XM_020095887.1	zinc finger BED domain-containing protein 4-like	3230	0
XM_020095888.1	troponin T, cardiac muscle	859	7.20E-128
XM_020095889.1	transmembrane protein 150A-like	1824	1.83E-171
XM_020095890.1	beta-1,4-galactosyltransferase 3-like isoform X1	4342	0
XM_020095891.1	beta-1,4-galactosyltransferase 3-like isoform X1	4246	0
XM_020095892.1	beta-1,4-galactosyltransferase 3-like isoform X1	4219	0
XM_020095893.1	V-type proton ATPase subunit S1-like	2964	0
XM_020095894.1	interferon regulatory factor 10	1897	0
XM_020095895.1	zinc finger CCCH domain-containing protein 13-like	3506	0
XM_020095896.1	zinc finger CCCH domain-containing protein 13-like	3511	0
XM_020095897.1	FERM domain-containing protein 4B-like isoform X1	4014	0
XM_020095898.1	FERM domain-containing protein 4B-like isoform X2	3894	0
XM_020095899.1	FERM domain-containing protein 4B-like isoform X1	3095	0
XM_020095900.1	FERM domain-containing protein 4B-like isoform X4	3846	0
XM_020095901.1	zinc finger protein 512B isoform X1	3767	0
XM_020095902.1	malate dehydrogenase, cytoplasmic-like	1655	0
XM_020095903.1	zinc finger protein 512B isoform X2	3764	0
XM_020095904.1	zinc finger protein 512B isoform X3	3665	0
XM_020095905.1	zinc finger protein 512B isoform X4	3662	0
XM_020095906.1	zinc finger protein 512B isoform X5	2869	0
XM_020095907.1	potassium voltage-gated channel subfamily A member 3-like	3956	0
XM_020095908.1	receptor tyrosine-protein kinase erbB-3-like isoform X2	4051	0
XM_020095909.1	receptor tyrosine-protein kinase erbB-3-like isoform X1	3712	0
XM_020095910.1	PREDICTED: leiomodin-3	2356	0
XM_020095911.1	matrix-remodeling-associated protein 8-like	2445	0
XM_020095912.1	PREDICTED: biglycan	2071	0
XM_020095913.1	NUAK family SNF1-like kinase 1	3066	0
XM_020095914.1	PREDICTED: uncharacterized protein C6orf226 homolog	582	6.60E-107
XM_020095915.1	leucine-rich repeat flightless-interacting protein 2 isoform X1	3388	0
XM_020095916.1	protein lifeguard 2	2164	0
XM_020095917.1	pancreatic progenitor cell differentiation and proliferation factor A-like	853	7.99E-68
XM_020095918.1	prickle-like protein 2	3966	0
XM_020095919.1	dysbindin-like isoform X2	2501	8.51E-108
XM_020095920.1	PREDICTED: uncharacterized protein LOC109635028 isoform X1	3764	0
XM_020095921.1	PREDICTED: uncharacterized protein LOC109635028 isoform X2	3758	0
XM_020095922.1	PREDICTED: uncharacterized protein LOC109635028 isoform X3	3722	0
XM_020095923.1	leucine-rich repeat flightless-interacting protein 2 isoform X1	3340	0
XM_020095924.1	sterile alpha motif domain-containing protein 10	2249	2.45E-102
XM_020095925.1	caM kinase-like vesicle-associated protein	3960	0
XM_020095926.1	beta-1,3-galactosyltransferase 4	2384	0
XM_020095927.1	homeobox protein Hox-C8a-like	1721	2.75E-61
XM_020095928.1	neuron navigator 1-like	2878	0
XM_020095929.1	patatin-like phospholipase domain-containing protein 2	1209	0
XM_020095930.1	leucine-rich repeat flightless-interacting protein 2 isoform X1	2944	0
XM_020095931.1	voltage-dependent calcium channel subunit alpha-2/delta-3-like	3600	0
XM_020095932.1	ERC protein 2-like	3294	0
XM_020095933.1	cytochrome P450 3A40-like	1496	0
XM_020095934.1	1-phosphatidylinositol 4,5-bisphosphate phosphodiesterase gamma-1-like	3868	0
XM_020095935.1	PREDICTED: opsin-5-like	452	2.45E-103
XM_020095936.1	lck-interacting transmembrane adapter 1	5148	0
XM_020095937.1	leucine-rich repeat flightless-interacting protein 2 isoform X1	3317	0

XM_020095938.1	amphoterin-induced protein 1-like	1637	0
XM_020095939.1	dedicator of cytokinesis protein 3-like	8634	0
XM_020095940.1	RCC1 and BTB domain-containing protein 2	2317	0
XM_020095941.1	eukaryotic translation initiation factor 2D	2026	0
XM_020095942.1	membrane-associated guanylate kinase, WW and PDZ domain-containing protein 1-like	6772	0
XM_020095943.1	leucine-rich repeat flightless-interacting protein 2 isoform X1	3288	0
XM_020095944.1	interleukin-17 receptor E-like	3104	0
XM_020095945.1	ninjurin-2-like isoform X3	423	4.12E-101
XM_020095946.1	FYVE, RhoGEF and PH domain-containing protein 3-like	2966	0
XM_020095947.1	protein tyrosine phosphatase domain-containing protein 1-like	1980	0
XM_020095948.1	adenylate cyclase type 6-like	5506	0

XM_020095949.1	signal peptide, CUB and EGF-like domain-containing protein 3	5873	0
XM_020095950.1	ankyrin repeat and SAM domain-containing protein 1A-like	1971	0
XM_020095951.1	leucine-rich repeat flightless-interacting protein 2 isoform X1	3190	0
XM_020095952.1	UHRF1-binding protein 1-like	6450	0
XM_020095953.1	immunoglobulin-like and fibronectin type III domain-containing protein 1	3396	0
XM_020095954.1	protein phosphatase 1 regulatory subunit 12A-like	716	4.48E-138
XM_020095955.1	protein FAM19A2-like	423	9.81E-90
XM_020095956.1	calmodulin-binding transcription activator 1-like	4102	0
XM_020095957.1	lysine-specific demethylase 5C-like	3974	0
XM_020095958.1	leucine-rich repeat flightless-interacting protein 2 isoform X1	3058	0
XM_020095959.1	PDZ domain-containing protein 4-like	3560	0
XM_020095960.1	homeobox protein Hox-C10a-like	3103	0
XM_020095961.1	homeobox protein Hox-C10a-like	2655	0
XM_020095962.1	homeobox protein Hox-C10a-like	2614	0
XM_020095963.1	homeobox protein Hox-C10a-like	2670	0
XM_020095964.1	homeobox protein Hox-C10a-like	2229	0
XM_020095965.1	homeobox protein Hox-C10a-like	2306	0
XM_020095966.1	homeobox protein Hox-C10a-like	2206	0
XM_020095967.1	homeobox protein Hox-C10a-like	2209	0
XM_020095968.1	homeobox protein Hox-C10a-like	2209	0
XM_020095969.1	leucine-rich repeat flightless-interacting protein 2 isoform X1	2468	0
XM_020095970.1	homeobox protein Hox-C10a-like	5164	0
XM_020095971.1	homeobox protein Hox-C11 isoform X1	2169	0
XM_020095972.1	homeobox protein Hox-C11 isoform X2	1321	0
XM_020095973.1	homeobox protein Hox-C4	3637	0
XM_020095974.1	homeobox protein Hox-C13	1278	0
XM_020095975.1	homeobox protein Hox-C12a	2737	8.82E-169
XM_020095976.1	homeobox protein Hox-C9	4048	8.07E-173
XM_020095977.1	protocadherin alpha-8-like	1500	0
XM_020095978.1	leucine-rich repeat flightless-interacting protein 2 isoform X7	2396	0
XM_020095979.1	homeobox protein Hox-B5a-like	1923	4.46E-165
XM_020095980.1	homeobox protein Hox-B5a-like	1914	3.44E-164
XM_020095981.1	homeobox protein Hox-B5a-like	1844	7.76E-139
XM_020095982.1	aurora kinase A-interacting protein	965	2.08E-153
XM_020095983.1	troponin I, slow skeletal muscle-like	1145	3.75E-132
XM_020095984.1	peptidyl-prolyl cis-trans isomerase FKBP5-like	1798	0
XM_020095985.1	troponin C, slow skeletal and cardiac muscles	1111	7.25E-100
XM_020095986.1	leucine-rich repeat flightless-interacting protein 2 isoform X8	2269	0
XM_020095987.1	coiled-coil domain-containing protein 115	1092	7.98E-151
XM_020095988.1	homeobox protein Hox-C1a-like	1295	0
XM_020095989.1	periphilin-1-like isoform X5	1463	0
XM_020095990.1	28S ribosomal protein S25, mitochondrial	949	1.72E-127
XM_020095991.1	mitochondrial ribosome-associated GTPase 2-like	1134	2.64E-147
XM_020095992.1	protein-serine O-palmitoleoyltransferase porcupine	3078	0
XM_020095993.1	protein-serine O-palmitoleoyltransferase porcupine	3056	0
XM_020095994.1	leucine-rich repeat flightless-interacting protein 2 isoform X9	2198	0
XM_020095995.1	bone morphogenetic protein 7 isoform X1	1586	0
XM_020095996.1	bone morphogenetic protein 7 isoform X2	1583	0
XM_020095997.1	cathepsin D	1689	0
XM_020095998.1	5-aminolevulinate synthase, nonspecific, mitochondrial-like isoform X1	2425	0
XM_020095999.1	5-aminolevulinate synthase, nonspecific, mitochondrial-like isoform X1	2431	0
XM_020096000.1	rho guanine nucleotide exchange factor 3-like isoform X1	1950	0
XM_020096001.1	rho guanine nucleotide exchange factor 3-like isoform X2	1791	0
XM_020096002.1	methyl-CpG-binding domain protein 3-like isoform X1	1738	0
XM_020096003.1	O-acetyl-ADP-ribose deacetylase 1 isoform X2	703	6.89E-139
XM_020096004.1	O-acetyl-ADP-ribose deacetylase 1 isoform X2	804	7.26E-139
XM_020096005.1	O-acetyl-ADP-ribose deacetylase 1 isoform X2	742	4.87E-139
XM_020096006.1	regulator of G-protein signaling 7	2419	0
XM_020096007.1	O-acetyl-ADP-ribose deacetylase 1 isoform X2	806	7.54E-139
XM_020096008.1	O-acetyl-ADP-ribose deacetylase 1 isoform X2	773	4.90E-139
XM_020096009.1	non-muscle caldesmon-like isoform X3	2098	0
XM_020096010.1	protein SOGA1-like	3045	0
XM_020096011.1	sushi domain-containing protein 3-like	2069	0
XM_020096012.1	sushi domain-containing protein 3-like	1862	0
XM_020096013.1	meiotic recombination protein SPO11 isoform X1	1454	0
XM_020096014.1	meiotic recombination protein SPO11 isoform X2	1433	0
XM_020096015.1	regulator of G-protein signaling 7	2229	0
XM_020096016.1	caspase recruitment domain-containing protein 19-like	1103	1.24E-152

XM_020096017.1	PREDICTED: uncharacterized protein LOC109635094 isoform X1	1369	0
XM_020096018.1	PREDICTED: uncharacterized protein LOC109635094 isoform X2	1366	0
XM_020096019.1	PREDICTED: uncharacterized protein LOC109635094 isoform X3	1188	0
XM_020096020.1	PREDICTED: uncharacterized protein LOC109635094 isoform X1	1191	0
XM_020096021.1	leucine-rich repeat-containing protein 23	1311	0
XM_020096022.1	class E basic helix-loop-helix protein 23	1665	5.78E-151
XM_020096023.1	PREDICTED: uncharacterized protein LOC109635097	995	1.86E-142
XM_020096024.1	regulator of G-protein signaling 7	2374	0
XM_020096025.1	nerve growth factor-like	1354	4.09E-160
XM_020096026.1	transcription factor ETV7-like isoform X1	2083	0
XM_020096027.1	transcription factor ETV7-like isoform X2	2080	0
XM_020096028.1	transcription factor ETV7-like isoform X3	1986	0
XM_020096029.1	adenosine receptor A3-like	1147	0
XM_020096030.1	regulator of G-protein signaling 7	2414	0

XM_020096031.1	kelch repeat and BTB domain-containing protein 12-like	2716	0
XM_020096032.1	protein FAM19A1-like isoform X1	1471	4.03E-100
XM_020096033.1	potassium channel subfamily K member 15-like	2218	0
XM_020096034.1	guanylyl cyclase-activating protein 2-like	850	2.13E-145
XM_020096035.1	tumor protein D54-like isoform X1	1010	1.36E-149
XM_020096036.1	tumor protein D54-like isoform X2	983	9.33E-143
XM_020096037.1	rho-related GTP-binding protein RhoE-like	584	4.02E-75
XM_020096038.1	extracellular matrix protein 2-like	1878	0
XM_020096039.1	transcription factor Spi-C-like	1154	3.14E-150
XM_020096040.1	PREDICTED: urotensin-2-like	666	1.69E-93
XM_020096041.1	ubiquitin-conjugating enzyme E2 D4-like isoform X1	948	2.67E-107
XM_020096042.1	cadherin-like protein 26	2742	0
XM_020096043.1	PREDICTED: fibromodulin	1133	0
XM_020096044.1	aquaporin AQPAe.a-like	1243	1.90E-156
XM_020096045.1	baculoviral IAP repeat-containing protein 2-like isoform X1	1245	0
XM_020096046.1	prolow-density lipoprotein receptor-related protein 1 isoform X4	12562	0
XM_020096047.1	ubiquitin-conjugating enzyme E2 D4-like isoform X2	768	1.24E-106
XM_020096048.1	prolow-density lipoprotein receptor-related protein 1 isoform X4	12559	0
XM_020096049.1	prolow-density lipoprotein receptor-related protein 1 isoform X4	12522	0
XM_020096050.1	prolow-density lipoprotein receptor-related protein 1 isoform X4	14665	0
XM_020096051.1	formin-like protein 3 isoform X1	6141	0
XM_020096052.1	formin-like protein 3 isoform X1	3326	0
XM_020096053.1	formin-like protein 3 isoform X1	5609	0
XM_020096054.1	formin-like protein 3 isoform X1	5653	0
XM_020096055.1	formin-like protein 3 isoform X1	5994	0
XM_020096056.1	galanin receptor type 1-like	1056	0
XM_020096057.1	zinc fingers and homeoboxes protein 3-like	5187	0
XM_020096058.1	zinc fingers and homeoboxes protein 3-like	4880	0
XM_020096059.1	protein phosphatase 1 regulatory subunit 21	4464	0
XM_020096060.1	PREDICTED: uncharacterized protein C20orf24 homolog	2188	1.45E-82
XM_020096061.1	neuron navigator 1-like isoform X1	7446	0
XM_020096062.1	neuron navigator 1-like isoform X2	7416	0
XM_020096063.1	neuron navigator 1-like isoform X3	7392	0
XM_020096064.1	neuron navigator 1-like isoform X4	7389	0
XM_020096065.1	neuron navigator 1-like isoform X5	7365	0
XM_020096066.1	myosin-binding protein H-like isoform X1	2026	0
XM_020096067.1	myosin-binding protein H-like isoform X1	1951	0
XM_020096068.1	myosin-binding protein H-like isoform X1	1885	0
XM_020096069.1	PREDICTED: uncharacterized protein LOC109635129	1369	0
XM_020096070.1	cysteine and glycine-rich protein 2-like	1071	1.48E-108
XM_020096071.1	pleckstrin homology-like domain family A member 3	1373	1.07E-94
XM_020096072.1	RING finger protein 122-like isoform X2	4439	1.27E-77
XM_020096073.1	lysine-specific demethylase 5C isoform X2	6699	0
XM_020096074.1	lysine-specific demethylase 5C isoform X2	6277	0
XM_020096075.1	lysine-specific demethylase 5C isoform X2	6703	0
XM_020096076.1	lysine-specific demethylase 5C isoform X2	6642	0
XM_020096077.1	IQ motif and SEC7 domain-containing protein 1-like isoform X1	7175	0
XM_020096078.1	IQ motif and SEC7 domain-containing protein 2-like isoform X2	3631	0
XM_020096079.1	monoacylglycerol lipase ABHD6-like	1542	0
XM_020096080.1	BTB/POZ domain-containing protein KCTD6	1208	3.41E-180
XM_020096081.1	RING finger protein 122-like isoform X2	4295	1.58E-65
XM_020096082.1	embryonic polyadenylate-binding protein A-like isoform X1	1758	0
XM_020096083.1	embryonic polyadenylate-binding protein A-like isoform X2	1743	0
XM_020096084.1	epiphycan isoform X1	1611	0
XM_020096085.1	14-3-3 protein beta/alpha-1	2750	1.26E-173
XM_020096086.1	small integral membrane protein 4	953	3.06E-52
XM_020096087.1	ankyrin repeat and SAM domain-containing protein 1A isoform X1	1644	0
XM_020096088.1	ankyrin repeat and SAM domain-containing protein 1A isoform X2	1641	0
XM_020096089.1	RING finger protein 122-like isoform X2	4108	2.32E-65
XM_020096090.1	ankyrin repeat and SAM domain-containing protein 1A isoform X3	2932	0
XM_020096091.1	ankyrin repeat and SAM domain-containing protein 1A isoform X4	1642	0
XM_020096092.1	ankyrin repeat and SAM domain-containing protein 1A isoform X5	1638	0
XM_020096093.1	ankyrin repeat and SAM domain-containing protein 1A isoform X6	1545	0
XM_020096094.1	ankyrin repeat and SAM domain-containing protein 1A isoform X7	2833	0
XM_020096095.1	transcription initiation factor TFIID subunit 11	1755	4.91E-107

XM_020096096.1	NACHT, LRR and PYD domains-containing protein 12-like	3830	0
XM_020096097.1	keratin, type II cytoskeletal 8-like	1513	0
XM_020096098.1	protocadherin gamma-C3-like	1848	0
XM_020096099.1	sarcolemmal membrane-associated protein-like isoform X1	1951	0
XM_020096100.1	sarcolemmal membrane-associated protein-like isoform X1	1812	0
XM_020096101.1	sarcolemmal membrane-associated protein-like isoform X1	1806	0
XM_020096102.1	sarcolemmal membrane-associated protein-like isoform X1	1863	0
XM_020096103.1	ceramide synthase 5-like	1646	0
XM_020096104.1	rho-related GTP-binding protein RhoQ	3573	1.30E-124
XM_020096105.1	SUN domain-containing protein 2	2156	0
XM_020096106.1	rhodopsin	1079	0
XM_020096107.1	6-phosphofructo-2-kinase/fructose-2,6-bisphosphatase 2-like	3422	0
XM_020096108.1	6-phosphofructo-2-kinase/fructose-2,6-bisphosphatase 2-like	3330	0
XM_020096109.1	disks large-associated protein 4-like isoform X1	5510	0
XM_020096110.1	bromodomain adjacent to zinc finger domain protein 2A	10097	0
XM_020096111.1	protein bicaudal D homolog 2-like	4043	0
XM_020096112.1	DNA-(apurinic or apyrimidinic site) lyase isoform X2	1992	0
XM_020096113.1	DNA-(apurinic or apyrimidinic site) lyase isoform X2	1999	0
XM_020096114.1	sodium-coupled neutral amino acid transporter 3-like	2967	0
XM_020096115.1	sodium-coupled neutral amino acid transporter 3-like	2805	0
XM_020096116.1	eukaryotic translation initiation factor 3 subunit L isoform X1	2198	0
XM_020096117.1	sodium-coupled neutral amino acid transporter 3-like	2794	0
XM_020096118.1	neurofascin isoform X18	3893	0
XM_020096119.1	neurofascin isoform X18	4694	0
XM_020096120.1	calcium-independent phospholipase A2-gamma-like isoform X2	3402	0
XM_020096121.1	calcium-independent phospholipase A2-gamma-like isoform X2	3386	0
XM_020096122.1	calcium-independent phospholipase A2-gamma-like isoform X2	3246	0
XM_020096123.1	monocarboxylate transporter 2-like	3337	0
XM_020096124.1	acidic mammalian chitinase-like	2114	0
XM_020096125.1	eukaryotic translation initiation factor 3 subunit L isoform X1	2003	0
XM_020096126.1	nuclear factor erythroid 2-related factor 1	1789	0
XM_020096127.1	nuclear factor erythroid 2-related factor 1	1716	0
XM_020096128.1	heterogeneous nuclear ribonucleoprotein A1-like isoform X1	1453	5.02E-136
XM_020096129.1	heterogeneous nuclear ribonucleoprotein A1-like isoform X2	1443	6.68E-145
XM_020096130.1	chromobox protein homolog 5	2686	5.69E-85
XM_020096131.1	glucose-6-phosphate 1-dehydrogenase isoform X2	2586	0
XM_020096132.1	glucose-6-phosphate 1-dehydrogenase isoform X2	2728	0
XM_020096133.1	glucose-6-phosphate 1-dehydrogenase isoform X2	2526	0
XM_020096134.1	glycerol-3-phosphate dehydrogenase 1-like protein	2353	0
XM_020096135.1	nuclear pore membrane glycoprotein 210 isoform X1	7213	0
XM_020096136.1	nuclear pore membrane glycoprotein 210 isoform X2	7209	0
XM_020096137.1	fibulin-2-like isoform X1	4605	0
XM_020096138.1	fibulin-2-like isoform X1	4645	0
XM_020096139.1	fibulin-2-like isoform X1	4610	0
XM_020096140.1	fibulin-2-like isoform X1	4593	0
XM_020096141.1	nuclear receptor coactivator 6 isoform X1	7608	0
XM_020096142.1	nuclear receptor coactivator 6 isoform X2	7566	0
XM_020096143.1	protein TASOR-like isoform X1	5691	0
XM_020096144.1	protein TASOR-like isoform X2	5688	0
XM_020096145.1	acyl-CoA desaturase-like	2715	0
XM_020096146.1	glucose-induced degradation protein 8 homolog	1784	4.44E-141
XM_020096147.1	glucose-induced degradation protein 8 homolog	1876	1.74E-140
XM_020096148.1	nascent polypeptide-associated complex subunit alpha isoform X1	2383	2.31E-118
XM_020096149.1	nascent polypeptide-associated complex subunit alpha isoform X1	2392	2.86E-118
XM_020096150.1	nascent polypeptide-associated complex subunit alpha isoform X1	2740	4.04E-117
XM_020096151.1	nascent polypeptide-associated complex subunit alpha isoform X1	2341	1.60E-118
XM_020096152.1	nascent polypeptide-associated complex subunit alpha isoform X3	1588	0
XM_020096153.1	nascent polypeptide-associated complex subunit alpha isoform X3	1594	0
XM_020096154.1	nascent polypeptide-associated complex subunit alpha isoform X3	1881	0
XM_020096155.1	nascent polypeptide-associated complex subunit alpha isoform X4	862	4.13E-100
XM_020096156.1	acyl-CoA desaturase-like	2694	0
XM_020096157.1	nascent polypeptide-associated complex subunit alpha isoform X4	862	5.03E-100
XM_020096158.1	nascent polypeptide-associated complex subunit alpha isoform X4	785	1.58E-100
XM_020096159.1	cat eye syndrome critical region protein 5-like isoform X1	2992	0
XM_020096160.1	cat eye syndrome critical region protein 5-like isoform X2	2789	0
XM_020096161.1	platelet glycoprotein IX-like	1108	1.97E-162
XM_020096162.1	cellular nucleic acid-binding protein isoform X1	1496	4.72E-101
XM_020096163.1	cellular nucleic acid-binding protein isoform X1	1475	2.13E-105
XM_020096164.1	serine/threonine-protein kinase WNK2-like isoform X1	11328	0
XM_020096165.1	serine/threonine-protein kinase WNK2-like isoform X2	11325	0
XM_020096166.1	serine/threonine-protein kinase WNK2-like isoform X3	11325	0
XM_020096167.1	serine/threonine-protein kinase WNK2-like isoform X4	11229	0
XM_020096168.1	serine/threonine-protein kinase WNK2-like isoform X5	10896	0
XM_020096169.1	probable palmitoyltransferase ZDHHC16 isoform X2	1986	0
XM_020096170.1	rho-related GTP-binding protein RhoA-D	2433	1.02E-135
XM_020096171.1	protein FAM19A2-like	1726	1.32E-75
XM_020096172.1	ubiquitin-like modifier-activating enzyme 1	4201	0
XM_020096173.1	ubiquitin-like modifier-activating enzyme 1	3459	0
XM_020096174.1	ubiquitin-like modifier-activating enzyme 1	3431	0

XM_020096175.1	protein bicaudal D homolog 2-like	8240	0
XM_020096176.1	sentrin-specific protease 5-like isoform X2	3868	0
XM_020096177.1	RNA-binding motif, single-stranded-interacting protein 2-like isoform X1	4794	0
XM_020096178.1	probable palmitoyltransferase ZDHHC16 isoform X2	1672	0
XM_020096179.1	DEAD (Asp-Glu-Ala-Asp) box helicase 5-2c	3014	0
XM_020096180.1	voltage-dependent L-type calcium channel subunit beta-3-like isoform X1	4380	0
XM_020096181.1	voltage-dependent L-type calcium channel subunit beta-4-like isoform X2	1793	0
XM_020096182.1	solute carrier family 26 member 6-like	3607	0
XM_020096183.1	sodium- and chloride-dependent creatine transporter 1-like	2606	0
XM_020096184.1	nuclear receptor subfamily 1 group D member 1-like isoform X1	3771	0
XM_020096185.1	nuclear receptor subfamily 1 group D member 1-like isoform X1	3801	0
XM_020096186.1	probable palmitoyltransferase ZDHHC16 isoform X2	1634	0
XM_020096187.1	retinoic acid receptor gamma isoform X1	2454	0
XM_020096188.1	retinoic acid receptor gamma isoform X1	2005	0
XM_020096189.1	retinoic acid receptor gamma isoform X1	1912	0
XM_020096190.1	retinoic acid receptor gamma isoform X1	1975	0
XM_020096191.1	molybdate-anion transporter	2310	0
XM_020096192.1	SLC2A4 regulator-like isoform X1	8102	0
XM_020096193.1	zinc finger protein 704-like isoform X2	7944	0
XM_020096194.1	probable palmitoyltransferase ZDHHC16 isoform X2	1905	0

XM_020096195.1	PREDICTED: uncharacterized protein C3orf18-like	2451	6.48E-64
XM_020096196.1	PREDICTED: uncharacterized protein C3orf18-like	2700	1.46E-63
XM_020096197.1	PREDICTED: uncharacterized protein C3orf18-like	2720	1.55E-63
XM_020096198.1	high mobility group protein HMG-I/HMG-Y	2471	4.23E-12
XM_020096199.1	high mobility group protein HMG-I/HMG-Y	2523	4.33E-12
XM_020096200.1	metabotropic glutamate receptor 4-like	2914	0
XM_020096201.1	probable serine/threonine-protein kinase kinX isoform X1	2071	0
XM_020096202.1	probable serine/threonine-protein kinase kinX isoform X2	2071	0
XM_020096203.1	probable palmitoyltransferase ZDHHC16 isoform X2	1984	0
XM_020096204.1	sodium- and chloride-dependent taurine transporter	3673	0
XM_020096205.1	rhodopsin	1059	0
XM_020096206.1	protein kinase C-binding protein 1 isoform X3	4921	0
XM_020096207.1	protein kinase C-binding protein 1 isoform X3	4897	0
XM_020096208.1	protein kinase C-binding protein 1 isoform X3	4542	0
XM_020096209.1	protein kinase C-binding protein 1 isoform X3	4529	0
XM_020096210.1	protein kinase C-binding protein 1 isoform X3	4616	0
XM_020096211.1	protein kinase C-binding protein 1 isoform X4	4847	0
XM_020096212.1	protein kinase C-binding protein 1 isoform X5	4821	0
XM_020096213.1	1-phosphatidylinositol 4,5-bisphosphate phosphodiesterase gamma-1	6075	0
XM_020096214.1	DNA topoisomerase 1-like	3385	0
XM_020096215.1	protein phosphatase 1 regulatory subunit 3A-like isoform X3	3164	0
XM_020096216.1	protein phosphatase 1 regulatory subunit 3A-like isoform X3	1692	0
XM_020096217.1	histidine protein methyltransferase 1 homolog	1805	0
XM_020096218.1	protein phosphatase 1 regulatory subunit 3A-like isoform X3	1677	0
XM_020096219.1	protein phosphatase 1 regulatory subunit 3A-like isoform X3	1666	0
XM_020096220.1	testis-specific Y-encoded-like protein 1 isoform X1	3787	0
XM_020096221.1	testis-specific Y-encoded-like protein 1 isoform X2	3787	0
XM_020096222.1	testis-specific Y-encoded-like protein 1 isoform X3	3776	0
XM_020096223.1	testis-specific Y-encoded-like protein 1 isoform X4	3776	0
XM_020096224.1	testis-specific Y-encoded-like protein 1 isoform X1	3704	0
XM_020096225.1	testis-specific Y-encoded-like protein 1 isoform X4	3693	0
XM_020096226.1	ubiquitin carboxyl-terminal hydrolase 21-like	1596	0
XM_020096227.1	rho-related GTP-binding protein RhoA-C-like	2207	1.13E-136
XM_020096228.1	poly(rC)-binding protein 3-like isoform X1	2233	0
XM_020096229.1	poly(rC)-binding protein 3-like isoform X1	2212	0
XM_020096230.1	ecto-NOX disulfide-thiol exchanger 2 isoform X1	3009	0
XM_020096231.1	protocadherin alpha-8-like	2469	0
XM_020096232.1	WD repeat-containing protein 11	5546	0
XM_020096233.1	protein SSUH2 homolog isoform X1	1966	0
XM_020096234.1	protein SSUH2 homolog isoform X1	1919	0
XM_020096235.1	protein SSUH2 homolog isoform X1	1859	0
XM_020096236.1	60S ribosomal protein L32	535	1.96E-88
XM_020096237.1	DNA primase small subunit	1895	0
XM_020096238.1	rho-related GTP-binding protein Rho6-like	4414	2.49E-158
XM_020096239.1	collagen alpha-1(II) chain-like isoform X2	6375	0
XM_020096240.1	collagen alpha-1(II) chain-like isoform X2	6168	0
XM_020096241.1	YTH domain-containing family protein 1-like	4904	0
XM_020096242.1	death-inducer obliterator 1-like	12069	0
XM_020096243.1	CTTNBP2 N-terminal-like protein	3268	0
XM_020096244.1	SH3 and PX domain-containing protein 2A isoform X1	4675	0
XM_020096245.1	peregrin isoform X1	5142	0
XM_020096246.1	peregrin isoform X1	5087	0
XM_020096247.1	peregrin isoform X1	5318	0
XM_020096248.1	peregrin isoform X1	5052	0
XM_020096249.1	peregrin isoform X1	5133	0
XM_020096250.1	synaptotagmin-2-like isoform X1	7644	0
XM_020096251.1	synaptotagmin-2-like isoform X1	7618	0
XM_020096252.1	synaptotagmin-2-like isoform X1	7513	0
XM_020096253.1	synaptotagmin-2-like isoform X1	7577	0

XM_020096254.1	synaptotagmin-2-like isoform X1	7490	0
XM_020096255.1	SH3 and PX domain-containing protein 2A isoform X2	4592	0
XM_020096256.1	synaptotagmin-2-like isoform X1	7635	0
XM_020096257.1	protein VPRBP	6106	0
XM_020096258.1	protein VPRBP	6155	0
XM_020096259.1	transcriptional enhancer factor TEF-5-like	3938	0
XM_020096260.1	PREDICTED: dystroglycan-like	4775	0
XM_020096261.1	1-phosphatidylinositol 3-phosphate 5-kinase isoform X2	4708	0
XM_020096262.1	thyrotropin releasing hormone isoform X1	1559	4.73E-153
XM_020096263.1	thyrotropin releasing hormone isoform X2	1560	2.42E-152
XM_020096264.1	RAF proto-oncogene serine/threonine-protein kinase isoform X1	3319	0
XM_020096265.1	RAF proto-oncogene serine/threonine-protein kinase isoform X1	3079	0
XM_020096266.1	RAF proto-oncogene serine/threonine-protein kinase isoform X1	3447	0
XM_020096267.1	exportin-1 isoform X1	4363	0
XM_020096268.1	tumor protein p53-inducible nuclear protein 2 isoform X1	5045	0
XM_020096269.1	tumor protein p53-inducible nuclear protein 2 isoform X2	5042	0
XM_020096270.1	tumor protein p53-inducible nuclear protein 2 isoform X3	5042	0
XM_020096271.1	tumor protein p53-inducible nuclear protein 2 isoform X4	4634	7.49E-111
XM_020096272.1	glutamine-rich protein 1-like	3511	0
XM_020096273.1	glutamine-rich protein 1-like	3354	0
XM_020096274.1	receptor-type tyrosine-protein phosphatase beta-like	1756	0
XM_020096275.1	receptor-type tyrosine-protein phosphatase beta-like	1652	0
XM_020096276.1	receptor-type tyrosine-protein phosphatase beta-like	1734	0

XM_020096277.1	transcription initiation factor TFIID subunit 4-like	5272	0
XM_020096278.1	transcription initiation factor TFIID subunit 4-like	5268	0
XM_020096279.1	calcium-responsive transactivator isoform X1	3147	6.94E-130
XM_020096280.1	calcium-responsive transactivator isoform X2	3172	2.73E-131
XM_020096281.1	kelch-like protein 31	3016	0
XM_020096282.1	calcium-responsive transactivator isoform X3	3115	6.40E-103
XM_020096283.1	calcium-responsive transactivator isoform X4	3070	4.23E-107
XM_020096284.1	calcium-responsive transactivator isoform X5	2992	4.38E-109
XM_020096285.1	protein LSM14 homolog B-like	1294	0
XM_020096286.1	transketolase-like isoform X1	2430	0
XM_020096287.1	serine/threonine-protein phosphatase 4 regulatory subunit 2-like isoform X2	2258	0
XM_020096288.1	serine/threonine-protein phosphatase 4 regulatory subunit 2-like isoform X2	2353	0
XM_020096289.1	serine/threonine-protein phosphatase 4 regulatory subunit 2-like isoform X2	2249	0
XM_020096290.1	eukaryotic translation initiation factor 4E type 3-like	3011	1.00E-125
XM_020096291.1	pre-mRNA-processing factor 6	3820	0
XM_020096292.1	plexin-A1 isoform X1	6669	0
XM_020096293.1	kelch domain-containing protein 3	4911	0
XM_020096294.1	beta-1,4-galactosyltransferase 2 isoform X1	2339	0
XM_020096295.1	IQ motif and SEC7 domain-containing protein 1-like isoform X1	5477	0
XM_020096296.1	IQ motif and SEC7 domain-containing protein 1-like isoform X2	4078	0
XM_020096297.1	IQ motif and SEC7 domain-containing protein 1-like isoform X3	3543	0
XM_020096298.1	IQ motif and SEC7 domain-containing protein 1-like isoform X4	3490	0
XM_020096299.1	IQ motif and SEC7 domain-containing protein 1-like isoform X5	3453	0
XM_020096300.1	IQ motif and SEC7 domain-containing protein 1-like isoform X6	3556	0
XM_020096301.1	IQ motif and SEC7 domain-containing protein 1-like isoform X7	3653	0
XM_020096302.1	IQ motif and SEC7 domain-containing protein 1-like isoform X8	3821	0
XM_020096303.1	kelch domain-containing protein 3	4824	0
XM_020096304.1	IQ motif and SEC7 domain-containing protein 1-like isoform X2	3474	0
XM_020096305.1	glutaminase liver isoform, mitochondrial	3088	0
XM_020096306.1	probable E3 ubiquitin-protein ligase MID2 isoform X1	1605	6.41E-161
XM_020096307.1	semaphorin-3F-like isoform X4	2704	0
XM_020096308.1	semaphorin-3F-like isoform X4	3600	0
XM_020096309.1	wiskott-Aldrich syndrome protein isoform X1	1867	0
XM_020096310.1	wiskott-Aldrich syndrome protein isoform X1	1859	0
XM_020096311.1	kelch domain-containing protein 3	4900	0
XM_020096312.1	protein Wnt-5a isoform X1	3671	0
XM_020096313.1	protein Wnt-5a isoform X2	2934	0
XM_020096314.1	WD repeat-containing protein 46	2152	0
XM_020096315.1	cytosolic sulfotransferase 1-like	1778	0
XM_020096316.1	RNA-binding protein 10-like	3599	0
XM_020096317.1	toll-like receptor 9	4104	0
XM_020096318.1	acylamino-acid-releasing enzyme-like	2927	0
XM_020096319.1	kelch domain-containing protein 3	5099	0
XM_020096320.1	F-actin-capping protein subunit alpha-1-like	2254	0
XM_020096321.1	uridine-cytidine kinase-like 1 isoform X1	3926	0
XM_020096322.1	uridine-cytidine kinase-like 1 isoform X2	2114	0
XM_020096323.1	uridine-cytidine kinase-like 1 isoform X3	1810	0
XM_020096324.1	uridine-cytidine kinase-like 1 isoform X4	2043	0
XM_020096325.1	zinc finger protein 740 isoform X1	3740	1.39E-152
XM_020096326.1	zinc finger protein 740 isoform X1	3588	1.34E-140
XM_020096327.1	complement component receptor 1-like protein isoform X1	1591	0
XM_020096328.1	complement component receptor 1-like protein isoform X2	1588	0
XM_020096329.1	kelch domain-containing protein 3	4599	0
XM_020096330.1	probable ATP-dependent RNA helicase DHX35	3876	0
XM_020096331.1	microphthalmia-associated transcription factor-like isoform X1	2289	0
XM_020096332.1	microphthalmia-associated transcription factor-like isoform X2	2286	0

XM_020096333.1	microphthalmia-associated transcription factor-like isoform X2	1727	0
XM_020096334.1	microphthalmia-associated transcription factor-like isoform X4	1586	0
XM_020096335.1	PREDICTED: uncharacterized protein LOC109635260	1444	0
XM_020096336.1	proliferation-associated protein 2G4-like	2266	0
XM_020096337.1	transcription cofactor vestigial-like protein 4	3366	0
XM_020096338.1	transcription cofactor vestigial-like protein 4	3271	0
XM_020096339.1	PREDICTED: plakophilin-1	3597	0
XM_020096340.1	gamma-glutamyltransferase 7-like isoform X1	4160	0
XM_020096341.1	tRNA (uracil-5-)-methyltransferase homolog A	2592	0
XM_020096342.1	gamma-glutamyltransferase 7-like isoform X2	4136	0
XM_020096343.1	trichohyalin-like isoform X1	2948	0
XM_020096344.1	trichohyalin-like isoform X1	2973	0
XM_020096345.1	trichohyalin-like isoform X1	2915	0
XM_020096346.1	trichohyalin-like isoform X1	2960	0
XM_020096347.1	tumor necrosis factor receptor superfamily member 9-like	3285	2.31E-174
XM_020096348.1	translocating chain-associated membrane protein 1-like 1	2122	0
XM_020096349.1	sodium/hydrogen exchanger 8	3789	0
XM_020096350.1	mitogen-activated protein kinase kinase kinase 12-like isoform X1	5071	0
XM_020096351.1	mitogen-activated protein kinase kinase kinase 12-like isoform X2	5059	0
XM_020096352.1	TAF5-like RNA polymerase II p300/CBP-associated factor-associated factor 65 kDa subunit 5L isoform X2	1853	0
XM_020096353.1	TAF5-like RNA polymerase II p300/CBP-associated factor-associated factor 65 kDa subunit 5L isoform X2	1859	0
XM_020096354.1	dnaJ homolog subfamily B member 12-like	1862	0
XM_020096355.1	prostaglandin E synthase 3 isoform X1	1574	2.08E-94
XM_020096356.1	prostaglandin E synthase 3 isoform X2	1691	1.31E-89
XM_020096357.1	glutamine--tRNA ligase	2945	0
XM_020096358.1	next to BRCA1 gene 1 protein-like	3073	0
XM_020096359.1	zinc finger and BTB domain-containing protein 46	2521	0
XM_020096360.1	zinc finger and BTB domain-containing protein 46	2680	0
XM_020096361.1	zinc finger and BTB domain-containing protein 46	2677	0
XM_020096362.1	protocadherin gamma-C3-like	2286	0
XM_020096363.1	zinc finger and BTB domain-containing protein 46	2518	0
XM_020096364.1	zinc finger and BTB domain-containing protein 46	2621	0
XM_020096365.1	zinc finger and BTB domain-containing protein 46	2618	0
XM_020096366.1	zinc finger and BTB domain-containing protein 46	2633	0
XM_020096367.1	zinc finger and BTB domain-containing protein 46	2414	0
XM_020096368.1	PWWP domain-containing protein 2B	4098	0
XM_020096369.1	zinc finger and BTB domain-containing protein 46	2314	0
XM_020096370.1	zinc finger and BTB domain-containing protein 46	2430	0
XM_020096371.1	zinc finger and BTB domain-containing protein 46	2435	0
XM_020096372.1	zinc finger protein 271-like	2997	0
XM_020096373.1	zinc finger and BTB domain-containing protein 46-like	1152	9.72E-110
XM_020096374.1	WD repeat-containing protein 13 isoform X1	2450	0
XM_020096375.1	WD repeat-containing protein 13 isoform X2	2076	0
XM_020096376.1	WD repeat-containing protein 13 isoform X1	2256	0
XM_020096377.1	RNA-binding protein 39 isoform X1	1654	0
XM_020096378.1	RNA-binding protein 39 isoform X2	1639	0
XM_020096379.1	DNA (cytosine-5)-methyltransferase 3B-like isoform X1	6615	0
XM_020096380.1	PREDICTED: erlin-1	3266	0
XM_020096381.1	DNA (cytosine-5)-methyltransferase 3B-like isoform X1	6500	0
XM_020096382.1	DNA (cytosine-5)-methyltransferase 3B-like isoform X1	6464	0
XM_020096383.1	metabotropic glutamate receptor 7-like isoform X1	5968	0
XM_020096384.1	metabotropic glutamate receptor 7-like isoform X2	5983	0
XM_020096385.1	potassium voltage-gated channel subfamily A member 2-like	2433	0
XM_020096386.1	potassium voltage-gated channel subfamily A member 2-like	2250	0
XM_020096387.1	potassium voltage-gated channel subfamily A member 2-like	2184	0
XM_020096388.1	sodium- and chloride-dependent GABA transporter 1-like isoform X1	2521	0
XM_020096389.1	sodium- and chloride-dependent GABA transporter 1-like isoform X1	2441	0
XM_020096390.1	sodium- and chloride-dependent GABA transporter 1-like isoform X1	4908	0
XM_020096391.1	sodium- and chloride-dependent GABA transporter 1-like isoform X1	4098	0
XM_020096392.1	transcription factor MafB-like	3774	4.03E-101
XM_020096393.1	protein kinase C and casein kinase substrate in neurons protein 1-like	4300	0
XM_020096394.1	sperm flagellar protein 1	1246	2.52E-177
XM_020096395.1	protein kinase C and casein kinase substrate in neurons protein 1-like	4194	0
XM_020096396.1	SAM pointed domain-containing Ets transcription factor-like isoform X1	2299	0
XM_020096397.1	SAM pointed domain-containing Ets transcription factor-like isoform X1	2291	0
XM_020096398.1	SAM pointed domain-containing Ets transcription factor-like isoform X1	2296	0
XM_020096399.1	proline-rich transmembrane protein 3	3940	0
XM_020096400.1	proline-rich transmembrane protein 3	3656	0
XM_020096401.1	N-glycosylase/DNA lyase isoform X1	5211	0
XM_020096402.1	N-glycosylase/DNA lyase isoform X2	5196	0
XM_020096403.1	collagen alpha-3(IX) chain isoform X1	3114	0
XM_020096404.1	collagen alpha-3(IX) chain isoform X2	2511	0
XM_020096405.1	transcription factor-like 5 protein	2232	0
XM_020096406.1	transcription factor-like 5 protein	1928	0
XM_020096407.1	nidogen-2 isoform X6	4175	0
XM_020096408.1	transcription factor-like 5 protein	2180	0
XM_020096409.1	cadherin-4-like isoform X1	7060	0
XM_020096410.1	cadherin-4-like isoform X2	7036	0
XM_020096411.1	cadherin-4-like isoform X1	6821	0

XM_020096412.1	dual specificity protein phosphatase 7-like	2911	0
XM_020096413.1	immunoglobulin superfamily member 1-like	3920	0
XM_020096414.1	U-editing enzyme APOBEC-2	1176	1.21E-134
XM_020096415.1	deoxyribonuclease-1-like isoform X1	1264	0
XM_020096416.1	deoxyribonuclease-1-like isoform X2	1234	0
XM_020096417.1	proteolipid protein 2-like	1983	7.09E-92
XM_020096418.1	glutamate receptor-interacting protein 2-like isoform X1	5194	0
XM_020096419.1	glutamate receptor-interacting protein 2-like isoform X2	5123	0
XM_020096420.1	forkhead box protein N3	4759	0
XM_020096421.1	cysteine sulfinic acid decarboxylase isoform X1	2035	0
XM_020096422.1	cysteine sulfinic acid decarboxylase isoform X2	2032	0
XM_020096423.1	cysteine sulfinic acid decarboxylase isoform X1	2146	3.85E-153
XM_020096424.1	solute carrier family 12 member 5-like isoform X1	3367	0
XM_020096425.1	solute carrier family 12 member 5-like isoform X1	3340	0
XM_020096426.1	ammonium transporter Rh type B-A-like	2356	0
XM_020096427.1	rap1 GTPase-activating protein 1-like isoform X1	3977	0
XM_020096428.1	rap1 GTPase-activating protein 1-like isoform X2	3974	0
XM_020096429.1	rap1 GTPase-activating protein 1-like isoform X3	3959	0
XM_020096430.1	rap1 GTPase-activating protein 1-like isoform X4	3956	0
XM_020096431.1	rap1 GTPase-activating protein 1-like isoform X5	4498	0
XM_020096432.1	rap1 GTPase-activating protein 1-like isoform X6	3941	0
XM_020096433.1	stromal membrane-associated protein 1-like isoform X1	4359	8.24E-155
XM_020096434.1	rap1 GTPase-activating protein 1-like isoform X7	4447	0
XM_020096435.1	rap1 GTPase-activating protein 1-like isoform X8	4025	0
XM_020096436.1	rap1 GTPase-activating protein 1-like isoform X9	3845	0
XM_020096437.1	rap1 GTPase-activating protein 1-like isoform X11	3886	0
XM_020096438.1	rap1 GTPase-activating protein 1-like isoform X1	3876	0
XM_020096439.1	rap1 GTPase-activating protein 1-like isoform X1	3912	0
XM_020096440.1	rap1 GTPase-activating protein 1-like isoform X1	3860	0

XM_020096441.1	rap1 GTPase-activating protein 1-like isoform X1	3872	0
XM_020096442.1	rap1 GTPase-activating protein 1-like isoform X6	3840	0
XM_020096443.1	rap1 GTPase-activating protein 1-like isoform X6	3876	0
XM_020096444.1	stromal membrane-associated protein 1-like isoform X2	1553	8.45E-165
XM_020096445.1	rap1 GTPase-activating protein 1-like isoform X11	3847	0
XM_020096446.1	deoxynucleoside triphosphate triphosphohydrolase SAMHD1	2987	0
XM_020096447.1	deoxynucleoside triphosphate triphosphohydrolase SAMHD1	2926	0
XM_020096448.1	calcium-binding and coiled-coil domain-containing protein 1-like	4207	0
XM_020096449.1	potassium voltage-gated channel subfamily D member 3-like	3408	0
XM_020096450.1	5-hydroxytryptamine receptor 1-like	3534	0
XM_020096451.1	5'-nucleotidase domain-containing protein 2-like	2567	0
XM_020096452.1	stromal membrane-associated protein 1-like isoform X3	1553	1.45E-156
XM_020096453.1	EMILIN-2 isoform X2	3489	0
XM_020096454.1	leucine-rich repeat and transmembrane domain-containing protein 1	3133	0
XM_020096455.1	leucine-rich repeat-containing G-protein coupled receptor 6	4231	0
XM_020096456.1	ubiquitin-conjugating enzyme E2 T	923	6.39E-145
XM_020096457.1	potassium voltage-gated channel subfamily A member 10-like	2790	0
XM_020096458.1	potassium voltage-gated channel subfamily A member 10-like	3078	0
XM_020096459.1	protein snail homolog Sna-like	1797	4.89E-141
XM_020096460.1	stromal membrane-associated protein 1-like isoform X4	1553	8.45E-165
XM_020096461.1	protein AAR2 homolog	2240	0
XM_020096462.1	opioid growth factor receptor-like protein 1	3127	0
XM_020096463.1	CD9 antigen-like	1399	6.06E-133
XM_020096464.1	nuclear receptor subfamily 2 group C member 2	4014	0
XM_020096465.1	intraflagellar transport protein 122 homolog isoform X1	4597	0
XM_020096466.1	intraflagellar transport protein 122 homolog isoform X2	4501	0
XM_020096467.1	stromal membrane-associated protein 1-like isoform X5	1550	4.60E-164
XM_020096468.1	acid-sensing ion channel 1-like	4671	0
XM_020096469.1	guanine nucleotide-binding protein G(s) subunit alpha	3196	0
XM_020096470.1	F-actin-capping protein subunit alpha-1-like	1644	0
XM_020096471.1	stromal membrane-associated protein 1-like isoform X6	1487	3.95E-154
XM_020096472.1	UPF0160 protein MYG1, mitochondrial	1566	0
XM_020096473.1	UPF0160 protein MYG1, mitochondrial	1469	0
XM_020096474.1	PREDICTED: plakophilin-1-like	2931	0
XM_020096475.1	eyes absent homolog 2-like	2896	0
XM_020096476.1	coiled-coil domain-containing glutamate-rich protein 1-like	1788	3.25E-99
XM_020096477.1	tubulin beta chain-like	1535	0
XM_020096478.1	sphingomyelin phosphodiesterase 2	2469	0
XM_020096479.1	stromal membrane-associated protein 1-like isoform X7	1409	1.03E-156
XM_020096480.1	protein SSUH2 homolog	3242	0
XM_020096481.1	protein SSUH2 homolog	3308	0
XM_020096482.1	glutaminase liver isoform, mitochondrial-like isoform X1	2397	0
XM_020096483.1	glutaminase kidney isoform, mitochondrial-like isoform X2	2313	0
XM_020096484.1	PAK4-inhibitor INKA2 isoform X1	3360	0
XM_020096485.1	PAK4-inhibitor INKA2 isoform X2	3329	0
XM_020096486.1	glycerol-3-phosphate dehydrogenase 1-like protein	1597	0
XM_020096487.1	immunoglobulin superfamily member 21 isoform X1	2982	0
XM_020096488.1	protocadherin alpha-2-like	1698	0
XM_020096489.1	immunoglobulin superfamily member 21 isoform X2	2963	0
XM_020096490.1	protein HGH1 homolog	1828	0

XM_020096491.1	tribbles homolog 2-like	3124	0
XM_020096492.1	tribbles homolog 2-like	2642	0
XM_020096493.1	galactosylgalactosylxylosylprotein 3-beta-glucuronosyltransferase 1 isoform X2	3959	0
XM_020096494.1	PRELI domain containing protein 3B-like	1904	1.44E-135
XM_020096495.1	28S ribosomal protein S16, mitochondrial	915	1.63E-96
XM_020096496.1	BTB/POZ domain-containing protein KCTD6	1145	0
XM_020096497.1	BTB/POZ domain-containing protein KCTD6	1131	0
XM_020096498.1	vesicular inhibitory amino acid transporter-like	2331	0
XM_020096499.1	PREDICTED: leiomodin-1-like	3698	0
XM_020096500.1	protein phosphatase 1H-like	3527	0
XM_020096501.1	filamin-A-like isoform X4	1635	0
XM_020096502.1	protein APCDD1-like	2845	0
XM_020096503.1	nck-associated protein 1-like isoform X1	3605	0
XM_020096504.1	titin homolog	9303	0
XM_020096505.1	nck-associated protein 1-like isoform X2	3145	0
XM_020096506.1	biogenesis of lysosome-related organelles complex 1 subunit 1	1317	9.69E-67
XM_020096507.1	sodium-coupled monocarboxylate transporter 1-like	2574	0
XM_020096508.1	thyrotropin subunit beta	788	6.58E-100
XM_020096509.1	kinesin heavy chain isoform X1	5676	0
XM_020096510.1	kinesin heavy chain isoform X2	5646	0
XM_020096511.1	protein deglycase DJ-1	865	1.84E-135
XM_020096512.1	60S ribosomal protein L22-like	531	7.61E-56
XM_020096513.1	potassium voltage-gated channel subfamily A member 2-like	2808	0
XM_020096514.1	angiopoietin-4-like isoform X1	3872	0
XM_020096515.1	angiopoietin-4-like isoform X2	3688	0
XM_020096516.1	regulating synaptic membrane exocytosis protein 1-like isoform X1	7088	0
XM_020096517.1	U1 small nuclear ribonucleoprotein C	947	9.91E-42
XM_020096518.1	1,25-dihydroxyvitamin D(3) 24-hydroxylase, mitochondrial	2072	0
XM_020096519.1	NFX1-type zinc finger-containing protein 1	6386	0
XM_020096520.1	protein FAM19A2-like	1637	1.05E-79
XM_020096521.1	CD9 antigen-like	1678	2.46E-124
XM_020096522.1	adenosine receptor A1-like isoform X1	1736	0
XM_020096523.1	adenosine receptor A1-like isoform X2	1466	0
XM_020096524.1	regulating synaptic membrane exocytosis protein 1-like isoform X2	6737	0
XM_020096525.1	synaptonemal complex protein 1	3420	0
XM_020096526.1	synaptonemal complex protein 1	3318	0
XM_020096527.1	flocculation protein FLO11-like	970	1.45E-155
XM_020096528.1	paired box protein Pax-7-like	3570	0
XM_020096529.1	telomeric repeat-binding factor 2-interacting protein 1	2160	0
XM_020096530.1	ubiquitin-like modifier-activating enzyme ATG7	4037	0
XM_020096531.1	isoleucine--tRNA ligase, cytoplasmic	4583	0
XM_020096532.1	regulating synaptic membrane exocytosis protein 1-like isoform X3	6647	0
XM_020096533.1	PREDICTED: tropomyosin-like	888	0
XM_020096534.1	PREDICTED: uncharacterized protein LOC109635385	1887	8.95E-57
XM_020096535.1	calcium/calmodulin-dependent 3',5'-cyclic nucleotide phosphodiesterase 1B-like	1920	0
XM_020096536.1	collagen alpha-1(II) chain	5272	0
XM_020096537.1	mitogen-activated protein kinase kinase kinase 12-like	4499	0
XM_020096538.1	immunoglobulin superfamily member 3-like	2234	0
XM_020096539.1	protein-glutamine gamma-glutamyltransferase 5-like	2278	0
XM_020096540.1	N-terminal EF-hand calcium-binding protein 1-like	1262	0
XM_020096541.1	TOX high mobility group box family member 2	2025	0
XM_020096542.1	PREDICTED: uncharacterized protein LOC109635390	978	3.73E-153
XM_020096543.1	polyhomeotic-like protein 2	2484	0
XM_020096544.1	putative homeodomain transcription factor 2	2712	0
XM_020096545.1	metabotropic glutamate receptor 4-like	4982	0
XM_020096546.1	immunoglobulin-like and fibronectin type III domain-containing protein 1	4082	0
XM_020096547.1	immunoglobulin-like and fibronectin type III domain-containing protein 1	3494	0
XM_020096548.1	immunoglobulin-like and fibronectin type III domain-containing protein 1	3857	0
XM_020096549.1	kinesin-like protein KIF17	3565	0
XM_020096550.1	PREDICTED: uncharacterized protein C1orf158 homolog	654	5.50E-139
XM_020096551.1	arf-GAP with SH3 domain, ANK repeat and PH domain-containing protein 1-like	3763	0
XM_020096552.1	fer-1-like protein 4	6460	0
XM_020096553.1	phosphatase and actin regulator 2 isoform X1	5276	0
XM_020096554.1	copine-3-like isoform X1	2677	0
XM_020096555.1	pyruvate dehydrogenase E1 component subunit beta, mitochondrial	766	3.60E-174
XM_020096556.1	hydroxyacyl-thioester dehydratase type 2, mitochondrial-like	486	2.42E-109
XM_020096557.1	sarcolemmal membrane-associated protein-like isoform X1	6774	0
XM_020096558.1	filamin-A-like isoform X2	1015	1.86E-168
XM_020096559.1	serine/threonine-protein kinase 4	1477	0
XM_020096560.1	phosphatase and actin regulator 2 isoform X2	4926	0
XM_020096561.1	cullin-associated NEDD8-dissociated protein 1-like	1650	0
XM_020096562.1	---NA---	1646	
XM_020096563.1	purine nucleoside phosphorylase-like	1404	0
XM_020096564.1	monoacylglycerol lipase ABHD6	1590	0
XM_020096565.1	transcription factor HES-5-like	1445	6.62E-125
XM_020096566.1	60S acidic ribosomal protein P2-like	518	3.84E-40
XM_020096567.1	phosphatase and actin regulator 2 isoform X3	5026	0
XM_020096568.1	cadherin-22 isoform X1	3465	0
XM_020096569.1	cadherin-22 isoform X2	3420	0

XM_020096570.1	proline-rich protein 13-like	1670	7.95E-38
XM_020096571.1	type-1 angiotensin II receptor-associated protein	1035	1.08E-103
XM_020096572.1	monocarboxylate transporter 1-like isoform X1	2728	0
XM_020096573.1	monocarboxylate transporter 1-like isoform X2	2215	0
XM_020096574.1	potassium voltage-gated channel subfamily A member 3-like	1797	0
XM_020096575.1	SH3 and cysteine-rich domain-containing protein 3-like	2067	0
XM_020096576.1	SH3 and cysteine-rich domain-containing protein 3-like	1929	0
XM_020096577.1	potassium voltage-gated channel subfamily A member 10-like	3469	0
XM_020096578.1	potassium voltage-gated channel subfamily A member 10-like	3484	0
XM_020096579.1	cytochrome c oxidase subunit 6C-1	503	5.53E-50
XM_020096580.1	AT-rich interactive domain-containing protein 5B	5967	0
XM_020096581.1	neurexophilin-4 isoform X1	1795	0
XM_020096582.1	neurexophilin-4 isoform X1	1661	1.86E-169
XM_020096583.1	transcription factor HES-5-like	1049	2.61E-92
XM_020096584.1	protein shisa-4-like	1262	5.79E-119
XM_020096585.1	protein shisa-4-like	1253	5.22E-119
XM_020096586.1	SAM pointed domain-containing Ets transcription factor-like	2147	0
XM_020096587.1	SAM pointed domain-containing Ets transcription factor-like	1947	0
XM_020096588.1	complement C1q subcomponent subunit B-like	1227	1.20E-118
XM_020096589.1	complement C1q subcomponent subunit C-like	1312	1.68E-117
XM_020096590.1	anti-Muellerian hormone type-2 receptor	2025	0
XM_020096591.1	sphingomyelin synthase-related protein 1-like	3141	0
XM_020096592.1	nuclear factor of activated T-cells, cytoplasmic 2-like	3111	0
XM_020096593.1	LIM and cysteine-rich domains protein 1 isoform X1	2377	0
XM_020096594.1	LIM and cysteine-rich domains protein 1 isoform X2	2373	0
XM_020096595.1	complement C1q subcomponent subunit C-like	1077	1.00E-162
XM_020096596.1	PREDICTED: uncharacterized protein LOC109635438	1895	0
XM_020096597.1	protein phosphatase 1 regulatory subunit 1A isoform X1	1367	2.34E-151
XM_020096598.1	protein phosphatase 1 regulatory subunit 1A isoform X2	1352	7.82E-147
XM_020096599.1	protein phosphatase 1 regulatory subunit 1A isoform X3	1238	4.06E-117
XM_020096600.1	protein phosphatase 1 regulatory subunit 1A isoform X4	1223	1.12E-112
XM_020096601.1	complement factor H-like isoform X1	972	8.65E-164
XM_020096602.1	complement factor H-like isoform X2	880	3.51E-154
XM_020096603.1	protocadherin beta-16-like	2592	0
XM_020096604.1	dual specificity protein phosphatase 13-like	789	2.17E-145

XM_020096605.1	peroxisomal membrane protein 4	764	2.02E-160
XM_020096606.1	GTP-binding protein RAD	1356	0
XM_020096607.1	PREDICTED: guanylin-like	696	9.48E-63
XM_020096608.1	potassium voltage-gated channel subfamily D member 3-like	2255	0
XM_020096609.1	PREDICTED: guanylin-like	811	1.85E-61
XM_020096610.1	tubby-related protein 1-like	2428	0
XM_020096611.1	11-cis retinol dehydrogenase	1008	0
XM_020096612.1	adenosine receptor A1-like	1180	0
XM_020096613.1	dual specificity protein phosphatase 13-like	1736	1.40E-115
XM_020096614.1	vomeronasal type-1 receptor 3-like	1114	0
XM_020096615.1	vomeronasal type-1 receptor 3-like	1110	0
XM_020096616.1	leucine-rich repeat-containing protein 52-like	1893	0
XM_020096617.1	pro-FMRFamide-related neuropeptide FF isoform X1	889	1.08E-66
XM_020096618.1	pro-FMRFamide-related neuropeptide FF isoform X2	886	7.76E-66
XM_020096619.1	beta-nerve growth factor	891	2.92E-131
XM_020096620.1	dual specificity protein phosphatase 13-like	1454	7.42E-117
XM_020096621.1	vomeronasal type-1 receptor 3-like	942	0
XM_020096622.1	kelch domain-containing protein 7A	2283	0
XM_020096623.1	CMRF35-like molecule 8	1188	2.15E-167
XM_020096624.1	coiled-coil domain-containing protein 13-like	1598	0
XM_020096625.1	histamine H1 receptor	1655	0
XM_020096626.1	interleukin-17 receptor D	4012	0
XM_020096627.1	DCC-interacting protein 13-alpha	3799	0
XM_020096628.1	protein DENND6A	1818	0
XM_020096629.1	serum amyloid P-component-like	4102	9.56E-146
XM_020096630.1	endothelial PAS domain-containing protein 1	3374	0
XM_020096631.1	ADP-ribosylation factor 4	1253	5.71E-128
XM_020096632.1	nucleolar protein 8 isoform X1	4299	0
XM_020096633.1	nucleolar protein 8 isoform X2	4296	0
XM_020096634.1	nucleolar protein 8 isoform X3	4271	0
XM_020096635.1	nucleolar protein 8 isoform X3	4267	0
XM_020096636.1	nucleolar protein 8 isoform X3	4290	0
XM_020096637.1	nucleolar protein 8 isoform X3	4241	0
XM_020096638.1	nucleolar protein 8 isoform X3	4291	0
XM_020096639.1	nucleolar protein 8 isoform X3	4239	0
XM_020096640.1	extracellular matrix protein 2 isoform X3	4581	0
XM_020096641.1	extracellular matrix protein 2 isoform X3	4392	0
XM_020096642.1	extracellular matrix protein 2 isoform X3	4353	0
XM_020096643.1	tyrosine-protein kinase Mer-like	3323	0
XM_020096644.1	PREDICTED: asporin	2533	0
XM_020096645.1	centromere protein P	3761	1.00E-171
XM_020096646.1	centromere protein P	3648	4.15E-172
XM_020096647.1	epiphycan isoform X1	2682	0
XM_020096648.1	eukaryotic translation initiation factor 4 gamma 3 isoform X8	7909	0

XM_020096649.1	eukaryotic translation initiation factor 4 gamma 3 isoform X8	7906	0
XM_020096650.1	eukaryotic translation initiation factor 4 gamma 3 isoform X3	7907	0
XM_020096651.1	eukaryotic translation initiation factor 4 gamma 3 isoform X8	7900	0
XM_020096652.1	eukaryotic translation initiation factor 4 gamma 3 isoform X4	7290	0
XM_020096653.1	eukaryotic translation initiation factor 4 gamma 3 isoform X11	7888	0
XM_020096654.1	eukaryotic translation initiation factor 4 gamma 3 isoform X11	7883	0
XM_020096655.1	eukaryotic translation initiation factor 4 gamma 3 isoform X8	7269	0
XM_020096656.1	eukaryotic translation initiation factor 4 gamma 3 isoform X4	7891	0
XM_020096657.1	eukaryotic translation initiation factor 4 gamma 3 isoform X6	7885	0
XM_020096658.1	lutropin-choriogonadotropic hormone receptor-like	2241	0
XM_020096659.1	eukaryotic translation initiation factor 4 gamma 3 isoform X1	7863	0
XM_020096660.1	heterochromatin protein 1-binding protein 3 isoform X2	2897	0
XM_020096661.1	heterochromatin protein 1-binding protein 3 isoform X1	2834	0
XM_020096662.1	serine/threonine-protein phosphatase 4 regulatory subunit 1	5277	0
XM_020096663.1	ras-related protein Rab-5A-like	3419	1.99E-129
XM_020096664.1	plasma membrane calcium-transporting ATPase 1-like isoform X1	7561	0
XM_020096665.1	plasma membrane calcium-transporting ATPase 1-like isoform X1	7281	0
XM_020096666.1	plasma membrane calcium-transporting ATPase 1-like isoform X1	4476	0
XM_020096667.1	neuron navigator 1-like	9702	0
XM_020096668.1	troponin T, cardiac muscle isoforms-like	1192	4.82E-154
XM_020096669.1	cysteine and glycine-rich protein 1-like	1758	2.28E-106
XM_020096670.1	bifunctional 3'-phosphoadenosine 5'-phosphosulfate synthase 2-like	3019	0
XM_020096671.1	troponin I, slow skeletal muscle-like	814	4.23E-104
XM_020096672.1	putative helicase mov-10-B.2 isoform X1	4349	0
XM_020096673.1	putative helicase mov-10-B.2 isoform X1	4338	0
XM_020096674.1	rho-related GTP-binding protein RhoA-D-like	3135	5.99E-133
XM_020096675.1	intermediate filament family orphan 2 isoform X1	5459	0
XM_020096676.1	intermediate filament family orphan 2 isoform X2	5429	0
XM_020096677.1	voltage-gated potassium channel subunit beta-1-like isoform X1	1236	0
XM_020096678.1	mRNA turnover protein 4 homolog	1243	2.79E-171
XM_020096679.1	multiple epidermal growth factor-like domains protein 6	10417	0
XM_020096680.1	voltage-gated potassium channel subunit beta-2 isoform X1	6596	0
XM_020096681.1	voltage-gated potassium channel subunit beta-2 isoform X2	7280	0
XM_020096682.1	DNA excision repair protein ERCC-6	5003	0
XM_020096683.1	voltage-gated potassium channel subunit beta-2 isoform X3	6754	0
XM_020096684.1	voltage-gated potassium channel subunit beta-2 isoform X3	6586	0
XM_020096685.1	voltage-gated potassium channel subunit beta-2 isoform X3	6734	0
XM_020096686.1	voltage-gated potassium channel subunit beta-2 isoform X3	6766	0
XM_020096687.1	voltage-gated potassium channel subunit beta-2 isoform X3	6712	0
XM_020096688.1	sterol O-acyltransferase 2-like	3862	0
XM_020096689.1	desmin-like isoform X2	2281	0
XM_020096690.1	60S ribosomal protein L29	373	2.02E-34
XM_020096691.1	60S ribosomal protein L29	343	1.01E-33
XM_020096692.1	DNA excision repair protein ERCC-6	4427	0
XM_020096693.1	diphosphoinositol polyphosphate phosphohydrolase 1 isoform X1	4637	3.81E-89
XM_020096694.1	diphosphoinositol polyphosphate phosphohydrolase 1 isoform X2	4288	2.88E-74
XM_020096695.1	coiled-coil domain-containing protein 51	2300	0
XM_020096696.1	coiled-coil domain-containing protein 51	2369	0
XM_020096697.1	deoxyribonuclease-1-like isoform X2	1408	0
XM_020096698.1	deoxyribonuclease-1-like isoform X2	1423	0
XM_020096699.1	membrane-spanning 4-domains subfamily A member 8-like	1472	4.24E-155
XM_020096700.1	membrane-spanning 4-domains subfamily A member 8-like	1470	8.98E-155
XM_020096701.1	membrane-spanning 4-domains subfamily A member 8-like	1448	2.43E-155
XM_020096702.1	membrane-spanning 4-domains subfamily A member 8-like	1444	4.41E-155
XM_020096703.1	translation machinery-associated protein 7	772	2.19E-06
XM_020096704.1	PX domain-containing protein kinase-like protein isoform X1	4326	0
XM_020096705.1	SMC5-SMC6 complex localization factor protein 2-like isoform X1	4730	0
XM_020096706.1	PX domain-containing protein kinase-like protein isoform X2	4322	0
XM_020096707.1	PX domain-containing protein kinase-like protein isoform X3	4245	0
XM_020096708.1	PX domain-containing protein kinase-like protein isoform X4	2028	0
XM_020096709.1	ribonuclease P protein subunit p14	781	3.04E-91
XM_020096710.1	ribonuclease P protein subunit p14	857	7.54E-91
XM_020096711.1	protein TASOR isoform X1	6863	0
XM_020096712.1	protein TASOR isoform X2	6860	0
XM_020096713.1	protein TASOR isoform X3	7375	0
XM_020096714.1	protein TASOR isoform X4	7363	0
XM_020096715.1	SMC5-SMC6 complex localization factor protein 2-like isoform X2	4616	0
XM_020096716.1	rho guanine nucleotide exchange factor 3 isoform X1	3613	0
XM_020096717.1	rho guanine nucleotide exchange factor 3 isoform X2	3610	0
XM_020096718.1	rho guanine nucleotide exchange factor 3 isoform X3	3233	0
XM_020096719.1	rho guanine nucleotide exchange factor 3 isoform X4	3520	0
XM_020096720.1	membrane-associated guanylate kinase, WW and PDZ domain-containing protein 3-like	8100	0
XM_020096721.1	leucine-rich repeats and immunoglobulin-like domains protein 2	6770	0
XM_020096722.1	leucine-rich repeat-containing protein 3-like	5698	9.60E-173
XM_020096723.1	SMC5-SMC6 complex localization factor protein 2-like isoform X1	4695	0
XM_020096724.1	tyrosine-protein kinase yes	5304	0
XM_020096725.1	protein MANBAL	740	6.91E-48
XM_020096726.1	protein phosphatase 1 regulatory inhibitor subunit 16B	3134	0
XM_020096727.1	protein phosphatase 1 regulatory inhibitor subunit 16B	3408	0

XM_020096728.1	protocadherin beta-16-like	1474	0
XM_020096729.1	protein phosphatase 1 regulatory inhibitor subunit 16B	3042	0
XM_020096730.1	protein FAM210B	1925	0
XM_020096731.1	cytochrome c oxidase subunit NDUFA4-like isoform X1	749	4.74E-67
XM_020096732.1	cytochrome c oxidase subunit NDUFA4-like isoform X2	706	2.98E-55
XM_020096733.1	SMC5-SMC6 complex localization factor protein 2-like isoform X4	4539	0
XM_020096734.1	coiled-coil domain-containing protein 66 isoform X1	3282	0
XM_020096735.1	coiled-coil domain-containing protein 66 isoform X2	3279	0
XM_020096736.1	inositol hexakisphosphate kinase 1	5607	0
XM_020096737.1	inositol hexakisphosphate kinase 1	5604	0
XM_020096738.1	inositol hexakisphosphate kinase 1	6113	0
XM_020096739.1	ephrin type-B receptor 2 isoform X1	3281	0
XM_020096740.1	ephrin type-B receptor 2 isoform X2	3278	0
XM_020096741.1	ephrin type-B receptor 2 isoform X3	3272	0
XM_020096742.1	ephrin type-B receptor 2 isoform X4	3269	0
XM_020096743.1	synapsin-1-like isoform X1	4731	0
XM_020096744.1	synapsin-1-like isoform X1	4414	0
XM_020096745.1	zinc finger CCCH domain-containing protein 6	5203	0
XM_020096746.1	phosphatidate cytidylyltransferase, mitochondrial	1985	0
XM_020096747.1	metalloproteinase inhibitor 4	3057	6.17E-148
XM_020096748.1	metalloproteinase inhibitor 4	2864	8.87E-150
XM_020096749.1	poly(rC)-binding protein 2 isoform X1	2431	0
XM_020096750.1	poly(rC)-binding protein 2 isoform X1	2450	0
XM_020096751.1	poly(rC)-binding protein 2 isoform X1	2404	0
XM_020096752.1	poly(rC)-binding protein 2 isoform X1	1191	0
XM_020096753.1	transcription factor Sp1 isoform X1	3806	0
XM_020096754.1	transcription factor Sp1 isoform X2	3803	0
XM_020096755.1	cell division cycle-associated protein 7-like isoform X1	1357	0
XM_020096756.1	cell division cycle-associated protein 7-like isoform X2	1402	0
XM_020096757.1	immunoglobulin-like and fibronectin type III domain-containing protein 1 isoform X1	5190	0
XM_020096758.1	monocarboxylate transporter 9	3701	0
XM_020096759.1	immunoglobulin-like and fibronectin type III domain-containing protein 1 isoform X2	5085	0
XM_020096760.1	protein phosphatase 1 regulatory subunit 12B-like isoform X1	1015	7.47E-93
XM_020096761.1	protein phosphatase 1 regulatory subunit 12B-like isoform X1	1005	6.73E-93
XM_020096762.1	protein phosphatase 1 regulatory subunit 12B-like isoform X1	964	3.59E-67
XM_020096763.1	rap1 GTPase-activating protein 1-like isoform X1	4557	0
XM_020096764.1	rap1 GTPase-activating protein 1-like isoform X2	5331	0
XM_020096765.1	rap1 GTPase-activating protein 1-like isoform X3	4303	0
XM_020096766.1	protein-arginine deiminase type-2 isoform X1	2999	0
XM_020096767.1	monocarboxylate transporter 9	3717	0
XM_020096768.1	protein-arginine deiminase type-2 isoform X2	2925	0

XM_020096769.1	inhibin beta B chain-like isoform X1	3461	0
XM_020096770.1	inhibin beta B chain-like isoform X1	2903	0
XM_020096771.1	R3H domain-containing protein 1-like isoform X1	5065	0
XM_020096772.1	solute carrier family 2, facilitated glucose transporter member 1-like	1476	0
XM_020096773.1	transcriptional enhancer factor TEF-5-like isoform X1	4065	0
XM_020096774.1	transcriptional enhancer factor TEF-5-like isoform X2	4053	0
XM_020096775.1	transcriptional enhancer factor TEF-5-like isoform X3	4053	0
XM_020096776.1	monocarboxylate transporter 9	3547	0
XM_020096777.1	transcriptional enhancer factor TEF-5-like isoform X4	4044	0
XM_020096778.1	transcriptional enhancer factor TEF-5-like isoform X5	4008	0
XM_020096779.1	transcriptional enhancer factor TEF-5-like isoform X6	4002	0
XM_020096780.1	transcriptional enhancer factor TEF-5-like isoform X7	4002	0
XM_020096781.1	transcriptional enhancer factor TEF-5-like isoform X8	3996	0
XM_020096782.1	transcriptional enhancer factor TEF-5-like isoform X9	3990	0
XM_020096783.1	transcriptional enhancer factor TEF-5-like isoform X10	3990	0
XM_020096784.1	transcriptional enhancer factor TEF-5-like isoform X11	3987	0
XM_020096785.1	transcriptional enhancer factor TEF-5-like isoform X12	3981	0
XM_020096786.1	transcriptional enhancer factor TEF-5-like isoform X13	3981	0
XM_020096787.1	transcriptional enhancer factor TEF-5-like isoform X14	3912	0
XM_020096788.1	nuclear receptor coactivator 5 isoform X1	2821	0
XM_020096789.1	nuclear receptor coactivator 5 isoform X2	2818	0
XM_020096790.1	nuclear receptor coactivator 5 isoform X3	2809	0
XM_020096791.1	zinc finger CCCH domain-containing protein 15	2312	0
XM_020096792.1	nuclear receptor coactivator 5 isoform X4	2794	0
XM_020096793.1	nuclear receptor coactivator 5 isoform X5	2713	0
XM_020096794.1	nuclear receptor coactivator 5 isoform X6	2472	0
XM_020096795.1	nuclear receptor coactivator 5 isoform X1	2445	0
XM_020096796.1	ras-related protein Rap-1A-like	1915	6.74E-111
XM_020096797.1	ras-related protein Rap-1A-like	1815	2.65E-111
XM_020096798.1	ras-related protein Rap-1A-like	1750	1.39E-111
XM_020096799.1	ras-related protein Rap-1A-like	1832	3.05E-111
XM_020096800.1	plexin-A4 isoform X1	8194	0
XM_020096801.1	hematopoietic progenitor cell antigen CD34	2535	0
XM_020096802.1	PREDICTED: uncharacterized protein C6orf106 homolog	5091	0
XM_020096803.1	endothelin-converting enzyme 1	3111	0
XM_020096804.1	glutamate--cysteine ligase catalytic subunit	3241	0
XM_020096805.1	6-phosphofructo-2-kinase/fructose-2,6-bisphosphatase 2 isoform X1	2897	0
XM_020096806.1	6-phosphofructo-2-kinase/fructose-2,6-bisphosphatase 2 isoform X1	2923	0

XM_020096807.1	6-phosphofructo-2-kinase/fructose-2,6-bisphosphatase 2 isoform X1	2879	0
XM_020096808.1	6-phosphofructo-2-kinase/fructose-2,6-bisphosphatase 2 isoform X1	2729	0
XM_020096809.1	6-phosphofructo-2-kinase/fructose-2,6-bisphosphatase 2 isoform X1	2734	0
XM_020096810.1	6-phosphofructo-2-kinase/fructose-2,6-bisphosphatase 2 isoform X5	2711	0
XM_020096811.1	CMP-N-acetylneuraminate-beta-galactosamide-alpha-2,3-sialyltransferase 2-like	4985	0
XM_020096812.1	CMP-N-acetylneuraminate-beta-galactosamide-alpha-2,3-sialyltransferase 2-like	5039	0
XM_020096813.1	specifically androgen-regulated gene protein-like	1900	0
XM_020096814.1	SH2 domain-containing protein 5	1759	0
XM_020096815.1	SH2 domain-containing protein 5	1700	0
XM_020096816.1	tripartite motif-containing protein 2 isoform X1	2599	0
XM_020096817.1	eukaryotic translation initiation factor 4E-binding protein 2	3037	1.15E-72
XM_020096818.1	zinc finger protein ZPR1	2228	0
XM_020096819.1	serine hydroxymethyltransferase, mitochondrial	3156	0
XM_020096820.1	PREDICTED: importin-9	3718	0
XM_020096821.1	E3 ubiquitin-protein ligase Itchy-like	5341	0
XM_020096822.1	nischarin isoform X1	6191	0
XM_020096823.1	nischarin isoform X2	6182	0
XM_020096824.1	nischarin isoform X3	1818	0
XM_020096825.1	ranBP-type and C3HC4-type zinc finger-containing protein 1-like	2853	0
XM_020096826.1	TBC1 domain family member 20 isoform X1	3810	0
XM_020096827.1	TBC1 domain family member 20 isoform X2	1626	0
XM_020096828.1	protein kinase C and casein kinase substrate in neurons protein 1-like	3020	0
XM_020096829.1	zinc finger protein 518A-like	4615	0
XM_020096830.1	40S ribosomal protein S10	644	4.06E-101
XM_020096831.1	potassium voltage-gated channel subfamily S member 3	2561	0
XM_020096832.1	serine/threonine-protein kinase 3/4-like	3834	0
XM_020096833.1	sperm-specific antigen 2-like isoform X1	2328	0
XM_020096834.1	sperm-specific antigen 2-like isoform X1	2406	0
XM_020096835.1	RISC-loading complex subunit TARBP2 isoform X3	2486	0
XM_020096836.1	RISC-loading complex subunit TARBP2 isoform X3	2467	0
XM_020096837.1	RISC-loading complex subunit TARBP2 isoform X3	2595	0
XM_020096838.1	RISC-loading complex subunit TARBP2 isoform X3	2600	0
XM_020096839.1	alkaline phosphatase, tissue-nonspecific isozyme	1840	0
XM_020096840.1	peroxisome proliferator-activated receptor gamma	1772	0
XM_020096841.1	B-cell linker protein-like isoform X1	2453	0
XM_020096842.1	SWI/SNF-related matrix-associated actin-dependent regulator of chromatin subfamily D member 1	2737	0
XM_020096843.1	acid-sensing ion channel 1 isoform X1	1674	0
XM_020096844.1	acid-sensing ion channel 1 isoform X2	3948	0
XM_020096845.1	acid-sensing ion channel 1 isoform X3	3012	0
XM_020096846.1	solute carrier family 12 member 5-like isoform X1	4442	0
XM_020096847.1	solute carrier family 12 member 5-like isoform X2	4427	0
XM_020096848.1	solute carrier family 12 member 5-like isoform X3	4567	0
XM_020096849.1	putative N-acetyltransferase 8B	2125	4.08E-161
XM_020096850.1	putative N-acetyltransferase 8B	1992	2.27E-162
XM_020096851.1	B-cell linker protein-like isoform X2	2329	0
XM_020096852.1	putative N-acetyltransferase 8B	1949	3.53E-162
XM_020096853.1	stabilin-1 isoform X1	8731	0
XM_020096854.1	stabilin-1 isoform X2	7453	0
XM_020096855.1	tripartite motif-containing protein 35-like	2433	0
XM_020096856.1	CTTNBP2 N-terminal-like protein isoform X1	2802	0
XM_020096857.1	CTTNBP2 N-terminal-like protein isoform X1	2899	0
XM_020096858.1	protein Wnt-2b-A-like	3437	0
XM_020096859.1	protocadherin beta-1-like	1011	0
XM_020096860.1	plasma membrane calcium-transporting ATPase 3-like isoform X1	4313	0
XM_020096861.1	plasma membrane calcium-transporting ATPase 3-like isoform X1	5196	0
XM_020096862.1	ubiquitin thioesterase OTU1	3151	0
XM_020096863.1	ubiquitin thioesterase OTU1	3205	0
XM_020096864.1	ubiquitin thioesterase OTU1	3143	0
XM_020096865.1	erythroid differentiation-related factor 1 isoform X1	4812	0
XM_020096866.1	5-aminolevulinate synthase, nonspecific, mitochondrial	2271	0
XM_020096867.1	transcription cofactor vestigial-like protein 4 isoform X1	5827	1.42E-144
XM_020096868.1	transcription cofactor vestigial-like protein 4 isoform X2	5670	6.33E-139
XM_020096869.1	transcription cofactor vestigial-like protein 4 isoform X2	5596	2.51E-124
XM_020096870.1	vesicle-associated membrane protein-associated protein B-like	2871	4.64E-134
XM_020096871.1	aurora kinase A isoform X1	2885	0
XM_020096872.1	aurora kinase A isoform X1	2795	0
XM_020096873.1	aurora kinase A isoform X1	2879	0
XM_020096874.1	erythroid differentiation-related factor 1 isoform X2	4803	0
XM_020096875.1	sodium- and chloride-dependent GABA transporter 1-like isoform X1	4833	0
XM_020096876.1	coiled-coil domain-containing protein 71	3458	0
XM_020096877.1	mitochondrial glutamate carrier 1-like	2401	0
XM_020096878.1	dynactin subunit 2 isoform X1	2172	0
XM_020096879.1	dynactin subunit 2 isoform X2	2169	0
XM_020096880.1	dynactin subunit 2 isoform X3	2162	0
XM_020096881.1	mannose-1-phosphate guanyltransferase beta	2833	0
XM_020096882.1	sodium- and chloride-dependent GABA transporter 2-like	848	1.29E-138
XM_020096883.1	zinc finger E-box-binding homeobox 2-like	5060	0
XM_020096884.1	rho GTPase-activating protein 9 isoform X1	3425	0
XM_020096885.1	rho GTPase-activating protein 9 isoform X1	3395	0

XM_020096886.1	autophagy-related protein 16-1-like isoform X1	992	2.03E-157
XM_020096887.1	rho GTPase-activating protein 9 isoform X1	3371	0
XM_020096888.1	rho GTPase-activating protein 9 isoform X1	3278	0
XM_020096889.1	ankyrin repeat and SAM domain-containing protein 1A-like isoform X1	2622	0
XM_020096890.1	ankyrin repeat and SAM domain-containing protein 1A-like isoform X1	2641	0
XM_020096891.1	ankyrin repeat and SAM domain-containing protein 1A-like isoform X1	2621	0
XM_020096892.1	ankyrin repeat and SAM domain-containing protein 1A-like isoform X1	2714	0
XM_020096893.1	T-complex protein 11 homolog isoform X2	1675	0
XM_020096894.1	T-complex protein 11 homolog isoform X2	1732	0
XM_020096895.1	autophagy-related protein 16-1-like isoform X2	1009	2.80E-142
XM_020096896.1	T-complex protein 11 homolog isoform X2	1657	0
XM_020096897.1	T-complex protein 11 homolog isoform X2	1721	0
XM_020096898.1	platelet-activating factor acetylhydrolase IB subunit alpha	3472	0
XM_020096899.1	B9 domain-containing protein 2	835	3.61E-128
XM_020096900.1	prosaposin receptor GPR37L1-like	3114	0
XM_020096901.1	pantothenate kinase 4	3252	0
XM_020096902.1	methionine--tRNA ligase, cytoplasmic	3336	0
XM_020096903.1	rho guanine nucleotide exchange factor 10-like protein	3919	0
XM_020096904.1	transmembrane protein 263-like	1893	6.09E-47
XM_020096905.1	integrin alpha-7 isoform X1	4181	0
XM_020096906.1	integrin alpha-7 isoform X2	4156	0
XM_020096907.1	semaphorin-4G-like isoform X1	5659	0
XM_020096908.1	zinc finger protein GLI1	7149	0
XM_020096909.1	suppressor of tumorigenicity 7 protein-like	1713	0
XM_020096910.1	synaptotagmin-2-like isoform X1	5261	4.82E-152
XM_020096911.1	synaptotagmin-2-like isoform X1	5459	1.03E-151
XM_020096912.1	synaptotagmin-2-like isoform X1	5252	4.65E-152
XM_020096913.1	ceramide synthase 5	1786	0
XM_020096914.1	semaphorin-4G-like isoform X1	5091	0
XM_020096915.1	7SK snRNA methylphosphate capping enzyme-like isoform X1	1055	0
XM_020096916.1	cytochrome c oxidase assembly protein COX14 homolog	437	1.73E-36
XM_020096917.1	beta-1,3-galactosyl-O-glycosyl-glycoprotein beta-1,6-N-acetylglucosaminyltransferase 7-like	2437	0
XM_020096918.1	protein RTF2 homolog	1620	0
XM_020096919.1	alpha-1-syntrophin isoform X1	3013	0
XM_020096920.1	alpha-1-syntrophin isoform X2	1640	0
XM_020096921.1	dual specificity protein phosphatase 7	6120	0
XM_020096922.1	elastase 2 precursor	946	0
XM_020096923.1	elastase 1 precursor	986	6.21E-175
XM_020096924.1	arylacetamide deacetylase-like 4 isoform X1	1310	0
XM_020096925.1	arylacetamide deacetylase-like 4 isoform X2	1268	0
XM_020096926.1	Hermansky-Pudlak syndrome 6 protein	4904	0
XM_020096927.1	epidermal retinol dehydrogenase 2 isoform X1	1705	0
XM_020096928.1	rho GTPase-activating protein 40-like	4950	0
XM_020096929.1	transcription factor AP-2 gamma isoform X1	3006	0
XM_020096930.1	transcription factor AP-2 gamma isoform X2	2675	0
XM_020096931.1	transcription factor AP-2 gamma isoform X3	2933	0
XM_020096932.1	transcription factor AP-2 gamma isoform X4	2603	0

XM_020096933.1	protein shisa-4-like	2779	6.97E-91
XM_020096934.1	PREDICTED: draxin	2312	0
XM_020096935.1	PREDICTED: draxin	2293	0
XM_020096936.1	putative beta-lactamase-like 1	2883	0
XM_020096937.1	neuromedin-U receptor 2-like isoform X1	2452	0
XM_020096938.1	neuromedin-U receptor 2-like isoform X1	2635	0
XM_020096939.1	methylcytosine dioxygenase TET3 isoform X3	9259	0
XM_020096940.1	CCR4-NOT transcription complex subunit 6	2678	0
XM_020096941.1	myosin-binding protein H-like isoform X1	2231	0
XM_020096942.1	myosin-binding protein H-like isoform X1	2231	0
XM_020096943.1	myosin-binding protein H-like isoform X1	2192	0
XM_020096944.1	myosin-binding protein H-like isoform X1	2192	0
XM_020096945.1	myosin-binding protein H-like isoform X1	2153	0
XM_020096946.1	myosin-binding protein H-like isoform X1	2128	0
XM_020096947.1	myosin-binding protein H-like isoform X1	2095	0
XM_020096948.1	myosin-binding protein H-like isoform X1	2032	0
XM_020096949.1	myosin-binding protein H-like isoform X1	1975	0
XM_020096950.1	myosin-binding protein H-like isoform X2	1905	0
XM_020096951.1	dynein heavy chain 12, axonemal-like	2445	0
XM_020096952.1	V-type proton ATPase subunit S1-like	1145	0
XM_020096953.1	synaptopodin 2-like protein	5681	0
XM_020096954.1	40S ribosomal protein S23	568	2.33E-101
XM_020096955.1	mitochondrial import inner membrane translocase subunit Tim17-A	1410	1.80E-89
XM_020096956.1	DNA damage-inducible transcript 3 protein	1845	2.73E-115
XM_020096957.1	DNA damage-inducible transcript 3 protein	1836	2.50E-115
XM_020096958.1	periostin-like isoform X1	3124	0
XM_020096959.1	periostin-like isoform X1	3068	0
XM_020096960.1	UDP-glucuronosyltransferase 2C1-like isoform X3	1953	0
XM_020096961.1	UDP-glucuronosyltransferase 2C1-like isoform X3	1941	0
XM_020096962.1	UDP-glucuronosyltransferase 2C1-like isoform X3	2632	0
XM_020096963.1	UDP-glucuronosyltransferase 2C1-like isoform X3	2438	0
XM_020096964.1	F-box only protein 9 isoform X1	2374	0

XM_020096965.1	UDP-glucuronosyltransferase 2C1-like isoform X3	2267	0
XM_020096966.1	UDP-glucuronosyltransferase 2C1-like isoform X3	2461	0
XM_020096967.1	UDP-glucuronosyltransferase 2C1-like isoform X3	1975	0
XM_020096968.1	PREDICTED: claudin-4-like	1084	1.99E-133
XM_020096969.1	PREDICTED: delta-sarcoglycan-like, partial	721	2.24E-82
XM_020096970.1	superoxide dismutase [Cu-Zn]	847	1.10E-105
XM_020096971.1	fibroblast growth factor 13-like isoform X1	2724	2.43E-163
XM_020096972.1	fibroblast growth factor 13-like isoform X2	2158	4.47E-143
XM_020096973.1	F-box only protein 9 isoform X2	2371	0
XM_020096974.1	fibroblast growth factor 13-like isoform X3	1990	1.42E-138
XM_020096975.1	fibroblast growth factor 13-like isoform X2	2083	1.05E-131
XM_020096976.1	dolichyl-phosphate beta-glucosyltransferase	1674	0
XM_020096977.1	gamma-aminobutyric acid receptor subunit beta-2	1793	0
XM_020096978.1	gamma-aminobutyric acid receptor subunit alpha-6-like isoform X1	5024	0
XM_020096979.1	gamma-aminobutyric acid receptor subunit alpha-6-like isoform X2	4992	0
XM_020096980.1	tripartite motif-containing protein 3-like isoform X1	5193	0
XM_020096981.1	tripartite motif-containing protein 3-like isoform X1	5151	0
XM_020096982.1	F-box only protein 9 isoform X3	2334	0
XM_020096983.1	solute carrier family 22 member 5-like	3863	0
XM_020096984.1	lathosterol oxidase	1532	0
XM_020096985.1	ubiquitin-like protein 3	1425	1.07E-80
XM_020096986.1	protocadherin beta-16-like	1479	0
XM_020096987.1	claudin-like protein ZF-A89	810	3.85E-100
XM_020096988.1	solute carrier family 13 member 2-like	2021	0
XM_020096989.1	protein unc-119 homolog A-like	3673	4.76E-166
XM_020096990.1	F-box only protein 9 isoform X4	2352	0
XM_020096991.1	transcription factor 15-like	1726	6.46E-139
XM_020096992.1	ELMO domain-containing protein 1	3155	0
XM_020096993.1	tumor suppressor candidate 5 homolog	1658	2.86E-101
XM_020096994.1	claudin-like protein ZF-A89	1130	2.31E-125
XM_020096995.1	replication protein A 70 kDa DNA-binding subunit-like isoform X1	2566	0
XM_020096996.1	replication protein A 70 kDa DNA-binding subunit-like isoform X2	2123	0
XM_020096997.1	replication protein A 70 kDa DNA-binding subunit-like isoform X3	2509	0
XM_020096998.1	replication protein A 70 kDa DNA-binding subunit-like isoform X1	3039	0
XM_020096999.1	neutrophil cytosol factor 1 isoform X1	2151	0
XM_020097000.1	neutrophil cytosol factor 1 isoform X2	1782	0
XM_020097001.1	neutrophil cytosol factor 1 isoform X2	1752	0
XM_020097002.1	potassium voltage-gated channel subfamily KQT member 5-like isoform X1	2371	0
XM_020097003.1	calcium-binding protein 8-like	3567	4.09E-151
XM_020097004.1	protein Mis18-alpha	1440	9.34E-168
XM_020097005.1	ubiquitin-fold modifier 1	934	6.25E-56
XM_020097006.1	olfactory receptor 1M1-like isoform X1	2033	0
XM_020097007.1	olfactory receptor 2AT4-like protein	1369	0
XM_020097008.1	cytochrome c oxidase assembly factor 4 homolog, mitochondrial	640	1.17E-56
XM_020097009.1	cytochrome c oxidase assembly factor 4 homolog, mitochondrial	648	8.29E-57
XM_020097010.1	potassium voltage-gated channel subfamily KQT member 5-like isoform X2	2254	0
XM_020097011.1	SET and MYND domain-containing protein 4	2505	0
XM_020097012.1	transcription regulator protein BACH2-like	4471	0
XM_020097013.1	gamma-aminobutyric acid receptor subunit alpha-1-like	3264	0
XM_020097014.1	gamma-aminobutyric acid receptor subunit alpha-1-like	3555	0
XM_020097015.1	gamma-aminobutyric acid receptor subunit alpha-1-like	3245	0
XM_020097016.1	smoothelin-like protein 2 isoform X1	3126	0
XM_020097017.1	smoothelin-like protein 2 isoform X1	2941	0
XM_020097018.1	smoothelin-like protein 2 isoform X1	3123	0
XM_020097019.1	esterase OVCA2	1141	1.17E-163
XM_020097020.1	actin-binding LIM protein 3-like isoform X1	3497	0
XM_020097021.1	actin-binding LIM protein 3-like isoform X1	3610	0
XM_020097022.1	beta-1,3-glucosyltransferase-like isoform X1	2721	0
XM_020097023.1	CB1 cannabinoid receptor-interacting protein 1	2743	8.93E-112
XM_020097024.1	beta-1,3-glucosyltransferase-like isoform X2	2708	0
XM_020097025.1	beta-1,3-glucosyltransferase-like isoform X3	2603	0
XM_020097026.1	coagulation factor IX	2006	0
XM_020097027.1	solute carrier family 22 member 4-like	2569	0
XM_020097028.1	LIM domain kinase 1-like	2925	0
XM_020097029.1	proto-oncogene DBL-like	6173	0
XM_020097030.1	adenosine deaminase domain-containing protein 2-like isoform X1	1910	0
XM_020097031.1	adenosine deaminase domain-containing protein 2-like isoform X1	1831	0
XM_020097032.1	adenosine deaminase domain-containing protein 2-like isoform X1	1897	0
XM_020097033.1	FH2 domain-containing protein 1-like isoform X1	2712	0
XM_020097034.1	FH2 domain-containing protein 1-like isoform X2	2639	0
XM_020097035.1	FH2 domain-containing protein 1-like isoform X2	2664	0
XM_020097036.1	regulator of G-protein signaling 20-like isoform X1	7064	6.82E-125
XM_020097037.1	calpain-5 isoform X1	3980	0
XM_020097038.1	calpain-5 isoform X1	2064	0
XM_020097039.1	calpain-5 isoform X1	3746	0
XM_020097040.1	olfactory marker protein-like	834	8.47E-116
XM_020097041.1	coiled-coil domain-containing protein 92	1772	1.41E-146
XM_020097042.1	serine/threonine-protein kinase 32A-like isoform X1	3933	0
XM_020097043.1	serine/threonine-protein kinase 32A-like isoform X1	1523	0

XM_020097044.1	cytokine receptor common subunit gamma-like	1281	0
XM_020097045.1	unconventional myosin-VIIa-like	1839	0
XM_020097046.1	testican-2-like isoform X1	2244	0
XM_020097047.1	gem-associated protein 5-like	1840	0
XM_020097048.1	drebrin-like protein isoform X2	2695	0
XM_020097049.1	pleckstrin homology domain-containing family B member 1	558	3.70E-124
XM_020097050.1	cell adhesion molecule 1-like	1536	0
XM_020097051.1	calponin homology domain-containing protein DDB_G0272472-like	1717	0
XM_020097052.1	zinc finger and BTB domain-containing protein 16	1660	0
XM_020097053.1	zinc finger and BTB domain-containing protein 16-A-like	6365	1.02E-89
XM_020097054.1	testican-2-like isoform X2	2236	0
XM_020097055.1	5-hydroxytryptamine receptor 3A-like	1661	0
XM_020097056.1	5-hydroxytryptamine receptor 3A-like	1344	0
XM_020097057.1	cell surface glycoprotein MUC18-like	3777	0
XM_020097058.1	PREDICTED: uncharacterized protein LOC109635703	765	4.58E-152
XM_020097059.1	protocadherin Fat 3-like	2439	0
XM_020097060.1	hormonally up-regulated neu tumor-associated kinase	1911	0
XM_020097061.1	short transient receptor potential channel 4	4173	0
XM_020097062.1	UDP-glucuronosyltransferase 1-3-like	570	4.74E-123
XM_020097063.1	protein FAM111A-like	2489	0
XM_020097064.1	carbonic anhydrase 4-like	1705	0
XM_020097065.1	ecto-NOX disulfide-thiol exchanger 2 isoform X1	3136	0
XM_020097066.1	DNA damage-inducible transcript 4 protein	1190	2.04E-161
XM_020097067.1	Golgi SNAP receptor complex member 1-like	927	1.53E-126
XM_020097068.1	septin-5-like isoform X1	1630	0
XM_020097069.1	---NA---	1932	
XM_020097070.1	---NA---	784	
XM_020097071.1	PREDICTED: elastin-like	985	4.22E-13
XM_020097072.1	protein ABHD15-like	3801	0
XM_020097073.1	olfactory receptor 1M1-like	1020	0
XM_020097074.1	olfactory receptor 13G1-like	978	0
XM_020097075.1	olfactory receptor 1F1-like	972	0
XM_020097076.1	protein FAM149B1 isoform X1	3399	0
XM_020097077.1	olfactory receptor 52E8-like	957	0
XM_020097078.1	relaxin receptor 2	2896	0
XM_020097079.1	sister chromatid cohesion protein PDS5 homolog A isoform X1	4829	0
XM_020097080.1	solute carrier family 22 member 4-like	1709	0
XM_020097081.1	acetyl-coenzyme A synthetase 2-like, mitochondrial	1342	0
XM_020097082.1	visual system homeobox 2-like	1154	4.73E-148
XM_020097083.1	rho GTPase-activating protein 26	4555	0
XM_020097084.1	protein FAM149B1 isoform X2	3330	0
XM_020097085.1	phosphatidylinositol-binding clathrin assembly protein-like	4836	0
XM_020097086.1	complement component C1q receptor-like	2013	0
XM_020097087.1	interferon regulatory factor 9-like	546	2.01E-109
XM_020097088.1	beta-arrestin-2 isoform X1	1251	0
XM_020097089.1	beta-2 adrenergic receptor-like	1719	0
XM_020097090.1	ubiquitin-like domain-containing CTD phosphatase 1	885	2.48E-122
XM_020097091.1	stanniocalcin 2	1942	0
XM_020097092.1	PREDICTED: tyrosinase-like	1837	0
XM_020097093.1	four and a half LIM domains protein 1-like	1412	0
XM_020097094.1	four and a half LIM domains protein 1-like	1398	0
XM_020097095.1	dnaJ homolog subfamily C member 9	1211	4.55E-155
XM_020097096.1	PREDICTED: claudin-4-like	1173	6.43E-123

XM_020097097.1	sulfatase-modifying factor 2 isoform X1	1288	0
XM_020097098.1	sulfatase-modifying factor 2 isoform X2	1068	0
XM_020097099.1	solute carrier family 35 member F2	1828	0
XM_020097100.1	HAUS augmin-like complex subunit 1	1204	0
XM_020097101.1	coiled-coil domain-containing protein 169	1860	3.25E-135
XM_020097102.1	protocadherin beta-1-like	1304	0
XM_020097103.1	ankyrin repeat domain-containing protein SOWAHA-like	2254	0
XM_020097104.1	beta-crystallin A1-like	1134	7.36E-148
XM_020097105.1	solute carrier family 22 member 5-like isoform X1	1910	0
XM_020097106.1	WASH complex subunit FAM21-like isoform X1	5253	0
XM_020097107.1	solute carrier family 22 member 5-like isoform X2	1805	0
XM_020097108.1	spartin-like isoform X1	2194	0
XM_020097109.1	BTB/POZ domain-containing protein KCTD16 isoform X1	2853	0
XM_020097110.1	BTB/POZ domain-containing protein KCTD16 isoform X2	2859	0
XM_020097111.1	serine-rich and transmembrane domain-containing protein 1	1530	5.31E-18
XM_020097112.1	reticulon-4 receptor-like 1	4028	0
XM_020097113.1	glutaredoxin domain-containing cysteine-rich protein 2 isoform X3	1726	0
XM_020097114.1	WASH complex subunit FAM21-like isoform X1	5260	0
XM_020097115.1	glutaredoxin domain-containing cysteine-rich protein 2 isoform X3	1665	0
XM_020097116.1	glutaredoxin domain-containing cysteine-rich protein 2 isoform X3	1660	4.91E-171
XM_020097117.1	glutaredoxin domain-containing cysteine-rich protein 2 isoform X3	1854	8.53E-171
XM_020097118.1	glutaredoxin domain-containing cysteine-rich protein 2 isoform X3	1665	9.87E-172
XM_020097119.1	glutaredoxin domain-containing cysteine-rich protein 2 isoform X3	1602	4.73E-172
XM_020097120.1	F-box only protein 40-like isoform X1	2421	0
XM_020097121.1	F-box only protein 40-like isoform X2	2418	0
XM_020097122.1	cornifelin homolog B-like	1206	1.09E-63

XM_020097123.1	WASH complex subunit FAM21-like isoform X1	5250	0
XM_020097124.1	cyclin-A1 isoform X1	1398	0
XM_020097125.1	cyclin-A1 isoform X2	1405	0
XM_020097126.1	ras-related protein Rab-34-like	1286	0
XM_020097127.1	PLAC8-like protein 1	1453	2.41E-111
XM_020097128.1	D(1)-like dopamine receptor	1511	0
XM_020097129.1	fibroblast growth factor 1	2335	7.84E-93
XM_020097130.1	POU domain, class 4, transcription factor 3	1220	0
XM_020097131.1	WASH complex subunit FAM21-like isoform X1	5226	0
XM_020097132.1	leukocyte cell-derived chemotaxin-2-like isoform X2	1411	0
XM_020097133.1	leukocyte cell-derived chemotaxin-2-like isoform X2	1277	0
XM_020097134.1	leukocyte cell-derived chemotaxin-2-like isoform X2	1102	3.24E-118
XM_020097135.1	polypeptide N-acetylgalactosaminyltransferase 10-like	2434	0
XM_020097136.1	mRNA decay activator protein ZFP36L3-like	1171	7.87E-08
XM_020097137.1	mRNA decay activator protein ZFP36L3-like	1127	7.46E-08
XM_020097138.1	trophoblast glycoprotein-like	1551	0
XM_020097139.1	WASH complex subunit FAM21-like isoform X1	5193	0
XM_020097140.1	protocadherin Fat 3-like	1980	0
XM_020097141.1	interleukin-12 subunit beta-like	1231	0
XM_020097142.1	arachidonate 5-lipoxygenase-activating protein	560	5.39E-113
XM_020097143.1	mesenteric estrogen-dependent adipogenesis protein	971	7.46E-156
XM_020097144.1	poly(U)-specific endoribonuclease-B	932	0
XM_020097145.1	MAP3K7 C-terminal-like protein	1219	2.23E-82
XM_020097146.1	cytokine-like protein 1	503	1.41E-82
XM_020097147.1	alpha-actinin-2 isoform X1	3608	0
XM_020097148.1	dipeptidyl peptidase 3 isoform X2	3130	0
XM_020097149.1	dipeptidyl peptidase 3 isoform X2	2984	0
XM_020097150.1	dipeptidyl peptidase 3 isoform X2	2898	0
XM_020097151.1	ras and Rab interactor 3-like	3591	0
XM_020097152.1	RNA-binding protein 4-like isoform X1	5048	0
XM_020097153.1	RNA-binding protein 4-like isoform X1	5059	0
XM_020097154.1	RNA-binding protein 4-like isoform X1	5045	0
XM_020097155.1	RNA-binding protein 4-like isoform X1	1780	0
XM_020097156.1	alpha-actinin-2 isoform X2	2691	0
XM_020097157.1	SUMO-specific isopeptidase USPL1 isoform X1	4649	0
XM_020097158.1	SUMO-specific isopeptidase USPL1 isoform X2	4526	0
XM_020097159.1	high mobility group-T protein-like	1292	2.87E-96
XM_020097160.1	high mobility group-T protein-like	1253	1.49E-97
XM_020097161.1	transcription elongation factor SPT6	7279	0
XM_020097162.1	stromal cell-derived factor 2-like	2072	1.72E-146
XM_020097163.1	cytoplasmic polyadenylation element-binding protein 4-like isoform X2	7503	0
XM_020097164.1	cytoplasmic polyadenylation element-binding protein 4-like isoform X2	7477	0
XM_020097165.1	homeobox protein MSH-D-like	1846	0
XM_020097166.1	neuron-specific protein family member 2-like	2256	1.81E-112
XM_020097167.1	PREDICTED: uncharacterized protein LOC109635790	1176	6.01E-179
XM_020097168.1	leucine-rich repeat and WD repeat-containing protein 1 isoform X1	3587	0
XM_020097169.1	leucine-rich repeat and WD repeat-containing protein 1 isoform X1	3673	0
XM_020097170.1	leucine-rich repeat and WD repeat-containing protein 1 isoform X1	3561	0
XM_020097171.1	leucine-rich repeat and WD repeat-containing protein 1 isoform X1	3526	0
XM_020097172.1	leucine-rich repeat and WD repeat-containing protein 1 isoform X1	3526	0
XM_020097173.1	leucine-rich repeat and WD repeat-containing protein 1 isoform X1	3584	0
XM_020097174.1	3-methyl-2-oxobutanoate dehydrogenase [lipoamide] kinase, mitochondrial	5325	0
XM_020097175.1	alpha-ketoglutarate-dependent dioxygenase alkB homolog 4	1642	1.16E-123
XM_020097176.1	septin-8 isoform X2	2919	0
XM_020097177.1	PREDICTED: cyclin-I-like	2011	0
XM_020097178.1	PREDICTED: cyclin-I-like	1361	0
XM_020097179.1	ubiquitin thioesterase Zranb1-like	4166	0
XM_020097180.1	splicing factor, arginine/serine-rich 15 isoform X2	5183	0
XM_020097181.1	splicing factor, arginine/serine-rich 15 isoform X2	5168	0
XM_020097182.1	leucine--tRNA ligase, cytoplasmic	4417	0
XM_020097183.1	RNA-binding protein 27 isoform X1	4184	0
XM_020097184.1	RNA-binding protein 27 isoform X2	4001	0
XM_020097185.1	E3 ubiquitin-protein ligase CBL-like	5716	0
XM_020097186.1	ubiquitin thioesterase Zranb1-like	4008	0
XM_020097187.1	serine/threonine-protein kinase DCLK1-like isoform X1	4699	0
XM_020097188.1	serine/threonine-protein kinase DCLK1-like isoform X2	2754	0
XM_020097189.1	serine/threonine-protein kinase DCLK1-like isoform X1	4303	0
XM_020097190.1	serine/threonine-protein kinase DCLK1-like isoform X4	3654	0
XM_020097191.1	serine/threonine-protein kinase DCLK1-like isoform X5	3515	0
XM_020097192.1	serine/threonine-protein kinase DCLK1-like isoform X6	2926	0
XM_020097193.1	mothers against decapentaplegic homolog 3	2252	0
XM_020097194.1	regulatory factor X-associated protein	1529	2.50E-121
XM_020097195.1	transcription factor 7 isoform X1	3696	0
XM_020097196.1	ubiquitin thioesterase Zranb1-like	4205	0
XM_020097197.1	transcription factor 7 isoform X2	3647	0
XM_020097198.1	transcription factor 7 isoform X1	1683	0
XM_020097199.1	transcription factor 7 isoform X4	1682	0
XM_020097200.1	transcription factor 7 isoform X2	1635	0
XM_020097201.1	transcription factor 7 isoform X6	1634	0

XM_020097202.1	transcription factor 7 isoform X4	1653	0
XM_020097203.1	S-phase kinase-associated protein 1	1374	6.48E-88
XM_020097204.1	HIV Tat-specific factor 1	1805	0
XM_020097205.1	HIV Tat-specific factor 1	1747	0
XM_020097206.1	HIV Tat-specific factor 1	1645	0
XM_020097207.1	HIV Tat-specific factor 1	1580	0
XM_020097208.1	vesicle transport protein SEC20	1224	4.20E-107
XM_020097209.1	serine/threonine-protein kinase VRK1-like isoform X1	1997	0
XM_020097210.1	protein furry homolog isoform X1	9207	0
XM_020097211.1	protein furry homolog isoform X2	12174	0
XM_020097212.1	ZAR1-like protein	1253	0
XM_020097213.1	protein FAM57B-like	7994	0
XM_020097214.1	radixin-like isoform X1	3875	0
XM_020097215.1	radixin-like isoform X1	2031	0
XM_020097216.1	radixin-like isoform X1	1867	0
XM_020097217.1	serine/threonine-protein kinase VRK1-like isoform X1	1985	0
XM_020097218.1	PREDICTED: claudin-4-like	2151	0
XM_020097219.1	heat shock 70 kDa protein 4L	3488	0
XM_020097220.1	zinc finger CCHC domain-containing protein 10	1247	4.45E-34
XM_020097221.1	protocadherin beta-1-like	1226	0
XM_020097222.1	protein phosphatase Slingshot homolog 2-like isoform X1	5400	0
XM_020097223.1	protein phosphatase Slingshot homolog 2-like isoform X2	5195	0
XM_020097224.1	protein phosphatase Slingshot homolog 2-like isoform X3	5646	0
XM_020097225.1	cationic amino acid transporter 3-like	2955	0
XM_020097226.1	serine/threonine-protein kinase VRK1-like isoform X1	1994	0
XM_020097227.1	cationic amino acid transporter 3-like	2608	0
XM_020097228.1	cationic amino acid transporter 3-like	2289	0
XM_020097229.1	cationic amino acid transporter 3-like	4451	0
XM_020097230.1	ras-related protein Rab-39B-like	3594	6.21E-146
XM_020097231.1	ras-related protein Rab-38-like	1252	3.74E-146
XM_020097232.1	telomerase-binding protein EST1A	7200	0
XM_020097233.1	cullin-5 isoform X2	3330	0
XM_020097234.1	microfibrillar-associated protein 3-like	2461	0
XM_020097235.1	serine/threonine-protein kinase VRK1-like isoform X1	2034	0
XM_020097236.1	BICD family-like cargo adapter 1	2001	0
XM_020097237.1	BICD family-like cargo adapter 1	1895	0
XM_020097238.1	BICD family-like cargo adapter 1	1859	0
XM_020097239.1	BICD family-like cargo adapter 1	1866	0
XM_020097240.1	BICD family-like cargo adapter 1	1769	0
XM_020097241.1	protein ABHD11	3245	0
XM_020097242.1	synaptojanin-1 isoform X1	8071	0
XM_020097243.1	synaptojanin-1 isoform X2	8031	0
XM_020097244.1	synaptojanin-1 isoform X3	8022	0
XM_020097245.1	synaptojanin-1 isoform X4	8006	0
XM_020097246.1	synaptojanin-1 isoform X2	7949	0
XM_020097247.1	neurobeachin-like isoform X1	9617	0
XM_020097248.1	neurobeachin-like isoform X2	9614	0
XM_020097249.1	E3 ubiquitin-protein ligase FANCL isoform X1	1566	0
XM_020097250.1	neurobeachin-like isoform X3	9611	0
XM_020097251.1	neurobeachin-like isoform X4	9602	0
XM_020097252.1	neurobeachin-like isoform X5	9533	0
XM_020097253.1	neurobeachin-like isoform X6	9509	0
XM_020097254.1	neurobeachin-like isoform X7	9497	0
XM_020097255.1	neurobeachin-like isoform X8	9491	0
XM_020097256.1	neurobeachin-like isoform X9	9482	0
XM_020097257.1	neurobeachin-like isoform X10	9476	0
XM_020097258.1	neurobeachin-like isoform X11	9374	0
XM_020097259.1	neurobeachin-like isoform X12	9488	0
XM_020097260.1	E3 ubiquitin-protein ligase FANCL isoform X1	1479	0
XM_020097261.1	gamma-aminobutyric acid receptor subunit gamma-2 isoform X1	4726	0
XM_020097262.1	gamma-aminobutyric acid receptor subunit gamma-2 isoform X2	4723	0
XM_020097263.1	gamma-aminobutyric acid receptor subunit gamma-2 isoform X3	4702	0
XM_020097264.1	gamma-aminobutyric acid receptor subunit gamma-2 isoform X4	4699	0
XM_020097265.1	adapter molecule crk-like	3062	0
XM_020097266.1	janus kinase and microtubule-interacting protein 2-like isoform X1	4997	0
XM_020097267.1	janus kinase and microtubule-interacting protein 2-like isoform X1	4826	0
XM_020097268.1	E3 ubiquitin-protein ligase FANCL isoform X1	1484	0
XM_020097269.1	janus kinase and microtubule-interacting protein 2-like isoform X1	3046	0
XM_020097270.1	sodium- and chloride-dependent GABA transporter 2-like isoform X1	2137	0
XM_020097271.1	platelet-activating factor acetylhydrolase IB subunit alpha	2371	0
XM_020097272.1	clustered mitochondria protein homolog	4651	0
XM_020097273.1	fatty aldehyde dehydrogenase-like	2439	0
XM_020097274.1	PREDICTED: vitronectin-like	1647	0
XM_020097275.1	cone-rod homeobox protein-like	2497	9.23E-138
XM_020097276.1	RING finger protein 145	5896	0
XM_020097277.1	von Willebrand factor A domain-containing protein 7-like	2906	0
XM_020097278.1	von Willebrand factor A domain-containing protein 7-like	3157	0
XM_020097279.1	AN1-type zinc finger protein 5-like isoform X1	3686	0
XM_020097280.1	coronin-6-like isoform X1	2622	0

XM_020097281.1	coronin-6-like isoform X1	1640	0
XM_020097282.1	T-lymphoma invasion and metastasis-inducing protein 1-like	5204	0
XM_020097283.1	T-lymphoma invasion and metastasis-inducing protein 1-like	7034	0
XM_020097284.1	T-lymphoma invasion and metastasis-inducing protein 1-like	5211	0
XM_020097285.1	AF4/FMR2 family member 4 isoform X1	5697	0
XM_020097286.1	AF4/FMR2 family member 4 isoform X2	5679	0
XM_020097287.1	AF4/FMR2 family member 4 isoform X3	5673	0
XM_020097288.1	AF4/FMR2 family member 4 isoform X4	5580	0
XM_020097289.1	AF4/FMR2 family member 4 isoform X1	5567	0
XM_020097290.1	AF4/FMR2 family member 4 isoform X6	5655	0
XM_020097291.1	dihydropyrimidinase-related protein 3 isoform X1	3497	0
XM_020097292.1	dihydropyrimidinase-related protein 3 isoform X2	3479	0
XM_020097293.1	PDZ and LIM domain protein 3	1706	0
XM_020097294.1	glucocorticoid receptor-like	5158	0
XM_020097295.1	glucocorticoid receptor-like	5131	0
XM_020097296.1	protein Shroom1-like	7819	0
XM_020097297.1	protein Shroom1-like	7769	0
XM_020097298.1	protein Shroom1-like	7649	0
XM_020097299.1	protein Shroom1-like	7622	0
XM_020097300.1	histone deacetylase complex subunit SAP30L isoform X1	3052	3.17E-160
XM_020097301.1	histone deacetylase complex subunit SAP30L isoform X2	3257	6.71E-149
XM_020097302.1	14-3-3 protein epsilon-like	1934	1.48E-161
XM_020097303.1	ubiquitin carboxyl-terminal hydrolase 28 isoform X1	5439	0
XM_020097304.1	ubiquitin carboxyl-terminal hydrolase 28 isoform X2	5429	0
XM_020097305.1	ubiquitin carboxyl-terminal hydrolase 28 isoform X3	5436	0
XM_020097306.1	ubiquitin carboxyl-terminal hydrolase 28 isoform X4	5436	0
XM_020097307.1	neurogenic differentiation factor 2	809	1.73E-105
XM_020097308.1	serine/threonine-protein kinase TAO1-like	5887	0
XM_020097309.1	serine/threonine-protein kinase TAO1-like	5241	0
XM_020097310.1	serine/threonine-protein kinase TAO1-like	5211	0
XM_020097311.1	tubulin-specific chaperone cofactor E-like protein isoform X1	5215	0
XM_020097312.1	tubulin-specific chaperone cofactor E-like protein isoform X1	4810	0
XM_020097313.1	tubulin-specific chaperone cofactor E-like protein isoform X1	5241	0
XM_020097314.1	tubulin-specific chaperone cofactor E-like protein isoform X1	4682	0
XM_020097315.1	tubulin-specific chaperone cofactor E-like protein isoform X1	5113	0
XM_020097316.1	tubulin-specific chaperone cofactor E-like protein isoform X1	4583	0
XM_020097317.1	protein NipSnap homolog 2-like	2758	0
XM_020097318.1	28S ribosomal protein S17, mitochondrial	837	1.11E-98
XM_020097319.1	protein YIPF5	2532	0
XM_020097320.1	elongation factor 1-alpha 1	2186	0
XM_020097321.1	SH3 and PX domain-containing protein 2B isoform X1	4774	0
XM_020097322.1	SH3 and PX domain-containing protein 2B isoform X2	4738	0
XM_020097323.1	SH3 and PX domain-containing protein 2B isoform X3	4689	0
XM_020097324.1	SH3 and PX domain-containing protein 2B isoform X4	4653	0
XM_020097325.1	stAR-related lipid transfer protein 13 isoform X1	5873	0
XM_020097326.1	stAR-related lipid transfer protein 13 isoform X1	5776	0
XM_020097327.1	stAR-related lipid transfer protein 13 isoform X1	5734	0
XM_020097328.1	stAR-related lipid transfer protein 13 isoform X1	5789	0
XM_020097329.1	stAR-related lipid transfer protein 13 isoform X1	4892	0
XM_020097330.1	elongation factor 1-alpha 1	2379	0
XM_020097331.1	voltage-dependent anion-selective channel protein 1	1969	0
XM_020097332.1	DNA-directed RNA polymerase III subunit RPC9	1988	1.92E-81
XM_020097333.1	NEDD4 family-interacting protein 1 isoform X1	1976	8.53E-149
XM_020097334.1	NEDD4 family-interacting protein 1 isoform X2	1916	1.35E-133
XM_020097335.1	NEDD4 family-interacting protein 1 isoform X3	1904	1.84E-130
XM_020097336.1	PREDICTED: protocadherin-16-like	12439	0
XM_020097337.1	zinc-binding protein A33-like	2714	0
XM_020097338.1	arfaptin-2-like isoform X1	4341	0
XM_020097339.1	arfaptin-2-like isoform X2	4282	0
XM_020097340.1	arfaptin-2-like isoform X3	4285	0
XM_020097341.1	arfaptin-2-like isoform X4	4226	0
XM_020097342.1	serine/threonine-protein kinase 10-like	5191	0
XM_020097343.1	neuroblast differentiation-associated protein AHNAK-like isoform X1	4494	0
XM_020097344.1	T-complex protein 1 subunit zeta	2037	0
XM_020097345.1	nectin-3-like protein isoform X1	1807	0
XM_020097346.1	melatonin receptor type 1B-like	1101	0
XM_020097347.1	transmembrane protein 120A-like	1773	0
XM_020097348.1	sodium/hydrogen exchanger 6-like	2118	0
XM_020097349.1	calpain-1 catalytic subunit-like	2707	0
XM_020097350.1	neuroblast differentiation-associated protein AHNAK-like isoform X2	4461	0
XM_020097351.1	nuclear speckle splicing regulatory protein 1	1909	1.96E-148
XM_020097352.1	protein sprouty homolog 2-like	4811	0
XM_020097353.1	argininosuccinate lyase isoform X1	1670	0
XM_020097354.1	argininosuccinate lyase isoform X1	1578	0
XM_020097355.1	protocadherin beta-14-like	4599	0
XM_020097356.1	argininosuccinate lyase isoform X1	1710	0
XM_020097357.1	lactase-phlorizin hydrolase-like	3424	0
XM_020097358.1	replication factor C subunit 3	1526	0
XM_020097359.1	protein orai-2	3411	0

XM_020097360.1	CREB3 regulatory factor	10990	0
XM_020097361.1	PDZ and LIM domain protein 3	3353	0
XM_020097362.1	keratinocyte-associated transmembrane protein 2	2525	0
XM_020097363.1	cytochrome c-a-like	1529	4.13E-69
XM_020097364.1	glutamate receptor 4 isoform X1	4325	0
XM_020097365.1	glutamate receptor 4 isoform X2	3150	0
XM_020097366.1	glutamate receptor 4 isoform X3	4086	0
XM_020097367.1	glutamate receptor 4 isoform X4	2117	0
XM_020097368.1	glutamate receptor 4 isoform X5	4325	0
XM_020097369.1	polypeptide N-acetylgalactosaminyltransferase 10-like	5654	0
XM_020097370.1	DNA repair protein RAD50	5424	0
XM_020097371.1	PREDICTED: claudin-3-like	1732	5.72E-117
XM_020097372.1	protein eva-1 homolog C isoform X1	2089	0
XM_020097373.1	protein eva-1 homolog C isoform X2	2077	0
XM_020097374.1	protein eva-1 homolog C isoform X3	1911	0
XM_020097375.1	UPF0769 protein C21orf59 homolog	1360	0
XM_020097376.1	regulator of microtubule dynamics protein 2-like	4030	0
XM_020097377.1	E3 ubiquitin-protein ligase RNF14-like	2541	0
XM_020097378.1	sorting nexin-12 isoform X1	761	2.77E-118
XM_020097379.1	sorting nexin-12 isoform X1	1605	3.05E-112
XM_020097380.1	neural cell adhesion molecule 1-like isoform X1	3233	0
XM_020097381.1	neural cell adhesion molecule 1-like isoform X2	2835	0
XM_020097382.1	neural cell adhesion molecule 1-like isoform X3	2740	0
XM_020097383.1	neural cell adhesion molecule 1-like isoform X4	4071	0
XM_020097384.1	FRAS1-related extracellular matrix protein 2-like	11455	0
XM_020097385.1	regulator of microtubule dynamics protein 2-like	4040	0
XM_020097386.1	PREDICTED: claudin-4-like	1496	1.09E-109
XM_020097387.1	solute carrier family 46 member 3	2915	0
XM_020097388.1	PREDICTED: claudin-4-like	1184	2.33E-124
XM_020097389.1	transcription factor COE1 isoform X1	5939	0
XM_020097390.1	transcription factor COE1 isoform X2	5933	0
XM_020097391.1	transcription factor COE1 isoform X3	5870	0
XM_020097392.1	IgGFc-binding protein-like	5550	0
XM_020097393.1	ubiquitin carboxyl-terminal hydrolase 2-like	2131	0
XM_020097394.1	cysteine and histidine-rich domain-containing protein 1 isoform X1	2199	0
XM_020097395.1	cysteine and histidine-rich domain-containing protein 1 isoform X2	2056	0
XM_020097396.1	prolyl 4-hydroxylase subunit alpha-2 isoform X1	2931	0
XM_020097397.1	dapper homolog 2-like isoform X1	3287	0
XM_020097398.1	prolyl 4-hydroxylase subunit alpha-2 isoform X2	2870	0
XM_020097399.1	prolyl 4-hydroxylase subunit alpha-2 isoform X3	2865	0
XM_020097400.1	Sjoegren syndrome/scleroderma autoantigen 1	1807	1.53E-122
XM_020097401.1	protein FAM114A2-like	2882	0
XM_020097402.1	glutamate receptor ionotropic, kainate 1-like	3472	0
XM_020097403.1	metabotropic glutamate receptor 5-like	5201	0
XM_020097404.1	ras-related protein Rab-38-like	2042	9.30E-160
XM_020097405.1	dapper homolog 2-like isoform X2	3272	0
XM_020097406.1	F-box only protein 40-like	3099	0
XM_020097407.1	apoptosis-inducing factor 3-like isoform X1	1380	0
XM_020097408.1	apoptosis-inducing factor 3-like isoform X2	3913	0
XM_020097409.1	protein LIAT1 isoform X1	2206	2.50E-136
XM_020097410.1	protein LIAT1 isoform X2	1013	1.50E-133
XM_020097411.1	CCR4-NOT transcription complex subunit 8	1382	0
XM_020097412.1	actin filament-associated protein 1-like 1	3390	0
XM_020097413.1	ubiquitin domain-containing protein 2	1905	5.01E-170
XM_020097414.1	PREDICTED: claudin-4-like	2268	1.18E-155
XM_020097415.1	poly(ADP-ribose) glycohydrolase-like isoform X2	3589	0
XM_020097416.1	translation initiation factor eIF-2B subunit delta	1809	0
XM_020097417.1	poly(ADP-ribose) glycohydrolase-like isoform X2	3556	0
XM_020097418.1	fibronectin type III domain-containing protein 1 isoform X1	6724	0
XM_020097419.1	fibronectin type III domain-containing protein 1 isoform X2	6649	0
XM_020097420.1	fibronectin type III domain-containing protein 1 isoform X1	6349	0
XM_020097421.1	protein eva-1 homolog C-like	3258	0
XM_020097422.1	acetyl-CoA acetyltransferase, cytosolic	1898	0
XM_020097423.1	superoxide dismutase [Mn], mitochondrial	1260	2.55E-168
XM_020097424.1	osteopetrosis-associated transmembrane protein 1	2086	0
XM_020097425.1	lactation elevated protein 1	2355	0
XM_020097426.1	cartilage intermediate layer protein 1-like	1572	1.83E-166
XM_020097427.1	cartilage intermediate layer protein 1-like	1176	1.03E-169
XM_020097428.1	cartilage intermediate layer protein 1-like	1148	7.88E-169
XM_020097429.1	rab11 family-interacting protein 2	4705	0
XM_020097430.1	sialomucin core protein 24 isoform X1	1411	5.99E-38
XM_020097431.1	sialomucin core protein 24 isoform X2	1293	1.11E-37
XM_020097432.1	centrosomal protein of 57 kDa-like	2054	0
XM_020097433.1	testis development-related protein-like	2046	8.28E-123
XM_020097434.1	KATNB1-like protein 1	1377	0
XM_020097435.1	neuronal acetylcholine receptor subunit alpha-2-like	3473	0
XM_020097436.1	jun dimerization protein 2-like	1614	9.49E-99
XM_020097437.1	transcription factor AP-2-beta isoform X1	2112	0
XM_020097438.1	transcription factor AP-2-beta isoform X2	2259	0

XM_020097439.1	PREDICTED: laforin	2357	0
XM_020097440.1	F-box only protein 25 isoform X1	1803	0
XM_020097441.1	protein kinase C epsilon type-like	2973	0
XM_020097442.1	F-box only protein 25 isoform X2	1776	0
XM_020097443.1	clusterin-like protein 1	2164	0
XM_020097444.1	acyl-coenzyme A thioesterase 1-like	2157	0
XM_020097445.1	acyl-coenzyme A thioesterase 1-like	2133	0
XM_020097446.1	acyl-coenzyme A thioesterase 1-like	1971	0
XM_020097447.1	low molecular weight phosphotyrosine protein phosphatase	772	2.89E-113
XM_020097448.1	ras-related protein Rab-15-like	2093	6.11E-105
XM_020097449.1	leucine-rich alpha-2-glycoprotein-like	1448	3.14E-171
XM_020097450.1	leucine-rich alpha-2-glycoprotein-like	1261	0
XM_020097451.1	transmembrane protein 87A-like isoform X1	3442	0
XM_020097452.1	scavenger receptor class A member 3	3690	0
XM_020097453.1	PR domain zinc finger protein 1-like	2557	0
XM_020097454.1	PR domain zinc finger protein 1-like	2450	0
XM_020097455.1	methyltransferase-like protein 24	2473	0
XM_020097456.1	protein FAM110C	3284	0
XM_020097457.1	mitogen-activated protein kinase kinase kinase 5	4371	0
XM_020097458.1	PREDICTED: nesprin-2-like	5787	0
XM_020097459.1	transmembrane protein 87A-like isoform X2	3442	0
XM_020097460.1	protein jagged-2-like	1681	0
XM_020097461.1	protein CASC5	4665	0
XM_020097462.1	adenylate kinase 7-like	2066	0
XM_020097463.1	PREDICTED: formin-1	5024	0
XM_020097464.1	ryanodine receptor 3	15009	0
XM_020097465.1	serine/threonine-protein kinase pim-1-like	966	0
XM_020097466.1	probable G-protein coupled receptor 176	3158	0
XM_020097467.1	protocadherin beta-14-like	1353	0
XM_020097468.1	laminin subunit alpha-2-like	2915	0
XM_020097469.1	laminin subunit alpha-2-like	1987	0
XM_020097470.1	laminin subunit alpha-2-like	2243	0
XM_020097471.1	stress-induced-phosphoprotein 1-like isoform X1	4170	0
XM_020097472.1	vesicular inhibitory amino acid transporter-like	1932	0
XM_020097473.1	receptor-type tyrosine-protein phosphatase kappa	7089	0
XM_020097474.1	phospholipase B1, membrane-associated-like	3660	0
XM_020097475.1	probable arginine--tRNA ligase, mitochondrial	2136	0
XM_020097476.1	EF-hand domain-containing protein 1-like	2406	0
XM_020097477.1	ELM2 and SANT domain-containing protein 1-like	2373	2.52E-136
XM_020097478.1	PREDICTED: extensin-3-like	854	1.33E-100
XM_020097479.1	stress-induced-phosphoprotein 1-like isoform X2	4167	0
XM_020097480.1	apolipoprotein B-100-like	11497	0
XM_020097481.1	absent in melanoma 1 protein-like	7968	0
XM_020097482.1	single-minded homolog 1-like	3406	0
XM_020097483.1	glutamate receptor ionotropic, kainate 2-like	5481	0
XM_020097484.1	ectonucleotide pyrophosphatase/phosphodiesterase family member 1-like	3578	0
XM_020097485.1	PREDICTED: T-complex-associated testis-expressed protein 1	843	0
XM_020097486.1	PREDICTED: gephyrin, partial	1598	0
XM_020097487.1	synaptotagmin-14-like isoform X1	3457	0
XM_020097488.1	brain-enriched guanylate kinase-associated protein-like	668	1.51E-77
XM_020097489.1	mRNA-capping enzyme-like	1666	0
XM_020097490.1	myoferlin-like isoform X2	6946	0
XM_020097491.1	forkhead box protein O3-like	970	3.41E-110
XM_020097492.1	spectrin beta chain, non-erythrocytic 1-like	8889	0
XM_020097493.1	PREDICTED: nesprin-1-like	18337	0
XM_020097494.1	SAM and SH3 domain-containing protein 1-like	5413	0
XM_020097495.1	polyphosphoinositide phosphatase	3510	0
XM_020097496.1	NHS-like protein 1	820	0
XM_020097497.1	prospero homeobox protein 1-like	3123	0
XM_020097498.1	cGMP-dependent protein kinase 1-like	2030	0
XM_020097499.1	phospholipase ABHD3-like	3274	0
XM_020097500.1	transcription factor GATA-4-like	737	1.41E-93
XM_020097501.1	delta-1-pyrroline-5-carboxylate synthase-like isoform X1	3012	0
XM_020097502.1	nuclear receptor coactivator 7-like	3718	0
XM_020097503.1	protein-tyrosine kinase 2-beta-like isoform X1	3639	0
XM_020097504.1	low density lipoprotein receptor adapter protein 1-like	4351	0
XM_020097505.1	translation initiation factor eIF-2B subunit beta	1249	0
XM_020097506.1	all-trans retinoic acid-induced differentiation factor	1480	5.06E-168
XM_020097507.1	TATA box-binding protein-associated factor RNA polymerase I subunit A	2462	0
XM_020097508.1	TATA box-binding protein-associated factor RNA polymerase I subunit A	2482	0
XM_020097509.1	TATA box-binding protein-associated factor RNA polymerase I subunit A	2492	0
XM_020097510.1	transmembrane protein 18	899	1.15E-137
XM_020097511.1	EF-hand domain-containing protein 1-like	1839	0
XM_020097512.1	sialin isoform X1	3741	0
XM_020097513.1	basic leucine zipper transcriptional factor ATF-like	1197	7.79E-76
XM_020097514.1	6.8 kDa mitochondrial proteolipid	454	2.49E-31
XM_020097515.1	H2.0-like homeobox protein isoform X1	1958	0
XM_020097516.1	H2.0-like homeobox protein isoform X2	1805	0
XM_020097517.1	H2.0-like homeobox protein isoform X3	1619	4.66E-176

XM_020097518.1	adenylate kinase 9	5244	0
XM_020097519.1	general transcription factor IIH subunit 5	546	1.58E-45
XM_020097520.1	sialin isoform X2	3705	0
XM_020097521.1	cleavage and polyadenylation specificity factor subunit 6-like	1779	2.45E-30
XM_020097522.1	PREDICTED: syndecan-1-like	1340	8.30E-82
XM_020097523.1	histidine triad nucleotide-binding protein 3-like isoform X1	1036	3.49E-104
XM_020097524.1	histidine triad nucleotide-binding protein 3-like isoform X2	969	2.25E-86
XM_020097525.1	proto-oncogene c-Fos-like	1799	0
XM_020097526.1	potassium voltage-gated channel subfamily H member 1 isoform X1	3457	0
XM_020097527.1	transmembrane protein 121-like	2163	0
XM_020097528.1	prospero homeobox protein 1-like	2301	0
XM_020097529.1	synaptotagmin-like protein 4	1427	0
XM_020097530.1	DBH-like monooxygenase protein 2 homolog	2158	0
XM_020097531.1	ras-related protein Rab-23	4208	4.55E-148
XM_020097532.1	G-protein coupled receptor 12-like	1008	0
XM_020097533.1	potassium channel subfamily K member 3	1221	0
XM_020097534.1	melanocortin-2 receptor accessory protein 2A-like isoform X1	1410	6.33E-159
XM_020097535.1	melanocortin-2 receptor accessory protein 2A-like isoform X1	1375	1.15E-151
XM_020097536.1	protein ripply2-like	641	5.12E-69
XM_020097537.1	transcription factor AP-2-delta	1679	0
XM_020097538.1	protein FAM150B	994	8.07E-116
XM_020097539.1	angiopoietin-related protein 7-like	1302	0
XM_020097540.1	RNA polymerase-associated protein RTF1 homolog	989	0
XM_020097541.1	WD repeat-containing protein 37-like	1537	0
XM_020097542.1	apolipoprotein B-100-like	10313	0
XM_020097543.1	estrogen receptor	2862	0
XM_020097544.1	estrogen receptor	2217	0
XM_020097545.1	zinc finger protein 106-like isoform X1	4159	0
XM_020097546.1	zinc finger protein 106-like isoform X2	4153	0
XM_020097547.1	zinc finger protein 106-like isoform X3	4410	0
XM_020097548.1	zinc finger protein 106-like isoform X4	4042	0
XM_020097549.1	calpain-3-like isoform X1	2719	0
XM_020097550.1	calpain-3-like isoform X2	2704	0
XM_020097551.1	calpain-3-like isoform X3	2435	0
XM_020097552.1	calpain-3-like isoform X1	1306	0
XM_020097553.1	RNA-binding protein PNO1	1081	0
XM_020097554.1	nuclear receptor-binding protein	3734	0
XM_020097555.1	protein Churchill	684	5.29E-80
XM_020097556.1	muscarinic acetylcholine receptor M5	1731	0
XM_020097557.1	cell death regulator Aven	1068	6.10E-124
XM_020097558.1	ER membrane protein complex subunit 7	1607	1.64E-134
XM_020097559.1	RNA-binding protein 25-like isoform X1	3386	0
XM_020097560.1	eukaryotic translation initiation factor 2 subunit 1	1699	0
XM_020097561.1	V-type proton ATPase subunit D	1519	7.13E-166
XM_020097562.1	disheveled-associated activator of morphogenesis 1-like isoform X1	4650	0
XM_020097563.1	disheveled-associated activator of morphogenesis 1-like isoform X1	4919	0
XM_020097564.1	tetratricopeptide repeat protein 27	3000	0
XM_020097565.1	disheveled-associated activator of morphogenesis 1-like isoform X1	4620	0
XM_020097566.1	apoptosis-stimulating of p53 protein 1 isoform X1	7550	0
XM_020097567.1	apoptosis-stimulating of p53 protein 1 isoform X2	7526	0
XM_020097568.1	A-kinase anchor protein 12 isoform X1	6861	0
XM_020097569.1	A-kinase anchor protein 12 isoform X2	6761	0
XM_020097570.1	actin, alpha cardiac	1395	0
XM_020097571.1	denticleless protein homolog	4167	0
XM_020097572.1	serine/threonine-protein phosphatase 2A 56 kDa regulatory subunit alpha isoform	4478	0
XM_020097573.1	melanoma inhibitory activity protein 3 isoform X1	6090	0
XM_020097574.1	melanoma inhibitory activity protein 3 isoform X2	6045	0
XM_020097575.1	melanoma inhibitory activity protein 3 isoform X3	5976	0
XM_020097576.1	melanoma inhibitory activity protein 3 isoform X4	6203	0
XM_020097577.1	collagen alpha-1(XIII) chain-like	6224	0
XM_020097578.1	axin interactor, dorsalization-associated protein isoform X1	3158	0
XM_020097579.1	axin interactor, dorsalization-associated protein isoform X2	3149	0
XM_020097580.1	protein lin-9 homolog isoform X1	2140	0
XM_020097581.1	protein lin-9 homolog isoform X1	2526	0
XM_020097582.1	protocadherin gamma-B4-like	1536	0
XM_020097583.1	protein TMED8	3481	2.45E-126
XM_020097584.1	homeobox protein Mix.1-like	1068	0
XM_020097585.1	heat shock protein HSP 90-beta	2762	0
XM_020097586.1	reticulon-4-interacting protein 1 homolog, mitochondrial-like	2309	0
XM_020097587.1	utrophin isoform X1	11250	0
XM_020097588.1	utrophin isoform X1	11214	0
XM_020097589.1	mitochondrial import receptor subunit TOM20 homolog	1589	8.92E-73
XM_020097590.1	utrophin isoform X1	11211	0
XM_020097591.1	utrophin isoform X1	10708	0
XM_020097592.1	utrophin isoform X1	11160	0
XM_020097593.1	utrophin isoform X1	3268	0
XM_020097594.1	armadillo repeat-containing protein 2	3378	0
XM_020097595.1	armadillo repeat-containing protein 2	3279	0
XM_020097596.1	sestrin-1 isoform X1	3560	0

XM_020097597.1	sestrin-1 isoform X2	3395	0
XM_020097598.1	YLP motif-containing protein 1 isoform X1	6761	0
XM_020097599.1	YLP motif-containing protein 1 isoform X2	6737	0
XM_020097600.1	protein farnesyltransferase subunit beta-like	1712	0
XM_020097601.1	protein max isoform X1	1581	1.24E-83
XM_020097602.1	protein max isoform X2	1582	8.18E-83
XM_020097603.1	arginyl-tRNA--protein transferase 1 isoform X1	2325	0
XM_020097604.1	protein max isoform X3	1565	8.42E-80
XM_020097605.1	protein max isoform X4	1566	1.86E-78
XM_020097606.1	protein max isoform X5	1551	4.59E-87
XM_020097607.1	protein max isoform X6	1552	2.73E-86
XM_020097608.1	protein max isoform X7	1551	1.86E-74
XM_020097609.1	protein max isoform X8	1534	3.13E-83
XM_020097610.1	protein max isoform X9	1535	1.21E-81
XM_020097611.1	protein max isoform X10	1521	7.18E-78
XM_020097612.1	PREDICTED: androglobin	5791	0
XM_020097613.1	arginyl-tRNA--protein transferase 1 isoform X2	2326	0
XM_020097614.1	metabotropic glutamate receptor 1 isoform X1	5182	0
XM_020097615.1	metabotropic glutamate receptor 1 isoform X2	5737	0
XM_020097616.1	metabotropic glutamate receptor 1 isoform X3	4226	0
XM_020097617.1	metabotropic glutamate receptor 1 isoform X4	3810	0
XM_020097618.1	ras-related protein Rab-38-like	1868	1.33E-149
XM_020097619.1	E3 ubiquitin-protein ligase RNF19B-like isoform X1	4306	0
XM_020097620.1	E3 ubiquitin-protein ligase RNF19B-like isoform X2	4287	0
XM_020097621.1	zinc finger protein 513	3836	0
XM_020097622.1	arginyl-tRNA--protein transferase 1 isoform X3	2299	0
XM_020097623.1	ralBP1-associated Eps domain-containing protein 1	4136	0
XM_020097624.1	costars family protein ABRACL isoform X1	846	2.71E-78
XM_020097625.1	costars family protein ABRACL isoform X1	751	4.76E-52
XM_020097626.1	exostosin-like 3	6661	0
XM_020097627.1	tubby-related protein 4 isoform X1	9363	0
XM_020097628.1	tubby-related protein 4 isoform X2	9336	0
XM_020097629.1	28S ribosomal protein S10, mitochondrial	981	3.08E-161
XM_020097630.1	arginyl-tRNA--protein transferase 1 isoform X4	2295	0
XM_020097631.1	sterile alpha motif domain-containing protein 5	2043	5.05E-120
XM_020097632.1	cleavage and polyadenylation specificity factor subunit 2	2891	0
XM_020097633.1	cleavage and polyadenylation specificity factor subunit 2	2762	0
XM_020097634.1	bifunctional lysine-specific demethylase and histidyl-hydroxylase NO66-like	1958	0
XM_020097635.1	NADH dehydrogenase [ubiquinone] 1 beta subcomplex subunit 1 isoform X1	539	2.66E-83
XM_020097636.1	NADH dehydrogenase [ubiquinone] 1 beta subcomplex subunit 1 isoform X2	568	1.20E-67
XM_020097637.1	pumilio homolog 2-like isoform X1	4112	0
XM_020097638.1	pumilio homolog 2-like isoform X2	4109	0
XM_020097639.1	arginyl-tRNA--protein transferase 1 isoform X5	2455	0
XM_020097640.1	pumilio homolog 2-like isoform X3	4106	0
XM_020097641.1	pumilio homolog 2-like isoform X4	4041	0
XM_020097642.1	pumilio homolog 2-like isoform X5	4037	0
XM_020097643.1	pumilio homolog 2-like isoform X6	4035	0
XM_020097644.1	pleckstrin homology domain-containing family G member 1 isoform X1	7059	0
XM_020097645.1	pleckstrin homology domain-containing family G member 1 isoform X2	7102	0
XM_020097646.1	pleckstrin homology domain-containing family G member 1 isoform X1	6875	0
XM_020097647.1	rho-associated protein kinase 2-like isoform X1	5245	0
XM_020097648.1	rho-associated protein kinase 2-like isoform X2	5153	0
XM_020097649.1	serine/arginine-rich splicing factor 5-like	2511	2.28E-115
XM_020097650.1	adenylate cyclase type 8-like	4181	0
XM_020097651.1	protein EFR3 homolog B isoform X1	4596	0
XM_020097652.1	protein EFR3 homolog B isoform X2	4710	0
XM_020097653.1	integrin alpha-9	5458	0
XM_020097654.1	protein EFR3 homolog B isoform X3	4605	0
XM_020097655.1	laminin subunit alpha-2-like isoform X1	3240	0
XM_020097656.1	laminin subunit alpha-2-like isoform X2	3157	0
XM_020097657.1	regulator of G-protein signaling 7	3290	0
XM_020097658.1	regulator of G-protein signaling 7	1779	0
XM_020097659.1	sorting nexin-9 isoform X1	3582	0
XM_020097660.1	sorting nexin-9 isoform X1	3511	0
XM_020097661.1	sorting nexin-9 isoform X1	3504	0
XM_020097662.1	inositol polyphosphate 1-phosphatase	1865	0
XM_020097663.1	C-1-tetrahydrofolate synthase, cytoplasmic-like	4677	0
XM_020097664.1	oncoprotein-induced transcript 3 protein isoform X1	1785	0
XM_020097665.1	interleukin-31 receptor subunit alpha-like	3226	0
XM_020097666.1	interleukin-31 receptor subunit alpha-like	3115	0
XM_020097667.1	brain-enriched guanylate kinase-associated protein-like	3005	0
XM_020097668.1	retinol dehydrogenase 14-like	1449	0
XM_020097669.1	transforming protein RhoA	1376	6.07E-145
XM_020097670.1	potassium voltage-gated channel subfamily F member 1-like	2190	0
XM_020097671.1	protein disulfide-isomerase A6 isoform X1	2002	0
XM_020097672.1	protein disulfide-isomerase A6 isoform X2	2003	0
XM_020097673.1	oncoprotein-induced transcript 3 protein isoform X2	1750	0
XM_020097674.1	neurocalcin-delta A	1585	4.48E-140
XM_020097675.1	macoilin-1-like isoform X2	3559	0

XM_020097676.1	ammonium transporter Rh type A	2657	0
XM_020097677.1	cysteine-rich venom protein TEL1-like	729	2.26E-171
XM_020097678.1	zinc finger and BTB domain-containing protein 2	3539	0
XM_020097679.1	zinc finger and BTB domain-containing protein 2	3440	0
XM_020097680.1	signal-induced proliferation-associated 1-like protein 1	5733	0
XM_020097681.1	signal-induced proliferation-associated 1-like protein 1	7434	0
XM_020097682.1	dihydrolipoyllysine-residue succinyltransferase component of 2-oxoglutarate dehydrogenase complex, mitochondrial-like	2459	0
XM_020097683.1	PREDICTED: uncharacterized protein LOC109636127	1267	0
XM_020097684.1	group XIIB secretory phospholipase A2-like protein	2663	8.73E-171
XM_020097685.1	sodium/potassium/calcium exchanger 4 isoform X1	5105	0
XM_020097686.1	sodium/potassium/calcium exchanger 4 isoform X2	5099	0
XM_020097687.1	nuclear receptor subfamily 2 group E member 1	3139	0
XM_020097688.1	sorting nexin-3	1248	6.29E-116
XM_020097689.1	flap endonuclease GEN homolog 1	3228	0
XM_020097690.1	peptidyl-prolyl cis-trans isomerase-like 6 isoform X1	1606	0
XM_020097691.1	peptidyl-prolyl cis-trans isomerase-like 6 isoform X2	1600	0
XM_020097692.1	peptidyl-prolyl cis-trans isomerase-like 6 isoform X3	1412	0
XM_020097693.1	group XIIB secretory phospholipase A2-like protein	1431	1.26E-173
XM_020097694.1	peptidyl-prolyl cis-trans isomerase-like 6 isoform X4	1576	0
XM_020097695.1	peptidyl-prolyl cis-trans isomerase-like 6 isoform X5	1573	0
XM_020097696.1	mesoderm posterior protein 2-like	1594	1.87E-80
XM_020097697.1	sorting nexin-17	4198	0
XM_020097698.1	CMP-sialic acid transporter	1936	0
XM_020097699.1	uridine diphosphate glucose pyrophosphatase	3407	4.12E-148
XM_020097700.1	cannabinoid receptor type 1A isoform X1	3317	0
XM_020097701.1	cannabinoid receptor type 1A isoform X1	3322	0
XM_020097702.1	PREDICTED: akirin-2-like	1505	1.65E-128
XM_020097703.1	sushi domain-containing protein 6	3776	0
XM_020097704.1	pre-mRNA-splicing regulator WTAP	2032	0
XM_020097705.1	S-adenosylmethionine synthase-like	3248	0
XM_020097706.1	pre-mRNA-splicing regulator WTAP	1929	0
XM_020097707.1	forkhead box protein O3-like	5798	0
XM_020097708.1	protein lin-28 homolog B-like isoform X1	7043	2.73E-125
XM_020097709.1	protein lin-28 homolog B-like isoform X1	7038	2.73E-125
XM_020097710.1	protocadherin gamma-A9-like	1899	0
XM_020097711.1	protein lin-28 homolog B-like isoform X1	6472	4.91E-119
XM_020097712.1	protein lin-28 homolog B-like isoform X1	6416	9.11E-126
XM_020097713.1	rho GTPase-activating protein 11A-like	1469	0
XM_020097714.1	neuroendocrine protein 7B2	2665	4.31E-140
XM_020097715.1	PREDICTED: gremlin-1	1457	2.17E-102
XM_020097716.1	poly [ADP-ribose] polymerase 1	3950	0
XM_020097717.1	transmembrane emp24 domain-containing protein 10	2002	1.28E-136
XM_020097718.1	phosphofurin acidic cluster sorting protein 2-like	4392	0
XM_020097719.1	PREDICTED: alpha-(1,6)-fucosyltransferase	3967	0
XM_020097720.1	PREDICTED: alpha-(1,6)-fucosyltransferase	4177	0
XM_020097721.1	PREDICTED: alpha-(1,6)-fucosyltransferase	4081	0
XM_020097722.1	PREDICTED: alpha-(1,6)-fucosyltransferase	3871	0
XM_020097723.1	solute carrier family 22 member 6	4505	0
XM_020097724.1	uronyl 2-sulfotransferase	3077	0
XM_020097725.1	cytospin-A isoform X2	3007	0
XM_020097726.1	SAYSvFN domain-containing protein 1	1180	1.01E-67
XM_020097727.1	protein C10	854	1.51E-69
XM_020097728.1	protein C10	1093	6.48E-69
XM_020097729.1	steroid hormone receptor ERR2-like isoform X2	5315	0
XM_020097730.1	steroid hormone receptor ERR2-like isoform X2	1800	0
XM_020097731.1	paired box protein Pax-2a isoform X1	1975	0
XM_020097732.1	steroid hormone receptor ERR2-like isoform X2	1519	0
XM_020097733.1	transforming growth factor beta-3-like	3819	0
XM_020097734.1	transmembrane protein 151B-like	2330	0
XM_020097735.1	NF-kappa-B inhibitor alpha-like	2348	0
XM_020097736.1	sphingosine-1-phosphate phosphatase 1	3409	0
XM_020097737.1	F-box only protein 30-like isoform X1	3090	0
XM_020097738.1	F-box only protein 30-like isoform X1	3000	0
XM_020097739.1	F-box only protein 30-like isoform X1	3042	0
XM_020097740.1	paired box protein Pax-2a isoform X2	1963	0
XM_020097741.1	F-box only protein 30-like isoform X1	3010	0
XM_020097742.1	prolyl endopeptidase-like	2837	0
XM_020097743.1	transmembrane protein 181 isoform X1	2896	0
XM_020097744.1	transmembrane protein 181 isoform X2	2770	0
XM_020097745.1	dynein light chain Tctex-type 1 isoform X1	880	3.17E-60
XM_020097746.1	dynein light chain Tctex-type 1 isoform X1	980	8.81E-60
XM_020097747.1	dynein light chain Tctex-type 1 isoform X1	564	1.32E-79
XM_020097748.1	paired box protein Pax-2a isoform X3	1931	0
XM_020097749.1	feline leukemia virus subgroup C receptor-related protein 2 isoform X1	2283	0
XM_020097750.1	headcase protein homolog isoform X1	2407	0
XM_020097751.1	headcase protein homolog isoform X2	2261	0
XM_020097752.1	headcase protein homolog isoform X1	2250	0
XM_020097753.1	translation initiation factor IF-3, mitochondrial	991	0
XM_020097754.1	thrombospondin-2 isoform X2	5656	0

XM_020097755.1	T-complex protein 1 subunit alpha	2052	0
XM_020097756.1	paired box protein Pax-2a isoform X4	1919	0
XM_020097757.1	acyl-CoA:lysophosphatidylglycerol acyltransferase 1 isoform X1	4116	0
XM_020097758.1	acyl-CoA:lysophosphatidylglycerol acyltransferase 1 isoform X1	4437	0
XM_020097759.1	ribosomal protein S6 kinase delta-1 isoform X1	5262	0
XM_020097760.1	ribosomal protein S6 kinase delta-1 isoform X2	5109	0
XM_020097761.1	dual specificity protein phosphatase 10	5250	0
XM_020097762.1	kinesin-like protein KIF25 isoform X1	2952	0
XM_020097763.1	kinesin-like protein KIF25 isoform X1	2866	0
XM_020097764.1	paired box protein Pax-2a isoform X5	1940	0
XM_020097765.1	coiled-coil domain-containing protein 167	1326	2.23E-59
XM_020097766.1	PREDICTED: nesprin-2-like	8067	0
XM_020097767.1	wiskott-Aldrich syndrome protein family member 1 isoform X1	3555	0
XM_020097768.1	wiskott-Aldrich syndrome protein family member 1 isoform X2	3375	0
XM_020097769.1	ATP-binding cassette sub-family D member 4	2697	0
XM_020097770.1	acetyl-coenzyme A synthetase 2-like, mitochondrial	1169	0
XM_020097771.1	adenosine 3'-phospho 5'-phosphosulfate transporter 1	2588	0
XM_020097772.1	paired box protein Pax-2a isoform X5	1288	0
XM_020097773.1	mRNA-capping enzyme-like	756	1.84E-51
XM_020097774.1	E3 ubiquitin-protein ligase RNF8 isoform X1	2878	0
XM_020097775.1	E3 ubiquitin-protein ligase RNF8 isoform X2	1724	0
XM_020097776.1	dynein regulatory complex protein 1 isoform X1	4427	0
XM_020097777.1	dynein regulatory complex protein 1 isoform X1	4411	0
XM_020097778.1	fer-1-like protein 6	8036	0
XM_020097779.1	solute carrier family 35 member F6	2431	0
XM_020097780.1	paired box protein Pax-2a isoform X5	1335	0
XM_020097781.1	sine oculis-binding protein homolog isoform X1	5099	0
XM_020097782.1	sine oculis-binding protein homolog isoform X2	5078	0
XM_020097783.1	adhesion G-protein coupled receptor G2-like	2496	0
XM_020097784.1	PREDICTED: uncharacterized protein LOC109636191	878	3.86E-145
XM_020097785.1	squalene synthase	4186	0
XM_020097786.1	structural maintenance of chromosomes protein 6	3748	0
XM_020097787.1	structural maintenance of chromosomes protein 6	3744	0
XM_020097788.1	paired box protein Pax-2a isoform X6	1912	0
XM_020097789.1	structural maintenance of chromosomes protein 6	3711	0
XM_020097790.1	structural maintenance of chromosomes protein 6	3722	0
XM_020097791.1	PREDICTED: uncharacterized protein C1orf115 homolog	870	6.50E-43
XM_020097792.1	syntaxin-binding protein 5-like	6120	0
XM_020097793.1	zinc finger CCCH domain-containing protein 14 isoform X1	2419	0
XM_020097794.1	zinc finger CCCH domain-containing protein 14 isoform X2	2415	0
XM_020097795.1	zinc finger CCCH domain-containing protein 14 isoform X3	2416	0
XM_020097796.1	NHS-like protein 1	1805	5.12E-133
XM_020097797.1	paired box protein Pax-2a isoform X7	1900	0
XM_020097798.1	lysosomal-associated transmembrane protein 4A	2119	5.73E-146
XM_020097799.1	cap-specific mRNA (nucleoside-2'-O-)-methyltransferase 1	3625	0
XM_020097800.1	PREDICTED: syntaxin-11-like	1523	0
XM_020097801.1	PREDICTED: syntaxin-11-like	1294	0
XM_020097802.1	PREDICTED: syntaxin-11-like	1884	0
XM_020097803.1	PREDICTED: uncharacterized protein LOC109636203 isoform X1	1412	1.18E-166
XM_020097804.1	PREDICTED: uncharacterized protein LOC109636203 isoform X1	1438	2.57E-167
XM_020097805.1	PREDICTED: uncharacterized protein LOC109636203 isoform X2	709	2.37E-150
XM_020097806.1	nucleoside diphosphate kinase 6 isoform X1	1826	8.97E-132
XM_020097807.1	nucleoside diphosphate kinase 6 isoform X1	1992	3.71E-130
XM_020097808.1	brain and acute leukemia cytoplasmic protein-like	913	5.88E-119
XM_020097809.1	integrator complex subunit 7	3760	0
XM_020097810.1	cathepsin B-like	1036	0
XM_020097811.1	hydroperoxide isomerase ALOXE3-like	952	7.17E-178
XM_020097812.1	hydroperoxide isomerase ALOXE3-like	2094	0
XM_020097813.1	protein RD3	1295	5.66E-143
XM_020097814.1	protein RD3	1233	2.76E-143
XM_020097815.1	protein FAM133-like	2092	1.34E-06
XM_020097816.1	tau-tubulin kinase 2-like isoform X1	3470	0
XM_020097817.1	tau-tubulin kinase 2-like isoform X2	3467	0
XM_020097818.1	translocating chain-associated membrane protein 2	1890	0
XM_020097819.1	potassium channel subfamily K member 2	1820	0
XM_020097820.1	protein SERAC1	2837	0
XM_020097821.1	myc target protein 1	1311	1.68E-84
XM_020097822.1	myc target protein 1	1186	4.58E-85
XM_020097823.1	MAGUK p55 subfamily member 5 isoform X1	3080	0
XM_020097824.1	MAGUK p55 subfamily member 5 isoform X1	3093	0
XM_020097825.1	MAGUK p55 subfamily member 5 isoform X1	3068	0
XM_020097826.1	protein-glutamate O-methyltransferase	2378	0
XM_020097827.1	PREDICTED: pro-opiomelanocortin-like	3575	2.92E-124
XM_020097828.1	heme-binding protein 2-like	2212	3.73E-139
XM_020097829.1	heme-binding protein 2-like	2052	7.84E-140
XM_020097830.1	heme-binding protein 2-like	2036	6.55E-140
XM_020097831.1	protocadherin gamma-C5-like	1593	0
XM_020097832.1	pre-mRNA-processing factor 17	2175	0
XM_020097833.1	sentrin-specific protease 2-like	2998	0
XM_020097834.1	kelch-like ECH-associated protein 1	2977	0

XM_020097835.1	guanine nucleotide-binding protein G(I)/G(S)/G(O) subunit gamma-4	1488	5.74E-42
XM_020097836.1	diphthine--ammonia ligase isoform X1	3077	0
XM_020097837.1	diphthine--ammonia ligase isoform X2	2169	0
XM_020097838.1	kelch domain-containing protein 1	1498	0
XM_020097839.1	PREDICTED: uncharacterized protein LOC109636229	2369	7.23E-146
XM_020097840.1	sideroflexin-5 isoform X2	2204	0
XM_020097841.1	peroxisomal N(1)-acetyl-spermine/spermidine oxidase-like	2492	0
XM_020097842.1	sideroflexin-5 isoform X2	2064	0
XM_020097843.1	sideroflexin-5 isoform X2	2209	1.37E-173
XM_020097844.1	sideroflexin-5 isoform X2	2313	0
XM_020097845.1	glycoprotein endo-alpha-1,2-mannosidase-like protein	2064	0
XM_020097846.1	probable carboxypeptidase X1 isoform X1	2955	0
XM_020097847.1	probable carboxypeptidase X1 isoform X1	2424	0
XM_020097848.1	atypical kinase COQ8A, mitochondrial-like	2430	0
XM_020097849.1	lactation elevated protein 1-like isoform X1	2781	0
XM_020097850.1	lactation elevated protein 1-like isoform X1	2752	0
XM_020097851.1	transmembrane protein 229B	3408	1.58E-126
XM_020097852.1	PREDICTED: paralemmin-1-like	1331	1.74E-99
XM_020097853.1	cysteine-rich protein 1	590	6.31E-41
XM_020097854.1	peroxisomal N(1)-acetyl-spermine/spermidine oxidase-like	1987	0
XM_020097855.1	PREDICTED: uncharacterized protein C15orf41 homolog	1994	7.90E-138
XM_020097856.1	dnaJ homolog subfamily C member 5-like isoform X1	2054	2.44E-145
XM_020097857.1	dnaJ homolog subfamily C member 5-like isoform X1	2058	2.59E-145
XM_020097858.1	dnaJ homolog subfamily C member 5-like isoform X1	1955	8.80E-146
XM_020097859.1	dnaJ homolog subfamily C member 5-like isoform X1	1979	1.13E-145
XM_020097860.1	dnaJ homolog subfamily C member 5-like isoform X1	2005	4.52E-119
XM_020097861.1	peroxisomal N(1)-acetyl-spermine/spermidine oxidase-like	1825	0
XM_020097862.1	collagen alpha-1(X) chain-like	2709	1.45E-97
XM_020097863.1	probable cationic amino acid transporter	3777	0
XM_020097864.1	EGF-containing fibulin-like extracellular matrix protein 2	2336	0
XM_020097865.1	homeobox protein not2-like	1027	3.24E-160
XM_020097866.1	homeobox protein EMX1-like	1301	1.96E-171
XM_020097867.1	A disintegrin and metalloproteinase with thrombospondin motifs 10-like isoform X2	4416	0
XM_020097868.1	claudin-19 isoform X2	2007	7.31E-138
XM_020097869.1	ecto-NOX disulfide-thiol exchanger 2 isoform X1	3156	0
XM_020097870.1	paired box protein Pax-9	1931	0
XM_020097871.1	spermatogenesis-associated protein 7	2878	0
XM_020097872.1	ephrin-A2-like isoform X1	2601	5.04E-124
XM_020097873.1	homeobox protein OTX1	2597	7.60E-159
XM_020097874.1	ephrin-A2-like isoform X1	2483	1.93E-124
XM_020097875.1	ephrin-A2-like isoform X1	2598	4.40E-123
XM_020097876.1	metabotropic glutamate receptor 1-like	1668	0
XM_020097877.1	parkin coregulated gene protein-like	731	1.14E-128
XM_020097878.1	B2 bradykinin receptor-like	1029	0
XM_020097879.1	B2 bradykinin receptor-like	1171	0
XM_020097880.1	psychosine receptor-like	1043	0
XM_020097881.1	transforming growth factor-beta receptor-associated protein 1	3078	0
XM_020097882.1	adapter protein CIKS	1055	0
XM_020097883.1	PREDICTED: uncharacterized protein LOC109636263	1843	0
XM_020097884.1	protein FAM53C-like	2593	0
XM_020097885.1	---NA---	1596	
XM_020097886.1	unconventional myosin-X-like	9195	0
XM_020097887.1	PREDICTED: uncharacterized protein C21orf58 homolog	863	1.07E-118
XM_020097888.1	PREDICTED: trichohyalin-like	1982	0
XM_020097889.1	UDP-glucose:glycoprotein glucosyltransferase 1	5466	0
XM_020097890.1	ankyrin repeat domain-containing protein 34B-like	2016	0
XM_020097891.1	glycosyltransferase family 92 protein F13G3.3-like isoform X2	1266	0
XM_020097892.1	tubulin polyglutamylase complex subunit 1-like	2137	4.35E-144
XM_020097893.1	PREDICTED: uncharacterized protein LOC109636273	2640	0
XM_020097894.1	prolyl endopeptidase-like isoform X1	2644	0
XM_020097895.1	tubby-related protein 4-like	2985	0
XM_020097896.1	transmembrane protein FLJ37396	5301	0
XM_020097897.1	PREDICTED: putative uncharacterized protein C6orf183	1020	0
XM_020097898.1	forkhead box protein O3-like	3680	0
XM_020097899.1	tudor domain-containing protein 6-like	3452	0
XM_020097900.1	adhesion G protein-coupled receptor F5-like	2682	0
XM_020097901.1	microtubule-associated protein 1S-like	6753	0
XM_020097902.1	prolyl endopeptidase-like isoform X1	2566	0
XM_020097903.1	fibronectin type III domain-containing protein 3B	1842	0
XM_020097904.1	traf2 and NCK-interacting protein kinase-like	5065	0
XM_020097905.1	synaptosomal-associated protein 23-like	3007	2.88E-123
XM_020097906.1	PREDICTED: afadin-like	3624	0
XM_020097907.1	homeodomain-interacting protein kinase 1-like	2058	0
XM_020097908.1	PREDICTED: pleckstrin-2	999	0
XM_020097909.1	prolyl endopeptidase-like isoform X3	2283	0
XM_020097910.1	zinc finger FYVE domain-containing protein 26-like	1049	0
XM_020097911.1	RING finger protein 145-like	3415	0
XM_020097912.1	zinc transporter ZIP8	1496	0
XM_020097913.1	dynactin subunit 1-like	1005	2.56E-84
XM_020097914.1	rab11 family-interacting protein 1-like isoform X1	5356	0

XM_020097915.1	prostatic spermine-binding protein-like	704	5.22E-93
XM_020097916.1	AP-5 complex subunit sigma-1	1661	3.70E-114
XM_020097917.1	pecanex-like protein 1 isoform X3	8064	0
XM_020097918.1	pecanex-like protein 1 isoform X3	8049	0
XM_020097919.1	pecanex-like protein 1 isoform X3	6755	0
XM_020097920.1	pecanex-like protein 1 isoform X3	6652	0
XM_020097921.1	legumain isoform X4	1871	0
XM_020097922.1	protein phosphatase 1B-like isoform X1	2751	0
XM_020097923.1	thyroid receptor-interacting protein 11 isoform X1	7643	0
XM_020097924.1	thyroid receptor-interacting protein 11 isoform X2	7640	0
XM_020097925.1	ninein isoform X1	5625	0
XM_020097926.1	ninein isoform X2	5939	0
XM_020097927.1	son of sevenless homolog 2	7525	0
XM_020097928.1	mitogen-activated protein kinase kinase kinase 9	5647	0
XM_020097929.1	sodium/calcium exchanger 3 isoform X1	3989	0
XM_020097930.1	sodium/calcium exchanger 3 isoform X2	3971	0
XM_020097931.1	sodium/calcium exchanger 3 isoform X2	1861	0
XM_020097932.1	mitogen-activated protein kinase kinase kinase kinase 5 isoform X1	4332	0
XM_020097933.1	mitogen-activated protein kinase kinase kinase kinase 5 isoform X2	4330	0
XM_020097934.1	mitogen-activated protein kinase kinase kinase kinase 5 isoform X3	4259	0
XM_020097935.1	chemokine CXCL12b	1943	1.84E-37
XM_020097936.1	mitogen-activated protein kinase kinase kinase kinase 5 isoform X4	4257	0
XM_020097937.1	phosphofurin acidic cluster sorting protein 2 isoform X1	4811	0
XM_020097938.1	phosphofurin acidic cluster sorting protein 2 isoform X2	4808	0
XM_020097939.1	DDB1- and CUL4-associated factor 5	3933	0
XM_020097940.1	golgin subfamily A member 5	2862	0
XM_020097941.1	golgin subfamily A member 5	2754	0
XM_020097942.1	rho guanine nucleotide exchange factor 33-like	2483	0
XM_020097943.1	RNA polymerase-associated protein RTF1 homolog	2737	0
XM_020097944.1	atlastin-1 isoform X1	4031	0
XM_020097945.1	atlastin-1 isoform X2	4007	0
XM_020097946.1	nucleolar and spindle-associated protein 1	1940	0
XM_020097947.1	protocadherin gamma-C5-like	2466	0
XM_020097948.1	protein FAM13A-like	3372	0
XM_020097949.1	L-2-hydroxyglutarate dehydrogenase, mitochondrial	2540	0
XM_020097950.1	protein salvador homolog 1	2814	0
XM_020097951.1	cyclin-dependent kinase-like 1	1315	0
XM_020097952.1	cyclin-dependent kinase-like 1	1373	0
XM_020097953.1	complex I intermediate-associated protein 30, mitochondrial	1221	0
XM_020097954.1	complex I intermediate-associated protein 30, mitochondrial	1124	0
XM_020097955.1	mediator of RNA polymerase II transcription subunit 6	1715	2.45E-129
XM_020097956.1	ATP synthase subunit s, mitochondrial isoform X1	862	3.43E-150
XM_020097957.1	ATP synthase subunit s, mitochondrial isoform X1	880	3.57E-143
XM_020097958.1	calcineurin B homologous protein 1	1434	2.18E-128
XM_020097959.1	protein Mis18-beta	975	4.55E-129
XM_020097960.1	phytanoyl-CoA hydroxylase-interacting protein-like	1950	0
XM_020097961.1	tetratricopeptide repeat protein 9A-like	1214	4.07E-52
XM_020097962.1	coiled-coil domain-containing protein 130	1433	5.27E-171
XM_020097963.1	pituitary tumor-transforming gene 1 protein-interacting protein-like	1396	2.32E-91
XM_020097964.1	DNA polymerase epsilon subunit 2	1815	0
XM_020097965.1	fatty acid-binding protein, brain-like	711	2.99E-94
XM_020097966.1	otolith matrix protein 1-like	2899	0
XM_020097967.1	homeobox protein Nkx-2.1	1953	0
XM_020097968.1	cytokine receptor-like factor 1 isoform X1	2334	0
XM_020097969.1	cytokine receptor-like factor 1 isoform X2	2338	0
XM_020097970.1	myopalladin isoform X1	8352	0
XM_020097971.1	four and a half LIM domains protein 2-like	1090	0
XM_020097972.1	phospholipase B1, membrane-associated	4637	0
XM_020097973.1	rab GTPase-activating protein 1-like isoform X1	1558	0
XM_020097974.1	rab GTPase-activating protein 1-like isoform X1	1373	0
XM_020097975.1	rab GTPase-activating protein 1-like isoform X1	1677	0
XM_020097976.1	homeobox protein Nkx-2.2 isoform X1	1540	3.30E-123
XM_020097977.1	tandem C2 domains nuclear protein	2421	0
XM_020097978.1	myopalladin isoform X2	8274	0
XM_020097979.1	pancreatic secretory granule membrane major glycoprotein GP2-like	1498	0
XM_020097980.1	ankyrin repeat domain-containing protein 66-like	767	8.14E-114
XM_020097981.1	lysophosphatidic acid receptor 4	1245	0
XM_020097982.1	PREDICTED: uncharacterized protein LOC109636340	745	1.46E-157
XM_020097983.1	interleukin-6 receptor subunit beta-like	3125	0
XM_020097984.1	B2 bradykinin receptor-like	1590	0
XM_020097985.1	glycosyltransferase family 92 protein F13G3.3-like isoform X2	2644	0
XM_020097986.1	glycosyltransferase family 92 protein F13G3.3-like isoform X2	2393	0
XM_020097987.1	potassium channel subfamily K member 10-like	2401	0
XM_020097988.1	galectin-related protein B-like	1050	3.92E-54
XM_020097989.1	PREDICTED: uncharacterized protein C14orf132 homolog	2085	2.06E-36
XM_020097990.1	protein FAM45A	2370	0
XM_020097991.1	zinc transporter 2-like	1316	0
XM_020097992.1	PREDICTED: syntaxin-11-like	1460	1.76E-173
XM_020097993.1	probable G-protein coupled receptor 151	1584	0

XM_020097994.1	myc target protein 1	1184	4.70E-121
XM_020097995.1	transcription factor Sp3-like	4477	0
XM_020097996.1	heparan-sulfate 6-O-sulfotransferase 1-A-like	5125	0
XM_020097997.1	ciliary neurotrophic factor receptor subunit alpha-like	821	3.44E-155
XM_020097998.1	sideroflexin-4 isoform X1	2108	0
XM_020097999.1	outer dense fiber protein 3-like protein 2	784	6.47E-177
XM_020098000.1	TRAF2 and NCK-interacting protein kinase-like	3299	0
XM_020098001.1	solute carrier family 2, facilitated glucose transporter member 2	2160	0
XM_020098002.1	eukaryotic translation initiation factor 5A-1-like	3832	5.45E-91
XM_020098003.1	eukaryotic translation initiation factor 5A-1-like	3730	2.19E-91
XM_020098004.1	PREDICTED: syntaxin-11-like	899	0
XM_020098005.1	sideroflexin-4 isoform X2	2105	0
XM_020098006.1	cytoplasmic dynein 1 heavy chain 1	14241	0
XM_020098007.1	PREDICTED: uncharacterized protein LOC109636367 isoform X1	1340	0
XM_020098008.1	PREDICTED: uncharacterized protein LOC109636367 isoform X2	1265	5.09E-162
XM_020098009.1	GTP-binding protein Di-Ras1	6093	4.71E-133
XM_020098010.1	receptor expression-enhancing protein 5-like	1245	1.15E-115
XM_020098011.1	growth arrest and DNA damage-inducible protein GADD45 beta-like isoform X1	1408	4.80E-104
XM_020098012.1	growth arrest and DNA damage-inducible protein GADD45 beta-like isoform X1	945	1.86E-96
XM_020098013.1	cocaine- and amphetamine-regulated transcript protein-like	758	1.31E-72
XM_020098014.1	guanine nucleotide-binding protein G(I)/G(S)/G(O) subunit gamma-12-like	740	2.13E-43
XM_020098015.1	guanine nucleotide-binding protein G(I)/G(S)/G(O) subunit gamma-12-like	777	3.05E-43
XM_020098016.1	protein bicaudal C homolog 1 isoform X1	6394	0
XM_020098017.1	PREDICTED: nesprin-1-like	27556	0
XM_020098018.1	coiled-coil domain-containing protein 170	1893	0
XM_020098019.1	required for meiotic nuclear division protein 1 homolog isoform X1	1975	0
XM_020098020.1	required for meiotic nuclear division protein 1 homolog isoform X1	1907	0
XM_020098021.1	required for meiotic nuclear division protein 1 homolog isoform X1	1888	0
XM_020098022.1	protein quaking-A isoform X1	2290	0
XM_020098023.1	protein quaking-A isoform X2	7925	0
XM_020098024.1	protein quaking-A isoform X3	2268	0
XM_020098025.1	protein quaking-A isoform X4	2149	0
XM_020098026.1	protein bicaudal C homolog 1 isoform X2	6372	0
XM_020098027.1	protein quaking-A isoform X5	7784	0
XM_020098028.1	sodium-dependent multivitamin transporter-like	2911	0
XM_020098029.1	sodium-dependent multivitamin transporter-like	2843	0
XM_020098030.1	sodium-dependent multivitamin transporter-like	2571	0
XM_020098031.1	sodium-dependent multivitamin transporter-like	2570	0
XM_020098032.1	SH2 domain-containing adapter protein F-like	2387	0
XM_020098033.1	quinone oxidoreductase PIG3	1331	0
XM_020098034.1	quinone oxidoreductase PIG3	1486	0
XM_020098035.1	quinone oxidoreductase PIG3	1221	0
XM_020098036.1	centromere protein O	1783	0
XM_020098037.1	guanine nucleotide-binding protein subunit alpha-11-like isoform X1	7536	0
XM_020098038.1	guanine nucleotide-binding protein G(q) subunit alpha-like isoform X2	7398	0
XM_020098039.1	potassium voltage-gated channel subfamily H member 1 isoform X1	4459	0
XM_020098040.1	guanine nucleotide-binding protein subunit alpha-11-like isoform X3	7403	0
XM_020098041.1	putative Polycomb group protein ASXL2 isoform X1	6329	0
XM_020098042.1	putative Polycomb group protein ASXL2 isoform X2	6326	0
XM_020098043.1	putative Polycomb group protein ASXL2 isoform X3	5923	0
XM_020098044.1	kinesin-like protein KIF3B	3039	0
XM_020098045.1	trifunctional enzyme subunit alpha, mitochondrial	3029	0
XM_020098046.1	ubiquitin-conjugating enzyme E2 D2-like isoform X1	2103	1.50E-102
XM_020098047.1	ubiquitin-conjugating enzyme E2 D2-like isoform X2	2044	7.28E-102
XM_020098048.1	potassium voltage-gated channel subfamily H member 1 isoform X1	4456	0
XM_020098049.1	intron-binding protein aquarius isoform X1	4787	0
XM_020098050.1	intron-binding protein aquarius isoform X2	4784	0
XM_020098051.1	zinc finger protein 770-like	3479	0
XM_020098052.1	pericentrin isoform X1	9916	0
XM_020098053.1	pericentrin isoform X2	9913	0
XM_020098054.1	pericentrin isoform X3	9907	0
XM_020098055.1	pericentrin isoform X4	9901	0
XM_020098056.1	pericentrin isoform X5	9772	0
XM_020098057.1	potassium voltage-gated channel subfamily H member 1 isoform X1	4631	0
XM_020098058.1	spectrin beta chain, non-erythrocytic 1-like	3343	0
XM_020098059.1	lysine-specific histone demethylase 1A isoform X1	3483	0
XM_020098060.1	lysine-specific histone demethylase 1A isoform X2	3480	0
XM_020098061.1	lysine-specific histone demethylase 1A isoform X3	3478	0
XM_020098062.1	lysine-specific histone demethylase 1A isoform X4	3471	0
XM_020098063.1	lysine-specific histone demethylase 1A isoform X1	3462	0
XM_020098064.1	cell cycle control protein 50A-like	1712	0
XM_020098065.1	nuclear export mediator factor NEMF isoform X1	4759	0
XM_020098066.1	protocadherin gamma-C5-like	2139	0
XM_020098067.1	potassium voltage-gated channel subfamily H member 1 isoform X1	4334	0
XM_020098068.1	nuclear export mediator factor NEMF isoform X2	4757	0
XM_020098069.1	serine/threonine-protein kinase ICK isoform X1	3104	0
XM_020098070.1	MAPK/MAK/MRK overlapping kinase isoform X2	3193	0
XM_020098071.1	kelch domain-containing protein 2	1661	0
XM_020098072.1	PREDICTED: beta-mannosidase	3456	0

XM_020098073.1	putative N-acetyltransferase 8B	1476	7.21E-157
XM_020098074.1	excitatory amino acid transporter 5-like	1680	0
XM_020098075.1	potassium voltage-gated channel subfamily H member 1 isoform X1	4432	0
XM_020098076.1	ELAV-like protein 1 isoform X1	2106	0
XM_020098077.1	ELAV-like protein 1 isoform X2	2100	0
XM_020098078.1	ELAV-like protein 1 isoform X3	2096	0
XM_020098079.1	ELAV-like protein 1 isoform X4	2090	0
XM_020098080.1	PREDICTED: tetraspanin-3-like	1248	1.96E-143
XM_020098081.1	lysyl oxidase homolog 3 isoform X1	2781	0
XM_020098082.1	lysyl oxidase homolog 3 isoform X2	2766	0
XM_020098083.1	lysyl oxidase homolog 3 isoform X3	2118	0
XM_020098084.1	lysyl oxidase homolog 3 isoform X3	1000	2.00E-170
XM_020098085.1	lysyl oxidase homolog 3 isoform X5	2937	0
XM_020098086.1	28S ribosomal protein S26, mitochondrial	1687	5.09E-102
XM_020098087.1	calmodulin-lysine N-methyltransferase-like isoform X1	822	5.48E-113
XM_020098088.1	amino-terminal enhancer of split-like	4516	4.02E-83
XM_020098089.1	tyrosine-protein phosphatase non-receptor type 21 isoform X1	6426	0
XM_020098090.1	tyrosine-protein phosphatase non-receptor type 21 isoform X1	6251	0
XM_020098091.1	tyrosine-protein phosphatase non-receptor type 21 isoform X1	6318	0
XM_020098092.1	protein FAM98B	1808	0
XM_020098093.1	RNA polymerase II elongation factor ELL-like isoform X1	2967	0
XM_020098094.1	RNA polymerase II elongation factor ELL-like isoform X2	2959	0
XM_020098095.1	calmodulin-lysine N-methyltransferase-like isoform X1	733	1.13E-113
XM_020098096.1	single-stranded DNA-binding protein 3-like isoform X1	4468	2.89E-132
XM_020098097.1	single-stranded DNA-binding protein 3-like isoform X2	4465	9.30E-131
XM_020098098.1	D-2-hydroxyglutarate dehydrogenase, mitochondrial	3447	0
XM_020098099.1	activin receptor type-1-like isoform X1	3171	0
XM_020098100.1	activin receptor type-1-like isoform X1	2928	0
XM_020098101.1	activin receptor type-1-like isoform X1	2843	0
XM_020098102.1	filamin-A-interacting protein 1-like	9928	0
XM_020098103.1	nardilysin isoform X1	4834	0
XM_020098104.1	calmodulin-lysine N-methyltransferase-like isoform X1	622	4.72E-96
XM_020098105.1	nardilysin isoform X1	4711	0
XM_020098106.1	nardilysin isoform X1	4743	0
XM_020098107.1	SLIT and NTRK-like protein 3	4477	0
XM_020098108.1	SLIT and NTRK-like protein 3	4273	0
XM_020098109.1	small ubiquitin-related modifier 3-like isoform X1	2941	3.64E-68
XM_020098110.1	small ubiquitin-related modifier 3 isoform X2	2998	1.19E-57
XM_020098111.1	dihydropyrimidinase-related protein 5 isoform X2	3396	0
XM_020098112.1	dihydropyrimidinase-related protein 5 isoform X2	3441	0
XM_020098113.1	dihydropyrimidinase-related protein 5 isoform X2	3674	0
XM_020098114.1	microtubule-associated protein RP/EB family member 3-like	2218	0
XM_020098115.1	microtubule-associated protein RP/EB family member 3-like	2207	0
XM_020098116.1	microtubule-associated protein RP/EB family member 3-like	2182	0
XM_020098117.1	transcription factor HIVEP3	5745	0
XM_020098118.1	calmodulin-lysine N-methyltransferase-like	3737	2.06E-95
XM_020098119.1	transcription factor HIVEP3	7678	0
XM_020098120.1	gamma-taxilin-like	1915	0
XM_020098121.1	dual specificity protein phosphatase 23	1328	1.91E-109
XM_020098122.1	dual specificity protein phosphatase 23	1290	3.82E-110
XM_020098123.1	dual specificity protein phosphatase 23	1938	8.38E-107
XM_020098124.1	stAR-related lipid transfer protein 9 isoform X1	12804	0
XM_020098125.1	stAR-related lipid transfer protein 9 isoform X2	12801	0
XM_020098126.1	transformer-2 protein homolog alpha-like isoform X2	1839	1.64E-77
XM_020098127.1	transformer-2 protein homolog alpha-like isoform X2	2119	5.99E-86
XM_020098128.1	transformer-2 protein homolog alpha-like isoform X2	1831	6.05E-87
XM_020098129.1	transformer-2 protein homolog alpha-like isoform X2	1998	3.63E-86
XM_020098130.1	obg-like ATPase 1	2496	0
XM_020098131.1	protein ecdysoneless homolog	2750	0
XM_020098132.1	fermitin family homolog 2 isoform X1	5269	0
XM_020098133.1	fermitin family homolog 2 isoform X2	5248	0
XM_020098134.1	fermitin family homolog 2 isoform X3	5235	0
XM_020098135.1	fermitin family homolog 2 isoform X4	5214	0
XM_020098136.1	polypyrimidine tract-binding protein 1-like	1317	0
XM_020098137.1	rab11 family-interacting protein 1-like isoform X1	4267	0
XM_020098138.1	rab11 family-interacting protein 1-like isoform X1	4364	0
XM_020098139.1	cohesin subunit SA-2-like	5852	0
XM_020098140.1	mRNA decay activator protein ZFP36L1	3483	0
XM_020098141.1	syntaxin-binding protein 5-like isoform X1	5334	0
XM_020098142.1	syntaxin-binding protein 5-like isoform X1	5229	0
XM_020098143.1	syntaxin-binding protein 5-like isoform X1	5205	0
XM_020098144.1	PREDICTED: uncharacterized protein LOC109636434	1149	4.39E-170
XM_020098145.1	syntaxin-binding protein 5-like isoform X3	5100	0
XM_020098146.1	syntaxin-binding protein 5-like isoform X3	5049	0
XM_020098147.1	SAM and SH3 domain-containing protein 1-like isoform X3	5521	0
XM_020098148.1	SAM and SH3 domain-containing protein 1-like isoform X3	5512	0
XM_020098149.1	SAM and SH3 domain-containing protein 1-like isoform X3	4845	0
XM_020098150.1	SAM and SH3 domain-containing protein 1-like isoform X3	4877	0
XM_020098151.1	SAM and SH3 domain-containing protein 1-like isoform X5	4011	0

XM_020098152.1	SAM and SH3 domain-containing protein 1-like isoform X6	4002	0
XM_020098153.1	SAM and SH3 domain-containing protein 1-like isoform X4	2698	0
XM_020098154.1	SAM and SH3 domain-containing protein 1-like isoform X3	2854	0
XM_020098155.1	PREDICTED: uncharacterized protein LOC109636434	1111	2.68E-170
XM_020098156.1	SAM and SH3 domain-containing protein 1-like isoform X3	2674	0
XM_020098157.1	D-aspartate oxidase	1702	0
XM_020098158.1	sex comb on midleg-like protein 4 isoform X1	3418	0
XM_020098159.1	sex comb on midleg-like protein 4 isoform X1	3414	0
XM_020098160.1	visinin-like protein 1	2700	1.88E-133
XM_020098161.1	visinin-like protein 1	2701	1.88E-133
XM_020098162.1	ribosomal protein S6 kinase alpha-5-like isoform X1	7329	0
XM_020098163.1	ribosomal protein S6 kinase alpha-5-like isoform X2	7306	0
XM_020098164.1	ubiquitin carboxyl-terminal hydrolase 40 isoform X1	5675	0
XM_020098165.1	ubiquitin carboxyl-terminal hydrolase 40 isoform X2	5672	0
XM_020098166.1	thrombospondin-type laminin G domain and EAR repeat-containing protein-like	3363	0
XM_020098167.1	graves disease carrier protein	3944	0
XM_020098168.1	transforming growth factor-beta receptor-associated protein 1	4588	0
XM_020098169.1	transforming growth factor-beta receptor-associated protein 1	4585	0
XM_020098170.1	serine/threonine-protein phosphatase 2A regulatory subunit B'' subunit alpha isoform X1	7437	0
XM_020098171.1	serine/threonine-protein phosphatase 2A regulatory subunit B'' subunit alpha isoform X2	4519	0
XM_020098172.1	F-box only protein 30-like isoform X1	4637	0
XM_020098173.1	glycogen phosphorylase, liver form	3186	0
XM_020098174.1	protein ABHD12B	1327	0
XM_020098175.1	protein ABHD12B	1281	0
XM_020098176.1	homeobox protein Meis2 isoform X1	4072	0
XM_020098177.1	homeobox protein Meis2 isoform X1	2479	0
XM_020098178.1	homeobox protein Meis2 isoform X1	3143	0
XM_020098179.1	homeobox protein Meis2 isoform X1	2536	0
XM_020098180.1	serine/threonine-protein kinase ICK isoform X1	3019	0
XM_020098181.1	homeobox protein Meis2 isoform X1	1611	0
XM_020098182.1	homeobox protein Meis2 isoform X1	2514	0
XM_020098183.1	homeobox protein Meis2 isoform X1	1584	0
XM_020098184.1	thioredoxin-related transmembrane protein 1-like	2116	3.42E-165
XM_020098185.1	E3 ubiquitin-protein ligase SHPRH isoform X1	6070	0
XM_020098186.1	E3 ubiquitin-protein ligase SHPRH isoform X2	6067	0
XM_020098187.1	E3 ubiquitin-protein ligase SHPRH isoform X1	5588	0
XM_020098188.1	serine/threonine-protein kinase ICK isoform X1	3015	0
XM_020098189.1	neutral alpha-glucosidase C-like	3098	0
XM_020098190.1	transmembrane protein 87A	2929	0
XM_020098191.1	SPARC-related modular calcium-binding protein 1 isoform X1	5382	0
XM_020098192.1	SPARC-related modular calcium-binding protein 1 isoform X2	5380	0
XM_020098193.1	SPARC-related modular calcium-binding protein 1 isoform X3	5333	0
XM_020098194.1	SPARC-related modular calcium-binding protein 1 isoform X4	5299	0
XM_020098195.1	solute carrier family 2, facilitated glucose transporter member 1-like	2099	0
XM_020098196.1	short transient receptor potential channel 7	3049	0
XM_020098197.1	serine/threonine-protein kinase ICK isoform X1	2550	0
XM_020098198.1	tyrosine-protein kinase Fyn isoform X1	3263	0
XM_020098199.1	tissue alpha-L-fucosidase-like	1754	0
XM_020098200.1	putative deoxyribonuclease TATDN3 isoform X1	1108	0
XM_020098201.1	putative deoxyribonuclease TATDN3 isoform X1	1103	0
XM_020098202.1	DNA (cytosine-5)-methyltransferase 3A-like isoform X1	5635	0
XM_020098203.1	DNA (cytosine-5)-methyltransferase 3A-like isoform X2	5620	0
XM_020098204.1	DNA (cytosine-5)-methyltransferase 3A-like isoform X3	5488	0
XM_020098205.1	PREDICTED: presenilin-1-like	3211	0
XM_020098206.1	myristoylated alanine-rich C-kinase substrate-like	1880	1.70E-47
XM_020098207.1	lysocardiolipin acyltransferase 1	3335	0
XM_020098208.1	hypoxia-inducible factor 1-alpha inhibitor	3369	0
XM_020098209.1	FERM domain-containing protein 6-like	5033	0
XM_020098210.1	FERM domain-containing protein 6-like	4732	0
XM_020098211.1	cysteine-rich protein 2-like isoform X1	2118	1.47E-173
XM_020098212.1	cysteine-rich protein 2-like isoform X2	2115	9.41E-173
XM_020098213.1	protein yippee-like 2	2480	7.69E-80
XM_020098214.1	E3 ubiquitin-protein ligase TRIM9 isoform X1	6943	0
XM_020098215.1	E3 ubiquitin-protein ligase TRIM9 isoform X2	6903	0
XM_020098216.1	WD repeat-containing protein 20-like	3009	0
XM_020098217.1	serine/threonine-protein phosphatase PP1-beta catalytic subunit	2448	0
XM_020098218.1	protein ANTAGONIST OF LIKE HETEROCHROMATIN PROTEIN 1-like	1881	0
XM_020098219.1	dystrobrevin beta isoform X1	5979	0
XM_020098220.1	dystrobrevin beta isoform X2	5958	0
XM_020098221.1	dystrobrevin beta isoform X3	5883	0
XM_020098222.1	propionyl-CoA carboxylase beta chain, mitochondrial	2520	0
XM_020098223.1	CUE domain-containing protein 2 isoform X2	1732	0
XM_020098224.1	sprouty-related, EVH1 domain-containing protein 1-like	5100	1.51E-161
XM_020098225.1	metastasis-associated protein MTA1	2465	0
XM_020098226.1	syntaxin-binding protein 6	2442	1.46E-138
XM_020098227.1	M-phase inducer phosphatase 1-like	2707	0
XM_020098228.1	programmed cell death protein 10	1708	8.06E-149
XM_020098229.1	pleckstrin homology domain-containing family H member 1	5237	0
XM_020098230.1	pantothenate kinase 3-like isoform X1	2873	0

XM_020098231.1	CUE domain-containing protein 2 isoform X2	1638	0
XM_020098232.1	pantothenate kinase 3-like isoform X2	1655	0
XM_020098233.1	alpha-(1,3)-fucosyltransferase 9	4611	0
XM_020098234.1	phospholipase DDHD1-like isoform X1	6415	0
XM_020098235.1	phospholipase DDHD1-like isoform X2	6313	0
XM_020098236.1	mitochondrial 2-oxodicarboxylate carrier isoform X1	2670	0
XM_020098237.1	mitochondrial 2-oxodicarboxylate carrier isoform X1	2329	0
XM_020098238.1	inositol-3-phosphate synthase 1	2507	0
XM_020098239.1	phospholipid phosphatase 2-like	1988	0
XM_020098240.1	CUE domain-containing protein 2 isoform X2	1593	0
XM_020098241.1	sprouty-related, EVH1 domain-containing protein 1-like	3943	0
XM_020098242.1	autophagy-related protein 9A isoform X1	4036	0
XM_020098243.1	autophagy-related protein 9A isoform X2	4021	0
XM_020098244.1	autophagy-related protein 9A isoform X3	4086	0
XM_020098245.1	protein LBH-like	2314	3.90E-67
XM_020098246.1	mitochondrial antiviral signaling protein IPS-1	3569	0
XM_020098247.1	PREDICTED: uncharacterized protein C1orf53 homolog	2350	3.89E-125
XM_020098248.1	protein YIPF4	1601	4.55E-124
XM_020098249.1	histone-binding protein N1/N2-like isoform X1	1936	0
XM_020098250.1	histone-binding protein N1/N2-like isoform X2	1787	0
XM_020098251.1	nuclear autoantigenic sperm protein-like isoform X3	1582	0
XM_020098252.1	probable rRNA-processing protein EBP2	1462	7.65E-150
XM_020098253.1	protein FAM183B-like	827	1.88E-51
XM_020098254.1	salivary glue protein Sgs-3-like	1643	9.59E-100
XM_020098255.1	protein CYR61-like	2071	0
XM_020098256.1	WD repeat-containing protein 47	4704	0
XM_020098257.1	pyroglutamyl-peptidase 1-like	3375	1.74E-135
XM_020098258.1	trimeric intracellular cation channel type A	1549	4.38E-168
XM_020098259.1	nuclear receptor subfamily 5 group A member 2	2404	0
XM_020098260.1	collagen alpha-1(XXIV) chain	6169	0
XM_020098261.1	unconventional myosin-If-like	4504	0
XM_020098262.1	ubiquitin-associated domain-containing protein 2	2918	0
XM_020098263.1	unconventional myosin-If-like	4386	0
XM_020098264.1	KN motif and ankyrin repeat domain-containing protein 4	5217	0
XM_020098265.1	low-density lipoprotein receptor-like	3001	0
XM_020098266.1	tyrosine-protein kinase receptor Tie-1 isoform X1	5027	0
XM_020098267.1	tyrosine-protein kinase receptor Tie-1 isoform X2	4994	0
XM_020098268.1	tyrosine-protein kinase receptor Tie-1 isoform X3	4691	0
XM_020098269.1	peptidyl-prolyl cis-trans isomerase FKBP2	1063	2.10E-96
XM_020098270.1	angiopoietin-related protein 4-like	1760	0
XM_020098271.1	tRNA modification GTPase GTPBP3, mitochondrial isoform X1	2490	0
XM_020098272.1	tRNA modification GTPase GTPBP3, mitochondrial isoform X2	2487	0
XM_020098273.1	cysteine protease ATG4D	2466	0
XM_020098274.1	thrombospondin-2 isoform X1	5343	0
XM_020098275.1	guanidinoacetate N-methyltransferase	1288	2.10E-176
XM_020098276.1	LIM/homeobox protein Lhx9	2915	0
XM_020098277.1	protein SREK1IP1	1464	1.75E-40
XM_020098278.1	adenylate kinase 4, mitochondrial	2682	4.04E-150
XM_020098279.1	receptor-type tyrosine-protein phosphatase C	3515	0
XM_020098280.1	receptor-type tyrosine-protein phosphatase C	3503	0
XM_020098281.1	probable proline--tRNA ligase, mitochondrial	1849	0
XM_020098282.1	thrombospondin-2 isoform X2	5312	0
XM_020098283.1	probable proline--tRNA ligase, mitochondrial	1846	0
XM_020098284.1	coiled-coil domain-containing protein 124	1219	1.89E-102
XM_020098285.1	coiled-coil domain-containing protein 124	1129	5.09E-102
XM_020098286.1	leptin receptor	5222	0
XM_020098287.1	thromboxane A2 receptor isoform X2	2504	0
XM_020098288.1	thromboxane A2 receptor isoform X2	2462	0
XM_020098289.1	thromboxane A2 receptor isoform X2	2441	0
XM_020098290.1	thromboxane A2 receptor isoform X2	2872	0
XM_020098291.1	thromboxane A2 receptor isoform X2	2762	0
XM_020098292.1	thromboxane A2 receptor isoform X2	2618	0
XM_020098293.1	thromboxane A2 receptor isoform X2	2589	0
XM_020098294.1	thromboxane A2 receptor isoform X2	2354	0
XM_020098295.1	thromboxane A2 receptor isoform X2	2402	0
XM_020098296.1	thromboxane A2 receptor isoform X2	2340	0
XM_020098297.1	excitatory amino acid transporter 1-like	5291	0
XM_020098298.1	thromboxane A2 receptor isoform X2	2656	0
XM_020098299.1	thromboxane A2 receptor isoform X2	2248	0
XM_020098300.1	tyrosine-protein kinase JAK2-like isoform X2	4083	0
XM_020098301.1	tyrosine-protein kinase JAK2-like isoform X2	4055	0
XM_020098302.1	tyrosine-protein kinase JAK2-like isoform X2	4049	0
XM_020098303.1	tyrosine-protein kinase JAK2-like isoform X2	3500	0
XM_020098304.1	tetratricopeptide repeat protein 22 isoform X1	1836	0
XM_020098305.1	tetratricopeptide repeat protein 22 isoform X2	1833	0
XM_020098306.1	cornifelin homolog B-like	901	1.20E-120
XM_020098307.1	box C/D snoRNA protein 1	1679	0
XM_020098308.1	NADH dehydrogenase [ubiquinone] iron-sulfur protein 7, mitochondrial	901	1.75E-150
XM_020098309.1	EF-hand calcium-binding domain-containing protein 7 isoform X1	2670	0

XM_020098310.1	core histone macro-H2A.2	1921	0
XM_020098311.1	EF-hand calcium-binding domain-containing protein 7 isoform X2	2661	0
XM_020098312.1	PREDICTED: anosmin-1-like	3382	0
XM_020098313.1	alkaline phosphatase-like isoform X1	2732	0
XM_020098314.1	alkaline phosphatase-like isoform X2	2531	0
XM_020098315.1	zinc-binding protein A33-like	2049	6.97E-170
XM_020098316.1	glypican-5-like isoform X1	3838	0
XM_020098317.1	transcription factor jun-D	2017	7.43E-180
XM_020098318.1	core histone macro-H2A.2	1693	0
XM_020098319.1	UDP-GlcNAc:betaGal beta-1,3-N-acetylglucosaminyltransferase 7	2202	0
XM_020098320.1	histidine-rich glycoprotein-like	1787	0
XM_020098321.1	transmembrane protein 125-like	2873	3.78E-100
XM_020098322.1	transmembrane protein 125-like	2669	1.05E-100
XM_020098323.1	adhesion G-protein coupled receptor D2-like	3477	0
XM_020098324.1	kinesin-like protein KIF14	5749	0
XM_020098325.1	PDZ domain-containing protein GIPC3-like	966	0
XM_020098326.1	low-density lipoprotein receptor-related protein 8-like	838	0

XM_020098327.1	potassium voltage-gated channel subfamily V member 2-like	1530	0
XM_020098328.1	carbohydrate sulfotransferase 3-like	4044	0
XM_020098329.1	sodium-coupled monocarboxylate transporter 1-like	1893	0
XM_020098330.1	R3H domain-containing protein 4	1104	0
XM_020098331.1	PREDICTED: uncharacterized protein LOC109636561	968	0
XM_020098332.1	protein crumbs homolog 1	2382	0
XM_020098333.1	DENN domain-containing protein 1B	549	1.61E-125
XM_020098334.1	proteoglycan 4	2559	0
XM_020098335.1	transcriptional protein SWT1	3633	0
XM_020098336.1	ribonucleoprotein PTB-binding 2	2443	0
XM_020098337.1	VWFA and cache domain-containing protein 1	2155	0
XM_020098338.1	PREDICTED: glypican-1-like	1085	0
XM_020098339.1	McKusick-Kaufman/Bardet-Biedl syndromes putative chaperonin	2481	0
XM_020098340.1	coiled-coil domain-containing protein 17	1923	0
XM_020098341.1	leucine-rich repeat-containing protein 7-like	2852	0
XM_020098342.1	U6 snRNA-associated Sm-like protein LSm4	1306	7.88E-57
XM_020098343.1	serine/arginine-rich splicing factor 2-like isoform X1	1108	1.73E-119
XM_020098344.1	contactin-associated protein-like 4	4514	0
XM_020098345.1	small glutamine-rich tetratricopeptide repeat-containing protein beta	1569	0
XM_020098346.1	NADH dehydrogenase [ubiquinone] 1 alpha subcomplex subunit 7	518	6.04E-71
XM_020098347.1	small integral membrane protein 7	973	1.14E-45
XM_020098348.1	alkaline phosphatase-like	1874	0
XM_020098349.1	plasmalemma vesicle-associated protein-like	1748	0
XM_020098350.1	protein SLX4IP	1816	0
XM_020098351.1	prostaglandin E2 receptor EP4 subtype-like	1881	0
XM_020098352.1	tyrosine-protein kinase SYK isoform X1	3364	0
XM_020098353.1	type III endosome membrane protein TEMP	1676	4.43E-68
XM_020098354.1	small conductance calcium-activated potassium channel protein 2-like isoform X2	3206	0
XM_020098355.1	small conductance calcium-activated potassium channel protein 2-like isoform X2	3191	0
XM_020098356.1	probable ATP-dependent RNA helicase DDX59	969	0
XM_020098357.1	doublesex- and mab-3-related transcription factor 2	1740	0
XM_020098358.1	doublesex- and mab-3-related transcription factor 2	1874	0
XM_020098359.1	protein SLX4IP	1719	0
XM_020098360.1	doublesex- and mab-3-related transcription factor 2	1637	0
XM_020098361.1	doublesex- and mab-3-related transcription factor 2	1719	0
XM_020098362.1	leucine rich adaptor protein 1-like	3286	0
XM_020098363.1	hydroxylysine kinase-like	1822	0
XM_020098364.1	alkaline ceramidase 1	2238	0
XM_020098365.1	cornifelin homolog B-like	593	1.11E-95
XM_020098366.1	FYN-binding protein-like	2235	0
XM_020098367.1	protein ABHD8	1751	0
XM_020098368.1	insulin-like peptide INSL5	973	1.33E-56
XM_020098369.1	lens fiber membrane intrinsic protein-like	525	1.28E-114
XM_020098370.1	leucine-rich PPR motif-containing protein, mitochondrial-like	572	4.90E-78
XM_020098371.1	disabled homolog 2-like	1367	0
XM_020098372.1	PREDICTED: uncharacterized protein LOC109636599	5156	0
XM_020098373.1	PREDICTED: occludin-like	3468	0
XM_020098374.1	nuclear receptor subfamily 2 group F member 6-like isoform X1	5013	0
XM_020098375.1	BRISC and BRCA1-A complex member 1	2142	0
XM_020098376.1	BRISC and BRCA1-A complex member 1	2287	0
XM_020098377.1	actin-related protein 2/3 complex subunit 5-like	2192	1.09E-96
XM_020098378.1	PREDICTED: hemicentin-1	18558	0
XM_020098379.1	RNA exonuclease 1 homolog isoform X1	6937	0
XM_020098380.1	potassium channel subfamily K member 1	3683	0
XM_020098381.1	MAP kinase-interacting serine/threonine-protein kinase 2	4545	0
XM_020098382.1	dedicator of cytokinesis protein 7 isoform X4	7186	0
XM_020098383.1	dedicator of cytokinesis protein 7 isoform X2	7180	0
XM_020098384.1	dedicator of cytokinesis protein 7 isoform X3	7171	0
XM_020098385.1	dedicator of cytokinesis protein 7 isoform X4	7067	0
XM_020098386.1	dedicator of cytokinesis protein 7 isoform X5	7162	0
XM_020098387.1	dedicator of cytokinesis protein 7 isoform X6	7206	0
XM_020098388.1	dedicator of cytokinesis protein 7 isoform X7	7156	0

XM_020098389.1	dedicator of cytokinesis protein 7 isoform X8	2895	0
XM_020098390.1	dedicator of cytokinesis protein 7 isoform X9	2874	0
XM_020098391.1	dedicator of cytokinesis protein 7 isoform X10	7141	0
XM_020098392.1	dedicator of cytokinesis protein 7 isoform X11	7135	0
XM_020098393.1	angiopoietin-related protein 3 isoform X1	2443	0
XM_020098394.1	angiopoietin-related protein 3 isoform X2	2329	0
XM_020098395.1	rho GTPase-activating protein 19-like	2845	0
XM_020098396.1	dynein heavy chain 12, axonemal-like	11679	0
XM_020098397.1	regulator of nonsense transcripts 1-like	6409	0
XM_020098398.1	regulator of nonsense transcripts 1-like	6406	0
XM_020098399.1	probable ATP-dependent RNA helicase DDX49	1721	0
XM_020098400.1	KN motif and ankyrin repeat domain-containing protein 3	3624	0
XM_020098401.1	KN motif and ankyrin repeat domain-containing protein 3	3217	0
XM_020098402.1	KN motif and ankyrin repeat domain-containing protein 3	3202	0
XM_020098403.1	KN motif and ankyrin repeat domain-containing protein 3	3213	0
XM_020098404.1	phosphatidylinositol 3-kinase regulatory subunit gamma-like	2602	0
XM_020098405.1	gamma-interferon-inducible lysosomal thiol reductase-like	1173	0
XM_020098406.1	GTP cyclohydrolase 1-like	1063	8.64E-168
XM_020098407.1	cytoplasmic polyadenylation element-binding protein 3-like isoform X1	3818	0
XM_020098408.1	GTP cyclohydrolase 1-like	1104	1.58E-167
XM_020098409.1	nucleoprotein TPR isoform X1	7438	0
XM_020098410.1	nucleoprotein TPR isoform X2	7396	0
XM_020098411.1	nucleoprotein TPR isoform X3	7393	0
XM_020098412.1	nucleoprotein TPR isoform X4	7390	0
XM_020098413.1	nucleoprotein TPR isoform X5	7390	0
XM_020098414.1	nucleoprotein TPR isoform X6	7387	0
XM_020098415.1	cytoplasmic polyadenylation element-binding protein 3-like isoform X2	4207	0
XM_020098416.1	nucleoprotein TPR isoform X7	7384	0
XM_020098417.1	nucleoprotein TPR isoform X8	7378	0
XM_020098418.1	microtubule-associated serine/threonine-protein kinase 3 isoform X1	7785	0
XM_020098419.1	microtubule-associated serine/threonine-protein kinase 3 isoform X2	7764	0
XM_020098420.1	microtubule-associated serine/threonine-protein kinase 3 isoform X3	7979	0
XM_020098421.1	microtubule-associated serine/threonine-protein kinase 3 isoform X4	7957	0
XM_020098422.1	N-acetyllactosaminide beta-1,3-N-acetylglucosaminyltransferase 3 isoform X1	2306	0
XM_020098423.1	N-acetyllactosaminide beta-1,3-N-acetylglucosaminyltransferase 3 isoform X1	2303	0
XM_020098424.1	N-acetyllactosaminide beta-1,3-N-acetylglucosaminyltransferase 3 isoform X1	2005	0
XM_020098425.1	cytoplasmic polyadenylation element-binding protein 3-like isoform X3	4183	0
XM_020098426.1	F-BAR domain only protein 1-like isoform X1	4363	0
XM_020098427.1	F-BAR domain only protein 1-like isoform X1	3949	0
XM_020098428.1	F-BAR domain only protein 1-like isoform X1	4342	0
XM_020098429.1	DNA-binding protein RFX2 isoform X1	4192	0
XM_020098430.1	DNA-binding protein RFX2 isoform X2	4177	0
XM_020098431.1	DNA-binding protein RFX2 isoform X3	4326	0
XM_020098432.1	ephrin type-B receptor 3 isoform X1	7814	0
XM_020098433.1	ephrin type-B receptor 3 isoform X2	7802	0
XM_020098434.1	ephrin type-B receptor 3 isoform X3	7802	0
XM_020098435.1	polyamine-modulated factor 1-like	585	1.10E-120
XM_020098436.1	cytoplasmic polyadenylation element-binding protein 3-like isoform X2	3751	0
XM_020098437.1	ephrin type-B receptor 3 isoform X4	7730	0
XM_020098438.1	ephrin type-B receptor 3 isoform X5	7718	0
XM_020098439.1	ephrin type-B receptor 3 isoform X6	7658	0
XM_020098440.1	ephrin type-B receptor 3 isoform X7	7562	0
XM_020098441.1	ephrin type-B receptor 3 isoform X8	7562	0
XM_020098442.1	ephrin type-B receptor 3 isoform X9	7706	0
XM_020098443.1	homer protein homolog 3 isoform X1	5019	0
XM_020098444.1	homer protein homolog 3 isoform X2	4972	0
XM_020098445.1	ubiquitin-60S ribosomal protein L40	554	1.76E-91
XM_020098446.1	cytoplasmic polyadenylation element-binding protein 3-like isoform X5	4138	0
XM_020098447.1	ubiquitin-60S ribosomal protein L40	519	8.45E-92
XM_020098448.1	receptor-type tyrosine-protein phosphatase S-like isoform X1	7127	0
XM_020098449.1	receptor-type tyrosine-protein phosphatase S-like isoform X2	6978	0
XM_020098450.1	receptor-type tyrosine-protein phosphatase S-like isoform X3	6966	0
XM_020098451.1	receptor-type tyrosine-protein phosphatase S-like isoform X4	5751	0
XM_020098452.1	tyrosine-protein kinase JAK1	6093	0
XM_020098453.1	AT-rich interactive domain-containing protein 3A isoform X1	9513	0
XM_020098454.1	AT-rich interactive domain-containing protein 3A isoform X2	9511	0
XM_020098455.1	cytoplasmic polyadenylation element-binding protein 3-like isoform X6	4114	0
XM_020098456.1	polyadenylate-binding protein 1-like	2997	0
XM_020098457.1	C-type natriuretic peptide	491	8.17E-68
XM_020098458.1	WD repeat-containing protein 63 isoform X2	2866	0
XM_020098459.1	WD repeat-containing protein 63 isoform X2	2930	0
XM_020098460.1	WD repeat-containing protein 63 isoform X2	2840	0
XM_020098461.1	G-protein-signaling modulator 2-like	3011	0
XM_020098462.1	mucolipin-3-like isoform X1	2092	0
XM_020098463.1	mucolipin-3-like isoform X1	2067	0
XM_020098464.1	influenza virus NS1A-binding protein	3399	0
XM_020098465.1	influenza virus NS1A-binding protein	3415	0
XM_020098466.1	lisH domain-containing protein ARMC9 isoform X1	4413	0
XM_020098467.1	lisH domain-containing protein ARMC9 isoform X2	4410	0

XM_020098468.1	serine palmitoyltransferase 3 isoform X1	2139	0
XM_020098469.1	lisH domain-containing protein ARMC9 isoform X3	4377	0
XM_020098470.1	lisH domain-containing protein ARMC9 isoform X4	4446	0
XM_020098471.1	lisH domain-containing protein ARMC9 isoform X1	4364	0
XM_020098472.1	lisH domain-containing protein ARMC9 isoform X5	4308	0
XM_020098473.1	major facilitator superfamily domain-containing protein 8-like	3319	0
XM_020098474.1	DNA-directed RNA polymerases I, II, and III subunit RPABC3	963	1.31E-105
XM_020098475.1	DNA-directed RNA polymerases I, II, and III subunit RPABC3	790	1.55E-106
XM_020098476.1	dolichyl pyrophosphate Man9GlcNAc2 alpha-1,3-glucosyltransferase	2479	0
XM_020098477.1	serine palmitoyltransferase 3 isoform X2	1943	0
XM_020098478.1	forkhead box protein D3	2665	3.49E-166
XM_020098479.1	casein kinase I isoform X1	4297	0
XM_020098480.1	casein kinase I isoform X1	4374	0
XM_020098481.1	casein kinase I isoform X1	4439	0
XM_020098482.1	casein kinase I isoform X1	4229	0
XM_020098483.1	casein kinase I isoform X1	4182	0
XM_020098484.1	casein kinase I isoform X1	4294	0
XM_020098485.1	casein kinase I isoform X1	4193	0
XM_020098486.1	casein kinase I isoform X4	4190	0
XM_020098487.1	diencephalon/mesencephalon homeobox protein 1-B-like	1414	0
XM_020098488.1	amyloid beta A4 precursor protein-binding family A member 1	3441	0
XM_020098489.1	ewing's tumor-associated antigen 1	2933	0
XM_020098490.1	amyloid beta A4 precursor protein-binding family A member 1	3438	0
XM_020098491.1	peroxisomal membrane protein 11C	1732	4.59E-143
XM_020098492.1	ephrin type-B receptor 1-B-like	8913	0
XM_020098493.1	lysine-specific demethylase 4B-like isoform X1	5409	0
XM_020098494.1	lysine-specific demethylase 4B-like isoform X2	5406	0
XM_020098495.1	low-density lipoprotein receptor-related protein 8-like isoform X1	5071	0
XM_020098496.1	low-density lipoprotein receptor-related protein 8-like isoform X1	5053	0
XM_020098497.1	low-density lipoprotein receptor-related protein 8-like isoform X1	5002	0
XM_020098498.1	low-density lipoprotein receptor-related protein 8-like isoform X1	3297	0
XM_020098499.1	ATP-binding cassette sub-family A member 1-like	8118	0
XM_020098500.1	SH3-containing GRB2-like protein 3-interacting protein 1 isoform X1	6363	0
XM_020098501.1	protocadherin Fat 3	7461	0
XM_020098502.1	SH3-containing GRB2-like protein 3-interacting protein 1 isoform X1	6312	0
XM_020098503.1	SH3-containing GRB2-like protein 3-interacting protein 1 isoform X1	6333	0
XM_020098504.1	SH3-containing GRB2-like protein 3-interacting protein 1 isoform X1	6360	0
XM_020098505.1	SH3-containing GRB2-like protein 3-interacting protein 1 isoform X1	6303	0
XM_020098506.1	SH3-containing GRB2-like protein 3-interacting protein 1 isoform X7	6303	0
XM_020098507.1	SH3-containing GRB2-like protein 3-interacting protein 1 isoform X1	6466	0
XM_020098508.1	SH3-containing GRB2-like protein 3-interacting protein 1 isoform X1	6243	0
XM_020098509.1	SH3-containing GRB2-like protein 3-interacting protein 1 isoform X7	6300	0
XM_020098510.1	cAMP-specific 3',5'-cyclic phosphodiesterase 4B isoform X1	5837	0
XM_020098511.1	cAMP-specific 3',5'-cyclic phosphodiesterase 4B isoform X2	6065	0
XM_020098512.1	cAMP-specific 3',5'-cyclic phosphodiesterase 4B isoform X3	5231	0
XM_020098513.1	cAMP-specific 3',5'-cyclic phosphodiesterase 4B isoform X4	4843	0
XM_020098514.1	cAMP-specific 3',5'-cyclic phosphodiesterase 4B isoform X5	5030	0
XM_020098515.1	E3 ubiquitin-protein ligase UHRF1	4105	0
XM_020098516.1	piggyBac transposable element-derived protein 5	3924	0
XM_020098517.1	ubiquitin carboxyl-terminal hydrolase 1 isoform X1	4618	0
XM_020098518.1	ubiquitin carboxyl-terminal hydrolase 1 isoform X2	4615	0
XM_020098519.1	thrombopoietin receptor	2480	0
XM_020098520.1	N(G),N(G)-dimethylarginine dimethylaminohydrolase 1 isoform X2	3729	0
XM_020098521.1	N(G),N(G)-dimethylarginine dimethylaminohydrolase 1 isoform X2	3541	3.46E-124
XM_020098522.1	N(G),N(G)-dimethylarginine dimethylaminohydrolase 1 isoform X2	3316	1.08E-125
XM_020098523.1	N(G),N(G)-dimethylarginine dimethylaminohydrolase 1 isoform X2	4551	1.61E-122
XM_020098524.1	protein ENL isoform X1	8274	0
XM_020098525.1	protein ENL isoform X2	8265	0
XM_020098526.1	rab GTPase-activating protein 1-like isoform X1	3943	0
XM_020098527.1	rab GTPase-activating protein 1-like isoform X1	4192	0
XM_020098528.1	rab GTPase-activating protein 1-like isoform X1	3940	0
XM_020098529.1	rab GTPase-activating protein 1-like isoform X1	3432	0
XM_020098530.1	rab GTPase-activating protein 1-like isoform X4	1730	0
XM_020098531.1	probable G-protein coupled receptor 21	1828	0
XM_020098532.1	long-chain-fatty-acid--CoA ligase ACSBG2-like	4007	0
XM_020098533.1	ras-related protein Rab-3A-like	3706	7.78E-154
XM_020098534.1	axonemal dynein light chain domain-containing protein 1 isoform X1	3848	0
XM_020098535.1	axonemal dynein light chain domain-containing protein 1 isoform X2	3845	0
XM_020098536.1	axonemal dynein light chain domain-containing protein 1 isoform X3	3822	0
XM_020098537.1	tudor domain-containing protein 5 isoform X1	3055	0
XM_020098538.1	tudor domain-containing protein 5 isoform X1	3053	0
XM_020098539.1	tudor domain-containing protein 5 isoform X1	3128	0
XM_020098540.1	tudor domain-containing protein 5 isoform X1	3683	0
XM_020098541.1	erythrocyte band 7 integral membrane protein isoform X1	1195	2.95E-165
XM_020098542.1	erythrocyte band 7 integral membrane protein isoform X1	1255	1.10E-164
XM_020098543.1	protein FAM163B-like	2327	4.12E-85
XM_020098544.1	calmodulin-regulated spectrin-associated protein 2 isoform X1	7337	0
XM_020098545.1	calmodulin-regulated spectrin-associated protein 2 isoform X2	7314	0
XM_020098546.1	calmodulin-regulated spectrin-associated protein 2 isoform X3	7304	0

XM_020098547.1	ankyrin repeat and SOCS box protein 7 isoform X1	5231	0
XM_020098548.1	sentrin-specific protease 5-like isoform X2	3886	0
XM_020098549.1	sentrin-specific protease 5-like isoform X2	3967	0
XM_020098550.1	A disintegrin and metalloproteinase with thrombospondin motifs 6	6882	0
XM_020098551.1	A disintegrin and metalloproteinase with thrombospondin motifs 6	6840	0
XM_020098552.1	exonuclease 1 isoform X1	2637	0
XM_020098553.1	phosphoglucomutase-like protein 5	2227	0
XM_020098554.1	disks large homolog 1 isoform X1	6195	0
XM_020098555.1	disks large homolog 1 isoform X2	5711	0
XM_020098556.1	disks large homolog 1 isoform X3	6043	0
XM_020098557.1	disks large homolog 1 isoform X4	5170	0
XM_020098558.1	disks large homolog 1 isoform X1	4196	0
XM_020098559.1	UPF0690 protein C1orf52 homolog	2740	3.45E-105
XM_020098560.1	cAMP-specific 3',5'-cyclic phosphodiesterase 4D-like isoform X1	5374	0
XM_020098561.1	cAMP-specific 3',5'-cyclic phosphodiesterase 4D-like isoform X2	2718	0
XM_020098562.1	paired box protein Pax-5-like	690	3.82E-151
XM_020098563.1	exonuclease 1 isoform X1	2631	0
XM_020098564.1	cAMP-specific 3',5'-cyclic phosphodiesterase 4D-like isoform X3	2649	0
XM_020098565.1	GRAM domain-containing protein 3-like isoform X1	2779	0
XM_020098566.1	GRAM domain-containing protein 3-like isoform X1	2779	0
XM_020098567.1	serine/threonine-protein kinase Nek7	3813	0
XM_020098568.1	CREB-regulated transcription coactivator 1 isoform X1	7846	0
XM_020098569.1	CREB-regulated transcription coactivator 1 isoform X2	7831	0
XM_020098570.1	CREB-regulated transcription coactivator 1 isoform X3	7759	0
XM_020098571.1	AP-1 complex subunit mu-2-like isoform X1	2878	0
XM_020098572.1	exonuclease 1 isoform X1	2634	0
XM_020098573.1	AP-1 complex subunit mu-1-like isoform X2	2872	0
XM_020098574.1	dipeptidyl peptidase 9-like isoform X1	3939	0
XM_020098575.1	serotransferrin precursor	3822	0
XM_020098576.1	PREDICTED: uncharacterized protein NCBP2-AS2-like	1032	9.04E-65
XM_020098577.1	arrestin domain-containing protein 2	2730	0
XM_020098578.1	putative tyrosine-protein phosphatase auxilin isoform X1	5652	0
XM_020098579.1	putative tyrosine-protein phosphatase auxilin isoform X2	5577	0
XM_020098580.1	exonuclease 1 isoform X1	2584	0
XM_020098581.1	putative tyrosine-protein phosphatase auxilin isoform X1	5696	0
XM_020098582.1	endogenous retrovirus group 3 member 1 Env polyprotein-like	2047	0
XM_020098583.1	DET1- and DDB1-associated protein 1	2573	1.30E-65
XM_020098584.1	anoctamin-8-like isoform X1	6543	0
XM_020098585.1	anoctamin-8-like isoform X2	6147	0
XM_020098586.1	cartilage oligomeric matrix protein	2760	0
XM_020098587.1	solute carrier family 35 member E2B	2789	0
XM_020098588.1	prostaglandin G/H synthase 2	2755	0
XM_020098589.1	phosducin-like protein isoform X2	2182	2.95E-149
XM_020098590.1	mediator of RNA polymerase II transcription subunit 26-like isoform X1	3169	0
XM_020098591.1	mediator of RNA polymerase II transcription subunit 26-like isoform X2	3166	0
XM_020098592.1	pleckstrin homology domain-containing family A member 3	2135	0
XM_020098593.1	transmembrane protein 79-like	1880	0
XM_020098594.1	CTD small phosphatase-like protein 2	1787	0
XM_020098595.1	inactive tyrosine-protein kinase transmembrane receptor ROR1 isoform X1	4697	0
XM_020098596.1	inactive tyrosine-protein kinase transmembrane receptor ROR1 isoform X2	4635	0
XM_020098597.1	interferon-induced protein 44-like isoform X1	4122	0
XM_020098598.1	interferon-induced protein 44-like isoform X2	4119	0
XM_020098599.1	interferon-induced protein 44-like isoform X3	4116	0
XM_020098600.1	MOB kinase activator 3A	1426	3.41E-163
XM_020098601.1	DAZ-associated protein 1 isoform X1	1884	0
XM_020098602.1	DAZ-associated protein 1 isoform X2	1883	0
XM_020098603.1	DAZ-associated protein 1 isoform X3	1867	0
XM_020098604.1	DAZ-associated protein 1 isoform X4	1862	0
XM_020098605.1	adhesion G protein-coupled receptor B3 isoform X1	5067	0
XM_020098606.1	DAZ-associated protein 1 isoform X5	1984	0
XM_020098607.1	cytosolic phospholipase A2	4923	0
XM_020098608.1	clusterin-associated protein 1	1708	0
XM_020098609.1	clusterin-associated protein 1	1706	0
XM_020098610.1	kelch-like protein 26	3649	0
XM_020098611.1	AFG3-like protein 1	4082	0
XM_020098612.1	adhesion G protein-coupled receptor B3 isoform X1	5439	0
XM_020098613.1	dysbindin domain-containing protein 1	1830	1.76E-105
XM_020098614.1	proteasome subunit beta type-1	1148	1.62E-177
XM_020098615.1	differentially expressed in FDCP 8 homolog	3012	0
XM_020098616.1	PREDICTED: aquaporin-9	1023	0
XM_020098617.1	hepatic triacylglycerol lipase	881	0
XM_020098618.1	transcription factor SOX-6-like isoform X2	2714	0
XM_020098619.1	transcription factor SOX-6-like isoform X2	5719	0
XM_020098620.1	adhesion G protein-coupled receptor B3 isoform X1	5300	0
XM_020098621.1	transcription factor SOX-6-like isoform X2	2663	0
XM_020098622.1	transcription factor SOX-6-like isoform X2	2604	0
XM_020098623.1	transmembrane gamma-carboxyglutamic acid protein 4	2100	4.86E-125
XM_020098624.1	ribonuclease T2 isoform X2	2134	1.06E-162
XM_020098625.1	ribonuclease T2 isoform X2	2254	9.72E-160

XM_020098626.1	ribonuclease T2 isoform X2	2250	9.43E-160
XM_020098627.1	ribonuclease T2 isoform X2	2134	2.83E-160
XM_020098628.1	eukaryotic translation initiation factor 3 subunit M	1365	0
XM_020098629.1	adhesion G protein-coupled receptor B3 isoform X1	4631	0
XM_020098630.1	putative tRNA (cytidine(32)/guanosine(34)-2'-O)-methyltransferase	3425	0
XM_020098631.1	PREDICTED: uncharacterized protein LOC109636723	1427	1.28E-87
XM_020098632.1	putative polypeptide N-acetylgalactosaminyltransferase-like protein 3	1320	0
XM_020098633.1	polypeptide N-acetylgalactosaminyltransferase 18-like	1353	0
XM_020098634.1	heterogeneous nuclear ribonucleoprotein H3	2086	0
XM_020098635.1	Wilms tumor protein isoform X1	2270	0
XM_020098636.1	Wilms tumor protein isoform X2	2267	0
XM_020098637.1	Wilms tumor protein isoform X3	2261	0
XM_020098638.1	adhesion G protein-coupled receptor B3 isoform X1	5207	0
XM_020098639.1	Wilms tumor protein isoform X4	2258	0
XM_020098640.1	PH domain leucine-rich repeat-containing protein phosphatase 2 isoform X1	7160	0
XM_020098641.1	PH domain leucine-rich repeat-containing protein phosphatase 2 isoform X2	7157	0
XM_020098642.1	synaptotagmin-7 isoform X1	5390	0
XM_020098643.1	synaptotagmin-7 isoform X2	5267	0
XM_020098644.1	synaptotagmin-7 isoform X3	5192	0
XM_020098645.1	AMP deaminase 3 isoform X1	3036	0
XM_020098646.1	AMP deaminase 3 isoform X2	2416	0
XM_020098647.1	adhesion G protein-coupled receptor B3 isoform X1	5334	0
XM_020098648.1	AMP deaminase 3 isoform X3	2357	0
XM_020098649.1	AMP deaminase 3 isoform X3	2359	0
XM_020098650.1	lymphatic vessel endothelial hyaluronic acid receptor 1	1012	8.75E-172
XM_020098651.1	ribosomal protein S6 kinase alpha-2-like	2668	0
XM_020098652.1	PREDICTED: uncharacterized protein LOC109636734 isoform X1	1431	3.11E-138
XM_020098653.1	PREDICTED: uncharacterized protein LOC109636734 isoform X2	1386	3.05E-125
XM_020098654.1	F-spondin extracellular matrix protein	3434	0
XM_020098655.1	glycine cleavage system H protein, mitochondrial	1013	1.23E-108
XM_020098656.1	adhesion G protein-coupled receptor B3 isoform X6	5189	0
XM_020098657.1	cysteine-rich secretory protein LCCL domain-containing 2	3769	0
XM_020098658.1	cysteine-rich secretory protein LCCL domain-containing 2	3772	0
XM_020098659.1	liprin-beta-2 isoform X1	3617	0
XM_020098660.1	liprin-beta-2 isoform X2	3584	0
XM_020098661.1	liprin-beta-2 isoform X3	3287	0
XM_020098662.1	centriole, cilia and spindle-associated protein isoform X1	2307	0
XM_020098663.1	centriole, cilia and spindle-associated protein isoform X2	2301	0
XM_020098664.1	synaptic vesicle membrane protein VAT-1 homolog	3617	0
XM_020098665.1	adhesion G protein-coupled receptor B3 isoform X7	5133	0
XM_020098666.1	cytochrome P450 2J2-like	2146	0
XM_020098667.1	forkhead box protein F1	2362	0
XM_020098668.1	protein RIC-3-like isoform X2	3372	0
XM_020098669.1	protein RIC-3-like isoform X2	3240	2.93E-166
XM_020098670.1	protein RIC-3-like isoform X2	3211	2.33E-166
XM_020098671.1	tripartite motif-containing protein 66	4985	0
XM_020098672.1	tripartite motif-containing protein 66	4948	0
XM_020098673.1	tripartite motif-containing protein 66	4892	0
XM_020098674.1	tripartite motif-containing protein 66	4450	0
XM_020098675.1	potassium channel subfamily K member 2-like	1047	0
XM_020098676.1	CUB and sushi domain-containing protein 1-like	12282	0
XM_020098677.1	diphosphomevalonate decarboxylase	1433	0
XM_020098678.1	tyrosine-protein kinase Fer-like isoform X1	3197	0
XM_020098679.1	PREDICTED: calretinin-like	1283	0
XM_020098680.1	GPI mannosyltransferase 3	1423	0
XM_020098681.1	DNA-binding protein RFX7-like	888	0
XM_020098682.1	transcription factor 12	1058	8.16E-171
XM_020098683.1	unconventional myosin-Ie-like	2400	0
XM_020098684.1	PREDICTED: copine-7-like	5937	0
XM_020098685.1	polycystic kidney disease protein 1-like 2	7760	0
XM_020098686.1	ecto-NOX disulfide-thiol exchanger 2 isoform X1	3198	0
XM_020098687.1	neuroblast differentiation-associated protein AHNAK-like	5280	0
XM_020098688.1	interferon-induced, double-stranded RNA-activated protein kinase-like	2548	0
XM_020098689.1	ubiquitin carboxyl-terminal hydrolase 47	5291	0
XM_020098690.1	lymphoid-restricted membrane protein-like	484	5.81E-70
XM_020098691.1	protein MRVI1	4606	0
XM_020098692.1	switch-associated protein 70-like	1965	0
XM_020098693.1	signal peptide, CUB and EGF-like domain-containing protein 2	2089	0
XM_020098694.1	cGMP-inhibited 3',5'-cyclic phosphodiesterase B	4066	0
XM_020098695.1	PREDICTED: exostosin-2	3362	0
XM_020098696.1	programmed cell death protein 5	1094	7.43E-66
XM_020098697.1	prolyl 3-hydroxylase OGFOD1	1774	0
XM_020098698.1	eukaryotic translation initiation factor 2-alpha kinase 3-like	1741	0
XM_020098699.1	tumor necrosis factor receptor type 1-associated DEATH domain protein	1571	0
XM_020098700.1	melanocyte-stimulating hormone receptor	2618	0
XM_020098701.1	heterogeneous nuclear ribonucleoprotein C-like	2048	5.38E-54
XM_020098702.1	interferon regulatory factor 8	1962	0
XM_020098703.1	LIM domain only protein 3 isoform X2	1710	7.39E-108
XM_020098704.1	cytochrome b-245 light chain	944	5.41E-102

XM_020098705.1	putative sodium-coupled neutral amino acid transporter 8	2449	0
XM_020098706.1	histone-lysine N-methyltransferase PRDM9-like	1947	0
XM_020098707.1	synaptotagmin-10-like isoform X2	2634	0
XM_020098708.1	serine-rich coiled-coil domain-containing protein 2-like	6198	0
XM_020098709.1	tubby protein homolog	1991	0
XM_020098710.1	DNA-directed RNA polymerases I, II, and III subunit RPABC5	528	3.04E-43
XM_020098711.1	C-type lectin domain family 18 member A-like isoform X1	1929	0
XM_020098712.1	C-type lectin domain family 18 member A-like isoform X1	1256	0
XM_020098713.1	ras association domain-containing protein 10-like	1976	0
XM_020098714.1	PREDICTED: ADM	1597	1.68E-108
XM_020098715.1	serine-rich coiled-coil domain-containing protein 2-like	6142	0
XM_020098716.1	DEP domain-containing protein 7	1706	0
XM_020098717.1	dickkopf-related protein 3	2031	0
XM_020098718.1	hepatocyte nuclear factor 4-beta-like	2224	0
XM_020098719.1	5-hydroxyisourate hydrolase-like	1000	5.45E-97
XM_020098720.1	stereocilin-like isoform X1	5925	0
XM_020098721.1	substance-K receptor	1602	0
XM_020098722.1	calcitonin-1-like isoform X1	1017	2.20E-118
XM_020098723.1	calcitonin-1-like isoform X1	968	1.23E-118
XM_020098724.1	calcitonin-1-like isoform X1	1284	1.07E-113
XM_020098725.1	fibulin-7-like isoform X1	2092	0
XM_020098726.1	fibulin-7-like isoform X2	2050	0
XM_020098727.1	heparan-alpha-glucosaminide N-acetyltransferase isoform X2	2784	0
XM_020098728.1	UPF0606 protein KIAA1549L-like	4278	0
XM_020098729.1	homeobox protein aristaless-like 4	1466	0
XM_020098730.1	protein PIGBOS1	326	2.45E-33
XM_020098731.1	C-type lectin domain family 3 member A	1265	1.07E-123
XM_020098732.1	PREDICTED: uncharacterized protein LOC109636803 isoform X1	1171	0
XM_020098733.1	PREDICTED: uncharacterized protein LOC109636803 isoform X2	1030	5.35E-163
XM_020098734.1	neuritin-like protein	507	2.78E-124
XM_020098735.1	ATM interactor	4851	0
XM_020098736.1	chromodomain Y-like protein 2	3117	0
XM_020098737.1	proto-oncogene tyrosine-protein kinase receptor Ret	4252	0
XM_020098738.1	centromere protein N	1758	0
XM_020098739.1	protein phosphatase 1 regulatory subunit 3D-like	3203	0
XM_020098740.1	COX assembly mitochondrial protein 2 homolog isoform X2	1941	3.96E-55
XM_020098741.1	COX assembly mitochondrial protein 2 homolog isoform X2	1915	1.48E-47
XM_020098742.1	COX assembly mitochondrial protein 2 homolog isoform X2	1688	5.52E-48
XM_020098743.1	AP-1 complex subunit gamma-1 isoform X1	7116	0
XM_020098744.1	AP-1 complex subunit gamma-1 isoform X2	7110	0
XM_020098745.1	AP-1 complex subunit gamma-1 isoform X3	7107	0
XM_020098746.1	zinc finger protein 821 isoform X1	4640	0
XM_020098747.1	zinc finger protein 821 isoform X2	4637	0
XM_020098748.1	zinc finger protein 821 isoform X3	4388	0
XM_020098749.1	zinc finger protein 821 isoform X2	4660	0
XM_020098750.1	proprotein convertase subtilisin/kexin type 5 isoform X1	7344	0
XM_020098751.1	proprotein convertase subtilisin/kexin type 5 isoform X1	6962	0
XM_020098752.1	proprotein convertase subtilisin/kexin type 5 isoform X1	7362	0
XM_020098753.1	kelch-like protein 10	2238	0
XM_020098754.1	pleckstrin homology domain-containing family A member 7-like isoform X1	4842	0
XM_020098755.1	pleckstrin homology domain-containing family A member 7-like isoform X2	4792	0
XM_020098756.1	pleckstrin homology domain-containing family A member 7-like isoform X3	4789	0
XM_020098757.1	pleckstrin homology domain-containing family A member 7-like isoform X4	4774	0
XM_020098758.1	pleckstrin homology domain-containing family A member 7-like isoform X5	4704	0
XM_020098759.1	pleckstrin homology domain-containing family A member 7-like isoform X6	4726	0
XM_020098760.1	pleckstrin homology domain-containing family A member 7-like isoform X7	4711	0
XM_020098761.1	pleckstrin homology domain-containing family A member 7-like isoform X8	4657	0
XM_020098762.1	pleckstrin homology domain-containing family A member 7-like isoform X9	4653	0
XM_020098763.1	pleckstrin homology domain-containing family A member 7-like isoform X10	4638	0
XM_020098764.1	pleckstrin homology domain-containing family A member 7-like isoform X11	4584	0
XM_020098765.1	pleckstrin homology domain-containing family A member 7-like isoform X12	4893	0
XM_020098766.1	pleckstrin homology domain-containing family A member 7-like isoform X13	4878	0
XM_020098767.1	pleckstrin homology domain-containing family A member 7-like isoform X5	4484	0
XM_020098768.1	caprin-1-like isoform X1	2661	0
XM_020098769.1	caprin-1-like isoform X2	2529	0
XM_020098770.1	nucleobindin-2 isoform X1	3200	0
XM_020098771.1	ubiquitin carboxyl-terminal hydrolase 10 isoform X1	5873	0
XM_020098772.1	ubiquitin carboxyl-terminal hydrolase 10 isoform X2	5870	0
XM_020098773.1	ubiquitin carboxyl-terminal hydrolase 10 isoform X3	5813	0
XM_020098774.1	ubiquitin carboxyl-terminal hydrolase 10 isoform X4	5810	0
XM_020098775.1	MARVEL domain-containing protein 3 isoform X2	1982	0
XM_020098776.1	MARVEL domain-containing protein 3 isoform X2	1468	0
XM_020098777.1	DET1- and DDB1-associated protein 1-like	2267	2.69E-68
XM_020098778.1	MARVEL domain-containing protein 3 isoform X2	1858	0
XM_020098779.1	unhealthy ribosome biogenesis protein 2 homolog	5696	0
XM_020098780.1	unhealthy ribosome biogenesis protein 2 homolog	5705	0
XM_020098781.1	unhealthy ribosome biogenesis protein 2 homolog	5693	0
XM_020098782.1	unhealthy ribosome biogenesis protein 2 homolog	5710	0
XM_020098783.1	unhealthy ribosome biogenesis protein 2 homolog	5702	0

XM_020098784.1	unhealthy ribosome biogenesis protein 2 homolog	5650	0
XM_020098785.1	ATP-binding cassette sub-family B member 10, mitochondrial	4843	0
XM_020098786.1	genetic suppressor element 1	9085	0
XM_020098787.1	DNA replication complex GINS protein PSF2	954	2.73E-123
XM_020098788.1	26S protease regulatory subunit 6A	1540	0
XM_020098789.1	programmed cell death protein 2 isoform X1	1869	0
XM_020098790.1	UPF0489 protein C5orf22 homolog	2600	0
XM_020098791.1	programmed cell death protein 2 isoform X2	1866	0
XM_020098792.1	TATA-box-binding protein	2581	0
XM_020098793.1	lysosomal protective protein-like	1822	0
XM_020098794.1	putative sodium-coupled neutral amino acid transporter 8	3688	0
XM_020098795.1	transcription factor E2F5-like	1493	0
XM_020098796.1	transcription factor E2F5-like	1111	0
XM_020098797.1	growth arrest-specific protein 8	1572	0
XM_020098798.1	conserved oligomeric Golgi complex subunit 4	5244	0
XM_020098799.1	guanine nucleotide-binding protein G(o) subunit alpha	2861	0
XM_020098800.1	mucin-5AC-like isoform X2	3375	0
XM_020098801.1	mucin-5AC-like isoform X2	3272	0
XM_020098802.1	outer dense fiber protein 2-like isoform X2	3489	0
XM_020098803.1	mucin-5AC-like isoform X2	2989	0
XM_020098804.1	transcriptional repressor CTCF-like isoform X1	3634	0
XM_020098805.1	transcriptional repressor CTCF-like isoform X1	2509	0
XM_020098806.1	actin, alpha cardiac	1728	0
XM_020098807.1	sodium/potassium/calcium exchanger 3-like	2318	0
XM_020098808.1	importin-8 isoform X4	4228	0
XM_020098809.1	nucleolar RNA helicase 2-like	2770	0
XM_020098810.1	mitochondrial pyruvate carrier 1	1344	5.48E-77
XM_020098811.1	outer dense fiber protein 2-like isoform X2	3563	0
XM_020098812.1	pre-mRNA-splicing factor ATP-dependent RNA helicase PRP16 isoform X1	4904	0
XM_020098813.1	pre-mRNA-splicing factor ATP-dependent RNA helicase PRP16 isoform X2	4901	0
XM_020098814.1	cytochrome b5	1464	1.57E-112
XM_020098815.1	centromere protein F	9215	0
XM_020098816.1	centromere protein F	9255	0
XM_020098817.1	homeodomain-interacting protein kinase 3	7207	0
XM_020098818.1	glucose-fructose oxidoreductase domain-containing protein 2-like	5408	0
XM_020098819.1	outer dense fiber protein 2-like isoform X2	3560	0
XM_020098820.1	THAP domain-containing protein 11	2579	7.31E-154
XM_020098821.1	DENN domain-containing protein 5A isoform X1	5446	0
XM_020098822.1	DENN domain-containing protein 5A isoform X2	5374	0
XM_020098823.1	DENN domain-containing protein 5A isoform X3	5356	0
XM_020098824.1	DENN domain-containing protein 5A isoform X4	5284	0
XM_020098825.1	proline-rich protein 12-like	6200	0
XM_020098826.1	putative hexokinase HKDC1 isoform X2	4429	0
XM_020098827.1	zinc finger homeobox protein 3	12735	0
XM_020098828.1	outer dense fiber protein 2-like isoform X2	3506	0
XM_020098829.1	zinc finger homeobox protein 3	12125	0
XM_020098830.1	RUN and FYVE domain-containing protein 2 isoform X3	3770	0
XM_020098831.1	RUN and FYVE domain-containing protein 2 isoform X3	4749	0
XM_020098832.1	RUN and FYVE domain-containing protein 2 isoform X3	3783	0
XM_020098833.1	RUN and FYVE domain-containing protein 2 isoform X3	4016	0
XM_020098834.1	RUN and FYVE domain-containing protein 2 isoform X4	3325	0
XM_020098835.1	cingulin-like protein 1	4918	0
XM_020098836.1	sn1-specific diacylglycerol lipase alpha isoform X1	7437	0
XM_020098837.1	sn1-specific diacylglycerol lipase alpha isoform X2	7420	0
XM_020098838.1	outer dense fiber protein 2-like isoform X2	3573	0
XM_020098839.1	sn1-specific diacylglycerol lipase alpha isoform X3	4992	0
XM_020098840.1	sn1-specific diacylglycerol lipase alpha isoform X4	7395	0
XM_020098841.1	E3 ubiquitin-protein ligase Arkadia isoform X1	4691	0
XM_020098842.1	E3 ubiquitin-protein ligase Arkadia isoform X1	5024	0
XM_020098843.1	E3 ubiquitin-protein ligase Arkadia isoform X1	4604	0
XM_020098844.1	protein FAM65A-like	3867	0
XM_020098845.1	inactive serine/threonine-protein kinase VRK3 isoform X1	2910	0
XM_020098846.1	inactive serine/threonine-protein kinase VRK3 isoform X2	2904	0
XM_020098847.1	SAFB-like transcription modulator	4110	0
XM_020098848.1	F-actin-methionine sulfoxide oxidase MICAL2 isoform X1	5739	0
XM_020098849.1	F-actin-methionine sulfoxide oxidase MICAL2 isoform X2	5631	0
XM_020098850.1	F-actin-methionine sulfoxide oxidase MICAL2 isoform X3	5382	0
XM_020098851.1	intraflagellar transport protein 22 homolog	1022	6.27E-137
XM_020098852.1	F-actin-methionine sulfoxide oxidase MICAL2 isoform X4	3647	0
XM_020098853.1	F-actin-methionine sulfoxide oxidase MICAL2 isoform X5	5274	0
XM_020098854.1	rho-related GTP-binding protein RhoU	6173	1.51E-128
XM_020098855.1	DNA replication ATP-dependent helicase/nuclease DNA2 isoform X1	5269	0
XM_020098856.1	DNA replication ATP-dependent helicase/nuclease DNA2 isoform X2	5266	0
XM_020098857.1	DNA replication ATP-dependent helicase/nuclease DNA2 isoform X3	5170	0
XM_020098858.1	phenazine biosynthesis-like domain-containing protein	1576	0
XM_020098859.1	transcriptional enhancer factor TEF-1 isoform X1	4996	0
XM_020098860.1	transcriptional enhancer factor TEF-1 isoform X1	5327	0
XM_020098861.1	transcriptional enhancer factor TEF-1 isoform X1	4856	0
XM_020098862.1	transcriptional enhancer factor TEF-1 isoform X1	4984	0

XM_020098863.1	transcriptional enhancer factor TEF-1 isoform X1	4932	0
XM_020098864.1	transcriptional enhancer factor TEF-1 isoform X1	4932	0
XM_020098865.1	60S ribosomal protein L5-like	1115	0
XM_020098866.1	transcriptional enhancer factor TEF-1 isoform X5	4920	0
XM_020098867.1	transcriptional enhancer factor TEF-1 isoform X6	4855	0
XM_020098868.1	N-lysine methyltransferase SMYD2-A-like	3985	0
XM_020098869.1	disintegrin and metalloproteinase domain-containing protein 10-like	4957	0
XM_020098870.1	protogenin isoform X1	7203	0
XM_020098871.1	protogenin isoform X2	7090	0
XM_020098872.1	polypeptide N-acetylgalactosaminyltransferase 2 isoform X1	5080	0
XM_020098873.1	polypeptide N-acetylgalactosaminyltransferase 2 isoform X2	4945	0
XM_020098874.1	GTPase HRas	2818	1.56E-132
XM_020098875.1	E3 ubiquitin-protein ligase NEDD4	5750	0
XM_020098876.1	myotubularin-related protein 10 isoform X1	5122	0
XM_020098877.1	guanine nucleotide-binding protein G(I)/G(S)/G(O) subunit gamma-12-like	1441	1.35E-43
XM_020098878.1	myotubularin-related protein 10 isoform X1	5014	0
XM_020098879.1	myotubularin-related protein 10 isoform X1	5119	0
XM_020098880.1	aspartate aminotransferase, cytoplasmic-like	2524	0
XM_020098881.1	coatomer subunit beta	3295	0
XM_020098882.1	kinesin-like protein KIF7 isoform X1	4604	0
XM_020098883.1	kinesin-like protein KIF7 isoform X1	4865	0
XM_020098884.1	kinesin-like protein KIF7 isoform X1	4601	0
XM_020098885.1	eukaryotic translation initiation factor 4 gamma 2-like	2728	0
XM_020098886.1	calumenin	2354	0
XM_020098887.1	UDP-N-acetylglucosamine--peptide N-acetylglucosaminyltransferase 110 kDa subunit isoform X9	3936	0
XM_020098888.1	meiosis-specific nuclear structural protein 1 isoform X2	2178	0
XM_020098889.1	meiosis-specific nuclear structural protein 1 isoform X2	2489	0
XM_020098890.1	MAP6 domain-containing protein 1-like isoform X1	1499	0
XM_020098891.1	meiosis-specific nuclear structural protein 1 isoform X2	2547	0
XM_020098892.1	testis-expressed sequence 9 protein isoform X1	1352	0
XM_020098893.1	testis-expressed sequence 9 protein isoform X2	1310	0
XM_020098894.1	cell cycle progression protein 1 isoform X1	2999	0
XM_020098895.1	cell cycle progression protein 1 isoform X2	2987	0
XM_020098896.1	cell cycle progression protein 1 isoform X3	2904	0
XM_020098897.1	cell cycle progression protein 1 isoform X4	2891	0
XM_020098898.1	ubiquitin carboxyl-terminal hydrolase MINDY-2 isoform X1	5604	0
XM_020098899.1	MAP6 domain-containing protein 1-like isoform X2	2029	0
XM_020098900.1	ubiquitin carboxyl-terminal hydrolase MINDY-2 isoform X2	5601	0

XM_020098901.1	myocardial zonula adherens protein-like isoform X1	4143	0
XM_020098902.1	myocardial zonula adherens protein-like isoform X2	4131	0
XM_020098903.1	myocardial zonula adherens protein-like isoform X3	4098	0
XM_020098904.1	protein GRINL1A	1839	0
XM_020098905.1	ER membrane protein complex subunit 8	2110	8.27E-147
XM_020098906.1	cytochrome c oxidase subunit 4 isoform 1, mitochondrial	847	9.34E-108
XM_020098907.1	cytochrome c oxidase subunit 4 isoform 1, mitochondrial	800	1.76E-102
XM_020098908.1	retinal dehydrogenase 2	2559	0
XM_020098909.1	vacuolar protein sorting-associated protein 26B-like	2734	0
XM_020098910.1	PREDICTED: ataxin-1-like	7869	0
XM_020098911.1	PREDICTED: ataxin-1-like	8048	0
XM_020098912.1	endothelin-converting enzyme 2-like	1665	1.55E-169
XM_020098913.1	nuclear factor of activated T-cells 5-like isoform X1	8624	0
XM_020098914.1	nuclear factor of activated T-cells 5-like isoform X1	8324	0
XM_020098915.1	methenyltetrahydrofolate synthase domain-containing protein isoform X1	2764	0
XM_020098916.1	methenyltetrahydrofolate synthase domain-containing protein isoform X2	2764	0
XM_020098917.1	transmembrane protein 41B	1497	0
XM_020098918.1	cleavage stimulation factor subunit 3	2732	0
XM_020098919.1	cleavage and polyadenylation specificity factor subunit 7	2117	0
XM_020098920.1	endothelin-converting enzyme 2-like	1644	1.47E-169
XM_020098921.1	succinate dehydrogenase assembly factor 2, mitochondrial	1046	9.35E-118
XM_020098922.1	paired box protein Pax-6 isoform X1	2832	0
XM_020098923.1	paired box protein Pax-6 isoform X1	2799	0
XM_020098924.1	paired box protein Pax-6 isoform X1	2804	0
XM_020098925.1	paired box protein Pax-6 isoform X1	2790	0
XM_020098926.1	paired box protein Pax-6 isoform X1	2873	0
XM_020098927.1	paired box protein Pax-6 isoform X5	2761	0
XM_020098928.1	paired box protein Pax-6 isoform X1	2847	0
XM_020098929.1	paired box protein Pax-6 isoform X5	2808	0
XM_020098930.1	paired box protein Pax-6 isoform X1	2698	0
XM_020098931.1	paired box protein Pax-6 isoform X1	2403	0
XM_020098932.1	PHD finger protein 10	2311	0
XM_020098933.1	lysine--tRNA ligase isoform X1	2514	0
XM_020098934.1	lysine--tRNA ligase isoform X2	2354	0
XM_020098935.1	hyaluronan synthase 1-like	4903	0
XM_020098936.1	transcription factor Maf-like	5046	2.87E-146
XM_020098937.1	PREDICTED: peripherin-2-like	1062	0
XM_020098938.1	transcription factor Maf-like	1634	1.27E-157
XM_020098939.1	BTB/POZ domain-containing protein 10 isoform X2	2326	0
XM_020098940.1	BTB/POZ domain-containing protein 10 isoform X2	2323	0
XM_020098941.1	BTB/POZ domain-containing protein 10 isoform X3	2217	0

XM_020098942.1	BTB/POZ domain-containing protein 10 isoform X4	2214	0
XM_020098943.1	proteasome subunit alpha type-4	1255	1.04E-170
XM_020098944.1	BTB/POZ domain-containing protein 10 isoform X2	2331	0
XM_020098945.1	BTB/POZ domain-containing protein 10 isoform X2	2334	0
XM_020098946.1	BTB/POZ domain-containing protein 10 isoform X2	2227	0
XM_020098947.1	myomesin-2-like isoform X1	4697	0
XM_020098948.1	myomesin-2-like isoform X1	4602	0
XM_020098949.1	tetraspanin-33-like isoform X3	3308	0
XM_020098950.1	transcription factor 25	2631	0
XM_020098951.1	putative hexokinase HKDC1 isoform X1	3581	0
XM_020098952.1	putative hexokinase HKDC1 isoform X2	3578	0
XM_020098953.1	dual specificity protein phosphatase 22-B-like	1623	3.63E-139
XM_020098954.1	bolA-like protein 2	981	1.28E-64
XM_020098955.1	PAS domain-containing serine/threonine-protein kinase	5082	0
XM_020098956.1	myotubularin-related protein 13 isoform X1	4612	0
XM_020098957.1	myotubularin-related protein 13 isoform X2	4537	0
XM_020098958.1	ATP-dependent RNA helicase SUPV3L1, mitochondrial	3243	0
XM_020098959.1	zinc finger protein 280D isoform X1	4299	0
XM_020098960.1	zinc finger protein 280D isoform X2	4296	0
XM_020098961.1	elongator complex protein 4	1939	0
XM_020098962.1	mitochondrial inner membrane protease subunit 1	825	2.90E-125
XM_020098963.1	suppression of tumorigenicity 5 protein	4156	0
XM_020098964.1	suppression of tumorigenicity 5 protein	4002	0
XM_020098965.1	60S ribosomal protein L13	794	8.27E-139
XM_020098966.1	transmembrane protein 9	1980	1.57E-101
XM_020098967.1	cell wall protein DAN4-like isoform X2	2453	3.25E-152
XM_020098968.1	cleavage and polyadenylation specificity factor subunit 5	1237	5.80E-169
XM_020098969.1	ras-related protein Rab-27B-like	1084	2.40E-156
XM_020098970.1	KIF1-binding protein	2660	0
XM_020098971.1	ras-related protein Rab-4A	3344	2.54E-148
XM_020098972.1	ras-related protein Rab-4A	3339	2.43E-148
XM_020098973.1	ras-related protein Rab-4A	2675	1.04E-150
XM_020098974.1	synaptosomal-associated protein 29	1567	4.78E-158
XM_020098975.1	synaptosomal-associated protein 29	1555	2.09E-158
XM_020098976.1	synaptosomal-associated protein 29	1513	2.91E-158
XM_020098977.1	methylcrotonoyl-CoA carboxylase beta chain, mitochondrial	2090	0
XM_020098978.1	microsomal glutathione S-transferase 3	1117	7.45E-111
XM_020098979.1	kinase suppressor of Ras 2 isoform X1	6080	0
XM_020098980.1	kinase suppressor of Ras 2 isoform X2	6077	0
XM_020098981.1	kinase suppressor of Ras 2 isoform X3	3616	0
XM_020098982.1	serine/arginine repetitive matrix protein 4 isoform X2	5422	7.88E-109
XM_020098983.1	serine/arginine repetitive matrix protein 4 isoform X2	5335	4.35E-141
XM_020098984.1	RING finger protein 37	3452	0
XM_020098985.1	proline dehydrogenase 1, mitochondrial-like	2946	0
XM_020098986.1	phospholipid phosphatase 1-like	1981	0
XM_020098987.1	protein NipSnap homolog 1-like	1567	0
XM_020098988.1	ras-specific guanine nucleotide-releasing factor 2 isoform X1	6274	0
XM_020098989.1	ras-specific guanine nucleotide-releasing factor 2 isoform X2	6229	0
XM_020098990.1	ras-specific guanine nucleotide-releasing factor 2 isoform X3	6184	0
XM_020098991.1	ubiquitin-conjugating enzyme E2 variant 2	823	5.17E-81
XM_020098992.1	ras-specific guanine nucleotide-releasing factor 2 isoform X4	6181	0
XM_020098993.1	centrosomal protein of 78 kDa	3215	0
XM_020098994.1	F-box only protein 21 isoform X1	2965	0
XM_020098995.1	F-box only protein 21 isoform X2	2949	0
XM_020098996.1	F-box only protein 21 isoform X3	2934	0
XM_020098997.1	hemicentin-2-like isoform X3	4901	0
XM_020098998.1	hemicentin-2-like isoform X3	4877	0
XM_020098999.1	hemicentin-2-like isoform X3	3437	0
XM_020099000.1	dual specificity protein phosphatase 2	1574	0
XM_020099001.1	coiled-coil domain-containing protein 84	1943	0
XM_020099002.1	protease-associated domain-containing protein 1 isoform X1	1012	1.26E-173
XM_020099003.1	protease-associated domain-containing protein 1 isoform X2	1153	1.37E-161
XM_020099004.1	spindlin-W-like isoform X2	1843	0
XM_020099005.1	protease-associated domain-containing protein 1 isoform X3	866	7.22E-162
XM_020099006.1	protease-associated domain-containing protein 1 isoform X2	917	3.84E-145
XM_020099007.1	GATS-like protein 3	2087	0
XM_020099008.1	cysteine dioxygenase type 1	1704	9.11E-149
XM_020099009.1	transcription factor BTF3	902	1.84E-97
XM_020099010.1	integrin alpha-2	4074	0
XM_020099011.1	flavin reductase (NADPH)-like	1658	5.36E-160
XM_020099012.1	flavin reductase (NADPH)-like	1697	8.34E-160
XM_020099013.1	spindlin-W-like isoform X2	1839	5.36E-159
XM_020099014.1	calsenilin-like isoform X1	1897	0
XM_020099015.1	calsenilin isoform X2	987	0
XM_020099016.1	calsenilin isoform X3	1278	4.70E-158
XM_020099017.1	calsenilin isoform X4	829	1.16E-160
XM_020099018.1	somatomedin-B and thrombospondin type-1 domain-containing protein-like isoform X1	2260	0
XM_020099019.1	somatomedin-B and thrombospondin type-1 domain-containing protein-like isoform X2	2258	0
XM_020099020.1	SH2B adapter protein 3	3679	0

XM_020099021.1	SH2B adapter protein 3	3676	0
XM_020099022.1	spindlin-W-like isoform X2	1737	2.74E-158
XM_020099023.1	reticulon-4 receptor-like 1	2526	0
XM_020099024.1	glycerol-3-phosphate acyltransferase 2, mitochondrial-like	3013	0
XM_020099025.1	glycerol-3-phosphate acyltransferase 2, mitochondrial-like	3040	0
XM_020099026.1	tissue-type plasminogen activator isoform X1	2976	0
XM_020099027.1	tissue-type plasminogen activator isoform X1	2957	0
XM_020099028.1	tissue-type plasminogen activator isoform X1	2882	0
XM_020099029.1	tissue-type plasminogen activator isoform X1	2747	0
XM_020099030.1	calcium-binding protein 7-like	4038	4.05E-147
XM_020099031.1	spindlin-W-like isoform X2	1805	1.75E-158
XM_020099032.1	transmembrane protein 8B	3729	0
XM_020099033.1	major prion protein homolog	2307	2.64E-174
XM_020099034.1	lymphocyte antigen 6G-like	824	1.00E-65
XM_020099035.1	prostate stem cell antigen-like	921	3.71E-57
XM_020099036.1	F-box/LRR-repeat protein 17	2100	0
XM_020099037.1	ataxin-2-like protein	4211	0
XM_020099038.1	MAP kinase-activated protein kinase 2-like	2593	0
XM_020099039.1	lipoxygenase homology domain-containing protein 1 isoform X1	6482	0
XM_020099040.1	secretory carrier-associated membrane protein 1-like	1199	0
XM_020099041.1	DNA mismatch repair protein Msh3	3600	0
XM_020099042.1	PREDICTED: midnolin-like	2095	0
XM_020099043.1	cytoskeleton-associated protein 5-A-like	465	2.36E-110
XM_020099044.1	shematrin-like protein 2	534	9.03E-17
XM_020099045.1	39S ribosomal protein L40, mitochondrial	568	1.61E-47
XM_020099046.1	tricarboxylate transport protein, mitochondrial-like	1114	5.32E-82
XM_020099047.1	interleukin-12 subunit beta-like	1084	0
XM_020099048.1	E3 ubiquitin-protein ligase KCMF1-like	1303	3.88E-172
XM_020099049.1	bifunctional methylenetetrahydrofolate dehydrogenase/cyclohydrolase, mitochondrial-like	1327	0
XM_020099050.1	prostaglandin G/H synthase 1-like	1944	0
XM_020099051.1	sia-alpha-2,3-Gal-beta-1,4-GlcNAc-R:alpha 2,8-sialyltransferase-like	1633	0
XM_020099052.1	MAU2 chromatid cohesion factor homolog	3527	0
XM_020099053.1	long-chain-fatty-acid--CoA ligase ACSBG2-like	2794	0
XM_020099054.1	ghrelin O-acyltransferase	648	6.62E-143
XM_020099055.1	neurogenic locus notch homolog protein 1-like	1797	0
XM_020099056.1	neurogenic locus notch homolog protein 1-like	3594	0
XM_020099057.1	nucleotide-binding oligomerization domain 1 protein	2652	0
XM_020099058.1	F-box/WD repeat-containing protein 8	2222	0
XM_020099059.1	WD repeat and SOCS box-containing protein 2	1539	0
XM_020099060.1	inositol-trisphosphate 3-kinase A-like	1651	0
XM_020099061.1	E3 ubiquitin-protein ligase znrf3-like	1115	1.33E-172
XM_020099062.1	kremen protein 1-like	654	9.58E-100
XM_020099063.1	SPRY domain-containing SOCS box protein 4-like	1322	0
XM_020099064.1	collagen alpha-1(XXVII) chain B-like isoform X1	7046	0
XM_020099065.1	collagen alpha-1(XXVII) chain B-like isoform X2	7001	0
XM_020099066.1	potassium channel subfamily T member 1-like isoform X1	4964	0
XM_020099067.1	potassium channel subfamily T member 1-like isoform X2	4943	0
XM_020099068.1	potassium channel subfamily T member 1-like isoform X3	4925	0
XM_020099069.1	potassium channel subfamily T member 1-like isoform X4	4901	0
XM_020099070.1	potassium channel subfamily T member 1-like isoform X5	5038	0
XM_020099071.1	potassium channel subfamily T member 1-like isoform X6	4999	0
XM_020099072.1	potassium channel subfamily T member 1-like isoform X7	4158	0
XM_020099073.1	SPRY domain-containing SOCS box protein 4-like	1466	0
XM_020099074.1	potassium channel subfamily T member 1-like isoform X8	4136	0
XM_020099075.1	potassium channel subfamily T member 1-like isoform X9	4904	0
XM_020099076.1	potassium channel subfamily T member 1-like isoform X10	4841	0
XM_020099077.1	potassium channel subfamily T member 1-like isoform X11	4880	0
XM_020099078.1	volume-regulated anion channel subunit LRRC8A	7763	0
XM_020099079.1	volume-regulated anion channel subunit LRRC8A	7732	0
XM_020099080.1	volume-regulated anion channel subunit LRRC8A	7774	0
XM_020099081.1	volume-regulated anion channel subunit LRRC8A	7750	0
XM_020099082.1	volume-regulated anion channel subunit LRRC8A	7929	0
XM_020099083.1	SPRY domain-containing SOCS box protein 4-like	3082	0
XM_020099084.1	cell wall protein DAN4-like isoform X1	2831	0
XM_020099085.1	cell wall protein DAN4-like isoform X1	2747	0
XM_020099086.1	kelch-like protein 20 isoform X1	2295	0
XM_020099087.1	kelch-like protein 20 isoform X2	2256	0
XM_020099088.1	iron-sulfur cluster co-chaperone protein HscB, mitochondrial isoform X1	1037	0
XM_020099089.1	iron-sulfur cluster co-chaperone protein HscB, mitochondrial isoform X2	928	0
XM_020099090.1	L-xylulose reductase	735	1.52E-146
XM_020099091.1	peptidyl-prolyl cis-trans isomerase-like 3	923	2.25E-107
XM_020099092.1	sperm flagellar protein 2 isoform X1	5746	0
XM_020099093.1	sperm flagellar protein 2 isoform X2	5740	0
XM_020099094.1	sperm flagellar protein 2 isoform X3	5650	0
XM_020099095.1	sperm flagellar protein 2 isoform X4	5605	0
XM_020099096.1	sperm flagellar protein 2 isoform X4	5594	0
XM_020099097.1	brain mitochondrial carrier protein 1-like	1181	0
XM_020099098.1	sperm flagellar protein 2 isoform X4	5647	0
XM_020099099.1	leucine zipper putative tumor suppressor 3-like isoform X2	5393	0

XM_020099100.1	leucine zipper putative tumor suppressor 3-like isoform X2	5261	0
XM_020099101.1	centrosomal protein of 131 kDa-like isoform X3	5165	0
XM_020099102.1	zinc finger CCCH domain-containing protein 13-like isoform X4	4925	0
XM_020099103.1	leukemia inhibitory factor receptor-like	6639	0
XM_020099104.1	leukemia inhibitory factor receptor-like	6655	0
XM_020099105.1	zinc finger protein 366	2743	0
XM_020099106.1	histone-lysine N-methyltransferase SMYD1 isoform X1	1850	0
XM_020099107.1	histone-lysine N-methyltransferase SMYD1 isoform X2	1890	0
XM_020099108.1	prostaglandin E2 receptor EP4 subtype-like	3451	0
XM_020099109.1	28S ribosomal protein S27, mitochondrial	1259	0
XM_020099110.1	mitochondrial import inner membrane translocase subunit TIM44-like isoform X1	3198	0
XM_020099111.1	sepiapterin reductase-like	1449	0
XM_020099112.1	isotocin-neurophysin IT 1-like	776	1.40E-83
XM_020099113.1	isotocin-neurophysin IT 1-like	792	1.74E-83
XM_020099114.1	fatty acid-binding protein, liver	501	5.48E-88
XM_020099115.1	cell division control protein 45 homolog	1977	0
XM_020099116.1	ubiquitin fusion degradation protein 1 homolog	1450	0
XM_020099117.1	creatine kinase U-type, mitochondrial-like	1277	0
XM_020099118.1	mitochondrial import inner membrane translocase subunit TIM44-like isoform X2	3126	0
XM_020099119.1	major facilitator superfamily domain-containing protein 10	2329	0
XM_020099120.1	major facilitator superfamily domain-containing protein 10	2363	0
XM_020099121.1	NADH dehydrogenase [ubiquinone] iron-sulfur protein 4, mitochondrial	769	5.38E-139
XM_020099122.1	keratin, type I cytoskeletal 18-like	1877	0
XM_020099123.1	nicotinamide riboside kinase 2-like isoform X1	1407	3.62E-140
XM_020099124.1	4-hydroxybenzoate polyprenyltransferase, mitochondrial	1410	0
XM_020099125.1	fibroblast growth factor 10-like	2000	4.75E-118
XM_020099126.1	arginine vasotocin precursor	645	2.29E-97
XM_020099127.1	transcription elongation factor A protein 1 isoform X1	1571	0
XM_020099128.1	intracellular hyaluronan-binding protein 4-like	1478	0
XM_020099129.1	homeobox protein orthopedia B-like isoform X1	1870	1.29E-176
XM_020099130.1	homeobox protein orthopedia B-like isoform X2	1793	8.83E-167
XM_020099131.1	corticotropin-releasing factor-binding protein-like isoform X1	1501	0
XM_020099132.1	corticotropin-releasing factor-binding protein-like isoform X2	1492	0
XM_020099133.1	calcineurin B homologous protein 3	1351	9.36E-145
XM_020099134.1	UDP-glucuronosyltransferase 3A2 isoform X1	2201	0
XM_020099135.1	UDP-glucuronosyltransferase 3A2 isoform X1	2031	0
XM_020099136.1	transcription elongation factor A protein 1 isoform X2	1598	0
XM_020099137.1	UDP-glucuronosyltransferase 3A2 isoform X1	2100	0
XM_020099138.1	UDP-N-acetylglucosamine/UDP-glucose/GDP-mannose transporter-like isoform X1	1765	0
XM_020099139.1	UDP-N-acetylglucosamine/UDP-glucose/GDP-mannose transporter-like isoform X2	1678	0
XM_020099140.1	UDP-N-acetylglucosamine/UDP-glucose/GDP-mannose transporter-like isoform X3	1506	0
XM_020099141.1	transmembrane protein 132D-like	3899	0
XM_020099142.1	COMM domain-containing protein 10 isoform X1	1195	1.18E-139
XM_020099143.1	COMM domain-containing protein 10 isoform X2	623	5.77E-123
XM_020099144.1	transmembrane protein 17A-like	922	2.23E-147
XM_020099145.1	selenoprotein M-like	993	3.32E-87
XM_020099146.1	enkurin domain-containing protein 1	1193	0
XM_020099147.1	enkurin domain-containing protein 1	1176	0
XM_020099148.1	glucagon family neuropeptides-like isoform X1	993	2.58E-122
XM_020099149.1	enkurin domain-containing protein 1	1133	0
XM_020099150.1	heat shock protein beta-1-like	1380	0
XM_020099151.1	N-acetylated-alpha-linked acidic dipeptidase-like protein	2492	0
XM_020099152.1	G2/mitotic-specific cyclin-B3	1478	0
XM_020099153.1	leucine-rich repeat-containing protein 74B isoform X1	1615	0
XM_020099155.1	LIM/homeobox protein Lhx6-like	1069	0
XM_020099156.1	glucagon family neuropeptides-like isoform X2	886	1.26E-95
XM_020099157.1	protein Largen	3500	2.22E-128
XM_020099158.1	pro-neuregulin-4, membrane-bound isoform	1325	3.20E-84
XM_020099159.1	B-cell linker protein-like	1204	0
XM_020099160.1	PREDICTED: multicilin	1154	0
XM_020099161.1	proheparin-binding EGF-like growth factor	1079	1.52E-122
XM_020099162.1	izumo sperm-egg fusion protein 1	1278	0
XM_020099163.1	3-ketoacyl-CoA thiolase, mitochondrial	1916	0
XM_020099164.1	DNA polymerase beta	2105	0
XM_020099165.1	leucine-rich repeat-containing protein 30	2042	0
XM_020099166.1	60S ribosomal protein L6	832	8.91E-161
XM_020099167.1	UPF0545 protein C22orf39 homolog isoform X1	1111	4.85E-91
XM_020099168.1	UPF0545 protein C22orf39 homolog isoform X2	1091	8.77E-74
XM_020099169.1	dynein light chain 2, cytoplasmic-like	701	9.65E-62
XM_020099170.1	terminal uridylyltransferase 7	5826	0
XM_020099171.1	terminal uridylyltransferase 7	5886	0
XM_020099172.1	kisspeptin receptor gpr54	1794	0
XM_020099173.1	iron-sulfur cluster assembly 1 homolog, mitochondrial	1745	3.82E-76
XM_020099174.1	ATP synthase subunit alpha, mitochondrial-like	2236	0
XM_020099175.1	E3 ubiquitin-protein ligase RNF165-like isoform X1	6315	0
XM_020099176.1	E3 ubiquitin-protein ligase RNF165-like isoform X1	6254	0
XM_020099177.1	E3 ubiquitin-protein ligase RNF165-like isoform X1	6312	0
XM_020099178.1	ras-related protein Rap-1A-like	1673	2.14E-132
XM_020099179.1	E3 ubiquitin-protein ligase RNF165-like isoform X1	6303	0

XM_020099180.1	E3 ubiquitin-protein ligase RNF165-like isoform X1	3632	0
XM_020099181.1	E3 ubiquitin-protein ligase RNF165-like isoform X5	3629	0
XM_020099182.1	PREDICTED: glucokinase	2058	0
XM_020099183.1	potassium channel subfamily K member 2-like	1200	0
XM_020099184.1	glycerol-3-phosphate acyltransferase 4	1943	0
XM_020099185.1	synaptobrevin homolog YKT6	2734	1.58E-129
XM_020099186.1	myosin regulatory light chain 2, atrial isoform	451	5.36E-86
XM_020099187.1	ras-related protein Rap-1A-like	1661	1.88E-132
XM_020099188.1	mucosa-associated lymphoid tissue lymphoma translocation protein 1 isoform X1	2818	0
XM_020099189.1	mucosa-associated lymphoid tissue lymphoma translocation protein 1 isoform X2	2770	0
XM_020099190.1	selenocysteine insertion sequence-binding protein 2	2613	0
XM_020099191.1	prolactin receptor-like	4207	0
XM_020099192.1	prolactin receptor-like	4252	0
XM_020099193.1	prolactin receptor-like	4182	0
XM_020099194.1	prolactin receptor-like	4185	0
XM_020099195.1	prolactin receptor-like	4287	0
XM_020099196.1	ras-related protein Rap-1A-like	1605	1.02E-132
XM_020099197.1	prolactin receptor-like	4170	0
XM_020099198.1	prolactin receptor-like	4099	0
XM_020099199.1	72 kDa inositol polyphosphate 5-phosphatase	4761	0
XM_020099200.1	72 kDa inositol polyphosphate 5-phosphatase	4638	0
XM_020099201.1	72 kDa inositol polyphosphate 5-phosphatase	4470	0
XM_020099202.1	mitochondrial-processing peptidase subunit alpha	2376	0
XM_020099203.1	granzyme A-like	1863	0
XM_020099204.1	sodium-dependent phosphate transporter 2 isoform X1	4200	0
XM_020099205.1	sodium-dependent phosphate transporter 2 isoform X2	2847	0
XM_020099206.1	sodium-dependent phosphate transporter 2 isoform X3	2847	0
XM_020099207.1	betaine--homocysteine S-methyltransferase 1-like	1474	0
XM_020099208.1	aquaporin-4 isoform X1	2994	0
XM_020099209.1	betaine--homocysteine S-methyltransferase 1-like	1602	0
XM_020099210.1	pyridoxal phosphate phosphatase	1967	0
XM_020099211.1	28S ribosomal protein S36, mitochondrial	954	1.42E-63
XM_020099212.1	clathrin heavy chain 1-like isoform X1	6996	0
XM_020099213.1	clathrin heavy chain 1-like isoform X2	6967	0
XM_020099214.1	serine/threonine-protein phosphatase with EF-hands 2-like	2431	0
XM_020099215.1	lysosome membrane protein 2-like isoform X1	6161	0
XM_020099216.1	aquaporin-4 isoform X2	3105	0
XM_020099217.1	lysosome membrane protein 2-like isoform X2	6114	0
XM_020099218.1	hypermethylated in cancer 2 protein	3086	0
XM_020099219.1	hypermethylated in cancer 2 protein	3079	0
XM_020099220.1	phosphatidylinositol 4-kinase alpha isoform X1	7351	0
XM_020099221.1	phosphatidylinositol 4-kinase alpha isoform X2	7253	0
XM_020099222.1	heparin cofactor 2	2013	0
XM_020099223.1	dmX-like protein 1 isoform X1	13009	0
XM_020099224.1	dmX-like protein 1 isoform X2	12970	0
XM_020099225.1	aquaporin-4 isoform X2	3106	0
XM_020099226.1	dmX-like protein 1 isoform X3	12946	0
XM_020099227.1	U3 small nucleolar RNA-associated protein 15 homolog	2210	0
XM_020099228.1	rho guanine nucleotide exchange factor 28-like isoform X1	6639	0
XM_020099229.1	rho guanine nucleotide exchange factor 28-like isoform X2	6561	0
XM_020099230.1	rho guanine nucleotide exchange factor 28-like isoform X3	6480	0
XM_020099231.1	rho guanine nucleotide exchange factor 28-like isoform X4	5520	0
XM_020099232.1	rho guanine nucleotide exchange factor 28-like isoform X5	5430	0
XM_020099233.1	glycine dehydrogenase (decarboxylating), mitochondrial	4179	0
XM_020099234.1	monocarboxylate transporter 13-like	2474	0
XM_020099235.1	monocarboxylate transporter 13-like	2441	0
XM_020099236.1	V-set and immunoglobulin domain-containing protein 10 isoform X1	2312	0
XM_020099237.1	V-set and immunoglobulin domain-containing protein 10 isoform X1	2307	0
XM_020099238.1	tyrosine-protein kinase Lyn-like	2578	0
XM_020099239.1	V-set and immunoglobulin domain-containing protein 10 isoform X3	1758	0
XM_020099240.1	serine/threonine-protein kinase TAO3	2678	0
XM_020099241.1	phosphatidylethanolamine-binding protein 1	1151	2.92E-137
XM_020099242.1	probable ATP-dependent RNA helicase DHX37	3635	0
XM_020099243.1	probable ATP-dependent RNA helicase DHX37	3778	0
XM_020099244.1	septin-5 isoform X1	1508	0
XM_020099245.1	septin-5 isoform X2	1276	0
XM_020099246.1	tyrosine-protein kinase Lyn-like	2575	0
XM_020099247.1	platelet glycoprotein Ib beta chain	1478	1.97E-99
XM_020099248.1	zinc finger protein 462	8173	0
XM_020099249.1	inhibitor of nuclear factor kappa-B kinase subunit alpha-like	3776	0
XM_020099250.1	inhibitor of nuclear factor kappa-B kinase subunit alpha-like	3771	0
XM_020099251.1	voltage-dependent anion-selective channel protein 2-like isoform X1	1846	0
XM_020099252.1	voltage-dependent anion-selective channel protein 2-like isoform X2	1884	0
XM_020099253.1	voltage-dependent anion-selective channel protein 2-like isoform X3	1816	0
XM_020099254.1	voltage-dependent anion-selective channel protein 2-like isoform X4	1812	0
XM_020099255.1	voltage-dependent anion-selective channel protein 2-like isoform X3	1776	0
XM_020099256.1	voltage-dependent anion-selective channel protein 2-like isoform X4	1763	0
XM_020099257.1	RNA exonuclease 1 homolog isoform X1	4554	0
XM_020099258.1	RNA exonuclease 1 homolog isoform X1	4517	0

XM_020099259.1	RNA exonuclease 1 homolog isoform X1	4368	0
XM_020099260.1	FYVE and coiled-coil domain-containing protein 1-like	7157	0
XM_020099261.1	RNA exonuclease 1 homolog isoform X1	4565	0
XM_020099262.1	UDP-glucuronosyltransferase 2A2-like	2033	0
XM_020099263.1	chondroitin sulfate proteoglycan 4-like	8975	0
XM_020099264.1	putative aminopeptidase W07G4.4	2016	0
XM_020099265.1	fumarylacetoacetate hydrolase domain-containing protein 2-like isoform X4	1143	0
XM_020099266.1	fumarylacetoacetate hydrolase domain-containing protein 2-like isoform X4	1217	0
XM_020099267.1	fumarylacetoacetate hydrolase domain-containing protein 2-like isoform X4	1104	0
XM_020099268.1	fumarylacetoacetate hydrolase domain-containing protein 2-like isoform X4	1103	0
XM_020099269.1	FYVE and coiled-coil domain-containing protein 1-like	7021	0
XM_020099270.1	fumarylacetoacetate hydrolase domain-containing protein 2-like isoform X4	1118	0
XM_020099271.1	fumarylacetoacetate hydrolase domain-containing protein 2-like isoform X4	1151	0
XM_020099272.1	fumarylacetoacetate hydrolase domain-containing protein 2-like isoform X4	1133	0
XM_020099273.1	fumarylacetoacetate hydrolase domain-containing protein 2-like isoform X4	1175	0
XM_020099274.1	fumarylacetoacetate hydrolase domain-containing protein 2-like isoform X4	1045	0
XM_020099275.1	RING finger and transmembrane domain-containing protein 2	3362	0
XM_020099276.1	UPF0454 protein C12orf49 homolog	1210	1.36E-133
XM_020099277.1	calphotin-like isoform X3	2629	4.80E-113
XM_020099278.1	calphotin-like isoform X3	2593	1.70E-113
XM_020099279.1	calphotin-like isoform X3	2575	3.16E-113
XM_020099280.1	dual specificity protein phosphatase CDC14A-like isoform X1	2414	0
XM_020099281.1	dual specificity protein phosphatase CDC14A-like isoform X2	2336	0
XM_020099282.1	dual specificity protein phosphatase CDC14A-like isoform X3	2118	0
XM_020099283.1	aspartate--tRNA ligase, mitochondrial	3201	0
XM_020099284.1	thioredoxin-like protein AAED1	1565	8.54E-168
XM_020099285.1	oocyte zinc finger protein XlCOF6.1-like isoform X1	2517	0
XM_020099286.1	zinc finger protein 260-like isoform X1	1058	9.83E-107
XM_020099287.1	28S ribosomal protein S30, mitochondrial	1802	0
XM_020099288.1	leucine-rich repeats and immunoglobulin-like domains protein 1	2397	0
XM_020099289.1	histidine triad nucleotide-binding protein 2, mitochondrial-like isoform X1	908	1.28E-143
XM_020099290.1	histidine triad nucleotide-binding protein 2, mitochondrial-like isoform X2	782	5.91E-119
XM_020099291.1	uncharacterized family 31 glucosidase KIAA1161-like	4962	0
XM_020099292.1	uncharacterized family 31 glucosidase KIAA1161-like	2591	0
XM_020099293.1	solute carrier family 23 member 2 isoform X1	6603	0
XM_020099294.1	solute carrier family 23 member 2 isoform X1	6552	0
XM_020099295.1	armadillo repeat-containing protein 6	2078	0
XM_020099296.1	threonine--tRNA ligase, cytoplasmic	3564	0
XM_020099297.1	tyrosine-protein kinase JAK2-like	4239	0
XM_020099298.1	tyrosine-protein kinase JAK2-like	4682	0
XM_020099299.1	phospholipid phosphatase 5-like	4348	0
XM_020099300.1	phospholipid phosphatase 5-like	4344	0
XM_020099301.1	septin-2B isoform X2	2405	0
XM_020099302.1	septin-2B isoform X2	2374	0
XM_020099303.1	coiled-coil domain-containing protein 158 isoform X1	3226	0
XM_020099304.1	coiled-coil domain-containing protein 158 isoform X1	3287	0
XM_020099305.1	coiled-coil domain-containing protein 158 isoform X1	3208	0
XM_020099306.1	coiled-coil domain-containing protein 158 isoform X1	3199	0
XM_020099307.1	SURP and G-patch domain-containing protein 1	2051	0
XM_020099308.1	coiled-coil domain-containing protein 158 isoform X1	2736	0
XM_020099309.1	PREDICTED: cyclin-G2-like	1825	0
XM_020099310.1	histone-lysine N-methyltransferase, H3 lysine-36 and H4 lysine-20 specific-like	3471	0
XM_020099311.1	E3 ubiquitin-protein ligase KCMF1-like	2192	5.83E-69

XM_020099312.1	protein FAM214B	3870	0
XM_020099313.1	protein FAM214B	3910	0
XM_020099314.1	protein FAM214B	4054	0
XM_020099315.1	growth arrest-specific protein 1-like	2538	1.38E-157
XM_020099316.1	replication factor C subunit 5	1651	0
XM_020099317.1	PREDICTED: claudin-5	1625	1.56E-141
XM_020099318.1	coiled-coil domain-containing protein 18	5482	0
XM_020099319.1	peroxisomal multifunctional enzyme type 2	2913	0
XM_020099320.1	nitric oxide synthase, brain	7850	0
XM_020099321.1	transducin-like enhancer protein 4 isoform X1	6213	0
XM_020099322.1	transducin-like enhancer protein 4 isoform X2	6210	0
XM_020099323.1	transducin-like enhancer protein 4 isoform X3	6201	0
XM_020099324.1	heat shock protein beta-1	2509	2.87E-126
XM_020099325.1	heat shock protein beta-1	2354	3.65E-125
XM_020099326.1	endothelial lipase	2429	0
XM_020099327.1	DNA polymerase kappa	2504	0
XM_020099328.1	ras-related protein Rab-27B-like	1189	8.84E-162
XM_020099329.1	A disintegrin and metalloproteinase with thrombospondin motifs 6	6265	0
XM_020099330.1	60 kDa SS-A/Ro ribonucleoprotein	3458	0
XM_020099331.1	cyclin-G-associated kinase isoform X1	4810	0
XM_020099332.1	cyclin-G-associated kinase isoform X2	4560	0
XM_020099333.1	radixin isoform X2	3553	0
XM_020099334.1	radixin isoform X2	3449	0
XM_020099335.1	endoplasmic reticulum aminopeptidase 1-like isoform X2	4560	0
XM_020099336.1	endoplasmic reticulum aminopeptidase 1-like isoform X2	4891	0
XM_020099337.1	endoplasmic reticulum aminopeptidase 1-like isoform X2	4576	0

XM_020099338.1	zinc finger protein 143	3655	0
XM_020099339.1	semaphorin-6D isoform X4	6444	0
XM_020099340.1	sin3 histone deacetylase corepressor complex component SDS3 isoform X1	1886	0
XM_020099341.1	sin3 histone deacetylase corepressor complex component SDS3 isoform X2	1871	0
XM_020099342.1	sin3 histone deacetylase corepressor complex component SDS3 isoform X3	1934	0
XM_020099343.1	dehydrogenase/reductase SDR family member 1	2033	0
XM_020099344.1	sin3 histone deacetylase corepressor complex component SDS3 isoform X1	1837	0
XM_020099345.1	sin3 histone deacetylase corepressor complex component SDS3 isoform X5	1812	0
XM_020099346.1	chondroitin sulfate N-acetylgalactosaminyltransferase 2	1880	0
XM_020099347.1	gamma-glutamyl hydrolase-like	1410	0
XM_020099348.1	RING finger protein 122 isoform X1	2323	8.33E-106
XM_020099349.1	RING finger protein 122 isoform X2	2320	4.16E-105
XM_020099350.1	proliferating cell nuclear antigen	1448	0
XM_020099351.1	osteoclast-stimulating factor 1	1661	2.28E-152
XM_020099352.1	polyadenylate-binding protein-interacting protein 1	2573	0
XM_020099353.1	follistatin isoform X1	2419	0
XM_020099354.1	follistatin isoform X2	2411	0
XM_020099355.1	follistatin isoform X1	2507	0
XM_020099356.1	mucolipin-3-like isoform X1	1907	0
XM_020099357.1	PREDICTED: sesquipedalian-1	3473	1.16E-170
XM_020099358.1	PREDICTED: sesquipedalian-1	3375	5.26E-171
XM_020099359.1	PREDICTED: sesquipedalian-1	3259	1.96E-171
XM_020099360.1	acetoacetyl-CoA synthetase	4861	0
XM_020099361.1	AP-3 complex subunit sigma-2	1048	4.56E-141
XM_020099362.1	lipoma HMGIC fusion partner-like 2 protein	1662	2.62E-152
XM_020099363.1	lipoma HMGIC fusion partner-like 2 protein	2269	1.50E-149
XM_020099364.1	phosphoserine aminotransferase	1255	0
XM_020099365.1	ras association domain-containing protein 6-like	3028	0
XM_020099366.1	PREDICTED: uncharacterized protein C9orf84 homolog	5077	0
XM_020099367.1	7-alpha-hydroxycholest-4-en-3-one 12-alpha-hydroxylase-like	2120	0
XM_020099368.1	T-cell acute lymphocytic leukemia protein 2-like	1460	2.26E-75
XM_020099369.1	scavenger receptor class F member 2	5778	0
XM_020099370.1	histone-lysine N-methyltransferase NSD3 isoform X1	5752	0
XM_020099371.1	histone-lysine N-methyltransferase NSD3 isoform X2	5881	0
XM_020099372.1	histone-lysine N-methyltransferase NSD3 isoform X3	5770	0
XM_020099373.1	histone-lysine N-methyltransferase NSD3 isoform X4	5746	0
XM_020099374.1	histone-lysine N-methyltransferase NSD3 isoform X5	5734	0
XM_020099375.1	cell division cycle protein 20 homolog B isoform X1	2814	0
XM_020099376.1	cell division cycle protein 20 homolog B isoform X2	2808	0
XM_020099377.1	cell division cycle protein 20 homolog B isoform X3	2762	0
XM_020099378.1	probable glutathione peroxidase 8	1186	9.84E-153
XM_020099379.1	ADP-ribosylation factor-like protein 15 isoform X1	1321	1.16E-152
XM_020099380.1	ADP-ribosylation factor-like protein 15 isoform X2	2442	1.02E-145
XM_020099381.1	matrix metalloproteinase-16-like isoform X1	5750	0
XM_020099382.1	E3 ubiquitin-protein ligase RNF128	2373	0
XM_020099383.1	PREDICTED: putative protein TPRXL	1613	1.17E-131
XM_020099384.1	methylthioribulose-1-phosphate dehydratase	1248	2.97E-164
XM_020099385.1	gamma-butyrobetaine dioxygenase	2410	0
XM_020099386.1	gamma-butyrobetaine dioxygenase	1757	0
XM_020099387.1	nicotinamide riboside kinase 2-like isoform X1	1160	4.90E-153
XM_020099388.1	matrix metalloproteinase-16-like isoform X2	5735	0
XM_020099389.1	nicotinamide riboside kinase 2-like isoform X1	1111	3.32E-108
XM_020099390.1	potassium voltage-gated channel subfamily A member 3-like	2091	0
XM_020099391.1	gonadotropin subunit beta-1-like isoform X1	689	2.44E-84
XM_020099392.1	gonadotropin subunit beta-1-like isoform X1	640	1.75E-74
XM_020099393.1	carbohydrate sulfotransferase 1-like	1791	0

XM_020099394.1	leucine-rich repeat and immunoglobulin-like domain-containing nogo receptor-interacting protein 2	3662	0
XM_020099395.1	leucine zipper protein 2	2087	0
XM_020099396.1	mortality factor 4-like protein 1 isoform X1	1381	0
XM_020099397.1	mortality factor 4-like protein 1 isoform X2	1267	0
XM_020099398.1	oxidoreductase HTATIP2 isoform X1	1744	0
XM_020099399.1	excitatory amino acid transporter 1-like	1817	0
XM_020099400.1	oxidoreductase HTATIP2 isoform X1	1492	1.08E-174
XM_020099401.1	ceroid-lipofuscinosis neuronal protein 6 isoform X1	1760	0
XM_020099402.1	ceroid-lipofuscinosis neuronal protein 6 isoform X2	1865	0
XM_020099403.1	proteasome subunit alpha type-4	971	3.04E-175
XM_020099404.1	proteasome subunit alpha type-4	1153	3.08E-174
XM_020099405.1	proteasome subunit alpha type-4	903	1.23E-175
XM_020099406.1	proteasome subunit alpha type-4	902	1.23E-175
XM_020099407.1	Bardet-Biedl syndrome 4 protein isoform X1	2432	0
XM_020099408.1	Bardet-Biedl syndrome 4 protein isoform X2	2408	0
XM_020099409.1	sentrin-specific protease 8	1484	2.07E-146
XM_020099410.1	sentrin-specific protease 8	1488	1.74E-146
XM_020099411.1	protein arginine N-methyltransferase 3	1796	0
XM_020099412.1	bone morphogenetic protein 2-like	1478	0
XM_020099413.1	neuronal acetylcholine receptor subunit alpha-7-like	2238	0
XM_020099414.1	GRAM domain-containing protein 2 isoform X2	3049	0
XM_020099415.1	GRAM domain-containing protein 2 isoform X2	2705	0
XM_020099416.1	GRAM domain-containing protein 2 isoform X2	2584	0

XM_020099417.1	GRAM domain-containing protein 2 isoform X2	2695	0
XM_020099418.1	PREDICTED: mucin-15	2270	0
XM_020099419.1	integrin alpha-11 isoform X1	5324	0
XM_020099420.1	integrin alpha-11 isoform X2	5178	0
XM_020099421.1	suppressor of cytokine signaling 5	2439	0
XM_020099422.1	sodium/potassium/calcium exchanger 5	1625	0
XM_020099423.1	EGF-like repeat and discoidin I-like domain-containing protein 3	2802	0
XM_020099424.1	protein fuzzy homolog	2963	0
XM_020099425.1	relaxin-3 receptor 1-like	1051	0
XM_020099426.1	transducin-like enhancer protein 3	2917	0
XM_020099427.1	proteoglycan 4-like	3169	0
XM_020099428.1	cytoplasmic polyadenylation element-binding protein 1	645	4.38E-163
XM_020099429.1	unconventional myosin-IXa-like	9473	0
XM_020099430.1	WD repeat and HMG-box DNA-binding protein 1-like	1036	0
XM_020099431.1	C-myc promoter-binding protein-like	3314	0
XM_020099432.1	olfactory receptor 10G4-like	932	0
XM_020099433.1	neuron navigator 2-like	1016	1.54E-165
XM_020099434.1	sodium- and chloride-dependent glycine transporter 2	2516	0
XM_020099435.1	N-acetyllactosaminide alpha-1,3-galactosyltransferase-like isoform X2	1232	0
XM_020099436.1	WD repeat-containing protein 47	3099	0
XM_020099437.1	guanylyl cyclase-activating protein 2-like	606	3.96E-138
XM_020099438.1	EF-hand domain-containing protein 1-like	1398	0
XM_020099439.1	very-long-chain 3-oxoacyl-CoA reductase-B-like	1767	0
XM_020099440.1	potassium voltage-gated channel subfamily KQT member 1-like isoform X1	2844	0
XM_020099441.1	PREDICTED: tetraspanin-4-like	1990	8.73E-88
XM_020099442.1	transmembrane protein 208	1054	8.90E-68
XM_020099443.1	ERO1-like protein alpha	2163	0
XM_020099444.1	WD repeat-containing protein 47	3043	0
XM_020099445.1	metallophosphoesterase MPPED2	1120	4.18E-128
XM_020099446.1	metallophosphoesterase MPPED2	767	4.96E-130
XM_020099447.1	metallophosphoesterase MPPED2	787	6.52E-130
XM_020099448.1	40S ribosomal protein S17-like	527	4.90E-96
XM_020099449.1	excitatory amino acid transporter 2 isoform X2	1641	0
XM_020099450.1	cyclin-dependent kinase inhibitor 1B	1082	7.79E-97
XM_020099451.1	transient receptor potential cation channel subfamily M member 5	4146	0
XM_020099452.1	WD repeat-containing protein 47	3035	0
XM_020099453.1	SKI family transcriptional corepressor 1 homolog-B-like isoform X1	2423	0
XM_020099454.1	GTP cyclohydrolase 1-like	1001	0
XM_020099455.1	membrane progestin receptor gamma-B-like	1465	0
XM_020099456.1	inactive serine protease PAMR1 isoform X2	2703	0
XM_020099457.1	guanylyl cyclase-activating protein 2-like	585	3.79E-146
XM_020099458.1	relaxin-3 receptor 1-like	1243	0
XM_020099459.1	cortexin-1-like	1054	6.96E-40
XM_020099460.1	PREDICTED: uncharacterized protein LOC109637239 isoform X1	3254	0
XM_020099461.1	selenocysteine insertion sequence-binding protein 2-like isoform X1	6344	0
XM_020099462.1	selenocysteine insertion sequence-binding protein 2-like isoform X2	6343	0
XM_020099463.1	selenocysteine insertion sequence-binding protein 2-like isoform X3	6319	0
XM_020099464.1	selenocysteine insertion sequence-binding protein 2-like isoform X4	6320	0
XM_020099465.1	selenocysteine insertion sequence-binding protein 2-like isoform X1	6138	0
XM_020099466.1	SHC-transforming protein 4	2471	0
XM_020099467.1	SHC-transforming protein 4	2504	0
XM_020099468.1	PREDICTED: uncharacterized protein LOC109637239 isoform X2	3252	0
XM_020099469.1	COP9 signalosome complex subunit 2 isoform X1	1932	0
XM_020099470.1	COP9 signalosome complex subunit 2 isoform X2	1919	0
XM_020099471.1	NAD(P) transhydrogenase, mitochondrial-like	2757	0
XM_020099472.1	spermatogenesis-associated protein 5-like protein 1	2467	0
XM_020099473.1	DTW domain-containing protein 1	1243	0
XM_020099474.1	fibroblast growth factor 7	1969	3.32E-148
XM_020099475.1	normal mucosa of esophagus-specific gene 1 protein	399	1.10E-44
XM_020099476.1	PREDICTED: uncharacterized protein LOC109637239 isoform X3	3287	0
XM_020099477.1	zinc finger protein 609-like	8063	0
XM_020099478.1	latent-transforming growth factor beta-binding protein 2	10472	0
XM_020099479.1	deoxyuridine 5'-triphosphate nucleotidohydrolase, mitochondrial isoform X1	757	1.23E-136
XM_020099480.1	deoxyuridine 5'-triphosphate nucleotidohydrolase, mitochondrial isoform X2	878	4.06E-122
XM_020099481.1	deoxyuridine 5'-triphosphate nucleotidohydrolase, mitochondrial isoform X3	590	1.06E-121
XM_020099482.1	MAP kinase-activating death domain protein isoform X1	5747	0
XM_020099483.1	MAP kinase-activating death domain protein isoform X2	5744	0
XM_020099484.1	MAP kinase-activating death domain protein isoform X3	5684	0
XM_020099485.1	MAP kinase-activating death domain protein isoform X4	5682	0
XM_020099486.1	MAP kinase-activating death domain protein isoform X5	5542	0
XM_020099487.1	MAP kinase-activating death domain protein isoform X6	5489	0
XM_020099488.1	oxysterols receptor LXR-alpha isoform X1	4062	0
XM_020099489.1	BMP/retinoic acid-inducible neural-specific protein 3-like	4810	0
XM_020099490.1	oxysterols receptor LXR-alpha isoform X1	3924	0
XM_020099491.1	oxysterols receptor LXR-alpha isoform X1	3924	0
XM_020099492.1	oxysterols receptor LXR-alpha isoform X1	4047	0
XM_020099493.1	ARL14 effector protein	1136	2.83E-147
XM_020099494.1	ARL14 effector protein	1113	2.10E-147
XM_020099495.1	tetratricopeptide repeat protein 17 isoform X1	4560	0

XM_020099496.1	tetratricopeptide repeat protein 17 isoform X2	4552	0
XM_020099497.1	TNF receptor-associated factor 6	3226	0
XM_020099498.1	TNF receptor-associated factor 6	3238	0
XM_020099499.1	protein Smaug homolog 1 isoform X1	5413	0
XM_020099500.1	protein Smaug homolog 1 isoform X2	5125	0
XM_020099501.1	ankycorbin isoform X1	4485	0
XM_020099502.1	Krueppel-like factor 2	2068	0
XM_020099503.1	neogenin isoform X1	4357	0
XM_020099504.1	neogenin isoform X2	7556	0
XM_020099505.1	neogenin isoform X3	4300	0
XM_020099506.1	cilia- and flagella-associated protein 70 isoform X1	3494	0
XM_020099507.1	cilia- and flagella-associated protein 70 isoform X2	3440	0
XM_020099508.1	CD151 antigen-like	2266	4.65E-163
XM_020099509.1	transmembrane protein 138	480	8.50E-70
XM_020099510.1	semaphorin-6D isoform X1	3578	0
XM_020099511.1	ecto-NOX disulfide-thiol exchanger 2 isoform X1	2873	0
XM_020099512.1	semaphorin-6D isoform X1	3611	0
XM_020099513.1	semaphorin-6D isoform X1	5999	0
XM_020099514.1	semaphorin-6D isoform X1	3520	0
XM_020099515.1	semaphorin-6D isoform X3	3394	0
XM_020099516.1	semaphorin-6D isoform X1	3335	0
XM_020099517.1	complement component C8 alpha chain	2006	0
XM_020099518.1	zinc transporter 2-like	2377	0
XM_020099519.1	biogenesis of lysosome-related organelles complex 1 subunit 6	1982	5.23E-108
XM_020099520.1	biogenesis of lysosome-related organelles complex 1 subunit 6	1129	7.63E-112
XM_020099521.1	cleavage and polyadenylation specificity factor subunit 1	4149	0
XM_020099522.1	monoacylglycerol lipase ABHD2-A	3961	0
XM_020099523.1	monoacylglycerol lipase ABHD2-A	3066	0
XM_020099524.1	A disintegrin and metalloproteinase with thrombospondin motifs 6	7623	0
XM_020099525.1	calmodulin-like protein 4	2079	7.01E-105
XM_020099526.1	D-glucuronyl C5-epimerase	6183	0
XM_020099527.1	acidic leucine-rich nuclear phosphoprotein 32 family member A	1540	4.64E-102
XM_020099528.1	complement component C8 beta chain	2018	0
XM_020099529.1	solute carrier family 12 member 1	4323	0
XM_020099530.1	mothers against decapentaplegic homolog 3	4482	0
XM_020099531.1	casein kinase I isoform X1	3492	0
XM_020099532.1	casein kinase I isoform X2	3468	0
XM_020099533.1	casein kinase I isoform X3	3381	0
XM_020099534.1	casein kinase I isoform X4	3357	0
XM_020099535.1	leucine-rich repeat-containing protein 4C-like	2225	0
XM_020099536.1	GDP-fucose transporter 1 isoform X1	2429	0
XM_020099537.1	GDP-fucose transporter 1 isoform X1	2285	0
XM_020099538.1	fin bud initiation factor-like	4611	1.67E-139
XM_020099539.1	protein kinase C-binding protein NELL1 isoform X1	2481	0
XM_020099540.1	protein ABHD4	2004	0
XM_020099541.1	protein kinase C-binding protein NELL1 isoform X2	4350	0
XM_020099542.1	protein kinase C-binding protein NELL1 isoform X3	2358	0
XM_020099543.1	piggyBac transposable element-derived protein 4-like	3892	0
XM_020099544.1	kinesin-like protein KIF23 isoform X1	3338	0
XM_020099545.1	kinesin-like protein KIF23 isoform X2	3104	0
XM_020099546.1	kinesin-like protein KIF23 isoform X3	3038	0
XM_020099547.1	kinesin-like protein KIF23 isoform X4	2804	0
XM_020099548.1	myelin expression factor 2 isoform X1	2056	0
XM_020099549.1	myelin expression factor 2 isoform X2	1997	0
XM_020099550.1	myelin expression factor 2 isoform X3	1959	0
XM_020099551.1	PREDICTED: transaldolase	1550	0
XM_020099552.1	dual specificity mitogen-activated protein kinase kinase 5	5465	0
XM_020099553.1	netrin receptor UNC5A	2406	0
XM_020099554.1	transmembrane protein 68	3436	0
XM_020099555.1	solute carrier family 22 member 18	1955	0
XM_020099556.1	very-long-chain (3R)-3-hydroxyacyl-CoA dehydratase-like	1761	0
XM_020099557.1	otogelin-like isoform X1	6362	0
XM_020099558.1	otogelin-like isoform X2	6290	0
XM_020099559.1	retinaldehyde-binding protein 1	1559	0
XM_020099560.1	zinc transporter ZIP1	2338	0
XM_020099561.1	RNA-binding protein with multiple splicing-like	2241	1.15E-153
XM_020099562.1	RNA-binding protein with multiple splicing-like	2225	9.03E-150
XM_020099563.1	transmembrane protein 68	3433	0
XM_020099564.1	secretory carrier-associated membrane protein 2-like isoform X1	2616	1.51E-173
XM_020099565.1	secretory carrier-associated membrane protein 2-like isoform X2	2581	1.82E-164
XM_020099566.1	C-Jun-amino-terminal kinase-interacting protein 1 isoform X1	4614	0
XM_020099567.1	C-Jun-amino-terminal kinase-interacting protein 1 isoform X2	4603	0
XM_020099568.1	C-Jun-amino-terminal kinase-interacting protein 1-like isoform X1	768	1.15E-84
XM_020099569.1	C-Jun-amino-terminal kinase-interacting protein 1-like isoform X2	659	9.78E-75
XM_020099570.1	E3 SUMO-protein ligase PIAS1-like isoform X1	6395	0
XM_020099571.1	E3 SUMO-protein ligase PIAS1-like isoform X2	6380	0
XM_020099572.1	coronin-2B isoform X1	3324	0
XM_020099573.1	coronin-2B isoform X1	3135	0
XM_020099574.1	coronin-2B isoform X1	3115	0

XM_020099575.1	coronin-2B isoform X1	3187	0
XM_020099576.1	transcription termination factor 4, mitochondrial-like	1199	0
XM_020099577.1	activating signal cointegrator 1	3359	0
XM_020099578.1	sorting nexin-1	2716	0
XM_020099579.1	insulin-induced gene 2 protein	1564	6.17E-155
XM_020099580.1	poly(rC)-binding protein 3 isoform X1	2232	0
XM_020099581.1	poly(rC)-binding protein 3 isoform X2	2229	0
XM_020099582.1	poly(rC)-binding protein 3 isoform X3	3734	0
XM_020099583.1	poly(rC)-binding protein 3 isoform X4	3731	0
XM_020099584.1	1-phosphatidylinositol 4,5-bisphosphate phosphodiesterase delta-4	3382	0
XM_020099585.1	1-phosphatidylinositol 4,5-bisphosphate phosphodiesterase delta-4	3379	0
XM_020099586.1	E3 ubiquitin-protein ligase HECW2	8014	0
XM_020099587.1	probable G-protein coupled receptor 148	1743	0
XM_020099588.1	UPF0538 protein C2orf76 homolog	824	5.76E-89
XM_020099589.1	inhibin beta B chain-like	3328	0
XM_020099590.1	transcription factor CP2-like protein 1 isoform X3	2182	0
XM_020099591.1	transcription factor CP2-like protein 1 isoform X3	2079	0
XM_020099592.1	transcription factor CP2-like protein 1 isoform X3	2163	0
XM_020099593.1	transcription factor CP2-like protein 1 isoform X3	2060	0
XM_020099594.1	inactive dipeptidyl peptidase 10 isoform X2	3753	0
XM_020099595.1	inactive dipeptidyl peptidase 10 isoform X2	4079	0
XM_020099596.1	inactive dipeptidyl peptidase 10 isoform X2	4039	0
XM_020099597.1	calcitonin gene-related peptide type 1 receptor	3335	0
XM_020099598.1	calcitonin gene-related peptide type 1 receptor	3304	0
XM_020099599.1	ras guanine nucleotide exchange factor F-like	2424	0
XM_020099600.1	uroporphyrinogen decarboxylase	1375	0
XM_020099601.1	ras guanine nucleotide exchange factor F-like	2689	0
XM_020099602.1	zinc finger protein 142	5553	0
XM_020099603.1	G-protein coupled receptor 39	2621	0
XM_020099604.1	ly6/PLAUR domain-containing protein 1	1405	3.09E-105
XM_020099605.1	interferon alpha/beta receptor 2-like	1614	0
XM_020099606.1	calcium/calmodulin-dependent 3',5'-cyclic nucleotide phosphodiesterase 1C-like	1017	0
XM_020099607.1	coiled-coil domain-containing protein 93	2657	0
XM_020099608.1	fibroblast growth factor 4A-like	582	1.42E-128
XM_020099609.1	glycine receptor subunit alpha-3-like	1469	0
XM_020099610.1	methylmalonic aciduria and homocystinuria type D homolog, mitochondrial-like	784	3.29E-140
XM_020099611.1	myosin light chain kinase 2, skeletal/cardiac muscle-like isoform X1	3996	0
XM_020099612.1	sodium/bile acid cotransporter	1576	0
XM_020099613.1	nck-associated protein 5-like	4619	0
XM_020099614.1	golgin subfamily A member 4-like	1090	0
XM_020099615.1	ras-related protein Rab-5C	692	2.04E-109
XM_020099616.1	serine/threonine-protein kinase pim-2-like	1535	0
XM_020099617.1	collagen alpha-3(VI) chain-like	4864	0
XM_020099618.1	myosin light chain kinase 2, skeletal/cardiac muscle-like isoform X2	3858	0
XM_020099619.1	collagen alpha-3(VI) chain-like	930	4.21E-146
XM_020099620.1	collagen alpha-3(VI) chain	6529	0
XM_020099621.1	inactive dipeptidyl peptidase 10 isoform X2	2439	0
XM_020099622.1	disintegrin and metalloproteinase domain-containing protein 8-like	465	1.51E-67
XM_020099623.1	actin-related protein 3-like	2604	0
XM_020099624.1	MKI67 FHA domain-interacting nucleolar phosphoprotein	1450	2.43E-141
XM_020099625.1	WD repeat-containing protein 75	3027	0
XM_020099626.1	ubiquitin carboxyl-terminal hydrolase 37-like	2679	0
XM_020099627.1	sterol 26-hydroxylase, mitochondrial-like	1563	0
XM_020099628.1	caseinolytic peptidase B protein homolog	1694	6.24E-64
XM_020099629.1	cadherin-8-like isoform X1	6222	0
XM_020099630.1	mitochondrial chaperone BCS1	1553	0
XM_020099631.1	mitochondrial chaperone BCS1	1549	0
XM_020099632.1	tRNA (guanine-N(7)-)-methyltransferase non-catalytic subunit WDR4	1524	0
XM_020099633.1	growth/differentiation factor 8	2462	0
XM_020099634.1	GTP-binding protein 8	1172	0
XM_020099635.1	GTP-binding protein 8	1128	0
XM_020099636.1	GTP-binding protein 8	1206	0
XM_020099637.1	formimidoyltransferase-cyclodeaminase-like	1220	0
XM_020099638.1	formimidoyltransferase-cyclodeaminase-like	1166	0
XM_020099639.1	formimidoyltransferase-cyclodeaminase-like	1217	0
XM_020099640.1	formimidoyltransferase-cyclodeaminase-like	1254	0
XM_020099641.1	formimidoyltransferase-cyclodeaminase-like	1152	0
XM_020099642.1	TIR domain-containing adapter molecule 1-like	4019	0
XM_020099643.1	formimidoyltransferase-cyclodeaminase-like	1183	0
XM_020099644.1	insulin-like growth factor-binding protein 2-B	1051	0
XM_020099645.1	interferon-induced 35 kDa protein	1406	0
XM_020099646.1	interleukin-20 receptor subunit beta-like	574	1.07E-83
XM_020099647.1	interleukin-10 receptor subunit beta-like	659	6.53E-59
XM_020099648.1	melanophilin-like isoform X1	2021	0
XM_020099649.1	melanophilin-like isoform X2	2018	0
XM_020099650.1	melanophilin-like isoform X3	1979	0
XM_020099651.1	melanophilin-like isoform X4	1931	0
XM_020099652.1	ly6/PLAUR domain-containing protein 6-like	828	2.43E-114
XM_020099653.1	caspase 10	2442	0

XM_020099654.1	leucine-rich repeat-containing protein 3-like	1539	0
XM_020099655.1	high affinity cGMP-specific 3',5'-cyclic phosphodiesterase 9A-like	1700	0
XM_020099656.1	homeobox protein engrailed-1-B-like	915	2.86E-161
XM_020099657.1	receptor activity-modifying protein 1-like	605	3.82E-110
XM_020099658.1	germinal-center associated nuclear protein isoform X1	7492	0
XM_020099659.1	caspase 10	2424	0
XM_020099660.1	germinal-center associated nuclear protein isoform X1	7434	0
XM_020099661.1	germinal-center associated nuclear protein isoform X1	7480	0
XM_020099662.1	cell differentiation protein RCD1 homolog isoform X1	2118	0
XM_020099663.1	cell differentiation protein RCD1 homolog isoform X2	2089	0
XM_020099664.1	putative ribonuclease	1207	1.31E-120
XM_020099665.1	signal transducer and activator of transcription 1-alpha/beta-like	3027	0
XM_020099666.1	signal transducer and activator of transcription 4 isoform X1	2800	0
XM_020099667.1	signal transducer and activator of transcription 4 isoform X2	2797	0
XM_020099668.1	glutaminase kidney isoform, mitochondrial isoform X1	2522	0
XM_020099669.1	glutaminase kidney isoform, mitochondrial isoform X2	2666	0
XM_020099670.1	solute carrier family 40 member 1	4307	0
XM_020099671.1	catenin alpha-1-like	4295	0
XM_020099672.1	rab effector MyRIP-like isoform X2	4165	0
XM_020099673.1	titin-like isoform X3	6014	0
XM_020099674.1	titin-like isoform X3	5975	0
XM_020099675.1	titin-like isoform X3	5972	0
XM_020099676.1	titin-like isoform X3	5963	0
XM_020099677.1	titin-like isoform X3	5881	0
XM_020099678.1	leucine-rich repeat flightless-interacting protein 1-like isoform X6	5981	0
XM_020099679.1	titin-like isoform X3	5597	0
XM_020099680.1	leucine-rich repeat flightless-interacting protein 1-like isoform X8	5434	0
XM_020099681.1	leucine-rich repeat flightless-interacting protein 1-like isoform X9	5397	0
XM_020099682.1	rab effector MyRIP-like isoform X2	4252	0
XM_020099683.1	leucine-rich repeat flightless-interacting protein 1-like isoform X10	5262	0
XM_020099684.1	leucine-rich repeat flightless-interacting protein 1-like isoform X11	5213	0
XM_020099685.1	leucine-rich repeat flightless-interacting protein 2-like isoform X12	3729	0
XM_020099686.1	leucine-rich repeat flightless-interacting protein 2-like isoform X13	3683	0
XM_020099687.1	homeobox protein PKNOX1 isoform X1	3280	0
XM_020099688.1	homeobox protein PKNOX1 isoform X2	3247	0
XM_020099689.1	homeobox protein PKNOX1 isoform X3	3117	0
XM_020099690.1	NADH dehydrogenase [ubiquinone] flavoprotein 3, mitochondrial isoform X1	1752	0
XM_020099691.1	NADH dehydrogenase [ubiquinone] flavoprotein 3, mitochondrial isoform X2	1749	0
XM_020099692.1	NADH dehydrogenase [ubiquinone] flavoprotein 3, mitochondrial isoform X3	1746	0
XM_020099693.1	NADH dehydrogenase [ubiquinone] flavoprotein 3, mitochondrial isoform X4	1743	0
XM_020099694.1	transmembrane protease serine 3	1406	0
XM_020099695.1	ATP-dependent RNA helicase DDX18 isoform X1	2753	0
XM_020099696.1	plexin domain-containing protein 2-like isoform X1	2901	0
XM_020099697.1	ATP-dependent RNA helicase DDX18 isoform X1	2598	0
XM_020099698.1	glycerol kinase-like isoform X1	2904	0
XM_020099699.1	glycerol kinase-like isoform X2	2829	0
XM_020099700.1	ribosomal RNA processing protein 1 homolog B-like isoform X1	3851	0
XM_020099701.1	ribosomal RNA processing protein 1 homolog B-like isoform X2	3837	0
XM_020099702.1	ribosomal RNA processing protein 1 homolog B-like isoform X3	3713	0
XM_020099703.1	tensin-1 isoform X1	8582	0
XM_020099704.1	tensin-1 isoform X2	8427	0
XM_020099705.1	plexin domain-containing protein 2-like isoform X1	2640	0
XM_020099706.1	tensin-1 isoform X3	8501	0
XM_020099707.1	tensin-1 isoform X4	8282	0
XM_020099708.1	tensin-1 isoform X5	8314	0
XM_020099709.1	tensin-1 isoform X6	7997	0
XM_020099710.1	tensin-1 isoform X7	8345	0
XM_020099711.1	serine/threonine-protein kinase 17A-like	4463	0
XM_020099712.1	UPF0565 protein C2orf69 homolog	3074	0
XM_020099713.1	collagen alpha-2(V) chain	5809	0
XM_020099714.1	plexin domain-containing protein 2-like isoform X1	2898	0
XM_020099715.1	PMS1 protein homolog 1	2958	0
XM_020099716.1	probable inactive tRNA-specific adenosine deaminase-like protein 3	1272	0
XM_020099717.1	alpha-ketoglutarate-dependent dioxygenase alkB homolog 6	1544	5.24E-153
XM_020099718.1	alpha-ketoglutarate-dependent dioxygenase alkB homolog 6	1468	2.20E-153
XM_020099719.1	ORM1-like protein 3	1578	6.15E-106
XM_020099720.1	integrin beta-2-like	3304	0
XM_020099721.1	integrin beta-2-like	3416	0
XM_020099722.1	UBX domain-containing protein 4	2579	0
XM_020099723.1	arginine and glutamate-rich protein 1 isoform X1	2506	1.74E-64
XM_020099724.1	arginine and glutamate-rich protein 1 isoform X2	2391	2.12E-35
XM_020099725.1	arginine and glutamate-rich protein 1 isoform X3	1660	1.00E-35
XM_020099726.1	CLIP-associating protein 1	7831	0
XM_020099727.1	tRNA (cytosine(34)-C(5))-methyltransferase, mitochondrial-like	2122	0
XM_020099728.1	GPI inositol-deacylase	7694	0
XM_020099729.1	COP9 signalosome complex subunit 8 isoform X1	1834	2.64E-131
XM_020099730.1	COP9 signalosome complex subunit 8 isoform X2	1896	0
XM_020099731.1	zinc finger protein GLI2 isoform X1	5845	0
XM_020099732.1	zinc finger protein GLI2 isoform X2	5728	0

XM_020099733.1	PREDICTED: symplekin	4288	0
XM_020099734.1	asparagine synthetase domain-containing protein 1	2286	0
XM_020099735.1	glucuronokinase 1-like	1036	0
XM_020099736.1	PTB domain-containing engulfment adapter protein 1 isoform X1	2324	0
XM_020099737.1	PTB domain-containing engulfment adapter protein 1 isoform X1	2333	0
XM_020099738.1	PTB domain-containing engulfment adapter protein 1 isoform X1	2321	0
XM_020099739.1	BTB/POZ domain-containing protein KCTD1 isoform X1	2260	0
XM_020099740.1	PTB domain-containing engulfment adapter protein 1 isoform X1	2330	0
XM_020099741.1	PTB domain-containing engulfment adapter protein 1 isoform X1	2060	0
XM_020099742.1	PTB domain-containing engulfment adapter protein 1 isoform X1	2056	0
XM_020099743.1	PTB domain-containing engulfment adapter protein 1 isoform X1	2238	0
XM_020099744.1	PTB domain-containing engulfment adapter protein 1 isoform X1	1741	0
XM_020099745.1	PTB domain-containing engulfment adapter protein 1 isoform X3	2088	0
XM_020099746.1	PTB domain-containing engulfment adapter protein 1 isoform X4	1506	0
XM_020099747.1	PTB domain-containing engulfment adapter protein 1 isoform X5	2003	0
XM_020099748.1	U3 small nucleolar RNA-associated protein 14 homolog A-like	2573	0
XM_020099749.1	BTB/POZ domain-containing protein KCTD1 isoform X2	2022	0
XM_020099750.1	PREDICTED: ephrin-B2	5220	0
XM_020099751.1	PREDICTED: ephrin-B2	1603	0
XM_020099752.1	insulin-like growth factor-binding protein 5	1309	0
XM_020099753.1	probable tRNA N6-adenosine threonylcarbamoyltransferase, mitochondrial	1632	0
XM_020099754.1	peptidyl-prolyl cis-trans isomerase FKBP7-like	1887	1.12E-160
XM_020099755.1	protein Jade-1	3140	0
XM_020099756.1	protein Jade-1	3058	0
XM_020099757.1	interferon alpha/beta receptor 1b-like isoform X1	3239	0
XM_020099758.1	interferon alpha/beta receptor 1b-like isoform X2	1685	0
XM_020099759.1	interferon alpha/beta receptor 1b-like isoform X3	1365	0
XM_020099760.1	rho-related GTP-binding protein RhoE-like	2102	3.32E-147
XM_020099761.1	fibrous sheath CABYR-binding protein-like	1837	8.04E-42
XM_020099762.1	NADH dehydrogenase [ubiquinone] flavoprotein 2, mitochondrial	988	3.53E-172
XM_020099763.1	anion exchange protein 3-like	5093	0
XM_020099764.1	solute carrier family 35 member F5 isoform X1	4586	0
XM_020099765.1	solute carrier family 35 member F5 isoform X1	4504	0
XM_020099766.1	solute carrier family 35 member F5 isoform X1	4453	0
XM_020099767.1	solute carrier family 35 member F5 isoform X1	4583	0
XM_020099768.1	interferon gamma receptor 2 precursor	2014	0
XM_020099769.1	interferon alpha/beta receptor 1-like isoform X2	2011	0
XM_020099770.1	tRNA wybutosine-synthesizing protein 5	1182	0
XM_020099771.1	m-AAA protease-interacting protein 1, mitochondrial	1586	1.57E-160
XM_020099772.1	tissue factor pathway inhibitor-like isoform X1	1791	0
XM_020099773.1	tissue factor pathway inhibitor-like isoform X2	1788	0
XM_020099774.1	40S ribosomal protein S15	638	3.08E-62
XM_020099775.1	interferon alpha/beta receptor 2-like	1867	0
XM_020099776.1	interferon alpha/beta receptor 2-like	3526	0
XM_020099777.1	protein FAM171B-like isoform X1	2527	0
XM_020099778.1	protein FAM171B-like isoform X2	2524	0
XM_020099779.1	protein FAM171B-like isoform X3	2293	0
XM_020099780.1	signal peptide, CUB and EGF-like domain-containing protein 1 isoform X1	7191	0
XM_020099781.1	signal peptide, CUB and EGF-like domain-containing protein 1 isoform X2	7101	0
XM_020099782.1	nuclear transcription factor Y subunit beta	1235	2.63E-150
XM_020099783.1	nuclear transcription factor Y subunit beta	1401	1.89E-149
XM_020099784.1	tubulin--tyrosine ligase-like protein 12	2187	0
XM_020099785.1	growth arrest-specific protein 2	2541	0
XM_020099786.1	growth arrest-specific protein 2	2576	0
XM_020099787.1	suppression of tumorigenicity 18 protein isoform X1	5535	0
XM_020099788.1	interleukin-17 receptor A-like	1659	0
XM_020099789.1	eukaryotic translation initiation factor 4 gamma 1-like	3729	2.20E-94
XM_020099790.1	LARGE xylosyl- and glucuronyltransferase 1	4523	0
XM_020099791.1	microsomal glutathione S-transferase 1	1257	6.13E-100
XM_020099792.1	microsomal glutathione S-transferase 1-like	631	1.45E-103
XM_020099793.1	suppression of tumorigenicity 18 protein isoform X2	5532	0
XM_020099794.1	deoxyribose-phosphate aldolase	1225	0
XM_020099795.1	patatin-like phospholipase domain-containing protein 2	2073	0
XM_020099796.1	heat shock 70 kDa protein 14	1699	0
XM_020099797.1	aryl hydrocarbon receptor nuclear translocator-like protein 2 isoform X1	3314	0
XM_020099798.1	aryl hydrocarbon receptor nuclear translocator-like protein 2 isoform X2	3311	0
XM_020099799.1	aryl hydrocarbon receptor nuclear translocator-like protein 2 isoform X3	3311	0
XM_020099800.1	aryl hydrocarbon receptor nuclear translocator-like protein 2 isoform X4	3311	0
XM_020099801.1	aryl hydrocarbon receptor nuclear translocator-like protein 2 isoform X5	3305	0
XM_020099802.1	aryl hydrocarbon receptor nuclear translocator-like protein 2 isoform X6	3299	0
XM_020099803.1	cadherin-related family member 2	3178	0
XM_020099804.1	suppression of tumorigenicity 18 protein isoform X1	5453	0
XM_020099805.1	aryl hydrocarbon receptor nuclear translocator-like protein 2 isoform X7	3272	0
XM_020099806.1	aryl hydrocarbon receptor nuclear translocator-like protein 2 isoform X8	3263	0
XM_020099807.1	aryl hydrocarbon receptor nuclear translocator-like protein 2 isoform X9	3224	0
XM_020099808.1	LIM domain only protein 3 isoform X2	2665	4.16E-106
XM_020099809.1	LIM domain only protein 3 isoform X2	3032	1.73E-98
XM_020099810.1	LIM domain only protein 3 isoform X2	2665	2.65E-99
XM_020099811.1	interleukin-1 receptor-associated kinase 3	2770	0

XM_020099812.1	mesencephalic astrocyte-derived neurotrophic factor-like	1639	4.58E-106
XM_020099813.1	suppression of tumorigenicity 18 protein isoform X4	5466	0
XM_020099814.1	PREDICTED: uncharacterized protein LOC109637451	497	4.19E-89
XM_020099815.1	rho guanine nucleotide exchange factor 39	3363	0
XM_020099816.1	glycosyltransferase 8 domain-containing protein 2 isoform X2	1643	0
XM_020099817.1	glycosyltransferase 8 domain-containing protein 2 isoform X2	1576	0
XM_020099818.1	glycosyltransferase 8 domain-containing protein 2 isoform X2	1752	0
XM_020099819.1	glycosyltransferase 8 domain-containing protein 2 isoform X2	1578	0
XM_020099820.1	sulfotransferase 4A1	5847	0
XM_020099821.1	suppression of tumorigenicity 18 protein isoform X5	5463	0
XM_020099822.1	amphoterin-induced protein 2-like	4021	0
XM_020099823.1	amphoterin-induced protein 2-like	4014	0
XM_020099824.1	amphoterin-induced protein 2-like	3955	0
XM_020099825.1	amphoterin-induced protein 2-like	4121	0
XM_020099826.1	pleckstrin homology-like domain family A member 1	3079	1.34E-87
XM_020099827.1	PREDICTED: ADM2-like	3990	7.03E-97
XM_020099828.1	suppression of tumorigenicity 18 protein isoform X1	5277	0
XM_020099829.1	metallophosphoesterase MPPED2	1567	0
XM_020099830.1	metallophosphoesterase MPPED2	1452	0
XM_020099831.1	acyl-CoA-binding protein homolog 1-like	662	7.93E-80
XM_020099832.1	zinc finger protein 850-like	4181	0
XM_020099833.1	L-lactate dehydrogenase B chain-like	1322	3.61E-168
XM_020099834.1	transmembrane protein 178B-like	1320	3.27E-168
XM_020099835.1	cat eye syndrome critical region protein 6	1316	0
XM_020099836.1	myotubularin-related protein 5-like	3198	0
XM_020099837.1	chloride anion exchanger	2354	0
XM_020099838.1	transmembrane 7 superfamily member 3	2761	0
XM_020099839.1	P2Y purinoceptor 1-like	1110	0
XM_020099840.1	mitochondrial 10-formyltetrahydrofolate dehydrogenase	5316	0
XM_020099841.1	solute carrier family 41 member 2	3617	0
XM_020099842.1	PREDICTED: uncharacterized protein C12orf45 homolog	1631	2.62E-72
XM_020099843.1	SH3-containing GRB2-like protein 3-interacting protein 1 isoform X1	5895	0
XM_020099844.1	ankyrin repeat domain-containing protein 16	1391	0
XM_020099845.1	selenoprotein O-like isoform X1	2445	0
XM_020099846.1	selenoprotein O-like isoform X2	2442	0
XM_020099847.1	selenoprotein O-like isoform X3	2292	0
XM_020099848.1	tubulin beta-4B chain-like	430	3.96E-66
XM_020099849.1	sorting nexin-4-like	2376	0
XM_020099850.1	SH3-containing GRB2-like protein 3-interacting protein 1 isoform X2	5889	0
XM_020099851.1	glycogen [starch] synthase, muscle-like	2510	0
XM_020099852.1	fish-egg lectin-like	1087	9.60E-154
XM_020099853.1	fish-egg lectin-like	1256	5.08E-165
XM_020099854.1	AT-rich interactive domain-containing protein 2-like	1351	3.20E-62
XM_020099855.1	AT-rich interactive domain-containing protein 2-like	615	1.69E-65
XM_020099856.1	angiopoietin-related protein 5-like	1237	0
XM_020099857.1	PREDICTED: uncharacterized protein LOC109637484	1677	0
XM_020099858.1	SH3-containing GRB2-like protein 3-interacting protein 1 isoform X3	5838	0
XM_020099859.1	sodium-coupled neutral amino acid transporter 2-like	907	3.86E-132
XM_020099860.1	ras-related and estrogen-regulated growth inhibitor-like protein	2001	7.55E-146
XM_020099861.1	PREDICTED: spexin	570	8.69E-67
XM_020099862.1	somatolactin precursor	693	9.26E-165
XM_020099863.1	prestin isoform X1	3085	0
XM_020099864.1	prestin isoform X1	3112	0
XM_020099865.1	SH3-containing GRB2-like protein 3-interacting protein 1 isoform X3	5826	0
XM_020099866.1	prestin isoform X1	2989	0
XM_020099867.1	prestin isoform X2	3034	0
XM_020099868.1	prestin isoform X3	3031	0
XM_020099869.1	prestin isoform X4	3028	0
XM_020099870.1	prestin isoform X1	2482	0
XM_020099871.1	dnaJ homolog subfamily C member 2	2199	0
XM_020099872.1	mitochondrial-processing peptidase subunit beta	1612	0
XM_020099873.1	26S protease regulatory subunit 7	1522	0
XM_020099874.1	SH3-containing GRB2-like protein 3-interacting protein 1 isoform X5	5823	0
XM_020099875.1	serine/threonine-protein phosphatase 6 regulatory subunit 2-like isoform X1	6994	0
XM_020099876.1	serine/threonine-protein phosphatase 6 regulatory subunit 2-like isoform X1	6678	0
XM_020099877.1	serine/threonine-protein phosphatase 6 regulatory subunit 2-like isoform X1	6991	0
XM_020099878.1	serine/threonine-protein phosphatase 6 regulatory subunit 2-like isoform X1	6985	0
XM_020099879.1	E3 ubiquitin-protein ligase Hakai isoform X1	1862	0
XM_020099880.1	E3 ubiquitin-protein ligase Hakai isoform X2	1859	0
XM_020099881.1	E3 ubiquitin-protein ligase Hakai isoform X3	1887	0
XM_020099882.1	serine/threonine-protein kinase B-raf isoform X1	2857	0
XM_020099883.1	serine/threonine-protein kinase B-raf isoform X2	2852	0
XM_020099884.1	serine/threonine-protein kinase B-raf isoform X3	2718	0
XM_020099885.1	serine/threonine-protein kinase B-raf isoform X4	2712	0

XM_020099886.1	28S ribosomal protein S33, mitochondrial	1180	8.87E-54
XM_020099887.1	28S ribosomal protein S33, mitochondrial	1158	1.41E-53
XM_020099888.1	growth arrest and DNA damage-inducible protein GADD45 alpha-like	1418	1.22E-105
XM_020099889.1	28S ribosomal protein S33, mitochondrial	1013	4.36E-54
XM_020099890.1	podocalyxin isoform X1	3393	0

XM_020099891.1	podocalyxin isoform X2	3369	0
XM_020099892.1	muskelin isoform X1	8297	0
XM_020099893.1	muskelin isoform X2	8244	0
XM_020099894.1	nucleosome assembly protein 1-like 1 isoform X5	3107	3.71E-174
XM_020099895.1	nucleosome assembly protein 1-like 1 isoform X5	3105	3.61E-174
XM_020099896.1	nucleosome assembly protein 1-like 1 isoform X3	3095	3.89E-169
XM_020099897.1	nucleosome assembly protein 1-like 1 isoform X3	1327	2.83E-177
XM_020099898.1	nucleosome assembly protein 1-like 1 isoform X5	1341	0
XM_020099899.1	thymopoietin isoform X1	2751	0
XM_020099900.1	thymopoietin isoform X2	2747	0
XM_020099901.1	thymopoietin isoform X3	2747	0
XM_020099902.1	adenylate cyclase type 1 isoform X1	3691	0
XM_020099903.1	thymopoietin isoform X4	2617	0
XM_020099904.1	thymopoietin isoform X5	2553	0
XM_020099905.1	thymopoietin isoform X6	2276	0
XM_020099906.1	serine-threonine kinase receptor-associated protein	2065	0
XM_020099907.1	zinc finger protein 420-like isoform X1	3539	0
XM_020099908.1	zinc finger protein 420-like isoform X2	3536	0
XM_020099909.1	DNA-(apurinic or apyrimidinic site) lyase isoform X2	1642	0
XM_020099910.1	DNA-(apurinic or apyrimidinic site) lyase isoform X2	1511	0
XM_020099911.1	adenylate cyclase type 1 isoform X2	3646	0
XM_020099912.1	DNA-(apurinic or apyrimidinic site) lyase isoform X2	1559	0
XM_020099913.1	DNA-(apurinic or apyrimidinic site) lyase isoform X2	1438	0
XM_020099914.1	probable tRNA N6-adenosine threonylcarbamoyltransferase	1203	0
XM_020099915.1	DENN domain-containing protein 5B	6074	0
XM_020099916.1	caprin-2 isoform X1	3405	0
XM_020099917.1	caprin-2 isoform X2	3402	0
XM_020099918.1	caprin-2 isoform X3	3387	0
XM_020099919.1	caprin-2 isoform X4	3384	0
XM_020099920.1	caprin-2 isoform X5	3187	0
XM_020099921.1	neuron navigator 3 isoform X1	11388	0
XM_020099922.1	neuron navigator 3 isoform X2	11364	0
XM_020099923.1	neuron navigator 3 isoform X3	11364	0
XM_020099924.1	transcription factor COE1	1268	4.33E-125
XM_020099925.1	histone H3.v1-like	1326	1.82E-55
XM_020099926.1	neuron navigator 3 isoform X4	11361	0
XM_020099927.1	neuron navigator 3 isoform X5	11340	0
XM_020099928.1	neuron navigator 3 isoform X6	11319	0
XM_020099929.1	neuron navigator 3 isoform X7	11214	0
XM_020099930.1	neuron navigator 3 isoform X8	10962	0
XM_020099931.1	neuron navigator 3 isoform X9	10920	0
XM_020099932.1	solute carrier family 13 member 4 isoform X1	4148	0
XM_020099933.1	solute carrier family 13 member 4 isoform X2	2093	0
XM_020099934.1	sushi domain-containing protein 3-like	3173	1.04E-149
XM_020099935.1	sushi domain-containing protein 3-like	2946	1.58E-150
XM_020099936.1	cytochrome c oxidase assembly factor 6 homolog	1248	5.22E-35
XM_020099937.1	cytochrome c oxidase assembly factor 6 homolog	1251	5.30E-35
XM_020099938.1	cytochrome c oxidase assembly factor 6 homolog	1201	2.22E-35
XM_020099939.1	palmitoyltransferase ZDHHC17	4615	0
XM_020099940.1	cysteine and glycine-rich protein 2	828	1.08E-123
XM_020099941.1	ATP-dependent DNA helicase Q1 isoform X1	3452	0
XM_020099942.1	ATP-dependent DNA helicase Q1 isoform X1	3581	0
XM_020099943.1	ATP-dependent DNA helicase Q1 isoform X1	3448	0
XM_020099944.1	ATP-dependent DNA helicase Q1 isoform X1	2636	0
XM_020099945.1	vesicle transport protein GOT1B	1167	5.50E-63
XM_020099946.1	oxysterol-binding protein-related protein 8 isoform X1	7569	0
XM_020099947.1	oxysterol-binding protein-related protein 8 isoform X1	7311	0
XM_020099948.1	oxysterol-binding protein-related protein 8 isoform X1	7503	0
XM_020099949.1	oxysterol-binding protein-related protein 8 isoform X1	7532	0
XM_020099950.1	oxysterol-binding protein-related protein 8 isoform X1	7268	0
XM_020099951.1	inner nuclear membrane protein Man1	4396	0
XM_020099952.1	tenascin-like isoform X3	4297	0
XM_020099953.1	pleckstrin homology domain-containing family A member 5-like isoform X1	7401	0
XM_020099954.1	pleckstrin homology domain-containing family A member 5-like isoform X2	7264	0
XM_020099955.1	pleckstrin homology domain-containing family A member 5-like isoform X3	7141	0
XM_020099956.1	pleckstrin homology domain-containing family A member 5-like isoform X3	937	8.07E-155
XM_020099957.1	conserved oligomeric Golgi complex subunit 5	2939	0
XM_020099958.1	probable G-protein coupled receptor 22 isoform X1	3219	0
XM_020099959.1	probable G-protein coupled receptor 22 isoform X1	3113	0
XM_020099960.1	probable G-protein coupled receptor 22 isoform X1	3072	0
XM_020099961.1	NADH dehydrogenase [ubiquinone] 1 alpha subcomplex subunit 5	522	3.85E-83
XM_020099962.1	neuroepithelial cell-transforming gene 1 protein isoform X1	3233	0
XM_020099963.1	neuroepithelial cell-transforming gene 1 protein isoform X2	3019	0
XM_020099964.1	neuroepithelial cell-transforming gene 1 protein isoform X3	3174	0
XM_020099965.1	cytochrome b-c1 complex subunit 10-like	711	6.28E-58
XM_020099966.1	sodium-coupled neutral amino acid transporter 4	5736	0
XM_020099967.1	phosphate carrier protein, mitochondrial isoform X1	1517	0
XM_020099968.1	phosphate carrier protein, mitochondrial isoform X1	1517	0
XM_020099969.1	phosphate carrier protein, mitochondrial isoform X1	1687	0

XM_020099970.1	protein lifeguard 1-like	1518	3.97E-142
XM_020099971.1	RecName: Full=Beta-2-microglobulin; Flags: Precursor	1136	1.89E-71
XM_020099972.1	PREDICTED: uncharacterized protein LOC109637536 isoform X1	3277	0
XM_020099973.1	PREDICTED: uncharacterized protein LOC109637536 isoform X2	3220	0
XM_020099974.1	PREDICTED: uncharacterized protein LOC109637536 isoform X1	3262	0
XM_020099975.1	PREDICTED: uncharacterized protein LOC109637536 isoform X3	3105	0
XM_020099976.1	PREDICTED: uncharacterized protein LOC109637536 isoform X3	3201	0
XM_020099977.1	KRR1 small subunit processome component homolog	1850	0
XM_020099978.1	zinc finger protein 568-like isoform X1	3478	0
XM_020099979.1	glioma pathogenesis-related protein 1-like	1486	1.65E-180
XM_020099980.1	rab GDP dissociation inhibitor beta-like	2319	0
XM_020099981.1	importin-8 isoform X1	2948	0
XM_020099982.1	importin-8 isoform X2	2939	0
XM_020099983.1	importin-8 isoform X3	2921	0
XM_020099984.1	importin-8 isoform X4	2912	0
XM_020099985.1	serine/threonine-protein kinase 38	6402	0
XM_020099986.1	zinc finger protein 773-like isoform X2	3421	0
XM_020099987.1	tubulin beta-4 chain-like	1777	0
XM_020099988.1	transmembrane and TPR repeat-containing protein 1 isoform X1	4704	0
XM_020099989.1	transmembrane and TPR repeat-containing protein 1 isoform X2	4536	0
XM_020099990.1	transcription factor E2F7 isoform X1	3830	0
XM_020099991.1	transcription factor E2F7 isoform X1	4774	0
XM_020099992.1	fish-egg lectin-like	1077	4.85E-180
XM_020099993.1	FGFR1 oncogene partner 2	1220	2.10E-145
XM_020099994.1	mediator of RNA polymerase II transcription subunit 21	941	6.75E-93
XM_020099995.1	E3 ubiquitin-protein ligase makorin-1	2959	0
XM_020099996.1	calcium/calmodulin-dependent 3',5'-cyclic nucleotide phosphodiesterase 1C	2967	0
XM_020099997.1	methionine-R-sulfoxide reductase B3 isoform X3	1475	3.72E-121
XM_020099998.1	methionine-R-sulfoxide reductase B3 isoform X3	1515	8.76E-117
XM_020099999.1	methionine-R-sulfoxide reductase B3 isoform X3	1514	4.99E-120
XM_020100000.1	methionine-R-sulfoxide reductase B3 isoform X3	1534	6.25E-120
XM_020100001.1	phosphorylated adapter RNA export protein	2240	0
XM_020100002.1	PREDICTED: uncharacterized protein LOC109637555	4557	0
XM_020100003.1	sorting and assembly machinery component 50 homolog	2501	0
XM_020100004.1	zinc finger protein aebp2-like	2555	0
XM_020100005.1	protein bicaudal D homolog 1-like isoform X3	7291	0
XM_020100006.1	protein bicaudal D homolog 1-like isoform X3	6112	0
XM_020100007.1	glutamate decarboxylase 1-like	2025	0
XM_020100008.1	protein bicaudal D homolog 1-like isoform X3	6117	0
XM_020100009.1	protein bicaudal D homolog 1-like isoform X3	6001	0
XM_020100010.1	potassium voltage-gated channel subfamily C member 2 isoform X2	3098	0
XM_020100011.1	potassium voltage-gated channel subfamily C member 2 isoform X2	4603	0
XM_020100012.1	potassium voltage-gated channel subfamily C member 2 isoform X2	3621	0
XM_020100013.1	potassium voltage-gated channel subfamily C member 2 isoform X2	2197	0
XM_020100014.1	potassium voltage-gated channel subfamily C member 2 isoform X2	3525	0
XM_020100015.1	potassium voltage-gated channel subfamily C member 2 isoform X2	4696	0
XM_020100016.1	cAMP-regulated D2 protein-like	2248	0
XM_020100017.1	glutamate decarboxylase 1-like	2595	0
XM_020100018.1	putative nuclease HARBI1	1973	0
XM_020100019.1	troponin T, skeletal muscle-like isoform X1	2597	0
XM_020100020.1	troponin T, skeletal muscle-like isoform X2	2597	0
XM_020100021.1	RecName: Full=Beta-2-microglobulin; Flags: Precursor	1683	2.24E-66
XM_020100022.1	anoctamin-4 isoform X3	3540	0
XM_020100023.1	anoctamin-4 isoform X3	3538	0
XM_020100024.1	anoctamin-4 isoform X3	3878	0
XM_020100025.1	RAD50-interacting protein 1 isoform X1	2745	0
XM_020100026.1	PREDICTED: pappalysin-2	7981	0
XM_020100027.1	RAD50-interacting protein 1 isoform X2	2410	0
XM_020100028.1	ankyrin repeat and SOCS box protein 13-like	1021	0
XM_020100029.1	ankyrin repeat and SOCS box protein 13-like	5126	0
XM_020100030.1	carbohydrate sulfotransferase 11 isoform X1	1264	0
XM_020100031.1	carbohydrate sulfotransferase 11 isoform X2	1208	0
XM_020100032.1	COP9 signalosome complex subunit 9	940	1.12E-28
XM_020100033.1	TAF6-like RNA polymerase II p300/CBP-associated factor-associated factor 65 kDa subunit 6L	3063	0
XM_020100034.1	TAF6-like RNA polymerase II p300/CBP-associated factor-associated factor 65 kDa subunit 6L	2849	0
XM_020100035.1	structure-specific endonuclease subunit slx1-like isoform X1	1293	0
XM_020100036.1	structure-specific endonuclease subunit slx1-like isoform X2	1287	0
XM_020100037.1	structure-specific endonuclease subunit slx1-like isoform X3	1028	0
XM_020100038.1	SH3 domain and tetratricopeptide repeat-containing protein 2-like	5830	0
XM_020100039.1	bolA-like protein 2	594	4.81E-58
XM_020100040.1	bolA-like protein 2	576	3.87E-58
XM_020100041.1	bolA-like protein 2	655	2.83E-57
XM_020100042.1	PREDICTED: uncharacterized protein C7orf43 homolog isoform X1	2277	0
XM_020100043.1	disabled homolog 1-like isoform X1	4003	0
XM_020100044.1	PREDICTED: uncharacterized protein C7orf43 homolog isoform X2	2329	0
XM_020100045.1	evolutionarily conserved signaling intermediate in Toll pathway, mitochondrial	2843	0
XM_020100046.1	evolutionarily conserved signaling intermediate in Toll pathway, mitochondrial	2874	0
XM_020100047.1	evolutionarily conserved signaling intermediate in Toll pathway, mitochondrial	2848	0
XM_020100048.1	evolutionarily conserved signaling intermediate in Toll pathway, mitochondrial	2878	0
XM_020100049.1	chromatin complexes subunit BAP18	1808	9.28E-82

XM_020100050.1	ubiquitin thioesterase OTUB1	1313	0
XM_020100051.1	nectin-4 isoform X1	4158	0
XM_020100052.1	disabled homolog 1-like isoform X1	4114	0
XM_020100053.1	nectin-4 isoform X1	4035	0
XM_020100054.1	nectin-4 isoform X1	4155	0
XM_020100055.1	NADH dehydrogenase [ubiquinone] iron-sulfur protein 2, mitochondrial	1631	0
XM_020100056.1	progestin and adipoQ receptor family member 9	5801	0
XM_020100057.1	RWD domain-containing protein 4	1376	1.82E-133
XM_020100058.1	galactose-3-O-sulfotransferase 2-like isoform X2	4692	0
XM_020100059.1	galactose-3-O-sulfotransferase 2-like isoform X2	4662	0
XM_020100060.1	IgGFc-binding protein-like	8604	0
XM_020100061.1	disabled homolog 1-like isoform X1	3877	0
XM_020100062.1	E3 ubiquitin-protein ligase pellino homolog 1-like	1715	0
XM_020100063.1	complement component C1q receptor-like	3750	0
XM_020100064.1	PREDICTED: uncharacterized protein LOC109637601	1565	1.85E-149
XM_020100065.1	PREDICTED: uncharacterized protein C11orf95 homolog	4701	0
XM_020100066.1	proline-, glutamic acid- and leucine-rich protein 1	3981	0
XM_020100067.1	P2Y purinoceptor 3-like	1529	0
XM_020100068.1	tyrosine-protein kinase Mer-like	3407	0
XM_020100069.1	thyrotropin receptor-like	2376	0
XM_020100070.1	protein c-ets-1-B-like	759	0
XM_020100071.1	alpha-N-acetylgalactosaminide alpha-2,6-sialyltransferase 5	3315	1.45E-174
XM_020100072.1	PREDICTED: focadhesin-like	724	2.50E-107
XM_020100073.1	PREDICTED: uncharacterized protein LOC109637611	1107	1.54E-169
XM_020100074.1	cytoplasmic aconitate hydratase	3938	0
XM_020100075.1	VPS10 domain-containing receptor SorCS2	5196	0
XM_020100076.1	kin of IRRE-like protein 1	4129	0
XM_020100077.1	snRNA-activating protein complex subunit 2	1585	0
XM_020100078.1	serine protease HTRA3	2088	0
XM_020100079.1	mRNA decay activator protein ZFP36L2-like	1363	0
XM_020100080.1	protein S100-A11-like	474	1.21E-66
XM_020100081.1	alpha-N-acetylgalactosaminide alpha-2,6-sialyltransferase 3	834	1.05E-166
XM_020100082.1	protein S100-P-like	500	1.32E-47
XM_020100083.1	metallo-beta-lactamase domain-containing protein 1	1338	1.52E-166
XM_020100084.1	cGMP-specific 3',5'-cyclic phosphodiesterase isoform X1	3050	0
XM_020100085.1	cGMP-specific 3',5'-cyclic phosphodiesterase isoform X2	3041	0
XM_020100086.1	HAUS augmin-like complex subunit 4	1188	0
XM_020100087.1	SH3 domain and tetratricopeptide repeat-containing protein 1	4588	0
XM_020100088.1	plasminogen activator inhibitor 1	2171	0
XM_020100089.1	PREDICTED: ependymin-like	821	4.59E-160
XM_020100090.1	high affinity immunoglobulin epsilon receptor subunit gamma-like	1285	6.84E-61
XM_020100091.1	arachidonate 5-lipoxygenase-like	2062	0
XM_020100092.1	forkhead box C1-A	2146	0
XM_020100093.1	lysophosphatidic acid receptor 6-like	1566	0
XM_020100094.1	C-C motif chemokine 3-like	571	1.31E-50
XM_020100095.1	PREDICTED: uncharacterized protein LOC109637632	835	4.81E-138
XM_020100096.1	peptidyl-glycine alpha-amidating monooxygenase isoform X1	3507	0
XM_020100097.1	peptidyl-glycine alpha-amidating monooxygenase isoform X2	3498	0
XM_020100098.1	peptidyl-glycine alpha-amidating monooxygenase isoform X3	3017	0
XM_020100099.1	peptidyl-glycine alpha-amidating monooxygenase isoform X4	3008	0
XM_020100100.1	tyrosine-protein kinase JAK2-like	1413	0
XM_020100101.1	ankyrin-2 isoform X1	12307	0
XM_020100102.1	ankyrin-2 isoform X2	11498	0
XM_020100103.1	zinc finger protein SNAI2 isoform X1	1040	6.35E-168
XM_020100104.1	ankyrin-2 isoform X3	11488	0
XM_020100105.1	ankyrin-2 isoform X4	11408	0
XM_020100106.1	ankyrin-2 isoform X5	7660	0
XM_020100107.1	putative transcription factor p65 homolog isoform X1	3207	0
XM_020100108.1	putative transcription factor p65 homolog isoform X2	3065	0
XM_020100109.1	UPF0696 protein C11orf68 homolog	1333	9.47E-161
XM_020100110.1	dr1-associated corepressor isoform X1	1124	3.90E-111
XM_020100111.1	dr1-associated corepressor isoform X2	998	7.96E-90
XM_020100112.1	zinc finger protein SNAI2 isoform X2	1019	1.16E-162
XM_020100113.1	ras-related protein Rab-1A-like	2581	1.01E-141
XM_020100114.1	RNA-binding protein 4.1-like	2458	0
XM_020100115.1	RNA-binding protein 4.1-like	2347	0
XM_020100116.1	RNA-binding protein 4.1-like	2931	0
XM_020100117.1	RNA-binding protein 4.1-like	1378	0
XM_020100118.1	calcium/calmodulin-dependent protein kinase type II delta 1 chain isoform X1	3469	0
XM_020100119.1	calcium/calmodulin-dependent protein kinase type II subunit delta isoform X2	3469	0
XM_020100120.1	calcium/calmodulin-dependent protein kinase type II delta 1 chain isoform X3	3466	0
XM_020100121.1	calcium/calmodulin-dependent protein kinase type II subunit delta isoform X4	3466	0
XM_020100122.1	calcium/calmodulin-dependent protein kinase type II delta 1 chain isoform X5	3447	0
XM_020100123.1	calcium/calmodulin-dependent protein kinase type II subunit delta isoform X6	3448	0
XM_020100124.1	calcium/calmodulin-dependent protein kinase type II delta 1 chain isoform X7	3444	0
XM_020100125.1	calcium/calmodulin-dependent protein kinase type II subunit delta isoform X8	3445	0
XM_020100126.1	small conductance calcium-activated potassium channel protein 2-like isoform X1	6366	0
XM_020100127.1	calcium/calmodulin-dependent protein kinase type II delta 1 chain isoform X9	3408	0
XM_020100128.1	calcium/calmodulin-dependent protein kinase type II delta 1 chain isoform X10	3405	0
XM_020100129.1	calcium/calmodulin-dependent protein kinase type II delta 1 chain isoform X11	3387	0

XM_020100130.1	calcium/calmodulin-dependent protein kinase type II delta 1 chain isoform X12	3387	0
XM_020100131.1	calcium/calmodulin-dependent protein kinase type II delta 1 chain isoform X13	3384	0
XM_020100132.1	calcium/calmodulin-dependent protein kinase type II delta chain isoform X14	3384	0
XM_020100133.1	calcium/calmodulin-dependent protein kinase type II delta chain isoform X15	3363	0
XM_020100134.1	calcium/calmodulin-dependent protein kinase type II delta chain isoform X16	3360	0
XM_020100135.1	calcium/calmodulin-dependent protein kinase type II delta chain isoform X17	3341	0
XM_020100136.1	calcium/calmodulin-dependent protein kinase type II delta chain isoform X18	3342	0
XM_020100137.1	small conductance calcium-activated potassium channel protein 2-like isoform X1	6357	0
XM_020100138.1	calcium/calmodulin-dependent protein kinase type II delta 1 chain isoform X19	3338	0
XM_020100139.1	calcium/calmodulin-dependent protein kinase type II delta chain isoform X20	3338	0
XM_020100140.1	actin-binding LIM protein 2 isoform X1	3659	0
XM_020100141.1	actin-binding LIM protein 2 isoform X2	3656	0
XM_020100142.1	actin-binding LIM protein 2 isoform X3	3653	0
XM_020100143.1	actin-binding LIM protein 2 isoform X4	3626	0
XM_020100144.1	actin-binding LIM protein 2 isoform X5	3617	0
XM_020100145.1	actin-binding LIM protein 2 isoform X6	3605	0
XM_020100146.1	actin-binding LIM protein 2 isoform X7	3587	0
XM_020100147.1	actin-binding LIM protein 2 isoform X8	3584	0
XM_020100148.1	actin-binding LIM protein 2 isoform X9	3371	0
XM_020100149.1	actin-binding LIM protein 2 isoform X10	3338	0
XM_020100150.1	actin-binding LIM protein 2 isoform X11	3317	0
XM_020100151.1	actin-binding LIM protein 2 isoform X12	3299	0
XM_020100152.1	actin-binding LIM protein 2 isoform X13	3266	0
XM_020100153.1	hepatocyte nuclear factor 6-like	1443	0
XM_020100154.1	actin-binding LIM protein 2 isoform X14	3245	0
XM_020100155.1	actin-binding LIM protein 2 isoform X15	3224	0
XM_020100156.1	actin-binding LIM protein 2 isoform X16	3137	0
XM_020100157.1	actin-binding LIM protein 2 isoform X17	3512	0
XM_020100158.1	zinc finger CCHC domain-containing protein 4	1716	0
XM_020100159.1	E3 ubiquitin-protein ligase TRIM62-like isoform X1	2337	0
XM_020100160.1	E3 ubiquitin-protein ligase TRIM62-like isoform X1	2066	0
XM_020100161.1	E3 ubiquitin-protein ligase TRIM62-like isoform X1	2266	0
XM_020100162.1	reticulon-3-like isoform X1	4412	3.62E-121
XM_020100163.1	reticulon-3-like isoform X2	4362	2.36E-129
XM_020100164.1	reticulon-3-like isoform X3	7034	0
XM_020100165.1	eukaryotic initiation factor 4A-I	3102	0
XM_020100166.1	autophagy-related protein 9A-like	891	0
XM_020100167.1	B-cell lymphoma/leukemia 10 isoform X1	2532	2.44E-119
XM_020100168.1	mediator of RNA polymerase II transcription subunit 11	4221	1.26E-68
XM_020100169.1	mitogen-activated protein kinase kinase kinase 11-like	12631	0
XM_020100170.1	PREDICTED: focadhesin	5847	0
XM_020100171.1	PREDICTED: focadhesin	5794	0
XM_020100172.1	PREDICTED: focadhesin	5620	0
XM_020100173.1	very-long-chain (3R)-3-hydroxyacyl-CoA dehydratase 4 isoform X1	1758	6.38E-124
XM_020100174.1	very-long-chain (3R)-3-hydroxyacyl-CoA dehydratase 4 isoform X1	1651	1.60E-95
XM_020100175.1	rap1 GTPase-GDP dissociation stimulator 1 isoform X1	4645	0
XM_020100176.1	B-cell lymphoma/leukemia 10 isoform X2	2529	1.27E-118
XM_020100177.1	rap1 GTPase-GDP dissociation stimulator 1 isoform X2	4499	0
XM_020100178.1	transcriptional adapter 2-beta	5033	0
XM_020100179.1	coiled-coil domain-containing protein 96-like	1666	0
XM_020100180.1	grpE protein homolog 1, mitochondrial	1507	4.53E-143
XM_020100181.1	PREDICTED: uncharacterized protein LOC109637663 isoform X1	16576	0
XM_020100182.1	PREDICTED: uncharacterized protein LOC109637663 isoform X2	16461	0
XM_020100183.1	PREDICTED: uncharacterized protein LOC109637663 isoform X3	16345	0
XM_020100184.1	PREDICTED: uncharacterized protein LOC109637663 isoform X4	16276	0
XM_020100185.1	PREDICTED: uncharacterized protein LOC109637663 isoform X5	16027	0
XM_020100186.1	PREDICTED: uncharacterized protein LOC109637663 isoform X6	15523	0
XM_020100187.1	PREDICTED: uncharacterized protein LOC109637663 isoform X7	12418	0
XM_020100188.1	EH domain-binding protein 1-like protein 1 isoform X8	5932	0
XM_020100189.1	biliverdin reductase A	1365	0
XM_020100190.1	FRAS1-related extracellular matrix protein 1	9999	0
XM_020100191.1	leucine-rich repeat-containing protein 72-like	720	7.63E-126
XM_020100192.1	alpha-protein kinase 1	3840	0
XM_020100193.1	RAS guanyl-releasing protein 2 isoform X1	3386	0
XM_020100194.1	RAS guanyl-releasing protein 2 isoform X2	3385	0
XM_020100195.1	RAS guanyl-releasing protein 2 isoform X3	3400	0
XM_020100196.1	RAS guanyl-releasing protein 2 isoform X3	3768	0
XM_020100197.1	PREDICTED: ependymin-like	1036	1.23E-148
XM_020100198.1	glycogen phosphorylase, muscle form	3872	0
XM_020100199.1	transcription factor Sox-3	3069	5.15E-166
XM_020100200.1	splicing factor 1 isoform X1	3122	0
XM_020100201.1	cyclic nucleotide-gated cation channel beta-1-like isoform X1	5400	0
XM_020100202.1	splicing factor 1 isoform X2	2022	0
XM_020100203.1	disintegrin and metalloproteinase domain-containing protein 33-like	3335	0
XM_020100204.1	leucine-rich repeat and calponin homology domain-containing protein 4 isoform X1	3885	0
XM_020100205.1	leucine-rich repeat and calponin homology domain-containing protein 4 isoform X2	2404	0
XM_020100206.1	PREDICTED: uncharacterized protein KIAA0232 homolog	5808	0
XM_020100207.1	actin filament-associated protein 1 isoform X1	4290	0
XM_020100208.1	actin filament-associated protein 1 isoform X2	4287	0

XM_020100209.1	cyclic nucleotide-gated cation channel beta-1-like isoform X1	5430	0
XM_020100210.1	actin filament-associated protein 1 isoform X3	4119	0
XM_020100211.1	actin filament-associated protein 1 isoform X4	4042	0
XM_020100212.1	PREDICTED: uncharacterized protein LOC109637679	2605	1.80E-137
XM_020100213.1	excitatory amino acid transporter 2 isoform X2	3958	0
XM_020100214.1	spermatogenesis-associated protein 6-like isoform X1	1585	0
XM_020100215.1	spermatogenesis-associated protein 6-like isoform X2	1492	0
XM_020100216.1	tRNA methyltransferase 10 homolog A	1536	2.12E-179
XM_020100217.1	PREDICTED: uncharacterized protein LOC109637683	6353	0
XM_020100218.1	cyclic nucleotide-gated cation channel beta-1-like isoform X3	2600	0
XM_020100219.1	PREDICTED: uncharacterized protein LOC109637683	6475	0
XM_020100220.1	PREDICTED: uncharacterized protein LOC109637683	6615	0
XM_020100221.1	PREDICTED: uncharacterized protein LOC109637683	6303	0
XM_020100222.1	tetraspanin-5-like isoform X1	4428	0
XM_020100223.1	tetraspanin-5-like isoform X2	4314	0
XM_020100224.1	sentrin-specific protease 5-like isoform X2	3552	0
XM_020100225.1	protein AF-9 isoform X1	2584	0
XM_020100226.1	protein AF-9 isoform X2	2586	0
XM_020100227.1	cyclic nucleotide-gated cation channel beta-1-like isoform X1	5293	0
XM_020100228.1	protein AF-9 isoform X3	2531	0
XM_020100229.1	protein AF-9 isoform X4	2017	0
XM_020100230.1	equilibrative nucleoside transporter 1	4253	0
XM_020100231.1	serine/threonine-protein phosphatase PP1-beta catalytic subunit	1573	0
XM_020100232.1	dedicator of cytokinesis protein 11 isoform X1	7620	0
XM_020100233.1	dedicator of cytokinesis protein 11 isoform X2	7581	0
XM_020100234.1	dedicator of cytokinesis protein 11 isoform X1	6572	0
XM_020100235.1	protein phosphatase 1 regulatory subunit 15B-like	882	9.23E-143
XM_020100236.1	neurexin-2-like isoform X1	6312	0
XM_020100237.1	neurexin-1-like isoform X2	5717	0
XM_020100238.1	neurexin-2-like isoform X3	6222	0
XM_020100239.1	neurexin-1-beta-like isoform X4	5332	0
XM_020100240.1	neurexin-2-like isoform X5	6348	0
XM_020100241.1	NAD-dependent protein deacetylase sirtuin-3-like	1730	0
XM_020100242.1	TBC1 domain family member 14 isoform X1	4261	0
XM_020100243.1	TBC1 domain family member 14 isoform X2	3144	0
XM_020100244.1	TBC1 domain family member 14 isoform X1	3047	0
XM_020100245.1	PREDICTED: macrosialin-like	3611	1.15E-125
XM_020100246.1	mitogen-activated protein kinase kinase kinase kinase 2-like isoform X1	4481	0
XM_020100247.1	mitogen-activated protein kinase kinase kinase kinase 2-like isoform X2	4478	0
XM_020100248.1	schwannomin-interacting protein 1-like	2913	4.79E-124
XM_020100249.1	echinoderm microtubule-associated protein-like 3	4185	0
XM_020100250.1	PREDICTED: menin	2180	0
XM_020100251.1	PREDICTED: menin	2215	0
XM_020100252.1	coiled-coil domain-containing protein 39-like isoform X1	3222	0
XM_020100253.1	PREDICTED: menin	2653	0
XM_020100254.1	inhibitor of growth protein 2-like	1659	3.81E-165
XM_020100255.1	neuroligin-3-like isoform X2	6381	0
XM_020100256.1	histone H2A.Z	799	2.18E-86
XM_020100257.1	AP-1 complex subunit sigma-1A isoform X1	1796	1.82E-144
XM_020100258.1	AP-1 complex subunit sigma-1A isoform X2	1852	1.66E-126
XM_020100259.1	AP-1 complex subunit sigma-1A isoform X3	1725	3.44E-108
XM_020100260.1	arylsulfatase J	2923	0
XM_020100261.1	coiled-coil domain-containing protein 39-like isoform X2	3015	0
XM_020100262.1	protein TSSC4	1750	4.10E-146
XM_020100263.1	mannose-P-dolichol utilization defect 1 protein	1280	2.06E-146
XM_020100264.1	zinc finger protein OZF-like isoform X2	4774	0
XM_020100265.1	mitotic spindle assembly checkpoint protein MAD2A	1095	3.70E-148
XM_020100266.1	maternal embryonic leucine zipper kinase	2188	0
XM_020100267.1	succinate--CoA ligase [ADP/GDP-forming] subunit alpha, mitochondrial	1448	0
XM_020100268.1	beta-galactosidase-1-like protein 2	3033	0
XM_020100269.1	claudin domain-containing protein 1-like	1633	9.76E-150
XM_020100270.1	phosphoglucomutase-like protein 5	1985	0
XM_020100271.1	PREDICTED: neurotrypsin	4287	0
XM_020100272.1	guanylate cyclase soluble subunit beta-1	2294	0
XM_020100273.1	SPARC-like protein 1	2729	0
XM_020100274.1	SPARC-like protein 1	2676	0
XM_020100275.1	osteocalcin 2-like	1284	2.54E-97
XM_020100276.1	dihydropyrimidinase-related protein 1-like isoform X1	3402	0
XM_020100277.1	dihydropyrimidinase-related protein 1-like isoform X2	3049	0
XM_020100278.1	GTPase IMAP family member 4-like	2107	0
XM_020100279.1	ADP/ATP translocase 1	1450	0
XM_020100280.1	N6-adenosine-methyltransferase subunit METTL14	1792	0
XM_020100281.1	PREDICTED: uncharacterized protein LOC109637725	1952	4.60E-179
XM_020100282.1	histone PARylation factor 1	1313	0
XM_020100283.1	solute carrier family 22 member 7-like	1967	0
XM_020100284.1	solute carrier family 22 member 7-like	1780	0
XM_020100285.1	proline-rich basic protein 1	4627	0
XM_020100286.1	serine/threonine-protein phosphatase 2A 55 kDa regulatory subunit B beta isoform isoform X1	4816	0
XM_020100287.1	coiled-coil domain-containing protein 86	1013	7.25E-124

XM_020100288.1	ankyrin repeat and SOCS box protein 12-like	2448	0
XM_020100289.1	mast/stem cell growth factor receptor kita-like isoform X1	3235	0
XM_020100290.1	mast/stem cell growth factor receptor kita-like isoform X2	3232	0
XM_020100291.1	ankyrin repeat domain-containing protein 49 isoform X2	939	1.01E-148
XM_020100292.1	ankyrin repeat domain-containing protein 49 isoform X2	1074	1.86E-152
XM_020100293.1	EMILIN-2 isoform X2	3808	0
XM_020100294.1	neuromedin-K receptor	3917	0
XM_020100295.1	wolframin-like isoform X1	4536	0
XM_020100296.1	wolframin-like isoform X2	4533	0
XM_020100297.1	RING finger protein 24	869	2.98E-70
XM_020100298.1	sodium-dependent phosphate transport protein 2B-like	1824	0
XM_020100299.1	PREDICTED: uncharacterized protein LOC109637742	670	4.90E-74
XM_020100300.1	multiple PDZ domain protein-like	5536	0
XM_020100301.1	PREDICTED: protocadherin-18-like	4217	1.13E-130
XM_020100302.1	cGMP-specific 3',5'-cyclic phosphodiesterase-like	2766	0
XM_020100303.1	protein NOXP20	3117	0
XM_020100304.1	PREDICTED: uncharacterized protein LOC109637748	1276	0
XM_020100305.1	small integral membrane protein 14	530	2.60E-47
XM_020100306.1	D(1) dopamine receptor-like	1368	0
XM_020100307.1	putative methyltransferase NSUN7	2358	0
XM_020100308.1	M-phase phosphoprotein 8 isoform X2	5011	0
XM_020100309.1	double C2-like domain-containing protein beta	832	5.90E-80
XM_020100310.1	rho GTPase-activating protein 7-like	7705	0
XM_020100311.1	PREDICTED: mucin-3A-like	1535	0
XM_020100312.1	5-hydroxytryptamine receptor 4-like	1077	0
XM_020100313.1	cyclic nucleotide-gated channel rod photoreceptor subunit alpha-like	2046	0
XM_020100314.1	transcription factor E2F5-like	2237	0
XM_020100315.1	solute carrier family 22 member 7-like	573	7.60E-134
XM_020100316.1	synaptopodin-2-like isoform X1	4483	0
XM_020100317.1	inactive ubiquitin carboxyl-terminal hydrolase 53-like	6508	0
XM_020100318.1	ufm1-specific protease 2	1373	0
XM_020100319.1	cilia- and flagella-associated protein 97	1549	0
XM_020100320.1	F-box only protein 41-like	5975	0
XM_020100321.1	N-acyl-aromatic-L-amino acid amidohydrolase (carboxylate-forming) B-like	1397	0
XM_020100322.1	rhomboid domain-containing protein 2	1479	0
XM_020100323.1	3-hydroxybutyrate dehydrogenase type 2	1041	9.38E-167
XM_020100324.1	ecto-NOX disulfide-thiol exchanger 2 isoform X1	2979	0
XM_020100325.1	leucine-rich repeat and transmembrane domain-containing protein 2	3874	0
XM_020100326.1	zinc finger CCHC domain-containing protein 7-like isoform X1	1411	0
XM_020100327.1	DNA-directed primase/polymerase protein	2016	0
XM_020100328.1	myozenin-2-like isoform X2	997	0
XM_020100329.1	alpha-1D adrenergic receptor isoform X1	2502	0
XM_020100330.1	alpha-1D adrenergic receptor isoform X2	2412	0
XM_020100331.1	thrombospondin type-1 domain-containing protein 4-like isoform X1	2249	0
XM_020100332.1	thrombospondin type-1 domain-containing protein 4-like isoform X2	2234	0
XM_020100333.1	thrombospondin type-1 domain-containing protein 4-like isoform X1	2004	0
XM_020100334.1	GPI-linked NAD(P)(+)--arginine ADP-ribosyltransferase 1-like	2330	2.31E-166
XM_020100335.1	fatty acid-binding protein, intestinal	638	5.92E-94
XM_020100336.1	interleukin-8-like isoform X1	1662	2.73E-62
XM_020100337.1	E3 ubiquitin-protein ligase RNF126-like	1864	5.37E-168
XM_020100338.1	interleukin-8-like isoform X2	1120	4.21E-64
XM_020100339.1	perilipin-2-like isoform X1	1327	0
XM_020100340.1	perilipin-2-like isoform X2	1334	0
XM_020100341.1	neuromedin-K receptor-like	1528	0
XM_020100342.1	extracellular superoxide dismutase [Cu-Zn]	942	6.89E-138
XM_020100343.1	guanylate cyclase soluble subunit alpha-3	2858	0
XM_020100344.1	hairy and enhancer of split-related protein HELT	856	1.39E-165
XM_020100345.1	WD40 repeat-containing protein SMU1-like isoform X1	1498	0
XM_020100346.1	WD40 repeat-containing protein SMU1-like isoform X2	1336	0
XM_020100347.1	WD40 repeat-containing protein SMU1-like isoform X1	1339	0
XM_020100348.1	rho-related GTP-binding protein RhoH	1558	1.02E-142
XM_020100349.1	transmembrane protein 70, mitochondrial	1051	6.08E-178
XM_020100350.1	PREDICTED: enamelin	1112	3.08E-120
XM_020100351.1	PREDICTED: enamelin	1066	1.80E-120
XM_020100352.1	PREDICTED: enamelin	1105	3.89E-120
XM_020100353.1	calcium-binding protein 2-like	768	3.01E-124
XM_020100354.1	PREDICTED: uncharacterized protein LOC109637788	822	2.56E-141
XM_020100355.1	neuronal acetylcholine receptor subunit alpha-9	1665	0
XM_020100356.1	PREDICTED: uncharacterized protein LOC109637790	956	2.77E-30
XM_020100357.1	probable G-protein coupled receptor 83	1386	0
XM_020100358.1	PREDICTED: uncharacterized protein LOC109637791	2171	0
XM_020100359.1	formin-like protein 14	521	1.22E-34
XM_020100360.1	ligand-dependent nuclear receptor corepressor-like protein isoform X1	8138	0
XM_020100361.1	ligand-dependent nuclear receptor corepressor-like protein isoform X2	8048	0
XM_020100362.1	ligand-dependent nuclear receptor corepressor-like protein isoform X3	5818	0
XM_020100363.1	condensin complex subunit 3 isoform X1	3368	0
XM_020100364.1	condensin complex subunit 3 isoform X2	3358	0
XM_020100365.1	lipoyl synthase, mitochondrial	2049	0
XM_020100366.1	60S ribosomal protein L9	824	2.39E-140

XM_020100367.1	UDP-glucose 6-dehydrogenase	4115	0
XM_020100368.1	stromal interaction molecule 2 isoform X1	6578	0
XM_020100369.1	MARVEL domain-containing protein 2-like	2214	0
XM_020100370.1	heparan-alpha-glucosaminide N-acetyltransferase isoform X2	3527	0
XM_020100371.1	heparan-alpha-glucosaminide N-acetyltransferase isoform X2	3739	0
XM_020100372.1	heparan-alpha-glucosaminide N-acetyltransferase isoform X2	3406	0
XM_020100373.1	recombining binding protein suppressor of hairless isoform X2	3537	0
XM_020100374.1	F-box only protein 10	4866	0
XM_020100375.1	zinc finger and BTB domain-containing protein 5	5432	0
XM_020100376.1	DNA-directed RNA polymerase I subunit RPA49	1428	0
XM_020100377.1	MARVEL domain-containing protein 2-like	2195	0

XM_020100378.1	DNA polymerase theta	8729	0
XM_020100379.1	tyrosine-protein phosphatase non-receptor type 9-like isoform X1	4793	0
XM_020100380.1	tyrosine-protein phosphatase non-receptor type 9-like isoform X1	4745	0
XM_020100381.1	E3 ubiquitin-protein ligase ZFP91-like	2964	0
XM_020100382.1	PREDICTED: uncharacterized protein LOC109637811	1021	2.40E-149
XM_020100383.1	long-chain-fatty-acid--CoA ligase 1-like isoform X3	3984	0
XM_020100384.1	long-chain-fatty-acid--CoA ligase 1-like isoform X3	3985	0
XM_020100385.1	long-chain-fatty-acid--CoA ligase 1-like isoform X3	3984	0
XM_020100386.1	MARVEL domain-containing protein 2-like	2155	0
XM_020100387.1	long-chain-fatty-acid--CoA ligase 1-like isoform X3	3997	0
XM_020100388.1	receptor-type tyrosine-protein phosphatase delta-like isoform X1	8341	0
XM_020100389.1	receptor-type tyrosine-protein phosphatase delta-like isoform X2	7111	0
XM_020100390.1	receptor-type tyrosine-protein phosphatase delta-like isoform X3	7102	0
XM_020100391.1	PREDICTED: uncharacterized protein LOC109637814	2010	0
XM_020100392.1	5,6-dihydroxyindole-2-carboxylic acid oxidase-like	1832	0
XM_020100393.1	proteasome-associated protein ECM29 homolog isoform X1	6313	0
XM_020100394.1	proteasome-associated protein ECM29 homolog isoform X2	6371	0
XM_020100395.1	MARVEL domain-containing protein 2-like	2312	0
XM_020100396.1	RNA-binding protein 47 isoform X1	5329	0
XM_020100397.1	RNA-binding protein 47 isoform X1	5325	0
XM_020100398.1	RNA-binding protein 47 isoform X1	5131	0
XM_020100399.1	RNA-binding protein 47 isoform X1	4974	0
XM_020100400.1	RNA-binding protein 47 isoform X1	5545	0
XM_020100401.1	RNA-binding protein 47 isoform X1	5523	0
XM_020100402.1	RNA-binding protein 47 isoform X1	5521	0
XM_020100403.1	RNA-binding protein 47 isoform X5	5337	0
XM_020100404.1	RNA-binding protein 47 isoform X6	5312	0
XM_020100405.1	PREDICTED: teneurin-1-like	5745	0
XM_020100406.1	amyloid beta A4 precursor protein-binding family B member 2 isoform X1	5958	0
XM_020100407.1	amyloid beta A4 precursor protein-binding family B member 2 isoform X2	5955	0
XM_020100408.1	amyloid beta A4 precursor protein-binding family B member 2 isoform X3	5952	0
XM_020100409.1	amyloid beta A4 precursor protein-binding family B member 2 isoform X4	5949	0
XM_020100410.1	amyloid beta A4 precursor protein-binding family B member 2 isoform X5	5907	0
XM_020100411.1	ATPase WRNIP1	2237	0
XM_020100412.1	amyloid beta A4 precursor protein-binding family B member 2 isoform X5	4022	1.47E-158
XM_020100413.1	amyloid beta A4 precursor protein-binding family B member 2 isoform X5	3806	1.00E-157
XM_020100414.1	sister chromatid cohesion protein PDS5 homolog A isoform X1	4482	0
XM_020100415.1	sister chromatid cohesion protein PDS5 homolog A isoform X1	4510	0
XM_020100416.1	sister chromatid cohesion protein PDS5 homolog A isoform X1	4772	0
XM_020100417.1	sister chromatid cohesion protein PDS5 homolog A isoform X1	4789	0
XM_020100418.1	sister chromatid cohesion protein PDS5 homolog A isoform X1	4290	0
XM_020100419.1	ankyrin-2-like isoform X1	9707	0
XM_020100420.1	ankyrin-2-like isoform X2	9677	0
XM_020100421.1	ankyrin-2-like isoform X3	9493	0
XM_020100422.1	lysine-specific demethylase 4C-like isoform X1	5979	0
XM_020100423.1	lysine-specific demethylase 4C-like isoform X2	5976	0
XM_020100424.1	lysine-specific demethylase 4C-like isoform X3	5964	0
XM_020100425.1	proproteinase E-like	961	1.34E-93
XM_020100426.1	pre-mRNA-splicing factor ATP-dependent RNA helicase DHX15	2856	0
XM_020100427.1	ubiquitin-like modifier-activating enzyme 1	4812	0
XM_020100428.1	ubiquitin-like modifier-activating enzyme 1	5099	0
XM_020100429.1	ubiquitin-like modifier-activating enzyme 1	4792	0
XM_020100430.1	glyoxylate reductase/hydroxypyruvate reductase-like	1235	0
XM_020100431.1	NEDD4-binding protein 2	6819	0
XM_020100432.1	LON peptidase N-terminal domain and RING finger protein 1	5027	0
XM_020100433.1	2-hydroxyacylsphingosine 1-beta-galactosyltransferase	3683	0
XM_020100434.1	structural maintenance of chromosomes protein 2	4448	0
XM_020100435.1	PREDICTED: uncharacterized protein LOC109637832	919	1.20E-162
XM_020100436.1	cysteine-rich hydrophobic domain-containing protein 2	4163	2.14E-82
XM_020100437.1	ranBP-type and C3HC4-type zinc finger-containing protein 1-like	1893	0
XM_020100438.1	dnaJ homolog subfamily A member 1-like	1538	0
XM_020100439.1	dnaJ homolog subfamily A member 1-like	1308	0
XM_020100440.1	dnaJ homolog subfamily A member 1-like	2915	0
XM_020100441.1	dnaJ homolog subfamily A member 1-like	1325	0
XM_020100442.1	aprataxin isoform X1	1283	0
XM_020100443.1	aprataxin isoform X2	1313	0
XM_020100444.1	aprataxin isoform X3	1186	0
XM_020100445.1	aprataxin isoform X4	1176	0

XM_020100446.1	transcription initiation factor IIE subunit beta	1684	2.33E-148
XM_020100447.1	transcription initiation factor IIE subunit beta	1553	5.43E-149
XM_020100448.1	transcription initiation factor IIE subunit beta	1552	5.25E-149
XM_020100449.1	dynactin subunit 6	1055	4.81E-136
XM_020100450.1	small integral membrane protein 18	2275	1.84E-47
XM_020100451.1	PREDICTED: secretogranin-2-like	2016	0
XM_020100452.1	PREDICTED: uncharacterized protein LOC109637840 isoform X1	981	2.32E-63
XM_020100453.1	PREDICTED: uncharacterized protein LOC109637840 isoform X2	1093	5.68E-53
XM_020100454.1	paired box protein Pax-5	1559	0
XM_020100455.1	slit homolog 2 protein isoform X1	7001	0
XM_020100456.1	slit homolog 2 protein isoform X2	7046	0
XM_020100457.1	circadian locomoter output cycles protein kaput-like isoform X1	6256	0
XM_020100458.1	circadian locomoter output cycles protein kaput-like isoform X1	6187	0
XM_020100459.1	circadian locomoter output cycles protein kaput-like isoform X1	6190	0
XM_020100460.1	circadian locomoter output cycles protein kaput-like isoform X1	6122	0
XM_020100461.1	circadian locomoter output cycles protein kaput-like isoform X1	3212	0
XM_020100462.1	olfactomedin-like protein 2B isoform X2	3657	0
XM_020100463.1	circadian locomoter output cycles protein kaput-like isoform X1	3206	0
XM_020100464.1	circadian locomoter output cycles protein kaput-like isoform X4	6175	0
XM_020100465.1	exocyst complex component 1-like	1107	2.36E-137
XM_020100466.1	exocyst complex component 1-like	1067	6.41E-135
XM_020100467.1	ubiquitin-conjugating enzyme E2 K-like	3043	1.55E-129
XM_020100468.1	catenin alpha-2	4161	0
XM_020100469.1	leucine-rich repeat transmembrane neuronal protein 1	3251	0
XM_020100470.1	serine/threonine-protein kinase haspin	5023	0
XM_020100471.1	olfactomedin-like protein 2B isoform X1	3384	0
XM_020100472.1	choline transporter-like protein 1	2802	0
XM_020100473.1	calcium/calmodulin-dependent protein kinase type II delta 1 chain isoform X1	1833	0
XM_020100474.1	calcium/calmodulin-dependent protein kinase type II subunit delta isoform X2	3297	0
XM_020100475.1	calcium/calmodulin-dependent protein kinase type II delta 1 chain isoform X3	1830	0
XM_020100476.1	calcium/calmodulin-dependent protein kinase type II delta 1 chain isoform X5	1803	0
XM_020100477.1	calcium/calmodulin-dependent protein kinase type II delta 1 chain isoform X5	3268	0
XM_020100478.1	calcium/calmodulin-dependent protein kinase type II delta 1 chain isoform X7	1800	0
XM_020100479.1	calcium/calmodulin-dependent protein kinase type II delta 1 chain isoform X9	1775	0
XM_020100480.1	calcium/calmodulin-dependent protein kinase type II delta 1 chain isoform X10	1771	0
XM_020100481.1	calcium/calmodulin-dependent protein kinase type II delta 1 chain isoform X11	1748	0
XM_020100482.1	calcium/calmodulin-dependent protein kinase type II delta 1 chain isoform X13	1744	0
XM_020100483.1	calcium/calmodulin-dependent protein kinase type II delta 1 chain isoform X13	2569	0
XM_020100484.1	calcium/calmodulin-dependent protein kinase type II delta 1 chain isoform X7	3264	0
XM_020100485.1	zinc finger protein 501-like	2371	0
XM_020100486.1	calcium/calmodulin-dependent protein kinase type II delta 2 chain-like	677	1.11E-61
XM_020100487.1	kelch-like protein 4 isoform X1	2613	0
XM_020100488.1	kelch-like protein 4 isoform X1	2033	0
XM_020100489.1	peroxisomal N(1)-acetyl-spermine/spermidine oxidase-like	2272	0
XM_020100490.1	microsomal triglyceride transfer protein large subunit	3290	0
XM_020100491.1	replication factor C subunit 1	4266	0
XM_020100492.1	40S ribosomal protein S6	875	4.31E-167
XM_020100493.1	mitochondrial sodium/hydrogen exchanger 9B2 isoform X1	2939	0
XM_020100494.1	mitochondrial sodium/hydrogen exchanger 9B2 isoform X2	2900	0
XM_020100495.1	CDGSH iron-sulfur domain-containing protein 2	1434	3.92E-76
XM_020100496.1	homer protein homolog 3-like isoform X1	3141	0
XM_020100497.1	CDGSH iron-sulfur domain-containing protein 2	1211	4.57E-77
XM_020100498.1	alkaline ceramidase 2	3844	0
XM_020100499.1	pre-mRNA 3'-end-processing factor FIP1-like isoform X1	2409	0
XM_020100500.1	pre-mRNA 3'-end-processing factor FIP1-like isoform X2	2406	0
XM_020100501.1	tetraspanin-5-like isoform X2	3697	0
XM_020100502.1	Krueppel-like factor 3	1977	0
XM_020100503.1	testican-3-like isoform X1	3433	0
XM_020100504.1	homer protein homolog 3-like isoform X2	3132	0
XM_020100505.1	testican-3-like isoform X2	3424	0
XM_020100506.1	bifunctional heparan sulfate N-deacetylase/N-sulfotransferase 4-like isoform X1	4482	0
XM_020100507.1	bifunctional heparan sulfate N-deacetylase/N-sulfotransferase 4-like isoform X2	4486	0
XM_020100508.1	WD repeat-containing protein 19	4362	0
XM_020100509.1	coiled-coil domain-containing protein 149-like isoform X1	2909	0
XM_020100510.1	coiled-coil domain-containing protein 149-like isoform X2	2872	0
XM_020100511.1	leucine-rich repeat LGI family member 2-like	2474	0
XM_020100512.1	adhesion G protein-coupled receptor L3-like isoform X1	5253	0
XM_020100513.1	homer protein homolog 3-like isoform X3	3120	0
XM_020100514.1	adhesion G protein-coupled receptor L3-like isoform X1	6901	0
XM_020100515.1	adhesion G protein-coupled receptor L3-like isoform X1	7246	0
XM_020100516.1	adhesion G protein-coupled receptor L3-like isoform X1	7207	0
XM_020100517.1	adhesion G protein-coupled receptor L3-like isoform X1	7168	0
XM_020100518.1	adhesion G protein-coupled receptor L3-like isoform X5	7150	0
XM_020100519.1	adhesion G protein-coupled receptor L3-like isoform X6	7117	0
XM_020100520.1	adhesion G protein-coupled receptor L3-like isoform X7	7099	0
XM_020100521.1	homer protein homolog 3-like isoform X4	3111	0
XM_020100522.1	zinc finger protein ZIC 5	3090	0
XM_020100523.1	transmembrane protein 182 isoform X1	2172	8.73E-154
XM_020100524.1	transmembrane protein 182 isoform X2	2140	9.70E-147

XM_020100525.1	mid1-interacting protein 1-B-like	1670	6.60E-93
XM_020100526.1	dachshund homolog 1-like isoform X1	4311	0
XM_020100527.1	dachshund homolog 1-like isoform X1	4301	0
XM_020100528.1	dachshund homolog 1-like isoform X1	4507	0
XM_020100529.1	dachshund homolog 1-like isoform X1	4296	0
XM_020100530.1	dachshund homolog 1-like isoform X1	4294	0
XM_020100531.1	dachshund homolog 1-like isoform X3	4279	0
XM_020100532.1	dachshund homolog 1-like isoform X1	4255	0
XM_020100533.1	connector enhancer of kinase suppressor of ras 2-like	3770	0
XM_020100534.1	protein Wnt-3a	3645	0
XM_020100535.1	dachshund homolog 1-like isoform X3	4228	0
XM_020100536.1	dachshund homolog 1-like isoform X3	4213	0
XM_020100537.1	dachshund homolog 1-like isoform X1	4371	0
XM_020100538.1	dachshund homolog 1-like isoform X3	4240	0
XM_020100539.1	target of Nesh-SH3 isoform X1	3261	0
XM_020100540.1	target of Nesh-SH3 isoform X2	3171	0
XM_020100541.1	target of Nesh-SH3 isoform X3	3003	0

XM_020100542.1	target of Nesh-SH3 isoform X4	2913	0
XM_020100543.1	spindle and kinetochore-associated protein 3 isoform X1	2183	0
XM_020100544.1	spindle and kinetochore-associated protein 3 isoform X2	2180	0
XM_020100545.1	PREDICTED: tetraspanin-7	3652	5.64E-170
XM_020100546.1	transcription factor Sox-1a-like	2176	3.37E-117
XM_020100547.1	angiopoietin-related protein 1	3715	0
XM_020100548.1	PREDICTED: uncharacterized protein LOC109637891 isoform X1	1748	2.19E-178
XM_020100549.1	PREDICTED: uncharacterized protein LOC109637891 isoform X2	1320	1.01E-170
XM_020100550.1	protein CREG2	1533	0
XM_020100551.1	interleukin-1 receptor accessory protein-like 1-B isoform X1	3562	0
XM_020100552.1	interleukin-1 receptor accessory protein-like 1-B isoform X1	3603	0
XM_020100553.1	interleukin-1 receptor accessory protein-like 1-B isoform X1	3559	0
XM_020100554.1	interleukin-1 receptor accessory protein-like 1-B isoform X1	3517	0
XM_020100555.1	interleukin-1 receptor accessory protein-like 1-B isoform X4	3514	0
XM_020100556.1	POU domain, class 3, transcription factor 3	1743	0
XM_020100557.1	probable G-protein coupled receptor 45	2661	0
XM_020100558.1	probable G-protein coupled receptor 45	2706	0
XM_020100559.1	probable G-protein coupled receptor 45	2770	0
XM_020100560.1	protein ABHD8-like	3840	0
XM_020100561.1	probable G-protein coupled receptor 45	2519	0
XM_020100562.1	gap junction alpha-3 protein-like	1646	0
XM_020100563.1	cyclic AMP receptor-like protein A isoform X1	3155	0
XM_020100564.1	cyclic AMP receptor-like protein A isoform X1	3294	0
XM_020100565.1	tryptophan--tRNA ligase, mitochondrial	1904	0
XM_020100566.1	major facilitator superfamily domain-containing protein 9	2519	0
XM_020100567.1	potassium/sodium hyperpolarization-activated cyclic nucleotide-gated channel 2-like	1638	0
XM_020100568.1	cytochrome c oxidase copper chaperone	547	1.93E-41
XM_020100569.1	T-box transcription factor TBX15	1644	0
XM_020100570.1	PREDICTED: uncharacterized protein KIAA1211-like homolog isoform X1	3684	0
XM_020100571.1	phosphatidylinositol 4-phosphate 5-kinase type-1 gamma-like	3825	0
XM_020100572.1	PREDICTED: uncharacterized protein KIAA1211-like homolog isoform X2	3681	0
XM_020100573.1	Krueppel-like factor 12	2728	0
XM_020100574.1	G-protein coupled receptor 183-like	3028	0
XM_020100575.1	phospholipase A1 member A isoform X3	1973	0
XM_020100576.1	phospholipase A1 member A isoform X3	1955	0
XM_020100577.1	phospholipase A1 member A isoform X3	1969	0
XM_020100578.1	phospholipase A1 member A isoform X3	1964	0
XM_020100579.1	popeye domain-containing protein 2 isoform X1	2937	0
XM_020100580.1	popeye domain-containing protein 2 isoform X2	2796	0
XM_020100581.1	filamin A-interacting protein 1-like isoform X2	2791	0
XM_020100582.1	Krueppel-like factor 5	3778	0
XM_020100583.1	short stature homeobox protein isoform X1	3022	3.61E-166
XM_020100584.1	short stature homeobox protein isoform X2	3001	1.56E-160
XM_020100585.1	type I inositol 3,4-bisphosphate 4-phosphatase	1187	0
XM_020100586.1	alpha-tocopherol transfer protein-like isoform X2	1717	0
XM_020100587.1	inosine-uridine preferring nucleoside hydrolase-like	1021	0
XM_020100588.1	AF4/FMR2 family member 3	3649	0
XM_020100589.1	probable phospholipid-transporting ATPase IH isoform X4	6062	0
XM_020100590.1	RCC1 and BTB domain-containing protein 2	1452	0
XM_020100591.1	importin subunit alpha-4	4438	0
XM_020100592.1	protein CMSS1	1203	2.79E-178
XM_020100593.1	alpha-tocopherol transfer protein-like isoform X2	1606	4.65E-166
XM_020100594.1	interphotoreceptor matrix proteoglycan 2	3371	0
XM_020100595.1	BCL-6 corepressor	1884	0
XM_020100596.1	putative nuclease HARBI1	1257	0
XM_020100597.1	G-protein coupled receptor 183-like	1029	0
XM_020100598.1	integrin beta-like protein 1	2226	0
XM_020100599.1	cohesin subunit SA-2-like	3627	0
XM_020100600.1	kelch-like protein 9	3173	0
XM_020100601.1	serine/threonine-protein kinase LATS2-like	1105	1.19E-76
XM_020100602.1	mitogen-activated protein kinase kinase kinase kinase 4-like	1173	0
XM_020100603.1	cAMP-dependent protein kinase inhibitor beta	2566	4.90E-45

XM_020100604.1	TBC1 domain family member 8-like	4272	0
XM_020100605.1	nebulin-related-anchoring protein	16492	0
XM_020100606.1	protein disulfide-isomerase A5	3758	0
XM_020100607.1	hydroxyacid oxidase 2 isoform X1	1699	0
XM_020100608.1	hydroxyacid oxidase 2 isoform X2	1696	0
XM_020100609.1	hydroxyacid oxidase 2 isoform X3	1693	0
XM_020100610.1	hydroxyacid oxidase 2 isoform X4	1690	0
XM_020100611.1	hydroxyacid oxidase 2 isoform X1	1565	0
XM_020100612.1	hydroxyacid oxidase 2 isoform X5	1591	0
XM_020100613.1	four and a half LIM domains protein 2-like	1372	0
XM_020100614.1	PREDICTED: ashwin	1184	1.03E-131
XM_020100615.1	lambda-crystallin homolog	1216	0
XM_020100616.1	XK-related protein 4	2672	0
XM_020100617.1	sciellin isoform X1	2104	0
XM_020100618.1	sciellin isoform X1	2067	0
XM_020100619.1	sciellin isoform X3	2032	0
XM_020100620.1	39S ribosomal protein L30, mitochondrial	888	6.28E-113
XM_020100621.1	MIT domain-containing protein 1 isoform X1	1243	0
XM_020100622.1	MIT domain-containing protein 1 isoform X2	1239	0
XM_020100623.1	sodium/hydrogen exchanger 2-like	1324	9.59E-171
XM_020100624.1	3 beta-hydroxysteroid dehydrogenase/Delta 5--	1853	0
XM_020100625.1	regulator of nonsense transcripts 3A	2695	5.31E-121
XM_020100626.1	P2Y purinoceptor 8	2467	0
XM_020100627.1	P2Y purinoceptor 8	1984	0
XM_020100628.1	CREB-regulated transcription coactivator 1-like isoform X1	4991	0
XM_020100629.1	P2Y purinoceptor 8	2080	0
XM_020100630.1	N-arachidonyl glycine receptor	2247	0
XM_020100631.1	N-arachidonyl glycine receptor	2229	0
XM_020100632.1	coiled-coil domain-containing protein 18-like	1688	0
XM_020100633.1	zona pellucida-like domain-containing protein 1	1791	0
XM_020100634.1	CREB-regulated transcription coactivator 1-like isoform X2	4988	0
XM_020100635.1	probable ubiquitin carboxyl-terminal hydrolase FAF-X isoform X1	8530	0
XM_020100636.1	probable ubiquitin carboxyl-terminal hydrolase FAF-X isoform X1	8239	0
XM_020100637.1	probable ubiquitin carboxyl-terminal hydrolase FAF-X isoform X1	8057	0
XM_020100638.1	probable ubiquitin carboxyl-terminal hydrolase FAF-X isoform X1	9763	0
XM_020100639.1	probable ubiquitin carboxyl-terminal hydrolase FAF-X isoform X1	8518	0
XM_020100640.1	probable ubiquitin carboxyl-terminal hydrolase FAF-X isoform X1	9733	0
XM_020100641.1	probable ubiquitin carboxyl-terminal hydrolase FAF-X isoform X1	8491	0
XM_020100642.1	probable ubiquitin carboxyl-terminal hydrolase FAF-X isoform X5	10382	0
XM_020100643.1	probable ubiquitin carboxyl-terminal hydrolase FAF-X isoform X6	10343	0
XM_020100644.1	CREB-regulated transcription coactivator 1-like isoform X3	4886	0
XM_020100645.1	WD repeat-containing protein 3	2690	0
XM_020100646.1	WD repeat-containing protein 3	2688	0
XM_020100647.1	60S ribosomal protein L8	847	4.82E-180
XM_020100648.1	60S ribosomal protein L8	891	8.77E-180
XM_020100649.1	adenylate cyclase type 6-like	9012	0
XM_020100650.1	adenylate cyclase type 6-like	8997	0
XM_020100651.1	signal transducing adapter molecule 2	3865	0
XM_020100652.1	very-long-chain (3R)-3-hydroxyacyl-CoA dehydratase 2	1626	3.44E-98
XM_020100653.1	calcium uptake protein 3, mitochondrial-like	2583	0
XM_020100654.1	CREB-regulated transcription coactivator 1-like isoform X4	4883	0
XM_020100655.1	E3 ubiquitin-protein ligase MYCBP2 isoform X1	16339	0
XM_020100656.1	E3 ubiquitin-protein ligase MYCBP2 isoform X2	15469	0
XM_020100657.1	E3 ubiquitin-protein ligase MYCBP2 isoform X3	15460	0
XM_020100658.1	E3 ubiquitin-protein ligase MYCBP2 isoform X4	15451	0
XM_020100659.1	E3 ubiquitin-protein ligase MYCBP2 isoform X5	16315	0
XM_020100660.1	E3 ubiquitin-protein ligase MYCBP2 isoform X6	15439	0
XM_020100661.1	E3 ubiquitin-protein ligase MYCBP2 isoform X7	15436	0
XM_020100662.1	E3 ubiquitin-protein ligase MYCBP2 isoform X8	16234	0
XM_020100663.1	E3 ubiquitin-protein ligase MYCBP2 isoform X9	16210	0
XM_020100664.1	E3 ubiquitin-protein ligase MYCBP2 isoform X10	15289	0
XM_020100665.1	E3 ubiquitin-protein ligase MYCBP2 isoform X11	15283	0
XM_020100666.1	E3 ubiquitin-protein ligase MYCBP2 isoform X12	15253	0
XM_020100667.1	E3 ubiquitin-protein ligase MYCBP2 isoform X13	15247	0
XM_020100668.1	E3 ubiquitin-protein ligase MYCBP2 isoform X14	16081	0
XM_020100669.1	ubiquitin-like-conjugating enzyme ATG10 isoform X1	1337	5.76E-144
XM_020100670.1	E3 ubiquitin-protein ligase MYCBP2 isoform X15	15004	0
XM_020100671.1	M-phase phosphoprotein 8 isoform X1	6917	0
XM_020100672.1	M-phase phosphoprotein 8 isoform X2	5942	0
XM_020100673.1	M-phase phosphoprotein 8 isoform X3	3714	0
XM_020100674.1	transcription termination factor 2	3576	0
XM_020100675.1	collagen alpha-1(VIII) chain isoform X2	3979	2.71E-97
XM_020100676.1	collagen alpha-1(VIII) chain isoform X2	3870	5.72E-97
XM_020100677.1	collagen alpha-1(VIII) chain isoform X2	3973	1.61E-96
XM_020100678.1	discoidin, CUB and LCCL domain-containing protein 2	4285	0
XM_020100679.1	cell cycle control protein 50A-like	2273	0
XM_020100680.1	PREDICTED: exportin-4	4639	0
XM_020100681.1	intraflagellar transport protein 88 homolog isoform X1	3195	0
XM_020100682.1	ubiquitin-like-conjugating enzyme ATG10 isoform X2	1301	7.61E-143

XM_020100683.1	intraflagellar transport protein 88 homolog isoform X2	3189	0
XM_020100684.1	protein-lysine N-methyltransferase N6AMT2 isoform X1	1071	3.83E-141
XM_020100685.1	protein-lysine N-methyltransferase N6AMT2 isoform X1	1108	9.96E-138
XM_020100686.1	protein-lysine N-methyltransferase N6AMT2 isoform X1	1005	1.68E-141
XM_020100687.1	protein-lysine N-methyltransferase N6AMT2 isoform X1	925	4.61E-104
XM_020100688.1	protein-lysine N-methyltransferase N6AMT2 isoform X1	866	1.26E-103
XM_020100689.1	PREDICTED: interleukin-17D	2001	7.70E-122
XM_020100690.1	zinc finger MYM-type protein 2 isoform X1	6672	0
XM_020100691.1	zinc finger MYM-type protein 2 isoform X1	6685	0
XM_020100692.1	zinc finger MYM-type protein 2 isoform X1	6609	0
XM_020100693.1	alpha-1,3-mannosyl-glycoprotein 4-beta-N-acetylglucosaminyltransferase A	4972	0
XM_020100694.1	cytochrome c oxidase assembly factor 5	710	6.32E-50
XM_020100695.1	acetyl-coenzyme A synthetase, cytoplasmic	2514	0
XM_020100696.1	tripartite motif-containing 13-like isoform X2	1841	0
XM_020100697.1	tripartite motif-containing 13-like isoform X2	2072	0
XM_020100698.1	tripartite motif-containing 13-like isoform X2	1636	0
XM_020100699.1	tripartite motif-containing 13-like isoform X2	1565	0
XM_020100700.1	F-box/LRR-repeat protein 3-like	4351	0
XM_020100701.1	ceroid-lipofuscinosis neuronal protein 5	4224	0
XM_020100702.1	protein TFG isoform X3	2294	2.32E-145
XM_020100703.1	protein TFG isoform X2	2291	1.45E-144
XM_020100704.1	protein TFG isoform X3	2292	2.25E-145
XM_020100705.1	protein TFG isoform X4	2261	2.20E-135

XM_020100706.1	protein TFG isoform X5	2257	1.42E-135
XM_020100707.1	protein jagunal homolog 1-A-like	1587	5.05E-128
XM_020100708.1	interferon regulatory factor 4-like	1169	0
XM_020100709.1	mediator of RNA polymerase II transcription subunit 14 isoform X1	5255	0
XM_020100710.1	mediator of RNA polymerase II transcription subunit 14 isoform X2	5226	0
XM_020100711.1	mediator of RNA polymerase II transcription subunit 14 isoform X3	5133	0
XM_020100712.1	TBC1 domain family member 4 isoform X1	3208	5.35E-180
XM_020100713.1	TBC1 domain family member 4 isoform X1	3290	2.15E-174
XM_020100714.1	rho guanine nucleotide exchange factor 7-like isoform X3	3203	0
XM_020100715.1	rho guanine nucleotide exchange factor 7-like isoform X2	3352	0
XM_020100716.1	rho guanine nucleotide exchange factor 7-like isoform X3	3152	0
XM_020100717.1	DNA polymerase kappa	6134	0
XM_020100718.1	serine/threonine-protein kinase 38	7941	0
XM_020100719.1	kelch repeat and BTB domain-containing protein 7	4404	0
XM_020100720.1	ras-related protein Rab-33B-like isoform X1	1361	3.38E-175
XM_020100721.1	retinal homeobox protein Rx2-like	2916	0
XM_020100722.1	ras-related protein Rab-33B-like isoform X2	1355	1.42E-173
XM_020100723.1	rho GTPase-activating protein 20-like	5469	0
XM_020100724.1	sentrin-specific protease 7 isoform X1	4764	0
XM_020100725.1	sentrin-specific protease 7 isoform X1	4747	0
XM_020100726.1	sentrin-specific protease 7 isoform X1	4710	0
XM_020100727.1	sentrin-specific protease 7 isoform X1	4698	0
XM_020100728.1	semaphorin-5B isoform X1	6335	0
XM_020100729.1	semaphorin-5B isoform X2	3760	0
XM_020100730.1	katanin p60 ATPase-containing subunit A1	4124	0
XM_020100731.1	vesicle-trafficking protein SEC22a	3417	0
XM_020100732.1	serine/threonine-protein phosphatase 2A regulatory subunit B'' subunit beta-like isoform X1	2607	0
XM_020100733.1	serine/threonine-protein phosphatase 2A regulatory subunit B'' subunit beta-like isoform X1	2036	0
XM_020100734.1	C-C chemokine receptor type 3-like	2547	0
XM_020100735.1	serine/threonine-protein phosphatase 2A regulatory subunit B'' subunit beta-like isoform X3	2394	0
XM_020100736.1	cytoplasmic protein NCK2	3193	0
XM_020100737.1	centromere protein J isoform X1	4633	0
XM_020100738.1	centromere protein J isoform X2	4631	0
XM_020100739.1	cell division cycle protein 16 homolog	2416	0
XM_020100740.1	PREDICTED: uncharacterized protein C17orf105 homolog	912	0
XM_020100741.1	guanine nucleotide exchange factor DBS isoform X1	4796	0
XM_020100742.1	guanine nucleotide exchange factor DBS isoform X2	4781	0
XM_020100743.1	guanine nucleotide exchange factor DBS isoform X3	4703	0
XM_020100744.1	guanine nucleotide exchange factor DBS isoform X4	4515	0
XM_020100745.1	guanine nucleotide exchange factor DBS isoform X5	4437	0
XM_020100746.1	thioredoxin domain-containing protein 9	2070	1.16E-144
XM_020100747.1	CMRF35-like molecule 8	2188	0
XM_020100748.1	ADP-ribosylation factor-like protein 5A	1505	3.83E-129
XM_020100749.1	PREDICTED: uncharacterized protein LOC109638005 isoform X1	5012	0
XM_020100750.1	PREDICTED: uncharacterized protein LOC109638005 isoform X2	4982	0
XM_020100751.1	PREDICTED: uncharacterized protein LOC109638005 isoform X3	4940	0
XM_020100752.1	PREDICTED: uncharacterized protein LOC109638005 isoform X4	4925	0
XM_020100753.1	PREDICTED: uncharacterized protein LOC109638005 isoform X5	4910	0
XM_020100754.1	radixin isoform X1	2586	0
XM_020100755.1	radixin isoform X1	2451	0
XM_020100756.1	radixin isoform X1	2497	0
XM_020100757.1	fibroblast growth factor 14 isoform X1	1539	8.19E-178
XM_020100758.1	fibroblast growth factor 14 isoform X2	1496	0
XM_020100759.1	fibroblast growth factor 14 isoform X1	1337	1.70E-135
XM_020100760.1	zinc finger protein ZIC 2	2648	0
XM_020100761.1	monoacylglycerol lipase ABHD2-A	3410	0

XM_020100762.1	sodium leak channel non-selective protein isoform X1	6180	0
XM_020100763.1	sodium leak channel non-selective protein isoform X2	6012	0
XM_020100764.1	SLAIN motif-containing protein 1	3076	0
XM_020100765.1	citrate lyase subunit beta-like protein, mitochondrial isoform X1	2622	0
XM_020100766.1	citrate lyase subunit beta-like protein, mitochondrial isoform X2	1674	0
XM_020100767.1	citrate lyase subunit beta-like protein, mitochondrial isoform X3	2465	0
XM_020100768.1	transmembrane and TPR repeat-containing protein 4	2903	0
XM_020100769.1	N-acetylserotonin O-methyltransferase-like protein isoform X3	2182	0
XM_020100770.1	N-acetylserotonin O-methyltransferase-like protein isoform X3	2382	0
XM_020100771.1	N-acetylserotonin O-methyltransferase-like protein isoform X3	2165	0
XM_020100772.1	N-acetylserotonin O-methyltransferase-like protein isoform X3	2168	0
XM_020100773.1	gap junction beta-2 protein-like	2453	0
XM_020100774.1	regulator of G-protein signaling 21-like	2181	6.66E-172
XM_020100775.1	RING finger protein 17	5449	0
XM_020100776.1	renin receptor	1441	0
XM_020100777.1	voltage-dependent L-type calcium channel subunit beta-4 isoform X1	3538	0
XM_020100778.1	voltage-dependent L-type calcium channel subunit beta-4 isoform X2	3535	0
XM_020100779.1	voltage-dependent L-type calcium channel subunit beta-4 isoform X3	3309	0
XM_020100780.1	voltage-dependent L-type calcium channel subunit beta-4 isoform X4	3478	0
XM_020100781.1	arrestin domain-containing protein 3-like	2803	0
XM_020100782.1	growth arrest-specific protein 2	6518	0
XM_020100783.1	phospholipase B-like 1	2484	0
XM_020100784.1	translation factor Guf1, mitochondrial-like	692	2.56E-106
XM_020100785.1	serine/arginine repetitive matrix protein 1-like	2649	0
XM_020100786.1	pleckstrin homology domain-containing family A member 2	2575	0
XM_020100787.1	eukaryotic translation initiation factor 4E-binding protein 2-like	1262	5.02E-77

XM_020100788.1	THO complex subunit 5 homolog	2328	0
XM_020100789.1	kinesin-like protein KIF2A isoform X4	5508	0
XM_020100790.1	Rieske domain-containing protein-like isoform X1	1472	3.57E-101
XM_020100791.1	Rieske domain-containing protein-like isoform X1	1417	1.34E-101
XM_020100792.1	Rieske domain-containing protein-like isoform X1	1440	1.59E-89
XM_020100793.1	small G protein signaling modulator 1 isoform X1	4486	0
XM_020100794.1	small G protein signaling modulator 1 isoform X2	4480	0
XM_020100795.1	serine/threonine-protein kinase RIO2	2021	0
XM_020100796.1	protein prune homolog 2-like isoform X1	7379	0
XM_020100797.1	protein prune homolog 2-like isoform X2	7376	0
XM_020100798.1	regulator of G-protein signaling 20-like isoform X1	1714	0
XM_020100799.1	protein prune homolog 2-like isoform X3	7349	0
XM_020100800.1	protein prune homolog 2-like isoform X4	7340	0
XM_020100801.1	protein prune homolog 2-like isoform X5	7337	0
XM_020100802.1	protein prune homolog 2-like isoform X6	7304	0
XM_020100803.1	protein prune homolog 2-like isoform X7	6701	0
XM_020100804.1	protein prune homolog 2-like isoform X8	5423	0
XM_020100805.1	protein prune homolog 2-like isoform X9	5420	0
XM_020100806.1	kinetochore protein NDC80 homolog	2293	0
XM_020100807.1	dimethyladenosine transferase 1, mitochondrial	1560	0
XM_020100808.1	regulator of G-protein signaling 20-like isoform X1	1618	0
XM_020100809.1	leucine-rich repeat and transmembrane domain-containing protein 1-like	1526	0
XM_020100810.1	iron-sulfur cluster assembly enzyme ISCU, mitochondrial	1209	4.98E-101
XM_020100811.1	dnaJ homolog subfamily B member 5	3349	0
XM_020100812.1	dnaJ homolog subfamily B member 5	3161	0
XM_020100813.1	agrin isoform X6	6269	0
XM_020100814.1	agrin isoform X6	6266	0
XM_020100815.1	T-box transcription factor TBX5	1685	0
XM_020100816.1	regulator of G-protein signaling 20-like isoform X1	1608	5.58E-133
XM_020100817.1	sodium- and chloride-dependent GABA transporter ine-like isoform X1	3715	0
XM_020100818.1	sodium- and chloride-dependent GABA transporter ine-like isoform X2	3649	0
XM_020100819.1	WSC domain-containing protein 2	4083	0
XM_020100820.1	apoptosis-inducing factor 3 isoform X1	2976	0
XM_020100821.1	apoptosis-inducing factor 3 isoform X2	2955	0
XM_020100822.1	protein limb expression 1 homolog	2423	0
XM_020100823.1	coiled-coil domain-containing protein 42 like-2-like	1362	0
XM_020100824.1	EGF-like repeat and discoidin I-like domain-containing protein 3 isoform X1	2769	0
XM_020100825.1	EGF-like repeat and discoidin I-like domain-containing protein 3 isoform X2	2768	0
XM_020100826.1	RILP-like protein 1	2483	0
XM_020100827.1	RILP-like protein 1	2517	0
XM_020100828.1	forkhead box protein F2-like	1801	0
XM_020100829.1	proteinase-activated receptor 1	1336	0
XM_020100830.1	versican core protein-like	3856	0
XM_020100831.1	arrestin domain-containing protein 3-like	1011	0
XM_020100832.1	arrestin domain-containing protein 3-like	837	0
XM_020100833.1	urea transporter 1-like	1684	0
XM_020100834.1	RGM domain family member B-like	3943	0
XM_020100835.1	phospholipase DDHD2	2561	0
XM_020100836.1	sarcalumenin-like isoform X2	2500	0
XM_020100837.1	PREDICTED: uncharacterized protein LOC109638058	1961	0
XM_020100838.1	forkhead box protein Q1-like	1235	2.91E-173
XM_020100839.1	dynein heavy chain 10, axonemal	8550	0
XM_020100840.1	PREDICTED: smoothelin-like	1584	0

XM_020100841.1	cytoplasmic phosphatidylinositol transfer protein 1-like isoform X1	2064	0
XM_020100842.1	CAP-Gly domain-containing linker protein 1-like isoform X1	8183	0
XM_020100843.1	leucine-rich repeat-containing protein 43	1778	0
XM_020100844.1	beta-1,3-galactosyl-O-glycosyl-glycoprotein beta-1,6-N-acetylglucosaminyltransferase	1278	0
XM_020100845.1	C2 calcium-dependent domain-containing protein 4C-like	1260	0
XM_020100846.1	vacuolar protein sorting-associated protein 13C-like	10121	0
XM_020100847.1	guanine nucleotide-binding protein subunit alpha-14-like	1102	0
XM_020100848.1	cyclin-dependent kinase 20	1434	0
XM_020100849.1	fibronectin type III domain-containing protein 7-like	3067	0
XM_020100850.1	transmembrane protein 248-like	1687	0
XM_020100851.1	transmembrane protein 248-like	1695	0
XM_020100852.1	arrestin domain-containing protein 3-like	1790	0
XM_020100853.1	arrestin domain-containing protein 3-like	2379	0
XM_020100854.1	solute carrier family 2, facilitated glucose transporter member 11-like	1906	0
XM_020100855.1	endoplasmic reticulum aminopeptidase 1-like isoform X2	3246	0
XM_020100856.1	oxysterol-binding protein 2-like isoform X1	2433	0
XM_020100857.1	oxysterol-binding protein 2-like isoform X2	2092	0
XM_020100858.1	oxysterol-binding protein 2-like isoform X3	2047	0
XM_020100859.1	oxysterol-binding protein 2-like isoform X4	2184	0
XM_020100860.1	acyl-CoA dehydrogenase family member 11-like	2310	0
XM_020100861.1	acyl-CoA dehydrogenase family member 11-like	2294	0
XM_020100862.1	transient receptor potential cation channel subfamily V member 4	3670	0
XM_020100863.1	coiled-coil domain-containing protein 117 isoform X2	1637	9.00E-166
XM_020100864.1	coiled-coil domain-containing protein 117 isoform X2	1845	2.11E-156
XM_020100865.1	coiled-coil domain-containing protein 117 isoform X2	1849	1.15E-155
XM_020100866.1	coiled-coil domain-containing protein 117 isoform X2	1842	8.69E-156
XM_020100867.1	coiled-coil domain-containing protein 117 isoform X2	1846	3.57E-155
XM_020100868.1	coiled-coil domain-containing protein 117 isoform X2	1842	5.67E-154
XM_020100869.1	chemokine-like receptor 1	1422	0
XM_020100870.1	probable peptide chain release factor C12orf65 homolog, mitochondrial	849	7.27E-69
XM_020100871.1	TBT-binding protein	963	7.49E-136
XM_020100872.1	prosaposin isoform X2	1457	0
XM_020100873.1	putative defense protein Hdd11	786	2.09E-134
XM_020100874.1	potassium voltage-gated channel subfamily V member 2-like	3355	0
XM_020100875.1	centromere protein H	995	7.69E-167
XM_020100876.1	betaine--homocysteine S-methyltransferase 1-like	1503	0
XM_020100877.1	WD repeat-containing protein 66 isoform X1	3358	0
XM_020100878.1	WD repeat-containing protein 66 isoform X2	2771	0
XM_020100879.1	PREDICTED: trichohyalin-like	4348	0
XM_020100880.1	PREDICTED: uncharacterized protein LOC109638090	987	1.33E-124
XM_020100881.1	IQ domain-containing protein D-like	1274	4.20E-119
XM_020100882.1	IQ domain-containing protein D-like	874	3.03E-118
XM_020100883.1	transmembrane protein 119-like	2462	4.12E-121
XM_020100884.1	ras-like protein family member 10A	2055	3.66E-134
XM_020100885.1	calcium/calmodulin-dependent protein kinase II inhibitor 2	2350	1.30E-41
XM_020100886.1	hydroxycarboxylic acid receptor 3-like	1148	0
XM_020100887.1	glial cell line-derived neurotrophic factor-like	925	1.84E-128
XM_020100888.1	putative claudin-24	857	9.49E-148
XM_020100889.1	forkhead box protein B2	996	0
XM_020100890.1	glycolipid transfer protein	825	3.81E-155
XM_020100891.1	acetyl-CoA carboxylase 2 isoform X4	8278	0
XM_020100892.1	acetyl-CoA carboxylase 2 isoform X4	8476	0
XM_020100893.1	acetyl-CoA carboxylase 2 isoform X3	8254	0
XM_020100894.1	acetyl-CoA carboxylase 2 isoform X4	8296	0
XM_020100895.1	PREDICTED: uncharacterized protein LOC109638101 isoform X1	4936	0
XM_020100896.1	acetyl-CoA carboxylase 2 isoform X4	8568	0
XM_020100897.1	acetyl-CoA carboxylase 2 isoform X4	7754	0
XM_020100898.1	DNA polymerase epsilon catalytic subunit A	7268	0
XM_020100899.1	forkhead box protein N4	2939	0
XM_020100900.1	protein phosphatase Slingshot homolog 1 isoform X1	4866	0
XM_020100901.1	protein phosphatase Slingshot homolog 1 isoform X2	4826	0
XM_020100902.1	protein phosphatase Slingshot homolog 1 isoform X1	4423	0
XM_020100903.1	coronin-1C-like isoform X1	4207	0
XM_020100904.1	AF4/FMR2 family member 2	5999	0
XM_020100905.1	fibronectin type III and SPRY domain-containing protein 2	6629	0
XM_020100906.1	junction-mediating and -regulatory protein-like	4242	0
XM_020100907.1	poly(A) RNA polymerase GLD2-like isoform X1	2631	0
XM_020100908.1	poly(A) RNA polymerase GLD2-like isoform X1	2659	0
XM_020100909.1	PREDICTED: uncharacterized protein LOC109638101 isoform X2	4921	0
XM_020100910.1	poly(A) RNA polymerase GLD2-like isoform X1	2645	0
XM_020100911.1	poly(A) RNA polymerase GLD2-like isoform X1	2628	0
XM_020100912.1	homer protein homolog 1-like isoform X1	5744	0
XM_020100913.1	homer protein homolog 1-like isoform X2	5663	0
XM_020100914.1	homer protein homolog 1-like isoform X3	5917	0
XM_020100915.1	homer protein homolog 1-like isoform X4	1328	1.25E-132
XM_020100916.1	ankyrin-1 isoform X1	920	3.34E-89
XM_020100917.1	ankyrin-1 isoform X2	4500	6.43E-65
XM_020100918.1	ankyrin-1 isoform X3	6790	0
XM_020100919.1	PREDICTED: uncharacterized protein LOC109638101 isoform X3	4909	0

XM_020100920.1	thyroid adenoma-associated protein isoform X1	6187	0
XM_020100921.1	V-type proton ATPase 116 kDa subunit a	4536	0
XM_020100922.1	tectonic-2 isoform X1	2384	0
XM_020100923.1	tectonic-2 isoform X2	2380	0
XM_020100924.1	tectonic-2 isoform X3	2382	0
XM_020100925.1	clustered mitochondria protein homolog	4761	0
XM_020100926.1	tuftelin-interacting protein 11 isoform X1	2650	0
XM_020100927.1	tuftelin-interacting protein 11 isoform X1	2805	0
XM_020100928.1	tuftelin-interacting protein 11 isoform X1	2630	0
XM_020100929.1	SRR1-like protein	1100	0
XM_020100930.1	SRR1-like protein	1083	0
XM_020100931.1	SRR1-like protein	1014	0
XM_020100932.1	diencephalon/mesencephalon homeobox protein 1-B-like	2606	0
XM_020100933.1	DNA polymerase kappa	3520	0
XM_020100934.1	DNA polymerase kappa	3519	0
XM_020100935.1	collagen type IV alpha-3-binding protein isoform X1	4134	0
XM_020100936.1	collagen type IV alpha-3-binding protein isoform X2	4054	0
XM_020100937.1	phosphatidylinositol 4-phosphate 5-kinase type-1 beta-like	3832	0
XM_020100938.1	ubiquitin-protein ligase E3B	5952	0
XM_020100939.1	ubiquitin-protein ligase E3B	5828	0
XM_020100940.1	ubiquitin-protein ligase E3B	5767	0
XM_020100941.1	BTB/POZ domain-containing adapter for CUL3-mediated RhoA degradation protein 2	2033	0
XM_020100942.1	unconventional myosin-Ih-like	3319	0
XM_020100943.1	membrane-associated phosphatidylinositol transfer protein 2-like	6850	0
XM_020100944.1	7-alpha-hydroxycholest-4-en-3-one 12-alpha-hydroxylase-like	2201	0
XM_020100945.1	ADP-ribosylation factor-like protein 6-interacting protein 4	1939	3.78E-60
XM_020100946.1	ubiquitin carboxyl-terminal hydrolase 30 isoform X3	2713	0
XM_020100947.1	ubiquitin carboxyl-terminal hydrolase 30 isoform X3	2612	0
XM_020100948.1	ubiquitin carboxyl-terminal hydrolase 30 isoform X3	2510	0
XM_020100949.1	ubiquitin carboxyl-terminal hydrolase 30 isoform X3	2570	0
XM_020100950.1	synaptic vesicle 2-related protein	3459	0
XM_020100951.1	D-amino-acid oxidase	1416	0

XM_020100952.1	uracil-DNA glycosylase	1355	0
XM_020100953.1	DNA oxidative demethylase ALKBH2	1999	0
XM_020100954.1	DNA oxidative demethylase ALKBH2	1902	0
XM_020100955.1	DNA oxidative demethylase ALKBH2	1881	0
XM_020100956.1	kinetochore-associated protein 1 isoform X1	7128	0
XM_020100957.1	protein Wnt-9a	5044	0
XM_020100958.1	kinetochore-associated protein 1 isoform X2	7123	0
XM_020100959.1	kinetochore-associated protein 1 isoform X3	7125	0
XM_020100960.1	kinetochore-associated protein 1 isoform X4	7120	0
XM_020100961.1	arginine/serine-rich coiled-coil protein 2 isoform X1	1893	5.33E-97
XM_020100962.1	arginine/serine-rich coiled-coil protein 2 isoform X2	1891	7.17E-95
XM_020100963.1	arginine/serine-rich coiled-coil protein 2 isoform X4	1824	5.49E-92
XM_020100964.1	arginine/serine-rich coiled-coil protein 2 isoform X3	1821	1.61E-92
XM_020100965.1	PREDICTED: uncharacterized protein LOC109638136	1551	0
XM_020100966.1	nipped-B-like protein isoform X1	7412	0
XM_020100967.1	nipped-B-like protein isoform X2	7409	0
XM_020100968.1	nipped-B-like protein isoform X3	7409	0
XM_020100969.1	nipped-B-like protein isoform X4	6836	0
XM_020100970.1	nipped-B-like protein isoform X1	1203	2.88E-128
XM_020100971.1	probable G-protein coupled receptor 160	2406	1.04E-176
XM_020100972.1	O(6)-methylguanine-induced apoptosis 2	2156	0
XM_020100973.1	WD repeat-containing protein 31 isoform X1	4430	0
XM_020100974.1	WD repeat-containing protein 31 isoform X2	1547	0
XM_020100975.1	BRI3-binding protein	4283	1.93E-137
XM_020100976.1	proprotein convertase subtilisin/kexin type 5 isoform X1	5354	0
XM_020100977.1	proprotein convertase subtilisin/kexin type 5 isoform X2	5345	0
XM_020100978.1	proprotein convertase subtilisin/kexin type 5 isoform X3	6839	0
XM_020100979.1	probable G-protein coupled receptor 160	2117	1.10E-177
XM_020100980.1	squamous cell carcinoma antigen recognized by T-cells 3	3229	0
XM_020100981.1	adenosine monophosphate-protein transferase FICD	2113	0
XM_020100982.1	ubiquitin-associated protein 2-like isoform X1	4466	0
XM_020100983.1	ubiquitin-associated protein 2-like isoform X2	4362	0
XM_020100984.1	ARF GTPase-activating protein GIT2 isoform X1	4854	0
XM_020100985.1	ARF GTPase-activating protein GIT2 isoform X2	4763	0
XM_020100986.1	ARF GTPase-activating protein GIT2 isoform X3	4750	0
XM_020100987.1	ARF GTPase-activating protein GIT2 isoform X4	4720	0
XM_020100988.1	ARF GTPase-activating protein GIT2 isoform X5	4616	0
XM_020100989.1	ARF GTPase-activating protein GIT2 isoform X6	4610	0
XM_020100990.1	ARF GTPase-activating protein GIT2 isoform X7	4714	0
XM_020100991.1	ARF GTPase-activating protein GIT2 isoform X8	4711	0
XM_020100992.1	trichoplein keratin filament-binding protein isoform X1	1829	0
XM_020100993.1	junctional protein associated with coronary artery disease	5815	0
XM_020100994.1	trichoplein keratin filament-binding protein isoform X2	1760	0
XM_020100995.1	thrombospondin-4-B-like isoform X1	4019	0
XM_020100996.1	thrombospondin-4-B-like isoform X2	3937	0
XM_020100997.1	serine incorporator 5	3179	0
XM_020100998.1	ras GTPase-activating-like protein IQGAP2 isoform X1	5925	0

XM_020100999.1	ras GTPase-activating-like protein IQGAP2 isoform X2	5903	0
XM_020101000.1	proteinase-activated receptor 3-like	2002	0
XM_020101001.1	neurofilament heavy polypeptide isoform X1	2997	0
XM_020101003.1	Fanconi anemia group G protein	2350	0
XM_020101004.1	AP-1 complex subunit beta-1	4389	0
XM_020101005.1	AP-1 complex subunit beta-1	4367	0
XM_020101006.1	angiopoietin-related protein 3-like	2163	0
XM_020101007.1	MOB kinase activator 1A	1779	5.78E-160
XM_020101008.1	bifunctional methylenetetrahydrofolate dehydrogenase/cyclohydrolase, mitochondrial	1691	0
XM_020101009.1	transitional endoplasmic reticulum ATPase	3008	0
XM_020101010.1	alcohol dehydrogenase [NADP(+)] A-like	1987	0
XM_020101011.1	alcohol dehydrogenase [NADP(+)] A-like	1936	0
XM_020101012.1	protein Mpv17	1349	9.89E-130
XM_020101013.1	glutamyl-tRNA(Gln) amidotransferase subunit C, mitochondrial	1592	7.98E-133
XM_020101014.1	polyubiquitin-like isoform X1	1237	3.07E-104
XM_020101016.1	rab5 GDP/GTP exchange factor-like	2420	0
XM_020101017.1	GTP-binding protein REM 2	2611	0
XM_020101018.1	general transcription factor IIH subunit 3	1487	0
XM_020101019.1	26S proteasome non-ATPase regulatory subunit 9	932	3.23E-147
XM_020101020.1	ankyrin repeat domain-containing protein 13A	3884	0
XM_020101021.1	sodium-dependent noradrenaline transporter	1999	0
XM_020101022.1	ectopic P granules protein 5 homolog	8907	0
XM_020101023.1	S-adenosylmethionine synthase isoform X1	3027	0
XM_020101024.1	S-adenosylmethionine synthase isoform X2	1252	0
XM_020101025.1	guanine nucleotide-binding protein G(q) subunit alpha	3790	0
XM_020101026.1	histone-lysine N-methyltransferase ASH1L-like isoform X1	5504	0
XM_020101027.1	histone-lysine N-methyltransferase ASH1L-like isoform X1	8916	0
XM_020101028.1	two pore calcium channel protein 1 isoform X1	4411	0
XM_020101029.1	coiled-coil domain-containing protein 13-like	2050	0
XM_020101030.1	two pore calcium channel protein 1 isoform X2	3046	0
XM_020101031.1	sodium/potassium/calcium exchanger 6, mitochondrial	1982	0
XM_020101032.1	RNA-binding protein EWS isoform X1	2024	1.46E-158
XM_020101033.1	RNA-binding protein EWS isoform X2	2090	8.68E-157
XM_020101034.1	matrix-remodeling-associated protein 5-like	7458	0
XM_020101035.1	RNA-binding protein EWS isoform X3	1994	6.01E-150

XM_020101036.1	endoplasmic reticulum aminopeptidase 1-like isoform X2	5705	0
XM_020101037.1	MLX-interacting protein isoform X1	5255	0
XM_020101038.1	MLX-interacting protein isoform X2	5186	0
XM_020101039.1	synaptic vesicle glycoprotein 2C-like	2641	0
XM_020101040.1	synaptic vesicle glycoprotein 2C-like	2621	0
XM_020101041.1	centrosomal protein POC5	2266	0
XM_020101042.1	centrosomal protein POC5	2199	0
XM_020101043.1	zinc finger CCCH domain-containing protein 13-like	6036	0
XM_020101044.1	ankyrin repeat and death domain-containing protein 1B	2023	0
XM_020101045.1	PREDICTED: transcobalamin-2-like	2166	0
XM_020101046.1	PREDICTED: transcobalamin-2-like	2160	0
XM_020101047.1	nodal homolog	1799	0
XM_020101048.1	deoxycytidine kinase-like isoform X1	1827	0
XM_020101049.1	deoxycytidine kinase-like isoform X2	1858	0
XM_020101050.1	LIM domain kinase 1-like	3536	0
XM_020101051.1	huntingtin-interacting protein 1-related protein	4810	0
XM_020101052.1	kinesin-like protein KIF2A isoform X1	3285	0
XM_020101053.1	kinesin-like protein KIF2A isoform X2	3239	0
XM_020101054.1	kinesin-like protein KIF2A isoform X3	3158	0
XM_020101055.1	kinesin-like protein KIF2A isoform X4	3112	0
XM_020101056.1	calcium-binding mitochondrial carrier protein SCaMC-2-like isoform X1	1895	0
XM_020101057.1	bone morphogenetic protein receptor type-1B isoform X2	4284	0
XM_020101058.1	bone morphogenetic protein receptor type-1B isoform X2	3913	0
XM_020101059.1	bone morphogenetic protein receptor type-1B isoform X2	3854	0
XM_020101060.1	single-stranded DNA-binding protein 2	1515	8.68E-162
XM_020101061.1	transforming acidic coiled-coil-containing protein 1 isoform X1	3919	0
XM_020101062.1	transforming acidic coiled-coil-containing protein 1 isoform X2	3917	0
XM_020101063.1	transforming acidic coiled-coil-containing protein 1 isoform X3	3737	0
XM_020101064.1	transforming acidic coiled-coil-containing protein 1 isoform X4	3570	0
XM_020101065.1	calcium-binding mitochondrial carrier protein SCaMC-2-like isoform X2	1784	0
XM_020101066.1	EMI domain-containing protein 1 isoform X1	2353	0
XM_020101067.1	EMI domain-containing protein 1 isoform X2	2347	0
XM_020101068.1	EMI domain-containing protein 1 isoform X3	2341	0
XM_020101069.1	rhomboid domain-containing protein 3	2763	0
XM_020101070.1	galactosylceramide sulfotransferase-like	2557	0
XM_020101071.1	pescadillo homolog	2258	0
XM_020101072.1	P2X purinoceptor 2	1811	0
XM_020101073.1	DNA repair protein XRCC4-like	4042	0
XM_020101074.1	protein kish-A	898	1.47E-34
XM_020101075.1	N-lysine methyltransferase KMT5A-like	1533	0
XM_020101076.1	RILP-like protein 2	1108	1.25E-139
XM_020101077.1	MORC family CW-type zinc finger protein 2 isoform X1	4440	0
XM_020101078.1	MORC family CW-type zinc finger protein 2 isoform X1	4330	0
XM_020101079.1	MORC family CW-type zinc finger protein 2 isoform X1	4319	0

XM_020101080.1	TRAF-type zinc finger domain-containing protein 1	3321	0
XM_020101081.1	protein FAM222A-like	4565	0
XM_020101082.1	UPF0561 protein C2orf68 homolog	1738	3.34E-65
XM_020101083.1	U4/U6.U5 tri-snRNP-associated protein 2	1836	0
XM_020101084.1	E3 ubiquitin/ISG15 ligase TRIM25-like	1400	0
XM_020101085.1	netrin receptor UNC5C isoform X1	6647	0
XM_020101086.1	netrin receptor UNC5C isoform X2	6590	0
XM_020101087.1	PDZ and LIM domain protein 5 isoform X1	3540	0
XM_020101088.1	PDZ and LIM domain protein 5 isoform X1	3596	0
XM_020101089.1	PDZ and LIM domain protein 5 isoform X1	3522	0
XM_020101090.1	PDZ and LIM domain protein 5 isoform X1	3501	0
XM_020101091.1	PDZ and LIM domain protein 5 isoform X1	3141	0
XM_020101092.1	PDZ and LIM domain protein 5 isoform X5	3123	0
XM_020101093.1	PREDICTED: melanopsin-A-like	2737	0
XM_020101094.1	eosinophil peroxidase-like	3676	0
XM_020101095.1	transcription factor COE2 isoform X1	3974	0
XM_020101096.1	transcription factor COE2 isoform X2	3968	0
XM_020101097.1	transcription factor COE2 isoform X3	3284	0
XM_020101098.1	xenotropic and polytropic retrovirus receptor 1 homolog	3569	0
XM_020101099.1	transcription factor COE2 isoform X4	3278	0
XM_020101100.1	aldehyde dehydrogenase family 3 member B1-like	2138	0
XM_020101101.1	mevalonate kinase	2144	0
XM_020101102.1	cob(I)yrinic acid a,c-diamide adenosyltransferase, mitochondrial	867	8.52E-174
XM_020101103.1	leucine-zipper-like transcriptional regulator 1 isoform X1	3494	0
XM_020101104.1	leucine-zipper-like transcriptional regulator 1 isoform X2	3491	0
XM_020101105.1	zinc finger CCHC domain-containing protein 8	2660	0
XM_020101106.1	hyaluronan and proteoglycan link protein 3	2619	0
XM_020101107.1	phosphatidylinositol 4,5-bisphosphate 5-phosphatase A-like	4134	0
XM_020101108.1	probable RNA-binding protein 19	3072	0
XM_020101109.1	WD repeat-containing protein 78-like	1118	1.24E-156
XM_020101110.1	protein unc-45 homolog B isoform X1	3010	0
XM_020101111.1	protein unc-45 homolog B isoform X2	2929	0
XM_020101112.1	riboflavin kinase	2107	1.38E-102
XM_020101113.1	metaxin-3 isoform X1	3012	0
XM_020101114.1	metaxin-3 isoform X2	2923	2.24E-177
XM_020101115.1	phosphoinositide-3-kinase-interacting protein 1	2490	4.65E-173
XM_020101116.1	polycystin-2 isoform X1	3950	0
XM_020101117.1	polycystin-2 isoform X1	3720	0
XM_020101118.1	polycystin-2 isoform X1	3865	0
XM_020101119.1	polycystin-2 isoform X1	3948	0
XM_020101120.1	methylmalonic aciduria type A protein, mitochondrial	1294	0
XM_020101121.1	choline transporter-like protein 5-A	2226	0
XM_020101122.1	methylmalonic aciduria type A protein, mitochondrial	2281	0
XM_020101123.1	tripartite motif-containing protein 2 isoform X1	3819	0
XM_020101124.1	tripartite motif-containing protein 2 isoform X1	3655	0
XM_020101125.1	tripartite motif-containing protein 2 isoform X1	3706	0
XM_020101126.1	tripartite motif-containing protein 2 isoform X1	3623	0
XM_020101127.1	tripartite motif-containing protein 2 isoform X1	3807	0
XM_020101128.1	voltage-dependent T-type calcium channel subunit alpha-1H-like	6080	0
XM_020101129.1	endothelin-1 receptor	3432	0
XM_020101130.1	endothelin-1 receptor	3271	0
XM_020101131.1	probable E3 ubiquitin-protein ligase HERC3	5206	0
XM_020101132.1	hydroxyacyl-coenzyme A dehydrogenase, mitochondrial	1251	0
XM_020101133.1	reverse transcriptase-like protein	852	0
XM_020101134.1	PRELI domain-containing protein 1, mitochondrial	2040	2.53E-140
XM_020101135.1	prominin-1 isoform X1	3299	0
XM_020101136.1	prominin-1 isoform X1	2948	0
XM_020101137.1	prominin-1 isoform X1	3290	0
XM_020101138.1	prominin-1 isoform X1	3275	0
XM_020101139.1	prominin-1 isoform X4	2947	0
XM_020101140.1	prominin-1 isoform X5	2914	0
XM_020101141.1	prominin-1 isoform X5	2882	0
XM_020101142.1	prominin-1 isoform X6	2830	0
XM_020101143.1	prominin-1 isoform X6	2799	0
XM_020101144.1	max dimerization protein 4	2909	2.80E-108
XM_020101145.1	cyclin-dependent kinase 4 inhibitor B-like	3035	1.94E-79
XM_020101146.1	aminoacyl tRNA synthase complex-interacting multifunctional protein 1-like	2465	0
XM_020101147.1	PREDICTED: LOW QUALITY PROTEIN: uncharacterized protein LOC109638246	720	5.05E-154
XM_020101148.1	ubiquitin carboxyl-terminal hydrolase isozyme L1	1276	5.06E-165
XM_020101149.1	dickkopf-related protein 2 isoform X1	2080	0
XM_020101150.1	dickkopf-related protein 2 isoform X2	2077	0
XM_020101151.1	dickkopf-related protein 2 isoform X3	1123	3.61E-159
XM_020101152.1	synaptogyrin-3-like isoform X1	1622	1.03E-150
XM_020101153.1	synaptogyrin-1-like isoform X2	2177	4.40E-116
XM_020101154.1	ovochymase-2-like isoform X1	4471	0
XM_020101155.1	ovochymase-2-like isoform X2	3686	0
XM_020101156.1	POU domain, class 4, transcription factor 2	2797	0
XM_020101157.1	protein disulfide-isomerase A2-like	1501	0
XM_020101158.1	ecto-NOX disulfide-thiol exchanger 2 isoform X4	2843	0

XM_020101159.1	mitogen-activated protein kinase kinase kinase kinase 4-like	7793	0
XM_020101160.1	PREDICTED: junctophilin-1-like	7424	1.75E-129
XM_020101161.1	E3 ubiquitin-protein ligase RBBP6-like	5552	0
XM_020101162.1	voltage-dependent calcium channel gamma-3 subunit-like	1008	0
XM_020101163.1	E3 ubiquitin-protein ligase RBBP6-like	654	4.78E-164
XM_020101164.1	E3 ubiquitin-protein ligase RBBP6-like	846	6.69E-165
XM_020101165.1	transmembrane protein 192	1348	2.33E-157
XM_020101166.1	glucocorticoid receptor-like	507	3.03E-126
XM_020101167.1	zinc finger protein 330	890	1.25E-135
XM_020101168.1	ATP-binding cassette sub-family G member 2	2569	0
XM_020101169.1	zinc finger protein basonuclin-2	8478	0
XM_020101170.1	dnaJ homolog subfamily B member 14-like	1891	0
XM_020101171.1	TBC domain-containing protein kinase-like protein	3027	0
XM_020101172.1	chromosome transmission fidelity protein 18 homolog	3092	0
XM_020101173.1	protein kinase C beta type isoform X1	4611	0
XM_020101174.1	protein kinase C beta type isoform X2	4282	0
XM_020101175.1	kelch-like protein 2	3610	0
XM_020101176.1	cytosol aminopeptidase	1989	0
XM_020101177.1	carboxypeptidase E	2762	0
XM_020101178.1	methylsterol monooxygenase 1	1220	0
XM_020101179.1	suppressor of cytokine signaling 1-like	1497	0
XM_020101180.1	thymocyte selection-associated high mobility group box protein TOX-like	3316	0
XM_020101181.1	suppressor of cytokine signaling 1-like	1499	0
XM_020101182.1	PREDICTED: clarin-2	1689	2.44E-128
XM_020101183.1	caskin-1-like isoform X2	2628	1.51E-31
XM_020101184.1	mediator of RNA polymerase II transcription subunit 28	791	5.54E-92
XM_020101185.1	E3 ubiquitin-protein ligase RBBP6-like	2428	5.39E-112
XM_020101186.1	guanine nucleotide-binding protein G(I)/G(S)/G(O) subunit gamma-13-like	858	4.01E-42
XM_020101187.1	meiotic nuclear division protein 1 homolog	1972	2.27E-144
XM_020101188.1	S-methyl-5'-thioadenosine phosphorylase	1020	0
XM_020101189.1	protein disulfide-isomerase-like	1863	0
XM_020101190.1	phospholipid phosphatase-related protein type 3-like isoform X2	3215	0
XM_020101191.1	lipopolysaccharide-induced tumor necrosis factor-alpha factor homolog	1782	5.90E-104
XM_020101192.1	lipopolysaccharide-induced tumor necrosis factor-alpha factor homolog	1795	6.78E-104
XM_020101193.1	NADH dehydrogenase [ubiquinone] 1 beta subcomplex subunit 6	555	1.39E-91
XM_020101194.1	arachidonate 15-lipoxygenase B-like	2903	0
XM_020101195.1	F-box only protein 41-like	955	7.79E-111
XM_020101196.1	epidermal growth factor-like protein 6 isoform X2	1598	0
XM_020101197.1	fibroblast growth factor-binding protein 1-like	1604	1.37E-131
XM_020101198.1	G protein-regulated inducer of neurite outgrowth 3-like	2967	0
XM_020101199.1	CXXC-type zinc finger protein 5-like isoform X1	2383	0

XM_020101200.1	CXXC-type zinc finger protein 5-like isoform X1	2145	0
XM_020101201.1	phospholipid phosphatase-related protein type 5-like isoform X1	915	0
XM_020101202.1	fibroblast growth factor-binding protein 2-like	1208	1.70E-105
XM_020101203.1	PREDICTED: uncharacterized protein LOC109638297	1046	6.85E-111
XM_020101204.1	PREDICTED: uncharacterized protein LOC109638297	994	3.72E-111
XM_020101205.1	early growth response protein 1-B-like	2598	0
XM_020101206.1	lipopolysaccharide-responsive and beige-like anchor protein isoform X1	10827	0
XM_020101207.1	lipopolysaccharide-responsive and beige-like anchor protein isoform X2	10824	0
XM_020101208.1	lipopolysaccharide-responsive and beige-like anchor protein isoform X3	10074	0
XM_020101209.1	protein mab-21-like 2	1866	0
XM_020101210.1	40S ribosomal protein S3a	906	0
XM_020101211.1	FRAS1-related extracellular matrix protein 2-like	10372	0
XM_020101212.1	ferric-chelate reductase 1-like	1714	0
XM_020101213.1	SWI/SNF-related matrix-associated actin-dependent regulator of chromatin subfamily A member 5 isoform X1	3705	0
XM_020101214.1	SWI/SNF-related matrix-associated actin-dependent regulator of chromatin subfamily A member 5 isoform X2	3686	0
XM_020101215.1	bifunctional 3'-phosphoadenosine 5'-phosphosulfate synthase 1	3034	0
XM_020101216.1	cytochrome P450 2U1	2983	0
XM_020101217.1	phosphatidylcholine:ceramide cholinephosphotransferase 2	1989	0
XM_020101218.1	phosphatidylcholine:ceramide cholinephosphotransferase 2	1994	0
XM_020101219.1	protein FAM160A1	5811	0
XM_020101220.1	glutamyl-tRNA(Gln) amidotransferase subunit B, mitochondrial	3147	0
XM_020101221.1	dCTP pyrophosphatase 1	1414	9.31E-125
XM_020101222.1	tRNA (adenine(37)-N6)-methyltransferase	1905	0
XM_020101223.1	hemogen isoform X1	2387	2.54E-156
XM_020101224.1	PREDICTED: cadherin-18-like	3171	0
XM_020101225.1	hemogen isoform X2	2374	2.87E-152
XM_020101226.1	acidic leucine-rich nuclear phosphoprotein 32 family member B isoform X2	1709	1.62E-92
XM_020101227.1	acidic leucine-rich nuclear phosphoprotein 32 family member B isoform X2	1553	6.10E-91
XM_020101228.1	acidic leucine-rich nuclear phosphoprotein 32 family member B isoform X2	1408	3.50E-80
XM_020101229.1	acidic leucine-rich nuclear phosphoprotein 32 family member B isoform X2	1548	6.34E-79
XM_020101230.1	acidic leucine-rich nuclear phosphoprotein 32 family member B isoform X2	1768	1.33E-92
XM_020101231.1	ras GTPase-activating protein-binding protein 2 isoform X1	4678	0
XM_020101232.1	ras GTPase-activating protein-binding protein 2 isoform X1	4697	0
XM_020101233.1	ras GTPase-activating protein-binding protein 2 isoform X1	4668	0
XM_020101234.1	ras GTPase-activating protein-binding protein 2 isoform X1	4619	0
XM_020101235.1	ras GTPase-activating protein-binding protein 2 isoform X4	4609	0
XM_020101236.1	F-box/WD repeat-containing protein 7 isoform X1	5159	0
XM_020101237.1	F-box/WD repeat-containing protein 7 isoform X2	1898	0

XM_020101238.1	serine/threonine-protein kinase pim-1-like	516	2.02E-111
XM_020101239.1	F-box/WD repeat-containing protein 7 isoform X3	1995	0
XM_020101240.1	calcium uniporter regulatory subunit MCUb, mitochondrial-like	1729	0
XM_020101241.1	caspase 3	2948	0
XM_020101242.1	GRB2-associated-binding protein 1 isoform X1	4406	0
XM_020101243.1	GRB2-associated-binding protein 1 isoform X2	4360	0
XM_020101244.1	GRB2-associated-binding protein 1 isoform X3	4313	0
XM_020101245.1	GRB2-associated-binding protein 1 isoform X4	4267	0
XM_020101246.1	GRB2-associated-binding protein 1 isoform X1	3988	0
XM_020101247.1	arginine N-methyltransferase 9	3868	0
XM_020101248.1	transmembrane protein 184C	2945	0
XM_020101249.1	protein FAM193A isoform X1	5990	0
XM_020101250.1	protein FAM193A isoform X2	5978	0
XM_020101251.1	carbonic anhydrase-related protein	1004	0
XM_020101252.1	protein FAM193A isoform X3	5941	0
XM_020101253.1	protein FAM193A isoform X4	5673	0
XM_020101254.1	LIM and calponin homology domains-containing protein 1-like isoform X1	6927	0
XM_020101255.1	LIM and calponin homology domains-containing protein 1-like isoform X2	6924	0
XM_020101256.1	LIM and calponin homology domains-containing protein 1-like isoform X3	6813	0
XM_020101257.1	LIM and calponin homology domains-containing protein 1-like isoform X4	6669	0
XM_020101258.1	LIM and calponin homology domains-containing protein 1-like isoform X5	6408	0
XM_020101259.1	LIM and calponin homology domains-containing protein 1-like isoform X6	5889	0
XM_020101260.1	rho guanine nucleotide exchange factor 38	2523	0
XM_020101261.1	integrator complex subunit 12	1790	1.12E-152
XM_020101262.1	inorganic pyrophosphatase 2, mitochondrial	1261	0
XM_020101263.1	TBC1 domain family member 9 isoform X1	6108	0
XM_020101264.1	TBC1 domain family member 9 isoform X2	6040	0
XM_020101265.1	chromodomain-helicase-DNA-binding protein 7-like	3475	0
XM_020101266.1	E3 ubiquitin-protein ligase MARCH8-like	1302	0
XM_020101267.1	transmembrane protein 154 isoform X1	1891	3.02E-90
XM_020101268.1	transmembrane protein 154 isoform X1	1910	3.50E-90
XM_020101269.1	transmembrane protein 154 isoform X1	1848	1.66E-83
XM_020101270.1	transmembrane protein 154 isoform X1	1837	1.40E-75
XM_020101271.1	translation machinery-associated protein 16	1615	9.07E-101
XM_020101272.1	general vesicular transport factor p115 isoform X1	3965	0
XM_020101273.1	general vesicular transport factor p115 isoform X2	3953	0
XM_020101274.1	general vesicular transport factor p115 isoform X3	3944	0
XM_020101275.1	general vesicular transport factor p115 isoform X4	3911	0
XM_020101276.1	programmed cell death protein 4-like	2876	0
XM_020101277.1	beta-1,3-galactosyltransferase 2-like	834	0
XM_020101278.1	protein odr-4 homolog	1086	0
XM_020101279.1	transmembrane protein 131-like isoform X1	4720	0
XM_020101280.1	transmembrane protein 131-like isoform X2	4717	0
XM_020101281.1	PREDICTED: protocadherin-23	10661	0

XM_020101282.1	secreted frizzled-related protein 2	2556	0
XM_020101283.1	arfaptin-1 isoform X1	4305	0
XM_020101284.1	arfaptin-1 isoform X1	3933	0
XM_020101285.1	RING finger protein 150 isoform X1	7085	0
XM_020101286.1	RING finger protein 150 isoform X2	7079	0
XM_020101287.1	mitochondrial uncoupling protein 2-like	1686	0
XM_020101288.1	mitochondrial uncoupling protein 2-like	1658	0
XM_020101289.1	ELMO domain-containing protein 2	1828	0
XM_020101290.1	mineralocorticoid receptor	2998	0
XM_020101291.1	mineralocorticoid receptor	3080	0
XM_020101292.1	mineralocorticoid receptor	2806	0
XM_020101293.1	mineralocorticoid receptor	2888	0
XM_020101294.1	mineralocorticoid receptor	2766	0
XM_020101295.1	methylcytosine dioxygenase TET3 isoform X3	8560	0
XM_020101296.1	zinc finger protein 827 isoform X3	4261	0
XM_020101297.1	zinc finger protein 827 isoform X3	4254	0
XM_020101298.1	zinc finger protein 827 isoform X3	8348	0
XM_020101299.1	kinesin-associated protein 3	2218	0
XM_020101300.1	aminoacyl tRNA synthase complex-interacting multifunctional protein 1	1193	2.08E-176
XM_020101301.1	group XIIA secretory phospholipase A2	1808	1.73E-124
XM_020101302.1	sperm-associated antigen 5	5343	0
XM_020101303.1	forkhead box protein E1	1487	0
XM_020101304.1	protein FAM13A isoform X1	3657	0
XM_020101305.1	protein FAM13A isoform X2	3503	0
XM_020101306.1	ubiquitin carboxyl-terminal hydrolase 38	4351	0
XM_020101307.1	serine/threonine-protein kinase DCLK2 isoform X1	3324	0
XM_020101308.1	serine/threonine-protein kinase DCLK2 isoform X2	3321	0
XM_020101309.1	serine/threonine-protein kinase DCLK2 isoform X3	3298	0
XM_020101310.1	serine/threonine-protein kinase DCLK2 isoform X4	3295	0
XM_020101311.1	potassium voltage-gated channel subfamily H member 6-like	5566	0
XM_020101312.1	serine/threonine-protein kinase DCLK2 isoform X5	3273	0
XM_020101313.1	serine/threonine-protein kinase DCLK2 isoform X6	3270	0
XM_020101314.1	serine/threonine-protein kinase DCLK2 isoform X7	3247	0
XM_020101315.1	serine/threonine-protein kinase DCLK2 isoform X8	3244	0
XM_020101316.1	serine/threonine-protein kinase DCLK2 isoform X9	2687	0

XM_020101317.1	serine/threonine-protein kinase DCLK2 isoform X10	2923	0
XM_020101318.1	mannosyl-oligosaccharide glucosidase	2806	0
XM_020101319.1	mannosyl-oligosaccharide glucosidase	3577	0
XM_020101320.1	lymphoid enhancer-binding factor 1 isoform X1	2893	0
XM_020101321.1	lymphoid enhancer-binding factor 1 isoform X2	2887	0
XM_020101322.1	lymphoid enhancer-binding factor 1 isoform X3	2884	0
XM_020101323.1	hedgehog-interacting protein isoform X1	5052	0
XM_020101324.1	hedgehog-interacting protein isoform X2	4630	0
XM_020101325.1	hedgehog-interacting protein isoform X3	4328	0
XM_020101326.1	PREDICTED: myosin-1-like	3340	0
XM_020101327.1	coronin-2B isoform X1	3250	0
XM_020101328.1	coronin-2B isoform X1	3281	0
XM_020101329.1	type II inositol 3,4-bisphosphate 4-phosphatase isoform X1	3415	0
XM_020101330.1	type II inositol 3,4-bisphosphate 4-phosphatase isoform X1	3502	0
XM_020101331.1	type II inositol 3,4-bisphosphate 4-phosphatase isoform X1	3471	0
XM_020101332.1	type II inositol 3,4-bisphosphate 4-phosphatase isoform X1	3368	0
XM_020101333.1	type II inositol 3,4-bisphosphate 4-phosphatase isoform X1	3281	0
XM_020101334.1	type II inositol 3,4-bisphosphate 4-phosphatase isoform X1	3298	0
XM_020101335.1	type II inositol 3,4-bisphosphate 4-phosphatase isoform X2	4095	0
XM_020101336.1	type II inositol 3,4-bisphosphate 4-phosphatase isoform X1	3364	0
XM_020101337.1	type II inositol 3,4-bisphosphate 4-phosphatase isoform X1	3831	0
XM_020101338.1	type II inositol 3,4-bisphosphate 4-phosphatase isoform X1	2887	0
XM_020101339.1	type II inositol 3,4-bisphosphate 4-phosphatase isoform X6	4044	0
XM_020101340.1	E3 ubiquitin-protein ligase Topors	3164	0
XM_020101341.1	rho GTPase-activating protein 10	3558	0
XM_020101342.1	PC4 and SFRS1-interacting protein isoform X1	2340	0
XM_020101343.1	PC4 and SFRS1-interacting protein isoform X1	2348	0
XM_020101344.1	PC4 and SFRS1-interacting protein isoform X1	2341	0
XM_020101345.1	PC4 and SFRS1-interacting protein isoform X1	2226	0
XM_020101346.1	snRNA-activating protein complex subunit 3	1336	0
XM_020101347.1	glutathione S-transferase C-terminal domain-containing protein isoform X1	4683	0
XM_020101348.1	glutathione S-transferase C-terminal domain-containing protein isoform X2	1730	0
XM_020101349.1	nephronectin isoform X1	4137	0
XM_020101350.1	nephronectin isoform X2	4084	0
XM_020101351.1	tetratricopeptide repeat protein 39B	2847	0
XM_020101352.1	PREDICTED: myosin-7-like	4784	0
XM_020101353.1	PREDICTED: centlein	5286	0
XM_020101354.1	mothers against decapentaplegic homolog 1	3054	0
XM_020101355.1	protein phosphatase 1K, mitochondrial	3266	0
XM_020101356.1	endophilin-A1 isoform X1	3681	0
XM_020101357.1	endophilin-A1 isoform X2	3654	0
XM_020101358.1	endophilin-A1 isoform X3	3642	0
XM_020101359.1	transmembrane anterior posterior transformation protein 1 homolog	3874	0
XM_020101360.1	annexin A10	1408	0
XM_020101361.1	proline-, glutamic acid- and leucine-rich protein 1-like isoform X1	6719	0
XM_020101362.1	WW domain binding protein 1-like isoform X2	6294	0
XM_020101363.1	SH3 domain-containing protein 19 isoform X1	3211	0
XM_020101364.1	astrotactin-2 isoform X1	7262	0
XM_020101365.1	SH3 domain-containing protein 19 isoform X2	3208	0
XM_020101366.1	polypyrimidine tract-binding protein 2-like isoform X1	1889	0
XM_020101367.1	polypyrimidine tract-binding protein 2-like isoform X2	1878	0
XM_020101368.1	ras suppressor protein 1 isoform X2	1337	0
XM_020101369.1	ras suppressor protein 1 isoform X2	1443	0
XM_020101370.1	ras suppressor protein 1 isoform X2	1336	0
XM_020101371.1	cyclic nucleotide-gated cation channel beta-1-like isoform X3	4984	0
XM_020101372.1	PREDICTED: palmdelphin	2304	0
XM_020101373.1	zinc finger SWIM domain-containing protein 5	7606	0
XM_020101374.1	serine/threonine-protein kinase 17A-like	4716	0
XM_020101375.1	serine/threonine-protein kinase 17A-like	4474	0
XM_020101376.1	E3 ubiquitin-protein ligase HECW1	5507	0
XM_020101377.1	V-set and transmembrane domain-containing protein 2-like protein	4246	6.13E-112
XM_020101378.1	tRNA (cytosine(38)-C(5))-methyltransferase	821	0
XM_020101379.1	bone morphogenetic protein 1-like isoform X2	3312	0
XM_020101380.1	putative ferric-chelate reductase 1	2552	0
XM_020101381.1	proteasome subunit beta type-5-like	1282	0
XM_020101382.1	PREDICTED: uncharacterized protein LOC109638404	5405	0
XM_020101383.1	zinc finger CCHC domain-containing protein 7-like isoform X1	2003	3.13E-175
XM_020101384.1	reversion-inducing cysteine-rich protein with Kazal motifs	7222	0
XM_020101385.1	PREDICTED: uncharacterized protein LOC109638407	919	5.75E-176
XM_020101386.1	mitochondrial import inner membrane translocase subunit TIM50-like	808	2.14E-72
XM_020101387.1	CAP-Gly domain-containing linker protein 3-like	685	6.48E-62
XM_020101388.1	transcriptional regulator ATRX-like	4048	0
XM_020101389.1	collagen alpha-1(XXI) chain-like	3530	0
XM_020101390.1	zinc finger CCHC domain-containing protein 2-like	2119	0
XM_020101391.1	TBC1 domain family member 1-like isoform X1	5505	0
XM_020101392.1	interferon regulatory factor 2	894	0
XM_020101393.1	proteasome subunit beta type-11-like	1017	0
XM_020101394.1	TBC1 domain family member 1-like isoform X2	5502	0
XM_020101395.1	TBC1 domain family member 1-like isoform X3	5271	0

XM_020101396.1	TBC1 domain family member 1-like isoform X4	4963	0
XM_020101397.1	phospholipid phosphatase-related protein type 3-like isoform X2	3126	0
XM_020101398.1	glucose 1,6-bisphosphate synthase isoform X2	2803	0
XM_020101399.1	sorting nexin-7	2807	0
XM_020101400.1	phospholipid phosphatase-related protein type 5-like isoform X1	3566	0
XM_020101401.1	phospholipid phosphatase-related protein type 5-like isoform X1	3504	0
XM_020101402.1	phospholipid phosphatase-related protein type 5-like isoform X1	3284	0
XM_020101403.1	phospholipid phosphatase-related protein type 5-like isoform X1	3377	6.71E-163
XM_020101404.1	C-C chemokine receptor type 3-like	2030	0
XM_020101405.1	E3 ubiquitin-protein ligase TRIM39-like	3511	0
XM_020101406.1	PREDICTED: enkurin-like	1552	0
XM_020101407.1	metalloreductase STEAP3	1917	0
XM_020101408.1	metalloreductase STEAP3	3416	0
XM_020101409.1	zinc finger protein PLAGL2-like	1425	0
XM_020101410.1	proto-oncogene serine/threonine-protein kinase mos	1731	0
XM_020101411.1	UDP-N-acetylglucosamine transferase subunit ALG14 homolog	938	1.13E-157
XM_020101412.1	coiled-coil-helix-coiled-coil-helix domain-containing protein 7	845	8.15E-58
XM_020101413.1	ATP-binding cassette sub-family D member 2-like	3119	0
XM_020101414.1	solute carrier family 22 member 13-like	1382	0
XM_020101415.1	PREDICTED: LOW QUALITY PROTEIN: calponin-3-like	2063	0
XM_020101416.1	mitochondrial import inner membrane translocase subunit TIM50	2348	2.28E-146
XM_020101417.1	fibroblast growth factor 20-like	2086	4.39E-156
XM_020101418.1	TGF-beta receptor type-1-like	9570	0
XM_020101419.1	lanC-like protein 1	4523	0
XM_020101420.1	vesicular, overexpressed in cancer, prosurvival protein 1	3188	5.33E-101
XM_020101421.1	cytochrome c-type heme lyase	2483	0
XM_020101422.1	PREDICTED: parathymosin-like	1367	1.78E-27
XM_020101423.1	solute carrier family 22 member 13-like	938	0
XM_020101424.1	ras and EF-hand domain-containing protein homolog	2526	0
XM_020101425.1	ras and EF-hand domain-containing protein homolog	2523	0
XM_020101426.1	probable tRNA pseudouridine synthase 2	1568	0
XM_020101427.1	probable tRNA pseudouridine synthase 2	1175	0
XM_020101428.1	protein ABHD14A	1732	0
XM_020101429.1	tumor suppressor candidate 2	1307	1.43E-61
XM_020101430.1	transmembrane protein 51-like	2317	1.41E-121
XM_020101431.1	transmembrane protein 51-like	2177	3.99E-122
XM_020101432.1	transmembrane protein 43	2010	0
XM_020101433.1	hyaluronidase-1-like isoform X1	2002	0
XM_020101434.1	hyaluronidase-1-like isoform X1	1892	0
XM_020101435.1	PREDICTED: uncharacterized protein CXorf38 homolog	819	1.98E-139
XM_020101436.1	N-fatty-acyl-amino acid synthase/hydrolase PM20D1	1862	0
XM_020101437.1	N-fatty-acyl-amino acid synthase/hydrolase PM20D1	1917	0
XM_020101438.1	taste receptor type 1 member 3	2552	0
XM_020101439.1	coiled-coil domain-containing protein 114-like	3180	0
XM_020101440.1	coiled-coil domain-containing protein 114-like	3084	0
XM_020101441.1	coiled-coil domain-containing protein 114-like	3042	0
XM_020101442.1	testis-expressed sequence 264 protein-like	2390	0
XM_020101443.1	PREDICTED: uncharacterized protein LOC109638455	1953	0
XM_020101444.1	proton-associated sugar transporter A isoform X1	4551	0
XM_020101445.1	proton-associated sugar transporter A isoform X2	3324	0
XM_020101446.1	proton-associated sugar transporter A isoform X3	3118	0
XM_020101447.1	migration and invasion-inhibitory protein isoform X1	1919	0
XM_020101448.1	endothelin B receptor-like	1707	0
XM_020101449.1	migration and invasion-inhibitory protein isoform X2	1907	0
XM_020101450.1	epigen-like isoform X1	2961	6.05E-118
XM_020101451.1	epigen-like isoform X2	2925	1.42E-110
XM_020101452.1	PAK4-inhibitor inka2-like	1241	0
XM_020101453.1	voltage-dependent calcium channel subunit alpha-2/delta-3	4377	0
XM_020101454.1	PREDICTED: uncharacterized protein LOC109638462 isoform X1	1416	0
XM_020101455.1	PREDICTED: uncharacterized protein LOC109638462 isoform X2	1365	0
XM_020101456.1	ubiquitin-like modifier-activating enzyme 1	3550	0
XM_020101457.1	PREDICTED: ninjurin-1-like	1471	5.22E-74
XM_020101458.1	membrane metallo-endopeptidase-like 1	2677	0
XM_020101459.1	dentin sialophosphoprotein-like isoform X1	3939	0
XM_020101460.1	unconventional myosin-VIIa-like	7121	0
XM_020101461.1	dentin sialophosphoprotein-like isoform X2	3167	0
XM_020101462.1	PREDICTED: caveolin-2-like	1176	3.39E-131
XM_020101463.1	solute carrier family 2, facilitated glucose transporter member 11-like	1545	0
XM_020101464.1	PREDICTED: mucin-3B-like, partial	1099	0
XM_020101465.1	E3 ubiquitin-protein ligase TRIM62-like	3368	0
XM_020101466.1	kazrin isoform X5	4365	0
XM_020101467.1	ski oncogene-like	3070	0
XM_020101468.1	1-phosphatidylinositol 4,5-bisphosphate phosphodiesterase eta-2-like	3603	0
XM_020101469.1	disks large homolog 2-like	450	1.64E-110
XM_020101470.1	tumor necrosis factor receptor superfamily member 4-like	863	1.49E-160
XM_020101471.1	homeobox protein Mohawk-like	1218	0
XM_020101472.1	tumor necrosis factor receptor superfamily member 3-like	696	1.28E-155
XM_020101473.1	green-sensitive opsin	954	0
XM_020101474.1	green-sensitive opsin-4-like	408	5.72E-96

XM_020101475.1	PREDICTED: uncharacterized protein C3orf67 homolog	3028	0
XM_020101476.1	retinol dehydrogenase 10-like	948	0
XM_020101477.1	somatomedin-B and thrombospondin type-1 domain-containing protein-like	827	0
XM_020101478.1	myosin heavy chain, fast skeletal muscle-like	5828	0
XM_020101479.1	ras association domain-containing protein 1-like	927	4.06E-119
XM_020101480.1	rab GDP dissociation inhibitor beta-like	1361	0
XM_020101481.1	cAMP-dependent protein kinase type II-alpha regulatory subunit	2683	0
XM_020101482.1	teleost multiple tissue opsin-3a	741	6.33E-156
XM_020101483.1	PREDICTED: rootletin	5066	0
XM_020101484.1	somatostatin receptor type 2-like	1005	0
XM_020101485.1	tachykinin-like peptides receptor 86C	999	0
XM_020101486.1	von Willebrand factor A domain-containing protein 1-like	1627	0
XM_020101487.1	protein bassoon-like	15539	0
XM_020101488.1	serine/threonine-protein kinase WNK2-like isoform X1	6880	0
XM_020101489.1	serine/threonine-protein kinase WNK2-like isoform X2	6877	0
XM_020101490.1	serine/threonine-protein kinase WNK2-like isoform X3	6769	0
XM_020101491.1	helicase ARIP4 isoform X1	7329	0
XM_020101492.1	helicase ARIP4 isoform X1	4805	0
XM_020101493.1	helicase ARIP4 isoform X1	7238	0
XM_020101494.1	dynamin-2 isoform X1	4340	0
XM_020101495.1	ERC protein 2 isoform X1	6157	0
XM_020101496.1	ERC protein 2 isoform X2	6713	0
XM_020101497.1	ERC protein 2 isoform X3	6689	0
XM_020101498.1	inter-alpha-trypsin inhibitor heavy chain H3-like isoform X1	2845	0
XM_020101499.1	inter-alpha-trypsin inhibitor heavy chain H3-like isoform X2	2812	0
XM_020101500.1	inter-alpha-trypsin inhibitor heavy chain H3-like isoform X3	2776	0
XM_020101501.1	inter-alpha-trypsin inhibitor heavy chain H3-like isoform X4	2743	0
XM_020101502.1	ubiquitin carboxyl-terminal hydrolase BAP1 isoform X1	3250	0
XM_020101503.1	ubiquitin carboxyl-terminal hydrolase BAP1 isoform X2	3247	0
XM_020101504.1	PREDICTED: uncharacterized protein LOC109638502	3282	0
XM_020101505.1	PREDICTED: uncharacterized protein LOC109638502	3218	0
XM_020101506.1	cysteine-rich with EGF-like domain protein 1 isoform X1	1743	9.61E-175
XM_020101507.1	cysteine-rich with EGF-like domain protein 1 isoform X2	1761	3.74E-174
XM_020101508.1	kelch-like protein 10	2020	0
XM_020101509.1	low-density lipoprotein receptor-related protein 8-like	2659	3.13E-77
XM_020101510.1	cysteine-rich with EGF-like domain protein 1 isoform X1	1901	4.13E-172
XM_020101511.1	cytochrome b561 domain-containing protein 1	2099	8.33E-132
XM_020101512.1	cytochrome b561 domain-containing protein 1	2012	3.56E-132
XM_020101513.1	myosin heavy chain, fast skeletal muscle-like	5902	0
XM_020101514.1	myosin heavy chain, fast skeletal muscle-like	5877	0
XM_020101515.1	myosin heavy chain, fast skeletal muscle-like	5903	0
XM_020101516.1	myosin heavy chain, fast skeletal muscle-like	5883	0
XM_020101517.1	voltage-dependent calcium channel subunit alpha-2/delta-2-like	7672	0
XM_020101518.1	nitrogen permease regulator 2-like protein isoform X1	1740	0
XM_020101519.1	membrane protein FAM159A-like	1002	4.90E-167
XM_020101521.1	glutathione peroxidase 1-like	727	4.86E-103
XM_020101522.1	protein BRICK1	836	5.50E-77
XM_020101523.1	tumor suppressor candidate 2	1183	1.30E-64
XM_020101524.1	tumor suppressor candidate 2	1209	1.67E-64
XM_020101525.1	tumor suppressor candidate 2	1145	8.96E-65
XM_020101526.1	protein FAM83D-like isoform X1	2953	0
XM_020101527.1	protein FAM83D-like isoform X1	2873	0
XM_020101528.1	protein FAM83D-like isoform X1	2812	0
XM_020101529.1	protein FAM83D-like isoform X1	2950	0
XM_020101530.1	excitatory amino acid transporter 5-like	1632	0
XM_020101531.1	filamin-binding LIM protein 1	2351	0
XM_020101532.1	formin-like protein 7	1926	0
XM_020101533.1	haloacid dehalogenase-like hydrolase domain-containing protein 3	1185	0
XM_020101534.1	calcium/calmodulin-dependent protein kinase type 1-like	1925	0
XM_020101535.1	protein FAM3A	1188	3.28E-156
XM_020101536.1	NADH dehydrogenase [ubiquinone] 1 alpha subcomplex assembly factor 3	1042	8.24E-154
XM_020101537.1	isotocin receptor-like	1574	0
XM_020101538.1	prostamide/prostaglandin F synthase	1050	1.21E-126
XM_020101539.1	protein B4-like	976	1.13E-117
XM_020101540.1	regulator of G-protein signaling 21-like	540	2.71E-134
XM_020101541.1	transmembrane and coiled-coil domains protein 1-like	1172	1.12E-176
XM_020101542.1	transketolase-like isoform X1	1947	0
XM_020101543.1	transketolase-like isoform X2	1833	0
XM_020101544.1	zinc finger MYND domain-containing protein 10	1976	0
XM_020101545.1	green-sensitive opsin-like	1190	0
XM_020101546.1	green-sensitive opsin-like	1038	0
XM_020101547.1	zinc finger protein 362-like isoform X1	2654	0
XM_020101548.1	zinc finger protein 362-like isoform X2	2621	0
XM_020101549.1	zinc finger protein 362-like isoform X2	2600	0
XM_020101550.1	prostacyclin synthase-like	1682	0
XM_020101551.1	PREDICTED: uncharacterized protein C3orf18 homolog isoform X1	1533	7.01E-70
XM_020101552.1	regulator of G-protein signaling 2-like	695	1.17E-126
XM_020101553.1	PREDICTED: uncharacterized protein C3orf18 homolog isoform X1	914	1.73E-72
XM_020101554.1	PREDICTED: uncharacterized protein C3orf18 homolog isoform X2	1568	5.03E-51

XM_020101555.1	potassium voltage-gated channel subfamily B member 1-like	2898	0
XM_020101556.1	caM kinase-like vesicle-associated protein	2463	0
XM_020101557.1	cytokine-inducible SH2-containing protein	2180	1.80E-138
XM_020101558.1	transcription factor 7-like 1-A isoform X1	1758	0
XM_020101559.1	transcription factor 7-like 1-A isoform X2	1704	0
XM_020101560.1	tumor necrosis factor receptor superfamily member 1B-like isoform X1	1975	0
XM_020101561.1	tumor necrosis factor receptor superfamily member 1B-like isoform X2	1756	0
XM_020101562.1	calmodulin-like protein 6	769	1.44E-111
XM_020101563.1	ephrin type-B receptor 3-like	2042	0
XM_020101564.1	synaptoporin isoform X2	1330	0
XM_020101565.1	probable G-protein coupled receptor	1701	0
XM_020101566.1	vertebrate ancient opsin-like	1020	0
XM_020101567.1	voltage-dependent calcium channel subunit alpha-2/delta-2 isoform X1	7871	0
XM_020101568.1	voltage-dependent calcium channel subunit alpha-2/delta-2 isoform X2	7826	0
XM_020101569.1	hepatocyte growth factor-like protein	4607	0
XM_020101570.1	protein ABHD14B isoform X1	1125	5.10E-154
XM_020101571.1	protein ABHD14B isoform X1	989	4.65E-144
XM_020101572.1	high mobility group protein HMGI-C-like	2727	1.54E-14
XM_020101573.1	high mobility group protein HMGI-C-like	2683	3.43E-14
XM_020101574.1	msx2-interacting protein-like isoform X1	10900	0
XM_020101575.1	major facilitator superfamily domain-containing protein 8-like	1514	0
XM_020101576.1	msx2-interacting protein-like isoform X2	10898	0
XM_020101577.1	green-sensitive opsin-like	1059	0
XM_020101578.1	glutamine-rich protein 1-like	3731	0
XM_020101579.1	glutamine-rich protein 1-like	3655	0
XM_020101580.1	RNA pseudouridylate synthase domain-containing protein 4-like	1714	0
XM_020101581.1	twinfilin-2-like isoform X1	1428	0
XM_020101582.1	WD repeat-containing protein 82-like isoform X2	1397	0
XM_020101583.1	U3 small nucleolar RNA-associated protein 15 homolog isoform X3	2177	0
XM_020101584.1	protein SFI1 homolog	4875	0
XM_020101585.1	protein SFI1 homolog	4602	0
XM_020101586.1	eukaryotic translation initiation factor 4E transporter	4009	0
XM_020101587.1	eukaryotic translation initiation factor 4E transporter	4052	0
XM_020101588.1	sodium- and chloride-dependent betaine transporter-like	483	1.78E-117
XM_020101589.1	serotransferrin precursor	1797	0
XM_020101590.1	guanine nucleotide-binding protein G(I)/G(S)/G(T) subunit beta-1-like	3267	0
XM_020101591.1	guanine nucleotide-binding protein G(I)/G(S)/G(T) subunit beta-1-like	3372	0
XM_020101592.1	guanine nucleotide-binding protein G(I)/G(S)/G(T) subunit beta-1-like	1665	0
XM_020101593.1	guanine nucleotide-binding protein G(I)/G(S)/G(T) subunit beta-1-like	3026	0
XM_020101594.1	semaphorin-3F-like isoform X1	4769	0
XM_020101595.1	semaphorin-3F-like isoform X2	4752	0
XM_020101596.1	semaphorin-3F-like isoform X3	4681	0
XM_020101597.1	semaphorin-3F-like isoform X4	4664	0
XM_020101598.1	guanine nucleotide-binding protein G(t) subunit alpha-1	1242	0
XM_020101599.1	F-box only protein 42	3113	0
XM_020101600.1	SUZ domain-containing protein 1-like	2781	3.62E-93
XM_020101601.1	PREDICTED: uncharacterized protein LOC109638563	2789	0
XM_020101602.1	protein bicaudal D homolog 2-like	4155	0
XM_020101603.1	caspase recruitment domain-containing protein 19-like	2393	1.68E-123
XM_020101604.1	TAR DNA-binding protein 43-like isoform X1	3056	0
XM_020101605.1	TAR DNA-binding protein 43-like isoform X2	3036	0
XM_020101606.1	centromere protein S-like	801	3.55E-111
XM_020101607.1	PR domain zinc finger protein 2	6897	0
XM_020101608.1	protein polybromo-1 isoform X1	5206	0
XM_020101609.1	protein polybromo-1 isoform X2	5194	0
XM_020101610.1	protein polybromo-1 isoform X3	5109	0

XM_020101611.1	receptor-type tyrosine-protein phosphatase gamma-like	6907	0
XM_020101612.1	procollagen galactosyltransferase 2-like	2727	0
XM_020101613.1	flocculation protein FLO11-like isoform X1	2781	0
XM_020101614.1	protein DDI1 homolog 2-like isoform X2	1758	0
XM_020101615.1	sodium- and chloride-dependent GABA transporter 2-like isoform X1	3367	0
XM_020101616.1	sodium- and chloride-dependent GABA transporter 2-like isoform X2	3320	0
XM_020101617.1	ephrin type-A receptor 2-like	3816	0
XM_020101618.1	POZ-, AT hook-, and zinc finger-containing protein 1 isoform X1	6027	0
XM_020101619.1	POZ-, AT hook-, and zinc finger-containing protein 1 isoform X2	2192	0
XM_020101620.1	POZ-, AT hook-, and zinc finger-containing protein 1 isoform X3	6399	0
XM_020101621.1	solute carrier family 2, facilitated glucose transporter member 11-like	1814	0
XM_020101622.1	arginine-glutamic acid dipeptide repeats protein-like isoform X1	4698	0
XM_020101623.1	arginine-glutamic acid dipeptide repeats protein-like isoform X2	4692	0
XM_020101624.1	arginine-glutamic acid dipeptide repeats protein-like isoform X3	4782	0
XM_020101625.1	zinc finger BED domain-containing protein 1-like	1734	0
XM_020101626.1	transcription initiation factor IIB	1276	0
XM_020101627.1	arginine-glutamic acid dipeptide repeats protein-like isoform X4	4704	0
XM_020101628.1	arginine-glutamic acid dipeptide repeats protein-like isoform X5	4776	0
XM_020101629.1	glucose-6-phosphate 1-dehydrogenase isoform X2	3702	0
XM_020101630.1	retinoid-binding protein 7	937	4.05E-98
XM_020101631.1	sodium-coupled neutral amino acid transporter 3-like isoform X2	5019	0
XM_020101632.1	sodium-coupled neutral amino acid transporter 3-like isoform X2	4998	0
XM_020101633.1	voltage-dependent L-type calcium channel subunit alpha-1D-like	9247	0

XM_020101634.1	transforming protein RhoA	1285	2.40E-141
XM_020101635.1	transforming protein RhoA	1341	8.57E-141
XM_020101636.1	double-stranded RNA-binding protein Staufen homolog 1 isoform X1	3101	0
XM_020101637.1	double-stranded RNA-binding protein Staufen homolog 1 isoform X2	3089	0
XM_020101638.1	putative RNA polymerase II subunit B1 CTD phosphatase RPAP2	2013	0
XM_020101639.1	RNA-binding protein 6-like isoform X1	4441	0
XM_020101640.1	RNA-binding protein 6-like isoform X2	4439	0
XM_020101641.1	metabotropic glutamate receptor 2-like isoform X1	4216	0
XM_020101642.1	metabotropic glutamate receptor 2-like isoform X2	4165	0
XM_020101643.1	metabotropic glutamate receptor 2-like isoform X3	4023	0
XM_020101644.1	U3 small nucleolar RNA-interacting protein 2 isoform X1	1620	0
XM_020101645.1	U3 small nucleolar RNA-interacting protein 2 isoform X2	1607	0
XM_020101646.1	PREDICTED: uncharacterized protein LOC109638593	1478	0
XM_020101647.1	PREDICTED: uncharacterized protein LOC109638593	1289	0
XM_020101648.1	SLIT-ROBO Rho GTPase-activating protein 3-like isoform X1	5447	0
XM_020101649.1	SLIT-ROBO Rho GTPase-activating protein 3-like isoform X2	5854	0
XM_020101650.1	SLIT-ROBO Rho GTPase-activating protein 3-like isoform X3	5809	0
XM_020101651.1	plasmalemma vesicle-associated protein-like	1522	0
XM_020101652.1	sodium- and chloride-dependent GABA transporter 2-like	1826	0
XM_020101653.1	peptidyl-prolyl cis-trans isomerase-like 1 isoform X1	730	1.04E-112
XM_020101654.1	peptidyl-prolyl cis-trans isomerase-like 1 isoform X2	727	2.89E-111
XM_020101655.1	bromodomain and PHD finger-containing protein 3-like isoform X1	4911	0
XM_020101656.1	bromodomain and PHD finger-containing protein 3-like isoform X2	4887	0
XM_020101657.1	bromodomain and PHD finger-containing protein 3-like isoform X3	3614	0
XM_020101658.1	glutamate receptor U1-like	2042	0
XM_020101659.1	coiled-coil domain-containing protein 30-like	2045	0
XM_020101660.1	N-acetyllactosaminide alpha-1,3-galactosyltransferase-like isoform X1	1447	0
XM_020101661.1	PREDICTED: calponin-2-like	1152	0
XM_020101662.1	N-acetyllactosaminide alpha-1,3-galactosyltransferase-like isoform X1	1537	0
XM_020101663.1	N-acetyllactosaminide alpha-1,3-galactosyltransferase-like isoform X2	1443	0
XM_020101664.1	N-acetyllactosaminide alpha-1,3-galactosyltransferase-like isoform X2	1533	0
XM_020101665.1	epithelial membrane protein 3-like	1087	6.69E-102
XM_020101666.1	epithelial membrane protein 3-like	1123	1.01E-101
XM_020101667.1	kelch repeat and BTB domain-containing protein 12	2728	0
XM_020101668.1	PREDICTED: uncharacterized protein LOC109638608	1075	4.94E-177
XM_020101669.1	PREDICTED: uncharacterized protein C1orf159 homolog	3513	1.77E-112
XM_020101670.1	glycerate kinase	2186	0
XM_020101671.1	glycerate kinase	2067	0
XM_020101672.1	phosphatidylinositol 3-kinase regulatory subunit beta-like	5400	0
XM_020101673.1	glycerate kinase	2164	0
XM_020101674.1	T-cell leukemia translocation-altered gene protein	1197	6.55E-69
XM_020101675.1	macrophage-stimulating protein receptor-like isoform X1	5186	0
XM_020101676.1	macrophage-stimulating protein receptor-like isoform X2	4949	0
XM_020101677.1	ras association domain-containing protein 1 isoform X1	2613	0
XM_020101678.1	ras association domain-containing protein 1 isoform X2	2692	0
XM_020101679.1	RNA-binding protein 5-like	3135	0
XM_020101680.1	solute carrier family 25 member 34	2187	0
XM_020101681.1	solute carrier family 25 member 34	1974	0
XM_020101682.1	transmembrane protein 82-like	1707	0
XM_020101683.1	hemK methyltransferase family member 1	1331	0
XM_020101684.1	nicotinamide/nicotinic acid mononucleotide adenylyltransferase 1	1966	0
XM_020101685.1	cytokine-inducible SH2-containing protein	2491	8.80E-120
XM_020101686.1	hyaluronidase-2-like isoform X1	2446	0
XM_020101687.1	adaptin ear-binding coat-associated protein 2-like	3988	8.83E-154
XM_020101688.1	solute carrier family 26 member 6-like	2725	0
XM_020101689.1	transmembrane prolyl 4-hydroxylase-like	3002	0
XM_020101690.1	something about silencing protein 10	1704	0
XM_020101691.1	phosphopantothenate--cysteine ligase	1118	0
XM_020101692.1	forkhead box protein J3-like isoform X1	6588	0
XM_020101693.1	rho guanine nucleotide exchange factor 15-like isoform X1	2397	0
XM_020101694.1	REM2- and Rab-like small GTPase 1	1749	0
XM_020101695.1	mitogen-activated protein kinase kinase kinase 13-A-like	2306	0
XM_020101696.1	heat shock protein beta-7-like	904	2.90E-78
XM_020101697.1	transmembrane and coiled-coil domains protein 1 isoform X1	3472	0
XM_020101698.1	transmembrane and coiled-coil domains protein 1 isoform X1	2492	0
XM_020101699.1	ER degradation-enhancing alpha-mannosidase-like protein 3 isoform X1	3654	0
XM_020101700.1	hyaluronidase-2-like isoform X1	4630	0
XM_020101701.1	hyaluronidase-2-like isoform X1	1884	0
XM_020101702.1	hyaluronidase-2-like isoform X1	1875	0
XM_020101703.1	hyaluronidase-2-like isoform X1	4631	0
XM_020101704.1	guanine nucleotide-binding protein G(i) subunit alpha-2	2512	0
XM_020101705.1	ERBB receptor feedback inhibitor 1	3562	0
XM_020101706.1	vacuolar fusion protein MON1 homolog B	2997	0
XM_020101707.1	dynein heavy chain 11, axonemal-like	13197	0
XM_020101708.1	twinfilin-2-like isoform X1	2150	0
XM_020101709.1	WD repeat-containing protein 82	1287	0
XM_020101710.1	calcium-dependent secretion activator 1 isoform X1	5059	0
XM_020101711.1	calcium-dependent secretion activator 1 isoform X2	4802	0
XM_020101712.1	calcium-dependent secretion activator 1 isoform X3	5047	0

XM_020101713.1	calcium-dependent secretion activator 1 isoform X4	5047	0
XM_020101714.1	calcium-dependent secretion activator 1 isoform X5	5023	0
XM_020101715.1	calcium-dependent secretion activator 1 isoform X6	5020	0
XM_020101716.1	calcium-dependent secretion activator 1 isoform X7	5017	0
XM_020101717.1	calcium-dependent secretion activator 1 isoform X8	5010	0
XM_020101718.1	calcium-dependent secretion activator 1 isoform X9	4751	0
XM_020101719.1	calcium-dependent secretion activator 1 isoform X10	4990	0
XM_020101720.1	calcium-dependent secretion activator 1 isoform X11	4981	0
XM_020101721.1	calcium-dependent secretion activator 1 isoform X12	4977	0
XM_020101722.1	PREDICTED: semaphorin-6B-like	5491	0
XM_020101723.1	calcium-dependent secretion activator 1 isoform X13	4978	0
XM_020101724.1	calcium-dependent secretion activator 1 isoform X14	4948	0
XM_020101725.1	calcium-dependent secretion activator 1 isoform X15	4898	0
XM_020101726.1	calcium-dependent secretion activator 1 isoform X16	4844	0
XM_020101727.1	calcium-dependent secretion activator 1 isoform X17	4787	0
XM_020101728.1	calcium-dependent secretion activator 1 isoform X18	4775	0
XM_020101729.1	calcium-dependent secretion activator 1 isoform X19	5011	0
XM_020101730.1	calcium-dependent secretion activator 1 isoform X20	4969	0
XM_020101731.1	protein kinase C and casein kinase substrate in neurons protein 2-like	3901	0
XM_020101732.1	alpha-enolase isoform X2	1691	0
XM_020101733.1	ADP-ribosylation factor-like protein 8B-A isoform X1	1571	1.15E-134
XM_020101734.1	ADP-ribosylation factor-like protein 8B-A isoform X2	1432	7.98E-107
XM_020101735.1	cytochrome b-c1 complex subunit 2, mitochondrial-like	1727	0
XM_020101736.1	platelet-derived growth factor subunit A	1765	3.96E-122
XM_020101737.1	testis-expressed sequence 2 protein-like isoform X1	3647	0
XM_020101738.1	testis-expressed sequence 2 protein-like isoform X2	3644	0
XM_020101739.1	60S ribosomal protein L3-like	1482	0
XM_020101740.1	zinc finger protein 574 isoform X2	1430	0
XM_020101741.1	methionine-R-sulfoxide reductase B1-A-like	1124	2.05E-110
XM_020101742.1	arf-GAP with dual PH domain-containing protein 1-like	2410	0
XM_020101743.1	cytosolic Fe-S cluster assembly factor NARFL	1793	0
XM_020101744.1	hydroxyacylglutathione hydrolase-like protein	1470	0
XM_020101745.1	hydroxyacylglutathione hydrolase-like protein	1506	0
XM_020101746.1	PREDICTED: uncharacterized protein LOC109638636	993	3.96E-170
XM_020101747.1	junctional sarcoplasmic reticulum protein 1	1576	2.41E-146
XM_020101748.1	centromere protein X isoform X1	1725	1.54E-93
XM_020101749.1	centromere protein X isoform X2	1721	2.39E-89
XM_020101750.1	centromere protein X isoform X3	1480	2.54E-98
XM_020101751.1	cAMP-dependent protein kinase type I-beta regulatory subunit	2253	0
XM_020101752.1	E3 SUMO-protein ligase CBX4-like	3155	0
XM_020101753.1	guanine nucleotide-binding protein G(o) subunit alpha-like	1371	0
XM_020101754.1	neuropeptide B-like	1048	1.12E-87
XM_020101755.1	NADPH oxidase organizer 1	2210	0
XM_020101756.1	chromobox protein homolog 8-like	2116	0
XM_020101757.1	extracellular serine/threonine protein kinase FAM20C-like	3948	0
XM_020101758.1	fizzy-related protein homolog	2644	0
XM_020101759.1	small integral membrane protein 10-like protein 1	1382	3.82E-38
XM_020101760.1	palmitoleoyl-protein carboxylesterase NOTUM	1782	0
XM_020101761.1	myeloid-associated differentiation marker-like protein 2	1916	0
XM_020101762.1	centriolar coiled-coil protein of 110 kDa	3776	0
XM_020101763.1	centriolar coiled-coil protein of 110 kDa	3736	0
XM_020101764.1	transmembrane channel-like protein 6 isoform X1	2818	0
XM_020101765.1	transmembrane channel-like protein 6 isoform X2	2765	0
XM_020101766.1	PREDICTED: uncharacterized protein LOC109638675	2281	0
XM_020101767.1	cytochrome P450 2K1-like	1382	0
XM_020101768.1	heat shock protein 75 kDa, mitochondrial-like	1395	0
XM_020101769.1	dynein assembly factor 5, axonemal	2454	0
XM_020101770.1	IQ domain-containing protein K	1530	2.09E-180
XM_020101771.1	putative short-chain dehydrogenase/reductase family 42E member 2	1579	0
XM_020101772.1	protein kinase C alpha type-like	548	4.22E-99
XM_020101773.1	sterile alpha motif domain-containing protein 9-like	5380	0
XM_020101774.1	TBC1 domain family member 16	5043	0
XM_020101775.1	rho GTPase-activating protein 17-like	3601	0
XM_020101776.1	sodium/myo-inositol cotransporter 2-like	1970	0
XM_020101777.1	fibronectin type III domain-containing protein 7-like	7739	0
XM_020101778.1	RING finger protein 151	878	1.42E-176
XM_020101779.1	platelet-activating factor acetylhydrolase IB subunit alpha	3080	0
XM_020101780.1	sterol regulatory element-binding protein 1-like	3106	0
XM_020101781.1	ras-related protein Rab-26-like	1321	1.19E-161
XM_020101782.1	ras-related protein Rab-26-like	1419	3.78E-161
XM_020101783.1	meteorin-like protein	1633	0
XM_020101784.1	somatostatin receptor type 2-like	2716	0
XM_020101785.1	somatostatin receptor type 2-like	2773	0
XM_020101786.1	somatostatin receptor type 2-like	2561	0
XM_020101787.1	ethanolamine-phosphate cytidylyltransferase-like isoform X1	1791	0
XM_020101788.1	ethanolamine-phosphate cytidylyltransferase-like isoform X1	1776	0
XM_020101789.1	fibronectin type III domain-containing protein 7-like	591	7.12E-108
XM_020101790.1	PREDICTED: aquaporin-8-like	1041	0
XM_020101791.1	Usher syndrome type-1G protein isoform X1	1903	0

XM_020101792.1	mediator of RNA polymerase II transcription subunit 9	657	5.55E-64
XM_020101793.1	disheveled-associated activator of morphogenesis 1-like isoform X2	7423	0
XM_020101794.1	PREDICTED: axin-2-like	1344	0
XM_020101795.1	heparan sulfate glucosamine 3-O-sulfotransferase 6	1343	0
XM_020101796.1	voltage-dependent calcium channel gamma-5 subunit-like	1932	0
XM_020101797.1	heterogeneous nuclear ribonucleoproteins A1 homolog	852	9.33E-97
XM_020101798.1	dexamethasone-induced Ras-related protein 1-like	1213	0
XM_020101799.1	dynein regulatory complex subunit 3	1873	0
XM_020101800.1	fibronectin type III domain-containing protein 7-like	2607	0
XM_020101801.1	methionine-R-sulfoxide reductase B1-A-like	606	6.86E-81
XM_020101802.1	decapping and exoribonuclease protein	2425	0
XM_020101803.1	decapping and exoribonuclease protein	2272	0
XM_020101804.1	decapping and exoribonuclease protein	2309	0
XM_020101805.1	peroxisomal membrane protein PMP34-like	3284	0
XM_020101806.1	serine/threonine-protein kinase 19 isoform X1	1289	0
XM_020101807.1	serine/threonine-protein kinase 19 isoform X2	1135	0
XM_020101808.1	ras-related C3 botulinum toxin substrate 1-like	2470	2.06E-134
XM_020101809.1	ras-related C3 botulinum toxin substrate 1-like	1784	2.64E-137
XM_020101810.1	ATP-binding cassette sub-family A member 3	7705	0
XM_020101811.1	cytochrome P450 2K1-like	1909	0
XM_020101812.1	transmembrane protease serine 2	2829	0
XM_020101813.1	heme oxygenase 2 isoform X2	3180	0
XM_020101814.1	heme oxygenase 2 isoform X2	2943	0
XM_020101815.1	heme oxygenase 2 isoform X2	3161	0
XM_020101816.1	heme oxygenase 2 isoform X2	2924	0
XM_020101817.1	heme oxygenase 2 isoform X2	3156	0
XM_020101818.1	heme oxygenase 2 isoform X2	2918	0
XM_020101819.1	leucine-rich repeat-containing protein 4-like	4118	0
XM_020101820.1	dnaJ homolog subfamily A member 3, mitochondrial	2434	0
XM_020101821.1	coronin-7-like isoform X1	1405	0
XM_020101822.1	coronin-7-like isoform X2	1251	0
XM_020101823.1	CREB-binding protein-like isoform X1	8514	0
XM_020101824.1	CREB-binding protein-like isoform X2	8291	0
XM_020101825.1	CREB-binding protein-like isoform X3	7425	0
XM_020101826.1	IgGFc-binding protein-like	6072	0
XM_020101827.1	adenylate cyclase type 9-like	9141	0
XM_020101828.1	E3 ubiquitin-protein ligase RNF213	15400	0
XM_020101829.1	SUN domain-containing protein 1 isoform X1	4424	0
XM_020101830.1	SUN domain-containing protein 1 isoform X1	4522	0
XM_020101831.1	SUN domain-containing protein 1 isoform X3	4233	0
XM_020101832.1	SUN domain-containing protein 1 isoform X4	4375	0
XM_020101833.1	SUN domain-containing protein 1 isoform X5	4313	0
XM_020101834.1	SUN domain-containing protein 1 isoform X6	4294	0
XM_020101835.1	SUN domain-containing protein 1 isoform X3	4284	0
XM_020101836.1	SUN domain-containing protein 1 isoform X4	4362	0
XM_020101837.1	SUN domain-containing protein 1 isoform X4	4339	0
XM_020101838.1	SUN domain-containing protein 1 isoform X8	4183	0
XM_020101839.1	SUN domain-containing protein 1 isoform X9	4098	0
XM_020101840.1	cullin-3 isoform X1	386	2.00E-88
XM_020101841.1	lysosomal alpha-glucosidase-like isoform X1	4188	0
XM_020101842.1	galectin-3-binding protein	2104	0
XM_020101843.1	soluble calcium-activated nucleotidase 1 isoform X2	1931	0
XM_020101844.1	soluble calcium-activated nucleotidase 1 isoform X2	1960	0
XM_020101845.1	soluble calcium-activated nucleotidase 1 isoform X2	1938	0
XM_020101846.1	hydroxyacylglutathione hydrolase-like protein	1660	0
XM_020101847.1	fumarylacetoacetate hydrolase domain-containing protein 2-like isoform X6	1354	2.43E-163
XM_020101848.1	serine/threonine-protein kinase/endoribonuclease IRE1	4304	0
XM_020101849.1	transcription factor Sox-9-A-like	3161	0
XM_020101850.1	cullin-3 isoform X1	1773	0
XM_020101851.1	ubiquitin carboxyl-terminal hydrolase 22 isoform X1	3607	0
XM_020101852.1	ubiquitin carboxyl-terminal hydrolase 22 isoform X2	3574	0
XM_020101853.1	ubiquitin carboxyl-terminal hydrolase 22 isoform X2	3106	0
XM_020101854.1	transcription factor AP-4-like isoform X1	4035	0
XM_020101855.1	transcription factor AP-4-like isoform X2	4014	0
XM_020101856.1	TOM1-like protein 2 isoform X1	4117	0
XM_020101857.1	TOM1-like protein 2 isoform X2	4042	0
XM_020101858.1	TOM1-like protein 2 isoform X3	3982	0
XM_020101859.1	PREDICTED: axin-2-like	4191	0
XM_020101860.1	40S ribosomal protein S2	988	2.32E-177
XM_020101861.1	NADH dehydrogenase [ubiquinone] 1 beta subcomplex subunit 10	656	4.50E-129
XM_020101862.1	skin secretory protein xP2-like	2593	1.06E-32
XM_020101863.1	fibroblast growth factor 12-like	1380	6.66E-116
XM_020101864.1	protein kinase C alpha type-like isoform X1	3486	0
XM_020101865.1	protein kinase C alpha type-like isoform X2	3460	0
XM_020101866.1	PREDICTED: uncharacterized protein LOC109638742	1029	0
XM_020101867.1	pyrroline-5-carboxylate reductase 1, mitochondrial-like	2157	0
XM_020101868.1	pyrroline-5-carboxylate reductase 1, mitochondrial-like	2115	0
XM_020101869.1	transcription factor MafG	2743	7.28E-94
XM_020101870.1	transcription factor MafG	2762	1.10E-93

XM_020101871.1	sarcalumenin-like isoform X1	3844	0
XM_020101872.1	sarcalumenin-like isoform X2	3808	0
XM_020101873.1	PREDICTED: uncharacterized protein LOC109638747	2235	0
XM_020101874.1	RNA-binding protein with serine-rich domain 1 isoform X1	1101	9.09E-91
XM_020101875.1	RNA-binding protein with serine-rich domain 1 isoform X1	923	7.26E-91
XM_020101876.1	interleukin-12 subunit alpha-like	597	2.46E-134
XM_020101877.1	transcription factor Sox-8-like	3383	0
XM_020101878.1	G2/mitotic-specific cyclin-B1	3402	0
XM_020101879.1	PREDICTED: uncharacterized protein C16orf52 homolog B	5030	1.87E-107
XM_020101880.1	cytohesin-1 isoform X1	3836	0
XM_020101881.1	testis-expressed sequence 2 protein-like	4861	0
XM_020101882.1	glucose-induced degradation protein 4 homolog	2730	4.66E-150
XM_020101883.1	oocyte zinc finger protein XlCOF6.1-like isoform X1	3090	0
XM_020101884.1	oocyte zinc finger protein XlCOF6.1-like isoform X1	3087	0
XM_020101885.1	centrosomal protein of 112 kDa isoform X1	3275	0
XM_020101886.1	centrosomal protein of 112 kDa isoform X1	3452	0
XM_020101887.1	centrosomal protein of 112 kDa isoform X1	2804	0
XM_020101888.1	selenoprotein T	1121	3.63E-121
XM_020101889.1	synaptogyrin-1 isoform X1	2007	2.69E-129
XM_020101890.1	kelch-like protein 20	2934	0
XM_020101891.1	ATP-dependent DNA helicase Q4 isoform X1	5184	0
XM_020101892.1	ATP-dependent DNA helicase Q4 isoform X2	5181	0
XM_020101893.1	cleft lip and palate transmembrane protein 1-like protein	2508	0
XM_020101894.1	NADH dehydrogenase [ubiquinone] flavoprotein 1, mitochondrial-like isoform X1	1677	0
XM_020101895.1	NADH dehydrogenase [ubiquinone] flavoprotein 1, mitochondrial-like isoform X1	1658	0
XM_020101896.1	tRNA-dihydrouridine(47) synthase [NAD(P)(+)]-like isoform X1	2228	0
XM_020101897.1	tRNA-dihydrouridine(47) synthase [NAD(P)(+)]-like isoform X2	2222	0
XM_020101898.1	grainyhead-like protein 2 homolog	2868	0
XM_020101899.1	protein patched homolog 1-like	2046	0
XM_020101900.1	cathepsin S-like	1165	0
XM_020101901.1	splicing factor 3B subunit 4	1412	2.40E-168
XM_020101902.1	vacuolar protein sorting-associated protein 72 homolog	1825	0
XM_020101903.1	biogenesis of lysosome-related organelles complex 1 subunit 4-like	1766	1.64E-152
XM_020101904.1	synaptic vesicle glycoprotein 2A-like	2571	0
XM_020101905.1	ribonucleoside-diphosphate reductase subunit M2 B isoform X1	1572	0
XM_020101906.1	ribonucleoside-diphosphate reductase subunit M2 B isoform X1	1624	0
XM_020101907.1	FERM domain-containing protein 8	2743	0
XM_020101908.1	60S acidic ribosomal protein P1	564	2.39E-40
XM_020101909.1	G-protein coupled receptor 20-like	1422	0
XM_020101910.1	tenascin-like isoform X3	6699	0
XM_020101911.1	zinc finger protein 217	4981	0
XM_020101912.1	lysosomal-associated transmembrane protein 4B-like	2913	1.81E-141
XM_020101913.1	ubiquitin-associated protein 2 isoform X3	3890	0
XM_020101914.1	potassium/sodium hyperpolarization-activated cyclic nucleotide-gated channel 2-like	1860	0
XM_020101915.1	histone-lysine N-methyltransferase 2A-like	7818	0
XM_020101916.1	40S ribosomal protein S19-like	1201	7.92E-82
XM_020101917.1	brevican core protein-like	4478	0
XM_020101918.1	lanosterol 14-alpha demethylase	656	6.88E-108
XM_020101919.1	cytosolic non-specific dipeptidase-like	1326	0
XM_020101920.1	zinc transporter ZIP4	2935	0
XM_020101921.1	gastrula zinc finger protein XlCGF8.2DB-like isoform X3	7008	0
XM_020101922.1	cilia- and flagella-associated protein 20	709	4.46E-135
XM_020101923.1	DENN domain-containing protein 3-like	4358	0
XM_020101924.1	relaxin-3 receptor 1-like	1179	0
XM_020101925.1	ras/Rap GTPase-activating protein SynGAP	5148	0
XM_020101926.1	partitioning defective 6 homolog gamma-like	1728	8.75E-178
XM_020101927.1	protein Jumonji-like	1334	2.05E-117
XM_020101928.1	39S ribosomal protein L24, mitochondrial	1044	5.81E-156
XM_020101929.1	PREDICTED: uncharacterized protein LOC109638797 isoform X1	1723	0
XM_020101930.1	PREDICTED: uncharacterized protein LOC109638797 isoform X1	1413	0
XM_020101931.1	PREDICTED: uncharacterized protein LOC109638797 isoform X1	1400	0
XM_020101932.1	espin-like protein	2025	0
XM_020101933.1	PREDICTED: uncharacterized protein LOC109638798	1121	2.20E-169
XM_020101934.1	PREDICTED: uncharacterized protein LOC109638798	1114	2.04E-169
XM_020101935.1	zona pellucida sperm-binding protein 4-like	1826	0
XM_020101936.1	cathepsin L1-like	1199	0
XM_020101937.1	protein S100-A1-like isoform X2	1085	5.76E-73
XM_020101938.1	protein S100-A1-like isoform X2	1020	2.75E-68

XM_020101939.1	protein S100-A1-like isoform X2	1083	8.03E-70
XM_020101940.1	ecto-NOX disulfide-thiol exchanger 2 isoform X1	2925	0
XM_020101941.1	nuclear transport factor 2-like	710	5.52E-95
XM_020101942.1	transcription termination factor 2, mitochondrial	1629	0
XM_020101943.1	HCLS1-associated protein X-1 isoform X1	1255	2.73E-172
XM_020101944.1	HCLS1-associated protein X-1 isoform X1	1203	2.06E-155
XM_020101945.1	chloride channel protein 2-like	1585	0
XM_020101946.1	IgGFc-binding protein-like	5624	0
XM_020101947.1	zinc finger protein 687 isoform X1	4437	0
XM_020101948.1	zinc finger protein 687 isoform X1	4705	0
XM_020101949.1	zinc finger protein 687 isoform X1	4330	0

XM_020101950.1	zinc finger protein 687 isoform X1	4350	0
XM_020101951.1	zinc finger protein 687 isoform X1	4621	0
XM_020101952.1	zinc finger protein 687 isoform X1	4765	0
XM_020101953.1	phosphatidylinositol 4-phosphate 5-kinase type-1 alpha-like isoform X1	4743	0
XM_020101954.1	phosphatidylinositol 4-phosphate 5-kinase type-1 alpha-like isoform X1	4680	0
XM_020101955.1	26S proteasome non-ATPase regulatory subunit 4	1400	0
XM_020101956.1	PREDICTED: aquaporin-10-like	1269	0
XM_020101957.1	extracellular matrix protein 1-like	1157	7.75E-130
XM_020101958.1	solute carrier family 12 member 9-like	3285	0
XM_020101959.1	leucine-rich repeat and immunoglobulin-like domain-containing nogo receptor-interacting protein 1	2977	0
XM_020101960.1	leucine-rich repeat and immunoglobulin-like domain-containing nogo receptor-interacting protein 1	3067	0
XM_020101961.1	DNA-directed RNA polymerases I, II, and III subunit RPABC4	346	1.78E-37
XM_020101962.1	transcriptional repressor scratch 1-like	2296	0
XM_020101963.1	PI-PLC X domain-containing protein 3	1342	0
XM_020101964.1	A-kinase anchor protein 9 isoform X1	14990	0
XM_020101965.1	A-kinase anchor protein 9 isoform X2	14978	0
XM_020101966.1	A-kinase anchor protein 9 isoform X3	14951	0
XM_020101967.1	histamine H3 receptor-like	1167	0
XM_020101968.1	A-kinase anchor protein 9 isoform X4	14930	0
XM_020101969.1	A-kinase anchor protein 9 isoform X5	14903	0
XM_020101970.1	serine/threonine-protein kinase 3	4198	0
XM_020101971.1	classical arabinogalactan protein 11-like	1155	1.21E-38
XM_020101972.1	ribonuclease UK114-like isoform X1	715	4.79E-94
XM_020101973.1	ribonuclease UK114-like isoform X2	785	4.82E-94
XM_020101974.1	60S ribosomal protein L30	526	1.17E-82
XM_020101975.1	14-3-3 protein eta-like	2281	4.65E-160
XM_020101976.1	zinc finger protein 706-like	1249	2.73E-46
XM_020101977.1	zinc finger protein 706-like	1164	1.42E-46
XM_020101978.1	myocyte-specific enhancer factor 2D isoform X1	6837	0
XM_020101979.1	UPF0602 protein C4orf47 homolog	939	0
XM_020101980.1	cadherin-4-like isoform X3	4092	0
XM_020101981.1	myocyte-specific enhancer factor 2D isoform X1	6868	0
XM_020101982.1	myocyte-specific enhancer factor 2D isoform X1	6844	0
XM_020101983.1	myocyte-specific enhancer factor 2D isoform X1	6729	0
XM_020101984.1	myocyte-specific enhancer factor 2D isoform X1	6671	0
XM_020101985.1	myocyte-specific enhancer factor 2D isoform X1	6786	0
XM_020101986.1	myocyte-specific enhancer factor 2D isoform X1	6717	0
XM_020101987.1	transmembrane protein 79-like	3242	0
XM_020101988.1	zinc finger protein 696-like	1602	0
XM_020101989.1	focal adhesion kinase 1 isoform X2	5461	0
XM_020101990.1	focal adhesion kinase 1 isoform X2	5539	0
XM_020101991.1	focal adhesion kinase 1 isoform X2	5449	0
XM_020101992.1	focal adhesion kinase 1 isoform X2	5431	0
XM_020101993.1	cadherin-4-like isoform X3	5325	0
XM_020101994.1	focal adhesion kinase 1 isoform X2	5731	0
XM_020101995.1	focal adhesion kinase 1 isoform X2	5422	0
XM_020101996.1	protein S100-A16-like	4329	1.50E-60
XM_020101997.1	protein S100-A1-like	538	2.41E-53
XM_020101998.1	E3 ubiquitin-protein ligase RNF19A-like	5131	0
XM_020101999.1	solute carrier family 45 member 4	8761	0
XM_020102000.1	polyadenylate-binding protein 1	2784	0
XM_020102001.1	pygopus homolog 2-like	4584	3.09E-60
XM_020102002.1	teashirt homolog 1-like isoform X1	5094	0
XM_020102003.1	teashirt homolog 1-like isoform X1	4980	0
XM_020102004.1	teashirt homolog 1-like isoform X1	4957	0
XM_020102005.1	meprin A subunit beta-like	1297	0
XM_020102006.1	teashirt homolog 1-like isoform X1	5592	0
XM_020102007.1	proline-rich protein PRCC	2807	0
XM_020102008.1	39S ribosomal protein L9, mitochondrial	1295	9.47E-159
XM_020102009.1	tyrosine-protein phosphatase non-receptor type 2-like isoform X1	3300	0
XM_020102010.1	tyrosine-protein phosphatase non-receptor type 2-like isoform X1	3177	0
XM_020102011.1	proteasome assembly chaperone 2	1216	0
XM_020102012.1	cytosolic non-specific dipeptidase-like	1600	0
XM_020102013.1	cytosolic non-specific dipeptidase-like	1973	0
XM_020102014.1	neurocalcin-delta A	946	4.61E-144
XM_020102015.1	neurocalcin-delta A	944	5.23E-144
XM_020102016.1	pre-B-cell leukemia transcription factor-interacting protein 1-like isoform X1	4961	0
XM_020102017.1	pre-B-cell leukemia transcription factor-interacting protein 1-like isoform X1	4730	0
XM_020102018.1	adhesion G protein-coupled receptor L2-like	3425	0
XM_020102019.1	pre-B-cell leukemia transcription factor-interacting protein 1-like isoform X1	4936	0
XM_020102020.1	pre-B-cell leukemia transcription factor-interacting protein 1-like isoform X1	4909	0
XM_020102021.1	pre-B-cell leukemia transcription factor-interacting protein 1-like isoform X4	4884	0
XM_020102022.1	cingulin-like protein 1	5324	0
XM_020102023.1	zinc finger transcription factor Trps1 isoform X1	11238	0
XM_020102024.1	zinc finger transcription factor Trps1 isoform X1	10999	0
XM_020102025.1	zinc finger transcription factor Trps1 isoform X1	11002	0
XM_020102026.1	zinc finger transcription factor Trps1 isoform X1	10752	0
XM_020102027.1	cyclin-dependent kinases regulatory subunit 1	2207	4.10E-37
XM_020102028.1	protein LYRIC isoform X1	4011	0

XM_020102029.1	protein LYRIC isoform X2	4007	0
XM_020102030.1	TBC1 domain family member 20-like	2958	0
XM_020102031.1	dystrobrevin alpha-like	3378	0
XM_020102032.1	LIX1-like protein	3658	0
XM_020102033.1	hepatoma-derived growth factor	2846	4.40E-149
XM_020102034.1	trypsinogen 1	824	8.63E-172
XM_020102035.1	sperm-associated antigen 1A-like	3252	0
XM_020102036.1	CUB and sushi domain-containing protein 3-like	13995	0
XM_020102037.1	origin recognition complex subunit 4-like	3037	0
XM_020102038.1	origin recognition complex subunit 4-like	3032	0
XM_020102039.1	tuftelin-like isoform X3	2337	0
XM_020102040.1	tuftelin-like isoform X3	2238	0
XM_020102041.1	tuftelin-like isoform X3	2278	0
XM_020102042.1	tuftelin-like isoform X3	2179	0
XM_020102043.1	autophagy-related protein 9A-like	3623	0
XM_020102044.1	PREDICTED: uncharacterized protein C1orf43 homolog	1695	4.57E-175
XM_020102045.1	SH2 domain-containing adapter protein E	3037	0
XM_020102046.1	type-4 ice-structuring protein	751	1.36E-74
XM_020102047.1	diacylglycerol O-acyltransferase 1	1786	0
XM_020102048.1	cilia- and flagella-associated protein 20-like isoform X1	916	2.21E-144
XM_020102049.1	cilia- and flagella-associated protein 20-like isoform X2	904	2.11E-141
XM_020102050.1	protein sprouty homolog 2-like	2420	0
XM_020102051.1	protein sprouty homolog 2-like	2424	0
XM_020102052.1	putative transferase CAF17, mitochondrial	956	0
XM_020102053.1	interleukin-1 receptor accessory protein-like 1-B isoform X1	5038	0
XM_020102054.1	interleukin-1 receptor accessory protein-like 1 isoform X2	2429	0
XM_020102055.1	SLIT and NTRK-like protein 1	2928	0
XM_020102056.1	hemK methyltransferase family member 2	1173	1.64E-158
XM_020102057.1	FERM and PDZ domain-containing protein 4-like isoform X1	6862	0
XM_020102058.1	FERM and PDZ domain-containing protein 4-like isoform X1	6315	0
XM_020102059.1	neural cell adhesion molecule 2 isoform X1	5368	0
XM_020102060.1	neural cell adhesion molecule 2 isoform X2	5365	0
XM_020102061.1	neural cell adhesion molecule 2 isoform X3	5409	0
XM_020102062.1	SLIT and NTRK-like protein 6	4010	0
XM_020102063.1	PREDICTED: transmembrane inner ear expressed protein	942	4.73E-87
XM_020102064.1	SLIT and NTRK-like protein 5	3988	0
XM_020102065.1	NF-kappa-B inhibitor zeta-like	2068	0
XM_020102066.1	coiled-coil domain-containing protein 122	1112	0
XM_020102067.1	FERM, RhoGEF and pleckstrin domain-containing protein 1-like isoform X1	3833	0
XM_020102068.1	FERM, RhoGEF and pleckstrin domain-containing protein 1-like isoform X2	3831	0
XM_020102069.1	FERM, RhoGEF and pleckstrin domain-containing protein 1-like isoform X3	3809	0
XM_020102070.1	ADP-ribosylation factor-like protein 13B isoform X1	2715	0
XM_020102071.1	parathyroid hormone/parathyroid hormone-related peptide receptor	4917	0
XM_020102072.1	ADP-ribosylation factor-like protein 13B isoform X2	2631	0
XM_020102073.1	ADP-ribosylation factor-like protein 13B isoform X3	2580	0
XM_020102074.1	ADP-ribosylation factor-like protein 13B isoform X4	2511	0
XM_020102075.1	PREDICTED: gamma-glutamylaminecyclotransferase	1373	1.69E-97
XM_020102076.1	PREDICTED: gamma-glutamylaminecyclotransferase	1156	2.74E-97
XM_020102077.1	PREDICTED: uncharacterized protein LOC109638895	1702	0
XM_020102078.1	tubulin alpha chain-like	1362	0
XM_020102079.1	NEDD4 family-interacting protein 2	3946	8.02E-107
XM_020102080.1	rho GTPase-activating protein 6 isoform X2	4639	0
XM_020102081.1	rho GTPase-activating protein 6 isoform X2	4300	0
XM_020102082.1	rho GTPase-activating protein 6 isoform X2	4189	0
XM_020102083.1	E3 ubiquitin-protein ligase Midline-1-like	4209	0
XM_020102084.1	corticotropin-releasing factor receptor 2-like	3133	0
XM_020102085.1	beta-chimaerin isoform X2	1778	0
XM_020102086.1	beta-chimaerin isoform X2	1746	0
XM_020102087.1	TSC22 domain family protein 1-like isoform X1	4228	0
XM_020102088.1	TSC22 domain family protein 1-like isoform X2	1713	4.64E-84
XM_020102089.1	peptide chain release factor 1, mitochondrial	1872	0
XM_020102090.1	WW domain-binding protein 4	1888	0
XM_020102091.1	male-specific lethal 3 homolog isoform X1	3062	0
XM_020102092.1	male-specific lethal 3 homolog isoform X1	3212	0
XM_020102093.1	glycerol kinase-like isoform X1	3685	0
XM_020102094.1	glycerol kinase-like isoform X2	3667	0
XM_020102095.1	glycerol kinase-like isoform X3	3646	0
XM_020102096.1	glycerol kinase-like isoform X4	3628	0
XM_020102097.1	alpha-1,3-mannosyl-glycoprotein 4-beta-N-acetylglucosaminyltransferase A-like	1496	0
XM_020102098.1	pituitary adenylate cyclase-activating polypeptide type I receptor-like	1621	0
XM_020102099.1	nuclear receptor coactivator 5-like	3454	0
XM_020102100.1	ras association domain-containing protein 8-like	2067	0
XM_020102101.1	ras association domain-containing protein 8-like	1821	0
XM_020102102.1	ras association domain-containing protein 8-like	1833	0
XM_020102103.1	ras association domain-containing protein 8-like	1962	0
XM_020102104.1	oxygen-regulated protein 1-like	5796	0
XM_020102105.1	PDZ domain-containing protein 7-like	2628	0
XM_020102106.1	MOB kinase activator 2 isoform X1	1046	0
XM_020102107.1	MOB kinase activator 2 isoform X2	1406	6.80E-161

XM_020102108.1	MOB kinase activator 2 isoform X3	1219	7.82E-161
XM_020102109.1	MOB kinase activator 2 isoform X4	1484	1.97E-154
XM_020102110.1	tyrosine 3-monooxygenase	1947	0
XM_020102111.1	dihydrofolate reductase-like	1545	3.61E-163
XM_020102112.1	cytosolic carboxypeptidase 2	3614	0
XM_020102113.1	tyrosine-protein phosphatase non-receptor type 5-like	4474	0
XM_020102114.1	tyrosine-protein phosphatase non-receptor type 5-like	4524	0
XM_020102115.1	SITS-binding protein-like isoform X1	5242	0
XM_020102116.1	scavenger receptor class A member 3	3565	0
XM_020102117.1	SITS-binding protein-like isoform X2	5033	0
XM_020102118.1	SITS-binding protein-like isoform X3	2365	0
XM_020102119.1	transmembrane protein 17B-like	632	2.82E-75
XM_020102120.1	solute carrier family 15 member 5	1725	0
XM_020102121.1	receptor-type tyrosine-protein phosphatase eta-like	3946	0
XM_020102122.1	immunoglobulin superfamily member 22	4149	0
XM_020102123.1	serine/threonine-protein kinase BRSK2-like	2997	0
XM_020102124.1	pleckstrin homology-like domain family A member 2	954	8.80E-92
XM_020102125.1	coiled-coil domain-containing protein 77	1725	0
XM_020102126.1	leucine-rich repeat-containing protein 56 isoform X1	2498	0
XM_020102127.1	leucine-rich repeat-containing protein 56 isoform X1	2124	0
XM_020102128.1	voltage-dependent L-type calcium channel subunit beta-2-like	2167	0
XM_020102129.1	electron transfer flavoprotein beta subunit lysine methyltransferase	952	0
XM_020102130.1	protein-glutamine gamma-glutamyltransferase 2-like	2704	0
XM_020102131.1	membrane protein MLC1 isoform X1	2468	0
XM_020102132.1	membrane protein MLC1 isoform X2	2468	0
XM_020102133.1	solute carrier family 41 member 2-like	2060	0
XM_020102134.1	pannexin-3 isoform X2	3549	0
XM_020102135.1	carbohydrate sulfotransferase 1-like	1852	0
XM_020102136.1	leucine-rich repeat-containing protein 10	1528	0
XM_020102137.1	USP6 N-terminal-like protein	4442	0
XM_020102138.1	USP6 N-terminal-like protein	4356	0
XM_020102139.1	protein SPT2 homolog isoform X1	3090	0
XM_020102140.1	MAM and LDL-receptor class A domain-containing protein 1-like	4765	0
XM_020102141.1	protein SPT2 homolog isoform X2	3087	0
XM_020102142.1	ubiquitin-conjugating enzyme E2 variant 3-like	2210	0
XM_020102143.1	lysoplasmalogenase-like protein TMEM86A	5909	7.37E-178
XM_020102144.1	CCR4-NOT transcription complex subunit 1 isoform X1	8565	0
XM_020102145.1	CCR4-NOT transcription complex subunit 1 isoform X1	8396	0
XM_020102146.1	CCR4-NOT transcription complex subunit 1 isoform X1	8559	0
XM_020102147.1	CCR4-NOT transcription complex subunit 1 isoform X1	8556	0
XM_020102148.1	CCR4-NOT transcription complex subunit 1 isoform X1	8552	0
XM_020102149.1	CCR4-NOT transcription complex subunit 1 isoform X1	8550	0
XM_020102150.1	CCR4-NOT transcription complex subunit 1 isoform X6	8546	0
XM_020102151.1	CCR4-NOT transcription complex subunit 1 isoform X7	8543	0
XM_020102152.1	CCR4-NOT transcription complex subunit 1 isoform X8	8537	0
XM_020102153.1	N-lysine methyltransferase SETD6	1657	0
XM_020102154.1	obg-like ATPase 1	2890	0
XM_020102155.1	WASH complex subunit SWIP isoform X1	4634	0
XM_020102156.1	WASH complex subunit SWIP isoform X2	4631	0
XM_020102157.1	CCR4-NOT transcription complex subunit 2	2171	0
XM_020102158.1	CCR4-NOT transcription complex subunit 2	2182	0
XM_020102159.1	formin-binding protein 4	3207	0
XM_020102160.1	seipin-like isoform X1	2574	0
XM_020102161.1	carnitine O-palmitoyltransferase 1, liver isoform-like isoform X1	4158	0
XM_020102162.1	carnitine O-palmitoyltransferase 1, liver isoform-like isoform X1	4047	0
XM_020102163.1	carnitine O-palmitoyltransferase 1, liver isoform-like isoform X2	4149	0
XM_020102164.1	guanine nucleotide-binding protein G(o) subunit alpha isoform X1	6006	0
XM_020102165.1	guanine nucleotide-binding protein G(o) subunit alpha isoform X2	1857	0
XM_020102166.1	guanine nucleotide-binding protein G(o) subunit alpha isoform X3	1857	0
XM_020102167.1	trithorax group protein osa-like isoform X2	1419	0
XM_020102168.1	cat eye syndrome critical region protein 5 homolog	2629	0
XM_020102169.1	coiled-coil domain-containing protein 34-like	1759	7.92E-168
XM_020102170.1	coiled-coil domain-containing protein 34-like	1757	7.65E-168
XM_020102171.1	ras-related protein Rab-19-like	1961	7.75E-171
XM_020102172.1	ras-related protein Rab-19-like	1991	1.08E-170
XM_020102173.1	UDP-GlcNAc:betaGal beta-1,3-N-acetylglucosaminyltransferase 9	2494	0
XM_020102174.1	UPF0183 protein C16orf70 homolog isoform X1	7736	0
XM_020102175.1	UPF0183 protein C16orf70 homolog isoform X2	7703	0
XM_020102176.1	ankyrin repeat and BTB/POZ domain-containing protein BTBD11-B isoform X1	4304	0
XM_020102177.1	ankyrin repeat and BTB/POZ domain-containing protein BTBD11-B isoform X2	4203	0
XM_020102178.1	ankyrin repeat and BTB/POZ domain-containing protein BTBD11-B isoform X1	3439	0
XM_020102179.1	sterile alpha motif domain-containing protein 7	1627	0
XM_020102180.1	PREDICTED: cryptochrome-1-like	4181	0
XM_020102181.1	PREDICTED: cryptochrome-1-like	4063	0
XM_020102182.1	GTPase HRas	3871	6.25E-115
XM_020102183.1	GTPase HRas	3869	6.17E-115
XM_020102184.1	ATP-binding cassette sub-family C member 8 isoform X1	5336	0
XM_020102185.1	ATP-binding cassette sub-family C member 8 isoform X2	4854	0
XM_020102186.1	ATP-sensitive inward rectifier potassium channel 11-like	1886	0

XM_020102187.1	insulin-like growth factor II isoform X1	3076	7.00E-137
XM_020102188.1	insulin-like growth factor II isoform X2	2994	2.84E-118
XM_020102189.1	nucleosome assembly protein 1-like 4 isoform X1	2870	0
XM_020102190.1	nucleosome assembly protein 1-like 4 isoform X2	2904	0
XM_020102191.1	MTSS1-like protein isoform X1	3505	0
XM_020102192.1	receptor-type tyrosine-protein phosphatase F-like	5108	0
XM_020102193.1	MTSS1-like protein isoform X2	3496	0
XM_020102194.1	MTSS1-like protein isoform X3	3349	0
XM_020102195.1	L-lactate dehydrogenase A chain	2184	0
XM_020102196.1	selenoprotein O	3470	0
XM_020102197.1	oxysterol-binding protein-related protein 5-like	3583	0
XM_020102198.1	oxysterol-binding protein-related protein 5-like	3521	0
XM_020102199.1	ankyrin repeat and BTB/POZ domain-containing protein 2	4750	0
XM_020102200.1	tumor susceptibility gene 101 protein-like	2648	0
XM_020102201.1	tumor susceptibility gene 101 protein-like	989	1.34E-141
XM_020102202.1	traB domain-containing protein isoform X2	2884	0
XM_020102203.1	traB domain-containing protein isoform X2	2662	0
XM_020102204.1	traB domain-containing protein isoform X2	2720	0
XM_020102205.1	PREDICTED: LOW QUALITY PROTEIN: complexin-4-like	1256	1.65E-107
XM_020102206.1	traB domain-containing protein isoform X2	2627	0
XM_020102207.1	traB domain-containing protein isoform X2	3410	0
XM_020102208.1	cytochrome P450 2B4-like isoform X1	2092	0
XM_020102209.1	cytochrome P450 2B4-like isoform X2	1598	0
XM_020102210.1	cytochrome P450 2B4-like isoform X2	1788	0
XM_020102211.1	beta-1,4-N-acetylgalactosaminyltransferase 3-like	5212	0
XM_020102212.1	N-acetyl-beta-glucosaminyl-glycoprotein 4-beta-N-acetylgalactosaminyltransferase 1-like	2616	1.62E-48
XM_020102213.1	PREDICTED: uncharacterized protein LOC109638986 isoform X1	5014	0
XM_020102214.1	PREDICTED: uncharacterized protein LOC109638986 isoform X2	4996	0
XM_020102215.1	mitogen-activated protein kinase 12-like	2027	0
XM_020102216.1	cytochrome P450 2F2-like	1768	0
XM_020102217.1	F-box only protein 38-like	3394	0
XM_020102218.1	excitatory amino acid transporter 1-like	1216	0
XM_020102219.1	heat shock factor protein 4	2563	0
XM_020102220.1	core-binding factor subunit beta isoform X1	3605	1.53E-93
XM_020102221.1	core-binding factor subunit beta isoform X1	3620	5.15E-94
XM_020102222.1	core-binding factor subunit beta isoform X1	3600	3.15E-87
XM_020102223.1	core-binding factor subunit beta isoform X1	3575	3.16E-86
XM_020102224.1	core-binding factor subunit beta isoform X4	814	8.12E-92
XM_020102225.1	core-binding factor subunit beta isoform X4	776	5.04E-92
XM_020102226.1	core-binding factor subunit beta isoform X4	784	3.43E-84
XM_020102227.1	tripartite motif-containing protein 16-like	2037	0
XM_020102228.1	transcription termination factor 2, mitochondrial	1986	0
XM_020102229.1	transcription termination factor 2, mitochondrial	1949	0
XM_020102230.1	transcription termination factor 2, mitochondrial	1916	0
XM_020102231.1	transcription termination factor 2, mitochondrial	1953	0
XM_020102232.1	transcription termination factor 2, mitochondrial	1840	0
XM_020102233.1	PREDICTED: uncharacterized protein LOC109638993	823	0
XM_020102234.1	transcription termination factor 2, mitochondrial	2190	0
XM_020102235.1	39S ribosomal protein L23, mitochondrial	1503	4.49E-107
XM_020102236.1	small cell adhesion glycoprotein homolog	3312	9.74E-45
XM_020102237.1	protein S100-B	503	1.20E-53
XM_020102238.1	sodium- and chloride-dependent GABA transporter 2-like isoform X1	1762	0
XM_020102239.1	SH3 domain-binding protein 4	5112	0
XM_020102240.1	ADP-ribosylation factor-like protein 4C	1514	9.03E-131
XM_020102241.1	cyclic nucleotide-gated channel cone photoreceptor subunit alpha-like	1944	0
XM_020102242.1	guanylate cyclase soluble subunit beta-2-like	2127	0
XM_020102243.1	pancreatic alpha-amylase-like	1034	0
XM_020102244.1	aldehyde oxidase 1-like	4078	0
XM_020102245.1	catenin delta-2 isoform X4	2792	0
XM_020102246.1	centromere protein L isoform X1	1544	0
XM_020102247.1	centromere protein L isoform X2	1558	0
XM_020102248.1	coiled-coil domain-containing protein 14 isoform X1	1962	0
XM_020102249.1	coiled-coil domain-containing protein 14 isoform X1	1903	0
XM_020102250.1	muscarinic acetylcholine receptor M4	1811	0
XM_020102251.1	PREDICTED: uncharacterized protein C7orf57 homolog	481	4.95E-109
XM_020102252.1	receptor-type tyrosine-protein phosphatase mu	1132	6.78E-142
XM_020102253.1	probable cysteine--tRNA ligase, mitochondrial	2148	0
XM_020102254.1	ubiquitin-like-conjugating enzyme ATG3 isoform X1	3078	0
XM_020102255.1	ubiquitin-like-conjugating enzyme ATG3 isoform X2	2988	0
XM_020102256.1	ubiquitin-like-conjugating enzyme ATG3 isoform X1	2977	0
XM_020102257.1	DDRGK domain-containing protein 1	1569	5.42E-108
XM_020102258.1	basic leucine zipper and W2 domain-containing protein 1-A isoform X1	2066	0
XM_020102259.1	basic leucine zipper and W2 domain-containing protein 1-A isoform X2	1678	0
XM_020102260.1	transcription factor 15-like	1943	9.70E-104
XM_020102261.1	von Willebrand factor D and EGF domain-containing protein-like isoform X1	7005	0
XM_020102262.1	von Willebrand factor D and EGF domain-containing protein-like isoform X2	6885	0
XM_020102263.1	microtubule cross-linking factor 1-like	3252	0
XM_020102264.1	PREDICTED: kynureninase	2961	0
XM_020102265.1	PREDICTED: kynureninase	2762	0
XM_020102266.1	rho GTPase-activating protein 1	6664	0

XM_020102267.1	activin receptor type-2A	8954	0
XM_020102268.1	diacylglycerol kinase zeta isoform X1	6443	0
XM_020102269.1	diacylglycerol kinase zeta isoform X2	6398	0
XM_020102270.1	diacylglycerol kinase zeta isoform X3	6395	0
XM_020102271.1	diacylglycerol kinase zeta isoform X4	6332	0
XM_020102272.1	microtubule cross-linking factor 1-like	1808	0
XM_020102273.1	diacylglycerol kinase zeta isoform X5	6597	0
XM_020102274.1	diacylglycerol kinase zeta isoform X6	5661	0
XM_020102275.1	pleiotrophin isoform X3	988	7.70E-95
XM_020102276.1	ubiquitin-protein ligase E3A isoform X1	4851	0
XM_020102277.1	ubiquitin-protein ligase E3A isoform X1	4672	0
XM_020102278.1	peripheral plasma membrane protein CASK isoform X1	4888	0
XM_020102279.1	peripheral plasma membrane protein CASK isoform X2	4885	0
XM_020102280.1	peripheral plasma membrane protein CASK isoform X3	4873	0
XM_020102281.1	peripheral plasma membrane protein CASK isoform X4	4867	0
XM_020102282.1	peripheral plasma membrane protein CASK isoform X5	4846	0
XM_020102283.1	peripheral plasma membrane protein CASK isoform X6	4843	0
XM_020102284.1	peripheral plasma membrane protein CASK isoform X7	4831	0
XM_020102285.1	peripheral plasma membrane protein CASK isoform X8	4828	0
XM_020102286.1	gamma-soluble NSF attachment protein	915	1.20E-106
XM_020102287.1	peripheral plasma membrane protein CASK isoform X9	4825	0
XM_020102288.1	peripheral plasma membrane protein CASK isoform X10	3176	0
XM_020102289.1	probable G-protein coupled receptor 34	1065	6.26E-131
XM_020102290.1	methyl-CpG-binding domain protein 5	8884	0
XM_020102291.1	methyl-CpG-binding domain protein 5	8739	0
XM_020102292.1	methyl-CpG-binding domain protein 5	8840	0
XM_020102293.1	origin recognition complex subunit 4	1592	0
XM_020102294.1	zinc finger E-box-binding homeobox 2 isoform X1	5716	0
XM_020102295.1	zinc finger E-box-binding homeobox 2 isoform X1	5579	0
XM_020102296.1	zinc finger E-box-binding homeobox 2 isoform X1	5713	0
XM_020102297.1	zinc finger E-box-binding homeobox 2 isoform X1	5637	0
XM_020102298.1	zinc finger E-box-binding homeobox 2 isoform X4	5634	0
XM_020102299.1	ubiquitin carboxyl-terminal hydrolase 20 isoform X1	2409	0
XM_020102300.1	kinesin heavy chain	5310	0
XM_020102301.1	sodium/potassium-transporting ATPase subunit beta-233-like	2341	0
XM_020102302.1	probable ATP-dependent RNA helicase DDX4	2453	0
XM_020102303.1	probable ATP-dependent RNA helicase DDX4	2330	0
XM_020102304.1	ankyrin repeat domain-containing protein 10-like isoform X2	1806	0
XM_020102305.1	ankyrin repeat domain-containing protein 10-like isoform X2	1919	0
XM_020102306.1	heat shock factor 2-binding protein	1280	0
XM_020102307.1	alpha-crystallin A chain	531	5.89E-109
XM_020102308.1	lysine-specific demethylase 6A-like	1368	2.88E-138
XM_020102309.1	FUN14 domain-containing protein 1 isoform X1	1856	7.15E-84
XM_020102310.1	FUN14 domain-containing protein 1 isoform X2	1853	3.78E-83
XM_020102311.1	adenylate kinase isoenzyme 5	1546	0
XM_020102312.1	FUN14 domain-containing protein 1 isoform X3	1850	1.66E-82
XM_020102313.1	FUN14 domain-containing protein 1 isoform X4	1847	8.32E-82
XM_020102314.1	thiamine transporter 1	3819	0
XM_020102315.1	thiamine transporter 1	1948	0
XM_020102316.1	ADP-ribosylation factor-like protein 6-interacting protein 6 isoform X1	1004	8.36E-98
XM_020102317.1	ADP-ribosylation factor-like protein 6-interacting protein 6 isoform X2	954	3.32E-94
XM_020102318.1	aldehyde oxidase isoform X1	4243	0
XM_020102319.1	aldehyde oxidase isoform X2	4237	0
XM_020102320.1	aldehyde oxidase isoform X3	4231	0
XM_020102321.1	neural proliferation differentiation and control protein 1 isoform X1	3989	3.81E-178
XM_020102322.1	neural proliferation differentiation and control protein 1 isoform X2	3987	2.06E-177
XM_020102323.1	E3 ubiquitin-protein ligase HECTD3	2366	0
XM_020102324.1	60S ribosomal protein L35	514	4.66E-52
XM_020102325.1	olfactomedin-like protein 2A	4037	0
XM_020102326.1	negative elongation factor B	2259	0
XM_020102327.1	NADPH oxidase activator 1-like isoform X1	2143	0
XM_020102328.1	neutrophil cytosol factor 2-like isoform X2	2047	0
XM_020102329.1	fibroblast growth factor 17 isoform X1	1451	1.07E-158
XM_020102330.1	fibroblast growth factor 17 isoform X2	1419	8.73E-150
XM_020102331.1	fibroblast growth factor 17 isoform X3	1405	5.07E-146
XM_020102332.1	PDZ domain-containing protein 2-like	7203	0
XM_020102333.1	PREDICTED: lymphotoxin-alpha-like	678	5.28E-146
XM_020102334.1	laminin subunit beta-2-like	3257	0
XM_020102335.1	autophagy-related protein 2 homolog B isoform X1	7855	0
XM_020102336.1	partitioning defective 3 homolog	728	6.53E-64
XM_020102337.1	FK506-binding protein 15	3706	0
XM_020102338.1	collagen alpha-1(XXIII) chain-like	1260	1.09E-98
XM_020102339.1	collagen alpha-1(V) chain-like	5325	0
XM_020102340.1	PDZ domain-containing protein 7-like	2907	0
XM_020102341.1	activated RNA polymerase II transcriptional coactivator p15	844	6.34E-56
XM_020102342.1	protein PAXX	1559	2.27E-134
XM_020102343.1	ectonucleoside triphosphate diphosphohydrolase 2-like	1825	0
XM_020102344.1	protein crumbs homolog 2	8052	0
XM_020102345.1	adhesion G-protein coupled receptor D2	5078	0
XM_020102346.1	nuclear receptor subfamily 6 group A member 1 isoform X1	6851	0

XM_020102347.1	nuclear receptor subfamily 6 group A member 1 isoform X2	6878	0
XM_020102348.1	partitioning defective 3 homolog	4795	0
XM_020102349.1	nuclear receptor subfamily 6 group A member 1 isoform X3	6854	0
XM_020102350.1	nuclear receptor subfamily 6 group A member 1 isoform X4	6871	0
XM_020102351.1	steroidogenic factor 1-like	4734	0
XM_020102352.1	LIM/homeobox protein Lhx2 isoform X1	2524	0
XM_020102353.1	LIM/homeobox protein Lhx2 isoform X2	2521	0
XM_020102354.1	ubiquinol-cytochrome-c reductase complex assembly factor 3	517	2.63E-54
XM_020102355.1	atrial natriuretic peptide receptor 3	2749	0
XM_020102356.1	Golgi-associated plant pathogenesis-related protein 1-like	754	3.04E-106
XM_020102357.1	folylpolyglutamate synthase, mitochondrial-like isoform X1	2613	0
XM_020102358.1	folylpolyglutamate synthase, mitochondrial-like isoform X2	2494	0
XM_020102359.1	PREDICTED: uncharacterized protein LOC109639082	1321	0
XM_020102360.1	neuropilin-2 isoform X2	1002	0
XM_020102361.1	PREDICTED: uncharacterized protein LOC109639083	1974	4.92E-152
XM_020102362.1	PREDICTED: uncharacterized protein LOC109639083	1897	2.15E-152
XM_020102363.1	syntaxin-binding protein 1-like	1841	0
XM_020102364.1	PREDICTED: uncharacterized protein LOC109639085 isoform X1	1629	6.88E-159
XM_020102365.1	PREDICTED: uncharacterized protein LOC109639085 isoform X2	658	9.07E-144
XM_020102366.1	collagen alpha-1(I) chain-like	1336	3.91E-98
XM_020102367.1	P2Y purinoceptor 2-like	2090	0
XM_020102368.1	PREDICTED: perforin-1-like	654	2.09E-83
XM_020102369.1	alpha-(1,3)-fucosyltransferase 7	1167	0
XM_020102370.1	PREDICTED: perforin-1-like	677	4.39E-73
XM_020102371.1	voltage-dependent N-type calcium channel subunit alpha-1B-like	9053	0
XM_020102372.1	contactin-associated protein-like 4	6366	0
XM_020102373.1	histone-lysine N-methyltransferase EHMT1-like	3639	0
XM_020102374.1	endoplasmic reticulum mannosyl-oligosaccharide 1,2-alpha-mannosidase	6029	0
XM_020102375.1	dipeptidyl peptidase 2	2657	0
XM_020102376.1	tubulin beta-4B chain	1639	0
XM_020102377.1	apoptosis regulator Bcl-2-like	1587	1.96E-156
XM_020102378.1	zinc finger RNA-binding protein isoform X1	4378	0
XM_020102379.1	zinc finger RNA-binding protein isoform X2	2812	0
XM_020102380.1	serine/threonine-protein phosphatase 6 catalytic subunit	1370	0
XM_020102381.1	Golgi phosphoprotein 3	4393	0
XM_020102382.1	endophilin-B2 isoform X1	3807	0
XM_020102383.1	endophilin-B2 isoform X2	3794	0
XM_020102384.1	rho GTPase-activating protein 12-like	944	1.29E-161
XM_020102385.1	endophilin-B2 isoform X3	3793	0
XM_020102386.1	endophilin-B2 isoform X4	3786	0
XM_020102387.1	endophilin-B2 isoform X5	3780	0
XM_020102388.1	endophilin-B2 isoform X6	3759	0
XM_020102389.1	protein phosphatase 1F	5322	0
XM_020102390.1	protein phosphatase 1F	5278	0
XM_020102391.1	C-X-C motif chemokine 11-like	598	1.44E-60
XM_020102392.1	ATP-binding cassette sub-family A member 2	9650	0
XM_020102393.1	niban-like protein 1	3799	0
XM_020102394.1	protein CYR61-like	2129	0
XM_020102395.1	LIM domain transcription factor LMO4.1-like	1647	5.40E-111
XM_020102396.1	contactin-associated protein-like 4	6036	0
XM_020102397.1	golgin subfamily A member 1 isoform X1	5408	0
XM_020102398.1	golgin subfamily A member 1 isoform X1	5405	0
XM_020102399.1	golgin subfamily A member 1 isoform X1	5333	0
XM_020102400.1	golgin subfamily A member 1 isoform X1	5279	0
XM_020102401.1	DNA topoisomerase 3-beta-1	2864	0
XM_020102402.1	arrestin domain-containing protein 1-like	2563	0
XM_020102403.1	brain-specific angiogenesis inhibitor 1-like	7106	0
XM_020102404.1	homocysteine-responsive endoplasmic reticulum-resident ubiquitin-like domain member 2 protein	2264	0
XM_020102405.1	homocysteine-responsive endoplasmic reticulum-resident ubiquitin-like domain member 2 protein	2262	0
XM_020102406.1	zinc finger E-box-binding homeobox 1-like	922	9.56E-76
XM_020102407.1	sperm acrosome membrane-associated protein 4-like	1512	1.65E-85
XM_020102408.1	ephrin-A1-like isoform X1	2459	2.41E-134
XM_020102409.1	ephrin-A1-like isoform X2	2456	1.79E-133
XM_020102410.1	protein FAM167B-like	1477	2.07E-71
XM_020102411.1	protein FAM167A-like	934	3.40E-76
XM_020102412.1	sperm acrosome membrane-associated protein 4-like	839	3.53E-78
XM_020102413.1	sperm acrosome membrane-associated protein 4-like	878	8.13E-69
XM_020102414.1	ephrin-A3-like isoform X1	2947	1.02E-147
XM_020102415.1	ephrin-A3-like isoform X2	3349	5.20E-146
XM_020102416.1	ephrin-A3-like isoform X3	1535	1.47E-160
XM_020102417.1	carnitine O-acetyltransferase-like	1909	0
XM_020102418.1	supervillin isoform X4	8012	0
XM_020102419.1	PREDICTED: nurim	2346	8.29E-160
XM_020102420.1	tripartite motif-containing protein 46-like	794	3.92E-95
XM_020102421.1	sorting nexin-27-like	5091	0
XM_020102422.1	circadian-associated transcriptional repressor	976	3.96E-157
XM_020102423.1	ATPase inhibitor, mitochondrial	461	8.05E-19
XM_020102424.1	TGF-beta receptor type-2-like	1835	0
XM_020102425.1	PREDICTED: phostensin	4245	0

XM_020102426.1	histone-lysine N-methyltransferase SETDB1-B-like	1558	0
XM_020102427.1	histone-lysine N-methyltransferase SETDB1-A-like isoform X1	1827	0
XM_020102428.1	histone-lysine N-methyltransferase SETDB1-A-like isoform X2	1824	0
XM_020102429.1	ETS translocation variant 3-like protein isoform X1	718	1.31E-152
XM_020102430.1	histone-lysine N-methyltransferase SETDB1-A-like isoform X1	1792	0
XM_020102431.1	histone-lysine N-methyltransferase EHMT2 isoform X1	4265	0
XM_020102432.1	histone-lysine N-methyltransferase EHMT2 isoform X2	4247	0
XM_020102433.1	histone-lysine N-methyltransferase EHMT2 isoform X3	4596	0
XM_020102434.1	choline transporter-like protein 4 isoform X1	4456	0
XM_020102435.1	choline transporter-like protein 4 isoform X2	4450	0
XM_020102436.1	double-strand-break repair protein rad21 homolog isoform X1	2833	0
XM_020102437.1	double-strand-break repair protein rad21 homolog isoform X2	2369	0
XM_020102438.1	zinc finger and BTB domain-containing protein 12-like	2741	0
XM_020102439.1	zinc finger and BTB domain-containing protein 12-like	2728	0
XM_020102440.1	negative elongation factor E	1250	5.52E-91
XM_020102441.1	negative elongation factor E	1214	3.22E-91
XM_020102442.1	DNA ligase 1	3053	0
XM_020102443.1	rRNA-processing protein UTP23 homolog	2131	1.80E-175
XM_020102444.1	E3 ubiquitin-protein ligase RNF5	2035	1.10E-93
XM_020102445.1	RNA-binding motif, single-stranded-interacting protein 3-like isoform X1	1254	0
XM_020102446.1	RNA-binding motif, single-stranded-interacting protein 3-like isoform X2	1897	1.57E-138
XM_020102447.1	exosome complex component RRP42	1028	0
XM_020102448.1	PREDICTED: uncharacterized protein LOC109639147 isoform X1	1437	0
XM_020102449.1	E3 ubiquitin-protein ligase RNF115-like isoform X2	1550	3.10E-175
XM_020102450.1	E3 ubiquitin-protein ligase RNF115-like isoform X3	1525	2.07E-170
XM_020102451.1	E3 ubiquitin-protein ligase RNF115-like isoform X4	1046	5.66E-155
XM_020102452.1	PREDICTED: uncharacterized protein LOC109639147 isoform X5	1041	4.22E-143
XM_020102453.1	PREDICTED: uncharacterized protein LOC109639147 isoform X1	1185	9.42E-145
XM_020102454.1	neuronal acetylcholine receptor subunit alpha-7-like	3553	0
XM_020102455.1	lysine-specific demethylase 3B isoform X1	6648	0
XM_020102456.1	sodium/potassium-transporting ATPase subunit alpha-1	3332	0
XM_020102457.1	protein S100-A1-like	542	3.05E-45
XM_020102458.1	small conductance calcium-activated potassium channel protein 1-like	2090	0
XM_020102459.1	sperm acrosome membrane-associated protein 4-like	637	1.11E-87
XM_020102460.1	C-type lectin domain family 3 member A	1386	7.89E-131
XM_020102461.1	LIM domain-containing protein 1-like	746	4.96E-131
XM_020102462.1	protein PRRC2A-like	5935	0
XM_020102463.1	protein PRRC2A-like	8946	0
XM_020102464.1	sodium bicarbonate cotransporter 3-like isoform X1	5974	0
XM_020102465.1	sodium bicarbonate cotransporter 3-like isoform X2	5765	0
XM_020102466.1	sodium bicarbonate cotransporter 3-like isoform X3	5938	0
XM_020102467.1	sodium bicarbonate cotransporter 3-like isoform X4	5932	0
XM_020102468.1	sodium bicarbonate cotransporter 3-like isoform X5	5588	0
XM_020102469.1	zinc finger protein 347-like	1205	0
XM_020102470.1	sodium bicarbonate cotransporter 3-like isoform X6	5552	0
XM_020102471.1	sodium bicarbonate cotransporter 3-like isoform X7	5546	0
XM_020102472.1	histone-lysine N-methyltransferase, H3 lysine-36 and H4 lysine-20 specific-like	11723	0
XM_020102473.1	histone-lysine N-methyltransferase, H3 lysine-36 and H4 lysine-20 specific-like	11726	0
XM_020102474.1	histone-lysine N-methyltransferase, H3 lysine-36 and H4 lysine-20 specific-like	11644	0
XM_020102475.1	histone-lysine N-methyltransferase, H3 lysine-36 and H4 lysine-20 specific-like	11696	0
XM_020102476.1	regulation of nuclear pre-mRNA domain-containing protein 2-like	3630	0
XM_020102477.1	CUGBP Elav-like family member 3 isoform X1	2724	0
XM_020102478.1	CUGBP Elav-like family member 3 isoform X2	2722	0
XM_020102479.1	CUGBP Elav-like family member 3 isoform X3	2708	0
XM_020102480.1	CUGBP Elav-like family member 3 isoform X4	2694	0
XM_020102481.1	CUGBP Elav-like family member 3 isoform X5	2677	0
XM_020102482.1	CUGBP Elav-like family member 3 isoform X6	2675	0
XM_020102483.1	nectin-4 isoform X1	1977	0
XM_020102484.1	dolichyl-diphosphooligosaccharide--protein glycosyltransferase subunit STT3A	5756	0
XM_020102485.1	palmitoyltransferase ZDHHC3-like	3822	0
XM_020102486.1	transmembrane protein 42	3589	8.59E-89
XM_020102487.1	heterogeneous nuclear ribonucleoprotein R isoform X2	3176	0
XM_020102488.1	heterogeneous nuclear ribonucleoprotein R isoform X2	2657	0
XM_020102489.1	casein kinase II subunit beta	3006	2.14E-141
XM_020102490.1	casein kinase II subunit beta	2935	3.51E-140
XM_020102491.1	N(G),N(G)-dimethylarginine dimethylaminohydrolase 2-like	1805	0
XM_020102492.1	N(G),N(G)-dimethylarginine dimethylaminohydrolase 2-like	1835	0
XM_020102493.1	N(G),N(G)-dimethylarginine dimethylaminohydrolase 2-like	1848	0
XM_020102494.1	40S ribosomal protein S5	756	1.96E-152
XM_020102495.1	chloride intracellular channel protein 4-like	1279	2.46E-160
XM_020102496.1	probable ATP-dependent DNA helicase HFM1 isoform X1	5048	0
XM_020102497.1	chloride intracellular channel protein 4-like	1310	2.54E-159
XM_020102498.1	chloride intracellular channel protein 4-like	1292	6.84E-160
XM_020102499.1	sperm acrosome membrane-associated protein 4-like	3078	2.54E-70
XM_020102500.1	ceramide synthase 2-like	3053	0
XM_020102501.1	cellular retinoic acid-binding protein 2-like	806	6.32E-101
XM_020102502.1	40S ribosomal protein S27-like isoform X1	571	1.38E-47
XM_020102503.1	CUB domain-containing protein 1-like isoform X1	4740	0
XM_020102504.1	probable ATP-dependent DNA helicase HFM1 isoform X1	5191	0

XM_020102505.1	CUB domain-containing protein 1-like isoform X2	4766	0
XM_020102506.1	oxysterol-binding protein-related protein 10	4702	0
XM_020102507.1	lysosomal thioesterase PPT2-A-like	2838	0
XM_020102508.1	dual specificity protein kinase CLK2 isoform X1	2908	0
XM_020102509.1	dual specificity protein kinase CLK2 isoform X2	2896	0
XM_020102510.1	dual specificity protein kinase CLK2 isoform X1	2373	0
XM_020102511.1	protein S100-A1-like	476	1.65E-46
XM_020102512.1	dolichol-phosphate mannosyltransferase subunit 3	1306	2.32E-48

XM_020102513.1	probable ATP-dependent DNA helicase HFM1 isoform X1	5047	0
XM_020102514.1	helicase SKI2W-like	4060	0
XM_020102515.1	secretory carrier-associated membrane protein 3	2213	2.30E-179
XM_020102516.1	eomesodermin homolog isoform X1	2773	0
XM_020102517.1	eomesodermin homolog isoform X1	2374	0
XM_020102518.1	eomesodermin homolog isoform X1	2410	0
XM_020102519.1	eomesodermin homolog isoform X1	2339	0
XM_020102520.1	eomesodermin homolog isoform X1	2524	0
XM_020102521.1	leucine-rich repeat-containing protein 71	2106	0
XM_020102522.1	probable ATP-dependent DNA helicase HFM1 isoform X1	4946	0
XM_020102523.1	28S ribosomal protein S21, mitochondrial	641	6.49E-60
XM_020102524.1	dnaJ homolog subfamily C member 8 isoform X1	1115	5.21E-157
XM_020102525.1	dnaJ homolog subfamily C member 8 isoform X1	1193	4.34E-148
XM_020102526.1	BMP/retinoic acid-inducible neural-specific protein 3-like	3619	0
XM_020102527.1	BMP/retinoic acid-inducible neural-specific protein 3-like	3616	0
XM_020102528.1	leukocyte elastase inhibitor-like	1337	0
XM_020102529.1	regulator of G-protein signaling 5-like	968	7.92E-121
XM_020102530.1	PREDICTED: uncharacterized protein C1orf226 homolog isoform X1	5122	0
XM_020102531.1	PREDICTED: uncharacterized protein C1orf226 homolog isoform X2	5101	0
XM_020102532.1	probable cation-transporting ATPase 13A3 isoform X1	5554	0
XM_020102533.1	ubiquitin carboxyl-terminal hydrolase isozyme L5	1464	0
XM_020102534.1	forkhead box C1-A-like	2162	0
XM_020102535.1	glutaredoxin 2 isoform X2	1292	2.32E-101
XM_020102536.1	glutaredoxin 2 isoform X2	1193	3.85E-71
XM_020102537.1	glutaredoxin 2 isoform X2	1149	4.48E-71
XM_020102538.1	glutaredoxin 2 isoform X2	1053	7.70E-72
XM_020102539.1	dual specificity protein phosphatase 22-B-like	1999	7.18E-125
XM_020102540.1	regulator of G-protein signaling 5-like	1217	5.39E-137
XM_020102541.1	probable cation-transporting ATPase 13A3 isoform X1	5286	0
XM_020102542.1	regulator of G-protein signaling 5-like	1648	9.54E-133
XM_020102543.1	alanine aminotransferase 2-like	1846	0
XM_020102544.1	regulator of G-protein signaling 21-like isoform X1	2054	4.28E-120
XM_020102545.1	regulator of G-protein signaling 21-like isoform X2	2051	2.78E-120
XM_020102546.1	regulator of G-protein signaling 1-like isoform X3	1991	2.41E-115
XM_020102547.1	BMP/retinoic acid-inducible neural-specific protein 3-like	2869	0
XM_020102548.1	BMP/retinoic acid-inducible neural-specific protein 3-like	2624	0
XM_020102549.1	probable cation-transporting ATPase 13A3 isoform X1	5474	0
XM_020102550.1	1-phosphatidylinositol 4,5-bisphosphate phosphodiesterase gamma-1-like	2779	9.16E-176
XM_020102551.1	regulator of G-protein signaling 8-like	788	6.66E-129
XM_020102552.1	thiamine transporter 1	1428	0
XM_020102553.1	myosin light chain kinase family member 4	2048	0
XM_020102554.1	GDP-mannose 4,6 dehydratase	495	1.83E-84
XM_020102555.1	insulin-like growth factor-binding protein 3	921	2.81E-180
XM_020102556.1	cartilage intermediate layer protein 1-like	467	5.68E-115
XM_020102557.1	regulator of G-protein signaling 21-like	1234	4.82E-147
XM_020102558.1	forkhead box protein Q1	1566	0
XM_020102559.1	forkhead box protein F2	2065	0
XM_020102560.1	valacyclovir hydrolase	1166	0
XM_020102561.1	interferon regulatory factor 4-like	1868	0
XM_020102562.1	regulator of G-protein signaling 8-like	1653	2.09E-87
XM_020102563.1	regulator of G-protein signaling 21-like	655	1.18E-139
XM_020102564.1	zinc finger protein 644-like	4033	0
XM_020102565.1	carboxyl-terminal PDZ ligand of neuronal nitric oxide synthase protein-like isoform X1	996	1.21E-122
XM_020102566.1	carboxyl-terminal PDZ ligand of neuronal nitric oxide synthase protein-like isoform X2	725	1.14E-64
XM_020102567.1	desmoplakin-like isoform X2	12346	0
XM_020102568.1	desmoplakin-like isoform X2	12298	0
XM_020102569.1	desmoplakin-like isoform X3	11014	0
XM_020102570.1	pyrroline-5-carboxylate reductase 3 isoform X3	2608	0
XM_020102571.1	pyrroline-5-carboxylate reductase 3 isoform X3	2160	0
XM_020102572.1	pyrroline-5-carboxylate reductase 3 isoform X3	2009	0
XM_020102573.1	zinc finger protein 644-like	3791	0
XM_020102574.1	pyrroline-5-carboxylate reductase 3 isoform X3	2074	0
XM_020102575.1	protein brambleberry-like	2419	0
XM_020102576.1	GDP-L-fucose synthase	1704	0
XM_020102577.1	GDP-L-fucose synthase	1604	0
XM_020102578.1	phosphatase and actin regulator 1-like isoform X1	2120	0
XM_020102579.1	phosphatase and actin regulator 1-like isoform X2	2085	0
XM_020102580.1	phosphatase and actin regulator 1-like isoform X3	1966	0
XM_020102581.1	NAD-dependent protein deacylase sirtuin-5, mitochondrial	1692	0
XM_020102582.1	lysine-specific demethylase 3B isoform X2	6624	0
XM_020102583.1	zinc finger protein 644-like	3779	0

XM_020102584.1	NAD-dependent protein deacylase sirtuin-5, mitochondrial	1716	0
XM_020102585.1	TBC1 domain family member 7-like	1444	0
XM_020102586.1	tripartite motif-containing protein 65-like isoform X1	2025	0
XM_020102587.1	tripartite motif-containing protein 65-like isoform X2	2022	0
XM_020102588.1	tripartite motif-containing protein 65-like isoform X3	2022	0
XM_020102589.1	elongation factor 1-delta-like isoform X1	1179	0
XM_020102590.1	elongation factor 1-delta-like isoform X1	1033	0
XM_020102591.1	elongation factor 1-delta-like isoform X1	1116	9.23E-163
XM_020102592.1	elongation factor 1-delta-like isoform X1	2304	0
XM_020102593.1	calcium-binding mitochondrial carrier protein SCaMC-1-like	2842	0
XM_020102594.1	transmembrane protein 69-like isoform X2	946	0

XM_020102595.1	solute carrier family 22 member 23	3742	0
XM_020102596.1	melanoma-associated antigen C1-like	3523	0
XM_020102597.1	proteasome assembly chaperone 4	716	8.88E-84
XM_020102598.1	exocyst complex component 2	3258	0
XM_020102599.1	tensin-3-like isoform X1	2480	0
XM_020102600.1	tensin-3-like isoform X2	2674	0
XM_020102601.1	histidine-rich glycoprotein-like	1962	0
XM_020102602.1	pituitary adenylate cyclase-activating polypeptide type I receptor-like	1881	0
XM_020102603.1	neutrophil cytosol factor 4	1403	0
XM_020102604.1	parvalbumin alpha-like	1185	4.16E-55
XM_020102605.1	glutamate receptor ionotropic, NMDA 2A-like	5116	0
XM_020102606.1	dynein light chain 2, cytoplasmic-like	1313	1.62E-71
XM_020102607.1	phosphatidylinositol 4,5-bisphosphate 3-kinase catalytic subunit alpha isoform-like	5406	0
XM_020102608.1	trichohyalin-like isoform X1	3480	0
XM_020102609.1	active regulator of SIRT1	1099	2.88E-73
XM_020102610.1	pseudokinase FAM20A-like isoform X1	642	4.56E-87
XM_020102611.1	pseudokinase FAM20A-like isoform X2	638	8.31E-91
XM_020102612.1	cytohesin-1 isoform X1	1991	0
XM_020102613.1	SUN domain-containing protein 2	3793	0
XM_020102614.1	SUN domain-containing protein 2	3617	0
XM_020102615.1	SUN domain-containing protein 2	3660	0
XM_020102616.1	PREDICTED: uncharacterized protein LOC109639247	1566	0
XM_020102617.1	neuronal pentraxin receptor-like	5619	0
XM_020102618.1	dynein heavy chain domain-containing protein 1	13465	0
XM_020102619.1	protein crumbs homolog 1-like	3057	0
XM_020102620.1	serine/threonine-protein kinase/endoribonuclease IRE1-like	4374	0
XM_020102621.1	protein sidekick-1	6771	0
XM_020102622.1	glutamate receptor ionotropic, NMDA 2A-like	4824	0
XM_020102623.1	unconventional myosin-XV-like	9648	0
XM_020102624.1	transmembrane protease serine 6	2436	0
XM_020102625.1	voltage-dependent calcium channel gamma-2 subunit-like	2416	6.52E-178
XM_020102626.1	transmembrane protease serine 6	458	3.83E-92
XM_020102627.1	platelet-derived growth factor subunit A-like	1841	3.65E-98
XM_020102628.1	intraflagellar transport protein 27 homolog	931	2.11E-139
XM_020102629.1	protein FAM83F	2005	0
XM_020102630.1	serine/threonine-protein kinase N2	5148	0
XM_020102631.1	voltage-dependent L-type calcium channel subunit alpha-1F-like	734	1.62E-77
XM_020102632.1	brain-specific angiogenesis inhibitor 1-associated protein 2-like protein 2	1518	0
XM_020102633.1	ATP-binding cassette sub-family G member 2-like isoform X1	1993	0
XM_020102634.1	ATP-binding cassette sub-family G member 2-like isoform X1	1933	0
XM_020102635.1	actin-related protein 2/3 complex subunit 1A	2269	0
XM_020102636.1	actin-related protein 2/3 complex subunit 1A	2027	0
XM_020102637.1	actin-related protein 2/3 complex subunit 1A	1956	0
XM_020102638.1	actin-related protein 2/3 complex subunit 1A	1957	0
XM_020102639.1	actin-related protein 2/3 complex subunit 1A	1957	0
XM_020102640.1	proline-rich protein 18-like	2109	0
XM_020102641.1	UPF0472 protein C16orf72 homolog	2321	2.62E-160
XM_020102642.1	lipopolysaccharide-induced tumor necrosis factor-alpha factor homolog	772	3.86E-101
XM_020102643.1	coiled-coil domain-containing protein 39	3598	0
XM_020102644.1	85/88 kDa calcium-independent phospholipase A2 isoform X1	3520	0
XM_020102645.1	85/88 kDa calcium-independent phospholipase A2 isoform X1	3513	0
XM_020102646.1	85/88 kDa calcium-independent phospholipase A2 isoform X1	3467	0
XM_020102647.1	85/88 kDa calcium-independent phospholipase A2 isoform X1	3436	0
XM_020102648.1	chromobox protein homolog 6	5087	0
XM_020102649.1	cyclic AMP-dependent transcription factor ATF-4-like	2368	8.86E-164
XM_020102650.1	guanine nucleotide-binding protein subunit alpha-12	4109	0
XM_020102651.1	somatostatin receptor type 5-like	1565	0
XM_020102652.1	protein CutA homolog isoform X1	926	1.99E-110
XM_020102653.1	protein CutA homolog isoform X1	891	5.50E-111
XM_020102654.1	protein CutA homolog isoform X1	896	1.77E-102
XM_020102655.1	zinc finger and BTB domain-containing protein 41	4968	0
XM_020102656.1	3-mercaptopyruvate sulfurtransferase-like	5309	0
XM_020102657.1	3-mercaptopyruvate sulfurtransferase-like	5294	0
XM_020102658.1	complement C1q tumor necrosis factor-related protein 6-like	2439	5.04E-134
XM_020102659.1	ras-related C3 botulinum toxin substrate 1-like	1062	5.28E-141
XM_020102660.1	leucine-rich repeat-containing protein 4C-like	5279	0
XM_020102661.1	leucine-rich repeat-containing protein 4C-like	5289	0
XM_020102662.1	TGF-beta-activated kinase 1 and MAP3K7-binding protein 1 isoform X1	2033	0

XM_020102663.1	TGF-beta-activated kinase 1 and MAP3K7-binding protein 1 isoform X2	2032	0
XM_020102664.1	TGF-beta-activated kinase 1 and MAP3K7-binding protein 1 isoform X3	2015	0
XM_020102665.1	TGF-beta-activated kinase 1 and MAP3K7-binding protein 1 isoform X4	2065	0
XM_020102666.1	TGF-beta-activated kinase 1 and MAP3K7-binding protein 1 isoform X1	2091	0
XM_020102667.1	TGF-beta-activated kinase 1 and MAP3K7-binding protein 1 isoform X1	1995	0
XM_020102668.1	volume-regulated anion channel subunit LRRC8D	3809	0
XM_020102669.1	synaptogyrin-1 isoform X1	2175	6.91E-162
XM_020102670.1	synaptogyrin-1 isoform X2	1414	8.52E-142
XM_020102671.1	actin-related protein 2/3 complex subunit 1A	1750	0
XM_020102672.1	actin-related protein 2/3 complex subunit 1A	1709	0
XM_020102673.1	PREDICTED: uncharacterized protein LOC109639286 isoform X1	1207	0
XM_020102674.1	PREDICTED: uncharacterized protein LOC109639286 isoform X2	1244	0
XM_020102675.1	PREDICTED: uncharacterized protein LOC109639286 isoform X2	1073	0
XM_020102676.1	uracil nucleotide/cysteinyl leukotriene receptor-like isoform X1	2075	0

XM_020102677.1	volume-regulated anion channel subunit LRRC8D	3049	0
XM_020102678.1	uracil nucleotide/cysteinyl leukotriene receptor-like isoform X1	2191	0
XM_020102679.1	hydroxycarboxylic acid receptor 2-like	923	0
XM_020102680.1	COBW domain-containing protein 1-like	1549	0
XM_020102681.1	ephrin-A5 isoform X1	6527	5.94E-133
XM_020102682.1	ephrin-A2-like isoform X1	6446	3.23E-114
XM_020102683.1	phosphoglucomutase-like protein 5	2755	0
XM_020102684.1	Fanconi anemia group C protein isoform X1	2669	0
XM_020102685.1	TSC22 domain family protein 2-like	4852	0
XM_020102686.1	Fanconi anemia group C protein isoform X2	2668	0
XM_020102687.1	growth arrest and DNA damage-inducible protein GADD45 gamma	1374	1.65E-102
XM_020102688.1	E3 ubiquitin-protein ligase RNF180-like	2398	0
XM_020102689.1	5-hydroxytryptamine receptor 1A	1867	0
XM_020102690.1	mitogen-activated protein kinase 6	4811	0
XM_020102691.1	serine/threonine-protein kinase NIM1-like	3755	0
XM_020102692.1	protein patched homolog 1	3398	0
XM_020102693.1	dedicator of cytokinesis protein 8	8171	0
XM_020102694.1	zinc finger SWIM domain-containing protein 6-like	4585	0
XM_020102695.1	B-cell CLL/lymphoma 6 member B protein-like	3146	0
XM_020102696.1	protamine-like protein	840	2.97E-52
XM_020102697.1	forkhead box protein D5-like	1215	0
XM_020102698.1	protein atonal homolog 7-like	2464	4.34E-160
XM_020102699.1	copper-transporting ATPase 1	4869	0
XM_020102700.1	regulator of G-protein signaling 7-binding protein	1710	2.00E-129
XM_020102701.1	transmembrane protein 252	1034	3.36E-120
XM_020102702.1	testosterone 17-beta-dehydrogenase 3	809	4.34E-101
XM_020102703.1	DNA excision repair protein ERCC-8	2388	0
XM_020102704.1	elongation of very long chain fatty acids protein 1-like	1536	0
XM_020102705.1	elongation of very long chain fatty acids protein 1-like	1279	0
XM_020102706.1	deoxyribodipyrimidine photo-lyase-like	2216	0
XM_020102707.1	mimitin, mitochondrial	1241	1.60E-114
XM_020102708.1	small integral membrane protein 15	820	2.79E-33
XM_020102709.1	SWI/SNF-related matrix-associated actin-dependent regulator of chromatin subfamily A containing DEAD/H box 1	4226	0
XM_020102710.1	macrophage-expressed gene 1 protein	2392	0
XM_020102711.1	PREDICTED: perforin-1-like	1885	0
XM_020102712.1	succinyl-CoA:3-ketoacid coenzyme A transferase 1, mitochondrial	2366	0
XM_020102713.1	succinyl-CoA:3-ketoacid coenzyme A transferase 1, mitochondrial	1877	0
XM_020102714.1	F-box only protein 4	1990	0
XM_020102715.1	UPF0600 protein C5orf51 homolog	1777	0
XM_020102716.1	selenoprotein P	2455	6.01E-167
XM_020102717.1	selenoprotein P	2410	3.82E-167
XM_020102718.1	cell division cycle 7-related protein kinase isoform X2	2577	0
XM_020102719.1	zinc finger protein 131 isoform X1	2848	0
XM_020102720.1	zinc finger protein 131 isoform X1	2925	0
XM_020102721.1	zinc finger protein 131 isoform X1	2741	0
XM_020102722.1	growth hormone receptor	6783	0
XM_020102723.1	importin-11 isoform X1	5446	0
XM_020102724.1	importin-11 isoform X1	3013	0
XM_020102725.1	mesoderm induction early response protein 3 isoform X3	3862	0
XM_020102726.1	mesoderm induction early response protein 3 isoform X3	3533	0
XM_020102727.1	cell division cycle 7-related protein kinase isoform X2	2438	0
XM_020102728.1	mesoderm induction early response protein 3 isoform X3	3515	0
XM_020102729.1	mesoderm induction early response protein 3 isoform X3	3480	0
XM_020102730.1	zinc phosphodiesterase ELAC protein 1	1687	0
XM_020102731.1	NAD-dependent malic enzyme, mitochondrial	4205	0
XM_020102732.1	NAD-dependent malic enzyme, mitochondrial	4202	0
XM_020102733.1	NAD-dependent malic enzyme, mitochondrial	4249	0
XM_020102734.1	NAD-dependent malic enzyme, mitochondrial	4246	0
XM_020102735.1	serine/threonine-protein kinase PLK2	3133	0
XM_020102736.1	cell division cycle 7-related protein kinase isoform X3	2285	0
XM_020102737.1	protein FAM173B-like	2018	2.07E-151
XM_020102738.1	ras-related protein Rab-3A-like	481	5.06E-85
XM_020102739.1	ras-related protein Rab-3A-like	562	1.37E-80
XM_020102740.1	aminopeptidase O	3564	0
XM_020102741.1	ecto-NOX disulfide-thiol exchanger 2 isoform X1	2895	0

XM_020102742.1	aminopeptidase O	3693	0
XM_020102743.1	cAMP-specific 3',5'-cyclic phosphodiesterase 4D isoform X1	6159	0
XM_020102744.1	cAMP-specific 3',5'-cyclic phosphodiesterase 4D isoform X2	5369	0
XM_020102745.1	complement component C7-like isoform X1	2796	0
XM_020102746.1	cell division cycle 7-related protein kinase isoform X4	2281	0
XM_020102747.1	complement component C7-like isoform X2	2787	0
XM_020102748.1	PI-PLC X domain-containing protein 3	1629	0
XM_020102749.1	DNA excision repair protein ERCC-6	5489	0
XM_020102750.1	DNA excision repair protein ERCC-6	5486	0
XM_020102751.1	cyclic AMP-responsive element-binding protein 3-like	2855	0
XM_020102752.1	cyclic AMP-responsive element-binding protein 3-like	2859	0
XM_020102753.1	PREDICTED: tetraspanin-3-like	1839	6.05E-167
XM_020102754.1	dehydrogenase/reductase SDR family member 13	2834	0
XM_020102755.1	sortilin-related receptor-like	1697	0
XM_020102756.1	A disintegrin and metalloproteinase with thrombospondin motifs 15-like	5668	0
XM_020102757.1	L-amino-acid oxidase-like	1568	0
XM_020102758.1	glomulin isoform X1	2231	0
XM_020102759.1	mitochondrial import inner membrane translocase subunit Tim8 B	979	1.02E-59
XM_020102760.1	ATP-binding cassette sub-family G member 4-like isoform X1	4209	0
XM_020102761.1	ATP-binding cassette sub-family G member 4-like isoform X2	4080	0
XM_020102762.1	A disintegrin and metalloproteinase with thrombospondin motifs 8	4467	0
XM_020102763.1	succinate dehydrogenase [ubiquinone] cytochrome b small subunit, mitochondrial	1935	5.90E-78
XM_020102764.1	unconventional myosin-XVIIIa	11397	0
XM_020102765.1	glomulin isoform X1	2258	0
XM_020102766.1	peroxisomal sarcosine oxidase	1499	0
XM_020102767.1	seizure protein 6 homolog	4329	0
XM_020102768.1	breast carcinoma-amplified sequence 3	2750	0
XM_020102769.1	beta,beta-carotene 9',10'-oxygenase isoform X1	2311	0
XM_020102770.1	beta,beta-carotene 9',10'-oxygenase isoform X1	2120	0
XM_020102771.1	transcobalamin-1-like isoform X1	645	7.82E-85
XM_020102772.1	glomulin isoform X1	2228	0
XM_020102773.1	CUE domain-containing protein 1 isoform X1	3358	4.88E-144
XM_020102774.1	CUE domain-containing protein 1 isoform X1	3363	5.10E-144
XM_020102775.1	CUE domain-containing protein 1 isoform X1	3355	3.21E-144
XM_020102776.1	WD repeat and SOCS box-containing protein 1	3359	0
XM_020102777.1	kinase suppressor of Ras 1-like isoform X1	4070	0
XM_020102778.1	kinase suppressor of Ras 1-like isoform X1	4233	0
XM_020102779.1	kinase suppressor of Ras 1-like isoform X1	4207	0
XM_020102780.1	copper-transporting ATPase 1	4574	0
XM_020102781.1	kinase suppressor of Ras 1-like isoform X1	2867	0
XM_020102782.1	galectin-9-like isoform X1	1499	0
XM_020102783.1	galectin-9-like isoform X2	2346	0
XM_020102784.1	PHD finger protein 12	4731	0
XM_020102785.1	receptor-interacting serine/threonine-protein kinase 2 isoform X1	4360	0
XM_020102786.1	zinc finger and BTB domain-containing protein 44 isoform X1	2829	0
XM_020102787.1	zinc finger and BTB domain-containing protein 44 isoform X1	2603	0
XM_020102788.1	zinc finger and BTB domain-containing protein 44 isoform X3	2316	0
XM_020102789.1	A-kinase anchor protein 1, mitochondrial	2884	0
XM_020102790.1	A-kinase anchor protein 1, mitochondrial	2847	0
XM_020102791.1	A-kinase anchor protein 1, mitochondrial	2714	0
XM_020102792.1	A-kinase anchor protein 1, mitochondrial	2695	0
XM_020102793.1	A-kinase anchor protein 1, mitochondrial	2809	0
XM_020102794.1	receptor-interacting serine/threonine-protein kinase 2 isoform X2	4357	0
XM_020102795.1	sorting nexin-19	5793	0
XM_020102796.1	LIM/homeobox protein Lhx1	2500	0
XM_020102797.1	general transcription factor IIF subunit 1	1976	0
XM_020102798.1	receptor-interacting serine/threonine-protein kinase 2 isoform X3	4306	0
XM_020102799.1	general transcription factor IIF subunit 1	1964	0
XM_020102800.1	general transcription factor IIF subunit 1	2050	0
XM_020102801.1	F-box/WD repeat-containing protein 9	3241	0
XM_020102802.1	sterile alpha motif domain-containing protein 9-like	5577	0
XM_020102803.1	immediate early response gene 2 protein-like	1129	4.06E-106
XM_020102804.1	interleukin-6 receptor subunit beta-like isoform X1	2577	0
XM_020102805.1	interleukin-6 receptor subunit beta-like isoform X2	2442	0
XM_020102806.1	Na(+)/H(+) exchange regulatory cofactor NHE-RF2	2119	0
XM_020102807.1	protein phosphatase 1 regulatory subunit 29-like	6109	0
XM_020102808.1	protein phosphatase 1 regulatory subunit 29-like	6032	0
XM_020102809.1	deoxyhypusine synthase	1442	0
XM_020102810.1	deoxyhypusine synthase	1601	0
XM_020102811.1	rhodopsin kinase	1674	0
XM_020102812.1	alpha-ketoglutarate-dependent dioxygenase alkB homolog 7, mitochondrial	2585	1.40E-141
XM_020102813.1	somatostatin receptor type 5-like	2749	0
XM_020102814.1	somatostatin receptor type 5-like	2738	0
XM_020102815.1	somatostatin receptor type 5-like	2735	0
XM_020102816.1	BUB3-interacting and GLEBS motif-containing protein ZNF207-like	1997	1.76E-60
XM_020102817.1	tectonin beta-propeller repeat-containing protein 1-like isoform X2	3033	0
XM_020102818.1	MHC class II regulatory factor RFX1-like	4542	0
XM_020102819.1	adhesion G protein-coupled receptor E1-like	2184	0
XM_020102820.1	G-protein coupled receptor family C group 6 member A-like	1830	0

XM_020102821.1	putative glycerol kinase 5 isoform X1	1984	0
XM_020102822.1	mitochondrial thiamine pyrophosphate carrier	1728	0
XM_020102823.1	glutathione S-transferase theta-3-like	1112	3.59E-170
XM_020102824.1	hematological and neurological expressed 1 protein	622	2.11E-98
XM_020102825.1	GDP-L-fucose synthase	1792	0
XM_020102826.1	beta-galactoside-binding lectin-like	744	5.56E-90
XM_020102827.1	cell death-inducing p53-target protein 1 homolog	1663	1.07E-77
XM_020102828.1	arf-GAP with dual PH domain-containing protein 2-like	1471	0
XM_020102829.1	putative glycerol kinase 5 isoform X2	3340	0
XM_020102830.1	cell death-inducing p53-target protein 1 homolog	1007	3.59E-74
XM_020102831.1	WAP, Kazal, immunoglobulin, Kunitz and NTR domain-containing protein 2-like	2849	0
XM_020102832.1	neuronal pentraxin-2 isoform X1	1399	0
XM_020102833.1	neuronal pentraxin-2 isoform X2	1454	0
XM_020102834.1	myeloid-associated differentiation marker homolog	1099	9.01E-166
XM_020102835.1	myeloid-associated differentiation marker homolog	957	0
XM_020102836.1	PREDICTED: uncharacterized protein LOC109639405 isoform X1	3086	0
XM_020102837.1	PREDICTED: uncharacterized protein LOC109639405 isoform X2	3050	0
XM_020102838.1	cytoplasmic phosphatidylinositol transfer protein 1-like isoform X1	2347	0
XM_020102839.1	cytoplasmic phosphatidylinositol transfer protein 1-like isoform X1	2386	0
XM_020102840.1	cytoplasmic phosphatidylinositol transfer protein 1-like isoform X1	2215	0
XM_020102841.1	leucine-rich repeat-containing protein 15-like	2129	0
XM_020102842.1	cytoplasmic phosphatidylinositol transfer protein 1-like isoform X1	2227	0
XM_020102843.1	aminoacyl tRNA synthase complex-interacting multifunctional protein 2 isoform X1	1606	0
XM_020102844.1	aminoacyl tRNA synthase complex-interacting multifunctional protein 2 isoform X2	1492	2.24E-169
XM_020102845.1	voltage-dependent calcium channel gamma-1 subunit-like isoform X1	2382	3.96E-136
XM_020102846.1	voltage-dependent calcium channel gamma-1 subunit-like isoform X2	2361	1.57E-130
XM_020102847.1	parvalbumin beta-like	881	1.50E-100
XM_020102848.1	parvalbumin beta-like	827	4.82E-68
XM_020102849.1	parvalbumin beta-like	1002	9.23E-71
XM_020102850.1	parvalbumin, thymic CPV3-like	796	2.86E-62
XM_020102851.1	serine/threonine-protein kinase LMTK2 isoform X1	7151	0
XM_020102852.1	serine/threonine-protein kinase LMTK2 isoform X2	7149	0
XM_020102853.1	sentrin-specific protease 5-like isoform X2	2390	0
XM_020102854.1	tectonin beta-propeller repeat-containing protein 1-like isoform X2	2737	0
XM_020102855.1	C-Jun-amino-terminal kinase-interacting protein 4-like isoform X1	6680	0
XM_020102856.1	C-Jun-amino-terminal kinase-interacting protein 4-like isoform X2	6676	0
XM_020102857.1	C-Jun-amino-terminal kinase-interacting protein 4-like isoform X3	6677	0
XM_020102858.1	copper-transporting ATPase 1	8914	0
XM_020102859.1	C-Jun-amino-terminal kinase-interacting protein 4-like isoform X4	6674	0
XM_020102860.1	C-Jun-amino-terminal kinase-interacting protein 4-like isoform X5	6668	0
XM_020102861.1	C-Jun-amino-terminal kinase-interacting protein 4-like isoform X6	6665	0
XM_020102862.1	C-Jun-amino-terminal kinase-interacting protein 4-like isoform X7	6652	0
XM_020102863.1	C-Jun-amino-terminal kinase-interacting protein 4-like isoform X8	6636	0
XM_020102864.1	sentrin-specific protease 5-like isoform X2	2286	0
XM_020102865.1	C-Jun-amino-terminal kinase-interacting protein 4-like isoform X9	6592	0
XM_020102866.1	C-Jun-amino-terminal kinase-interacting protein 4-like isoform X10	6646	0
XM_020102867.1	C-Jun-amino-terminal kinase-interacting protein 4-like isoform X11	6649	0
XM_020102868.1	interferon-induced protein 44-like	1831	0
XM_020102869.1	trinucleotide repeat-containing gene 6C protein-like isoform X2	5743	0
XM_020102870.1	trinucleotide repeat-containing gene 6C protein-like isoform X2	5563	0
XM_020102871.1	zona pellucida sperm-binding protein 3-like	1646	0
XM_020102872.1	PREDICTED: uncharacterized protein LOC109639421	1016	3.85E-178
XM_020102873.1	sentrin-specific protease 5-like isoform X2	2198	0
XM_020102874.1	ADP-ribosylation factor-binding protein GGA3	3857	0
XM_020102875.1	28S ribosomal protein S7, mitochondrial	1321	1.49E-170
XM_020102876.1	MIF4G domain-containing protein	2399	1.92E-157
XM_020102877.1	MIF4G domain-containing protein	2615	1.51E-156
XM_020102878.1	transportin-2-like isoform X1	5647	0
XM_020102879.1	transportin-2-like isoform X1	5643	0
XM_020102880.1	transportin-2-like isoform X1	5611	0
XM_020102881.1	small G protein signaling modulator 3	5114	0
XM_020102882.1	sentrin-specific protease 5-like isoform X2	2252	0
XM_020102883.1	GTP-binding protein 1-like isoform X1	3184	0
XM_020102884.1	GTP-binding protein 1-like isoform X2	3137	0
XM_020102885.1	GTP-binding protein 1-like isoform X3	3133	0
XM_020102886.1	GTP-binding protein 1-like isoform X4	3085	0
XM_020102887.1	voltage-dependent P/Q-type calcium channel subunit alpha-1A-like	5400	0
XM_020102888.1	voltage-dependent P/Q-type calcium channel subunit alpha-1A-like	4835	6.39E-106
XM_020102889.1	F-box-like/WD repeat-containing protein TBL1XR1 isoform X1	4469	0
XM_020102890.1	ATPase family AAA domain-containing protein 5-like	6275	0
XM_020102891.1	nuclear pore complex protein Nup85	2259	0
XM_020102892.1	small ubiquitin-related modifier 2 isoform X1	1180	1.06E-63
XM_020102893.1	small ubiquitin-related modifier 2 isoform X2	1062	6.97E-61
XM_020102894.1	brain-specific angiogenesis inhibitor 1-associated protein 2-like protein 1	2626	0
XM_020102895.1	brain protein I3	1217	2.19E-77
XM_020102896.1	brain protein I3	2439	1.94E-73
XM_020102897.1	F-box-like/WD repeat-containing protein TBL1XR1 isoform X1	4608	0
XM_020102898.1	neuronal-specific septin-3-like isoform X1	2607	0
XM_020102899.1	neuronal-specific septin-3-like isoform X2	2569	0

XM_020102900.1	neuronal-specific septin-3-like isoform X3	2725	0
XM_020102901.1	neuronal-specific septin-3-like isoform X4	2629	0
XM_020102902.1	neuronal-specific septin-3-like isoform X5	2544	0
XM_020102903.1	neuronal-specific septin-3-like isoform X1	1784	0
XM_020102904.1	PREDICTED: uncharacterized protein LOC109639440	2727	0
XM_020102905.1	mitochondrial Rho GTPase 1-A isoform X1	2336	0
XM_020102906.1	mitochondrial Rho GTPase 1-A isoform X2	2309	0
XM_020102907.1	F-box-like/WD repeat-containing protein TBL1XR1 isoform X1	4612	0
XM_020102908.1	mitochondrial Rho GTPase 1-A isoform X3	3252	0
XM_020102909.1	mitochondrial Rho GTPase 1-A isoform X4	2998	0
XM_020102910.1	protein Tob1-like	2522	0
XM_020102911.1	voltage-dependent T-type calcium channel subunit alpha-1I-like isoform X1	10095	0
XM_020102912.1	voltage-dependent T-type calcium channel subunit alpha-1I-like isoform X2	10092	0
XM_020102913.1	voltage-dependent T-type calcium channel subunit alpha-1I-like isoform X3	9945	0
XM_020102914.1	ankyrin repeat domain-containing protein 40	4821	0
XM_020102915.1	nucleus accumbens-associated protein 1-like isoform X3	5884	0
XM_020102916.1	F-box-like/WD repeat-containing protein TBL1XR1 isoform X1	4329	0
XM_020102917.1	nucleus accumbens-associated protein 1-like isoform X3	5883	0
XM_020102918.1	nucleus accumbens-associated protein 1-like isoform X3	2243	0
XM_020102919.1	nucleus accumbens-associated protein 1-like isoform X3	2110	0
XM_020102920.1	luc7-like protein 3	2253	0
XM_020102921.1	deoxyhypusine synthase	1763	0
XM_020102922.1	deoxyhypusine synthase	1787	0

XM_020102923.1	guanine nucleotide-binding protein G(I)/G(S)/G(O) subunit gamma-12-like	337	2.22E-48
XM_020102924.1	relaxin-3-like isoform X1	908	8.59E-91
XM_020102925.1	F-box-like/WD repeat-containing protein TBL1XR1 isoform X1	4469	0
XM_020102926.1	relaxin-3-like isoform X1	1142	2.51E-88
XM_020102927.1	far upstream element-binding protein 2 isoform X1	3594	0
XM_020102928.1	far upstream element-binding protein 2 isoform X2	3579	0
XM_020102929.1	protein Njmu-R1 isoform X1	2853	0
XM_020102930.1	protein Njmu-R1 isoform X2	2778	0
XM_020102931.1	F-box-like/WD repeat-containing protein TBL1XR1 isoform X1	4473	0
XM_020102932.1	reticulocalbin-3 isoform X1	1189	0
XM_020102933.1	reticulocalbin-3 isoform X2	1553	0
XM_020102934.1	nitric oxide synthase-interacting protein	1505	0
XM_020102935.1	nitric oxide synthase-interacting protein	1174	0
XM_020102936.1	methyltransferase-like protein 22	2172	0
XM_020102937.1	methyltransferase-like protein 22	2219	0
XM_020102938.1	F-box-like/WD repeat-containing protein TBL1XR1 isoform X1	4118	0
XM_020102939.1	prostatic spermine-binding protein-like isoform X2	1729	1.60E-43
XM_020102940.1	prostatic spermine-binding protein-like isoform X2	1572	7.31E-44
XM_020102941.1	bcl-2-like protein 12	2471	0
XM_020102942.1	PREDICTED: tetraspanin-4-like	2158	3.83E-127
XM_020102943.1	PREDICTED: tetraspanin-4-like	2201	5.66E-127
XM_020102944.1	junction plakoglobin-like	4754	0
XM_020102945.1	RNA binding protein fox-1 homolog 1	4090	0
XM_020102946.1	F-box-like/WD repeat-containing protein TBL1XR1 isoform X1	1734	0
XM_020102947.1	serine/threonine-protein kinase SMG1	11956	0
XM_020102948.1	dynein heavy chain 10, axonemal-like	1624	0
XM_020102949.1	carbonic anhydrase-like	1649	5.04E-147
XM_020102950.1	hydroxyacid oxidase 2 isoform X5	1343	0
XM_020102951.1	transmembrane gamma-carboxyglutamic acid protein 2 isoform X1	2025	4.28E-112
XM_020102952.1	transmembrane gamma-carboxyglutamic acid protein 2 isoform X2	2016	9.43E-110
XM_020102953.1	transmembrane gamma-carboxyglutamic acid protein 2 isoform X3	685	8.55E-123
XM_020102954.1	AP-2 complex subunit alpha-1 isoform X1	6036	0
XM_020102955.1	AP-2 complex subunit alpha-1 isoform X2	5973	0
XM_020102956.1	AP-2 complex subunit alpha-1 isoform X3	5910	0
XM_020102957.1	Y+L amino acid transporter 2-like	3001	0
XM_020102958.1	U1 small nuclear ribonucleoprotein 70 kDa	2189	8.48E-116
XM_020102959.1	PHD and RING finger domain-containing protein 1 isoform X1	5765	0
XM_020102960.1	neuronal-specific septin-3-like isoform X2	4218	0
XM_020102961.1	neuronal-specific septin-3-like isoform X2	2710	0
XM_020102962.1	neuronal-specific septin-3-like isoform X2	2586	0
XM_020102963.1	multidrug resistance-associated protein 1-like isoform X1	5427	0
XM_020102964.1	multidrug resistance-associated protein 1-like isoform X2	5424	0
XM_020102965.1	multidrug resistance-associated protein 1-like isoform X3	5370	0
XM_020102966.1	Y+L amino acid transporter 2-like	3053	0
XM_020102967.1	ornithine aminotransferase, mitochondrial	3350	0
XM_020102968.1	protein arginine N-methyltransferase 1 isoform X1	1756	0
XM_020102969.1	protein arginine N-methyltransferase 1 isoform X2	1721	0
XM_020102970.1	F-box/LRR-repeat protein 16	4867	0
XM_020102971.1	sarcalumenin-like isoform X2	2022	0
XM_020102972.1	epithelial membrane protein 2-like	3390	2.46E-94
XM_020102973.1	cytosolic Fe-S cluster assembly factor NUBP1	1010	0
XM_020102974.1	voltage-dependent calcium channel gamma-3 subunit	3110	0
XM_020102975.1	activating transcription factor 7-interacting protein 2 isoform X1	1797	0
XM_020102976.1	activating transcription factor 7-interacting protein 2 isoform X2	1794	0
XM_020102977.1	centromere protein F	6200	0
XM_020102978.1	arf-GAP domain and FG repeat-containing protein 1-like isoform X1	1890	0

XM_020102979.1	activating transcription factor 7-interacting protein 2 isoform X3	1981	0
XM_020102980.1	PREDICTED: uncharacterized protein LOC109639488 isoform X1	2008	5.46E-167
XM_020102981.1	PREDICTED: uncharacterized protein LOC109639488 isoform X2	2000	3.86E-166
XM_020102982.1	cerebellar degeneration-related protein 2-like	2542	0
XM_020102983.1	butyrophilin subfamily 3 member A3-like isoform X1	1761	0
XM_020102984.1	butyrophilin subfamily 3 member A3-like isoform X2	1758	0
XM_020102985.1	platelet endothelial cell adhesion molecule-like	2650	0
XM_020102986.1	protein phosphatase 1 regulatory subunit 27-like	1796	3.56E-142
XM_020102987.1	arf-GAP domain and FG repeat-containing protein 1-like isoform X2	1783	0
XM_020102988.1	neuronal pentraxin-1-like	2285	0
XM_020102989.1	protein kinase C beta type-like	5913	0
XM_020102990.1	voltage-dependent T-type calcium channel subunit alpha-1H-like	5934	0
XM_020102991.1	Na(+)/H(+) exchange regulatory cofactor NHE-RF1-like	1842	1.35E-85
XM_020102992.1	28S ribosomal protein S34, mitochondrial-like	1000	3.54E-132
XM_020102993.1	mapk-regulated corepressor-interacting protein 1 isoform X1	959	2.42E-63
XM_020102994.1	mapk-regulated corepressor-interacting protein 1 isoform X1	952	2.27E-63
XM_020102995.1	mapk-regulated corepressor-interacting protein 1 isoform X1	944	6.04E-60
XM_020102996.1	suppressor of cytokine signaling 1b	1916	0
XM_020102997.1	DBIRD complex subunit ZNF326	2549	0
XM_020102998.1	GTP-binding protein Rhes-like	1376	1.83E-151
XM_020102999.1	sphingosine kinase 1-like	2169	0
XM_020103000.1	PREDICTED: cytoglobin-1-like	1111	8.81E-132
XM_020103001.1	PREDICTED: cytoglobin-1-like	1205	3.01E-131
XM_020103002.1	mitogen-activated protein kinase 1	4312	0
XM_020103003.1	epsin-2 isoform X1	4906	0
XM_020103004.1	epsin-2 isoform X1	4943	0
XM_020103005.1	epsin-2 isoform X1	4825	0
XM_020103006.1	B9 domain-containing protein 1	1047	1.56E-156
XM_020103007.1	protodermal factor 1-like	5373	3.20E-100
XM_020103008.1	sarcoplasmic/endoplasmic reticulum calcium ATPase 1-like isoform X1	4065	0
XM_020103009.1	neuromedin-U receptor 1-like	1742	0
XM_020103010.1	sarcoplasmic/endoplasmic reticulum calcium ATPase 1-like isoform X2	4110	0
XM_020103011.1	rab GTPase-binding effector protein 2	2207	0
XM_020103012.1	protein FAM83G-like isoform X1	5692	0
XM_020103013.1	flocculation protein FLO11-like isoform X2	5524	0
XM_020103014.1	GRB2-related adapter protein-like	1299	1.44E-161
XM_020103015.1	trinucleotide repeat-containing gene 6A protein isoform X1	6311	0
XM_020103016.1	trinucleotide repeat-containing gene 6A protein isoform X1	6094	0
XM_020103017.1	trinucleotide repeat-containing gene 6A protein isoform X1	6262	0
XM_020103018.1	rho GDP-dissociation inhibitor 1	2216	5.46E-129
XM_020103019.1	E3 ubiquitin-protein ligase RBBP6-like isoform X1	6250	0
XM_020103020.1	E3 ubiquitin-protein ligase RBBP6-like isoform X2	6247	0
XM_020103021.1	serine/threonine-protein kinase 25	2008	0
XM_020103022.1	E3 ubiquitin-protein ligase RBBP6-like isoform X1	6176	0
XM_020103023.1	ATP-dependent DNA helicase Q5	4274	0
XM_020103024.1	phosphoribosyl pyrophosphate synthase-associated protein 1 isoform X1	3934	0
XM_020103025.1	phosphoribosyl pyrophosphate synthase-associated protein 1 isoform X2	3887	0
XM_020103026.1	THO complex subunit 4	1225	2.77E-99
XM_020103027.1	protein CLEC16A isoform X1	3852	0
XM_020103028.1	protein CLEC16A isoform X2	3813	0
XM_020103029.1	protein CLEC16A isoform X3	3807	0
XM_020103030.1	protein CLEC16A isoform X4	3786	0
XM_020103031.1	protein CLEC16A isoform X5	3714	0
XM_020103032.1	glycerophosphodiester phosphodiesterase domain-containing protein 3-like	2414	0
XM_020103033.1	eukaryotic initiation factor 4A-II	2044	0
XM_020103034.1	protein yippee-like 2	1821	2.01E-81
XM_020103035.1	V-type proton ATPase 16 kDa proteolipid subunit	1665	1.53E-81
XM_020103036.1	SPRY domain-containing SOCS box protein 3-like isoform X1	3439	0
XM_020103037.1	SPRY domain-containing SOCS box protein 3-like isoform X1	3284	0
XM_020103038.1	SPRY domain-containing SOCS box protein 3-like isoform X1	3414	0
XM_020103039.1	SPRY domain-containing SOCS box protein 3-like isoform X1	3279	0
XM_020103040.1	MHC class II transactivator	5715	0
XM_020103041.1	dexamethasone-induced protein	1543	1.75E-52
XM_020103042.1	SEC14-like protein 1	2700	0
XM_020103043.1	alpha-1,6-mannosylglycoprotein 6-beta-N-acetylglucosaminyltransferase B-like isoform X2	3733	0
XM_020103044.1	alpha-1,6-mannosylglycoprotein 6-beta-N-acetylglucosaminyltransferase B-like isoform X2	3726	0
XM_020103045.1	zinc finger protein Gfi-1	1569	0
XM_020103046.1	fas-binding factor 1 isoform X1	4036	0
XM_020103047.1	fas-binding factor 1 isoform X2	3865	0
XM_020103048.1	fas-binding factor 1 isoform X3	3862	0
XM_020103049.1	ADP-ribose pyrophosphatase, mitochondrial isoform X1	2707	0
XM_020103050.1	ADP-ribose pyrophosphatase, mitochondrial isoform X1	2635	0
XM_020103051.1	glutamate receptor ionotropic, NMDA 2A	7847	0
XM_020103052.1	glutamate receptor ionotropic, NMDA 2A-like	1071	2.94E-130
XM_020103053.1	nucleoside diphosphate kinase A-like isoform X1	1862	1.72E-127
XM_020103054.1	nucleoside diphosphate kinase 3-like isoform X2	1498	2.66E-120
XM_020103055.1	coiled-coil domain-containing protein 85C-A-like isoform X1	4855	0
XM_020103056.1	coiled-coil domain-containing protein 85C-A-like isoform X2	4756	4.35E-180
XM_020103057.1	lactosylceramide 1,3-N-acetyl-beta-D-glucosaminyltransferase A-like	2411	0

XM_020103058.1	B-cell lymphoma/leukemia 11B isoform X1	4537	0
XM_020103059.1	B-cell lymphoma/leukemia 11B isoform X2	3245	0
XM_020103060.1	B-cell lymphoma/leukemia 11B isoform X2	2534	0
XM_020103061.1	isthmin-1 isoform X1	2772	0
XM_020103062.1	HHIP-like protein 1	2948	0
XM_020103063.1	serine/threonine-protein kinase VRK1	1728	0
XM_020103064.1	protein phosphatase 1 regulatory subunit 37-like	747	0
XM_020103065.1	adenylate kinase 7-like	2132	0
XM_020103066.1	GTPase IMAP family member 7-like	552	3.91E-124
XM_020103067.1	PREDICTED: nesprin-3	2391	0
XM_020103068.1	homeobox protein goosecoid	726	2.37E-149
XM_020103069.1	histidine-rich glycoprotein-like	1419	0
XM_020103070.1	melanoma differentiation-associated protein 5	6159	0
XM_020103071.1	galactocerebrosidase isoform X1	2936	0
XM_020103072.1	galactocerebrosidase isoform X2	2932	0
XM_020103073.1	histone-lysine N-methyltransferase setd3	6494	0
XM_020103074.1	histone-lysine N-methyltransferase setd3	6491	0
XM_020103075.1	noelin-2-like isoform X1	2530	0
XM_020103076.1	noelin-2-like isoform X1	1970	0
XM_020103077.1	THUMP domain-containing protein 1	1757	0
XM_020103078.1	acid ceramidase	1798	0
XM_020103079.1	septin-2B isoform X1	2372	0
XM_020103080.1	IQ domain-containing protein E	3569	0
XM_020103081.1	IQ domain-containing protein E	3533	0
XM_020103082.1	putative RNA exonuclease NEF-sp	2673	0
XM_020103083.1	putative RNA exonuclease NEF-sp	2636	0
XM_020103084.1	storkhead-box protein 1	3309	0
XM_020103085.1	galanin receptor type 2-like	1893	0
XM_020103086.1	collagen alpha-1(XI) chain-like	6178	0
XM_020103087.1	proto-oncogene vav-like	2711	0
XM_020103088.1	septin-2B isoform X2	2333	0
XM_020103089.1	microfibril-associated glycoprotein 4-like	1268	4.83E-166
XM_020103090.1	protein FRG1 isoform X1	715	4.39E-134
XM_020103091.1	protein FRG1 isoform X2	693	6.97E-134
XM_020103092.1	protein FRG1 isoform X1	576	8.91E-122
XM_020103093.1	protein ELFN1-like	3201	0
XM_020103094.1	probable phospholipid-transporting ATPase VB	4699	0
XM_020103095.1	intercellular adhesion molecule 5-like	1455	0
XM_020103096.1	ERI1 exoribonuclease 2	1047	8.89E-175
XM_020103097.1	protein FAM183B-like	1100	1.88E-83
XM_020103098.1	heparan sulfate 2-O-sulfotransferase 1	2049	0
XM_020103099.1	actin, cytoplasmic 2	1772	0
XM_020103100.1	pericentriolar material 1 protein isoform X1	7173	0
XM_020103101.1	pericentriolar material 1 protein isoform X1	7170	0
XM_020103102.1	pericentriolar material 1 protein isoform X1	7586	0
XM_020103103.1	pericentriolar material 1 protein isoform X1	7055	0
XM_020103104.1	pericentriolar material 1 protein isoform X1	7169	0
XM_020103105.1	pericentriolar material 1 protein isoform X1	7115	0
XM_020103106.1	pericentriolar material 1 protein isoform X1	7072	0
XM_020103107.1	pericentriolar material 1 protein isoform X1	7005	0
XM_020103108.1	U4/U6.U5 tri-snRNP-associated protein 1	2755	0
XM_020103109.1	protocadherin Fat 1 isoform X1	15325	0
XM_020103110.1	protocadherin Fat 1 isoform X2	15289	0
XM_020103111.1	protocadherin Fat 1 isoform X3	15256	0
XM_020103112.1	V-type proton ATPase subunit d 1-like	1080	0
XM_020103113.1	protocadherin Fat 1 isoform X4	15220	0
XM_020103114.1	BRCA1-associated ATM activator 1 isoform X1	3012	0
XM_020103115.1	BRCA1-associated ATM activator 1 isoform X2	3009	0
XM_020103116.1	eukaryotic translation initiation factor 3 subunit B	2768	0
XM_020103117.1	carbohydrate sulfotransferase 12 isoform X1	1897	0
XM_020103118.1	carbohydrate sulfotransferase 12 isoform X1	2037	0
XM_020103119.1	carbohydrate sulfotransferase 12 isoform X1	1870	0
XM_020103120.1	breast cancer anti-estrogen resistance protein 3-like isoform X3	3066	0
XM_020103121.1	breast cancer anti-estrogen resistance protein 3-like isoform X3	2646	0
XM_020103122.1	breast cancer anti-estrogen resistance protein 3-like isoform X3	2659	0
XM_020103123.1	breast cancer anti-estrogen resistance protein 3-like isoform X3	2585	0
XM_020103124.1	breast cancer anti-estrogen resistance protein 3-like isoform X3	2685	0
XM_020103125.1	probable flap endonuclease 1 homolog	1960	0
XM_020103126.1	peptidyl-prolyl cis-trans isomerase NIMA-interacting 1	1389	1.07E-111
XM_020103127.1	ubiquitin-like protein 5	465	7.30E-49
XM_020103128.1	protein FAM83G-like	4435	0
XM_020103129.1	sodium/glucose cotransporter 5-like	2454	0
XM_020103130.1	GRB2-related adapter protein-like	2353	0
XM_020103131.1	axin-1 isoform X1	6017	0
XM_020103132.1	oxysterol-binding protein 1-like isoform X1	4128	0
XM_020103133.1	oxysterol-binding protein 1-like isoform X1	4166	0
XM_020103134.1	oxysterol-binding protein 1-like isoform X1	4192	0
XM_020103135.1	oxysterol-binding protein 1-like isoform X1	4205	0
XM_020103136.1	oxysterol-binding protein 1-like isoform X1	4149	0

XM_020103137.1	UDP-N-acetylglucosamine transporter-like	2116	0
XM_020103138.1	oxysterol-binding protein 1-like isoform X1	4125	0
XM_020103139.1	oxysterol-binding protein 1-like isoform X1	4119	0
XM_020103140.1	oxysterol-binding protein 1-like isoform X1	4089	0
XM_020103141.1	oxysterol-binding protein 1-like isoform X1	4089	0
XM_020103142.1	oxysterol-binding protein 1-like isoform X1	4056	0
XM_020103143.1	oxysterol-binding protein 1-like isoform X1	4041	0
XM_020103144.1	oxysterol-binding protein 1-like isoform X8	4017	0
XM_020103145.1	oxysterol-binding protein 1-like isoform X9	4002	0
XM_020103146.1	oxysterol-binding protein 1-like isoform X10	3969	0
XM_020103147.1	oxysterol-binding protein 1-like isoform X11	3966	0
XM_020103148.1	oxysterol-binding protein 1-like isoform X12	3960	0
XM_020103149.1	cdc42-interacting protein 4 homolog isoform X1	4177	0
XM_020103150.1	cdc42-interacting protein 4 homolog isoform X2	4153	0
XM_020103151.1	cdc42-interacting protein 4 homolog isoform X1	3761	0
XM_020103152.1	UAP56-interacting factor	1513	3.39E-175
XM_020103153.1	general transcription factor 3C polypeptide 1	7208	0
XM_020103154.1	protein tweety homolog 3-like isoform X1	4924	0
XM_020103155.1	protein tweety homolog 3-like isoform X2	4461	0
XM_020103156.1	protein tweety homolog 3-like isoform X3	4452	0
XM_020103157.1	caspase 3	2932	0
XM_020103158.1	mitotic spindle assembly checkpoint protein MAD1	4065	0
XM_020103159.1	poly(A)-specific ribonuclease PARN	2346	0
XM_020103160.1	retinol dehydrogenase 8-like	1279	0
XM_020103161.1	beta-1,3-N-acetylglucosaminyltransferase lunatic fringe	2986	0
XM_020103162.1	adhesion G protein-coupled receptor L4	5001	0
XM_020103163.1	mitochondrial fission factor homolog A-like	2891	9.04E-150
XM_020103164.1	unconventional myosin-VIIa-like	5913	0
XM_020103165.1	probable polyketide synthase 1	6339	0
XM_020103166.1	probable polyketide synthase 1	6291	0
XM_020103167.1	probable G-protein coupled receptor 158	3495	0
XM_020103168.1	adhesion G-protein coupled receptor G2	2068	0
XM_020103169.1	collagen alpha-1(XV) chain-like	3848	0
XM_020103170.1	retinol dehydrogenase 12-like	885	5.22E-177
XM_020103171.1	mitochondrial fission factor homolog A-like	3081	4.35E-149
XM_020103172.1	PREDICTED: uncharacterized protein C8orf59 homolog	1054	2.92E-39
XM_020103173.1	adhesion G protein-coupled receptor L4	3426	0
XM_020103174.1	amyloid beta A4 precursor protein-binding family B member 1-interacting protein	2744	0
XM_020103175.1	selenoprotein O-like isoform X3	2369	0
XM_020103176.1	selenoprotein O-like isoform X3	2363	0
XM_020103177.1	abl interactor 1 isoform X1	3245	0
XM_020103178.1	abl interactor 1 isoform X2	3227	0
XM_020103179.1	abl interactor 1 isoform X3	3156	0
XM_020103180.1	abl interactor 1 isoform X4	3105	0
XM_020103181.1	abl interactor 1 isoform X5	3087	0
XM_020103182.1	mitochondrial fission factor homolog A-like	1375	1.65E-156
XM_020103183.1	abl interactor 1 isoform X7	2871	3.21E-162
XM_020103184.1	abl interactor 1 isoform X7	2732	0
XM_020103185.1	abl interactor 1 isoform X8	2712	0
XM_020103186.1	abl interactor 1 isoform X9	3016	0
XM_020103187.1	abl interactor 1 isoform X10	2998	0
XM_020103188.1	acyl-CoA-binding domain-containing protein 5 isoform X1	3356	0
XM_020103189.1	acyl-CoA-binding domain-containing protein 5 isoform X1	3118	0
XM_020103190.1	acyl-CoA-binding domain-containing protein 5 isoform X1	3366	0
XM_020103191.1	acyl-CoA-binding domain-containing protein 5 isoform X1	3358	0
XM_020103192.1	decaprenyl-diphosphate synthase subunit 1	2333	0
XM_020103193.1	apolipoprotein D-like	1016	2.69E-125
XM_020103194.1	apolipoprotein D-like	1019	1.40E-130
XM_020103195.1	coiled-coil domain-containing protein 106-like	1318	0
XM_020103196.1	SET domain-containing protein 9	1063	0
XM_020103197.1	toll-like receptor 3	3219	0
XM_020103198.1	connector enhancer of kinase suppressor of ras 2	3078	0
XM_020103199.1	kelch-like protein 34	2776	0
XM_020103200.1	mitogen-activated protein kinase kinase kinase 15	1798	4.01E-169
XM_020103201.1	C-C motif chemokine 20-like	627	8.45E-70
XM_020103202.1	small muscular protein	1222	8.59E-57
XM_020103203.1	small muscular protein	1226	8.81E-57
XM_020103204.1	small muscular protein	1269	1.71E-56
XM_020103205.1	small muscular protein	651	2.67E-59
XM_020103206.1	small muscular protein	648	2.57E-59
XM_020103207.1	small muscular protein	551	7.81E-60
XM_020103208.1	protein FAM78B-like	3531	0
XM_020103209.1	serine/threonine-protein kinase 38	3248	0
XM_020103210.1	ATP-dependent zinc metalloprotease YME1L1 isoform X1	3136	0
XM_020103211.1	ATP-dependent zinc metalloprotease YME1L1 isoform X2	3112	0
XM_020103212.1	ATP-dependent zinc metalloprotease YME1L1 isoform X3	3127	0
XM_020103213.1	dipeptidyl aminopeptidase-like protein 6 isoform X1	4078	0
XM_020103214.1	dipeptidyl aminopeptidase-like protein 6 isoform X2	3972	0
XM_020103215.1	dipeptidyl aminopeptidase-like protein 6 isoform X3	4329	0

XM_020103216.1	dipeptidyl aminopeptidase-like protein 6 isoform X4	4005	0
XM_020103217.1	dipeptidyl aminopeptidase-like protein 6 isoform X5	3975	0
XM_020103218.1	dipeptidyl aminopeptidase-like protein 6 isoform X6	4063	0
XM_020103219.1	gamma-glutamyl hydrolase-like	1474	0
XM_020103220.1	carbonic anhydrase 1	2614	0
XM_020103221.1	heat shock 70 kDa protein 4L	3130	0
XM_020103222.1	sodium/potassium-transporting ATPase subunit beta-3	2533	0
XM_020103223.1	annexin A13-like isoform X1	2592	0
XM_020103224.1	annexin A13-like isoform X2	2589	0
XM_020103225.1	transferrin receptor protein 1-like	4740	0
XM_020103226.1	RNA-binding Raly-like protein isoform X1	3945	3.79E-172
XM_020103227.1	RNA-binding Raly-like protein isoform X2	3945	2.67E-171
XM_020103228.1	RNA-binding Raly-like protein isoform X3	4700	9.28E-166
XM_020103229.1	RNA-binding Raly-like protein isoform X3	3949	1.51E-167
XM_020103230.1	RNA-binding Raly-like protein isoform X4	3933	8.79E-174
XM_020103231.1	RNA-binding Raly-like protein isoform X3	3426	3.90E-108
XM_020103232.1	membrane-bound transcription factor site-2 protease	2256	0
XM_020103233.1	rho GTPase-activating protein 21-like	4802	0
XM_020103234.1	glutamate decarboxylase 1-like	6186	0
XM_020103235.1	DCN1-like protein 1	1383	0
XM_020103236.1	Fanconi anemia core complex-associated protein 20	4103	5.94E-166
XM_020103237.1	PREDICTED: uncharacterized protein LOC109639647	2481	0
XM_020103238.1	mitochondrial carrier homolog 1 isoform X1	2071	0
XM_020103239.1	mitochondrial carrier homolog 1 isoform X1	1686	0
XM_020103240.1	protein kinase C zeta type isoform X1	4049	0
XM_020103241.1	protein kinase C zeta type isoform X2	3583	0
XM_020103242.1	ligand-dependent nuclear receptor-interacting factor 1 isoform X1	5271	0
XM_020103243.1	ligand-dependent nuclear receptor-interacting factor 1 isoform X2	5193	0
XM_020103244.1	deoxyribonuclease gamma-like	1073	3.27E-89
XM_020103245.1	insulin-like growth factor I	1835	1.89E-127
XM_020103246.1	claudin-19 isoform X1	824	2.48E-143
XM_020103247.1	nudix hydrolase 24, chloroplastic-like	956	0
XM_020103248.1	peroxisome biogenesis factor 10	1350	0
XM_020103249.1	probable glutamate receptor	1813	0
XM_020103250.1	proenkephalin-B isoform X1	1026	1.91E-135

XM_020103251.1	proenkephalin-B isoform X2	998	3.11E-125
XM_020103252.1	serine/threonine-protein kinase 35-like	5278	0
XM_020103253.1	1-phosphatidylinositol 4,5-bisphosphate phosphodiesterase eta-2 isoform X1	5774	0
XM_020103254.1	1-phosphatidylinositol 4,5-bisphosphate phosphodiesterase eta-2 isoform X1	3580	0
XM_020103255.1	1-phosphatidylinositol 4,5-bisphosphate phosphodiesterase eta-2 isoform X3	3419	0
XM_020103256.1	serine/threonine-protein kinase 38	3967	0
XM_020103257.1	serine/threonine-protein kinase 38	4124	0
XM_020103258.1	Krueppel-like factor 13	5560	3.91E-122
XM_020103259.1	ski oncogene	4646	0
XM_020103260.1	glucocorticoid modulatory element-binding protein 2-like	2547	0
XM_020103261.1	glucocorticoid modulatory element-binding protein 2-like	2362	0
XM_020103262.1	glucocorticoid modulatory element-binding protein 2-like	2454	0
XM_020103263.1	glucocorticoid modulatory element-binding protein 2-like	2458	0
XM_020103264.1	targeting protein for Xklp2 isoform X1	2472	0
XM_020103265.1	targeting protein for Xklp2 isoform X1	2398	0
XM_020103266.1	targeting protein for Xklp2 isoform X1	2437	0
XM_020103267.1	targeting protein for Xklp2 isoform X1	2454	0
XM_020103268.1	N-acylglucosamine 2-epimerase	1388	0
XM_020103269.1	suppression of tumorigenicity 5 protein	2132	0
XM_020103270.1	suppression of tumorigenicity 5 protein	2217	0
XM_020103271.1	transcription factor HES-1-B-like	1166	3.78E-172
XM_020103272.1	protein crumbs homolog 2-like	1716	1.11E-150
XM_020103273.1	tumor necrosis factor receptor superfamily member 14 isoform X1	3629	3.42E-139
XM_020103274.1	tumor necrosis factor receptor superfamily member 14 isoform X2	1263	3.73E-116
XM_020103275.1	endonuclease domain-containing 1 protein-like	2053	0
XM_020103276.1	endonuclease domain-containing 1 protein-like	1471	0
XM_020103277.1	inactive Ufm1-specific protease 1	1257	0
XM_020103278.1	fibroblast growth factor 14-like	800	6.90E-85
XM_020103279.1	zona pellucida sperm-binding protein 3-like	1087	0
XM_020103280.1	F-box only protein 36	1373	8.49E-175
XM_020103281.1	endonuclease domain-containing 1 protein-like	615	9.66E-156
XM_020103282.1	PREDICTED: ephrin-B2-like	2020	4.55E-116
XM_020103283.1	serpin H1-like isoform X2	1192	0
XM_020103284.1	dynein heavy chain 2, axonemal-like	1759	0
XM_020103285.1	lysine-specific demethylase 6B-like	7136	0
XM_020103286.1	neuronal acetylcholine receptor subunit beta-2-like isoform X2	2367	0
XM_020103287.1	fibroblast growth factor 11-like isoform X1	1585	6.49E-179
XM_020103288.1	fibroblast growth factor 11-like isoform X1	1633	0
XM_020103289.1	fibroblast growth factor 14-like isoform X3	1035	1.27E-132
XM_020103290.1	acetylcholine receptor subunit beta-like	1675	0
XM_020103291.1	calcium-activated potassium channel subunit beta-2-like	1519	9.28E-176
XM_020103292.1	prostaglandin D2 receptor 2-like	2103	0
XM_020103293.1	cornifelin homolog B-like	1948	7.85E-81
XM_020103294.1	cornifelin homolog B-like	779	5.04E-86

XM_020103295.1	PREDICTED: neuroligin-2	7656	0
XM_020103296.1	calcium-activated potassium channel subunit beta-2-like	1219	2.34E-177
XM_020103297.1	PREDICTED: LOW QUALITY PROTEIN: erythropoietin	1789	2.74E-126
XM_020103298.1	ribonuclease P protein subunit p20	1341	9.69E-68
XM_020103299.1	ribonuclease P protein subunit p20	1393	1.56E-67
XM_020103300.1	ribonuclease P protein subunit p20	1247	4.14E-68
XM_020103301.1	protein MB21D2-like	5128	0
XM_020103302.1	transmembrane protein 256	1112	1.08E-60
XM_020103303.1	telomerase Cajal body protein 1	3277	0
XM_020103304.1	UMP-CMP kinase	1496	3.92E-159
XM_020103305.1	PERQ amino acid-rich with GYF domain-containing protein 1 isoform X1	6752	0
XM_020103306.1	PERQ amino acid-rich with GYF domain-containing protein 1 isoform X2	4897	0
XM_020103307.1	PERQ amino acid-rich with GYF domain-containing protein 1 isoform X3	4881	0
XM_020103308.1	PERQ amino acid-rich with GYF domain-containing protein 1 isoform X4	6708	0
XM_020103309.1	PERQ amino acid-rich with GYF domain-containing protein 1 isoform X5	4857	0
XM_020103310.1	claudin-7-B-like isoform X2	2445	4.76E-130
XM_020103311.1	dynein heavy chain 2, axonemal	11624	0
XM_020103312.1	serpin H1-like isoform X2	626	2.47E-53
XM_020103313.1	cholecystokinin receptor-like	7127	0
XM_020103314.1	ephrin type-B receptor 3-like	1130	1.56E-160
XM_020103315.1	carboxypeptidase O isoform X1	1279	0
XM_020103316.1	carboxypeptidase O isoform X2	1177	0
XM_020103317.1	carboxypeptidase O isoform X1	1235	0
XM_020103318.1	nucleotide triphosphate diphosphatase NUDT15	545	3.63E-101
XM_020103319.1	nucleotide triphosphate diphosphatase NUDT15	549	4.16E-101
XM_020103320.1	splicing factor U2AF 35 kDa subunit-like isoform X1	911	6.98E-116
XM_020103321.1	splicing factor U2AF 35 kDa subunit-like isoform X1	914	8.43E-105
XM_020103322.1	OX-2 membrane glycoprotein-like	1864	0
XM_020103323.1	B- and T-lymphocyte attenuator-like	1623	0
XM_020103324.1	DNA annealing helicase and endonuclease ZRANB3	4256	0
XM_020103325.1	vesicle-trafficking protein SEC22b-like	1200	7.92E-155
XM_020103326.1	glucokinase regulatory protein	2141	0
XM_020103327.1	retinitis pigmentosa 1-like 1 protein	7774	0
XM_020103328.1	trace amine-associated receptor 13c-like	951	0
XM_020103329.1	putative E3 ubiquitin-protein ligase SH3RF2	3279	0
XM_020103330.1	trace amine-associated receptor 13c-like	600	3.90E-91
XM_020103331.1	cilia- and flagella-associated protein 221	2755	0
XM_020103332.1	secretin receptor-like	1460	0

XM_020103333.1	vascular endothelial growth factor C-like	2259	0
XM_020103334.1	PREDICTED: uncharacterized protein C8orf74 homolog	1120	0
XM_020103335.1	XK-related protein 6-like	672	6.80E-108
XM_020103336.1	neurobeachin-like protein 1	11320	0
XM_020103337.1	claudin-7-B-like isoform X2	822	1.45E-141
XM_020103338.1	paraspeckle component 1-like	2654	0
XM_020103339.1	methylmalonic aciduria and homocystinuria type D homolog, mitochondrial-like	1007	0
XM_020103340.1	methylmalonic aciduria and homocystinuria type D homolog, mitochondrial-like	1100	0
XM_020103341.1	ly6/PLAUR domain-containing protein 6-like	784	1.47E-105
XM_020103342.1	ly6/PLAUR domain-containing protein 6-like	727	6.05E-106
XM_020103343.1	ly6/PLAUR domain-containing protein 6-like	1324	3.86E-103
XM_020103344.1	ly6/PLAUR domain-containing protein 6-like	1810	4.76E-101
XM_020103345.1	trafficking kinesin-binding protein 2 isoform X1	5280	0
XM_020103346.1	trafficking kinesin-binding protein 2 isoform X2	5218	0
XM_020103347.1	E3 ubiquitin/ISG15 ligase TRIM25-like isoform X1	2913	0
XM_020103348.1	E3 ubiquitin/ISG15 ligase TRIM25-like isoform X2	2847	0
XM_020103349.1	STE20-related kinase adapter protein beta isoform X1	3254	0
XM_020103350.1	STE20-related kinase adapter protein beta isoform X1	3051	0
XM_020103351.1	STE20-related kinase adapter protein beta isoform X2	3177	0
XM_020103352.1	STE20-related kinase adapter protein beta isoform X2	3116	0
XM_020103353.1	R3H domain-containing protein 2-like isoform X1	1800	0
XM_020103354.1	R3H domain-containing protein 2-like isoform X1	830	4.23E-130
XM_020103355.1	rho-related GTP-binding protein RhoE-like	2723	2.15E-160
XM_020103356.1	zinc finger protein 654	8334	0
XM_020103357.1	gamma-aminobutyric acid receptor subunit beta-3-like isoform X1	5048	0
XM_020103358.1	gamma-aminobutyric acid receptor subunit beta-3-like isoform X2	1644	0
XM_020103359.1	gamma-aminobutyric acid receptor subunit beta-3-like isoform X3	5034	0
XM_020103360.1	gamma-aminobutyric acid receptor subunit beta-3-like isoform X4	1623	0
XM_020103361.1	gamma-aminobutyric acid receptor subunit beta-3-like isoform X3	5038	0
XM_020103362.1	15 kDa selenoprotein	1555	7.01E-79
XM_020103363.1	gamma-aminobutyric acid receptor subunit beta-3-like isoform X6	5227	0
XM_020103364.1	gamma-aminobutyric acid receptor subunit beta-3-like isoform X7	1802	0
XM_020103365.1	gamma-aminobutyric acid receptor subunit alpha-5	2310	0
XM_020103366.1	gamma-aminobutyric acid receptor subunit gamma-3-like	2498	0
XM_020103367.1	metalloreductase STEAP3	2862	0
XM_020103368.1	metalloreductase STEAP3	2259	0
XM_020103369.1	metalloreductase STEAP3	2709	0
XM_020103370.1	metalloreductase STEAP3	2869	0
XM_020103371.1	metalloreductase STEAP3	2342	0
XM_020103372.1	homeobox protein engrailed-1-B-like isoform X1	1366	1.15E-146
XM_020103373.1	homeobox protein engrailed-1-B-like isoform X2	1354	7.93E-144

XM_020103374.1	complement C1q-like protein 2	904	6.63E-151
XM_020103375.1	tRNA-splicing endonuclease subunit Sen15	1523	1.13E-91
XM_020103376.1	alpha-1,6-mannosylglycoprotein 6-beta-N-acetylglucosaminyltransferase A	5664	0
XM_020103377.1	transmembrane protein 163 isoform X1	2449	0
XM_020103378.1	transmembrane protein 163 isoform X2	2355	0
XM_020103379.1	transmembrane protein 163 isoform X1	2389	0
XM_020103380.1	transmembrane protein 163 isoform X3	2043	5.17E-136
XM_020103381.1	cyclin-T2-like isoform X2	2863	0
XM_020103382.1	cyclin-T2-like isoform X2	2851	0
XM_020103383.1	OX-2 membrane glycoprotein-like	2939	0
XM_020103384.1	OX-2 membrane glycoprotein-like	3071	0
XM_020103385.1	OX-2 membrane glycoprotein-like	2936	0
XM_020103386.1	OX-2 membrane glycoprotein-like	3068	0
XM_020103387.1	OX-2 membrane glycoprotein-like isoform X1	2219	0
XM_020103388.1	OX-2 membrane glycoprotein-like isoform X2	1657	0
XM_020103389.1	profilin-2-like isoform X1	2311	1.46E-94
XM_020103390.1	OX-2 membrane glycoprotein-like isoform X3	1638	0
XM_020103391.1	OX-2 membrane glycoprotein-like isoform X4	1631	0
XM_020103392.1	bis(5'-adenosyl)-triphosphatase ENPP4	1962	0
XM_020103393.1	chloride intracellular channel protein 5-like isoform X1	2358	0
XM_020103394.1	chloride intracellular channel protein 5-like isoform X2	2009	0
XM_020103395.1	cystathionine beta-synthase-like	2715	0
XM_020103396.1	proteoglycan 3-like isoform X4	1220	0
XM_020103397.1	proteoglycan 3-like isoform X4	1178	0
XM_020103398.1	profilin-2-like isoform X2	914	3.43E-99
XM_020103400.1	proteoglycan 3-like isoform X4	1213	0
XM_020103401.1	proteoglycan 3-like isoform X4	1225	0
XM_020103402.1	PREDICTED: uncharacterized protein LOC109639758 isoform X5	1094	0
XM_020103403.1	adrenodoxin, mitochondrial	1719	1.74E-113
XM_020103404.1	transmembrane protein 106B-like	2504	3.17E-174
XM_020103405.1	transmembrane protein 106B-like	2496	1.80E-175
XM_020103406.1	ankyrin repeat and MYND domain-containing protein 2	2221	0
XM_020103407.1	isoprenoid synthase domain-containing protein	2385	0
XM_020103408.1	von Willebrand factor D and EGF domain-containing protein	5223	0
XM_020103409.1	von Willebrand factor D and EGF domain-containing protein-like	998	1.85E-127
XM_020103410.1	PREDICTED: uncharacterized protein C1orf21 homolog	1651	7.28E-74
XM_020103411.1	collagen alpha-1(XXVIII) chain-like	4492	0
XM_020103412.1	elongator complex protein 6	1491	1.39E-163
XM_020103413.1	semaphorin-6D-like isoform X2	1350	0
XM_020103414.1	ubiquitin-conjugating enzyme E2 E2-like	618	3.06E-125
XM_020103415.1	PREDICTED: uncharacterized protein LOC109639771	1710	0

XM_020103416.1	thrombospondin type-1 domain-containing protein 7A	4358	0
XM_020103417.1	alkylglycerol monooxygenase	1418	0
XM_020103418.1	T-box transcription factor TBX20	4535	0
XM_020103419.1	homeodomain-interacting protein kinase 3-like	1374	0
XM_020103420.1	SWI/SNF complex subunit SMARCC1-like	1378	0
XM_020103421.1	chondroitin sulfate proteoglycan 5	2372	0
XM_020103422.1	sterol regulatory element-binding protein cleavage-activating protein	3721	0
XM_020103423.1	PREDICTED: uncharacterized protein LOC109639779	1216	0
XM_020103424.1	sclerostin domain-containing protein 1	1320	5.00E-139
XM_020103425.1	homeobox protein MOX-2-like	1510	1.79E-81
XM_020103426.1	serine/threonine-protein kinase Nek10	3588	0
XM_020103427.1	nuclear receptor subfamily 1 group D member 2-like	2703	0
XM_020103428.1	retinoic acid receptor beta-like isoform X1	2989	0
XM_020103429.1	retinoic acid receptor beta-like isoform X2	3256	0
XM_020103430.1	transcription factor Sox-14-like	902	1.12E-140
XM_020103431.1	retinoic acid receptor beta-like isoform X3	2849	0
XM_020103432.1	retinoic acid receptor beta-like isoform X3	2845	0
XM_020103433.1	macrophage mannose receptor 1-like isoform X1	2845	0
XM_020103434.1	septin-7 isoform X1	2621	0
XM_020103435.1	septin-7 isoform X2	2600	0
XM_020103436.1	protachykinin-like isoform X1	1152	1.15E-57
XM_020103437.1	protachykinin-like isoform X2	1128	1.09E-51
XM_020103438.1	PREDICTED: uncharacterized protein LOC109639789	902	0
XM_020103439.1	probable C-mannosyltransferase DPY19L1	4152	0
XM_020103440.1	glucocorticoid-induced transcript 1 protein-like isoform X1	3753	0
XM_020103441.1	glucocorticoid-induced transcript 1 protein-like isoform X2	3751	0
XM_020103442.1	islet cell autoantigen 1 isoform X1	3450	0
XM_020103443.1	PREDICTED: nucleolin-like	2131	0
XM_020103444.1	islet cell autoantigen 1 isoform X2	3324	0
XM_020103445.1	WD repeat-containing protein mio	4482	0
XM_020103446.1	WD repeat-containing protein mio	4339	0
XM_020103447.1	homocysteine-responsive endoplasmic reticulum-resident ubiquitin-like domain member 2 protein	2399	2.77E-163
XM_020103448.1	homocysteine-responsive endoplasmic reticulum-resident ubiquitin-like domain member 2 protein	2314	1.19E-163
XM_020103449.1	ubiquitin-conjugating enzyme E2 E1-like	1028	8.32E-113
XM_020103450.1	diacylglycerol kinase beta isoform X1	4558	0
XM_020103451.1	diacylglycerol kinase beta isoform X2	4537	0
XM_020103452.1	glucocorticoid receptor-like	2718	0
XM_020103453.1	ADP-ribosylation factor-like protein 4A	1763	1.82E-118

XM_020103454.1	excitatory amino acid transporter 1-like	2034	0
XM_020103455.1	excitatory amino acid transporter 1-like	2031	0
XM_020103456.1	glutamate receptor ionotropic, delta-2	2614	0
XM_020103457.1	U3 small nucleolar ribonucleoprotein protein IMP3	1344	2.78E-130
XM_020103458.1	Golgi membrane protein 1	4107	0
XM_020103459.1	BDNF/NT-3 growth factors receptor	2618	0
XM_020103460.1	BDNF/NT-3 growth factors receptor-like	1559	0
XM_020103461.1	kinesin-like protein KIF7 isoform X1	4205	0
XM_020103462.1	transducin-like enhancer protein 1	4484	0
XM_020103463.1	transmembrane emp24 domain-containing protein 7	1596	5.75E-108
XM_020103464.1	coiled-coil domain-containing protein 112	1862	0
XM_020103465.1	serine-rich coiled-coil domain-containing protein 1	3627	0
XM_020103466.1	UPF0553 protein C9orf64 homolog	1447	0
XM_020103467.1	solute carrier family 28 member 3 isoform X1	2105	0
XM_020103468.1	solute carrier family 28 member 3 isoform X1	1930	0
XM_020103469.1	ras and EF-hand domain-containing protein homolog	3799	0
XM_020103470.1	FERM domain-containing protein 3	2077	0
XM_020103471.1	trypsinogen 2 precursor	2023	3.19E-179
XM_020103472.1	protein fem-1 homolog C	4385	0
XM_020103473.1	histone-lysine N-methyltransferase 2C isoform X1	15711	0
XM_020103474.1	recQ-mediated genome instability protein 1 isoform X1	3777	0
XM_020103475.1	recQ-mediated genome instability protein 1 isoform X1	3525	0
XM_020103476.1	recQ-mediated genome instability protein 1 isoform X1	3752	0
XM_020103477.1	heterogeneous nuclear ribonucleoprotein K isoform X1	1531	0
XM_020103478.1	heterogeneous nuclear ribonucleoprotein K isoform X2	1526	0
XM_020103479.1	heterogeneous nuclear ribonucleoprotein K isoform X3	1484	0
XM_020103480.1	cytosolic carboxypeptidase 1 isoform X1	6030	0
XM_020103481.1	cytosolic carboxypeptidase 1 isoform X2	6027	0
XM_020103482.1	histone-lysine N-methyltransferase 2C isoform X2	15663	0
XM_020103483.1	N-alpha-acetyltransferase 35, NatC auxiliary subunit	2765	0
XM_020103484.1	ubiquilin-1 isoform X1	2831	0
XM_020103485.1	ubiquilin-1 isoform X2	2847	0
XM_020103486.1	G kinase-anchoring protein 1 isoform X1	1496	0
XM_020103487.1	G kinase-anchoring protein 1 isoform X1	1475	0
XM_020103488.1	G kinase-anchoring protein 1 isoform X1	1403	0
XM_020103489.1	probable gluconokinase	927	2.14E-134
XM_020103490.1	geranylgeranyl transferase type-1 subunit beta	2764	0
XM_020103491.1	histone-lysine N-methyltransferase 2C isoform X1	15611	0
XM_020103492.1	E3 ubiquitin-protein ligase TRIM36 isoform X1	5167	0
XM_020103493.1	E3 ubiquitin-protein ligase TRIM36 isoform X2	5155	0
XM_020103494.1	LMBR1 domain-containing protein 2-B-like isoform X1	3513	0
XM_020103495.1	LMBR1 domain-containing protein 2-B-like isoform X1	3559	0
XM_020103496.1	LMBR1 domain-containing protein 2-B-like isoform X1	3475	0
XM_020103497.1	LMBR1 domain-containing protein 2-B-like isoform X1	3507	0
XM_020103498.1	LMBR1 domain-containing protein 2-B-like isoform X1	3512	0
XM_020103499.1	LMBR1 domain-containing protein 2-B-like isoform X4	3507	0
XM_020103500.1	histone-lysine N-methyltransferase 2C isoform X4	15069	0
XM_020103501.1	PHD finger protein 24 isoform X1	2950	0
XM_020103502.1	PHD finger protein 24 isoform X2	1711	0
XM_020103503.1	leucine-rich repeat transmembrane neuronal protein 4-like	2674	0
XM_020103504.1	bromodomain-containing protein 3-like	1943	0
XM_020103505.1	guanine nucleotide exchange factor VAV2	5607	0
XM_020103506.1	phospholipase A2-like isoform X1	851	5.30E-96
XM_020103507.1	phospholipase A2-like isoform X2	1029	2.66E-84
XM_020103508.1	histone-lysine N-methyltransferase 2C isoform X5	15690	0
XM_020103509.1	phospholipase A2, minor isoenzyme-like	1069	1.57E-94
XM_020103510.1	protein phosphatase 1 regulatory subunit 3C-like isoform X1	1068	2.43E-150
XM_020103511.1	alpha-1A adrenergic receptor-like	1395	0
XM_020103512.1	leucine-rich repeat transmembrane neuronal protein 4-like	3517	0
XM_020103513.1	amine oxidase [flavin-containing] A-like isoform X1	1796	0
XM_020103514.1	amine oxidase [flavin-containing] A-like isoform X1	1727	0
XM_020103515.1	coxsackievirus and adenovirus receptor homolog	1368	0
XM_020103516.1	coxsackievirus and adenovirus receptor homolog	1355	0
XM_020103517.1	protein turtle homolog A-like	5625	0
XM_020103518.1	rho GTPase-activating protein 21-like	7895	0
XM_020103519.1	GPI ethanolamine phosphate transferase 3	3374	0
XM_020103520.1	dihydropyrimidinase-related protein 2-like	1806	9.12E-96
XM_020103521.1	5'-AMP-activated protein kinase subunit beta-1-like	1887	0
XM_020103522.1	5'-AMP-activated protein kinase subunit beta-1-like	1547	0
XM_020103523.1	5'-AMP-activated protein kinase subunit beta-1 isoform X3	1623	8.23E-134
XM_020103524.1	zinc finger protein ZIC 4-like	5264	0
XM_020103525.1	heterogeneous nuclear ribonucleoprotein L-like	5413	0
XM_020103526.1	adenine phosphoribosyltransferase	2111	2.43E-124
XM_020103527.1	DNA-directed RNA polymerases I and III subunit RPAC1	1538	0
XM_020103528.1	F-box/WD repeat-containing protein 4 isoform X1	1391	0
XM_020103529.1	F-box/WD repeat-containing protein 4 isoform X2	1367	0
XM_020103530.1	glucocorticoid receptor-like	6444	0
XM_020103531.1	kinesin-like protein KIF1A isoform X1	9400	0
XM_020103532.1	F-box/WD repeat-containing protein 4 isoform X3	1325	0

XM_020103533.1	F-box/WD repeat-containing protein 4 isoform X4	1250	0
XM_020103534.1	solute carrier family 2, facilitated glucose transporter member 9-like	4595	0
XM_020103535.1	spermatogenesis-associated protein 2	3131	0
XM_020103536.1	zinc finger BED domain-containing protein 1-like	2865	0
XM_020103537.1	cytosolic purine 5'-nucleotidase	2516	0
XM_020103538.1	kinesin-like protein KIF1A isoform X2	9376	0
XM_020103539.1	cyclin-dependent kinase 1-like	1151	0
XM_020103540.1	nuclear valosin-containing protein-like	2494	0
XM_020103541.1	transmembrane 9 superfamily member 2-like	4519	0
XM_020103542.1	vacuolar protein sorting-associated protein 8 homolog	5566	0
XM_020103543.1	fibroblast growth factor 8-like	2213	5.19E-145
XM_020103544.1	sodium/potassium/calcium exchanger 3-like	2951	0
XM_020103545.1	kinesin-like protein KIF1A isoform X3	9306	0
XM_020103546.1	aldose 1-epimerase isoform X1	1302	0
XM_020103547.1	aldose 1-epimerase isoform X1	1297	0
XM_020103548.1	zinc finger SWIM domain-containing protein 8 isoform X1	9350	0
XM_020103549.1	zinc finger SWIM domain-containing protein 8 isoform X2	9341	0
XM_020103550.1	zinc finger SWIM domain-containing protein 8 isoform X3	9299	0
XM_020103551.1	zinc finger SWIM domain-containing protein 8 isoform X4	9289	0
XM_020103552.1	kinesin-like protein KIF1A isoform X4	9283	0
XM_020103553.1	coiled-coil-helix-coiled-coil-helix domain-containing protein 1	818	9.65E-87
XM_020103554.1	PREDICTED: LOW QUALITY PROTEIN: neurotrypsin-like	8035	0
XM_020103555.1	propionyl-CoA carboxylase alpha chain, mitochondrial	2793	0
XM_020103556.1	bifunctional heparan sulfate N-deacetylase/N-sulfotransferase 2	4610	0
XM_020103557.1	lymphoid-specific helicase isoform X1	3349	0
XM_020103558.1	lymphoid-specific helicase isoform X2	3281	0
XM_020103559.1	lymphoid-specific helicase isoform X3	3206	0
XM_020103560.1	kinesin-like protein KIF1A isoform X5	9198	0
XM_020103561.1	rap1 GTPase-GDP dissociation stimulator 1-like	2228	0
XM_020103562.1	cytoplasmic tRNA 2-thiolation protein 2	1819	0
XM_020103563.1	RING finger protein 166	4003	1.46E-130
XM_020103564.1	sphingolipid delta(4)-desaturase DES1	2497	0
XM_020103565.1	DELTA-stichotoxin-Hmg2b-like isoform X1	620	4.46E-133
XM_020103566.1	DELTA-stichotoxin-Hmg2b-like isoform X2	739	2.73E-120
XM_020103567.1	ras and Rab interactor 2-like isoform X1	4698	0
XM_020103568.1	kinesin-like protein KIF1A isoform X6	9174	0
XM_020103569.1	ras and Rab interactor 2-like isoform X2	4621	0
XM_020103570.1	F-box only protein 28	2817	0
XM_020103571.1	steroid 17-alpha-hydroxylase/17,20 lyase	1831	0
XM_020103572.1	BLOC-1-related complex subunit 7 isoform X1	548	5.04E-70
XM_020103573.1	BLOC-1-related complex subunit 7 isoform X2	527	7.38E-65
XM_020103574.1	piezo-type mechanosensitive ion channel component 1	9042	0
XM_020103575.1	DNA replication factor Cdt1 isoform X1	2577	0
XM_020103576.1	DNA replication factor Cdt1 isoform X2	2575	0
XM_020103577.1	kinesin-like protein KIF1A isoform X7	9237	0
XM_020103578.1	thymidine kinase 2, mitochondrial isoform X2	1972	0
XM_020103579.1	thymidine kinase 2, mitochondrial isoform X2	2050	0
XM_020103580.1	thymidine kinase 2, mitochondrial isoform X2	2270	5.35E-165
XM_020103581.1	UPF0606 protein KIAA1549-like isoform X1	8692	0
XM_020103582.1	UPF0606 protein KIAA1549-like isoform X2	8650	0
XM_020103583.1	solute carrier family 23 member 1-like	2558	0
XM_020103584.1	protein FAM180A-like	677	3.95E-124
XM_020103585.1	kinesin-like protein KIF1A isoform X8	9180	0
XM_020103586.1	membrane-associated tyrosine- and threonine-specific cdc2-inhibitory kinase	2130	0
XM_020103587.1	probable polypeptide N-acetylgalactosaminyltransferase 8 isoform X1	2780	0
XM_020103588.1	probable polypeptide N-acetylgalactosaminyltransferase 8 isoform X2	2609	0
XM_020103589.1	probable polypeptide N-acetylgalactosaminyltransferase 8 isoform X3	1680	0
XM_020103590.1	dual specificity tyrosine-phosphorylation-regulated kinase 4-like isoform X1	3400	0
XM_020103591.1	dual specificity tyrosine-phosphorylation-regulated kinase 4-like isoform X2	3378	0
XM_020103592.1	dual specificity tyrosine-phosphorylation-regulated kinase 4-like isoform X3	3621	0
XM_020103593.1	dual specificity tyrosine-phosphorylation-regulated kinase 4-like isoform X4	3516	0
XM_020103594.1	protein LCHN-like	4780	0
XM_020103595.1	protein bicaudal D homolog 2-like	4524	0
XM_020103596.1	homeodomain-interacting protein kinase 2-like	378	1.61E-92
XM_020103597.1	NADH dehydrogenase [ubiquinone] 1 alpha subcomplex subunit 9, mitochondrial isoform X1	1305	0
XM_020103598.1	eukaryotic translation initiation factor 4 gamma 1-like isoform X1	5696	0
XM_020103599.1	NADH dehydrogenase [ubiquinone] 1 alpha subcomplex subunit 9, mitochondrial isoform X2	1329	0
XM_020103600.1	WAP four-disulfide core domain protein 1 isoform X1	2257	1.87E-135
XM_020103601.1	WAP four-disulfide core domain protein 1 isoform X1	2197	4.16E-122
XM_020103602.1	WAP four-disulfide core domain protein 1 isoform X3	2206	1.12E-121
XM_020103603.1	monocarboxylate transporter 13-like	2764	0
XM_020103604.1	monocarboxylate transporter 13-like	2741	0
XM_020103605.1	monocarboxylate transporter 13-like	2748	0
XM_020103606.1	F-actin-methionine sulfoxide oxidase MICAL3 isoform X1	9867	0
XM_020103607.1	eukaryotic translation initiation factor 4 gamma 1-like isoform X2	6894	0
XM_020103608.1	F-actin-methionine sulfoxide oxidase MICAL3 isoform X2	8583	0
XM_020103609.1	ras and EF-hand domain-containing protein homolog	3801	0
XM_020103610.1	ras and EF-hand domain-containing protein homolog	3622	0
XM_020103611.1	protein arginine N-methyltransferase 8-B-like	3535	0

XM_020103612.1	tetratricopeptide repeat protein 38	1914	0
XM_020103613.1	PREDICTED: tetraspanin-9	7623	0
XM_020103614.1	tetraspanin-33-like isoform X1	1082	0
XM_020103615.1	tetraspanin-33-like isoform X2	991	1.58E-178
XM_020103616.1	tetraspanin-33-like isoform X3	1025	1.56E-169
XM_020103617.1	tetraspanin-33-like isoform X4	947	1.73E-149
XM_020103618.1	tetraspanin-33-like isoform X1	939	3.00E-169
XM_020103619.1	tetraspanin-33-like isoform X5	1075	1.07E-156
XM_020103620.1	period circadian protein homolog 2-like isoform X1	4871	0
XM_020103621.1	PHD finger protein 11-like isoform X1	459	2.81E-98
XM_020103622.1	histone-lysine N-methyltransferase trithorax-like isoform X2	456	1.70E-97
XM_020103623.1	PHD finger protein 6-like isoform X3	526	3.13E-92
XM_020103624.1	potassium voltage-gated channel subfamily G member 4-like	1630	5.43E-151
XM_020103625.1	cat eye syndrome critical region protein 2	7889	0
XM_020103626.1	bcl-2-like protein 13 isoform X1	3075	0
XM_020103627.1	period circadian protein homolog 2-like isoform X1	4755	0
XM_020103628.1	bcl-2-like protein 13 isoform X1	2942	0
XM_020103629.1	bcl-2-like protein 13 isoform X1	3060	0
XM_020103630.1	mitochondrial glutamate carrier 1-like	2320	2.35E-174
XM_020103631.1	mitochondrial glutamate carrier 1-like	2562	2.70E-173
XM_020103632.1	mitochondrial glutamate carrier 1-like	2316	2.21E-174
XM_020103633.1	PREDICTED: uncharacterized protein LOC109639913 isoform X1	1874	1.45E-166
XM_020103634.1	PREDICTED: uncharacterized protein LOC109639913 isoform X2	1871	8.46E-166
XM_020103635.1	PREDICTED: uncharacterized protein LOC109639913 isoform X1	1859	6.72E-165
XM_020103636.1	V-type proton ATPase subunit E 1-like	1268	5.99E-112
XM_020103637.1	period circadian protein homolog 2-like isoform X1	4867	0
XM_020103638.1	homeobox protein aristaless-like 4	812	3.80E-150
XM_020103639.1	nuclear pore complex protein Nup205	6472	0
XM_020103640.1	sphingosine kinase 1-like	1811	0
XM_020103641.1	FYVE, RhoGEF and PH domain-containing protein 4 isoform X1	4428	0
XM_020103642.1	FYVE, RhoGEF and PH domain-containing protein 4 isoform X1	4356	0
XM_020103643.1	transmembrane and TPR repeat-containing protein 2-like	5448	0
XM_020103644.1	period circadian protein homolog 2-like isoform X1	4818	0
XM_020103645.1	adenosine deaminase CECR1-A-like	3186	0
XM_020103646.1	RIB43A-like with coiled-coils protein 2 isoform X1	1989	0
XM_020103647.1	RIB43A-like with coiled-coils protein 2 isoform X2	1400	0
XM_020103648.1	homeobox protein Hox-B8 isoform X1	2032	0
XM_020103649.1	homeobox protein Hox-B8 isoform X2	2026	2.53E-171
XM_020103650.1	homeobox protein Hox-B2	2175	0
XM_020103651.1	homeobox protein Hox-B13a	1594	0
XM_020103652.1	kinesin-like protein KIF3A isoform X1	3204	0
XM_020103653.1	period circadian protein homolog 2-like isoform X4	4815	0
XM_020103654.1	homeobox protein Hox-B1a-like	3333	0
XM_020103655.1	monocarboxylate transporter 4-like	2844	0
XM_020103656.1	pyruvate dehydrogenase (acetyl-transferring) kinase isozyme 2, mitochondrial-like	1266	0
XM_020103657.1	TANK-binding kinase 1-binding protein 1-like	2514	0
XM_020103658.1	probable E3 ubiquitin-protein ligase TRIML1	1258	0
XM_020103659.1	chromobox protein homolog 1-like	4409	1.24E-80
XM_020103660.1	period circadian protein homolog 2-like isoform X1	4752	0
XM_020103661.1	PREDICTED: coilin-like	783	9.58E-86
XM_020103662.1	PREDICTED: battenin-like, partial	1039	3.12E-175
XM_020103663.1	retinoid-inducible serine carboxypeptidase	1565	0
XM_020103664.1	homeobox protein Hox-B9	1156	3.90E-176
XM_020103665.1	proline-rich protein 15-like protein A	477	4.03E-69
XM_020103666.1	homeobox even-skipped homolog protein 1-like	1047	1.04E-143
XM_020103667.1	period circadian protein homolog 2-like isoform X1	4664	0
XM_020103668.1	phospholipase A2 inhibitor and Ly6/PLAUR domain-containing protein-like	690	8.91E-151
XM_020103669.1	nuclear factor erythroid 2-related factor 1	8072	0
XM_020103670.1	PRA1 family protein 2	920	1.06E-77
XM_020103671.1	cytosolic endo-beta-N-acetylglucosaminidase isoform X1	2697	0
XM_020103672.1	cytosolic endo-beta-N-acetylglucosaminidase isoform X2	2601	0
XM_020103673.1	casein kinase I	4282	0
XM_020103674.1	insulin-like growth factor 2 mRNA-binding protein 1 isoform X1	5293	0
XM_020103675.1	insulin-like growth factor 2 mRNA-binding protein 1 isoform X2	5287	0
XM_020103676.1	homeobox protein Hox-B3a	4452	0
XM_020103677.1	homeobox protein Hox-B3a	4453	0
XM_020103678.1	homeobox protein Hox-B3a	4439	0
XM_020103679.1	homeobox protein Hox-B3a	4438	0
XM_020103680.1	zinc finger protein 521 isoform X1	6288	0
XM_020103681.1	homeobox protein Hox-B3a	4811	0
XM_020103682.1	homeobox protein Hox-B5a-like	2167	0
XM_020103683.1	homeobox protein Hox-A5-like	1421	4.58E-73
XM_020103684.1	ubiquitin-conjugating enzyme E2 Z	1876	0
XM_020103685.1	zinc finger protein 646-like isoform X1	3473	0
XM_020103686.1	Krueppel homolog 1-like isoform X2	3470	0
XM_020103687.1	zinc finger protein 646-like isoform X3	3425	0
XM_020103688.1	rab11 family-interacting protein 4A-like	5031	0
XM_020103689.1	zinc finger protein 521 isoform X2	6285	0
XM_020103690.1	coatomer subunit zeta-1-like isoform X1	2126	2.99E-163

XM_020103691.1	coatomer subunit zeta-1-like isoform X2	2094	1.27E-154
XM_020103692.1	potassium voltage-gated channel subfamily C member 1-like isoform X1	2027	0
XM_020103693.1	potassium voltage-gated channel subfamily C member 1-like isoform X2	2091	0
XM_020103694.1	potassium voltage-gated channel subfamily C member 1-like isoform X3	11302	0
XM_020103695.1	oxysterol-binding protein-related protein 6-like isoform X1	2705	0
XM_020103696.1	oxysterol-binding protein-related protein 6-like isoform X1	2600	0
XM_020103697.1	zinc finger protein 521 isoform X3	6258	0
XM_020103698.1	tRNA-dihydrouridine(16/17) synthase [NAD(P)(+)]-like	3208	0
XM_020103699.1	ATP synthase F(0) complex subunit C3, mitochondrial-like	920	3.33E-75
XM_020103700.1	mannosyl-oligosaccharide 1,2-alpha-mannosidase IA	3119	0
XM_020103701.1	thioredoxin-like protein AAED1	1407	0
XM_020103702.1	C-type lectin domain family 11 member A	1566	0
XM_020103703.1	protein ARV1	2441	1.02E-160
XM_020103704.1	zinc finger protein 521 isoform X4	6087	0
XM_020103705.1	gap junction Cx32.2 protein-like	1334	0
XM_020103706.1	gap junction Cx32.2 protein-like	1329	0
XM_020103707.1	cation-independent mannose-6-phosphate receptor-like	7468	0
XM_020103708.1	ras-interacting protein 1-like isoform X1	3106	0
XM_020103709.1	protein FAM184A-like	2488	0
XM_020103710.1	protein broad-minded	5041	0
XM_020103711.1	PREDICTED: exostosin-1-like	1440	0
XM_020103712.1	zinc finger protein 521 isoform X5	6084	0
XM_020103713.1	scavenger receptor class A member 5-like	1298	0
XM_020103714.1	protein unc-93 homolog A-like	1858	0
XM_020103715.1	solute carrier family 22 member 2-like	1824	0
XM_020103716.1	sterile alpha motif domain-containing protein 12-like	451	1.94E-35
XM_020103717.1	dihydroxyacetone phosphate acyltransferase	4016	0
XM_020103718.1	PREDICTED: uncharacterized protein C1orf131 homolog	1324	2.41E-107
XM_020103719.1	zinc finger protein 521 isoform X1	5736	0
XM_020103720.1	tripartite motif-containing protein 67	5918	0
XM_020103721.1	protein FAM89A	5247	1.71E-92
XM_020103722.1	inactive hydroxysteroid dehydrogenase-like protein 1	1877	0
XM_020103723.1	gap junction Cx32.7 protein-like	1331	0
XM_020103724.1	gap junction Cx32.7 protein-like	1326	0
XM_020103725.1	gap junction Cx32.7 protein-like	1327	0
XM_020103726.1	PREDICTED: uncharacterized protein C1orf198 homolog	2925	0
XM_020103727.1	zinc finger protein 521 isoform X2	5733	0
XM_020103728.1	gap junction Cx32.2 protein-like	1462	0
XM_020103729.1	UDP-N-acetylglucosamine--peptide N-acetylglucosaminyltransferase 110 kDa subunit isoform X9	4708	0
XM_020103730.1	UDP-N-acetylglucosamine--peptide N-acetylglucosaminyltransferase 110 kDa subunit isoform X9	4705	0
XM_020103731.1	exocyst complex component 8	2704	0
XM_020103732.1	kinesin-like protein KIF3A isoform X2	3195	0
XM_020103733.1	sprT-like domain-containing protein Spartan	2733	0
XM_020103734.1	angiopoietin-1 isoform X1	2124	0
XM_020103735.1	angiopoietin-1 isoform X2	1849	0
XM_020103736.1	angiopoietin-1 isoform X1	1346	0
XM_020103737.1	zinc finger protein 521 isoform X3	5705	0
XM_020103738.1	UPF0500 protein C1orf216 homolog isoform X1	2204	0
XM_020103739.1	UPF0500 protein C1orf216 homolog isoform X2	2020	0
XM_020103740.1	UPF0500 protein C1orf216 homolog isoform X1	2086	0
XM_020103741.1	PREDICTED: exostosin-1c	1390	0
XM_020103742.1	absent in melanoma 1 protein-like	7857	0
XM_020103743.1	ephrin type-A receptor 7-like	1081	2.02E-166

XM_020103744.1	transcription factor AP-2-epsilon	1462	0
XM_020103745.1	dyslexia-associated protein KIAA0319-like protein homolog	1163	1.95E-143
XM_020103746.1	thiopurine S-methyltransferase isoform X2	1112	0
XM_020103747.1	thiopurine S-methyltransferase isoform X2	951	0
XM_020103748.1	PERQ amino acid-rich with GYF domain-containing protein 2 isoform X1	5038	0
XM_020103749.1	thiopurine S-methyltransferase isoform X2	986	0
XM_020103750.1	annexin A2-like isoform X1	1354	0
XM_020103751.1	annexin A2-like isoform X1	1196	0
XM_020103752.1	annexin A2-like isoform X1	1356	0
XM_020103753.1	PREDICTED: uncharacterized protein LOC109639992	3129	0
XM_020103754.1	PREDICTED: uncharacterized protein LOC109639992	3010	0
XM_020103755.1	actin-binding Rho-activating protein-like	3389	0
XM_020103756.1	protein argonaute-4	620	8.34E-62
XM_020103757.1	PERQ amino acid-rich with GYF domain-containing protein 2 isoform X1	5053	0
XM_020103758.1	PREDICTED: sororin	1435	9.94E-98
XM_020103759.1	proteasome subunit beta type-2	827	1.17E-146
XM_020103760.1	sterile alpha motif domain-containing protein 12 isoform X1	1123	2.24E-141
XM_020103761.1	sterile alpha motif domain-containing protein 12 isoform X2	1120	6.63E-143
XM_020103762.1	sterile alpha motif domain-containing protein 12 isoform X3	950	1.00E-129
XM_020103763.1	sterile alpha motif domain-containing protein 12 isoform X4	3504	2.76E-144
XM_020103764.1	sterile alpha motif domain-containing protein 12 isoform X5	7032	1.63E-125
XM_020103765.1	PERQ amino acid-rich with GYF domain-containing protein 2 isoform X1	5053	0
XM_020103766.1	sterile alpha motif domain-containing protein 12 isoform X6	1372	8.23E-136
XM_020103767.1	sterile alpha motif domain-containing protein 12 isoform X4	1349	8.85E-131
XM_020103768.1	PREDICTED: claspin	4743	0
XM_020103769.1	mediator of RNA polymerase II transcription subunit 30-like	1646	2.95E-86

XM_020103770.1	PREDICTED: neurochondrin	3171	0
XM_020103771.1	syndecan-4-like isoform X2	1092	7.26E-118
XM_020103772.1	PREDICTED: complexin-2-like	4085	2.15E-56
XM_020103773.1	PERQ amino acid-rich with GYF domain-containing protein 2 isoform X1	4963	0
XM_020103774.1	PREDICTED: complexin-2-like	3907	2.05E-56
XM_020103775.1	PREDICTED: complexin-2-like	3944	2.07E-56
XM_020103776.1	spermatogenesis-associated protein 5-like	1385	0
XM_020103777.1	protocadherin Fat 4-like	7137	0
XM_020103778.1	testicular haploid expressed gene protein-like	1410	9.45E-167
XM_020103779.1	homeodomain-only protein	709	5.72E-49
XM_020103780.1	major facilitator superfamily domain-containing protein 7-like	929	2.07E-109
XM_020103781.1	signal recognition particle subunit SRP72	3242	0
XM_020103782.1	snRNA-activating protein complex subunit 1-like	1282	0
XM_020103783.1	coiled-coil domain-containing protein 186-like isoform X1	1154	0
XM_020103784.1	coiled-coil domain-containing protein 186-like isoform X2	1151	0
XM_020103785.1	oxysterol-binding protein-related protein 1-like isoform X1	4280	0
XM_020103786.1	ADP-ribosylation factor-like protein 9	2683	2.02E-134
XM_020103787.1	leptin receptor overlapping transcript-like 1 isoform X1	3783	1.49E-80
XM_020103788.1	leptin receptor overlapping transcript-like 1 isoform X2	713	1.42E-88
XM_020103789.1	ankyrin repeat domain-containing protein 50-like	6931	0
XM_020103790.1	protein transport protein Sec24B-like isoform X1	4753	0
XM_020103791.1	protein transport protein Sec24B-like isoform X2	4606	0
XM_020103792.1	nuclear cap-binding protein subunit 1 isoform X1	2998	0
XM_020103793.1	nuclear cap-binding protein subunit 1 isoform X2	2995	0
XM_020103794.1	oxysterol-binding protein-related protein 1-like isoform X2	4276	0
XM_020103795.1	thiosulfate sulfurtransferase/rhodanese-like domain-containing protein 2	1867	0
XM_020103796.1	protein sprouty homolog 2-like	2489	0
XM_020103797.1	polycomb group RING finger protein 3	5419	1.43E-169
XM_020103798.1	receptor-type tyrosine-protein phosphatase gamma-like	4799	0
XM_020103799.1	LIM domain-binding protein 2 isoform X1	1426	0
XM_020103800.1	LIM domain-binding protein 2 isoform X2	1420	0
XM_020103801.1	LIM domain-binding protein 2 isoform X2	1415	0
XM_020103802.1	oxysterol-binding protein-related protein 1-like isoform X3	4255	0
XM_020103803.1	transmembrane anterior posterior transformation protein 1 homolog isoform X1	3485	0
XM_020103804.1	transmembrane anterior posterior transformation protein 1 homolog isoform X2	1832	0
XM_020103805.1	solute carrier family 25 member 45-like	2382	0
XM_020103806.1	follistatin-related protein 5 isoform X1	4471	0
XM_020103807.1	follistatin-related protein 5 isoform X2	2693	0
XM_020103808.1	follistatin-related protein 5 isoform X3	2684	0
XM_020103809.1	follistatin-related protein 5 isoform X4	2681	0
XM_020103810.1	protein FAM198B	2507	0
XM_020103811.1	glycine receptor subunit beta	1623	0
XM_020103812.1	glutamate receptor 2	2677	0
XM_020103813.1	relaxin receptor 1	5272	0
XM_020103814.1	dipeptidyl aminopeptidase-like protein 6 isoform X1	5551	0
XM_020103815.1	PREDICTED: uncharacterized protein C4orf45 homolog	434	1.01E-71
XM_020103816.1	transmembrane protein 144 isoform X1	1495	0
XM_020103817.1	transmembrane protein 144 isoform X2	1441	0
XM_020103818.1	platelet-derived growth factor C isoform X1	1331	0
XM_020103819.1	platelet-derived growth factor C isoform X2	1328	0
XM_020103820.1	electron transfer flavoprotein-ubiquinone oxidoreductase, mitochondrial	2261	0
XM_020103821.1	peptidyl-prolyl cis-trans isomerase-like	1611	0
XM_020103822.1	rap guanine nucleotide exchange factor 2 isoform X1	4495	0
XM_020103823.1	dipeptidyl aminopeptidase-like protein 6 isoform X2	5536	0
XM_020103824.1	rap guanine nucleotide exchange factor 2 isoform X2	4742	0
XM_020103825.1	rap guanine nucleotide exchange factor 6-like	1849	7.58E-114
XM_020103826.1	PREDICTED: complexin-2	743	9.76E-61
XM_020103827.1	dihydropteridine reductase	1786	5.91E-155
XM_020103828.1	CD99 antigen-like isoform X1	1623	6.59E-120
XM_020103829.1	CD99 antigen-like isoform X2	2304	5.99E-104
XM_020103830.1	matrix-remodeling-associated protein 5	9331	0
XM_020103831.1	dipeptidyl aminopeptidase-like protein 6 isoform X3	2678	0
XM_020103832.1	runt-related transcription factor 1 isoform X1	3017	0
XM_020103833.1	runt-related transcription factor 1 isoform X2	1959	0
XM_020103834.1	neuroligin-4, X-linked	4423	0
XM_020103835.1	patatin-like phospholipase domain-containing protein 2	1747	1.27E-168
XM_020103836.1	protein downstream neighbor of son homolog	1897	0
XM_020103837.1	PREDICTED: anosmin-1	3024	0
XM_020103838.1	PREDICTED: glycogenin-2	1161	0
XM_020103839.1	dipeptidyl aminopeptidase-like protein 6 isoform X1	4760	0
XM_020103840.1	dehydrogenase/reductase SDR family member on chromosome X	1496	2.40E-150
XM_020103841.1	protein FAM207A	1213	3.54E-167
XM_020103842.1	PREDICTED: pseudouridine-5'-phosphatase	1318	1.42E-169
XM_020103843.1	arylsulfatase G	1883	0
XM_020103844.1	serine/threonine-protein kinase pim-2-like	2141	2.21E-152
XM_020103845.1	chloride intracellular channel protein 4-like	1935	2.56E-180
XM_020103846.1	ankyrin repeat domain-containing protein SOWAHC-like	2201	0
XM_020103847.1	growth arrest-specific protein 8-like isoform X1	1548	0
XM_020103848.1	growth arrest-specific protein 8-like isoform X1	1665	0

XM_020103849.1	angiomotin-like protein 2 isoform X1	2789	0
XM_020103850.1	lymphocyte antigen 6D-like	546	6.85E-46
XM_020103851.1	nuclear RNA export factor 1 isoform X1	2528	0
XM_020103852.1	protein NLRC3-like isoform X1	3521	0
XM_020103853.1	protein NLRC3-like isoform X2	3517	0
XM_020103854.1	protein NLRC3-like isoform X1	2308	0
XM_020103855.1	calcipressin-3 isoform X1	1440	3.71E-112
XM_020103856.1	calcipressin-1 isoform X2	1527	3.21E-103
XM_020103857.1	cAMP-dependent protein kinase catalytic subunit PRKX	2516	0
XM_020103858.1	cAMP-dependent protein kinase catalytic subunit PRKX	2257	0
XM_020103859.1	serotransferrin precursor	2373	0
XM_020103860.1	rho guanine nucleotide exchange factor 7-like isoform X2	3525	0
XM_020103861.1	rho guanine nucleotide exchange factor 7-like isoform X2	3366	0
XM_020103862.1	transcription factor Sox-1a-like	1804	1.22E-138
XM_020103863.1	sodium/myo-inositol cotransporter-like	3489	0
XM_020103864.1	voltage-dependent L-type calcium channel subunit beta-4-like	3034	0
XM_020103865.1	transportin-1 isoform X2	4219	0
XM_020103866.1	cyclic AMP-responsive element-binding protein 3-like protein 1	3191	0
XM_020103867.1	ras-related and estrogen-regulated growth inhibitor-like	1109	4.23E-127
XM_020103868.1	tetratricopeptide repeat protein 39C	2962	0
XM_020103869.1	rho guanine nucleotide exchange factor 7-like	408	6.28E-98
XM_020103870.1	potassium voltage-gated channel subfamily H member 4-like	710	2.70E-162
XM_020103871.1	keratin, type II cytoskeletal 8-like	1639	0
XM_020103872.1	small integral membrane protein 11A-like	1087	1.86E-20
XM_020103873.1	cardiac phospholamban	1120	2.75E-17
XM_020103874.1	keratin, type I cytoskeletal 18-like isoform X1	1728	0
XM_020103875.1	keratin, type I cytoskeletal 18-like isoform X2	1404	0
XM_020103876.1	tetratricopeptide repeat protein 39C	2927	0
XM_020103877.1	T-cell surface antigen CD2	3075	0
XM_020103878.1	G-protein coupled bile acid receptor 1	2109	0
XM_020103879.1	G-protein coupled bile acid receptor 1	2019	0
XM_020103880.1	G-protein coupled bile acid receptor 1	2014	0
XM_020103881.1	autophagy-related protein 13 isoform X1	3665	0
XM_020103882.1	autophagy-related protein 13 isoform X1	3763	0
XM_020103883.1	autophagy-related protein 13 isoform X1	3647	0
XM_020103884.1	autophagy-related protein 13 isoform X1	3566	0
XM_020103885.1	autophagy-related protein 13 isoform X4	3548	0
XM_020103886.1	putative nuclease HARBI1	1973	0
XM_020103887.1	ATP-dependent RNA helicase DDX3X-like isoform X1	11230	0
XM_020103888.1	UBX domain-containing protein 7	2966	0
XM_020103889.1	ATP-dependent RNA helicase DDX3X-like isoform X2	11227	0
XM_020103890.1	ATP-dependent RNA helicase DDX3X-like isoform X3	11216	0
XM_020103891.1	ATP-dependent RNA helicase DDX3X-like isoform X4	11213	0
XM_020103892.1	ATP-dependent RNA helicase DDX3X-like isoform X5	11187	0
XM_020103893.1	ATP-dependent RNA helicase DDX3X-like isoform X6	11187	0
XM_020103894.1	ATP-dependent RNA helicase DDX3X-like isoform X7	11179	0
XM_020103895.1	ATP-dependent RNA helicase DDX3X-like isoform X8	11173	0
XM_020103896.1	ATP-dependent RNA helicase DDX3X-like isoform X9	11166	0
XM_020103897.1	ATP-dependent RNA helicase DDX3X-like isoform X10	11164	0
XM_020103898.1	ATP-dependent RNA helicase DDX3X-like isoform X11	11135	0
XM_020103899.1	ATP-dependent RNA helicase DDX3X-like isoform X12	11170	0
XM_020103900.1	protein-tyrosine kinase 2-beta-like isoform X1	3476	0
XM_020103901.1	protein-tyrosine kinase 2-beta-like isoform X1	3430	0
XM_020103902.1	protein-tyrosine kinase 2-beta-like isoform X1	3473	0
XM_020103903.1	vacuolar protein sorting-associated protein 4B isoform X1	3120	0
XM_020103904.1	inosine-uridine preferring nucleoside hydrolase-like	1649	0
XM_020103905.1	inosine-uridine preferring nucleoside hydrolase-like	1092	0
XM_020103906.1	inosine-uridine preferring nucleoside hydrolase-like	2020	0
XM_020103907.1	inosine-uridine preferring nucleoside hydrolase-like	1941	0
XM_020103908.1	protein unc-50 homolog	1426	0
XM_020103909.1	cytidine and dCMP deaminase domain-containing protein 1 isoform X1	3409	0
XM_020103910.1	cytidine and dCMP deaminase domain-containing protein 1 isoform X2	3406	0
XM_020103911.1	calcium-binding protein 39-like	3681	0
XM_020103912.1	vacuolar protein sorting-associated protein 4B isoform X2	2916	0
XM_020103913.1	probable phospholipid-transporting ATPase IH isoform X4	8143	0
XM_020103914.1	probable phospholipid-transporting ATPase IH isoform X4	3837	0
XM_020103915.1	probable phospholipid-transporting ATPase IH isoform X4	3834	0
XM_020103916.1	probable phospholipid-transporting ATPase IH isoform X4	3821	0
XM_020103917.1	probable phospholipid-transporting ATPase IH isoform X4	3824	0
XM_020103918.1	probable phospholipid-transporting ATPase IH isoform X4	3794	0
XM_020103919.1	semaphorin-5B isoform X1	4511	0
XM_020103920.1	immunoglobulin superfamily member 3-like	3304	7.71E-106
XM_020103921.1	integral membrane protein GPR180	2533	0
XM_020103922.1	dTDP-D-glucose 4,6-dehydratase	1854	0
XM_020103923.1	gamma-tubulin complex component 6	4468	0
XM_020103924.1	cell division cycle protein 20 homolog	1739	0
XM_020103925.1	fibroblast growth factor 14-like isoform X1	6903	3.96E-180
XM_020103926.1	fibroblast growth factor 14-like isoform X2	6822	1.17E-169
XM_020103927.1	fibroblast growth factor 14-like isoform X2	6843	1.98E-121

XM_020103928.1	NADH-ubiquinone oxidoreductase 75 kDa subunit, mitochondrial-like	2508	0
XM_020103929.1	angio-associated migratory cell protein	1532	0
XM_020103930.1	angio-associated migratory cell protein	1859	0
XM_020103931.1	protein FAM184A-like isoform X1	3510	0
XM_020103932.1	protein FAM184A-like isoform X2	3480	0
XM_020103933.1	60S ribosomal protein L26	580	1.06E-88
XM_020103934.1	nuclear RNA export factor 1 isoform X1	2774	0
XM_020103935.1	kinesin light chain 1-like	3326	0
XM_020103936.1	fatty aldehyde dehydrogenase-like	1967	0
XM_020103937.1	autoimmune regulator isoform X1	2083	0
XM_020103938.1	eukaryotic translation initiation factor 3 subunit K isoform X1	941	2.08E-163
XM_020103939.1	eukaryotic translation initiation factor 3 subunit K isoform X2	938	2.06E-162
XM_020103940.1	Fanconi anemia group J protein	4301	0
XM_020103941.1	tyrosine kinase receptor Cad96Ca-like	930	0
XM_020103942.1	putative uncharacterized zinc finger protein 814	5312	0
XM_020103943.1	MAP/microtubule affinity-regulating kinase 4-like	5935	0
XM_020103944.1	mitogen-activated protein kinase kinase kinase kinase 5-like	3080	0
XM_020103945.1	autoimmune regulator isoform X2	2080	0
XM_020103946.1	ubiquitin-like modifier-activating enzyme 1	1317	0
XM_020103947.1	calpain-1 catalytic subunit-like	3213	0
XM_020103948.1	aldehyde dehydrogenase, dimeric NADP-preferring-like isoform X1	2045	0
XM_020103949.1	aldehyde dehydrogenase, dimeric NADP-preferring-like isoform X1	2049	0
XM_020103950.1	eukaryotic translation initiation factor 3 subunit K-like isoform X1	618	3.82E-101
XM_020103951.1	eukaryotic translation initiation factor 3 subunit K-like isoform X2	615	1.61E-100
XM_020103952.1	transmembrane protein 97	901	2.32E-110
XM_020103953.1	sushi domain-containing protein 6-like isoform X1	1388	2.14E-168
XM_020103954.1	sushi domain-containing protein 6-like isoform X2	1333	4.81E-157
XM_020103955.1	T-box transcription factor TBX4	1679	0
XM_020103956.1	serine--pyruvate aminotransferase-like	1500	0
XM_020103957.1	ubiquitin carboxyl-terminal hydrolase 32 isoform X1	8545	0
XM_020103958.1	ubiquitin carboxyl-terminal hydrolase 32 isoform X2	8515	0
XM_020103959.1	ubiquitin carboxyl-terminal hydrolase 32 isoform X3	8497	0
XM_020103960.1	amyloid protein-binding protein 2	2936	0
XM_020103961.1	PREDICTED: uncharacterized protein LOC109640130	2393	5.45E-141
XM_020103962.1	alpha-actinin-4-like isoform X1	4677	0
XM_020103963.1	alpha-actinin-4-like isoform X2	4678	0
XM_020103964.1	alpha-actinin-4-like isoform X3	4642	0
XM_020103965.1	alpha-actinin-4-like isoform X4	4643	0
XM_020103966.1	alpha-actinin-4-like isoform X5	4631	0
XM_020103967.1	alpha-actinin-4-like isoform X6	4631	0
XM_020103968.1	alpha-actinin-4-like isoform X7	4713	0
XM_020103969.1	zinc finger protein 431-like	3678	0
XM_020103970.1	pre-B-cell leukemia transcription factor 1 isoform X1	3518	0
XM_020103971.1	coiled-coil domain-containing protein 9 isoform X1	4376	0
XM_020103972.1	coiled-coil domain-containing protein 9 isoform X1	4375	0
XM_020103973.1	coiled-coil domain-containing protein 9 isoform X1	4373	0
XM_020103974.1	coiled-coil domain-containing protein 9 isoform X1	4265	0
XM_020103975.1	PREDICTED: uncharacterized protein LOC109640135	4192	7.56E-125
XM_020103976.1	PREDICTED: uncharacterized protein LOC109640135	4057	2.85E-125
XM_020103977.1	PREDICTED: uncharacterized protein LOC109640135	4070	3.01E-125
XM_020103978.1	zinc finger CCCH domain-containing protein 4	6453	0
XM_020103979.1	pre-B-cell leukemia transcription factor 1 isoform X2	1655	0
XM_020103980.1	WD repeat-containing protein 20-like	4182	0
XM_020103981.1	serine/threonine-protein kinase ULK2	7383	0
XM_020103982.1	serine/threonine-protein kinase PAK 4	6083	0
XM_020103983.1	serine/threonine-protein kinase PAK 4	6085	0
XM_020103984.1	serine/threonine-protein kinase PAK 4	5977	0
XM_020103985.1	hypoxia-inducible factor 1-alpha-like	3364	0
XM_020103986.1	TFIIH basal transcription factor complex helicase XPD subunit	2425	0
XM_020103987.1	pre-B-cell leukemia transcription factor 1 isoform X3	3405	0
XM_020103988.1	cdc42 effector protein 3-like	2452	5.68E-175
XM_020103989.1	creatine kinase M-type-like	1684	0
XM_020103990.1	molybdopterin synthase catalytic subunit-like	524	6.71E-108
XM_020103991.1	molybdopterin synthase sulfur carrier subunit-like isoform X2	352	2.70E-55
XM_020103992.1	molybdopterin synthase sulfur carrier subunit-like isoform X2	359	5.35E-59
XM_020103993.1	pre-B-cell leukemia transcription factor 1 isoform X4	2888	0
XM_020103994.1	frataxin, mitochondrial	906	2.87E-163
XM_020103995.1	E3 ubiquitin-protein ligase DTX3L-like	1968	0
XM_020103996.1	coiled-coil domain-containing protein 125	2639	0
XM_020103997.1	carbohydrate deacetylase	889	4.36E-153
XM_020103998.1	AT-rich interactive domain-containing protein 3A-like	1701	0
XM_020103999.1	insulin gene enhancer protein ISL-1 isoform X1	1871	0
XM_020104000.1	insulin gene enhancer protein ISL-1 isoform X1	1911	0
XM_020104001.1	insulin gene enhancer protein ISL-1 isoform X1	1868	0
XM_020104002.1	ubiquitin-conjugating enzyme E2 L3	2015	9.22E-92
XM_020104003.1	amyloid beta A4 precursor protein-binding family A member 1	4904	0
XM_020104004.1	amyloid beta A4 precursor protein-binding family A member 1	4834	0
XM_020104005.1	amyloid beta A4 precursor protein-binding family A member 1	4733	0
XM_020104006.1	zona pellucida sperm-binding protein 4-like	1343	0

XM_020104007.1	cell cycle checkpoint protein RAD17	2684	0
XM_020104008.1	adenylate kinase isoenzyme 6	879	4.15E-117
XM_020104009.1	protein prenyltransferase alpha subunit repeat-containing protein 1 isoform X1	4175	0
XM_020104010.1	protein prenyltransferase alpha subunit repeat-containing protein 1 isoform X2	4066	0
XM_020104011.1	3-hydroxy-3-methylglutaryl-Coenzyme A reductase isoform X1	3488	0
XM_020104012.1	3-hydroxy-3-methylglutaryl-Coenzyme A reductase isoform X2	3485	0
XM_020104013.1	nuclear RNA export factor 1 isoform X1	2421	0
XM_020104014.1	3-hydroxy-3-methylglutaryl-Coenzyme A reductase isoform X1	3370	0
XM_020104015.1	E3 SUMO-protein ligase PIAS2 isoform X1	1965	0
XM_020104016.1	E3 SUMO-protein ligase PIAS2 isoform X2	1962	0
XM_020104017.1	glutamine synthetase-like	2026	0
XM_020104018.1	nucleus accumbens-associated protein 1-like isoform X1	4615	0
XM_020104019.1	nucleus accumbens-associated protein 1-like isoform X1	3418	0
XM_020104020.1	nucleus accumbens-associated protein 1-like isoform X1	3209	0
XM_020104021.1	nucleus accumbens-associated protein 1-like isoform X1	4612	0
XM_020104022.1	nucleus accumbens-associated protein 1-like isoform X1	3248	0
XM_020104023.1	nucleus accumbens-associated protein 1-like isoform X1	3279	0
XM_020104024.1	beta-1,3-N-acetylglucosaminyltransferase manic fringe-like	1952	0
XM_020104025.1	WD repeat domain-containing protein 83	1228	0
XM_020104026.1	PREDICTED: artemin-like	1914	1.29E-121
XM_020104027.1	PREDICTED: artemin-like	1378	4.63E-124
XM_020104028.1	PREDICTED: artemin-like	1407	6.48E-124
XM_020104029.1	protein phosphatase 1 regulatory subunit 29-like	2925	0
XM_020104030.1	3-ketodihydrosphingosine reductase	2417	0
XM_020104031.1	PREDICTED: uncharacterized protein LOC109640171	1262	0
XM_020104032.1	voltage-dependent P/Q-type calcium channel subunit alpha-1A-like	3991	0
XM_020104033.1	alpha-mannosidase 2x	2571	0
XM_020104034.1	metal transporter CNNM1-like	3877	0
XM_020104035.1	CREB3 regulatory factor-like	507	6.42E-99
XM_020104036.1	protein Asterix	782	3.58E-72
XM_020104037.1	homeobox protein Nkx-2.5-like	1717	0
XM_020104038.1	GTP-binding protein 1-like isoform X1	3974	0
XM_020104039.1	GTP-binding protein 1-like isoform X1	2065	0
XM_020104040.1	urotensin-2 receptor-like isoform X2	1105	0
XM_020104041.1	protein phosphatase 1 regulatory subunit 7	2447	0
XM_020104042.1	beta-galactoside-binding lectin-like	918	3.07E-93
XM_020104043.1	PREDICTED: syntaxin-10	2887	1.14E-138
XM_020104044.1	immediate early response gene 2 protein	1565	4.90E-102
XM_020104045.1	PREDICTED: mitoferrin-2-like	3288	0
XM_020104046.1	transcription factor IIIA-like	1509	0
XM_020104047.1	coiled-coil domain-containing protein 130	1379	0
XM_020104048.1	coiled-coil domain-containing protein 130	1377	0
XM_020104049.1	ectonucleoside triphosphate diphosphohydrolase 7-like isoform X1	4423	0
XM_020104050.1	ectonucleoside triphosphate diphosphohydrolase 7-like isoform X2	4420	0
XM_020104051.1	aspartate aminotransferase, cytoplasmic-like	2576	0
XM_020104052.1	5'-AMP-activated protein kinase subunit gamma-2-like	1265	0
XM_020104053.1	tRNA (guanine(26)-N(2))-dimethyltransferase	2251	0
XM_020104054.1	tRNA (guanine(26)-N(2))-dimethyltransferase	2126	0
XM_020104055.1	complement C3-like	5613	0
XM_020104056.1	voltage-dependent P/Q-type calcium channel subunit alpha-1A-like isoform X1	5988	1.09E-54
XM_020104057.1	voltage-dependent P/Q-type calcium channel subunit alpha-1A-like isoform X2	5976	2.57E-68
XM_020104058.1	coiled-coil domain-containing protein 28A isoform X1	1754	1.87E-109
XM_020104059.1	coiled-coil domain-containing protein 28A isoform X1	1807	3.15E-109
XM_020104060.1	coiled-coil domain-containing protein 28A isoform X1	1807	3.15E-109
XM_020104061.1	coiled-coil domain-containing protein 28A isoform X1	1747	1.31E-96
XM_020104062.1	abl interactor 2-like isoform X1	1161	0
XM_020104063.1	abl interactor 2-like isoform X2	1143	0
XM_020104064.1	abl interactor 2-like isoform X3	1014	0
XM_020104065.1	abl interactor 2-like isoform X1	1383	2.23E-161
XM_020104066.1	aspartyl aminopeptidase	1692	0
XM_020104067.1	prospero homeobox protein 1-like	5153	0
XM_020104068.1	protein FAM131A-like isoform X2	3446	1.34E-175
XM_020104069.1	tubulin alpha chain-like	744	1.03E-154
XM_020104070.1	receptor-type tyrosine-protein phosphatase-like N	3184	0
XM_020104071.1	sterol 26-hydroxylase, mitochondrial-like	2366	0

XM_020104072.1	collagen alpha-1(XVIII) chain-like	6091	0
XM_020104073.1	MAGUK p55 subfamily member 4-like	1801	0
XM_020104074.1	cytochrome P450 20A1	1856	0
XM_020104075.1	protein FAM131A-like isoform X2	3462	6.87E-177
XM_020104076.1	transmembrane protein 237	1758	0
XM_020104077.1	ATP synthase-coupling factor 6, mitochondrial isoform X1	521	3.89E-100
XM_020104078.1	ATP synthase-coupling factor 6, mitochondrial isoform X1	468	1.28E-74
XM_020104079.1	obscurin-like protein 1 isoform X1	7471	0
XM_020104080.1	obscurin-like protein 1 isoform X2	7386	0
XM_020104081.1	X-linked retinitis pigmentosa GTPase regulator-like isoform X1	3292	0
XM_020104082.1	X-linked retinitis pigmentosa GTPase regulator-like isoform X2	3289	0
XM_020104083.1	tubulin alpha chain-like	1701	0
XM_020104084.1	tubulin alpha chain-like	576	5.95E-112
XM_020104085.1	NACHT, LRR and PYD domains-containing protein 3-like isoform X1	5599	0

XM_020104086.1	elongation of very long chain fatty acids protein 1-like	2360	0
XM_020104087.1	NACHT, LRR and PYD domains-containing protein 3-like isoform X2	4021	0
XM_020104088.1	small ubiquitin-related modifier 1	2435	2.71E-63
XM_020104089.1	PREDICTED: claudin-34-like	294	1.08E-50
XM_020104090.1	sodium/potassium-transporting ATPase subunit alpha-1	3438	0
XM_020104091.1	sodium/potassium-transporting ATPase subunit alpha-1	3638	0
XM_020104092.1	myc box-dependent-interacting protein 1 isoform X1	2900	0
XM_020104093.1	myc box-dependent-interacting protein 1 isoform X2	2890	0
XM_020104094.1	myc box-dependent-interacting protein 1 isoform X3	2854	0
XM_020104095.1	elongation of very long chain fatty acids protein 1-like	2229	0
XM_020104096.1	myc box-dependent-interacting protein 1 isoform X4	2845	0
XM_020104097.1	myc box-dependent-interacting protein 1 isoform X5	2806	0
XM_020104098.1	myc box-dependent-interacting protein 1 isoform X6	2796	0
XM_020104099.1	myc box-dependent-interacting protein 1 isoform X7	2695	0
XM_020104100.1	myc box-dependent-interacting protein 1 isoform X7	2672	0
XM_020104101.1	myc box-dependent-interacting protein 1 isoform X7	2586	0
XM_020104102.1	myc box-dependent-interacting protein 1 isoform X8	2742	0
XM_020104103.1	myc box-dependent-interacting protein 1 isoform X9	2697	0
XM_020104104.1	indian hedgehog B protein-like isoform X1	3596	0
XM_020104105.1	elongation of very long chain fatty acids protein 1-like	2205	0
XM_020104106.1	indian hedgehog B protein-like isoform X2	3297	0
XM_020104107.1	amyloid beta A4 protein-like	4128	0
XM_020104108.1	filamin A-interacting protein 1-like isoform X2	5286	0
XM_020104109.1	filamin A-interacting protein 1-like isoform X2	4307	0
XM_020104110.1	filamin A-interacting protein 1-like isoform X2	4456	0
XM_020104111.1	epithelial cell-transforming sequence 2 oncogene-like isoform X1	3486	0
XM_020104112.1	epithelial cell-transforming sequence 2 oncogene-like isoform X2	3402	0
XM_020104113.1	PREDICTED: uncharacterized protein LOC109640227	1618	0
XM_020104114.1	desmin-like isoform X2	2173	0
XM_020104115.1	transmembrane protein 198-B-like	1556	0
XM_020104116.1	transmembrane protein 198-B-like	2725	0
XM_020104117.1	lysophosphatidic acid receptor 4	1006	0
XM_020104118.1	mitogen-activated protein kinase kinase kinase 2	5417	0
XM_020104119.1	methylcrotonoyl-CoA carboxylase beta chain, mitochondrial-like	2442	0
XM_020104120.1	histidine-rich glycoprotein-like	1504	0
XM_020104121.1	histidine-rich glycoprotein-like	1520	0
XM_020104122.1	zinc finger matrin-type protein 3	2981	0
XM_020104123.1	zinc finger matrin-type protein 3	1917	0
XM_020104124.1	retinal Mueller cells isomerohydrolase-like	1874	0
XM_020104125.1	formin-binding protein 1-like	6261	0
XM_020104126.1	breast cancer anti-estrogen resistance protein 3-like	3502	0
XM_020104127.1	probable phospholipid-transporting ATPase IH isoform X4	5946	0
XM_020104128.1	methylcrotonoyl-CoA carboxylase subunit alpha, mitochondrial	1744	0
XM_020104129.1	hypoxanthine-guanine phosphoribosyltransferase-like	1339	2.15E-173
XM_020104130.1	E3 ubiquitin-protein ligase ARIH2	1693	0
XM_020104131.1	ankyrin repeat and MYND domain-containing protein 1	3041	0
XM_020104132.1	lysosome-associated membrane glycoprotein 3	726	8.03E-123
XM_020104133.1	fermitin family homolog 3	2104	0
XM_020104134.1	fragile X mental retardation syndrome-related protein 1 isoform X1	3997	0
XM_020104135.1	fragile X mental retardation syndrome-related protein 1 isoform X1	3840	0
XM_020104136.1	fragile X mental retardation syndrome-related protein 1 isoform X3	3762	0
XM_020104137.1	mitochondrial import inner membrane translocase subunit TIM14 isoform X1	597	8.95E-63
XM_020104138.1	mitochondrial import inner membrane translocase subunit TIM14 isoform X1	549	1.63E-68
[truncated: 857,475 more chars]
