# Supplementary material for: Investigating the effects of Carpesii fructus extract on the liver transcriptome of olive flounder (Paralichthys olivaceus) as a potential antiparasitic agent
Source: Genet Mol Biol. 2024 Mar 4;47(1):e20230146. doi: 10.1590/1678-4685-GMB-2023-0146 (PMC10941726; doi:10.1590/1678-4685-GMB-2023-0146)
Supplement: Table S3 - [file 1415-4757-GMB-47-1-e20230146-s6.pdf]

Genetics and Molecular Biology

https://doi.org/10.1590/1678-4685-GMB-2023-0146

Supplementary Material to “Investigating the effects of Carpesii fructus extract on the liver transcriptome of olive flounder (*Paralichthys olivaceus*) as a potential antiparasitic agent”

Table S3 - Formalin DEGs.

Name	FI-12h vs. F-0h - Fold change	FI-12h vs. F-0h - P-value	FI-24h vs. F-0h - Fold change	FI-24h vs. F-0h - P-value	FI-72h vs. F-0h - Fold change	FI-72h vs. F-0h - P-value	FI-3h vs. F-0h - Fold change	FI-3h vs. F-0h - P-value	FI-6h vs. F-0h - Fold change	FI-6h vs. F-0h - P-value	FI-168h vs. F-0h - Fold change	FI-168h vs. F-0h - P-value	
XM_020077998.1	-1263.050081	0.00891359	-2.520340595	1.45387E-06	-1225.10311	0.009380963	-2.57012648	1.86647E-06	-1247.329995	0.01094762	-2.414050367	3.75425E-06	protocadherin gamma-C5-like isoform X4
XM_020078495.1	-5.015255911	1.58509E-08	-5.541606462	1.75575E-08	-2.08353136	0.001025384	-696.6476775	0.032594104	-6.037407401	7.8305E-09	-3.172207804	5.49898E-06	nucleotide exchange factor SIL1 isoform X1
XM_020078631.1	-11.47707373	2.71659E-08	-5.351091083	5.49845E-07	-2.114852156	0.002392221	-27.3553865	3.17331E-06	-2.43400195	0.000565907	-10.47188318	8.93413E-08	cationic amino acid transporter 2-like
XM_020079199.1	-2.514042843	0.000679197	-2.31794969	0.001946486	-2.158400898	0.003408902	-4.102508202	3.0913E-05	-5.618535262	1.83807E-06	-3.913957636	2.11413E-05	single Ig IL-1-related receptor
XM_020079238.1	-981.6892222	0.011644165	-953.7008112	0.014398105	-2.398563479	1.6971E-05	-1011.357091	0.023871922	-969.4709907	0.014122399	-948.7721163	0.014779513	complement C1q tumor necrosis factor-related protein 4-like isoform X2
XM_020079246.1	-796.7949439	0.014444204	-774.0779537	0.017670557	-772.8561036	0.015143104	-820.8750775	0.028464404	-786.8779305	0.017342003	-2.164099913	0.000348954	selenoprotein H
XM_020079321.1	-3.559134445	6.88338E-15	-8.052741793	0	-2.095685133	2.36924E-07	-2.743566961	2.98949E-10	-5.081502987	0	-3.295123508	3.33289E-13	ADP-dependent glucokinase isoform X1
XM_020079911.1	-499.3563221	0.02296488	-485.1194438	0.027458054	-484.3537028	0.023985513	-514.4474909	0.041588465	-493.1412685	0.026981158	-482.6123622	0.028072931	dmX-like protein 2 isoform X1
XM_020080092.1	-2.156396421	0.003604264	-422.6419282	0.031125669	-26.46795379	3.727E-06	-448.1928776	0.046331078	-429.6306388	0.030596173	-420.4577284	0.031798042	aryl hydrocarbon receptor nuclear translocator 2 isoform X3
XM_020080136.1	-4.813441186	1.92219E-05	-3.182706406	0.00040896	-351.7985457	0.032374786	-7.819401292	1.98389E-05	-4.111266204	6.99052E-05	-10.77189707	6.75311E-06	threonine--tRNA ligase, cytoplasmic-like isoform X2
XM_020080938.1	-668.1728372	0.01725383	-649.1229224	0.020922902	-648.0983087	0.018062719	-688.3658509	0.032914278	-2.89349202	1.62042E-05	-645.7682762	0.021427437	actin, alpha cardiac
XM_020080977.1	-10.60564299	0	-1695.62131	0.007944444	-1692.944841	0.006571619	-1798.130624	0.014341009	-2.07444503	2.98849E-07	-2.512984338	8.83608E-10	omega-amidase NIT2 isoform X1
XM_020081117.1	-6.735953383	0	-2.113976143	1.30548E-07	-1755.323739	0.006313006	-1864.385237	0.013876298	-1787.170347	0.007485581	-1749.013027	0.007878523	trafficking protein particle complex subunit 8 isoform X1
XM_020081705.1	-7.480602886	1.69875E-08	-11.40394423	2.73843E-08	-8.011572556	2.15018E-08	-580.7021042	0.037768614	-8.747700357	2.56008E-08	-16.74068119	6.17895E-08	electrogenic sodium bicarbonate cotransporter 4-like
XM_020081970.1	-2.039568129	0.000555911	-5.320580116	1.48197E-09	-2.414754936	4.60609E-05	-845.7205575	0.027764664	-810.6944166	0.016845596	-793.3855316	0.017601645	nuclear pore complex protein Nup214 isoform X4
XM_020081997.1	-1182.661264	0.009564429	-1148.943048	0.011942658	-1147.129488	0.010060307	-1218.402757	0.020331716	-1167.941708	0.011707996	-1143.005347	0.012273269	growth arrest-specific protein 1-like
XM_020082132.1	-15.51986727	3.94129E-14	-2.459573338	8.38294E-06	-13.5030224	6.71685E-14	-1085.893531	0.022461528	-1040.920449	0.013152946	-1018.696079	0.013773691	sodium-dependent phosphate transporter 1-B-like
XM_020082277.1	-780.7171805	0.014747343	-758.4585748	0.018022858	-757.2613793	0.015458324	-804.3114242	0.028951492	-771.0002731	0.017688726	-754.5388855	0.018474532	electrogenic sodium bicarbonate cotransporter 1-like isoform X2
XM_020082662.1	-636.0173105	0.018122717	-617.8841646	0.021923249	-616.9088599	0.018964776	-2.682257486	0.000112911	-628.1013564	0.021528649	-614.6909592	0.022445414	mothers against decapentaplegic homolog 5
XM_020082702.1	-298.3842804	0.037156255	-289.8772074	0.04338759	-289.4196482	0.038652972	-5.379520922	0.000202573	-294.6705509	0.042691333	-288.3791314	0.044234171	inactive rhomboid protein 1-like isoform X2
XM_020082916.1	-4.737948226	0.005310557	-5.773083722	0.004954943	-3.082569729	0.019579716	-7.611070637	0.005936209	-2.998043105	0.022854533	-4.325610225	0.0087284	thyrotroph embryonic factor-like isoform X2
XM_020083069.1	-2.568922329	4.97489E-06	-4.376062103	1.48762E-09	-6.855360022	5.30487E-12	-5.243406968	7.53883E-10	-2.936818152	6.84426E-07	-2.341353624	3.84147E-05	catechol O-methyltransferase domain-containing protein 1-like isoform X1
XM_020083146.1	-4.817346148	0	-29.43431898	0	-2.436252099	1.46779E-11	-14.62111888	0	-2374.643671	0.005500979	-6.833696533	0	histone acetyltransferase p300-like isoform X3
XM_020083276.1	-2.355532562	7.5236E-10	-6.869051425	0	-3.363062044	3.21965E-15	-2005.176291	0.012981486	-2.249712428	6.64072E-09	-2.802341802	4.28946E-12	titin homolog
XM_020083411.1	-2.500053495	0.000602725	-5.718997548	1.36022E-06	-3.344738281	4.12699E-05	-3.924559503	3.13955E-05	-8.267850801	3.47958E-07	-5.146335168	2.58595E-06	mediator of RNA polymerase II transcription subunit 24 isoform X1
XM_020083443.1	-6.128572308	6.49569E-09	-4.950819072	8.03399E-08	-3.199402666	5.16816E-06	-663.5203709	0.033915417	-3.845130263	7.60524E-07	-622.4602885	0.022182246	exocyst complex component 6 isoform X5
XM_020083473.1	-619.9395471	0.018588774	-602.2647857	0.022458847	-601.3141356	0.019448474	-638.6748909	0.034982319	-612.223699	0.022056113	-2.701432957	7.39749E-05	MAGUK p55 subfamily member 2 isoform X2
XM_020083513.1	-3.646363164	1.55981E-08	-899.032985	0.015264548	-2.102773436	0.000223307	-10.08108215	3.69735E-11	-9.62494317	3.13927E-12	-12.42749489	4.52327E-12	interleukin-21 receptor-like
XM_020083610.1	-2.739978637	6.65009E-07	-1031.797706	0.013310627	-2.547041327	3.29633E-06	-3.068594392	3.24521E-07	-3.106275148	9.49052E-08	-9.214622518	3.02092E-13	serine/threonine-protein kinase BRSK1-like isoform X10
XM_020083633.1	-780.7171805	0.014747343	-758.4585748	0.018022858	-757.2613793	0.015458324	-804.3114242	0.028951492	-771.0002731	0.017688726	-754.5388855	0.018474532	pleckstrin homology domain-containing family A member 4 isoform X1
XM_020084045.1	-668.1728372	0.01725383	-4.836323682	6.18627E-08	-2.699445538	3.45885E-05	-688.3658509	0.032914278	-659.8566712	0.020543598	-2.093050646	0.001192358	transcription factor Sp2-like
XM_020084276.1	-427.0063871	0.026669025	-414.8322387	0.031652942	-414.1774431	0.027820013	-439.9110509	0.047006552	-421.6918101	0.031115998	-412.6883991	0.032333352	actin filament-associated protein 1-like 1 isoform X2
XM_020084599.1	-3361.198196	0.002918283	-2.973676483	0	-16.69703141	0	-2.268862644	1.57662E-11	-3319.364287	0.003780326	-9.12903474	0	tuberoinfundibular peptide of 39 residues isoform X2
XM_020084914.1	-467.2007954	0.024481285	-453.880686	0.029179064	-453.1642541	0.025555881	-481.3201842	0.043823838	-461.3859537	0.028677309	-451.5350453	0.029821277	rab9 effector protein with kelch motifs-like isoform X7
XM_020085278.1	-322.5009254	0.034621891	-313.3062758	0.040568481	-312.8117347	0.036037849	-2.204479925	0.013035584	-318.487037	0.039909455	-311.6871191	0.041377172	signal recognition particle 14 kDa protein
XM_020085871.1	-1142.466856	0.0099234	-1109.8946	0.012368213	-4.921953528	8.99991E-12	-1176.993624	0.02095196	-20.21774278	7.60503E-14	-65.83834752	2.34235E-09	transcriptional regulator ATRX-like isoform X2
XM_020085991.1	-660.1339555	0.017463599	-641.3132329	0.02116463	-2.58647737	6.59972E-05	-680.0840242	0.033241076	-651.9178425	0.020781616	-637.9989469	0.021673452	myelin proteolipid protein-like isoform X2
XM_020086059.1	-6.87137215	5.94786E-09	-3.323088409	6.16152E-06	-3.808195927	9.86809E-07	-2.194215932	0.001271685	-4.565514098	2.1385E-07	-2.522095527	0.000169617	nectin-3-like protein isoform X1
XM_020086474.1	-2.292712743	1.58592E-05	-4.284396558	2.35043E-10	-7.533399238	1.08469E-13	-2.439685433	1.21406E-05	-4.359180346	1.43747E-10	-13.4380808	6.92779E-14	PREDICTED: uncharacterized protein KIAA1257 homolog

XM_020086502.1	-402.8897421	0.028171536	-391.4031703	0.033346209	-390.7853566	0.029374051	-415.0655709	0.049165748	-2.967061556	0.000354103	-3.264405466	0.000188915	ubiquitin-conjugating enzyme E2 variant 1-like
XM_020086723.1	-3562.170238	0.002721778	-3460.611127	0.003619792	-3455.148695	0.002891171	-3.529881241	0	-8.150507387	0	-3442.726801	0.003747918	gastrula zinc finger protein XlCGF57.1-like isoform X27
XM_020086781.1	-4.198297919	2.95825E-05	-2.911604757	0.000623786	-4.41654284	2.7342E-05	-8.166024124	1.26816E-05	-15.27532573	4.79268E-06	-5.086572293	1.95936E-05	calpastatin isoform X9
XM_020086786.1	-8.270423143	6.07925E-12	-5.633194881	3.49229E-10	-2.445800131	2.63551E-05	-15.66966951	2.05397E-10	-5.188949843	6.33047E-10	-8.784373455	2.52268E-11	calpastatin isoform X14
XM_020086840.1	-5.1256999	4.81848E-12	-4.033315598	6.35508E-10	-4.485703093	6.2833E-11	-1143.866317	0.021476471	-1096.49225	0.012481711	-32.97577399	1.01249E-11	sorting nexin-32-like
XM_020087052.1	-3.158810636	2.88434E-06	-664.7423013	0.020455078	-663.693033	0.017641484	-18.34385325	2.10582E-08	-675.7343287	0.020082975	-661.3069346	0.020951265	cyclin-dependent kinase 2
XM_020087066.1	-5.301874237	2.24379E-08	-2.068010138	0.001636125	-616.9088599	0.018964776	-2.789091093	7.25435E-05	-6.614996412	1.12468E-08	-4.833767057	1.28261E-07	PREDICTED: uncharacterized protein C1orf50 homolog
XM_020087136.1	-619.9395471	0.018588774	-602.2647857	0.022458847	-601.3141356	0.019448474	-638.6748909	0.034982319	-612.223699	0.022056113	-599.1523008	0.022990333	receptor-type tyrosine-protein phosphatase epsilon-like isoform X1
XM_020087175.1	-5.026814122	1.04779E-05	-7.663220299	4.93395E-06	-2.711675344	0.00092678	-10.15440557	1.14569E-05	-3.381792175	0.000187062	-41.19942607	0.000108683	leucine-rich repeat-containing protein 1
XM_020087623.1	-1624.799756	0.006772169	-3.242190351	4.06541E-10	-1575.984409	0.007142709	-1673.903224	0.015345386	-1604.577287	0.00842625	-3.972785862	3.34488E-12	phosphatidylinositol 5-phosphate 4-kinase type-2 alpha isoform X3

XM_020087877.1	-4.564941202	1.47258E-11	-1125.513979	0.012194626	-1123.737402	0.010281995	-1193.557277	0.02069932	-1144.125222	0.011955691	-1119.697359	0.012530586	receptor-type tyrosine-protein phosphatase-like N
XM_020088285.1	-595.8229021	0.019331844	-3.496150583	4.99773E-06	-4.794328457	1.69828E-07	-613.8294109	0.03612195	-588.4072129	0.022895839	-575.8443131	0.023857602	homeobox protein SIX3 isoform X1
XM_020088436.1	-3.347946345	1.07577E-06	-5.739244202	8.28315E-09	-2.448387988	9.69609E-05	-3.834807628	1.01129E-06	-4.388812805	7.36108E-08	-20.79945439	4.60703E-09	phosphatidylinositol-binding clathrin assembly protein-like isoform X6
XM_020088463.1	-1584.605347	0.006962189	-3.111896045	1.49815E-09	-16.9539603	0	-1632.494091	0.01569521	-1564.883143	0.008651344	-16.01547163	0	transmembrane protease serine 4-like isoform X2
XM_020088623.1	-2.073078203	0.00174932	-4.51825846	5.00946E-07	-2.919865976	3.16148E-05	-605.5475842	0.036519339	-2.837632929	5.1154E-05	-2.867119127	5.25005E-05	transforming growth factor beta-1-like
XM_020088632.1	-306.4231621	0.036272031	-297.6868968	0.04240511	-297.2170104	0.037740756	-2.786609076	0.003502013	-302.6093796	0.041721759	-17.65862575	5.88315E-05	protein moonraker isoform X2
XM_020089059.1	-523.4729671	0.021940616	-508.5485122	0.026292472	-507.7457894	0.022924299	-539.2929709	0.040063871	-516.9577546	0.025832599	-505.9203499	0.026888455	GDNF family receptor alpha-like
XM_020089123.1	-17.78161117	0	-1430.091868	0.009537967	-1427.834527	0.007955435	-1516.548517	0.016765137	-1453.739542	0.009344897	-1422.7012	0.009815727	bromodomain and PHD finger-containing protein 3-like isoform X1
XM_020089433.1	-3.390549077	8.89289E-14	-3.845043769	1.13243E-14	-2.015590568	1.19174E-06	-14.70280672	0	-2.213581375	8.89533E-08	-2.785979115	1.16408E-10	plasma membrane calcium-transporting ATPase 3-like isoform X1
XM_020089578.1	-475.2396771	0.024084526	-461.6903754	0.028729285	-460.9616163	0.025145086	-489.6020109	0.043241382	-469.3247824	0.028233996	-459.3043745	0.029364417	histone-lysine N-methyltransferase SUV39H1 isoform X1
XM_020090282.1	-8.564319163	5.37578E-11	-2.51236331	4.51068E-05	-749.4640171	0.015620584	-6.030514796	6.25291E-09	-2.942030309	4.15571E-06	-746.7695562	0.018659148	growth factor receptor-bound protein 14 isoform X1
XM_020090726.1	-410.9286238	0.027652896	-4.200630467	2.58616E-05	-398.5827188	0.028837707	-2.994871812	0.000526913	-3.668885713	5.8893E-05	-397.1497407	0.033459372	importin subunit alpha-7 isoform X1
XM_020090798.1	-2.678550614	8.54872E-15	-6.863588402	0	-5.641896725	0	-2.208623003	4.66716E-10	-8.897723721	0	-2735.71784	0.004851763	gamma-aminobutyric acid type B receptor subunit 2 isoform X2
XM_020091151.1	-29.36047852	8.78028E-09	-664.7423013	0.020455078	-663.693033	0.017641484	-704.9295042	0.032280349	-675.7343287	0.020082975	-39.43215386	1.82815E-07	tumor necrosis factor receptor superfamily member 6B-like
XM_020091401.1	-8.49836508	2.22045E-16	-1297.327147	0.010549058	-1295.27937	0.008837955	-13.23750157	7.66054E-15	-1318.779454	0.010338314	-2.238206469	1.26568E-05	ATPase family AAA domain-containing protein 2 isoform X1
XM_020091459.1	-7.695302714	0	-2.719141311	1.07581E-13	-3.410044721	0	-4.625409407	0	-5.575464714	0	-9.342727606	0	rhotekin-2-like isoform X1
XM_020091484.1	-402.8897421	0.028171536	-391.4031703	0.033346209	-390.7853566	0.029374051	-415.0655709	0.049165748	-397.875324	0.032785529	-389.3804114	0.034052063	28S ribosomal protein S18a, mitochondrial
XM_020091710.1	-459.1619138	0.024890764	-4.336142024	8.08087E-06	-445.3668919	0.025979787	-473.0383576	0.044423227	-453.447125	0.029134489	-443.7657161	0.030292361	gap junction delta-2 protein-like
XM_020091818.1	-4.626559305	0	-3.418912482	0	-2.491318109	0	-4.520499261	0	-4.376936996	0	-5.324973694	0	serine/threonine-protein phosphatase 2A 56 kDa regulatory subunit gamma isoform-like isoform X2
XM_020091985.1	-2316.143579	0.004540093	-2250.109261	0.005883615	-2246.557556	0.004803465	-2386.140317	0.011082081	-2287.316555	0.005757181	-2238.480769	0.006073213	clathrin coat assembly protein AP180-like isoform X10
XM_020092119.1	-6.990931641	4.71845E-14	-2.845346102	2.66404E-07	-2.070609207	0.000104924	-6.299856474	5.07461E-12	-3.708892392	1.4289E-09	-8.743907816	5.89528E-14	PREDICTED: liprin-alpha-2-like
XM_020092158.1	-2.923615059	0	-2.711542324	2.22045E-16	-3439.553971	0.002906836	-32.24812839	0	-3.192023172	0	-204.3550476	1.4877E-14	copine-5-like isoform X2
XM_020092192.1	-10.41956539	1.05413E-07	-461.6903754	0.028729285	-3.824047548	1.12161E-05	-24.83483554	6.29642E-06	-469.3247824	0.028233996	-459.3043745	0.029364417	39S ribosomal protein L42, mitochondrial isoform X2
XM_020092262.1	-603.8617838	0.019077998	-586.6454067	0.023020363	-585.7194112	0.019956105	-622.1112375	0.035733521	-596.3460416	0.022609144	-4.58938326	3.6145E-07	zinc finger CCHC-type and RNA-binding motif-containing protein 1
XM_020092309.1	-4.361431616	6.10527E-07	-4.021712787	2.44587E-06	-2.113234749	0.001912347	-14.89566475	2.26681E-07	-5.77890009	1.45167E-07	-16.50192983	7.289E-08	cleavage and polyadenylation specificity factor subunit 6 isoform X2
XM_020092324.1	-5.38740008	7.77156E-16	-3.459371169	1.89956E-11	-3.622126325	3.34199E-12	-2.319162084	7.35745E-07	-3.519758059	9.95448E-12	-3.147241838	1.95046E-10	3-oxo-5-beta-steroid 4-dehydrogenase
XM_020092368.1	-507.3952038	0.022613482	-492.9291333	0.027058465	-492.151065	0.023621485	-522.7293176	0.041066797	-501.0800972	0.026587388	-490.3816915	0.0276669	protein kinase C and casein kinase substrate in neurons protein 2 isoform X4
XM_020092886.1	-732.4838905	0.015732348	-711.600438	0.019165196	-710.4772062	0.016482217	-754.6204642	0.030522104	-2.246484535	0.000280793	-2.294504947	0.000231856	PREDICTED: uncharacterized protein C8orf88 homolog
XM_020092928.1	-9.721018713	1.05827E-10	-5.009343775	1.35347E-08	-6.283437086	1.20982E-09	-4.220800891	2.37839E-07	-2.302467885	0.000199032	-707.92291	0.019638017	zinc finger protein 585A-like isoform X2
XM_020093428.1	-6.262738969	4.06616E-06	-2.332975752	0.003717844	-2.442310442	0.00222609	-6.828837625	1.68484E-05	-2.635577856	0.001295248	-7.577215775	5.57176E-06	phosphatase and actin regulator 4A-like isoform X2
XM_020093443.1	-2.993910397	2.22045E-16	-3.829591627	0	-3.787565297	0	-12.52981936	0	-16.47953435	0	-5.02603317	0	cathepsin S-like
XM_020093795.1	-1544.410939	0.007162063	-1500.379073	0.009072639	-2.441120107	5.45865E-07	-1591.084957	0.016061306	-1525.189	0.008887801	-1492.625163	0.009339755	usherin-like isoform X2
XM_020093999.1	-2.216252876	0.000646672	-594.4550962	0.022736248	-593.5167734	0.019699188	-630.3930642	0.035353743	-604.2848703	0.022329317	-591.3829715	0.023272531	breakpoint cluster region protein
XM_020094348.1	-2380.454633	0.004377777	-2312.586777	0.005685041	-5.816170144	0	-2.618076684	6.34393E-12	-2350.827185	0.005562304	-2300.635403	0.005869673	60S ribosomal protein L19 isoform X1
XM_020094511.1	-3.866182698	1.71016E-05	-4.846808492	6.61404E-06	-421.9748053	0.027336799	-448.1928776	0.046331078	-6.011902225	1.99909E-06	-420.4577284	0.031798042	tubulin-specific chaperone D isoform X1
XM_020094711.1	-764.6394172	0.01506258	-742.8391959	0.018388843	-2.995941252	2.9183E-06	-787.7477708	0.029456126	-755.1226157	0.018048939	-4.052343559	8.24679E-08	RUN domain-containing protein 3A-like isoform X1
XM_020094786.1	-603.8617838	0.019077998	-586.6454067	0.023020363	-4.57542226	2.16619E-07	-2.877284202	7.25638E-05	-24.3527999	6.06836E-08	-583.6136423	0.023561537	calcium-activated potassium channel subunit alpha-1-like isoform X8
XM_020095238.1	-636.0173105	0.018122717	-617.8841646	0.021923249	-616.9088599	0.018964776	-655.2385442	0.03426342	-628.1013564	0.021528649	-3.102393622	1.18334E-05	RAC-beta serine/threonine-protein kinase-like
XM_020095277.1	-394.8508604	0.028709303	-383.5934809	0.033951173	-382.9879944	0.029930083	-406.7837442	0.049933602	-389.9364953	0.033382079	-2.00585021	0.011663778	Friend leukemia integration 1 transcription factor isoform X1
XM_020095362.1	-644.0561921	0.017897869	-625.693854	0.021664611	-624.7062221	0.01873138	-663.5203709	0.033915417	-2.248656852	0.000519586	-622.4602885	0.022182246	5,6-dihydroxyindole-2-carboxylic acid oxidase
XM_020095373.1	-6.127355824	0	-3.287567988	4.44089E-16	-3.566682028	0	-5.45399234	0	-2.740416731	3.87246E-13	-4.233325368	0	RING finger protein B-like isoform X1

XM_020095756.1	-933.4559322	0.012271869	-3.035131748	5.57181E-07	-905.4112608	0.012882502	-961.6661308	0.024917233	-2.135815799	0.000182088	-902.1561409	0.015530894	adhesion G protein-coupled receptor L3-like isoform X10
XM_020096139.1	-1528.333176	0.007244942	-1484.759694	0.009172365	-1482.416062	0.0076373	-1574.521304	0.016212561	-1509.311342	0.008985757	-1477.086504	0.009441775	heterogeneous nuclear ribonucleoprotein A1-like isoform X1
XM_020096150.1	-386.8119788	0.029267285	-375.7837914	0.034578295	-3.318173634	0.000163255	-4.676458817	5.08613E-05	-4.817569904	1.73095E-05	-2.939792627	0.000540837	fibulin-2-like isoform X1
XM_020096187.1	-370.7342154	0.030448675	-5.03534114	2.27202E-05	-6.746639937	5.42421E-06	-2.701932616	0.001879658	-7.631385379	5.28844E-06	-11.01064844	5.44779E-06	sentrin-specific protease 5-like isoform X2
XM_020096631.1	-11.2757056	4.58042E-06	-10.41058693	9.47029E-06	-7.336118311	8.60305E-06	-18.11336631	5.17797E-05	-10.59204378	8.13318E-06	-5.961017615	2.30575E-05	dual specificity protein phosphatase 13-like
XM_020096645.1	-2.802195433	3.53422E-07	-6.085502898	2.96585E-12	-2.164957585	6.08682E-05	-1119.020837	0.021887685	-6.806957113	7.19869E-13	-1049.773396	0.013367183	nucleolar protein 8 isoform X3
XM_020096837.1	-2.085225081	0.00087529	-2.113952397	0.000920447	-2.764400358	2.13246E-05	-2.346408127	0.000364636	-3.731833911	5.7077E-07	-5.936579762	1.05286E-08	TBC1 domain family member 20 isoform X1
XM_020096885.1	-603.8617838	0.019077998	-586.6454067	0.023020363	-585.7194112	0.019956105	-622.1112375	0.035733521	-596.3460416	0.022609144	-583.6136423	0.023561537	erythroid differentiation-related factor 1 isoform X2
XM_020097150.1	-555.6284938	0.02070332	-539.78727	0.024880911	-538.9352381	0.021641803	-572.4202775	0.038205274	-548.7130694	0.024441867	-536.9976669	0.025453582	WASH complex subunit FAM21-like isoform X1
XM_020097333.1	-579.7451388	0.019859193	-3.757506289	3.07915E-06	-3.933537805	1.38369E-06	-597.2657575	0.036926016	-572.5295555	0.023490883	-560.3056546	0.024471985	SH3 and PX domain-containing protein 2B isoform X2
XM_020097479.1	-2.102527553	7.55647E-09	-2.661718718	1.36702E-12	-16.46640643	0	-2369.576664	0.011119531	-2.115704658	9.40036E-09	-2222.942111	0.006093123	laminin subunit alpha-2-like
XM_020097571.1	-4.003148455	0.000781464	-3.288385617	0.002672033	-6.122408915	0.000232613	-3.288134984	0.003986584	-2.018751739	0.03719456	-3.651417299	0.001715707	eukaryotic translation initiation factor 2 subunit 1
XM_020097659.1	-660.1339555	0.017463599	-641.3132329	0.02116463	-640.3009465	0.01828053	-680.0840242	0.033241076	-651.9178425	0.020781616	-637.9989469	0.021673452	rho-associated protein kinase 2-like isoform X2
XM_020097679.1	-2.600296198	2.12879E-05	-735.0295064	0.018577219	-733.8692927	0.01595487	-779.4659442	0.029715332	-2.320448851	0.000149941	-731.2308978	0.01903921	retinol dehydrogenase 14-like
XM_020098041.1	-2.369962626	1.22446E-12	-4.784999428	0	-5.427158063	0	-5.261054668	0	-5.752009343	0	-7.373241008	0	sodium-dependent multivitamin transporter-like
XM_020098153.1	-700.3283638	0.016460151	-680.3616802	0.020006988	-679.2877574	0.017238404	-721.4931575	0.031671313	-691.6119861	0.019641813	-5.011762683	2.79607E-08	syntaxin-binding protein 5-like isoform X1
XM_020098465.1	-8.759346191	0	-2.896298468	9.37709E-10	-2.878782618	6.48692E-10	-11.0149847	7.77156E-16	-1294.962968	0.010494274	-2.083802645	3.9952E-06	AT-rich interactive domain-containing protein 3A isoform X2
XM_020098802.1	-587.7840205	0.019592159	-571.0260278	0.023609743	-570.1246868	0.020489493	-605.5475842	0.036519339	-580.4683842	0.023189662	-568.0749838	0.024160992	programmed cell death protein 2 isoform X2
XM_020098837.1	-660.1339555	0.017463599	-641.3132329	0.02116463	-640.3009465	0.01828053	-2.263708175	0.000669551	-39.13419708	1.94992E-07	-637.9989469	0.021673452	putative hexokinase HKDC1 isoform X2
XM_020098876.1	-3039.642929	0.003289439	-2952.981312	0.004333345	-2948.320153	0.003489084	-3131.504717	0.008535097	-6.171307186	0	-2937.7204	0.004481678	60S ribosomal protein L5-like
XM_020099468.1	-700.3283638	0.016460151	-680.3616802	0.020006988	-679.2877574	0.017238404	-721.4931575	0.031671313	-2.588418038	5.11832E-05	-2.375950873	0.00018342	guanylyl cyclase-activating protein 2-like
XM_020099921.1	-772.6782988	0.014903405	-750.6488853	0.01820409	-749.4640171	0.015620584	-9.341484861	1.12098E-09	-763.0614444	0.017867098	-746.7695562	0.018659148	DNA-(apurinic or apyrimidinic site) lyase isoform X2
XM_020099989.1	-2.271901328	0.001500215	-461.6903754	0.028729285	-460.9616163	0.025145086	-24.83483554	6.29642E-06	-469.3247824	0.028233996	-459.3043745	0.029364417	zinc finger protein 568-like isoform X1
XM_020100327.1	-4.784163771	1.84494E-06	-2.504356361	0.000789139	-3.169930834	6.7553E-05	-3.646357499	5.30282E-05	-2.172352191	0.003019147	-4.053448817	1.2162E-05	synaptopodin-2-like isoform X1
XM_020100520.1	-973.6503406	0.011744619	-2.735369022	2.25554E-06	-944.3980717	0.012333347	-1003.075264	0.024039874	-961.532162	0.014238479	-941.0027871	0.014899906	coiled-coil domain-containing protein 149-like isoform X1
XM_020100525.1	-3.683269394	0.000962228	-9.91546135	0.000150137	-7.855912716	0.000123901	-6.706217072	0.000409456	-3.882158172	0.000986727	-2.528254809	0.009797702	adhesion G protein-coupled receptor L3-like isoform X1
XM_020100752.1	-25.89325083	4.41657E-06	-399.2128598	0.032762231	-398.5827188	0.028837707	-423.3473976	0.048422768	-405.8141527	0.032209706	-3.565229683	8.54604E-05	guanine nucleotide exchange factor DBS isoform X1
XM_020100806.1	-2.809657558	0.000149424	-2.208560517	0.002352304	-2.082051305	0.003793875	-8.712949338	9.31581E-07	-2.092815774	0.003858841	-14.35316754	3.47507E-07	serine/threonine-protein kinase RIO2
XM_020100960.1	-9.110664397	9.26635E-08	-6.563927223	5.1968E-07	-2.01517709	0.005283812	-8.712949338	9.31581E-07	-3.779761912	1.29954E-05	-27.85048997	2.61004E-06	ubiquitin carboxyl-terminal hydrolase 30 isoform X3
XM_020101327.1	-2.184733957	0.000519056	-3.053518762	9.05745E-06	-5.376497002	1.52952E-08	-12.04637775	1.14637E-08	-2.469571009	0.000127702	-645.7682762	0.021427437	serine/threonine-protein kinase DCLK2 isoform X9
XM_020101493.1	-57.04000718	2.44249E-15	-1703.430999	0.007938024	-37.11095839	0	-1806.412451	0.014323326	-2.374911725	5.15144E-07	-1694.627723	0.008178537	teleost multiple tissue opsin-3a
XM_020101596.1	-3.655987729	0	-12.9358565	0	-24.28189665	0	-10.3515204	0	-15.43798208	0	-15.67941693	0	protein SFI1 homolog
XM_020102004.1	-3.358510387	9.45442E-05	-5.472080021	7.42853E-06	-2.001388281	0.010009371	-415.0655709	0.049165747	-397.875324	0.032785529	-389.3804114	0.034052063	cadherin-4-like isoform X3
XM_020102188.1	-3.235536557	1.25076E-07	-2.982854904	8.67092E-07	-3.123102571	2.94901E-07	-2.518349299	2.51732E-05	-905.960361	0.015104403	-2.039627804	0.000449913	ankyrin repeat and BTB/POZ domain-containing protein BTBD11-B isoform X2
XM_020102309.1	-3.811010976	1.0951E-07	-719.4101275	0.018965281	-2.395389228	9.55961E-05	-3.688055921	8.78536E-07	-7.70192163	4.92411E-10	-3.473900335	7.72647E-07	zinc finger E-box-binding homeobox 2 isoform X4
XM_020102385.1	-346.6175704	0.032404928	-336.7353441	0.03809425	-336.2038213	0.033748864	-2.705242777	0.002509926	-342.3035231	0.037468415	-334.9951068	0.038868687	endoplasmic reticulum mannosyl-oligosaccharide 1,2-alpha-mannosidase
XM_020102473.1	-3.225618935	0	-3.636257104	0	-4.844506598	0	-3388.241344	0.007909453	-3247.914829	0.003875135	-3.595007352	0	protein PRRC2A-like
XM_020102886.1	-17.29575794	1.11022E-16	-4.129199692	3.95416E-11	-6.73155532	8.21565E-15	-8.908428121	2.76446E-14	-1287.024139	0.010601112	-10.55951043	1.22125E-15	28S ribosomal protein S7, mitochondrial
XM_020103022.1	-3296.887143	0.002986474	-3202.891375	0.003953355	-3197.835743	0.003170084	-5.104801455	0	-3255.853657	0.003864392	-3186.338936	0.00409103	rab GTPase-binding effector protein 2
XM_020103243.1	-1263.050081	0.00891359	-2.367887447	5.29116E-06	-1225.10311	0.009380963	-1301.221024	0.019196268	-1247.329995	0.01094762	-10.95826228	2.44249E-15	membrane-bound transcription factor site-2 protease
XM_020104206.1	-418.9675054	0.027152372	-407.0225492	0.032198147	-3.37124979	8.42431E-05	-2.547553387	0.001697765	-2.022640882	0.008577407	-2.128363245	0.005810728	CCR4-NOT transcription complex subunit 6
XM_020104808.1	-579.7451388	0.019859193	-563.2163384	0.023915544	-562.3273247	0.020766465	-597.2657575	0.036926016	-572.5295555	0.023490883	-560.3056546	0.024471985	neuropeptide FF receptor 2
XM_020104999.1	-3.435397711	1.09915E-11	-2.928536442	1.39332E-09	-4.16335577	2.94542E-13	-2.507426397	1.62517E-07	-2.771026743	4.38902E-09	-2.558260865	4.04595E-08	integrator complex subunit 6 isoform X1
XM_020105086.1	-555.6284938	0.02070332	-13.4336127	4.00536E-08	-538.9352381	0.021641803	-572.4202775	0.038205274	-548.7130694	0.024441867	-3.560469304	8.23675E-06	60S ribosomal protein L17
XM_020105112.1	-708.3672455	0.016272353	-688.1713697	0.019789955	-687.0851196	0.01704331	-729.7749842	0.031375662	-699.5508148	0.019428147	-684.6149223	0.020274159	cyclic AMP-responsive element-binding protein 1
XM_020105211.1	-724.4450088	0.015908574	-703.7907486	0.019369195	-702.679844	0.016665342	-746.3386375	0.030801221	-715.4284722	0.019013932	-700.1535808	0.019845746	nucleolar GTP-binding protein 1
XM_020105496.1	-274.2676354	0.040082736	-3.357305787	0.00133008	-266.0275616	0.04167075	-5.912975721	0.00028178	-6.746675248	9.32435E-05	-15.80555953	0.000117756	trace amine-associated receptor 13c-like
XM_020105552.1	-732.4838905	0.015732348	-3.475535287	7.83501E-07	-3.380005745	7.97482E-07	-2.869273381	1.94738E-05	-2.302467885	0.000199032	-2.630678248	3.50274E-05	neuronal tyrosine-phosphorylated phosphoinositide-3-kinase adapter 2 isoform X1
XM_020105629.1	-1118.350211	0.010150853	-33.58941692	7.81575E-12	-2.07013472	0.000120419	-1152.148144	0.021342862	-1104.431079	0.012391051	-16.86641501	1.0858E-13	endoplasmic reticulum mannosyl-oligosaccharide 1,2-alpha-mannosidase-like
XM_020106014.1	-2.819747512	0.000243231	-430.4516176	0.030615444	-2.288630794	0.00207395	-456.4747042	0.045675986	-2.224196129	0.002948885	-428.2270576	0.031279987	exosome complex component MTR3
XM_020106439.1	-1214.816791	0.009293973	-1180.181805	0.011621517	-1178.318937	0.009778053	-1251.530064	0.019861633	-1199.697023	0.011392324	-1174.082664	0.011945262	rho guanine nucleotide exchange factor 11-like

XM_020107781.1	-997.7669856	0.011447947	-4.559748678	4.61091E-10	-967.7901582	0.012024262	-2.237037234	9.9098E-05	-985.3486481	0.013895521	-15.04783734	1.17173E-12	cysteine and tyrosine-rich protein 1
XM_020108113.1	-427.0063871	0.026669025	-414.8322387	0.031652942	-2.205585476	0.003409736	-439.9110509	0.047006552	-421.6918101	0.031115998	-412.6883991	0.032333352	protein NLRC5
XM_020108215.1	-10.49117471	9.29409E-06	-3.045570143	0.001047204	-19.62080777	2.95935E-05	-16.85309083	8.1477E-05	-3.655649886	0.000309741	-5.546267302	5.29153E-05	constitutive coactivator of peroxisome proliferator-activated receptor gamma isoform X1
XM_020108496.1	-812.8727072	0.014152485	-789.6973326	0.017331186	-788.450828	0.014839706	-837.4387308	0.027993936	-802.7555879	0.017008024	-785.6162024	0.017769837	NAD-dependent protein deacylase sirtuin-5, mitochondrial
XM_020108917.1	-41.08952342	1.28918E-07	-2.683301963	5.49082E-05	-27.01357596	2.07416E-08	-671.8021975	0.033574714	-3.89312382	5.85421E-07	-3.180818453	7.19929E-06	transcription factor SOX-18-like
XM_020109679.1	-668.1728372	0.01725383	-649.1229224	0.020922902	-648.0983087	0.018062719	-688.3658509	0.032914278	-659.8566712	0.020543598	-645.7682762	0.021427437	palmitoyltransferase ZDHHC3-like isoform X2
XM_020110042.1	-4.151892328	2.19824E-13	-3.126407957	3.70514E-10	-2.326514142	3.06434E-07	-7.533398246	9.54792E-15	-8.620743008	1.11022E-16	-4.449565761	3.32401E-13	prokineticin receptor 1-like
XM_020110205.1	-603.8617838	0.019077998	-586.6454067	0.023020363	-585.7194112	0.019956105	-2.365437063	0.000618473	-3.605162481	2.83441E-06	-2.377761402	0.000397024	E3 ubiquitin-protein ligase HECW2
XM_020110837.1	-676.2117188	0.017048753	-39.40160866	1.83718E-07	-4.588058065	5.41561E-08	-696.6476775	0.032594104	-667.7954999	0.020310774	-653.5376054	0.021186766	PREDICTED: osteocrin
XM_020110905.1	-571.7062571	0.020133211	-555.4066489	0.024229134	-554.5299625	0.021050648	-588.9839309	0.037342321	-564.5907268	0.023799787	-552.5363254	0.024790873	low-density lipoprotein receptor-related protein 6-like
XM_020111760.1	-563.6673755	0.020414489	-547.5969595	0.024550816	-546.7326003	0.021342327	-580.7021042	0.037768614	-556.6518981	0.024116676	-544.7669961	0.025117964	rho GTPase-activating protein 30
XM_020112181.1	-772.6782988	0.014903405	-750.6488853	0.01820409	-6.217407123	5.48934E-10	-796.0295975	0.029201555	-2.942030309	4.15571E-06	-746.7695562	0.018659148	guanylyl cyclase-activating protein 2-like
XM_020112193.1	-11.85821511	1.60982E-13	-6.046514838	2.14844E-11	-2.542093078	6.01201E-06	-5.969211624	1.49049E-10	-2.939314512	5.39994E-07	-56.57301641	8.97563E-09	MAP7 domain-containing protein 1-like
XM_020114014.1	-8.354084076	1.75415E-14	-6.037831517	1.58329E-12	-2.652275252	9.60313E-07	-17.45003897	1.25111E-12	-6.143212748	9.67781E-13	-11.38431887	4.57412E-14	switch-associated protein 70-like

XR_002202336.1	-4.946870149	0	-2.187565916	2.45332E-11	-2.086513274	1.7678E-10	-3.394459005	0	-4.071330313	0	-7.616012832	0	transcriptional repressor NF-X1
XR_002202752.1	-8.522329484	4.2558E-08	-500.7388227	0.026670094	-3.905411224	4.55681E-06	-531.0111442	0.040558782	-9.121414513	8.43443E-08	-498.1510207	0.027272234	semaphorin-5A isoform X1
XR_002203147.1	-31.27918581	0	-13.30513065	0	-4.474277932	0	-91.76422325	4.10783E-15	-45.82465415	0	-61.9052898	0	---NA---

XR_002203320.1	-6.644638878	0.011316265	-6.144442135	0.015315849	-3.311659074	0.037834072	-5.510611502	0.023203578	-3.222406996	0.042542442	-3.131853401	0.047859843	---NA---
XR_002203466.1	-3.359267578	1.07692E-14	-4.368571889	0	-12.89823351	0	-3.473518749	1.24567E-13	-3.19501423	1.57763E-13	-6.470551887	0	---NA---
XR_002203636.1	-3.806509456	0.002427546	-4.085136543	0.002539387	-6.341161502	0.000687476	-4.353172981	0.003262136	-2.790306111	0.011212474	-3.045224743	0.00816375	---NA---

Table S3.2 - Carpesii Fructus DEGs

Name	HI-12h vs. H-0h - Fold change	HI-12h vs. H-0h - P-value	HI-24h vs. H-0h - Fold change	HI-24h vs. H-0h - P-value	HI-72h vs. H-0h - Fold change	HI-72h vs. H-0h - P-value	HI-3h vs. H-0h - Fold change	HI-3h vs. H-0h - P-value	HI-6h vs. H-0h - Fold change	HI-6h vs. H-0h - P-value	HI-168h vs. H-0h - Fold change	HI-168h vs. H-0h - P-value	HO-12h vs. H-0h - Fold change	HO-12h vs. H-0h - P-value	HO-24h vs. H-0h - Fold change	HO-24h vs. H-0h - P-value	HO-72h vs. H-0h - Fold change	HO-72h vs. H-0h - P-value	HO-3h vs. H-0h - Fold change	HO-3h vs. H-0h - P-value	HO-6h vs. H-0h - Fold change	HO-6h vs. H-0h - P-value	HO-168h vs. H-0h - Fold change	HO-168h vs. H-0h - P-value	
XM_020077992.1	-11.79164024	0	-5.748649572	0	-4.086397793	4.44089E-16	-7.577931059	0	-3.977020432	2.43361E-13	-10.16164512	0	-6.530620301	0	-3.325707518	1.32572E-12	-3.865773889	1.00031E-13	-54.63966002	2.22045E-15	-5.736094387	0	-3.187870092	1.62493E-11	protocadherin gamma-C5-like isoform X4
XM_020078079.1	-4.837087703	0	-2.431696114	0	-9.39045028	0	-6.143430843	0	-6.378545109	0	-42.10800622	0	-3.30072487	0	-3.710501251	0	-8.290344951	0	-4.127290552	0	-2.665527023	0	-19.10851512	0	inositol 1,4,5-trisphosphate receptor type 1-like
XM_020078442.1	-747.3952993	0.023235049	-11.24325944	3.45789E-10	-4.816899068	2.0416E-09	-5.146145939	3.1515E-08	-7.453922315	3.41914E-09	-16.28671641	6.98064E-12	-841.537418	0.020095042	-744.3534661	0.017000634	-7.433295489	1.04397E-09	-948.1948945	0.01902773	-825.1245137	0.009818186	-5.853479991	5.37966E-09	excitatory amino acid transporter 2 isoform X2
XM_020078445.1	-6.327749473	4.29455E-05	-4.934949544	7.6736E-05	-42.66543391	8.46382E-05	-17.28298779	7.16103E-05	-11.66478048	4.29655E-05	-6.270939334	5.32624E-06	-5.435251474	2.7649E-05	-3.021999288	0.00088739	-18.6109841	4.26646E-05	-3.031072534	0.000515374	-2.541350702	0.00158695	-12.44366847	2.66073E-05	LIM domain only protein 3 isoform X2
XM_020079439.1	-28.55862208	2.28003E-06	-4.142613426	4.93144E-06	-5.404975652	1.16686E-07	-9.310279005	4.22288E-07	-2.356551201	0.001420109	-4.398072758	4.29005E-07	-4.027881441	3.06214E-06	-510.4673098	0.024461677	-54.91395187	2.93704E-05	-8.372205187	1.42027E-08	-565.8589768	0.014842062	-497.1485357	0.030346712	vascular endothelial growth factor receptor 3
XM_020079902.1	-2785.410501	0.006461177	-2674.293011	0.004886855	-2826.027926	0.002326112	-2947.507597	0.009743566	-2606.630523	0.006806996	-2997.891332	0.002343761	-3136.348694	0.005413696	-44.59758738	0	-2674.124728	0.005185241	-3533.853344	0.005146315	-3075.178995	0.001989799	-2.615688688	2.23821E-13	disks large homolog 3 isoform X3
XM_020079913.1	-730.6215116	0.023714248	-4.438093265	5.87019E-08	-21.17618798	7.67089E-11	-773.1400651	0.031560447	-683.7269882	0.024862601	-786.3558695	0.011369287	-3.763186476	1.6632E-07	-727.6473121	0.017385074	-701.410826	0.020403943	-926.913755	0.019429496	-4.106763941	8.40739E-09	-708.6620217	0.022029747	protein FAM189A1 isoform X1
XM_020080931.1	-2131.232782	0.008504463	-3.769393006	0	-3.606913716	0	-2255.259975	0.012487355	-1994.440826	0.008951479	-2293.810654	0.003272888	-2399.742605	0.007201108	-2122.611173	0.005670905	-2046.077037	0.006956041	-2703.888904	0.006838069	-2352.939285	0.002820098	-2067.229412	0.007708268	alpha-aminoadipic semialdehyde dehydrogenase
XM_020080945.1	-3196.368299	0.005593188	-3068.856601	0.004189226	-3242.978395	0.001948891	-3382.381103	0.008554058	-2.584884756	8.08242E-14	-24.70366975	0	-3599.088416	0.004670275	-7.54769667	0	-3068.667509	0.004453535	-7.918457043	0	-3528.893684	0.001661884	-3100.39144	0.004990075	protein SZT2-like isoform X1
XM_020081068.1	-554.4967411	0.030261505	-3.739859321	5.79701E-06	-5.847175161	2.40924E-08	-586.7657052	0.039363121	-518.9066853	0.031692825	-6.567714739	6.91201E-09	-4.116951035	1.15542E-06	-552.2326948	0.022705222	-2.771722496	0.000129312	-2.493494457	0.000183962	-6.909436318	2.68546E-09	-6.487815154	1.7803E-07	unconventional myosin-Va-like
XM_020082219.1	-1007.389008	0.017675452	-967.2015607	0.01430172	-1022.078961	0.007983743	-1066.014059	0.024186823	-942.7303215	0.018553944	-1084.236156	0.00796177	-2.458807701	8.48353E-06	-1003.298854	0.012598699	-967.1233108	0.014974904	-1278.052557	0.014383836	-1112.168501	0.006985533	-977.1214462	0.016288403	serine/threonine-protein kinase TAO3-like isoform X1
XM_020082853.1	-3.775543829	4.57613E-06	-556.5333345	0.02433637	-588.1101057	0.014683849	-3.364464395	2.05788E-05	-542.4524428	0.030495343	-8.896077965	9.16572E-10	-4.078671486	7.99894E-07	-577.2919259	0.021763493	-556.4767434	0.025318301	-735.3834995	0.023970244	-639.9348445	0.012997338	-4.86119701	5.45692E-07	liprin-alpha-3-like isoform X1
XM_020082861.1	-4.354521828	3.78813E-06	-500.1671074	0.026862392	-528.545753	0.016442738	-3.190719734	7.38552E-05	-487.5123419	0.03344535	-560.6889856	0.016308718	-586.5583874	0.027853041	-518.8203868	0.024089763	-500.113489	0.02791308	-660.8995112	0.02635995	-575.1184603	0.014586082	-8.662751863	1.7593E-07	myosin light chain kinase, smooth muscle-like
XM_020082904.1	-4.322722873	1.82964E-11	-18.21249936	8.88178E-16	-39.40749218	2.22045E-15	-5.562425767	7.9925E-13	-6.521452894	1.13132E-13	-1463.356521	0.005640538	-1530.925168	0.011345965	-17.47147526	1.11022E-16	-1305.302837	0.011102452	-10.06538802	0	-1501.066806	0.004903489	-1318.797077	0.01216383	lethal(3)malignant brain tumor-like protein 2 isoform X1
XM_020082960.1	-5.098887695	0	-3.433117361	0	-6.166885108	0	-4.062436806	0	-2.825433602	0	-4.606387832	0	-3.361292428	0	-4.353945618	0	-6.717868807	0	-13.30008395	0	-5.727694806	0	-3.231592203	0	cytoplasmic polyadenylation element-binding protein 4-like isoform X4
XM_020083293.1	-6.408094623	0	-3.979517617	0	-4.785027265	0	-2.905010513	0	-3.880330762	0	-97.49544851	0	-4.707929799	0	-2.421807963	0	-5.182392363	0	-3.01098124	0	-37.07112026	0	-3.350345383	0	NADH dehydrogenase [ubiquinone] 1 alpha subcomplex assembly factor 8
XM_020083443.1	-2.051743898	0.000472663	-886.6783791	0.015584473	-2.922163552	3.88035E-07	-977.264364	0.026017173	-864.2444629	0.020104693	-2.867386269	4.24772E-07	-1039.854442	0.016489057	-919.7680834	0.01376211	-5.033719397	6.87822E-10	-4.891541349	6.34597E-11	-2.126702722	9.74613E-05	-895.7701054	0.017694679	elongator complex protein 1
XM_020083634.1	-2441.547854	0.007432344	-2344.147966	0.005676482	-2477.151004	0.002764993	-22.36633271	0	-2284.838503	0.007826424	-2627.797642	0.002781642	-2.20277697	6.90135E-10	-2431.675023	0.004849037	-2.028932233	3.89904E-08	-88.31329742	0	-2695.540173	0.00235849	-2368.229373	0.006658499	poly(ADP-ribose) glycohydrolase-like isoform X2
XM_020083681.1	-5460.829634	0.003128674	-5242.982504	0.002252279	-5540.460568	0.000955151	-6.720437675	0	-5110.329411	0.003303297	-5877.40077	0.000970158	-6148.878723	0.002576844	-5438.782747	0.001882778	-5242.678748	0.002413015	-6928.195096	0.002457536	-6028.954218	0.000804378	-42.76974238	0	deoxycytidylate deaminase isoform X1
XM_020083808.1	-15.80117813	0	-2843.391692	0.004563989	-3.677193027	0	-3133.881957	0.009196496	-3.247631339	0	-3187.451514	0.002168281	-3.491384473	0	-4.052724442	0	-2843.214491	0.004846775	-3757.305309	0.00482103	-3269.628147	0.001836982	-2.80331039	3.10862E-15	rho GDP-dissociation inhibitor 1-like
XM_020084098.1	-453.8540151	0.035944634	-435.7485621	0.030458301	-460.4722071	0.018987456	-480.266071	0.046011694	-3.83630084	4.67793E-05	-488.4755828	0.018813529	-511.0090449	0.031439117	-451.9957707	0.027411944	-25.74868687	4.67502E-06	-11.04868795	5.89307E-08	-9.125471374	2.07105E-08	-5.310198107	4.18639E-06	leucine-rich repeat protein SHOC-2
XM_020084289.1	-2.741490326	7.20212E-05	-604.8472435	0.022513132	-639.1652651	0.013430399	-666.6404309	0.035569053	-4.620594339	5.82553E-07	-8.800832649	2.19043E-10	-3.51384902	1.75617E-06	-4.523208563	1.57097E-07	-604.7881042	0.023443489	-5.480959299	6.70297E-09	-4.458864064	1.96212E-08	-611.0404128	0.025227914	kelch-like ECH-associated protein 1
XM_020084323.1	-5.306145339	0	-3.562663717	0	-10.83765201	0	-4.95768437	0	-3.507025615	0	-6.055127721	0	-8764.774704	0.001708462	-4.603367343	0	-13.01556668	0	-12.5848283	0	-5.819716834	0	-3.715593736	0	interleukin enhancer-binding factor 3-like isoform X3
XM_020084952.1	-3.982133785	0	-11.98968392	0	-8.180661617	0	-4.761286075	0	-2.361135329	3.68233E-10	-13.57084824	0	-2.765142418	7.33857E-14	-10.5736664	0	-20.20066018	0	-2.108568341	2.8682E-09	-2.105196391	1.60971E-09	-11.61713155	0	cysteine-rich hydrophobic domain-containing protein 2-like
XM_020085600.1	-7.055920061	0	-2.561950781	0	-6.361770961	0	-4.042135702	0	-3.049851456	0	-2.980040552	0	-5.138035589	0	-3.386551333	0	-4.037030517	0	-11.6911909	0	-4.085135234	0	-2.689751022	0	BICD family-like cargo adapter 2 isoform X1
XM_020085697.1	-4420.854799	0.003949086	-4244.495052	0.002888557	-4485.320607	0.001271375	-4678.126653	0.006248994	-4137.104765	0.004166734	-4758.093026	0.001287961	-4977.863915	0.003270367	-4403.001198	0.002431716	-3.371728974	0	-5608.764446	0.003115489	-4880.778269	0.001076027	-4288.121015	0.003488312	transmembrane protein 135
XM_020085744.1	-5485.990315	0.003112693	-2.242277268	1.90958E-14	-3.510247339	0	-3.534670119	0	-5133.875168	0.00328647	-10.93000282	0	-6177.209726	0.002563375	-5463.841978	0.001872213	-43.74123819	0	-133.5594025	0	-8.014910057	0	-10.29351737	0	acetyl-CoA carboxylase 1 isoform X5
XM_020086008.1	-1275.769611	0.014142291	-1224.875742	0.011275642	-1294.373144	0.006078342	-1350.013084	0.019767688	-1193.885069	0.014858115	-1373.089768	0.006074785	-1436.48849	0.012079388	-1270.597318	0.009866385	-1224.783902	0.011840721	-1618.550789	0.011451576	-1408.471972	0.005291485	-1237.445737	0.012952475	transcription initiation factor TFIID subunit 1-like isoform X5
XM_020086126.1	-1569.310895	0.011534663	-13.58122454	0	-5.554622229	0	-6.666157821	0	-2.256476731	2.07258E-07	-4.597417129	0	-3.054536932	4.62042E-12	-4.334631866	5.55112E-16	-1506.600174	0.009550994	-3.286344207	1.10245E-13	-25.33624483	0	-14.16413793	0	V-type proton ATPase catalytic subunit A
XM_020086260.1	-906.7462821	0.019485442	-2.04813922	0.000474396	-6.107770961	5.1692E-12	-959.514425	0.026418123	-2.637730789	1.39611E-05	-3.43112758	1.77648E-08	-1020.967106	0.016777141	-903.0619294	0.014019258	-5.176237418	6.72032E-10	-6.394693035	3.2776E-12	-3.290551833	2.57662E-08	-3.057624492	9.71247E-07	F-box only protein 50 isoform X3
XM_020086263.1	-7.610770107	4.82639E-08	-2.556064195	0.000211725	-3.632944799	1.08962E-06	-3.413143847	1.60019E-05	-3.087646316	4.3087E-05	-2.338848518	0.00026153	-3.279939509	7.22574E-06	-2.929496298	3.24127E-05	-5.402823099	1.88126E-07	-4.833717041	5.25939E-08	-4.349963208	6.20477E-08	-5.307354676	2.53452E-07	Down syndrome cell adhesion molecule homolog
XM_020086405.1	-579.6574226	0.029113016	-556.5333345	0.02433637	-588.1101057	0.014683849	-3.19711799	3.37622E-05	-542.4524428	0.030495343	-623.8757131	0.014576057	-652.664062	0.025324691	-577.2919259	0.021763493	-556.4767434	0.025318301	-40.76968819	1.62312E-07	-639.9348445	0.012997338	-562.2296083	0.027195707	histone deacetylase 11
XM_020086617.1	-1124.805522	0.015939694	-1079.934015	0.012809679	-1141.207666	0.007036055	-2.146605866	0.000110254	-1052.610523	0.016738762	-1210.609611	0.007023699	-1266.502469	0.013655215	-1120.241932	0.011249208	-1079.849819	0.013430673	-1427.020533	0.012941232	-1241.801269	0.00614185	-3.494476319	1.14266E-08	sulfatase-modifying factor 1
XM_020087044.1	-2.583959888	1.49301E-09	-1579.177741	0.00863493	-1668.777646	0.004471982	-3.714276091	1.72751E-13	-4.134004741	1.40998E-14	-1770.263483	0.00448081	-3.29685512	1.22573E-10	-1638.132706	0.007529625	-1579.067215	0.009133187	-2086.735858	0.008902476	-1815.889244	0.003892161	-17.51496509	0	claudin-19 isoform X1
XM_020087086.1	-4.623616531	0	-2.852423148	7.51155E-12	-7.219413466	0	-2.108124689	2.8161E-07	-3.462638783	8.54872E-14	-9.53464052	0	-7.732494921	0	-3.634087193	1.11022E-15	-3.765470049	1.66533E-15	-7.829048186	0	-5.292712552	0	-2.763364117	3.13223E-11	lysine-specific demethylase PHF2 isoform X2
XM_020087258.1	-6.047640827	0	-3.135303048	0	-6.566085312	0	-2.610530719	1.36557E-14	-2.683714182	3.44169E-15	-2.831241399	0	-3.350369058	0	-3.522146884	0	-4.227385898	0	-9.643925968	0	-6.321618093	0	-2.482151417	7.56062E-14	solute carrier family 35 member G2
XM_020087676.1	-4.277037697	0	-3.604840398	0	-2.200845172	1.02864E-09	-5.327634636	0	-3.954150253	0	-160.8053384	1.82632E-13	-9.576359999	0	-2.742922767	5.32019E-13	-2126.595972	0.006647399	-2810.294601	0.006544896	-7.69768369	0	-2148.580753	0.007375232	phospholipid scramblase 2-like isoform X2
XM_020088464.1	-537.7229534	0.031079225	-516.2717437	0.026089996	-545.5641395	0.015902279	-569.0157662	0.040326213	-503.2095136	0.032545271	-3.959336973	7.38197E-07	-605.445723	0.027080795	-535.5265408	0.023377758	-516.217276	0.027119976	-682.1806507	0.025630033	-593.6374272	0.014097516	-521.553938	0.029083575	protein KIAA0100 homolog isoform X1
XM_020088776.1	-4.001449841	0	-2.437399132	0	-5.90753869	0	-2.397993206	0	-2.321431162	0	-4.69046491	0	-2.888920072	0	-3.692246452	0	-5.595856807	0	-6.081832626	0	-4.08031572	0	-3.057379448	0	ATP synthase subunit epsilon, mitochondrial
XM_020088791.1	-361.5981829	0.043478792	-347.1730623	0.037293707	-17.14103465	1.87052E-06	-3.968128656	0.000191206	-338.3892106	0.045457009	-4.282935389	1.59393E-05	-407.1286991	0.038219987	-4.646300907	3.16556E-05	-347.1275129	0.038600739	-458.7286859	0.036160676	-3.668609231	3.77076E-05	-3.532699048	0.000269977	synaptonemal complex protein 2 isoform X3
XM_020088808.1	-6.998551001	2.64155E-12	-2.729596999	1.26163E-06	-4.835795108	5.60274E-12	-4.942918727	2.39149E-10	-3.125681355	2.2149E-07	-3.270877467	6.54898E-09	-2.487204705	4.38846E-06	-4.098248504	5.03954E-10	-4.95990543	7.33038E-11	-11.33500992	1.66533E-15	-3.510511342	9.36775E-10	-7.432311461	1.58729E-12	RBBP8 N-terminal-like protein isoform X1
XM_020089041.1	-739.0084055	0.023472224	-709.5273796	0.019348373	-749.7847771	0.011289879	-782.0150346	0.031268548	-691.5755741	0.024609936	-795.3825449	0.011228837	-832.0937502	0.020305419	-736.0003891	0.017190817	-709.4627195	0.020184865	-937.5543248	0.019226552	-815.8650303	0.009943034	-716.7971558	0.021798829	collagen alpha-1(XIX) chain-like
XM_020089168.1	-30.33520729	0	-2497.142011	0.005295602	-2638.825675	0.00255304	-2752.258268	0.010421888	-2433.961634	0.007334948	-2799.304474	0.002570188	-2928.588002	0.005822474	-2590.383486	0.004514492	-2496.983072	0.00558879	-3299.76081	0.005533339	-4.298054853	0	-2522.79692	0.006227172	TELO2-interacting protein 1 homolog
XM_020089196.1	-1745.435666	0.010423852	-1675.805559	0.008143949	-1770.887965	0.004184508	-1847.011377	0.015002793	-1633.405877	0.010963784	-1878.583588	0.004194906	-2.468721096	7.58247E-10	-2.766599371	2.44617E-11	-5.521172518	0	-2214.422695	0.008354425	-1927.003045	0.003597795	-50.14993175	7.66054E-15	agrin isoform X6
XM_020089439.1	-1611.245364	0.011279037	-7.539748246	0	-13.24550584	0	-6.59177598	2.10942E-15	-1507.828503	0.011859902	-4.102680482	9.448E-14	-1814.235202	0.009580142	-1604.720398	0.007698706	-1546.859641	0.009330098	-2044.173579	0.009088475	-10.49741893	0	-1562.8511	0.01026528	cAMP-dependent protein kinase type II-alpha regulatory subunit
XM_020089513.1	-780.9428746	0.022332004	-749.7889704	0.018348732	-4.824674391	1.04616E-09	-826.3898822	0.029889618	-730.8185034	0.023419403	-4.033420038	7.59027E-09	-879.3120892	0.019294563	-777.7657742	0.016278184	-749.7221869	0.019154311	-990.7571736	0.018271216	-20.8103735	2.46014E-12	-757.4728261	0.020711783	copine-5-like isoform X2
XM_020090008.1	-1258.995823	0.014322359	-3.74269157	5.69716E-10	-1277.354758	0.006173139	-1332.263145	0.019995196	-1178.187897	0.015046571	-1355.036417	0.006168757	-5.273399592	3.32512E-13	-1253.891164	0.010004204	-36.80245915	2.13762E-12	-1597.269649	0.01160061	-3.319123969	4.73586E-10	-1221.175468	0.013121995	alkyldihydroxyacetonephosphate synthase, peroxisomal
XM_020090011.1	-663.526361	0.025844873	-637.0565161	0.021438187	-4.913145082	8.89508E-09	-2.93081492	3.19084E-05	-620.9383014	0.027086316	-714.1424667	0.012618391	-747.10074	0.022413056	-660.822696	0.019102987	-636.9956782	0.022337308	-2.735967812	1.67783E-05	-5.990182843	4.16932E-10	-643.5809491	0.024065186	cyclin-T2-like isoform X2
XM_020090164.1	-780.9428746	0.022332004	-749.7889704	0.018348732	-792.3307433	0.010623844	-826.3898822	0.029889618	-730.8185034	0.023419403	-840.5159217	0.010571262	-879.3120892	0.019294563	-777.7657742	0.016278184	-749.7221869	0.019154311	-990.7571736	0.018271216	-862.1624476	0.009345662	-757.4728261	0.020711783	PREDICTED: secernin-3
XM_020090688.1	-697.0739363	0.024734006	-669.2657887	0.020458166	-707.2388109	0.012035144	-36.72457862	3.61038E-07	-652.3326448	0.025927041	-750.2491681	0.011964332	-784.8754112	0.021425586	-694.2350041	0.01820558	-669.2032521	0.021328241	-884.351476	0.020285196	-769.567613	0.0106123	-2.652678988	5.51355E-05	trafficking kinesin-binding protein 1-like isoform X1
XM_020090979.1	-1326.090974	0.013627389	-1273.189651	0.010838431	-8.192605849	0	-1403.262901	0.019115629	-1240.976584	0.014319165	-3.074867361	2.59684E-09	-1493.150496	0.011588603	-2.12781475	1.70834E-06	-2.047019544	6.88376E-06	-2.465045576	1.20047E-08	-1464.028872	0.005026238	-1286.256541	0.012424687	regulation of nuclear pre-mRNA domain-containing protein 1A isoform X1
XM_020091033.1	-839.6511315	0.020908171	-4.254806283	1.64855E-08	-851.8950959	0.009802502	-4.212105968	5.00539E-08	-7.097335782	6.43376E-10	-4.815917644	2.08678E-10	-3.043175567	8.91583E-07	-2.019275345	0.000683868	-3.056832449	1.71193E-06	-1065.241162	0.017080083	-3.913970338	2.87569E-09	-4.708033092	7.1995E-09	protein ATP1B4
XM_020091113.1	-3.476099868	0	-2.132028513	0	-3.015193799	0	-2.802363824	0	-2.159493295	0	-3.051050745	0	-2.868299181	0	-3.194764765	0	-3.586311347	0	-3.442360283	0	-2.288771432	0	-2.24566762	0	regulator of G-protein signaling 22
XM_020091172.1	-11.74429077	0	-3068.856601	0.004189226	-3242.978395	0.001948891	-3382.381103	0.008554058	-2991.21123	0.005895249	-4.107873637	0	-5.724599297	0	-3183.451954	0.003563944	-3068.667509	0.004453535	-4055.241262	0.004442251	-11.59977331	0	-3100.39144	0.004990075	V-type proton ATPase subunit C 1-A

XM_020091319.1	-3.442791079	2.37223E-08	-4.394468141	3.06828E-10	-2.641885018	7.27404E-07	-4.783072421	3.20073E-10	-13.1684333	1.23512E-12	-5.24484284	6.76126E-13	-2.308057669	1.75742E-05	-9.316678647	5.68434E-14	-1039.590352	0.013946133	-39.16798879	1.03784E-12	-14.60444356	1.11022E-16	-1050.337653	0.015195411	CCR4-NOT transcription complex subunit 10 isoform X1
XM_020091378.1	-5.361536645	0	-4.539763611	0	-6.211617578	0	-2.411984099	0	-6.271045647	0	-7.132171413	0	-6.833830627	0	-5.493712222	0	-9.984425334	0	-10.50719425	0	-16.87253028	0	-6.158539315	0	non-canonical poly(A) RNA polymerase PAPD7-like isoform X3
XM_020091536.1	-3.852514297	4.44089E-16	-6.030530287	0	-9.877877432	0	-6.275045565	0	-4.356932474	1.11022E-16	-2077.170445	0.003695097	-6.637288425	0	-5.567009595	0	-1852.831593	0.007696421	-7.743715224	0	-2.339737375	2.34746E-10	-5.558482256	0	transcription regulator protein BACH2-like
XM_020091845.1	-10.57570408	1.58015E-11	-870.5737428	0.015867586	-919.9686418	0.008993881	-959.514425	0.026418123	-848.5472912	0.020445822	-975.916052	0.008960803	-1020.967106	0.016777141	-903.0619294	0.014019258	-870.500589	0.016593475	-12.14666732	1.26232E-13	-1001.054699	0.00788697	-879.4998373	0.018004496	PREDICTED: microcephalin
XM_020092337.1	-915.133176	0.019320876	-878.6260609	0.015724818	-928.477835	0.008901159	-968.3893945	0.026216065	-856.3958771	0.020273848	-984.9427274	0.008869134	-1030.410774	0.01663189	-911.4150064	0.013889568	-4.574501836	2.44479E-09	-1161.00629	0.015754731	-1010.314183	0.007804139	-887.6349713	0.017848287	putative homeodomain transcription factor 2 isoform X3
XM_020092845.1	-445.4671213	0.036517729	-427.6962439	0.030975361	-451.9630139	0.019357046	-471.3911015	0.046676672	-3.064584771	0.000284354	-479.4489074	0.019177151	-2.385734019	0.001289715	-443.6426937	0.027890595	-427.6464477	0.032132016	-565.1343834	0.030236062	-491.7831092	0.017226009	-2.497717394	0.001256816	PREDICTED: uncharacterized protein LOC109633199
XM_020092956.1	-6.799052405	0	-5.398474312	0	-8.588028498	0	-2.56089943	1.26453E-10	-3.714608831	6.66134E-16	-5.298581366	0	-3.250805465	1.66533E-15	-4.03535267	0	-8.062711315	0	-8.580627425	0	-12.1124731	0	-4.72844358	0	myocardin-like isoform X4
XM_020093161.1	-4.934035362	2.2633E-12	-1289.294287	0.01069966	-1362.44669	0.005723496	-1421.01284	0.018907859	-1256.673756	0.01414781	-34.02508305	2.22045E-16	-1512.037832	0.011485771	-7.899194639	7.77156E-16	-1289.19905	0.011243092	-94.45182374	9.28034E-11	-1482.547839	0.004977049	-3.448311329	1.74633E-09	protein 4.1-like isoform X1
XM_020093203.1	-3.695227249	2.13741E-10	-2.199415697	5.82021E-06	-4.667413838	6.9833E-14	-5.05866469	5.58942E-12	-6.06090406	8.94618E-13	-2.163193485	2.15052E-06	-6.133635549	5.9841E-13	-5.08880259	1.17526E-11	-3.005397071	1.91128E-07	-9.56734527	1.66533E-15	-11.15707951	1.11022E-16	-3.932020722	2.04825E-09	polypeptide N-acetylgalactosaminyltransferase 1
XM_020093226.1	-537.7229534	0.031079225	-516.2717437	0.026089996	-545.5641395	0.015902279	-569.0157662	0.040326213	-503.2095136	0.032545271	-578.7423363	0.015776444	-605.445723	0.027080795	-535.5265408	0.023377758	-516.217276	0.027119976	-682.1806507	0.025630033	-593.6374272	0.014097516	-521.553938	0.029083575	rho guanine nucleotide exchange factor 1 isoform X1
XM_020093332.1	-495.7884843	0.033333044	-476.0101529	0.028108635	-503.0181733	0.01731945	-524.6409186	0.042969134	-463.9665843	0.034894151	-533.6089596	0.017171925	-558.2273839	0.029097531	-493.7611558	0.025239788	-475.9578086	0.029192177	-628.977802	0.027536261	-547.3400099	0.01537931	-480.8782676	0.031251367	lethal(3)malignant brain tumor-like protein 3 isoform X5
XM_020093359.1	-831.2642376	0.021100504	-798.1028793	0.017272694	-2.630251509	6.01468E-06	-879.6396993	0.028393048	-777.9100185	0.022133205	-894.6759738	0.009868906	-935.974096	0.018204338	-827.8842363	0.015297382	-798.0335477	0.018044253	-1054.600592	0.017240861	-917.7193483	0.008708706	-806.2836306	0.019539349	putative Ras-related protein Rab-42
XM_020093409.1	-7222.077339	0.002281889	-213.9351272	0	-7327.391147	0.00064811	-131.1831922	0	-6758.53244	0.002411299	-7773.002595	0.000660688	-10.66335591	0	-7192.92892	0.001330962	-6933.576378	0.001729445	-106.3113735	0	-7973.445745	0.000542169	-7005.255796	0.001983914	Na(+)/H(+) exchanger beta-like isoform X1
XM_020093443.1	-2022.203162	0.008967369	-2.904330947	7.7871E-13	-12.49319525	0	-2139.885371	0.013099723	-1892.40921	0.009436999	-2176.463874	0.003490553	-2.322920079	9.07635E-10	-4.775074703	0	-1941.402422	0.007325396	-2.978383091	1.82077E-14	-8.685228696	0	-1961.472669	0.008108161	polycomb protein SCMH1 isoform X1
XM_020093542.1	-2.140565682	0.00115517	-2.749940528	4.51652E-05	-664.6928448	0.012874314	-3.144260662	1.58673E-05	-613.0897155	0.027392485	-705.1157913	0.012792149	-737.6570722	0.022674084	-652.469619	0.019340637	-628.9437847	0.022604215	-831.1486273	0.021465131	-723.2701957	0.011366889	-2.970292923	2.0848E-05	Golgi-specific brefeldin A-resistance guanine nucleotide exchange factor 1 isoform X2
XM_020093566.1	-9.988788138	5.28695E-11	-2.545945162	2.18762E-05	-868.9134824	0.00958846	-906.2646079	0.027701563	-2.956749586	4.01526E-06	-921.7559999	0.009548479	-964.3050995	0.017703106	-852.9434673	0.014847729	-822.1892282	0.017534422	-1086.522301	0.01676715	-945.4977987	0.008418512	-830.6890328	0.019000313	vacuolar protein sorting-associated protein 33A
XM_020093600.1	-5.208333566	0	-2.960262111	4.81792E-12	-4.542600661	0	-3.111315456	4.23594E-12	-2.882271827	3.90947E-11	-5.091297619	0	-6.089255862	0	-3.109349277	4.1378E-13	-6.257249928	0	-12.48705752	0	-7.410328898	0	-2.276361507	2.60885E-08	phosphatidylethanolamine-binding protein 4
XM_020093706.1	-2.961074767	9.64046E-06	-4.438093265	5.87018E-08	-6.006193541	2.94906E-10	-3.103552215	7.75916E-06	-3.836276886	6.70314E-07	-3.147116916	7.17788E-07	-2.967814594	4.31526E-06	-2.561517922	4.22911E-05	-5.82524065	6.19744E-09	-5.691604867	4.94803E-10	-3.505452731	9.16717E-08	-2.006633809	0.001658756	dihydrofolate reductase
XM_020093725.1	-7.269575718	0	-4.23510039	0	-6.565798151	0	-4.221089674	0	-2.693121459	2.24265E-14	-4.660173263	0	-5.18426112	0	-4.459257542	0	-6.633925239	0	-24.05134873	0	-7.422728006	0	-2.831862517	7.77156E-16	serine/threonine-protein phosphatase 4 regulatory subunit 3B isoform X1
XM_020093726.1	-12.79872216	5.16774E-09	-8.888004411	3.38066E-09	-16.1029323	1.7609E-10	-5.614549511	7.95951E-08	-65.78177415	1.28847E-05	-20.08012099	1.82304E-10	-747.10074	0.022413056	-3.437082158	1.77737E-06	-4.672451161	1.32174E-07	-5.772844011	1.99112E-09	-2.462428033	5.24943E-05	-3.398636686	3.91664E-06	homeobox protein Nkx-3.1-like
XM_020093812.1	-4.204957569	0	-2.540208675	2.03171E-14	-3.797404335	0	-3586.505402	0.008088298	-3.444195603	0	-3647.811957	0.001823824	-4.178732323	0	-2.449454823	6.4837E-14	-3.851530363	0	-2.741542815	0	-2.545593505	5.55112E-16	-4.555224109	0	rhotekin isoform X4
XM_020093823.1	-1200.287566	0.014989019	-1152.404878	0.01199683	-1217.790405	0.00652637	-1270.138358	0.020835216	-1123.247796	0.01574419	-1291.849689	0.006518821	-1351.495479	0.012821122	-1195.419625	0.010515861	-1152.316861	0.012588499	-2.288310937	7.47333E-06	-1325.136621	0.005688995	-1164.22953	0.013750095	armadillo repeat protein deleted in velo-cardio-facial syndrome isoform X4
XM_020093984.1	-7.970276763	0	-7.569753842	0	-53.73726517	2.33147E-15	-4.236016975	8.58202E-14	-2.467965307	3.34913E-08	-14.40577271	0	-2.447321981	7.82739E-09	-4.508800818	8.88178E-16	-10.51956434	0	-12.99699064	0	-20.14708615	0	-14.59509997	0	RIMS-binding protein 2 isoform X8
XM_020094129.1	-5.774062048	0	-3.267844953	0	-8.323812431	0	-397.2777176	0	-7056.778703	0.002295107	-9.161788315	0	-3.027329141	0	-460.728696	0	-7.508099511	0	-9567.056394	0.001694553	-5.612263073	0	-3.306335718	0	PREDICTED: synaptotagmin-3
XM_020094515.1	-437.0802274	0.037109757	-5.85473497	2.98856E-06	-443.4538207	0.019740154	-4.003968946	4.69281E-05	-409.0264833	0.038828115	-11.07460071	5.71349E-08	-4.164461993	1.00657E-05	-435.2896167	0.028385786	-4.737620022	9.52913E-06	-7.1392113	2.06111E-07	-5.446265523	3.06108E-07	-6.373190675	2.87981E-06	ADP-ribosylation factor-like protein 16 isoform X1

XM_020094613.1	-3598.939203	0.004931089	-3455.367873	0.003661852	-3651.41967	0.001669763	-2.731472706	5.55112E-16	-3367.943351	0.005199416	-3873.478841	0.001687383	-2.419504514	3.38618E-14	-4.293180724	0	-3.285000836	0	-4565.98861	0.003906781	-3973.34889	0.001419978	-4.016591929	0	forkhead box protein K1
XM_020095138.1	-2.391718186	0.0008622	-508.2194255	0.026470688	-537.0549462	0.016168373	-560.1407967	0.040826808	-2.015779368	0.005793626	-569.715661	0.016038522	-2.150153066	0.001839452	-527.1734638	0.023728612	-508.1653825	0.027510908	-671.540081	0.025989873	-584.3779438	0.014338019	-513.4188039	0.029492823	14-3-3 protein epsilon isoform X1
XM_020095328.1	-3.196061565	0	-3.081960623	0	-2.117042158	5.96131E-11	-3471.130799	0.00834531	-4.036191253	0	-11.06747019	0	-4.452328429	0	-3266.982724	0.003458394	-2.285028344	7.74714E-12	-2.994945668	0	-3.177404872	0	-10.46553402	0	poly(A) polymerase gamma isoform X2
XM_020095820.1	-2.161259836	3.94574E-05	-1232.92806	0.011200502	-1302.882337	0.006031897	-1358.888053	0.019655893	-1201.733655	0.014765588	-1382.116443	0.006028741	-1445.932157	0.012002011	-1278.950395	0.009798783	-1232.835796	0.011762778	-1629.191359	0.011378425	-1417.731455	0.005250309	-2.810128418	1.91359E-07	meiosis 1 arrest protein
XM_020095871.1	-579.6574226	0.029113016	-556.5333345	0.02433637	-588.1101057	0.014683849	-613.3906138	0.038006477	-542.4524428	0.030495343	-623.8757131	0.014576057	-652.664062	0.025324691	-577.2919259	0.021763493	-556.4767434	0.025318301	-20.96602263	5.29217E-09	-639.9348445	0.012997338	-562.2296083	0.027195707	ATP-dependent RNA helicase DDX39A
XM_020096208.1	-1829.304604	0.009949488	-27.51869898	0	-33.49646246	0	-16.75774461	0	-95.75016637	7.5008E-11	-68.71665518	1.11022E-16	-40.21696557	0	-1821.900401	0.006709136	-103.7875705	3.2226E-11	-33.56222088	0	-19.78569485	0	-1774.364585	0.009023503	probable palmitoyltransferase ZDHHC16 isoform X2
XM_020096477.1	-5.026482268	9.88098E-15	-2.568154881	1.48343E-08	-4.422450081	7.77156E-16	-2.656491581	1.59611E-08	-2.523286092	5.51875E-08	-3.663100967	4.38538E-14	-6.320424286	0	-3.624826222	1.09657E-12	-3.048131684	2.22687E-10	-6.152006689	0	-5.875539283	0	-2.156800133	1.62464E-06	UPF0160 protein MYG1, mitochondrial
XM_020097676.1	-5.651638194	0	-2.744597913	0	-3.930141192	0	-3.663879574	0	-2.675195343	0	-2.818856597	0	-3.961495455	0	-2.506620109	0	-4.20654402	0	-5.562995118	0	-3.542947737	0	-2.212456357	0	protein disulfide-isomerase A6 isoform X1
XM_020098354.1	-554.4967411	0.030261505	-532.37638	0.02535982	-562.5825259	0.01539347	-586.7657052	0.039363121	-2.659621838	0.000286843	-7.746345011	3.04244E-09	-624.3330586	0.026350073	-552.2326948	0.022705222	-8.229468074	6.79927E-08	-703.4617902	0.024939379	-8.148974225	1.22606E-09	-3.835412925	5.7761E-06	plasmalemma vesicle-associated protein-like
XM_020098511.1	-4.775149191	7.53012E-07	-6.896627124	6.59047E-08	-2.73613593	4.69059E-05	-2.627999465	0.000274389	-29.90166212	1.58487E-06	-17.28820739	1.64328E-09	-3.819866285	1.78792E-06	-568.9388489	0.022068944	-11.24661211	3.41198E-08	-7.652532678	3.64538E-09	-3.721763272	5.43831E-07	-3.362322308	1.5494E-05	SH3-containing GRB2-like protein 3-interacting protein 1 isoform X7
XM_020098753.1	-5.370032401	1.11022E-16	-3.641386687	1.84408E-13	-9.210172361	0	-3.654800913	8.84737E-13	-2.096435097	1.62453E-06	-9.613041259	0	-2.818035076	6.41575E-11	-8.042375661	0	-26.2762207	0	-2.080845027	3.41289E-07	-2.514860946	3.30332E-10	-8.311896059	0	zinc finger protein 821 isoform X3
XM_020098864.1	-4.878920433	6.54699E-13	-5.52523331	4.36318E-14	-1473.066202	0.005218938	-1536.387444	0.017661282	-1358.705372	0.013123649	-3.090416001	8.43286E-10	-1634.805513	0.010631475	-1446.011935	0.008606415	-4.984871716	3.1819E-13	-1842.002754	0.010082634	-13.87208478	0	-2.860123885	4.24408E-08	transcriptional enhancer factor TEF-1 isoform X1
XM_020099027.1	-3.316483953	9.18918E-06	-2.609124959	0.000120153	-3.294467635	2.18203E-06	-2.021048649	0.003253951	-581.6953721	0.028689675	-5.644702753	3.73365E-09	-699.882401	0.023781064	-3.498256215	2.64178E-06	-596.7362108	0.023736888	-788.5863483	0.022511334	-11.13214941	7.6726E-11	-602.9052787	0.025536097	spindlin-W-like isoform X2
XM_020099110.1	-437.0802274	0.037109757	-419.6439257	0.03151003	-443.4538207	0.019740154	-462.516132	0.047362644	-409.0264833	0.038828115	-470.4222321	0.019554029	-492.1217093	0.032485332	-435.2896167	0.028385786	-419.5945542	0.032679962	-554.4938137	0.030738725	-482.5236257	0.017573687	-423.9323291	0.034892231	zinc finger protein 366
XM_020099293.1	-1208.67446	0.014890135	-1160.457197	0.011912469	-1226.299598	0.006473754	-1279.013328	0.02071084	-1131.096382	0.015640723	-1300.876365	0.006466686	-1360.939147	0.012734442	-1203.772702	0.01043983	-1160.368754	0.012501056	-1533.426231	0.012070833	-1334.396104	0.005642284	-1172.364664	0.013656882	leucine-rich repeats and immunoglobulin-like domains protein 1
XM_020099368.1	-9.332084023	5.92789E-08	-16.67395354	6.6707E-08	-4.167926288	3.73584E-07	-6.855442382	2.44744E-07	-6.168551808	3.69922E-07	-21.14436304	4.44871E-09	-4.697754868	3.05191E-07	-7.232890693	3.18173E-08	-9.526329072	4.41552E-08	-5.201035005	4.84796E-08	-8.272235687	9.00709E-10	-5.993800251	2.13317E-07	lipoma HMGIC fusion partner-like 2 protein
XM_020099481.1	-2005.429374	0.009075838	-1925.427422	0.007025372	-2034.672955	0.003531785	-3.084111454	3.94723E-10	-1876.712038	0.00955059	-2158.410524	0.003545617	-4.985311646	0	-2.660427029	9.51228E-12	-1925.298635	0.007390378	-3.468151712	0	-2214.047033	0.003026687	-1945.202401	0.00817832	PREDICTED: uncharacterized protein LOC109637239 isoform X3
XM_020099890.1	-3.654164092	8.65974E-14	-14.23446578	0	-17.34436239	0	-1740.511743	0.015774464	-5.04965863	2.22045E-16	-3.813856081	1.11022E-16	-2.761915114	4.67159E-11	-3.010243743	4.72378E-12	-5.988129367	0	-4.444294416	0	-2.832312648	1.89981E-12	-1595.391636	0.01001694	serine/threonine-protein kinase B-raf isoform X4
XM_020099893.1	-1963.494905	0.009270375	-10.84903038	0	-1992.126989	0.00362473	-10.82971534	0	-6.574150181	0	-2113.277147	0.003638126	-2210.869249	0.007836372	-16.89348513	0	-1885.039167	0.007588195	-138.1054285	2.01428E-12	-2167.749615	0.003125825	-109.5845608	1.93932E-11	growth arrest and DNA damage-inducible protein GADD45 alpha-like
XM_020100115.1	-13.73148172	0	-2247.520148	0.005917931	-14.46213979	0	-234.9582888	1.14706E-08	-2.880383374	2.61791E-13	-2519.477538	0.002914525	-2.940050595	6.77236E-15	-15.1382097	0	-3.757871447	0	-2.618975709	2.66343E-13	-2584.426371	0.002488868	-3.009588909	1.85407E-14	dr1-associated corepressor isoform X1
XM_020100634.1	-453.8540151	0.035944634	-435.7485621	0.030458301	-460.4722071	0.018987456	-480.266071	0.046011694	-424.723655	0.037614743	-2.424497636	0.000631639	-511.0090449	0.031439117	-451.9957707	0.027411944	-3.019587521	0.000195196	-575.7749532	0.029749702	-501.0425926	0.016890733	-440.2025972	0.033767962	P2Y purinoceptor 8
XM_020100647.1	-62961.37376	0.000147866	-60449.67582	8.59532E-05	-63879.48938	2.22696E-05	-95.50259448	0	-92.9410838	0	-256.9833647	0	-525.4938287	0	-62707.47875	6.55246E-05	-60446.46044	9.59904E-05	-2.571763698	0	-113.1784883	0	-61071.35686	0.000118511	probable ubiquitin carboxyl-terminal hydrolase FAF-X isoform X5
XM_020100725.1	-3078.951785	0.005818159	-2956.124147	0.004369394	-3123.849689	0.002045482	-3258.13153	0.008863954	-2881.331028	0.00613161	-3313.824969	0.002063335	-3466.877067	0.004862702	-3066.508876	0.003721651	-2955.941	0.004642638	-3906.273286	0.004624519	-5.995038264	0	-2986.499563	0.005196625	ras-related protein Rab-33B-like isoform X1
XM_020100918.1	-6.856131477	0	-4.004029882	0	-9.94197364	0	-3.197144236	0	-3.124643486	0	-4.803772235	0	-3.556285704	0	-6.290788968	0	-7.897135222	0	-7.885878045	0	-5.086887352	0	-3.293909424	0	homer protein homolog 1-like isoform X2
XM_020100978.1	-2.945796894	0.004748341	-14.51551442	0.000200549	-4.624792052	0.000206529	-2.31370952	0.020073331	-3.934338055	0.001584726	-3.876202634	0.000393909	-2.107774394	0.024309121	-4.043496617	0.000717174	-3.748223619	0.001380976	-2.197256287	0.015605214	-2.924569459	0.001803776	-2.954964012	0.004624346	WD repeat-containing protein 31 isoform X1
XM_020101054.1	-1082.871053	0.016520116	-1039.672424	0.013307499	-6.423955339	7.54952E-14	-1145.888785	0.022751643	-1013.367594	0.017345844	-1165.476234	0.007335102	-9.03772215	3.21965E-14	-1078.476547	0.011698995	-1039.590352	0.013946133	-1373.817685	0.013423201	-1195.503852	0.006421605	-1050.337653	0.015195411	deoxycytidine kinase-like isoform X2
XM_020101615.1	-9.178017793	0	-3.46333422	0	-5.269012463	0	-3630.88025	0.007993493	-3210.971634	0.005469928	-3.254497749	0	-3.137741361	0	-2.755068434	1.11022E-16	-3.563411695	0	-4353.177215	0.004114747	-3.246856396	0	-3.469734314	0	protein polybromo-1 isoform X3
XM_020101965.1	-14.34225605	0	-7715.04418	0.001409721	-15.95215496	0	-10.9864894	0	-35.47361665	0	-36.64041743	0	-2.874325812	0	-15.13216787	0	-7714.610046	0.001519248	-7.336764065	0	-2.50711365	0	-5.22572944	0	leucine-rich repeat and immunoglobulin-like domain-containing nogo receptor-interacting protein 1
XM_020101968.1	-14.27929464	0	-11.03455831	0	-7.519727349	0	-3.844477702	0	-836.7653342	0	-17205.87834	0.00020922	-4.788860939	0	-4.07166923	0	-15347.80506	0.000638344	-20282.11014	0.000682346	-58.01575407	0	-15506.4709	0.00075088	PI-PLC X domain-containing protein 3
XM_020102405.1	-7.844060387	1.38897E-09	-5.2753321	9.76048E-09	-3.91678094	3.44469E-08	-782.0150346	0.031268548	-5.42027467	2.45063E-08	-2.606368153	1.32378E-05	-832.0937502	0.020305419	-736.0003891	0.017190817	-709.4627195	0.020184865	-4.563416845	4.70412E-09	-815.8650303	0.009943034	-2.237190567	0.000376242	golgin subfamily A member 1 isoform X1
XM_020102696.1	-7.694856224	1.88629E-07	-2.682331158	0.00022768	-7.782243883	1.07616E-08	-2.016758128	0.005765938	-2.015779368	0.005793626	-19.88416987	9.55568E-09	-3.539452693	7.77016E-06	-3.800567493	4.87973E-06	-3.727463172	9.8528E-06	-19.14582601	1.61197E-08	-2.697663143	6.12614E-05	-2.229219694	0.001771756	serine/threonine-protein kinase NIM1-like
XM_020102765.1	-613.2049979	0.02771126	-588.7426071	0.023090655	-622.1468786	0.013825911	-648.8904918	0.03634409	-573.8467863	0.029033432	-659.9824146	0.013730454	-690.4387332	0.024074694	-610.7042339	0.020618768	-588.6843173	0.024037534	-777.9457785	0.022788846	-676.9727784	0.012223769	-594.7701446	0.0258518	ATP-binding cassette sub-family G member 4-like isoform X1
XM_020103552.1	-1141.579309	0.015718476	-1096.038651	0.012620247	-1158.226052	0.006916842	-1208.013571	0.021750406	-1068.307695	0.016507355	-1228.662962	0.006905634	-1285.389805	0.013461006	-1136.948086	0.011078183	-1095.953606	0.013234465	-1448.301673	0.012757652	-1260.320236	0.006035871	-2.869215046	3.63124E-07	aldose 1-epimerase isoform X1
XM_020103863.1	-5.354627984	0	-5.599825555	0	-2.966423926	0	-19.69883537	0	-17.15780171	0	-6.038600304	0	-53.45125053	0	-102.2448651	0	-12.58944137	0	-6.396051764	0	-4.105815364	0	-3.352192606	0	cAMP-dependent protein kinase catalytic subunit PRKX
XM_020103972.1	-2969.922165	0.00604257	-2.253544895	4.93245E-11	-3013.230177	0.00214267	-27.20662106	0	-2779.299412	0.006367348	-4.315579035	0	-4.337446688	0	-3.677616052	0	-2851.266385	0.004831658	-3767.945879	0.004806486	-3278.887631	0.001830201	-4.502559541	0	alpha-actinin-4-like isoform X6
XM_020104173.1	-4.232310606	0	-4.323435483	0	-5.502721452	0	-2.825338385	1.06581E-13	-2.942469498	2.13163E-14	-4.263292186	0	-2.558506595	5.75096E-13	-4.419480889	0	-9.882506916	0	-9.019032814	0	-5.025956525	0	-3.67846051	0	protein-methionine sulfoxide oxidase mical2b-like
XM_020104412.1	-1015.775902	0.017539369	-975.2538789	0.014184393	-1030.588154	0.007908679	-1074.889029	0.024018238	-950.5789073	0.018411666	-1093.262832	0.007887498	-3.323401823	2.9634E-08	-1011.651931	0.01249243	-975.1752043	0.014853545	-1288.693127	0.014270601	-3.948604022	1.77422E-10	-985.2565802	0.016159562	serine/arginine-rich splicing factor 4-like isoform X2
XM_020104820.1	-11.01920467	4.51551E-08	-2.574690148	0.000229644	-9.31695476	1.9735E-09	-30.09676356	1.62892E-06	-2.863958362	0.00011272	-5.187732287	2.55133E-08	-2.833893793	4.9653E-05	-6.681168695	3.61895E-08	-3.800833459	4.20125E-06	-13.90727129	1.97704E-09	-3.579430957	8.82672E-07	-3.203137298	2.52998E-05	protein PAT1 homolog 2-like isoform X2
XM_020104925.1	-11.10857866	4.16633E-10	-5.793180587	2.77996E-09	-8.671099631	1.17462E-11	-4.214797637	1.51716E-07	-2.723023051	2.9321E-05	-6.557000832	3.35209E-11	-4.867823072	4.34851E-09	-2.753289681	1.08838E-05	-8.283246731	4.70423E-10	-6.281530342	9.44772E-11	-5.408679001	1.13379E-10	-2.051207541	0.00104099	Hermansky-Pudlak syndrome 3 protein isoform X3
XM_020104989.1	-1326.090974	0.013627389	-1273.189651	0.010838431	-1345.428304	0.00580874	-2.934816017	9.26525E-08	-4.30986719	6.41041E-11	-49.81375764	2.24265E-14	-84.17417432	2.67336E-10	-5.926086949	3.28626E-14	-1273.095263	0.011387113	-1682.394207	0.011025677	-101.1315142	3.56808E-11	-1286.256541	0.012468057	protein FAM124A-like isoform X3
XM_020105063.1	-8.435627691	0	-3.307941924	0	-4.926881512	0	-2.255471548	1.6852E-11	-2.323963668	4.03333E-12	-3.023033224	0	-2.854383899	0	-3.578442367	0	-4.707085894	0	-7.52025544	0	-5.741939168	0	-3.478215464	0	phosphatidylserine decarboxylase proenzyme, mitochondrial isoform X1
XM_020105190.1	-13.10897099	0	-1061.572099	1.72751E-13	-2.022095315	2.15383E-14	-10358.10714	0.00277351	-2.090877429	8.43769E-15	-2.098727496	8.88178E-16	-3.332956864	0	-3.702926153	0	-2.610993146	0	-2.328334426	0	-2.242622683	0	-2.170527739	3.33067E-16	microtubule-associated protein 4-like isoform X2
XM_020105518.1	-16.87623028	8.11744E-05	-3.053582122	0.001557952	-8.778901128	7.87534E-06	-5.501750469	0.000171249	-6.561802803	0.000112952	-9.166276911	5.2013E-06	-2.527771227	0.004048699	-3.250221671	0.000835361	-8.853326547	4.33184E-05	-21.30254013	1.99775E-05	-6.941828274	5.56058E-06	-5.036494072	0.000153796	cullin-5 isoform X2
XM_020105629.1	-2.062347636	8.47467E-12	-24.29782536	0	-22.68463546	0	-5494.623849	0.005327779	-2.714401649	0	-3.655613866	0	-9.299551467	0	-5.402080177	0	-4985.018157	0.002559869	-3.465964904	0	-3.428446277	0	-2.832424267	0	B-cell receptor-associated protein 31
XM_020105967.1	-3.793459156	0.001209141	-6.222494363	0.000199306	-33.93920799	0.00023507	-2.208122268	0.023793715	-4.729359902	0.000695982	-6.612006919	3.63775E-05	-3.469010141	0.001102572	-3.720228926	0.000880793	-3.107771082	0.002928853	-2.962507804	0.001935372	-3.251509329	0.000756774	-3.053098766	0.003409373	OTU domain-containing protein 4-like
XM_020106193.1	-14.07245624	0.000244543	-4.332535229	0.000718031	-17.54900247	6.3667E-05	-3.45560681	0.002707978	-3.071401963	0.004656002	-3.876202634	0.000393909	-4.184531706	0.000531775	-5.362592264	0.000253283	-5.942040864	0.000297401	-6.148259374	7.70313E-05	-9.974431508	1.85889E-05	-4.885649375	0.000552117	AT-hook-containing transcription factor-like
XM_020106301.1	-3.173025067	1.4899E-05	-3.286018567	8.12196E-06	-630.6560718	0.013625548	-2.452515732	0.000368062	-581.6953721	0.028689675	-669.0090899	0.013532924	-699.882401	0.023781064	-619.0573109	0.02035041	-596.7362108	0.023736888	-788.5863483	0.022511334	-686.2322618	0.012043247	-602.9052787	0.025536097	histone-lysine N-methyltransferase, H3 lysine-36 and H4 lysine-20 specific-like

XM_020106621.1	-2.335724185	5.69968E-06	-1313.451242	0.010497616	-1387.97427	0.005599674	-3.439499285	2.74716E-09	-1280.219513	0.013898066	-1472.383197	0.005600119	-1540.368835	0.011277269	-1362.481165	0.009167044	-2.655619642	3.28773E-07	-5.971687882	1.22125E-15	-1510.32629	0.004867415	-2.246514659	1.2611E-05	complement C1q tumor necrosis factor-related protein 6-like
XM_020106806.1	-780.9428746	0.022332004	-749.7889704	0.018348732	-792.3307433	0.010623844	-826.3898822	0.029889618	-730.8185034	0.023419403	-840.5159217	0.010571262	-879.3120892	0.019294563	-777.7657742	0.016278184	-749.7221869	0.019154311	-990.7571736	0.018271216	-862.1624476	0.009345662	-757.4728261	0.020711783	ankyrin repeat domain-containing protein 45 isoform X2
XM_020106908.1	-1560.924001	0.011635161	-3.173950461	1.38862E-09	-1583.685714	0.004787301	-1651.762047	0.016570141	-1460.736988	0.012232983	-1679.99673	0.004793836	-4.364129568	2.22045E-16	-2.386946438	1.26192E-08	-1498.54828	0.009603938	-1980.330161	0.009347997	-1723.294409	0.004126814	-2.563291243	3.7258E-09	HEAT repeat-containing protein 5B isoform X2
XM_020106974.1	-2324.13134	0.007787299	-4.573706423	0	-2.643715805	1.94622E-13	-2459.384273	0.011532322	-2174.958301	0.008199062	-8.015282583	0	-2616.946965	0.006554231	-2314.731945	0.00512523	-2231.270587	0.006313092	-2948.622008	0.006225984	-5.506962995	0	-5.841483711	0	nuclear receptor coactivator 2 isoform X1
XM_020107664.1	-2600.898837	0.006964121	-2497.142011	0.005295602	-2638.825675	0.00255304	-2752.258268	0.010421888	-2433.961634	0.007334948	-2799.304474	0.002570188	-2928.588002	0.005846265	-2590.383486	0.004535791	-2496.983072	0.005613013	-3299.76081	0.005555798	-2871.470358	0.002187814	-2522.79692	0.006252795	PREDICTED: myozenin-1-like
XM_020108357.1	-5.617901393	0	-4.151507548	0	-5.343115385	0	-4.374073568	0	-3.701253632	0	-5.476080902	0	-3.700682884	0	-5.133784283	0	-8.252989312	0	-14.5126201	0	-4.474486681	0	-3.791307119	0	receptor-type tyrosine-protein phosphatase beta
XM_020108639.1	-2.376775789	0.001988219	-427.6962439	0.030975361	-451.9630139	0.019357046	-471.3911015	0.046676672	-11.99382746	2.25609E-06	-479.4489074	0.019177151	-501.5653771	0.031953608	-443.6426937	0.027890595	-427.6464477	0.032132016	-2.641199624	0.000324777	-491.7831092	0.017226009	-432.0674632	0.034320847	mitochondrial inner membrane protease subunit 2
XM_020109341.1	-697.0739363	0.024734006	-669.2657887	0.020458166	-707.2388109	0.012035144	-737.640187	0.032787407	-652.3326448	0.025927041	-750.2491681	0.011964332	-784.8754112	0.021425586	-694.2350041	0.01820558	-669.2032521	0.021328241	-884.351476	0.020285196	-769.567613	0.0106123	-676.1214854	0.023003377	transcription elongation regulator 1 isoform X2
XM_020109416.1	-780.9428746	0.022332004	-749.7889704	0.018348732	-792.3307433	0.010623844	-826.3898822	0.029889618	-730.8185034	0.023419403	-840.5159217	0.010571262	-879.3120892	0.019294563	-777.7657742	0.016278184	-749.7221869	0.019154311	-990.7571736	0.018271216	-862.1624476	0.009345662	-757.4728261	0.020711783	BTB/POZ domain-containing protein 6-B-like isoform X1
XM_020109535.1	-1686.727409	0.010782271	-1619.439332	0.00844301	-1711.323613	0.004361336	-1784.88659	0.015468256	-1578.465776	0.011339396	-1815.39686	0.004370665	-2.763351389	1.02679E-08	-1679.898092	0.00732759	-1619.326683	0.00889767	-15.5858554	0	-1862.186661	0.003773699	-1636.067306	0.009800817	ER membrane protein complex subunit 3-like
XM_020109913.1	-3.87600338	0	-2.61440458	0	-7.040285428	0	-3.751006338	0	-2.59115868	0	-3.113753826	0	-3.22125643	0	-3.161374426	0	-5.357783586	0	-6.748982488	0	-5.017384219	0	-2.09751352	1.63314E-13	serine/threonine-protein kinase 11-interacting protein-like
XM_020109914.1	-4.388677446	1.73006E-11	-4.545339037	5.67912E-12	-5.739269902	4.10783E-15	-2.396537924	2.10232E-06	-2.603646643	4.06137E-07	-3.172354147	1.84898E-10	-2.506619112	1.96789E-07	-5.195881859	2.72449E-13	-9.245051102	2.20934E-14	-2.638689495	2.56394E-08	-5.047759945	3.88578E-15	-3.872040827	1.19268E-10	gap junction gamma-1 protein-like
XM_020110140.1	-4.605126904	0	-5.116514806	0	-3.853450343	0	-2.211813998	0	-3.982809798	0	-6.553155839	0	-3.757648745	0	-2.251453446	0	-3.761100914	0	-3.408316543	0	-6.977688034	0	-5.916618127	0	RAB6-interacting golgin
XM_020110205.1	-965.454539	0.018388007	-4.052484544	5.1632E-09	-979.5329944	0.00837884	-1021.639212	0.025067634	-903.4873922	0.019298846	-1039.102779	0.008352622	-1087.072781	0.015809138	-961.5334685	0.013156384	-926.8638434	0.015611136	-3.334681266	2.13195E-08	-1065.871084	0.007337862	-936.4457758	0.016963463	ubiquitin carboxyl-terminal hydrolase 43-like
XM_020110258.1	-7.107334377	2.6544E-06	-411.5916076	0.032063239	-434.9446274	0.020137526	-453.6411625	0.048070669	-401.1778974	0.03946531	-2.013713733	0.005694015	-3.016383588	0.000170009	-3.949199379	2.18604E-05	-2.568740163	0.001103793	-543.8532439	0.031258545	-4.349379257	2.05199E-06	-415.797195	0.035483081	zinc transporter 1-like isoform X1
XM_020110374.1	-2.180652573	7.63322E-07	-2.258948773	2.36106E-07	-15.22180475	0	-1527.512474	0.017703005	-3.258775279	5.33984E-11	-4.002903641	9.99201E-16	-4.035843122	1.37668E-14	-1437.658858	0.008624362	-2.348516443	9.22909E-08	-4.124920389	1.11022E-15	-8.402251144	0	-3.964633446	2.1938E-13	renalase isoform X1
XM_020110543.1	-3.610701598	0	-5.538885974	0	-7.74646823	0	-3.863465335	0	-3.052113392	0	-5.330824026	0	-2.435801766	0	-4.086537895	0	-6.604809915	0	-7.940641568	0	-5.16970237	0	-2.767352818	0	glucocorticoid modulatory element-binding protein 2-like
XM_020111478.1	-520.9491658	0.031942771	-2.114097063	0.00300402	-528.545753	0.016442738	-551.2658272	0.04134081	-487.5123419	0.03344535	-560.6889856	0.016308718	-586.5583874	0.027853041	-518.8203868	0.024089763	-500.113489	0.02791308	-660.8995112	0.02635995	-2.831041913	3.69909E-05	-505.2836698	0.029913698	histone-lysine N-methyltransferase KMT5B
XM_020111598.1	-10.04855877	1.60606E-07	-6.289125669	3.12533E-07	-528.545753	0.016442738	-551.2658272	0.04134081	-487.5123419	0.03344535	-8.869363935	5.30905E-09	-2.683192572	0.000166916	-3.740347404	6.52896E-06	-2.71662544	0.000232067	-12.68216415	8.00752E-09	-575.1184603	0.014586082	-8.662751863	1.7593E-07	trafficking protein particle complex subunit 1
XM_020111726.1	-5.957478005	1.32639E-11	-7.61022078	8.80185E-13	-5.858507405	3.47722E-13	-3.571024241	2.71721E-08	-9.755030638	1.95055E-12	-11.73461779	6.66134E-16	-2.308884255	1.48063E-06	-2.387452324	8.77626E-07	-5.559597375	7.89258E-13	-4.165334141	1.22247E-12	-2.051387551	7.89613E-06	-6.603011855	2.40141E-13	C-X-C chemokine receptor type 1-like
XM_020111977.1	-5.309618133	8.60367E-12	-1128.247924	0.012256772	-8.28987206	7.77156E-16	-3.023054912	1.66998E-07	-5.187661069	2.95307E-11	-3.20164857	3.79672E-09	-5.248938559	1.34692E-12	-8.930051688	9.4369E-15	-4.411377332	9.80651E-11	-2.426733001	2.42357E-06	-7.656014944	2.22045E-16	-5.131488732	1.2943E-11	GTPase IMAP family member 8-like
XM_020112063.1	-3.785137071	2.65251E-06	-2.983661357	2.70241E-05	-4.540535685	5.66602E-08	-4.22218659	1.6047E-06	-3.556578881	7.59317E-06	-7.263082368	7.99157E-10	-2.10880882	0.001269508	-3.777692134	1.28799E-06	-9.100821162	1.33828E-08	-7.536405935	1.24569E-09	-3.030832275	4.30419E-06	-3.010668135	2.90266E-05	sodium/calcium exchanger 2-like
XM_020112269.1	-2.537225961	1.51515E-06	-2.286049693	1.08297E-05	-1311.391531	0.005986073	-1367.763023	0.019545375	-1209.582241	0.014674168	-1391.143118	0.00598331	-3.213118077	3.95485E-09	-1287.303472	0.009732031	-1240.887689	0.011685795	-1639.831928	0.011306162	-1426.990939	0.005209689	-2.679932668	4.81305E-07	serine/threonine-protein kinase DCLK2-like
XM_020112351.1	-319.6637138	0.04810078	-2.281885075	0.008734547	-324.3251154	0.027074399	-3.507945567	0.000780797	-3.887323688	0.000460817	-3.082684339	0.000438423	-359.9103601	0.042396781	-318.3465385	0.037700864	-306.8680455	0.042924515	-4.705156706	3.36409E-05	-3.243127965	0.000238343	-310.040452	0.045538545	PH domain leucine-rich repeat-containing protein phosphatase 1-like
XM_020112369.1	-1091.257946	0.01640077	-1047.724742	0.013205044	-1107.170893	0.007285688	-1154.763754	0.022602871	-1021.21618	0.017221025	-1174.50291	0.00727088	-1228.727798	0.014060223	-1086.829624	0.011606386	-1047.642246	0.013840067	-1384.458254	0.013324063	-1204.763336	0.006363884	-1058.472787	0.015082618	signal peptidase complex subunit 2
XM_020112487.1	-1997.042481	0.009114125	-1917.375104	0.007057	-2026.163762	0.003550043	-105.2120009	3.43369E-11	-5.660901459	1.11022E-16	-2149.383848	0.003563791	-2248.643921	0.007701106	-1988.961941	0.006093718	-1917.246741	0.007453473	-2533.639788	0.007311141	-2204.787549	0.003060265	-1937.067267	0.00824556	carnitine O-palmitoyltransferase 1, liver isoform-like
XM_020112560.1	-554.4967411	0.030261505	-532.37638	0.02535982	-562.5825259	0.01539347	-586.7657052	0.039363121	-518.9066853	0.031692825	-596.7956871	0.015275241	-624.3330586	0.026350073	-552.2326948	0.022705222	-532.3210629	0.026369971	-703.4617902	0.024939379	-612.1563941	0.013637871	-537.8242061	0.028298051	UNC119-binding protein C5orf30 homolog
XM_020113371.1	-7.463061119	0	-2.997289511	0	-9.194786318	0	-3.762642483	0	-3.365380559	0	-3.96427006	0	-4.431899812	0	-3.787422864	0	-5.037951535	0	-10.0409932	0	-4.915952844	0	-3.212321044	0	E3 ubiquitin-protein ligase RING2-A-like
XR_002202478.1	-5.255305004	0	-2.998291926	0	-8.920357796	0	-3.474364895	0	-5.45418428	0	-6.272363945	0	-3.392769277	0	-3.146144945	0	-4.397176916	0	-3.606279241	0	-2.184183376	0	-5.136777771	0	---NA---
XR_002202725.1	-479.0146966	0.034330073	-459.9055166	0.02900437	-485.9997868	0.017953054	-506.8909796	0.044133124	-2.297575176	0.002405204	-515.5556089	0.017795606	-539.3400483	0.0299909	-3.865669218	1.01751E-05	-459.8540216	0.030111116	-607.6966625	0.028380718	-528.821043	0.015953096	-464.6079994	0.032211555	cAMP-dependent protein kinase inhibitor alpha-like
XR_002202944.1	-5.808214238	0	-2368.304921	0.005588531	-59.86384698	0	-2.118664991	1.25265E-08	-5.457581609	0	-8.506991674	0	-4.303219063	0	-9.140732023	0	-12.86384834	0	-3.191241145	0	-6.609470468	0	-12.63507929	0	---NA---
XR_002203535.1	-504.1753781	0.032856209	-484.062471	0.027680828	-511.5273665	0.017017853	-533.5158881	0.042411352	-471.8151701	0.034397282	-542.6356349	0.016875004	-567.6710517	0.028670532	-502.1142328	0.024844844	-484.0097021	0.028753168	-8.235205866	1.89363E-08	-556.5994934	0.015106337	-489.0134017	0.030792409	---NA---
