# Supplementary material for: Investigating the effects of Carpesii fructus extract on the liver transcriptome of olive flounder (Paralichthys olivaceus) as a potential antiparasitic agent
Source: Genet Mol Biol. 2024 Mar 4;47(1):e20230146. doi: 10.1590/1678-4685-GMB-2023-0146 (PMC10941726; doi:10.1590/1678-4685-GMB-2023-0146)
Supplement: Table S4 - [file 1415-4757-GMB-47-1-e20230146-s7.pdf]

Genetics and Molecular Biology

https://doi.org/10.1590/1678-4685-GMB-2023-0146

Supplementary Material to "Investigating the effects of Carpesii fructus extract on the liver transcriptome of olive flounder (*Paralichthys olivaceus*) as a potential antiparasitic agent"

Table S4 - Primer sequences for qRT-PCR validation of differentially expressed genes in the whole transcriptome assembly.

Genes	Primers	Sequences (5´ - 3´)
CYP1A1	qPO_CYP1A1_FW1	GAAGCTGCACTTCCACACAA
	qPO_CYP1A1_RV1	TGGTGATGAGCTTCACTGCT
CYP24A1	qPO_CYP24A1_FW1	CAATGTGCTGGTTGGTCAGA
	qPO_CYP24A1_RV1	TGGATTTGGAGCCTCATCTC
CYP27B1	qPO_CYP27B1_FW1	CCTCGTAGAGTTCGATGTGA
	qPO_CYP27B1_RV1	CATCGCTCGATAAACTGGAG
CYP25	qPO_CYP25_FW1	TCTTCAGCTGAGCACGTGAT
	qPO_CYP25_RV1	TGAACCACAGTGGGTGTACT
UGT	qPO_UGT_FW1	AGAAGGGCAACTGCAAAGAC
	qPO_UGT_RV1	GTTGAGTCGGCTTCAGTCAA
NR1D1	qPO_NR1D1_FW1	AAAGCACTGCGCTCTCTCAT
	qPO_NR1D1_RV1	TCTGAGCTCAAGGGTCAATG
ACTB	qPO_ACTB_FW1	CAGCATCATGAAGTGTGACG
	qPO_ACTB_RV1	CTTCTGCATACGGTCAGCAA
